# Distinct and Overlapping Roles of Hippo Effectors YAP and TAZ During Human and Mouse Hepatocarcinogenesis

**DOI:** 10.1016/j.jcmgh.2020.11.008

**Published:** 2020-11-21

**Authors:** Haichuan Wang, Jingxiao Wang, Shanshan Zhang, Jiaoyuan Jia, Xianqiong Liu, Jie Zhang, Pan Wang, Xinhua Song, Li Che, Ke Liu, Silvia Ribback, Antonio Cigliano, Matthias Evert, Hong Wu, Diego F. Calvisi, Yong Zeng, Xin Chen

**Affiliations:** 1 Liver Transplantation Division, Department of Liver Surgery, Laboratory of Liver Surgery, West China Hospital, Sichuan University, Chengdu, Sichuan, People's Republic of China; 2 Department of Bioengineering and Therapeutic Sciences and Liver Center, University of California, San Francisco, California; 3 School of Life Sciences, Beijing University of Chinese Medicine, Beijing, China; 4 Department of Oncology and Hematology, The Second Hospital, Jilin University, Changchun, China; 5 School of Pharmacy, Hubei University of Chinese Medicine Wuhan, Hubei, China; 6 Department of Pediatrics and Human Development, East Lansing, Michigan; 7 Department of Pharmacology and Toxicology, College of Human Medicine, Michigan State University, East Lansing, Michigan; 8 Institute of Pathology, University of Greifswald, Greifswald, Germany; 9 Institute of Pathology, University Clinic of Regensburg, Regensburg, Germany

**Keywords:** YAP, TAZ, Hepatocellular Carcinoma, Cell Cycle, Akt, protein kinase B, BrdU, bromodeoxyuridine, Ct, cycle threshold, HCC, hepatocellular carcinoma, ICC, intrahepatic cholangiocarcinoma, IHC, immunohistochemistry, KEGG, Kyoto encyclopedia of genes and genomes, KO, knockout, LATS1/2, large tumor-suppressor kinase 1/2, MAPK, mitogen-activated protein kinase, mRNA, messenger RNA, myr-Akt-HA, myristoylated-protein kinase B-hemagglutinin tag, NEDD4, developmentally down-regulated protein 4, NRas, Neuroblastoma Ras viral oncogene homolog, pCMV, cytomegalovirus promoter, p-Erk1/2, phosphorylated extracellular regulated kinase 1/2, PTX3, pentraxin 3, qPCR, quantitative polymerase chain reaction, siRNA, small interfering RNA, siSC, scrambled small interfering RNA, siTAZ, small interfering targeting transcriptional co-activator with PDZ-binding motif, TAZ, transcriptional co-activator with PDZ-binding motif, siYAP, small interfering RNA targeting yes-associated protein, siYT, small interfering RNA targeting yes-associated protein/transcriptional co-activator with PDZ-binding motif, TCGA, the cancer genome atlas, TEAD, TEA domain, V12, mutation at position 12 replacing amino acid glycine with valine, YAP, yes-associated protein

## Abstract

**Background & Aims:**

Yes-associated protein (YAP) and its paralog transcriptional co-activator with post synaptic density protein, drosophila disc large tumor suppressor and zonula occludens-1-binding motif (TAZ) are 2 co-activators downstream of Hippo tumor-suppressor cascade. Both have been implicated in the development of hepatocellular carcinoma (HCC). However, whether YAP and TAZ have distinct or overlapping functions during hepatocarcinogenesis remains unknown.

**Methods:**

Expression patterns of YAP and TAZ were analyzed in human HCC samples. The requirement of Yap and/or Taz in protein kinase B (Akt)/ neuroblastoma RAS viral oncogene homolog (NRas) -driven liver tumorigenesis was analyzed using conditional *Yap*, *Taz*, and *Yap*;*Taz* knockout mice. Transcriptional programs regulated by YAP and/or TAZ were identified via RNA sequencing.

**Results:**

We found that in human HCC samples, an almost ubiquitous activation of YAP or TAZ occurs, underlying their role in this tumor type. Intriguingly, 70% of HCC samples showed only nuclear YAP or TAZ immunoreactivity. In the Akt/NRas liver tumor model, where nuclear Yap and Taz can be detected readily, deletion of *Yap* or *Taz* alone only mildly delayed liver tumor development, whereas their concomitant ablation strongly inhibited tumor cell proliferation and significantly suppressed Akt/NRas-driven hepatocarcinogenesis. In HCC cell lines, silencing of either YAP or TAZ led to decreased expression of both overlapping and distinct sets of genes, with the most prominent gene signatures related to cell-cycle progression and DNA replication.

**Conclusions:**

YAP and TAZ have overlapping and distinct roles in hepatocarcinogenesis. HCCs may display unique activation of YAP or TAZ, thus relying on either YAP or TAZ for their growth.

## Graphical abstract

**Figure:**
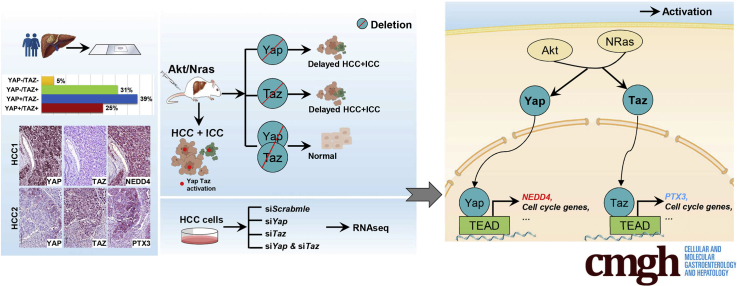

**Summary**

Yes-associated protein (YAP) and transcriptional co-activator with post synaptic density protein, drosophila disc large tumor suppressor and zonula occludens-1-binding motif (TAZ) coordinately regulate liver cancer development. Human liver tumors show ubiquitous activation of YAP or TAZ, thus relying on either YAP or TAZ for their growth.

Hepatocellular carcinoma (HCC), comprising more than 80% of primary liver cancer cases, continues to increase in incidence and lethality, thus representing a major health concern worldwide.^1^^,^^2^ Treatment options for HCC are limited and generally ineffective. Surgical resection and liver transplantation are the only available curative treatments for HCC. However, they can be applied only to early stage HCC patients. The most recent clinical study showed that atezolizumab (a monoclonal antibody that attaches to programmed death-ligand 1) in combination with bevacizumab (a monoclonal antibody targeting vascular endothelial growth factor) provides better overall and progression-free survival outcomes than the multikinase inhibitor sorafenib, although approximately 20% of HCC patients still progressed.^3^ Thus, a better understanding of the molecular pathways underlying HCC pathogenesis is needed to develop novel and more effective therapies against HCC.

The Hippo tumor-suppressor cascade is an evolutionally conserved pathway that controls organ size, tissue regeneration, stem cell self-renewal, and tumor development.^4^ Macrophage stimulating 1 and serine/threonine kinase 3 together with Salvador family WW domain containing protein 1 form the core Hippo regulatory complex and activate large tumor-suppressor kinase 1/2 (LATS1/2). Once activated, LATS1/2 proteins phosphorylate and inhibit Yes-associated protein (YAP) and/or transcriptional co-activator with post synaptic density protein, drosophila disc large tumor suppressor and zonula occludens-1 (PDZ) -binding motif (TAZ) transcriptional co-activators, leading to their cytoplasmic sequestration. YAP and TAZ function mainly via interacting with the DNA binding proteins TEA domain (TEAD) to promote target gene expression.^5^ In addition, they have been found to regulate additional signaling cascades in a TEAD-independent manner.^6^ YAP and TAZ are highly similar proteins and share multiple functional domains, including TEAD binding and transcriptional activation domains. Comparison of the most similar variants of human YAP and TAZ has shown an overall similarity of approximately 53%. Although several functional features are shared between YAP and TAZ, different roles played by the 2 proteins also have been described.^7^ In liver cancer, these commonalities and differences have not been explored in detail.

Mounting evidence has indicated there is coordinated activation of protein kinase B (AKT)/ the mammalian target of rapamycin (mTOR) and Ras guanosine triphosphate (GTP)ase (RAS)/mitogen-activated protein kinase (MAPK) pathways in a large subset of human HCC.^8^ This biochemical cross-talk can be reproduced via liver-specific co-expression of activated forms of Akt and neuroblastoma Ras GTPase viral oncogene homolog (NRas in mice (Akt/NRas).^8^ Recently, we showed that Hippo signaling is inactivated in the Akt/NRas mouse liver tumor model, leading to nuclear accumulation of Yap and Taz. Restoring Hippo activity via either overexpression of Lats2 or blocking Yap and Taz function by hydrodynamic transfection of the dominant-negative form of Tead family member 2 strongly inhibited Akt/NRas-induced hepatocarcinogenesis in mice.^9^

Here, using human HCC samples, we examined YAP and TAZ expression patterns in this tumor type. Furthermore, using in vitro and in vivo approaches, we investigated whether YAP and TAZ possess distinct or redundant roles along hepatocellular carcinogenesis.

## Results

### Distinct and Overlapping Activation of YAP and TAZ in Human HCC Samples

**Figure 1:**
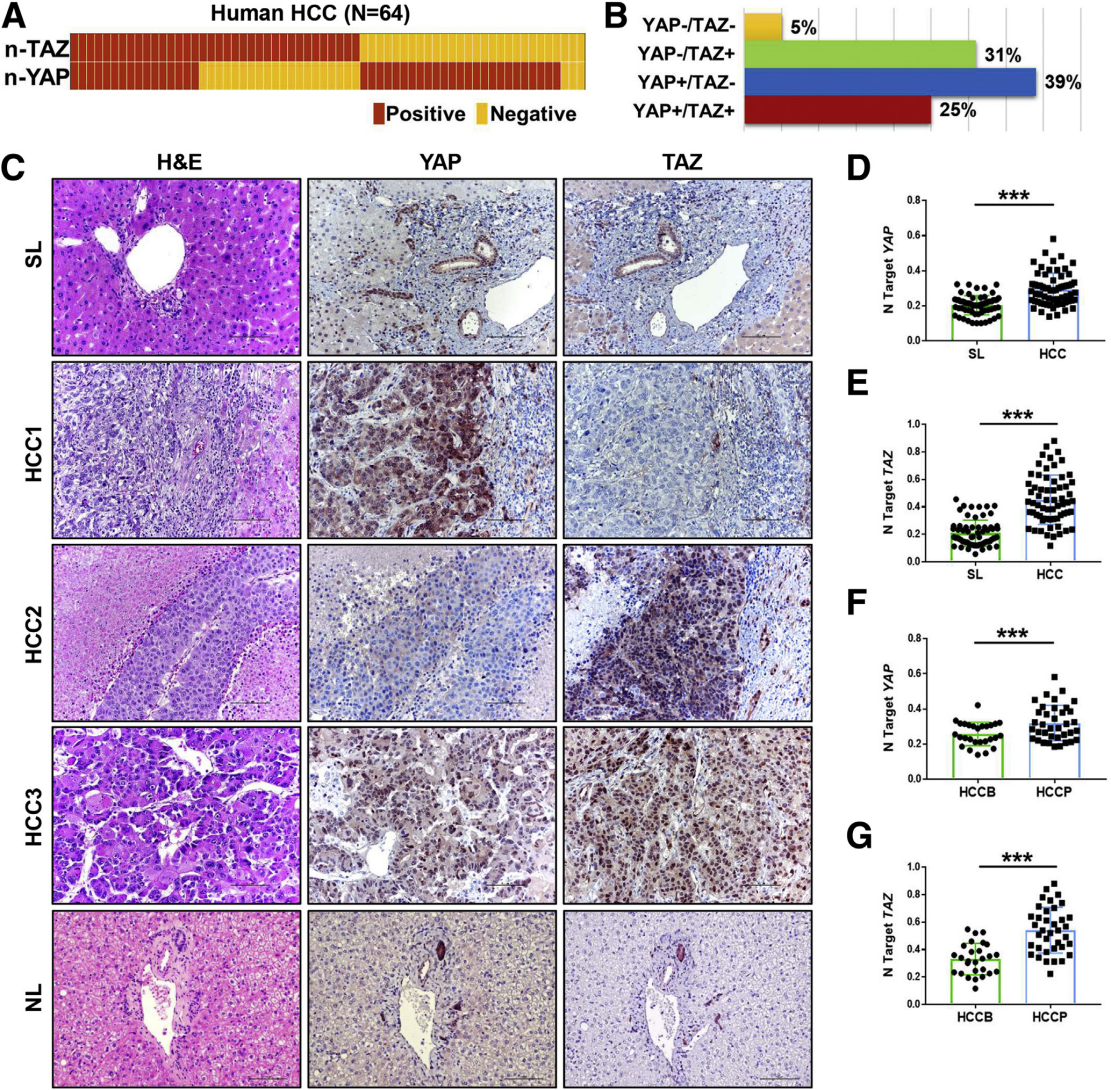
**YAP and TAZ expression in human HCC samples.** (*A*) Heatmap of nuclear TAZ and YAP staining patterns among HCC samples (N = 64). (*B*) Percentage of 4 different nuclear YAP and TAZ staining patterns. (*C*) Representative images of IHC staining against YAP and TAZ in human normal liver (NL), nontumorous surrounding tumor tissues (SL), and HCC. Original magnification: 200×. *Scale bar*: 100 μm. (*D* and *E*) Real-time qPCR analysis of *YAP* and *TAZ* mRNA levels in HCC (N = 64) and corresponding nontumorous surrounding liver tissues (SL) (N = 64). (*F* and *G*) Real-time qPCR analysis of *YAP* and *TAZ* mRNA levels in HCC with a better prognosis (HCCB) (*n* = 27) and HCC with a poorer prognosis (HCCP) (*n* = 37). Quantitative values were calculated by using the PE Biosystems Analysis software and expressed as a number target (N Target). N Target = 2^−ΔCt^, wherein the ΔCt value of each sample was calculated by subtracting the average Ct value of the gene of interest from the average Ct value of the *β-actin* gene. The *P* value was calculated using the Student *t* test: ∗∗∗*P* < .001 when compared with SL or HCCB.

To test the activation status of YAP and TAZ in human HCCs, we first determined the staining patterns of YAP and TAZ in a collection of human HCC specimens (N = 64) (Table 1), with normal liver tissue used as a negative control. Nuclear immunoreactivity for YAP and TAZ (a readout of their activation) was found in 36 and 41 HCC specimens, respectively (56.3% and 64.1%, respectively) (Figure 1*A*). Of note, immunoreactivity for YAP and/or TAZ was found in 61 of 64 (95.3%) samples, implying the almost-ubiquitous activation of at least 1 Hippo pathway member in human HCC. However, only 16 HCC samples showed both YAP and TAZ nuclear staining, whereas 20 (31%) HCCs and 25 (39%) HCCs of the 64 samples analyzed showed only TAZ or YAP immunoreactivity, respectively (Figure 1*B* and *C*). Consistent with the fact that YAP/TAZ are expressed in most HCCs, real-time quantitative polymerase chain reaction (qPCR) analysis showed significant up-regulation of *YAP* and *TAZ* messenger RNA (mRNA) as in HCC when compared with corresponding nontumorous surrounding livers (Figure 1*D* and *E*). Furthermore, higher levels of *YAP* and *TAZ* mRNA were detected in the most aggressive clinical tumors (HCC with shorter/poorer survival), when compared with those with a better survival (Figure 1*F* and *G*).

**Table 1:** Clinicopathologic Features of HCC Patients

Variables Features		
	HCCB	HCCP
Patients, n	27	37
Male	21	27
Female	6	10
Age, *y*, mean ± SD	66.4 ± 9.8	67.8 ± 10.0
Etiology		
HBV	14	14
HCV	19	11
Ethanol	4	8
Wilson disease	0	1
Hemochromatosis	0	1
NA	0	2
Cirrhosis		
Positive	20	28
Negative	7	9
Tumor size		
>5 cm	15	21
<5 cm	12	16
Edmondson and Steiner grade		
I	6	6
II	8	10
III	8	12
IV	5	9
Serum α-fetoprotein level, *ng/mL*		
>300	11	15
<300	14	21
N/A	2	1
Survival after partial liver resection, *mo* Means ± SD		
	57.2 ± 17.1	17.4 ± 10.1

HBV, hepatitis B virus; HCCB, HCC with longer/better survival; HCCP, HCC with shorter/poorer survival; HCV, hepatitis C virus.

In summary, the data indicate that inactivation of Hippo cascade occurs in most HCC samples (61 of 64; 95.3%), leading to nuclear localization and activation of YAP and/or TAZ in human HCCs.

### Yap and Taz Are Functionally Redundant in the Akt/NRas Murine Liver Tumor Model

**Figure 2:**
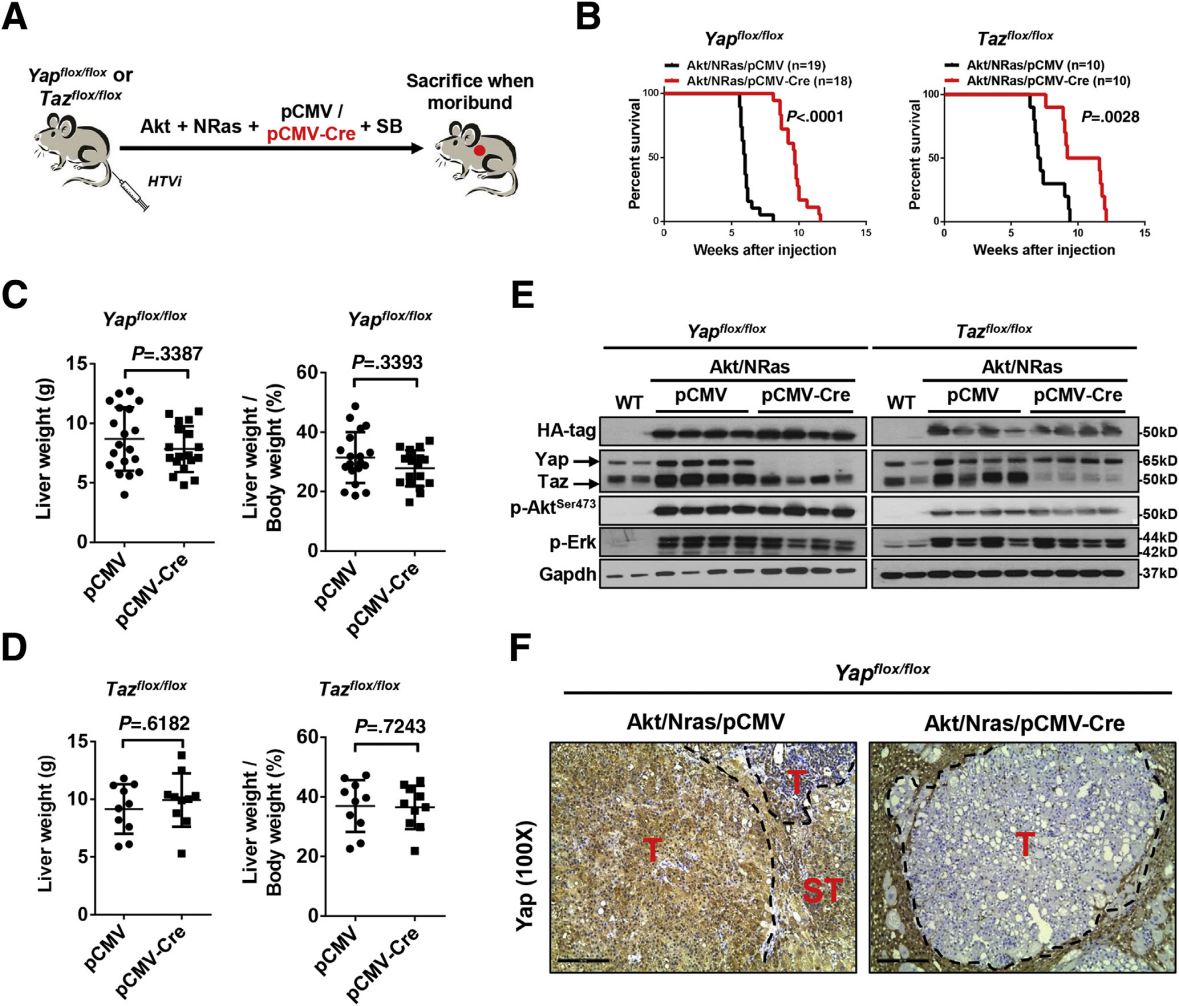
**Yap and Taz are functionally redundant during Akt/NRas-driven mouse hepatocarcinogenesis.** (*A*) Study design. *Yap*^*flox/flox*^ or *Taz*^*flox/flox*^ conditional KO mice were injected hydrodynamically with Akt/NRas/pCMV (control) or Akt/NRas/pCMV-Cre plasmids. (*B*) Survival curves of *Yap*^*flox/flox*^ and *Taz*^*flox/flox*^ conditional KO mice injected with Akt and NRas. Mice number in each arm is labeled in the figure. (*C* and *D*) Comparisons of liver weight and liver-weight-to-body-weight ratios in (*C*) *Yap*^*flox/flox*^ and (*D*) *Taz*^*flox/flox*^ murine Akt/NRas liver tumors. (*E*) Western blot analysis showing the expression levels of HA-tag, Yap (*upper band*), Taz (*lower band*), and activation of Akt and Ras pathways in wild-type (WT) normal livers, Akt/NRas/pCMV tumors, and Akt/NRas/pCMV-Cre tumors. (*F*) Immunohistochemical staining of Yap in *Yap*^*flox/flox*^ murine Akt/NRas liver tumors. T, tumor; ST, surrounding tissues. *Scale bar*: 200 μm.

Previously, we showed increased nuclear Yap and Taz in Akt/NRas-induced liver tumor cells.^9^ To compare the functional requirements of Yap and Taz in Akt/NRas-driven hepatocarcinogenesis, we hydrodynamically injected myristoylated-protein kinase B with hemagglutinin tag (myr-Akt-HA-tagged) and NRasV12 (mutation at position 12 replacing amino acid glycine with valine) plasmids together with cytomegalovirus promoter (pCMV)-Cre into *Yap*^*flox/flox*^ or *Taz*^*flox/flox*^ mice. Consequently, Akt/NRas oncogenes were expressed exclusively in *Yap* or *Taz* knockout (KO) hepatocytes, depending on the model used. Mice injected with Akt, NRas, and pCMV (empty vector) plasmids were used as control (Figure 2*A*). Deletion of either *Yap* or *Taz* alone slightly delayed Akt/NRas-induced liver tumor development in mice (Figure 2*B*). Liver weight and liver-weight-to-body-weight ratio at the time of death showed no significant difference between the pCMV group and the pCMV-Cre group among *Yap*^*flox/flox*^ or *Taz*^*flox/flox*^ mice (Figure 2*C* and *D* and Table 2).

**Table 2:** Basic Information of Akt/NRas Murine Liver Cancer Models

Mouse strain	Plasmids	Sex	Weeks PI	BW, g	LW, g
*Yap*^*flox/flox*^	Akt/NRas/pCMV	F	5.6	22.2	6.5
*Yap*^*flox/flox*^	Akt/NRas/pCMV	F	5.7	26.5	7.5
*Yap*^*flox/flox*^	Akt/NRas/pCMV	F	5.7	25.1	6.8
*Yap*^*flox/flox*^	Akt/NRas/pCMV	F	5.7	28.2	11.9
*Yap*^*flox/flox*^	Akt/NRas/pCMV	F	5.8	27.6	8.2
*Yap*^*flox/flox*^	Akt/NRas/pCMV	F	5.8	25	11.2
*Yap*^*flox/flox*^	Akt/NRas/pCMV	F	5.9	23.2	11.3
*Yap*^*flox/flox*^	Akt/NRas/pCMV	F	6	20.5	5.9
*Yap*^*flox/flox*^	Akt/NRas/pCMV	F	7.1	25.1	7
*Yap*^*flox/flox*^	Akt/NRas/pCMV	F	8.1	20.3	4
*Yap*^*flox/flox*^	Akt/NRas/pCMV	M	5.6	34.8	7.8
*Yap*^*flox/flox*^	Akt/NRas/pCMV	M	5.9	30.8	12.7
*Yap*^*flox/flox*^	Akt/NRas/pCMV	M	6	30.2	10
*Yap*^*flox/flox*^	Akt/NRas/pCMV	M	6	35.1	11.9
*Yap*^*flox/flox*^	Akt/NRas/pCMV	M	6.1	32.8	12.5
*Yap*^*flox/flox*^	Akt/NRas/pCMV	M	6.1	29.5	9.4
*Yap*^*flox/flox*^	Akt/NRas/pCMV	M	6.2	28.6	9.1
*Yap*^*flox/flox*^	Akt/NRas/pCMV	M	6.2	30.5	5.7
*Yap*^*flox/flox*^	Akt/NRas/pCMV	M	6.5	28.6	5.6
*Yap*^*flox/flox*^	Akt/NRas/pCMV-Cre	F	8.1	22.5	5.2
*Yap*^*flox/flox*^	Akt/NRas/pCMV-Cre	F	8.6	27.1	9.5
*Yap*^*flox/flox*^	Akt/NRas/pCMV-Cre	F	8.6	29.1	10.8
*Yap*^*flox/flox*^	Akt/NRas/pCMV-Cre	F	8.7	29.9	10.2
*Yap*^*flox/flox*^	Akt/NRas/pCMV-Cre	F	8.7	22.8	8
*Yap*^*flox/flox*^	Akt/NRas/pCMV-Cre	F	9.2	26.4	8
*Yap*^*flox/flox*^	Akt/NRas/pCMV-Cre	F	9.6	27.5	6.9
*Yap*^*flox/flox*^	Akt/NRas/pCMV-Cre	F	9.7	21.6	6.8
*Yap*^*flox/flox*^	Akt/NRas/pCMV-Cre	F	9.7	24.7	4.8
*Yap*^*flox/flox*^	Akt/NRas/pCMV-Cre	F	9.8	24.6	6.2
*Yap*^*flox/flox*^	Akt/NRas/pCMV-Cre	M	9.9	24.5	7.2
*Yap*^*flox/flox*^	Akt/NRas/pCMV-Cre	M	9.2	32.1	7.3
*Yap*^*flox/flox*^	Akt/NRas/pCMV-Cre	M	9.8	33.5	5.5
*Yap*^*flox/flox*^	Akt/NRas/pCMV-Cre	M	10	31	7.1
*Yap*^*flox/flox*^	Akt/NRas/pCMV-Cre	M	10	35.4	11
*Yap*^*flox/flox*^	Akt/NRas/pCMV-Cre	M	10.6	33.6	7.1
*Yap*^*flox/flox*^	Akt/NRas/pCMV-Cre	M	11.5	29.1	9.1
*Yap*^*flox/flox*^	Akt/NRas/pCMV-Cre	M	11.6	33.5	10.3
*Taz*^*flox/flox*^	Akt/NRas/pCMV	F	6.4	25.7	10.7
*Taz*^*flox/flox*^	Akt/NRas/pCMV	F	6.7	24.4	11.3
*Taz*^*flox/flox*^	Akt/NRas/pCMV	F	6.8	23.7	11.2
*Taz*^*flox/flox*^	Akt/NRas/pCMV	F	6.9	24.2	8.2
*Taz*^*flox/flox*^	Akt/NRas/pCMV	F	7	23.9	9.2
*Taz*^*flox/flox*^	Akt/NRas/pCMV	F	7.2	24.2	9.6
*Taz*^*flox/flox*^	Akt/NRas/pCMV	M	7.4	26.9	11.8
*Taz*^*flox/flox*^	Akt/NRas/pCMV	M	9.4	27	6.1
*Taz*^*flox/flox*^	Akt/NRas/pCMV	M	9.5	24.2	5.9
*Taz*^*flox/flox*^	Akt/NRas/pCMV	M	9.3	24.3	7.6
*Taz*^*flox/flox*^	Akt/NRas/pCMV-Cre	F	7.6	31.3	13.8
*Taz*^*flox/flox*^	Akt/NRas/pCMV-Cre	F	8.9	31.1	12.5
*Taz*^*flox/flox*^	Akt/NRas/pCMV-Cre	F	9	27.4	10
*Taz*^*flox/flox*^	Akt/NRas/pCMV-Cre	F	9.1	24.2	10.3
*Taz*^*flox/flox*^	Akt/NRas/pCMV-Cre	F	9.2	22.9	10.4
*Taz*^*flox/flox*^	Akt/NRas/pCMV-Cre	F	11.4	27.1	8.1
*Taz*^*flox/flox*^	Akt/NRas/pCMV-Cre	M	11.5	28	9.9
*Taz*^*flox/flox*^	Akt/NRas/pCMV-Cre	M	11.6	28.2	8.8
*Taz*^*flox/flox*^	Akt/NRas/pCMV-Cre	M	11.7	24.2	5.3
*Taz*^*flox/flox*^	Akt/NRas/pCMV-Cre	M	11.8	27	10.2
*Yap*^*flox/flox*^*;Taz*^*flox/flox*^	Akt/NRas/pCMV	F	5.9	24.6	3.1
*Yap*^*flox/flox*^*;Taz*^*flox/flox*^	Akt/NRas/pCMV	F	6.3	24.1	4.5
*Yap*^*flox/flox*^*;Taz*^*flox/flox*^	Akt/NRas/pCMV	F	6	24.1	7.6
*Yap*^*flox/flox*^*;Taz*^*flox/flox*^	Akt/NRas/pCMV	F	6.9	23.2	8.8
*Yap*^*flox/flox*^*;Taz*^*flox/flox*^	Akt/NRas/pCMV	F	7.2	26.2	4.1
*Yap*^*flox/flox*^*;Taz*^*flox/flox*^	Akt/NRas/pCMV	F	7.4	22	5.2
*Yap*^*flox/flox*^*;Taz*^*flox/flox*^	Akt/NRas/pCMV	F	8.3	18.4	3.7
*Yap*^*flox/flox*^*;Taz*^*flox/flox*^	Akt/NRas/pCMV	M	7	30.6	9.4
*Yap*^*flox/flox*^*;Taz*^*flox/flox*^	Akt/NRas/pCMV	M	7.1	32.1	9.1
*Yap*^*flox/flox*^*;Taz*^*flox/flox*^	Akt/NRas/pCMV	M	8.5	37.5	10.7
*Yap*^*flox/flox*^*;Taz*^*flox/flox*^	Akt/NRas/pCMV	M	10.5	28.5	12.5
*Yap*^*flox/flox*^*;Taz*^*flox/flox*^	Akt/NRas/pCMV	M	10.6	26.3	8.6
*Yap*^*flox/flox*^*;Taz*^*flox/flox*^	Akt/NRas/pCMV	M	10.4	25.9	8.7
*Yap*^*flox/flox*^*;Taz*^*flox/flox*^	Akt/NRas/pCMV-Cre	F	8.3	17.5	0.8
*Yap*^*flox/flox*^*;Taz*^*flox/flox*^	Akt/NRas/pCMV-Cre	F	12.5	33	1.3
*Yap*^*flox/flox*^*;Taz*^*flox/flox*^	Akt/NRas/pCMV-Cre	F	17.7	33.4	4.6
*Yap*^*flox/flox*^*;Taz*^*flox/flox*^	Akt/NRas/pCMV-Cre	F	16	28.6	5.4
*Yap*^*flox/flox*^*;Taz*^*flox/flox*^	Akt/NRas/pCMV-Cre	F	24	30.8	3.3
*Yap*^*flox/flox*^*;Taz*^*flox/flox*^	Akt/NRas/pCMV-Cre	F	27.7	29	3.5
*Yap*^*flox/flox*^*;Taz*^*flox/flox*^	Akt/NRas/pCMV-Cre	M	10	29.8	1.3
*Yap*^*flox/flox*^*;Taz*^*flox/flox*^	Akt/NRas/pCMV-Cre	M	11	36.1	1.2
*Yap*^*flox/flox*^*;Taz*^*flox/flox*^	Akt/NRas/pCMV-Cre	M	17.5	47.8	1.8
*Yap*^*flox/flox*^*;Taz*^*flox/flox*^	Akt/NRas/pCMV-Cre	M	17.6	43.5	1.7
*Yap*^*flox/flox*^*;Taz*^*flox/flox*^	Akt/NRas/pCMV-Cre	M	22.5	35.4	3.3

BW, body weight; F, female; LW, liver weight; M, male; PI, postinjection.

**Figure 3:**
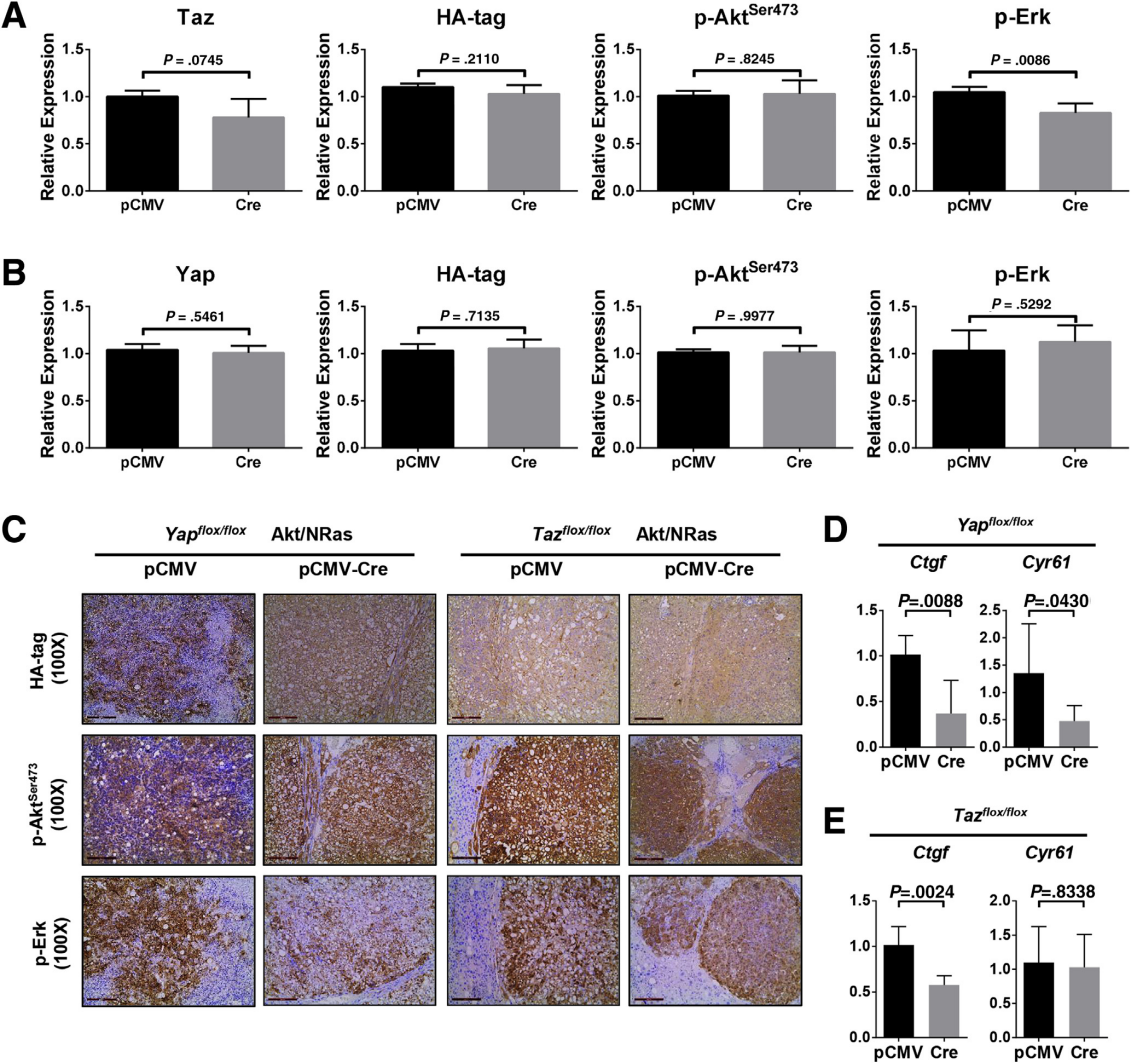
**Molecular biological analysis of Yap or Taz-depleted Akt/NRas liver tumors.** (*A*) Quantification of Taz, HA-tag, p-Akt^Ser473^, and p-Erk1/2 expression in *Yap*^*flox/flox*^ murine Akt/NRas/pCMV and Akt/NRas/pCMV-Cre liver tumors. (*B*) Quantification of Yap, HA-tag, p-Akt^Ser473^, and p-Erk1/2 expression in *Taz*^*flox/flox*^ murine Akt/NRas/pCMV and Akt/NRas/pCMV-Cre liver tumors. (*C*) Immunohistochemical staining pattern of HA-tagged Akt (*upper panel*), activation form of Akt (p-Akt^Ser473^; *middle panel*), and Ras downstream target p-Erk (*lower panel*) in *Yap*^*flox/flox*^ and *Taz*^*flox/flox*^ murine Akt/NRas liver tumors. *Scale bar*: 200 μm. (*D*) Expression of canonical Yap/Taz target genes, *Ctgf* and *Cyr61*, in Akt/NRas liver tumors developed in *Yap*^*flox/flox*^ or *Taz*^*flox/flox*^ conditional knockout mice.

**Figure 4:**
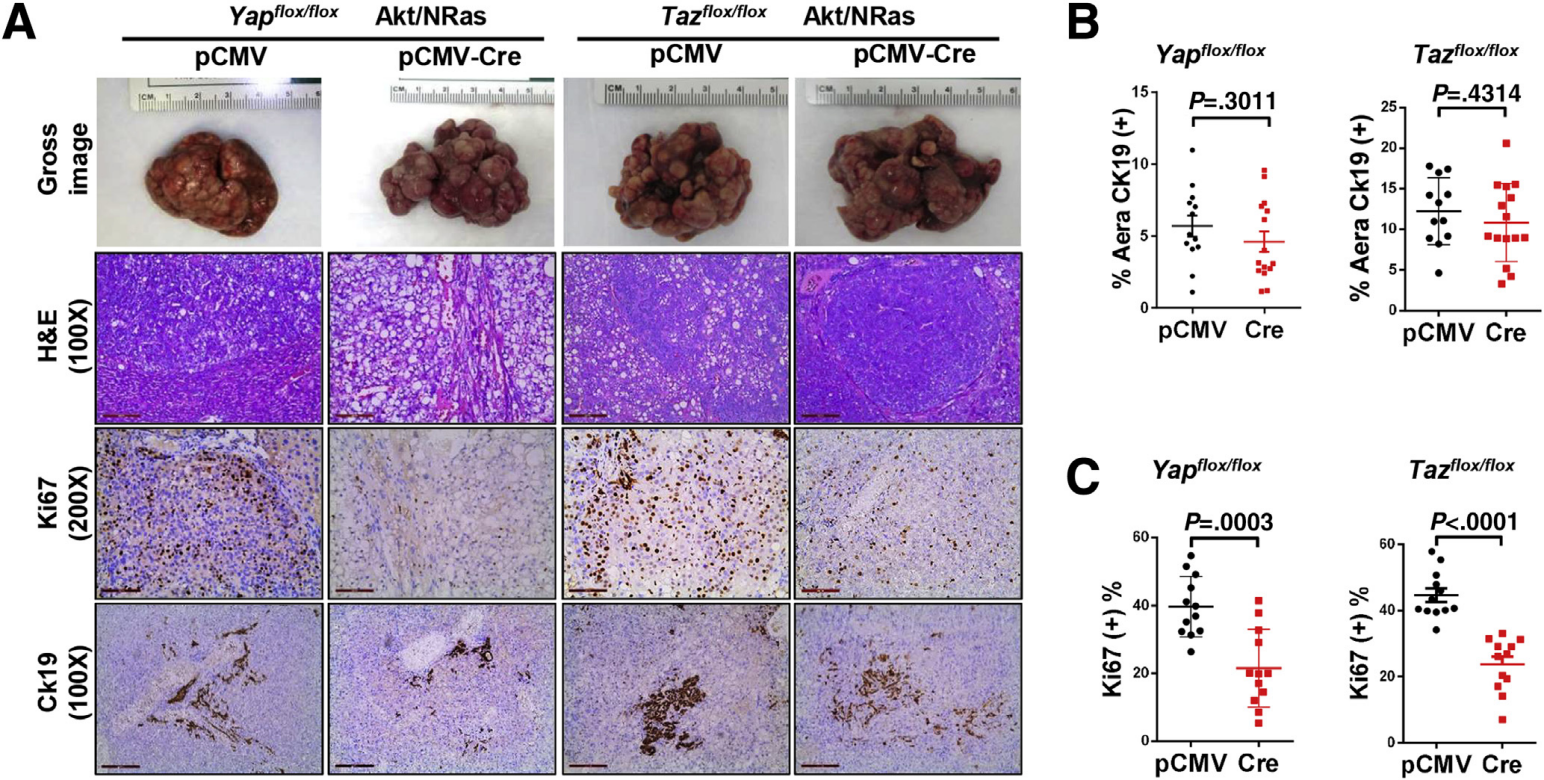
**Tumor features in Yap or Taz-depleted Akt/NRas murine livers.** (*A*) Representative images of gross images, H&E staining, and immunohistochemistry for Ki67 and cytokeratin 19 (Ck19). *Scalebar*: 200 μm for magnification of 100×, 100 μm for magnification of 200×. (*B*) Percentages of Ck19-positive area in the 2 groups. (*C*) Percentage of Ki67-positive cells in tumors.

Next, we analyzed the tumor tissues from pCMV and pCMV-Cre groups, using wild-type normal liver as a control. Effective depletion of Yap or Taz protein in the tumors from each model was validated using Western blot (Figure 2*E*). Furthermore, the tumors in *Yap KO* mice showed no immunoreactivity for Yap (Figure 2*F*). Altogether, the present data indicate that the tumors developed in a *Yap* or *Taz* KO background, and they were not escapers. Noticeably, ablation of Yap or Taz did not affect the expression of the other paralog. Similar expression levels of HA-tagged Akt and activated/phosphorylated Akt (p-Akt^Ser473^) also were observed. In addition, a slight decrease of Ras downstream kinases activated/phosphorylated phosphorylated extracellular regulated kinase 1/2 (p-Erk1/2) occurred in *Yap KO* Akt/NRas liver tumors, which was not observed in *Taz KO* Akt/NRas corresponding lesions (Figures 2*E* and 3*A* and *B*). These observations also were validated using immunohistochemistry (IHC) (Figure 3*C*). The knockout of Yap or Taz was accompanied by the decreased mRNA expression of canonical Yap/Taz targets, such as *Ctgf* and/or *Cyr61* genes (Figure 3*D* and *E*). At the histopathologic level, no major morphologic changes accompanied the knocking out of Yap or Taz, with the liver alterations consisting of both hepatocellular and cholangiocellular lesions as reported for Akt/NRas wild-type mice (Figure 4*A* and *B*).^9^ Proliferation of tumor cells was reduced after *Yap* or *Taz* depletion, as indicated by Ki67 immunostaining and quantification (Figure 4*A* and *C*).

Altogether, the present data suggest that ablation of *Yap* or *Taz* mildly delayed Akt/NRas-driven liver tumor development in vivo, leading to decreased tumor cell proliferation. The results indicate the overall redundant roles of Yap and Taz in this murine liver tumor model.

### Yap and Taz Are Required for Akt/NRas-Induced Mouse Hepatocarcinogenesis

**Figure 5:**
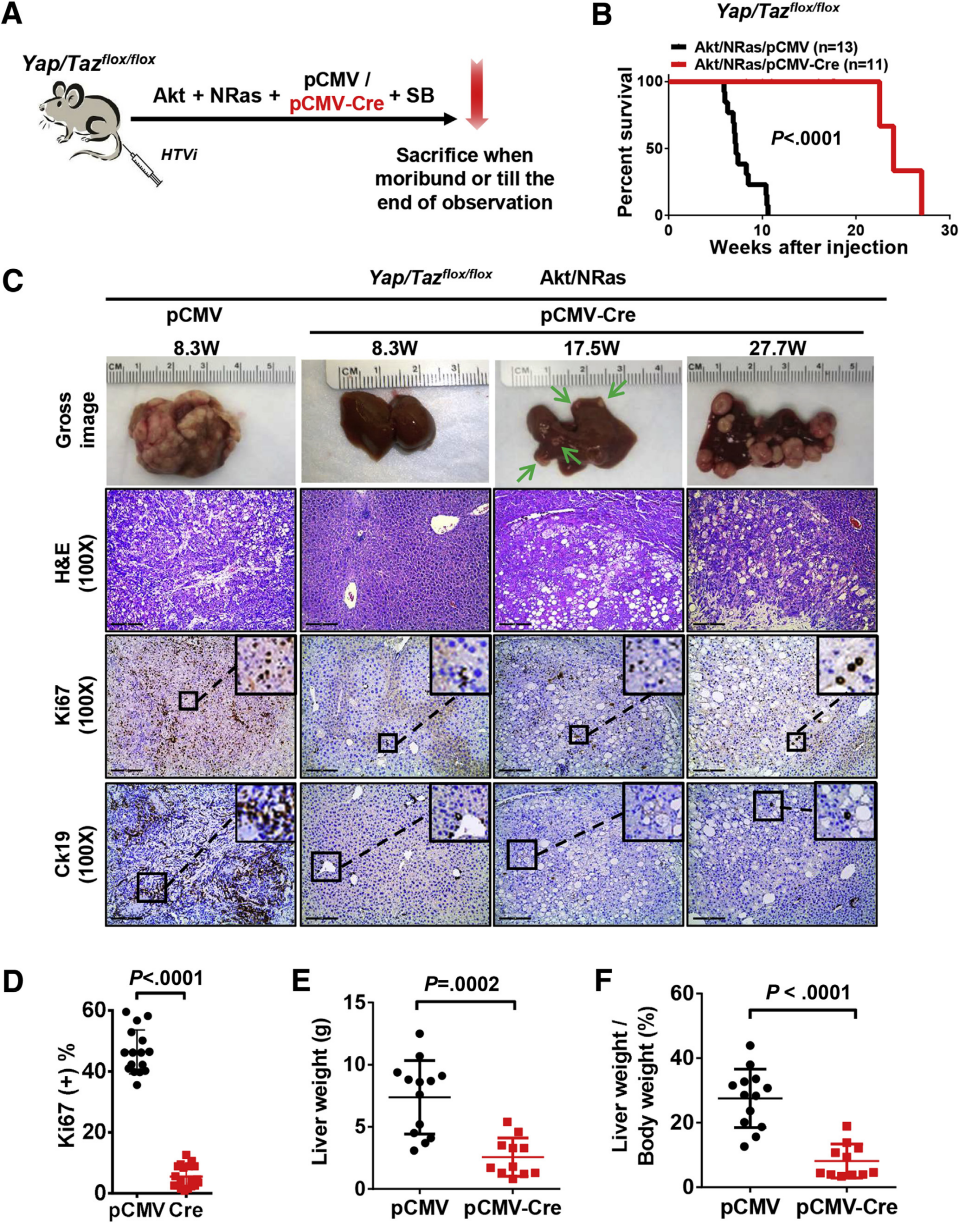
**Concomitant depletion of Yap and Taz significantly impairs Akt/NRas-induced murine hepatocarcinogenesis.** (*A*) Study design. *Yap/Taz*^*flox/flox*^ conditional KO mice were injected hydrodynamically with Akt/NRas/pCMV (control; N = 13) or Akt/NRas/pCMV-Cre (N = 11) plasmids. (*B*) Survival curves of *Yap/Taz*^*flox/flox*^ conditional KO mice injected with Akt and NRas. (*C*) Macroscopy comparisons of Akt/NRas liver tumor development in pCMV and pCMV-Cre groups (*upper row*), histopathologic features of the lesions (second row), Ki67 (third row), and Ck19 (fourth row) immunohistochemistry in both pCMV and pCMV-Cre groups. *Scale bar*: 200 μm for magnification of 100×, 100 μm for magnification of 200×. (*D*) Percentage of Ki67-positive cells in tumors. (*E* and *F*) Comparisons of (*E*) liver weight and (*F*) liver-weight-to-body-weight ratios in *Yap/Taz*^*flox/flox*^ murine Akt/NRas liver tumors.

Next, we tested the hypothesis that concomitant deletion of *Yap* and *Taz* would significantly suppress Akt/NRas-driven hepatocarcinogenesis by using *Yap;Taz* double KO mice in the Akt/NRas mouse liver cancer model. Specifically, *Yap/Taz*^*flox/flox*^ mice were injected with myr-Akt-HA-tagged and NRasV12 plasmids together with pCMV-Cre or control pCMV empty vector (Figure 5*A*). Remarkably, combined deletion of *Yap* and *Taz* strongly impaired Akt/NRas liver tumor development (Figure 5*B*). Indeed, mice had to be euthanized owing to lethal tumor burden at 8–10 weeks after injection in control Akt/NRas/pCMV mice, while only small tumor nodules could be observed until 17.5 weeks after injection in the *Yap;Taz* double KO (Akt/NRas/pCMV-Cre) group (Figure 5*C*). As expected, the liver weight and liver-weight-to-body-weight ratio decreased significantly in the pCMV-Cre group (Figure 5*E* and *F* and Table 2).

**Figure 6:**
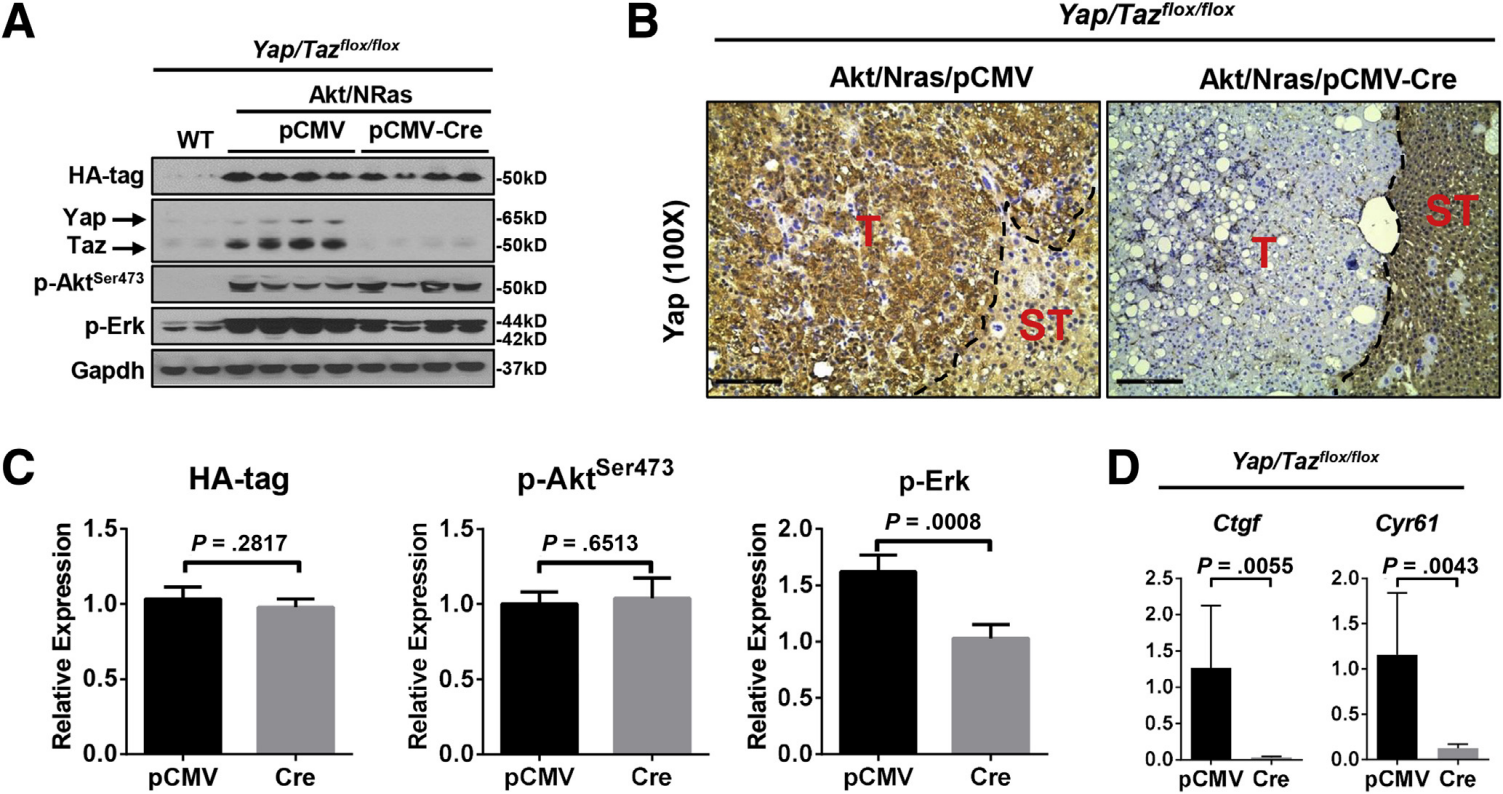
**Molecular biological analysis of Yap and Taz-depleted Akt/NRas liver tumors.** (*A*) Western blot analysis showing the expression levels of HA-tag, Yap/Taz, and activation of Akt and Ras pathways. Glyceraldehyde-3-phosphate dehydrogenase (Gapdh) was used as a loading control. (*B*) Immunohistochemical staining pattern of Yap in *Yap/Taz*^*flox/flox*^ murine Akt/NRas liver tumors. *Scale bar*: 100 μm. (*C*) Quantification of HA-tag, p-Akt^Ser473^, and p-Erk1/2 expression in *Yap/Taz*^*flox/flox*^ murine Akt/NRas/pCMV and Akt/NRas/pCMV-Cre liver tumors. (*D*) Expression of *Ctgf* and *Cyr61* in Akt/NRas liver tumors developed in *Yap/Taz*^*flox/flox*^ conditional knockout mice. T, tumor; ST, surrounding tissues.

In addition, concomitant deletion of *Yap* and *Taz* resulted in the most significant proliferation restraint, as indicated by Ki67 staining (Figure 5*C*). The slowly growing lesions of Akt/NRas in *Yap;Taz* double KO mice were exclusively pure hepatocellular lesions (Figure 5*C*), lacking cytokeratin 19 (Ck19) (+) cholangiocellular-like tumors, thus recapitulating the phenotype of co-expressing Akt/NRas and large tumor suppressor kinase 2 (Last2) in the mouse liver.^9^ Cell proliferation was reduced significantly in *Yap;Taz* double KO Akt/NRas tumors, as measured by Ki67+ percentages (Figure 5*D*). Depletion of Yap and Taz among tumor tissues was confirmed by Western blot analysis (Figure 6*A*). Deletion of Yap also was validated by IHC, which showed exclusively Yap depletion in the tumor lesions (Figure 6*B*). Similar expression levels of HA-tag and activation form of Akt (p-Akt^Ser473^) were observed, indicating the stable activation of Akt in all tumor lesions. Consistent with that observed in *Yap* KO tumors, levels of Ras downstream effectors p-Erk1/2 were decreased in *Yap;Taz* double KO tumors (Figure 6*C*). Intriguingly, mRNA expression of canonical Yap/Taz targets, *Ctgf* and *Cyr61* genes, were almost annulled after the concomitant depletion of Yap and Taz (Figure 6*D*).

Altogether, our study shows that although Yap or Taz alone has limited roles in regulating Akt/NRas-driven liver tumor formation, simultaneous deletion of *Yap* and *Taz* significantly impaired tumorigenesis in this model.

### YAP and TAZ Coordinately Regulate Cell Proliferation in Human HCC Cell Lines

**Figure 7:**
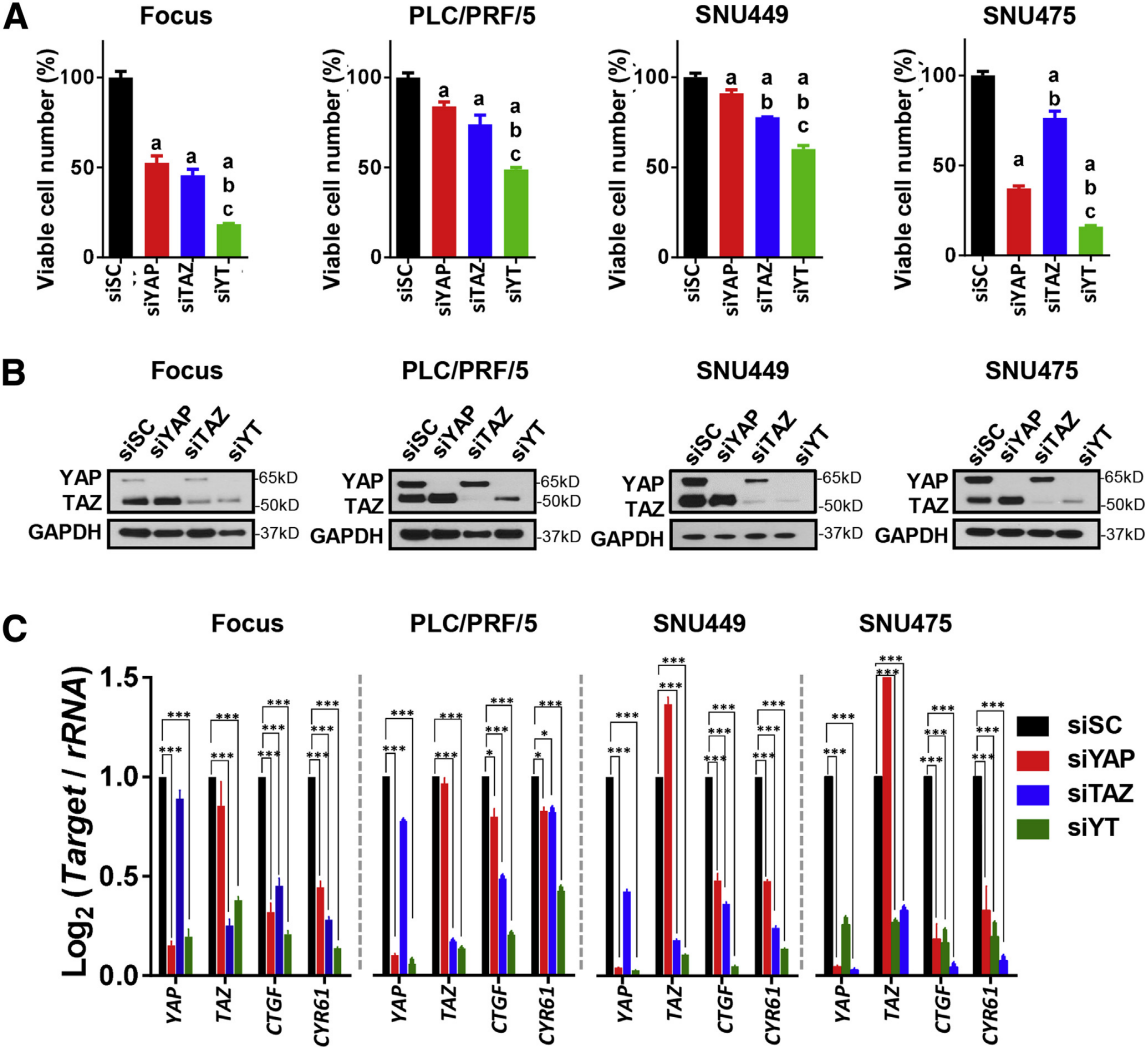
**YAP and TAZ coordinately regulate HCC cell proliferation in vitro.** (*A*) Focus, PLC/PRF/5, SNU 449, and SNU 475 human HCC cell lines were transfected with siSC, si*YAP*, si*TAZ*, and si*YAP*, and si*TAZ* (siYT) for 72 hours. Crystal violet assay was used to determine the number of viable cells. Data are presented as the percentage to siSC group in each HCC cell line respectively. The results show that inhibition of YAP or/and TAZ reduces cell proliferation. *P* < .05 (a) vs siSC, (b) vs si*YAP*, (c) vs si*TAZ*. (*B*) Expression of YAP and TAZ was detected by Western blot after treatment. (*C*) mRNA expression of *YAP*, *TAZ*, *CTGF*, and *CYR61*.

Next, to explore the potential functional contribution of YAP and TAZ in vitro, we applied human HCC cell lines for comparison. Specifically, 4 human HCC cell lines (Focus, PLC/PRF/5, SNU449, and SNU475) were transfected with small interfering RNAs (siRNAs) against *YAP* and *TAZ*, either alone or in combination (which are referred to as si*YAP*, si*TAZ*, and si*YT*), as well as scrambled siRNA (siSC) as control. Similar to the in vivo data, silencing of *YAP* and/or *TAZ* resulted in cell proliferation constraint in all 4 human HCC cell lines. Notably, this growth-inhibitory effect showed a tendency to be cell-type–dependent. For instance, silencing of *YAP* contributed to cell proliferation inhibition in SNU475 more significantly. However, si*YT* contributed to the most significant inhibition readouts (Figure 7*A*). Depletion of YAP and/or TAZ was confirmed further by Western blot. Importantly, a slight up-regulation of TAZ levels was observed among si*YAP*-treated samples when compared with control-treated (siSC) samples in each cell line (Figure 7*B*), implying the potential existence of compensational mechanisms between YAP and TAZ. Consistently, down-regulation of *YAP* and/or *TAZ*, as well as *CTGF* and *CYR61* mRNAs, also were detected (Figure 7*C*).

Collectively, YAP and TAZ coordinately regulate cell growth in vitro and repression of YAP and TAZ concurrently leads to the most significant cell growth restraint. The results are consistent with the findings in the Akt/NRas mouse liver tumor model.

### Redundant Roles of YAP and TAZ in Regulating Cell Cycle and DNA Replication

**Figure 8:**
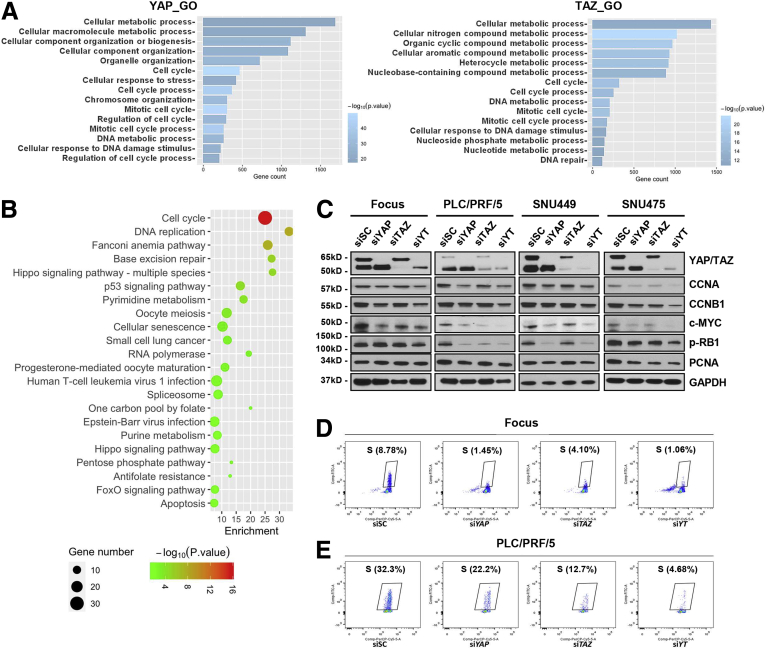
**YAP and TAZ regulate cell cycle and DNA replication during hepatocarcinogenesis.** (*A*) Gene Ontology (GO) analysis of *YAP* and *TAZ* correlated genes from the TCGA Liver Hepatocellular Carcinoma (LIHC) data set. (*B*) KEGG analysis of differentially expressed genes in *YAP* and *TAZ* concomitantly silenced (si*YT*) HCC cell lines. Ranked by *P* value. (*C*) Western blot analysis showing the level changes of cell-cycle–related proteins after silencing of YAP (si*YAP*), TAZ (si*TAZ*), and the 2 genes combined (si*YT*). Glyceraldehyde-3-phosphate dehydrogenase (GAPDH) was used as a loading control. (*D* and *E*) Enhanced cell-cycle arrest in the (*D*) Focus and (*E*) PLC/PRF/5 human HCC cell lines treated with specific siRNA against *YAP* and/or *TAZ* (si*YAP* and/or si*TAZ*). The percentages of cells in the S phase are shown. FoxO, Forkhead box subfamily O.

To investigate the potential mechanisms of YAP and TAZ in regulating cell proliferation during hepatocarcinogenesis, we first retrieved genes that correlated positively with *YAP* or *TAZ* mRNA expression from the The Cancer Genome Atlas (TCGA) data set (Supplementary Table 1). The top 2500 genes ranked by the Spearman correlation score were subjected to gene ontology analysis (Figure 8*A*). We found that genes that correlated positively with *YAP* or *TAZ* mRNA expression shared overall similar biological processes, such as cellular metabolic processes, cell cycle, and mitotic cell cycle (Figure 8*A*).

**Figure 9:**
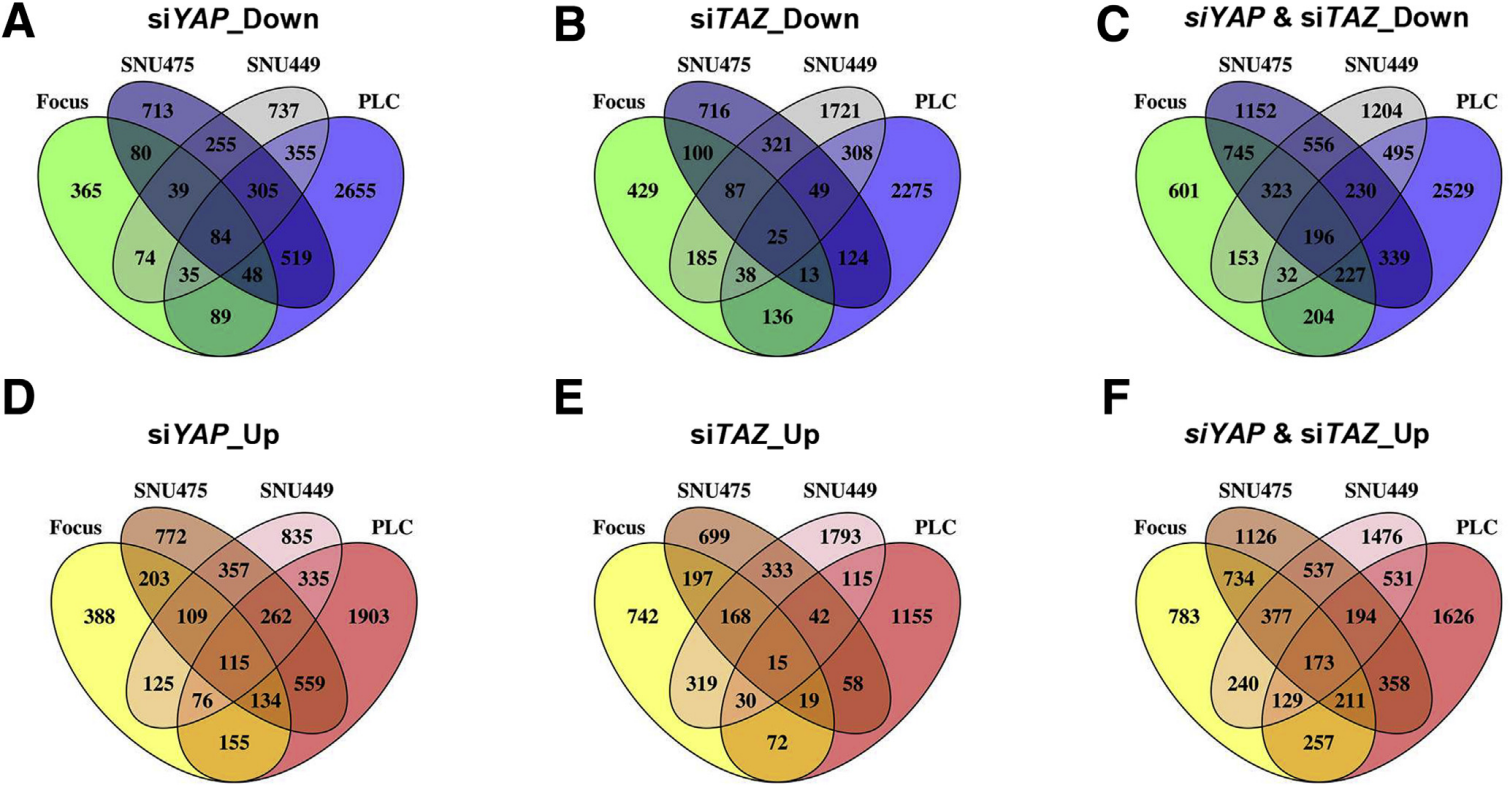
**Venn graphs showing differentially expressed genes after silencing *YAP* and/or *TAZ* in human HCC cell lines.** (*A–C*) Down-regulated genes in si*YAP*-treated, si*TAZ*-treated, and si*YAP*- and si*TAZ*-treated HCC cell lines. (*D–F*) Up-regulated genes in si*YAP*-treated, si*TAZ*-treated, and si*YAP*- and si*TAZ*-treated HCC cell lines.

Subsequently, RNA sequencing was used for global expression analysis of cells subjected to *YAP* and *TAZ* silencing. Specifically, the aforementioned 4 human HCC cell lines treated with siSC, si*YAP*, si*TAZ*, or si*YT* were subject to RNA sequencing. We first discerned clusters of differentially up-regulated (log2 fold change, >1.5; *P* adj < .05) and down-regulated (log2 fold change, < −1.5; *P* adj < .05) genes in each cell line. As expected, deregulated genes shared both overlapping and distinct patterns across the 4 HCC cell lines (Figure 9 and Supplementary Table 2). For better representation, the same genes that were deregulated in at least 3 cell lines were output for Kyoto Encyclopedia of Genes and Genomes (KEGG) analysis. Differentially expressed genes regulated by YAP or TAZ shared a highly similar functional clustering, such as cell cycle and DNA replication (Figure 8*B*). Moreover, expression of cell-cycle–related proteins (CCNA, CCNB1, c-MYC, pRB-1, and PCNA), tested by Western blot (Figure 8*C*), were showed to be commonly decreased when YAP or TAZ expression was inhibited. Furthermore, bromodeoxyuridine (BrdU) incorporation significantly decreased in Focus and PLC/PRF/5 cell lines when treated with siRNAs against *YAP* or *TAZ*, indicating the inhibition of cell-cycle progression and mitosis (Figures 8*D* and *E*).

**Figure 10:**
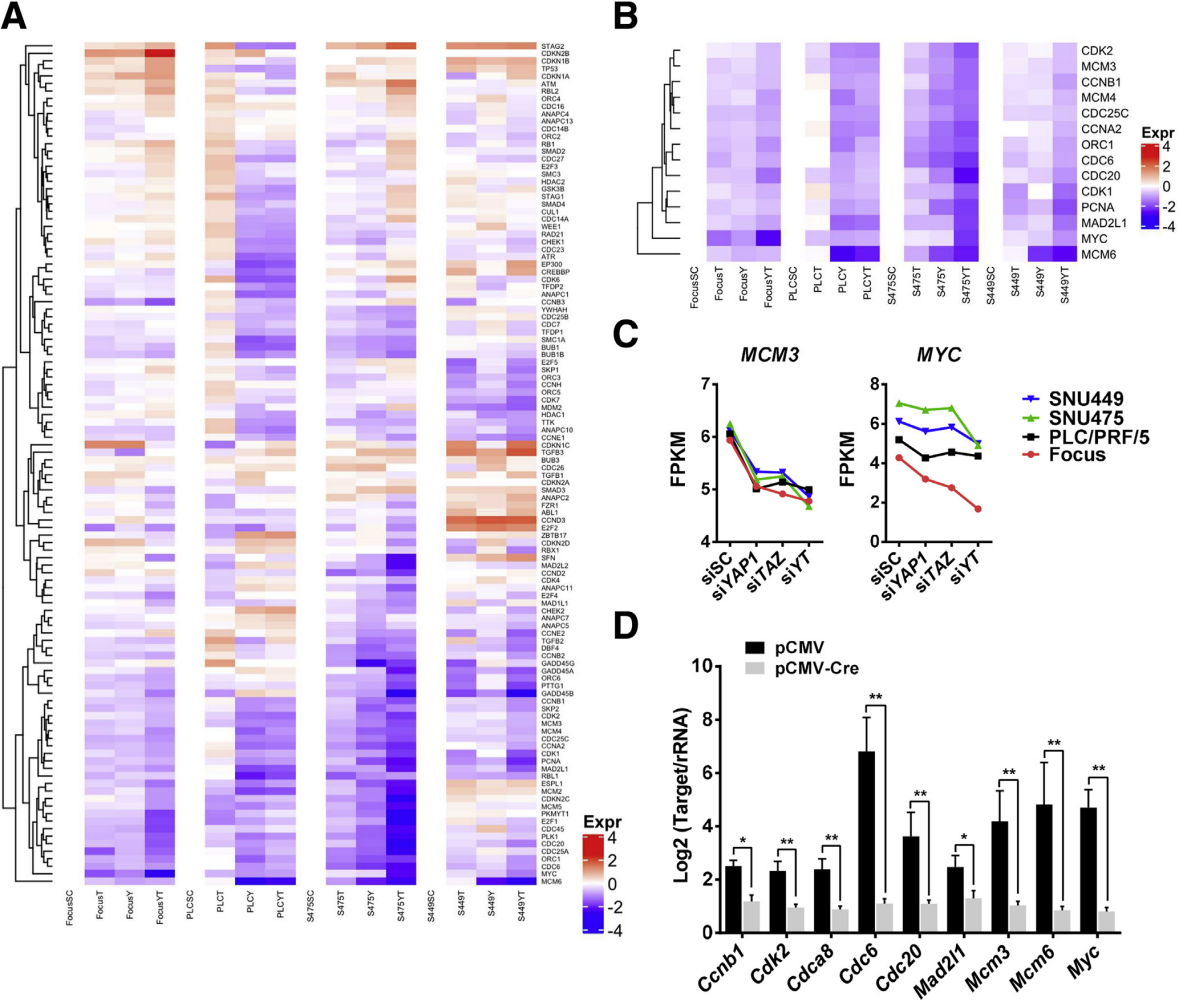
**Functional redundancy of YAP and TAZ in regulating cell cycle and DNA replication–related genes.** (*A*) Heatmap showing differential expression of cell-cycle–related genes after silencing of YAP (si*YAP*), TAZ (si*TAZ*), and the 2 genes combined (si*YT*). (*B*) Heatmap showing the normalized expression of DNA replication signature genes (normalized with siSC). (*C*) Fragments per kilobase of transcript per million mapped reads (FPKM) results of Minichromosome maintenance complex component 3 (MCM3) and c-MYC expression. (*D*) Expression of DNA replication signature genes in *Yap;Taz* double KO murine Akt/NRas liver cancer tissues. ∗*P* < .05, ∗∗*P* < .01.

**Figure 11:**
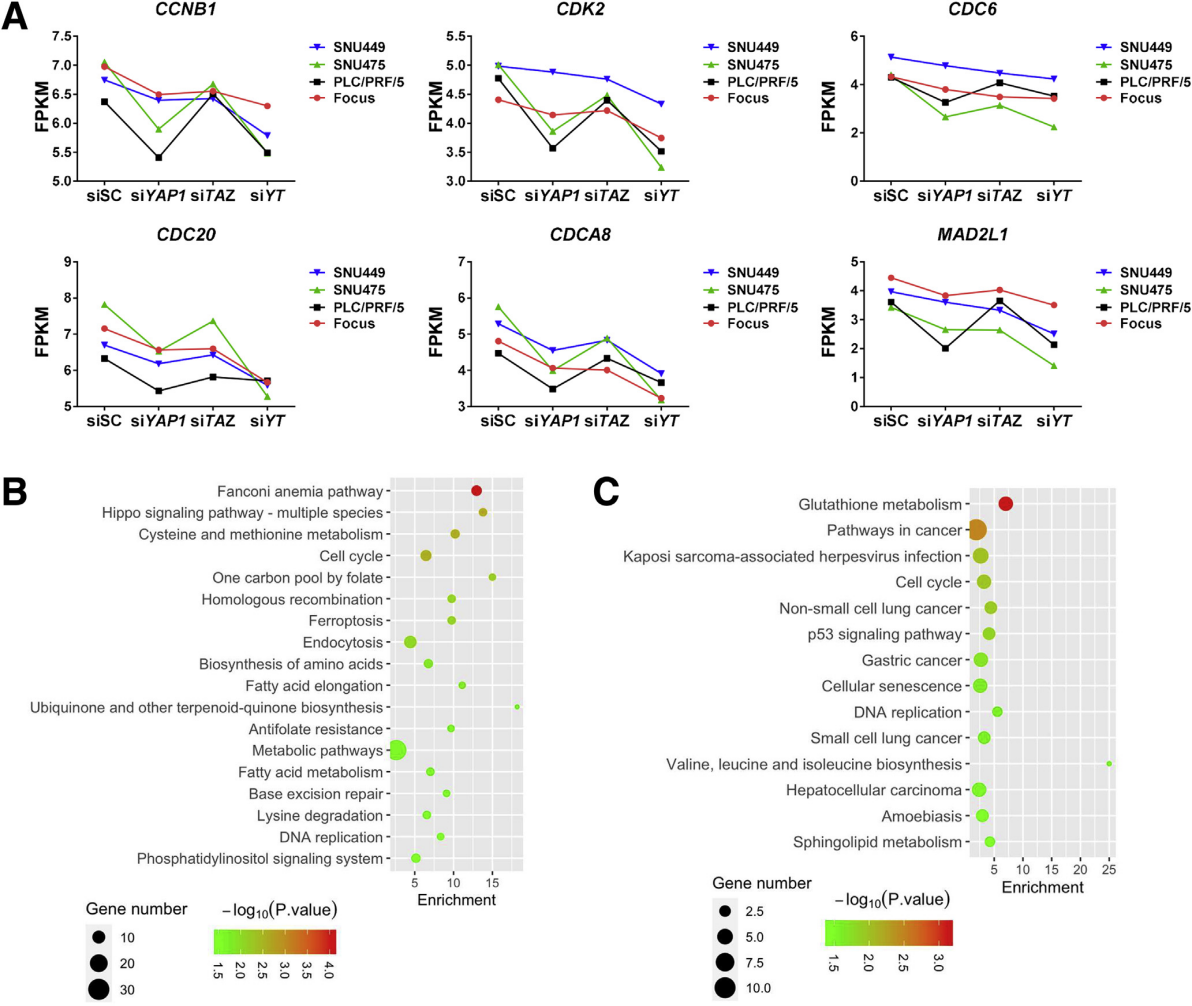
**Overlapping and distinct transcriptome profiles of YAP and TAZ during hepatocarcinogenesis.** (*A*) Fragments per kilobase of transcript per million mapped reads (FPKM) results of DNA replication–related genes (*CCNB1*, *CDK2*, *CDC6*, *CDC20*, *CDCA8*, and *MAD2L1*) expression in si*YAP*-treated, si*TAZ*-treated, and si*YAP*- and si*TAZ*-treated HCC cell lines. (*B* and *C*) KEGG analysis results of genes that were found to be down-regulated in at least 3 cell lines after silencing of (*B*) *YAP* or (*C*) *TAZ*.

Further analysis indicated that variations in genomic profiles and biological activities related to cell cycle and mitosis processes were coordinately regulated by YAP and TAZ. Therefore, the data suggest that the functional contribution of YAP and TAZ in modulating the cell cycle is transcriptionally redundant (Figure 10*A*). This functional redundancy was even more evident when further analyzing the expression patterns of the DNA replication gene signature.^6^^,^^10^ Inhibition of *YAP* or *TAZ*, either alone or in combination, led to significant down-regulation of the chromosomal instability signature genes, including *MCM3*, *MYC*, *CCNB1*, *CDK2*, *CDC6*, *CDC20*, *CDCA8*, and *MAD2L1* (Figures 10*B* and *C* and 11*A*). The results were consistent with those from mouse models, in which ablation of *Yap* and/or *Taz* consistently led to decreased tumor cell proliferation in vivo. Furthermore, all of these genes also were down-regulated significantly in Akt/NRas tumors in *Yap;Taz* double KO mice (Figure 10*D*).

Altogether, YAP and TAZ coordinately regulate cell cycle and DNA replication at the transcriptional level. Our study, therefore, provides strong evidence that Hippo cascade and its downstream effectors YAP and TAZ are major regulators of tumor cell proliferation during hepatocarcinogenesis.

### Identification of Putative YAP- and TAZ-Specific Targets in HCC

Our results also suggested that YAP and TAZ might have distinct targets. The KEGG analyses showed specific pathways that are regulated by YAP or TAZ. For instance, silencing of YAP led to the deregulated fatty acid metabolism and metabolic pathways, whereas knockdown of TAZ resulted in the aberrant expression of genes involved in glutathione metabolism (Figure 11*B* and *C*).

**Figure 12:**
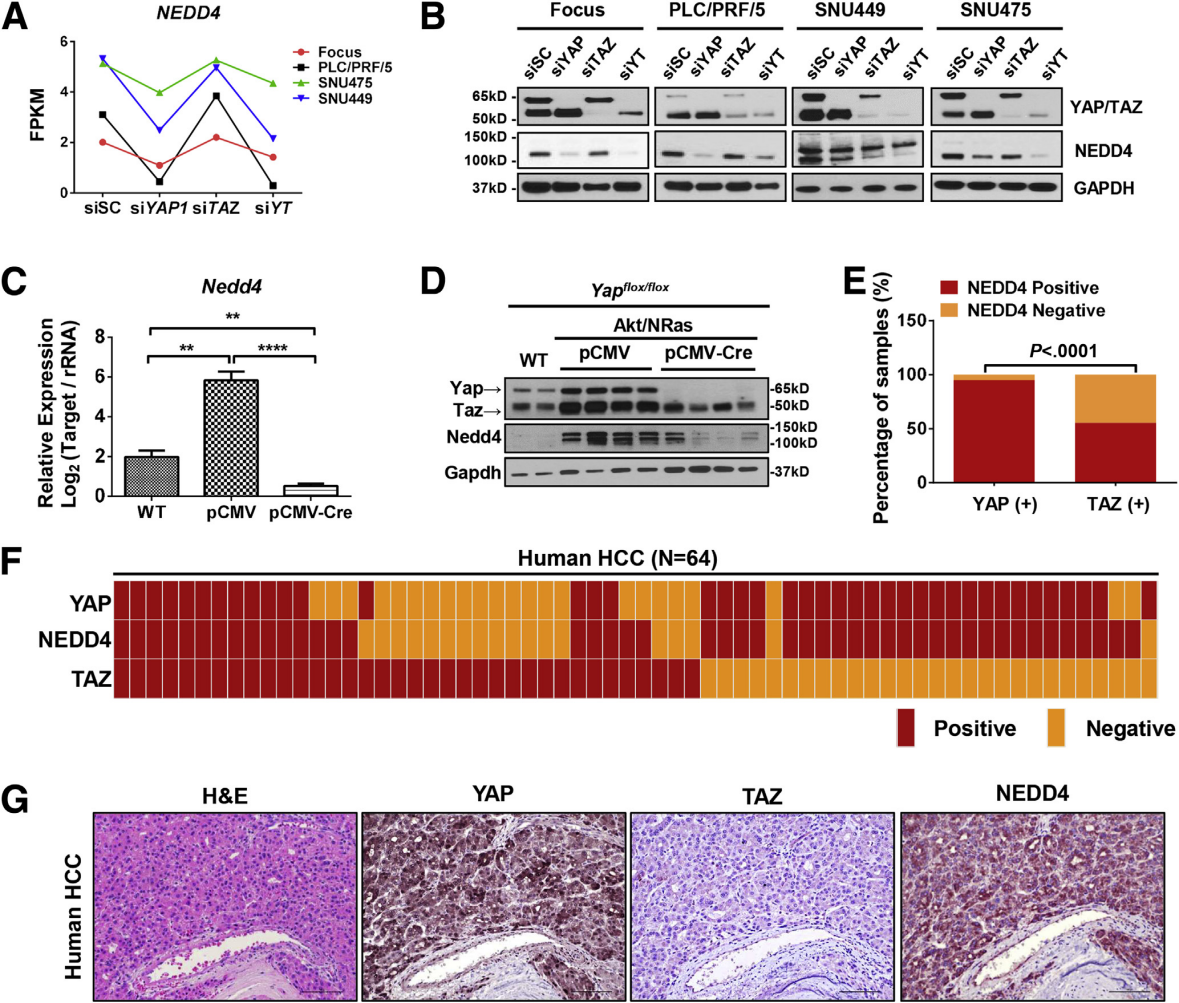
**NEDD4 is a potential YAP target in HCC.** (*A*) Fragments per kilobase of transcript per million mapped reads (FPKM) results of *NEDD4* expression in si*YAP*-treated, si*TAZ*-treated, and si*YAP*- and si*TAZ*-treated HCC cell lines. (*B*) Western blot results of YAP, TAZ, and NEDD4 expression in si*YAP*-treated, si*TAZ*-treated, and si*YAP*- and si*TAZ*-treated HCC cell lines. Glyceraldehyde-3-phosphate dehydrogenase (GAPDH) was used as a loading control. (*C*) Real-time qPCR results showing *Nedd4* mRNA expression in *Yap*^*flox/flox*^ wild-type normal liver tissues (WT) and Akt/NRas/pCMV (pCMV) or Akt/NRas/pCMV-Cre (pCMV-Cre) murine liver tumors. ∗∗*P* < .01, ∗∗∗∗*P* < .0001. (*D*) Western blot results of Yap/Taz and Nedd4 expression in wild-type normal liver tissues (WT) and Akt/NRas liver tumors developed in *Yap*^*flox/flox*^ conditional KO mice. Gapdh was used as a loading control. (*E*) Comparison of NEDD4-positive and -negative immunoreactivity frequency between YAP-positive (YAP [+]) and TAZ-positive (TAZ [+]) human HCC samples. The Fisher exact test was applied, *P* < .0001. (*F*) Heatmap of nuclear YAP, nuclear TAZ, and NEDD4 staining patterns in human HCC samples (N = 64). (*G*) Representative immunohistochemical pattern of YAP, TAZ, and NEDD4 staining in a serial section from 1 human HCC. A strong immunoreactivity for nuclear and cytoplasmic YAP, as well as for cytoplasmic NEDD4, is evident, whereas low cytoplasmic immunolabeling for TAZ can be appreciated. Original magnification: 200×. *Scale bar*: 100 μm.

Intriguingly, we noticed that YAP may be involved in the regulation of endocytosis, in which E3 ubiquitin-protein ligase neural precursor cell expressed, developmentally down-regulated protein 4 (NEDD4) is a main component.^11^ NEDD4 has been shown to negatively regulate the stability of WW45 and LATS kinases, leading to alterations in cell proliferation.^12^ *NEDD4* mRNA was specifically down-regulated after knocking down *Yap* in all HCC cell lines and Akt//NRas murine liver tumor tissues (Figure 12*A* and *B*). Consistently, NEDD4 protein levels also were decreased upon YAP deletion (Figure 12*C* and *D*). Next, we analyzed NEDD4 expression among the same collection of human HCC samples (Figure 1) using IHC. We found that 39 of 41 (95.1%) YAP-positive samples also were positive for NEDD4, whereas only 20 of 36 (55.6%) TAZ-positive samples were positive for NEDD4 (*P* < .0001) (Figures 12*E–G*), suggesting that NEDD4 may be a direct target of YAP in HCC.

**Figure 13:**
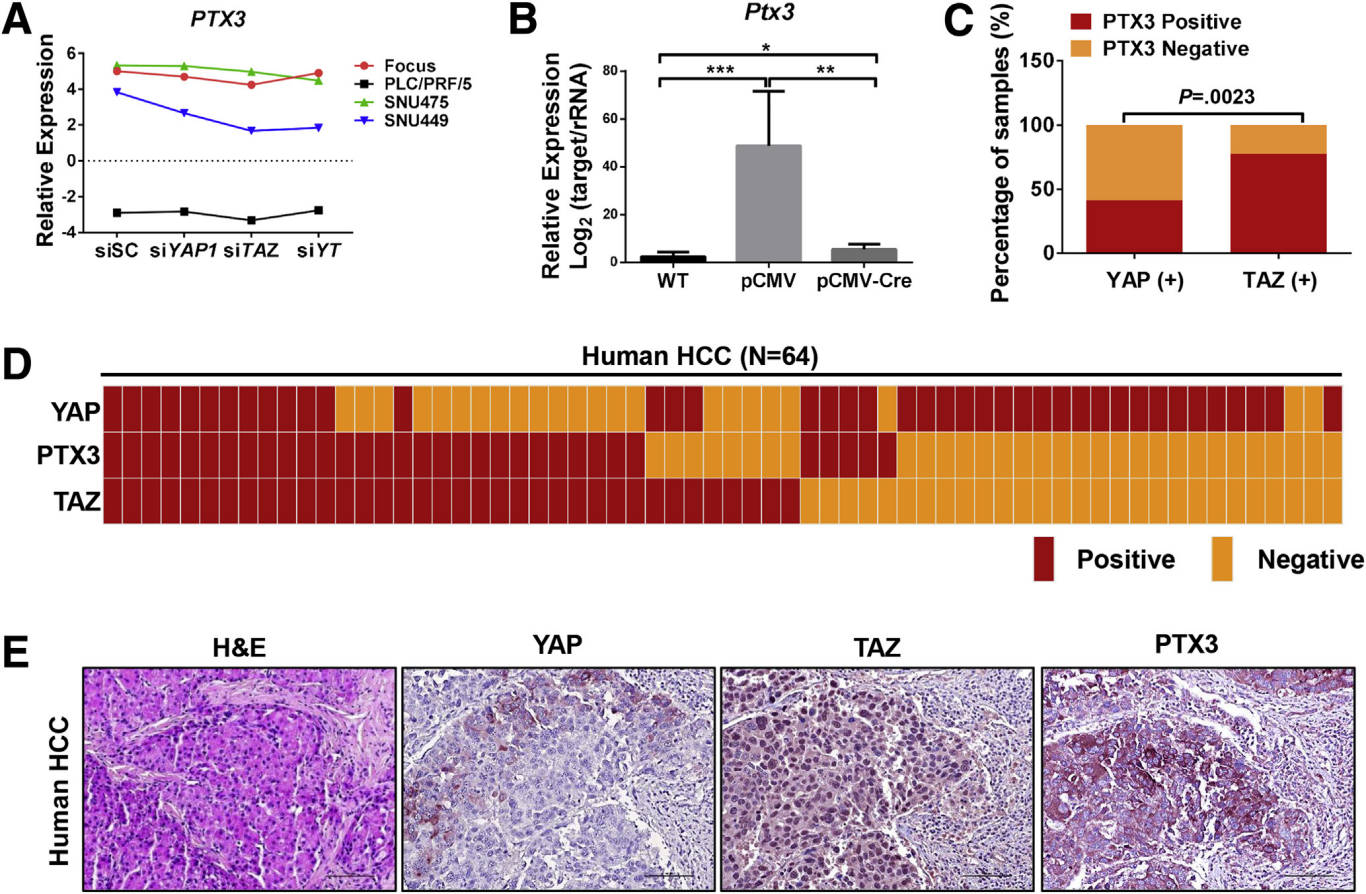
**PTX3 is a potential target of TAZ in HCC.** (*A*) Fragments per kilobase of transcript per million mapped reads results of *PTX3* expression in si*YAP*-treated, si*TAZ*-treated, and si*YAP*- and si*TAZ*-treated HCC cell lines. (*B*) Real-time qPCR results showing *Nedd4* mRNA expression in *Taz*^*flox/flox*^ wild-type normal liver tissues (WT) and Akt/NRas/pCMV (pCMV) or Akt/NRas/pCMV-Cre (pCMV-Cre) murine liver tumors. ∗*P* < .05, ∗∗*P* < .01, ∗∗∗*P* < .0001. (*C*) Comparison of PTX3-positive and -negative immunoreactivity frequency between YAP-positive (YAP [+]) and TAZ-positive (TAZ [+]) human HCC samples. The Fisher exact test was applied, *P* = .0023. (*D*) Heatmap of nuclear TAZ staining and PTX3 staining patterns in human HCC samples (N = 64). (*E*) Representative immunohistochemical pattern of YAP, TAZ, and PTX3 staining in a serial section from 1 human HCC. A strong immunoreactivity for nuclear and cytoplasmic TAZ as well as for cytoplasmic PTX3 is appreciable, whereas faint and scattered cytoplasmic immunolabeling for YAP is present. Original magnification: 200×. *Scale bar*: 100 μm.

**Figure 14:**
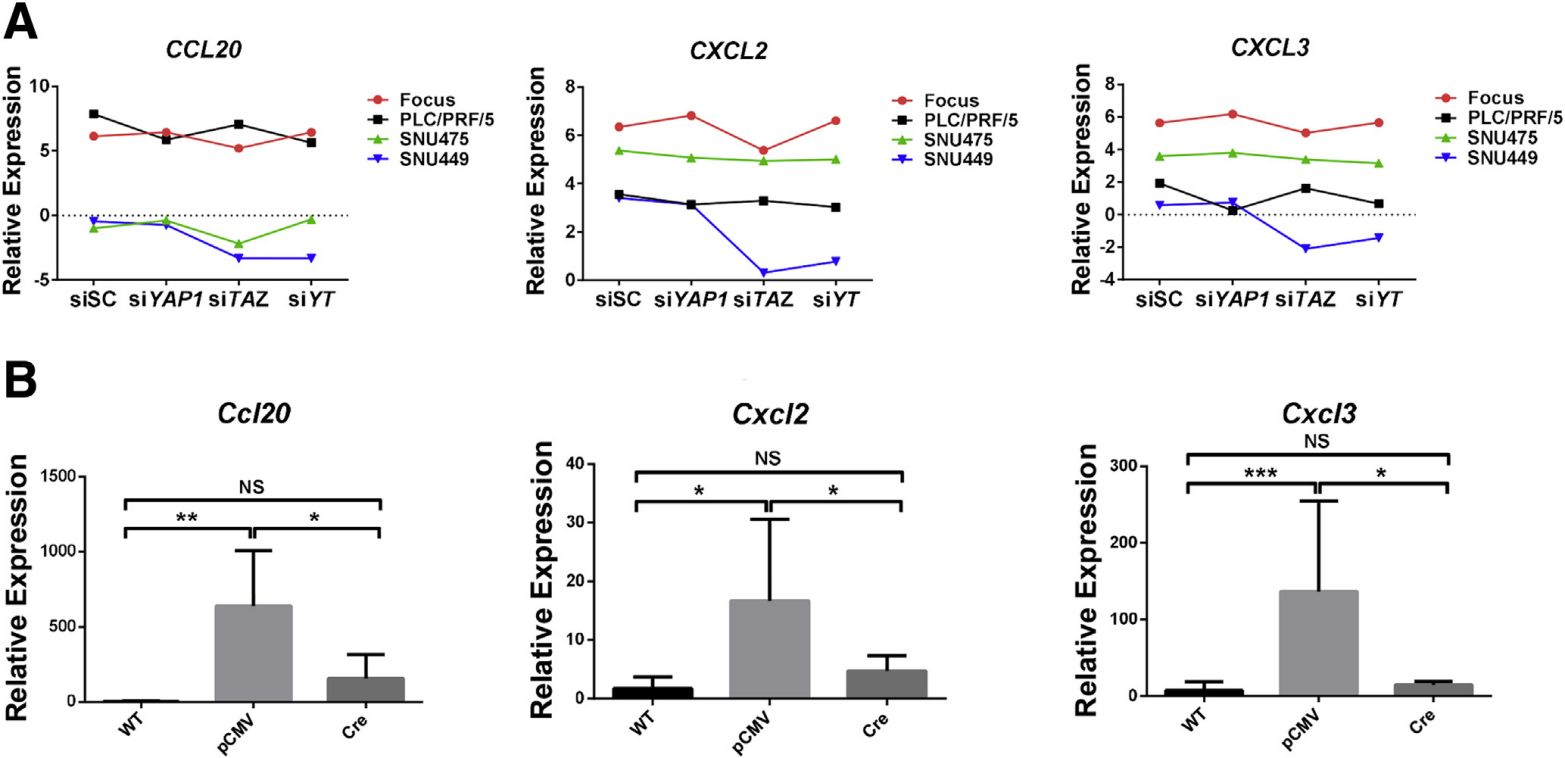
**TAZ is associated with expression of inflammatory-associated genes during hepatocarcinogenesis.** (*A*) Fragments per kilobase of transcript per million mapped read results of *CCL20*, *CXCL2*, and *CXCL3* expression in si*YAP*-treated, si*TAZ*-treated, and si*YAP*- and si*TAZ*-treated HCC cell lines. (*B*) Real-time qPCR results showing *Ccl20*, *Cxcl2*, and *Cxcl3* mRNA expression in wild-type normal liver tissues (WT) and Akt/NRas liver tumors developed in *Taz*^*flox/flox*^ conditional KO mice. ∗*P* < .05, ∗∗*P* < .01, and ∗∗∗*P* < .0001.

TAZ has been reported to be associated with inflammation response and tissue infiltration during hepatocarcinogenesis.^13^ Accordingly, we found that the levels of inflammatory-related genes, including *PTX3*, *CCL20*, *CXCL2*, and *CXCL3*, were specifically down-regulated after knocking down TAZ in most HCC cell lines as well as in *Taz* KO Akt/NRas murine liver tumor tissues (Figures 13*A* and *B* and 14). In human HCCs, 28 of 36 (77.8%) TAZ-positive samples also were positive for pentraxin 3 (PTX3) IHC staining (Figure 13), whereas only 17 of 41 (41.5%) YAP-positive samples showed PTX3-positive immunoreactivity (*P* = .0023) (Figure 13*C–E*). The results suggest that PTX3 may be a major target of TAZ in HCC.

Altogether, our results indicate that YAP and TAZ regulate both overlapping and distinct gene expression programs in HCC. NEDD4 might be a putative target regulated by YAP, whereas PTX3 might be a specific TAZ target.

## Discussion

Hippo signaling downstream co-activators YAP and TAZ are master regulators of oncogenic events in human hepatocarcinogenesis. Although multiple studies investigated the functional role(s) of YAP and TAZ in HCC,^14^ whether these 2 proteins have overlapping or distinct roles in liver cancer remains unclear. In the current study, we attempted to address this fundamental question using human HCC samples and murine HCC models. A major conclusion of the investigation is the discovery of the almost ubiquitous inactivation of the Hippo cascade, leading to the nuclear localization and activation of YAP and/or TAZ in human HCC samples. This observation was validated in various mouse HCC models, such as liver tumors induced by diethylnitrosamine^15^ and Akt/NRas oncogenes.^9^ Altogether, this concordant body of evidence supports the inactivation of the Hippo pathway as a major signaling event in hepatocarcinogenesis.

Another important finding from this investigation was the imbalance of YAP and TAZ activation in most human HCC samples because approximately 70% of HCCs showed only nuclear YAP or TAZ immunoreactivity. The activation of YAP and TAZ in human HCCs has been reported by several other studies.^16^^,^^17^ However, none of these studies provided a comprehensive description of the percentage of HCCs with unique YAP or TAZ activation. Clearly, the next step is to investigate the mechanisms whereby YAP or TAZ are differentially activated during hepatocarcinogenesis. For instance, AKT has been reported to specifically phosphorylate YAP at site Ser127, leading to the inhibition of p73-mediated apoptosis and cell death.^18^ A previous study showed that RAS activation lead to increased YAP activity via phosphorylation of Ajuba proteins, which inactivate LATS kinases.^19^ In addition, protein phosphatase 1A has been characterized as a bona fide phosphatase of TAZ by decreasing its interaction with β-transducin repeat containing E3 ubiquitin protein ligase (β-TrCP),^20^ although protein phosphatase 1A was found to stabilize chromatin-bound MYC in proliferating cells,^21^ suggesting a potential protein–protein link between c-MYC and TAZ via protein phosphatase 1A. Additional studies are required to elucidate the precise molecular mechanisms responsible for the differential activation of YAP and TAZ along liver tumorigenesis.

When investigating the functional contribution of Yap and/or Taz in regulating Akt/NRas-driven hepatocarcinogenesis using conditional *Yap*, *Taz*, or *Yap/Taz* double KO mice, we discovered that Yap and Taz share redundant roles in regulating tumor cell proliferation and cholangiocellular-like tumor formation in Akt/NRas mice. The latter phenotype likely is owing to the fact that both Yap and Taz are able to induce Notch signaling.^9^ Previously, we have shown that activation of Lats2 in Akt/NRas murine liver tumors resulted in delayed hepatocarcinogenesis and the elimination of intrahepatic cholangiocarcinoma (ICC)-like lesions in the liver.^9^ Here, we show that only loss of both *Yap* and *Taz* is able to recapitulate this phenotype. Thus, YAP and TAZ might be functionally redundant in regulating the differentiation status of liver tumors. The contribution of YAP in promoting ICC development has been investigated extensively.^22^ Studies have shown the key role of the NOTCH cascade downstream of YAP in ICCs.^23^ In contrast, few studies of the functional role of TAZ in cholangiocarcinogenesis have been conducted to date.^24^ Additional studies are required to address the biochemical cross-talk between YAP/TAZ and NOTCH cascades in ICCs.

Moreover, we found that deletion of *Yap* or concomitant deletion of *Yap* and *Taz* led to decreased p-Erk in Akt/NRas liver tumors (Figures 2*E* and 6*A*), whereas ablation of Taz alone did not affect p-Erk levels (Figure 2*E*). The data suggest that Yap, but not Taz, may control the activation of MAPK signaling downstream of activated Ras in cancer. Multiple studies have suggested a molecular cross-talk between MAPK and Hippo signaling cascades during tumor development.^25^ However, the specific biochemical interplay between Yap and MAPK pathways during hepatocarcinogenesis has not been characterized satisfactorily. It would be of high importance to determine the molecular mechanisms whereby YAP, but not TAZ, modulates p-ERK activity in HCC.

**Figure 15:**
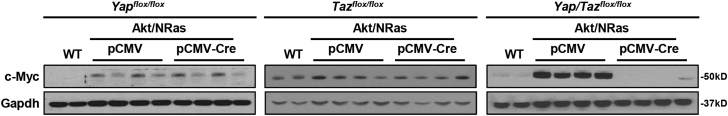
**c-MYC is a potential target coordinately regulated by YAP and TAZ. Western blot results showing expression of c-Myc in *Yap***^***flox/flox***^**, *Taz***^***flox/flox***^**, and *Yap/Taz***^***flox/flox***^**murine Akt/NRas liver tumors.** Glyceraldehyde-3-phosphate dehydrogenase (Gapdh) was used as a loading control.

At the transcriptional level, we showed that YAP and TAZ coordinately regulated the cell cycle and DNA replication through bioinformatic analysis of TCGA liver hepatocellular carcinoma (LIHC) sequence results^26^ and our RNA sequencing data. Among the potential targets of YAP and TAZ, MCM3 and c-MYC were shown as common targets for the 2 protooncogenes. Our results are consistent with previous studies showing that YAP regulates c-MYC via abelson non-receptor tyrosine kinase (c-ABL) at the transcriptional level^27^ and interaction between c-MYC and YAP integrates mitogenic cues to induce cell-cycle entry and cell proliferation both in vitro and in vivo.^28^ Intriguingly, decreased c-MYC protein expression also was found in *Yap;Taz* double KO murine Akt/NRas HCCs (Figure 15), implying a pivotal role of c-MYC downstream of YAP/TAZ. Therefore, although YAP and TAZ also have been reported to regulate tumor development at the post-transcriptional level,^5^ at least our present data suggest that YAP and TAZ function coordinately at a transcriptional level to a great extent. However, they may have distinct expression patterns because HCCs may show only activated YAP or TAZ. Thus, HCC may depend on either YAP or TAZ for its growth.

Our study has important translational implications. Because inactivation of Hippo and activation of YAP or TAZ are almost ubiquitous in human HCCs, it is conceivable that targeting the Hippo/YAP/TAZ cascade may be highly effective for the treatment of HCC. However, because this pathway has critical roles in regulating normal organ homeostasis, it is possible that such treatment might be highly toxic, which can limit the therapeutic efficacy. Targeting YAP or TAZ individually, for instance, using siRNA-based gene silencing or clustered regularly interspaced short alindromic repeats (CRISPR) CIRSPR-associated (Cas) 9–based gene deletion in HCC cells, may assist in avoiding this potential toxicity. In this scenario, studies to determine whether YAP and/or TAZ are activated in specific HCC samples would be needed. Such investigations will be of help to identify HCC patients who will benefit from this treatment.

## Materials and Methods

### Constructs and Reagents

The constructs used for mouse injection in this study, including pT3 (vector), pT3-EF1a-HA-myr-Akt (human), pT2CAGGS-NRasV12 (human), pCMV, pCMV-Cre, and pCMV-Sleeping Beauty transposase were described previously.^29^ Plasmids were purified using the Endotoxin free Maxi Prep Kit (Sigma-Aldrich, St. Louis, MO).

### Mouse Treatment and Tail Vein Hydrodynamic Gene Delivery

Wild-type *FVB/N* mice were obtained from Charles River (Wilmington, MA). *Yap*^*flox/flox*^ and *Taz*^*flox/flox*^ mice were kindly provided by Dr Eric Olson from the University of Texas Southwestern Medical Center (Dallas, TX). *Yap*^*flox/flox*^*;Taz*^*flox/flox*^ mice were generated by crossing the *Yap*^*flox/flox*^ and *Taz*^*flox/flox*^ mice followed by genotyping for validation. Mice used for hydrodynamic injection were 5.5 to 6 weeks old. Hydrodynamic injections were performed as described.^29^ The dosages of plasmids used for murine tumor models are as follows: 15 μg AKT, 15 μg NRas, 60 μg pCMV, 60 μg pCMV-Cre, and 3.6 μg Sleeping Beauty transposase. Mice were monitored by abdominal palpation and euthanized when they developed a high burden of liver tumors (ie, large abdominal masses). Mice were housed, fed, and monitored in accordance with protocols approved by the Committee for Animal Research at the University of California, San Francisco.

### Protein Extraction and Western Blot Analysis

For total protein extraction, mouse liver tissues and cells were homogenized in Mammalian Protein Extraction Reagent (cat 78501; Thermo Fisher Scientific, Waltham, MA) containing the Halt Protease Inhibitor Cocktail (cat 78429; Thermo Fisher Scientific). For nuclear protein extraction, procedures were conducted using NE-PER Nuclear and Cytoplasmic Extraction Reagents (cat 78835; Thermo Fisher Scientific), following the manufacturer’s instructions. Subsequently, protein concentration was determined using the Pierce Microplate BCA Protein Assay Kit (cat 23252; Thermo Fisher Scientific). For Western blot, extracted proteins were boiled in Tris-Glycine Sodium Dodecyl Sulfate Sample Buffer (Bio-Rad Laboratories, Inc, Hercules, CA) for denaturation and subsequently separated by sodium dodecyl sulfate–polyacrylamide gel electrophoresis, and transferred onto nitrocellulose membranes (Bio-Rad Laboratories, Inc). Membranes were blocked in 10% nonfat milk in Tris-buffered saline containing 0.05% Tween-20 for 1 hour at room temperature and then incubated with primary antibodies (summarized in Table 3) at 4°C overnight. Membranes then were incubated with horseradish-peroxidase secondary antibody (1:5000; Jackson ImmunoResearch Laboratories, Inc, West Grove, PA) at room temperature for 1 hour. After appropriate washing, membranes were developed with the Clarity Western ECL Substrate (cat 170-5061; Bio-Rad Laboratories, Inc) or Clarity Max Western ECL Substrate (cat 170-5062; Bio-Rad Laboratories, Inc). Western blots were quantified using ImageJ software (Version 1.51n, National Institutes of Health, Bethesda, MD, https://imagej.nih.gov/ij), as previously described.^30^

**Table 3:** Antibodies Used for IHC and Western Blot

Application	Concentration	Company	Catalogue number
IHC			
Cleaved caspase 3	1:500	Cell Signaling Technology (Danvers, MA)	9664
CK19	1:1000	Abcam (Cambridge, United Kingdom)	Ab133496
HA-tag	1:500	Cell Signaling Technology	2367
Ki67	1:150	Thermo Fisher Scientific (Waltham, MA)	RM-9106-S1
Phospho-Akt^Ser473^	1:100	Cell Signaling Technology	4060
Phospho-Erk1/2	1:400	Cell Signaling Technology	4370
NEDD4	1:100	Proteintech (Rosemont, IL)	21698-1-ap
PTX3	1:100	Sigma-Aldrich (St. Louis, MO)	HPA069320
YAP	1:100	Cell Signaling Technology	10474
TAZ	1:100	Cell Signaling Technology	83669
WB			
p-Akt ^S473^	1:1000	Cell Signaling Technology	3787
c-Myc	1:10,000	Abcam	Ab32072
Cyclin A	1:750	Santa Cruz Biotechnology (Dallas, TX)	SC-751
Cyclin B1	1:250	Santa Cruz Biotechnology	SC-245
p-ERK1/2	1:1000	Cell Signaling Technology	9101
GAPDH	1:10,000	EMD Millipore (Burlington, MA)	MAB374
HA-tag	1:1000	Cell Signaling Technology	2367
NEDD4	1:1000	Santa Cruz Biotechnology	SC-390628
PCNA	1:1000	Cell Signaling Technology	2586
p-Rb	1:1000	Cell Signaling Technology	9307
Yap/Taz	1:1000	Cell Signaling Technology	8418
Second antibodies			
Goat anti-mouse	1:500	Invitrogen (Carlsbad, CA)	A11001
Goat anti-rabbit	1:500	Invitrogen	B2770

CK19, cytokeratin 19; GAPDH, glyceraldehyde-3-phosphate dehydrogenase; PCNA, proliferating cell nuclear antigen; WB, Western blot.

### RNA Extraction and Real-Time qPCR

Total mRNA from mouse liver tissues and cells was extracted by using the Quick RNA miniprep kit (Zymo Research, Irvine, CA). Next, mRNA expression was detected by real-time qPCR using SYBR Green Master Mix (Applied Biosystems, Foster City, CA) in a QuantStudio 6 Flex system (Applied Biosystems). Expression of each gene was normalized to the 18S ribosomal RNA. Thermal cycling started with an initial hold period at 95°C for 10 minutes, and then was followed by a 3-step PCR program of 95°C for 15 seconds, 60°C for 1 minute, and 72°C for 30 seconds for a total of 40 cycles. Primers used are listed in Table 4. For human specimens, Gene Expression Assays for human *YAP* (ID Hs00902712_g1), *TAZ* (WWTR1; Hs00210007_m1), and *β-*a*ctin* (ID 4333762T) genes were purchased from Applied Biosystems. Quantitative values for each gene were calculated using the PE Biosystems Analysis software (Foster City, CA) and expressed as a number target. The number target is equal to 2^−ΔCt^, wherein ΔCt value of each sample was calculated by subtracting the average cycle threshold (Ct) value of the target gene from the average Ct value of the *β-*a*ctin* gene.

**Table 4:** Sequences of Real-Time PCR Primers

Genes	Forward primer sequences, 5’-3’	Reverse primer sequences, 5’-3’
18s rRNA	CGGCTACCACATCCAAGGAA	GCTGGAATTACCGCGGCT
Ccnb1 (mouse)	AAGGTGCCTGTGTGTGAACC	GTCAGCCCCATCATCTGCG
Ccl20 (mouse)	GCCTCTCGTACATACAGACGC	CCAGTTCTGCTTTGGATCAGC
Cdk2 (mouse)	CCTGCTTATCAATGCAGAGGG	TGCGGGTCACCATTTCAGC
Cdca8 (mouse)	CAAATTGAGTCCGACAGACAGA	GCCGAAGGATCTCGATGTTGT
Cdc6 (mouse)	TGGCATCATACAAGTTTGTGTGG	CAGGCTGGACGTTTCTAAGTTTT
Cdc20 (mouse)	TTCGTGTTCGAGAGCGATTTG	ACCTTGGAACTAGATTTGCCAG
Ctgf (mouse)	GGGCCTCTTCTGCGATTTC	ATCCAGGCAAGTGCATTGGTA
Cxcl2 (mouse)	CATCCAGAGCTTGAGTGTGACG	GGCTTCAGGGTCAAGGCAAACT
Cxcl3 (mouse)	TGAGACCATCCAGAGCTTGACG	CCTTGGGGGTTGAGGCAAACTT
Cyr61 (mouse)	CTGCGCTAAACAACTCAACGA	GCAGATCCCTTTCAGAGCGG
CTGF (human)	CAGCATGGACGTTCGTCTG	AACCACGGTTTGGTCCTTGG
CYR61 (human)	GGTCAAAGTTACCGGGCAGT	GGAGGCATCGAATCCCAGC
Mad2l1 (mouse)	GTGGCCGAGTTTTTCTCATTTG	AGGTGAGTCCATATTTCTGCACT
Mcm3 (mouse)	AGCGCAGAGAGACTACTTGGA	CAGCCGATACTGGTTGTCACT
Mcm6 (mouse)	GCTGTTCCTAGACTTCCTGGA	CAACCAGCGTGTTTCTCTCAG
Myc (mouse)	ATGCCCCTCAACGTGAACTTC	CGCAACATAGGATGGAGAGCA
Nedd4 (mouse)	TCGGAGGACGAGGTATGGG	GGTACGGATCAGCAGTGAACA
NEDD4 (human)	TCAGGACAACCTAACAGATGCT	TTCTGCAAGATGAGTTGGAACA
Ptx3 (mouse)	CGCAGGTTGTGAAACAGCAAT	GGGTTCCACTTTGTGCCATAAG
Yap1 (mouse)	TACTGATGCAGGTACTGCGG	TCAGGGATCTCAAAGGAGGAC
Wwtr1 (mouse)	CATGGCGGAAAAAGATCCTCC	GTCGGTCACGTCATAGGACTG
YAP1 (human)	TAGCCCTGCGTAGCCAGTTA	TCATGCTTAGTCCACTGTCTGT
WWTR1 (human)	TCCCAGCCAAATCTCGTGATG	AGCGCATTGGGCATACTCAT

rRNA, ribosomal RNA.

### RNA Sequencing Analysis

RNA was isolated from 4 different HCC cell lines (Focus, PLC/PRF/5, SNU449, and SNU475) subjected to silencing of YAP (si*YAP*), TAZ (si*TAZ*), or YAP/TAZ (si*YT*). RNA was extracted using the Zymo Quick-RNA Miniprep Kit (cat D7001; Zymo Research), followed by quantification with Nanodrop (Thermo Fisher Scientific). RNA quality control was conducted with Bioanalyzer (Agilent, Santa Clara, CA). Library preparation and sequencing was performed by Novogene (Sacramento, CA). Poor quality reads were trimmed using fastq-mcf (1.05).^31^^,^^32^ Read quality was checked using fastqc (v0.11.7). We aligned reads to the respective genome (human reference genome version 38 [hg38]) using STAR (version 2.6.0). Gene read counts were in Ensembl ID and converted to Entrez ID using the Bioconductor Package Maintainer org.Hs.eg.db (version 3.8.2). Duplicated and not annotated genes were removed. Data were filtered out with genes with row means less than 5 or greater than 5000. The Edge R package (version 3.28.1) was used to calculate fragments per kilobase of transcript per million mapped reads, normalize data, and conduct Gene Ontology and KEGG analysis. A Venn diagram was drawn using the VennDiagram package.^33^ Heatmaps were generated using ComplexHeatmap (version 2.2.0).^34^

### Human HCC Samples

Formalin-fixed, paraffin-embedded tissues from a collection of 64 human HCC samples and corresponding non-neoplastic surrounding livers were used for the study. Tumors were divided in HCC with shorter/poorer (*n* = 37) and longer/better (*n* = 37) survival, characterized by <3 and 3 or more years' survival after partial liver resection, respectively. The HCC specimens were collected at the Institute of Pathology at the Medical University of Greifswald (Greifswald, Germany). Clinicopathologic features of the human HCC samples are reported in Table 1. All subjects provided their informed consent for inclusion in the investigation. The study was performed under the Institutional Review Board approval of the local Ethical Committee of the Medical University of Greifswald (approval code: BB 67/10).

### Cell Culture and In Vitro Studies

The SNU449 and SUN475 human HCC cell lines were a kind gift from Dr John Gordan at the University of California, San Francisco (San Francisco, CA). PLC/PRF/5 and Focus cells were purchased from American Type Culture Collection (Manassas, VA). All cell lines were authenticated (Genetica DNA Laboratories, Burlington, NC) and tested clear of mycoplasma contamination. Cells were cultured in Dulbecco’s modified Eagle medium (Gibco, Grand Island, NY) with 5% fetal bovine serum (Gibco), 100 μg/mL streptomycin, and 100 U/mL penicillin at 37°C in a 5% CO_2_ humidified incubator. For siRNA transfection, SNU449, SNU475, PLC/PRF/5, and Focus were transfected with siSC, si*YAP* (cat AM16708, assay ID 17374; Thermofisher Scientific), si*TAZ* (cat AM16708, assay ID 23134; Thermofisher Scientific), or si*YAP*/si*TAZ* by using the Thermofisher siRNA Transfection Kit. Procedures were conducted as indicated by the manufacturer. After 72 hours of transfection, cells were harvested for Western blot or RNA analysis. Proliferation assays were conducted as before.^35^

### Histology, Immunohistochemistry, and Proliferation and Apoptotic Indices

Histopathologic examination of the mouse lesions was conducted by 2 board-certified pathologists and liver experts (M.E. and S.R), in accordance with the criteria by Frith et al.^36^ Antigen unmasking was achieved by placing the slides in a microwave oven on high for 10 minutes in 10 mmol/L sodium citrate buffer (pH 6.0), followed by a 20-minute cool down at room temperature. After a blocking step with 5% goat serum and the avidin–biotin blocking kit (Vector Laboratories, Burlingame, CA), the slides were incubated with primary antibodies overnight at 4°C. Slides then were subjected to 3% hydrogen peroxide for 10 minutes to quench endogenous peroxidase activity and, subsequently, the biotin-conjugated secondary antibody was applied at a 1:500 dilution for 30 minutes at room temperature. The antibodies used are described in Table 3. Immunoreactivity was visualized with the Vectastain Elite ABC kit (cat PK-6100; Vector Laboratories) and ImmPACT 3,3′-diaminobenzidine tetra hydrochloride peroxidase (horseradish peroxidase) substrate (cat SK-4105; Vector Laboratories). Slides were counterstained with hematoxylin. Proliferation indices were determined in mouse HCC lesions by counting Ki-67–positive cells. ImageJ version 1.8.0 (National Institutes of Health, Bethesda, MD, https://imagej.nih.gov/ij/download.html) and Image Pro Plus 7 (Media Cybernetics, Rockville, MD) software were used for quantification.

### BrdU Incorporation and Flow Cytometric Analysis

For the BrdU incorporation assay, control or siRNA-treated cells were incubated with BrdU for 1 hour and the assay was performed using the fluorescein isothiocyanate BrdU Flow Kit (cat 559619; BD Biosciences, San Jose, CA), following the manufacturer’s instructions. Briefly, the cells were fixed after removing the medium with BrdU. Then, DNase was used to expose incorporated BrdU. Next, the anti-BrdU antibody was added and bound to newly synthesized cellular DNA, which is labeled with BrdU. 7-aminoactinomycin D was used for total DNA staining. Measurement of cell-cycle parameters was performed with the Becton Dickinson LSRII Flow Cytometer (BD Biosciences, San Jose, CA) and acquired data were analyzed by BD FACSDiva software 8.0 and FlowJo V10 (BD Biosciences). Each experiment was conducted 3 times.

### TCGA Data Analysis

For YAP and TAZ correlated gene analysis, liver hepatocellular carcinoma (TCGA, PanCancer Atlas) data at the public cBioPortal site (http://www.cbioprotal.org) were used. The top 2500 genes ranked by Spearman correlation score were subjected to bioinformatic analysis. The Edge R package (version 3.28.1) was used to conduct Gene Ontology analysis. A Venn diagram was drawn using the VennDiagram package (version 1.6.20).^33^ Heatmaps were drawn using ComplexHeatmap (version 2.2.0).^34^

### Statistical Analysis

Prism 7.0 software (GraphPad, San Diego, CA) was used to analyze the data, which are presented as means ± SD. Comparisons between the 2 groups were performed with a 2-tailed unpaired *t* test or the Fisher exact test. Welch correction was applied when necessary. *P* values less than .05 were considered statistically significant. Kaplan–Meier survival data were evaluated using a log-rank (Mantel–Cox) test.

All authors had access to the study data and reviewed and approved the final manuscript.
