# Supplementary material for: Distinct and Overlapping Roles of Hippo Effectors YAP and TAZ During Human and Mouse Hepatocarcinogenesis
Source: Cell Mol Gastroenterol Hepatol. 2020 Nov 21;11(4):1095–117. doi: 10.1016/j.jcmgh.2020.11.008 (PMC7903139; doi:10.1016/j.jcmgh.2020.11.008)
Supplement: Supplementary Table [file mmc1.pdf]

Supplementary Table 1 Genes positively correlated with *YAP* or *TAZ* mRNA expression from TCGA LIHC dataset.

YAP Correlated Genes					TAZ correlated genes				
Gene	Cytoband	Spearman's Correlation	p-Value	q-Value	Gene	Cytoband	Spearman's Correlation	p-Value	q-Value
PTPRG	3p14.2	0.716215117	5.30E-56	1.06E-51	EXOC6B	2p13.2	0.670476476	8.91E-47	1.79E-42
PARD3B	2q33.3	0.643736768	4.14E-42	4.16E-38	LIMS1	2q12.3	0.661708884	3.41E-45	3.43E-41
ROCK2	2p25.1	0.641925497	8.25E-42	5.52E-38	MAML2	11q21	0.650542271	2.98E-43	1.99E-39
ENC1	5q13.3	0.609964672	7.67E-37	3.08E-33	KIRREL1	1q23.1	0.647093071	1.14E-42	5.72E-39
BCL9L	11q23.3	0.60395383	5.72E-36	1.91E-32	UBXN7	3q29	0.63865897	2.83E-41	1.14E-37
MAP3K1	5q11.2	0.59984476	2.20E-35	4.91E-32	PIK3CA	3q26.32	0.635119948	1.06E-40	3.53E-37
MED17	11q21	0.598151702	3.82E-35	7.67E-32	CCDC186	10q25.3	0.630013722	6.86E-40	1.97E-36
RNF38	9p13.2	0.597673055	4.46E-35	8.14E-32	RASA2	3q23	0.625842332	3.08E-39	7.74E-36
NPAT	11q22.3	0.597166504	5.25E-35	8.78E-32	MGAT5	2q21.2-q21	0.625079132	4.05E-39	9.03E-36
LIMS1	2q12.3	0.594333635	1.30E-34	2.01E-31	HIPK3	11p13	0.623258002	7.73E-39	1.55E-35
ATRX	Xq21.1	0.593892189	1.50E-34	2.10E-31	STRN	2p22.2	0.621212436	1.59E-38	2.90E-35
CUL5	11q22.3	0.593755045	1.57E-34	2.10E-31	BOD1L1	4p15.33	0.620290736	2.20E-38	3.44E-35
TNKS2	10q23.32	0.593234827	1.85E-34	2.32E-31	ATF7	12q13.13	0.620250872	2.23E-38	3.44E-35
NRP1	10p11.22	0.591765473	2.95E-34	3.29E-31	NCKAP1	2q32.1	0.617527343	5.76E-38	8.27E-35
LATS2	13q12.11	0.591286073	3.44E-34	3.63E-31	RNF169	11q13.4	0.613353637	2.43E-37	3.25E-34
KIF13A	6p22.3	0.59112007	3.62E-34	3.64E-31	ZNF704	8q21.13	0.613166607	2.59E-37	3.25E-34
TNS3	7p12.3	0.588439293	8.41E-34	7.35E-31	ETV3	1q23.1	0.609026464	1.05E-36	1.24E-33
TCF12	15q21.3	0.588111232	9.32E-34	7.80E-31	MINDY2	15q21.3-q2	0.608742572	1.16E-36	1.29E-33
RUFY2	10q21.3	0.587733668	1.05E-33	8.22E-31	LEPROT	1p31.3	0.606154294	2.75E-36	2.81E-33
BIRC2	11q22.2	0.585731709	1.96E-33	1.46E-30	RIF1	2q23.3	0.606111298	2.79E-36	2.81E-33
PAFAH1B2	11q23.3	0.585311392	2.23E-33	1.58E-30	TCP11L2	12q23.3	0.605261637	3.71E-36	3.54E-33
ARHGAP12	10p11.22	0.582606655	5.13E-33	3.32E-30	PHC3	3q26.2	0.603427917	6.80E-36	6.21E-33
ANAPC1	2q13	0.582270094	5.69E-33	3.57E-30	CLOCK	4q12	0.59989402	2.17E-35	1.89E-32
SMAD3	15q22.33	0.581432107	7.35E-33	4.47E-30	PRKG1	10q11.23-q	0.597850554	4.21E-35	3.52E-32
RYK	3q22.2	0.580935806	8.56E-33	5.05E-30	TCF12	15q21.3	0.595132043	1.01E-34	7.90E-32
ZNF710	15q26.1	0.580008981	1.13E-32	6.51E-30	NRP1	10p11.22	0.595094216	1.02E-34	7.90E-32
RNF111	15q22.1-q2	0.579457726	1.34E-32	7.48E-30	BMPR2	2q33.1-q33	0.594369512	1.29E-34	9.59E-32
SLC11A2	12q13.12	0.577780328	2.23E-32	1.21E-29	MAST4	5q12.3	0.59392959	1.48E-34	1.06E-31
TNFRSF19	13q12.12	0.576738467	3.05E-32	1.57E-29	ELK3	12q23.1	0.593659658	1.62E-34	1.12E-31
CBL	11q23.3	0.57401864	6.89E-32	3.37E-29	TGFBRAP1	2q12.1-q12	0.592661642	2.22E-34	1.49E-31
NFAT5	16q22.1	0.573086404	9.09E-32	4.15E-29	LOC284441	19p12	0.592354013	2.45E-34	1.59E-31
ZBTB44	11q24.3	0.572604808	1.05E-31	4.68E-29	ADAM17	2p25.1	0.591722293	2.99E-34	1.88E-31
BOD1L1	4p15.33	0.56915615	2.90E-31	1.21E-28	PKD2	4q22.1	0.591505791	3.20E-34	1.95E-31
ZBTB38	3q23	0.567875678	4.21E-31	1.73E-28	UEVLD	11p15.1	0.591313692	3.41E-34	2.01E-31
CSNK2A3	11p15.4	0.567378239	4.87E-31	1.92E-28	ASXL2	2p23.3	0.590420181	4.52E-34	2.59E-31
EPC2	2q23.1	0.567174691	5.17E-31	2.00E-28	MOB1A	2p13.1	0.590005316	5.15E-34	2.87E-31
AASDHPPT	11q22.3	0.564252089	1.20E-30	4.40E-28	CTTNBP2NL	1p13.2	0.589645121	5.76E-34	3.13E-31
ARID4B	1q42.3	0.563680048	1.42E-30	5.09E-28	PTPRG	3p14.2	0.588736845	7.67E-34	4.05E-31
TGFBRAP1	2q12.1-q12.	0.562053306	2.26E-30	7.84E-28	ZEB1	10p11.22	0.588011573	9.62E-34	4.95E-31
PPP4R2	3p13	0.561870531	2.39E-30	8.03E-28	SWAP70	11p15.4	0.580834155	8.82E-33	4.32E-30
MFHAS1	8p23.1	0.560855721	3.19E-30	1.05E-27	ATRX	Xq21.1	0.580660363	9.31E-33	4.45E-30
LCOR	10q24.1	0.559610841	4.54E-30	1.47E-27	EYA3	1p35.3	0.580432957	9.97E-33	4.66E-30
MRE11	11q21	0.557292496	8.74E-30	2.74E-27	ADAM10	15q21.3	0.579571052	1.30E-32	5.92E-30
KMT2A	11q23.3	0.557160132	9.07E-30	2.80E-27	DPY19L3	19q13.11	0.579369457	1.38E-32	6.15E-30
CEMIP2	9q21.13	0.557083782	9.27E-30	2.82E-27	VCL	10q22.2	0.579270551	1.42E-32	6.20E-30
TRUB1	10q25.3	0.554724719	1.79E-29	5.30E-27	MAP3K2	2q14.3	0.578341833	1.88E-32	7.87E-30
DAAM1	14q23.1	0.554032518	2.18E-29	6.34E-27	ANKRD36BP1	1q24.2	0.57811414	2.02E-32	8.26E-30
CLASP1	2q14.2-q14.	0.553236387	2.72E-29	7.68E-27	P2RY1	3q25.2	0.577599344	2.35E-32	9.45E-30
RNMT	18p11.21	0.551881314	3.95E-29	1.09E-26	WDFY3	4q21.23	0.576517693	3.26E-32	1.28E-29
CDKL5	Xp22.13	0.551424889	4.48E-29	1.20E-26	PPP1R12A	12q21.2-q2	0.575192375	4.85E-32	1.84E-29
ACVR1	2q24.1	0.550893269	5.19E-29	1.37E-26	RSF1	11q14.1	0.575122288	4.95E-32	1.84E-29
ADNP	20q13.13	0.550521685	5.75E-29	1.50E-26	SLC30A6	2p22.3	0.574761524	5.52E-32	2.01E-29
MAPK1	22q11.22	0.549670212	7.27E-29	1.87E-26	DPP8	15q22.31	0.573902466	7.13E-32	2.51E-29
RAB3GAP1	2q21.3	0.549351977	7.93E-29	2.02E-26	CCNT1	12q13.11-q	0.573367734	8.36E-32	2.89E-29
DDX21	10q22.1	0.549068661	8.57E-29	2.15E-26	SLK	10q24.33-q	0.571948302	1.27E-31	4.28E-29
HIPK3	11p13	0.546373606	1.79E-28	4.32E-26	KMT2E	7q22.3	0.571930648	1.28E-31	4.28E-29
DDX6	11q23.3	0.545690232	2.15E-28	5.02E-26	ADAMTS12	5p13.3-p13	0.571881517	1.30E-31	4.28E-29
USP46	4q12	0.545305834	2.39E-28	5.41E-26	HIF1A	14q23.2	0.57171567	1.36E-31	4.33E-29
AKAP11	13q14.11	0.545281346	2.40E-28	5.41E-26	HIP1	7q11.23	0.571673569	1.38E-31	4.33E-29
CMIP	16q23.2-q2	0.545246608	2.42E-28	5.41E-26	ATP7A	Xq21.1	0.571116863	1.63E-31	5.03E-29
RASAL2	1q25.2	0.544517961	2.95E-28	6.37E-26	ROCK2	2p25.1	0.570280015	2.08E-31	6.34E-29
MPZL3	11q23.3	0.544311161	3.12E-28	6.66E-26	TAOK1	17q11.2	0.570145049	2.17E-31	6.49E-29
RSF1	11q14.1	0.544023654	3.37E-28	7.13E-26	BROX	1q41	0.569456265	2.65E-31	7.83E-29
ZFR	5p13.3	0.541093404	7.39E-28	1.47E-25	SCAF11	12q12	0.569052505	2.99E-31	8.69E-29
ASXL2	2p23.3	0.540849952	7.88E-28	1.55E-25	CDKL5	Xp22.13	0.568675628	3.33E-31	9.57E-29
KMT2E	7q22.3	0.539179672	1.23E-27	2.35E-25	DNAJC13	3q22.1	0.568028583	4.03E-31	1.14E-28

TBC1D9	4q31.21	0.536687921	2.37E-27	4.31E-25	SAMD8	10q22.2	0.567935474	4.14E-31	1.15E-28
DSP	6p24.3	0.536667135	2.38E-27	4.31E-25	SKIL	3q26.2	0.567841123	4.25E-31	1.17E-28
CDK13	7p14.1	0.536107622	2.76E-27	4.95E-25	ATF7IP	12p13.1	0.566584671	6.13E-31	1.66E-28
RASA1	5q14.3	0.536019638	2.82E-27	5.02E-25	KIAA0754	1p34.3	0.565896742	7.49E-31	2.01E-28
BMPR1A	10q23.2	0.535927382	2.89E-27	5.10E-25	PAFAH1B2	11q23.3	0.564767466	1.04E-30	2.74E-28
XRN2	20p11.22	0.53558598	3.16E-27	5.53E-25	SNX29	16p13.13-p	0.564321949	1.18E-30	3.08E-28
ZNF236	18q23	0.535329146	3.38E-27	5.86E-25	SMG1P3	16p12.2	0.564158979	1.24E-30	3.19E-28
JARID2	6p22.3	0.534963825	3.72E-27	6.39E-25	SMURF2	17q23.3-q2	0.563865698	1.35E-30	3.42E-28
WAPL	10q23.2	0.534390361	4.32E-27	7.36E-25	BTBD8	1p22.1	0.563612573	1.45E-30	3.63E-28
FUT11	10q22.2	0.533205315	5.88E-27	9.77E-25	CLASP1	2q14.2-q14	0.563289386	1.59E-30	3.94E-28
PTPN12	7q11.23	0.531648039	8.81E-27	1.44E-24	PDZD8	10q25.3-q2	0.563139044	1.66E-30	4.06E-28
GTF2I	7q11.23	0.530502248	1.18E-26	1.92E-24	LCOR	10q24.1	0.562882494	1.79E-30	4.32E-28
ATF7	12q13.13	0.530283284	1.25E-26	2.01E-24	CRIM1	2p22.2	0.562809031	1.82E-30	4.36E-28
RNF169	11q13.4	0.529874399	1.39E-26	2.22E-24	KLHL11	17q21.2	0.562315715	2.10E-30	4.96E-28
8-Mar	10q11.21-q1	0.529402871	1.57E-26	2.48E-24	PDE3A	12p12.2	0.562269369	2.13E-30	4.97E-28
ZNRF3	22q12.1	0.529128952	1.68E-26	2.64E-24	CEP170	1q43	0.560792793	3.24E-30	7.49E-28
ATF1	12q13.12	0.52883026	1.82E-26	2.83E-24	GPR176	15q14-q15.	0.560541313	3.49E-30	7.95E-28
REST	4q12	0.527738286	2.40E-26	3.71E-24	PCDHGB7	5q31.3	0.560398544	3.63E-30	8.19E-28
RASA2	3q23	0.527105808	2.82E-26	4.31E-24	LOC653653	17q23.1	0.560068104	3.99E-30	8.90E-28
RAB11FIP2	10q26.11	0.526640902	3.18E-26	4.80E-24	TACC1	8p11.22	0.559504064	4.68E-30	1.03E-27
OTUD4	4q31.21	0.526286117	3.48E-26	5.21E-24	IL6ST	5q11.2	0.559467902	4.73E-30	1.03E-27
DIXDC1	11q23.1	0.525769885	3.96E-26	5.89E-24	REST	4q12	0.559189143	5.12E-30	1.10E-27
LDB1	10q24.32	0.524697273	5.19E-26	7.53E-24	LRCH3	3q29	0.558814426	5.69E-30	1.20E-27
DPY19L3	19q13.11	0.524687022	5.21E-26	7.53E-24	RNF168	3q29	0.5586302	5.99E-30	1.25E-27
CTNND1	11q12.1	0.524479162	5.49E-26	7.82E-24	GTF3C4	9q34.13	0.558412944	6.37E-30	1.32E-27
ATM	11q22.3	0.523765321	6.57E-26	9.29E-24	CSNK2A3	11p15.4	0.558191702	6.78E-30	1.39E-27
NCOA3	20q13.12	0.523622952	6.81E-26	9.50E-24	KIAA2026	9p24.1	0.558124788	6.91E-30	1.40E-27
KDM3B	5q31.2	0.522501544	9.02E-26	1.24E-23	BPTF	17q24.2	0.557719604	7.75E-30	1.56E-27
NFKB1	4q24	0.522306213	9.48E-26	1.29E-23	ACAP2	3q29	0.557437428	8.39E-30	1.67E-27
TGOLN2	2p11.2	0.522014176	1.02E-25	1.37E-23	SOCS5	2p21	0.557286231	8.76E-30	1.72E-27
EEA1	12q22	0.522005064	1.02E-25	1.37E-23	ACTR2	2p14	0.556787938	1.01E-29	1.96E-27
BICC1	10q21.1	0.521609954	1.13E-25	1.49E-23	LUZP1	1p36.12	0.556281102	1.16E-29	2.23E-27
MAP4K5	14q22.1	0.521302328	1.22E-25	1.60E-23	KIDINS220	2p25.1	0.556269468	1.17E-29	2.23E-27
TGFBR2	3p24.1	0.521008762	1.31E-25	1.70E-23	MED1	17q12	0.5558856	1.30E-29	2.41E-27
SBF2	11p15.4	0.52098342	1.32E-25	1.70E-23	NHLRC2	10q25.3	0.554217638	2.07E-29	3.81E-27
DENND5A	11p15.4	0.520868955	1.36E-25	1.73E-23	TASOR2	10p15.1	0.554093452	2.14E-29	3.91E-27
CWF19L2	11q22.3	0.520712349	1.41E-25	1.78E-23	ATP2C1	3q22.1	0.553507459	2.52E-29	4.56E-27
USP34	2p15	0.520670207	1.43E-25	1.79E-23	EPC2	2q23.1	0.552937451	2.95E-29	5.29E-27
LGR5	12q21.1	0.520544924	1.47E-25	1.83E-23	AKAP10	17p11.2	0.552713606	3.14E-29	5.58E-27
PICALM	11q14.2	0.520393441	1.53E-25	1.89E-23	EPS8	12p12.3	0.552594016	3.25E-29	5.72E-27
LAMC1	1q25.3	0.519980216	1.69E-25	2.09E-23	SCRN1	7p14.3	0.552296464	3.52E-29	6.10E-27
ATF2	2q31.1	0.519944692	1.71E-25	2.09E-23	FUT11	10q22.2	0.551725743	4.13E-29	7.08E-27
PUM1	1p35.2	0.519778231	1.78E-25	2.17E-23	ZBTB38	3q23	0.550372197	5.99E-29	1.02E-26
ARHGAP32	11q24.3	0.519482276	1.92E-25	2.29E-23	PARD3B	2q33.3	0.549363548	7.90E-29	1.33E-26
ARHGEF12	11q23.3	0.51942903	1.94E-25	2.31E-23	GMCL1	2p13.3	0.549297876	8.05E-29	1.35E-26
CREBRF	5q35.1	0.518721453	2.31E-25	2.72E-23	REL	2p16.1	0.549220142	8.22E-29	1.36E-26
CFAP97	4q35.1	0.516768428	3.74E-25	4.32E-23	EPC1	10p11.22	0.548500607	1.00E-28	1.65E-26
TACC1	8p11.22	0.516461194	4.04E-25	4.63E-23	EDIL3	5q14.3	0.54814691	1.10E-28	1.80E-26
EXOC6B	2p13.2	0.516133923	4.38E-25	4.97E-23	KIF5B	10p11.22	0.547561861	1.29E-28	2.09E-26
BICRAL	6p21.1	0.515825941	4.72E-25	5.32E-23	ZMYM4	1p34.3	0.547169736	1.44E-28	2.31E-26
ZKSCAN8	6p22.1	0.515616373	4.97E-25	5.55E-23	BICC1	10q21.1	0.546832475	1.58E-28	2.51E-26
CDON	11q24.2	0.515609255	4.98E-25	5.55E-23	LATS2	13q12.11	0.545639833	2.18E-28	3.45E-26
RCBTB1	13q14.2	0.515569143	5.02E-25	5.58E-23	CEP97	3q12.3	0.545174285	2.47E-28	3.82E-26
VPS54	2p15-p14	0.51541791	5.21E-25	5.75E-23	SRPK2	7q22.3	0.545072063	2.54E-28	3.89E-26
SLK	10q24.33-q2	0.514542337	6.46E-25	7.05E-23	UHMK1	1q23.3	0.544985787	2.60E-28	3.94E-26
PLEKHA7	11p15.2-p15	0.514313407	6.83E-25	7.41E-23	ADAT1	16q23.1	0.544976391	2.61E-28	3.94E-26
KIDINS220	2p25.1	0.514088358	7.21E-25	7.79E-23	MFN1	3q26.33	0.544172857	3.24E-28	4.85E-26
WWTR1	3q25.1	0.51387377	7.60E-25	8.16E-23	C9ORF129	9q22.31	0.5439674	3.42E-28	5.09E-26
SMURF2	17q23.3-q24	0.513541195	8.24E-25	8.71E-23	ZMYND11	10p15.3	0.543500873	3.88E-28	5.73E-26
PPP1R12A	12q21.2-q21	0.513319847	8.70E-25	9.15E-23	TP53BP1	15q15.3	0.543052409	4.38E-28	6.41E-26
SOCS5	2p21	0.513002753	9.40E-25	9.83E-23	MACF1	1p34.3	0.542975244	4.47E-28	6.50E-26
TTC30B	2q31.2	0.512850418	9.75E-25	1.01E-22	ARHGAP21	10p12.1|10p	0.542855939	4.61E-28	6.66E-26
NUP160	11p11.2	0.512838459	9.78E-25	1.01E-22	WDR47	1p13.3	0.54279956	4.68E-28	6.72E-26
GIGYF2	2q37.1	0.512478548	1.07E-24	1.09E-22	NCK1	3q22.3	0.541913168	5.94E-28	8.40E-26
NCKAP1	2q32.1	0.512468298	1.07E-24	1.09E-22	NBEAL1	2q33.2	0.541791584	6.13E-28	8.61E-26
MTMR12	5p13.3	0.511145152	1.47E-24	1.47E-22	AKAP2	9q31.3	0.541644115	6.38E-28	8.90E-26
RC3H2	9q33.2	0.510913338	1.56E-24	1.54E-22	BBX	3q13.12	0.541598816	6.46E-28	8.94E-26
ATE1	10q26.13	0.509957184	1.96E-24	1.92E-22	MAP3K1	5q11.2	0.541249726	7.09E-28	9.62E-26
KIAA0754	1p34.3	0.509863789	2.01E-24	1.96E-22	TANC1	2q24.2	0.541070055	7.43E-28	1.00E-25
PDZD8	10q25.3-q26	0.509058262	2.43E-24	2.34E-22	SMG1P1	16p12.2	0.540887372	7.81E-28	1.04E-25
GPATCH8	17q21.31	0.508970278	2.48E-24	2.37E-22	DOCK1	10q26.2	0.540620167	8.38E-28	1.11E-25
CEP170	1q43	0.50886948	2.55E-24	2.41E-22	IPMK	10q21.1	0.539886424	1.02E-27	1.34E-25
AXIN2	17q24.1	0.508649947	2.68E-24	2.52E-22	IQGAP1	15q26.1	0.53975513	1.05E-27	1.38E-25
SLC30A6	2p22.3	0.508207462	2.98E-24	2.79E-22	ARHGAP29	1p22.1	0.539715267	1.07E-27	1.38E-25

AKAP10	17p11.2	0.507691515	3.37E-24	3.14E-22	ATF2	2q31.1	0.538968965	1.30E-27	1.67E-25
EP300	22q13.2	0.507512699	3.52E-24	3.24E-22	LATS1	6q25.1	0.538738612	1.38E-27	1.77E-25
TMF1	3p14.1	0.507510705	3.52E-24	3.24E-22	SPATA13	13q12.12	0.538568622	1.44E-27	1.84E-25
ITGA2	5q11.2	0.507470842	3.56E-24	3.26E-22	TTBK2	15q15.2	0.53778577	1.78E-27	2.24E-25
BRWD3	Xq21.1	0.50699305	3.98E-24	3.62E-22	MYO9A	15q23	0.537739747	1.80E-27	2.26E-25
BPTF	17q24.2	0.506846979	4.13E-24	3.72E-22	PAPOLG	2p16.1	0.537677958	1.83E-27	2.28E-25
ELF1	13q14.11	0.505779777	5.32E-24	4.66E-22	ADAM9	8p11.22	0.537433083	1.95E-27	2.42E-25
WWC2	4q35.1	0.505592988	5.56E-24	4.85E-22	RNF38	9p13.2	0.536354491	2.59E-27	3.19E-25
TAF1	Xq13.1	0.505410185	5.80E-24	5.04E-22	TAF1	Xq13.1	0.536030743	2.82E-27	3.45E-25
POLR2B	4q12	0.505288886	5.97E-24	5.13E-22	LCA5	6q14.1	0.5355789	3.17E-27	3.86E-25
C9ORF129	9q22.31	0.50528756	5.97E-24	5.13E-22	KLF7	2q33.3	0.535043747	3.65E-27	4.41E-25
SEPTIN10	2q13	0.504926129	6.51E-24	5.56E-22	ZBTB4	17p13.1	0.534744291	3.94E-27	4.74E-25
MINDY2	15q21.3-q22	0.504523508	7.16E-24	6.06E-22	WDFY3-AS2	4q21.23	0.534444127	4.26E-27	5.10E-25
MAP3K8	10p11.23	0.50443894	7.30E-24	6.16E-22	RECQL	12p12.1	0.533898901	4.91E-27	5.81E-25
DYNC1I2	2q31.1	0.504415307	7.34E-24	6.17E-22	FRMD3	9q21.32	0.53339846	5.60E-27	6.57E-25
EPC1	10p11.22	0.504201468	7.72E-24	6.43E-22	RAB3GAP1	2q21.3	0.533363592	5.65E-27	6.59E-25
WAC	10p12.1|10p	0.503932105	8.23E-24	6.80E-22	ZNF791	19p13.13	0.533294969	5.75E-27	6.67E-25
AMOTL2	3q22.2	0.503407615	9.31E-24	7.63E-22	ROR1	1p31.3	0.531699589	8.69E-27	9.92E-25
TET3	2p13.1	0.503043434	1.01E-23	8.24E-22	OSBPL8	12q21.2	0.531654588	8.79E-27	9.98E-25
TXLNG	Xp22.2	0.502743888	1.09E-23	8.74E-22	PEAK1	15q24.3	0.531513642	9.12E-27	1.03E-24
JMJD1C	10q21.3	0.502656758	1.11E-23	8.88E-22	ITGAV	2q32.1	0.5314562	9.26E-27	1.04E-24
PCNX4	14q23.1	0.502099239	1.27E-23	1.01E-21	ERCC6L2	9q22.32	0.530755666	1.11E-26	1.23E-24
STRN	2p22.2	0.501992462	1.30E-23	1.03E-21	NPAT	11q22.3	0.530488296	1.19E-26	1.31E-24
ATXN1L	16q22.2	0.501752711	1.37E-23	1.09E-21	ZKSCAN8	6p22.1	0.530142338	1.30E-26	1.42E-24
RDX	11q22.3	0.50165736	1.40E-23	1.11E-21	JAG1	20p12.2	0.52945327	1.55E-26	1.69E-24
PTCH1	9q22.32	0.501423446	1.48E-23	1.16E-21	JAK1	1p31.3	0.528056909	2.21E-26	2.40E-24
IPO7	11p15.4	0.501276628	1.53E-23	1.20E-21	TRAF6	11p12	0.527852969	2.33E-26	2.52E-24
MIB1	18q11.2	0.501242459	1.55E-23	1.20E-21	LUZP6	7q33	0.527227179	2.74E-26	2.92E-24
DENND4C	9p22.1	0.501215124	1.56E-23	1.20E-21	RYK	3q22.2	0.527171086	2.78E-26	2.95E-24
CCAR1	10q21.3	0.501107493	1.60E-23	1.23E-21	CCNI	4q21.1	0.526176492	3.57E-26	3.76E-24
KAT6B	10q22.2	0.499784025	2.17E-23	1.64E-21	STAG1	3q22.3	0.526149727	3.60E-26	3.76E-24
DENND4A	15q22.31	0.49915105	2.52E-23	1.87E-21	DYNC1I2	2q31.1	0.525258209	4.51E-26	4.67E-24
KIAA2026	9p24.1	0.499024341	2.59E-23	1.92E-21	DNAJB14	4q23	0.524265608	5.79E-26	5.97E-24
ADAM10	15q21.3	0.498853213	2.70E-23	1.99E-21	ANKS1A	6p21.31	0.523919687	6.32E-26	6.48E-24
NCOA2	8q13.3	0.498805662	2.73E-23	2.00E-21	MYOF	10q23.33	0.523701255	6.68E-26	6.81E-24
LRCH3	3q29	0.498352642	3.03E-23	2.21E-21	RNF111	15q22.1-q2	0.523473179	7.07E-26	7.17E-24
KLHL11	17q21.2	0.498256502	3.10E-23	2.25E-21	TRIM44	11p13	0.523403133	7.20E-26	7.26E-24
PHF2	9q22.31	0.49809837	3.21E-23	2.33E-21	NR2C2	3p25.1	0.523142028	7.68E-26	7.72E-24
RBM12	20q11.22	0.498049964	3.25E-23	2.35E-21	JMJD1C	10q21.3	0.522848462	8.27E-26	8.22E-24
ZNF264	19q13.43	0.498012948	3.27E-23	2.36E-21	HIVEP2	6q24.2	0.522728586	8.52E-26	8.43E-24
FAM168A	11q13.4	0.497940909	3.33E-23	2.39E-21	CREBRF	5q35.1	0.522632914	8.73E-26	8.60E-24
NFRKB	11q24.3	0.497534587	3.66E-23	2.60E-21	ZXDA	Xp11.21	0.522410712	9.23E-26	9.04E-24
NKAPD1	11q23.1	0.497349114	3.82E-23	2.70E-21	SLIT2	4p15.31	0.521440133	1.18E-25	1.15E-23
SEC23IP	10q26.11-q2	0.497266362	3.89E-23	2.74E-21	MBNL1	3q25.1-q25	0.521233706	1.24E-25	1.20E-23
FKBP14	7p14.3	0.496994436	4.14E-23	2.87E-21	RSPRY1	16q13	0.520681597	1.42E-25	1.37E-23
ATXN7L3B	12q21.1	0.496839254	4.29E-23	2.96E-21	TRIP12	2q36.3	0.520509899	1.48E-25	1.41E-23
NR2C2	3p25.1	0.496302805	4.86E-23	3.32E-21	PCDHGA12	5q31.3	0.520379712	1.53E-25	1.45E-23
FAM217B	20q13.33	0.496162891	5.01E-23	3.41E-21	ATE1	10q26.13	0.519993952	1.69E-25	1.58E-23
AMMECR1L	2q14.3	0.495791129	5.46E-23	3.69E-21	CBL	11q23.3	0.51968501	1.82E-25	1.70E-23
GTF3C4	9q34.13	0.495257243	6.17E-23	4.16E-21	ZMIZ1	10q22.3	0.519590192	1.86E-25	1.73E-23
BROX	1q41	0.49511943	6.37E-23	4.28E-21	ZNF148	3q21.2	0.5194669	1.92E-25	1.78E-23
ANKRD36BP1	1q24.2	0.494904408	6.69E-23	4.48E-21	TMTC2	12q21.31	0.518793355	2.27E-25	2.09E-23
DOCK7	1p31.3	0.494158435	7.94E-23	5.26E-21	ZNF81	Xp11.23	0.518787797	2.28E-25	2.09E-23
MALT1	18q21.32	0.494057637	8.12E-23	5.36E-21	WIPF2	17q21.2	0.518741385	2.30E-25	2.10E-23
NEU3	11q13.4	0.49366185	8.89E-23	5.85E-21	TMF1	3p14.1	0.518630906	2.37E-25	2.15E-23
KIF5B	10p11.22	0.493546566	9.12E-23	5.99E-21	ROCK1	18q11.1	0.518614676	2.37E-25	2.15E-23
RSPRY1	16q13	0.493113158	1.01E-22	6.50E-21	ARMC8	3q22.3	0.518553494	2.41E-25	2.17E-23
TOGARAM1	14q21.2	0.493072155	1.02E-22	6.54E-21	LDLRAD3	11p13	0.518545321	2.42E-25	2.17E-23
MTMR3	22q12.2	0.493021187	1.03E-22	6.60E-21	TOR1AIP2	1q25.2	0.518081644	2.71E-25	2.42E-23
PARG	10q11.23	0.492922418	1.05E-22	6.73E-21	TEAD1	11p15.3	0.518016154	2.75E-25	2.45E-23
USP25	21q21.1	0.492897326	1.06E-22	6.74E-21	ITGB1	10p11.22	0.517761882	2.93E-25	2.59E-23
C2CD3	11q13.4	0.492684341	1.11E-22	7.05E-21	ZNF827	4q31.21-q3	0.516897715	3.63E-25	3.19E-23
DCP1A	3p21.1	0.492553361	1.14E-22	7.24E-21	CEP68	2p14	0.516717744	3.79E-25	3.32E-23
UPF2	10p14	0.49217512	1.25E-22	7.87E-21	ZFHX3	16q22.2-q2	0.516505081	3.99E-25	3.47E-23
STARD13	13q13.1-q13	0.491981035	1.30E-22	8.20E-21	TXLNG	Xp22.2	0.51637862	4.12E-25	3.57E-23
SOS1	2p22.1	0.491581262	1.43E-22	8.95E-21	MRTFB	16p13.12	0.516063983	4.45E-25	3.84E-23
GTF2A1	14q31.1	0.490335705	1.89E-22	1.17E-20	GPATCH8	17q21.31	0.515579642	5.01E-25	4.30E-23
PRPF4B	6p25.2	0.490287977	1.91E-22	1.18E-20	SBNO1	12q24.31	0.515055869	5.70E-25	4.85E-23
ARID2	12q12	0.489855173	2.10E-22	1.28E-20	AMOTL2	3q22.2	0.514907373	5.91E-25	5.01E-23
ARCN1	11q23.3	0.489735902	2.16E-22	1.31E-20	YAP1	11q22.1	0.51387377	7.60E-25	6.36E-23
DLG5	10q22.3	0.48944401	2.31E-22	1.40E-20	ITGA8	10p13	0.513495585	8.33E-25	6.95E-23
DLG1	3q29	0.489335809	2.36E-22	1.43E-20	SVIL	10p11.23	0.512660497	1.02E-24	8.47E-23
PCF11	11q14.1	0.489024874	2.54E-22	1.52E-20	PARVA	11p15.3	0.51247684	1.07E-24	8.82E-23
TOP2B	3p24.2	0.488956252	2.57E-22	1.54E-20	PROX1	1q32.3	0.512034925	1.19E-24	9.78E-23

SCAF11	12q12	0.488422081	2.90E-22	1.73E-20	ANKIB1	7q21.2	0.511648534	1.30E-24	1.07E-22
KANSL1	17q21.31	0.488309325	2.98E-22	1.77E-20	KIF27	9q21.32	0.511271576	1.43E-24	1.17E-22
CCDC186	10q25.3	0.488119688	3.11E-22	1.84E-20	BMP2K	4q21.21	0.511178324	1.46E-24	1.19E-22
RIF1	2q23.3	0.487698844	3.41E-22	2.02E-20	TAF3	10p14	0.511101871	1.49E-24	1.20E-22
KDM3A	2p11.2	0.487455962	3.60E-22	2.12E-20	ATR	3q23	0.510831333	1.59E-24	1.28E-22
WDR82	3p21.2	0.487439447	3.62E-22	2.12E-20	PPP2R5E	14q23.2	0.510775524	1.61E-24	1.29E-22
LOC284441	19p12	0.487365553	3.68E-22	2.15E-20	ZNF366	5q13.2|5q1	0.510711617	1.64E-24	1.30E-22
CTNNB1	3p22.1	0.487060459	3.93E-22	2.28E-20	PPP1R2B	5q33.3	0.510617672	1.67E-24	1.33E-22
ANKRD50	4q28.1	0.487011199	3.98E-22	2.30E-20	CACNA2D1	7q21.11	0.509709683	2.08E-24	1.65E-22
ATP11A	13q34	0.486883351	4.09E-22	2.36E-20	RRAGC	1p34.3	0.509619483	2.13E-24	1.67E-22
SPATA13	13q12.12	0.486725321	4.24E-22	2.43E-20	SHPRH	6q24.3	0.508917068	2.52E-24	1.97E-22
DHX33	17p13.2	0.486581243	4.38E-22	2.50E-20	FKBP9P1	7p11.2	0.508573144	2.73E-24	2.13E-22
LEPROT	1p31.3	0.486145023	4.82E-22	2.73E-20	USP34	2p15	0.507766686	3.31E-24	2.56E-22
IL1R1	2q11.2-q12.	0.486102312	4.87E-22	2.75E-20	WDR31	9q32	0.507427321	3.59E-24	2.76E-22
STAG1	3q22.3	0.4858973	5.10E-22	2.87E-20	GUCY1A1	4q32.1	0.507254725	3.74E-24	2.87E-22
FNBP1L	1p22.1	0.485815757	5.19E-22	2.91E-20	PPP4R3B	2p16.1	0.507190552	3.80E-24	2.90E-22
PPP1R2B	5q33.3	0.485698444	5.33E-22	2.98E-20	RAPGEF6	5q31.1	0.506890828	4.08E-24	3.11E-22
SMG1P1	16p12.2	0.485639276	5.40E-22	3.01E-20	GTF2A1	14q31.1	0.506837761	4.13E-24	3.13E-22
LEMD3	12q14.3	0.485597077	5.45E-22	3.03E-20	TAF1L	9p21.1	0.506286082	4.71E-24	3.55E-22
BTBD7	14q32.12	0.485497561	5.57E-22	3.08E-20	BARD1	2q35	0.505925283	5.14E-24	3.85E-22
HIVEP1	6p24.1	0.48549354	5.57E-22	3.08E-20	CYSLTR2	13q14.2	0.50588501	5.18E-24	3.87E-22
SP1	12q13.13	0.485151033	6.01E-22	3.32E-20	GCC2	2q12.3	0.505859788	5.22E-24	3.88E-22
ANKIB1	7q21.2	0.484812444	6.48E-22	3.57E-20	LRRC37A4P	17q21.31	0.505346119	5.89E-24	4.33E-22
SINHCAF	12p11.21	0.484656691	6.71E-22	3.67E-20	ZBTB10	8q21.13	0.505036323	6.34E-24	4.65E-22
NUP153	6p22.3	0.484523468	6.91E-22	3.77E-20	VGLL4	3p25.3-p25	0.504846117	6.63E-24	4.84E-22
ANKRD17	4q13.3	0.484476167	6.98E-22	3.80E-20	TTC30B	2q31.2	0.504510979	7.18E-24	5.20E-22
RABGAP1	9q33.2-q33.	0.48407497	7.63E-22	4.13E-20	LPP	3q27.3-q28	0.504415592	7.34E-24	5.30E-22
UBXN7	3q29	0.483951108	7.84E-22	4.23E-20	EFCAB14	1p33	0.504364338	7.43E-24	5.33E-22
YLPM1	14q24.3	0.48351745	8.63E-22	4.63E-20	ARHGAP12	10p11.22	0.503825327	8.44E-24	6.01E-22
DOCK1	10q26.2	0.483035957	9.59E-22	5.14E-20	FLNA	Xq28	0.503654769	8.78E-24	6.22E-22
KBTBD7	13q14.11	0.482970467	9.73E-22	5.20E-20	POLR2A	17p13.1	0.503645372	8.80E-24	6.22E-22
RNF214	11q23.3	0.482551615	1.07E-21	5.68E-20	SPTLC2	14q24.3	0.503544326	9.01E-24	6.35E-22
USP28	11q23.2	0.481998937	1.20E-21	6.37E-20	CYTH3	7p22.1	0.503528914	9.05E-24	6.35E-22
SMC3	10q25.2	0.481871658	1.24E-21	6.53E-20	ZNF281	1q32.1	0.503370884	9.39E-24	6.57E-22
DCAF17	2q31.1	0.481825531	1.25E-21	6.58E-20	FAM168A	11q13.4	0.503037454	1.02E-23	7.06E-22
GMCL1	2p13.3	0.481700815	1.29E-21	6.74E-20	USP46	4q12	0.50283529	1.06E-23	7.35E-22
ZNRF2P1	7p14.3	0.481413228	1.37E-21	7.16E-20	LINC02035	3q21.1	0.502470823	1.16E-23	7.98E-22
AKAP2	9q31.3	0.481237978	1.42E-21	7.42E-20	ASAP1	8q24.21-q2	0.502211782	1.23E-23	8.42E-22
AEBP2	12p12.3	0.480721028	1.59E-21	8.27E-20	VPS13C	15q22.2	0.502012394	1.29E-23	8.73E-22
NCOA6	20q11.22	0.480536517	1.66E-21	8.59E-20	CMIP	16q23.2-q2	0.501966835	1.31E-23	8.80E-22
RAB30	11q14.1	0.48040614	1.71E-21	8.79E-20	ANKRD17	4q13.3	0.501855787	1.34E-23	9.00E-22
TAF1L	9p21.1	0.480116539	1.82E-21	9.34E-20	BIRC6	2p22.3	0.501538303	1.44E-23	9.63E-22
WIPF2	17q21.2	0.479803598	1.95E-21	9.95E-20	MAP4K3	2p22.1	0.501126855	1.59E-23	1.06E-21
ZBTB33	Xq24	0.47974722	1.97E-21	1.00E-19	ANKRD44	2q33.1	0.500893084	1.68E-23	1.11E-21
SAMD8	10q22.2	0.479599725	2.03E-21	1.03E-19	ZNF41	Xp11.3	0.500424688	1.87E-23	1.23E-21
GLUD2	Xq24	0.479378483	2.13E-21	1.08E-19	C10ORF12	10q24.1	0.500332433	1.91E-23	1.25E-21
AP3M1	10q22.2	0.479238676	2.20E-21	1.11E-19	EMSY	11q13.5	0.499913866	2.11E-23	1.37E-21
LUZP6	7q33	0.478844028	2.40E-21	1.20E-19	SCYL2	12q23.1	0.499056517	2.57E-23	1.66E-21
PHF20	20q11.22-q1	0.478812422	2.41E-21	1.21E-19	RGPD3	2q12.2	0.498805947	2.73E-23	1.75E-21
BMPR2	2q33.1-q33.	0.47863987	2.51E-21	1.25E-19	RC3H2	9q33.2	0.498588691	2.87E-23	1.84E-21
ETV3	1q23.1	0.478397755	2.64E-21	1.31E-19	ACER2	9p22.1	0.498391152	3.00E-23	1.92E-21
SP3	2q31.1	0.478228137	2.74E-21	1.35E-19	SEPTIN7	7p14.2	0.49822935	3.11E-23	1.99E-21
MAML1	5q35.3	0.478038501	2.85E-21	1.40E-19	DHX15	4p15.2	0.497924394	3.34E-23	2.12E-21
PREPL	2p21	0.477943398	2.91E-21	1.42E-19	ZNF800	7q31.33	0.497528607	3.66E-23	2.32E-21
HIF1A	14q23.2	0.477676028	3.09E-21	1.51E-19	OSMR	5p13.1	0.497114312	4.03E-23	2.54E-21
MAP4K3	2p22.1	0.477421756	3.26E-21	1.58E-19	ADAMTS15	11q24.3	0.497075934	4.06E-23	2.56E-21
MAML2	11q21	0.4772045	3.42E-21	1.65E-19	SNTB2	16q22.1	0.496856373	4.28E-23	2.68E-21
MLLT10	10p12.31	0.477056151	3.53E-21	1.69E-19	FRYL	4p11	0.496798714	4.33E-23	2.71E-21
SPTLC2	14q24.3	0.477039528	3.54E-21	1.69E-19	ITGA2	5q11.2	0.496538	4.60E-23	2.87E-21
TRIM56	7q22.1	0.476926879	3.63E-21	1.73E-19	SON	21q22.11	0.496356621	4.80E-23	2.98E-21
ZNF551	19q13.43	0.476606222	3.89E-21	1.85E-19	RPS6KA5	14q32.11	0.49624422	4.92E-23	3.05E-21
PACSIN2	22q13.2	0.476489235	3.99E-21	1.88E-19	PTPRE	10q26.2	0.4961126	5.07E-23	3.13E-21
EIF4G2	11p15.4	0.476065184	4.37E-21	2.05E-19	TMOD3	15q21.2	0.495894774	5.33E-23	3.29E-21
GOLIM4	3q26.2	0.476057001	4.38E-21	2.05E-19	GOLIM4	3q26.2	0.4958122	5.44E-23	3.34E-21
TP53BP1	15q15.3	0.47589527	4.53E-21	2.12E-19	GIGYF2	2q37.1	0.495557643	5.76E-23	3.53E-21
SIRT1	10q21.3	0.47573827	4.69E-21	2.18E-19	RANBP2	2q13	0.495506675	5.83E-23	3.56E-21
SLC35F5	2q14.1	0.475691396	4.74E-21	2.20E-19	8-Mar	10q11.21-q	0.495114304	6.38E-23	3.88E-21
GCC2	2q12.3	0.475513719	4.92E-21	2.28E-19	HIVEP1	6p24.1	0.495044543	6.48E-23	3.93E-21
POLR3A	10q22.3	0.475394413	5.05E-21	2.32E-19	ZDHHC21	9p22.3	0.494914133	6.68E-23	4.01E-21
SKIL	3q26.2	0.475190859	5.27E-21	2.42E-19	BICRAL	6p21.1	0.494756957	6.92E-23	4.15E-21
MYO9A	15q23	0.474611381	5.97E-21	2.74E-19	ARID1B	6q25.3	0.494519769	7.31E-23	4.37E-21
ZNF776	19q13.43	0.474348743	6.31E-21	2.89E-19	PREPL	2p21	0.494450577	7.42E-23	4.42E-21
CHD7	8q12.2	0.474291334	6.39E-21	2.92E-19	ZNF532	18q21.32	0.493954277	8.31E-23	4.93E-21
ANKS1A	6p21.31	0.474158395	6.58E-21	2.99E-19	NCOA2	8q13.3	0.493887933	8.44E-23	4.98E-21

ZMYND11	10p15.3	0.474125901	6.62E-21	3.00E-19	TRIO	5p15.2	0.493817033	8.58E-23	5.04E-21
PRRC2B	9q34.13	0.474074933	6.70E-21	3.03E-19	CTDSPL2	15q15.3-q2	0.493617716	8.98E-23	5.26E-21
CTDSPL2	15q15.3-q2	0.474055001	6.72E-21	3.03E-19	SP3	2q31.1	0.493298238	9.65E-23	5.62E-21
ADD3	10q25.1-q2	0.473844579	7.03E-21	3.17E-19	RASAL2	1q25.2	0.49323645	9.79E-23	5.68E-21
UBE2H	7q32.2	0.473725558	7.21E-21	3.24E-19	ERN1	17q23.3	0.493097497	1.01E-22	5.85E-21
ATXN1	6p22.3	0.473648393	7.33E-21	3.29E-19	CTNND1	11q12.1	0.493068739	1.02E-22	5.87E-21
MGA	15q15.1	0.473541331	7.50E-21	3.35E-19	DDR2	1q23.3	0.492890525	1.06E-22	6.09E-21
ZNF516	18q23	0.473436263	7.67E-21	3.41E-19	SP1	12q13.13	0.492668003	1.11E-22	6.39E-21
DPP8	15q22.31	0.473246057	7.99E-21	3.54E-19	STAM	10p12.33	0.49203873	1.29E-22	7.33E-21
TCF20	22q13.2|22q	0.472900099	8.60E-21	3.80E-19	ADNP	20q13.13	0.491782003	1.36E-22	7.75E-21
MFSD14A	1p21.2	0.472874472	8.65E-21	3.82E-19	AQR	15q14	0.491742139	1.37E-22	7.80E-21
CCNT1	12q13.11-q	0.472395716	9.57E-21	4.21E-19	USP49	6p21.1	0.491606187	1.42E-22	8.02E-21
SLF2	10q24.31	0.472382728	9.60E-21	4.21E-19	PDPR	16q22.1	0.491430635	1.47E-22	8.32E-21
MAGI1	3p14.1	0.471703341	1.11E-20	4.85E-19	DYNC2H1	11q22.3	0.491153936	1.57E-22	8.83E-21
SENP1	12q13.11	0.471586598	1.14E-20	4.95E-19	MYSM1	1p32.1	0.490670381	1.75E-22	9.74E-21
SMG1P3	16p12.2	0.471246334	1.22E-20	5.30E-19	MFHAS1	8p23.1	0.489637632	2.21E-22	1.22E-20
HS3ST3B1	17p12	0.471194563	1.24E-20	5.35E-19	ZBTB26	9q33.2	0.489571181	2.24E-22	1.23E-20
IPMK	10q21.1	0.470655117	1.38E-20	5.97E-19	VRK2	2p16.1	0.48954794	2.25E-22	1.23E-20
ZNF836	19q13.41	0.470281638	1.50E-20	6.43E-19	RUNX1T1	8q21.3	0.489518817	2.27E-22	1.24E-20
BBS1	11q13.2	0.470222128	1.52E-20	6.50E-19	FGD6	12q22	0.489508646	2.27E-22	1.24E-20
CAMSAP2	1q32.1	0.469927992	1.61E-20	6.90E-19	GAS2L3	12q23.1	0.489123753	2.48E-22	1.35E-20
LIMA1	12q13.12	0.469859261	1.64E-20	6.98E-19	RP2	Xp11.3	0.488869122	2.63E-22	1.42E-20
TET2	4q24	0.469826056	1.65E-20	7.02E-19	ZDHHC20	13q12.11	0.488862278	2.63E-22	1.42E-20
PRKAR2A	3p21.31	0.46979986	1.66E-20	7.04E-19	ZNF641	12q13.11	0.488712515	2.72E-22	1.46E-20
MAPK8	10q11.22	0.469719454	1.69E-20	7.15E-19	PTPN12	7q11.23	0.48870739	2.72E-22	1.46E-20
BCOR	Xp11.4	0.469322068	1.83E-20	7.74E-19	HEG1	3q21.2	0.488370259	2.94E-22	1.57E-20
EMSY	11q13.5	0.468787328	2.05E-20	8.62E-19	CSGALNACT2	10q11.21	0.487498388	3.57E-22	1.88E-20
ERCC6L2	9q22.32	0.468656348	2.11E-20	8.84E-19	SF3B1	2q33.1	0.4873489	3.69E-22	1.93E-20
TTBK2	15q15.2	0.468289495	2.28E-20	9.53E-19	MAN1A2	1p12	0.486848613	4.12E-22	2.16E-20
KDM5A	12p13.33	0.468242906	2.30E-20	9.60E-19	FAM171B	2q32.1	0.486821563	4.15E-22	2.16E-20
JRKL	11q21	0.467763975	2.54E-20	1.06E-18	CDYL2	16q23.2	0.486783831	4.18E-22	2.18E-20
RFX7	15q21.3	0.467754009	2.55E-20	1.06E-18	MAP4K5	14q22.1	0.486732724	4.23E-22	2.19E-20
EPHB2	1p36.12	0.467396586	2.75E-20	1.14E-18	KMT2A	11q23.3	0.486545258	4.41E-22	2.28E-20
ITGA6	2q31.1	0.46700945	2.98E-20	1.23E-18	ZBTB11	3q12.3	0.486471049	4.49E-22	2.31E-20
LMTK2	7q21.3	0.466992902	2.99E-20	1.23E-18	RLIM	Xq13.2	0.486293087	4.67E-22	2.39E-20
PROSER3	19q13.12	0.46697884	3.00E-20	1.23E-18	ASH1L	1q22	0.486270023	4.69E-22	2.40E-20
HS3ST3A1	17p12	0.466918903	3.03E-20	1.24E-18	ANOS1	Xp22.31	0.486084184	4.89E-22	2.49E-20
TASOR2	10p15.1	0.466712718	3.17E-20	1.29E-18	ABL1	9q34.12	0.485728449	5.29E-22	2.68E-20
TAOK1	17q11.2	0.466678265	3.19E-20	1.30E-18	GNL3L	Xp11.22	0.485449405	5.63E-22	2.84E-20
NRIP1	21q11.2-q2	0.466064828	3.62E-20	1.46E-18	ARHGEF12	11q23.3	0.485336079	5.77E-22	2.91E-20
ADAM17	2p25.1	0.466054686	3.63E-20	1.46E-18	WAC	10p12.1|10	0.485251796	5.88E-22	2.95E-20
HACD2	3q21.1	0.465241472	4.30E-20	1.73E-18	ZNF37A	10p11.1	0.485231864	5.91E-22	2.96E-20
ARID5B	10q21.2	0.46489893	4.62E-20	1.85E-18	LDB1	10q24.32	0.485211363	5.93E-22	2.96E-20
ROCK1	18q11.1	0.46462615	4.89E-20	1.95E-18	SEPTIN10	2q13	0.48517093	5.99E-22	2.98E-20
PIK3C2A	11p15.1	0.464453598	5.06E-20	2.01E-18	TNKS2	10q23.32	0.484936021	6.31E-22	3.13E-20
ZNF148	3q21.2	0.464337994	5.19E-20	2.05E-18	PCDH17	13q21.1	0.484935736	6.31E-22	3.13E-20
CDK6	7q21.2	0.463795282	5.80E-20	2.28E-18	NHSL2	Xq13.1	0.484922367	6.33E-22	3.13E-20
SIN3A	15q24.2	0.463781045	5.82E-20	2.29E-18	PIK3C2A	11p15.1	0.484408968	7.09E-22	3.50E-20
LRRC37A4P	17q21.31	0.463654621	5.97E-20	2.33E-18	GPATCH2L	14q24.3	0.483949684	7.84E-22	3.84E-20
ZNF354C	5q35.3	0.463376577	6.33E-20	2.46E-18	KAT6B	10q22.2	0.483648715	8.38E-22	4.10E-20
PIKFYVE	2q34	0.463337136	6.38E-20	2.48E-18	CCNT2	2q21.3	0.483387609	8.88E-22	4.31E-20
NUAK2	1q32.1	0.463274494	6.46E-20	2.50E-18	POMK	8p11.21	0.483319395	9.01E-22	4.36E-20
RBM7	11q23.2	0.463123013	6.67E-20	2.58E-18	FUBP1	1p31.1	0.483287666	9.08E-22	4.38E-20
DCAF16	4p15.31	0.462992887	6.85E-20	2.64E-18	SOS1	2p22.1	0.483282256	9.09E-22	4.38E-20
PIAS1	15q23	0.462970108	6.88E-20	2.65E-18	ITSN2	2p23.3	0.483188292	9.28E-22	4.46E-20
TAF8	6p21.1	0.46248178	7.61E-20	2.92E-18	MRE11	11q21	0.483098884	9.46E-22	4.52E-20
SMARCC1	3p21.31	0.462304136	7.89E-20	3.01E-18	CREBBP	16p13.3	0.48251887	1.07E-21	5.12E-20
GRK3	22q12.1	0.462223807	8.02E-20	3.05E-18	NID2	14q22.1	0.482478722	1.08E-21	5.15E-20
TSC22D1	13q14.11	0.462220675	8.03E-20	3.05E-18	HACD2	3q21.1	0.482436581	1.09E-21	5.18E-20
SRPK2	7q22.3	0.461982633	8.43E-20	3.19E-18	CLASP2	3p22.3	0.482408107	1.10E-21	5.20E-20
CHD6	20q12	0.46184357	8.67E-20	3.27E-18	UNC5C	4q22.3	0.481808523	1.26E-21	5.92E-20
HMG20A	15q24.3	0.461558941	9.19E-20	3.46E-18	PDZD2	5p13.3	0.481802752	1.26E-21	5.92E-20
SENP7	3q12.3	0.461442373	9.42E-20	3.53E-18	RFX3	9p24.2	0.481383291	1.38E-21	6.45E-20
KIF21A	12q12	0.461421697	9.46E-20	3.54E-18	PUM1	1p35.2	0.480794593	1.57E-21	7.32E-20
PATL1	11q12.1	0.461216684	9.86E-20	3.68E-18	CHSY1	15q26.3	0.480705401	1.60E-21	7.45E-20
ITPRID2	2q31.3	0.461108768	1.01E-19	3.76E-18	HEATR5B	2p22.2	0.480666358	1.61E-21	7.50E-20
TNPO1	5q13.2	0.461017542	1.03E-19	3.82E-18	CAPRIN1	11p13	0.480496369	1.67E-21	7.73E-20
OSBPL8	12q21.2	0.460781888	1.08E-19	4.00E-18	PFKFB3	10p15.1	0.480486403	1.68E-21	7.73E-20
RALBP1	18p11.22	0.460356772	1.18E-19	4.34E-18	7-Mar	2q24.2	0.480471312	1.68E-21	7.73E-20
ZNF432	19q13.41	0.46001387	1.26E-19	4.64E-18	MYH9	22q12.3	0.48026516	1.76E-21	8.05E-20
BRPF3	6p21.31	0.459937067	1.28E-19	4.70E-18	BICD1	12p11.21	0.480209585	1.78E-21	8.13E-20
TIRAP	11q24.2	0.459914003	1.29E-19	4.72E-18	GCNT4	5q13.3	0.48011471	1.82E-21	8.28E-20
KCTD18	2q33.1	0.459762412	1.33E-19	4.85E-18	PYGO1	15q21.3	0.480010734	1.86E-21	8.45E-20
EBLN2	3p13	0.45953589	1.39E-19	5.05E-18	PIAS1	15q23	0.479861116	1.92E-21	8.71E-20

PRR12	19q13.33	0.459514799	1.40E-19	5.06E-18	BCLAF3	Xp22.12	0.47981869	1.94E-21	8.77E-20
VEZF1	17q22	0.459413717	1.43E-19	5.16E-18	FAM114A1	4p14	0.479701947	1.99E-21	8.98E-20
KMT2D	12q13.12	0.459269069	1.47E-19	5.30E-18	RSRC1	3q25.32	0.479459634	2.10E-21	9.42E-20
MEX3C	18q21.2	0.459264798	1.47E-19	5.30E-18	FOXC2	16q24.1	0.478944248	2.35E-21	1.05E-19
PEAK1	15q24.3	0.458851357	1.60E-19	5.75E-18	MIGA1	1p31.1	0.478805303	2.42E-21	1.08E-19
AQR	15q14	0.457941615	1.92E-19	6.86E-18	SAMD4B	19q13.2	0.478772843	2.43E-21	1.08E-19
CACNB4	2q23.3	0.457724503	2.01E-19	7.15E-18	ATXN1L	16q22.2	0.478727569	2.46E-21	1.09E-19
ACTR2	2p14	0.457683927	2.02E-19	7.18E-18	NFAT5	16q22.1	0.478725576	2.46E-21	1.09E-19
TBCEL	11q23.3	0.457567753	2.07E-19	7.34E-18	CAMSAP2	1q32.1	0.478544767	2.56E-21	1.12E-19
SEPTIN7	7p14.2	0.457242581	2.21E-19	7.83E-18	DHX33	17p13.2	0.478330643	2.68E-21	1.18E-19
UEVLD	11p15.1	0.4570575	2.30E-19	8.10E-18	CFLAR	2q33.1	0.478034514	2.86E-21	1.25E-19
SHPRH	6q24.3	0.456994179	2.33E-19	8.19E-18	ZNF236	18q23	0.477731837	3.05E-21	1.33E-19
BIRC6	2p22.3	0.456556929	2.54E-19	8.92E-18	KMT2C	7q36.1	0.477727566	3.05E-21	1.33E-19
ARL6IP6	2q23.3	0.456248512	2.71E-19	9.47E-18	TLR6	4p14	0.477456726	3.24E-21	1.40E-19
NFXL1	4p12	0.456205276	2.73E-19	9.53E-18	UBR1	15q15.2	0.477455355	3.24E-21	1.40E-19
PLEKHA5	12p12.3	0.456165982	2.75E-19	9.59E-18	FBXO11	2p16.3	0.477399329	3.28E-21	1.42E-19
USP53	4q26	0.456085116	2.80E-19	9.73E-18	CERS6	2q24.3	0.477120787	3.48E-21	1.50E-19
RSBN1	1p13.2	0.45584622	2.93E-19	1.02E-17	DHX36	3q25.2	0.476977563	3.59E-21	1.54E-19
HEATR1	1q43	0.455639785	3.06E-19	1.06E-17	KIF3C	2p23.3	0.476711332	3.80E-21	1.63E-19
SBNO1	12q24.31	0.455344355	3.25E-19	1.12E-17	ANAPC1	2q13	0.476596012	3.90E-21	1.67E-19
POMK	8p11.21	0.455251199	3.31E-19	1.14E-17	PROSER3	19q13.12	0.476575688	3.92E-21	1.67E-19
ADNP2	18q23	0.455247414	3.31E-19	1.14E-17	FOXJ3	1p34.2	0.476445385	4.03E-21	1.71E-19
ARHGAP26	5q31.3	0.4552026	3.34E-19	1.14E-17	NF1	17q11.2	0.476374201	4.09E-21	1.73E-19
PIK3CA	3q26.32	0.455124407	3.39E-19	1.16E-17	PCDHGA11	5q31.3	0.476234385	4.21E-21	1.78E-19
ARL5A	2q23.3	0.454978083	3.49E-19	1.19E-17	ZNF551	19q13.43	0.476153202	4.29E-21	1.81E-19
RGPD3	2q12.2	0.454963814	3.50E-19	1.19E-17	ARL13B	3q11.1-q11	0.476128186	4.31E-21	1.81E-19
SMARCAD1	4q22.3	0.454892946	3.55E-19	1.21E-17	ADCY3	2p23.3	0.476084906	4.35E-21	1.82E-19
ALKBH8	11q22.3	0.454842515	3.59E-19	1.22E-17	ATF1	12q13.12	0.476065544	4.37E-21	1.83E-19
ELK3	12q23.1	0.454756809	3.65E-19	1.24E-17	ARL5B	10p12.31	0.476017992	4.41E-21	1.84E-19
PDGFC	4q32.1	0.454024744	4.23E-19	1.43E-17	LMTK2	7q21.3	0.475912354	4.52E-21	1.88E-19
SPTBN1	2p16.2	0.453859596	4.37E-19	1.48E-17	KCTD10	12q24.11	0.47575692	4.67E-21	1.94E-19
ZNF623	8q24.3	0.453819448	4.41E-19	1.49E-17	INIP	9q32	0.475591487	4.84E-21	2.01E-19
ARIH1	15q24.1	0.453540008	4.66E-19	1.57E-17	ELF1	13q14.11	0.47512505	5.35E-21	2.21E-19
TCP11L2	12q23.3	0.453141199	5.04E-19	1.68E-17	RGP1	9p13.3	0.474716911	5.84E-21	2.41E-19
ZNF587	19q13.43	0.453070584	5.12E-19	1.70E-17	ZNF462	9q31.2	0.47468598	5.87E-21	2.41E-19
ZNF800	7q31.33	0.452943021	5.25E-19	1.74E-17	CAND1	12q14.3-q1	0.474542474	6.06E-21	2.48E-19
TBCK	4q24	0.452903727	5.29E-19	1.76E-17	KIAA1217	10p12.2-p1	0.474421745	6.22E-21	2.54E-19
ANKRD12	18p11.22	0.452613862	5.60E-19	1.86E-17	LAMC1	1q25.3	0.474323008	6.35E-21	2.59E-19
ZFC3H1	12q21.1	0.452508638	5.72E-19	1.89E-17	SMARCA5	4q31.21	0.474291653	6.39E-21	2.60E-19
LRCH1	13q14.13-q1	0.452440741	5.80E-19	1.91E-17	FOSL2	2p23.2	0.473980969	6.83E-21	2.77E-19
CEP295	11q21	0.452349055	5.91E-19	1.94E-17	CARNMT1	9q21.13	0.473754811	7.17E-21	2.90E-19
LOC653653	17q23.1	0.45226234	6.01E-19	1.98E-17	UGCG	9q31.3	0.473663485	7.31E-21	2.95E-19
POLR2A	17p13.1	0.452008792	6.32E-19	2.07E-17	ZNF146	19q13.12	0.473480289	7.60E-21	3.07E-19
NR2F1	5q15	0.451979463	6.36E-19	2.08E-17	MAGI1	3p14.1	0.473456194	7.64E-21	3.07E-19
VHL	3p25.3	0.451952413	6.39E-19	2.09E-17	MED13L	12q24.21	0.473211319	8.05E-21	3.23E-19
FAM161B	14q24.3	0.451738042	6.67E-19	2.17E-17	DDX21	10q22.1	0.473152947	8.15E-21	3.27E-19
ACAP2	3q29	0.451394894	7.14E-19	2.32E-17	TWSG1	18p11.22	0.473107674	8.23E-21	3.29E-19
UTP14C	13q14.3	0.451298083	7.28E-19	2.36E-17	ASAP2	2p25.1|2p2	0.473043323	8.34E-21	3.33E-19
ABL1	9q34.12	0.451200987	7.42E-19	2.40E-17	SMG1	16p12.3	0.472951922	8.51E-21	3.38E-19
ZNF217	20q13.2	0.451174506	7.46E-19	2.41E-17	SMARCAD1	4q22.3	0.472719039	8.94E-21	3.55E-19
HIVEP2	6q24.2	0.451098196	7.57E-19	2.44E-17	SPTBN1	2p16.2	0.472264561	9.85E-21	3.88E-19
VPS41	7p14.1	0.450986009	7.74E-19	2.50E-17	POLR2B	4q12	0.472252033	9.87E-21	3.88E-19
ABLIM1	10q25.3	0.45086471	7.93E-19	2.55E-17	C20ORF194	20p13	0.47218939	1.00E-20	3.92E-19
SLMAP	3p14.3	0.450715791	8.17E-19	2.62E-17	PTAR1	9q21.12	0.472084606	1.02E-20	4.00E-19
DHX9	1q25.3	0.450473763	8.57E-19	2.74E-17	NR2F2	15q26.2	0.471918922	1.06E-20	4.14E-19
SLC39A6	18q12.2	0.449504796	1.04E-18	3.29E-17	SP2	17q21.32	0.471714161	1.11E-20	4.31E-19
NEMF	14q21.3	0.449269633	1.09E-18	3.44E-17	WWC3	Xp22.2	0.471628169	1.13E-20	4.39E-19
ZDHHC20	13q12.11	0.449134607	1.12E-18	3.52E-17	DBR1	3q22.3	0.47143113	1.17E-20	4.56E-19
GPATCH2L	14q24.3	0.449122107	1.12E-18	3.53E-17	SBF2	11p15.4	0.471258293	1.22E-20	4.72E-19
RNF168	3q29	0.449111856	1.12E-18	3.53E-17	RUFY2	10q21.3	0.470482664	1.44E-20	5.52E-19
BBS10	12q21.2	0.449105022	1.12E-18	3.53E-17	PRKAR2A	3p21.31	0.470293028	1.49E-20	5.74E-19
MATR3	5q31.2	0.449056332	1.13E-18	3.56E-17	MSN	Xq12	0.470184542	1.53E-20	5.86E-19
SNX12	Xq13.1	0.448963966	1.15E-18	3.62E-17	REV3L	6q21	0.469911762	1.62E-20	6.20E-19
SETD5	3p25.3	0.448871252	1.18E-18	3.68E-17	DDX6	11q23.3	0.469809541	1.65E-20	6.31E-19
VPS13B	8q22.2	0.448792379	1.19E-18	3.73E-17	RALGAPA2	20p11.23	0.4698047	1.66E-20	6.31E-19
ZNF134	19q13.43	0.448756217	1.20E-18	3.75E-17	BAZ2A	12q13.3	0.469687388	1.70E-20	6.46E-19
ZEB1	10p11.22	0.448522161	1.26E-18	3.92E-17	PDCD6IP	3p22.3	0.469436817	1.79E-20	6.80E-19
MTR	1q43	0.44847404	1.27E-18	3.94E-17	CCP110	16p12.3	0.469396669	1.81E-20	6.83E-19
PKD2	4q22.1	0.448421253	1.28E-18	3.98E-17	PPP1CB	2p23.2	0.469286475	1.85E-20	6.96E-19
PRR14L	22q12.2	0.448378368	1.29E-18	4.01E-17	DENND5A	11p15.4	0.469182545	1.89E-20	7.10E-19
CLOCK	4q12	0.448164529	1.35E-18	4.17E-17	MAPK8	10q11.22	0.46914058	1.90E-20	7.15E-19
PCDHGB7	5q31.3	0.448099229	1.37E-18	4.21E-17	TAF2	8q24.12	0.468703614	2.09E-20	7.82E-19
PUM2	2p24.1	0.447895404	1.42E-18	4.38E-17	ARIH1	15q24.1	0.468567969	2.15E-20	8.00E-19
CDK12	17q12	0.447851031	1.44E-18	4.41E-17	AEBP2	12p12.3	0.468408055	2.22E-20	8.26E-19

CSNK1G1	15q22.31	0.447825405	1.44E-18	4.42E-17	WWC2	4q35.1	0.468247177	2.30E-20	8.51E-19
GSTCD	4q24	0.447499126	1.54E-18	4.71E-17	ETS1	11q24.3	0.468214323	2.31E-20	8.56E-19
BMP4	14q22.2	0.447050662	1.68E-18	5.09E-17	COL8A1	3q12.1	0.468054721	2.39E-20	8.83E-19
ZBTB11	3q12.3	0.44659163	1.84E-18	5.56E-17	SEC23IP	10q26.11-q	0.467999739	2.42E-20	8.92E-19
PRRC2C	1q24.3	0.446553475	1.85E-18	5.59E-17	SYNE2	14q23.2	0.467716993	2.57E-20	9.44E-19
RB1	13q14.2	0.446222925	1.97E-18	5.93E-17	CSF1	1p13.3	0.467526218	2.67E-20	9.81E-19
CREB1	2q33.3	0.446206663	1.98E-18	5.94E-17	ELMSAN1	14q24.3	0.467060561	2.95E-20	1.08E-18
ASH1L	1q22	0.446198975	1.98E-18	5.94E-17	RASSF8	12p12.1	0.467008847	2.98E-20	1.09E-18
EIF3A	10q26.11	0.446106435	2.02E-18	6.04E-17	WAPL	10q23.2	0.46680953	3.10E-20	1.13E-18
TRIP12	2q36.3	0.445988268	2.07E-18	6.17E-17	YLPM1	14q24.3	0.466712434	3.17E-20	1.15E-18
THBS1	15q14	0.445878644	2.11E-18	6.30E-17	ZNF25	10p11.21	0.46665634	3.20E-20	1.16E-18
CD2AP	6p12.3	0.445708259	2.18E-18	6.48E-17	SUSD6	14q24.1	0.466615053	3.23E-20	1.17E-18
QSER1	11p13	0.445610704	2.22E-18	6.60E-17	FAM98B	15q14	0.46635651	3.41E-20	1.23E-18
ZNF704	8q21.13	0.445553646	2.25E-18	6.66E-17	TRUB1	10q25.3	0.466341419	3.42E-20	1.23E-18
CCDC93	2q14.1	0.445514462	2.27E-18	6.70E-17	DENND1B	1q31.3	0.466324619	3.43E-20	1.23E-18
FAM168B	2q21.1	0.445137183	2.44E-18	7.19E-17	ZSWIM4	19p13.13-p	0.466207625	3.52E-20	1.26E-18
SKI	1p36.33-p3	0.445117821	2.45E-18	7.21E-17	IGFBP5	2q35	0.466187375	3.53E-20	1.26E-18
TNKS	8p23.1	0.444987869	2.51E-18	7.38E-17	KIAA1109	4q27	0.466177124	3.54E-20	1.27E-18
SETD2	3p21.31	0.444946977	2.53E-18	7.43E-17	BEND3P3	10q22.3	0.466041104	3.64E-20	1.30E-18
RAPGEF6	5q31.1	0.444689858	2.66E-18	7.80E-17	KDM1B	6p22.3	0.465973569	3.69E-20	1.32E-18
CSPP1	8q13.1-q13.	0.444390028	2.82E-18	8.24E-17	ZNF623	8q24.3	0.465910324	3.74E-20	1.33E-18
ZBTB26	9q33.2	0.444217507	2.91E-18	8.51E-17	PREX2	8q13.2	0.465809983	3.82E-20	1.36E-18
ATP7A	Xq21.1	0.443973454	3.06E-18	8.91E-17	RLF	1p34.2	0.465780767	3.85E-20	1.36E-18
KBTBD4	11p11.2	0.443741961	3.20E-18	9.30E-17	ELK4	1q32.1	0.465744606	3.87E-20	1.37E-18
PBRM1	3p21.1	0.443715481	3.21E-18	9.32E-17	CLCN3	4q33	0.465656336	3.95E-20	1.39E-18
KDM1B	6p22.3	0.443715228	3.21E-18	9.32E-17	CHD9	16q12.2	0.465621314	3.97E-20	1.40E-18
ZNF189	9q31.1	0.443663943	3.24E-18	9.40E-17	MAN2A1	5q21.3	0.465444206	4.12E-20	1.45E-18
RHOBTB1	10q21.2	0.443646859	3.25E-18	9.42E-17	DENND4C	9p22.1	0.465238339	4.30E-20	1.51E-18
NF1	17q11.2	0.44346491	3.37E-18	9.74E-17	TOGARAM1	14q21.2	0.465211574	4.33E-20	1.51E-18
CLASP2	3p22.3	0.442999077	3.69E-18	1.06E-16	PICALM	11q14.2	0.464913452	4.60E-20	1.61E-18
POLI	18q21.2	0.442749076	3.87E-18	1.11E-16	GPRC5B	16p12.3	0.464833156	4.68E-20	1.63E-18
LOX	5q23.1	0.442699816	3.91E-18	1.12E-16	SLC4A7	3p24.1	0.464770513	4.74E-20	1.64E-18
MAST4	5q12.3	0.442599873	3.98E-18	1.14E-16	MFSD14A	1p21.2	0.464752575	4.76E-20	1.65E-18
LMBR1	7q36.3	0.442583674	4.00E-18	1.14E-16	IDE	10q23.33	0.464583615	4.93E-20	1.70E-18
CRIM1	2p22.2	0.442550044	4.02E-18	1.15E-16	TNS1	2q35	0.464499157	5.02E-20	1.73E-18
ADAM9	8p11.22	0.44242305	4.12E-18	1.18E-16	JAK2	9p24.1	0.464414874	5.10E-20	1.75E-18
YY2	Xp22.12	0.442377167	4.16E-18	1.19E-16	PCDH18	4q28.3	0.464345146	5.18E-20	1.77E-18
ZBTB39	12q13.3	0.442368665	4.16E-18	1.19E-16	GLI3	7p14.1	0.464189536	5.35E-20	1.83E-18
NCOR1	17p12-p11.2	0.442283813	4.23E-18	1.20E-16	ZNF426	19p13.2	0.464154185	5.39E-20	1.84E-18
INO80D	2q33.3	0.442087912	4.40E-18	1.25E-16	TOPBP1	3q22.1	0.464003995	5.56E-20	1.89E-18
ARID1B	6q25.3	0.442084495	4.40E-18	1.25E-16	PGM2L1	11q13.4	0.463873015	5.71E-20	1.94E-18
AGPS	2q31.2	0.442059043	4.42E-18	1.25E-16	LOC100190986	16p12.2	0.463821368	5.77E-20	1.96E-18
ZBTB10	8q21.13	0.442056021	4.42E-18	1.25E-16	GOLGB1	3q13.33	0.463466123	6.21E-20	2.10E-18
MGAT5	2q21.2-q21.	0.441732478	4.71E-18	1.33E-16	ADAMTS5	21q21.3	0.463457012	6.22E-20	2.10E-18
CCDC82	11q21	0.441703799	4.73E-18	1.33E-16	ITPR1	3p26.1	0.463359346	6.35E-20	2.14E-18
PROX1	1q32.3	0.441641726	4.79E-18	1.35E-16	SSH1	12q24.11	0.463275348	6.46E-20	2.17E-18
7-Mar	2q24.2	0.441600723	4.83E-18	1.35E-16	DOCK7	1p31.3	0.463258264	6.48E-20	2.18E-18
WDFY1	2q36.1	0.441505621	4.92E-18	1.38E-16	ADAMTS9	3p14.1	0.463223022	6.53E-20	2.19E-18
CXADR	21q21.1	0.441471737	4.95E-18	1.38E-16	NIPBL	5p13.2	0.463214305	6.54E-20	2.19E-18
SSH1	12q24.11	0.441449812	4.97E-18	1.38E-16	ERC1	12p13.33	0.462943627	6.92E-20	2.31E-18
SMNDC1	10q25.2	0.441448388	4.97E-18	1.38E-16	PLCL1	2q33.1	0.462844253	7.06E-20	2.35E-18
MAP4K4	2q11.2	0.441401976	5.01E-18	1.39E-16	FKBP14	7p14.3	0.462368454	7.79E-20	2.58E-18
KIRREL1	1q23.1	0.441266809	5.15E-18	1.43E-16	KCTD20	6p21.31	0.46228047	7.93E-20	2.63E-18
RAB6D	2q21.1	0.441239674	5.17E-18	1.44E-16	USP53	4q26	0.462118738	8.20E-20	2.71E-18
SDE2	1q42.12	0.441179025	5.23E-18	1.45E-16	RBM43	2q23.3	0.461987473	8.42E-20	2.78E-18
TMEM150C	4q21.22	0.441043489	5.37E-18	1.49E-16	QTRT2	3q13.31	0.461934796	8.51E-20	2.80E-18
LINC02035	3q21.1	0.440945539	5.47E-18	1.51E-16	CHD6	20q12	0.461902369	8.57E-20	2.82E-18
UHMK1	1q23.3	0.440800322	5.63E-18	1.55E-16	EIF4G2	11p15.4	0.461840044	8.68E-20	2.85E-18
HECTD2	10q23.32	0.440689874	5.75E-18	1.58E-16	ZBTB44	11q24.3	0.461815524	8.72E-20	2.86E-18
MED13L	12q24.21	0.440557155	5.90E-18	1.62E-16	PDE10A	6q27	0.461683547	8.96E-20	2.93E-18
SNX25	4q35.1	0.440223614	6.28E-18	1.72E-16	MIS18BP1	14q21.2	0.461650057	9.02E-20	2.94E-18
ZFHX3	16q22.2-q2	0.440220624	6.29E-18	1.72E-16	BDP1	5q13.2	0.46164664	9.03E-20	2.94E-18
KLF3	4p14	0.440165354	6.35E-18	1.73E-16	LEMD3	12q14.3	0.461379303	9.54E-20	3.08E-18
KIAA1109	4q27	0.44012008	6.41E-18	1.74E-16	ZNF460	19q13.43	0.461191627	9.91E-20	3.20E-18
DNAJC14	12q13.2	0.43995664	6.61E-18	1.80E-16	ZNRF2P1	7p14.3	0.461094247	1.01E-19	3.25E-18
STAM2	2q23.3	0.439888872	6.70E-18	1.82E-16	SLMAP	3p14.3	0.460784735	1.08E-19	3.46E-18
SNORD116-4	15q11.2	0.439839846	6.76E-18	1.83E-16	KANSL1	17q21.31	0.46064806	1.11E-19	3.55E-18
MED1	17q12	0.439777539	6.84E-18	1.85E-16	BCOR	Xp11.4	0.460587411	1.12E-19	3.59E-18
DNAJB14	4q23	0.439550033	7.15E-18	1.93E-16	SNX12	Xq13.1	0.46052836	1.14E-19	3.62E-18
KMT5B	11q13.2	0.439487105	7.23E-18	1.95E-16	KIF13A	6p22.3	0.460380975	1.17E-19	3.73E-18
TSC22D2	3q25.1	0.439393995	7.36E-18	1.98E-16	AKT3	1q43-q44	0.460290603	1.19E-19	3.79E-18
ZNF143	11p15.4	0.439307039	7.49E-18	2.01E-16	ZFP91	11q12.1	0.459910871	1.29E-19	4.08E-18
UTP20	12q23.2	0.439217173	7.62E-18	2.04E-16	ZFR	5p13.3	0.459837408	1.31E-19	4.13E-18
NEK7	1q31.3	0.43902298	7.90E-18	2.11E-16	YY2	Xp22.12	0.459547985	1.39E-19	4.35E-18

TRIB2	2p24.3	0.438900859	8.09E-18	2.16E-16	HIPK1	1p13.2	0.459523911	1.39E-19	4.37E-18
G3BP2	4q21.1	0.438599858	8.56E-18	2.28E-16	KIF2A	5q12.1	0.459236894	1.48E-19	4.62E-18
MED12	Xq13.1	0.438466316	8.78E-18	2.33E-16	ZNF432	19q13.41	0.458965175	1.56E-19	4.88E-18
RBFOX2	22q12.3	0.438423605	8.86E-18	2.35E-16	NSD3	8p11.23	0.458854664	1.60E-19	4.98E-18
TTC30A	2q31.2	0.438262158	9.13E-18	2.41E-16	IPP	1p34.1	0.458827723	1.61E-19	5.00E-18
C10ORF12	10q24.1	0.437945528	9.70E-18	2.55E-16	DDX3X	Xp11.4	0.458736892	1.64E-19	5.08E-18
GAS2L3	12q23.1	0.437859168	9.86E-18	2.59E-16	EP300	22q13.2	0.458637233	1.67E-19	5.18E-18
IL6ST	5q11.2	0.4376158	1.03E-17	2.71E-16	TOP1	20q12	0.458560813	1.69E-19	5.25E-18
CCNT2	2q21.3	0.437463464	1.06E-17	2.79E-16	CEP135	4q12	0.458518782	1.71E-19	5.29E-18
PPFIA1	11q13.3	0.437367823	1.08E-17	2.83E-16	SEPTIN2	2q37.3	0.458285328	1.79E-19	5.51E-18
SON	21q22.11	0.437329637	1.09E-17	2.84E-16	CDK12	17q12	0.458269635	1.80E-19	5.52E-18
WDR11	10q26.12	0.437318247	1.09E-17	2.84E-16	CRYBG3	3q11.2	0.458214194	1.82E-19	5.57E-18
RBBP5	1q32.1	0.437263578	1.10E-17	2.87E-16	RASEF	9q21.32	0.458109571	1.86E-19	5.67E-18
RNF43	17q22	0.4371568	1.13E-17	2.92E-16	RSBN1	1p13.2	0.458097937	1.86E-19	5.67E-18
TNRC18	7p22.1	0.437005889	1.16E-17	3.00E-16	ANKRD50	4q28.1	0.457905169	1.94E-19	5.88E-18
PHC3	3q26.2	0.436784077	1.21E-17	3.13E-16	PIK3R4	3q22.1	0.457693892	2.02E-19	6.13E-18
ARID1A	1p36.11	0.436737553	1.22E-17	3.14E-16	COPB2	3q23	0.457689654	2.02E-19	6.13E-18
ZFP91	11q12.1	0.436716024	1.22E-17	3.15E-16	NPEPPS	17q21.32	0.457397512	2.15E-19	6.46E-18
GNAI1	7q21.11	0.436417222	1.29E-17	3.33E-16	MED14	Xp11.4	0.457289311	2.19E-19	6.58E-18
DCDC2	6p22.3	0.436409736	1.30E-17	3.33E-16	CAB39	2q37.1	0.457236886	2.22E-19	6.64E-18
ANKRD26	10p12.1	0.436284075	1.33E-17	3.40E-16	RBM12	20q11.22	0.457198162	2.23E-19	6.69E-18
CSNK2A1	20p13	0.436275533	1.33E-17	3.40E-16	ACVR1	2q24.1	0.457162854	2.25E-19	6.72E-18
UBR1	15q15.2	0.436177298	1.35E-17	3.46E-16	PXDN	2p25.3	0.456988594	2.33E-19	6.94E-18
RBMS1	2q24.2	0.435966022	1.41E-17	3.59E-16	CHM	Xq21.2	0.456749128	2.45E-19	7.27E-18
SLC16A7	12q14.1	0.435823314	1.45E-17	3.68E-16	ACTR3	2q14.1	0.456711148	2.46E-19	7.31E-18
VGLL4	3p25.3-p25.	0.435712888	1.48E-17	3.75E-16	ZNF776	19q13.43	0.456699616	2.47E-19	7.32E-18
HEATR5B	2p22.2	0.435451498	1.55E-17	3.93E-16	DZIP1	13q32.1	0.456461257	2.59E-19	7.67E-18
RIC1	9p24.1	0.435420177	1.56E-17	3.95E-16	CD93	20p11.21	0.456157155	2.76E-19	8.11E-18
ZNF518A	10q24.1	0.435352124	1.58E-17	4.00E-16	KRBA2	17p13.1	0.456154308	2.76E-19	8.11E-18
ITGA8	10p13	0.435261015	1.61E-17	4.06E-16	APBB2	4p14-p13	0.456042975	2.82E-19	8.26E-18
DNAJC13	3q22.1	0.434972282	1.70E-17	4.27E-16	CPLANE1	5p13.2	0.455946449	2.88E-19	8.39E-18
FRYL	4p11	0.434955656	1.71E-17	4.28E-16	ESYT2	7q36.3	0.455917405	2.89E-19	8.43E-18
USP6NL	10p14	0.434802008	1.76E-17	4.40E-16	DLG1	3q29	0.455599352	3.08E-19	8.95E-18
SNX6	14q13.1	0.434751894	1.77E-17	4.44E-16	NFKB1	4q24	0.455507129	3.14E-19	9.10E-18
REL	2p16.1	0.434666188	1.80E-17	4.50E-16	NBPF10	1q21.1	0.455468119	3.17E-19	9.16E-18
BCLAF3	Xp22.12	0.434499046	1.86E-17	4.63E-16	SPICE1	3q13.2	0.455347357	3.24E-19	9.37E-18
PTPN9	15q24.2	0.434278942	1.94E-17	4.82E-16	RABGAP1	9q33.2-q33	0.455316321	3.26E-19	9.42E-18
PWAR5	15q11.2	0.433976556	2.05E-17	5.10E-16	GUCY1B1	4q32.1	0.455231753	3.32E-19	9.55E-18
RBM12B	8q22.1	0.433887711	2.08E-17	5.17E-16	RBM12B	8q22.1	0.455089669	3.42E-19	9.81E-18
KRBA2	17p13.1	0.433887141	2.08E-17	5.17E-16	TRIM56	7q22.1	0.455036138	3.45E-19	9.91E-18
MAN2A1	5q21.3	0.433648245	2.18E-17	5.40E-16	ZEB2	2q22.3	0.454878392	3.56E-19	1.02E-17
SCYL2	12q23.1	0.433369201	2.30E-17	5.68E-16	SFMBT2	10p14	0.45487726	3.56E-19	1.02E-17
ZNF765	19q13.42	0.433059405	2.43E-17	6.00E-16	CMTM1	16q21	0.454794394	3.62E-19	1.03E-17
CRKL	22q11.21	0.433034348	2.44E-17	6.02E-16	FRMD4B	3p14.1	0.454516489	3.83E-19	1.09E-17
RCOR1	14q32.31-q3	0.432964302	2.48E-17	6.09E-16	LNPEP	5q15	0.45447979	3.86E-19	1.10E-17
CCNI	4q21.1	0.432648811	2.63E-17	6.45E-16	TSC22D2	3q25.1	0.454004243	4.25E-19	1.20E-17
USF3	3q13.2	0.432636852	2.63E-17	6.45E-16	BAZ2B	2q24.2	0.453631804	4.57E-19	1.29E-17
ZNF813	19q13.42	0.432619897	2.64E-17	6.46E-16	NOTCH2	1p12	0.453554103	4.65E-19	1.31E-17
ABL2	1q25.2	0.432559972	2.67E-17	6.52E-16	HDGFL3	15q25.2	0.453424514	4.77E-19	1.34E-17
SECISBP2L	15q21.1	0.432373753	2.76E-17	6.74E-16	NEMF	14q21.3	0.453340264	4.85E-19	1.36E-17
TRIO	5p15.2	0.432140267	2.89E-17	7.03E-16	PIKFYVE	2q34	0.453150026	5.04E-19	1.41E-17
KLHL28	14q21.2	0.432046018	2.94E-17	7.13E-16	SMG1P2	16p11.2	0.453053499	5.13E-19	1.44E-17
RALGPS2	1q25.2	0.431727395	3.12E-17	7.54E-16	GSTCD	4q24	0.452995872	5.19E-19	1.45E-17
SPOPL	2q22.1	0.431577511	3.21E-17	7.75E-16	PRRC2C	1q24.3	0.452962952	5.23E-19	1.46E-17
PRKD3	2p22.2	0.431467602	3.27E-17	7.90E-16	BRWD3	Xq21.1	0.452950709	5.24E-19	1.46E-17
FAM13B	5q31.2	0.431423863	3.30E-17	7.95E-16	UBE2H	7q32.2	0.45286187	5.33E-19	1.48E-17
TGS1	8q12.1	0.431316405	3.36E-17	8.10E-16	MDFIC	7q31.1-q31	0.452694728	5.51E-19	1.53E-17
WDFY3	4q21.23	0.431020846	3.55E-17	8.54E-16	STAM2	2q23.3	0.452581402	5.64E-19	1.56E-17
SMAD5	5q31.1	0.430716175	3.76E-17	9.02E-16	RBMS2	12q13.3	0.452336811	5.92E-19	1.63E-17
KIAA0753	17p13.1	0.430470272	3.94E-17	9.41E-16	AGO4	1p34.3	0.452232881	6.04E-19	1.67E-17
ZNF407	18q22.3	0.430276365	4.08E-17	9.74E-16	PPFIBP1	12p11.23-p	0.452208394	6.07E-19	1.67E-17
SMCHD1	18p11.32	0.430087298	4.22E-17	1.01E-15	PCNP	3q12.3	0.452106172	6.20E-19	1.70E-17
ZMYM2	13q12.11	0.429942081	4.34E-17	1.03E-15	PTCH1	9q22.32	0.452058938	6.26E-19	1.72E-17
KAT6A	8p11.21	0.42992158	4.36E-17	1.04E-15	WRN	8p12	0.451827413	6.55E-19	1.79E-17
LATS1	6q25.1	0.429809393	4.45E-17	1.05E-15	FAR2	12p11.22	0.451615852	6.83E-19	1.86E-17
NIPAL2	8q22.2	0.429731974	4.51E-17	1.07E-15	XRN2	20p11.22	0.451399165	7.13E-19	1.93E-17
FNDC3B	3q26.31	0.429685247	4.55E-17	1.08E-15	RASSF3	12q14.2	0.451352826	7.20E-19	1.95E-17
ZNF146	19q13.12	0.429656234	4.57E-17	1.08E-15	NUP153	6p22.3	0.451286014	7.29E-19	1.97E-17
PDS5B	13q13.1	0.429629722	4.60E-17	1.08E-15	SYNJ1	21q22.11	0.451239427	7.36E-19	1.99E-17
PCDHGB5	5q31.3	0.429467522	4.74E-17	1.11E-15	DCUN1D1	3q26.33	0.45113151	7.52E-19	2.03E-17
SYNRG	17q12	0.429216851	4.96E-17	1.16E-15	CNTRL	9q33.2	0.451087504	7.59E-19	2.04E-17
PDPK1	16p13.3	0.429049709	5.12E-17	1.20E-15	BMPR1A	10q23.2	0.450995975	7.73E-19	2.08E-17
MACF1	1p34.3	0.428837009	5.32E-17	1.25E-15	PHF20	20q11.22-q	0.450834243	7.98E-19	2.14E-17
SMARCA5	4q31.21	0.428143984	6.04E-17	1.41E-15	SLC39A6	18q12.2	0.450786976	8.05E-19	2.16E-17

ICE2	15q22.2	0.428100277	6.09E-17	1.42E-15	TBC1D9	4q31.21	0.450769322	8.08E-19	2.16E-17
ZNF791	19p13.13	0.428055115	6.14E-17	1.43E-15	ABI1	10p12.1	0.450748426	8.11E-19	2.17E-17
RNF44	5q35.2	0.427938087	6.28E-17	1.46E-15	ITGA9	3p22.2	0.450723764	8.15E-19	2.17E-17
NFATC2	20q13.2	0.427904993	6.31E-17	1.47E-15	TBC1D23	3q12.1-q12	0.450398307	8.70E-19	2.31E-17
CCP110	16p12.3	0.42726411	7.10E-17	1.64E-15	RBBP5	1q32.1	0.450324275	8.82E-19	2.34E-17
INTS2	17q23.2	0.427088995	7.33E-17	1.69E-15	QSER1	11p13	0.450174787	9.09E-19	2.41E-17
SGPL1	10q22.1	0.426975385	7.49E-17	1.72E-15	EXOC1	4q12	0.450166814	9.10E-19	2.41E-17
AHR	7p21.1	0.426943951	7.53E-17	1.73E-15	SLC12A6	15q14	0.450107589	9.21E-19	2.43E-17
LTN1	21q21.3	0.426770657	7.77E-17	1.78E-15	SPTY2D1	11p15.1	0.449534978	1.03E-18	2.71E-17
TASOR	3p14.3	0.426749586	7.80E-17	1.79E-15	ZNF430	19p12	0.449523914	1.03E-18	2.71E-17
ZNF420	19q13.12	0.426719119	7.85E-17	1.79E-15	FMN1	15q13.3	0.449497015	1.04E-18	2.72E-17
ELK4	1q32.1	0.426660463	7.93E-17	1.81E-15	NEU3	11q13.4	0.449439875	1.05E-18	2.75E-17
BRD1	22q13.33	0.426330166	8.42E-17	1.92E-15	NPHP3	3q22.1	0.449340217	1.07E-18	2.80E-17
KLHL23	2q31.1	0.42632895	8.43E-17	1.92E-15	SRGAP1	12q14.2	0.449288964	1.08E-18	2.82E-17
TRRAP	7q22.1	0.426246737	8.55E-17	1.94E-15	VPS13B	8q22.2	0.449038678	1.14E-18	2.96E-17
PAPOLG	2p16.1	0.426231077	8.58E-17	1.95E-15	KLHL24	3q27.1	0.449005648	1.14E-18	2.97E-17
BTRC	10q24.32	0.42617003	8.67E-17	1.96E-15	KIT	4q12	0.448961261	1.15E-18	3.00E-17
PPP4R3B	2p16.1	0.426005166	8.94E-17	2.02E-15	ANKRD26	10p12.1	0.448914817	1.17E-18	3.02E-17
PIGN	18q21.33	0.425927829	9.07E-17	2.05E-15	TNPO1	5q13.2	0.448758812	1.20E-18	3.11E-17
SNX30	9q32	0.425806246	9.27E-17	2.09E-15	KDM3B	5q31.2	0.448605337	1.24E-18	3.20E-17
LUZP1	1p36.12	0.425711997	9.43E-17	2.12E-15	RGS7BP	5q12.3	0.448298789	1.32E-18	3.39E-17
PRRG1	Xp21.1	0.425673273	9.50E-17	2.13E-15	KDM6A	Xp11.3	0.448128082	1.36E-18	3.49E-17
POLR2M	15q21.3	0.425639958	9.55E-17	2.14E-15	ALDH1L2	12q23.3	0.448125979	1.36E-18	3.49E-17
ESF1	20p12.1	0.425536028	9.74E-17	2.17E-15	INPP4A	2q11.2	0.447884061	1.43E-18	3.65E-17
CARF	2q33.2	0.425472247	9.85E-17	2.20E-15	DOCK5	8p21.2	0.447294651	1.60E-18	4.10E-17
NEK9	14q24.3	0.425418889	9.95E-17	2.22E-15	RPGRIP1L	16q12.2	0.447171074	1.64E-18	4.19E-17
KCTD20	6p21.31	0.425363477	1.00E-16	2.24E-15	HUWE1	Xp11.22	0.447128364	1.65E-18	4.22E-17
SAMD4B	19q13.2	0.424749579	1.12E-16	2.50E-15	TJP1	15q13.1	0.44701567	1.69E-18	4.30E-17
ZNF235	19q13.31	0.424718827	1.13E-16	2.51E-15	C18ORF25	18q21.1	0.446980584	1.70E-18	4.32E-17
SMAD7	18q21.1	0.424715695	1.13E-16	2.51E-15	ZNF699	19p13.2	0.446975773	1.70E-18	4.32E-17
SNORD116-28	15q11.2	0.424643145	1.15E-16	2.54E-15	KRR1	12q21.2	0.446927907	1.72E-18	4.36E-17
ELF2	4q31.1	0.424632836	1.15E-16	2.54E-15	PIK3CG	7q22.3	0.446874092	1.74E-18	4.39E-17
YTHDC1	4q13.2	0.424619341	1.15E-16	2.54E-15	MED12	Xq13.1	0.446586505	1.84E-18	4.63E-17
TMOD3	15q21.2	0.424582152	1.16E-16	2.56E-15	CCN4	8q24.22	0.446375331	1.92E-18	4.82E-17
TRPM7	15q21.2	0.424490182	1.18E-16	2.59E-15	FBXO21	12q24.22	0.446298349	1.94E-18	4.88E-17
NFE2L2	2q31.2	0.424336138	1.21E-16	2.66E-15	MMP16	8q21.3	0.446129614	2.01E-18	5.04E-17
SNURF	15q11.2	0.424149187	1.25E-16	2.75E-15	ARHGEF17	11q13.4	0.446023006	2.05E-18	5.14E-17
TOP1	20q12	0.424051998	1.27E-16	2.80E-15	PRRC2B	9q34.13	0.445841912	2.13E-18	5.30E-17
TOX3	16q12.1	0.424018144	1.28E-16	2.81E-15	MOB1B	4q13.3	0.44581404	2.14E-18	5.32E-17
ACTN1	14q24.1|14q	0.423991034	1.29E-16	2.82E-15	NEO1	15q24.1	0.445694417	2.19E-18	5.44E-17
TNRC6B	22q13.1	0.423852366	1.32E-16	2.89E-15	COL6A3	2q37.3	0.445197832	2.41E-18	5.97E-17
KRIT1	7q21.2	0.423720247	1.35E-16	2.96E-15	DHX9	1q25.3	0.445059449	2.48E-18	6.13E-17
WDR35	2p24.1	0.423520645	1.40E-16	3.06E-15	APPBP2	17q23.2	0.445030722	2.49E-18	6.16E-17
FAM107B	10p13	0.423479643	1.41E-16	3.08E-15	POLI	18q21.2	0.445007911	2.50E-18	6.18E-17
NPNT	4q24	0.423311931	1.46E-16	3.17E-15	ITGA4	2q31.3	0.444985987	2.51E-18	6.19E-17
N4BP2	4p14	0.423305128	1.46E-16	3.17E-15	GTF2I	7q11.23	0.444937011	2.53E-18	6.25E-17
PHLDA1	12q21.2	0.423163867	1.50E-16	3.25E-15	AKAP11	13q14.11	0.444894585	2.56E-18	6.28E-17
LDLRAD3	11p13	0.42314868	1.50E-16	3.25E-15	PRRG1	Xp21.1	0.444843617	2.58E-18	6.34E-17
RNF41	12q13.3	0.423054812	1.53E-16	3.30E-15	POGLUT3	11q22.3	0.444835075	2.59E-18	6.34E-17
TBC1D4	13q22.2	0.423027477	1.53E-16	3.32E-15	TUT4	1p32.3	0.444752501	2.63E-18	6.43E-17
PRDM10	11q24.3	0.422861474	1.58E-16	3.41E-15	RNF4	4p16.3	0.444698685	2.65E-18	6.49E-17
TPR	1q31.1	0.422848122	1.58E-16	3.41E-15	RERE	1p36.23	0.44450193	2.76E-18	6.73E-17
MOB1A	2p13.1	0.422666713	1.64E-16	3.52E-15	ZMYM1	1p34.3	0.444490145	2.76E-18	6.74E-17
KCTD10	12q24.11	0.422238637	1.77E-16	3.79E-15	DUSP18	22q12.2	0.444474026	2.77E-18	6.75E-17
STT3B	3p23	0.422206574	1.78E-16	3.81E-15	KPNA4	3q25.33	0.444300509	2.87E-18	6.97E-17
SMG1	16p12.3	0.421964831	1.86E-16	3.97E-15	SIN3A	15q24.2	0.444244526	2.90E-18	7.03E-17
RAD54L2	3p21.2	0.42170942	1.95E-16	4.14E-15	FGFR1	8p11.23	0.444192703	2.93E-18	7.08E-17
KLF7	2q33.3	0.421648273	1.97E-16	4.18E-15	TRIM32	9q33.1	0.444192703	2.93E-18	7.08E-17
EYA3	1p35.3	0.421408735	2.05E-16	4.36E-15	EPHA3	3p11.1	0.4441654	2.94E-18	7.11E-17
GTF2H3	12q24.31	0.42135435	2.07E-16	4.40E-15	GK5	3q23	0.444142479	2.96E-18	7.13E-17
ATXN7	3p14.1	0.421220807	2.12E-16	4.50E-15	LTBP1	2p22.3	0.44410358	2.98E-18	7.17E-17
ZKSCAN1	7q22.1	0.42105395	2.19E-16	4.63E-15	TET2	4q24	0.443849308	3.13E-18	7.52E-17
RERE	1p36.23	0.420989315	2.21E-16	4.68E-15	LIMA1	12q13.12	0.443745125	3.19E-18	7.65E-17
BARD1	2q35	0.420932084	2.24E-16	4.72E-15	NAV1	1q32.1	0.443557166	3.31E-18	7.92E-17
GALNT1	18q12.2	0.420605771	2.37E-16	4.99E-15	RAB10	2p23.3	0.443405969	3.41E-18	8.14E-17
LMO7	13q22.2	0.420600646	2.37E-16	4.99E-15	PLS1	3q23	0.443202381	3.55E-18	8.46E-17
MYCBP2	13q22.3	0.420378264	2.47E-16	5.18E-15	PTPN9	15q24.2	0.4430882	3.63E-18	8.64E-17
BBX	3q13.12	0.420241305	2.53E-16	5.31E-15	TGFBR2	3p24.1	0.44307197	3.64E-18	8.65E-17
TEAD1	11p15.3	0.420038286	2.63E-16	5.50E-15	ZNF765	19q13.42	0.443033531	3.66E-18	8.71E-17
CDYL	6p25.1	0.420014428	2.64E-16	5.51E-15	NCOA1	2p23.3	0.442921628	3.74E-18	8.88E-17
ROR1	1p31.3	0.420009676	2.64E-16	5.51E-15	COL12A1	6q13-q14.1	0.442772456	3.85E-18	9.12E-17
KDM2B	12q24.31	0.420001555	2.64E-16	5.52E-15	SH3RF1	4q32.3-q33	0.442766445	3.86E-18	9.12E-17
CDK8	13q12.13	0.419851047	2.71E-16	5.66E-15	GPATCH11	2p22.2	0.442527264	4.04E-18	9.53E-17
FRMD3	9q21.32	0.419730363	2.77E-16	5.77E-15	CEP85L	6q22.31	0.442505436	4.06E-18	9.56E-17

PCDHGB6	5q31.3	0.419725005	2.78E-16	5.77E-15	TSHZ3	19q12	0.442447822	4.10E-18	9.66E-17
REV3L	6q21	0.419601781	2.84E-16	5.88E-15	SLC25A24	1p13.3	0.442266443	4.25E-18	9.99E-17
DHX15	4p15.2	0.419473648	2.90E-16	6.00E-15	SYNJ2	6q25.3	0.442246227	4.26E-18	1.00E-16
CAPRIN1	11p13	0.419448591	2.92E-16	6.01E-15	MEX3B	15q25.2	0.44198142	4.49E-18	1.05E-16
SNX19	11q24.3-q25	0.4194257	2.93E-16	6.03E-15	FAM161B	14q24.3	0.441902434	4.56E-18	1.06E-16
ZNF827	4q31.21-q31	0.419373721	2.96E-16	6.08E-15	TGFB2	1q41	0.441833925	4.62E-18	1.08E-16
MIS18BP1	14q21.2	0.419301666	2.99E-16	6.15E-15	KLHL9	9p21.3	0.441684152	4.75E-18	1.10E-16
NIPBL	5p13.2	0.419255568	3.02E-16	6.20E-15	CTTNBP2	7q31.31	0.441673646	4.76E-18	1.11E-16
ITSN2	2p23.3	0.418703144	3.33E-16	6.81E-15	MBD5	2q23.1	0.441649303	4.78E-18	1.11E-16
RBM26	13q31.1	0.418628258	3.38E-16	6.90E-15	CNNM4	2q11.2	0.441646566	4.78E-18	1.11E-16
TBC1D12	10q23.33	0.418598645	3.39E-16	6.93E-15	LIMCH1	4p13	0.441592782	4.83E-18	1.12E-16
ZNF41	Xp11.3	0.418439761	3.49E-16	7.11E-15	ZNF292	6q14.3	0.441592181	4.84E-18	1.12E-16
MSL1	17q21.1	0.418437483	3.49E-16	7.11E-15	BAG4	8p11.23	0.441585917	4.84E-18	1.12E-16
SLC38A11	2q24.3	0.418397161	3.52E-16	7.15E-15	CDH11	16q21	0.441402545	5.01E-18	1.16E-16
RANBP2	2q13	0.418256104	3.61E-16	7.32E-15	MECOM	3q26.2	0.441376634	5.04E-18	1.16E-16
CEP97	3q12.3	0.417857469	3.87E-16	7.84E-15	UPF2	10p14	0.44130904	5.11E-18	1.17E-16
ZNF543	19q13.43	0.417852629	3.87E-16	7.84E-15	PCDHGA6	5q31.3	0.441272215	5.14E-18	1.18E-16
SUSD6	14q24.1	0.417733323	3.96E-16	8.00E-15	RASA1	5q14.3	0.441110972	5.30E-18	1.21E-16
PDE3A	12p12.2	0.417587156	4.06E-16	8.18E-15	NEK7	1q31.3	0.441043774	5.37E-18	1.23E-16
SHOC2	10q25.2	0.417434804	4.17E-16	8.38E-15	ZSWIM6	5q12.1	0.441010459	5.41E-18	1.24E-16
ERN1	17q23.3	0.41692865	4.56E-16	9.16E-15	USP12	13q12.13	0.440858013	5.57E-18	1.27E-16
NR2F2	15q26.2	0.416891379	4.59E-16	9.21E-15	ZNF354C	5q35.3	0.440833215	5.59E-18	1.27E-16
PLEKHA1	10q26.13	0.416860882	4.62E-16	9.25E-15	SRRM2	16p13.3	0.44055687	5.90E-18	1.34E-16
METAP1	4q23	0.416839242	4.64E-16	9.27E-15	PEAR1	1q23.1	0.440498561	5.96E-18	1.35E-16
TOR1AIP2	1q25.2	0.416380243	5.03E-16	1.00E-14	ZC3HAV1L	7q34	0.440383911	6.09E-18	1.38E-16
ZC3H12C	11q22.3	0.416331837	5.07E-16	1.01E-14	MLXIP	12q24.31	0.440293202	6.20E-18	1.40E-16
USP49	6p21.1	0.416008822	5.37E-16	1.07E-14	THUMPD1	16p12.3	0.439935284	6.64E-18	1.49E-16
GABPA	21q21.3	0.415912701	5.46E-16	1.09E-14	RAI1	17p11.2	0.439805159	6.81E-18	1.53E-16
ZNF318	6p21.1	0.415836961	5.53E-16	1.10E-14	SASH1	6q24.3-q25	0.439673609	6.98E-18	1.56E-16
PDPR	16q22.1	0.415549374	5.82E-16	1.16E-14	PUS7L	12q12	0.439342204	7.44E-18	1.66E-16
ZNF106	15q15.1	0.41545541	5.92E-16	1.17E-14	COG5	7q22.3	0.439324978	7.46E-18	1.67E-16
BTBD3	20p12.2	0.415356606	6.02E-16	1.19E-14	NUFIP2	17q11.2	0.439244507	7.58E-18	1.69E-16
CARNMT1	9q21.13	0.415334598	6.05E-16	1.20E-14	RTL6	22q13.31	0.439231979	7.59E-18	1.69E-16
ZNF430	19p12	0.414839708	6.60E-16	1.30E-14	ATM	11q22.3	0.439178163	7.67E-18	1.71E-16
REV1	2q11.2	0.414785419	6.66E-16	1.31E-14	PCNX4	14q23.1	0.43914371	7.72E-18	1.72E-16
FKBP9P1	7p11.2	0.414758095	6.69E-16	1.32E-14	SLC25A40	7q21.12	0.43901273	7.92E-18	1.76E-16
TNFAIP3	6q23.3	0.414656432	6.81E-16	1.34E-14	SEC14L1	17q25.2-q2	0.438969165	7.98E-18	1.77E-16
OXR1	8q23.1	0.414621979	6.85E-16	1.35E-14	NFATC2	20q13.2	0.438937828	8.03E-18	1.78E-16
ITGB1	10p11.22	0.414616284	6.86E-16	1.35E-14	WDR11	10q26.12	0.438858401	8.15E-18	1.80E-16
ACER2	9p22.1	0.414602058	6.88E-16	1.35E-14	LINC00472	6q13	0.438810046	8.23E-18	1.82E-16
MAGI3	1p13.2	0.414498402	7.00E-16	1.37E-14	PDGFD	11q22.3	0.438639722	8.50E-18	1.87E-16
URB1	21q22.11	0.414444871	7.07E-16	1.38E-14	YME1L1	10p12.1	0.438571384	8.61E-18	1.89E-16
SNORD116-20	15q11.2	0.414411506	7.11E-16	1.39E-14	ARHGAP5	14q12	0.438400541	8.90E-18	1.95E-16
UTRN	6q24.2	0.414235873	7.33E-16	1.43E-14	VEZF1	17q22	0.438323376	9.03E-18	1.98E-16
TSHZ1	18q22.3	0.414174084	7.41E-16	1.45E-14	CCAR1	10q21.3	0.438184139	9.27E-18	2.03E-16
TGFB2	1q41	0.413957113	7.70E-16	1.49E-14	HSPG2	1p36.12	0.438131462	9.36E-18	2.05E-16
SNHG4	5q31.2	0.413706962	8.05E-16	1.55E-14	ARHGAP42	11q22.1	0.437983967	9.63E-18	2.10E-16
HUNK	21q22.11	0.413618959	8.17E-16	1.58E-14	KDM5A	12p13.33	0.43790481	9.77E-18	2.13E-16
MFAP3	5q33.2	0.413448	8.42E-16	1.62E-14	PHF2	9q22.31	0.437583055	1.04E-17	2.26E-16
SF3B1	2q33.1	0.413227896	8.75E-16	1.68E-14	RAB11FIP2	10q26.11	0.437514433	1.05E-17	2.29E-16
CCNG2	4q21.1	0.413215967	8.77E-16	1.68E-14	CDC42EP3	2p22.2	0.437367507	1.08E-17	2.35E-16
CHSY1	15q26.3	0.412956142	9.17E-16	1.76E-14	SNX6	14q13.1	0.437345013	1.09E-17	2.36E-16
UBN2	7q34	0.412683475	9.62E-16	1.84E-14	CASTOR2	7q11.23	0.437233521	1.11E-17	2.40E-16
FAM8A1	6p22.3	0.412340934	1.02E-15	1.95E-14	SP4	7p15.3	0.437087039	1.14E-17	2.47E-16
ZNF284	19q13.31	0.412156812	1.05E-15	2.01E-14	PRPF4B	6p25.2	0.436882312	1.19E-17	2.56E-16
DYRK1A	21q22.13	0.411849646	1.11E-15	2.11E-14	SLC16A7	12q14.1	0.43686774	1.19E-17	2.57E-16
SVIL	10p11.23	0.411796513	1.12E-15	2.13E-14	PLA2R1	2q24.2	0.436565317	1.26E-17	2.71E-16
FRS2	12q15	0.411714508	1.14E-15	2.16E-14	KLHL20	1q25.1	0.436392561	1.30E-17	2.80E-16
EPG5	18q12.3-q21	0.411680339	1.15E-15	2.17E-14	DYNC1LI2	16q22.1	0.436085612	1.38E-17	2.95E-16
GCNT4	5q13.3	0.411558927	1.17E-15	2.22E-14	SECISBP2L	15q21.1	0.435957764	1.41E-17	3.02E-16
UBR2	6p21.1	0.411493835	1.18E-15	2.24E-14	SPRED1	15q14	0.435327636	1.59E-17	3.39E-16
CNOT9	2q35	0.411351524	1.21E-15	2.29E-14	ZNF619	3p22.1	0.435319868	1.59E-17	3.39E-16
CHD2	15q26.1	0.411339507	1.22E-15	2.29E-14	G3BP2	4q21.1	0.435210609	1.63E-17	3.45E-16
VCL	10q22.2	0.411163852	1.25E-15	2.36E-14	FRS2	12q15	0.435201212	1.63E-17	3.46E-16
IREB2	15q25.1	0.410431474	1.42E-15	2.66E-14	FAM217B	20q13.33	0.434998936	1.69E-17	3.59E-16
CEP68	2p14	0.410312168	1.45E-15	2.72E-14	PTAFR	1p35.3	0.43492046	1.72E-17	3.63E-16
RNF219	13q31.1	0.410165812	1.49E-15	2.78E-14	DCK	4q13.3	0.434866359	1.73E-17	3.67E-16
EIF4ENIF1	22q12.2	0.410017463	1.53E-15	2.85E-14	ATXN7	3p14.1	0.434639422	1.81E-17	3.82E-16
ZNF699	19p13.2	0.409901305	1.56E-15	2.90E-14	CDYL	6p25.1	0.434522456	1.85E-17	3.90E-16
ATXN2	12q24.12	0.409900151	1.56E-15	2.90E-14	PCDHGA9	5q31.3	0.434348085	1.91E-17	4.02E-16
P2RY1	3q25.2	0.409851119	1.57E-15	2.92E-14	CDK17	12q23.1	0.434250753	1.95E-17	4.09E-16
ADD1	4p16.3	0.409841494	1.58E-15	2.92E-14	ATN1	12p13.31	0.434088625	2.01E-17	4.21E-16
CELF1	11p11.2	0.409661853	1.63E-15	3.01E-14	TGOLN2	2p11.2	0.43405229	2.02E-17	4.23E-16
BRD3	9q34.2	0.40933352	1.72E-15	3.18E-14	KAT6A	8p11.21	0.433995057	2.04E-17	4.27E-16

GOLGA4	3p22.2	0.409312734	1.73E-15	3.18E-14	GNA11	19p13.3	0.433974556	2.05E-17	4.28E-16
GNA11	19p13.3	0.409225888	1.75E-15	3.22E-14	TNS3	7p12.3	0.433858841	2.09E-17	4.37E-16
ERC1	12p13.33	0.409188588	1.76E-15	3.24E-14	DSTYK	1q32.1	0.433432128	2.27E-17	4.72E-16
ZNF37A	10p11.1	0.409167802	1.77E-15	3.25E-14	MED17	11q21	0.43339198	2.29E-17	4.75E-16
CDKN2AIP	4q35.1	0.408922072	1.85E-15	3.38E-14	AFAP1L2	10q25.3	0.433354679	2.30E-17	4.78E-16
TAF1A	1q41	0.408818598	1.88E-15	3.44E-14	TPBG	6q14.1	0.43305002	2.44E-17	5.05E-16
INO80	15q15.1	0.408795078	1.89E-15	3.45E-14	RAD54L2	3p21.2	0.432941808	2.49E-17	5.15E-16
TSPY26P	20q11.21	0.40874596	1.90E-15	3.47E-14	ZNF397	18q12.2	0.432762707	2.57E-17	5.32E-16
MOSPD2	Xp22.2	0.408641918	1.94E-15	3.53E-14	PATL1	11q12.1	0.432711169	2.60E-17	5.36E-16
ZNF92	7q11.21	0.40859633	1.95E-15	3.56E-14	UBXN4	2q21.3	0.432641581	2.63E-17	5.43E-16
RPEL1	10q24.33	0.408389496	2.02E-15	3.68E-14	XRN1	3q23	0.432532068	2.68E-17	5.52E-16
CNOT6L	4q21.1	0.408377651	2.03E-15	3.68E-14	ZNF267	16p11.2	0.432503309	2.70E-17	5.55E-16
ZC3H11A	1q32.1	0.408320418	2.05E-15	3.72E-14	FLG	1q21.3	0.432475565	2.71E-17	5.56E-16
NSD1	5q35.3	0.408200459	2.09E-15	3.78E-14	PDGFC	4q32.1	0.432457751	2.72E-17	5.58E-16
WASHC2A	10q11.23	0.408015654	2.16E-15	3.90E-14	MYNN	3q26.2	0.432366065	2.77E-17	5.67E-16
TRAF6	11p12	0.407965122	2.18E-15	3.93E-14	ANTXR1	2p13.3	0.432239814	2.83E-17	5.80E-16
IDE	10q23.33	0.407920389	2.19E-15	3.95E-14	YTHDC1	4q13.2	0.432136027	2.89E-17	5.90E-16
COL4A3BP	5q13.3	0.407877649	2.21E-15	3.98E-14	RNF150	4q31.21	0.432111918	2.90E-17	5.92E-16
RGPD4	2q12.3	0.407559482	2.33E-15	4.19E-14	PHF3	6q12	0.432092604	2.91E-17	5.94E-16
LOC100190986	16p12.2	0.407401879	2.40E-15	4.30E-14	RNF41	12q13.3	0.432032636	2.95E-17	5.99E-16
CORO1C	12q24.11	0.407215915	2.48E-15	4.44E-14	FZD1	7q21.13	0.431876029	3.03E-17	6.16E-16
TNPO3	7q32.1	0.407118534	2.52E-15	4.51E-14	NCOR1	17p12-p11.	0.431845847	3.05E-17	6.19E-16
KIT	4q12	0.407061331	2.54E-15	4.54E-14	QKI	6q26	0.431759001	3.10E-17	6.28E-16
BCLAF1	6q23.3	0.406934593	2.60E-15	4.63E-14	ZNF689	16p11.2	0.431444426	3.29E-17	6.65E-16
ZMYND8	20q13.12	0.406901278	2.61E-15	4.65E-14	VPS41	7p14.1	0.431338442	3.35E-17	6.78E-16
EDIL3	5q14.3	0.406866142	2.63E-15	4.68E-14	ARL15	5q11.2	0.431296585	3.38E-17	6.82E-16
NORAD	20q11.23	0.406853157	2.63E-15	4.68E-14	PTPN13	4q21.3	0.430623799	3.83E-17	7.69E-16
FBXO21	12q24.22	0.406683738	2.71E-15	4.81E-14	SETD5	3p25.3	0.43060951	3.84E-17	7.70E-16
WBP11P1	18q12.1	0.406606396	2.75E-15	4.87E-14	YEATS2	3q27.1	0.430544304	3.88E-17	7.79E-16
MIGA1	1p31.1	0.405883336	3.11E-15	5.50E-14	NEDD1	12q23.1	0.430406979	3.98E-17	7.98E-16
SERINC5	5q14.1	0.405636752	3.24E-15	5.72E-14	SRCAP	16p11.2	0.430339038	4.03E-17	8.07E-16
PANX1	11q21	0.405397856	3.38E-15	5.95E-14	AAK1	2p13.3	0.430276823	4.08E-17	8.16E-16
SMCR8	17p11.2	0.405380772	3.39E-15	5.96E-14	IDS	Xq28	0.430256718	4.09E-17	8.18E-16
VPS13C	15q22.2	0.405276272	3.45E-15	6.06E-14	TRAPPC8	18q12.1	0.430095017	4.22E-17	8.42E-16
MYH9	22q12.3	0.405194268	3.50E-15	6.14E-14	BCL9L	11q23.3	0.430048574	4.25E-17	8.49E-16
CGGBP1	3p11.1	0.405146716	3.52E-15	6.18E-14	PPP4R2	3p13	0.429932685	4.35E-17	8.66E-16
MCTS2P	20q11.21	0.405064135	3.57E-15	6.26E-14	MAP3K21	1q42.2	0.429916739	4.36E-17	8.68E-16
HNRNPLL	2p22.1	0.40484233	3.71E-15	6.49E-14	IGF1R	15q26.3	0.42991076	4.36E-17	8.68E-16
ZDHHC17	12q21.2	0.404821259	3.72E-15	6.51E-14	TNPO3	7q32.1	0.429887127	4.38E-17	8.71E-16
CCDC191	3q13.31	0.404727865	3.78E-15	6.61E-14	MIER1	1p31.3	0.429846694	4.42E-17	8.76E-16
TFAM	10q21.1	0.404656395	3.83E-15	6.68E-14	TOPORS	9p21.1	0.429503868	4.70E-17	9.30E-16
MATN3	2p24.1	0.404599742	3.87E-15	6.74E-14	EFNB2	13q33.3	0.429437808	4.76E-17	9.40E-16
RPS6KA3	Xp22.12	0.404405255	4.00E-15	6.96E-14	ONECUT1	15q21.3	0.429296599	4.89E-17	9.63E-16
SYT15	10q11.22	0.404317442	4.06E-15	7.06E-14	VTCN1	1p13.1-p12	0.429278979	4.90E-17	9.66E-16
METTL14	4q26	0.404150443	4.17E-15	7.26E-14	KBTBD7	13q14.11	0.429135415	5.04E-17	9.90E-16
ZNF619	3p22.1	0.404046257	4.25E-15	7.37E-14	FLT1	13q12.3	0.429119755	5.05E-17	9.91E-16
ZNF621	3p22.1	0.403934582	4.33E-15	7.50E-14	ZFPM2	8q23.1	0.429099945	5.07E-17	9.94E-16
PTBP3	9q32	0.403783955	4.44E-15	7.68E-14	FNBP1L	1p22.1	0.429065258	5.10E-17	9.99E-16
FUBP1	1p31.1	0.403445685	4.70E-15	8.11E-14	NOTCH3	19p13.12	0.429036611	5.13E-17	1.00E-15
AMER1	Xq11.2	0.40315098	4.94E-15	8.51E-14	POLK	5q13.3	0.428879435	5.28E-17	1.03E-15
BTAF1	10q23.32	0.402953656	5.11E-15	8.80E-14	APOOL	Xq21.1	0.428806826	5.35E-17	1.04E-15
NUMA1	11q13.4	0.40288902	5.17E-15	8.88E-14	GLIS3	9p24.2	0.428653922	5.50E-17	1.07E-15
ZNF292	6q14.3	0.40285656	5.20E-15	8.93E-14	RBL1	20q11.23	0.428639115	5.52E-17	1.07E-15
TAB3	Xp21.2	0.4025905	5.43E-15	9.31E-14	FBN1	15q21.1	0.428375731	5.79E-17	1.12E-15
MFSD9	2q12.1	0.402053026	5.95E-15	1.02E-13	PPTC7	12q24.11	0.428331312	5.84E-17	1.13E-15
PLEKHM3	2q33.3	0.402046192	5.96E-15	1.02E-13	EIF2AK3	2p11.2	0.428175275	6.01E-17	1.16E-15
HNRNPUL2	11q12.3	0.40198896	6.01E-15	1.03E-13	EDNRA	4q31.22-q3	0.428145977	6.04E-17	1.17E-15
ZNF641	12q13.11	0.401760599	6.25E-15	1.06E-13	N4BP2	4p14	0.428109246	6.08E-17	1.17E-15
RASSF8	12p12.1	0.401591179	6.43E-15	1.09E-13	CRAMP1	16p13.3	0.427749875	6.50E-17	1.25E-15
NDST2	10q22.2	0.401457095	6.58E-15	1.12E-13	IL1R1	2q11.2-q12	0.427730797	6.52E-17	1.25E-15
GPR157	1p36.22	0.401400868	6.64E-15	1.13E-13	ZNF417	19q13.43	0.427427266	6.89E-17	1.32E-15
ZNF281	1q32.1	0.401141291	6.93E-15	1.17E-13	PRR12	19q13.33	0.426973676	7.49E-17	1.43E-15
SNTB2	16q22.1	0.40106202	7.03E-15	1.19E-13	FBXO30	6q24.3	0.426939223	7.54E-17	1.44E-15
RBM43	2q23.3	0.400774263	7.37E-15	1.24E-13	SERINC5	5q14.1	0.426729655	7.83E-17	1.49E-15
CHM	Xq21.2	0.400631608	7.55E-15	1.27E-13	NCEH1	3q26.31	0.426711147	7.86E-17	1.49E-15
SUFU	10q24.32	0.400517713	7.70E-15	1.30E-13	ANKRD13C	1p31.1	0.426700926	7.87E-17	1.50E-15
UBA3	3p14.1	0.40044499	7.79E-15	1.31E-13	MTMR12	5p13.3	0.426537059	8.11E-17	1.54E-15
MAP3K2	2q14.3	0.4004172	7.83E-15	1.32E-13	STOX2	4q35.1	0.426533696	8.12E-17	1.54E-15
NUTM2D	10q23.2	0.400258885	8.04E-15	1.35E-13	GEM	8q22.1	0.426530337	8.12E-17	1.54E-15
ZMIZ1	10q22.3	0.400212472	8.10E-15	1.36E-13	RPEL1	10q24.33	0.426417469	8.29E-17	1.57E-15
SP4	7p15.3	0.400117654	8.23E-15	1.38E-13	PTBP3	9q32	0.426382273	8.34E-17	1.58E-15
KMT2C	7q36.1	0.40009516	8.26E-15	1.38E-13	SH3GLB1	1p22.3	0.426073901	8.83E-17	1.66E-15
ZNF616	19q13.41	0.400078645	8.29E-15	1.38E-13	ZNF420	19q13.12	0.425957442	9.02E-17	1.70E-15
RALGAPA2	20p11.23	0.400000057	8.39E-15	1.40E-13	SLAIN2	4p11	0.425561655	9.69E-17	1.82E-15

ATR	3q23	0.399906093	8.53E-15	1.42E-13	SLFN11	17q12	0.425559377	9.69E-17	1.82E-15
QTRT2	3q13.31	0.399829498	8.64E-15	1.44E-13	RSBN1L	7q11.23	0.425496734	9.81E-17	1.84E-15
CASD1	7q21.3	0.399742938	8.76E-15	1.46E-13	G2E3	14q12	0.425424726	9.94E-17	1.86E-15
ZNF514	2q11.1	0.399675625	8.86E-15	1.47E-13	ERCC4	16p13.12	0.425181843	1.04E-16	1.94E-15
TNFSF15	9q32	0.399558948	9.04E-15	1.50E-13	MYLIP	6p22.3	0.425177003	1.04E-16	1.94E-15
ELMSAN1	14q24.3	0.399515317	9.10E-15	1.51E-13	RIN2	20p11.23	0.425165013	1.04E-16	1.94E-15
PCDH18	4q28.3	0.399490972	9.14E-15	1.51E-13	SAMD15	14q24.3	0.425069158	1.06E-16	1.97E-15
FRAS1	4q21.21	0.399386325	9.30E-15	1.54E-13	MPZL3	11q23.3	0.425050869	1.06E-16	1.98E-15
MINK1	17p13.2	0.399344872	9.37E-15	1.55E-13	CYP1B1	2p22.2	0.425050264	1.06E-16	1.98E-15
INSIG2	2q14.1-q14.	0.39888761	1.01E-14	1.67E-13	LMBRD2	5p13.2	0.425047986	1.06E-16	1.98E-15
ADAT1	16q23.1	0.39882323	1.02E-14	1.68E-13	MIB1	18q11.2	0.425033179	1.07E-16	1.98E-15
ZNF445	3p21.31	0.398614232	1.06E-14	1.74E-13	PCDHB15	5q31.3	0.424987117	1.08E-16	1.99E-15
UBAP2	9p13.3	0.398453639	1.09E-14	1.78E-13	NCR3LG1	11p15.1	0.424793078	1.11E-16	2.06E-15
SP2	17q21.32	0.398442249	1.09E-14	1.78E-13	SNAPC3	9p22.3	0.424738759	1.13E-16	2.08E-15
PRKG1	10q11.23-q2	0.398191339	1.13E-14	1.86E-13	UBR2	6p21.1	0.424621446	1.15E-16	2.12E-15
ZNF280D	15q21.3	0.398117362	1.15E-14	1.88E-13	AHNAK	11q12.3	0.424436651	1.19E-16	2.19E-15
PAN3	13q12.2	0.397990084	1.17E-14	1.92E-13	CLDN10	13q32.1	0.424434023	1.19E-16	2.19E-15
CAMK1D	10p13	0.39783006	1.21E-14	1.96E-13	ITPR3	6p21.31	0.424343256	1.21E-16	2.22E-15
RAI1	17p11.2	0.397820094	1.21E-14	1.96E-13	WDR82	3p21.2	0.424274065	1.22E-16	2.25E-15
TASP1	20p12.1	0.397774536	1.22E-14	1.98E-13	SLC8A1	2p22.1	0.42427082	1.23E-16	2.25E-15
BAG4	8p11.23	0.397748625	1.22E-14	1.98E-13	ATP11B	3q26.33	0.424022355	1.28E-16	2.34E-15
ZC3H13	13q14.13	0.397702782	1.23E-14	2.00E-13	EIF2AK2	2p22.2	0.42400641	1.29E-16	2.35E-15
ADAMTS12	5p13.3-p13.	0.397558381	1.26E-14	2.04E-13	GJA1	6q22.31	0.423972526	1.29E-16	2.36E-15
SPRED2	2p14	0.39744253	1.29E-14	2.07E-13	USP8	15q21.2	0.423943798	1.30E-16	2.37E-15
BCL7A	12q24.31	0.397351414	1.30E-14	2.10E-13	APPL2	12q23.3	0.423747297	1.35E-16	2.45E-15
JAK1	1p31.3	0.397329774	1.31E-14	2.11E-13	TMEM184C	4q31.23	0.423724234	1.35E-16	2.46E-15
ASAP1	8q24.21-q24	0.397303065	1.32E-14	2.11E-13	HMBOX1	8p21.1-p12	0.42364656	1.37E-16	2.49E-15
DARS	2q21.3	0.397275958	1.32E-14	2.12E-13	GALNT10	5q33.2	0.42361948	1.38E-16	2.50E-15
ZNF175	19q13.41	0.397179431	1.34E-14	2.15E-13	BBS1	11q13.2	0.423348093	1.45E-16	2.62E-15
PHACTR2	6q24.2	0.397075786	1.37E-14	2.19E-13	IFT140	16p13.3	0.423337273	1.45E-16	2.62E-15
STAG2	Xq25	0.396604543	1.48E-14	2.36E-13	ICE1	5p15.32	0.423181521	1.49E-16	2.69E-15
ZNF562	19p13.2	0.396558985	1.49E-14	2.37E-13	MMP14	14q11.2	0.422952875	1.56E-16	2.80E-15
RAB3GAP2	1q41	0.396497481	1.50E-14	2.39E-13	CXORF38	Xp11.4	0.422938354	1.56E-16	2.80E-15
DCAF1	3p21.2	0.39648239	1.51E-14	2.40E-13	UTRN	6q24.2	0.422758683	1.61E-16	2.89E-15
ETNK1	12p12.1	0.396420887	1.52E-14	2.42E-13	GON4L	1q22	0.422744446	1.61E-16	2.89E-15
PCDHGA9	5q31.3	0.39634418	1.54E-14	2.45E-13	MARF1	16p13.11	0.422655608	1.64E-16	2.94E-15
CLCN3	4q33	0.396277663	1.56E-14	2.47E-13	RAB30	11q14.1	0.422624032	1.65E-16	2.95E-15
HUWE1	Xp11.22	0.396219576	1.57E-14	2.50E-13	DYRK1A	21q22.13	0.422540034	1.68E-16	2.99E-15
ABI1	10p12.1	0.395886317	1.66E-14	2.63E-13	ARID5B	10q21.2	0.42232531	1.74E-16	3.10E-15
ASAP2	2p25.1|2p24	0.395706761	1.71E-14	2.70E-13	PCSK5	9q21.13	0.422266085	1.76E-16	3.13E-15
RAB6A	11q13.4	0.395672307	1.72E-14	2.71E-13	SLC9A7	Xp11.3|Xp1	0.42199248	1.85E-16	3.29E-15
TMEM255A	Xq24	0.395551381	1.76E-14	2.76E-13	COL4A1	13q34	0.421958566	1.86E-16	3.30E-15
PDCD6IP	3p22.3	0.39514554	1.88E-14	2.94E-13	ZNF713	7p11.2	0.421790286	1.92E-16	3.40E-15
TBC1D5	3p24.3	0.395059862	1.90E-14	2.98E-13	PRPF40A	2q23.3	0.421731345	1.94E-16	3.43E-15
BTBD10	11p15.3	0.394859947	1.97E-14	3.07E-13	ZNF611	19q13.41	0.421570272	1.99E-16	3.53E-15
ZMYM4	1p34.3	0.394821792	1.98E-14	3.09E-13	WDR35	2p24.1	0.42152918	2.01E-16	3.55E-15
SCRN1	7p14.3	0.39478563	1.99E-14	3.10E-13	DYRK2	12q15	0.421397346	2.06E-16	3.63E-15
ARFIP1	4q31.3	0.394784405	1.99E-14	3.10E-13	PACSIN2	22q13.2	0.421293701	2.10E-16	3.70E-15
PAXIP1	7q36.2	0.394747475	2.00E-14	3.11E-13	TMEM131	2q11.2	0.421199654	2.13E-16	3.76E-15
SUPT20H	13q13.3	0.394734662	2.01E-14	3.12E-13	PLSCR1	3q24	0.421098684	2.17E-16	3.82E-15
PCDHGA11	5q31.3	0.394435854	2.11E-14	3.27E-13	NRP2	2q33.3	0.421037436	2.20E-16	3.86E-15
MPZL2	11q23.3	0.394379307	2.13E-14	3.30E-13	SOCS7	17q12	0.420860613	2.27E-16	3.98E-15
ERCC6	10q11.23	0.394203338	2.19E-14	3.39E-13	CDKL1	14q21.3	0.420729318	2.32E-16	4.07E-15
CRLF3	17q11.2	0.393757722	2.36E-14	3.65E-13	B4GALT5	20q13.13	0.420526898	2.41E-16	4.22E-15
C11ORF58	11p15.2	0.393722129	2.37E-14	3.67E-13	ZNF718	4p16.3	0.420430656	2.45E-16	4.28E-15
CREB3L2	7q33	0.393635853	2.41E-14	3.72E-13	DAAM1	14q23.1	0.42030651	2.50E-16	4.38E-15
RALGAPB	20q11.23	0.393623894	2.41E-14	3.72E-13	MATR3	5q31.2	0.420259528	2.52E-16	4.41E-15
CAB39	2q37.1	0.393465864	2.47E-14	3.81E-13	VEZT	12q22	0.42022724	2.54E-16	4.43E-15
HMGXB4	22q12.3	0.393289611	2.55E-14	3.92E-13	PTER	10p13	0.420209729	2.55E-16	4.44E-15
TTLL5	14q24.3	0.393239781	2.57E-14	3.95E-13	DCAF17	2q31.1	0.420207991	2.55E-16	4.44E-15
KDM6A	Xp11.3	0.393227822	2.57E-14	3.95E-13	ZC3H7A	16p13.13	0.42018638	2.56E-16	4.45E-15
APLP2	11q24.3	0.393150259	2.60E-14	4.00E-13	THSD7A	7p21.3	0.420157337	2.57E-16	4.47E-15
VPS36	13q14.3	0.393148095	2.60E-14	4.00E-13	CFAP126	1q23.3	0.420110782	2.59E-16	4.50E-15
DBR1	3q22.3	0.392996045	2.67E-14	4.10E-13	MOSPD2	Xp22.2	0.420032052	2.63E-16	4.56E-15
ARMC8	3q22.3	0.39278024	2.77E-14	4.24E-13	FMNL3	12q13.12	0.420006965	2.64E-16	4.58E-15
ZNF304	19q13.43	0.392778789	2.77E-14	4.24E-13	ARHGAP23	17q12	0.419984186	2.65E-16	4.59E-15
FAM76B	11q21	0.392756579	2.78E-14	4.25E-13	SLC35F5	2q14.1	0.419981053	2.65E-16	4.59E-15
AMBRA1	11p11.2	0.392735508	2.79E-14	4.26E-13	MAP4	3p21.31	0.41995987	2.66E-16	4.61E-15
SNORA8	11q21	0.392666032	2.82E-14	4.30E-13	S1PR3	9q22.1	0.419784299	2.75E-16	4.75E-15
CHD9	16q12.2	0.392636989	2.83E-14	4.32E-13	STXBP5	6q24.3	0.419647654	2.81E-16	4.86E-15
ERO1A	14q22.1	0.392584312	2.86E-14	4.35E-13	6-Mar	5p15.2	0.419607191	2.84E-16	4.89E-15
BAZ1B	7q11.23	0.392557262	2.87E-14	4.37E-13	ZNF134	19q13.43	0.41958612	2.85E-16	4.90E-15
FLG	1q21.3	0.392006308	3.14E-14	4.77E-13	TTC37	5q15	0.419344377	2.97E-16	5.11E-15
CREBZF	11q14.1	0.391961872	3.16E-14	4.80E-13	ARID2	12q12	0.419287999	3.00E-16	5.16E-15

BCL2L1	20q11.21	0.391924856	3.18E-14	4.83E-13	RGPD4	2q12.3	0.419211291	3.04E-16	5.22E-15
NTN4	12q22	0.391916057	3.18E-14	4.83E-13	PGBD3	10q11.23	0.419112314	3.10E-16	5.31E-15
TFCP2	12q13.12-q1	0.391906918	3.19E-14	4.83E-13	PXK	3p14.3	0.419079	3.12E-16	5.33E-15
ZBTB1	14q23.3	0.391864492	3.21E-14	4.86E-13	KITLG	12q21.32	0.419061661	3.13E-16	5.35E-15
PTPN11	12q24.13	0.391730095	3.28E-14	4.96E-13	F2R	5q13.3	0.418922109	3.20E-16	5.48E-15
PRPF40A	2q23.3	0.391649514	3.33E-14	5.02E-13	MAGI3	1p13.2	0.418787712	3.28E-16	5.60E-15
BDP1	5q13.2	0.391496039	3.41E-14	5.14E-13	FCHSD2	11q13.4	0.418726493	3.32E-16	5.66E-15
MINPP1	10q23.2	0.391481517	3.42E-14	5.14E-13	CUL5	11q22.3	0.418649898	3.36E-16	5.73E-15
ZNF397	18q12.2	0.391456176	3.43E-14	5.16E-13	TRRAP	7q22.1	0.418609181	3.39E-16	5.75E-15
RGP1	9p13.3	0.391432713	3.44E-14	5.18E-13	GTF3C3	2q33.1	0.418605194	3.39E-16	5.75E-15
HNRNPR	1p36.12	0.391219273	3.57E-14	5.36E-13	SCAF8	6q25.2	0.418597506	3.39E-16	5.76E-15
YTHDF3	8q12.3	0.391069785	3.65E-14	5.47E-13	CCDC191	3q13.31	0.418553941	3.42E-16	5.80E-15
ZNF280C	Xq26.1	0.391045582	3.67E-14	5.49E-13	STAG2	Xq25	0.418526037	3.44E-16	5.82E-15
PUS7	7q22.3	0.390776788	3.83E-14	5.72E-13	FILIP1L	3q12.1	0.418244999	3.61E-16	6.11E-15
SOS2	14q21.3	0.39066033	3.90E-14	5.82E-13	TBCEL	11q23.3	0.418051092	3.74E-16	6.32E-15
ARHGAP5	14q12	0.390650079	3.91E-14	5.83E-13	NTN4	12q22	0.417952744	3.81E-16	6.43E-15
SDAD1	4q21.1	0.390625022	3.93E-14	5.85E-13	NUP160	11p11.2	0.417864018	3.87E-16	6.52E-15
NFIX	19p13.13	0.390599708	3.94E-14	5.87E-13	LTN1	21q21.3	0.417798528	3.91E-16	6.59E-15
PCNP	3q12.3	0.390323199	4.12E-14	6.12E-13	APPL1	3p14.3	0.417779736	3.93E-16	6.61E-15
PTPN21	14q31.3	0.390298996	4.14E-14	6.14E-13	RAPGEF5	7p15.3	0.417732184	3.96E-16	6.66E-15
VPS26A	10q22.1	0.390219269	4.19E-14	6.22E-13	VANGL1	1p13.1	0.417702286	3.98E-16	6.69E-15
UHRF2	9p24.1	0.390036467	4.32E-14	6.40E-13	SMAD9	13q13.3	0.417669953	4.00E-16	6.72E-15
TMEM106B	7p21.3	0.390000902	4.34E-14	6.43E-13	JRKL	11q21	0.417600635	4.05E-16	6.80E-15
TRIM44	11p13	0.389915452	4.40E-14	6.51E-13	TSHZ1	18q22.3	0.417571591	4.07E-16	6.83E-15
HELZ	17q24.2	0.389847969	4.45E-14	6.58E-13	RALGAPB	20q11.23	0.41737626	4.22E-16	7.05E-15
FCHO2	5q13.2	0.389785896	4.50E-14	6.64E-13	ZNF621	3p22.1	0.417370565	4.22E-16	7.05E-15
PEG3	19q13.43	0.389763145	4.51E-14	6.65E-13	ABCC1	16p13.11	0.417351488	4.23E-16	7.07E-15
NEO1	15q24.1	0.389719552	4.55E-14	6.69E-13	CDC73	1q31.2	0.417328424	4.25E-16	7.09E-15
DCAF7	17q23.3	0.389462603	4.74E-14	6.96E-13	SIRT1	10q21.3	0.417290299	4.28E-16	7.13E-15
CTCF	16q22.1	0.38923037	4.92E-14	7.21E-13	TET3	2p13.1	0.417172102	4.37E-16	7.27E-15
SEC14L1	17q25.2-q25	0.389210723	4.93E-14	7.23E-13	LMBR1	7q36.3	0.417146648	4.39E-16	7.30E-15
CTDSP2	12q14.1	0.389086862	5.03E-14	7.37E-13	KLHL23	2q31.1	0.416998596	4.51E-16	7.48E-15
CEP290	12q21.32	0.389026782	5.08E-14	7.44E-13	ZFX	Xp22.11	0.416966805	4.53E-16	7.52E-15
ARFGEF1	8q13.2	0.388947909	5.15E-14	7.52E-13	SYNRG	17q12	0.416891065	4.59E-16	7.61E-15
NCAPD3	11q25	0.388933957	5.16E-14	7.54E-13	NHS	Xp22.2-p22	0.416853598	4.62E-16	7.65E-15
SETX	9q34.13	0.388877321	5.21E-14	7.60E-13	CFAP97	4q35.1	0.416848923	4.63E-16	7.65E-15
SLC24A1	15q22.31	0.388759696	5.31E-14	7.73E-13	TLN1	9p13.3	0.416847215	4.63E-16	7.65E-15
CHST15	10q26.13	0.388757703	5.31E-14	7.73E-13	RGL1	1q25.3	0.416666975	4.78E-16	7.88E-15
TARBP1	1q42.2	0.388707589	5.35E-14	7.79E-13	RBMS3	3p24.1	0.41664645	4.80E-16	7.90E-15
PCDHGA12	5q31.3	0.388616926	5.43E-14	7.90E-13	EPG5	18q12.3-q2	0.416540551	4.89E-16	8.03E-15
FNDC3A	13q14.2	0.388501438	5.53E-14	8.03E-13	MAMDC2	9q21.12	0.416526204	4.90E-16	8.05E-15
PHACTR1	6p24.1	0.38849161	5.54E-14	8.04E-13	SIRPB1	20p13	0.416390778	5.02E-16	8.23E-15
RRAGC	1p34.3	0.388393807	5.63E-14	8.16E-13	ETV3L	1q23.1	0.416261934	5.13E-16	8.41E-15
PUS7L	12q12	0.387916896	6.08E-14	8.78E-13	AFTPH	2p14	0.416201344	5.19E-16	8.49E-15
SYNE2	14q23.2	0.387905194	6.09E-14	8.79E-13	DDI2	1p36.21	0.416094554	5.29E-16	8.65E-15
RBAK	7p22.1	0.387806418	6.19E-14	8.91E-13	GDAP2	1p12	0.415713099	5.66E-16	9.23E-15
RREB1	6p24.3	0.387476662	6.52E-14	9.36E-13	BAZ1A	14q13.1-q1	0.415695161	5.67E-16	9.25E-15
ARFGEF2	20q13.13	0.387183835	6.83E-14	9.79E-13	CD2AP	6p12.3	0.415652906	5.72E-16	9.31E-15
RBM25	14q24.2	0.387150948	6.87E-14	9.83E-13	DOCK9	13q32.3	0.415567028	5.80E-16	9.45E-15
KIF27	9q21.32	0.387126888	6.90E-14	9.86E-13	KBTBD4	11p11.2	0.415512643	5.86E-16	9.52E-15
CAMSAP1	9q34.3	0.387086285	6.94E-14	9.92E-13	DISC1	1q42.2	0.415502392	5.87E-16	9.53E-15
UBFD1	16p12.2	0.387065784	6.96E-14	9.95E-13	BNC2	9p22.3-p22	0.415464979	5.91E-16	9.59E-15
MFN1	3q26.33	0.38692797	7.12E-14	1.02E-12	TRAK1	3p22.1	0.41540319	5.97E-16	9.68E-15
IKZF4	12q13.2	0.386923414	7.12E-14	1.02E-12	PDS5A	4p14	0.415320159	6.06E-16	9.81E-15
ZRANB3	2q21.3	0.386679677	7.41E-14	1.06E-12	XPO7	8p21.3	0.415207972	6.18E-16	1.00E-14
CWC22	2q31.3	0.386670736	7.42E-14	1.06E-12	ERCC6	10q11.23	0.41518861	6.20E-16	1.00E-14
ZNF627	19p13.2	0.386519084	7.60E-14	1.08E-12	SERTAD4	1q32.2	0.415176889	6.22E-16	1.00E-14
PDE5A	4q26	0.386377854	7.77E-14	1.11E-12	SNRNP200	2q11.2	0.415145045	6.25E-16	1.01E-14
CELSR1	22q13.31	0.386329448	7.83E-14	1.11E-12	PDE5A	4q26	0.414946866	6.47E-16	1.04E-14
BTBD19	1p34.1	0.386306874	7.86E-14	1.12E-12	KAT7	17q21.33	0.414865716	6.57E-16	1.05E-14
TSTD2	9q22.33	0.386284459	7.89E-14	1.12E-12	ZNF436	1p36.12	0.414785704	6.66E-16	1.07E-14
ZNF417	19q13.43	0.386145792	8.07E-14	1.14E-12	CRTC3	15q26.1	0.414723631	6.73E-16	1.08E-14
SLC12A6	15q14	0.386026771	8.22E-14	1.17E-12	ARHGAP31	3q13.32-q1	0.414635646	6.84E-16	1.10E-14
HNRNPF	10q11.21	0.386010256	8.24E-14	1.17E-12	NCOA6	20q11.22	0.414619701	6.86E-16	1.10E-14
TJP1	15q13.1	0.386007463	8.25E-14	1.17E-12	SPAG9	17q21.33	0.414612583	6.86E-16	1.10E-14
SLTM	15q22.1	0.385987362	8.27E-14	1.17E-12	TLR1	4p14	0.414560191	6.93E-16	1.11E-14
ZNF366	5q13.2|5q13	0.385937646	8.34E-14	1.18E-12	PKN2	1p22.2	0.414431773	7.09E-16	1.13E-14
ZNF689	16p11.2	0.385537074	8.89E-14	1.25E-12	APP	21q21.3	0.414416113	7.11E-16	1.13E-14
DDX18	2q14.1	0.38544334	9.02E-14	1.27E-12	FCHO2	5q13.2	0.414349484	7.19E-16	1.15E-14
IMPAD1	8q12.1	0.385386392	9.10E-14	1.28E-12	CEMIP2	9q21.13	0.414315884	7.23E-16	1.15E-14
USP12	13q12.13	0.385200912	9.38E-14	1.32E-12	PLSCR4	3q24	0.414298231	7.25E-16	1.15E-14
UVRAG	11q13.5	0.385120161	9.50E-14	1.33E-12	MLLT10	10p12.31	0.414266909	7.29E-16	1.16E-14
DIP2B	12q13.12	0.384907773	9.82E-14	1.38E-12	ZBTB20	3q13.31	0.41413781	7.46E-16	1.18E-14
TCF7L2	10q25.2-q25	0.384800115	9.99E-14	1.40E-12	MCAM	11q23.3	0.414135645	7.46E-16	1.18E-14

RNF6	13q12.13	0.384672836	1.02E-13	1.42E-12	KIAA0355	19q13.11	0.414100337	7.51E-16	1.19E-14
RLIM	Xq13.2	0.384641515	1.02E-13	1.43E-12	PTPRM	18p11.23	0.414061897	7.56E-16	1.20E-14
MTMR1	Xq28	0.384498861	1.05E-13	1.46E-12	SENP7	3q12.3	0.413994159	7.65E-16	1.21E-14
ZBTB4	17p13.1	0.384484908	1.05E-13	1.46E-12	CTCF	16q22.1	0.413750677	7.98E-16	1.26E-14
HDAC4	2q37.3	0.38440091	1.06E-13	1.48E-12	KCTD18	2q33.1	0.413651618	8.12E-16	1.28E-14
LPCAT2	16q12.2	0.384380409	1.07E-13	1.49E-12	TBL1XR1	3q26.32	0.413641907	8.14E-16	1.28E-14
CDC14C	7p12.3	0.38427195	1.09E-13	1.51E-12	BBS10	12q21.2	0.413593786	8.21E-16	1.29E-14
TAF2	8q24.12	0.38422124	1.10E-13	1.52E-12	KIAA1958	9q32	0.413528866	8.30E-16	1.30E-14
ZNF440	19p13.2	0.383999998	1.13E-13	1.57E-12	GALNT4	12q21.33	0.41343066	8.44E-16	1.33E-14
THOC2	Xq25	0.383972663	1.14E-13	1.58E-12	ZIK1	19q13.43	0.413417164	8.46E-16	1.33E-14
FRMD4B	3p14.1	0.383925396	1.15E-13	1.59E-12	ZNF616	19q13.41	0.41333325	8.59E-16	1.35E-14
EPB41L5	2q14.2	0.383736899	1.18E-13	1.64E-12	PRSS23	11q14.2	0.412996973	9.11E-16	1.42E-14
COPB1	11p15.2	0.383729835	1.18E-13	1.64E-12	GPRIN3	4q22.1	0.412913829	9.24E-16	1.44E-14
CS	12q13.3	0.383715543	1.19E-13	1.64E-12	ZNF562	19p13.2	0.412752952	9.51E-16	1.48E-14
SEC31A	4q21.22	0.383634108	1.20E-13	1.66E-12	TUBGCP4	15q15.3	0.412743187	9.52E-16	1.48E-14
ZBTB34	9q33.3	0.383543561	1.22E-13	1.68E-12	DUBR	3q13.12	0.412735612	9.53E-16	1.48E-14
SYNJ1	21q22.11	0.383532456	1.22E-13	1.68E-12	SLC6A6	3p25.1	0.412698851	9.60E-16	1.49E-14
KANK1	9p24.3	0.383494586	1.23E-13	1.69E-12	RFX7	15q21.3	0.412641334	9.69E-16	1.50E-14
BEND3P3	10q22.3	0.383460839	1.24E-13	1.70E-12	MYO5C	15q21.2	0.412480456	9.97E-16	1.54E-14
ABCE1	4q31.21	0.383341111	1.26E-13	1.73E-12	SACS	13q12.12	0.412418668	1.01E-15	1.56E-14
MED23	6q23.2	0.383191339	1.29E-13	1.77E-12	CSGALNACT1	8p21.3	0.412248679	1.04E-15	1.60E-14
RPL23AP64	11q23.3	0.38294043	1.34E-13	1.83E-12	PRDM2	1p36.21	0.412238173	1.04E-15	1.61E-14
WDR3	1p12	0.382924253	1.34E-13	1.84E-12	VEPH1	3q25.31-q2	0.412224907	1.04E-15	1.61E-14
ZNF703	8p11.23	0.38281907	1.37E-13	1.87E-12	DHX40	17q23.1	0.412004657	1.08E-15	1.67E-14
NCK1	3q22.3	0.382759105	1.38E-13	1.88E-12	NME9	3q22.3	0.412002052	1.08E-15	1.67E-14
WASHC4	12q23.3	0.382723797	1.39E-13	1.89E-12	CWC22	2q31.3	0.411972226	1.09E-15	1.67E-14
PRKAA2	1p32.2	0.382683379	1.40E-13	1.90E-12	HEATR5A	14q12	0.411836092	1.12E-15	1.71E-14
RSBN1L	7q11.23	0.382347087	1.47E-13	2.00E-12	RBM18	9q33.2	0.411759497	1.13E-15	1.73E-14
PXK	3p14.3	0.382146631	1.52E-13	2.06E-12	STT3B	3p23	0.411680624	1.15E-15	1.76E-14
ZSCAN29	15q15.3	0.382092246	1.53E-13	2.07E-12	ITIH5	10p14	0.411417525	1.20E-15	1.83E-14
ZNF550	19q13.43	0.382071745	1.54E-13	2.08E-12	RUSC2	9p13.3	0.411354883	1.21E-15	1.85E-14
COG5	7q22.3	0.381955883	1.57E-13	2.12E-12	TMEM43	3p25.1	0.411133356	1.26E-15	1.92E-14
ZNF528	19q13.41	0.381880685	1.59E-13	2.14E-12	PELI1	2p14	0.410998674	1.29E-15	1.97E-14
WDR37	10p15.3	0.381753691	1.62E-13	2.18E-12	RPL23AP64	11q23.3	0.410985249	1.29E-15	1.97E-14
NAF1	4q32.2	0.381753122	1.62E-13	2.18E-12	CHST3	10q22.1	0.410621395	1.38E-15	2.09E-14
C20ORF194	20p13	0.38155836	1.67E-13	2.24E-12	CREB1	2q33.3	0.410556759	1.39E-15	2.11E-14
LINC01881	2q37.3	0.381491568	1.68E-13	2.26E-12	TRIP11	14q32.12	0.410420654	1.43E-15	2.16E-14
HIF1AN	10q24.31	0.381375842	1.72E-13	2.30E-12	MFSD14B	9q22.32	0.410326405	1.45E-15	2.19E-14
PABPC3	13q12.13	0.381375273	1.72E-13	2.30E-12	XYLT1	16p12.3	0.410138192	1.50E-15	2.26E-14
GBF1	10q24.32	0.381196457	1.76E-13	2.36E-12	C2CD3	11q13.4	0.410111996	1.50E-15	2.27E-14
MYADM	19q13.42	0.380738596	1.90E-13	2.53E-12	BICD2	9q22.31	0.409832383	1.58E-15	2.37E-14
UBQLN2	Xp11.21	0.380482928	1.97E-13	2.63E-12	CDR2L	17q25.1	0.409774865	1.59E-15	2.40E-14
PCDHGB3	5q31.3	0.380419406	1.99E-13	2.66E-12	HELZ	17q24.2	0.409484431	1.68E-15	2.52E-14
EXOC2	6p25.3	0.380257102	2.04E-13	2.72E-12	RAB18	10p12.1	0.409376117	1.71E-15	2.56E-14
STAM	10p12.33	0.379962282	2.14E-13	2.85E-12	DPYSL2	8p21.2	0.409363133	1.71E-15	2.57E-14
DCAF12	9p13.3	0.379957584	2.14E-13	2.85E-12	RASSF6	4q13.3	0.409276082	1.74E-15	2.60E-14
USP42	7p22.1	0.379865301	2.17E-13	2.88E-12	EHF	11p13	0.409199421	1.76E-15	2.63E-14
TRIP11	14q32.12	0.379601918	2.26E-13	3.00E-12	ZNF484	9q22.31	0.409180074	1.77E-15	2.64E-14
ZNF625	19p13.2	0.379318745	2.37E-13	3.13E-12	ADAMTS1	21q21.3	0.409139043	1.78E-15	2.65E-14
PEX26	22q11.21	0.379292406	2.38E-13	3.14E-12	THBS1	15q14	0.409026571	1.81E-15	2.70E-14
DNAJC10	2q32.1	0.379244855	2.39E-13	3.16E-12	SH3PXD2B	5q35.1	0.408875944	1.86E-15	2.76E-14
NIPA1	15q11.2	0.379179961	2.42E-13	3.19E-12	CLIC4	1p36.11	0.408807322	1.88E-15	2.79E-14
RASSF3	12q14.2	0.379155417	2.43E-13	3.20E-12	ZNF678	1q42.13	0.408763673	1.90E-15	2.81E-14
SLX4IP	20p12.2	0.378975332	2.50E-13	3.29E-12	FSD1L	9q31.2	0.408539269	1.97E-15	2.92E-14
MEX3B	15q25.2	0.378734602	2.59E-13	3.41E-12	ARL6	3q11.2	0.408367969	2.03E-15	3.01E-14
TSHZ2	20q13.2	0.378437789	2.71E-13	3.56E-12	ZNF154	19q13.43	0.408348665	2.04E-15	3.01E-14
ZNF609	15q22.31	0.378436765	2.72E-13	3.56E-12	ERBIN	5q12.3	0.408213356	2.09E-15	3.07E-14
IRF2BPL	14q24.3	0.378284999	2.78E-13	3.64E-12	IKZF3	17q12-q21.	0.408194445	2.09E-15	3.08E-14
ZNF567	19q13.12	0.378275888	2.78E-13	3.64E-12	GTF2H3	12q24.31	0.40819314	2.09E-15	3.08E-14
TANC1	2q24.2	0.378125545	2.85E-13	3.73E-12	ZNF528	19q13.41	0.408127365	2.12E-15	3.11E-14
UBE4A	11q23.3	0.378023324	2.90E-13	3.78E-12	USP33	1p31.1	0.40807298	2.14E-15	3.14E-14
CDC73	1q31.2	0.37801165	2.90E-13	3.78E-12	NOTCH1	9q34.3	0.407956237	2.18E-15	3.20E-14
CREBBP	16p13.3	0.378007948	2.90E-13	3.78E-12	HRNR	1q21.3	0.407808154	2.24E-15	3.27E-14
FAM171A1	10p13	0.377977481	2.92E-13	3.80E-12	ZNF860	3p23-p22.3	0.407756724	2.26E-15	3.30E-14
NPHP3	3q22.1	0.377902025	2.95E-13	3.84E-12	TSTD2	9q22.33	0.407578103	2.33E-15	3.39E-14
TBC1D23	3q12.1-q12.	0.377873836	2.96E-13	3.86E-12	CCDC39	3q26.33	0.407554914	2.34E-15	3.40E-14
ZNF112	19q13.31	0.377627137	3.08E-13	4.00E-12	1-Mar	4q32.2-q32	0.407511053	2.35E-15	3.43E-14
FKBP9	7p14.3	0.377500712	3.14E-13	4.08E-12	MYO10	5p15.1	0.407427761	2.39E-15	3.47E-14
PARD3	10p11.22-p1	0.377467513	3.16E-13	4.10E-12	HELB	12q14.3|12c	0.407421642	2.39E-15	3.47E-14
STAU1	20q13.13	0.377211818	3.28E-13	4.26E-12	ZNF609	15q22.31	0.407382487	2.41E-15	3.49E-14
CEP192	18p11.21	0.377170815	3.30E-13	4.28E-12	FKBP9	7p14.3	0.4073082	2.44E-15	3.54E-14
ARID4A	14q23.1	0.377113298	3.33E-13	4.31E-12	TRAK2	2q33.1	0.407289264	2.44E-15	3.55E-14
SOX9	17q24.3	0.376976392	3.40E-13	4.40E-12	MIA2	14q13.2	0.407250089	2.46E-15	3.57E-14
CKAP5	11p11.2	0.376911987	3.44E-13	4.44E-12	ZNF175	19q13.41	0.407242396	2.46E-15	3.57E-14

ZNF549	19q13.43	0.376762784	3.52E-13	4.54E-12	RBBP6	16p12.1	0.407234423	2.47E-15	3.57E-14
DCK	4q13.3	0.376694447	3.56E-13	4.58E-12	CMTR2	16q22.2	0.407153842	2.50E-15	3.62E-14
ZBTB14	18p11.31	0.376634936	3.59E-13	4.62E-12	FAM135A	6q13	0.406913237	2.61E-15	3.76E-14
PLCG1	20q12	0.376569162	3.63E-13	4.66E-12	PEX26	22q11.21	0.406535958	2.78E-15	4.00E-14
PREX2	8q13.2	0.376408828	3.72E-13	4.78E-12	WDPCP	2p15	0.406465343	2.81E-15	4.04E-14
TVP23B	17p11.2	0.376221495	3.83E-13	4.91E-12	NPNT	4q24	0.406403839	2.84E-15	4.08E-14
ZFX	Xp22.11	0.375804637	4.08E-13	5.22E-12	GAN	16q23.2	0.406390456	2.85E-15	4.09E-14
SH3BP5	3p25.1	0.375682199	4.16E-13	5.32E-12	PPP1R3D	20q13.33	0.406350024	2.87E-15	4.12E-14
MBNL3	Xq26.2	0.375669101	4.17E-13	5.33E-12	USP9X	Xp11.4	0.406323828	2.88E-15	4.13E-14
CHAMP1	13q34	0.375426219	4.32E-13	5.51E-12	MEIS3P1	17p12	0.406321265	2.89E-15	4.13E-14
WDR31	9q32	0.375331225	4.39E-13	5.58E-12	MAML1	5q35.3	0.406264032	2.91E-15	4.17E-14
UBE3B	12q24.11	0.37518533	4.49E-13	5.70E-12	BAHCC1	17q25.3	0.406261185	2.91E-15	4.17E-14
PPP2R5E	14q23.2	0.37508681	4.56E-13	5.78E-12	SELENON	1p36.11	0.406234591	2.93E-15	4.18E-14
PIK3CG	7q22.3	0.375084247	4.56E-13	5.78E-12	ANKRD40	17q21.33	0.40614615	2.97E-15	4.24E-14
RALGAPA1	14q13.2	0.374565479	4.94E-13	6.25E-12	PDE1A	2q32.1	0.406111421	2.99E-15	4.27E-14
GON4L	1q22	0.374539826	4.95E-13	6.27E-12	HECTD2	10q23.32	0.406093787	3.00E-15	4.27E-14
MSANTD2	11q24.2	0.374487434	4.99E-13	6.31E-12	NIPAL2	8q22.2	0.406090513	3.00E-15	4.27E-14
GPR176	15q14-q15.	0.374346167	5.10E-13	6.45E-12	TNFSF15	9q32	0.406082055	3.01E-15	4.28E-14
MCM3AP-AS1	21q22.3	0.3742825	5.15E-13	6.51E-12	HNRNPLL	2p22.1	0.406048485	3.02E-15	4.30E-14
NUDT4	12q22	0.373961521	5.41E-13	6.82E-12	CASP8AP2	6q15	0.406023713	3.04E-15	4.31E-14
LRIG2	1p13.2	0.373718354	5.62E-13	7.08E-12	CAMK1D	10p13	0.405999794	3.05E-15	4.33E-14
PAPSS1	4q25	0.373615848	5.71E-13	7.17E-12	FAM102B	1p13.3	0.405820124	3.14E-15	4.46E-14
SYNGAP1	6p21.32	0.373609041	5.71E-13	7.17E-12	SSPN	12p12.1	0.405804181	3.15E-15	4.47E-14
DPF2	11q13.1	0.373542385	5.77E-13	7.24E-12	RIC1	9p24.1	0.405798199	3.15E-15	4.47E-14
PRDM4	12q23.3	0.373413113	5.89E-13	7.38E-12	CRLF3	17q11.2	0.405685727	3.22E-15	4.55E-14
PLS1	3q23	0.373347054	5.95E-13	7.45E-12	TASOR	3p14.3	0.405680032	3.22E-15	4.55E-14
FCF1	14q24.3	0.372975185	6.29E-13	7.85E-12	SENP1	12q13.11	0.405619952	3.25E-15	4.60E-14
CAND1	12q14.3-q15	0.372960378	6.31E-13	7.86E-12	MAP3K20	2q31.1	0.405581797	3.27E-15	4.62E-14
CEP170P1	4q26	0.372959612	6.31E-13	7.86E-12	NECTIN3	3q13.13	0.405477021	3.33E-15	4.70E-14
LRBA	4q31.3	0.372877804	6.39E-13	7.95E-12	UNC5B	10q22.1	0.405429747	3.36E-15	4.73E-14
POLR1A	2p11.2	0.372770457	6.49E-13	8.07E-12	ARID1A	1p36.11	0.405020606	3.60E-15	5.05E-14
RUNX1T1	8q21.3	0.372708337	6.55E-13	8.15E-12	ENTPD4	8p21.3	0.404897285	3.68E-15	5.15E-14
ARHGEF11	1q23.1	0.372676146	6.59E-13	8.18E-12	PLEKHM3	2q33.3	0.40486938	3.69E-15	5.17E-14
CSTF2T	10q21.1	0.372526436	6.74E-13	8.36E-12	SOAT1	1q25.2	0.404756624	3.77E-15	5.27E-14
PHF12	17q11.2	0.372523019	6.74E-13	8.36E-12	ZNF518A	10q24.1	0.404746658	3.77E-15	5.28E-14
TMCC1	3q22.1	0.372516755	6.75E-13	8.36E-12	ZKSCAN1	7q22.1	0.404724733	3.79E-15	5.29E-14
ZNF234	19q13.31	0.372427631	6.84E-13	8.45E-12	GPR157	1p36.22	0.404663001	3.83E-15	5.34E-14
ACTR3	2q14.1	0.372213249	7.07E-13	8.72E-12	GSR	8p12	0.404628206	3.85E-15	5.36E-14
PPP1R2	3q29	0.372196423	7.09E-13	8.74E-12	CX3CR1	3p22.2	0.404527693	3.92E-15	5.45E-14
TSPAN5	4q23	0.371898605	7.41E-13	9.13E-12	TTC30A	2q31.2	0.404330654	4.05E-15	5.62E-14
ZNF713	7p11.2	0.371841069	7.48E-13	9.21E-12	SPRED2	2p14	0.404288797	4.08E-15	5.66E-14
TBC1D8	2q11.2	0.371743688	7.59E-13	9.33E-12	STON1	2p16.3	0.404179742	4.15E-15	5.76E-14
MAU2	19p13.11	0.371743403	7.59E-13	9.33E-12	APAF1	12q23.1	0.404021427	4.27E-15	5.90E-14
HPS4	22q12.1	0.371701831	7.64E-13	9.38E-12	MSRB3	12q14.3	0.403954798	4.31E-15	5.96E-14
PHF6	Xq26.2	0.371563164	7.80E-13	9.57E-12	P2RY12	3q25.1	0.403923537	4.34E-15	5.99E-14
EXOC5	14q22.3	0.371485741	7.89E-13	9.68E-12	TULP3	12p13.33	0.403914679	4.34E-15	5.99E-14
NHLRC2	10q25.3	0.371454989	7.93E-13	9.72E-12	RAB6D	2q21.1	0.403809866	4.42E-15	6.10E-14
NEK4	3p21.1	0.371378653	8.02E-13	9.82E-12	MPHOSPH9	12q24.31	0.403740105	4.47E-15	6.16E-14
SPG11	15q21.1	0.371320851	8.09E-13	9.91E-12	HRH2	5q35.2	0.403465321	4.69E-15	6.45E-14
RABL2B	22q13.33	0.371246818	8.18E-13	1.00E-11	ZNF347	19q13.42	0.403410123	4.73E-15	6.50E-14
SPRY2	13q31.1	0.370694425	8.90E-13	1.09E-11	BCLAF1	6q23.3	0.403330081	4.80E-15	6.58E-14
WWC3	Xp22.2	0.370689299	8.91E-13	1.09E-11	UVRAG	11q13.5	0.403271709	4.84E-15	6.64E-14
SMAD9	13q13.3	0.370569997	9.07E-13	1.10E-11	ZNF567	19q13.12	0.403039077	5.04E-15	6.89E-14
PCMTD2	20q13.33	0.370558604	9.08E-13	1.11E-11	PAK2	3q29	0.402965615	5.10E-15	6.97E-14
PCDHGA6	5q31.3	0.370448783	9.24E-13	1.12E-11	KMT2D	12q13.12	0.402747789	5.29E-15	7.22E-14
PKN2	1p22.2	0.370421075	9.27E-13	1.13E-11	PUM2	2p24.1	0.402746452	5.29E-15	7.22E-14
NUP58	13q12.13	0.370044935	9.82E-13	1.19E-11	EBLN2	3p13	0.402699701	5.33E-15	7.27E-14
LCA5	6q14.1	0.369988867	9.90E-13	1.20E-11	PPM1L	3q25.33-q2	0.402520283	5.50E-15	7.49E-14
TGIF1	18p11.31	0.369935025	9.98E-13	1.21E-11	SLC40A1	2q32.2	0.402483836	5.53E-15	7.52E-14
POLR1B	2q14.1	0.369824831	1.01E-12	1.23E-11	FAM13B	5q31.2	0.402303311	5.70E-15	7.75E-14
ANKMY2	7p21.1	0.369690176	1.04E-12	1.25E-11	CPEB2	4p15.32	0.402238391	5.77E-15	7.83E-14
ZSCAN25	7q22.1	0.369650286	1.04E-12	1.26E-11	INTS2	17q23.2	0.402213619	5.79E-15	7.86E-14
IPP	1p34.1	0.369638897	1.04E-12	1.26E-11	SGMS2	4q25	0.402138163	5.86E-15	7.95E-14
VPS50	7q21.2-q21.	0.369583088	1.05E-12	1.27E-11	RARB	3p24.2	0.401877342	6.13E-15	8.30E-14
KAT7	17q21.33	0.369544363	1.06E-12	1.28E-11	KLHDC10	7q32.2	0.401830645	6.18E-15	8.35E-14
SNRNP200	2q11.2	0.369445844	1.07E-12	1.29E-11	ZC3H12C	11q22.3	0.401701373	6.31E-15	8.52E-14
G3BP1	5q33.1	0.369400596	1.08E-12	1.30E-11	PROB1	5q31.2	0.401641199	6.38E-15	8.61E-14
FAM98B	15q14	0.369387472	1.08E-12	1.30E-11	ARL5A	2q23.3	0.401543799	6.48E-15	8.74E-14
POLK	5q13.3	0.369296925	1.10E-12	1.32E-11	ATXN1	6p22.3	0.401533662	6.49E-15	8.74E-14
DPY19L4	8q22.1	0.369188608	1.12E-12	1.34E-11	KCNE4	2q36.1	0.401518001	6.51E-15	8.76E-14
NFYA	6p21.1	0.369171925	1.12E-12	1.34E-11	BTBD10	11p15.3	0.401390153	6.65E-15	8.95E-14
ZSCAN30	18q12.2	0.368917252	1.16E-12	1.39E-11	PAWR	12q21.2	0.401367403	6.68E-15	8.97E-14
SMAD2	18q21.1	0.368694702	1.20E-12	1.44E-11	ZMYM2	13q12.11	0.40134488	6.70E-15	9.00E-14
DDX3X	Xp11.4	0.368685021	1.20E-12	1.44E-11	ALMS1	2p13.1	0.401339185	6.71E-15	9.00E-14

TRAPPC8	18q12.1	0.368677074	1.21E-12	1.44E-11	PHLDB1	11q23.3	0.401333205	6.71E-15	9.01E-14
TNS1	2q35	0.36863092	1.21E-12	1.45E-11	PWAR5	15q11.2	0.401315929	6.73E-15	9.03E-14
SUCNR1	3q25.1	0.368514371	1.24E-12	1.47E-11	SMARCA2	9p24.3	0.401281383	6.77E-15	9.07E-14
MPHOSPH8	13q12.11	0.368464944	1.25E-12	1.48E-11	NMD3	3q26.1	0.401272556	6.78E-15	9.08E-14
FUBP3	9q34.11-q34	0.368345896	1.27E-12	1.51E-11	EEA1	12q22	0.401215893	6.85E-15	9.16E-14
ERBIN	5q12.3	0.368326819	1.27E-12	1.51E-11	WDR60	7q36.3	0.400963614	7.14E-15	9.54E-14
TRIM33	1p13.2	0.368284419	1.28E-12	1.52E-11	ADGRF5	6p12.3	0.400951655	7.16E-15	9.55E-14
NCOA4	10q11.22	0.368078526	1.32E-12	1.57E-11	SLC49A4	3q21.1	0.400847042	7.29E-15	9.71E-14
ST14	11q24.3	0.367903127	1.35E-12	1.61E-11	ZNF587	19q13.43	0.400723294	7.44E-15	9.91E-14
TMEM168	7q31.1	0.367789801	1.38E-12	1.63E-11	TSPY26P	20q11.21	0.400633825	7.55E-15	1.00E-13
GTF3C3	2q33.1	0.367745951	1.39E-12	1.64E-11	ATP13A3	3q29	0.400625059	7.56E-15	1.01E-13
DLEU7	13q14.3	0.367587862	1.42E-12	1.68E-11	PAN3	13q12.2	0.400622497	7.56E-15	1.01E-13
STARD7-AS1	2q11.2	0.367561156	1.43E-12	1.68E-11	PCDHGB5	5q31.3	0.400614117	7.58E-15	1.01E-13
PHF3	6q12	0.36747875	1.44E-12	1.70E-11	SMCHD1	18p11.32	0.400503191	7.72E-15	1.02E-13
BTBD8	1p22.1	0.36745493	1.45E-12	1.71E-11	ZNF570	19q13.12	0.400495788	7.73E-15	1.02E-13
CSNK1A1	5q32	0.36736497	1.47E-12	1.73E-11	CDK13	7p14.1	0.400423464	7.82E-15	1.04E-13
GATAD2B	1q21.3	0.36724538	1.49E-12	1.76E-11	DNAJC27	2p23.3	0.400379045	7.88E-15	1.04E-13
ATG16L1	2q37.1	0.367205232	1.50E-12	1.77E-11	SLTM	15q22.1	0.400193423	8.13E-15	1.07E-13
GOLGA6L5P	15q25.2	0.367165037	1.51E-12	1.77E-11	LOX	5q23.1	0.400000342	8.39E-15	1.11E-13
CEMIP	15q25.1	0.366945267	1.56E-12	1.83E-11	FAM199X	Xq22.2	0.399995957	8.40E-15	1.11E-13
PIP5K1A	1q21.3	0.366725731	1.62E-12	1.89E-11	FYTTD1	3q29	0.399977278	8.43E-15	1.11E-13
MBTD1	17q21.33	0.366665936	1.63E-12	1.90E-11	CBLB	3q13.11	0.399883029	8.56E-15	1.13E-13
RBM27	5q32	0.36649882	1.67E-12	1.95E-11	SNX25	4q35.1	0.399879498	8.57E-15	1.13E-13
PLS3	Xq23	0.366466618	1.68E-12	1.95E-11	ELF2	4q31.1	0.399861389	8.59E-15	1.13E-13
TMEM184C	4q31.23	0.366409671	1.69E-12	1.97E-11	ZNF585B	19q13.12	0.399822693	8.65E-15	1.14E-13
CACNA2D1	7q21.11	0.36636973	1.70E-12	1.98E-11	EXOC5	14q22.3	0.399783684	8.70E-15	1.14E-13
SPTY2D1	11p15.1	0.366271287	1.73E-12	2.01E-11	PLA2G4A	1q31.1	0.399772436	8.72E-15	1.14E-13
KRR1	12q21.2	0.366261322	1.73E-12	2.01E-11	DNAJC10	2q32.1	0.399627049	8.93E-15	1.17E-13
ATXN7L3	17q21.31	0.36612066	1.77E-12	2.05E-11	CNOT9	2q35	0.399617282	8.95E-15	1.17E-13
LAMA3	18q11.2	0.36582083	1.85E-12	2.14E-11	NSRP1	17q11.2	0.399532117	9.08E-15	1.19E-13
ZNF829	19q13.12	0.365677191	1.89E-12	2.19E-11	SLX4IP	20p12.2	0.399180451	9.63E-15	1.26E-13
STRIP1	1p13.3	0.365582788	1.92E-12	2.22E-11	PIEZO2	18p11.22-p	0.399178015	9.63E-15	1.26E-13
DHX36	3q25.2	0.365484553	1.94E-12	2.24E-11	MED23	6q23.2	0.39906896	9.81E-15	1.28E-13
CA5B	Xp22.2	0.365403118	1.97E-12	2.27E-11	SYDE1	19p13.12	0.399051591	9.83E-15	1.28E-13
MTMR2	11q21	0.365294063	2.00E-12	2.30E-11	ATP11A	13q34	0.399034222	9.86E-15	1.29E-13
GSE1	16q24.1	0.36526986	2.01E-12	2.31E-11	PHF6	Xq26.2	0.399019985	9.89E-15	1.29E-13
QKI	6q26	0.3652374	2.02E-12	2.32E-11	ZNF106	15q15.1	0.398914347	1.01E-14	1.31E-13
PTAR1	9q21.12	0.365224302	2.02E-12	2.32E-11	DZIP1L	3q22.3	0.398889489	1.01E-14	1.31E-13
SERTAD4	1q32.2	0.365174939	2.03E-12	2.34E-11	SPIN1	9q22.1	0.398862524	1.01E-14	1.32E-13
PKHD1	6p12.3-p12.	0.365130955	2.05E-12	2.35E-11	MFAP3	5q33.2	0.398752045	1.03E-14	1.34E-13
YEATS2	3q27.1	0.365063994	2.07E-12	2.37E-11	HERC3	4q22.1	0.398706203	1.04E-14	1.35E-13
SART3	12q23.3	0.365022448	2.08E-12	2.39E-11	TOP2B	3p24.2	0.398670325	1.05E-14	1.36E-13
ZNF268	12q24.33	0.364953515	2.10E-12	2.41E-11	SMC3	10q25.2	0.39858889	1.06E-14	1.37E-13
CNTRL	9q33.2	0.364738356	2.17E-12	2.49E-11	DYNLL2	17q22	0.398531373	1.07E-14	1.39E-13
ZNF28	19q13.41	0.364602173	2.22E-12	2.54E-11	CASK	Xp11.4	0.398378753	1.10E-14	1.42E-13
ZNF614	19q13.41	0.364594459	2.22E-12	2.54E-11	MAP3K14-AS1	17q21.31	0.398260213	1.12E-14	1.45E-13
LAMA5	20q13.33	0.364515017	2.24E-12	2.56E-11	PRR11	17q22	0.398129476	1.15E-14	1.48E-13
CBX6	22q13.1	0.364374356	2.29E-12	2.62E-11	STXBP4	17q22	0.397994355	1.17E-14	1.51E-13
BCL6	3q27.3	0.364326519	2.31E-12	2.63E-11	LRIG2	1p13.2	0.397796176	1.21E-14	1.56E-13
ZIK1	19q13.43	0.3642682	2.33E-12	2.65E-11	CELF1	11p11.2	0.397775703	1.22E-14	1.56E-13
LZTFL1	3p21.31	0.364132897	2.37E-12	2.71E-11	ARL6IP6	2q23.3	0.397681084	1.24E-14	1.59E-13
KIAA0355	19q13.11	0.364083637	2.39E-12	2.72E-11	EMP1	12p13.1	0.397565538	1.26E-14	1.61E-13
FBXO34	14q22.3	0.363880618	2.46E-12	2.80E-11	HS3ST1	4p15.33	0.397473624	1.28E-14	1.64E-13
MPHOSPH9	12q24.31	0.363872076	2.47E-12	2.81E-11	SLC34A2	4p15.2	0.397461233	1.28E-14	1.64E-13
SRFBP1	5q23.1	0.36382965	2.48E-12	2.82E-11	ZNF264	19q13.43	0.397444808	1.28E-14	1.64E-13
SACM1L	3p21.31	0.363806469	2.49E-12	2.83E-11	ANKRD12	18p11.22	0.397287063	1.32E-14	1.69E-13
PDCL	9q33.2	0.363789786	2.50E-12	2.83E-11	ZNF836	19q13.41	0.397252325	1.33E-14	1.69E-13
TULP3	12p13.33	0.363683889	2.54E-12	2.88E-11	RSPH3	6q25.3	0.397231254	1.33E-14	1.70E-13
EFCAB14	1p33	0.363640298	2.55E-12	2.89E-11	ADGRA2	8p11.23	0.397155513	1.35E-14	1.72E-13
PPP3R1	2p14	0.363549751	2.59E-12	2.93E-11	GNB4	3q26.33	0.397054431	1.37E-14	1.75E-13
ANKFY1	17p13.2	0.363379192	2.65E-12	3.00E-11	CDH6	5p13.3	0.397041048	1.37E-14	1.75E-13
UBP1	3p22.3	0.363258748	2.70E-12	3.06E-11	VHL	3p25.3	0.397028235	1.38E-14	1.75E-13
ZBTB6	9q33.2	0.363230274	2.71E-12	3.07E-11	CWF19L2	11q22.3	0.396827779	1.42E-14	1.81E-13
MIER3	5q11.2	0.363199522	2.73E-12	3.08E-11	ZNF644	1p22.2	0.396798166	1.43E-14	1.81E-13
FOXN3	14q31.3-q32	0.363151116	2.75E-12	3.10E-11	LRRC58	3q13.33	0.3967005	1.45E-14	1.84E-13
BRD2	6p21.32	0.363060854	2.78E-12	3.14E-11	UBR5	8q22.3	0.396385294	1.53E-14	1.94E-13
DNAJC22	12q13.12	0.362868086	2.86E-12	3.22E-11	HMGXB4	22q12.3	0.396328916	1.54E-14	1.96E-13
COG6	13q14.11	0.362867232	2.86E-12	3.22E-11	WASHC4	12q23.3	0.396248335	1.57E-14	1.98E-13
ARHGEF17	11q13.4	0.362785227	2.90E-12	3.26E-11	TRAPPC11	4q35.1	0.396240077	1.57E-14	1.98E-13
ZNF81	Xp11.23	0.362632607	2.96E-12	3.33E-11	TFCP2	12q13.12-q	0.396181136	1.58E-14	2.00E-13
UBR5	8q22.3	0.36251928	3.01E-12	3.38E-11	MEF2A	15q26.3	0.396126751	1.60E-14	2.02E-13
PRRC2A	6p21.33	0.362514181	3.01E-12	3.38E-11	CEP170P1	4q26	0.39605836	1.62E-14	2.04E-13
CRAMP1	16p13.3	0.362471159	3.03E-12	3.40E-11	GMEB1	1p35.3	0.395968721	1.64E-14	2.07E-13
DOCK4	7q31.1	0.362307719	3.11E-12	3.48E-11	TNFSF8	9q32-q33.1	0.395952045	1.64E-14	2.07E-13

ZNF224	19q13.31	0.362201512	3.16E-12	3.53E-11	ADD3	10q25.1-q2	0.395875326	1.67E-14	2.10E-13
WDR48	3p22.2	0.362191261	3.16E-12	3.53E-11	ZBTB1	14q23.3	0.395837741	1.68E-14	2.11E-13
ARPP19	15q21.2	0.362171614	3.17E-12	3.54E-11	RALBP1	18p11.22	0.395835463	1.68E-14	2.11E-13
LINC01622	6p25.3	0.362079815	3.21E-12	3.59E-11	EHD3	2p23.1	0.395806732	1.68E-14	2.12E-13
BTN2A1	6p22.2	0.361937843	3.28E-12	3.66E-11	GAREM1	18q12.1	0.395750326	1.70E-14	2.14E-13
EPS8	12p12.3	0.36185897	3.32E-12	3.70E-11	TSPAN5	4q23	0.395643091	1.73E-14	2.17E-13
ALG10B	12q12	0.361611817	3.44E-12	3.83E-11	DENND4A	15q22.31	0.395487797	1.77E-14	2.22E-13
ZFP36L2	2p21	0.361514436	3.49E-12	3.88E-11	RAB8B	15q22.2	0.39534799	1.82E-14	2.27E-13
NKD1	16q12.1	0.361499743	3.50E-12	3.89E-11	ARPP19	15q21.2	0.395219003	1.86E-14	2.32E-13
RECQL	12p12.1	0.361472295	3.51E-12	3.90E-11	B3GNT2	2p15	0.394978683	1.93E-14	2.41E-13
B4GALNT3	12p13.33	0.36136971	3.57E-12	3.96E-11	FKTN	9q31.2	0.394846592	1.97E-14	2.46E-13
LRATD1	2p24.3	0.361212043	3.65E-12	4.04E-11	VPS8	3q27.2	0.394816666	1.98E-14	2.47E-13
BMP2K	4q21.21	0.360807597	3.87E-12	4.29E-11	IFT81	12q24.11	0.394749753	2.00E-14	2.49E-13
KIF26B	1q44	0.360763342	3.90E-12	4.31E-11	CFAP221	2q14.2	0.394725023	2.01E-14	2.50E-13
TMEM67	8q22.1	0.360682881	3.94E-12	4.36E-11	KLHL8	4q22.1	0.394709605	2.02E-14	2.51E-13
ZNF548	19q13.43	0.360643704	3.97E-12	4.38E-11	RBAK	7p22.1	0.394709063	2.02E-14	2.51E-13
FYCO1	3p21.31	0.360639433	3.97E-12	4.38E-11	HNRNPUL2	11q12.3	0.394606244	2.05E-14	2.55E-13
ICE1	5p15.32	0.360558567	4.02E-12	4.43E-11	IFT80	3q25.33	0.394604251	2.05E-14	2.55E-13
PRAG1	8p23.1	0.360241368	4.21E-12	4.63E-11	TANC2	17q23.2-q2	0.394436255	2.11E-14	2.61E-13
CYSLTR2	13q14.2	0.360128706	4.28E-12	4.70E-11	DHX8	17q21.31	0.394313532	2.15E-14	2.67E-13
ZNF12	7p22.1	0.360060558	4.32E-12	4.75E-11	DCP1A	3p21.1	0.394042746	2.25E-14	2.78E-13
KDM5B	1q32.1	0.360047745	4.33E-12	4.75E-11	KCTD12	13q22.3	0.394027939	2.26E-14	2.78E-13
TRAPPC11	4q35.1	0.360038349	4.33E-12	4.76E-11	SPOPL	2q22.1	0.393939413	2.29E-14	2.82E-13
OXSR1	3p22.2	0.359942392	4.39E-12	4.82E-11	ZNF548	19q13.43	0.393660341	2.40E-14	2.95E-13
CXORF56	Xq24	0.359829803	4.47E-12	4.89E-11	PTPRB	12q15	0.39362902	2.41E-14	2.96E-13
SETD1B	12q24.31	0.35964159	4.59E-12	5.02E-11	PPFIA1	11q13.3	0.393411365	2.50E-14	3.07E-13
ATG14	14q22.3	0.359500761	4.69E-12	5.12E-11	TAF13	1p13.3	0.393356809	2.52E-14	3.09E-13
TCAF1	7q35	0.359476558	4.70E-12	5.13E-11	TMPPE	3p22.3	0.393272468	2.55E-14	3.13E-13
SH3D19	4q31.3	0.359466308	4.71E-12	5.14E-11	PLS3	Xq23	0.393209314	2.58E-14	3.16E-13
CFLAR	2q33.1	0.359414485	4.74E-12	5.18E-11	ZZZ3	1p31.1	0.393167173	2.60E-14	3.18E-13
SLC7A6OS	16q22.1	0.359261037	4.85E-12	5.28E-11	VPS35	16q11.2	0.393118938	2.62E-14	3.20E-13
ERCC3	2q14.3	0.359173311	4.91E-12	5.34E-11	CSNK2A1	20p13	0.393062389	2.64E-14	3.23E-13
ZNF468	19q13.41	0.359133448	4.94E-12	5.37E-11	LAMC2	1q25.3	0.392738282	2.79E-14	3.40E-13
MBNL1	3q25.1-q25.	0.359078493	4.98E-12	5.41E-11	ARID4B	1q42.3	0.392704472	2.80E-14	3.42E-13
UBR3	2q31.1	0.358965736	5.06E-12	5.50E-11	REV1	2q11.2	0.39261905	2.84E-14	3.46E-13
KCTD7	7q11.21	0.358927012	5.09E-12	5.53E-11	EPS15	1p32.3	0.392602421	2.85E-14	3.47E-13
SPATA5	4q28.1	0.358915907	5.10E-12	5.53E-11	ZBTB6	9q33.2	0.392396384	2.94E-14	3.59E-13
ZNF611	19q13.41	0.358887225	5.12E-12	5.55E-11	TRIM33	1p13.2	0.392383029	2.95E-14	3.59E-13
TIA1	2p13.3	0.358862376	5.14E-12	5.57E-11	LOC646214	15q11.2	0.392381578	2.95E-14	3.59E-13
SEMA7A	15q24.1	0.358833048	5.16E-12	5.59E-11	PRDM10	11q24.3	0.392293593	2.99E-14	3.64E-13
LRIG3	12q14.1	0.358783219	5.20E-12	5.63E-11	PARG	10q11.23	0.392293337	2.99E-14	3.64E-13
LINC00205	21q22.3	0.358743355	5.23E-12	5.66E-11	MYCBP2	13q22.3	0.39208488	3.10E-14	3.76E-13
FAM133CP	10q21.1	0.358697797	5.26E-12	5.69E-11	SEPTIN11	4q21.1	0.392066656	3.11E-14	3.77E-13
WDR60	7q36.3	0.358543184	5.38E-12	5.81E-11	PRKCI	3q26.2	0.391841883	3.22E-14	3.91E-13
EIF2AK3	2p11.2	0.35842217	5.48E-12	5.91E-11	SLFN5	17q12	0.391811245	3.24E-14	3.92E-13
CPSF6	12q15	0.358398536	5.50E-12	5.92E-11	ZNF319	16q21	0.391780493	3.26E-14	3.94E-13
USP47	11p15.3	0.358204939	5.65E-12	6.09E-11	NEXN	1p31.1	0.391707885	3.29E-14	3.99E-13
ADAMTS5	21q21.3	0.358092157	5.75E-12	6.18E-11	VPS4B	18q21.33	0.391575766	3.37E-14	4.07E-13
APBB2	4p14-p13	0.357871199	5.93E-12	6.36E-11	CXORF56	Xq24	0.391494786	3.41E-14	4.12E-13
CCDC39	3q26.33	0.35787107	5.93E-12	6.36E-11	LRRC49	15q23	0.391487581	3.41E-14	4.12E-13
SIRPB1	20p13	0.357809981	5.99E-12	6.42E-11	OTUD4	4q31.21	0.391393248	3.47E-14	4.18E-13
FAM199X	Xq22.2	0.357560859	6.21E-12	6.65E-11	GNA13	17q24.1	0.391340287	3.50E-14	4.22E-13
SPRED1	15q14	0.357394831	6.36E-12	6.80E-11	ZNF813	19q13.42	0.391212933	3.57E-14	4.30E-13
NPEPPS	17q21.32	0.357359264	6.39E-12	6.83E-11	FNDC3B	3q26.31	0.391105092	3.63E-14	4.37E-13
ZNF708	19p12	0.357259865	6.48E-12	6.92E-11	ZNF304	19q13.43	0.39084057	3.79E-14	4.55E-13
KDM2A	11q13.2	0.356923872	6.81E-12	7.24E-11	DNAJB4	1p31.1	0.390454748	4.04E-14	4.83E-13
ZFP30	19q13.12	0.356812138	6.92E-12	7.35E-11	ZNF708	19p12	0.390361069	4.10E-14	4.90E-13
RFFL	17q12	0.356723986	7.00E-12	7.44E-11	SETX	9q34.13	0.390358819	4.10E-14	4.90E-13
MOB1B	4q13.3	0.356630759	7.10E-12	7.54E-11	MGA	15q15.1	0.390308962	4.13E-14	4.94E-13
FBXO3	11p13	0.356624896	7.10E-12	7.54E-11	SCRN3	2q31.1	0.390284474	4.15E-14	4.95E-13
SLCO1B3	12p12.2	0.356595907	7.13E-12	7.57E-11	ZBTB43	9q33.3	0.390194497	4.21E-14	5.02E-13
MRTFB	16p13.12	0.356515557	7.22E-12	7.65E-11	MAP3K3	17q23.3	0.390093984	4.28E-14	5.10E-13
ZBED4	22q13.33	0.356509745	7.22E-12	7.65E-11	SYTL2	11q14.1	0.390046432	4.31E-14	5.14E-13
TRAF3IP1	2q37.3	0.356431157	7.31E-12	7.73E-11	MTMR2	11q21	0.389973824	4.36E-14	5.20E-13
ANKHD1-EIF4E	5q31.3	0.356384292	7.36E-12	7.77E-11	SEC14L1P1	11p11.2	0.389961947	4.37E-14	5.20E-13
IKZF3	17q12-q21.1	0.356321007	7.42E-12	7.84E-11	CA5B	Xp22.2	0.389914598	4.40E-14	5.24E-13
IGSF10	3q25.1	0.356290502	7.46E-12	7.87E-11	ADAMTSL1	9p22.2-p22	0.389833448	4.46E-14	5.30E-13
UBE2Q2P1	15q25.2	0.356281386	7.47E-12	7.88E-11	UTP20	12q23.2	0.389765395	4.51E-14	5.36E-13
RELN	7q22.1	0.356248409	7.50E-12	7.91E-11	RELCH	18q21.33	0.389736636	4.53E-14	5.38E-13
LOC728024	8p11.23	0.356074148	7.69E-12	8.11E-11	NAB1	2q32.2	0.389720691	4.55E-14	5.39E-13
FAM114A1	4p14	0.356019256	7.75E-12	8.17E-11	NSUN3	3q11.2	0.389650388	4.60E-14	5.45E-13
KDM4C	9p24.1	0.355986227	7.79E-12	8.20E-11	AKAP13	15q25.3	0.389561806	4.66E-14	5.52E-13
DENND5B	12p11.21	0.355921591	7.86E-12	8.27E-11	CHMP1B	18p11.21	0.389386862	4.80E-14	5.67E-13
TMEM185B	2q14.2	0.35591675	7.87E-12	8.27E-11	ZNF136	19p13.2	0.389305257	4.86E-14	5.74E-13

NPTN	15q24.1	0.35586749	7.92E-12	8.32E-11	BTN2A3P	6p22.2	0.389215279	4.93E-14	5.81E-13
SLC35E2A	1p36.33	0.355863789	7.93E-12	8.32E-11	NEK1	4q33	0.389213369	4.93E-14	5.81E-13
CSRNP2	12q13.12	0.355813105	7.98E-12	8.37E-11	PDCL	9q33.2	0.389136406	4.99E-14	5.88E-13
KBTBD2	7p14.3	0.355761852	8.04E-12	8.43E-11	PCDHGB2	5q31.3	0.389119458	5.01E-14	5.89E-13
TAF4	20q13.33	0.355719141	8.09E-12	8.47E-11	ADCY5	3q21.1	0.389005658	5.10E-14	5.98E-13
FIP1L1	4q12	0.355652512	8.17E-12	8.55E-11	ADNP2	18q23	0.388957875	5.14E-14	6.03E-13
RRAGB	Xp11.21	0.355649096	8.18E-12	8.55E-11	SLC24A1	15q22.31	0.388884697	5.20E-14	6.09E-13
ARMC1	8q13.1	0.355605246	8.23E-12	8.60E-11	DCAF16	4p15.31	0.388726382	5.34E-14	6.24E-13
SCAPER	15q24.3	0.355553993	8.29E-12	8.66E-11	COL3A1	2q32.2	0.388710437	5.35E-14	6.25E-13
IKZF5	10q26.13	0.355473411	8.38E-12	8.74E-11	CNOT6L	4q21.1	0.388565504	5.48E-14	6.39E-13
TANK	2q24.2	0.355429846	8.44E-12	8.79E-11	ZNF710	15q26.1	0.388561233	5.48E-14	6.39E-13
STOX2	4q35.1	0.355369385	8.51E-12	8.86E-11	AP1G1	16q22.2	0.388518807	5.52E-14	6.43E-13
MYSM1	1p32.1	0.355337876	8.55E-12	8.90E-11	EFCAB7	1p31.3	0.388462539	5.57E-14	6.48E-13
MLXIP	12q24.31	0.355171588	8.75E-12	9.10E-11	THBD	20p11.21	0.388349415	5.67E-14	6.60E-13
ZNF124	1q44	0.3551098	8.83E-12	9.17E-11	CEP112	17q24.1	0.388308385	5.71E-14	6.64E-13
RP2	Xp11.3	0.355016405	8.95E-12	9.29E-11	PAFAH1B1	17p13.3	0.388235207	5.77E-14	6.71E-13
PCDH17	13q21.1	0.354995904	8.98E-12	9.31E-11	LAMB1	7q31.1	0.388189079	5.82E-14	6.76E-13
CAMK2G	10q22.2	0.354919024	9.08E-12	9.40E-11	PDE8B	5q13.3	0.388184335	5.82E-14	6.76E-13
HECTD4	12q24.13	0.354799149	9.23E-12	9.55E-11	KDM3A	2p11.2	0.388163453	5.84E-14	6.78E-13
RPRD2	1q21.2	0.354717429	9.34E-12	9.66E-11	SFRP1	8p11.21	0.38813593	5.87E-14	6.80E-13
RAD50	5q31.1	0.354692657	9.38E-12	9.69E-11	B3GALNT1	3q26.1	0.388028486	5.97E-14	6.91E-13
ZNF184	6p22.1	0.354654787	9.43E-12	9.73E-11	USH1C	11p15.1	0.387992385	6.00E-14	6.94E-13
ANKRD42	11q14.1	0.354550597	9.57E-12	9.87E-11	CUEDC1	17q22	0.387836572	6.16E-14	7.11E-13
AVL9	7p14.3	0.354536787	9.59E-12	9.88E-11	ARFIP1	4q31.3	0.387783097	6.21E-14	7.17E-13
TOPORS	9p21.1	0.354406494	9.77E-12	1.01E-10	ZNF486	19p12	0.387561717	6.43E-14	7.42E-13
STK4	20q13.12	0.354395389	9.78E-12	1.01E-10	PNMA2	8p21.2	0.387494427	6.50E-14	7.50E-13
DICER1	14q32.13	0.354388556	9.79E-12	1.01E-10	POLR3A	10q22.3	0.387441639	6.56E-14	7.56E-13
RNF145	5q33.3	0.354385423	9.80E-12	1.01E-10	EDN1	6p24.1	0.387114189	6.91E-14	7.96E-13
ZNF202	11q24.1	0.35438343	9.80E-12	1.01E-10	DACH1	13q21.33	0.387094731	6.93E-14	7.98E-13
ZCCHC4	4p15.2	0.354372066	9.82E-12	1.01E-10	PCDHGB3	5q31.3	0.386948272	7.10E-14	8.16E-13
OGT	Xq13.1	0.354311961	9.90E-12	1.02E-10	ADD1	4p16.3	0.38693338	7.11E-14	8.17E-13
SULT1C4	2q12.3	0.354229177	1.00E-11	1.03E-10	RFWD3	16q23.1	0.386882127	7.17E-14	8.24E-13
LONRF1	8p23.1	0.354211733	1.00E-11	1.03E-10	FTO	16q12.2	0.386863619	7.19E-14	8.25E-13
RC3H1	1q25.1	0.354048292	1.03E-11	1.05E-10	BTBD19	1p34.1	0.386749225	7.33E-14	8.40E-13
RPGRIP1L	16q12.2	0.354012415	1.03E-11	1.06E-10	SPG11	15q21.1	0.386572046	7.54E-14	8.63E-13
ZNF665	19q13.42	0.353939915	1.04E-11	1.07E-10	TMEM200C	18p11.31	0.386494328	7.63E-14	8.73E-13
LRRC58	3q13.33	0.3537123	1.08E-11	1.10E-10	MALT1	18q21.32	0.386443629	7.69E-14	8.80E-13
KIF2A	5q12.1	0.353608655	1.09E-11	1.12E-10	SPEN	1p36.21-p3	0.386378708	7.77E-14	8.88E-13
LINC00472	6q13	0.35341758	1.12E-11	1.15E-10	ATAD2B	2p24.1-p23	0.386315496	7.85E-14	8.96E-13
ERMP1	9p24.1	0.353055407	1.18E-11	1.20E-10	EGR3	8p21.3	0.386297158	7.87E-14	8.98E-13
RESF1	12p11.21	0.353034621	1.19E-11	1.21E-10	PLEKHB2	2q21.1	0.386117318	8.10E-14	9.23E-13
CDK5R1	17q11.2	0.352882001	1.21E-11	1.23E-10	ATP6V1A	3q13.31	0.386112477	8.11E-14	9.23E-13
NAB1	2q32.2	0.352827331	1.22E-11	1.24E-10	INTS4P2	7q11.21	0.386087773	8.14E-14	9.26E-13
ZNF644	1p22.2	0.352813094	1.23E-11	1.24E-10	PDLIM3	4q35.1	0.386059543	8.18E-14	9.30E-13
C20ORF197	20q13.33	0.352718842	1.24E-11	1.26E-10	PKHD1	6p12.3-p12	0.385988503	8.27E-14	9.40E-13
NBEAL1	2q33.2	0.352579323	1.27E-11	1.28E-10	SMAD2	18q21.1	0.385885825	8.41E-14	9.54E-13
ATF7IP	12p13.1	0.352520667	1.28E-11	1.29E-10	ARAP2	4p14	0.385845677	8.46E-14	9.59E-13
SPAST	2p22.3	0.352520382	1.28E-11	1.29E-10	VCAN	5q14.2-q14	0.385761394	8.58E-14	9.71E-13
ZNF507	19q13.11	0.352515257	1.28E-11	1.29E-10	FAT1	4q35.2	0.38562415	8.77E-14	9.90E-13
MFSD14B	9q22.32	0.352494756	1.28E-11	1.29E-10	MTMR3	22q12.2	0.385622726	8.77E-14	9.90E-13
SKP2	5p13.2	0.352455177	1.29E-11	1.30E-10	TNFSF18	1q25.1	0.385622477	8.77E-14	9.90E-13
SEPTIN2	2q37.3	0.352251756	1.33E-11	1.33E-10	SEMA3C	7q21.11	0.385602569	8.80E-14	9.93E-13
GEMIN5	5q33.2	0.35223422	1.33E-11	1.34E-10	CC2D2A	4p15.32	0.385521359	8.91E-14	1.01E-12
KANSL1L	2q34	0.352211893	1.34E-11	1.34E-10	NDE1	16p13.11	0.385462987	8.99E-14	1.01E-12
MAN1A2	1p12	0.352140825	1.35E-11	1.35E-10	ZNF124	1q44	0.385229786	9.33E-14	1.05E-12
TTC37	5q15	0.352104663	1.36E-11	1.36E-10	KIAA1211	4q12	0.38522705	9.34E-14	1.05E-12
UNC119B	12q24.31	0.351975417	1.38E-11	1.38E-10	KLHL28	14q21.2	0.385111904	9.51E-14	1.07E-12
PPFIBP1	12p11.23-p1	0.35193382	1.39E-11	1.39E-10	ZNF469	16q24.2	0.385099945	9.53E-14	1.07E-12
CPD	17q11.2	0.351843558	1.41E-11	1.40E-10	ZNF543	19q13.43	0.38505638	9.59E-14	1.08E-12
CSGALNACT2	10q11.21	0.351790881	1.42E-11	1.41E-10	ARHGAP32	11q24.3	0.385015377	9.66E-14	1.08E-12
BMI1	10p12.2	0.351759559	1.42E-11	1.42E-10	CASC4	15q15.3	0.384981493	9.71E-14	1.09E-12
CTTNBP2NL	1p13.2	0.351750448	1.43E-11	1.42E-10	FOXJ2	12p13.31	0.384876709	9.87E-14	1.11E-12
ZFAND4	10q11.22	0.351728238	1.43E-11	1.42E-10	KLHL4	Xq21.31	0.38480511	9.99E-14	1.12E-12
ZNF160	19q13.41-q	0.351722543	1.43E-11	1.42E-10	SAMHD1	20q11.23	0.384724374	1.01E-13	1.13E-12
ZNF322	6p22.2	0.351664055	1.44E-11	1.43E-10	MXRA5	Xp22.33	0.384721242	1.01E-13	1.13E-12
CRTC3	15q26.1	0.35166104	1.44E-11	1.43E-10	RNF19A	8q22.2	0.384692768	1.02E-13	1.13E-12
HNRNPK	9q21.32	0.351658192	1.44E-11	1.43E-10	TBC1D19	4p15.2	0.384691914	1.02E-13	1.13E-12
ZNF660	3p21.31	0.351480759	1.48E-11	1.47E-10	HMG20A	15q24.3	0.384677107	1.02E-13	1.14E-12
ZNF570	19q13.12	0.351469695	1.48E-11	1.47E-10	RPL23AP53	8p23.3	0.384645786	1.02E-13	1.14E-12
FBXL19-AS1	16p11.2	0.351288032	1.52E-11	1.51E-10	B4GALNT3	12p13.33	0.38461233	1.03E-13	1.15E-12
BAZ2B	2q24.2	0.351222826	1.54E-11	1.52E-10	THRA	17q21.1	0.384567767	1.04E-13	1.15E-12
AHNAK	11q12.3	0.351145947	1.55E-11	1.54E-10	OSBPL11	3q21.2	0.384502277	1.05E-13	1.17E-12
FAM193A	4p16.3	0.351103805	1.56E-11	1.54E-10	KIAA0753	17p13.1	0.384491457	1.05E-13	1.17E-12
ENTPD7	10q24.2	0.351063372	1.57E-11	1.55E-10	LOC728024	8p11.23	0.384388644	1.07E-13	1.18E-12

DDI2	1p36.21	0.351014249	1.58E-11	1.56E-10	GLP2R	17p13.1	0.384296756	1.08E-13	1.20E-12
ZNF70	22q11.23	0.350998737	1.59E-11	1.56E-10	SESTD1	2q31.2	0.384240318	1.09E-13	1.21E-12
U2SURP	3q23	0.350950641	1.60E-11	1.57E-10	NUP205	7q33	0.384189234	1.10E-13	1.22E-12
MBD5	2q23.1	0.350904798	1.61E-11	1.58E-10	MTF1	1p34.3	0.384044986	1.13E-13	1.25E-12
PRKAR1A	17q24.2	0.350758869	1.64E-11	1.61E-10	MBNL1-AS1	3q25.1	0.383606773	1.21E-13	1.33E-12
INS-IGF2	11p15.5	0.350639311	1.67E-11	1.64E-10	APC	5q22.2	0.38360421	1.21E-13	1.33E-12
CDK17	12q23.1	0.350600671	1.68E-11	1.65E-10	TAF4B	18q11.2	0.383555963	1.22E-13	1.34E-12
KIAA1958	9q32	0.350559099	1.69E-11	1.65E-10	GNAQ	9q21.2	0.383544415	1.22E-13	1.34E-12
TMEM43	3p25.1	0.350543154	1.69E-11	1.66E-10	TFAM	10q21.1	0.383501989	1.23E-13	1.35E-12
DZIP3	3q13.13	0.350523792	1.70E-11	1.66E-10	EIF3A	10q26.11	0.383350793	1.26E-13	1.38E-12
BICD2	9q22.31	0.350457732	1.71E-11	1.67E-10	UBA6	4q13.2	0.383315485	1.26E-13	1.39E-12
TMED4	7p13	0.350450044	1.71E-11	1.68E-10	CCN3	8q24.12	0.383180803	1.29E-13	1.42E-12
FAM45BP	Xq26.1	0.350429828	1.72E-11	1.68E-10	PLEKHA7	11p15.2-p1	0.383141794	1.30E-13	1.43E-12
RAB18	10p12.1	0.350424728	1.72E-11	1.68E-10	GABPA	21q21.3	0.383084562	1.31E-13	1.44E-12
ACBD3	1q42.12	0.350324759	1.74E-11	1.70E-10	TRAM2	6p12.2	0.383013974	1.33E-13	1.45E-12
FAM118B	11q24.2	0.350228233	1.77E-11	1.73E-10	TM6SF1	15q25.2	0.382932511	1.34E-13	1.47E-12
FNBP4	11p11.2	0.350189223	1.78E-11	1.73E-10	DCAF12	9p13.3	0.382837151	1.36E-13	1.49E-12
ATP7B	13q14.3	0.35015477	1.79E-11	1.74E-10	ZNF235	19q13.31	0.382749993	1.38E-13	1.51E-12
ZMYM5	13q12.11	0.349984781	1.83E-11	1.78E-10	USP3	15q22.31	0.382713262	1.39E-13	1.52E-12
CCDC138	2q13	0.34955943	1.94E-11	1.89E-10	KBTBD2	7p14.3	0.382554662	1.43E-13	1.56E-12
SPTLC1	9q22.31	0.349388252	1.99E-11	1.93E-10	ZDHHC17	12q21.2	0.382346803	1.47E-13	1.61E-12
TWSG1	18p11.22	0.349221395	2.04E-11	1.98E-10	F3	1p21.3	0.382305231	1.48E-13	1.62E-12
APPL1	3p14.3	0.349139106	2.06E-11	2.00E-10	RHBDD1	2q36.3	0.382227497	1.50E-13	1.64E-12
SCART1	10q26.3	0.349088439	2.07E-11	2.01E-10	SLC37A3	7q34	0.382223795	1.50E-13	1.64E-12
GAB1	4q31.21	0.348974124	2.11E-11	2.05E-10	CDKN2AIP	4q35.1	0.382179661	1.51E-13	1.65E-12
AGO4	1p34.3	0.348820198	2.15E-11	2.09E-10	CSNK1G1	15q22.31	0.382126984	1.52E-13	1.66E-12
ATF6B	6p21.32	0.348792293	2.16E-11	2.09E-10	SPART	13q13.3	0.382095947	1.53E-13	1.66E-12
POU2F3	11q23.3	0.348747395	2.18E-11	2.11E-10	TPM4	19p13.12-p	0.381998282	1.56E-13	1.69E-12
VRK2	2p16.1	0.348641382	2.21E-11	2.14E-10	MAPK10	4q21.3	0.381915424	1.58E-13	1.71E-12
TUT4	1p32.3	0.348466837	2.26E-11	2.19E-10	NUP98	11p15.4	0.381867302	1.59E-13	1.72E-12
TECPR2	14q32.31	0.348211023	2.35E-11	2.27E-10	CTNNB1	3p22.1	0.381846516	1.59E-13	1.72E-12
APPBP2	17q23.2	0.348109799	2.38E-11	2.30E-10	WASHC2A	10q11.23	0.381839531	1.60E-13	1.72E-12
XIAP	Xq25	0.348071049	2.39E-11	2.31E-10	ZFP30	19q13.12	0.381770518	1.61E-13	1.74E-12
SAMD15	14q24.3	0.348027304	2.41E-11	2.32E-10	MON2	12q14.1	0.381764511	1.61E-13	1.74E-12
ELMO2	20q13.12	0.347998441	2.42E-11	2.33E-10	ELF4	Xq26.1	0.381550387	1.67E-13	1.80E-12
SPIN1	9q22.1	0.347968828	2.43E-11	2.34E-10	ZNF845	19q13.42	0.381467813	1.69E-13	1.82E-12
FBXO11	2p16.3	0.347968166	2.43E-11	2.34E-10	GPRASP1	Xq22.1	0.381315762	1.73E-13	1.86E-12
INCENP	11q12.3	0.347907324	2.45E-11	2.36E-10	TMOD2	15q21.2	0.381255967	1.75E-13	1.88E-12
FNIP1	5q31.1	0.347898782	2.45E-11	2.36E-10	LAMA5	20q13.33	0.381101069	1.79E-13	1.92E-12
ZNF426	19p13.2	0.34764347	2.54E-11	2.44E-10	KIAA0825	5q15	0.381075293	1.80E-13	1.93E-12
MTMR6	13q12.13	0.347629988	2.54E-11	2.44E-10	EBF2	8p21.2	0.381002386	1.82E-13	1.95E-12
YY1	14q32.2	0.347564214	2.57E-11	2.46E-10	COL4A2	13q34	0.380997709	1.82E-13	1.95E-12
SLC28A3	9q21.32-q21	0.34755298	2.57E-11	2.46E-10	CHST15	10q26.13	0.380933358	1.84E-13	1.97E-12
MCMBP	10q26.11	0.34744804	2.61E-11	2.50E-10	TNRC6B	22q13.1	0.380847936	1.86E-13	2.00E-12
MBLAC2	5q14.3	0.347183233	2.71E-11	2.59E-10	DDHD2	8p11.23	0.380795829	1.88E-13	2.01E-12
DIDO1	20q13.33	0.347116604	2.73E-11	2.61E-10	SLC33A1	3q25.31	0.380795544	1.88E-13	2.01E-12
ZNF136	19p13.2	0.347049405	2.76E-11	2.63E-10	AFAP1	4p16.1	0.380697024	1.91E-13	2.04E-12
B4GALT6	18q12.1	0.346937812	2.80E-11	2.67E-10	PRKD3	2p22.2	0.380610348	1.93E-13	2.06E-12
KIF16B	20p12.1	0.346923551	2.81E-11	2.68E-10	NAF1	4q32.2	0.380589108	1.94E-13	2.07E-12
NBPF10	1q21.1	0.346904213	2.81E-11	2.68E-10	ADAMTS7	15q25.1	0.380483497	1.97E-13	2.10E-12
P2RY12	3q25.1	0.34680916	2.85E-11	2.71E-10	LRRN2	1q32.1	0.380467721	1.98E-13	2.10E-12
ALDH1L2	12q23.3	0.346726821	2.89E-11	2.74E-10	ZNF407	18q22.3	0.380408584	2.00E-13	2.12E-12
LNX1	4q12	0.346633117	2.92E-11	2.78E-10	HNRNPR	1p36.12	0.38030323	2.03E-13	2.15E-12
NRSN1	6p22.3	0.346577569	2.95E-11	2.80E-10	FNIP2	4q32.1	0.380290417	2.03E-13	2.16E-12
ARL5B	10p12.31	0.346458856	2.99E-11	2.84E-10	GPD2	2q24.1	0.380072591	2.10E-13	2.23E-12
KIAA1217	10p12.2-p12	0.34636176	3.04E-11	2.88E-10	TMEM87B	2q13	0.38001906	2.12E-13	2.25E-12
POLDIP3	22q13.2	0.346300281	3.06E-11	2.90E-10	ENDOD1	11q21	0.379998844	2.13E-13	2.25E-12
ZSCAN23	6p22.1	0.346195511	3.11E-11	2.94E-10	MAP3K13	3q27.2	0.379966838	2.14E-13	2.26E-12
SEPTIN11	4q21.1	0.346187785	3.11E-11	2.94E-10	SMAD3	15q22.33	0.379955848	2.14E-13	2.27E-12
MBTPS2	Xp22.12	0.346139379	3.13E-11	2.96E-10	ZNF160	19q13.41-q	0.379953855	2.14E-13	2.27E-12
CHMP1B	18p11.21	0.346125309	3.14E-11	2.96E-10	SYNGAP1	6p21.32	0.379897504	2.16E-13	2.28E-12
NSMAF	8q12.1	0.346097523	3.15E-11	2.97E-10	ZNF597	16p13.3	0.379812909	2.19E-13	2.31E-12
LEF1	4q25	0.34601865	3.18E-11	3.00E-10	PLAT	8p11.21	0.379677089	2.24E-13	2.36E-12
GALNT4	12q21.33	0.346014403	3.19E-11	3.00E-10	PIP5K1A	1q21.3	0.379596508	2.27E-13	2.39E-12
TRAK2	2q33.1	0.345864631	3.25E-11	3.06E-10	DAPK1	9q21.33	0.379576006	2.27E-13	2.39E-12
PBX2	6p21.32	0.345720243	3.32E-11	3.12E-10	AMPD3	11p15.4	0.379542407	2.29E-13	2.40E-12
NUP98	11p15.4	0.345570471	3.39E-11	3.18E-10	ZFC3H1	12q21.1	0.379295646	2.38E-13	2.49E-12
CCDC15	11q24.2	0.34553692	3.40E-11	3.20E-10	GPR20	8q24.3	0.379293624	2.38E-13	2.49E-12
ZNF319	16q21	0.345500425	3.42E-11	3.21E-10	ZNF184	6p22.1	0.379280732	2.38E-13	2.50E-12
TTC21B	2q24.3	0.345489889	3.43E-11	3.21E-10	SMC2	9q31.1	0.379094797	2.45E-13	2.57E-12
CHD1	5q15-q21.1	0.345310788	3.51E-11	3.29E-10	USF3	3q13.2	0.379040697	2.47E-13	2.59E-12
ZNF585B	19q13.12	0.345233079	3.55E-11	3.32E-10	POLR2M	15q21.3	0.379004535	2.49E-13	2.60E-12
ABRAXAS2	10q26.13	0.345211012	3.56E-11	3.33E-10	KLF3	4p14	0.37896638	2.50E-13	2.62E-12
BMT2	7q31.1	0.345162155	3.58E-11	3.35E-10	CKAP5	11p11.2	0.378788703	2.57E-13	2.69E-12

NCOA1	2p23.3	0.345131972	3.60E-11	3.37E-10	IFT57	3q13.12-q1	0.378695593	2.61E-13	2.73E-12
ABRAXAS1	4q21.23	0.345128555	3.60E-11	3.37E-10	NFIX	19p13.13	0.378688074	2.61E-13	2.73E-12
SHH	7q36.3	0.345006245	3.66E-11	3.42E-10	ZNF284	19q13.31	0.378666083	2.62E-13	2.73E-12
ATP2C1	3q22.1	0.344947461	3.69E-11	3.45E-10	SMNDC1	10q25.2	0.37860049	2.65E-13	2.76E-12
BRCA2	13q13.1	0.34493183	3.70E-11	3.45E-10	GPR173	Xp11.22	0.378447066	2.71E-13	2.82E-12
KSR1	17q11.2	0.344929808	3.70E-11	3.45E-10	CARF	2q33.2	0.378342232	2.76E-13	2.87E-12
CASC4	15q15.3	0.34492155	3.71E-11	3.45E-10	TTC26	7q34	0.378315209	2.77E-13	2.88E-12
PHF21A	11p11.2	0.344915595	3.71E-11	3.45E-10	CHRNA7	15q13.3	0.37821827	2.81E-13	2.92E-12
NME9	3q22.3	0.344858778	3.74E-11	3.48E-10	PPP1R2	3q29	0.378117288	2.85E-13	2.96E-12
ZNF566	19q13.12	0.344855206	3.74E-11	3.48E-10	ISLR2	15q24.1	0.378040506	2.89E-13	3.00E-12
SPRY1	4q28.1	0.344834135	3.75E-11	3.49E-10	KRIT1	7q21.2	0.377942173	2.93E-13	3.04E-12
BAMBI	10p12.1	0.344790855	3.77E-11	3.51E-10	NOS3	7q36.1	0.377937333	2.93E-13	3.04E-12
OLR1	12p13.2	0.34473741	3.80E-11	3.53E-10	TMEM65	8q24.13	0.377862189	2.97E-13	3.07E-12
CEP164	11q23.3	0.344692644	3.82E-11	3.55E-10	EGR2	10q21.3	0.377848548	2.97E-13	3.08E-12
ANKRD40	17q21.33	0.34467098	3.84E-11	3.56E-10	CD200	3q13.2	0.377663414	3.06E-13	3.16E-12
ZNF510	9q22.33	0.344653611	3.85E-11	3.57E-10	MAU2	19p13.11	0.377619279	3.08E-13	3.18E-12
KTN1	14q22.3	0.344622574	3.86E-11	3.58E-10	SMC5	9q21.12	0.377544393	3.12E-13	3.22E-12
KLHL24	3q27.1	0.344565911	3.89E-11	3.60E-10	IRAK3	12q14.3	0.377309225	3.23E-13	3.33E-12
GNL3L	Xp11.22	0.344540569	3.91E-11	3.61E-10	USP25	21q21.1	0.377300087	3.24E-13	3.34E-12
CNOT6	5q35.3	0.344504123	3.93E-11	3.63E-10	TCHH	1q21.3	0.377234176	3.27E-13	3.37E-12
PFAS	17p13.1	0.344249566	4.07E-11	3.76E-10	SCD5	4q21.22	0.377224916	3.28E-13	3.37E-12
TENT2	5q14.1	0.344068472	4.17E-11	3.85E-10	PIGN	18q21.33	0.377093081	3.34E-13	3.44E-12
TINAGL1	1p35.2	0.344030317	4.19E-11	3.87E-10	AGFG1	2q36.3	0.377023801	3.38E-13	3.47E-12
ZC3H4	19q13.32	0.343823881	4.31E-11	3.98E-10	ABLIM1	10q25.3	0.376855894	3.47E-13	3.56E-12
OSMR	5p13.1	0.343798254	4.33E-11	3.99E-10	PIK3CB	3q22.3	0.376621838	3.60E-13	3.68E-12
APAF1	12q23.1	0.343767503	4.35E-11	4.00E-10	CCDC82	11q21	0.376602476	3.61E-13	3.69E-12
PCDHGA10	5q31.3	0.343706942	4.38E-11	4.03E-10	CSPP1	8q13.1-q13	0.376544959	3.64E-13	3.72E-12
TRIM32	9q33.1	0.343653607	4.41E-11	4.06E-10	SETD2	3p21.31	0.376513922	3.66E-13	3.74E-12
ZNF142	2q35	0.343586124	4.46E-11	4.10E-10	COPB1	11p15.2	0.376491054	3.67E-13	3.75E-12
IFT81	12q24.11	0.343550531	4.48E-11	4.11E-10	MTMR9	8p23.1	0.376428243	3.71E-13	3.78E-12
KIAA0586	14q23.1	0.34351978	4.50E-11	4.13E-10	KCNQ3	8q24.22	0.376382093	3.73E-13	3.80E-12
STK36	2q35	0.343411579	4.56E-11	4.18E-10	CALU	7q32.1	0.376346211	3.75E-13	3.82E-12
PDZD2	5p13.3	0.343207421	4.69E-11	4.29E-10	ZNF703	8p11.23	0.376336841	3.76E-13	3.83E-12
TAF3	10p14	0.343023788	4.81E-11	4.40E-10	PTGS2	1q31.1	0.376327929	3.76E-13	3.83E-12
DENND1B	1q31.3	0.342653034	5.06E-11	4.62E-10	CAMSAP1	9q34.3	0.376316883	3.77E-13	3.84E-12
AREL1	14q24.3	0.34262456	5.08E-11	4.64E-10	NBPF14	1q21.2	0.376157144	3.86E-13	3.93E-12
FERMT1	20p12.3	0.342550423	5.14E-11	4.68E-10	DR1	1p22.1	0.376040401	3.93E-13	3.99E-12
MIA2	14q13.2	0.342548636	5.14E-11	4.68E-10	SMAD7	18q21.1	0.375935617	4.00E-13	4.06E-12
NUDT21	16q13	0.342543979	5.14E-11	4.68E-10	ZNF608	5q23.2	0.375892621	4.02E-13	4.08E-12
USO1	4q21.1	0.342492441	5.18E-11	4.71E-10	CALCRL	2q32.1	0.37588294	4.03E-13	4.08E-12
MAPK14	6p21.31	0.342430083	5.22E-11	4.75E-10	WDR3	1p12	0.375718076	4.13E-13	4.18E-12
ZNF772	19q13.43	0.342424388	5.23E-11	4.75E-10	SPTLC1	9q22.31	0.375496549	4.28E-13	4.33E-12
FILIP1L	3q12.1	0.342342953	5.28E-11	4.80E-10	RBL2	16q12.2	0.375493159	4.28E-13	4.33E-12
COL27A1	9q32	0.342188624	5.40E-11	4.90E-10	PCDHB18P	5q31.3	0.375426222	4.32E-13	4.37E-12
SNCAIP	5q23.2	0.342182645	5.40E-11	4.90E-10	TCF4	18q21.2	0.375412267	4.33E-13	4.37E-12
LARP1	5q33.2	0.34203458	5.51E-11	5.00E-10	BAZ1B	7q11.23	0.375300934	4.41E-13	4.45E-12
STK35	20p13	0.342026323	5.52E-11	5.00E-10	SLC39A10	2q32.3	0.375149737	4.51E-13	4.55E-12
UGCG	9q31.3	0.341942894	5.58E-11	5.06E-10	PPP1R12B	1q32.1	0.375118701	4.53E-13	4.57E-12
ZNHIT6	1p22.3	0.341938623	5.58E-11	5.06E-10	LIFR	5p13.1	0.37501534	4.61E-13	4.64E-12
FMNL2	2q23.3	0.341919261	5.60E-11	5.07E-10	PCDHGB6	5q31.3	0.374979036	4.63E-13	4.66E-12
CASS4	20q13.31	0.341854723	5.65E-11	5.10E-10	MAPKBP1	15q15.1	0.37474159	4.80E-13	4.83E-12
SPIN4	Xq11.1	0.341823019	5.67E-11	5.12E-10	ACBD3	1q42.12	0.374738004	4.81E-13	4.83E-12
FCHSD2	11q13.4	0.341811345	5.68E-11	5.13E-10	SOCS4	14q22.3	0.374635214	4.88E-13	4.90E-12
TUG1	22q12.2	0.341761231	5.72E-11	5.16E-10	PCDHAC2	5q31.3	0.374626956	4.89E-13	4.90E-12
BAZ2A	12q13.3	0.34175639	5.73E-11	5.16E-10	SULT1C4	2q12.3	0.374562153	4.94E-13	4.95E-12
JADE3	Xp11.3	0.341754967	5.73E-11	5.16E-10	SDE2	1q42.12	0.374523026	4.97E-13	4.98E-12
CSGALNACT1	8p21.3	0.341752689	5.73E-11	5.16E-10	CARMIL1	6p22.2	0.374497969	4.99E-13	4.99E-12
EXOC1	4q12	0.341564476	5.88E-11	5.29E-10	ITPRID2	2q31.3	0.374470065	5.01E-13	5.01E-12
RHBDD1	2q36.3	0.34156049	5.88E-11	5.29E-10	DOCK10	2q36.2	0.374384074	5.07E-13	5.08E-12
GAPVD1	9q33.3	0.341363735	6.04E-11	5.43E-10	NFE2L2	2q31.2	0.374298652	5.14E-13	5.14E-12
WWP1	8q21.3	0.341331275	6.07E-11	5.45E-10	STXBP3	1p13.3	0.374133103	5.27E-13	5.27E-12
TMEM245	9q31.3	0.341295113	6.10E-11	5.47E-10	FLRT2	14q31.3	0.374125948	5.28E-13	5.27E-12
CLUHP3	16p11.2	0.341254989	6.13E-11	5.50E-10	ANKFY1	17p13.2	0.374064312	5.33E-13	5.31E-12
CCDC121	2p23.3	0.341219942	6.16E-11	5.52E-10	ANK3	10q21.2	0.374042956	5.35E-13	5.33E-12
ARHGAP29	1p22.1	0.341186627	6.19E-11	5.55E-10	THRAP3	1p34.3	0.374024902	5.36E-13	5.34E-12
DIP2A	21q22.3	0.34116214	6.21E-11	5.56E-10	SNORD116-4	15q11.2	0.374024168	5.36E-13	5.34E-12
USP40	2q37.1	0.341142778	6.23E-11	5.57E-10	GSK3B	3q13.33	0.373955826	5.42E-13	5.39E-12
ZNF286A	17p12	0.341119026	6.25E-11	5.59E-10	SLC6A17	1p13.3	0.373850831	5.51E-13	5.47E-12
SNX29	16p13.13-p1	0.341057608	6.30E-11	5.63E-10	TNFAIP6	2q23.3	0.373676698	5.65E-13	5.62E-12
ZNF717	3p12.3	0.34083141	6.50E-11	5.80E-10	DST	6p12.1	0.373635779	5.69E-13	5.65E-12
EIF4B	12q13.13	0.340764075	6.56E-11	5.85E-10	SMCR8	17p11.2	0.373564025	5.75E-13	5.71E-12
TXNDC5	6p24.3	0.340749553	6.57E-11	5.86E-10	AFAP1L1	5q32	0.373546683	5.77E-13	5.72E-12
SLC38A7	16q21	0.340716238	6.60E-11	5.88E-10	MSL2	3q22.3	0.373362999	5.93E-13	5.88E-12
CHORDC1	11q14.3	0.340702001	6.61E-11	5.89E-10	ARHGEF10	8p23.3	0.37335847	5.94E-13	5.88E-12

PPTC7	12q24.11	0.340604051	6.70E-11	5.97E-10	GLS	2q32.2	0.373291815	6.00E-13	5.93E-12
DISC1	1q42.2	0.340598356	6.71E-11	5.97E-10	KCTD9	8p21.2	0.372898305	6.37E-13	6.28E-12
TUBGCP4	15q15.3	0.340535193	6.76E-11	6.02E-10	RCBTB1	13q14.2	0.37287541	6.39E-13	6.30E-12
ZNF347	19q13.42	0.340419664	6.87E-11	6.11E-10	SLC30A9	4p13	0.372846483	6.42E-13	6.33E-12
ZZZ3	1p31.1	0.340414984	6.87E-11	6.11E-10	SNX13	7p21.1	0.372690161	6.57E-13	6.47E-12
ELP1	9q31.3	0.340342945	6.94E-11	6.17E-10	MYLK	3q21.1	0.372623247	6.64E-13	6.53E-12
NRXN3	14q24.3-q3	0.340321031	6.96E-11	6.18E-10	ESRRG	1q41	0.372600981	6.66E-13	6.55E-12
SPIN3	Xp11.21	0.340195735	7.08E-11	6.29E-10	ARFGEF2	20q13.13	0.372485602	6.78E-13	6.66E-12
NEPRO	3q13.2	0.340044824	7.23E-11	6.41E-10	FAM193A	4p16.3	0.372182756	7.10E-13	6.96E-12
PRKDC	8q11.21	0.339913274	7.36E-11	6.52E-10	CASC15	6p22.3	0.372152706	7.13E-13	6.99E-12
PDGFRB	5q32	0.339809084	7.46E-11	6.61E-10	IGSF9B	11q25	0.372141559	7.15E-13	7.00E-12
INTS4P1	7q11.21	0.339603459	7.68E-11	6.79E-10	ADCY7	16q12.1	0.372087083	7.20E-13	7.05E-12
HRNR	1q21.3	0.339573658	7.71E-11	6.82E-10	GATAD2B	1q21.3	0.372079396	7.21E-13	7.05E-12
PPP4R1	18p11.22	0.339506121	7.78E-11	6.87E-10	PAPPA	9q33.1	0.372009965	7.29E-13	7.12E-12
DDX42	17q23.3	0.339499454	7.79E-11	6.87E-10	ZNHIT6	1p22.3	0.371920226	7.39E-13	7.21E-12
WDR47	1p13.3	0.339490437	7.79E-11	6.88E-10	HDAC9	7p21.1	0.37191285	7.40E-13	7.22E-12
NCBP3	17p13.2	0.339410994	7.88E-11	6.95E-10	EOGT	3p14.1	0.371905989	7.41E-13	7.22E-12
TMEM51-AS1	1p36.21	0.33940739	7.88E-11	6.95E-10	NIBAN2	9q34.11	0.371794372	7.53E-13	7.34E-12
VPS11	11q23.3	0.339307349	7.99E-11	7.04E-10	EDNRB	13q22.3	0.371764189	7.57E-13	7.37E-12
IPW	15q11.2	0.33921367	8.09E-11	7.13E-10	UBAP2	9p13.3	0.371693859	7.65E-13	7.44E-12
C5ORF24	5q31.1	0.339160709	8.15E-11	7.17E-10	ARMCX1	Xq22.1	0.371683608	7.66E-13	7.45E-12
ARHGAP21	10p12.1|10p	0.339082121	8.24E-11	7.24E-10	WDFY1	2q36.1	0.371644884	7.71E-13	7.49E-12
EP400	12q24.33	0.339019075	8.31E-11	7.30E-10	ZNF117	7q11.21	0.371603481	7.75E-13	7.53E-12
ZNF718	4p16.3	0.338986733	8.35E-11	7.33E-10	CEP290	12q21.32	0.371505361	7.87E-13	7.64E-12
SMG6	17p13.3	0.338952564	8.38E-11	7.36E-10	RP1L1	8p23.1	0.371391614	8.01E-13	7.77E-12
TMEM39A	3q13.33	0.338912132	8.43E-11	7.39E-10	DLG5	10q22.3	0.371111567	8.35E-13	8.09E-12
DMTF1	7q21.12	0.338846926	8.50E-11	7.45E-10	SOS2	14q21.3	0.37108167	8.39E-13	8.12E-12
GDPD1	17q22	0.338782956	8.58E-11	7.52E-10	ZNF45	19q13.31	0.371062307	8.42E-13	8.14E-12
NARS2	11q14.1	0.338743851	8.62E-11	7.55E-10	CH25H	10q23.31	0.371043541	8.44E-13	8.16E-12
NAALADL2	3q26.31	0.338741486	8.63E-11	7.55E-10	PELI2	14q22.3	0.370989868	8.51E-13	8.22E-12
ARSB	5q14.1	0.338612895	8.78E-11	7.68E-10	RBBP4	1p35.1	0.370932493	8.58E-13	8.29E-12
XPOT	12q14.2	0.338576424	8.82E-11	7.71E-10	LRRK1	15q26.3	0.370838218	8.71E-13	8.40E-12
ZNF649	19q13.41	0.338561618	8.84E-11	7.72E-10	ARAP3	5q31.3	0.370784972	8.78E-13	8.46E-12
CTTNBP2	7q31.31	0.338553664	8.85E-11	7.73E-10	LXN	3q25.32	0.370744565	8.83E-13	8.51E-12
MLH3	14q24.3	0.338303644	9.15E-11	7.99E-10	VPS54	2p15-p14	0.370737705	8.84E-13	8.51E-12
WDR26	1q42.11-q4	0.338272038	9.19E-11	8.02E-10	CAP1	1p34.2	0.370712078	8.88E-13	8.54E-12
ESYT2	7q36.3	0.338245273	9.22E-11	8.04E-10	CSRNP2	12q13.12	0.370696133	8.90E-13	8.56E-12
LINC00662	19q11	0.338178074	9.31E-11	8.11E-10	TNFSF13	17p13.1	0.370602169	9.02E-13	8.67E-12
ACLY	17q21.2	0.338110021	9.39E-11	8.18E-10	SLC9A1	1p36.11	0.370549208	9.10E-13	8.73E-12
MLLT6	17q12	0.33803542	9.49E-11	8.26E-10	LPAR1	9q31.3	0.37054806	9.10E-13	8.73E-12
PIK3C3	18q12.3	0.338007967	9.52E-11	8.28E-10	ZNFX1	20q13.13	0.370396187	9.31E-13	8.92E-12
TBRG1	11q24.2	0.337971093	9.57E-11	8.32E-10	NBPF1	1p36.13	0.370333091	9.40E-13	9.00E-12
INPP5F	10q26.11	0.337862583	9.71E-11	8.44E-10	EML1	14q32.2	0.370232578	9.54E-13	9.13E-12
NAA16	13q14.11	0.337837526	9.75E-11	8.47E-10	RAD50	5q31.1	0.370211792	9.57E-13	9.16E-12
ZNF608	5q23.2	0.337763494	9.84E-11	8.55E-10	IPO7	11p15.4	0.370186165	9.61E-13	9.19E-12
HIP1	7q11.23	0.337695038	9.94E-11	8.62E-10	PAPSS1	4q25	0.370089639	9.75E-13	9.31E-12
MNT	17p13.3	0.337559051	1.01E-10	8.77E-10	C3ORF52	3q13.2	0.370089569	9.75E-13	9.31E-12
SEMA4C	2q11.2	0.337471067	1.02E-10	8.88E-10	NCAPD3	11q25	0.37008793	9.75E-13	9.31E-12
C2CD5	12p12.1	0.337398174	1.03E-10	8.96E-10	ITGA3	17q21.33	0.370019308	9.85E-13	9.41E-12
TBL1XR1	3q26.32	0.337211385	1.06E-10	9.17E-10	TCAF1	7q35	0.369920219	1.00E-12	9.54E-12
BACH1	21q21.3	0.33706446	1.08E-10	9.35E-10	TTF2	1p13.1	0.369667371	1.04E-12	9.91E-12
APCDD1	18p11.22	0.337051931	1.08E-10	9.37E-10	RBM27	5q32	0.369527732	1.06E-12	1.01E-11
ZNF740	12q13.13	0.336901304	1.11E-10	9.55E-10	CDC27	17q21.32	0.369423233	1.08E-12	1.03E-11
CASP8AP2	6q15	0.336889345	1.11E-10	9.56E-10	PATJ	1p31.3	0.369339921	1.09E-12	1.04E-11
PLAGL2	20q11.21	0.336887376	1.11E-10	9.56E-10	RBM41	Xq22.3	0.369245582	1.11E-12	1.05E-11
NOLC1	10q24.32	0.33685036	1.11E-10	9.60E-10	TNKS	8p23.1	0.369224058	1.11E-12	1.06E-11
NATD1	17p11.2	0.336712522	1.13E-10	9.77E-10	KTN1	14q22.3	0.369198975	1.12E-12	1.06E-11
DST	6p12.1	0.336673513	1.14E-10	9.82E-10	PALLD	4q32.3	0.369189578	1.12E-12	1.06E-11
PCDHGB2	5q31.3	0.336610052	1.15E-10	9.89E-10	UBA3	3p14.1	0.369129525	1.13E-12	1.07E-11
FBXL3	13q22.3	0.336594925	1.15E-10	9.91E-10	MINK1	17p13.2	0.369064008	1.14E-12	1.08E-11
METTL24	6q21	0.336549545	1.16E-10	9.96E-10	GOLGA7B	10q24.2	0.369021558	1.15E-12	1.09E-11
WDPCP	2p15	0.336524025	1.16E-10	9.99E-10	SLC16A4	1p13.3	0.369009444	1.15E-12	1.09E-11
FAM3B	21q22.3	0.336507435	1.17E-10	1.00E-09	ST6GAL2	2q12.3	0.368882614	1.17E-12	1.11E-11
ZNF678	1q42.13	0.336392238	1.18E-10	1.02E-09	KLHL5	4p14	0.36887722	1.17E-12	1.11E-11
ADAMTS9	3p14.1	0.336364476	1.19E-10	1.02E-09	CENPC	4q13.2	0.368755921	1.19E-12	1.12E-11
CAPN7	3p25.1	0.336253523	1.21E-10	1.03E-09	ARNT2	15q25.1	0.368600379	1.22E-12	1.15E-11
FAM135A	6q13	0.336250106	1.21E-10	1.03E-09	CYSLTR1	Xq21.1	0.368564127	1.23E-12	1.16E-11
ODAM	4q13.3	0.336249745	1.21E-10	1.03E-09	BACH1	21q21.3	0.368441284	1.25E-12	1.18E-11
PPP6R3	11q13.2	0.336218524	1.21E-10	1.04E-09	PCDHGC5	5q31.3	0.368413069	1.26E-12	1.18E-11
FOXJ3	1p34.2	0.336205971	1.21E-10	1.04E-09	ICE2	15q22.2	0.368403867	1.26E-12	1.18E-11
TADA2A	17q12	0.336205971	1.21E-10	1.04E-09	DACT1	14q23.1	0.368396037	1.26E-12	1.18E-11
IGFBP5	2q35	0.336186039	1.22E-10	1.04E-09	EFCAB6	22q13.2-q1	0.368175343	1.30E-12	1.22E-11
TMEM127	2q11.2	0.335920426	1.26E-10	1.08E-09	RNF115	1q21.1	0.368115827	1.31E-12	1.23E-11
ZMYM3	Xq13.1	0.335888202	1.27E-10	1.08E-09	ENC1	5q13.3	0.368087923	1.32E-12	1.24E-11

NID2	14q22.1	0.335635639	1.31E-10	1.12E-09	PHLPP2	16q22.2	0.368059734	1.32E-12	1.24E-11
CSF1	1p13.3	0.335600616	1.32E-10	1.12E-09	SF3A1	22q12.2	0.36805831	1.32E-12	1.24E-11
UBA6	4q13.2	0.335582677	1.32E-10	1.12E-09	CEP192	18p11.21	0.367993674	1.34E-12	1.25E-11
ANGPTL2	9q33.3	0.335510354	1.33E-10	1.13E-09	UBE2Q2P1	15q25.2	0.367970678	1.34E-12	1.25E-11
STAU2	8q21.11	0.335459101	1.34E-10	1.14E-09	SPRED3	19q13.2	0.367954036	1.34E-12	1.26E-11
LUC7L2	7q34	0.335399472	1.35E-10	1.15E-09	MORC3	21q22.12	0.36792733	1.35E-12	1.26E-11
EHMT1	9q34.3	0.335362859	1.36E-10	1.16E-09	SLC7A6OS	16q22.1	0.367872402	1.36E-12	1.27E-11
EXD2	14q24.1	0.335358303	1.36E-10	1.16E-09	NFASC	1q32.1	0.367746236	1.39E-12	1.29E-11
SWAP70	11p15.4	0.335295376	1.37E-10	1.16E-09	FAM126B	2q33.1	0.367575276	1.42E-12	1.33E-11
IGDCC3	15q22.31	0.335187882	1.39E-10	1.18E-09	TCF20	22q13.2|22c	0.367562864	1.43E-12	1.33E-11
PTPRK	6q22.33	0.335187744	1.39E-10	1.18E-09	AHCYL2	7q32.1	0.367458417	1.45E-12	1.35E-11
UBXN4	2q21.3	0.335166697	1.39E-10	1.18E-09	SYT11	1q22	0.367328524	1.48E-12	1.37E-11
JAG1	20p12.2	0.335134783	1.40E-10	1.19E-09	SPRY1	4q28.1	0.367224594	1.50E-12	1.39E-11
ZNF761	19q13.42	0.335102607	1.41E-10	1.19E-09	INO80D	2q33.3	0.367224309	1.50E-12	1.39E-11
PCDHB8	5q31.3	0.334947127	1.44E-10	1.21E-09	PRPF38B	1p13.3	0.367170377	1.51E-12	1.40E-11
MORC3	21q22.12	0.334912401	1.44E-10	1.22E-09	BNIP3L	8p21.2	0.367102441	1.53E-12	1.42E-11
HIPK1	1p13.2	0.334797367	1.46E-10	1.24E-09	RBMS1	2q24.2	0.366859274	1.58E-12	1.47E-11
HNRNPA1P33	10q11.22	0.334727036	1.48E-10	1.25E-09	ADAMTS2	5q35.3	0.366813715	1.59E-12	1.48E-11
MIER1	1p31.3	0.334719918	1.48E-10	1.25E-09	UACA	15q23	0.366618838	1.64E-12	1.52E-11
SAP130	2q14.3	0.334647879	1.49E-10	1.26E-09	ZNF507	19q13.11	0.366569694	1.65E-12	1.53E-11
SLC13A3	20q13.12	0.334295657	1.57E-10	1.32E-09	CHST4	16q22.2	0.366524072	1.66E-12	1.54E-11
CIC	19q13.2	0.334272023	1.57E-10	1.32E-09	MAPK1	22q11.22	0.366490136	1.67E-12	1.54E-11
FAT1	4q35.2	0.334103742	1.61E-10	1.35E-09	PHACTR2	6q24.2	0.366484842	1.67E-12	1.54E-11
TRIM24	7q33-q34	0.334006362	1.63E-10	1.37E-09	DNAL1	14q24.3	0.366459785	1.68E-12	1.55E-11
DUSP19	2q32.1	0.333990986	1.63E-10	1.37E-09	THOC2	Xq25	0.366400844	1.70E-12	1.56E-11
LMBRD2	5p13.2	0.33398643	1.63E-10	1.37E-09	ARHGAP18	6q22.33	0.366187859	1.75E-12	1.61E-11
BICD1	12p11.21	0.33380574	1.67E-10	1.40E-09	MASTL	10p12.1	0.366083243	1.78E-12	1.63E-11
SLAIN2	4p11	0.333785974	1.68E-10	1.40E-09	DCAF1	3p21.2	0.366081936	1.78E-12	1.63E-11
GTF3C2	2p23.3	0.333754368	1.68E-10	1.41E-09	HS3ST3A1	17p12	0.366034695	1.79E-12	1.64E-11
HEATR3	16q12.1	0.333547647	1.73E-10	1.44E-09	IREB2	15q25.1	0.365813142	1.85E-12	1.70E-11
MARK2	11q13.1	0.33348033	1.75E-10	1.46E-09	MAP1B	5q13.2	0.365791502	1.86E-12	1.70E-11
ZNF443	19p13.2	0.333476486	1.75E-10	1.46E-09	PPM1D	17q23.2	0.365633187	1.90E-12	1.74E-11
ZC3HAV1L	7q34	0.333412628	1.76E-10	1.47E-09	ANKRD36B	2q11.2	0.365613998	1.91E-12	1.74E-11
NUP205	7q33	0.333409999	1.76E-10	1.47E-09	NUDT21	16q13	0.365534667	1.93E-12	1.76E-11
RAB8B	15q22.2	0.33330448	1.79E-10	1.49E-09	DIP2A	21q22.3	0.365458642	1.95E-12	1.78E-11
PTPN4	2q14.2	0.333227885	1.80E-10	1.50E-09	CREB3L2	7q33	0.365378346	1.97E-12	1.80E-11
ARHGAP1	11p11.2	0.333218773	1.81E-10	1.50E-09	KIAA1107	1p22.1	0.365359294	1.98E-12	1.81E-11
PARP11	12p13.32	0.333203967	1.81E-10	1.51E-09	ARFGEF1	8q13.2	0.365343323	1.98E-12	1.81E-11
ZC3H7A	16p13.13	0.333022612	1.85E-10	1.54E-09	LPCAT2	16q12.2	0.365307161	2.00E-12	1.82E-11
PLSCR4	3q24	0.333004365	1.86E-10	1.54E-09	HACE1	6q16.3	0.365243664	2.01E-12	1.83E-11
TRIM67	1q42.2	0.332712103	1.93E-10	1.60E-09	FRMD4A	10p13	0.365219176	2.02E-12	1.84E-11
CFAP221	2q14.2	0.332493959	1.99E-10	1.65E-09	MAP9	4q32.1	0.365199933	2.03E-12	1.84E-11
PDS5A	4p14	0.332418656	2.01E-10	1.66E-09	KDM4C	9p24.1	0.365185008	2.03E-12	1.85E-11
MYORG	9p13.3	0.332337791	2.03E-10	1.68E-09	TBC1D5	3p24.3	0.365095199	2.06E-12	1.87E-11
AP3B1	5q14.1	0.332269738	2.05E-10	1.69E-09	LNX1	4q12	0.365041499	2.08E-12	1.89E-11
ASXL1	20q11.21	0.332268765	2.05E-10	1.69E-09	UHRF2	9p24.1	0.365015873	2.08E-12	1.89E-11
DYNLL2	17q22	0.332181754	2.07E-10	1.71E-09	PLK2	5q11.2	0.364982274	2.09E-12	1.90E-11
TBC1D14	4p16.1	0.332112562	2.09E-10	1.73E-09	CHD8	14q11.2	0.364913367	2.12E-12	1.92E-11
QRICH1	3p21.31	0.332061499	2.11E-10	1.74E-09	FBXL14	12p13.33	0.364881476	2.13E-12	1.93E-11
RASGRF1	15q25.1	0.332050058	2.11E-10	1.74E-09	BTC	4q13.3	0.364868887	2.13E-12	1.93E-11
TRIM4	7q22.1	0.332023723	2.12E-10	1.74E-09	AGO3	1p34.3	0.364857843	2.13E-12	1.93E-11
ANKRD52	12q13.3	0.331955955	2.14E-10	1.76E-09	MYO6	6q14.1	0.364772136	2.16E-12	1.96E-11
PRKCI	3q26.2	0.331777305	2.19E-10	1.80E-09	EME2	16p13.3	0.364769059	2.16E-12	1.96E-11
HFE	6p22.2	0.331595191	2.24E-10	1.84E-09	SUCNR1	3q25.1	0.364515737	2.24E-12	2.03E-11
CFAP126	1q23.3	0.331556167	2.25E-10	1.85E-09	IFI16	1q23.1	0.364504197	2.25E-12	2.03E-11
ARL15	5q11.2	0.331542799	2.25E-10	1.85E-09	CDK8	13q12.13	0.364492057	2.25E-12	2.04E-11
RBM18	9q33.2	0.331523721	2.26E-10	1.86E-09	DPYSL3	5q32	0.364429595	2.27E-12	2.05E-11
NNAT	20q11.23	0.331382234	2.30E-10	1.89E-09	CHMP3	2p11.2	0.364351887	2.30E-12	2.08E-11
PTPN1	20q13.13	0.331170811	2.37E-10	1.94E-09	USP6NL	10p14	0.364307727	2.31E-12	2.09E-11
ANO6	12q12	0.331136476	2.38E-10	1.95E-09	CYFIP1	15q11.2	0.36430374	2.32E-12	2.09E-11
KDM6B	17p13.1	0.331101169	2.39E-10	1.96E-09	ZNF829	19q13.12	0.364244515	2.34E-12	2.10E-11
NHSL2	Xq13.1	0.331082696	2.40E-10	1.96E-09	HP1BP3	1p36.12	0.36418415	2.36E-12	2.12E-11
YME1L1	10p12.1	0.330894733	2.46E-10	2.01E-09	MBOAT4	8p12	0.364176982	2.36E-12	2.12E-11
SACS	13q12.12	0.330851168	2.47E-10	2.02E-09	ZNF225	19q13.31	0.36405801	2.40E-12	2.16E-11
SLC25A40	7q21.12	0.330832944	2.48E-10	2.02E-09	DIP2B	12q13.12	0.364052626	2.40E-12	2.16E-11
VPS4B	18q21.33	0.330831805	2.48E-10	2.02E-09	RICTOR	5p13.1	0.364031154	2.41E-12	2.16E-11
SMG8	17q22	0.330679755	2.53E-10	2.06E-09	NUDT11	Xp11.22	0.363704654	2.53E-12	2.26E-11
RTF1	15q15.1	0.330627078	2.54E-10	2.07E-09	UVSSA	4p16.3	0.363700378	2.53E-12	2.26E-11
RNF216	7p22.1	0.330620529	2.55E-10	2.07E-09	RNMT	18p11.21	0.363655105	2.55E-12	2.28E-11
6-Mar	5p15.2	0.330560734	2.57E-10	2.09E-09	LRRFIP1	2q37.3	0.363565697	2.58E-12	2.31E-11
KLHL9	9p21.3	0.330554185	2.57E-10	2.09E-09	AKAP9	7q21.2	0.363493942	2.61E-12	2.33E-11
ZNF561	19p13.2	0.330466793	2.60E-10	2.11E-09	KIF16B	20p12.1	0.363458635	2.62E-12	2.34E-11
SFMBT2	10p14	0.33045741	2.60E-10	2.11E-09	ZFYVE9	1p32.3	0.363384318	2.65E-12	2.37E-11
LSM14A	19q13.11	0.330242419	2.68E-10	2.17E-09	SHANK1	19q13.33	0.36337864	2.65E-12	2.37E-11

ZFP28	19q13.43	0.329895488	2.80E-10	2.27E-09	YWHAG	7q11.23	0.363373782	2.66E-12	2.37E-11
ZNF749	19q13.43	0.329878214	2.81E-10	2.27E-09	STK4	20q13.12	0.363339614	2.67E-12	2.38E-11
PROB1	5q31.2	0.329763375	2.85E-10	2.31E-09	KANSL1L	2q34	0.363302908	2.68E-12	2.39E-11
ZCCHC2	18q21.33	0.329622234	2.90E-10	2.35E-09	PBRM1	3p21.1	0.363220023	2.72E-12	2.42E-11
TM9SF3	10q24.1	0.329499511	2.95E-10	2.38E-09	BLZF1	1q24.2	0.36309303	2.77E-12	2.46E-11
FUT10	8p12	0.329409272	2.98E-10	2.41E-09	PCDHGA2	5q31.3	0.363028496	2.80E-12	2.48E-11
CUL4A	13q34	0.329397598	2.99E-10	2.41E-09	LHFPL6	13q13.3-q1	0.362893712	2.85E-12	2.53E-11
ZHX2	8q24.13	0.329279693	3.03E-10	2.45E-09	ZNF516	18q23	0.362854988	2.87E-12	2.54E-11
UBE2D1	10q21.1	0.329193583	3.07E-10	2.48E-09	ZNF217	20q13.2	0.362850432	2.87E-12	2.54E-11
KPNA4	3q25.33	0.329185325	3.07E-10	2.48E-09	CLDND1	3q11.2	0.36280345	2.89E-12	2.56E-11
TMEM87B	2q13	0.329135899	3.09E-10	2.49E-09	ZNF627	19p13.2	0.362706069	2.93E-12	2.59E-11
NUFIP2	17q11.2	0.329082938	3.11E-10	2.51E-09	RHOJ	14q23.2	0.362540066	3.00E-12	2.65E-11
C11ORF95	11q13.1	0.329070979	3.12E-10	2.51E-09	CCDC93	2q14.1	0.362512731	3.02E-12	2.66E-11
KLLN	10q23.31	0.328960713	3.16E-10	2.55E-09	SUSD5	3p22.3	0.362498237	3.02E-12	2.67E-11
UBA2	19q13.11	0.328954236	3.17E-10	2.55E-09	FGD4	12p11.21	0.362413642	3.06E-12	2.70E-11
KITLG	12q21.32	0.328899732	3.19E-10	2.56E-09	IPPK	9q22.31	0.362341603	3.09E-12	2.73E-11
CUEDC1	17q22	0.328886753	3.19E-10	2.57E-09	EXD2	14q24.1	0.362279245	3.12E-12	2.75E-11
NAA15	4q31.1	0.328621091	3.31E-10	2.65E-09	ZNF701	19q13.41	0.362229234	3.14E-12	2.77E-11
VCPIP1	8q13.1	0.328599736	3.32E-10	2.66E-09	ZNF33A	10p11.1	0.362125654	3.19E-12	2.81E-11
ZNF132	19q13.43	0.328588797	3.32E-10	2.66E-09	BCL10	1p22.3	0.362048658	3.23E-12	2.84E-11
SHQ1	3p13	0.328578688	3.33E-10	2.67E-09	FAM155A	13q33.3	0.362036532	3.23E-12	2.84E-11
ZFAND3	6p21.2	0.328543642	3.34E-10	2.68E-09	SNORD116-20	15q11.2	0.362036481	3.23E-12	2.84E-11
SPICE1	3q13.2	0.328539086	3.34E-10	2.68E-09	ZNF514	2q11.1	0.361860847	3.32E-12	2.92E-11
NDFIP2	13q31.1	0.328459667	3.38E-10	2.70E-09	GJC1	17q21.31	0.36178411	3.36E-12	2.95E-11
HERC4	10q21.3	0.328455942	3.38E-10	2.70E-09	PODXL	7q32.3	0.361733685	3.38E-12	2.97E-11
ZNF45	19q13.31	0.328451387	3.38E-10	2.71E-09	MICAL2	11p15.3	0.361539778	3.48E-12	3.05E-11
AP1AR	4q25	0.328435157	3.39E-10	2.71E-09	VLDLR	9p24.2	0.361477962	3.51E-12	3.08E-11
ZNF107	7q11.21	0.328374388	3.42E-10	2.73E-09	PRR16	5q23.1	0.361456915	3.52E-12	3.09E-11
SOCS6	18q22.2	0.328247513	3.47E-10	2.77E-09	LINC00654	20p12.3	0.361405709	3.55E-12	3.11E-11
HERC3	4q22.1	0.328238971	3.48E-10	2.78E-09	TMEM51-AS1	1p36.21	0.361227659	3.64E-12	3.18E-11
AGFG1	2q36.3	0.328217947	3.49E-10	2.78E-09	USP32	17q23.1-q2	0.361163806	3.68E-12	3.21E-11
KIAA1549	7q34	0.327969608	3.60E-10	2.87E-09	MAP4K4	2q11.2	0.360941826	3.80E-12	3.31E-11
HBP1	7q22.3	0.327824961	3.67E-10	2.92E-09	PPP2R2A	8p21.2	0.360924457	3.81E-12	3.32E-11
MEF2A	15q26.3	0.327727295	3.72E-10	2.96E-09	ITPRIPL2	16p12.3	0.360918192	3.81E-12	3.32E-11
ZNF780B	19q13.2	0.327688286	3.73E-10	2.97E-09	SYT13	11p11.2	0.360894864	3.82E-12	3.33E-11
ZNF503	10q22.2	0.32756414	3.79E-10	3.02E-09	SNX18	5q11.2	0.360894274	3.82E-12	3.33E-11
SPRTN	1q42.2	0.327549618	3.80E-10	3.02E-09	ALG10B	12q12	0.360840174	3.85E-12	3.35E-11
SLIT2	4p15.31	0.327432548	3.86E-10	3.06E-09	SRBD1	2p21	0.360813408	3.87E-12	3.36E-11
LNPEP	5q15	0.327419515	3.87E-10	3.07E-09	BTBD3	20p12.2	0.360742793	3.91E-12	3.39E-11
RCOR3	1q32.2-q32.	0.32740599	3.87E-10	3.07E-09	CASP2	7q34	0.360742224	3.91E-12	3.39E-11
ATAD2B	2p24.1-p23.	0.327315562	3.92E-10	3.10E-09	PTPN4	2q14.2	0.360731688	3.92E-12	3.40E-11
MAPK6	15q21.2	0.327312596	3.92E-10	3.10E-09	EIF4G3	1p36.12	0.360575082	4.01E-12	3.47E-11
PDGFB	22q13.1	0.326997509	4.08E-10	3.23E-09	USP24	1p32.3	0.36051187	4.04E-12	3.50E-11
PELI1	2p14	0.326954228	4.11E-10	3.24E-09	PCDHGA5	5q31.3	0.360507174	4.05E-12	3.50E-11
TTC3	21q22.13	0.326952235	4.11E-10	3.24E-09	LOXL2	8p21.3	0.360455776	4.08E-12	3.53E-11
DSTYK	1q32.1	0.326949388	4.11E-10	3.24E-09	ARHGAP6	Xp22.2	0.360305887	4.17E-12	3.60E-11
RHOT1	17q11.2	0.326877064	4.15E-10	3.27E-09	LNPK	2q31.1	0.360264432	4.19E-12	3.62E-11
RBBP9	20p11.23	0.326840333	4.17E-10	3.28E-09	KRT80	12q13.13	0.360091143	4.30E-12	3.71E-11
CTR9	11p15.4	0.326838363	4.17E-10	3.28E-09	SCN7A	2q24.3	0.360033184	4.34E-12	3.74E-11
PTPRF	1p34.2	0.326579797	4.31E-10	3.39E-09	SNORD116-28	15q11.2	0.359905343	4.42E-12	3.81E-11
PRTG	15q21.3	0.326544628	4.33E-10	3.40E-09	SH3BP5	3p25.1	0.359802015	4.48E-12	3.86E-11
PPAT	4q12	0.326422621	4.40E-10	3.46E-09	DNAH7	2q32.3	0.359768149	4.51E-12	3.88E-11
RPS6KA5	14q32.11	0.326381522	4.42E-10	3.47E-09	MDM1	12q15	0.359684703	4.56E-12	3.92E-11
CPEB2	4p15.32	0.326377632	4.43E-10	3.47E-09	LINC01881	2q37.3	0.359667196	4.57E-12	3.93E-11
ZNF687	1q21.3	0.326124783	4.57E-10	3.58E-09	TOR1AIP1	1q25.2	0.359630486	4.60E-12	3.95E-11
MKLN1	7q32.3	0.325940842	4.68E-10	3.66E-09	OSBPL5	11p15.4	0.359609532	4.61E-12	3.96E-11
ZNF33B	10q11.21	0.325932869	4.69E-10	3.67E-09	INO80	15q15.1	0.359536638	4.66E-12	4.00E-11
NUP54	4q21.1	0.32587763	4.72E-10	3.69E-09	AFF1	4q21.3-q22	0.35952411	4.67E-12	4.01E-11
NT5C2	10q24.32-q2	0.325846309	4.74E-10	3.71E-09	ZDHHC1	16q22.1	0.35938174	4.77E-12	4.09E-11
TRAM2	6p12.2	0.325758775	4.79E-10	3.74E-09	CDC42BPA	1q42.13	0.35936608	4.78E-12	4.10E-11
DIAPH2	Xq21.33	0.325733552	4.81E-10	3.75E-09	ENTPD7	10q24.2	0.359328209	4.80E-12	4.12E-11
BAHCC1	17q25.3	0.325617948	4.88E-10	3.80E-09	MICALCL	11p15.3	0.359326034	4.81E-12	4.12E-11
SH3BP4	2q37.2	0.325436569	5.00E-10	3.89E-09	TPR	1q31.1	0.359283246	4.84E-12	4.14E-11
RANBP6	9p24.1	0.325309006	5.08E-10	3.94E-09	DSP	6p24.3	0.359207195	4.89E-12	4.18E-11
SLC25A36	3q23	0.32525519	5.11E-10	3.97E-09	SAMD9	7q21.2	0.359078778	4.98E-12	4.26E-11
ZBTB12	6p21.33	0.32523953	5.13E-10	3.97E-09	ABL2	1q25.2	0.359019267	5.02E-12	4.29E-11
DACH1	13q21.33	0.325186142	5.16E-10	4.00E-09	TTLL7	1p31.1	0.359001256	5.04E-12	4.30E-11
PCDHB11	5q31.3	0.325138408	5.19E-10	4.02E-09	ZNF322	6p22.2	0.358996229	5.04E-12	4.30E-11
SNAPC3	9p22.3	0.32509175	5.22E-10	4.05E-09	TAF1B	2p25.1	0.358939876	5.08E-12	4.34E-11
PYGO1	15q21.3	0.325048367	5.25E-10	4.07E-09	RCOR1	14q32.31-q	0.358795463	5.19E-12	4.42E-11
ZEB2	2q22.3	0.325021989	5.27E-10	4.08E-09	MTUS1	8p22	0.358647683	5.30E-12	4.51E-11
ROBO2	3p12.3	0.324965968	5.31E-10	4.10E-09	PCYT1B	Xp22.11	0.358583645	5.35E-12	4.56E-11
ZNF451	6p12.1	0.324961909	5.31E-10	4.10E-09	MED21	12p11.23	0.35855799	5.37E-12	4.57E-11
RELCH	18q21.33	0.32459004	5.57E-10	4.30E-09	NFIC	19p13.3	0.358446088	5.46E-12	4.64E-11

SEL1L	14q31.1	0.324431725	5.69E-10	4.38E-09	RUBCN	3q29	0.358442956	5.46E-12	4.64E-11
THUMPD1	16p12.3	0.324299891	5.78E-10	4.46E-09	FKBP15	9q32	0.358437571	5.47E-12	4.64E-11
PCDHGB4	5q31.3	0.324264008	5.81E-10	4.48E-09	VGLL3	3p12.1	0.35842006	5.48E-12	4.65E-11
CHD8	14q11.2	0.324215608	5.85E-10	4.50E-09	ZSCAN23	6p22.1	0.358403264	5.49E-12	4.66E-11
DOCK9	13q32.3	0.324203649	5.86E-10	4.51E-09	CAV1	7q31.2	0.358294606	5.58E-12	4.73E-11
MSL2	3q22.3	0.324087475	5.94E-10	4.57E-09	SLFN12	17q12	0.358276383	5.60E-12	4.74E-11
UBE3C	7q36.3	0.324013751	6.00E-10	4.61E-09	INCENP	11q12.3	0.358244208	5.62E-12	4.76E-11
BACE1	11q23.3	0.323965322	6.04E-10	4.63E-09	PRIMPOL	4q35.1	0.3581921	5.67E-12	4.79E-11
WIF1	12q14.3	0.323795331	6.17E-10	4.73E-09	ZNF107	7q11.21	0.358167211	5.69E-12	4.81E-11
FTO	16q12.2	0.323762018	6.20E-10	4.75E-09	FILIP1	6q14.1	0.358144742	5.70E-12	4.82E-11
ZFP64	20q13.2	0.323633032	6.30E-10	4.82E-09	RNF145	5q33.3	0.358039195	5.79E-12	4.89E-11
SMG1P2	16p11.2	0.323615947	6.31E-10	4.83E-09	TBC1D8	2q11.2	0.357840732	5.96E-12	5.02E-11
MYO10	5p15.1	0.323324659	6.55E-10	5.01E-09	DMXL1	5q23.1	0.357796598	6.00E-12	5.05E-11
DACT2	6q27	0.323247303	6.62E-10	5.05E-09	BRPF1	3p25.3	0.357713169	6.07E-12	5.11E-11
SUCLA2	13q14.2	0.323168907	6.68E-10	5.10E-09	TTLL5	14q24.3	0.357523818	6.24E-12	5.25E-11
UBAP2L	1q21.3	0.323015433	6.82E-10	5.20E-09	ASB3	2p16.2	0.357520569	6.24E-12	5.25E-11
ZC3H6	2q14.1	0.323009476	6.82E-10	5.20E-09	CCSER1	4q22.1	0.357518006	6.25E-12	5.25E-11
G2E3	14q12	0.322926617	6.89E-10	5.26E-09	NFXL1	4p12	0.357485093	6.28E-12	5.27E-11
RIN2	20p11.23	0.322906378	6.91E-10	5.27E-09	RNF6	13q12.13	0.35737433	6.38E-12	5.36E-11
MYO5C	15q21.2	0.322895273	6.92E-10	5.27E-09	DOK6	18q22.2	0.357317594	6.43E-12	5.40E-11
PRR5L	11p13-p12	0.322846582	6.97E-10	5.30E-09	RAB31	18p11.22	0.357224842	6.52E-12	5.47E-11
EFCAB6	22q13.2-q1	0.322719131	7.08E-10	5.39E-09	FSTL1	3q13.33	0.357095001	6.64E-12	5.57E-11
KREMEN1	22q12.1	0.322662641	7.13E-10	5.42E-09	NIBAN1	1q25.3	0.357015441	6.72E-12	5.63E-11
SCRN3	2q31.1	0.322529098	7.25E-10	5.51E-09	GALNT1	18q12.2	0.356772391	6.96E-12	5.82E-11
AP2B1	17q12	0.322439998	7.34E-10	5.57E-09	GPX8	5q11.2	0.3567405	6.99E-12	5.85E-11
TTC14	3q26.33	0.322437412	7.34E-10	5.57E-09	DNAH9	17p12	0.356668096	7.06E-12	5.91E-11
LANCL1	2q34	0.32238547	7.39E-10	5.61E-09	MSI2	17q22	0.356578367	7.15E-12	5.98E-11
ZNF225	19q13.31	0.322260589	7.51E-10	5.69E-09	SPIN4	Xq11.1	0.356519258	7.21E-12	6.02E-11
ZFP14	19q13.12	0.322247941	7.52E-10	5.70E-09	INSYN2B	5q35.1	0.35642635	7.31E-12	6.10E-11
LYST	1q42.3	0.322228698	7.54E-10	5.71E-09	KPNB1	17q21.32	0.356397959	7.34E-12	6.12E-11
TCERG1	5q32	0.321941681	7.82E-10	5.92E-09	ZNF121	19p13.2	0.35638657	7.35E-12	6.12E-11
SNX10	7p15.2	0.321837467	7.92E-10	6.00E-09	SCIN	7p21.3	0.356355125	7.39E-12	6.15E-11
PPIP5K2	5q21.1	0.32182323	7.94E-10	6.00E-09	CDC14C	7p12.3	0.356353492	7.39E-12	6.15E-11
MED28	4p15.32	0.321757171	8.00E-10	6.05E-09	GTF2E1	3q13.33	0.356351832	7.39E-12	6.15E-11
VPS8	3q27.2	0.321684562	8.08E-10	6.10E-09	AFDN	6q27	0.356333039	7.41E-12	6.16E-11
NBEAL2	3p21.31	0.321566965	8.20E-10	6.19E-09	TEK	9p21.2	0.356318517	7.43E-12	6.17E-11
LNPK	2q31.1	0.321563263	8.20E-10	6.19E-09	ZNF654	3p11.1	0.356283494	7.46E-12	6.20E-11
RPL23AP53	8p23.3	0.321527101	8.24E-10	6.22E-09	KIF3A	5q31.1	0.35627754	7.47E-12	6.20E-11
CASK	Xp11.4	0.321434276	8.34E-10	6.29E-09	ZSCAN20	1p35.1	0.356249351	7.50E-12	6.23E-11
TNFRSF21	6p12.3	0.321248626	8.54E-10	6.44E-09	STARD13	13q13.1-q1	0.356175009	7.58E-12	6.29E-11
FBXL14	12p13.33	0.321242077	8.54E-10	6.44E-09	BCL2L11	2q13	0.356029222	7.74E-12	6.42E-11
FGD6	12q22	0.32120677	8.58E-10	6.46E-09	PCNX2	1q42.2	0.355993345	7.78E-12	6.45E-11
EDRF1	10q26.2	0.321152669	8.64E-10	6.50E-09	CACNA1C	12p13.33	0.35587888	7.91E-12	6.55E-11
ITGA4	2q31.3	0.321098854	8.70E-10	6.55E-09	PLAU	10q22.2	0.355790896	8.01E-12	6.63E-11
URB2	1q42.13	0.3210644	8.74E-10	6.57E-09	ZNF17	19q13.43	0.355671305	8.15E-12	6.73E-11
CX3CR1	3p22.2	0.321038489	8.77E-10	6.59E-09	KMT5B	11q13.2	0.355662194	8.16E-12	6.74E-11
LOC652276	16p13.3	0.320941296	8.88E-10	6.67E-09	CHD1	5q15-q21.1	0.355644824	8.18E-12	6.75E-11
NEDD1	12q23.1	0.320912111	8.91E-10	6.69E-09	GJD3	17q21.2	0.35558446	8.25E-12	6.81E-11
SP6	17q21.32	0.320906085	8.92E-10	6.70E-09	FBXO34	14q22.3	0.355570223	8.27E-12	6.82E-11
RSRC1	3q25.32	0.32083547	9.00E-10	6.75E-09	MBTPS1	16q23.3-q2	0.355397671	8.48E-12	6.98E-11
ZFYVE26	14q24.1	0.320832623	9.00E-10	6.75E-09	FNDC1	6q25.3	0.355383531	8.49E-12	6.99E-11
ETS1	11q24.3	0.32080887	9.03E-10	6.77E-09	HFE	6p22.2	0.355362079	8.52E-12	7.01E-11
LRIG1	3p14.1	0.320793329	9.04E-10	6.78E-09	FSCN1	7p22.1	0.355357238	8.52E-12	7.01E-11
CHST3	10q22.1	0.320625902	9.24E-10	6.91E-09	TRPV3	17p13.2	0.355209927	8.71E-12	7.16E-11
PTPN14	1q32.3-q41	0.320601415	9.27E-10	6.93E-09	ASAH1	8p22	0.355204333	8.71E-12	7.16E-11
COBLL1	2q24.3	0.320587178	9.28E-10	6.94E-09	PIAS3	1q21.1	0.355157066	8.77E-12	7.21E-11
INIP	9q32	0.320578231	9.29E-10	6.94E-09	UST	6q25.1	0.355130753	8.81E-12	7.23E-11
JCAD	10p11.23	0.320575503	9.30E-10	6.94E-09	GAPVD1	9q33.3	0.355120335	8.82E-12	7.24E-11
ZNF461	19q13.12	0.3205513	9.33E-10	6.96E-09	MAP3K12	12q13.13	0.35510954	8.83E-12	7.25E-11
SLC37A3	7q34	0.320482678	9.41E-10	7.01E-09	PDPK1	16p13.3	0.355083604	8.87E-12	7.27E-11
CMTR2	16q22.2	0.320154374	9.81E-10	7.30E-09	TNFRSF21	6p12.3	0.355079617	8.87E-12	7.27E-11
SUSD1	9q31.3-q32	0.320118497	9.85E-10	7.32E-09	DNAH10	12q24.31	0.355000995	8.97E-12	7.35E-11
SGPP1	14q23.2	0.320094864	9.88E-10	7.34E-09	LOC283922	16q23.1	0.354982372	9.00E-12	7.36E-11
TMTC4	13q32.3	0.320088315	9.89E-10	7.34E-09	GAB1	4q31.21	0.354903959	9.10E-12	7.44E-11
TAF13	1p13.3	0.319941105	1.01E-09	7.47E-09	NEK11	3q22.1	0.354774946	9.27E-12	7.58E-11
FIGNL1	7p12.2	0.319813849	1.02E-09	7.59E-09	SNIP1	1p34.3	0.354736791	9.32E-12	7.62E-11
KRAS	12p12.1	0.319749356	1.03E-09	7.65E-09	IGSF10	3q25.1	0.354608806	9.49E-12	7.75E-11
SLC4A7	3p24.1	0.319743211	1.03E-09	7.65E-09	WNK1	12p13.33	0.35456865	9.54E-12	7.79E-11
TTC28	22q12.1	0.319658359	1.04E-09	7.73E-09	ZNF283	19q13.31	0.354528078	9.60E-12	7.83E-11
SCAF8	6q25.2	0.319590875	1.05E-09	7.79E-09	MCTS2P	20q11.21	0.354397763	9.78E-12	7.97E-11
PFKFB3	10p15.1	0.319422595	1.08E-09	7.95E-09	PCDHGC4	5q31.3	0.354142137	1.01E-11	8.26E-11
DEK	6p22.3	0.319401809	1.08E-09	7.96E-09	TMEM127	2q11.2	0.353998656	1.04E-11	8.43E-11
BMS1P1	10q11.22	0.319379599	1.08E-09	7.98E-09	SMC1A	Xp11.22	0.353942085	1.04E-11	8.49E-11
FAT4	4q28.1	0.319215396	1.10E-09	8.14E-09	NUMA1	11q13.4	0.35392557	1.05E-11	8.50E-11

PPRC1	10q24.32	0.319118209	1.12E-09	8.24E-09	SLC4A4	4q13.3	0.353915319	1.05E-11	8.51E-11
KPNB1	17q21.32	0.319080908	1.12E-09	8.28E-09	KIF21A	12q12	0.353835307	1.06E-11	8.61E-11
ZNF532	18q21.32	0.319057844	1.13E-09	8.30E-09	PABPC5	Xq21.31	0.353804782	1.06E-11	8.64E-11
VEPH1	3q25.31-q2	0.318869056	1.15E-09	8.48E-09	CSTF2T	10q21.1	0.35373992	1.07E-11	8.71E-11
TMEM52B	12p13.2	0.318853933	1.16E-09	8.50E-09	SSH2	17q11.2	0.353709738	1.08E-11	8.75E-11
CAP2	6p22.3	0.318844005	1.16E-09	8.50E-09	ZNF423	16q12.1	0.353705802	1.08E-11	8.75E-11
CCDC6	10q21.2	0.318792752	1.16E-09	8.55E-09	RAB21	12q21.1	0.353689546	1.08E-11	8.76E-11
EAF1	3p25.1	0.318737797	1.17E-09	8.61E-09	NCBP3	17p13.2	0.353575056	1.10E-11	8.90E-11
GPATCH2	1q41	0.318630451	1.19E-09	8.72E-09	NBPF9	1q21.2	0.353485246	1.11E-11	9.01E-11
TNFSF8	9q32-q33.1	0.318460058	1.21E-09	8.90E-09	SLC28A3	9q21.32-q2	0.353411063	1.13E-11	9.11E-11
WDFY3-AS2	4q21.23	0.318376138	1.23E-09	8.99E-09	FOXF1	16q24.1	0.353367959	1.13E-11	9.15E-11
DNMBP	10q24.2	0.318312704	1.24E-09	9.06E-09	PRICKLE1	12q12	0.353323941	1.14E-11	9.21E-11
INTS13	12p11.23	0.318224982	1.25E-09	9.16E-09	DCBLD2	3q12.1|3	0.3531992	1.16E-11	9.37E-11
RAG1	11p12	0.318162986	1.26E-09	9.23E-09	RAB11FIP5	2p13.2	0.353183255	1.16E-11	9.38E-11
RSPO2	8q23.1	0.318105157	1.27E-09	9.29E-09	ADAMTS4	1q23.3	0.353147663	1.17E-11	9.43E-11
SH3PXD2B	5q35.1	0.318060688	1.28E-09	9.33E-09	DAAM2	6p21.2	0.353079895	1.18E-11	9.52E-11
ENTPD4	8p21.3	0.318052715	1.28E-09	9.34E-09	FRMD6	14q22.1	0.353026079	1.19E-11	9.58E-11
RAB5B	12q13.2	0.318015864	1.28E-09	9.38E-09	BTBD7	14q32.12	0.353006742	1.19E-11	9.60E-11
INTS4P2	7q11.21	0.318001189	1.29E-09	9.39E-09	MEGF6	1p36.32	0.352927275	1.21E-11	9.71E-11
RASEF	9q21.32	0.31799846	1.29E-09	9.39E-09	ZBTB8A	1p35.1	0.352788607	1.23E-11	9.89E-11
SSPN	12p12.1	0.317971393	1.29E-09	9.41E-09	C8ORF37	8q22.1	0.35271496	1.24E-11	9.99E-11
USP3	15q22.31	0.317878455	1.31E-09	9.52E-09	STC1	8p21.2	0.35267585	1.25E-11	1.00E-10
DAAM2	6p21.2	0.31778506	1.32E-09	9.62E-09	PHACTR1	6p24.1	0.352527911	1.28E-11	1.03E-10
ATN1	12p13.31	0.317638157	1.35E-09	9.78E-09	CLTC	17q23.1	0.352470553	1.29E-11	1.03E-10
MAP3K21	1q42.2	0.317635857	1.35E-09	9.78E-09	PTPN11	12q24.13	0.352298001	1.32E-11	1.06E-10
MYLIP	6p22.3	0.317591175	1.35E-09	9.84E-09	WDR44	Xq24	0.352284903	1.32E-11	1.06E-10
SREK1	5q12.3	0.317576084	1.36E-09	9.85E-09	TBC1D12	10q23.33	0.352276361	1.32E-11	1.06E-10
GOLGA7B	10q24.2	0.317408617	1.38E-09	1.00E-08	ZNF510	9q22.33	0.352194926	1.34E-11	1.07E-10
MYOF	10q23.33	0.317402086	1.39E-09	1.00E-08	MAP1A	15q15.3	0.35219037	1.34E-11	1.07E-10
ARHGEF40	14q11.2	0.317372188	1.39E-09	1.01E-08	TNFRSF19	13q12.12	0.352160472	1.34E-11	1.08E-10
PPP1R3D	20q13.33	0.317355674	1.39E-09	1.01E-08	LTBP2	14q24.3	0.351972829	1.38E-11	1.10E-10
RAPGEF2	4q32.1	0.31727253	1.41E-09	1.02E-08	AHCYL1	1p13.3	0.351869469	1.40E-11	1.12E-10
CD46	1q32.2	0.317156071	1.43E-09	1.03E-08	RARG	12q13.13	0.35185751	1.40E-11	1.12E-10
VANGL1	1p13.1	0.317132723	1.43E-09	1.04E-08	SNRK	3p22.1	0.351642816	1.45E-11	1.15E-10
PCDHGC5	5q31.3	0.317113604	1.44E-09	1.04E-08	SGCB	4q12	0.351629718	1.45E-11	1.16E-10
TECTA	11q23.3	0.316952202	1.47E-09	1.06E-08	BRCA2	13q13.1	0.351589603	1.46E-11	1.16E-10
C8ORF37	8q22.1	0.316911001	1.47E-09	1.06E-08	HECTD4	12q24.13	0.351374023	1.50E-11	1.20E-10
DDX10	11q22.3	0.316880159	1.48E-09	1.07E-08	NLGN3	Xq13.1	0.351218886	1.54E-11	1.22E-10
ZNF180	19q13.31	0.31672628	1.51E-09	1.09E-08	GPR82	Xp11.4	0.351184702	1.54E-11	1.23E-10
NUMB	14q24.2-q2	0.316534771	1.54E-09	1.11E-08	PLEKHA2	8p11.22	0.35117015	1.55E-11	1.23E-10
DCUN1D1	3q26.33	0.316509144	1.55E-09	1.11E-08	MEX3C	18q21.2	0.351059386	1.57E-11	1.25E-10
CALU	7q32.1	0.316451057	1.56E-09	1.12E-08	MARVELD1	10q24.2	0.351047142	1.57E-11	1.25E-10
PARD6G	18q23	0.316382458	1.57E-09	1.13E-08	PBX1	1q23.3	0.351042871	1.58E-11	1.25E-10
ZNF436	1p36.12	0.316356809	1.58E-09	1.13E-08	IL7R	5p13.2	0.351001918	1.58E-11	1.26E-10
MED13	17q23.2	0.316194508	1.61E-09	1.16E-08	KRAS	12p12.1	0.350976267	1.59E-11	1.26E-10
RSRC2	12q24.31	0.31619199	1.61E-09	1.16E-08	PMEPA1	20q13.31	0.350946345	1.60E-11	1.27E-10
RBSN	3p25.1	0.316057713	1.64E-09	1.17E-08	SFRP4	7p14.1	0.3508581	1.62E-11	1.28E-10
OSBPL11	3q21.2	0.316009997	1.65E-09	1.18E-08	GPRC5A	12p13.1	0.350819293	1.63E-11	1.29E-10
LRRC34	3q26.2	0.315945076	1.66E-09	1.19E-08	FAM13C	10q21.1	0.350765555	1.64E-11	1.30E-10
EVI5	1p22.1	0.31590806	1.67E-09	1.19E-08	UHRF1BP1	6p21.31	0.350708587	1.65E-11	1.31E-10
FAM149B1	10q22.2	0.315849119	1.68E-09	1.20E-08	B3GALT5	21q22.2	0.35060551	1.68E-11	1.32E-10
SUZ12	17q11.2	0.315828618	1.69E-09	1.21E-08	PAXIP1-AS2	7q36.2	0.350589851	1.68E-11	1.32E-10
ANKRD49	11q21	0.315823208	1.69E-09	1.21E-08	HECW2	2q32.3	0.350360067	1.73E-11	1.37E-10
GRPR	Xp22.2	0.315762934	1.70E-09	1.21E-08	PACRGL	4p15.31	0.350350411	1.74E-11	1.37E-10
MID1	Xp22.2	0.315647808	1.73E-09	1.23E-08	ZNF132	19q13.43	0.350332472	1.74E-11	1.37E-10
THSD1	13q14.3	0.315631578	1.73E-09	1.23E-08	TRAF3IP1	2q37.3	0.350244345	1.76E-11	1.39E-10
PTK2	8q24.3	0.315627877	1.73E-09	1.23E-08	CHRFAM7A	15q13.2	0.350195911	1.78E-11	1.39E-10
AGAP1	2q37.2	0.315557261	1.74E-09	1.24E-08	DCLRE1B	1p13.2	0.350192096	1.78E-11	1.39E-10
RFX3	9p24.2	0.315490985	1.76E-09	1.25E-08	PPP2R3A	3q22.2-q22	0.350162173	1.78E-11	1.40E-10
SMAD1	4q31.21	0.315453047	1.77E-09	1.26E-08	LZTS1	8p21.3	0.350089565	1.80E-11	1.41E-10
USP44	12q22	0.31538597	1.78E-09	1.27E-08	GPR161	1q24.2	0.349995366	1.83E-11	1.43E-10
EML1	14q32.2	0.315359653	1.79E-09	1.27E-08	HGF	7q21.11	0.349986656	1.83E-11	1.43E-10
RGL1	1q25.3	0.31535709	1.79E-09	1.27E-08	MTCL1	18p11.22	0.349945202	1.84E-11	1.44E-10
GPM6B	Xp22.2	0.315212662	1.82E-09	1.29E-08	SLC41A1	1q32.1	0.349938368	1.84E-11	1.44E-10
RARB	3p24.2	0.315050141	1.86E-09	1.32E-08	ANXA2P2	9p13.3	0.349924985	1.84E-11	1.44E-10
NEK5	13q14.3	0.315003668	1.87E-09	1.33E-08	LPAR4	Xq21.1	0.349908969	1.85E-11	1.45E-10
JAK2	9p24.1	0.314999458	1.87E-09	1.33E-08	AHR	7p21.1	0.349755021	1.89E-11	1.48E-10
ZFYVE9	1p32.3	0.314834024	1.91E-09	1.35E-08	ZNF93	19p12	0.349718396	1.90E-11	1.48E-10
SYTL2	11q14.1	0.314824628	1.91E-09	1.35E-08	TSPYL5	8q22.1	0.349672707	1.91E-11	1.49E-10
GXYLT2	3p13	0.314696849	1.94E-09	1.37E-08	COL5A1	9q34.3	0.349639392	1.92E-11	1.50E-10
PWARSN	15q11.2	0.314399602	2.01E-09	1.42E-08	YTHDC2	5q22.2	0.349628287	1.92E-11	1.50E-10
XPO4	13q12.11	0.314185673	2.07E-09	1.46E-08	ZNF550	19q13.43	0.349558811	1.94E-11	1.51E-10
ATP13A3	3q29	0.314121322	2.08E-09	1.47E-08	ITGB8	7p21.1	0.349518471	1.95E-11	1.52E-10
WASHC5	8q24.13	0.314002586	2.12E-09	1.49E-08	ZNF234	19q13.31	0.349503287	1.96E-11	1.52E-10

FLT1	13q12.3	0.31399319	2.12E-09	1.49E-08	COL13A1	10q22.1	0.349499386	1.96E-11	1.52E-10
GPALPP1	13q14.12	0.313835444	2.16E-09	1.52E-08	LRRC4C	11p12	0.349420026	1.98E-11	1.54E-10
FOXRED2	22q12.3	0.313652664	2.21E-09	1.55E-08	PRR14L	22q12.2	0.349394801	1.99E-11	1.55E-10
TBC1D8B	Xq22.3	0.313597972	2.22E-09	1.56E-08	LRCH1	13q14.13-q	0.349349813	2.00E-11	1.56E-10
FAM160B1	10q25.3	0.313467277	2.26E-09	1.59E-08	GPM6B	Xp22.2	0.349341791	2.00E-11	1.56E-10
DDX46	5q31.1	0.313349679	2.29E-09	1.61E-08	WNT9A	1q42.13	0.349239415	2.03E-11	1.58E-10
SRRM2	16p13.3	0.31331608	2.30E-09	1.61E-08	ZNF224	19q13.31	0.349229083	2.03E-11	1.58E-10
DCBLD2	3q12.1|3	0.313009416	2.39E-09	1.67E-08	SLC6A20	3p21.31	0.349178711	2.05E-11	1.59E-10
DDHD2	8p11.23	0.312919154	2.42E-09	1.69E-08	MATN3	2p24.1	0.349136712	2.06E-11	1.60E-10
CNTF	11q12.1	0.312903103	2.42E-09	1.69E-08	SPARC	5q33.1	0.348917863	2.12E-11	1.65E-10
GOPC	6q22.1	0.312803572	2.45E-09	1.72E-08	FMNL2	2q23.3	0.348798558	2.16E-11	1.67E-10
LRRN4	20p12.3	0.312677734	2.49E-09	1.74E-08	MMGT1	Xq26.3	0.348783751	2.16E-11	1.68E-10
CTNNA1	5q31.2	0.312659472	2.50E-09	1.74E-08	RDX	11q22.3	0.348709744	2.19E-11	1.69E-10
PLCL1	2q33.1	0.312657194	2.50E-09	1.74E-08	SLC5A4	22q12.3	0.348610731	2.22E-11	1.72E-10
RLF	1p34.2	0.31265634	2.50E-09	1.74E-08	LAMA2	6q22.33	0.348607162	2.22E-11	1.72E-10
C1ORF109	1p34.3	0.312551271	2.53E-09	1.77E-08	GOLGA6L5P	15q25.2	0.348456513	2.27E-11	1.75E-10
ZNF207	17q11.2	0.311999731	2.71E-09	1.89E-08	SNURF	15q11.2	0.348421891	2.28E-11	1.76E-10
SRC	20q11.23	0.311885266	2.74E-09	1.91E-08	ZNF28	19q13.41	0.348230813	2.34E-11	1.81E-10
LRPPRC	2p21	0.311881849	2.75E-09	1.91E-08	OPA1	3q29	0.34809212	2.38E-11	1.84E-10
RPA1	17p13.3	0.31169022	2.81E-09	1.96E-08	CCDC160	Xq26.2	0.348070679	2.39E-11	1.85E-10
SULT4A1	22q13.31	0.311462398	2.89E-09	2.01E-08	GALNT7	4q34.1	0.348023213	2.41E-11	1.86E-10
IQGAP1	15q26.1	0.311379285	2.92E-09	2.03E-08	PIK3C3	18q12.3	0.348015692	2.41E-11	1.86E-10
BTN2A3P	6p22.2	0.311348248	2.93E-09	2.04E-08	TPH1	11p15.1	0.348013749	2.41E-11	1.86E-10
MED14	Xp11.4	0.311238219	2.97E-09	2.06E-08	NCOA3	20q13.12	0.347996163	2.42E-11	1.86E-10
ZDHHC21	9p22.3	0.311214136	2.98E-09	2.07E-08	ATP10D	4p12	0.347944625	2.43E-11	1.87E-10
PCNX1	14q24.2	0.311054113	3.04E-09	2.11E-08	PCDHB8	5q31.3	0.347817451	2.48E-11	1.91E-10
ZBTB43	9q33.3	0.310959864	3.07E-09	2.13E-08	ARHGAP1	11p11.2	0.347720535	2.51E-11	1.93E-10
FRMD4A	10p13	0.310935376	3.08E-09	2.14E-08	SLC12A2	5q23.3	0.347635114	2.54E-11	1.95E-10
CBX1	17q21.32	0.310910035	3.09E-09	2.14E-08	JHY	11q24.1	0.34745626	2.61E-11	2.00E-10
TAF4B	18q11.2	0.310733931	3.16E-09	2.19E-08	DYRK3	1q32.1	0.347378279	2.63E-11	2.02E-10
WDR43	2p23.2	0.310704168	3.17E-09	2.20E-08	LRRC41	1p34.1-p33	0.347368883	2.64E-11	2.02E-10
NAA40	11q13.1	0.310612198	3.21E-09	2.22E-08	TTF1	9q34.13	0.347322427	2.66E-11	2.03E-10
B4GALT5	20q13.13	0.31058771	3.22E-09	2.23E-08	CXCL6	4q13.3	0.347320691	2.66E-11	2.03E-10
RCN2	15q24.3	0.310584742	3.22E-09	2.23E-08	BNIP2	15q22.2	0.34728944	2.67E-11	2.04E-10
PRR11	17q22	0.31052995	3.24E-09	2.24E-08	NT5C1B	2p24.2	0.34728047	2.67E-11	2.04E-10
PAFAH1B1	17p13.3	0.310438222	3.28E-09	2.26E-08	AMPH	7p14.1	0.347185469	2.71E-11	2.07E-10
DVL3	3q27.1	0.310414873	3.29E-09	2.27E-08	HCN4	15q24.1	0.347181807	2.71E-11	2.07E-10
TUBGCP3	13q34	0.310405762	3.29E-09	2.27E-08	GPATCH2	1q41	0.34716444	2.71E-11	2.08E-10
PCDHGA2	5q31.3	0.310388544	3.30E-09	2.27E-08	FAM107B	10p13	0.347123438	2.73E-11	2.09E-10
IGF1R	15q26.3	0.310340272	3.32E-09	2.29E-08	ANKHD1-EIF4EB	5q31.3	0.34702805	2.77E-11	2.11E-10
HSPA13	21q11.2	0.310333153	3.32E-09	2.29E-08	ARMCX2	Xq22.1	0.346982492	2.78E-11	2.13E-10
CTBP1-DT	4p16.3	0.310276205	3.34E-09	2.30E-08	B3GNT5	3q27.1	0.346977651	2.79E-11	2.13E-10
HTR2B	2q37.1	0.31022723	3.36E-09	2.32E-08	ZNF92	7q11.21	0.346928391	2.81E-11	2.14E-10
PHYHIPL	10q21.1	0.310216126	3.37E-09	2.32E-08	ZCCHC4	4p15.2	0.346868621	2.83E-11	2.16E-10
ZBTB41	1q31.3	0.310175693	3.38E-09	2.33E-08	NACC2	9q34.3	0.346816774	2.85E-11	2.17E-10
ZNF254	19p12	0.310093972	3.42E-09	2.35E-08	SLC38A7	16q21	0.346805953	2.85E-11	2.17E-10
PCYT1B	Xp22.11	0.310081895	3.42E-09	2.35E-08	ADORA2B	17p12	0.346761942	2.87E-11	2.19E-10
NIPSNAP3B	9q31.1	0.310003141	3.45E-09	2.38E-08	CXADR	21q21.1	0.346703447	2.89E-11	2.20E-10
KANSL2	12q13.11	0.309980077	3.46E-09	2.38E-08	RCSD1	1q24.2	0.34662429	2.93E-11	2.23E-10
CNOT1	16q21	0.309955305	3.47E-09	2.39E-08	PDE1C	7p14.3	0.346600398	2.94E-11	2.23E-10
SF3A1	22q12.2	0.309945623	3.48E-09	2.39E-08	AMMECR1L	2q14.3	0.346552536	2.96E-11	2.25E-10
DSC2	18q12.1	0.309759689	3.56E-09	2.44E-08	CUL2	10p11.21	0.346429244	3.01E-11	2.28E-10
SLCO1B7	12p12.2	0.309758927	3.56E-09	2.44E-08	SEC31A	4q21.22	0.346413583	3.01E-11	2.29E-10
GLYR1	16p13.3	0.309721534	3.57E-09	2.45E-08	GNG12	1p31.3	0.346288867	3.07E-11	2.33E-10
SRCAP	16p11.2	0.309691516	3.59E-09	2.46E-08	CASS4	20q13.31	0.34601704	3.18E-11	2.41E-10
JKAMP	14q23.1	0.309688219	3.59E-09	2.46E-08	SHOC2	10q25.2	0.345967279	3.21E-11	2.43E-10
SLC34A2	4p15.2	0.309630924	3.61E-09	2.47E-08	DZIP3	3q13.13	0.345774059	3.29E-11	2.49E-10
SLC16A12	10q23.31	0.309559883	3.65E-09	2.49E-08	CCSER2	10q23.1	0.345569332	3.39E-11	2.56E-10
ZNF227	19q13.31	0.309472694	3.68E-09	2.52E-08	RXFP1	4q32.1	0.345525421	3.41E-11	2.58E-10
ALMS1	2p13.1	0.309346532	3.74E-09	2.55E-08	COL16A1	1p35.2	0.34544824	3.45E-11	2.60E-10
TTPA	8q12.3	0.309285249	3.77E-09	2.57E-08	BTN2A1	6p22.2	0.345441484	3.45E-11	2.60E-10
BCL9	1q21.2	0.309188502	3.81E-09	2.60E-08	LINC01559	12p13.1	0.345419665	3.46E-11	2.61E-10
ROBO1	3p12.3	0.309015665	3.89E-09	2.65E-08	EFNB3	17p13.1	0.345399555	3.47E-11	2.62E-10
ABCC10	6p21.1	0.308974094	3.91E-09	2.66E-08	ZFP92	Xq28	0.345313621	3.51E-11	2.65E-10
FAM133B	7q21.2	0.308906632	3.95E-09	2.68E-08	OLR1	12p13.2	0.345306769	3.51E-11	2.65E-10
GNAQ	9q21.2	0.308860767	3.97E-09	2.70E-08	MYADM	19q13.42	0.345260959	3.54E-11	2.66E-10
PCDHB18P	5q31.3	0.308851816	3.97E-09	2.70E-08	GEMIN5	5q33.2	0.345251278	3.54E-11	2.67E-10
ZMYM1	1p34.3	0.308849115	3.97E-09	2.70E-08	LOC100126784	11p15.1	0.345203874	3.56E-11	2.68E-10
SMYD4	17p13.3	0.308820334	3.99E-09	2.71E-08	RBM7	11q23.2	0.345199455	3.57E-11	2.68E-10
C1GALT1	7p22.1-p21.	0.308812077	3.99E-09	2.71E-08	DKK2	4q25	0.345184407	3.57E-11	2.69E-10
SNX13	7p21.1	0.308727225	4.03E-09	2.74E-08	FUT10	8p12	0.345105658	3.61E-11	2.72E-10
HHAT	1q32.2	0.308625573	4.08E-09	2.77E-08	CCDC144B	17p11.2	0.344971563	3.68E-11	2.76E-10
RAB2B	14q11.2	0.308566512	4.11E-09	2.78E-08	EXOC2	6p25.3	0.344969956	3.68E-11	2.76E-10
ZNF583	19q13.43	0.308471244	4.16E-09	2.82E-08	MYOCD	17p12	0.34494829	3.69E-11	2.77E-10

KDM5C	Xp11.22	0.308461278	4.17E-09	2.82E-08	IQCK	16p12.3	0.344919272	3.71E-11	2.78E-10
MBTPS1	16q23.3-q24	0.308405754	4.19E-09	2.84E-08	ZNF646	16p11.2	0.344889659	3.72E-11	2.79E-10
ZNF329	19q13.43	0.308362189	4.22E-09	2.85E-08	FBXO28	1q42.11	0.344841823	3.75E-11	2.81E-10
MAP2K4	17p12	0.308168567	4.32E-09	2.91E-08	GIT2	12q24.11	0.344774365	3.78E-11	2.83E-10
ZNF395	8p21.1	0.308164865	4.32E-09	2.91E-08	CCN1	1p22.3	0.344756117	3.79E-11	2.84E-10
YEATS4	12q15	0.308124169	4.34E-09	2.93E-08	TMEM185B	2q14.2	0.344704579	3.82E-11	2.86E-10
VEZT	12q22	0.308005718	4.40E-09	2.97E-08	KIF26B	1q44	0.344604016	3.87E-11	2.89E-10
COL6A3	2q37.3	0.307849089	4.48E-09	3.02E-08	TLL1	4q32.3	0.34453814	3.91E-11	2.92E-10
PGM5P2	9p11.2	0.307803676	4.51E-09	3.04E-08	USP28	11q23.2	0.344508678	3.92E-11	2.93E-10
ZSCAN12	6p22.1	0.307765946	4.53E-09	3.05E-08	SEPTIN8	5q31.1	0.34445771	3.95E-11	2.95E-10
ARMCX5	Xq22.1	0.307748861	4.54E-09	3.06E-08	ZNF318	6p21.1	0.34440304	3.98E-11	2.97E-10
RICTOR	5p13.1	0.307578916	4.63E-09	3.12E-08	KCNJ16	17q24.3	0.344340274	4.02E-11	2.99E-10
HECW1	7p14.1-p13	0.307522755	4.67E-09	3.14E-08	GPR75	2p16.2	0.344241935	4.07E-11	3.03E-10
KLHL8	4q22.1	0.307395785	4.74E-09	3.18E-08	OXR1	8q23.1	0.344217106	4.08E-11	3.04E-10
LGALS8	1q43	0.307381833	4.75E-09	3.19E-08	LRRC8C	1p22.2	0.344100932	4.15E-11	3.09E-10
KLHDC10	7q32.2	0.307281035	4.80E-09	3.22E-08	ZBTB41	1q31.3	0.34395543	4.23E-11	3.15E-10
ARL13B	3q11.1-q11.	0.307275625	4.81E-09	3.22E-08	DCLK1	13q13.3	0.343943419	4.24E-11	3.15E-10
APOOL	Xq21.1	0.307234053	4.83E-09	3.24E-08	COL1A2	7q21.3	0.343905886	4.26E-11	3.17E-10
IPPK	9q22.31	0.307185932	4.86E-09	3.25E-08	SCG2	2q36.1	0.343854616	4.29E-11	3.19E-10
MYD88	3p22.2	0.30716657	4.87E-09	3.26E-08	MAP2K1	15q22.31	0.343819325	4.31E-11	3.20E-10
1-Mar	4q32.2-q32.	0.30710557	4.91E-09	3.28E-08	KLF8	Xp11.21	0.343794008	4.33E-11	3.21E-10
SNRNP48	6p24.3	0.307023346	4.95E-09	3.31E-08	SERINC3	20q13.12	0.343752981	4.35E-11	3.23E-10
ADGRB3	6q12-q13	0.306989513	4.97E-09	3.32E-08	PLEKHH2	2p21	0.343735881	4.36E-11	3.24E-10
PCMTD1	8q11.23	0.306974656	4.98E-09	3.33E-08	LGI2	4p15.2	0.343674678	4.40E-11	3.26E-10
MAP4	3p21.31	0.306946204	5.00E-09	3.34E-08	NAA35	9q21.33	0.343576158	4.46E-11	3.30E-10
NOL8	9q22.31	0.306752844	5.12E-09	3.41E-08	UBQLN2	Xp11.21	0.343559952	4.47E-11	3.31E-10
NOTCH1	9q34.3	0.306640941	5.19E-09	3.46E-08	FBXL19-AS1	16p11.2	0.343512661	4.50E-11	3.33E-10
GMFB	14q22.2	0.306586436	5.22E-09	3.48E-08	KDM5B	1q32.1	0.343498424	4.51E-11	3.33E-10
CMTM1	16q21	0.306547547	5.25E-09	3.50E-08	TNFAIP3	6q23.3	0.343464255	4.53E-11	3.35E-10
RNF144A	2p25.1	0.306232626	5.45E-09	3.62E-08	ARCN1	11q23.3	0.343449331	4.54E-11	3.35E-10
IARS2	1q41	0.306159163	5.50E-09	3.65E-08	MDGA1	6p21.2	0.34343424	4.55E-11	3.36E-10
SYNJ2	6q25.3	0.306048399	5.57E-09	3.70E-08	IKZF4	12q13.2	0.343371146	4.59E-11	3.39E-10
CASTOR2	7q11.23	0.306038321	5.58E-09	3.70E-08	SETBP1	18q12.3	0.343315622	4.62E-11	3.41E-10
ZNF280B	22q11.22	0.305983935	5.61E-09	3.73E-08	FUT4	11q21	0.343291704	4.64E-11	3.42E-10
BEND7	10p13	0.305978923	5.62E-09	3.73E-08	STK17B	2q32.3	0.343272366	4.65E-11	3.43E-10
FBXW2	9q33.2	0.305978638	5.62E-09	3.73E-08	ZNF280D	15q21.3	0.343268355	4.65E-11	3.43E-10
TSSK6	19p13.11	0.305973907	5.62E-09	3.73E-08	LBH	2p23.1	0.343070746	4.78E-11	3.52E-10
AP3M2	8p11.21	0.305866736	5.69E-09	3.77E-08	SH3TC2	5q32	0.342829739	4.94E-11	3.63E-10
SH3GLB1	1p22.3	0.305842248	5.71E-09	3.78E-08	NUP155	5p13.2	0.342774048	4.98E-11	3.65E-10
DKK3	11p15.3	0.30577448	5.76E-09	3.81E-08	CDON	11q24.2	0.3427712	4.98E-11	3.65E-10
ZNF610	19q13.41	0.305623808	5.86E-09	3.87E-08	SART3	12q23.3	0.342718833	5.02E-11	3.68E-10
KPNA3	13q14.2	0.305619298	5.86E-09	3.87E-08	GTF3C2	2p23.3	0.342612601	5.09E-11	3.73E-10
SNAP23	15q15.1-q15	0.305584012	5.89E-09	3.89E-08	NUP58	13q12.13	0.342568181	5.12E-11	3.75E-10
PNMA1	14q24.3	0.305510527	5.94E-09	3.92E-08	HNRNPUL1	19q13.2	0.342566473	5.12E-11	3.75E-10
MMP16	8q21.3	0.305485508	5.96E-09	3.93E-08	ALOX5	10q11.21	0.342522908	5.16E-11	3.77E-10
IWS1	2q14.3	0.305484331	5.96E-09	3.93E-08	PFKP	10p15.2	0.34251738	5.16E-11	3.77E-10
RPAP1	15q15.1	0.305478921	5.96E-09	3.93E-08	TMEM106B	7p21.3	0.342503855	5.17E-11	3.78E-10
ZNF343	20p13	0.305441905	5.99E-09	3.95E-08	LRRC37A6P	10p12.1	0.342499966	5.17E-11	3.78E-10
ZNF260	19q13.12	0.305441336	5.99E-09	3.95E-08	KCNQ4	1p34.2	0.342474234	5.19E-11	3.79E-10
FAM13C	10q21.1	0.305319943	6.08E-09	4.00E-08	PLCH1	3q25.31	0.342460536	5.20E-11	3.80E-10
ATP9A	20q13.2	0.305288146	6.10E-09	4.02E-08	TMEM173	5q31.2	0.342457703	5.20E-11	3.80E-10
GOLGA6L10	15q25.2	0.305278465	6.11E-09	4.02E-08	XCR1	3p21.31	0.342322883	5.30E-11	3.86E-10
TM9SF2	13q32.3	0.305140366	6.21E-09	4.08E-08	MKLN1	7q32.3	0.342118578	5.45E-11	3.97E-10
NBPF1	1p36.13	0.305036152	6.29E-09	4.13E-08	GOLGA4	3p22.2	0.342026323	5.52E-11	4.02E-10
PIEZO2	18p11.22-p1	0.305015081	6.30E-09	4.14E-08	OSR1	2p24.1	0.341972307	5.56E-11	4.04E-10
FBXW11	5q35.1	0.304986323	6.33E-09	4.15E-08	PHF20L1	8q24.22	0.341929227	5.59E-11	4.07E-10
KLHL4	Xq21.31	0.304965294	6.34E-09	4.16E-08	CDC42SE1	1q21.3	0.341841243	5.66E-11	4.11E-10
LRRC36	16q22.1	0.304847731	6.43E-09	4.22E-08	FZD6	8q22.3	0.341825061	5.67E-11	4.12E-10
SLC12A1	15q21.1	0.304802399	6.47E-09	4.24E-08	SUGP2	19p13.11	0.341819602	5.68E-11	4.12E-10
UGGT1	2q14.3	0.304758816	6.50E-09	4.26E-08	PRDM5	4q27	0.341764563	5.72E-11	4.15E-10
CBFB	16q22.1	0.304735183	6.52E-09	4.27E-08	GLI2	2q14.2	0.341685239	5.78E-11	4.19E-10
WRN	8p12	0.304699021	6.55E-09	4.28E-08	ATP8A2	13q12.13	0.341568227	5.87E-11	4.26E-10
HNRNPA1L2	13q14.3	0.304684214	6.56E-09	4.29E-08	FAM171A1	10p13	0.341509237	5.92E-11	4.29E-10
CNOT8	5q33.2	0.304623017	6.61E-09	4.32E-08	PALD1	10q22.1	0.341475922	5.95E-11	4.30E-10
WBP11	12p12.3	0.304622995	6.61E-09	4.32E-08	TEX9	15q21.3	0.341405819	6.01E-11	4.34E-10
INPP4A	2q11.2	0.304570603	6.65E-09	4.34E-08	ZFP90	16q22.1	0.341357756	6.05E-11	4.37E-10
GRIP1	12q14.3	0.30451857	6.69E-09	4.37E-08	CLEC5A	7q34	0.341301668	6.09E-11	4.40E-10
WNK1	12p13.33	0.304415674	6.77E-09	4.42E-08	NUMBL	19q13.2	0.341074322	6.28E-11	4.53E-10
CENPC	4q13.2	0.304412858	6.77E-09	4.42E-08	TNC	9q33.1	0.340914132	6.42E-11	4.63E-10
PTCD3	2p11.2	0.304382106	6.80E-09	4.43E-08	FUT8	14q23.3	0.340777742	6.54E-11	4.71E-10
YWHAG	7q11.23	0.304347938	6.83E-09	4.45E-08	SOGA3	6q22.33	0.340753998	6.56E-11	4.73E-10
DET1	15q26.1	0.304086163	7.04E-09	4.59E-08	ARID4A	14q23.1	0.340744143	6.57E-11	4.73E-10
MMS19	10q24.1	0.304047538	7.07E-09	4.61E-08	DLG4	17p13.1	0.340737309	6.58E-11	4.73E-10
CAPZA2	7q31.2	0.303980624	7.13E-09	4.64E-08	PLXNA1	3q21.3	0.34067125	6.64E-11	4.77E-10

IPO8	12p11.21	0.303917455	7.18E-09	4.67E-08	OTUD1	10p12.2	0.340618858	6.69E-11	4.80E-10
GPR107	9q34.11	0.303890077	7.21E-09	4.68E-08	PDP1	8q22.1	0.340528026	6.77E-11	4.86E-10
MED12L	3q25.1	0.30380468	7.28E-09	4.73E-08	VCPIP1	8q13.1	0.340510372	6.79E-11	4.87E-10
COL12A1	6q13-q14.1	0.303759403	7.32E-09	4.75E-08	CCDC102B	18q22.1-q2	0.340377708	6.91E-11	4.96E-10
UBLCP1	5q33.3	0.303740205	7.34E-09	4.76E-08	NCOA7	6q22.31-q2	0.340345508	6.94E-11	4.98E-10
MPP5	14q23.3	0.303698448	7.37E-09	4.78E-08	BVES	6q21	0.340047318	7.23E-11	5.18E-10
CNTNAP2	7q35-q36.1	0.303686212	7.38E-09	4.79E-08	FAM117B	2q33.2	0.339956603	7.32E-11	5.24E-10
KLHL7	7p15.3	0.303579591	7.48E-09	4.84E-08	SPATA6	1p33	0.339893674	7.38E-11	5.28E-10
FAM169A	5q13.3	0.303529882	7.52E-09	4.87E-08	TRERF1	6p21.1	0.339856896	7.42E-11	5.31E-10
HEPN1	11q24.2	0.303470493	7.58E-09	4.90E-08	SEMA6D	15q21.1	0.339833571	7.44E-11	5.32E-10
SMYD2	1q32.3	0.303231191	7.80E-09	5.04E-08	ALDH1A3	15q26.3	0.339718443	7.56E-11	5.40E-10
KCNJ5	11q24.3	0.303166577	7.85E-09	5.07E-08	TNRC6A	16p12.1	0.339589811	7.69E-11	5.49E-10
UNC5C	4q22.3	0.303107707	7.91E-09	5.10E-08	ACTN1	14q24.1|14c	0.339588672	7.69E-11	5.49E-10
KLF10	8q22.3	0.303081418	7.93E-09	5.12E-08	OSBPL10	3p23	0.33951808	7.77E-11	5.54E-10
ZNF701	19q13.41	0.30293344	8.08E-09	5.21E-08	NEK4	3p21.1	0.339483033	7.80E-11	5.57E-10
TTC17	11p12-p11.2	0.302863308	8.14E-09	5.25E-08	ITPRIP	10q25.1	0.339444878	7.84E-11	5.59E-10
CNOT7	8p22	0.302737738	8.26E-09	5.32E-08	U2SURP	3q23	0.339369874	7.92E-11	5.65E-10
TEAD3	6p21.31	0.302726064	8.28E-09	5.33E-08	CNTN4	3p26.3-p26	0.339327523	7.97E-11	5.68E-10
INHBA	7p14.1	0.302674405	8.33E-09	5.36E-08	IL17RD	3p14.3	0.33922786	8.08E-11	5.75E-10
NR2F1-AS1	5q15	0.30264292	8.36E-09	5.38E-08	ORC2	2q33.1	0.339188898	8.12E-11	5.78E-10
TBX20	7p14.2	0.302625826	8.37E-09	5.39E-08	DLGAP1	18p11.31	0.339174018	8.14E-11	5.79E-10
BBS5	2q31.1	0.302513364	8.49E-09	5.45E-08	PLAGL1	6q24.2	0.339167258	8.14E-11	5.79E-10
FICD	12q23.3	0.302486313	8.51E-09	5.47E-08	ZMYND8	20q13.12	0.339148465	8.16E-11	5.80E-10
HIST2H2BA	1p11.2	0.302396023	8.61E-09	5.52E-08	KCNJ2	17q24.3	0.33904425	8.28E-11	5.88E-10
HNRNPH2	Xq22.1	0.302385537	8.62E-09	5.53E-08	JARID2	6p22.3	0.33901236	8.32E-11	5.90E-10
IMPACT	18q11.2	0.302340812	8.66E-09	5.56E-08	CORO1C	12q24.11	0.33891925	8.42E-11	5.98E-10
FBXL4	6q16.1-q16.	0.302309206	8.69E-09	5.58E-08	MCM9	6q22.31	0.338910423	8.43E-11	5.98E-10
TLL1	4q32.3	0.30222906	8.78E-09	5.63E-08	KLHL7	7p15.3	0.338878984	8.47E-11	6.00E-10
CEP350	1q25.2	0.301962963	9.06E-09	5.80E-08	SMAD5	5q31.1	0.338856632	8.49E-11	6.02E-10
NUP133	1q42.13	0.301922124	9.10E-09	5.83E-08	NPY5R	4q32.2	0.338798694	8.56E-11	6.06E-10
PDP2	16q22.1	0.301806356	9.23E-09	5.90E-08	EVC	4p16.2	0.338790548	8.57E-11	6.07E-10
RASL10B	17q12	0.301801231	9.23E-09	5.91E-08	KIAA0556	16p12.1	0.338785707	8.58E-11	6.07E-10
ZNF558	19p13.2	0.301792689	9.24E-09	5.91E-08	GABRR2	6q15	0.338754591	8.61E-11	6.09E-10
TAS2R5	7q34	0.301785438	9.25E-09	5.91E-08	SGCD	5q33.2-q33	0.338518616	8.89E-11	6.28E-10
ZNF391	6p22.1	0.30169223	9.35E-09	5.98E-08	ACE	17q23.3	0.338513212	8.90E-11	6.29E-10
GTF2IP1	7q11.23	0.301528451	9.53E-09	6.09E-08	RANBP6	9p24.1	0.338479328	8.94E-11	6.31E-10
USP14	18p11.32	0.301453565	9.62E-09	6.14E-08	WDR19	4p14	0.338361161	9.08E-11	6.41E-10
ATP2B1	12q21.33	0.301407722	9.67E-09	6.17E-08	PPP1R9B	17q21.33	0.33829197	9.17E-11	6.46E-10
SH3YL1	2p25.3	0.301308063	9.78E-09	6.24E-08	PRDM4	12q23.3	0.338182915	9.30E-11	6.56E-10
EFNB2	13q33.3	0.301159429	9.96E-09	6.35E-08	CHD2	15q26.1	0.338135363	9.36E-11	6.60E-10
PCDHB15	5q31.3	0.301113752	1.00E-08	6.38E-08	ZNF737	19p12	0.338075568	9.44E-11	6.65E-10
PGM2L1	11q13.4	0.301095648	1.00E-08	6.39E-08	NIPA1	15q11.2	0.337985615	9.55E-11	6.72E-10
NRP2	2q33.3	0.301009656	1.01E-08	6.45E-08	SLC10A6	4q21.3	0.337966684	9.58E-11	6.74E-10
VPS13A	9q21.2	0.3009342	1.02E-08	6.51E-08	XIAP	Xq25	0.337935192	9.62E-11	6.77E-10
TCTN2	12q24.31	0.300910282	1.03E-08	6.52E-08	FBXO3	11p13	0.3378492	9.73E-11	6.84E-10
CASC15	6p22.3	0.300798714	1.04E-08	6.60E-08	ZNF468	19q13.41	0.337815886	9.77E-11	6.87E-10
PDGFD	11q22.3	0.300710396	1.05E-08	6.67E-08	DNM1L	12p11.21	0.337803239	9.79E-11	6.88E-10
SEC23B	20p11.23	0.300647753	1.06E-08	6.71E-08	RAPGEF2	4q32.1	0.337690886	9.94E-11	6.97E-10
ING3	7q31.31	0.300637666	1.06E-08	6.72E-08	CCDC144A	17p11.2	0.337676019	9.96E-11	6.98E-10
ARHGEF3	3p14.3	0.300624974	1.06E-08	6.73E-08	DGKI	7q33	0.337624412	1.00E-10	7.03E-10
NLRX1	11q23.3	0.300604209	1.06E-08	6.74E-08	JCAD	10p11.23	0.337603755	1.01E-10	7.04E-10
ANKRD11	16q24.3	0.30058741	1.07E-08	6.75E-08	TENM4	11q14.1	0.337545469	1.01E-10	7.09E-10
SNORD1C	17q25.1	0.300492405	1.08E-08	6.82E-08	RGS5	1q23.3	0.33750552	1.02E-10	7.13E-10
NKTR	3p22.1	0.300439203	1.08E-08	6.86E-08	RNF217	6q22.31	0.337459204	1.03E-10	7.17E-10
STAG3L1	7q11.23	0.300339544	1.10E-08	6.94E-08	CGAS	6q13	0.337249136	1.05E-10	7.36E-10
HEATR5A	14q12	0.300330838	1.10E-08	6.94E-08	DCAF7	17q23.3	0.337197314	1.06E-10	7.41E-10
HDGFL3	15q25.2	0.300226054	1.11E-08	7.03E-08	HRH1	3p25.3	0.337149763	1.07E-10	7.45E-10
PTPN23	3p21.31	0.300224346	1.11E-08	7.03E-08	ERCC3	2q14.3	0.337145325	1.07E-10	7.45E-10
PDE10A	6q27	0.300217391	1.11E-08	7.03E-08	LSG1	3q29	0.337062775	1.08E-10	7.53E-10
EDNRA	4q31.22-q3	0.300086981	1.13E-08	7.14E-08	RREB1	6p24.3	0.33680193	1.12E-10	7.79E-10
ZNF615	19q13.41	0.299709253	1.18E-08	7.45E-08	FBXW2	9q33.2	0.336782568	1.12E-10	7.81E-10
AFAP1L2	10q25.3	0.299704982	1.18E-08	7.45E-08	ZDBF2	2q33.3	0.336763017	1.13E-10	7.82E-10
USP8	15q21.2	0.299609473	1.19E-08	7.53E-08	CHST9	18q11.2	0.336658865	1.14E-10	7.93E-10
RPGR	Xp11.4	0.299605038	1.20E-08	7.53E-08	BCL2	18q21.33	0.336626246	1.15E-10	7.96E-10
HOOK3	8p11.21	0.299436473	1.22E-08	7.68E-08	ATP1A2	1q23.2	0.33650123	1.17E-10	8.09E-10
GLUL	1q25.3	0.299403158	1.22E-08	7.70E-08	DCHS1	11p15.4	0.336458559	1.17E-10	8.13E-10
SNORA71E	20q11.23	0.299381025	1.23E-08	7.72E-08	SAP130	2q14.3	0.336416393	1.18E-10	8.17E-10
SENP6	6q14.1	0.299276044	1.24E-08	7.81E-08	PDCD10	3q26.1	0.336243841	1.21E-10	8.36E-10
STRN3	14q12	0.299245413	1.25E-08	7.84E-08	CSRNP3	2q24.3	0.336239868	1.21E-10	8.36E-10
MED21	12p11.23	0.299239149	1.25E-08	7.84E-08	ANXA2P1	4q31.3	0.336056198	1.24E-10	8.56E-10
ATP2A2	12q24.11	0.299223773	1.25E-08	7.85E-08	NXNL2	9q22.1	0.336056017	1.24E-10	8.56E-10
NAA35	9q21.33	0.299183909	1.26E-08	7.89E-08	FOXF2	6p25.3	0.336055101	1.24E-10	8.56E-10
EXT2	11p11.2	0.2991617	1.26E-08	7.90E-08	BRD1	22q13.33	0.336045948	1.24E-10	8.57E-10
TOPBP1	3q22.1	0.299071437	1.27E-08	7.99E-08	TGFB3	14q24.3	0.336030287	1.24E-10	8.58E-10

BLOC1S5	6p24.3	0.298987155	1.28E-08	8.06E-08	PCDH7	4p15.1	0.335994111	1.25E-10	8.62E-10
GABPB1	15q21.2	0.298984592	1.28E-08	8.06E-08	ZSCAN30	18q12.2	0.335973078	1.25E-10	8.64E-10
ZNF25	10p11.21	0.298943874	1.29E-08	8.10E-08	PLEKHG1	6q25.1	0.335957679	1.25E-10	8.66E-10
UBXN2A	2p23.3	0.29889964	1.30E-08	8.13E-08	PTPN14	1q32.3-q41	0.335866847	1.27E-10	8.76E-10
STXBP4	17q22	0.298781573	1.32E-08	8.25E-08	FPR3	19q13.41	0.335782849	1.28E-10	8.85E-10
ZNF720	16p11.2	0.298576276	1.35E-08	8.44E-08	ABR	17p13.3	0.33573245	1.29E-10	8.90E-10
ADCY6	12q13.12	0.298545403	1.35E-08	8.47E-08	LOXL3	2p13.1	0.335572996	1.32E-10	9.09E-10
NCL	2q37.1	0.298386925	1.38E-08	8.62E-08	CCDC6	10q21.2	0.335489852	1.34E-10	9.19E-10
CHUK	10q24.31	0.298377243	1.38E-08	8.62E-08	HCG11	6p22.2	0.335470395	1.34E-10	9.21E-10
TMEM109	11q12.2	0.298331564	1.39E-08	8.66E-08	RESF1	12p11.21	0.335456253	1.34E-10	9.22E-10
BRMS1L	14q13.2	0.298303375	1.39E-08	8.69E-08	MEDAG	13q12.3	0.335429192	1.35E-10	9.25E-10
SETDB2	13q14.2	0.298281002	1.39E-08	8.71E-08	C1QTNF7	4p15.32	0.335371805	1.36E-10	9.31E-10
CCSER2	10q23.1	0.298278439	1.40E-08	8.71E-08	CPSF6	12q15	0.335201981	1.39E-10	9.52E-10
PPP3CB	10q22.2	0.298251673	1.40E-08	8.73E-08	RBM25	14q24.2	0.335196737	1.39E-10	9.52E-10
NXF1	11q12.3	0.298100762	1.42E-08	8.88E-08	AP3M2	8p11.21	0.335156423	1.40E-10	9.57E-10
CASP2	7q34	0.298097914	1.42E-08	8.88E-08	PTPRK	6q22.33	0.335076981	1.41E-10	9.66E-10
GPATCH11	2p22.2	0.298082538	1.43E-08	8.90E-08	TMX3	18q22.1	0.335039395	1.42E-10	9.70E-10
RASAL2-AS1	1q25.2	0.298046946	1.43E-08	8.93E-08	RHOQ	2p21	0.335031138	1.42E-10	9.71E-10
CAAP1	9p21.2	0.298024452	1.44E-08	8.95E-08	FRAS1	4q21.21	0.334951306	1.44E-10	9.80E-10
PTPRA	20p13	0.298016479	1.44E-08	8.96E-08	SRSF11	1p31.1	0.334688881	1.49E-10	1.01E-09
DDX52	17q12	0.297953573	1.45E-08	9.02E-08	DOCK4	7q31.1	0.334664963	1.49E-10	1.02E-09
UBN1	16p13.3	0.297926786	1.45E-08	9.04E-08	DDX18	2q14.1	0.334627093	1.50E-10	1.02E-09
DYNC2H1	11q22.3	0.297845051	1.47E-08	9.13E-08	BTAF1	10q23.32	0.334426921	1.54E-10	1.05E-09
TTYH3	7p22.3	0.297829405	1.47E-08	9.14E-08	DPH3P1	20q13.33	0.334358696	1.55E-10	1.06E-09
CBLN4	20q13.2	0.29775042	1.48E-08	9.22E-08	BMT2	7q31.1	0.334314734	1.56E-10	1.06E-09
EPHA3	3p11.1	0.297668122	1.50E-08	9.30E-08	PGM5P2	9p11.2	0.334296329	1.57E-10	1.06E-09
SEMA3C	7q21.11	0.297648132	1.50E-08	9.32E-08	ZNF665	19q13.42	0.334292716	1.57E-10	1.06E-09
SUGP2	19p13.11	0.2975347	1.52E-08	9.44E-08	MPHOSPH8	13q12.11	0.334286569	1.57E-10	1.06E-09
DLAT	11q23.1	0.297489427	1.53E-08	9.49E-08	EPB41L4A	5q22.1-q22	0.334272023	1.57E-10	1.07E-09
VGLL3	3p12.1	0.297475211	1.53E-08	9.50E-08	METTL24	6q21	0.33421795	1.58E-10	1.07E-09
CEP44	4q34.1	0.297437889	1.54E-08	9.54E-08	RNF13	3q25.1	0.334060462	1.62E-10	1.10E-09
ZNF512	2p23.3	0.297154574	1.59E-08	9.86E-08	CCDC50	3q28	0.334002945	1.63E-10	1.10E-09
IL7R	5p13.2	0.297101935	1.60E-08	9.91E-08	LRRC8B	1p22.2	0.333755506	1.68E-10	1.14E-09
CASC2	10q26.11	0.297081726	1.60E-08	9.93E-08	TUSC3	8p22	0.333747819	1.68E-10	1.14E-09
DZIP1	13q32.1	0.296988001	1.62E-08	1.00E-07	MFSD1	3q25.32	0.333714504	1.69E-10	1.14E-09
ZNF462	9q31.2	0.296951999	1.63E-08	1.01E-07	ADAM12	10q26.2	0.333698298	1.70E-10	1.15E-09
LOC100129034	9q33.3	0.296876668	1.64E-08	1.02E-07	FAM168B	2q21.1	0.33363136	1.71E-10	1.16E-09
PPIP5K1	15q15.3	0.296871258	1.64E-08	1.02E-07	THBS2	6q27	0.333627421	1.71E-10	1.16E-09
IGSF9B	11q25	0.296835356	1.65E-08	1.02E-07	CAPN6	Xq23	0.333490891	1.74E-10	1.18E-09
MINAR1	15q25.1	0.296823233	1.65E-08	1.02E-07	ZNF207	17q11.2	0.333449412	1.75E-10	1.18E-09
PTPRE	10q26.2	0.296741133	1.67E-08	1.03E-07	GVINP1	11p15.4	0.333383946	1.77E-10	1.19E-09
COMMD2	3q25.1	0.296715242	1.67E-08	1.03E-07	ZDHHC2	8p22	0.333359743	1.77E-10	1.20E-09
RFX1	19p13.12	0.296698422	1.68E-08	1.03E-07	TBX20	7p14.2	0.333355707	1.77E-10	1.20E-09
FLG2	1q21.3	0.296639397	1.69E-08	1.04E-07	INHBA	7p14.1	0.333191462	1.81E-10	1.22E-09
GATC	12q24.31	0.296596079	1.70E-08	1.04E-07	ULBP3	6q25.1	0.333191129	1.81E-10	1.22E-09
USH1C	11p15.1	0.296564464	1.70E-08	1.05E-07	LLGL1	17p11.2	0.333057896	1.85E-10	1.24E-09
PXDN	2p25.3	0.296543239	1.71E-08	1.05E-07	MN1	22q12.1	0.332906415	1.88E-10	1.26E-09
FKTN	9q31.2	0.29652162	1.71E-08	1.05E-07	ZC3H6	2q14.1	0.332719222	1.93E-10	1.30E-09
CCN4	8q24.22	0.296451902	1.72E-08	1.06E-07	FAM106C	17p11.2	0.332656792	1.95E-10	1.31E-09
PAPOLA	14q32.2	0.296450983	1.72E-08	1.06E-07	DDX52	17q12	0.33264761	1.95E-10	1.31E-09
CIPC	14q24.3	0.296364708	1.74E-08	1.07E-07	ZBTB34	9q33.3	0.332544796	1.98E-10	1.32E-09
TES	7q31.2	0.296342783	1.75E-08	1.07E-07	RAB6A	11q13.4	0.332437734	2.00E-10	1.34E-09
GAB2	11q14.1	0.296324275	1.75E-08	1.07E-07	XPOT	12q14.2	0.332432893	2.01E-10	1.34E-09
CDC42BPB	14q32.32	0.296297224	1.76E-08	1.08E-07	MEF2C	5q14.3	0.332373098	2.02E-10	1.35E-09
TMEM131	2q11.2	0.296170558	1.78E-08	1.09E-07	FRMD8	11q13.1	0.332365125	2.02E-10	1.35E-09
ZNF33A	10p11.1	0.296164842	1.78E-08	1.09E-07	SEL1L3	4p15.2	0.332352882	2.03E-10	1.36E-09
SNX4	3q21.2	0.296036973	1.81E-08	1.11E-07	PMP22	17p12	0.332297073	2.04E-10	1.36E-09
NSD3	8p11.23	0.296032153	1.81E-08	1.11E-07	RGS18	1q31.2	0.332246503	2.06E-10	1.37E-09
GJA1	6q22.31	0.296022451	1.81E-08	1.11E-07	HNRNPK	9q21.32	0.332217061	2.06E-10	1.38E-09
A4GNT	3q22.3	0.295986869	1.82E-08	1.11E-07	TTC14	3q26.33	0.332209088	2.07E-10	1.38E-09
TMPPE	3p22.3	0.295865054	1.85E-08	1.13E-07	DCLK2	4q31.23-q3	0.332194021	2.07E-10	1.38E-09
CDC42BPA	1q42.13	0.295790673	1.86E-08	1.14E-07	DSG2	18q12.1	0.332126229	2.09E-10	1.39E-09
PRMT3	11p15.1	0.295701002	1.88E-08	1.15E-07	PPIL4	6q25.1	0.33203312	2.11E-10	1.41E-09
PROSER1	13q13.3	0.295671083	1.89E-08	1.15E-07	SLC66A3	2p25.1	0.332000944	2.12E-10	1.41E-09
ZNF624	17p11.2	0.295544395	1.92E-08	1.17E-07	EEF2K	16p12.2	0.331965067	2.13E-10	1.42E-09
ZNF638	2p13.3-p13.	0.295415693	1.94E-08	1.18E-07	NSD1	5q35.3	0.331919841	2.15E-10	1.43E-09
TRIM52	5q35.3	0.29528697	1.97E-08	1.20E-07	TAB2	6q25.1	0.331913814	2.15E-10	1.43E-09
GOLGB1	3q13.33	0.295268747	1.98E-08	1.20E-07	TCTN2	12q24.31	0.331831809	2.17E-10	1.44E-09
AMMECR1	Xq23	0.295212653	1.99E-08	1.21E-07	GPR141	7p14.1	0.331765262	2.19E-10	1.45E-09
ADORA2B	17p12	0.294941544	2.05E-08	1.25E-07	CALHM5	6q22.1	0.331660681	2.22E-10	1.47E-09
PCDHB10	5q31.3	0.294925487	2.06E-08	1.25E-07	POSTN	13q13.3	0.331638301	2.23E-10	1.48E-09
GNA12	7p22.3-p22.	0.294818574	2.08E-08	1.26E-07	RTN1	14q23.1	0.331621411	2.23E-10	1.48E-09
PTAFR	1p35.3	0.294808893	2.08E-08	1.26E-07	PLXDC2	10p12.31	0.331574975	2.25E-10	1.49E-09
THRAP3	1p34.3	0.294651596	2.12E-08	1.29E-07	EIF3C	16p11.2	0.331509739	2.26E-10	1.50E-09

USP54	10q22.2	0.294601034	2.13E-08	1.29E-07	FOXC1	6p25.3	0.331490122	2.27E-10	1.50E-09
CAMK2D	4q26	0.294534689	2.15E-08	1.30E-07	ZNF169	9q22.32	0.331426056	2.29E-10	1.52E-09
POGZ	1q21.3	0.294321704	2.20E-08	1.33E-07	ITGA11	15q23	0.331293937	2.33E-10	1.54E-09
CH25H	10q23.31	0.294220785	2.23E-08	1.35E-07	IARS	9q22.31	0.331277707	2.34E-10	1.54E-09
ZNF675	19p12	0.294193857	2.24E-08	1.35E-07	LYST	1q42.3	0.331215064	2.35E-10	1.56E-09
RAP2A	13q32.1	0.294187471	2.24E-08	1.35E-07	KATNAL1	13q12.3	0.331124802	2.38E-10	1.57E-09
5-Mar	10q23.32-q2	0.294163674	2.24E-08	1.36E-07	LINC00662	19q11	0.331035109	2.41E-10	1.59E-09
UHRF1BP1	6p21.31	0.294127797	2.25E-08	1.36E-07	DENND6A	3p14.3	0.331030269	2.41E-10	1.59E-09
KPNA5	6q22.1	0.294076871	2.27E-08	1.37E-07	AGPS	2q31.2	0.331029296	2.41E-10	1.59E-09
DUSP18	22q12.2	0.293980018	2.29E-08	1.38E-07	TLR7	Xp22.2	0.330998378	2.42E-10	1.60E-09
PAQR8	6p12.2	0.29387324	2.32E-08	1.40E-07	OCLN	5q13.2	0.330915519	2.45E-10	1.62E-09
PPWD1	5q12.3	0.29386299	2.32E-08	1.40E-07	CCDC121	2p23.3	0.330803901	2.49E-10	1.64E-09
ZNFX1	20q13.13	0.293853045	2.33E-08	1.40E-07	MAGEE1	Xq13.3	0.330785417	2.49E-10	1.64E-09
PFKM	12q13.11	0.293835655	2.33E-08	1.40E-07	ZNF493	19p12	0.330776566	2.49E-10	1.64E-09
TSR1	17p13.3	0.293818855	2.33E-08	1.41E-07	FIP1L1	4q12	0.330768309	2.50E-10	1.64E-09
SLC16A6	17q24.2	0.293759683	2.35E-08	1.41E-07	ZNF260	19q13.12	0.330751224	2.50E-10	1.65E-09
XPO7	8p21.3	0.293658262	2.38E-08	1.43E-07	HAS2	8q24.13	0.330659376	2.53E-10	1.67E-09
ZNF91	19p12	0.293552055	2.41E-08	1.44E-07	PMAIP1	18q21.32	0.330655738	2.53E-10	1.67E-09
AASDH	4q12	0.293462931	2.43E-08	1.46E-07	NAP1L3	Xq21.32	0.330572921	2.56E-10	1.68E-09
ZCCHC12	Xq24	0.293441622	2.44E-08	1.46E-07	SREK1	5q12.3	0.330517619	2.58E-10	1.70E-09
ZNF845	19q13.42	0.2934262	2.44E-08	1.46E-07	GRID2IP	7p22.1	0.33050594	2.58E-10	1.70E-09
UBE2G1	17p13.2	0.293414526	2.45E-08	1.47E-07	RHOT1	17q11.2	0.330460221	2.60E-10	1.71E-09
LRIF1	1p13.3	0.293411394	2.45E-08	1.47E-07	ARSJ	4q26	0.330414283	2.62E-10	1.72E-09
RBBP6	16p12.1	0.293380642	2.45E-08	1.47E-07	CASC2	10q26.11	0.330404306	2.62E-10	1.72E-09
PXYLP1	3q23	0.29319727	2.51E-08	1.50E-07	RALB	2q14.2	0.330312441	2.65E-10	1.74E-09
SLC35A5	3q13.2	0.293185596	2.51E-08	1.50E-07	CLSPN	1p34.3	0.330274682	2.66E-10	1.75E-09
SOCS7	17q12	0.293108431	2.53E-08	1.52E-07	COL4A3BP	5q13.3	0.330249229	2.67E-10	1.75E-09
CEP135	4q12	0.293089354	2.54E-08	1.52E-07	ZNF500	16p13.3	0.330239263	2.68E-10	1.75E-09
CDKL1	14q21.3	0.293077777	2.54E-08	1.52E-07	HCFC1	Xq28	0.330102589	2.72E-10	1.78E-09
LAPTM4A	2p24.1	0.293056324	2.55E-08	1.52E-07	BRD3	9q34.2	0.330088352	2.73E-10	1.79E-09
FOXJ2	12p13.31	0.292995675	2.57E-08	1.53E-07	RUNDC1	17q21.31	0.330063603	2.74E-10	1.79E-09
PLA2R1	2q24.2	0.292908344	2.59E-08	1.54E-07	IVNS1ABP	1q25.3	0.329950989	2.78E-10	1.82E-09
IRF2	4q35.1	0.29280917	2.62E-08	1.56E-07	FAM163A	1q25.2	0.329906535	2.80E-10	1.83E-09
PCDHGA5	5q31.3	0.292795103	2.62E-08	1.56E-07	GUCY1A2	11q22.3	0.329906235	2.80E-10	1.83E-09
TLE4	9q21.31	0.292748969	2.64E-08	1.57E-07	HEATR1	1q43	0.329859706	2.81E-10	1.84E-09
NMD3	3q26.1	0.29272204	2.65E-08	1.57E-07	ZNF12	7p22.1	0.329809307	2.83E-10	1.85E-09
EBF2	8p21.2	0.292691896	2.66E-08	1.58E-07	CD84	1q23.3	0.329723031	2.86E-10	1.87E-09
PRPF38B	1p13.3	0.292554492	2.70E-08	1.60E-07	CTBP2	10q26.13	0.329675765	2.88E-10	1.88E-09
PCM1	8p22	0.292542085	2.70E-08	1.61E-07	B3GALT5-AS1	21q22.2	0.329495754	2.95E-10	1.92E-09
AMPD3	11p15.4	0.292511333	2.71E-08	1.61E-07	APOBEC3C	22q13.1	0.329487837	2.95E-10	1.92E-09
WDR91	7q33	0.292461504	2.73E-08	1.62E-07	ATXN7L3B	12q21.1	0.329475593	2.96E-10	1.92E-09
DYNC1I1	7q21.3	0.292460733	2.73E-08	1.62E-07	RAP2B	3q25.2	0.329262893	3.04E-10	1.98E-09
PCDHAC2	5q31.3	0.292437147	2.73E-08	1.62E-07	TMEM39A	3q13.33	0.328774281	3.24E-10	2.10E-09
RPUSD4	11q24.2	0.292416515	2.74E-08	1.62E-07	SHROOM4	Xp11.22	0.328715055	3.27E-10	2.12E-09
ARHGEF18	19p13.2	0.292194419	2.81E-08	1.67E-07	C5ORF51	5p13.1	0.328666649	3.29E-10	2.13E-09
COPB2	3q23	0.292156427	2.82E-08	1.67E-07	NPY4R	10q11.22	0.328646686	3.30E-10	2.13E-09
SNAPC1	14q23.2	0.29211099	2.84E-08	1.68E-07	ARHGEF35	7q35	0.328342901	3.43E-10	2.22E-09
DDR1	6p21.33	0.291964634	2.88E-08	1.71E-07	CHRNA6	8p11.21	0.328293129	3.45E-10	2.23E-09
NOC3L	10q23.33	0.291931035	2.90E-08	1.71E-07	HAUS6	9p22.1	0.32828396	3.46E-10	2.24E-09
ANGEL2	1q32.3	0.291810021	2.94E-08	1.73E-07	LMLN	3q29	0.328205087	3.49E-10	2.26E-09
IGDCC4	15q22.31	0.291802806	2.94E-08	1.74E-07	PNMA1	14q24.3	0.328184017	3.50E-10	2.26E-09
DNAAF5	7p22.3	0.291661957	2.99E-08	1.76E-07	PPIP5K2	5q21.1	0.328155543	3.51E-10	2.27E-09
NID1	1q42.3	0.291627503	3.00E-08	1.77E-07	GPR107	9q34.11	0.328123082	3.53E-10	2.28E-09
CD200	3q13.2	0.291558596	3.02E-08	1.78E-07	CLSTN2	3q23	0.328110799	3.53E-10	2.28E-09
ESCO1	18q11.2	0.291539255	3.03E-08	1.78E-07	GAS7	17p13.1	0.32801716	3.58E-10	2.31E-09
POM121	7q11.23	0.291528129	3.03E-08	1.79E-07	SULF1	8q13.2-q13	0.327992672	3.59E-10	2.32E-09
RGPD6	2q13	0.291198422	3.15E-08	1.85E-07	TSPAN11	12p11.21	0.32787614	3.64E-10	2.35E-09
STAG3L2	7q11.23	0.291166511	3.16E-08	1.86E-07	NNAT	20q11.23	0.327854828	3.65E-10	2.35E-09
LSM14B	20q13.33	0.291139176	3.17E-08	1.86E-07	ZNF91	19p12	0.32784233	3.66E-10	2.36E-09
UBE3A	15q11.2	0.291131488	3.17E-08	1.87E-07	E2F3	6p22.3	0.327731851	3.71E-10	2.39E-09
TAF1B	2p25.1	0.291115299	3.18E-08	1.87E-07	RASSF2	20p13	0.327731281	3.71E-10	2.39E-09
PLA2G4A	1q31.1	0.291110154	3.18E-08	1.87E-07	TENM3	4q34.3-q35	0.327728249	3.71E-10	2.39E-09
BCORL1	Xq26.1	0.291093617	3.18E-08	1.87E-07	LRBA	4q31.3	0.327617386	3.77E-10	2.42E-09
FOXC2	16q24.1	0.291086404	3.19E-08	1.87E-07	PITPNM3	17p13.2-p1	0.32753577	3.81E-10	2.45E-09
FTX	Xq13.2	0.291010758	3.21E-08	1.89E-07	MCMBP	10q26.11	0.327481281	3.84E-10	2.46E-09
HLTF	3q24	0.291005348	3.22E-08	1.89E-07	ZDHHC15	Xq13.3	0.327464344	3.84E-10	2.47E-09
EXTL3	8p21.1	0.290833366	3.28E-08	1.92E-07	ADGRB3	6q12-q13	0.327411758	3.87E-10	2.48E-09
GPR173	Xp11.22	0.290773733	3.30E-08	1.94E-07	RPRD2	1q21.2	0.327407248	3.87E-10	2.48E-09
NELFA	4p16.3	0.290725165	3.32E-08	1.94E-07	TSHZ2	20q13.2	0.327386201	3.88E-10	2.49E-09
TMEM128	4p16.3	0.290699254	3.33E-08	1.95E-07	SETD1B	12q24.31	0.32735972	3.90E-10	2.49E-09
SHANK2	11q13.3-q1	0.290563718	3.38E-08	1.98E-07	P3H2	3q28	0.327237544	3.96E-10	2.53E-09
ZNF117	7q11.21	0.290509211	3.40E-08	1.99E-07	PCDHB11	5q31.3	0.327187818	3.98E-10	2.54E-09
PWWP2A	5q33.3	0.290423626	3.44E-08	2.01E-07	LAMA1	18p11.31	0.327134294	4.01E-10	2.56E-09
CAD	2p23.3	0.290383478	3.45E-08	2.02E-07	COMMD2	3q25.1	0.327091211	4.03E-10	2.57E-09

MAP3K7	6q15	0.290349025	3.46E-08	2.02E-07	BCL9	1q21.2	0.327016302	4.07E-10	2.60E-09
AHCTF1	1q44	0.290348455	3.46E-08	2.02E-07	XPR1	1q25.3	0.326983557	4.09E-10	2.61E-09
DMD	Xp21.2-p21.	0.290210926	3.52E-08	2.05E-07	NBEAL2	3p21.31	0.326976438	4.10E-10	2.61E-09
DHX57	2p22.1	0.290194411	3.53E-08	2.06E-07	HNRNPH2	Xq22.1	0.326877799	4.15E-10	2.64E-09
ZSWIM4	19p13.13-p1	0.290099187	3.56E-08	2.08E-07	MMRN1	4q22.1	0.326874502	4.15E-10	2.64E-09
FGF2	4q28.1	0.290001521	3.60E-08	2.10E-07	RAB23	6p12.1-p11	0.32684859	4.16E-10	2.65E-09
BNIP2	15q22.2	0.289991677	3.61E-08	2.10E-07	PDS5B	13q13.1	0.326830367	4.17E-10	2.66E-09
NOTCH3	19p13.12	0.289935299	3.63E-08	2.11E-07	MAP3K7	6q15	0.3267999	4.19E-10	2.67E-09
ZNF195	11p15.4	0.289931882	3.63E-08	2.11E-07	MTMR6	13q12.13	0.326776551	4.20E-10	2.67E-09
TMEM170A	16q23.1	0.289839626	3.67E-08	2.14E-07	PRKCH	14q23.1	0.326769433	4.21E-10	2.67E-09
ASB7	15q26.3	0.289831369	3.67E-08	2.14E-07	S100PBP	1p35.1	0.32676687	4.21E-10	2.67E-09
WDR19	4p14	0.289796346	3.69E-08	2.14E-07	DLEU7	13q14.3	0.326723798	4.23E-10	2.69E-09
PIAS3	1q21.1	0.289618099	3.76E-08	2.19E-07	DUSP7	3p21.2	0.326721597	4.23E-10	2.69E-09
WASHC2C	10q11.22	0.289597313	3.77E-08	2.19E-07	CSDE1	1p13.2	0.32669056	4.25E-10	2.70E-09
ENOX2	Xq26.1	0.289593612	3.77E-08	2.19E-07	WASHC5	8q24.13	0.326639307	4.28E-10	2.71E-09
SERPINB9	6p25.2	0.289567131	3.78E-08	2.20E-07	PFKM	12q13.11	0.326595172	4.30E-10	2.73E-09
KDM7A	7q34	0.289535403	3.80E-08	2.21E-07	RNF44	5q35.2	0.326593464	4.30E-10	2.73E-09
BLOC1S6	15q21.1	0.289510753	3.81E-08	2.21E-07	ZSCAN12	6p22.1	0.326562143	4.32E-10	2.74E-09
TCF3	19p13.3	0.289508475	3.81E-08	2.21E-07	RAB5C	17q21.2	0.326515611	4.35E-10	2.75E-09
SOWAHB	4q21.1	0.289507418	3.81E-08	2.21E-07	PIP4P2	8q21.3	0.3264286	4.40E-10	2.78E-09
MEX3D	19p13.3	0.289332527	3.89E-08	2.25E-07	RNF219	13q31.1	0.326373361	4.43E-10	2.80E-09
NAPEPLD	7q22.1	0.289310581	3.89E-08	2.26E-07	ZNF521	18q11.2	0.326351459	4.44E-10	2.81E-09
ZNF345	19q13.12	0.289256786	3.92E-08	2.27E-07	ZFYVE16	5q14.1	0.326321823	4.46E-10	2.82E-09
USP10	16q24.1	0.289242244	3.92E-08	2.27E-07	WNT2B	1p13.2	0.326315828	4.46E-10	2.82E-09
KLHL20	1q25.1	0.289204658	3.94E-08	2.28E-07	LRATD1	2p24.3	0.326211629	4.52E-10	2.86E-09
MCPH1	8p23.1	0.289105569	3.99E-08	2.31E-07	ZNF280C	Xq26.1	0.326151264	4.56E-10	2.88E-09
ZNF654	3p11.1	0.289096457	3.99E-08	2.31E-07	ATP2A2	12q24.11	0.326139875	4.56E-10	2.88E-09
GNA13	17q24.1	0.288964054	4.05E-08	2.34E-07	LONRF1	8p23.1	0.326078086	4.60E-10	2.90E-09
PKP2	12p11.21	0.288543209	4.25E-08	2.45E-07	POLA1	Xp22.11-p2	0.32607455	4.60E-10	2.90E-09
MAML3	4q31.1	0.288494804	4.27E-08	2.46E-07	KIAA1549L	11p13	0.326052775	4.62E-10	2.91E-09
TEDDM1	1q25.3	0.288438322	4.30E-08	2.48E-07	YWHAQ	2p25.1	0.32605246	4.62E-10	2.91E-09
IGSF5	21q22.2	0.288348078	4.34E-08	2.50E-07	PSMD5	9q33.2	0.325934008	4.69E-10	2.95E-09
ADAMTS15	11q24.3	0.288333673	4.35E-08	2.51E-07	ASPN	9q22.31	0.325776548	4.78E-10	3.01E-09
FSD1L	9q31.2	0.28831074	4.36E-08	2.51E-07	WDR66	12q24.31	0.325695722	4.83E-10	3.04E-09
RNF4	4p16.3	0.288163083	4.43E-08	2.55E-07	ADGRG1	16q21	0.325680021	4.84E-10	3.05E-09
PAK2	3q29	0.288160805	4.43E-08	2.55E-07	MED13	17q23.2	0.325461341	4.98E-10	3.13E-09
USP24	1p32.3	0.288131477	4.45E-08	2.56E-07	PAMR1	11p13	0.325423067	5.01E-10	3.14E-09
WDR92	2p14	0.288102148	4.46E-08	2.57E-07	YBX3P1	16p11.2	0.325372842	5.04E-10	3.16E-09
MYRF	11q12.2	0.288077946	4.47E-08	2.57E-07	CNTN1	12q12	0.325210235	5.14E-10	3.23E-09
CCR4	3p22.3	0.287934246	4.54E-08	2.61E-07	FICD	12q23.3	0.325182297	5.16E-10	3.24E-09
FAM83G	17p11.2	0.287866669	4.58E-08	2.63E-07	RIC8B	12q23.3	0.325020565	5.27E-10	3.30E-09
KIAA0556	16p12.1	0.287830792	4.60E-08	2.64E-07	RBM26	13q31.1	0.324969597	5.31E-10	3.32E-09
SRPK1	6p21.31	0.287799471	4.61E-08	2.65E-07	CPNE8	12q12	0.324933435	5.33E-10	3.34E-09
RPL32P3	3q21.3	0.28775078	4.64E-08	2.66E-07	FAM102A	9q34.11	0.324919483	5.34E-10	3.34E-09
SAMHD1	20q11.23	0.287678741	4.68E-08	2.68E-07	TP53I11	11p11.2	0.324865952	5.38E-10	3.37E-09
TMEM184B	22q13.1	0.287667067	4.68E-08	2.68E-07	USP14	18p11.32	0.324754619	5.46E-10	3.41E-09
CNNM4	2q11.2	0.287658525	4.69E-08	2.69E-07	BBS12	4q27	0.324735541	5.47E-10	3.42E-09
ABCA1	9q31.1	0.287657671	4.69E-08	2.69E-07	CACHD1	1p31.3	0.324697386	5.50E-10	3.43E-09
GABRQ	Xq28	0.287470836	4.79E-08	2.74E-07	ZBTB21	21q22.3	0.324681345	5.51E-10	3.44E-09
NACC2	9q34.3	0.287438706	4.80E-08	2.75E-07	CEP44	4q34.1	0.32466037	5.52E-10	3.45E-09
SH3GL3	15q25.2	0.287426371	4.81E-08	2.75E-07	CCDC144CP	17p11.2	0.324656369	5.52E-10	3.45E-09
SLC35E2B	1p36.33	0.287362681	4.84E-08	2.77E-07	PGR	11q22.1	0.324580531	5.58E-10	3.48E-09
PPP1R12B	1q32.1	0.287285232	4.89E-08	2.79E-07	DENND2C	1p13.2	0.324574949	5.58E-10	3.48E-09
SMC2	9q31.1	0.287283524	4.89E-08	2.79E-07	STRN3	14q12	0.324498069	5.64E-10	3.51E-09
TTC5	14q11.2	0.287171621	4.95E-08	2.82E-07	ZNF675	19p12	0.324453934	5.67E-10	3.53E-09
LOC646762	7p14.3	0.287083637	5.00E-08	2.85E-07	ZNF461	19q13.12	0.324445392	5.68E-10	3.54E-09
ARHGAP42	11q22.1	0.287032384	5.03E-08	2.87E-07	DDX60	4q32.3	0.324249492	5.82E-10	3.62E-09
PRXL2C	9q22.33	0.28699012	5.05E-08	2.88E-07	ZFP36L2	2p21	0.324197669	5.86E-10	3.65E-09
PAXBP1	21q22.11	0.2869817	5.06E-08	2.88E-07	ZNF702P	19q13.41	0.324168885	5.88E-10	3.66E-09
RYBP	3p13	0.286957213	5.07E-08	2.89E-07	CTDSP2	12q14.1	0.324148694	5.90E-10	3.66E-09
SESTD1	2q31.2	0.286931871	5.08E-08	2.89E-07	PLEKHA3	2q31.2	0.324117942	5.92E-10	3.68E-09
PRIMPOL	4q35.1	0.286890014	5.11E-08	2.91E-07	BIRC2	11q22.2	0.324044218	5.98E-10	3.71E-09
ZNF362	1p35.1	0.286889445	5.11E-08	2.91E-07	FANCM	14q21.2	0.324001934	6.01E-10	3.73E-09
SLFN11	17q12	0.286798898	5.16E-08	2.93E-07	PDP2	16q22.1	0.32400063	6.01E-10	3.73E-09
LARGE2	11p11.2	0.286739674	5.19E-08	2.95E-07	CDK19	6q21	0.32399522	6.01E-10	3.73E-09
ZNF460	19q13.43	0.286732838	5.20E-08	2.95E-07	PPL	16p13.3	0.32378736	6.18E-10	3.83E-09
ZNF24	18q12.2	0.286710059	5.21E-08	2.96E-07	IQSEC2	Xp11.22	0.323757059	6.20E-10	3.84E-09
MBD6	12q13.3	0.286698669	5.22E-08	2.96E-07	GPR34	Xp11.4	0.32374211	6.21E-10	3.85E-09
HNRNPDL	4q21.22	0.286697246	5.22E-08	2.96E-07	ARF3	12q13.12	0.323717884	6.23E-10	3.86E-09
BAG5	14q32.33	0.286672758	5.23E-08	2.97E-07	STC2	5q35.2	0.323696813	6.25E-10	3.87E-09
DMXL2	15q21.2	0.286644284	5.25E-08	2.98E-07	CYBRD1	2q31.1	0.323577223	6.34E-10	3.93E-09
TRIM8	10q24.32	0.286622359	5.26E-08	2.98E-07	KCND2	7q31.31	0.323567045	6.35E-10	3.93E-09
R3HDM1	2q21.3	0.286569113	5.29E-08	3.00E-07	DUSP11	2p13.1	0.323561562	6.36E-10	3.93E-09
PHF20L1	8q24.22	0.286556869	5.30E-08	3.00E-07	RNF11	1p32.3	0.323505753	6.40E-10	3.96E-09

PDCD7	15q22.31	0.286459773	5.36E-08	3.04E-07	SRRM1	1p36.11	0.323504518	6.40E-10	3.96E-09
ZNF773	19q13.43	0.286429591	5.38E-08	3.04E-07	R3HDM2	12q13.3	0.323478703	6.43E-10	3.97E-09
APC	5q22.2	0.286428737	5.38E-08	3.04E-07	ZPLD1	3q12.3	0.323429666	6.47E-10	3.99E-09
ATP1A2	1q23.2	0.28627683	5.47E-08	3.09E-07	CASD1	7q21.3	0.323414922	6.48E-10	4.00E-09
FANCF	11p14.3	0.286272984	5.47E-08	3.09E-07	SPECC1	17p11.2	0.323141737	6.71E-10	4.13E-09
DVL2	17p13.1	0.286224009	5.50E-08	3.11E-07	MB21D2	3q29	0.323058713	6.78E-10	4.18E-09
ZNF573	19q13.12	0.286193054	5.52E-08	3.12E-07	PAXIP1	7q36.2	0.323051879	6.79E-10	4.18E-09
MRS2	6p22.3	0.286179875	5.53E-08	3.12E-07	ZNF717	3p12.3	0.322962279	6.86E-10	4.22E-09
FBXW8	12q24.22	0.286153272	5.54E-08	3.13E-07	SENP6	6q14.1	0.322944413	6.88E-10	4.23E-09
RBL1	20q11.23	0.286116947	5.57E-08	3.14E-07	DIPK2A	3q24	0.322866514	6.95E-10	4.27E-09
ZNF423	16q12.1	0.286101897	5.58E-08	3.14E-07	RUNX1	21q22.12	0.322850284	6.96E-10	4.28E-09
TOR1AIP1	1q25.2	0.286095043	5.58E-08	3.15E-07	FYCO1	3p21.31	0.322719873	7.08E-10	4.34E-09
SCAI	9q33.3	0.286090751	5.58E-08	3.15E-07	AASDHPPT	11q22.3	0.322660648	7.13E-10	4.38E-09
ATAD1	10q23.31	0.285985683	5.65E-08	3.18E-07	RIPOR1	16q22.1	0.322573233	7.21E-10	4.42E-09
GOLPH3	5p13.3	0.285951514	5.67E-08	3.19E-07	PRSS35	6q14.2	0.322519392	7.26E-10	4.45E-09
BCL2L2	14q11.2	0.285940857	5.68E-08	3.20E-07	LRIG3	12q14.1	0.322506604	7.27E-10	4.45E-09
UPRT	Xq13.3	0.285891434	5.71E-08	3.21E-07	CRMP1	4p16.2	0.322430009	7.35E-10	4.49E-09
LRRK1	15q26.3	0.285714611	5.82E-08	3.27E-07	TNFRSF10A	8p21.3	0.322404098	7.37E-10	4.51E-09
GPR20	8q24.3	0.285670764	5.85E-08	3.29E-07	CAPZA2	7q31.2	0.322328642	7.44E-10	4.55E-09
RNF13	3q25.1	0.285667629	5.85E-08	3.29E-07	HEPH	Xq12	0.3223201	7.45E-10	4.55E-09
KLF3-AS1	4p14	0.285662219	5.85E-08	3.29E-07	TMEM130	7q22.1	0.32216486	7.60E-10	4.64E-09
SLC35E3	12q15	0.285537503	5.94E-08	3.33E-07	TMEM150C	4q21.22	0.322098573	7.66E-10	4.67E-09
TRAPPC10	21q22.3	0.285457207	5.99E-08	3.36E-07	ACTN4	19q13.2	0.322032798	7.73E-10	4.71E-09
PDE8B	5q13.3	0.285433917	6.00E-08	3.37E-07	MMP2	16q12.2	0.322009734	7.75E-10	4.72E-09
ZNF135	19q13.43	0.285381222	6.04E-08	3.39E-07	PABPC4L	4q28.3	0.321864736	7.89E-10	4.81E-09
LILRA1	19q13.42	0.285347205	6.06E-08	3.40E-07	UBR3	2q31.1	0.321846579	7.91E-10	4.82E-09
MAP3K13	3q27.2	0.285269015	6.11E-08	3.43E-07	TMTC1	12p11.22	0.321824654	7.93E-10	4.83E-09
RCHY1	4q21.1	0.285177878	6.18E-08	3.46E-07	PCDHB10	5q31.3	0.321808027	7.95E-10	4.84E-09
PJA2	5q21.3	0.285176454	6.18E-08	3.46E-07	SEC22B	1p12	0.321728412	8.03E-10	4.88E-09
RBMS3	3p24.1	0.285106887	6.23E-08	3.48E-07	KIF1BP	10q22.1	0.321723879	8.04E-10	4.88E-09
RAB5A	3p24.3	0.285094449	6.23E-08	3.49E-07	ZNF471	19q13.43	0.321712895	8.05E-10	4.89E-09
CXADRP3	18p11.21	0.285080747	6.24E-08	3.49E-07	GLYR1	16p13.3	0.321699084	8.06E-10	4.89E-09
OGA	10q24.32	0.284794334	6.44E-08	3.60E-07	EPB41L2	6q23.1-q23	0.321629607	8.13E-10	4.93E-09
VLDLR	9p24.2	0.284790978	6.45E-08	3.60E-07	TMEM233	12q24.23	0.321628095	8.14E-10	4.93E-09
SLC30A1	1q32.3	0.284595017	6.59E-08	3.68E-07	BRMS1L	14q13.2	0.321582364	8.18E-10	4.96E-09
TSSK4	14q12	0.284556267	6.62E-08	3.69E-07	UBE2W	8q21.11	0.321552158	8.21E-10	4.98E-09
PTPN13	4q21.3	0.284530462	6.64E-08	3.70E-07	CSPG4	15q24.2	0.321536378	8.23E-10	4.99E-09
ZNF594	17p13.2	0.284529812	6.64E-08	3.70E-07	PCDHGA10	5q31.3	0.321471476	8.30E-10	5.03E-09
TMEM209	7q32.2	0.284507317	6.65E-08	3.71E-07	RPL32P3	3q21.3	0.321358251	8.42E-10	5.10E-09
ABI2	2q33.2	0.284415347	6.72E-08	3.74E-07	KCNJ12	17p11.2	0.321358081	8.42E-10	5.10E-09
ZNF770	15q14	0.284368365	6.75E-08	3.76E-07	OLFML2A	9q33.3	0.321297886	8.48E-10	5.13E-09
DLG4	17p13.1	0.284342738	6.77E-08	3.77E-07	TCEANC2	1p32.3	0.321243239	8.54E-10	5.17E-09
RTL6	22q13.31	0.28428579	6.82E-08	3.79E-07	AASDH	4q12	0.321071804	8.73E-10	5.28E-09
PLXNA1	3q21.3	0.284092737	6.96E-08	3.87E-07	GOLGA2	9q34.11	0.32104817	8.76E-10	5.29E-09
HECTD1	14q12	0.284034935	7.01E-08	3.90E-07	GNAI3	1p13.3	0.321022828	8.78E-10	5.30E-09
DHFR2	3q11.2	0.283994502	7.04E-08	3.91E-07	TTN	2q31.2	0.321014286	8.79E-10	5.31E-09
PIGG	4p16.3	0.283973147	7.06E-08	3.92E-07	CCDC14	3q21.1	0.320977555	8.84E-10	5.33E-09
HCG11	6p22.2	0.283920083	7.10E-08	3.94E-07	LRP12	8q22.3	0.320885015	8.94E-10	5.39E-09
HRH2	5q35.2	0.28389506	7.12E-08	3.95E-07	SLC16A6	17q24.2	0.320835491	9.00E-10	5.42E-09
HERC2	15q13.1	0.28374621	7.23E-08	4.01E-07	CAVIN1	17q21.2	0.320691107	9.16E-10	5.52E-09
CCSER1	4q22.1	0.283673903	7.29E-08	4.04E-07	ADAM28	8p21.2	0.320687696	9.17E-10	5.52E-09
GREB1	2p25.1	0.283572234	7.37E-08	4.09E-07	MID2	Xq22.3	0.320628465	9.23E-10	5.56E-09
ZNF470	19q13.43	0.283356687	7.55E-08	4.18E-07	FBXL5	4p15.32	0.32059802	9.27E-10	5.58E-09
CDC27	17q21.32	0.283305169	7.59E-08	4.20E-07	HSPA13	21q11.2	0.320591449	9.28E-10	5.58E-09
HCG18	6p22.1	0.283251903	7.64E-08	4.22E-07	UGT1A8	2q37.1	0.320510493	9.37E-10	5.64E-09
SMG1P5	16p11.2	0.283251623	7.64E-08	4.22E-07	ST8SIA6	10p12.33	0.320485598	9.40E-10	5.65E-09
GPRASP1	Xq22.1	0.283244215	7.64E-08	4.23E-07	WASF2	1p36.11	0.320457337	9.44E-10	5.67E-09
TSHZ3	19q12	0.283152813	7.72E-08	4.26E-07	ZNF112	19q13.31	0.320449529	9.45E-10	5.67E-09
TBX4	17q23.2	0.283105536	7.76E-08	4.28E-07	ACTBL2	5q11.2	0.320377329	9.53E-10	5.72E-09
FGFR1	8p11.23	0.283028952	7.83E-08	4.32E-07	CHAMP1	13q34	0.320375332	9.54E-10	5.72E-09
MRTFA	22q13.1-q1:	0.282775819	8.05E-08	4.44E-07	PPP3CA	4q24	0.320331647	9.59E-10	5.75E-09
GDF11	12q13.2	0.282645408	8.16E-08	4.50E-07	CORO2B	15q23	0.320296265	9.63E-10	5.77E-09
MERTK	2q13	0.282634304	8.17E-08	4.50E-07	COL10A1	6q22.1	0.320296034	9.63E-10	5.77E-09
ACTL6A	3q26.33	0.282554292	8.25E-08	4.54E-07	STK38L	12p11.23	0.320269693	9.66E-10	5.79E-09
CBLL1	7q22.3	0.28248026	8.31E-08	4.57E-07	SLC36A1	5q33.1	0.320259466	9.68E-10	5.80E-09
BBS2	16q13	0.28245264	8.34E-08	4.58E-07	ANP32A-IT1	15q23	0.320252742	9.68E-10	5.80E-09
LINC00598	13q14.11	0.282397216	8.39E-08	4.61E-07	GRAMD1B	11q24.1	0.320136357	9.83E-10	5.88E-09
WNT3	17q21.31-q2	0.282271831	8.50E-08	4.67E-07	NR2F1	5q15	0.320091732	9.88E-10	5.91E-09
SPEN	1p36.21-p36	0.282247628	8.53E-08	4.68E-07	SFRP5	10q24.2	0.320090081	9.89E-10	5.91E-09
SEPTIN8	5q31.1	0.282158505	8.61E-08	4.72E-07	IRF2	4q35.1	0.320067813	9.91E-10	5.93E-09
PLIN1	15q26.1	0.282153949	8.62E-08	4.72E-07	PCDHB4	5q31.3	0.320060491	9.92E-10	5.93E-09
SLC10A7	4q31.22	0.282133448	8.63E-08	4.73E-07	TBC1D8B	Xq22.3	0.319988656	1.00E-09	5.98E-09
ARHGAP18	6q22.33	0.282009301	8.75E-08	4.80E-07	TMEM51	1p36.21	0.319950216	1.01E-09	6.01E-09
DYNC1LI2	16q22.1	0.281922171	8.84E-08	4.84E-07	ZNF638	2p13.3-p13	0.319912084	1.01E-09	6.04E-09

CYTH3	7p22.1	0.281758731	9.00E-08	4.93E-07	ZBTB2	6q25.1	0.319882448	1.01E-09	6.05E-09
MDFIC	7q31.1-q31.	0.281734528	9.02E-08	4.94E-07	MARK1	1q41	0.319866042	1.02E-09	6.06E-09
KATNBL1P6	6q24.3	0.281657689	9.10E-08	4.97E-07	NTM	11q25	0.319847687	1.02E-09	6.08E-09
EFCAB7	1p31.3	0.281551806	9.20E-08	5.03E-07	CSF2RB	22q12.3	0.319785922	1.03E-09	6.12E-09
HS6ST2	Xq26.2	0.281476213	9.28E-08	5.07E-07	SIN3B	19p13.11	0.319758587	1.03E-09	6.14E-09
DCAF10	9p13.2	0.281366523	9.39E-08	5.13E-07	MITF	3p13	0.319737231	1.03E-09	6.16E-09
HAUS6	9p22.1	0.28135611	9.40E-08	5.13E-07	HSPB7	1p36.13	0.319703334	1.04E-09	6.18E-09
TMEM182	2q12.1	0.28118128	9.58E-08	5.22E-07	RAB19	7q34	0.319648377	1.05E-09	6.22E-09
R3HCC1L	10q24.2	0.281177191	9.58E-08	5.22E-07	MAGEL2	15q11.2	0.319637705	1.05E-09	6.23E-09
LBR	1q42.12	0.28116505	9.60E-08	5.23E-07	MINDY3	10p13	0.319467037	1.07E-09	6.36E-09
PLAGL1	6q24.2	0.281103261	9.66E-08	5.26E-07	GFPT1	2p13.3	0.319467014	1.07E-09	6.36E-09
PPFIA2	12q21.31	0.28106444	9.70E-08	5.28E-07	RGPD6	2q13	0.319464616	1.07E-09	6.36E-09
HEG1	3q21.2	0.281063683	9.70E-08	5.28E-07	AVL9	7p14.3	0.319352572	1.08E-09	6.44E-09
MTX3	5q14.1	0.28100645	9.76E-08	5.31E-07	PCYT1A	3q29	0.319341444	1.09E-09	6.45E-09
FAM117B	2q33.2	0.280999229	9.77E-08	5.31E-07	AMMECR1	Xq23	0.319306991	1.09E-09	6.48E-09
RFXAP	13q13.3	0.280996769	9.78E-08	5.31E-07	ENTPD1	10q24.1	0.319250328	1.10E-09	6.52E-09
PCID2	13q34	0.280989935	9.78E-08	5.32E-07	RIT1	1q22	0.319155509	1.11E-09	6.59E-09
THSD1P1	13q14.3	0.28094694	9.83E-08	5.34E-07	ANXA3	4q21.21	0.319139228	1.11E-09	6.61E-09
SMAD4	18q21.2	0.280932988	9.84E-08	5.35E-07	ATXN2	12q24.12	0.319126751	1.12E-09	6.61E-09
LIN54	4q21.22	0.280656953	1.01E-07	5.50E-07	FAR1	11p15.3	0.318984096	1.14E-09	6.73E-09
ZNF169	9q22.32	0.280640276	1.02E-07	5.51E-07	RAB5A	3p24.3	0.318974985	1.14E-09	6.74E-09
LRP5	11q13.2	0.280434694	1.04E-07	5.63E-07	LOC441204	7p15.2	0.318934037	1.14E-09	6.77E-09
RBBP4	1p35.1	0.280368227	1.05E-07	5.67E-07	DNASE1L1	Xq28	0.318894119	1.15E-09	6.80E-09
GNPDA2	4p12	0.280361231	1.05E-07	5.67E-07	MCPH1	8p23.1	0.318863937	1.15E-09	6.83E-09
VN1R1	19q13.43	0.280316697	1.05E-07	5.69E-07	LUM	12q21.33	0.318836734	1.16E-09	6.85E-09
HMBOX1	8p21.1-p12	0.280284819	1.06E-07	5.71E-07	BRD2	6p21.32	0.318728116	1.17E-09	6.94E-09
PPIG	2q31.1	0.280165351	1.07E-07	5.78E-07	DDX46	5q31.1	0.318617353	1.19E-09	7.03E-09
FAM120A	9q22.31	0.280020683	1.09E-07	5.87E-07	ZC3H13	13q14.13	0.318615359	1.19E-09	7.03E-09
KCNQ4	1p34.2	0.279973411	1.09E-07	5.89E-07	FAT4	4q28.1	0.318609471	1.19E-09	7.03E-09
EVC	4p16.2	0.279745341	1.12E-07	6.03E-07	GNPTAB	12q23.2	0.318578343	1.20E-09	7.06E-09
AJUBA	14q11.2	0.27970804	1.12E-07	6.05E-07	TRPM7	15q21.2	0.318546168	1.20E-09	7.09E-09
CDH22	20q13.12	0.27968844	1.13E-07	6.06E-07	NXPE3	3q12.3	0.31854332	1.20E-09	7.09E-09
TP63	3q28	0.279611743	1.14E-07	6.11E-07	C3ORF70	3q27.2	0.318537626	1.20E-09	7.09E-09
VPS35	16q11.2	0.279581493	1.14E-07	6.13E-07	TLR10	4p14	0.318412572	1.22E-09	7.20E-09
ZXDA	Xp11.21	0.279580212	1.14E-07	6.13E-07	PDGFRA	4q12	0.318384889	1.23E-09	7.22E-09
EIF3C	16p11.2	0.27947683	1.15E-07	6.19E-07	DNAJC14	12q13.2	0.318378456	1.23E-09	7.22E-09
LRCH2	Xq23	0.279427383	1.16E-07	6.22E-07	PAQR3	4q21.21	0.318348844	1.23E-09	7.25E-09
ERLIN1	10q24.31	0.279422162	1.16E-07	6.23E-07	KPNA6	1p35.2	0.318345614	1.23E-09	7.25E-09
CUL2	10p11.21	0.279397674	1.16E-07	6.24E-07	COL4A3	2q36.3	0.318308147	1.24E-09	7.28E-09
DDX50	10q22.1	0.279315385	1.17E-07	6.29E-07	MTR	1q43	0.318273957	1.24E-09	7.31E-09
ADAM21P1	14q24.2	0.279229423	1.18E-07	6.35E-07	TMEM273	10q11.23	0.31820867	1.25E-09	7.37E-09
DNAJC27	2p23.3	0.279192662	1.19E-07	6.37E-07	PHF12	17q11.2	0.318181702	1.26E-09	7.39E-09
ANOS1	Xp22.31	0.279191172	1.19E-07	6.37E-07	SERPINB9	6p25.2	0.318149241	1.26E-09	7.42E-09
COL8A1	3q12.1	0.279103095	1.20E-07	6.43E-07	EPHA4	2q36.1	0.31814497	1.26E-09	7.42E-09
KDM4D	11q21	0.279096175	1.20E-07	6.43E-07	LYN	8q12.1	0.318126178	1.27E-09	7.43E-09
KLHL42	12p11.22	0.278947787	1.22E-07	6.53E-07	TLR3	4q35.1	0.318093148	1.27E-09	7.46E-09
HMGN4	6p22.2	0.278947502	1.22E-07	6.53E-07	ANXA4	2p13.3	0.318089731	1.27E-09	7.46E-09
ZMAT1	Xq22.1	0.278930702	1.22E-07	6.54E-07	ACER3	11q13.5	0.318033637	1.28E-09	7.51E-09
PDCD4	10q25.2	0.278888011	1.23E-07	6.57E-07	LARP6	15q23	0.318027253	1.28E-09	7.51E-09
SIPA1L3	19q13.13-q1	0.278832467	1.24E-07	6.61E-07	GCLM	1p22.1	0.317997476	1.29E-09	7.54E-09
DHX40	17q23.1	0.278749893	1.25E-07	6.66E-07	TLE4	9q21.31	0.317971587	1.29E-09	7.56E-09
ZBTB49	4p16.3	0.278731954	1.25E-07	6.68E-07	SRFBP1	5q23.1	0.317940528	1.30E-09	7.59E-09
RBM41	Xq22.3	0.278727296	1.25E-07	6.68E-07	ZNF720	16p11.2	0.317924298	1.30E-09	7.60E-09
PABPC5	Xq21.31	0.27867818	1.26E-07	6.71E-07	SH3BGRL	Xq21.1	0.317832492	1.31E-09	7.68E-09
MADD	11p11.2	0.278517261	1.28E-07	6.83E-07	ACTL6A	3q26.33	0.317831473	1.31E-09	7.68E-09
ZSWIM6	5q12.1	0.278512136	1.28E-07	6.83E-07	CDH17	8q22.1	0.317731232	1.33E-09	7.77E-09
HNF4G	8q21.13	0.278398525	1.30E-07	6.91E-07	RASL11B	4q12	0.317693744	1.34E-09	7.81E-09
MASTL	10p12.1	0.278386586	1.30E-07	6.91E-07	OPHN1	Xq12	0.317480674	1.37E-09	8.01E-09
MYEF2	15q21.1	0.278376021	1.30E-07	6.92E-07	ARL6IP5	3p14.1	0.317431579	1.38E-09	8.05E-09
NEDD9	6p24.2	0.278240495	1.32E-07	7.01E-07	ZMYM6	1p34.3	0.317416892	1.38E-09	8.07E-09
EME2	16p13.3	0.278171711	1.33E-07	7.07E-07	C9ORF47	9q22.1	0.317394442	1.39E-09	8.09E-09
GPR82	Xp11.4	0.278114428	1.34E-07	7.11E-07	ERO1A	14q22.1	0.317325776	1.40E-09	8.15E-09
AZIN1	8q22.3	0.27805458	1.34E-07	7.15E-07	SOX7	8p23.1	0.317292177	1.41E-09	8.18E-09
CYSLTR1	Xq21.1	0.278050487	1.34E-07	7.15E-07	AXL	19q13.2	0.317282496	1.41E-09	8.19E-09
ZNF667	19q13.43	0.277896177	1.37E-07	7.26E-07	PHF21A	11p11.2	0.317278674	1.41E-09	8.19E-09
HSP90AB2P	4p15.33	0.277874055	1.37E-07	7.28E-07	KLHL42	12p11.22	0.31720106	1.42E-09	8.27E-09
PIP4K2B	17q12	0.277827623	1.38E-07	7.31E-07	MYO3A	10p12.1	0.317186603	1.42E-09	8.28E-09
STXBP5	6q24.3	0.277820524	1.38E-07	7.32E-07	MAPRE1	20q11.21	0.31712532	1.43E-09	8.34E-09
ZNF471	19q13.43	0.277794846	1.38E-07	7.34E-07	HOOK3	8p11.21	0.317044738	1.45E-09	8.42E-09
SLC33A1	3q25.31	0.277755869	1.39E-07	7.37E-07	KATNBL1P6	6q24.3	0.317016594	1.45E-09	8.45E-09
RAPGEF1	9q34.13	0.277726541	1.39E-07	7.39E-07	ITGB3	17q21.32	0.317008577	1.46E-09	8.45E-09
INTS6	13q14.3	0.277700345	1.40E-07	7.40E-07	GALNT5	2q24.1	0.316813327	1.49E-09	8.66E-09
SLC6A6	3p25.1	0.277615208	1.41E-07	7.47E-07	MMP7	11q22.2	0.316808651	1.49E-09	8.66E-09
STK24	13q32.2	0.27760211	1.41E-07	7.48E-07	VPS50	7q21.2-q21	0.316806412	1.49E-09	8.66E-09

SH3RF3	2q13	0.277479692	1.43E-07	7.57E-07	SKI	1p36.33-p3	0.316779362	1.50E-09	8.69E-09
SNX27	1q21.3	0.277372895	1.45E-07	7.65E-07	URB1	21q22.11	0.316763986	1.50E-09	8.70E-09
MAN1A1	6q22.31	0.277345275	1.45E-07	7.67E-07	STAT3	17q21.2	0.316747471	1.50E-09	8.72E-09
TBC1D3	17q12	0.277339865	1.45E-07	7.68E-07	OGFRL1	6q13	0.316693655	1.51E-09	8.77E-09
ZNF449	Xq26.3	0.277268111	1.46E-07	7.73E-07	RPE	2q34	0.316613928	1.53E-09	8.85E-09
EPDR1	7p14.1	0.277267826	1.46E-07	7.73E-07	RERG	12p12.3	0.316525944	1.55E-09	8.95E-09
GPR75	2p16.2	0.277217978	1.47E-07	7.77E-07	DIAPH2	Xq21.33	0.316506012	1.55E-09	8.96E-09
SGMS2	4q25	0.277198634	1.47E-07	7.78E-07	ARHGEF3	3p14.3	0.316353677	1.58E-09	9.13E-09
R3HDM2	12q13.3	0.27717557	1.48E-07	7.80E-07	KDR	4q12	0.31634485	1.58E-09	9.14E-09
TSEN2	3p25.2	0.277149964	1.48E-07	7.82E-07	UBE3C	7q36.3	0.316267566	1.60E-09	9.22E-09
ZNF792	19q13.11	0.277082338	1.49E-07	7.87E-07	UTP14C	13q14.3	0.316027081	1.65E-09	9.49E-09
IARS	9q22.31	0.277080468	1.49E-07	7.87E-07	CPEB4	5q35.2	0.315963015	1.66E-09	9.56E-09
F2R	5q13.3	0.27703975	1.50E-07	7.90E-07	UBE2D1	10q21.1	0.31591392	1.67E-09	9.61E-09
CYFIP1	15q11.2	0.277001595	1.51E-07	7.93E-07	USP1	1p31.3	0.315884996	1.67E-09	9.65E-09
E2F3	6p22.3	0.276968565	1.51E-07	7.96E-07	PPP1R18	6p21.33	0.315785053	1.70E-09	9.76E-09
DHX35	20q11.23-q1	0.276877611	1.53E-07	8.04E-07	RPL13P5	12p13.31	0.315762101	1.70E-09	9.78E-09
PPP1R13B	14q32.33	0.276856947	1.53E-07	8.05E-07	CEP152	15q21.1	0.315762011	1.70E-09	9.78E-09
ZNF568	19q13.12	0.276846127	1.53E-07	8.06E-07	KLRA1P	12p13.2	0.315707309	1.71E-09	9.84E-09
MON2	12q14.1	0.276844988	1.53E-07	8.06E-07	DVL3	3q27.1	0.315678276	1.72E-09	9.88E-09
NUAK1	12q23.3	0.276801993	1.54E-07	8.09E-07	JAKMIP3	10q26.3	0.315620598	1.73E-09	9.94E-09
C2CD6	2q33.1	0.276793044	1.54E-07	8.10E-07	CDSN	6p21.33	0.315590974	1.74E-09	9.98E-09
BLZF1	1q24.2	0.276785478	1.54E-07	8.10E-07	ZNF431	19p12	0.315513127	1.75E-09	1.01E-08
IFT80	3q25.33	0.276633427	1.57E-07	8.23E-07	NTRK2	9q21.33	0.315413149	1.78E-09	1.02E-08
FMN1	15q13.3	0.276512273	1.59E-07	8.34E-07	HS3ST3B1	17p12	0.315396751	1.78E-09	1.02E-08
ZNF17	19q13.43	0.276509566	1.59E-07	8.34E-07	CXORF21	Xp21.2	0.31538366	1.78E-09	1.02E-08
DCLRE1A	10q25.3	0.276471126	1.59E-07	8.37E-07	NFATC2IP	16p11.2	0.315364493	1.79E-09	1.02E-08
CLDN16	3q28	0.276457134	1.60E-07	8.38E-07	PRRC2A	6p21.33	0.315227841	1.82E-09	1.04E-08
ZNF223	19q13.31	0.276417025	1.60E-07	8.41E-07	RCHY1	4q21.1	0.315222408	1.82E-09	1.04E-08
SLC36A1	5q33.1	0.276397256	1.61E-07	8.43E-07	FAM180A	7q33	0.314993317	1.87E-09	1.07E-08
PCDHB17P	5q31.3	0.276365099	1.61E-07	8.46E-07	ZNF143	11p15.4	0.314991792	1.87E-09	1.07E-08
N4BP1	16q12.1	0.276331604	1.62E-07	8.48E-07	GASK1B	4q32.1	0.314808113	1.91E-09	1.09E-08
KIF3C	2p23.3	0.276321068	1.62E-07	8.49E-07	SAMD14	17q21.33	0.314791905	1.92E-09	1.10E-08
B3GALT5-AS1	21q22.2	0.27622722	1.64E-07	8.57E-07	PDGFRB	5q32	0.314777953	1.92E-09	1.10E-08
ADGRF5	6p12.3	0.276204325	1.64E-07	8.59E-07	LINC01622	6p25.3	0.314411752	2.01E-09	1.15E-08
PIBF1	13q21.33-q2	0.27619977	1.64E-07	8.59E-07	C2CD5	12p12.1	0.314395811	2.01E-09	1.15E-08
LPAR4	Xq21.1	0.276199672	1.64E-07	8.59E-07	TNIK	3q26.2-q26	0.314351414	2.03E-09	1.15E-08
DKK1	10q21.1	0.276127534	1.65E-07	8.65E-07	LHFPL2	5q14.1	0.314306403	2.04E-09	1.16E-08
SH2D4A	8p21.3	0.276110361	1.66E-07	8.66E-07	RCN2	15q24.3	0.314303578	2.04E-09	1.16E-08
SOX4	6p22.3	0.276094701	1.66E-07	8.68E-07	NFE2L3	7p15.2	0.314177701	2.07E-09	1.18E-08
CREG2	2q11.2	0.276067219	1.66E-07	8.69E-07	KCNK2	1q41	0.314167185	2.07E-09	1.18E-08
GPAM	10q25.2	0.276014689	1.67E-07	8.74E-07	GRIP1	12q14.3	0.313998585	2.12E-09	1.20E-08
NAA25	12q24.13	0.275968277	1.68E-07	8.78E-07	NPY1R	4q32.2	0.313973585	2.12E-09	1.21E-08
USP16	21q21.3	0.275782342	1.71E-07	8.95E-07	TTC21B	2q24.3	0.313819784	2.16E-09	1.23E-08
ZDHHC5	11q12.1	0.275774085	1.72E-07	8.95E-07	GABRG1	4p12	0.313814493	2.16E-09	1.23E-08
GALNT10	5q33.2	0.275732532	1.72E-07	8.99E-07	ANKRD27	19q13.11	0.313811811	2.17E-09	1.23E-08
SIGLEC6	19q13.41	0.275731457	1.72E-07	8.99E-07	COL5A2	2q32.2	0.313800137	2.17E-09	1.23E-08
CDC42SE2	5q31.1	0.275730804	1.72E-07	8.99E-07	FAM160A1	4q31.3	0.313759997	2.18E-09	1.24E-08
FLNA	Xq28	0.275475678	1.77E-07	9.22E-07	ANO6	12q12	0.313759988	2.18E-09	1.24E-08
KIAA0825	5q15	0.275461397	1.77E-07	9.23E-07	CEP126	11q22.1	0.313672758	2.20E-09	1.25E-08
HCFC1	Xq28	0.275422147	1.78E-07	9.27E-07	HMCN1	1q25.3-q31	0.313608222	2.22E-09	1.26E-08
SLC8A1	2p22.1	0.275403232	1.79E-07	9.29E-07	TMEM159	16p12.3	0.313526787	2.24E-09	1.27E-08
ADAMTSL1	9p22.2-p22	0.275259561	1.81E-07	9.43E-07	CLINT1	5q33.3	0.31334911	2.29E-09	1.30E-08
USP22	17p11.2	0.275243331	1.82E-07	9.44E-07	TRIB2	2p24.3	0.313290333	2.31E-09	1.31E-08
DDB1	11q12.2	0.275235358	1.82E-07	9.45E-07	BMP5	6p12.1	0.313248029	2.32E-09	1.31E-08
SRSF10	1p36.11	0.275224842	1.82E-07	9.46E-07	PRKDC	8q11.21	0.313145237	2.35E-09	1.33E-08
DUSP11	2p13.1	0.275218274	1.82E-07	9.46E-07	XPO1	2p15	0.312959871	2.41E-09	1.36E-08
TMEM65	8q24.13	0.275173305	1.83E-07	9.50E-07	SMURF1	7q22.1	0.312860782	2.43E-09	1.37E-08
CDK2	12q13.2	0.275105232	1.84E-07	9.56E-07	USP47	11p15.3	0.312791471	2.46E-09	1.39E-08
SLC5A4	22q12.3	0.275092883	1.85E-07	9.57E-07	COL27A1	9q32	0.312790452	2.46E-09	1.39E-08
KLHL5	4p14	0.274930972	1.88E-07	9.74E-07	RB1	13q14.2	0.312779796	2.46E-09	1.39E-08
KIF3A	5q31.1	0.274900098	1.88E-07	9.76E-07	PLEKHG5	1p36.31	0.312736636	2.47E-09	1.39E-08
NBPF3	1p36.12	0.274884275	1.89E-07	9.78E-07	RNF122	8p12	0.312725816	2.48E-09	1.40E-08
PIK3R4	3q22.1	0.274857225	1.89E-07	9.80E-07	KIAA1324L	7q21.12	0.312684431	2.49E-09	1.40E-08
SMC1A	Xp11.22	0.274823341	1.90E-07	9.83E-07	SLC25A36	3q23	0.31267257	2.49E-09	1.40E-08
CEP85L	6q22.31	0.274794045	1.90E-07	9.86E-07	BRD4	19p13.12	0.312640679	2.50E-09	1.41E-08
CNNM1	10q24.2	0.274754167	1.91E-07	9.90E-07	TAGAP	6q25.3	0.312639255	2.50E-09	1.41E-08

Supplementary Table 2. RNA-seq results of gene expressions among four human HCC cell lines after scrambled siRNA (siSC), siYAP, siTAZ or siYAP&siTAZ (siYT) treatment. Data are presented as log_FPKM values.

**Cell lines**	**Focus**				**PLC/PRF/5**				**SNU475**				**SNU449**			
**Sample** / **Gene**	siSC	si*YAP*	si*TAZ*	si*YT*	siSC	si*YAP*	si*TAZ*	si*YT*	siSC	si*YAP*	si*TAZ*	si*YT*	siSC	si*YAP*	si*TAZ*	si*YT*
WASH7P	5.31457	4.98619	5.14106	4.62557	4.90983	5.90092	3.81721	6.15995	4.51118	5.2049	5.14044	4.42646	3.86711	3.79831	3.62424	3.83061
MIR6859-1	5.47939	5.64563	5.72343	5.53004	5.79078	6.90827	4.55695	7.38437	4.87217	5.38967	5.70388	3.5852	2.66884	2.66377	3.3085	3.97208
CICP27	-0.37355	-0.3482	-1.27379	-0.48024	-0.9572	-0.7022	-1.78564	-0.15338	-2.54796	-1.20503	-1.26405	-1.43356	-2.35326	-1.70548	-2.49204	-1.84261
WASH9P	5.60291	5.45325	5.66433	5.11295	4.61871	5.76302	3.719	6.00443	5.04305	5.46566	5.38433	4.67161	4.21029	4.15688	4.24261	4.22465
MIR6859-2	4.89604	4.75501	4.83269	3.12847	4.28463	5.41946	3.10821	6.1155	3.74056	3.84878	4.3363	3.4489	3.02636	3.1715	3.59536	3.49032
RPL23AP24	0.38906	0.00728	1.16957	0.16085	2.68607	2.81351	1.54725	3.34631	0.98153	0.10025	1.12984	0.52381	0.44726	0.74303	-0.20112	-0.17316
CICP7	-1.07097	-0.67225	-1.08556	-0.79922	-0.82175	-1.63594	-2.02187	-0.18013	-2.87387	-1.54913	-2.60018	-2.19222	-1.87922	-1.43808	-2.00245	-1.67099
MTND1P23	6.38464	6.32108	6.44059	5.16671	10.2278	12.5788	10.0608	13.6578	5.04794	5.22961	4.20721	5.14989	4.37119	4.37742	4.53646	5.34398
MTCO2P12	6.19523	5.93741	6.16913	4.04242	4.47168	7.64772	3.80897	7.87782	5.4204	5.95146	4.87464	5.95978	2.97388	3.25047	3.46908	4.6929
MTATP8P1	7.08165	6.63541	7.07827	5.66521	8.17868	10.8933	8.43997	11.3591	6.25488	5.96085	5.25415	5.46514	6.39418	6.57326	7.54936	7.77172
MTCO3P12	6.17066	6.06581	6.15049	4.83692	4.61502	7.70421	4.41216	7.96988	5.10451	5.46007	4.33893	5.74869	3.4291	3.31583	4.1243	5.33106
CICP3	-0.96866	-0.36924	-0.44083	-0.43723	-0.14306	-1.36708	-1.85174	0.10789	-2.24989	-1.41748	-1.22601	-2.00087	-1.74595	-0.87773	-1.83084	-1.97566
FAM87B	-0.29067	-0.37496	0.45954	-0.50662	-3.32193	-1.54868	-2.51938	-2.0455	-1.80926	-1.59669	-3.32193	-0.95544	-2.36819	-2.15539	-2.85665	-2.20622
LINC01128	1.88349	1.95973	1.83049	2.03337	0.65928	0.72929	0.84665	0.80521	0.30289	-0.16764	-0.21072	0.21959	0.69178	0.73765	0.71499	0.70979
LINC00115	2.099	1.65198	1.63326	1.26677	0.70197	1.12791	-0.20071	1.13933	-0.96862	-1.8864	-1.82038	-1.90186	-1.24067	-0.42653	-0.59369	0.25419
LINC02593	-3.0747	-2.61757	-3.32193	-1.06624	0.2384	1.38607	-1.39906	1.4097	-3.32193	-2.03385	-3.06656	-1.85097	-3.32193	-3.32193	-2.88157	-2.87321
SAMD11	5.51314	5.13098	4.85755	4.65636	4.29769	5.12865	3.13096	5.37477	3.48098	2.97796	2.85998	1.79629	3.29363	2.85323	2.61164	2.81284
NOC2L	6.885	6.56191	6.23932	5.93413	6.06019	5.87795	5.14305	6.09797	5.70603	5.0043	5.10495	4.14462	5.10221	4.71322	4.69223	4.77428
KLHL17	4.13843	4.01915	4.55958	3.83782	3.00929	4.02295	1.35276	3.96084	2.33413	2.77982	2.82502	1.88656	2.03346	2.56505	1.51102	1.72791
PLEKHN1	3.15284	3.42481	3.66227	3.12864	0.6471	1.97176	0.16722	2.00366	1.95954	2.45659	2.74076	1.87107	1.36075	0.2518	1.34057	0.72299
PERM1	1.83514	1.64504	1.87641	2.19028	-1.04373	0.62652	-0.87746	0.5607	-0.34918	0.28066	0.34946	0.03421	-1.02716	-1.13957	0.10281	-1.36751
HES4	6.16475	6.35191	6.53332	4.94358	5.79745	7.15709	5.03952	7.07525	4.47833	3.62648	3.65687	1.84316	4.50272	4.36729	4.66027	3.44247
ISG15	6.62047	6.79293	6.74518	6.73756	5.66784	6.65316	5.84866	6.36487	4.49859	4.38102	4.96594	4.68912	3.84448	3.33187	3.02212	3.03925
C1orf159	3.77506	3.44846	3.64625	2.85997	2.1482	2.46901	1.02619	2.71238	1.49121	1.28222	1.56632	0.7206	1.62813	1.50146	1.73769	1.82306
SDF4	6.891	6.75125	6.66976	6.41978	6.74714	7.18002	5.76043	7.36275	6.10552	6.02325	5.46827	5.22178	5.40072	5.34442	5.08792	5.57665
B3GALT6	6.06265	6.3421	6.28558	5.89944	4.87164	5.52326	4.43968	5.56286	4.51759	4.71083	4.74385	4.03745	3.2895	3.55999	3.90587	4.14276
C1QTNF12	5.69	5.61034	5.80252	5.30293	-0.57867	0.88725	-1.54669	1.52539	-0.4079	-0.83936	-0.37286	-1.71283	-2.30987	-1.30776	-0.96284	0.26022
UBE2J2	6.30132	6.09888	6.09589	5.65472	4.55628	4.6863	4.2511	4.67849	4.40196	3.79726	4.26127	2.91612	4.01138	3.60111	3.93759	3.52711
SCNN1D	2.35973	2.91968	2.63214	2.19502	0.3179	1.30948	-1.5064	1.83316	-0.86706	0.63258	0.52442	-0.33165	-1.54918	-0.57347	-1.27589	-0.71771
ACAP3	4.4204	4.75637	5.06661	4.43665	3.66195	3.89861	2.37365	4.09925	2.65674	2.84341	2.85911	2.03883	1.96467	2.36218	2.80168	2.79038
PUSL1	5.34582	5.17588	5.44684	4.50681	4.46577	4.76792	3.49158	4.70386	3.74435	3.02829	3.61801	2.14268	3.57279	3.2766	3.47833	2.95192
INTS11	5.51247	5.5145	5.47455	4.99229	4.28068	4.72873	3.6761	4.83976	4.15844	4.10728	4.32367	3.32832	3.44425	3.57115	3.79996	3.93401
MIR6727	6.81172	6.91548	6.80546	5.11973	6.32595	5.3627	4.25153	6.09317	4.28766	3.91344	5.51938	2.52599	2.25616	2.5091	2.79494	3.23599
CPTP	6.2096	6.65648	6.50902	5.58428	5.20323	5.7658	4.24401	5.90468	4.42312	4.54346	4.63185	3.61102	3.71547	4.07744	4.52555	4.67379
TAS1R3	1.46869	1.50614	1.29688	1.00876	0.29088	0.98138	-0.67214	0.94748	-0.94217	-0.6326	-0.35997	-0.55923	-0.30675	-1.14252	-1.39365	-1.64106
DVL1	7.51473	7.47834	7.66067	6.37322	6.1119	6.47778	5.28546	6.3012	5.57347	5.85326	5.87797	4.34598	4.62544	4.29915	5.06766	4.84575
MIR6808	6.70074	6.44646	7.11689	4.90648	4.59236	4.80893	4.00565	5.04251	3.43339	4.43725	4.91074	0.4298	2.87065	2.38805	-3.32193	0.46854
MXRA8	1.92867	2.65101	2.71666	2.57118	1.27539	1.34946	0.26315	1.59978	5.25485	5.28265	6.03038	4.39946	-2.44272	-1.58128	-1.71509	-0.78352
AURKAIP1	8.65345	8.61192	8.42874	7.88643	7.07079	7.71446	6.34775	7.69552	6.69577	6.37682	6.56548	4.75205	6.51923	6.31864	6.19073	6.1271
CCNL2	5.40392	5.22886	5.73414	5.52981	3.55102	5.44123	2.89322	5.10309	2.10473	2.68146	2.94815	2.7477	3.42172	3.80412	2.33755	2.73
MRPL20-AS1	5.23854	5.30877	5.25101	4.99632	4.23419	5.04489	3.78512	5.06989	3.38696	3.5324	3.53146	2.99413	3.92357	4.07042	2.945	2.84377
MRPL20	6.30161	5.99993	5.92796	5.91575	5.13225	5.73672	4.92716	5.70368	4.7334	4.46676	4.65969	3.78347	5.17941	5.06987	4.53087	4.48038
RN7SL657P	3.10038	2.73137	2.93199	2.0401	1.16188	1.94467	1.49561	2.60553	0.03377	-3.32193	-0.62413	-0.22881	1.25261	1.58252	0.44838	1.5575

LINC01770	-3.32193	-1.29657	-1.23737	-2.2167	0.94146	3.6809	0.36942	3.15162	-3.32193	-3.32193	-3.32193	-3.32193	-3.32193	-2.54629	-3.32193	-2.40354
VWA1	1.10656	2.43222	2.4809	1.36515	5.79208	7.37731	4.69187	7.39039	1.82999	3.17861	2.49974	1.94947	1.33625	1.86422	2.89328	3.16709
ATAD3C	2.3497	1.85895	1.8261	1.06765	1.95002	2.12533	0.91506	2.13594	0.613	0.15892	0.6378	-0.878	1.25011	0.71286	0.90343	0.74938
ATAD3B	6.15699	5.8226	5.57055	5.03682	5.60019	5.807	5.01498	5.87779	3.14166	2.6365	3.08642	1.85017	3.98434	3.92632	3.67305	3.9376
ATAD3A	6.94376	6.58172	6.42067	5.80983	6.04469	6.01199	5.34284	6.04381	5.5323	4.75868	5.47506	3.64045	5.46797	5.13752	5.80723	5.32123
TMEM240	0.29256	-0.725	0.13677	-0.43638	-0.67695	-0.06953	-0.80471	0.82565	-1.22817	-0.21884	-0.43269	-1.2519	0.36696	0.26013	0.5812	-0.0319
SSU72	5.4387	5.11134	5.13123	4.92493	4.56011	4.82006	4.26482	4.7276	4.61317	3.684	4.26295	3.81579	4.56904	3.59945	4.79663	4.19198
FNDC10	-3.32193	-2.44567	-2.79486	-3.32193	3.52337	3.72629	2.15163	3.89175	3.90536	4.05413	4.07676	2.54892	-3.32193	-3.32193	-3.32193	-3.32193
MIB2	4.18968	4.60907	4.74148	3.96767	3.23696	4.08282	2.08126	4.26861	2.21668	3.03585	2.94364	2.2805	1.03971	1.34062	1.73497	2.0597
MMP23B	-1.29581	-0.08445	-0.20324	-0.34066	-0.0013	1.04812	-1.94696	1.47531	-1.74446	-1.91795	-1.52448	-1.2389	-1.07551	-0.38388	-1.34593	-0.81986
CDK11B	6.31678	6.22592	6.27607	6.4347	5.31949	6.33666	5.27702	6.52007	5.13527	4.72396	5.29341	4.21882	4.91006	5.53578	5.3972	5.49131
SLC35E2B	3.7235	3.84061	4.06877	3.88463	3.74529	3.27238	3.47174	3.43579	3.57018	3.61169	4.04825	4.17736	3.32624	2.75884	4.01477	3.76423
MMP23A	-0.65698	0.27921	0.71106	0.6277	0.57887	1.67192	-1.13234	2.09606	-1.44963	-1.34241	-0.80002	-0.15874	-1.32577	-1.32963	-3.32193	-0.68662
CDK11A	5.43484	5.29957	5.38072	5.60285	4.37355	5.44351	4.20347	5.80295	4.10421	3.794	4.336	3.27011	4.09117	4.66818	4.47346	4.52319
SLC35E2A	2.38849	2.34031	2.80191	2.03293	2.26484	1.14773	1.6409	1.72066	2.75132	2.75478	3.05358	2.58096	0.98658	0.68125	2.15667	1.59634
NADK	4.8057	4.82741	4.7641	4.80181	4.38387	4.64025	3.83563	4.63577	4.31057	4.07283	4.55006	3.83528	3.98063	3.96952	4.10614	4.00695
TMEM52	0.16671	-0.03741	-0.33604	-0.91521	2.31621	3.21814	1.19003	3.3307	0.891	-0.08141	-0.85215	-0.51446	-1.05454	-0.84655	0.90034	0.36499
PRKCZ	0.32023	0.77277	1.13437	1.05379	1.99459	2.28037	1.62249	2.38231	2.71681	2.7911	2.98225	2.45834	2.32338	2.29753	2.94089	2.80805
PRKCZ-AS1	0.61569	0.85446	1.43206	0.23091	0.60947	1.64711	0.24644	1.76582	0.31565	0.43474	0.62048	0.58519	-0.45171	0.54861	0.37467	0.8853
FAAP20	3.45096	3.89292	3.97114	3.24618	2.83811	4.07956	2.03397	4.1191	3.03108	3.53917	3.345	2.34893	2.01963	2.60351	2.44422	2.5927
SKI	4.67474	4.98281	5.07159	4.43599	3.89991	3.56619	3.81037	3.7829	4.26274	4.33901	4.55091	4.57694	3.90215	4.24971	5.16178	5.30702
MORN1	1.29572	1.37699	1.56706	1.03176	0.30325	0.23004	-0.36333	0.28159	-1.1516	-0.81425	-0.46576	-1.21429	-0.58099	-0.87947	-1.20642	-0.67168
RER1	5.65484	5.61218	5.56753	5.68864	5.1812	5.62749	4.98042	5.59662	4.57472	4.29394	4.17782	4.57902	4.59459	4.38633	4.03004	4.23254
PEX10	4.40726	4.44952	4.31836	4.26568	4.48913	4.99973	3.85773	5.03006	4.12376	4.24514	4.05388	3.84133	3.63696	3.4768	3.67278	3.79831
PLCH2	-2.95684	-2.46752	-2.64655	-3.08099	-2.90366	-2.72785	-3.05698	-2.7238	-2.44475	-0.8234	-2.14063	-2.35443	-0.59077	-0.02576	-0.88927	0.743
PANK4	3.44292	3.06572	3.31968	2.93713	2.37821	3.41776	2.2574	3.16594	2.76445	2.2421	2.82259	1.79544	3.00528	2.95897	2.58277	2.80961
TNFRSF14-AS1	0.50105	1.03186	1.20317	0.90467	-2.80623	-2.30548	-3.32193	-2.39402	-0.02622	0.50291	0.70737	0.70892	-3.32193	-2.33116	-2.64051	-1.42853
TNFRSF14	2.96118	3.49184	3.56733	3.6291	-2.04034	-0.58249	-2.88953	-0.40905	1.79905	2.57548	2.75108	2.51891	-2.74773	-1.4618	-2.11362	-1.02998
PRXL2B	4.70307	4.9913	4.91058	4.32261	3.592	4.00411	2.79818	4.02782	3.77274	4.13889	4.06796	3.62324	4.06595	4.39496	4.47907	4.61339
MMEL1	3.01351	3.34831	3.08271	2.71679	1.16591	2.3509	0.78309	2.63779	1.33185	1.54749	1.6378	1.5986	1.92651	2.15895	2.29276	2.21767
PRDM16	-3.32193	-3.32193	-3.32193	-3.05814	-3.32193	-2.91989	-3.32193	-3.19164	-2.18394	-1.98135	-1.59731	-2.08561	-3.32193	-2.93218	-3.21643	-2.93216
ARHGEF16	2.01233	2.90519	2.86918	2.52003	2.86774	3.59575	1.79073	3.26678	0.67078	1.68759	0.82906	1.27175	-0.15539	1.12445	0.79775	1.19659
MEGF6	4.24279	4.76042	5.54156	4.06826	2.84124	2.18879	1.63951	1.65194	0.1703	1.55525	1.40444	0.36916	2.89576	3.2478	3.04035	3.18512
TPRG1L	4.62429	4.66656	5.22311	5.13949	5.32582	4.97884	5.66643	4.54773	4.31787	3.73078	4.59775	4.4362	5.19267	4.4846	5.67832	4.96747
WRAP73	2.95134	2.60681	2.80831	2.43869	1.71066	2.34022	1.17197	2.02925	1.42564	0.91938	1.35223	1.04559	1.86803	2.104	1.17225	1.46957
TP73	-0.02378	0.99528	1.22855	0.2111	-0.59837	-0.88652	-1.40157	-0.69272	0.69659	-0.76878	0.31183	-1.42671	0.45173	0.75332	1.49429	1.0108
TP73-AS1	1.71666	1.95239	2.18631	1.95542	2.29173	2.14096	2.07389	2.48043	-1.99903	-1.96734	-1.40041	-1.92523	0.92976	1.58472	1.78267	1.75164
CCDC27	-1.0578	-1.10181	-0.75258	-1.17469	2.63E-05	-1.41621	-0.33427	-1.31615	-3.32193	-3.08969	-3.32193	-2.7225	-0.88928	-1.817	-0.27526	-0.54944
SMIM1	-3.32193	-2.386	-3.32193	-2.30257	1.3127	3.42101	0.69492	2.97106	0.30422	1.34025	0.15229	0.49682	-0.7134	0.64218	1.18577	1.8768
LRRC47	5.22473	5.09935	4.96305	4.39034	4.628	4.28738	4.22689	4.31524	4.35021	4.48371	4.5716	3.85335	4.30236	4.37111	4.41276	4.3583
CEP104	2.82711	2.68901	2.72306	2.8561	2.85846	2.48793	2.90779	2.32847	2.23406	1.95325	2.26792	2.45931	2.46252	2.16504	2.9766	2.65153
DFFB	0.85173	0.70545	0.16563	0.05205	1.36179	1.35248	0.78964	1.48307	0.53807	0.92487	0.65541	0.69684	1.00079	1.18657	0.46767	0.91145
C1orf174	3.4425	3.01092	3.09149	3.07528	3.13976	3.16799	3.07965	3.25169	2.91108	2.22372	2.48934	2.21537	3.16484	2.77066	2.58747	2.26148
LINC01134	-0.34463	-0.70265	-0.49232	0.2151	0.04648	-0.59955	-0.61569	-0.63229	-3.32193	-2.68736	-3.32193	-3.08187	-2.00222	-2.64717	-3.32193	-2.00525
AJAP1	-3.07376	-2.88778	-3.1225	-2.92585	-0.6512	-1.24258	-0.77416	-1.92295	2.42166	2.08978	2.7823	1.20934	-3.32193	-3.32193	-3.23823	-3.32193
NPHP4	-3.32193	-3.2295	-3.22454	-3.03123	0.49239	0.92587	-0.14671	0.95893	1.11712	1.03134	1.23088	0.84697	-2.2146	-2.48824	-1.92575	-1.87533
KCNAB2	5.99051	5.64272	5.54599	5.18004	-1.91076	-1.46044	-1.8637	-1.18808	3.59897	3.53314	3.42866	3.10248	-2.17675	-2.72892	-2.76972	-2.47842
CHD5	-2.76528	-3.32193	-3.07589	-3.06114	-2.76945	-2.54448	-3.03534	-2.96576	-2.45724	-1.68091	-2.91574	-2.09598	0.76424	2.64116	2.51016	3.73447
RNF207	3.62871	3.52398	3.69135	3.28415	3.35705	3.71424	3.23212	3.49298	2.65986	2.76189	2.66984	2.43192	3.04658	2.99563	2.26415	2.3152
ICMT	5.53109	5.48915	5.46603	5.32442	5.58816	5.17741	5.74895	5.16806	4.75579	3.81844	4.13118	4.14871	4.97352	4.9472	5.09623	5.06725

LINC00337	-0.33841	0.76779	0.96742	0.88316	0.61168	0.97293	0.2325	0.80203	-0.78128	-1.00074	-0.91746	-1.65142	-1.65967	-0.64532	-3.32193	-0.98696
GPR153	2.44433	2.8578	2.93045	2.22047	1.47136	2.49479	0.36636	2.54139	1.91404	3.08387	2.80607	2.61357	0.09519	0.7427	1.96124	2.59393
ACOT7	6.74546	6.8094	6.65742	6.60779	4.47405	4.34531	3.64615	4.37267	4.78987	4.04329	3.8529	3.50332	4.81091	4.43055	4.60199	4.4309
HES2	3.33901	4.09296	4.05566	4.14527	-3.07962	-2.95427	-2.9794	-2.89813	-2.32116	-2.68626	-2.29273	-2.69497	-2.67829	-2.68014	-0.96822	-1.34315
ESPN	-0.46197	0.29773	0.58861	0.33017	1.19448	1.33913	0.08402	1.47806	-2.49037	-0.88376	-1.40383	-1.08651	-1.57152	-1.95975	-0.49748	-0.38566
TNFRSF25	2.59047	3.07685	3.62375	3.31147	-1.22395	0.48733	-1.90825	0.76737	-0.72129	0.90802	0.74856	0.62852	0.20826	-0.2744	-0.58341	1.05086
PLEKHG5	2.82082	3.39226	3.85075	3.31659	-0.41577	0.97758	-0.94026	0.41176	1.49141	2.2728	2.20252	2.08456	1.24849	0.50883	0.54132	1.77776
NOL9	3.71434	3.40265	2.98493	2.73466	2.95117	2.61229	2.6088	2.83289	2.87019	2.58018	2.66695	3.04995	2.9235	2.53166	2.49244	2.81289
ZBTB48	2.92553	2.72248	2.95175	3.04222	2.24739	3.35646	1.72317	3.70212	1.36973	1.73307	1.84066	1.44057	1.3849	1.64493	1.63756	1.69834
KLHL21	5.40937	5.22794	5.2273	4.79182	3.0731	2.42658	2.56203	2.53567	2.13778	2.52905	2.32335	2.31083	3.3858	3.31477	3.81231	3.81355
PHF13	3.97293	4.24499	4.38079	4.31878	3.58613	3.35116	3.47016	3.43072	3.51072	3.46181	3.42747	3.45857	3.57016	3.74052	4.3499	3.98553
THAP3	4.63459	4.31386	4.3505	4.30613	3.49038	4.5086	3.11989	4.68339	3.38795	3.3308	3.45413	3.15372	3.76855	3.50253	3.3607	3.37912
DNAJC11	4.78816	4.56466	4.4634	4.46931	4.03929	4.45378	3.83894	4.66666	3.60088	3.43549	3.75632	3.58824	4.3831	4.07441	4.15362	4.06427
CAMTA1	3.55648	3.74154	3.83291	4.35481	1.91196	2.76946	2.05843	2.6931	2.06116	1.62471	1.99227	1.25581	2.18725	2.43511	2.31027	1.62379
VAMP3	5.28817	5.51436	5.52589	5.83122	4.97114	5.05781	5.47785	4.91177	5.3549	5.2086	5.20752	6.21559	5.9466	5.98161	5.77157	5.61635
PER3	-0.02675	-0.3042	0.54831	0.17413	0.93134	0.72268	1.08515	1.33787	-0.11361	-0.3345	-0.56537	0.11254	1.94064	1.86627	1.98253	1.71761
UTS2	-0.90686	-0.89499	-0.11805	-0.51095	-0.22671	0.58694	0.56102	0.80683	-0.40581	-1.34919	-1.43694	-0.85755	1.59438	1.68357	1.22291	1.11912
TNFRSF9	3.46403	3.67187	3.81472	3.40077	0.9506	0.58555	2.00898	0.40578	0.27674	1.17843	0.56974	2.10278	-2.21592	-0.02852	-1.5817	-0.24859
PARK7	6.93464	6.9272	6.85828	6.84446	6.96043	7.60164	6.8227	7.56782	6.92184	6.79527	6.77012	5.80249	6.86803	6.99968	6.47285	6.55906
ERRFI1	7.70005	8.06201	8.08894	7.61529	4.80365	5.29639	5.06916	5.2075	5.23922	6.10976	3.72658	6.44671	6.16994	5.70412	5.48705	6.27282
SLC45A1	-2.46895	-1.99529	-1.94791	-2.57474	-3.0253	-3.32193	-3.32193	-2.92178	-0.98255	-0.95839	-0.54659	-2.30825	-1.26601	-0.72859	-0.70567	0.16709
RERE	3.21562	3.26251	3.23211	2.75354	3.49403	4.06392	3.16776	3.95794	3.69182	3.6136	3.77702	3.12044	3.03061	3.28529	3.96873	4.17382
MIR6728	5.61279	5.49583	5.90741	4.53719	4.50412	3.91542	4.87795	2.87981	5.82942	6.42413	5.50046	5.40127	4.88151	4.621	4.49696	4.09761
ENO1-AS1	0.84962	2.03975	1.54744	0.68171	2.67555	1.42293	2.6904	1.21837	1.79211	2.42008	2.11491	1.23812	-0.38326	1.06556	1.14568	2.14354
GPR157	2.88956	2.89643	3.10261	2.71692	5.1653	5.11647	5.16014	4.77004	1.3324	1.74847	1.15398	2.21634	2.07723	1.93426	2.421	2.24872
MIR34AHG	0.24692	0.17519	0.64934	0.202	-0.18638	-0.74214	-0.87892	-0.67789	-0.30313	-0.14836	0.27278	0.02501	-2.02138	-1.25804	-2.22785	-1.26945
LNCTAM34A	-2.05833	-1.46566	-1.21108	-2.65334	-3.32193	-3.32193	-3.32193	-3.32193	0.543	-0.64302	-0.36352	-0.38828	-2.25323	-2.87351	-1.27895	-0.35188
H6PD	3.01529	3.49419	3.82447	3.21195	3.93204	4.54656	3.20186	4.65001	2.01405	2.98706	2.45256	3.39608	1.29567	2.32222	2.64642	3.40576
SPSB1	4.87023	4.86565	4.72342	4.1912	4.03067	3.57646	3.70998	3.58926	2.54254	2.83989	2.72703	2.65159	3.6356	3.77821	4.05547	4.42848
SLC25A33	3.08705	3.07733	2.5947	2.9451	3.69255	4.10119	3.86162	4.02183	1.40677	0.74239	1.24728	0.83187	3.08631	3.22883	2.54815	2.55042
TMEM201	3.6232	3.05155	2.84828	2.1249	2.88685	2.09357	2.09437	2.29585	2.26334	1.39809	1.86984	0.75217	2.65673	2.26725	3.09885	2.67209
PIK3CD	4.18908	4.22179	4.27897	3.89652	2.44111	2.86549	2.42612	2.91084	5.20668	4.57277	5.2242	4.66917	3.23194	2.9263	3.50561	3.15437
PIK3CD-AS2	-2.45281	-0.60685	-1.02418	-2.29094	2.75909	4.18197	1.52481	3.91102	1.4803	1.78556	0.98822	1.09575	0.60841	1.73385	1.80459	1.13562
CLSTN1	5.77577	5.54693	5.84247	5.46533	5.42905	5.12032	5.18927	5.22164	5.98508	5.62097	5.95896	5.63613	5.15752	4.69839	5.47967	5.59975
CTNNBIP1	2.89179	3.06078	2.96543	2.71272	2.85152	3.01203	2.36	2.78104	4.22407	3.52186	3.82399	3.4194	2.84838	2.5623	3.45313	3.27723
LZIC	2.72249	2.86216	2.9514	2.62448	3.83598	3.28294	3.91028	3.189	3.71283	3.19437	2.83798	3.81859	4.14613	4.06455	3.53732	3.66425
NMNAT1	1.35502	1.40654	1.35236	2.07104	0.90553	1.58195	0.78128	1.52777	0.57473	0.74394	0.36285	1.11026	1.38184	1.19837	1.13066	0.85491
RBP7	-3.32193	-2.31347	-2.27374	-2.2257	1.64358	0.36156	1.3449	1.02139	4.63386	4.54816	3.96359	3.9284	4.38769	4.2232	3.65296	3.24981
UBE4B	3.39681	3.3228	3.10126	3.68629	3.36881	2.79735	3.5625	2.74063	3.54634	3.17191	3.29505	3.91653	3.34081	3.31181	3.68041	3.81058
KIF1B	2.3853	2.46658	2.48551	2.7266	2.78442	2.17759	3.06255	2.23426	3.19085	3.12925	2.94995	3.84633	3.18539	3.47595	3.64324	3.9641
RN7SL731P	3.34565	4.14089	3.42892	4.53439	1.23265	2.76458	3.12994	3.25577	2.41527	2.15131	2.68063	4.16422	4.50587	4.0205	4.41574	4.32222
PGD	6.65524	6.62963	6.53787	6.79776	7.30011	7.34963	7.11624	7.21096	6.68389	6.38275	6.39089	6.43423	6.58002	6.69465	6.90982	6.97659
CENPS-CORT	3.98836	3.79908	3.61703	3.62356	3.67709	3.76163	3.57093	3.81627	3.56056	3.20764	3.35105	2.62428	3.3629	3.51599	2.82121	2.99352
CENPS	4.3729	4.11954	4.0203	4.00907	4.03405	4.16746	3.94774	4.19019	3.93054	3.56145	3.71568	2.92386	3.76515	3.87069	3.19788	3.30099
CORT	1.6425	2.09183	1.34546	1.33786	0.74432	1.65788	0.9223	2.17357	1.01152	0.29387	0.60694	0.5532	1.58768	1.6083	0.68548	1.07777
DFFA	5.03922	4.72803	4.49877	4.5541	4.19796	4.4327	4.20615	4.49645	4.37807	4.17101	4.43961	4.39989	4.37379	3.81184	4.21179	3.85226
PEX14	4.20937	4.04224	4.02497	3.72445	3.64044	4.13983	3.3925	3.89374	4.63573	4.39167	4.74763	3.91977	4.16552	4.02175	4.45592	4.18237
CASZ1	0.73206	0.55371	0.16609	-0.98215	1.38049	0.72299	1.01695	0.87541	0.30802	-0.0615	-0.39668	-0.95626	0.0246	0.89819	1.48862	0.98363
TARDBP	5.19825	5.08041	5.03092	5.01547	4.98317	4.5222	5.27752	4.40531	5.27999	4.4566	4.98516	4.70037	5.35518	5.2038	5.33202	5.03005
MASP2	-1.41943	-1.22474	-1.56521	-1.32328	-1.34135	-0.65443	-1.63689	-0.11218	-1.67938	-1.68378	-1.69877	-0.77443	-0.77026	-0.45977	-0.42477	-0.6974

SRM	7.18741	6.46981	6.45352	5.68547	6.91647	7.87866	6.29323	7.65711	7.42242	5.90015	7.21381	5.58886	6.23679	4.72264	6.09721	4.7674
EXOSC10	4.45444	4.1635	4.35016	4.30743	3.9066	4.06191	4.28316	3.94061	4.56828	3.90434	4.5838	4.4708	4.1227	3.69129	3.96041	3.44459
EXOSC10-AS1	3.29301	3.04022	3.3535	3.1496	3.84471	2.16738	4.71816	2.13935	4.12397	3.51222	4.31472	3.50538	4.31023	3.8077	4.64296	3.78727
MTOR	3.40551	3.34121	3.15398	3.50202	3.74886	3.02079	3.7071	3.11084	4.41583	3.89331	4.20143	4.32813	3.57173	3.4058	4.14893	4.07672
MTOR-AS1	2.22571	2.18671	2.22631	2.76073	3.40569	2.17708	3.17397	1.18761	4.0679	3.43183	3.69552	3.75529	3.02657	2.74216	3.72364	3.58637
UBIAD1	2.79849	2.69523	2.462	2.40747	3.44445	3.25782	3.32096	3.07372	2.79744	2.63838	2.78058	2.35721	3.0461	2.7219	2.91664	2.81048
FBXO2	0.75738	2.00061	2.09496	2.8099	4.57048	5.02905	3.67847	4.61961	1.32378	2.79402	2.58115	1.4131	-0.09452	0.89211	1.35118	2.02459
FBXO44	2.91834	2.93693	3.01854	2.88326	4.00226	4.12916	3.08415	3.9941	3.32541	3.59815	3.60936	2.66862	3.00542	2.27022	2.61582	2.7145
FBXO6	1.92053	2.45612	1.74392	3.05717	3.89523	3.54464	2.89154	3.7825	2.79171	2.78187	2.6487	2.01465	2.89127	2.21719	1.80574	2.44709
MAD2L2	5.07764	5.24875	5.21381	5.09731	4.28368	4.76686	3.47049	4.617	5.12151	4.38883	4.61362	2.9434	4.57883	4.25081	4.51198	4.14303
DRAXIN	1.84683	3.96557	3.54264	4.98868	-3.32193	-3.32193	-3.18624	-3.15115	-3.15482	-2.93717	-3.17208	-2.05757	-2.74022	-2.59091	-2.73278	-1.91613
AGTRAP	6.22212	6.26843	6.30002	5.77033	5.22943	6.13258	4.14918	5.91232	4.86339	4.81092	4.80911	4.22131	4.67638	4.75473	4.56771	4.82649
C1orf167	-0.29242	0.38555	0.23298	0.58693	1.46875	1.31657	0.98834	1.21154	0.7974	1.72307	1.41189	1.49998	-1.29074	0.10951	-0.19212	0.58349
MTHFR	0.60929	1.12913	1.27856	1.76403	2.31828	2.36853	1.96955	2.37691	1.55921	2.39706	2.25048	2.61781	0.15737	1.37781	1.0434	1.77118
CLCN6	1.54548	1.46563	1.80436	1.42455	2.17581	3.35052	1.74306	3.48328	1.83136	2.66088	2.14108	2.64158	1.6752	3.14427	2.19379	2.91762
NPPA	0.09782	-0.18806	0.00442	0.30712	-1.57585	1.73246	-1.76585	2.18612	-1.24404	-1.44501	-2.54131	-1.75109	-0.41222	-0.05061	-2.58935	-0.03045
NPPB	-2.239	-1.51028	-3.32193	-3.32193	-2.76125	-1.61643	-2.29061	-1.00276	5.65924	5.03929	3.29742	2.18673	-3.32193	-3.32193	-3.32193	-3.32193
KIAA2013	6.02886	6.24909	6.18579	5.39322	6.35079	6.44951	5.57743	6.42764	6.15944	6.2671	6.24265	5.46921	4.96418	4.72625	5.46623	5.42748
MFN2	5.78189	5.59729	5.43355	5.52002	5.40604	4.69836	5.15212	4.64664	5.40118	5.18882	5.35199	5.32201	4.99002	5.02052	5.29055	5.35138
MIIP	4.35855	4.07884	4.27768	3.2655	4.04892	5.11704	2.81432	5.07777	4.44928	4.10755	4.59253	2.89315	3.35478	3.22599	3.53995	3.23927
TNFRSF8	2.7844	1.85558	1.53815	1.00965	-2.88606	-2.44245	-2.84931	-2.74283	-3.01003	-2.47324	-2.2685	-2.48412	-3.32193	-3.32193	-3.32193	-3.32193
MIR7846	5.28193	5.90664	5.91747	5.91445	1.11112	-3.32193	-0.2266	-3.32193	3.82739	4.85524	3.75491	4.08416	-3.32193	-3.32193	-3.32193	0.74665
TNFRSF1B	5.33519	5.98769	5.63553	5.90476	-0.32156	-0.34444	-0.64694	-1.53483	2.63978	3.75215	2.96478	3.29423	-2.75209	-2.3472	-2.84319	-2.83421
VPS13D	0.99671	1.06228	1.09688	1.46312	1.71897	0.96622	2.05769	0.93498	2.09949	1.81866	2.05645	2.9645	1.69596	1.79653	2.31973	2.57513
DHRS3	4.68454	4.56769	4.80546	5.1287	5.76429	6.8172	4.62935	6.68684	4.01309	4.74003	4.17434	5.35441	4.25596	4.59139	5.16711	6.0104
MIR6730	3.39582	2.98205	3.27896	2.88982	3.44935	4.98745	1.74152	5.27346	2.45572	2.5072	2.60883	2.74177	0.93737	1.49203	2.17756	3.77243
LRRC38	1.07222	1.86757	1.7782	0.4712	-3.12128	-3.32193	-3.32193	-3.32193	-3.32193	-3.32193	-3.32193	-3.32193	-2.68919	-3.32193	-3.32193	-3.32193
PDPN	2.03524	2.55602	2.68371	2.45032	-3.08579	-3.04491	-3.0644	-3.32193	-3.32193	-3.32193	-3.32193	-3.32193	-3.32193	-3.32193	-3.32193	-3.32193
PRDM2	0.95099	0.85969	0.69923	1.01403	1.62362	0.52229	1.70063	0.74878	2.2678	1.4852	1.6061	2.41879	1.81926	0.97764	1.63786	1.32542
KAZN	0.31489	-0.23623	-0.26751	-0.27035	-2.66973	-1.7722	-2.87156	-1.94304	1.76192	1.5392	1.44043	1.12332	-2.48137	-2.98548	-2.60609	-2.45735
KAZN-AS1	-1.30486	-3.32193	-3.32193	-3.32193	-2.80446	-3.32193	-3.32193	-3.32193	3.72322	3.82845	3.51924	2.85329	-3.32193	-3.32193	-3.32193	-3.32193
TMEM51-AS1	-2.91274	-2.74587	-2.04421	-2.68781	-2.86627	-1.74206	-2.93665	-1.82611	-2.31009	-1.09254	-1.95156	-0.90175	-2.89695	-2.89826	-2.36639	-2.70522
TMEM51	4.81761	4.75758	5.0964	4.62793	3.24392	4.18678	3.16378	3.87258	4.33604	4.41613	4.21328	4.23438	4.10896	3.99792	4.10178	4.17696
FHAD1	-1.79639	-1.68551	-1.39575	-1.8979	-3.18054	-3.07845	-3.09575	-3.2215	1.43654	1.71348	1.6182	1.78172	-3.07104	-2.9092	-3.09098	-3.08627
EFHD2	7.40063	7.34232	7.28024	6.09236	5.05505	4.82343	4.43463	4.82611	8.96742	8.85584	8.7094	7.47768	5.64307	5.50888	5.48374	5.50194
CELA2B	-1.25287	-3.32193	-1.50458	-0.99	-0.05936	0.58526	-1.32794	0.63899	-2.62667	-0.83059	-1.41153	-0.98855	-2.15075	-1.35445	-1.13287	-1.27965
CASP9	1.3726	1.27179	1.28595	1.6805	3.46323	3.71167	3.08061	3.80124	1.54846	2.09626	1.8396	2.02352	0.64668	0.59869	0.96818	1.10805
DNAJC16	1.63652	1.38557	1.69876	1.88917	3.21544	2.98994	3.32904	3.17756	2.06251	1.53882	2.58638	2.64037	2.28021	1.40829	2.15749	1.17514
AGMAT	2.28715	1.94981	0.81154	1.48758	5.94812	5.52522	5.53899	5.72023	2.36511	2.4246	2.16151	1.42754	-2.75008	-3.00886	-2.94742	-2.38921
CHCHD2P6	2.6669	2.87	3.22598	1.76036	2.22402	3.06193	1.66874	2.27794	1.64491	2.20808	1.95503	-0.1156	0.40515	1.18184	0.6708	0.70039
DDI2	1.30977	1.6224	1.39121	1.87515	2.37722	1.40469	2.38621	1.97588	1.8425	1.96758	1.78477	2.70091	2.3562	2.32982	2.05247	2.36075
RSC1A1	2.04221	1.76087	1.85648	2.14842	3.19649	1.819	2.90406	1.34469	2.97918	2.51376	2.72355	3.72203	2.9526	2.58068	2.9362	2.88995
PLEKHM2	6.01615	6.02473	6.1473	5.30587	4.76854	5.1739	4.11617	5.10567	6.05056	6.08488	6.21829	5.8523	4.39954	4.43419	5.17351	5.04877
SLC25A34	0.70521	1.28972	0.67492	0.2678	-2.29925	-0.95028	-2.39063	-0.28223	-1.8921	-1.79889	-1.99431	-2.37997	-1.78434	-1.30969	-1.69557	-2.35977
SLC25A34-AS1	2.47655	3.32938	2.80879	1.91835	-1.70681	-1.09415	-3.32193	-1.36242	-1.38701	-0.31701	-1.06652	-3.32193	-1.78434	-1.37915	-3.32193	-3.32193
TMEM82	-0.97572	-0.69948	-0.77611	-1.48263	2.70943	2.84253	1.78088	2.93183	-3.32193	-3.32193	-2.70938	-2.34512	-3.32193	-3.32193	-3.32193	-3.32193
FBLIM1	4.87178	5.31855	5.62612	5.5632	4.93093	4.46471	5.01339	4.12941	4.6141	5.1259	5.26014	5.53329	4.64365	4.18395	5.60068	5.14632
RPL12P14	5.5638	5.0747	5.07423	4.34308	4.43022	3.72595	3.92416	3.55831	4.51913	3.53048	3.97159	2.71296	1.62205	0.27837	0.21235	0.03902
UQCRHL	2.47173	2.51962	2.13822	1.79526	0.87879	1.70826	1.06622	1.00399	1.64196	2.1459	1.29889	0.27949	0.65832	0.77564	-0.18149	0.6408
SPEN	3.20281	3.03433	2.95414	2.89112	3.44732	2.29073	3.30001	2.34646	3.91425	3.62909	3.70649	3.91475	3.51292	3.25927	3.75784	4.11791

ZBTB17	3.8963	3.71433	3.82078	3.53988	2.6347	3.61799	1.8762	3.77917	3.86685	3.99715	3.81898	3.38196	2.44599	2.71028	2.48223	2.77656
FAM131C	-0.62698	0.51755	0.48268	-2.21625	-0.29161	1.65294	-1.18205	1.81516	3.42762	4.19498	3.18281	2.72932	1.95942	2.4631	1.95975	2.74198
ARHGEF19-AS1	1.63482	1.87649	1.49716	1.68911	2.24952	3.17593	1.76575	3.08936	3.85646	3.85004	4.08317	3.05525	-2.06791	0.26794	-0.36741	0.5658
ARHGEF19	0.46978	0.53519	0.56065	0.43913	1.509	1.99147	0.96872	1.81815	3.53786	3.37183	3.66945	3.02072	-1.6113	-0.21697	-1.01238	-0.34444
ANO7L1	-1.14877	-0.40235	-1.40647	-0.75548	0.44239	0.77968	-0.33698	0.43561	0.75417	0.78003	0.8776	0.13523	-2.63389	-2.63584	-1.62853	-1.30587
CPLANE2	1.93257	1.93406	1.89931	0.80638	2.24173	1.15912	1.81682	1.85806	2.28925	2.81292	2.94444	2.06884	1.04806	1.5282	2.27744	2.73746
FBXO42	1.50515	1.44256	1.45447	1.36492	1.55392	1.84902	1.43974	1.87805	2.53194	2.17211	2.26525	2.61339	1.70516	1.41287	1.57061	1.48426
SZRD1	5.38459	4.85447	5.11664	4.66935	4.9912	4.34345	4.86506	4.01981	6.37828	5.33113	6.24495	4.82949	5.99371	4.77546	6.15829	4.91606
SPATA21	3.4955	2.86242	3.13968	2.76492	3.29815	2.29981	3.41455	1.95978	4.78703	3.61173	4.63093	3.86625	4.54451	3.38774	4.52094	3.29256
NECAP2	4.26823	4.4527	4.59592	4.54737	3.20563	3.06314	3.01824	2.9049	4.17769	4.41762	4.20924	4.20303	4.19984	4.21856	3.95505	4.11789
LINC01772	-0.62126	-0.65086	0.1636	0.31887	-1.8747	0.30634	-1.84371	0.87602	-1.63305	-0.42318	-1.24434	-0.09092	-1.25657	-0.7724	-1.4188	-0.36426
CROCCP3	-0.46087	-0.02584	0.65175	-0.0546	-0.52473	0.78578	-0.7375	1.40452	-1.08695	-0.59894	-0.22554	-0.26127	-0.87242	-0.3996	-0.49599	-0.49122
NBPF1	2.27913	2.07096	2.04025	2.09761	2.92362	2.03451	3.06825	2.2613	2.18495	2.42341	2.29459	3.25596	2.90186	3.31514	3.63362	3.86301
CROCCP2	3.00343	1.96034	2.25039	1.80347	3.64663	4.51831	2.71118	4.51353	2.46126	2.55269	2.57673	2.00871	1.16136	1.3401	0.50772	1.14249
MST1P2	-0.8879	-1.17719	-0.14769	-0.70558	2.23391	5.63509	0.1935	4.51258	-1.57234	-0.23217	0.11456	-0.25386	-1.94764	-0.93476	-2.91763	-1.2156
EIF1AXP1	4.37828	4.55644	3.97653	4.17942	5.18271	4.02994	6.03456	4.18095	4.83349	4.09869	3.745	3.95343	5.07838	5.13173	5.01325	4.65376
CROCC	2.72449	2.19158	2.59188	1.78052	2.87104	3.40238	2.01596	3.24109	3.1549	3.20212	3.36278	2.70477	0.94352	0.71079	1.31071	1.32142
MST1L	-2.51527	-1.99221	-0.97634	-2.22778	0.85738	4.4195	-1.06608	3.4509	-2.55926	-1.20988	-1.72844	-1.6755	-2.2017	-0.90843	-2.12818	-1.93879
MFAP2	1.36521	1.55532	1.91036	2.23413	-2.67541	-1.06831	-2.7398	-1.37702	5.36236	5.52697	5.64373	4.61069	-3.32193	-3.32193	-3.32193	-3.32193
ATP13A2	4.97306	5.11242	4.94823	4.47143	5.25176	5.61099	4.38144	5.46512	5.9798	5.82104	5.94303	5.39545	4.62151	4.85068	5.86269	5.71135
SDHB	5.70711	5.60632	5.54679	5.376	4.86755	5.38178	4.79953	5.13731	6.01406	5.48497	5.7413	5.22712	5.18994	5.20034	4.62055	4.47769
PADI2	-3.32193	-3.12586	-3.32193	-2.45905	-2.41017	-2.98591	-2.34481	-2.7732	0.26367	1.47325	0.65806	1.26258	6.881	6.44598	8.22962	7.51833
PADI1	-1.6366	-1.1924	-1.54037	-1.47481	-2.90385	-2.75619	-2.65196	-3.07306	-0.22705	0.41837	0.07191	0.00055	0.44883	0.96611	0.50065	0.94321
PADI3	-2.34117	-0.69196	-1.26131	0.30658	-2.82271	-3.00454	-3.32193	-2.66297	0.6517	2.00692	1.33363	2.15723	5.5323	5.91152	6.37233	7.57799
RCC2	5.53173	5.30752	5.36358	5.0635	4.86372	4.71847	4.88573	4.65552	5.98237	6.1605	6.52292	6.35353	6.12829	6.25354	6.54394	6.39402
ARHGEF10L	2.66565	2.76973	2.9534	2.19365	3.35275	4.67512	2.3227	4.55929	2.47774	2.66502	2.8597	2.25068	0.25912	0.57276	1.73248	1.75897
KLHDC7A	-3.11923	-3.32193	-3.08471	-3.32193	1.25143	-1.821	0.29452	-0.73125	-0.16969	-1.52579	-0.81398	-1.42776	1.30767	1.51971	1.34142	0.49586
ALDH4A1	2.2478	2.129	1.81342	1.97253	4.18113	5.51701	3.48993	5.41415	3.86563	4.05081	4.10509	3.43699	3.48235	3.672	3.46562	3.75561
IFFO2	2.83517	2.55381	2.57399	1.88526	2.01691	1.57465	2.22579	1.33154	2.80948	2.93261	3.46977	3.11054	4.09915	3.57029	4.62902	4.16259
UBR4	4.23887	4.28771	4.32642	4.36713	3.91459	3.28757	4.22653	3.24189	4.19058	3.96898	4.2153	4.86373	3.58223	3.45453	4.28959	4.38131
EMC1-AS1	4.50818	4.41991	4.70234	4.01254	5.78913	3.72857	5.40586	3.33831	5.74573	5.26049	5.75537	5.12034	4.56838	4.20035	4.8453	4.98959
EMC1	4.46031	4.35913	4.41232	4.1869	4.61504	4.245	4.58106	4.35649	4.64219	4.19369	4.65049	4.51002	3.82877	3.3279	3.95497	3.85202
MRTO4	6.2339	5.78178	5.52895	5.3366	5.1551	5.5953	5.02683	5.61483	5.73244	5.13696	5.41692	4.74405	6.07167	5.70319	5.56012	5.19802
AKR7L	-1.19605	-0.45517	-0.58919	-0.8361	-1.61592	0.05037	-2.28848	0.70849	-2.91494	-2.69829	-2.20965	-1.80558	-1.54948	-1.98155	-2.70331	-2.07819
AKR7A3	0.71648	0.56116	0.41872	0.44417	-1.10083	0.80903	-1.68116	1.17591	-1.6849	-1.01771	-0.81051	-1.87775	-0.58009	-0.30647	-1.47393	-0.15642
AKR7A2	5.35978	5.75753	5.64157	5.6829	4.89299	5.16853	4.28262	5.01984	5.56557	5.68276	5.37336	5.12109	4.25928	4.65372	4.45022	4.70035
CAPZB	6.67875	6.63008	6.80091	6.59613	6.23357	6.49512	6.17504	6.53446	7.44069	7.19111	7.18593	7.30378	6.93171	6.87575	7.29564	7.31663
NBL1	5.22474	5.30177	5.28058	4.50775	4.43544	4.49461	3.75287	4.5409	5.23531	5.40643	5.27468	4.5817	6.16734	6.00689	6.52345	6.84725
TMCO4	1.93133	1.54346	1.60811	1.10721	2.3157	2.3099	1.7141	2.04629	3.33507	3.02007	3.65699	2.91477	-0.6782	-1.91924	-0.58505	-0.59842
OTUD3	1.22435	0.52487	0.77985	0.8777	1.10807	0.94156	1.51167	1.54785	1.60069	0.68178	1.38237	0.69794	2.09699	1.23648	1.43624	0.72748
CAMK2N1	1.73772	2.52685	2.20187	2.40816	5.45042	6.08318	5.56063	6.21535	1.66464	2.26972	0.98223	2.77073	-2.20917	-1.59209	-1.74489	-0.33076
MUL1	4.33993	4.11158	4.09353	4.0157	4.98318	4.1977	4.63016	3.83018	4.84871	4.10936	4.85085	4.1936	3.93436	3.05757	4.15785	3.46404
FAM43B	0.3863	1.15948	1.21194	0.35717	-1.48877	-2.93777	-2.05387	-1.66498	-0.45136	0.49511	-0.34424	-1.17372	-2.54859	-3.32193	-2.95668	-2.94956
CDA	8.33017	8.54328	8.65578	8.15728	3.42101	4.84716	2.95855	4.86993	7.35294	7.17877	7.50269	5.91604	-3.32193	-2.14669	-3.32193	-2.4848
PINK1	4.72707	4.92763	5.0079	4.70786	4.18017	4.41772	3.93596	4.1946	5.59905	5.53713	6.06576	5.22223	2.47097	2.09065	2.9984	3.21619
MIR6084	6.83796	7.41917	7.38612	6.85663	7.05363	6.5477	7.07803	6.27253	8.61815	8.31213	8.84042	7.75586	4.3995	4.35609	5.54167	5.87346
PINK1-AS	5.31491	5.45748	5.53783	5.38011	4.89857	6.32005	4.72609	6.22083	5.4838	5.47145	5.8446	5.29271	4.04395	3.90996	3.72344	3.93278
KIF17	-0.60908	-0.58823	-0.39926	-0.00191	0.82694	1.37453	0.83637	1.18169	-0.04719	1.40323	1.29647	1.20516	-0.76197	-1.59763	-1.38906	-2.4366
SH2D5	3.17779	2.53894	2.14537	1.06603	2.6139	2.30553	2.43918	2.28054	1.8594	1.96614	1.94286	0.58239	3.39133	3.30258	3.61031	3.17063
HP1BP3	3.44546	3.21731	3.66423	3.68304	3.91664	3.61933	4.11687	3.83448	5.02839	4.71795	4.7903	5.03496	5.11522	4.94641	4.70696	4.45551

EIF4G3	3.58854	3.6984	3.87238	3.98588	3.97947	3.51216	4.33554	3.08715	3.49903	2.88297	3.68958	3.74364	3.2599	2.25274	3.6306	2.83759
ECE1	5.0366	4.94018	5.13942	4.57587	4.53257	4.25546	4.01122	4.38195	6.2972	6.49946	6.38848	6.29976	4.19996	4.3842	5.09107	5.08978
HS6ST1P1	1.24136	1.10246	0.85901	-1.25983	1.12665	-0.0141	-0.11729	-0.23773	1.28652	1.22898	1.08393	0.82332	0.97995	0.17767	1.09873	1.45375
NBPF3	0.70197	1.12727	1.02164	0.78684	1.32197	0.6549	1.14127	0.63227	1.84648	1.82327	1.33319	1.6584	-0.25008	-0.39941	0.32545	0.40384
ALPL	-1.38114	-1.59179	-1.67094	-1.92733	-1.18503	1.70383	-1.55247	1.9431	3.82787	3.72427	4.23393	2.82739	-3.32193	-3.32193	-3.32193	-3.32193
RAP1GAP	-0.13739	-0.01003	-0.20605	-0.20737	1.85469	1.66975	1.38829	1.54751	1.14809	1.44037	1.49073	0.50266	-1.29905	-0.85546	-0.01679	0.25378
USP48	2.92641	2.91788	3.08318	3.13531	2.71427	2.07831	3.1873	2.26441	3.64781	3.21669	3.47543	3.81438	3.15848	3.17597	3.43467	3.12053
LDLRAD2	3.62304	3.82831	3.96369	3.53603	4.00671	4.20698	3.15277	4.59914	6.01246	6.12427	6.40597	5.92629	2.33035	1.95038	4.12475	2.90139
LINC00339	1.38714	1.44107	1.49137	1.57983	0.9939	1.58393	0.45451	1.86061	1.73794	2.08937	2.26283	1.73348	1.50902	1.42842	1.28848	1.47312
CDC42	6.26801	6.48518	6.54737	6.41181	6.31007	5.89664	6.89186	5.71188	7.22797	7.14681	7.22363	7.38728	6.83204	7.01145	7.23247	6.97174
ZBTB40	2.75405	2.38002	2.55017	2.48327	2.13867	2.07393	2.17218	2.23242	2.56533	2.41104	2.53052	2.90471	2.34007	2.24561	2.78112	2.77897
EPHB2	3.99379	4.10207	4.11193	3.72636	-1.88265	-2.27519	-1.44905	-2.40731	1.3262	2.46559	2.31117	2.88431	2.94812	2.85311	4.15206	3.87556
MIR4253	7.27914	7.65268	7.63569	6.33606	2.44152	1.83268	2.44014	-3.32193	5.3655	6.58275	6.71878	6.43729	6.45497	5.96506	7.89933	7.50502
KDM1A	5.02434	4.73739	4.85693	4.70172	4.14865	3.74545	4.13983	3.69383	5.65707	5.31068	5.50028	5.57952	4.67532	5.0636	5.43785	5.35658
LUZP1	3.39213	3.38855	3.29997	3.60792	2.78701	1.75115	3.24777	1.48305	4.16818	3.88829	4.20929	4.89776	3.35558	3.22349	3.61417	3.93601
HTR1D	1.58588	1.32004	1.43011	1.09696	2.6967	1.85412	2.55465	2.30792	1.78292	2.76667	1.53986	2.78108	-0.70196	-1.57742	-0.0948	-0.85161
LINC01355	-1.13159	-1.25143	-0.93719	-0.35126	-2.13828	-0.8708	-2.18595	-0.39344	-2.21091	-2.56715	-1.85087	-1.72392	-1.23821	-1.32018	-2.25918	-1.57128
HNRNPR	5.27428	4.85882	4.87219	4.8364	4.5634	3.41347	4.89327	3.53496	5.33563	4.3558	5.07835	4.41285	5.1893	4.30843	4.89707	4.15765
ZNF436	1.03128	1.12605	1.22657	1.40225	1.6711	1.09547	2.12291	1.00911	2.55381	2.89945	2.74206	3.67976	1.62776	2.06463	2.6103	2.61316
ZNF436-AS1	0.24074	-0.46713	0.18773	0.13275	0.11209	1.21208	-0.35445	0.70474	-0.42354	-0.71929	-0.124	-0.43952	-0.47726	-0.39256	-1.20119	-0.154
TCEA3	-0.82704	-1.08392	-0.35821	0.28091	2.54574	1.8589	2.06382	1.54106	-0.07864	0.34603	-0.18819	-0.12603	-2.29485	-1.28539	-0.82678	-0.85597
ASAP3	1.37271	1.36734	1.22065	1.75716	1.61689	2.4951	1.03297	2.29625	2.43275	3.10558	2.53356	3.30014	0.00296	0.99629	1.60475	2.00631
E2F2	-0.43438	-0.74937	-1.57391	-1.43707	2.52753	1.32277	1.74527	2.14198	0.19092	0.29232	-0.44705	-0.80617	0.79736	2.21442	2.0136	2.04438
ID3	4.72497	4.71308	4.4289	5.2184	7.77807	8.51189	7.16451	8.04125	2.85373	2.81014	3.17051	2.51331	3.10881	0.96669	2.57421	2.70186
ELOA	4.49654	4.48849	4.30367	4.59796	3.543	3.81494	4.2738	3.79539	3.87203	3.80413	4.37129	4.3626	3.04912	3.4448	3.85299	3.49069
ELOA-AS1	3.45052	3.46329	3.48886	3.69761	1.9967	2.41767	2.71238	2.86708	2.61532	2.87243	3.29709	3.32786	2.17751	2.53912	3.07154	2.39861
PITHD1	4.63291	4.39924	4.48227	4.51813	4.23826	4.47797	4.3351	4.42411	4.48977	4.19756	4.54049	3.95874	4.44413	4.53699	3.89524	4.14574
LYPLA2	6.56494	6.87675	6.67381	6.62436	6.06738	6.88727	5.56001	6.55474	6.3607	6.80323	6.60717	5.91266	5.60071	6.39658	5.88303	6.56641
GALE	4.33039	4.33648	4.74276	4.18219	4.66096	4.9492	4.04516	4.73592	4.60003	4.71637	4.57338	4.33531	3.91303	3.81105	3.05243	3.64763
HMGCL	1.74459	2.08461	2.38829	2.63925	2.44111	3.08826	2.16319	2.98758	2.36579	3.18292	2.56016	3.01745	1.69029	2.13201	1.82554	2.0919
FUCA1	2.38365	2.54722	2.65508	3.44854	5.137	4.96476	5.29436	4.74158	4.70364	5.19763	4.70607	5.82829	3.29034	3.40295	3.72292	3.67705
PNRC2	5.89016	5.78768	5.752	5.91464	5.17737	5.387	6.08561	4.87277	5.73413	5.44502	6.1881	5.93395	4.97064	4.6236	5.95026	5.11155
SRSF10	3.96806	3.8326	3.7523	4.13469	3.49786	2.89489	3.79802	2.79029	3.78688	3.41406	3.68569	3.48225	3.73716	3.52968	3.32708	2.77625
MYOM3	-0.32896	0.12223	0.32491	0.44353	-2.57624	-3.2045	-2.55251	-2.83751	-0.00453	1.22165	0.55851	1.50337	-3.32193	-3.32193	-3.2108	-3.32193
IL22RA1	-0.23627	-0.34934	-0.17024	-0.59887	3.12294	1.85028	2.71087	1.92415	1.74693	1.26324	1.57217	1.17393	0.27296	0.04419	1.06091	1.26355
IFNLR1	0.78561	0.91624	1.19236	1.56846	2.01806	1.32591	1.98924	0.86799	-0.14195	-0.11884	-0.47272	-0.02654	0.53231	0.80663	1.18108	1.56635
STPG1	0.51741	0.85542	0.718	0.76566	-0.13083	-0.03519	0.09812	-0.45584	1.69958	1.22795	1.63416	0.83652	0.89886	1.05093	1.54209	1.21153
NIPAL3	1.96825	2.06207	1.85597	2.1722	0.74916	0.37101	0.78056	0.05986	3.67821	3.21846	3.55603	3.05029	1.75622	2.26785	2.39754	2.95592
RCAN3	2.4583	2.9041	2.93276	3.11829	2.40797	2.57937	2.73592	2.26179	2.83522	2.62284	2.5255	3.50185	3.73767	4.20326	4.34325	4.35441
NCMAP	-3.32193	-3.32193	-3.32193	-3.01427	1.64281	1.27652	1.48884	1.55786	-3.0364	-3.32193	-2.84673	-3.32193	1.43091	2.17297	2.17705	2.74733
SRRM1	4.89187	4.47513	4.61172	4.62658	3.73906	3.35687	3.93066	3.5409	4.68935	3.91028	4.58968	4.04389	4.25699	3.90279	4.1233	3.62742
CLIC4	5.76926	6.05012	6.01841	6.29989	4.18804	3.57938	4.92448	3.3247	7.25237	6.78732	6.84353	7.5766	5.84366	5.92587	5.97101	5.8708
RUNX3	-2.18366	-2.09038	-1.92888	-0.68992	3.67506	3.58933	2.78814	3.4539	2.96327	2.74106	2.94305	2.41189	2.98376	2.85593	3.85804	3.91843
SYF2	4.47607	4.85281	4.84816	5.17713	2.81451	3.13893	2.9142	3.31236	4.25395	4.65761	4.38242	4.33996	3.07958	3.59163	3.17779	3.13342
RSRP1	1.94593	1.86649	2.28004	2.39769	1.82557	2.28532	1.42047	1.95803	0.8563	1.1453	1.83759	0.97081	0.58786	1.09102	0.03662	0.06513
TMEM50A	5.56391	5.27141	5.69387	5.35504	5.27021	4.37927	5.22194	3.96706	5.65814	4.56776	4.86883	4.1997	5.04353	3.25047	4.20819	3.33555
RHCE	1.81282	1.70079	1.51543	1.62293	0.22687	0.44134	0.0743	0.97023	1.26237	0.23466	-0.8297	1.06757	1.53556	-0.62628	-0.9258	-1.39529
MACO1	3.28329	3.38919	3.41422	3.72244	3.04755	3.38049	3.53238	3.45691	2.89203	2.92691	3.00739	3.44119	2.68057	2.79426	3.24011	2.98955
LDLRAP1	2.21545	1.89358	2.43711	1.72826	2.55375	1.4877	2.4046	1.469	2.77322	1.61056	2.85813	1.50598	2.53342	0.46442	2.51142	0.92318
MAN1C1	-3.09299	-2.57726	-2.9372	-3.03848	-0.46499	-0.9509	-1.30399	-0.59399	-2.94359	-2.90869	-2.98025	-3.00631	-2.90312	-2.46953	-2.74412	-2.44291

SELENON	4.11208	4.16193	4.3829	3.93268	6.06321	5.23868	5.26806	5.13225	6.19386	6.1456	6.28662	6.05473	4.92038	5.0243	5.67201	6.051
MTFR1L	2.41057	1.90843	2.73207	2.29288	2.65646	1.84556	2.4008	1.88225	3.20218	1.84264	3.12054	1.48239	2.68345	1.50022	2.57216	1.16048
AUNIP	3.26909	2.45687	2.57101	2.441	2.80716	2.20073	3.09661	2.2561	3.08609	1.61005	2.54669	1.54153	3.47697	2.72609	2.7074	1.61192
PAQR7	2.65331	2.83353	2.79342	2.34837	3.23386	3.76249	3.14493	3.74109	3.95465	4.3676	4.46095	4.01303	2.73269	3.10264	3.79231	3.60138
STMN1	5.81072	5.89507	5.92941	5.96126	4.81075	5.11758	4.91353	5.16526	6.05174	6.11874	6.49397	5.58246	5.76699	6.67444	5.9442	5.74851
MIR3917	8.96093	9.06326	9.31474	8.58735	8.67636	8.25526	8.55893	7.96715	9.60555	9.75151	10.2048	8.40052	9.07194	10.0815	9.8964	9.69691
PAFAH2	2.47618	2.61341	2.32629	3.16434	2.28909	2.21492	1.87592	2.10043	2.04353	2.11597	0.87979	2.25158	3.03595	2.76186	2.06911	2.69138
EXTL1	-2.56981	-2.85308	-3.32193	-2.60134	-2.66089	-2.17742	-3.1082	-2.04438	-0.40322	-0.0873	0.53288	-0.29771	-3.32193	-2.58407	-3.32193	-3.32193
TRIM63	-1.55083	0.54028	-0.57395	1.13379	-3.32193	-3.32193	-3.32193	-2.87962	-2.28501	-2.02658	-2.15205	-2.66941	-3.32193	-3.32193	-3.32193	-3.32193
PDIK1L	1.34897	0.8247	0.88259	1.72137	0.96602	0.55285	1.72725	0.66535	0.88795	0.55557	0.89155	1.0895	0.76171	0.72224	0.72444	0.72525
ZNF593	5.82834	5.84632	5.27504	4.92711	5.91567	6.43174	5.43455	6.31846	5.64263	5.846	5.81633	4.31507	5.2135	5.30353	4.7745	5.00049
CNKSR1	-2.85315	-2.42519	-1.42309	-1.76675	-1.92655	-0.32422	-2.54224	0.17309	-2.78959	-2.24422	-2.06368	-2.87412	-2.39414	-2.28701	-3.32193	-1.70808
CEP85	2.91796	2.45899	2.3271	2.33148	2.52756	2.22893	2.23565	2.3424	3.2148	2.37284	2.47044	2.16011	3.4929	3.39736	2.87374	2.89407
SH3BGRL3	7.84837	8.11651	8.29364	8.04123	5.62739	6.99206	5.15845	6.68227	7.99023	7.83935	7.86236	7.27033	6.22156	6.02602	5.66676	5.72742
UBXN11	5.00191	5.01613	5.16159	5.19262	3.73278	4.54872	2.96072	4.34294	4.78465	4.48068	4.6568	3.79492	3.73026	3.75848	2.69244	2.90193
CRYBG2	0.35617	0.6625	-0.005	-0.85367	2.4374	2.30748	1.97937	2.20177	0.01355	-0.18572	-0.36281	-2.02379	-3.18379	-3.32193	-3.00588	-3.15179
DHDDS	3.038	2.59982	2.56878	2.75931	3.25254	3.36184	3.22796	3.21286	3.6875	3.14731	3.49176	3.16377	2.82729	2.78417	2.8738	2.91791
HMGN2	7.4407	7.25483	7.24379	7.24539	5.28493	5.62526	5.42749	5.92006	7.75282	7.25461	7.39768	6.63338	7.39393	7.57387	6.78207	6.63933
RPS6KA1	3.47087	3.46864	3.34979	3.52226	3.5665	3.56674	3.34996	3.49044	4.22715	4.09749	4.26837	3.82068	2.57011	2.83446	3.41153	3.11005
ARID1A	2.95748	3.00892	3.15771	2.91333	4.00813	2.59897	3.51069	2.87834	4.53711	4.30089	4.789	4.34067	3.63143	3.66016	4.56399	4.63628
PIGV	2.53388	2.56589	2.65508	2.44109	1.82394	1.50483	1.56023	1.50056	2.52481	2.90519	2.74097	3.37715	1.65002	2.07122	1.52737	2.4762
ZDHHC18	3.85857	3.67534	3.49145	3.51336	3.32309	3.42284	3.18309	3.26705	4.07849	3.61227	3.81527	3.35008	4.34743	3.9984	4.7274	4.37493
SFN	7.29921	7.35316	7.38552	6.21866	7.26958	7.25963	6.71325	6.93563	7.30727	6.55359	7.20638	5.13128	1.14985	1.94999	1.59412	2.33185
GPN2	3.60652	3.72486	3.69112	3.20643	3.57897	3.56194	3.11606	3.72132	3.53447	3.56736	3.61583	3.1669	3.29404	3.26905	3.0864	3.25726
GPATCH3	3.64994	3.5805	3.59012	3.4093	4.13595	3.92215	3.82436	3.94185	2.84708	3.21977	3.58358	3.07039	2.90799	3.47967	3.57564	3.63488
NUDC	7.82057	7.59626	7.39753	7.1451	6.93524	7.31581	6.76715	7.19877	6.9661	6.40187	6.78178	5.40339	6.6026	6.7058	6.62221	6.61484
NR0B2	-2.58088	-3.32193	-1.56225	-3.32193	3.86924	6.15187	3.80866	6.2119	-3.32193	-2.60172	-3.32193	-3.32193	-1.95571	-1.46572	-2.60735	-2.11381
TRNP1	4.54063	5.88151	5.31105	5.30448	8.0138	8.7412	7.56551	8.48004	6.94039	7.98747	7.07054	7.2799	7.45579	7.51227	7.46353	7.97399
TENT5B	2.29807	1.9998	2.21508	1.36879	1.95836	0.67014	1.67355	0.76411	4.8223	3.26975	4.24625	2.06015	3.93312	3.67411	3.93622	2.98261
SLC9A1	3.96098	3.7393	3.9902	3.30135	3.25825	2.65233	3.17206	2.4164	4.57979	3.7406	4.81907	3.76143	2.06046	1.20846	3.49198	2.2372
NPM1P39	2.86026	2.49215	2.59432	1.75584	1.48508	1.06433	0.9548	0.46224	2.19353	2.2773	2.32623	2.06122	0.01678	-0.9454	0.17071	0.08533
WDTC1	3.10057	3.0429	3.18917	2.98313	2.68133	2.47459	2.24554	2.2271	4.10703	4.242	4.16656	4.39384	2.59576	3.08029	3.57693	3.77915
TMEM222	3.77367	3.69215	3.61845	3.40097	3.43692	3.55993	2.70612	3.40285	3.76807	3.85762	3.77587	3.2632	3.18903	2.87612	3.21794	3.05134
SYTL1	-2.56068	-1.58206	-1.527	-1.77099	-2.15867	-0.82688	-2.84766	-0.96215	-2.75179	-0.994	-1.95242	-1.56845	-2.04957	-1.81375	-1.35129	-1.78642
MAP3K6	2.25443	2.46446	2.56841	2.36908	3.24296	2.66195	3.09228	2.6097	3.3904	3.00106	3.54179	2.7941	2.49538	2.52754	3.34236	3.16554
GPR3	1.76666	0.83891	0.61792	-0.09873	0.25211	-0.01383	-1.29236	-0.40603	0.98315	-0.37271	-1.17745	-0.68544	1.9827	0.76909	-1.2677	-0.35772
WASF2	4.65486	4.12835	4.82043	3.93455	4.60489	3.07665	4.72627	2.89432	6.3279	4.70972	6.04833	5.04888	5.53868	3.38156	5.86476	3.7082
AHDC1	3.34378	3.63856	3.79682	3.07908	1.04081	1.05212	0.63935	1.14751	4.48935	4.68363	4.72546	4.39724	2.84506	2.90991	3.00182	3.59321
IFI6	5.94396	6.04138	6.06528	6.06455	5.4124	5.66193	5.2994	5.42273	5.53947	6.0952	5.92369	5.78485	2.98231	4.00627	3.36375	4.29467
FAM76A	1.47212	1.10533	1.59338	2.00373	-0.0497	0.5667	0.58573	0.86098	1.39344	1.03423	1.36257	1.6947	1.47661	1.65483	2.04362	1.61658
STX12	2.96882	3.65048	3.34556	4.27115	2.53163	2.92373	3.6755	2.87276	2.84945	4.03453	4.05078	4.83624	2.67274	4.04569	3.59502	3.61709
PPP1R8	4.24274	4.12143	4.10637	3.88771	3.93222	3.27853	4.07078	3.22763	4.23673	4.10189	4.02427	3.95895	4.59006	4.49566	4.26378	4.22436
THEMIS2	1.91128	1.91687	2.11049	2.04562	-1.70434	-1.24247	-2.48691	-2.64448	0.41133	0.34039	0.61044	0.7315	-1.46132	-2.03373	-0.86514	-1.17074
RPA2	4.87357	4.71643	4.73117	4.88776	4.6763	3.93516	4.51408	4.13845	5.12361	4.35145	4.74099	4.28714	5.81881	5.49737	4.77834	4.58696
SMPDL3B	-1.15579	-1.34506	-0.16477	-0.32695	2.03993	3.55317	1.81509	2.97804	-0.30482	0.63931	0.27997	0.76407	1.16391	1.69218	2.47698	2.25716
XKR8	2.13776	2.838	3.01401	3.04334	4.24629	4.51911	3.79289	4.35478	3.6849	4.12088	4.19857	4.14753	2.45589	2.93622	3.45058	3.37839
EYA3	2.19453	2.32323	2.06513	2.3469	1.02656	1.25562	1.53848	0.91065	1.79968	2.30026	2.2061	2.99981	1.74609	1.90846	2.20627	2.18991
SPCS2P4	4.58696	4.4827	4.3279	4.28709	4.36616	4.73351	4.68584	4.35284	4.20015	3.74392	3.37455	3.18069	3.4229	3.12596	2.55262	2.41338
DNAJC8	6.05074	5.93573	6.02911	6.11143	5.40244	5.52163	5.70257	5.40995	6.0956	5.6509	5.79444	5.48003	5.76548	5.86686	5.58043	5.27831
ATP5IF1	6.62972	6.58998	6.61105	6.38374	5.97563	6.79328	5.56315	6.74894	5.86699	5.73914	5.65226	4.09566	5.84243	5.55178	4.81292	4.86136

SESN2	4.12904	4.16074	3.70798	3.84238	3.57852	4.6377	3.40182	4.50909	4.76098	4.72433	4.68442	4.52927	2.41885	3.24331	2.70408	3.72668
MED18	4.55524	4.62605	4.51256	3.97062	3.80162	4.13668	3.74162	4.30788	4.30778	4.19965	4.44383	3.66362	3.1961	3.36497	3.11905	3.6284
PHACTR4	3.16544	2.79761	2.83946	3.05358	3.31077	3.17774	3.63441	3.3838	4.23761	3.12715	3.90803	3.72831	3.61655	3.05458	3.83064	3.38888
RCC1	5.5202	4.74093	4.84733	4.17935	5.28591	4.0022	4.89099	4.11581	5.82448	4.92957	5.69598	4.52935	5.1107	4.13108	4.67164	3.66717
SNHG3	5.0453	4.57005	4.6425	4.38236	3.47852	3.86907	3.20858	4.40845	3.02764	3.71534	2.93219	3.51586	3.63578	3.33685	1.95243	2.10001
SNORA73A	4.6419	4.01518	3.9332	4.02424	2.29288	3.32253	2.60374	3.49863	2.91548	2.56387	1.50186	2.01035	3.53746	1.8597	1.88639	1.41881
SNORA73B	4.82036	4.66074	3.98873	4.56278	2.30667	2.73103	2.3425	3.32988	1.46117	1.20239	2.14905	1.39213	3.35528	1.98584	1.40199	0.96816
TRNAU1AP	2.61191	2.88649	3.13436	3.19664	2.32005	2.35211	2.39021	2.45279	3.01682	2.76578	3.00139	2.92131	2.88319	2.70857	2.43138	2.22277
SNHG12	3.4404	2.47734	3.11263	2.41207	2.49675	3.32178	1.88074	3.73369	1.39935	2.18028	1.59857	1.02791	2.185	2.08555	0.33681	-0.3305
SNORA61	6.22356	5.2591	5.54787	5.45329	5.1434	6.41297	4.94253	6.70714	3.18924	3.53779	3.4896	2.78484	4.4024	5.01157	2.38681	2.82605
SNORA44	3.41731	3.41401	4.34347	3.04698	1.37692	1.87455	2.33329	3.20028	0.55273	2.37929	1.65329	2.00063	3.21919	2.08097	2.19878	1.82484
SNORA16A	4.33783	3.19122	3.97869	2.37304	3.02811	3.23271	3.36403	3.05408	1.78292	2.75543	2.59828	0.79495	2.05435	3.25166	1.76271	1.22904
TAF12	3.34521	3.28604	3.3184	3.66894	2.75645	3.16963	2.77124	3.34662	3.00935	3.1806	2.86599	2.9425	2.60504	2.63652	1.69556	1.76794
RAB42	1.32131	1.58041	1.55682	1.89039	-2.78639	-2.88184	-3.32193	-2.14637	-0.75616	0.6752	-0.62551	-0.14777	-0.1496	-0.39413	0.31636	-0.11054
GMEB1	2.39367	2.41493	2.03571	2.47532	1.66315	1.86212	1.56568	1.54376	1.88344	1.74174	1.79116	2.09011	1.68102	1.74225	1.26661	1.69192
YTHDF2	5.27107	5.26756	5.07349	5.5411	4.37113	4.08903	4.65104	4.21283	4.9435	4.70531	4.86201	5.15924	4.75971	4.8218	4.8192	4.79053
OPRD1	-2.90811	-2.86473	-3.18653	-2.4432	-3.04497	-3.2056	-3.21421	-3.18602	-3.32193	-2.92101	-3.32193	-2.68861	-1.69802	-1.09065	-1.49924	-0.44975
EPB41	2.46665	2.25225	2.14531	2.50912	2.5885	2.11708	2.80483	1.91804	2.21818	2.12601	1.93958	2.77001	2.31795	2.22162	2.44927	2.64221
TMEM200B	2.74153	2.56308	2.24307	2.89745	4.60327	4.08153	3.8455	3.85218	3.60897	3.5726	3.57543	2.98303	3.55562	2.81422	3.84439	3.36295
SRSF4	4.39245	4.08724	4.19276	4.08002	3.50736	3.60226	3.56944	3.76935	4.40738	3.53659	4.26126	3.37513	3.72323	3.26363	3.93609	3.30409
MECR	2.70302	2.36231	2.54112	2.23927	1.51008	1.97876	1.20047	1.95082	3.10997	2.88547	2.90304	2.31543	2.31716	2.00855	1.83087	1.77465
PTPRU	-0.94857	-0.3867	-0.53176	-0.02948	4.03159	2.92457	3.14028	3.3425	-0.15754	1.07373	-0.34767	1.60405	0.18568	0.64974	1.77689	2.30118
MATN1	-2.44025	-2.50922	-3.05934	-2.27677	-1.95769	-1.23889	-1.79214	-0.94098	-2.48727	-1.61052	-1.95959	-2.13422	-2.48031	-2.98498	-2.60515	-2.91173
MATN1-AS1	-2.34786	-1.56435	-1.60603	-1.64776	-1.04396	-0.72018	-1.93218	-0.11114	-1.52863	-1.02746	-2.32264	-1.73156	-3.11891	-3.11959	-3.07808	-2.86099
LAPTM5	4.06534	5.07489	4.36272	5.43872	-1.9374	-1.66332	-2.40728	-2.23255	-1.04168	0.25972	-1.02058	1.7284	-3.32193	-3.07697	-3.32193	-3.32193
SDC3	3.40587	3.61083	3.81894	3.37673	2.81852	2.26546	2.23981	2.07095	4.23667	4.54507	4.54633	4.48615	2.61417	2.11037	4.17317	3.46917
PUM1	3.72177	3.89229	3.93871	4.23415	3.66114	2.70045	3.99691	2.98072	4.72601	4.86823	4.91838	5.53312	4.6104	4.49835	5.14288	5.11796
NKAIN1	-0.0241	0.08733	-0.10293	0.59499	-2.17535	-0.87142	-3.00864	-1.526	-0.85458	-0.23164	-1.33611	0.27315	-3.05462	-1.23604	-0.42469	-0.34994
SNRNP40	4.29411	3.81596	3.78597	3.63457	3.61716	3.18869	3.70858	3.2217	4.6355	3.8508	4.60516	3.5696	3.77764	3.6068	3.7347	3.05588
ZCCHC17	4.96392	5.07089	4.76255	5.38966	4.29223	4.41836	4.1156	4.56063	5.13669	4.77666	4.80389	4.34231	4.86418	5.06473	4.46261	4.58689
FABP3	-0.50397	0.45793	-1.12509	1.07409	-0.63004	-1.30069	-0.7508	-2.39613	-0.483	-0.14818	-1.96169	-0.29139	-0.1052	-0.66323	-0.2824	-0.02058
SERINC2	7.69013	7.98183	8.01147	7.21662	3.74816	4.92351	3.10981	4.35336	5.91209	5.44291	5.71798	4.7398	1.01353	2.15486	2.53606	2.87051
LINC01226	2.52768	2.11966	2.51856	1.57783	-3.01824	-3.21132	-2.87005	-2.96442	-1.48805	-1.90405	-2.2223	-2.9447	-0.39472	-0.29751	-1.31841	-1.24123
TINAGL1	6.63545	6.03896	6.4052	4.6901	-1.96555	-2.09707	-2.5684	-1.69077	1.94791	0.91506	1.17753	0.22993	2.81849	3.04362	2.13541	1.96898
HCRTR1	2.68053	2.50716	2.61797	2.50897	2.04729	2.03139	1.37423	1.54342	2.45834	2.24853	2.50964	2.13281	2.13046	1.85017	2.85322	1.80941
PEF1	5.48698	5.27456	5.30338	5.16286	4.6757	4.78209	4.24549	4.55328	4.83682	4.63621	4.96109	4.6858	5.09367	4.58415	5.27664	4.56074
COL16A1	1.60361	1.84267	2.15218	2.23857	-2.35095	-2.65053	-3.32193	-2.94477	0.35351	1.15287	1.24649	1.09227	-3.06275	-2.4346	-2.75666	-2.39682
ADGRB2	1.15797	1.95793	2.34122	2.04606	-1.34651	1.26104	-1.54432	1.35825	3.42216	4.22974	3.83394	3.88604	-2.00607	0.1577	0.03806	1.07167
SPOCD1	3.01147	2.90959	3.47318	3.02652	-2.48124	-1.13789	-2.45956	-2.03906	2.19196	3.12252	3.20891	3.11905	-3.20314	-3.32193	-3.17842	-2.607
PTP4A2	5.87047	5.66065	5.91686	5.60741	5.61545	5.38054	6.14121	5.00817	6.5457	6.17644	6.16391	6.48365	5.59512	4.47814	5.37159	4.337
KHDRBS1	6.67584	6.73523	6.69818	6.83597	5.64552	5.38492	6.04228	5.37492	6.88504	6.63862	6.69743	6.93906	6.47656	6.73492	6.7709	6.65732
TMEM39B	3.71951	3.66715	3.51688	3.27639	2.40002	2.78445	1.86602	2.80206	3.90145	3.9286	3.92973	3.85819	2.49603	2.67209	2.32589	2.61262
KPNA6	4.60392	4.50937	4.28159	4.70674	4.2206	3.89138	4.45743	3.98055	4.66006	4.47717	4.88202	5.18415	4.72495	4.51734	4.86864	4.82012
TXLNA	4.16513	3.56617	3.99443	3.37995	4.90536	4.20879	4.81011	4.02755	4.85907	4.12637	4.99952	4.66943	5.18751	4.50499	5.11579	4.82876
CCDC28B	2.72243	2.57107	2.60196	2.47473	0.89107	2.20512	-0.28659	2.37226	2.57493	2.68277	2.84313	2.17392	2.16353	2.23955	1.9189	1.81285
IQCC	1.5454	0.92057	1.09817	0.68031	1.28756	0.92982	0.58815	0.94515	0.7359	0.54508	0.78434	-0.10258	1.68537	1.33373	0.6464	0.97411
DCDC2B	0.71058	1.10487	1.92211	1.49874	1.0034	1.77268	-0.47354	1.54365	-1.04054	-0.35709	-0.26654	-0.23793	-0.4777	-0.08492	-1.26127	-0.49847
TMEM234	2.05051	1.86282	2.54008	2.21864	2.01157	2.39014	1.39831	1.89495	2.00566	2.01439	1.99008	1.83245	2.0361	1.87967	1.326	1.66692
EIF3I	7.85933	7.85575	7.70329	7.66482	6.90144	7.26759	6.925	7.05154	8.02218	7.8436	8.04491	7.9517	7.59125	7.44667	7.54627	7.49455
MTMR9LP	0.44165	0.70472	1.14511	0.91596	-1.58305	-2.27924	-2.01858	-1.39371	-1.20046	-0.05984	-0.57729	0.62406	-1.36778	-0.91677	-0.41377	0.32314

HDAC1	5.23205	5.36013	5.37072	5.56064	5.73783	5.05429	5.66394	4.89495	6.12239	5.2307	5.95652	5.56811	5.75569	4.77795	5.52953	4.89905
MARCKSL1	5.01369	5.76955	5.78076	6.10759	6.48318	6.84855	6.17573	6.38655	5.21782	5.282	5.53989	5.58093	6.85418	6.77204	7.56118	7.56412
TSSK3	1.38684	1.44578	1.68056	1.47669	0.73519	2.01424	-0.05934	2.0066	-0.18064	0.21277	0.57962	-0.03933	0.54579	0.67196	0.39784	0.81498
FAM229A	2.31706	2.26765	2.59617	1.90708	0.95767	2.7602	-0.21101	3.19932	-0.11334	0.83479	0.8892	0.55646	0.46709	0.74493	0.40507	0.76325
BSDC1	3.14367	3.33256	3.35706	3.51047	3.07934	2.74299	2.89226	2.39696	3.3645	3.57905	3.81243	3.69197	2.83185	2.84823	3.05115	3.13485
ZBTB8A	0.85703	0.87664	0.95045	1.4785	0.26805	0.48304	0.87225	0.51689	1.26779	0.85892	0.98191	1.7641	1.19629	0.38676	1.74729	0.95701
ZBTB8OS	3.5079	3.62776	3.49125	3.85809	2.83754	3.58104	2.86414	3.37858	2.85362	2.82238	2.72514	2.4198	3.47518	3.34654	2.44104	2.36196
RBBP4	3.9365	4.04612	3.8704	4.3715	3.42312	3.10463	3.49919	3.42031	4.3825	4.08179	3.82371	4.36045	4.29369	4.6731	4.31672	4.28382
SYNC	2.64226	2.95782	3.09391	3.36057	2.14404	1.84323	2.41565	2.39569	3.21077	3.31491	3.21004	3.6495	3.81783	4.15423	4.09774	3.5957
KIAA1522	3.57619	3.69991	4.0173	3.37834	5.29589	3.56653	4.75303	3.43005	4.18011	4.00196	4.20724	3.94322	5.00985	4.16559	6.25219	4.86548
S100PBP	1.82888	1.91696	2.14091	2.4546	1.41327	1.3742	1.57343	1.57124	2.01945	1.97003	2.05577	2.53552	1.77675	1.73795	1.42607	1.65066
FNDC5	-2.52139	-2.24464	-2.65667	-1.65068	-2.14554	-2.15204	-3.02234	-2.01718	-1.87601	-3.32193	-2.72521	-1.58038	-0.2583	2.24842	1.50504	3.33818
HPCA	5.02382	5.30775	5.36303	4.52864	4.38157	4.00914	3.61509	3.42813	6.59817	6.37086	6.48327	4.96419	2.50809	3.128	3.55904	3.61294
TMEM54	6.09479	6.26461	6.30862	5.6643	5.25034	5.06341	4.54297	4.5894	7.46157	7.2718	7.34824	6.04193	3.5987	4.19492	4.4738	4.50522
RNF19B	5.12727	5.30076	5.22391	4.93283	3.87271	2.94046	3.85858	2.48994	4.85949	5.10653	5.15952	5.07719	4.21521	4.00219	3.7968	4.37985
AK2	5.30114	4.91076	4.80011	4.50569	4.69308	4.61893	4.67678	4.52488	5.49367	5.09791	5.09358	4.66142	5.28346	4.51181	4.68257	4.2468
AZIN2	0.11388	0.35844	0.75199	0.43806	-2.39245	-0.94692	-2.49559	-1.75128	0.74994	1.5508	1.33749	1.18663	-1.51335	-0.90469	-1.66817	-1.16353
TRIM62	1.50894	1.60349	1.39062	1.77914	0.39116	0.38434	-0.12867	0.73965	2.84825	3.16473	2.77566	3.70435	0.91411	1.98376	1.83809	2.81521
ZNF362	-1.69106	-0.94777	-1.03962	-0.11468	1.2425	1.40286	0.29978	1.81095	3.6691	4.28466	3.96088	4.30762	2.87352	3.74764	4.57911	4.496
PHC2	5.21775	5.18995	5.44649	5.33184	4.58686	4.82927	4.33285	4.40834	5.89976	6.38735	6.18315	6.52657	5.13253	5.0302	5.19109	5.34379
MIR3605	6.64702	6.83563	6.71317	6.90247	6.85956	5.71907	6.44353	5.31449	7.92346	8.26887	8.17363	7.95876	7.26616	7.10467	7.21238	7.09202
ZSCAN20	0.72238	0.41236	0.73035	0.70669	-0.3077	-0.62818	-0.35397	-0.60709	1.13124	0.88625	1.12112	1.16897	0.20647	0.4267	0.52624	0.49849
SMIM12	2.9851	3.01015	2.80697	2.67464	3.23653	2.71567	2.75548	2.73807	3.00495	2.64627	2.53455	1.96388	2.80864	2.87304	1.96712	2.37377
GJB3	-0.41448	0.01772	-0.40574	-0.97012	1.59154	-3.32193	2.33546	-2.21077	0.06328	-0.28431	0.42897	-1.29253	-3.32193	-3.32193	-3.32193	-3.32193
DLGAP3	-2.65703	-3.32193	-2.56183	-3.00421	0.92489	1.31469	0.0666	1.14552	0.86707	1.28303	0.76308	0.23023	-3.11668	-2.27481	-2.23927	-1.6059
TMEM35B	4.64967	3.90049	4.98249	3.76509	5.02352	3.42475	4.85876	2.58669	4.51096	3.22553	5.04713	2.7492	3.90728	0.85481	3.01016	0.32198
ZMYM6	1.24726	1.42196	1.50197	2.09699	0.65163	0.4436	1.33179	0.66197	0.95871	1.07946	0.75222	1.97661	1.58667	1.89518	1.35001	1.58027
ZMYM1	2.01955	1.97275	1.94555	2.27155	1.72334	1.167	2.4717	1.31616	2.5171	2.63763	2.50558	3.54621	2.29968	2.26857	2.11127	1.9563
ZMYM4	2.81602	2.94879	2.78023	3.35178	3.06628	2.07903	3.56536	2.10061	4.21433	4.19407	4.21024	5.13274	3.78577	3.34176	4.00884	3.85215
RPL5P4	2.44454	2.01179	2.34523	2.24627	1.86169	2.61415	2.14851	2.40858	2.0958	2.83007	2.06474	4.10491	0.62402	1.01057	0.02854	0.46703
ZMYM4-AS1	2.11345	2.38522	2.17221	2.65313	3.50775	0.37488	4.16474	0.93361	4.7137	4.56941	4.59082	5.4758	4.01157	3.55038	4.4736	4.59641
KIAA0319L	2.74272	2.84835	3.12954	3.3337	3.70181	3.67956	3.71244	3.58119	3.90385	4.13844	4.31513	4.81038	3.02364	3.07243	3.63466	3.8621
NCDN	4.11181	3.97345	3.71934	3.45677	3.46561	3.0341	2.89767	2.68833	3.4807	3.61068	4.04589	3.33566	3.09524	2.75043	3.20673	3.23086
TFAP2E	-0.01977	0.28009	0.39851	0.77511	-0.01679	-0.1703	-0.16118	-0.08402	0.77169	1.91008	1.45656	1.35256	-2.24395	-1.51828	-0.98218	-0.86303
PSMB2	6.41868	6.29408	6.13176	5.98277	5.04325	5.3968	5.15734	5.38825	6.19623	5.69465	5.98569	5.69866	5.74373	5.89932	5.48075	5.48881
C1orf216	2.38168	2.25727	2.19682	2.44512	2.2835	1.66472	2.43568	1.17772	3.40808	3.6456	3.6599	3.64944	3.17478	3.13447	3.26225	3.39715
CLSPN	4.58214	4.29249	4.34132	4.74945	3.77126	2.92555	3.94642	3.72271	3.78881	2.26798	2.86919	2.04909	3.7141	3.41093	3.63834	3.04131
AGO4	1.09754	1.32047	1.66084	1.83935	1.28718	1.6728	1.61394	1.52705	2.33365	1.8973	2.42693	2.84137	2.37262	2.07105	2.99308	2.30637
AGO1	2.1891	2.03427	1.84384	2.099	3.2825	2.87226	3.39132	2.8887	3.3032	3.3236	3.44222	3.8283	2.22251	2.85388	3.4171	3.54793
AGO3	0.25037	0.45224	0.48038	0.7675	0.15607	-0.15612	0.63719	0.19258	0.55458	0.51965	0.78466	1.3116	0.86965	0.74911	1.22745	0.85121
ADPRHL2	5.57311	5.51555	5.39215	5.16007	4.64155	4.79017	4.15196	4.6974	5.48109	5.61227	5.76104	5.1143	4.74915	4.93428	4.88091	4.19442
COL8A2	-3.32193	-2.86452	-2.65229	-2.14636	-2.96316	-3.09785	-2.93207	-3.32193	-1.8685	-1.69742	-0.7387	-2.04814	-2.7171	-2.98911	-1.31661	-1.6124
TRAPPC3	4.63296	4.57653	4.5424	4.39354	4.24272	4.38254	4.38169	4.22365	4.73882	4.52596	4.74752	4.12221	4.46469	4.14068	3.66262	3.61774
MAP7D1	6.4922	6.51637	6.68337	5.9209	5.07628	5.26937	4.78888	5.26375	7.04968	6.86613	7.21833	6.27327	5.78099	6.10196	6.76885	6.61444
THRAP3	5.58254	5.41491	5.43364	5.26278	5.26723	4.6431	5.355	4.80093	6.38754	6.04984	6.44861	6.29419	5.41957	5.30945	5.79246	5.55817
SH3D21	3.57243	3.7461	4.37107	3.27352	1.73101	3.48122	0.96049	2.85991	4.55522	4.76461	5.26759	3.45553	2.42939	1.32016	2.79843	2.34398
EVA1B	5.28549	5.55683	6.19114	4.99531	3.56455	5.28464	2.727	4.68441	6.54944	6.89563	7.37977	5.28256	4.62071	3.2714	4.89612	4.50718
STK40	3.14745	2.69806	2.90093	2.35905	4.47674	4.40496	4.36839	3.8348	6.05513	5.81209	6.29042	5.77024	4.82573	4.62409	5.49007	5.38556
LSM10	4.91849	4.79792	4.37942	4.14177	5.26282	5.93691	4.65727	5.75572	5.45945	5.49425	5.44734	4.18187	4.95371	5.06136	4.337	4.61783
OSCP1	0.24572	-0.08371	0.20835	0.59044	-0.20342	0.96912	-0.22842	0.57594	1.05229	1.31563	1.24988	1.91389	0.98227	1.80326	1.46353	1.76005

MRPS15	5.31703	4.94674	4.76983	4.82224	5.06622	5.71633	4.9253	5.55411	5.52293	4.88202	5.14088	4.53211	5.24269	4.54149	4.75683	4.16542
LINC01137	2.42906	3.27116	2.92867	2.95524	2.29874	2.72797	2.1745	2.44892	2.46785	3.05457	2.8342	2.42607	1.16076	2.00318	1.11877	1.96366
ZC3H12A	5.6662	5.54401	5.48804	5.51159	3.94122	3.60211	3.06013	3.1939	5.35351	5.36546	5.77248	5.36753	1.48954	2.23885	0.69556	1.14488
MEAF6	3.6841	3.79598	3.77122	4.08635	3.26021	3.24209	3.49804	3.0123	4.22508	4.29666	4.04788	4.28161	3.93924	3.88237	3.28506	3.30048
SNIP1	2.42952	2.27092	2.09083	2.45069	1.75199	1.40252	2.0509	1.12446	2.42068	2.57309	2.78099	2.54891	1.47357	1.91274	1.97839	1.91184
DNALI1	-0.06876	-0.02955	-0.3567	-0.02987	-1.20797	0.23171	-0.35321	0.57338	-0.9292	-1.14335	-1.29783	-1.52419	-0.51376	-0.83062	-2.00194	-1.30064
GNL2	4.39505	4.18023	3.99358	4.27459	3.81582	4.01097	4.19053	3.96402	4.00205	3.5998	3.97438	3.47264	3.65649	3.29623	3.5415	3.29516
C1orf109	1.9433	1.70623	1.14743	1.6449	2.70223	1.7239	2.6906	1.84705	2.27517	1.86575	1.93937	1.90072	2.54739	1.82512	1.58874	1.37511
CDCA8	4.80969	4.06637	4.00688	3.23049	4.47216	3.48442	4.33625	3.6625	5.75473	3.99304	5.08882	3.18106	5.28578	4.55069	5.03621	3.91065
EPHA10	-2.95946	-3.32193	-3.32193	-3.32193	0.43235	-0.38798	-0.35053	-0.88461	-1.54179	-0.5213	-1.90149	-1.50343	-3.03061	-3.03155	-3.19641	-2.86603
MANEAL	2.9757	3.30524	2.85384	2.8693	5.53327	4.67407	5.01196	4.72107	4.81447	4.69847	4.74812	4.20422	3.039	3.29519	3.64941	3.53841
YRDC	6.14362	6.08515	5.75166	5.81039	5.86761	5.75232	5.75113	5.80117	5.17259	4.91767	4.99307	3.93458	5.15905	4.79384	4.5304	4.42922
C1orf122	5.72542	5.7956	5.64178	5.29871	6.2335	6.26987	5.5503	6.24373	5.57572	5.40946	5.59452	4.13034	4.95193	4.6627	4.85944	4.65854
MTF1	1.97465	2.12651	1.89129	2.52444	1.87247	0.99801	2.08241	1.18546	2.41966	2.28555	2.47273	3.06853	2.19962	2.59692	2.5147	2.81123
INPP5B	1.03339	0.87687	0.85538	0.6204	0.2541	0.78716	-0.04683	1.17003	2.40396	1.76641	1.93298	2.03131	0.96999	0.90365	0.96992	0.93736
SF3A3	5.53367	4.99605	5.04693	4.80868	4.17269	3.64574	4.26341	3.75478	5.49603	3.98855	5.13451	4.24131	4.85355	3.78818	4.36202	3.29968
FHL3	4.56122	4.55909	4.6683	4.73859	2.45812	3.66329	2.35754	3.49154	3.09053	4.21035	3.73331	4.42411	3.18765	3.47311	3.86737	3.81596
UTP11	5.23395	5.10236	4.89411	5.05617	3.25484	3.87593	3.65522	3.97511	4.28553	3.95517	4.03927	3.90374	4.23259	4.35282	3.32746	3.43828
RRAGC	3.68736	4.04033	4.18993	4.73898	2.5913	2.67524	3.48963	2.40571	3.22551	3.41206	3.32814	4.07918	3.78757	3.96577	3.77712	3.83388
MYCBP	3.81452	3.69077	3.56553	3.75271	2.7822	2.62481	3.17245	2.66845	4.22135	3.75982	3.9216	4.06668	3.57344	3.59952	3.06871	3.05301
GJA9	-0.95725	-1.18839	-0.55405	0.51972	-1.69751	-1.44599	-2.55267	-1.43728	-2.11048	-1.65153	-1.0266	-1.24497	-1.44995	-1.70756	-2.03449	-1.48302
RHBDL2	2.12053	1.90557	2.06326	2.25165	-2.27831	-1.89725	-1.58519	-2.32856	1.50767	1.99372	1.80504	1.45017	-2.26	-3.03887	-2.47766	-2.08626
AKIRIN1	4.51689	4.52843	4.65491	4.78035	5.71578	4.85731	5.58632	4.80488	5.21821	4.71015	4.7086	5.03065	5.22884	4.50681	4.67157	4.15029
NDUFS5	9.00144	8.97823	8.89823	8.91592	8.25523	8.88243	7.93627	8.81499	8.67196	8.5906	8.68267	7.21914	8.0079	8.0684	7.45405	7.46429
BMP8A	-2.77724	-1.97452	-3.32193	-2.66286	3.00869	1.59541	2.4581	1.47163	0.08367	-0.38933	0.07089	-0.90281	-1.01364	-0.65037	-1.15086	-0.07325
OXCT2P1	-0.72352	-0.85203	-1.83802	-0.87091	2.31456	2.4099	1.66346	2.49384	2.20956	2.00019	1.6972	0.57534	-1.26226	-0.61577	-0.55791	-0.169
PABPC4	5.89522	5.58296	5.43568	5.39117	4.9264	4.82024	5.05143	4.97423	6.00274	5.51091	5.82397	5.97207	5.75609	4.97447	5.94313	5.41443
PABPC4-AS1	7.02828	6.69123	6.45192	6.55167	6.7171	5.74547	6.90544	5.71991	7.69383	7.13629	7.43352	7.36759	7.33282	6.56139	7.44506	6.90479
HEYL	-1.25844	-1.7841	-1.50981	-1.94023	0.57197	0.3549	0.49165	0.14875	2.30871	2.1419	2.15328	1.56257	-1.91005	-2.00611	-1.72545	-0.7963
PPIE	2.47061	2.39779	2.18005	2.21417	4.19107	3.73055	4.32646	3.55659	2.7904	2.85601	2.72791	2.51488	2.17462	2.4389	2.14244	1.68505
BMP8B	-1.85178	-2.69716	-2.22154	-2.82994	4.44785	3.16235	4.21215	2.88633	1.01934	0.89052	0.45123	0.31542	0.0645	-0.02984	0.72428	0.38894
OXCT2	-1.05129	-1.37243	-1.73015	-1.24488	2.76875	2.64278	2.3725	2.96057	2.00107	1.90604	1.47815	0.70589	-0.73822	0.40889	0.25783	0.63388
TRIT1	3.63892	2.90553	3.2519	2.97511	4.35746	3.44427	4.71091	3.60036	3.24546	2.57864	3.43538	2.57467	2.82513	1.46567	2.93897	1.3482
MYCL	-1.41147	-1.48163	-1.57307	-1.15737	2.02089	0.98213	1.50234	0.79988	0.15786	-0.73076	-0.71747	-1.15595	-1.77522	-2.03742	-1.34845	-1.69232
MFSD2A	2.51038	1.95708	1.84316	2.04772	2.49683	2.54547	2.12061	2.53938	1.39042	0.28766	0.11529	0.05166	-0.12122	-0.89302	-0.83054	-0.6683
RLF	2.73032	2.72839	2.92189	3.17151	3.43751	2.42333	4.1641	2.27626	3.61093	3.81345	3.49318	4.68156	2.57539	2.69874	3.14074	2.99014
ZMPSTE24	4.50167	4.68682	4.48989	5.22406	7.31142	5.65243	8.06533	5.72091	3.92147	4.08283	4.21914	4.97594	4.88916	5.00403	4.83182	4.87478
COL9A2	-0.96765	-0.19021	-0.82324	-0.11073	0.77567	0.602	-0.52825	0.78528	1.87349	2.78977	2.34422	2.46566	-1.93718	-2.79799	-0.73737	-0.20701
SMAP2	4.15541	3.93644	3.81542	3.77323	5.56089	4.71067	5.60832	4.71064	4.69151	4.45197	4.27698	4.5055	3.29708	3.89243	3.3962	3.7562
ZFP69B	1.50201	0.97232	0.99154	1.21253	3.61554	2.22388	3.75708	2.09709	0.93723	-0.17761	0.27739	0.86935	0.28838	0.65331	0.58259	0.8843
ZFP69	1.37023	1.00305	1.13415	1.41363	2.83449	2.1137	3.10403	2.23397	1.82542	1.64087	1.20358	2.05479	1.27579	1.09305	1.04405	0.9919
EXO5	2.26873	1.81105	1.71454	1.95263	3.93664	2.70506	3.75261	3.07035	2.91455	2.03344	2.50198	2.00944	2.83194	2.70643	2.64041	2.25658
ZNF684	0.99267	1.1326	0.54748	1.55441	2.0815	1.30776	2.25472	1.64966	0.79301	0.7021	0.33809	0.27939	0.69652	1.13312	0.6443	-0.00404
RIMS3	0.85252	1.1103	1.22451	1.8401	2.39433	1.27564	2.06192	0.78276	1.71351	1.94673	2.03584	2.23473	1.11036	1.69239	2.50896	2.67379
NFYC-AS1	2.18985	1.93278	1.98907	1.74518	3.95752	2.38314	3.89459	2.0143	3.27383	2.55268	2.80954	2.27913	2.24776	2.11698	2.54944	2.40814
NFYC	3.68656	3.5669	3.40595	3.53388	4.82984	4.26011	4.65444	4.44924	4.48088	3.89174	3.97118	4.06448	3.10029	3.18619	3.05556	3.1186
KCNQ4	0.92582	0.38722	0.36402	0.14689	-0.68947	-2.11206	-1.40695	-1.37	-2.80457	-2.26986	-2.66509	-2.88706	0.25119	0.7474	1.26478	0.63088
CITED4	5.4645	5.80654	6.11127	4.72956	3.02949	3.60229	2.04218	3.51015	6.24766	6.69255	6.66819	5.27757	7.73411	6.68774	7.8043	7.03314
CTPS1	4.12866	3.76645	3.71291	3.71403	5.64339	4.64455	5.98689	4.75251	5.17746	3.6312	4.53945	3.44313	5.27987	4.85058	4.82435	4.2874
SLFNL1-AS1	0.68144	0.41586	0.38607	-0.03251	0.80012	0.60625	-0.03594	1.01662	1.09071	0.34414	1.17682	0.33908	0.62675	-0.09994	0.54449	-0.22198

SLFNL1	-0.56439	-0.7243	-1.49003	-1.10867	0.10959	-0.55085	-0.99018	0.30471	-1.42622	-1.33936	-1.07981	-2.30453	-1.14341	-1.50213	-1.70693	-1.14734
SCMH1	2.57631	2.23371	2.27965	1.98391	2.34512	2.31333	1.9003	2.17018	3.67798	3.01986	3.81514	3.26505	2.88245	2.27131	3.15349	2.39053
FOXO6	0.22411	0.7947	-0.65105	-1.3733	-0.65271	0.97471	-1.99392	1.33641	-1.31847	0.29108	-1.90162	-0.10715	-1.84949	-1.31048	-0.35879	-0.16876
EDN2	-3.32193	-3.32193	-2.2365	-2.64585	-3.32193	-3.32193	-3.32193	-3.32193	-2.25097	-1.25278	-1.34253	-3.32193	2.10167	1.62958	1.44478	0.03451
HIVEP3	1.20155	0.68456	0.75226	-0.38	-2.56021	-2.54163	-2.58866	-2.91634	0.67	0.49101	-0.02597	1.17499	-0.48234	-0.65081	-0.0987	-0.53263
GUCA2A	-2.08069	-3.32193	-3.32193	-3.32193	2.34539	-0.10863	1.53659	1.14344	-3.32193	-3.32193	-3.32193	-3.32193	-3.32193	-3.32193	-3.32193	-3.32193
FOXJ3	3.02291	3.14492	3.26269	3.36139	2.5337	2.5482	3.09062	2.4796	3.46483	3.4366	3.48147	4.18696	3.38381	3.53893	3.62954	3.86284
RIMKLA	-3.32193	-3.18837	-3.1813	-3.32193	0.8267	0.5141	1.18264	0.1336	-1.17943	-1.00001	-2.20836	-1.01965	-0.40218	0.29467	1.00427	1.2644
ZMYND12	-2.55032	-3.32193	-2.81715	-2.78949	-2.64324	-1.84295	-2.59065	-2.81547	-0.05426	0.37113	-0.32924	0.68562	-1.51743	-1.64017	-2.57762	-1.06023
PPCS	3.60956	3.65671	3.94496	4.02264	4.11112	4.47755	4.17678	4.18917	4.56988	4.5514	4.7672	4.38701	4.18588	3.41596	3.33822	3.09612
CCDC30	-1.15012	-1.26503	-1.42523	-0.86292	-2.45015	-1.83578	-2.47015	-2.35326	-2.64274	-2.44904	-2.07741	-1.78242	-2.11669	-1.81629	-1.91801	-1.35237
PPIH	5.58949	5.04608	5.13953	4.70527	4.22704	4.34837	4.058	4.53469	5.63531	5.09388	5.2486	4.04053	4.83616	4.85005	4.1416	3.51467
P3H1	4.24125	3.48686	4.40633	3.40778	4.58442	4.557	4.415	4.25076	4.82476	3.54065	5.10361	3.30869	3.07563	1.9355	3.41232	2.05777
C1orf50	2.29084	2.27814	2.09373	2.07723	1.8427	2.48973	2.06817	2.47475	3.07008	3.16997	3.12278	2.91544	2.7595	3.15339	2.77976	2.92869
TMEM269	0.26349	0.07886	0.59338	-0.0383	0.76382	0.49565	0.79918	0.1548	1.36488	1.52232	1.63082	0.48081	0.46383	0.8063	0.82989	1.11172
SVBP	2.69167	2.14625	2.73249	2.42545	3.02188	2.88274	3.05592	2.48596	3.78308	3.83669	3.86883	3.04641	3.03629	3.0312	2.89936	3.20025
ERMAP	0.93358	0.79621	0.91606	1.06264	0.96918	0.80211	1.00615	0.56113	1.80305	2.00974	2.14503	1.75397	0.99325	1.02593	1.29404	1.16509
ZNF691	2.0631	1.85138	1.92723	2.17864	1.71069	2.19247	1.1371	2.60523	2.22151	2.17347	2.36588	2.02684	1.69409	1.81645	1.65864	1.37428
SLC2A1-AS1	0.27194	0.50657	1.15833	0.08561	-2.74669	-2.20667	-2.80519	-1.91785	-2.09489	-1.28115	-2.35401	-1.29973	-1.57789	-1.83597	-2.10678	-1.43372
EBNA1BP2	6.15239	5.68777	5.58568	5.28558	5.05717	4.60395	5.25723	4.45466	6.12553	5.10432	5.92094	4.36431	6.52752	5.36775	5.7603	4.47653
CFAP57	2.76957	2.57626	2.41045	1.82808	2.44696	1.17928	2.74386	0.83318	3.46059	2.44268	3.26081	1.28448	3.93255	2.8172	3.78703	2.45957
TIE1	4.66537	5.5141	5.36258	5.27632	-3.18642	-3.16201	-3.03925	-2.97052	-2.0907	-1.16298	-1.60732	-1.21847	-3.32193	-3.32193	-3.17046	-2.78331
CDC20	7.15511	6.56126	6.59874	5.6714	6.32714	5.43378	5.81782	5.70945	7.8184	6.52498	7.36395	5.27803	6.69982	6.18265	6.42411	5.58841
ELOVL1	5.66086	5.6979	5.86558	5.63388	6.68553	6.18797	6.48815	6.039	6.60161	6.34941	6.46372	5.79237	6.25782	5.86409	6.21851	5.95143
MED8	4.52206	4.41484	4.20387	4.54612	4.06834	4.74878	4.25518	4.29482	4.55586	4.05036	4.42484	4.45075	4.11475	3.81803	3.87567	3.45152
SZT2	2.70262	2.65283	2.81487	2.19294	1.5919	1.66644	0.78743	1.85186	2.76959	3.04241	3.02789	2.28534	1.74268	1.71554	1.88693	1.78211
SZT2-AS1	3.50008	3.50031	3.60904	3.46377	2.21008	2.26754	0.8968	2.85428	3.12879	2.92502	2.85428	2.81703	2.06638	1.52288	1.9695	1.31253
HYI	5.09848	5.17096	5.21521	4.29192	3.60479	4.26965	2.94268	4.48997	5.29088	5.74366	5.54527	4.31009	4.25181	4.29461	4.27061	4.11214
HYI-AS1	0.60794	0.62157	-0.368	0.23714	0.174	1.17617	-0.66265	1.40093	-0.39192	0.8056	0.35637	-0.36772	2.11753	1.73287	2.97268	2.46463
KDM4A	3.25836	2.97229	3.09502	3.06015	2.92261	2.48738	3.01261	2.51208	4.12992	3.61361	4.27969	4.14893	3.30983	2.99071	3.82615	3.6066
KDM4A-AS1	3.50001	3.41973	3.30876	3.38554	2.50203	2.65006	2.58262	2.71232	3.59772	2.97298	3.76009	3.35009	3.1308	2.85872	3.51353	3.05948
ST3GAL3	1.28734	1.40537	1.9433	2.03261	0.45274	0.75665	0.01881	0.57731	1.43241	1.90893	1.73951	1.73517	0.92049	1.13751	0.88845	1.00665
ARTN	-0.74567	-0.40455	0.09574	-0.61502	-2.00686	-0.8814	-2.97419	-1.40415	-0.88571	-0.21719	-0.93379	-1.91004	-2.38456	-1.71242	-2.06256	-2.96022
IPO13	3.4992	3.67991	3.43174	3.63425	3.48652	3.10281	3.02476	3.43496	3.90256	3.91682	4.10822	4.19451	2.23432	2.91414	2.89955	3.51967
DPH2	5.05359	4.32336	4.32391	3.84921	4.81428	3.73664	4.11757	3.74707	4.37357	3.23622	4.41913	2.86926	4.27639	3.11449	3.80574	2.66802
ATP6V0B	6.2082	6.11677	5.99256	6.27686	6.29857	6.76767	6.15035	6.4794	4.70765	4.82167	4.79478	4.14296	4.94981	5.15361	4.52296	4.68629
B4GALT2	4.97911	4.938	4.89206	3.76903	5.34664	4.8578	4.21541	4.91891	6.49112	6.32158	6.64269	4.98194	4.45775	4.57823	4.96337	5.05793
CCDC24	1.1062	1.47077	2.11007	1.87039	2.36069	3.1942	1.12381	3.06795	1.22265	2.00606	1.83239	1.34646	0.93621	1.46436	0.95217	1.33882
SLC6A9	1.14154	0.93111	1.31515	0.3533	1.77771	1.91875	1.23072	2.00028	1.83282	1.50681	1.00096	0.55495	1.34465	1.18436	1.10698	1.95345
DMAP1	3.10388	2.7535	2.90498	3.10803	2.14827	2.82937	1.74875	2.81243	2.89358	2.82868	3.05053	2.85509	2.05334	2.38359	2.38819	2.67585
ERI3	5.07998	4.85223	4.7735	4.78165	4.20964	4.27179	3.80478	3.98397	5.28258	4.91299	4.97475	4.8444	4.52206	4.44737	4.57796	4.19632
RNF220	3.20063	2.93449	2.8327	2.68517	2.83184	3.04144	2.50186	2.88931	3.38752	3.30353	3.56309	2.42115	3.31809	3.26874	3.53188	3.27235
TMEM53	3.61207	3.50023	3.29847	3.19904	3.98569	4.41273	3.51856	4.13313	3.985	4.04342	4.38331	3.1966	3.65481	3.80572	4.09431	3.68154
ARMH1	-2.74634	-2.97126	-3.32193	-1.97294	-2.15242	0.1903	-3.32193	-0.08075	-2.22447	-1.91336	-2.31057	-2.06229	-2.85298	-2.09568	-3.01853	-2.75784
KIF2C	4.71372	4.23143	4.32541	3.73275	4.69499	4.03118	4.62755	4.13088	5.97947	4.81979	5.27802	4.02719	5.24563	5.14819	5.19862	4.90553
SNORD55	6.26195	5.95809	5.29198	5.34041	5.58181	5.29777	4.7691	6.04161	5.2216	4.43408	4.6568	4.05785	3.93554	3.61086	3.48736	3.63315
SNORD38A	4.40369	3.78545	4.79101	4.49152	3.25062	3.34897	3.38536	4.72656	1.42089	2.92298	2.54669	2.10579	3.61597	1.43113	0.21235	2.72069
SNORD38B	5.82937	4.96829	4.92087	4.87514	4.44273	5.93081	4.36768	6.04951	3.76178	2.766	3.278	2.16755	3.45814	3.95166	2.17756	2.78295
PLK3	4.24928	4.25019	3.90496	4.2626	1.37923	2.48054	1.3068	2.1629	2.89499	3.40572	3.51692	3.75802	1.46286	1.46702	0.50646	1.46256
TCTEX1D4	2.09441	2.15007	2.11031	1.61044	-1.31328	0.62278	-2.35883	0.2496	-0.10836	0.73934	1.20969	1.79703	-0.03418	-0.0388	-1.35347	-0.33124

BTBD19	2.5977	2.91156	3.27989	2.72537	-1.28975	-0.93355	-1.91784	-1.18139	-0.16086	0.93149	1.16805	1.86923	-2.42377	-1.0629	-2.815	-1.1705
EIF2B3	3.88962	3.34151	3.16923	3.70648	3.14809	2.98584	3.43072	3.01501	3.64431	3.08805	3.38837	2.71612	3.77996	3.36891	3.23233	2.96848
HECTD3	4.70023	4.69234	4.6419	4.6143	4.25527	4.46862	4.14638	4.33309	4.97636	4.91091	5.09974	5.24835	3.78919	4.09335	4.66971	4.80102
UROD	5.97437	5.86183	5.56881	5.32406	4.68847	5.2938	4.42125	5.14048	5.02593	5.33823	5.25234	4.71626	4.47661	4.6449	4.18117	4.45284
ZSWIM5	-2.72406	-2.53917	-2.9388	-3.10525	1.35854	0.77349	1.07113	1.20078	-2.51783	-1.47413	-2.83759	-1.70426	-3.18368	-3.18415	-2.8702	-2.86164
LINC01144	-0.73875	-0.9784	-2.26852	-1.8789	-0.36836	0.02545	-1.18794	0.53565	-3.32193	-2.7982	-1.79549	-2.42621	-2.54696	-2.27724	-3.32193	-1.13818
MUTYH	3.22532	3.0259	2.96196	2.68736	2.5256	2.58542	1.61604	2.80647	2.70393	2.36046	2.4126	1.97119	1.93266	2.09923	1.21281	1.41011
TOE1	3.89484	3.39076	3.12955	3.09302	3.06533	2.38479	2.96135	2.53346	2.95714	2.49589	2.93277	2.51977	2.64722	2.5742	1.79827	1.65003
TESK2	0.1127	-0.87359	0.13282	-0.1112	0.45425	-0.22449	-0.10671	-0.4361	1.03931	0.60343	1.20316	0.83451	0.19319	-0.54874	-0.22028	-1.42047
CCDC163	1.08873	0.8016	0.34174	0.08341	1.52294	2.02017	1.25467	1.88076	0.19797	-0.36096	-0.37569	-0.59853	-0.23473	-0.09685	-0.71155	-0.24857
MMACHC	6.44494	6.25436	6.11705	6.19038	5.5002	5.98537	5.26928	6.09247	5.63626	5.37475	5.45361	5.20127	5.93388	5.92955	5.05513	4.93701
AKR1A1	4.72437	4.58282	4.70025	4.6198	4.71657	5.64048	4.4844	5.43477	4.56952	4.8203	4.82579	4.53477	3.95334	4.20211	3.99853	4.19191
NASP	5.02046	4.62091	4.58267	4.62578	3.85849	4.01983	3.81966	4.1901	4.70882	3.79778	4.26762	3.85159	4.35443	4.32693	3.83766	3.96565
CCDC17	-1.15091	-1.36452	-0.67637	-0.47558	-0.58769	0.90389	-2.59593	0.40307	-2.08473	-0.5928	-1.03763	-0.71489	-1.04617	-0.99678	-1.13041	-0.39408
GPBP1L1	3.60655	3.37873	3.52114	3.63972	3.25886	2.68341	3.63569	2.48267	3.65128	3.28031	3.61183	3.98358	3.59307	2.75864	3.43653	2.79858
TMEM69	4.72162	4.77355	4.52016	5.04089	3.51416	4.01388	3.83776	4.03007	3.86416	4.09589	3.84536	4.54586	3.83894	4.15474	2.96097	3.41808
IPP	2.10787	2.15372	2.19129	2.2888	2.27558	2.06616	2.57679	2.46119	2.86934	2.53516	2.30429	3.02902	2.94148	2.93639	2.51602	2.74917
MAST2	3.62036	3.49012	3.62313	3.18475	4.40278	4.17327	4.23179	3.88141	4.51887	3.92666	4.23964	4.01904	4.32914	4.765	5.60605	5.45827
PIK3R3	-0.35696	0.40341	-0.33117	1.08612	1.7159	0.21287	2.01088	0.31744	2.07553	0.36938	2.05966	1.19184	2.43537	2.01763	3.86691	3.23128
TSPAN1	-1.01848	0.08035	-0.82023	0.87911	-0.23055	-0.91278	0.56354	-0.54531	1.00464	1.63461	1.32925	1.7373	-0.89733	0.6878	-0.12928	1.24659
POMGNT1	3.90943	3.98435	3.89058	4.01584	4.37508	3.82824	4.24353	3.76647	4.20131	4.41058	4.79476	4.30611	3.92113	3.77891	3.87986	4.07706
RAD54L	3.97356	3.5726	3.58944	3.06987	3.4389	2.93989	3.16416	3.47345	3.98954	2.7328	3.30558	2.11693	3.64376	3.80559	3.56027	3.17204
LRRC41	3.64285	3.5732	3.60691	3.54295	3.85811	3.39285	3.49129	3.48291	4.43084	4.29961	4.55877	4.55614	3.57789	3.37944	3.83604	3.66605
UQCRH	8.43045	8.51666	8.34599	8.32188	7.06493	7.60854	7.05756	7.43891	7.63135	7.6043	7.36844	6.44383	7.41184	7.34274	6.45838	6.31282
NSUN4	2.29626	2.12056	1.84655	2.30346	1.79695	1.47891	1.50472	1.43585	2.01118	1.94055	1.90168	2.33949	2.43464	1.90373	1.81619	2.11753
FAAH	-2.68115	-2.92818	-2.10475	-2.05183	-2.44915	-1.89107	-2.98739	-1.10224	-2.59951	-2.09049	-1.72385	-1.80829	-0.40145	0.35452	-0.19413	0.00051
FAAHP1	-2.92856	-1.53	-2.52059	-1.40738	-3.32193	-2.92475	-3.32193	-3.32193	-0.47514	-0.22488	-0.29621	-1.33222	-1.87885	-1.25806	-2.64517	-2.38257
DMBX1	0.41445	0.7348	1.45169	0.99609	-2.78063	-3.32193	-2.73666	-2.61042	-0.98575	-2.29781	-2.24433	-2.13904	-3.32193	-3.32193	-3.32193	-2.98675
MKNK1-AS1	0.88431	1.09142	1.32587	1.85853	1.35255	1.26952	1.49876	1.26489	1.85008	1.52882	1.94439	1.66422	1.4521	1.60825	0.63666	1.45051
MKNK1	1.92702	2.09312	2.12477	2.36815	1.93395	1.77634	1.86276	1.82036	2.27495	2.28298	2.34242	2.46114	1.72012	2.02025	1.37204	1.89635
MOB3C	1.12825	1.26128	1.03055	1.32274	0.40076	0.45772	0.14295	-0.10935	1.54649	1.8698	1.74919	1.88527	-0.08501	0.23306	0.33898	0.4379
ATPAF1	2.78834	2.34247	2.75635	2.66622	2.60968	1.93293	2.46893	1.60559	3.96289	3.11588	3.70068	3.31091	3.61546	2.59562	3.61649	2.38858
EFCAB14-AS1	2.16531	1.39824	2.56694	1.64148	4.0217	1.73329	4.67416	1.47881	3.97287	2.15613	3.76468	3.51586	4.15378	2.63713	4.0416	2.77481
EFCAB14	3.51709	2.51436	3.47461	2.79569	4.56238	2.97792	4.80829	2.67499	5.11296	2.84263	4.94376	3.64759	4.54221	2.9278	4.82222	3.18234
PDZK1IP1	-0.31897	-0.07689	-0.70949	0.95578	-3.32193	-3.32193	-3.32193	-2.60652	2.59928	3.14463	2.61788	0.70926	-0.36488	1.02723	-1.28919	-0.45002
STIL	2.26775	1.92292	1.90483	1.70991	1.89755	0.6549	2.46864	1.15515	3.39312	1.65944	2.38923	1.78673	3.14809	1.80339	2.68292	1.77575
CMPK1	5.3866	5.36329	5.24024	5.75101	5.48795	5.14218	6.13127	5.06835	5.53397	5.34537	5.27623	6.0161	5.64992	5.56441	5.45034	5.1691
FOXD2-AS1	0.31561	0.05302	0.21758	-0.31669	1.47512	1.21449	1.06512	1.08651	1.10399	0.69713	0.49963	0.34822	1.04472	1.23005	1.66517	1.81193
FOXD2	-0.29714	-0.5614	0.14153	-1.132	1.60415	0.29233	0.74902	0.54841	1.79783	1.65153	1.92508	-0.17314	-0.08101	-0.23164	0.43528	0.46453
SLC5A9	-3.32193	-3.06844	-3.05559	-3.32193	2.15503	2.40356	1.50168	3.14283	-3.06019	-3.32193	-3.32193	-3.32193	-3.32193	-3.14102	-3.10359	-2.73598
SPATA6	-1.88957	-1.34609	-1.73088	-0.78981	-0.22631	-0.86449	-0.04977	-0.38013	-0.38015	-0.18229	-0.17594	1.02334	0.16838	0.21532	0.41707	0.73863
FAF1	3.40214	3.23886	3.15953	3.21023	2.88756	2.81924	3.24505	2.63064	3.81653	3.36416	3.67952	3.80896	3.26323	3.49131	3.86068	3.33027
CDKN2C	3.49283	3.21553	3.58691	2.61446	3.84954	3.26769	2.91979	3.3504	4.75144	3.69817	3.89779	1.86709	3.6924	3.82191	4.028	3.76695
RNF11	4.79152	5.00093	4.99452	5.43154	4.03669	3.63122	4.70797	3.32271	5.61021	5.27676	5.37989	5.61128	5.37726	4.85199	5.42669	4.86006
TTC39A	-0.80456	-0.15165	-0.73481	0.2104	-0.62315	0.38625	-0.35665	0.03826	-3.06767	-2.8368	-2.72234	-2.42315	-1.9665	-1.67318	-1.15759	-0.81294
EPS15	2.96322	3.16782	3.09649	3.5075	2.68902	2.14203	3.20386	1.93911	3.89774	3.87637	3.71034	4.87929	3.83006	3.53586	3.45759	3.30939
OSBPL9	3.55614	3.66632	3.69611	4.09651	3.4698	3.71907	3.80175	3.45121	4.12164	3.99634	4.03664	4.59034	4.11862	4.15994	4.40511	4.19354
NRDC	4.80336	4.872	4.8561	5.13252	4.64963	4.47061	4.95825	4.37684	5.07147	4.64359	4.9336	5.23722	5.21562	5.12283	5.53027	5.35774
MIR761	4.40241	4.88897	4.23277	4.54528	4.83758	3.23422	5.54721	4.23844	5.00942	5.70344	5.73461	5.22926	4.37235	4.74412	4.33592	5.00866
RAB3B	1.20891	1.50592	1.33326	1.60621	3.21729	2.41903	4.06664	1.8394	3.69761	3.4802	3.55068	3.93252	4.75784	4.62063	5.17899	4.76532

TXNDC12	4.59415	4.35573	4.36047	4.38613	5.54089	5.6505	5.67558	5.16922	5.05648	4.84797	4.93438	4.64094	5.46663	5.15992	5.22083	4.91086
KTI12	4.54127	4.54075	4.46913	4.38988	3.74709	3.90931	3.39781	4.00378	3.80685	3.67387	3.93087	3.41992	3.86177	4.06905	3.67923	3.87599
BTF3L4	3.54676	3.53067	3.37729	3.6113	2.52051	2.90968	3.16452	2.91355	3.94441	3.86455	3.70014	3.9457	4.11228	4.25512	4.00867	3.61316
ZFYVE9	2.08468	2.12891	2.0972	2.34837	2.59615	1.25275	3.15256	1.11495	3.17963	2.52827	3.08299	3.69129	3.18945	2.59823	3.5368	3.22204
CC2D1B	3.73029	3.82292	4.00436	3.87536	3.62365	3.99181	3.2963	3.95723	4.27039	4.14685	4.42561	4.23948	3.30268	3.03417	3.40459	3.26439
ORC1	3.78847	3.23673	3.11484	2.67108	3.18589	1.79928	3.17053	2.38325	3.41975	1.89805	2.31897	1.66737	3.34939	3.10463	2.83623	2.43988
PRPF38A	3.54225	3.39551	3.31207	3.46521	2.92003	3.16834	3.19014	3.14221	3.67173	3.27057	3.72886	3.44708	3.51706	3.37273	3.45895	3.31307
TUT4	1.18902	1.0333	1.18882	1.4685	1.27604	1.04737	1.55357	1.2192	1.61219	1.40615	1.53077	2.26333	1.71995	1.43264	1.76668	1.56173
GPX7	-3.32193	-3.32193	-3.32193	-2.66255	-2.65764	-3.32193	-2.8075	-2.69348	-3.32193	-3.32193	-2.76076	-3.32193	-0.00155	0.47492	0.95639	1.01859
COA7	3.87317	3.63206	3.26529	3.5452	3.78404	3.3503	4.15015	3.29066	3.71104	3.57792	3.83287	3.69209	4.04134	4.28364	4.30835	3.95272
ZYG11B	1.20531	1.5905	1.53246	1.96368	2.51424	1.45012	2.93487	1.58745	2.69899	2.90762	2.75267	3.68809	2.73477	2.8661	3.0465	2.97801
RPS13P2	3.02096	3.12358	3.46108	1.17207	1.5486	2.61736	1.40467	2.05578	2.44713	3.16708	2.52471	1.87086	1.07348	0.97395	1.14871	0.67639
ZYG11A	0.62151	0.30106	0.21137	-0.23205	1.26501	1.23686	1.29946	1.39886	0.43213	-0.37773	-0.06428	-0.62459	2.39737	2.25473	2.08806	1.44037
ECHDC2	0.95932	0.86104	0.90451	1.4461	1.38848	2.22567	0.68596	2.40196	1.0993	1.50372	1.45062	1.38485	1.12828	1.36245	1.17501	1.67321
SCP2	2.53852	2.95746	2.96658	3.47493	3.70254	3.6854	4.21128	3.61066	3.56853	3.81895	3.52288	3.87426	3.02611	3.6872	3.17065	3.29461
TUBBP10	-2.18895	-1.73277	-1.38571	-1.06931	-0.84672	0.09093	-1.02975	-0.74496	-0.33386	0.10828	-0.35883	0.93335	-1.07738	1.00673	-0.15619	0.69029
CPT2	0.9682	1.28806	1.00015	1.66235	1.35784	1.53998	1.12393	1.3485	1.59328	1.68794	1.60701	1.92436	0.94174	1.53595	0.59534	1.31269
MAGOH	5.62905	5.54605	5.50708	5.27582	4.23269	4.76484	4.6067	4.6847	5.4123	5.09465	5.37059	3.62302	4.95712	4.96352	4.02749	3.67036
LRP8	2.438	2.18852	2.30199	2.47099	2.09236	1.83404	2.14477	1.66389	1.53874	0.71319	1.03677	0.4385	2.09321	1.723	1.75374	1.48662
NDC1	4.38212	3.93227	3.76947	3.94509	4.88074	3.62534	5.34559	3.5028	4.5164	3.7927	4.00635	3.85369	4.92211	4.81545	4.64509	4.42538
YIPF1	3.39413	3.70122	3.62621	3.98608	3.60955	3.70204	3.90976	3.6677	3.35065	3.1618	3.4134	4.01982	2.5416	2.79542	3.03252	3.09489
DIO1	-2.57328	-2.22081	-2.83348	-2.42763	-0.90689	0.8359	-1.49828	1.87177	-3.32193	-3.32193	-3.32193	-3.32193	-2.70567	-3.32193	-2.59996	-3.32193
HSPB11	4.0066	4.02253	4.2411	4.07411	3.21693	3.6761	3.26188	3.63832	3.71052	3.3449	3.33457	2.54902	3.86101	3.63454	3.0367	2.62338
LRRC42	4.09294	4.38123	4.49588	4.46765	4.44333	4.0333	4.45385	4.05662	4.90672	4.71523	4.71943	4.53042	4.41853	4.47339	3.97389	3.84102
HNRNPA3P12	1.05056	0.3188	0.48284	-1.40923	-1.286	-0.88118	-0.63213	-0.0463	0.36528	0.02187	-0.22371	-0.95654	-1.17397	-0.57785	-0.45922	-0.92276
TMEM59	3.5605	3.94139	3.99903	4.35081	4.14713	4.42797	4.39916	4.22464	3.76437	4.24205	3.8886	4.44181	2.7426	3.08583	2.70726	3.03788
TCEANC2	0.84565	0.85143	0.71539	0.69079	0.03799	0.50174	0.22157	0.55179	0.25111	-0.0849	0.27542	0.63933	0.02102	0.20895	0.01962	0.16486
CYB5RL	0.5338	-0.09033	-0.23109	0.01587	0.99864	0.34397	0.5089	0.37815	0.80521	0.40507	0.34594	0.43607	0.96054	0.23786	0.97053	0.43915
MRPL37	6.31966	6.02461	5.8872	5.5997	6.07817	5.76158	5.65552	5.54451	6.75074	6.25456	6.51087	5.83123	6.14913	5.68284	6.33003	5.88534
SSBP3	4.91281	4.53391	4.86246	4.55417	2.11063	2.38801	1.86717	2.48804	3.62286	3.11951	3.79036	3.20717	0.7502	0.84182	1.62155	1.54154
SSBP3-AS1	0.10577	-0.21163	-0.38498	0.09871	-1.99573	-1.83943	-1.63993	-1.68464	-2.56696	-2.0433	-2.63235	-1.89842	-3.02125	-2.56261	-2.4366	-1.3635
ACOT11	-0.67944	-0.87328	-0.95964	-0.70945	1.67251	1.16151	0.80447	1.48202	-0.49503	-0.77918	-0.44656	-0.74387	-3.23481	-3.32193	-2.78075	-3.21419
FAM151A	0.72104	-0.11169	0.08822	0.60135	2.90334	2.64262	2.16094	2.86442	0.34465	0.14774	-0.08604	-0.32694	-3.32193	-3.32193	-2.24555	-3.32193
MROH7-TTC4	2.63673	2.58733	2.53761	2.55153	2.4342	2.19962	2.40349	2.25527	2.36497	2.26858	2.44992	2.21577	2.54742	2.42789	2.46305	2.21746
TTC4	3.65523	3.59736	3.55179	3.55671	3.43929	3.21202	3.40547	3.25922	3.37249	3.28612	3.45297	3.21717	3.55718	3.45145	3.46269	3.22578
PARS2	2.19039	2.27825	2.36565	2.34135	1.93953	1.76979	1.03186	1.2277	2.17643	2.10742	2.13163	2.3899	1.11939	1.24591	1.57196	1.64774
DHCR24-DT	-0.30369	-1.60936	-3.32193	-0.43367	1.89506	2.0358	1.68936	1.64004	-0.80666	-1.34558	-1.68873	-0.37101	-0.44251	0.53244	-0.36208	-0.74898
PCSK9	-3.11302	-2.88921	-2.8686	-3.32193	3.46366	5.66219	2.1392	5.38348	-2.69587	-2.94285	-2.46827	-3.12317	2.95718	3.85694	4.25643	3.91287
USP24	2.79976	2.60738	2.57863	2.84455	3.96926	3.00584	4.51928	3.02741	3.69419	3.4871	3.60911	4.39662	3.81577	3.58486	4.35938	4.23438
LINC01767	0.30948	0.28555	-0.368	1.48369	1.1086	1.99551	0.6436	2.22444	-3.32193	-3.32193	-3.32193	-3.32193	-3.32193	-3.32193	-3.32193	-3.32193
PLPP3	3.42755	4.18153	3.44884	4.81475	4.44876	3.05216	4.71122	2.85651	-0.09503	0.38235	-0.32252	0.2792	0.64233	-0.05945	0.01604	0.95159
PRKAA2	0.98198	1.09099	0.86412	1.02699	2.48471	1.25714	3.64293	1.45672	-0.06138	0.1585	-0.5289	0.76277	0.38134	0.41914	0.63933	0.56216
C8A	-3.32193	-3.32193	-3.32193	-3.32193	2.19441	-0.65992	1.95688	-0.05589	-3.32193	-3.32193	-3.32193	-3.32193	-3.32193	-3.32193	-2.61791	-2.91978
DAB1	0.03622	-0.92407	-0.57229	-0.32021	-0.25493	0.07924	-0.08218	0.09245	-0.62196	-0.50464	-0.63655	-0.31862	-0.66276	-0.6856	-1.30638	-0.93943
RPS26P15	2.94163	2.42136	2.8011	-0.59272	2.6234	4.25107	1.12551	3.52437	3.15606	4.1131	2.43666	2.18267	1.24573	-0.68432	0.6695	0.69909
OMA1	2.08175	1.24653	1.43121	1.81795	1.83807	1.88283	2.05413	1.93519	1.59056	1.70501	1.59424	1.96329	1.65159	1.63151	0.79433	1.26418
TACSTD2	2.18798	2.43259	2.52883	2.09339	-2.44126	-3.32193	-2.9344	-3.32193	-3.32193	-3.32193	-3.32193	-2.92911	-3.32193	-3.32193	-3.32193	-3.32193
MYSM1	0.44264	0.14257	0.60375	0.76591	1.23468	1.90439	2.07363	1.95609	1.82447	2.26479	1.98187	3.06857	2.77719	2.59408	2.09707	2.30656
LINC01358	-1.54718	-0.69489	-1.09268	-1.20144	-3.32193	-3.32193	-3.17443	-3.32193	-0.25626	0.01704	0.31832	-0.30291	-2.97592	-2.87822	-3.32193	-2.57756
FGGY	1.04515	1.34079	1.32298	1.03962	-1.77478	-1.40671	-1.81361	-1.78802	1.68029	2.20742	1.83215	1.49552	-0.79967	-1.58248	-0.97763	-1.32689

HOOK1	0.10592	-0.17413	-0.06315	0.21208	2.17912	2.29149	2.99432	2.01221	-0.68823	-0.28659	-0.21521	0.39985	2.28881	2.50952	2.75843	2.42121
CYP2J2	-1.03103	-1.24989	-1.81344	-1.92225	2.75201	0.93424	2.9822	1.27234	-1.21127	-0.99859	-0.64882	-1.092	0.28506	0.3508	0.4213	0.18721
NFIA	0.64472	0.32631	0.27252	0.90716	0.52918	0.75524	0.57146	1.45898	-2.20977	-1.67393	-1.73855	-0.94457	0.70203	1.42832	0.88348	1.46528
TM2D1	2.02925	2.12043	2.47705	2.7458	2.36611	2.23986	2.62783	2.08759	1.58246	1.88578	1.47611	1.61409	2.30104	2.50304	1.51294	1.10157
PATJ	0.63405	0.80266	0.87016	1.24518	1.98887	1.135	2.46974	1.43613	0.79446	0.93834	0.79875	1.71669	1.31182	1.32169	1.9529	1.72974
USP1	4.54979	4.20716	4.21269	4.43378	4.24244	2.722	4.67662	2.77284	5.22202	3.92081	4.29428	3.91631	5.08457	4.80875	5.03697	4.44344
DOCK7	1.13345	1.24249	1.46172	1.47237	1.51629	0.68535	2.01054	0.62299	2.20656	2.03846	1.92405	2.97874	2.00412	1.87046	2.19184	2.19708
ATG4C	2.66424	2.59412	2.17161	2.74079	1.2111	1.53643	1.88259	1.63131	2.04685	2.05063	2.36211	2.13161	2.11203	2.0873	2.03662	1.35597
FOXD3-AS1	-1.24728	-2.53857	-0.84503	-3.32193	1.24171	1.20704	-0.51515	1.88426	2.73808	3.2411	3.11867	1.62501	-3.32193	-3.32193	-3.32193	-3.32193
FOXD3	-3.32193	-3.32193	-2.40998	-2.36633	0.39695	0.3827	-1.16069	1.06447	1.81414	2.33661	2.14301	0.75464	-3.32193	-3.32193	-2.88339	-3.32193
ALG6	1.80834	1.58676	1.70391	2.04197	2.43844	2.10467	2.982	2.20572	1.2049	0.90224	0.94049	1.49573	2.05008	1.49428	1.31045	1.4975
ITGB3BP	3.1118	3.21344	3.2323	3.07208	3.0018	2.52338	2.97274	2.67212	3.6201	3.79618	3.76458	3.51707	3.03322	2.92439	2.6247	2.48749
EFCAB7	-0.04272	-0.11295	-0.04322	0.62561	-0.11937	0.12502	0.45428	-0.18964	0.38073	0.15095	0.31236	0.80824	0.50773	0.80437	0.48449	-0.13611
PGM1	4.3892	5.05858	5.14	5.13138	4.66067	4.90003	4.68145	4.76949	5.35733	5.98337	5.89688	6.34333	2.93305	3.9671	3.91832	4.44891
ROR1	1.47585	1.03275	0.97251	1.17597	2.74147	-0.17958	2.78143	0.62617	2.41306	1.47243	1.95293	2.28606	-2.26079	-2.97633	-2.32688	-2.08713
ROR1-AS1	1.24948	1.4359	1.29924	1.53219	-2.96688	-3.32193	-3.32193	-2.29201	-3.32193	-3.04552	-3.32193	-2.44975	-3.32193	-3.32193	-3.32193	-3.32193
CACHD1	-1.02496	-1.91729	-1.65575	-0.71412	-2.93049	-3.1537	-2.56912	-3.32193	1.77695	1.66301	1.63374	2.36944	1.50625	1.34195	1.19149	1.74278
RAVER2	0.81738	0.81946	0.66404	1.30613	1.0915	0.91382	1.64136	0.83842	1.47258	1.85849	1.48652	2.97853	1.91696	2.9328	2.71053	2.93761
JAK1	4.91478	4.51174	4.8627	4.81149	5.83242	4.67403	6.22751	4.24311	6.35406	5.33563	6.01781	6.18614	5.84273	4.89043	6.002	4.90642
AK4	4.13019	4.46612	4.35988	4.39188	3.88135	4.28044	4.33116	4.26949	4.74875	5.09518	4.2175	5.71718	3.34279	3.96	4.04851	3.7952
DNAJC6	1.18673	1.18883	0.71986	1.2353	0.34033	-0.20534	0.87135	-0.38885	2.67915	1.99465	1.93903	2.21381	3.54122	3.46391	3.05582	2.3128
LEPROT	4.2588	4.16042	4.3761	4.64717	4.22538	3.59541	4.51451	3.32285	4.89713	4.17729	4.32391	4.42512	6.06481	5.18555	5.3839	4.50674
LEPR	0.80329	0.7204	1.0962	0.59762	1.53341	-0.63212	1.6298	-1.12899	2.03523	1.3043	1.4751	0.4387	2.57558	2.21247	2.71337	1.80897
PDE4B	-1.88579	-1.72951	-1.67694	-0.53657	0.38405	0.9915	0.50165	0.92965	-1.42915	-0.39969	-1.75961	-0.79421	-0.20635	-0.06626	-1.06737	-0.40135
WDR78	-2.46563	-2.04136	-1.84528	-1.71498	-2.44548	-2.4585	-2.60087	-2.25045	-1.7939	-1.78696	-1.60882	-0.37806	-1.14013	-1.39532	-0.94336	-1.47456
MIER1	2.10215	2.48994	2.56384	3.15945	2.55807	2.20336	3.47914	2.27236	3.40465	3.52564	3.47519	4.10452	2.66277	2.92446	2.4945	2.66115
SLC35D1	1.02834	1.25713	1.15952	1.76861	3.81588	3.18843	4.4269	3.1406	1.94407	2.26314	2.83183	3.24202	2.05846	2.33504	2.29929	2.17001
C1orf141	-0.12173	-0.30189	-0.03085	0.02426	-3.32193	-3.32193	-3.32193	-3.32193	-3.32193	-3.32193	-3.32193	-3.32193	-3.32193	-3.32193	-3.32193	-3.32193
GADD45A	6.21235	6.00503	6.11745	5.53499	2.75348	3.1475	2.77039	2.95868	6.57416	6.06261	6.52301	4.58478	4.54509	3.90457	3.40513	3.04671
GNG12	4.19924	3.95398	3.88708	4.22528	0.31537	-1.66225	0.39125	-2.20259	6.01238	5.31016	4.84861	6.11022	6.24428	5.27059	5.65504	5.36934
GNG12-AS1	0.33885	0.49294	0.82521	1.16929	-3.32193	-3.32193	-3.32193	-3.32193	-1.10626	-0.74473	-0.82604	0.01872	-1.04081	-0.94439	-0.656	-1.15286
WLS	4.82998	4.64339	5.00052	4.92073	-2.86903	-3.32193	-2.91679	-3.05103	4.28534	3.40374	3.92717	3.67919	3.53226	1.79168	3.73138	2.67527
DEPDC1	3.61431	3.04604	3.17375	2.79435	3.52433	1.74117	3.99694	1.96904	4.10291	2.08582	2.87137	2.14952	4.62965	2.45076	4.12373	2.24996
LRRC7	-2.39555	-2.81992	-2.36488	-2.76799	-0.3307	-1.50751	-0.55957	-1.24702	-2.42509	-1.98144	-2.14427	-2.51191	-2.86409	-2.663	-3.11814	-2.99027
LRRC40	3.44155	3.2145	3.38265	3.7165	4.37523	3.6971	4.73617	3.66805	4.02432	3.82163	3.75887	4.23463	3.97089	3.87985	3.67724	3.57822
SRSF11	4.60618	4.44785	4.57096	5.09985	4.17172	5.07429	4.31115	5.21034	3.92779	3.8942	4.02661	4.25744	4.09049	4.09083	3.73302	3.49144
ANKRD13C	3.25937	3.21346	3.33132	3.46447	3.20438	2.65907	3.55088	2.85036	2.92595	3.09096	3.2178	2.81748	3.24114	3.2461	3.49099	3.24219
HHLA3	1.87054	2.22105	2.40378	2.51411	1.36827	3.25394	1.33953	3.28827	2.03087	1.24248	2.06325	1.58017	1.80054	1.83617	1.59007	0.87611
CTH	3.086	2.8058	2.63096	3.12847	2.30268	4.21947	2.65816	3.79354	2.02194	1.31454	1.39576	1.07834	1.30314	1.05849	-0.10657	0.43829
ZRANB2-AS1	4.1762	3.79525	3.91874	4.27434	3.71008	3.78808	4.17037	3.63398	3.69758	4.00391	3.99073	4.18655	3.3975	3.06529	2.65694	2.59755
ZRANB2	4.37993	4.25679	4.32377	4.72325	4.01276	4.14842	4.41366	4.00333	4.00972	4.15034	4.21739	4.45926	3.50025	3.12156	2.93341	2.74711
NEGR1	-2.89179	-3.32193	-3.05198	-2.53045	-3.32193	-3.32193	-3.32193	-3.32193	-0.55911	-0.93159	-0.69952	-0.80593	-0.40039	0.12996	0.34688	-0.32093
LRRIQ3	-1.63701	-1.74222	-1.79107	-1.05793	-2.2575	-2.14928	-1.45532	-2.81445	-2.03391	-1.45451	-1.72765	-1.25429	-2.89208	-2.96953	-2.90142	-2.42299
FPGT	1.03534	0.69718	0.34012	0.71985	3.59247	2.10607	4.08143	2.08058	2.57258	2.60834	2.38306	2.60099	2.17698	1.80991	1.68706	1.62746
FPGT-TNNI3K	-1.74155	-1.95316	-2.33456	-2.52874	1.04893	-1.61525	1.18612	-1.75005	-0.07653	0.14756	-0.03452	-0.30827	-0.95501	-1.1954	-0.87984	-1.0099
CRYZ	3.6136	4.00268	3.81306	4.18654	6.79202	5.91783	7.4803	5.65867	3.8688	4.45901	3.86261	4.561	5.28019	5.68543	5.18504	5.01535
TYW3	2.64861	2.43878	2.43259	2.73064	4.64839	3.37878	5.28392	3.3044	2.28929	2.31151	2.46842	2.47695	3.97837	3.02141	3.4824	2.53853
SLC44A5	-2.24542	-1.98779	-1.58399	-1.4563	-2.21676	-2.62336	-1.70358	-2.79621	-1.66737	-0.62918	-0.71178	0.78542	-1.29021	-0.35308	-0.4208	0.08667
ACADM	4.08678	4.17165	3.97889	4.19945	5.45963	4.45204	5.89494	4.38448	3.72546	4.06328	3.73835	3.64369	4.07945	4.18798	3.36373	3.45663
RABGGTB	5.34595	5.11344	5.14311	5.06273	5.33742	5.32692	5.76198	5.45329	3.11166	3.59075	3.63292	2.98218	3.36755	3.55335	2.31465	2.57688

SNORD45C	3.57158	3.73685	3.55325	3.49339	4.30089	3.58733	5.78457	4.23237	2.79735	2.75093	2.85283	2.91661	4.02352	3.62886	2.518	1.57345
SNORD45A	4.28563	3.86974	3.94718	4.22977	4.30161	2.73103	4.59984	3.95284	2.13614	3.02125	2.28859	2.63954	3.93554	3.6278	1.85941	2.87051
SNORD45B	5.24139	4.73972	5.48534	5.30609	5.35209	5.03547	5.29799	5.1699	3.14905	3.07308	3.34343	2.85862	3.76679	3.35019	3.63808	3.78399
TPI1P1	4.18662	4.24155	4.36275	2.86493	3.60196	3.81969	2.48695	3.12364	4.90933	5.2103	4.63596	3.69502	2.02591	1.77065	1.83107	1.59864
ST6GALNAC5	0.79788	0.19176	0.18309	-0.07342	-3.11448	-3.32193	-3.32193	-3.32193	-1.68215	-2.0562	-1.70152	-3.09223	1.32795	-0.27784	0.27794	-1.31462
PIGK	2.49742	2.3031	2.3515	2.59781	4.43099	3.47655	4.84931	3.64281	3.4805	3.30961	2.95542	3.61252	4.43782	4.15418	3.58601	3.62857
AK5	-2.23669	-1.85588	-1.87429	-1.49455	-3.25478	-3.32193	-3.17827	-2.83624	1.90216	2.22512	1.6475	1.77754	2.49026	2.76899	2.833	2.80433
USP33	3.4423	3.51671	3.47661	4.15637	4.13639	3.30418	4.67096	3.24296	3.79124	3.84818	3.69403	4.64187	4.16598	4.45332	4.11378	4.30711
MIGA1	1.92177	2.0403	2.04114	2.48395	3.98853	2.72836	4.89389	2.60293	2.0703	2.00015	2.28097	2.71478	3.32001	2.73992	3.37037	2.91541
RNA5SP21	1.73719	0.01066	-3.32193	1.09842	4.8721	2.06594	5.27005	1.98052	2.26261	1.99927	0.82784	1.26258	1.00008	-0.45183	1.67243	-0.16983
NEXN-AS1	1.34374	1.70247	1.54013	1.18912	0.5662	0.88447	0.95105	1.16003	-2.19707	-2.11646	-1.68746	-2.13034	1.34518	1.23075	1.44293	1.01193
NEXN	2.79513	2.89235	2.59172	3.3744	1.41336	1.76891	2.08129	2.40099	-2.63478	-2.43943	-1.9262	-1.91126	3.07617	2.90477	3.07074	2.12711
FUBP1	4.36593	3.78807	3.69337	3.97088	4.91592	4.42331	5.24543	4.22668	5.0217	4.3553	4.96304	4.49684	5.32111	4.86399	4.92236	4.39642
DNAJB4	2.14025	1.9288	1.68309	2.7451	3.91771	2.55762	4.46748	2.6771	5.27812	4.77513	5.14661	4.45174	3.51492	2.59114	2.69287	2.2218
GIPC2	-2.65649	-1.86036	-1.93234	-1.87501	-0.11817	-1.40921	-0.43789	-1.58923	-1.10997	-1.01279	-0.58504	-1.47688	-1.14562	-2.27406	-2.11681	-1.17416
PTGFR	-1.01409	-1.33762	-0.54548	0.23975	-3.32193	-3.32193	-3.32193	-3.32193	0.65404	0.99225	0.85246	1.47745	-3.32193	-3.32193	-3.32193	-3.32193
IFI44L	-1.90647	-1.60082	-1.50872	-1.36943	-3.32193	-3.32193	-3.32193	-3.32193	-3.32193	-3.03592	-3.21288	-1.66404	-3.23871	-3.239	-3.32193	-2.65135
IFI44	5.76771	5.90959	6.0894	6.12137	-3.10564	-2.852	-2.88313	-1.83042	1.63694	2.45897	2.39728	3.24588	-1.56201	-0.72407	-3.32193	-0.77813
ADGRL4	3.43034	3.78343	3.81543	3.97158	-3.32193	-2.80912	-3.06247	-2.99878	-3.32193	-3.32193	-3.32193	-3.32193	-3.32193	-3.32193	-3.32193	-3.32193
ADGRL2	-0.51342	-0.69716	-0.5795	-0.47563	5.39122	5.13404	6.21026	4.99489	2.49815	2.80361	3.05883	3.56646	2.99653	3.50085	3.94215	4.02721
TTLL7	-0.17189	0.1372	-0.1206	0.87799	0.8611	-0.08764	1.65579	0.02767	-0.44773	-0.13726	-0.69299	1.03354	2.40967	2.46443	2.31751	2.19393
PRKACB	1.25302	1.3918	1.35572	2.00583	3.62923	1.7253	4.41103	1.33005	2.25647	2.90167	2.46253	3.83034	4.25686	4.19188	4.39443	3.95795
SAMD13	-2.7866	-2.99754	-2.9815	-2.96175	-0.70091	-1.18218	-0.4559	-0.85148	-2.48736	-0.84971	-1.92743	-1.40797	0.02167	1.2076	0.44931	0.28451
RPF1	4.99749	4.82686	4.63168	4.80424	5.49806	5.03946	5.52906	5.0656	5.33641	5.12785	4.95396	5.03751	5.16107	5.3741	4.74269	4.41075
GNG5	7.16028	7.19952	7.27049	7.28753	7.77894	8.25144	7.90128	8.12299	7.27283	6.99365	6.71043	6.64304	7.789	7.82118	7.18819	7.17078
SPATA1	1.90492	2.09506	2.44039	2.95344	2.42433	2.41783	2.86668	2.23745	1.54815	1.84689	1.61734	2.31676	2.5423	2.45397	2.05956	1.91191
CTBS	1.3375	1.84399	2.01277	2.79955	2.29772	2.11796	2.57461	1.81516	1.04484	1.72862	1.35412	2.55811	2.10048	2.1757	1.51894	1.65818
SSX2IP	1.6697	1.73848	1.24309	1.85884	3.42512	2.09706	3.96632	2.10414	2.1953	1.90581	1.38046	2.18451	4.10576	4.23029	3.83317	3.80783
LPAR3	-3.03616	-3.00477	-2.29471	-2.96969	1.3221	-3.32193	0.65282	-2.71681	4.33008	4.48176	4.43685	4.81322	0.77668	-0.28944	-0.41821	-0.31215
MCOLN2	-3.32193	-3.32193	-2.98035	-3.32193	0.06774	-0.90734	-0.1523	-0.84702	1.23512	0.91301	0.9426	1.26819	-1.60101	-2.88679	-3.04064	-3.32193
WDR63	-3.32193	-3.32193	-3.32193	-3.32193	-2.99761	-2.83644	-2.86845	-2.7655	-1.41138	-1.37272	-0.91487	0.15416	-1.23282	-1.13539	-1.79142	-0.59971
MCOLN3	-1.48274	-1.3608	-1.30247	-0.50117	0.97552	0.72433	1.56848	0.79141	-0.86381	-0.07778	-0.62976	0.74631	0.19735	0.66172	0.10217	0.53547
SYDE2	-0.46273	-0.73784	-1.27378	-1.10621	1.66489	-0.14746	1.90683	0.10174	0.55627	-0.1512	0.49432	0.20074	1.25724	0.50066	0.58628	-0.42327
C1orf52	3.548	3.57824	3.41495	3.73721	3.35604	3.58321	3.62076	3.37628	2.77113	3.31434	3.01669	3.04502	3.76868	3.61295	2.84064	2.863
BCL10	3.48483	3.87937	3.65449	3.85343	3.02171	2.94475	3.85202	2.55977	4.06255	4.19893	3.98324	5.03002	3.51124	4.1068	3.78057	3.79803
DDAH1	3.82159	3.43403	2.38636	3.00523	5.21024	4.80067	5.02467	4.93013	5.70847	4.65033	3.23038	4.50015	7.04207	6.87094	5.80441	6.02541
ZNHIT6	2.5419	2.86457	2.30365	2.61815	2.95694	2.69363	3.57945	2.92181	3.09726	3.62174	3.3237	3.55571	2.14632	2.93502	1.55869	1.86081
ODF2L	1.246	1.49045	1.61819	2.17162	1.63527	1.14389	2.48138	0.81577	0.87843	0.80585	0.97565	1.57994	0.91912	0.46909	0.41218	0.35779
CLCA2	-3.32193	-3.32193	-3.32193	-3.32193	-3.32193	-3.32193	-3.32193	-3.32193	-2.31958	-1.10093	-1.84255	0.54426	-3.32193	-3.32193	-3.32193	-3.32193
SH3GLB1	3.57129	3.77842	3.79184	4.32646	4.34992	4.04431	4.99053	3.88786	4.75853	4.67899	4.69289	5.3579	4.69845	4.43307	4.75549	4.50654
SELENOF	6.00544	6.00315	6.07819	6.2654	6.94526	6.08216	7.46025	5.43754	6.15862	5.9322	6.09312	5.44701	6.55205	5.78543	5.73717	5.07475
HS2ST1	2.4815	2.85387	2.78813	3.40932	4.71481	3.82689	5.48676	3.74237	2.59119	3.34398	3.14657	3.58073	3.37002	3.96154	4.14851	4.3881
LMO4	3.57477	3.75288	3.74803	4.00213	3.27898	3.48006	3.32355	3.17406	3.16133	2.90505	2.75061	2.61574	3.51092	4.03262	3.39116	3.22219
PKN2	2.49192	2.70125	2.61493	2.98943	4.54393	2.65814	5.23867	2.75819	3.79831	3.37066	3.66429	4.36108	4.57219	4.13596	4.55393	4.21325
GTF2B	3.82704	3.85486	3.64834	4.2338	3.69753	3.96839	3.88603	3.79865	3.98103	4.10045	4.05117	3.92085	3.46349	3.70122	2.72119	3.19985
KYAT3	3.4776	3.73571	3.82532	3.86255	3.93899	3.68493	4.45306	3.30855	2.94288	3.04939	2.83652	3.4484	3.80146	3.57251	3.68449	3.25192
RBMXL1	2.108	2.12565	1.94847	2.12955	3.25804	2.58691	3.76865	2.64426	3.11824	2.39212	2.88792	3.08311	3.43424	3.26547	3.32688	3.17524
GBP3	2.73084	2.7591	2.75593	3.10898	1.50515	-0.47212	2.29426	-0.60262	-0.15555	0.43726	0.8798	0.75907	3.76396	3.61352	2.91312	3.15019
GBP1	2.72898	2.49805	2.40943	3.29215	0.95281	-1.57436	1.2588	-2.01196	2.9225	2.63618	2.8225	3.55199	3.14293	3.62313	2.89391	3.42025
PTGES3P1	2.74162	3.15987	2.33618	2.84796	2.30608	1.34312	1.82775	1.13905	2.55828	2.5821	2.03389	2.2234	0.68764	2.31965	1.59212	2.35555

GBP2	-0.22709	0.47598	0.44358	2.14547	2.2569	2.73916	2.76451	2.58807	0.08825	1.20547	0.39729	2.38491	1.87392	2.84128	2.58579	3.5496
GBP4	0.5288	0.41894	0.44424	1.72169	-3.24633	-3.14807	-3.32193	-3.32193	1.52009	1.40759	1.83893	2.82312	-2.74063	-2.4811	-3.0094	-3.0032
GBP5	0.23514	-0.06518	0.28438	1.20281	-3.15658	-2.85156	-2.62347	-2.57331	-1.36571	-1.21467	-1.70206	-0.70129	-3.32193	-3.32193	-3.32193	-3.32193
LRRC8B	-0.54394	-0.71521	-1.12577	-0.73446	1.9829	1.53579	2.43568	1.36961	1.01595	0.60554	-0.13206	0.99294	3.04975	3.45694	2.74703	3.15641
LRRC8C-DT	-1.63029	-1.20502	-0.88799	-1.39008	0.90156	0.05596	1.52392	-0.56759	0.68902	0.14224	-0.83116	0.41501	1.96893	2.56153	1.8102	2.11902
LRRC8C	0.27757	0.59622	0.36972	1.0512	-2.0221	-2.97968	-1.68921	-2.92692	1.69575	1.1789	1.09543	1.7418	0.99492	0.4771	1.09435	0.64154
LRRC8D	2.71421	2.62909	2.37788	2.83334	4.4065	3.96738	4.59599	3.82955	3.2264	3.48816	3.17763	4.16209	3.3019	3.29497	3.24211	3.14938
GEMIN8P4	1.34975	-0.29164	0.14931	0.88534	1.97941	2.2739	1.75679	2.76794	0.41953	0.52066	0.81093	0.14961	1.59418	1.77584	1.34455	1.31589
ZNF326	2.34525	2.05953	2.13841	2.52936	2.20489	1.57833	2.42667	1.83971	2.07264	1.37506	1.71229	2.09303	2.33437	2.14551	1.99975	1.9835
LINC02609	0.58536	0.68686	0.74138	0.82768	-1.44978	-0.41107	-1.87832	-0.35954	-0.51518	0.24352	-0.99752	-0.52716	-0.11585	0.20171	-0.88946	0.12299
ZNF644	2.23052	2.17332	2.29296	2.79266	3.25091	1.89216	3.9837	1.98569	3.03407	3.26714	2.82345	4.16919	3.67514	3.60728	3.79182	3.86221
CDC7	2.92713	2.58549	2.64439	2.92453	3.57586	2.90506	3.88604	3.13653	2.28335	1.76696	2.01186	1.23551	3.4285	3.72787	3.0581	2.96889
TGFBR3	-0.39636	-0.64299	-0.42599	0.23941	3.37371	2.30474	3.86848	2.42789	1.01976	-0.56484	0.35866	-0.10732	3.11727	1.76808	3.77908	2.82686
EPHX4	-3.32193	-3.32193	-3.32193	-3.32193	-0.07504	-1.15648	-0.25114	-0.8596	-1.11813	-0.45065	-2.04173	-0.76051	-3.32193	-3.32193	-3.32193	-2.31516
SETSIP	1.70449	2.20294	1.60096	2.36903	-1.3581	-0.33895	-1.00435	-2.31076	-1.33013	-0.11507	-0.66497	-1.23866	-2.58303	-1.00025	-0.97826	-0.23465
BTBD8	-1.53908	-0.90886	-1.15175	-0.32661	0.55002	-0.39123	0.96113	-0.1122	-0.34568	0.17412	-0.3856	1.3003	-1.68331	-0.96804	-2.01628	-0.34088
GLMN	2.35449	2.20797	1.91277	3.03221	3.41526	3.0433	4.1579	3.02813	2.23853	2.0843	2.04749	2.34509	3.18012	3.11041	2.49203	2.48999
RPAP2	-0.10694	-0.0155	0.12031	0.37883	1.01916	0.20524	1.62665	0.51615	0.65884	0.11996	0.77691	1.28895	1.41579	0.12384	1.49518	0.27381
GFI1	-1.2204	-0.34716	-0.73363	-0.95585	-0.47573	-0.13884	-1.01438	0.34997	0.75282	0.75451	-0.49632	0.35763	-2.68725	-2.68908	-2.78691	-2.77706
EVI5	1.51929	1.64198	1.45641	1.77682	2.55169	1.61721	2.84435	1.78036	1.96103	1.85871	1.86245	2.94674	2.49559	2.5745	1.99813	2.04819
DIPK1A	3.12723	2.62934	3.14982	2.92834	4.86324	3.41427	5.40378	3.15545	2.48205	1.58761	2.91442	2.63632	-0.66401	-0.62409	-0.56629	-0.88615
MTF2	2.68501	2.82234	2.60666	3.33842	3.68455	3.31908	4.3671	3.47022	2.69295	2.56092	3.11185	2.83493	3.52114	3.95138	3.85135	3.74051
TMED5	3.53889	3.51758	3.49976	4.11078	5.64345	4.68272	6.43091	4.35177	3.77856	3.29477	3.55314	3.82479	5.04251	4.34675	4.79763	4.04412
CCDC18	3.12289	2.99485	3.1588	3.39011	5.00093	3.30848	5.02793	3.22131	3.42412	2.86022	3.15024	2.76843	3.72762	3.31033	3.83865	3.4862
CCDC18-AS1	1.2252	1.37496	1.68363	1.96633	3.02578	1.89667	3.11059	1.80517	2.07353	1.99774	1.89865	0.94816	2.01378	2.19975	1.93785	1.92716
DR1	3.58686	3.6052	3.4606	3.2364	4.52174	3.47173	4.51568	3.34579	3.96255	3.68501	3.64756	3.29132	3.91749	3.90174	3.6677	3.6394
FNBP1L	1.76255	2.42185	2.46148	2.80972	4.83245	3.98059	5.79839	3.69827	3.48663	3.95414	3.34982	4.64664	4.74246	5.1613	5.35023	5.35985
BCAR3	4.17994	3.99644	4.06923	3.75161	2.71205	2.18624	2.95577	1.57263	5.38814	5.11317	5.69482	5.07684	5.74728	4.94889	5.49132	5.14693
DNTTIP2	4.73207	4.6748	4.29854	4.92706	4.43051	3.86339	4.904	4.00433	3.74091	3.73858	3.5877	4.15552	4.10867	4.13565	3.83507	3.77276
GCLM	4.8056	4.74309	4.6979	5.32244	5.8707	4.47113	6.49361	4.13403	3.52999	2.38866	3.09184	2.97043	4.46665	2.54461	3.99259	2.24469
ABCA4	-2.67316	-2.70735	-2.39824	-1.96967	-1.79132	-1.48056	-1.94892	-1.43382	-3.17137	-3.32193	-3.32193	-3.19798	-3.03218	-3.2191	-2.97589	-3.32193
ARHGAP29	1.75688	1.94638	1.91996	1.92301	3.18227	2.2496	4.08295	2.49318	3.82643	3.77	3.50187	3.79863	4.81756	4.91247	5.08563	4.28815
ABCD3	2.65145	2.7697	2.66095	3.33177	5.82862	4.57042	6.28469	4.67529	3.51578	3.22295	2.85658	3.72275	4.03657	4.26765	3.86661	4.13972
F3	5.05648	5.51894	6.87778	5.24778	2.4074	2.38718	3.16104	2.49948	4.01487	4.4435	3.29749	3.53807	2.07863	2.04442	1.81883	1.50577
SLC44A3-AS1	-1.67984	-1.45962	-1.18099	-1.52015	-1.3482	-0.25961	-1.64741	-0.1489	-2.34475	-1.32369	-2.36458	-1.69775	-2.08007	-1.64923	-1.90858	-1.509
SLC44A3	-1.33071	-0.84827	-0.43249	-0.12091	4.49356	3.93616	4.46558	3.89421	1.26028	1.276	0.92109	1.74714	1.86365	2.2273	2.53803	2.40616
CNN3	5.69223	5.51936	5.71217	4.94445	6.31872	5.48131	6.6979	5.22405	7.30339	6.70886	7.09914	6.4248	7.32514	7.01316	7.69582	6.58438
ALG14	-0.13013	-0.43039	-0.0603	0.00751	2.23127	1.61597	2.32942	1.31947	-0.57505	-0.5672	0.02589	-0.46135	0.62802	-0.40715	0.41226	-0.48748
RWDD3	2.239	2.36582	2.17224	2.55673	3.27491	3.81517	3.45845	3.781	2.57811	3.02555	2.6935	2.68882	3.27374	3.57653	2.77967	2.31942
EEF1A1P11	7.32108	6.87425	6.956	6.08056	5.20023	6.63215	4.81025	6.33596	6.27041	7.38763	6.30688	4.68284	4.69883	3.92526	4.81015	4.90073
RPL7P9	5.58488	5.57882	5.45277	5.20262	4.68826	4.74321	5.05186	4.44954	5.31149	6.14227	5.47393	5.71423	4.83738	4.45936	4.56736	4.65792
PTBP2	0.33097	0.60482	0.5079	1.22291	1.52211	1.77055	2.31868	2.16619	0.66476	1.7546	1.16614	2.09957	0.59902	1.73208	0.67351	1.08789
DPYD	0.47396	0.76164	0.8466	1.29693	-2.88032	-2.53052	-2.90258	-2.95842	3.64772	3.77756	3.84932	4.21188	2.06567	2.86292	2.12659	2.22428
DPYD-AS1	-1.95776	-0.80794	-0.24121	-1.4541	-3.32193	-3.32193	-3.32193	-3.32193	2.29827	2.1707	2.59107	2.06325	0.27501	1.04705	0.63501	0.99621
MIR137HG	-2.84926	-2.95446	-3.32193	-3.32193	-3.24485	-3.32193	-3.32193	-3.32193	2.50018	3.26607	3.1443	3.14515	0.11371	1.55319	0.20001	0.72342
MIR2682	0.59563	-3.32193	-3.32193	-3.32193	-3.32193	-3.32193	-3.32193	-3.32193	4.67376	5.42504	5.10657	5.57258	2.62769	2.96552	1.79418	2.93996
LINC01776	-2.29012	-3.32193	-1.91591	-3.32193	-3.32193	-3.32193	-3.32193	-3.32193	-1.23861	-1.19839	-0.72502	-0.83105	0.81883	0.51791	-0.91005	-1.07775
SNX7	4.07116	3.72322	4.37129	4.29144	5.28106	3.26168	5.47823	2.88354	5.28976	4.07588	5.11441	4.54936	5.33524	2.9433	5.1822	2.89305
PALMD	-3.32193	-3.32193	-3.16938	-3.15992	-2.96592	-2.95953	-3.20041	-2.78796	-3.32193	-3.32193	-3.32193	-3.32193	-1.60542	0.12716	0.48884	0.3722
FRRS1	0.55034	-0.32525	0.4685	0.71822	4.31503	1.38716	4.78046	1.66843	1.05828	0.17055	0.63695	0.59226	1.38689	0.97377	1.96093	1.24573

AGL	0.98351	1.02252	1.10407	1.51259	3.34314	2.05218	3.9844	1.99875	0.85712	1.17963	1.01159	1.97762	2.18528	2.6016	2.61314	2.87892
SLC35A3	0.81878	1.22659	1.19094	1.58543	2.45334	1.19537	2.60743	1.38154	1.1899	1.52513	0.05519	1.26516	2.32601	2.84115	1.11098	2.09123
MFSD14A	4.02612	3.93959	3.84581	4.51968	5.1499	3.59678	5.72593	3.38124	4.42394	4.15993	4.37338	4.6162	5.68311	5.5322	5.53537	5.41936
SASS6	2.11123	1.77972	1.62586	2.15102	2.77193	1.35036	3.5349	2.09089	2.27705	1.45307	1.64906	1.49722	3.44319	3.06478	2.9883	2.26026
TRMT13	2.03557	1.81332	1.84513	2.46074	1.84847	1.35008	2.23575	1.78673	1.35981	0.83516	1.07293	1.55116	2.37989	0.90595	1.33365	0.46757
LRRC39	1.50014	1.19387	1.2959	1.92249	1.04971	1.05009	0.96771	0.96687	-0.20045	-0.43631	0.20156	0.81798	1.72535	0.61375	1.22222	-0.03662
DBT	1.04051	1.17502	1.32181	1.97504	1.58436	0.7628	2.21148	0.68788	0.9823	1.005	1.02993	1.85662	2.38289	2.1301	2.16538	2.15342
RTCA	4.78113	5.0852	4.97573	5.31168	5.10331	4.59737	5.59994	4.54553	4.94415	4.8814	4.68668	4.88436	5.05113	5.09903	4.71054	4.71483
CDC14A	0.13964	0.44865	0.07849	0.37207	0.81576	0.00028	1.1568	0.03788	0.872	0.69231	0.45228	1.57926	1.53109	1.7429	1.26967	1.49321
VCAM1	0.86338	0.45133	0.3625	0.93667	2.49895	-0.60423	2.20638	-0.31833	-3.32193	-3.32193	-3.14189	-2.63651	-2.94169	-2.7362	-3.32193	-3.15174
EXTL2	2.19012	2.31197	2.17407	2.88687	3.1354	3.0499	3.72066	2.87155	2.94402	3.13513	2.84507	3.24888	3.13819	3.42673	2.76201	3.13423
SLC30A7	2.56311	2.32449	2.41585	2.93669	3.45541	2.47558	3.83554	2.45082	2.87109	1.56959	1.92433	2.7378	3.10078	1.46565	2.63736	1.73078
DPH5	3.07658	2.74403	2.78242	2.84652	3.71352	3.47821	3.84496	3.35072	4.18613	4.44602	4.10281	4.09335	4.26924	3.98256	3.39629	3.22997
S1PR1	-0.45045	-0.07251	-0.94571	1.16194	-2.9192	-3.32193	-2.76434	-2.94276	6.11192	5.09978	5.75785	5.01667	-0.95516	0.22076	-0.66466	0.90743
LINC01709	0.77811	0.12256	1.37459	-2.16131	0.04997	0.41893	-1.10337	0.19873	1.78909	3.60648	1.71973	1.68176	-0.20631	-0.57129	0.49385	0.57035
RPSAP19	3.36887	3.3915	3.5482	2.22183	2.26891	3.08443	1.40713	3.06818	4.03561	5.35182	3.99977	3.45538	2.15477	1.76754	2.0793	1.92633
COL11A1	-3.08026	-3.32193	-3.17394	-3.32193	-0.13098	2.5029	-0.26543	2.67828	-2.92363	-2.71071	-3.19171	-2.49172	-0.97947	0.18802	-0.22201	-0.25407
RNPC3	0.41353	0.69802	0.88025	1.37678	0.31872	0.49993	0.2973	0.00098	-0.00966	0.37724	0.64482	0.79851	0.19513	0.37842	-0.054	0.11319
AMY2B	-1.28341	-1.19351	-0.78909	-0.48453	-1.68403	-0.97556	-1.97291	-0.91237	-1.65849	-2.18515	-1.9379	-1.95932	-1.57609	-1.083	-2.25973	-1.50322
PRMT6	4.53862	3.59549	3.97969	3.64351	4.98533	3.40175	4.63403	3.2405	4.33169	2.72121	3.954	2.3363	3.50161	1.50228	3.50762	1.83764
VAV3	-2.06634	-1.71941	-1.72578	-1.86157	-0.79585	-1.61423	-0.3086	-1.30506	-3.32193	-3.32193	-3.32193	-3.32193	3.88846	4.29205	4.47081	3.95476
SLC25A24	2.01685	1.46505	2.36996	2.07034	3.96482	1.61356	4.96963	1.28702	4.30351	3.67659	4.30438	4.09503	3.20601	2.73356	3.38918	2.03228
NBPF4	-2.64363	-3.32193	-3.32193	-2.85843	1.20485	1.4381	1.31107	1.66446	-2.8902	-2.42026	-3.32193	-2.67205	-3.32193	-3.32193	-3.32193	-3.32193
SLC25A24P1	-3.32193	-3.32193	-3.32193	-3.32193	3.3904	3.08421	3.07951	2.99948	-3.32193	-2.83891	-3.32193	-2.84586	-3.32193	-3.32193	-3.32193	-3.32193
NBPF5P	-3.32193	-3.32193	-3.32193	-3.32193	1.38072	1.1058	1.02837	2.12218	-2.67879	-2.77003	-3.32193	-2.77781	-3.32193	-3.32193	-3.32193	-3.32193
SLC25A24P2	-3.32193	-3.32193	-3.32193	-3.32193	4.90496	3.95294	4.43908	4.00256	-3.32193	-3.32193	-3.32193	-2.03347	-3.32193	-3.32193	-3.32193	-3.32193
NBPF6	-2.94328	-3.32193	-3.32193	-3.32193	1.45339	1.33776	1.3748	1.84732	-3.32193	-2.03089	-2.93136	-2.22562	-3.32193	-3.32193	-3.32193	-3.32193
FAM102B	-0.57251	-0.60228	-0.27346	-0.17544	4.86531	2.37152	5.47915	2.3569	2.98563	2.16995	2.25821	2.53264	0.94589	0.31768	0.91243	0.13201
HENMT1	-3.32193	-3.32193	-2.22631	-3.32193	4.19751	3.49719	4.34884	3.63117	2.81077	2.87768	2.99777	2.83514	-0.03325	0.96205	-0.62194	-0.13335
PRPF38B	4.01592	4.08427	3.94496	4.45587	4.23697	4.53238	4.61605	4.91896	3.76802	3.8818	3.84161	3.90444	3.57935	4.1467	3.71497	3.81195
STXBP3	2.40612	2.26279	2.57269	3.34889	3.00217	2.71868	3.80982	2.88088	2.50345	2.32574	2.33654	3.04442	2.98143	2.60445	2.46077	2.2791
AKNAD1	-1.50367	-1.7614	-2.00195	-2.06166	-3.32193	-2.92214	-3.12356	-3.32193	-1.15605	-1.41855	-1.467	-1.03147	-3.00298	-3.32193	-3.11934	-3.32193
GPSM2	1.3305	1.35183	1.55497	1.75117	3.8672	3.38915	4.32084	3.63342	3.87803	3.18302	3.24357	3.52484	3.50128	3.80651	3.58865	3.3425
CLCC1	2.32464	2.31573	2.53272	2.78445	4.83324	4.3118	5.26177	4.56788	3.98864	3.37187	3.52997	3.93754	3.9459	4.14169	3.87326	3.92937
WDR47	1.70422	2.17997	2.0472	2.44323	2.53819	1.56385	2.91385	1.35771	2.75035	2.96329	2.42535	3.2578	3.1535	3.74426	3.65128	3.72431
TAF13	5.63565	5.9044	5.52795	5.92044	5.69956	5.3621	6.48663	5.18451	5.0343	5.10293	5.05471	4.93706	4.95728	5.24667	4.68468	4.74259
TMEM167B	1.83934	1.97382	1.94075	2.2474	3.56551	3.18696	3.83228	2.92363	2.72864	2.94338	2.73096	3.01409	2.63882	2.49488	2.06436	2.53509
SCARNA2	-0.25297	0.42703	0.25656	-1.58519	1.20005	1.79205	0.72458	2.59321	-0.06014	-0.01309	0.52609	-1.86155	0.75913	0.48366	-1.85502	-1.8348
KIAA1324	-1.95141	-1.62235	-2.3301	-2.21165	-2.61001	-2.37471	-2.3128	-1.94999	1.94273	0.64582	1.68361	0.33682	-2.157	-2.91513	-2.24094	-3.32193
CELSR2	-1.61592	-1.931	-1.56376	-2.19093	2.67672	1.30092	1.83951	1.49621	0.2928	1.12751	0.29438	1.44442	1.02505	1.70035	2.63981	3.20478
PSRC1	3.23223	2.1282	2.75845	2.07661	4.12729	3.53126	3.3396	3.65685	4.89888	3.86235	3.92548	2.61598	4.31824	4.40703	3.88658	3.69965
SORT1	2.01735	1.96496	2.36434	2.2585	5.05697	3.67858	5.5316	3.48746	3.08928	2.38032	2.79261	2.71502	4.49884	4.60651	5.58952	5.14053
PSMA5	4.87113	4.70057	4.68851	4.94362	5.43326	5.05523	5.37195	4.86678	5.27475	5.14311	5.26794	4.69117	4.72891	4.70985	4.15981	4.10396
SYPL2	-2.337	-2.1085	-2.21084	-1.33396	4.00252	3.31914	3.89795	3.22099	-2.78063	-2.85967	-2.83058	-2.86637	-3.32193	-2.50275	-2.23658	-2.35434
ATXN7L2	1.06197	0.74995	0.64031	0.89554	1.25897	2.21552	0.52921	2.76278	0.1148	0.19437	0.35983	-0.50493	-0.88466	-1.19819	-0.92992	-0.90433
CYB561D1	0.219	0.28405	0.1817	0.32618	1.62181	1.14407	1.87382	0.93889	0.59557	2.06263	1.64985	2.06561	0.82132	1.77041	2.01182	2.6221
AMIGO1	-0.85559	-0.4608	-1.01961	-0.41967	1.68735	-0.43302	1.05082	0.19011	1.44923	1.33065	1.40474	1.09157	1.23736	0.97155	1.61705	1.3388
GNAI3	1.93846	2.08903	2.17063	2.32192	2.91977	1.91358	3.56444	1.62893	3.45363	3.41331	3.28123	3.73506	2.90983	2.97147	2.98364	2.9117
GNAT2	1.00777	1.55511	1.24109	1.36913	3.61178	1.02287	3.35398	1.16288	2.48214	3.01193	2.6353	3.32331	2.08362	2.35089	2.74412	2.39377
AMPD2	3.91139	4.03598	4.05735	3.91294	5.38915	4.98401	4.71611	4.91405	4.14985	5.02441	4.71933	4.7089	3.37752	3.84942	3.9017	4.24149

GSTM4	1.2281	1.31949	1.11413	1.46492	3.7771	4.12829	3.13975	4.06165	3.5663	3.7358	3.54348	3.33843	-0.26834	0.381	0.27411	0.68069
GSTM2	-0.67149	-0.86478	-1.38676	-1.35774	2.24683	2.80279	1.64518	2.8128	2.91871	2.93759	2.76805	2.63829	-3.05877	-2.83777	-2.67208	-2.51643
GSTM1	1.92888	1.71827	1.39268	1.26377	2.206	3.05585	1.44597	3.09008	5.26913	5.12633	5.03892	4.86859	-2.75234	-3.0102	-2.65293	-3.32193
GSTM3	2.49127	2.1821	2.15558	2.42219	4.67507	4.60987	4.40346	4.44293	3.34566	3.43484	3.10385	3.28082	3.69367	3.43827	2.88769	2.76826
EPS8L3	-3.32193	-3.32193	-2.70944	-3.32193	5.82638	5.17201	5.19887	5.08987	-3.32193	-3.32193	-3.32193	-3.32193	-3.32193	-3.32193	-3.32193	-3.32193
CSF1	6.25918	6.26713	6.24136	6.07832	4.19159	4.00547	3.89226	3.67348	3.49474	4.03439	4.33789	4.22216	3.09791	3.49745	2.94821	3.15718
AHCYL1	4.34368	4.3499	4.32506	4.50049	5.63427	4.64702	5.89954	4.3791	5.33938	4.82826	5.36419	5.64024	4.78691	4.42151	5.28907	4.82369
STRIP1	1.42566	1.33179	1.34414	1.56165	2.91676	2.26193	2.80486	2.30646	2.66578	2.54881	3.05155	2.89444	2.50442	2.04607	2.69598	2.55312
ALX3	1.0147	0.44913	0.24872	-0.80002	1.25539	1.08825	0.73016	1.25352	1.44387	0.93482	1.17791	1.18701	0.94982	1.03947	2.05857	1.78275
SLC6A17	-2.88849	-2.98596	-2.82094	-2.94905	-2.40124	-2.36225	-2.50366	-2.6843	-0.57716	0.06518	-0.43174	-0.12111	-3.19559	-3.08023	-3.32193	-3.16618
KCNC4	-3.11408	-3.09058	-3.21969	-2.9727	0.15173	-0.4056	-0.19211	-0.27543	0.25719	0.86272	0.78356	0.7962	-2.5946	-2.22626	-2.15663	-1.31313
RBM15-AS1	-1.44408	-1.90607	-2.88666	-1.62082	0.3917	0.00234	0.24137	0.45711	-1.28293	-1.17148	-1.81218	-2.05352	-1.9268	-1.40181	-1.4924	-1.86751
RBM15	2.19238	2.44226	2.317	2.49	3.33247	1.84603	3.19774	1.73207	2.86132	2.08226	2.78671	2.11181	2.76113	2.54457	2.9656	2.52204
LAMTOR5-AS1	1.98578	2.10559	2.16538	1.73332	2.74055	2.65243	3.1729	2.42495	2.27727	2.33959	2.14923	0.96638	2.4094	2.43021	1.52091	1.52337
SLC16A4	-1.88073	-2.45388	-2.02816	-0.35732	0.31601	-0.24582	0.82367	0.27218	-1.17846	-0.07288	-0.01661	1.89693	1.59372	1.4742	1.41886	1.75547
LAMTOR5	5.12819	5.21295	5.15229	4.97858	5.51692	5.90306	5.75597	5.74315	5.15272	5.21994	4.91002	4.26091	5.5134	5.65204	4.23827	4.3025
LRIF1	2.78097	2.95425	2.72261	3.56928	2.00516	2.89237	2.7618	2.87582	3.63313	3.45571	3.18822	4.11357	3.51108	3.72989	2.88863	3.22421
DRAM2	1.79231	2.13844	2.09114	2.70751	4.47663	4.30417	5.04535	4.24464	3.36135	3.85851	3.22883	4.32744	3.68584	4.025	3.94502	3.79008
CEPT1	1.24453	1.43794	1.56197	1.93171	2.87664	2.14054	3.23796	2.25861	2.04304	2.53219	2.06046	2.18355	2.02937	2.19742	2.43358	2.12374
DENND2D	-3.13197	-3.32193	-3.32193	-3.08601	-2.70095	-1.88499	-2.98539	-2.20311	0.19773	0.44578	0.46772	0.68485	-0.94632	-0.7775	-0.8103	-0.45062
CHI3L2	-1.1414	-1.08888	-1.50984	-0.24574	-3.18438	-3.32193	-3.0352	-3.32193	-1.77768	-0.63235	-1.10155	0.40939	1.16387	2.02563	1.24571	2.35359
CHIA	-3.32193	-3.32193	-3.32193	-3.32193	-3.32193	-3.32193	-3.32193	-3.05974	-3.32193	-3.32193	-3.32193	-3.32193	-2.19666	-0.6229	-0.59966	-0.34602
PIFO	-3.32193	-3.32193	-3.32193	-2.381	-2.49526	-1.2126	-2.08303	-1.76131	-1.89232	-1.60676	-1.38445	-1.167	-0.40276	0.83012	-0.36174	0.79518
OVGP1	-1.82927	-2.08472	-2.03932	-2.22092	-0.73507	-0.21051	-1.38752	-0.56623	-3.32193	-2.95282	-1.79623	-2.42673	-2.19474	-1.91476	-2.66444	-2.40725
WDR77	4.33542	3.98599	3.64659	3.90497	4.49397	3.98898	4.39549	3.85616	4.93798	4.00978	4.56271	4.11547	5.03703	4.83197	4.32517	4.32776
ATP5PB	5.25227	5.12254	4.94674	5.25929	5.51134	4.83833	5.66948	4.92676	6.25889	6.01861	5.90381	5.8311	6.22039	6.15089	5.47214	5.59065
RAP1A	3.91358	3.97317	3.92998	4.14028	3.09678	2.20946	3.74474	2.18229	3.88685	3.45643	3.43254	3.93564	3.28545	3.02288	2.84516	2.40297
INKA2	-2.21341	-2.28319	-1.83759	-1.92025	0.40887	-0.64233	0.48736	-0.34443	-0.42125	-0.53049	-0.10321	-0.5371	-0.9307	-0.01258	0.22579	0.28839
INKA2-AS1	-3.32193	-2.82748	-3.32193	-2.77618	-2.06364	-0.98547	-1.71796	-0.90728	-0.36339	0.29142	-0.29185	0.1837	-0.70465	0.23926	0.00588	0.22187
DDX20	2.14814	1.84897	1.75239	1.7743	2.08224	1.29084	2.4059	1.07665	2.56447	2.18632	2.38665	2.457	2.53794	1.92762	2.32542	2.25731
CTTNBP2NL	1.63621	1.65949	1.84884	1.95286	1.76884	0.16022	2.58266	0.10098	3.50723	2.67521	3.30986	3.39564	3.14503	1.6897	3.60997	2.64337
WNT2B	-2.20159	-2.24494	-2.15558	-2.15327	-0.3494	-0.65004	-0.0512	-0.55516	-1.31601	-1.44175	-1.51609	-1.02195	0.20216	0.7336	0.58882	0.6352
ST7L	1.57077	1.36188	1.44291	1.47595	1.58964	1.07222	1.72142	1.26926	1.16775	1.00057	1.20201	1.238	1.39714	1.61259	1.19121	1.40557
CAPZA1	5.74421	5.4341	5.8554	5.81751	5.81228	4.91086	6.61959	4.72534	5.94614	5.54055	6.28125	6.15776	5.76582	5.14312	6.23757	5.19715
MOV10	3.54425	3.53393	3.75099	3.863	4.66389	4.56199	4.24023	4.57281	5.11546	4.90499	5.3961	5.33216	3.76932	3.69435	4.3431	4.27396
PPM1J	-1.54353	-0.77357	-0.78612	-1.29754	1.12162	1.2755	0.91818	0.94922	0.4389	0.79956	0.44243	-0.29192	0.9247	1.15652	1.16349	1.49281
LINC01356	-3.32193	-2.75843	-2.31562	-3.32193	-2.14198	-0.91471	-2.4781	-1.989	1.30762	1.75886	1.84165	0.34532	0.1383	1.01978	-0.4947	0.47294
SLC16A1	4.83601	4.57567	4.3422	4.64493	5.29138	4.04288	5.6451	4.24055	5.58981	5.36153	5.46813	5.3493	5.31539	5.08106	5.02691	5.0094
SLC16A1-AS1	0.69907	0.71201	0.3754	1.16703	0.10488	0.50167	0.17508	1.05375	-0.26938	-0.48858	-0.21534	-0.27623	0.27447	0.09511	-0.20513	-0.37263
LRIG2	-0.07795	0.02313	0.36567	0.49938	0.78866	0.643	1.07888	1.00868	0.54755	0.92792	0.85641	1.65441	1.04087	1.37047	1.34092	1.6141
RLIMP2	-1.7436	-1.02874	-1.57642	-0.7334	-0.21892	-0.5095	0.16314	0.06347	-0.05025	0.42756	0.52351	1.34926	0.29649	1.10732	1.43696	1.44185
MAGI3	-0.31812	-0.12029	0.40625	0.65375	2.42295	0.72603	2.85566	0.93026	1.96526	1.59389	1.79549	2.36092	1.76568	1.86703	2.56675	2.40948
PHTF1	1.70042	1.96001	1.99947	2.54579	3.9073	3.20254	4.42086	3.04435	2.19905	2.26531	2.47534	3.0934	1.71	1.98303	1.84525	2.46716
RSBN1	0.98404	1.30063	1.14862	1.30496	1.86904	0.74551	2.19642	0.93662	2.72154	2.7668	2.12706	2.82835	1.28463	0.98814	1.48842	1.34053
PTPN22	-0.43885	-0.59724	-0.86452	-0.96182	-3.03552	-3.32193	-2.60512	-3.32193	-0.31978	-0.05251	0.48894	0.91149	-2.97703	-3.32193	-3.32193	-3.09765
AP4B1-AS1	2.13648	2.45297	2.08274	2.35863	2.22102	1.87779	2.54197	1.54673	2.67698	2.29009	2.42918	2.29123	2.3742	2.11363	2.19756	1.86314
AP4B1	2.02178	2.24993	1.95785	1.98868	2.04309	1.83346	2.24542	1.90498	2.39922	1.94475	2.09189	2.01185	2.19682	1.94776	1.88299	1.70483
DCLRE1B	2.57777	2.51497	2.5916	2.07146	2.89959	1.59824	2.93335	1.89979	3.38849	2.38645	2.60868	1.93916	2.807	3.04976	3.07258	2.62632
HIPK1	1.13149	1.46777	1.46227	1.76302	3.5857	2.20119	4.06825	2.1942	3.56125	3.01365	3.64463	3.56874	3.4675	3.33284	3.94253	3.44379
OLFML3	-1.31245	-0.60859	0.79054	1.17207	-2.65815	-2.68377	-2.49783	-2.1147	3.46426	3.24665	3.7544	3.07382	1.34232	1.14062	0.57741	1.61037

TRIM33	2.86442	3.23947	3.31105	3.85786	2.86907	2.61068	3.3194	2.91157	3.11681	2.90442	2.9574	3.93627	2.98975	2.34313	2.89477	2.79554
PKMP1	-1.71861	-0.34671	-0.38815	-1.041	-0.90817	-0.82695	-1.37554	-1.31093	0.81438	1.53656	0.55834	-0.36524	-1.55834	-2.18873	-2.34148	-2.32583
BCAS2	5.50995	5.37656	5.23868	5.67063	5.45606	5.11746	5.71645	4.91797	5.4385	4.9317	5.15992	5.11245	5.11259	5.01476	4.6908	4.59575
DENND2C	-3.04764	-2.12492	-2.51934	-2.10675	1.79328	0.66174	1.90131	0.73903	-0.07116	-0.56604	-0.61453	0.0606	-0.79653	-0.78039	-0.04446	-0.22422
NRAS	3.79146	4.18369	4.43532	4.51823	5.67219	4.77444	6.85605	4.56125	4.94674	5.23445	5.45515	6.03393	5.06439	5.55252	6.1974	5.58358
SIKE1	2.54302	2.10349	2.12912	2.24425	3.45867	2.98705	3.91254	3.02963	3.58592	3.36584	3.31058	4.02376	3.77684	3.67055	2.93182	3.04162
TSPAN2	-2.78071	-1.98121	-1.6633	-2.03705	-3.32193	-3.32193	-3.32193	-2.97629	0.5087	1.67564	0.82704	1.14698	1.88824	2.18328	2.95402	1.66225
NGF	0.79146	1.37632	0.82261	0.9092	-3.32193	-3.32193	-3.32193	-3.32193	3.69358	4.51744	3.79935	2.6993	1.31094	1.90521	0.45831	-1.11499
VANGL1	2.52422	2.42177	2.28059	2.53283	3.32208	1.57704	2.83495	1.97087	3.40228	2.59409	1.52903	2.45497	3.23261	2.90455	2.77871	2.97534
SLC22A15	0.11453	0.16426	0.29793	0.64397	0.28826	1.36532	0.83499	0.85559	-0.45257	0.10544	-0.21818	0.82243	-2.56543	-1.76303	-3.32193	-2.66889
MAB21L3	-3.32193	-2.97989	-2.96308	-2.9424	0.51216	-0.84189	1.13193	-1.74439	-2.44914	-3.32193	-3.32193	-3.32193	-3.32193	-3.32193	-2.57111	-3.32193
ATP1A1-AS1	6.23542	5.89219	5.81472	6.20482	6.9565	6.58596	7.14571	6.49079	6.64865	6.43126	6.51763	7.25059	5.30851	5.61117	6.06858	6.31019
CD58	1.87554	1.64577	2.00694	2.58009	3.23816	2.63634	3.98779	2.3856	1.84444	1.95521	1.66281	3.14539	3.18091	2.72775	2.57528	2.03315
IGSF3	2.48163	2.70652	2.70025	2.86581	5.37701	3.71974	5.54688	3.84133	3.34448	3.54349	3.12546	3.86164	4.17748	4.90336	5.21296	5.72386
PTGFRN	0.71329	0.32422	0.51778	0.67464	5.37576	3.69328	5.31971	3.64848	0.01285	-0.41884	-0.13592	-0.44013	3.53441	3.92952	4.27152	4.21302
TTF2	1.01511	0.298	0.25435	0.33851	2.58471	1.2092	2.31244	1.41958	2.06519	0.50166	1.66539	0.76309	2.37362	1.4771	2.44421	1.51224
TRIM45	-0.43506	-0.78927	-0.67516	-0.21316	1.13611	0.71808	0.74471	1.01662	0.20578	-0.7865	-0.46229	-0.98775	0.42105	0.70112	0.62477	1.0177
MAN1A2	1.30359	1.37135	1.22356	2.07897	3.36772	2.4135	4.07976	2.43546	2.5342	2.21793	2.81143	3.22457	2.4831	1.31854	2.56323	2.19335
GDAP2	0.73138	0.85576	0.81923	1.06233	1.532	1.0729	2.06562	0.9998	0.93625	0.57573	0.5867	1.46663	1.24188	1.21559	0.99425	0.98269
WDR3	2.98236	2.7212	2.33591	2.42833	3.33711	2.21302	3.78286	2.0475	3.25772	3.23623	3.40933	3.08737	2.79755	2.61778	2.48236	2.47179
SPAG17	0.5904	0.26723	-0.13829	-0.2192	0.58263	-0.33994	0.88908	-0.32948	0.55527	0.47798	0.63018	-0.00939	-0.08889	-0.06449	-0.44199	-0.36964
TBX15	-0.86611	-1.10095	-1.3588	-1.04054	-0.15718	-0.32011	-0.06407	0.19275	-2.76214	-2.84344	-3.04543	-3.32193	1.16398	0.79878	1.36323	0.6892
WARS2	0.96796	1.12649	1.17065	1.44534	1.60356	1.20351	1.57593	1.11347	2.90435	2.7389	2.49529	3.19922	2.33093	2.67799	2.09755	1.92649
WARS2-AS1	0.33934	0.20492	0.17235	0.5134	0.87816	0.64654	0.76969	0.13376	1.86177	2.02631	1.80749	1.94795	0.69061	0.52826	0.35647	0.55855
LINC00622	-0.08867	1.46639	0.27829	2.40046	-2.81614	-3.32193	-2.77454	-3.32193	-2.21812	-1.37326	-2.30456	-0.81442	-3.32193	-3.32193	-3.32193	-2.75398
ZNF697	3.11185	3.20272	2.95622	3.23418	1.03921	0.41117	1.12688	0.17056	2.57336	2.72631	2.39715	3.13972	0.93302	1.38512	1.50289	1.73564
PHGDH	2.79441	2.27636	2.50756	1.61125	2.68561	3.6246	2.25365	3.45902	4.77702	3.66145	3.75102	2.60572	2.56039	2.66406	2.97419	2.61043
HMGCS2	-3.32193	-3.32193	-3.32193	-3.32193	0.1035	0.61779	-0.16976	1.28324	-3.32193	-3.32193	-3.32193	-3.32193	-3.32193	-3.32193	-3.32193	-3.32193
REG4	-3.32193	-3.32193	-3.32193	-3.32193	2.51946	-1.83793	2.70698	-2.10694	-3.32193	-3.32193	-3.32193	-3.32193	-3.32193	-3.32193	-3.32193	-3.32193
SEC22B	4.42975	4.89862	4.45964	5.34676	3.30375	3.41447	3.75491	3.23872	3.74322	4.07263	3.76281	4.25124	3.4678	3.06855	3.3053	3.33774
PFN1P2	-0.29764	-0.3099	-0.62812	0.00118	-0.9218	-0.81899	-0.46017	-1.15367	-0.54481	-0.13681	-0.68547	0.27999	-0.75325	-0.40479	-0.2839	0.53779
NBPF8	3.46028	3.52456	3.48001	3.90058	3.22427	3.56306	3.0334	3.55957	2.71863	2.98826	3.0646	4.44717	3.51016	3.29556	2.69627	3.45183
NBPF26	3.59596	3.81553	3.43512	4.0477	2.98347	2.45912	2.89031	2.41933	3.83573	3.97461	4.13322	5.46818	4.30498	4.1232	4.03684	4.35517
LINC00623	3.51988	3.41676	3.56595	3.18133	2.66877	3.37662	2.81719	3.41794	-0.68568	-0.72511	-1.0366	-0.92824	0.24392	0.5292	-0.17521	0.60741
FAM72B	3.43588	2.87535	2.75258	2.52436	2.769	2.37765	2.67402	2.49553	3.46153	2.75226	2.81666	1.79574	3.64954	3.67634	2.84501	2.6131
SRGAP2C	2.34147	2.57587	2.48116	3.04171	2.45028	2.11043	2.37115	2.1476	3.9553	3.7809	3.38673	4.21623	3.28378	3.14991	3.36405	3.58035
EMBP1	-2.03922	-1.93879	-2.27856	-1.83189	-0.84572	-0.59637	-0.90317	-0.03877	-3.04907	-2.06988	-2.37499	-2.30841	-2.05693	-1.34507	-1.79528	-2.05988
LINC02591	1.1291	0.84376	1.21863	1.53759	-0.05693	0.96783	-0.12777	0.88212	-3.09288	-3.32193	-3.32193	-2.96504	-2.375	-2.21385	-2.67546	-1.87119
FAM72C	4.08411	3.48206	3.42599	3.02131	3.8299	3.39031	3.67213	3.37688	3.85151	2.92484	3.02691	2.04822	4.69095	4.5511	3.92269	3.6283
SRGAP2D	3.24031	2.95956	2.83279	3.12707	3.88333	2.39217	3.69411	2.34277	4.15149	3.93296	3.30227	4.07198	3.90001	3.82413	4.01898	4.49677
NBPF15	3.89041	3.92329	3.95955	4.14698	3.4565	3.66848	3.23028	3.86542	3.49664	3.71667	3.36851	4.25843	3.68418	3.79754	3.23495	4.12104
PFN1P6	1.99651	1.75484	2.16302	2.52302	1.36645	1.51267	1.49751	1.92667	1.60373	2.014	1.97402	2.29114	2.1761	2.31008	2.24576	3.47374
SRGAP2B	2.68078	2.78313	2.74426	3.21746	3.13879	2.52746	3.01545	2.5442	3.97471	3.83398	3.26757	4.07297	3.82731	3.62269	4.01071	4.34394
FAM72D	3.9969	3.39147	3.34889	2.94538	3.36507	3.08975	3.21067	3.21191	3.81768	2.99424	3.02088	2.15662	4.51276	4.39255	3.69256	3.36982
LINC01145	2.16446	2.13309	2.45681	2.48561	1.20438	1.68831	1.12533	1.75586	-2.94572	-2.33014	-2.02304	-1.95489	-1.59543	-0.41448	-1.13657	-0.64888
NBPF20	1.04637	0.9519	0.92656	1.27875	0.51526	1.05863	0.50939	1.30828	0.98603	1.60933	0.98502	1.62593	0.87826	0.97287	0.6692	1.26344
NBPF25P	2.86779	2.64291	2.69844	3.33775	1.89767	2.60095	1.63688	2.817	1.76561	2.07941	2.00276	2.89237	2.34734	2.54374	2.0587	2.45498
GPR89A	2.27342	1.76213	2.05446	2.01142	3.01083	2.81424	3.4729	2.72868	2.34131	1.77108	2.30653	1.98291	3.41989	2.71007	3.43931	2.57877
PDZK1	-0.81241	-1.14797	-1.20528	-0.70465	5.34806	2.7056	5.0568	3.71048	-0.09441	-1.31553	-0.48202	0.57955	0.07886	-0.70596	0.03739	-0.55585
CD160	-2.65622	-2.79712	-1.31827	-2.16252	-2.45003	-1.7485	-1.49243	-0.94775	-3.02658	-2.36413	-2.17474	-2.24352	-0.91939	-1.30348	-0.24057	-0.49343

RNF115	1.86785	1.60546	2.04348	1.8607	2.61321	1.41068	2.8714	1.58957	2.346	1.50168	2.02715	1.83129	3.06835	2.33462	3.21222	2.04018
POLR3C	3.81531	3.8682	3.72275	3.78828	4.17207	3.16191	4.37303	3.10337	4.30998	3.92976	4.1115	4.54276	4.20401	3.76935	4.32561	3.88993
NUDT17	3.20689	2.79377	3.253	2.98278	2.9484	3.88103	2.9215	3.75769	3.38464	3.06445	3.49935	3.1307	3.49253	2.86852	2.90279	2.71037
PIAS3	3.15692	2.75564	3.49512	2.90725	3.27142	3.2662	3.019	3.04029	3.98318	2.98532	3.93956	3.29846	3.79366	2.55458	3.65613	2.80817
ITGA10	-2.74544	-2.97067	-2.7994	-1.88614	-1.10443	1.43693	-0.54664	0.14775	-2.32251	-0.0522	-1.25828	1.03971	1.87929	1.9727	2.30518	3.54998
PEX11B	3.28209	3.30208	3.20239	3.8032	4.02151	4.21618	3.83292	3.71239	4.61435	5.01566	4.97364	4.74848	4.70791	4.77577	4.51845	4.56502
RBM8A	4.91515	4.76681	4.60101	4.58563	4.53569	4.71258	4.73341	4.64245	5.6664	5.29237	5.52338	5.10232	5.716	5.80457	5.14752	5.23616
GNRHR2	2.50957	2.601	2.38587	2.48587	3.00282	2.46928	3.27911	2.29748	3.90437	3.82457	3.74977	3.72222	4.32923	4.38702	3.76407	4.25113
LIX1L-AS1	4.93839	4.7635	4.74393	4.54209	4.5441	4.4587	4.72233	4.28417	5.65536	5.19826	5.52493	4.85258	5.61683	5.72491	5.24944	5.10343
LIX1L	3.80755	3.64683	3.74673	3.45549	3.2882	3.57113	3.25961	3.3376	4.58211	4.53031	4.37795	5.22127	4.98722	5.15991	4.84746	4.82271
ANKRD34A	-0.77233	-0.43507	-0.41471	-0.48831	1.39395	2.50336	0.65782	1.94137	1.43331	0.60859	1.23401	-0.47333	0.03707	0.05548	0.38862	0.48303
POLR3GL	2.83433	2.73054	2.60407	2.86572	2.45597	3.16966	1.89698	2.81297	4.1252	4.22143	4.19648	4.05864	3.56414	4.04155	3.80539	4.02384
TXNIP	-0.38889	0.29802	-0.0938	1.17777	0.83367	0.76715	1.3324	0.56085	6.35625	7.55156	7.02514	7.7097	0.00829	1.10821	0.59499	1.45026
LINC01719	-0.45106	-0.07314	-0.77028	-0.22204	-1.866	-1.62828	-1.85286	-1.19098	-2.52779	-0.92612	-1.60397	-0.2373	-1.0122	0.01722	-0.44796	-0.25237
NBPF10	2.61124	2.7697	2.91053	3.34235	1.55762	1.29706	1.57082	1.59092	2.55603	2.67772	2.66825	3.72613	2.28748	2.00684	1.79365	2.17415
NOTCH2NLA	2.59998	3.12085	2.89717	2.99036	3.52464	3.43534	3.35646	3.19682	3.44378	3.3886	3.483	3.48392	2.82253	2.8136	3.01053	2.96558
NUDT4P2	3.8678	3.49419	3.30268	3.70267	4.03562	2.65076	4.18152	2.11329	4.65617	4.15236	4.30977	4.36336	3.57341	3.21879	3.39918	3.74596
SEC22B4P	1.47616	0.29735	1.31216	1.4002	-0.17932	-3.32193	-0.46218	0.76131	-1.03006	0.90926	-0.04653	2.23524	-1.46335	-1.46708	-0.43406	-1.92367
HYDIN2	-2.71318	-2.65363	-2.86445	-2.83897	-3.16946	-3.17638	-3.18705	-3.32193	-2.87187	-3.25069	-3.1039	-2.89074	-1.71016	-1.63808	-1.73469	-1.4689
NBPF12	2.14136	1.96693	1.9085	2.31537	2.16452	2.29336	2.1614	2.75527	1.5004	1.81005	1.54565	3.18822	2.5997	2.58666	2.40789	2.92579
PRKAB2	1.12125	1.09909	1.44737	1.16054	3.37	2.85943	3.44106	2.53126	2.07523	2.70072	2.57905	3.07516	2.89506	2.77774	2.59233	2.60046
FMO5	-0.66294	-0.71467	-0.10551	-0.97371	4.01164	2.87762	3.75722	2.99924	1.28449	0.96377	1.33977	0.80491	1.24811	0.88831	1.09931	1.44983
PDIA3P1	2.70595	2.92595	3.1878	3.07131	3.75523	4.32594	4.37701	3.9717	3.80944	3.86518	3.46145	3.69096	2.77075	2.83221	2.71757	2.55292
CCT8P1	-0.93912	-0.17042	-1.63503	-1.33771	2.84034	3.57192	3.07909	3.65934	-0.40866	0.70988	-1.03452	-0.3845	-1.48655	-1.82204	-1.44098	-2.07628
CHD1L	4.34232	4.03176	4.14966	4.05175	5.68036	5.35132	5.64736	5.19177	4.96491	4.55112	4.94254	4.84046	5.13687	4.69626	4.87137	4.75603
LINC00624	-0.60393	-0.77263	-0.66928	-0.55405	1.51037	-0.05843	1.70664	-1.10469	0.79708	0.28265	0.69356	0.66087	1.12189	0.65079	0.58339	0.79367
BCL9	2.21208	2.23677	2.28372	2.2213	2.56741	2.2426	2.59081	2.23745	2.68332	2.39942	2.72514	2.86279	3.33917	3.70261	4.60105	4.54332
ACP6	1.59154	1.25523	1.39892	1.57706	0.07531	0.50466	-0.51523	0.64755	0.57327	0.78301	0.56376	0.90437	0.7494	0.83747	0.98352	1.30359
GJA5	-3.32193	-2.70912	-3.32193	-3.32193	-3.32193	-3.32193	-3.32193	-3.32193	0.32303	-1.03954	-0.14247	-0.75707	-3.32193	-3.07821	-3.32193	-3.32193
GPR89B	2.18165	1.82999	1.94653	1.90986	3.12861	2.66459	3.59208	2.64123	2.14244	1.37206	1.986	1.96018	3.29023	2.56549	3.24702	2.46451
PDZK1P1	-2.28192	-1.67395	-2.32115	-1.44741	3.03904	0.53166	2.45502	1.34324	-1.75421	-2.4621	-2.41477	-1.47257	-1.66591	-2.00917	-1.38698	-1.91638
NBPF11	1.80454	1.86586	1.8764	1.98374	1.84696	2.31296	1.69731	2.36312	1.00817	1.60038	1.26203	2.4579	2.43466	2.4159	2.03112	2.47271
PFN1P4	0.0913	0.87466	1.11007	-0.26677	0.49767	-0.54454	0.78403	0.02649	-0.00341	0.97212	-0.15199	-0.26517	0.0108	0.54243	0.2702	2.20843
ABHD17AP1	2.46129	2.86579	2.35787	0.32786	0.95391	2.01899	-0.06773	2.39093	1.9162	2.14784	1.5148	0.06532	-0.18109	-0.18565	-0.16131	0.30625
LINC01138	1.41581	1.4098	1.47219	0.81573	0.92838	1.51277	0.87961	1.27967	-1.71492	-0.78761	-1.44151	-1.30644	-2.1734	-1.89027	-1.37067	-1.50377
NBPF14	3.62394	3.80269	3.70059	4.08755	3.19011	2.59743	3.12372	2.58244	4.13791	4.37159	4.41111	5.91824	4.18996	3.72699	3.98404	4.03424
NUDT4B	3.22761	3.21312	2.86132	3.23922	3.3523	2.23872	3.4077	1.85641	3.90553	3.53364	3.60488	3.8271	3.41105	3.01575	3.16153	3.36091
PDE4DIP	2.98385	3.58137	2.97805	3.52444	1.02624	0.30936	1.04021	0.14523	1.44493	2.05517	1.67011	2.57721	-0.33393	-0.85771	-0.36089	-0.43663
NBPF9	2.97381	3.00458	3.06416	3.29823	2.90912	3.06022	2.76112	3.25545	3.27408	3.46757	2.95302	3.80234	2.77436	2.83441	2.32386	3.0142
NBPF19	2.8318	3.3564	3.07988	3.62664	2.22482	2.3925	2.03944	2.41511	2.14161	2.54229	2.43033	3.88404	2.41169	2.2935	2.19446	2.76574
LINC00869	1.73636	1.58987	1.71368	1.2311	1.41095	2.05071	1.32379	1.86396	-2.52049	-1.68366	-2.58897	-2.71852	-2.08377	-2.00192	-1.18459	-1.37669
BOLA1	3.69551	3.49184	3.44955	2.6172	3.6985	4.51221	2.48649	4.12728	3.29195	3.07015	3.39999	1.77558	3.48696	3.43837	2.75364	2.42968
SV2A	1.30162	1.81269	1.81224	2.33212	-2.89792	-1.68284	-2.64321	-2.35343	-3.32193	-3.11386	-2.73696	-2.93783	-2.99759	-3.15124	-3.11578	-2.36437
SF3B4	6.99234	6.77152	6.65085	6.55889	5.67959	5.67716	5.29794	5.86094	6.36099	5.81179	6.02303	5.62042	6.37086	6.33422	6.00374	6.13627
MTMR11	1.25079	1.95637	2.15496	2.15002	2.59782	2.60956	2.43827	2.13417	0.91053	1.55489	1.20352	2.28029	2.27248	2.92417	2.61114	3.11824
OTUD7B	1.73062	1.90078	2.16337	1.6951	3.28783	2.03401	3.37417	2.25852	2.24644	1.70719	2.39235	2.16174	2.47319	1.85109	3.19262	2.90839
VPS45	2.40413	2.15066	2.28891	2.48333	2.41617	2.38898	2.6423	2.20967	2.47135	1.98128	2.26281	2.21997	3.18604	3.05056	2.81684	2.60287
PLEKHO1	5.11011	5.16646	5.44633	5.22018	1.62875	2.74631	1.49297	1.97773	1.34823	0.7814	0.70564	0.206	3.45593	3.74501	4.12875	3.94789
ANP32E	5.89687	5.79464	5.51266	5.78279	5.19884	5.06101	5.65824	5.18932	6.04361	5.62722	5.43927	5.31752	6.21913	6.77314	5.92401	6.31355
CA14	-1.93243	-1.10895	-1.45848	-1.20989	-1.55305	-0.61538	-1.53237	-0.41454	-1.57622	-1.49887	-2.81652	-1.51647	-2.13984	-0.57984	-1.50915	-0.26689

APH1A	5.43173	5.53925	5.576	5.5611	6.17171	5.95599	5.86488	5.69483	5.88104	6.17874	6.06169	5.76715	5.74235	6.2602	5.90615	6.07653
C1orf54	3.70965	3.61902	4.06821	3.8173	4.84762	3.62711	4.55337	3.15615	4.72662	4.95473	4.84724	4.97836	4.63014	5.07231	5.13763	5.26055
CIART	-1.7297	-2.12503	-1.29707	-0.80479	-2.87114	-2.95337	-2.7014	-2.89711	1.02912	0.95022	0.3706	-0.35426	2.53192	2.03568	2.16917	1.71128
MRPS21	6.58093	6.64087	6.49595	6.35258	5.33391	6.11566	5.0068	5.92862	5.47636	5.70299	4.95722	4.85447	6.06326	6.11763	4.89784	5.29759
PRPF3	3.85382	3.59471	3.4301	3.74542	3.66755	3.318	3.77661	3.45395	3.12322	2.60633	2.86743	2.5386	4.18458	3.98667	3.96869	3.77194
RPRD2	2.79478	2.35462	2.19621	2.05625	2.53322	1.78272	2.40782	2.09524	2.95039	2.25336	2.18404	2.76606	3.01461	2.59342	3.0517	2.96931
TARS2	3.75304	3.69868	3.41953	3.55525	3.60822	3.89363	3.3695	3.90458	3.64029	3.52121	3.81981	3.37414	3.15124	3.95241	3.29627	3.69961
ECM1	2.68979	3.40248	3.45832	3.87578	-0.47786	-0.05272	-0.59816	-0.34584	3.06515	2.97295	2.9195	2.53985	0.67986	-0.41668	-0.1978	1.31579
ADAMTSL4	1.62878	2.08093	2.11486	1.74839	2.12727	4.46657	1.05822	4.11696	2.08324	2.79289	2.26681	2.38218	0.55653	0.53307	0.36358	1.36994
ADAMTSL4-AS1	0.36066	1.08857	1.16296	1.62733	0.83557	4.06523	0.33396	3.86144	0.72096	1.14549	0.73454	1.40792	-0.17064	0.3281	-0.0773	0.65368
MCL1	6.0049	6.40804	6.38203	6.36198	5.83436	6.27735	6.56134	5.46105	5.77538	6.72279	6.51861	7.12821	6.36595	6.77887	7.05299	7.13786
ENSA	5.17737	5.07827	4.94877	4.91347	4.96467	4.822	5.00433	4.59287	5.09293	4.96373	5.01091	4.60946	5.62716	5.3277	4.96757	4.87504
GOLPH3L	2.36713	2.61935	2.30264	3.00308	2.98951	2.83755	3.18047	2.32429	3.47278	3.59218	3.41663	4.19817	3.69174	3.87737	3.68918	3.96077
HORMAD1	-3.32193	-3.32193	-3.32193	-2.8289	1.02326	1.47277	1.50651	0.70959	-3.32193	-3.32193	-3.32193	-3.32193	-3.32193	-3.32193	-3.32193	-3.32193
CTSS	4.92546	5.44402	5.24485	5.8096	2.67792	3.11372	2.81198	2.81173	-0.10137	0.53137	0.39731	1.16379	2.37998	2.20151	1.01996	1.80144
CTSK	-0.18202	-1.24076	-0.10723	-0.02521	-0.45631	2.21188	0.27903	2.29107	-1.77977	-0.89895	-1.49148	-0.46623	1.90347	2.23001	1.36397	2.09568
ARNT	2.81642	2.78481	3.06994	3.22225	3.48981	2.8706	3.66262	2.81305	3.42023	2.91289	3.41109	3.38915	3.45072	3.03048	3.80818	3.25549
SETDB1	2.82173	2.72178	2.84618	2.97001	2.51209	2.6313	2.59804	2.84659	2.96015	3.1926	3.24056	3.71007	2.572	2.53327	2.87162	3.0808
CERS2	6.14758	5.36781	5.93689	5.51054	7.18599	5.45664	7.04232	5.18574	6.04464	4.53165	5.95042	4.77265	6.45867	3.39203	6.24344	3.7327
ANXA9	1.15393	1.00595	1.00971	1.13266	4.49661	5.09865	3.76442	5.116	0.41697	0.98941	0.67542	0.90528	1.55109	1.88106	2.57134	2.50031
MINDY1	2.06815	2.70252	2.00142	2.68489	3.33453	2.93217	3.16426	3.02819	2.13949	2.9775	2.54744	3.24951	2.63441	2.63306	3.25375	3.73794
PRUNE1	2.48504	1.65219	2.49916	1.96588	4.36912	2.4338	3.89326	2.6224	3.41595	2.26042	3.04675	2.51624	3.23421	2.27127	3.52287	2.23999
C1orf56	4.01035	4.01187	3.98181	3.82587	3.69059	4.10446	3.65822	4.07143	3.44595	3.25875	3.58494	3.63343	4.30318	3.92117	4.31503	3.91756
CDC42SE1	5.29902	5.18546	5.12468	5.36201	4.40208	4.2035	4.84147	4.03021	4.8736	4.92861	5.09468	5.0628	5.69181	6.02627	5.71633	5.66171
MLLT11	4.13151	4.52191	4.45711	5.11281	2.58419	3.21198	2.81059	2.92136	3.13392	3.47978	3.26157	3.05262	5.07158	6.04037	4.79861	5.19756
GABPB2	-0.13474	-1.27634	-0.7362	-0.63535	-0.39587	-1.48927	-1.11196	-0.9947	0.34288	-1.34664	-0.29075	-0.97609	1.31924	-1.48543	-0.10378	-0.79072
SEMA6C	-0.23753	-0.5149	0.2536	-0.00879	2.38983	2.96844	1.86961	3.12055	-0.24224	0.53882	0.9771	0.39607	0.53631	0.25717	2.20254	2.72379
SCNM1	4.69838	4.51635	4.5906	4.42398	3.57433	4.37304	3.00784	4.26612	3.67919	3.12033	3.42466	2.31905	3.80659	3.54816	3.56757	3.28197
LYSMD1	1.51308	1.54272	1.78084	1.65405	2.17084	2.18363	2.22157	2.06544	0.92486	1.78333	1.44824	1.82064	2.43547	2.6683	2.82828	2.72748
VPS72	4.26495	3.93718	4.07304	3.87294	3.81807	3.84584	3.29151	3.77911	4.15422	4.02417	4.09742	3.2561	4.37637	4.39063	4.29512	4.2401
PIP5K1A	4.26719	4.03795	3.85549	4.06217	3.92134	3.30529	4.23867	3.54803	4.67544	4.17244	4.54536	4.35216	5.20367	4.89694	4.72966	4.56922
PSMD4	6.41364	6.32536	6.30908	6.38838	6.25739	6.2859	6.17702	6.22351	6.45924	6.35385	6.53728	5.85794	6.29314	6.33597	6.13209	6.08496
ZNF687-AS1	1.50849	1.7317	1.80737	1.83408	1.94956	2.21467	1.93627	2.40448	-0.90209	0.92968	0.60586	0.81233	1.96807	1.85065	0.822	2.08409
ZNF687	3.14411	3.03236	3.16635	2.62405	3.63096	3.32016	3.08329	3.40369	3.77889	3.76147	3.76949	3.81515	3.13524	3.5548	3.99649	4.24581
PI4KB	3.782	3.73034	3.4036	3.54631	4.03667	3.48586	3.78137	3.51124	4.35549	4.26776	4.49085	4.45944	4.08133	4.35358	4.35271	4.6687
RFX5	3.80503	3.4322	3.4204	3.52732	4.16334	3.32545	3.48206	3.20454	4.21959	3.49137	3.76734	3.86029	4.03048	3.35516	3.44325	3.41991
SELENBP1	2.62504	2.83	2.59227	2.99733	1.11112	0.59538	0.74099	0.50309	-3.32193	-3.32193	-3.32193	-3.32193	-1.27807	-1.16548	-2.01141	-1.03189
PSMB4	8.1485	8.20172	8.1757	8.07057	7.28913	7.86467	7.0648	7.73761	7.58983	7.46058	7.72119	7.24168	7.07417	7.00203	6.67746	6.46489
POGZ	2.71539	2.4171	2.59757	2.59966	2.54616	2.4108	2.74901	2.72388	2.82595	2.01496	2.94637	3.03455	3.17698	2.36757	3.24677	2.73761
CGN	2.15452	1.78975	1.22501	1.10008	5.20354	4.19801	4.84417	4.5511	0.48066	0.89894	0.25547	0.3673	1.49116	2.13283	2.48812	2.46331
TUFT1	3.28784	3.00127	2.90187	2.45455	2.98237	2.0092	3.0468	2.21617	4.32286	3.82072	4.07129	2.93567	4.40092	4.39473	4.41473	3.88434
SNX27	3.10821	2.76973	2.75216	2.68947	4.12138	3.42851	3.99052	3.24915	2.7794	2.87707	3.04457	3.36296	3.65529	3.86545	4.31381	4.04008
MRPL9	6.17623	5.86232	5.69935	5.64409	5.8223	5.43984	5.71028	5.12708	6.01731	5.72367	6.08439	5.27028	5.79007	5.20869	5.62936	5.22113
OAZ3	3.32441	2.95818	2.76726	2.58502	3.27234	2.48227	3.17202	2.04434	3.42641	3.09294	3.42232	2.38668	3.16751	2.66697	3.17643	2.88578
TDRKH	1.86844	1.76629	1.74431	1.92386	2.02667	1.96016	2.12705	1.84027	0.87675	0.60946	0.44697	0.781	2.98574	2.96975	2.93046	3.12055
TDRKH-AS1	-0.23747	0.31639	0.29655	0.17688	-0.44309	-0.22345	-1.49828	-0.987	-2.48145	-1.22444	-2.05331	-1.48223	0.97463	1.51661	0.54973	0.64187
THEM4	1.83396	1.21565	1.33892	1.669	2.35915	2.32643	2.51788	2.32784	2.55949	2.32967	2.40636	2.70466	2.71244	2.47286	2.17708	2.5892
S100A10	7.63615	8.18553	8.54196	7.84526	8.17474	8.30666	8.33533	8.18447	8.51907	8.16295	8.06655	7.65407	7.95169	8.25581	7.02294	7.35103
FLG-AS1	-2.63892	-2.67454	-2.75807	-2.21992	-3.20328	-3.32193	-3.32193	-3.32193	0.55814	0.06199	-0.12752	0.10539	-3.32193	-3.11085	-3.0677	-3.06256
HRNR	-2.67616	-3.32193	-3.32193	-3.18204	-3.32193	-3.10396	-2.94215	-3.32193	-0.36777	0.60715	0.98543	1.11904	-3.32193	-2.61741	-2.16356	-2.84318

FLG	-2.63588	-2.57056	-2.87761	-2.06882	-3.17993	-3.23574	-3.24218	-3.03829	3.61406	2.38884	2.66117	1.59485	-3.32193	-2.62781	-2.95382	-2.35796
S100A9	-3.32193	-1.31492	-1.94699	0.70332	4.79735	6.21637	4.6703	5.05386	-3.32193	-3.32193	-3.32193	-3.32193	-3.32193	-3.32193	-3.32193	-3.32193
S100A4	1.15065	1.9409	1.05209	1.70239	1.5976	1.06384	1.83321	0.78333	1.62874	2.1638	1.82837	0.61883	4.24066	4.68669	3.72083	3.73624
S100A3	2.90603	3.73622	3.61253	3.66892	0.59944	-0.25236	0.3937	-3.32193	6.04518	5.63537	5.85225	3.83551	0.75848	1.81797	1.02842	0.75374
S100A2	2.75209	3.1868	2.87134	2.22168	-0.45194	-0.4131	-0.33372	-0.30949	2.86717	3.12051	2.94368	1.73975	1.97872	2.99774	1.84772	2.18482
S100A16	7.54647	7.61386	7.59466	7.39581	7.99216	8.01524	7.90799	7.34407	8.15001	8.48976	8.08107	8.11495	5.90282	5.93428	5.33457	5.94464
S100A14	-1.82454	-2.57161	-1.66195	-2.50042	3.9013	5.0726	3.69169	4.43576	-2.55093	-3.32193	-2.6174	-1.87383	-2.7601	-3.32193	-3.32193	-1.33443
S100A13	4.68382	4.65647	4.81077	4.88922	4.20014	4.77622	3.73773	4.58814	4.75755	5.09638	4.81672	4.85397	4.50346	4.57977	3.71601	4.05084
S100A1	-0.83377	-1.59947	-1.82584	-0.42137	-0.02743	0.1506	-0.24948	0.02004	-1.44238	-1.16553	-0.85963	-0.77189	-0.43022	0.0563	-0.22075	0.53586
CHTOP	4.6005	4.3518	4.32147	4.21574	4.23708	4.22681	4.29652	4.23961	4.54961	4.25748	4.57714	4.00167	4.15779	3.9969	3.61887	3.58043
SNAPIN	4.58357	4.61978	4.54014	4.57104	5.00073	5.2959	4.62292	4.90372	5.0695	5.20896	5.14166	4.48552	5.12077	5.50034	4.99851	5.05785
ILF2	6.6677	6.41258	6.28429	6.15618	6.8263	6.35655	6.83683	6.20785	7.40642	6.91425	7.17364	6.75699	7.40924	7.35367	7.1014	6.89044
NPR1	-1.08218	0.12761	-0.19775	-0.14731	0.22322	-1.21966	0.17392	-0.45928	-2.14419	-1.91838	-2.75427	-2.52673	-2.87345	-1.95774	-2.29564	-1.61228
INTS3	2.76693	2.62691	2.86043	2.49118	3.24933	2.86519	3.14099	3.02949	3.14768	2.91752	3.31108	3.49759	2.63311	3.00252	3.27393	3.41118
SLC27A3	0.71946	1.15964	1.11609	0.91929	1.24231	2.51329	0.49691	3.02977	-0.49367	0.04318	0.19358	-0.11951	-1.73656	-1.8509	-1.8567	-0.74066
GATAD2B	2.10038	2.11117	2.28185	2.27072	1.4754	0.81827	1.96917	1.24253	2.88702	2.52339	3.056	3.66262	2.80144	2.89243	3.80935	3.51032
DENND4B	3.82594	3.53435	3.60304	3.18863	4.18811	4.06772	3.28794	4.27381	4.04989	3.88114	4.2048	3.67251	3.69954	3.97362	3.97123	4.20676
CRTC2	4.88763	4.72576	4.79419	4.58808	4.84298	5.02299	4.08986	5.08264	4.45641	4.44586	4.53777	4.03705	3.79739	3.73528	4.10513	3.89355
SLC39A1	6.64289	6.39273	6.34569	6.18706	7.49482	6.9321	7.17344	6.7221	6.4633	6.24333	6.56373	5.86167	6.31737	5.88235	6.28874	6.10474
CREB3L4	2.11193	1.63214	1.74901	2.01701	2.30316	2.62127	1.80439	2.58799	1.51843	1.89066	1.73955	1.34043	1.59993	1.55454	1.64914	1.79262
JTB	6.53781	6.31968	6.15157	6.23054	6.99881	7.45883	6.6495	7.31339	6.12037	6.09249	5.84815	5.92694	6.66774	6.73952	5.78736	6.2659
RAB13	7.21566	7.41196	7.22372	7.57553	6.04073	6.51739	5.84242	6.53838	6.20911	6.64185	6.45146	6.62238	6.40329	6.84656	6.40713	6.7419
C1orf43	6.13013	6.19351	5.98801	6.4315	7.11616	7.0416	7.02206	6.88138	6.4407	6.66652	6.29545	6.4649	6.22363	6.37997	5.93909	6.14228
UBAP2L	5.20289	4.98814	4.99722	5.06117	4.71319	4.0498	4.62884	4.17004	5.37953	4.62515	5.35688	4.84469	5.35756	4.74742	5.43623	5.1145
HAX1	5.80072	5.49477	5.42706	5.26576	6.22446	6.74455	5.82901	6.49961	5.98536	5.60646	5.94102	5.08469	5.94043	5.66489	5.67797	5.33546
ATP8B2	3.0531	3.35063	3.24776	3.19497	3.84408	3.03818	3.34285	2.86649	3.77169	3.22212	3.69111	3.55703	3.43457	2.94211	3.44158	3.38441
RPSAP17	0.20758	0.36049	0.63956	-2.24496	-1.32685	-0.65923	-1.53058	-0.68334	0.87722	2.27692	0.45918	0.82089	-0.00697	-0.2264	-1.19868	-0.70217
IL6R	0.87337	0.71276	0.5815	0.76064	4.8717	4.22069	4.90187	4.28738	2.45478	1.60135	1.93561	1.67351	3.76779	3.60686	3.88063	4.40541
UBE2Q1	4.22612	3.92944	4.16809	4.24674	4.68608	4.43541	4.85113	4.2698	4.56215	3.88527	4.46904	4.05903	4.82658	4.07547	4.30193	3.81549
UBE2Q1-AS1	4.64803	4.35519	4.37476	4.57978	5.95729	4.6219	5.99607	4.4526	5.59511	4.60609	5.25751	4.78244	5.89472	5.10099	5.05333	5.04703
CHRNB2	-3.32193	-2.9482	-3.11272	-3.1	-1.87078	-2.37162	-2.01422	-1.96199	-1.05057	0.14205	-0.08003	-0.0753	-2.93281	-2.09741	-2.6181	-1.48564
ADAR	4.81365	4.58211	4.16224	4.42225	5.50316	4.11697	5.07637	4.37334	6.10403	5.8563	5.04639	6.33972	5.7651	5.76189	5.39569	6.16857
KCNN3	0.27176	0.13668	-0.19949	0.51175	-3.03722	-2.71845	-3.32193	-2.6961	-2.90864	-3.17127	-3.16068	-3.17369	-3.32193	-3.32193	-3.32193	-3.32193
PMVK	4.68641	4.57602	4.42672	3.75439	6.19192	6.14038	5.62306	6.02892	5.14321	5.07052	4.86393	4.21792	5.88272	5.88685	5.82129	6.0512
PBXIP1	3.89068	4.26921	4.46534	4.92728	4.49514	4.94619	4.08851	4.85007	4.38396	5.3816	5.11665	5.34287	4.38284	4.66934	5.68312	6.2527
PYGO2	4.83257	4.86718	5.04124	4.92908	5.42217	5.49599	5.4259	5.09387	5.35516	5.53498	5.9955	5.69899	5.30059	4.98468	5.87294	5.36177
CKS1B	5.25709	4.6981	5.12212	4.37573	5.80336	5.20499	5.62098	5.10811	6.43708	5.10901	5.4756	3.89329	7.24228	6.7344	6.36028	5.58044
FLAD1	3.9243	3.82006	3.61184	3.77388	4.11874	4.08655	3.52938	4.12256	3.48396	3.47562	3.57084	2.9883	3.66359	3.85915	3.66174	3.83698
ZBTB7B	3.23373	3.92238	3.72235	3.59417	5.36582	5.64476	5.01306	5.90991	4.60127	5.33811	5.14169	4.83294	3.87187	4.70475	4.66169	5.1207
DCST1	-0.71206	-0.459	-0.44706	-1.19253	-0.28169	-0.25621	-1.25964	-0.56018	-1.38634	-1.30504	-1.99377	-1.93622	-1.0003	-1.30901	-2.06345	-1.37841
DCST1-AS1	1.50495	1.21754	1.41834	-0.29484	0.38184	1.00668	0.21115	0.64148	-0.20384	0.01503	-1.3458	-0.65052	0.51749	0.26975	0.00211	-0.2565
ADAM15	5.35895	5.44373	5.41221	5.22524	4.09269	4.07633	3.2919	3.90323	4.85306	4.92999	4.85152	4.44119	3.9919	3.91994	4.53501	4.71284
EFNA4	2.92543	2.88968	3.05773	3.01618	3.46063	4.34288	2.9068	4.06734	2.91236	2.80194	3.08797	2.49421	3.28933	3.28423	3.18214	3.55258
EFNA3	1.81357	2.40656	2.23999	1.737	2.42394	4.0481	1.27199	3.65814	2.15303	3.26703	2.58409	1.86625	0.78609	2.05741	1.55267	2.14553
EFNA1	3.20787	3.08846	3.18863	3.55032	3.07411	4.21103	2.5865	4.00876	3.9091	4.20664	3.71314	3.64751	3.63333	3.82324	2.99108	3.43814
SLC50A1	3.95633	3.89523	4.34601	3.90344	5.29344	5.47682	4.87716	4.99711	4.02268	3.54176	4.46308	3.25765	4.01189	2.46918	3.57431	2.36436
DPM3	6.52844	6.38734	6.64644	6.06708	6.24366	7.36514	5.69527	6.88553	5.57068	5.65176	5.6769	4.45057	6.14321	6.19672	5.30932	5.35374
KRTCAP2	4.57495	4.57989	4.57411	4.1984	5.00979	6.43956	4.28744	6.2114	4.5009	4.19929	4.44817	3.75322	4.22817	4.09521	3.91742	3.80701
TRIM46	-0.28787	-0.02327	0.64198	-0.22347	-2.87453	-0.30444	-2.60887	0.02348	1.1307	1.87429	1.7347	1.35101	-2.27015	-2.09753	-2.74622	-0.85955
MUC1	-1.59792	-0.19158	0.22912	0.16065	-0.41527	0.96094	-0.72063	0.10904	1.22505	2.85911	1.17933	2.82853	-0.55626	0.20569	0.42334	1.40762

THBS3	0.54582	1.02917	1.33715	1.90507	0.982	2.30877	0.42008	2.1931	1.12961	2.5754	2.33816	2.86664	0.96679	1.93432	1.99818	3.34471
MTX1	4.81663	4.80205	4.76535	4.65747	5.31613	5.89753	4.93646	5.76016	4.62986	4.49023	4.75393	4.0253	4.53334	4.66483	4.52351	4.64162
GBAP1	4.0434	4.46704	4.68244	5.09091	4.17542	5.18908	4.29123	4.98813	2.08592	2.90101	3.13532	3.27191	2.40297	3.00683	3.13002	3.68728
MTX1P1	6.32576	6.30946	6.21691	6.21839	6.71971	7.34074	6.34172	7.2354	5.78144	5.85507	6.04259	5.27871	5.67343	5.96732	5.73932	6.11732
GBA	5.55822	6.06425	6.19963	6.65675	5.99368	6.24037	6.20317	5.94879	4.06121	4.78827	5.09945	5.22466	3.94181	4.49746	4.90885	5.40994
FAM189B	4.57566	4.5651	4.63199	4.34159	4.33705	4.84084	3.68127	4.83333	4.94818	4.97377	5.14004	4.83902	3.41049	3.93938	4.39891	4.6292
SCAMP3	6.20809	6.16738	6.08242	6.1731	6.8456	6.78345	6.39889	6.70331	5.62588	5.44182	5.74333	5.27073	5.67717	5.80281	5.51817	5.55538
CLK2	3.77812	3.65094	3.85859	3.75793	3.36499	4.50602	3.02401	4.59224	3.01155	3.48985	3.44632	3.09765	3.20095	4.06102	2.82086	3.01554
HCN3	0.75018	0.23642	0.78977	0.58759	2.72658	3.69524	1.78983	3.90006	0.92225	0.88241	1.36613	0.83047	1.182	1.83579	0.89697	0.86995
PKLR	-3.32193	-2.58598	-2.55433	-3.32193	3.14781	4.86664	2.50838	4.94046	-2.89476	-2.95918	-2.93555	-2.67846	-2.0628	-2.37655	-3.32193	-2.66328
FDPS	5.75485	5.65814	5.47707	5.40314	5.94549	7.08141	5.3961	6.96593	6.16931	5.73775	5.74416	4.65493	6.19025	6.7858	6.26773	6.24215
RUSC1-AS1	5.61388	5.52697	5.37769	5.3341	5.71054	6.98897	5.16801	6.88021	5.9485	5.4984	5.51259	4.40697	5.93499	6.52378	5.97653	5.93168
RUSC1	3.9925	4.02361	4.15148	3.78311	4.29834	4.36381	3.59705	4.45554	4.05657	3.81735	4.17912	3.64756	3.71481	3.98459	3.60041	3.87342
ASH1L	1.57988	1.80209	1.66785	2.10691	2.62906	1.49307	3.10708	1.65758	3.09547	3.02965	3.12183	3.9366	3.30009	3.43833	3.62706	3.80079
MIR555	3.61199	3.95663	3.53526	4.12517	4.83787	2.89854	5.05765	3.4165	5.34013	5.3722	5.52867	5.66344	5.27559	5.67864	5.79896	5.96409
ASH1L-AS1	1.64024	1.6773	1.25194	1.55405	1.69393	1.24593	1.83815	1.0602	0.80041	1.14059	1.31851	1.47389	2.12923	2.20483	2.30508	2.77942
MSTO1	3.99489	3.69551	3.52708	3.62339	4.91438	5.13962	4.42972	5.18525	4.24207	3.85683	4.35932	3.32896	4.18513	4.26458	4.19163	4.21884
YY1AP1	3.66342	3.36318	3.41824	3.39812	3.57266	3.57286	3.51454	3.73114	4.41738	4.10728	4.15387	4.34862	4.27037	4.1008	4.16031	4.0551
DAP3	5.8505	5.74379	5.60168	5.78203	4.81607	5.05959	5.03432	5.07473	5.38763	5.24954	5.35822	5.53398	5.59712	5.74012	5.038	5.10033
MSTO2P	4.64833	4.41654	4.24267	4.33383	5.44411	5.47875	5.04605	5.54583	4.78946	4.55098	4.83606	3.90236	4.83426	4.91809	4.81077	4.67209
GON4L	2.74185	2.52691	2.56847	2.93984	2.75295	2.73352	2.63842	3.03556	2.75143	2.62295	2.61824	3.06724	3.01292	2.98403	3.07524	3.37549
SYT11	-3.32193	-1.51718	-1.95496	-0.9676	-1.92384	-0.98754	-1.911	-0.84156	-0.69786	-0.02104	-0.58269	-0.26857	-3.16354	-0.89065	-0.68217	-1.11607
RIT1	3.47135	3.69959	3.57875	4.38201	2.42815	3.24045	2.85142	3.14388	2.79426	3.47495	2.91986	3.95546	3.79949	3.99235	3.72488	4.07019
KHDC4	3.17614	2.78378	3.13437	3.14783	3.06343	3.24367	2.8002	3.2804	1.80488	1.52398	1.64379	1.65779	2.98014	2.86864	2.37912	1.85122
ARHGEF2	4.64349	4.58996	4.20315	4.20817	2.84969	3.33368	2.96506	3.01493	4.8088	5.08196	5.0635	5.24561	3.47886	3.62567	3.93099	4.30134
SSR2	5.89388	5.67383	5.77441	5.76513	5.83789	7.04498	5.67153	6.65827	5.76579	5.64239	5.68109	5.61393	5.04288	4.97186	4.59152	4.65525
UBQLN4	4.19631	3.87564	3.84535	3.75386	5.12894	4.2843	4.62445	4.2095	5.57508	5.13928	5.44272	5.45578	5.70081	5.49539	6.16038	6.07972
LAMTOR2	5.01683	4.89056	4.90193	5.1117	4.91633	5.41865	4.32918	5.05975	5.7546	5.55277	5.73228	5.31671	5.25699	5.58131	4.45268	4.59829
MEX3A	1.49815	1.25712	1.47123	1.40263	0.30533	0.83074	-0.24249	0.78909	2.36534	2.06412	2.7222	1.80242	1.52609	1.57481	3.51419	2.98708
SEMA4A	-0.90964	-1.34485	-1.19444	-1.42322	-1.23879	0.09251	-1.94735	-0.70322	-2.58971	-2.91146	-2.27879	-2.8045	-2.40256	-2.88338	-3.32193	-3.17004
SLC25A44	2.34194	2.23592	2.09105	2.33069	4.46403	3.77615	4.49188	3.74859	2.74001	2.49082	2.78472	2.91366	3.36757	3.78477	3.46636	3.72879
PMF1-BGLAP	4.83491	4.53588	4.50166	4.23865	5.60804	5.12973	5.19562	5.02465	5.39322	4.74315	4.94296	4.15823	5.67757	5.60804	5.54925	5.30419
PMF1	4.12174	3.83172	3.6901	3.56581	4.72983	4.53057	4.21386	4.56403	4.46001	3.81337	4.03874	3.24813	4.8726	4.77167	4.56217	4.36019
BGLAP	1.80383	1.19919	1.07654	0.73496	0.9678	1.55362	-0.92644	1.64403	1.90259	0.84645	1.37844	0.03904	1.29301	0.87267	0.9304	1.09537
PAQR6	-0.38036	-0.42877	-0.16539	0.03413	1.61975	3.44336	-0.58996	3.02888	-0.78379	0.30492	0.28783	0.09129	-2.15426	-1.32883	-1.98904	-0.2965
SMG5	4.91576	4.35629	4.3456	3.95303	5.9396	5.02108	5.51778	4.66412	4.98727	4.12912	4.58709	4.16504	5.58379	4.82172	5.78111	5.1555
TMEM79	4.79488	4.89495	5.05042	5.23405	5.60552	6.53481	5.25732	6.48855	3.52515	3.80578	3.82285	3.41999	3.67224	3.98229	3.63481	4.13212
GLMP	5.63503	5.76047	5.86935	6.0122	6.63262	7.32238	6.24019	7.21589	4.31504	4.74961	4.83282	4.73547	4.34574	4.73508	4.72603	5.09615
TSACC	0.82422	1.09174	0.5868	0.50394	0.97265	1.61159	1.12378	2.16606	-0.77238	-0.99214	-1.36426	-2.10796	-0.04021	1.34778	0.51516	0.7332
MEF2D	3.82819	3.52093	3.63873	3.08305	4.28779	4.07562	3.96526	4.12173	4.64102	4.38728	4.62484	4.16897	3.98917	3.49814	4.18008	4.1474
IQGAP3	4.10198	3.62178	4.11706	3.08	3.81586	3.05813	3.38069	3.28382	4.4961	3.60089	4.01254	3.10821	4.03771	4.67422	4.96569	4.87046
NAXE	6.23079	6.03321	5.99856	5.73114	7.18358	7.0633	6.34481	6.85446	6.71316	6.23066	6.55934	5.6262	6.66551	6.47862	6.84052	6.76905
GPATCH4	4.95703	4.51194	3.97691	4.27631	4.32636	4.10154	4.36168	4.46811	3.74604	3.02606	3.69056	2.46963	4.66269	3.91059	4.12669	3.56401
BCAN	1.62786	1.93643	1.99901	1.466	2.86646	3.28449	2.13906	2.71752	0.75845	2.08009	1.87611	2.15122	2.45967	2.95508	3.37556	3.63085
NES	0.36395	1.06385	0.9656	1.32045	1.95744	1.49097	1.22771	1.65812	-1.84403	0.09696	-0.62215	0.99495	5.35345	5.63197	6.70022	6.75305
CRABP2	1.85911	2.75536	2.37823	2.70334	-0.45546	0.73935	-1.01403	0.89711	-0.12011	1.83589	-0.26722	1.12002	2.24585	3.05429	2.80139	3.17807
ISG20L2	4.1333	3.99036	3.6426	3.75556	3.99307	3.55055	3.84985	3.39275	3.34778	2.84173	3.3221	2.91699	4.1266	3.51763	3.74801	3.23018
RRNAD1	2.72031	3.15323	3.20332	2.4783	3.21966	4.04807	2.58451	3.98725	2.27919	2.61406	2.6415	2.34156	2.57338	3.02661	2.88664	3.23953
MRPL24	6.04518	5.64839	5.60922	5.15112	5.74201	6.21283	5.26449	6.15656	5.60563	5.58119	5.62459	4.34231	6.04198	5.88019	5.46916	5.3989
PRCC	5.40089	5.164	4.91671	4.79031	4.96647	4.59734	4.23622	4.77743	5.21603	4.74264	4.82587	4.10471	4.6064	4.38103	5.0476	4.94128

SH2D2A	6.07849	6.31187	6.10981	5.50161	-1.81753	-1.27793	-2.66493	-2.24985	0.09713	0.93915	-0.70814	0.24333	-3.32193	-2.75401	-3.32193	-3.32193
NTRK1	-0.87453	0.21401	-0.1486	0.06307	-3.32193	-3.32193	-3.32193	-3.32193	-3.32193	-1.48311	-1.81312	-2.57723	-3.32193	-3.32193	-3.32193	-2.56023
PEAR1	1.45341	1.10289	1.31865	0.49681	-2.1896	-1.38156	-2.28768	-1.4458	3.87939	4.05759	4.13314	3.47635	1.55962	-1.33711	0.34002	-1.71104
LRRC71	-2.19762	-1.3292	-2.06014	-2.51887	-3.32193	-2.94535	-3.32193	-1.86023	-0.47957	0.13173	0.29947	-0.1345	-2.21389	-2.77541	-2.2334	-2.2166
ARHGEF11	2.50706	2.20366	2.37732	2.391	3.7496	2.87793	3.54838	2.75986	2.96363	2.71823	3.10856	3.07231	3.50207	3.03139	3.59951	3.46049
KRT8P45	-1.4415	-0.39172	-0.44331	-1.72198	0.99329	0.7882	0.63406	-0.10571	1.30399	2.18525	1.93463	1.40183	-2.4419	-1.50954	-2.30458	-1.4671
ETV3L	-0.91036	0.96464	0.82797	0.83661	-0.3512	-2.47855	-0.08428	-2.37009	-0.268	-0.58181	-0.00701	-1.08208	-0.83248	-1.29827	0.45944	-0.93892
ETV3	2.81146	3.11208	2.88088	3.09484	2.69471	1.21598	2.78764	0.97955	3.2523	3.49153	3.43042	3.73302	2.93749	2.59118	3.27318	3.1606
KIRREL1-IT1	-0.39386	-0.5278	0.73675	0.95311	-3.32193	-3.32193	-3.32193	-1.95869	-0.83379	-0.71314	-0.96886	-1.51175	1.8974	1.58015	0.4461	1.11399
IFI16	5.2344	5.2415	5.32364	5.94788	-2.86399	-2.79118	-2.97291	-2.55853	4.50002	4.88909	4.82287	5.80119	2.2484	1.64216	1.50762	1.98651
AIM2	2.33484	3.0895	2.86506	3.93697	-3.32193	-3.32193	-3.32193	-3.32193	-3.32193	-3.32193	-3.32193	-1.64503	-3.32193	-3.32193	-3.32193	-3.32193
DUSP23	4.05808	3.98486	3.94196	3.53851	2.27934	3.98999	1.81752	3.70926	5.93091	6.55863	6.10243	5.09457	5.62662	6.11588	5.22399	5.26926
SLAMF8	-0.06662	-0.27421	-0.61611	-0.34601	-3.17134	-2.48539	-3.32193	-3.32193	-1.43051	-0.23547	-0.23207	0.01242	-2.6359	-2.46681	-3.32193	-2.50767
SNHG28	-0.09253	0.19526	0.02732	0.00149	-3.32193	-2.14877	-2.95493	-2.8687	-0.9082	0.16019	0.36677	0.13781	-2.17424	-1.89124	-2.65058	-1.68046
CFAP45	1.5257	1.49632	1.42872	1.29364	-2.14835	-1.12429	-2.07101	-1.26361	-0.47673	0.01771	-0.55226	-0.08794	0.24935	0.37873	-0.36311	-0.05052
IGSF9	-0.72853	-0.83648	-0.38478	-1.70391	-3.18513	-2.88344	-2.6929	-2.68306	-2.16814	-1.52112	-1.88989	-2.1003	-3.1953	-2.25365	-2.02716	-1.70987
SLAMF9	-0.14295	1.41258	1.7827	-0.2129	-3.32193	-3.32193	-3.32193	-3.32193	-1.58158	-0.42002	-2.0643	-2.61134	-3.32193	-3.32193	-3.32193	-3.32193
PIGM	2.29747	2.57757	2.01383	2.64855	3.0512	2.85606	2.96643	3.08743	2.31231	2.51022	2.43938	2.96662	2.45361	2.91089	2.63409	3.31293
IGSF8	5.18551	5.60776	5.41751	5.19435	6.01998	7.01369	5.34934	6.9342	4.61868	5.00285	4.99102	4.28027	3.16465	3.53899	3.98659	4.28988
PEA15	6.86852	6.82348	6.8015	6.35352	5.43178	4.71105	5.33083	4.63074	6.87232	6.53041	6.60595	5.41912	6.91799	6.50302	6.53633	6.03641
DCAF8	3.59686	3.50343	3.47933	3.69624	4.26294	4.0778	4.03223	3.99363	3.61495	3.64835	3.74525	4.06866	3.91825	3.88923	4.30169	4.33808
RPSAP18	0.30892	-0.02197	0.72831	-0.88274	-1.33051	-0.15664	-1.53406	-0.68745	0.79005	2.22077	0.54715	-0.23891	-2.56809	-1.71242	-3.32193	-1.17809
PEX19	3.79485	3.83759	3.60137	4.04111	3.74259	3.1387	3.77535	2.96691	3.66661	3.72276	3.71685	4.02355	4.12411	4.02583	3.61968	3.71393
SUMO1P3	2.30765	2.31365	2.47102	1.42135	2.54762	3.41729	2.35177	3.20317	2.36312	2.64494	2.28113	1.33357	1.63235	1.80243	0.853	1.73934
NCSTN	4.63585	4.64822	4.80643	5.04652	5.87872	5.62646	5.89895	5.48069	4.87138	5.02996	5.18895	5.55497	4.70416	4.62799	4.44198	4.72766
SLAMF7	-1.42275	-0.72574	-0.70444	-1.10499	-3.32193	-3.32193	-3.32193	-3.32193	-1.89973	-2.62645	-2.20789	-2.17274	-3.32193	-3.32193	-3.11613	-3.32193
F11R	4.12453	4.49772	4.48702	4.66878	6.43149	6.78461	6.55958	6.51917	5.60259	6.01445	6.03594	6.54645	2.36203	2.80995	3.44555	3.4033
TSTD1	1.63909	2.02135	2.71352	2.21241	-1.22804	0.7281	-2.18609	0.22414	1.84966	2.1672	2.05425	1.59393	3.47964	3.78341	3.38537	3.36497
USF1	4.54168	4.40646	4.42976	4.71776	4.60118	5.06424	4.41155	4.99243	4.49672	4.77254	4.95935	4.8109	3.98618	4.47906	4.46277	4.679
ARHGAP30	2.45808	2.73423	2.76939	2.45892	-2.43738	-1.6577	-2.3209	-1.22389	-2.04384	-1.50946	-1.20617	-1.65318	-3.01143	-2.64547	-2.79688	-2.39792
NECTIN4	0.76187	2.04605	2.05083	3.22228	-2.7655	-2.39874	-2.62539	-2.98439	-1.708	-0.75747	0.18032	-0.58368	-3.32193	-3.09172	-3.32193	-3.32193
KLHDC9	-1.43152	0.33899	0.41094	0.01638	-2.85383	-1.82372	-2.81482	-1.66804	-1.6906	-1.15811	-0.94147	-2.80817	0.07652	0.52461	0.60567	1.23005
PFDN2	8.59539	8.52449	8.41536	8.10216	6.87578	7.85893	6.78696	7.70356	7.63891	7.2331	7.50058	6.28382	6.81897	6.57201	5.71803	5.25313
NIT1	3.66262	3.80207	4.0046	4.18647	4.71371	4.77111	4.55444	4.72826	4.18395	3.94039	4.38212	3.87458	3.77714	3.58498	3.7488	3.21184
DEDD	3.62883	3.60207	3.79571	3.72936	4.49705	4.1755	4.35328	4.11101	4.47441	3.84796	4.47912	4.09416	4.36441	3.73659	4.35083	3.49427
UFC1	5.59677	5.57411	5.32953	5.64243	5.5773	5.56819	5.38223	5.50861	5.4043	5.08182	4.92099	4.36776	5.09218	4.43304	4.66969	4.18998
USP21	3.26388	3.07582	3.29968	3.191	3.13252	3.37256	2.36254	3.7301	3.16091	3.34214	3.788	3.36235	2.58405	2.8477	3.1665	3.03369
PPOX	2.56777	2.65148	2.53311	2.30089	3.06909	2.80351	2.5224	2.83859	2.70549	2.85755	2.75098	2.04981	2.83215	2.93024	3.04522	3.08201
B4GALT3	4.28784	3.78478	3.94865	3.77886	4.59989	4.43053	4.22105	4.44716	4.14016	4.00618	4.19449	3.7438	3.95385	3.57549	3.97104	3.93237
ADAMTS4	-2.39498	-2.14277	-2.04476	-1.00519	-2.75784	-2.8576	-3.32193	-2.97939	-3.32193	-2.87608	-3.32193	-2.80875	-3.32193	-3.08818	-3.22202	-2.87149
NDUFS2	5.41845	5.63946	5.52116	5.71325	5.69552	5.39365	5.65207	5.26144	5.27318	5.40495	5.57349	5.39428	5.36071	5.34973	5.02646	5.0444
FCER1G	4.57108	5.42402	5.1297	5.62031	-0.1739	1.67504	-0.23189	0.86685	-0.5962	-1.5868	-1.20551	-1.05404	-1.78975	-3.32193	-3.32193	-1.27136
APOA2	-0.11827	0.27045	-3.32193	-1.702	7.34866	11.2743	6.95047	10.9687	-1.78127	-0.79791	0.27029	-0.8182	-3.32193	-3.32193	-2.48671	-3.32193
TOMM40L	3.21036	3.09576	2.76756	2.93857	4.07525	3.8779	3.71104	3.77742	3.08631	3.41082	3.17354	2.95432	3.20673	3.35755	2.71996	3.26462
MIR5187	4.73615	4.45141	4.67282	4.54258	6.32018	4.51194	6.14241	4.87043	5.20545	5.88716	5.44209	5.12729	5.56385	5.65315	4.61324	5.85551
NR1I3	2.21902	2.00981	1.67272	2.06731	2.6074	3.00117	2.36223	3.0503	1.59487	1.79663	1.63996	1.69552	1.73896	2.18734	1.32136	1.5225
MPZ	-0.90947	-1.72637	-0.61057	-0.97319	0.51737	1.83224	-0.50348	0.82489	-2.44408	-3.32193	-2.86432	-2.898	-2.05205	-3.32193	-3.32193	-2.28289
SDHC	3.48533	3.5691	3.51163	3.74628	3.7269	3.48901	3.91412	3.38038	2.94497	3.03327	2.93794	3.13976	3.40584	3.4147	3.13707	3.3026
CFAP126	3.0039	2.99609	3.39974	3.20386	4.23559	2.98306	4.51379	2.70938	2.65016	2.80018	2.81962	2.87273	3.32167	3.33907	3.46126	3.80686
FCGR2A	0.10909	0.446	0.18674	0.17952	-3.15321	-2.9486	-2.97408	-3.09084	-2.30858	-1.38958	-1.99251	-0.80906	-3.16563	-3.02559	-3.1336	-2.80836

HSPA6	0.06941	0.74833	-0.06919	-0.11019	-0.01487	-0.45634	-0.11249	-0.63246	0.85773	0.87663	-0.71631	0.35524	-1.38801	-0.6515	-1.64942	-0.86982
FCGR2C	0.04574	0.39687	0.32859	0.5271	-3.32193	-3.32193	-3.32193	-3.32193	-3.32193	-3.32193	-3.32193	-3.32193	-3.32193	-3.32193	-3.05052	-3.32193
HSPA7	-0.27758	0.67066	0.56926	-1.49727	-2.18614	-2.4624	-3.32193	-2.75726	-1.58175	-1.03392	-2.44657	-2.50338	-3.32193	-2.6181	-2.85252	-1.41454
FCRLB	0.56304	0.62775	1.30846	1.07272	-2.13816	-1.82804	-2.0604	-0.9231	0.14969	0.29405	-0.53667	-0.40952	-1.30416	-1.58318	-2.42846	-1.46203
DUSP12	3.8844	3.6843	3.55941	3.73706	3.89337	3.69223	3.89741	3.86392	3.79417	3.78863	3.8599	3.43717	4.29003	4.31013	3.33155	3.47859
ATF6	3.08087	2.91063	2.93138	3.36737	3.65867	2.93147	4.111	2.48817	3.27183	2.92298	3.28994	3.70318	3.80282	2.87602	4.09583	3.55267
OLFML2B	-1.94849	-1.98249	-2.7004	-2.66767	-3.18899	-2.21831	-3.32193	-2.97674	3.62421	3.98906	4.30443	4.6664	3.37481	4.74516	4.45046	4.32947
NOS1AP	-1.83767	-2.09222	-1.84952	-2.22788	0.61701	-0.85098	0.09701	-0.55199	-0.32819	-0.42417	-0.27886	-0.80239	-1.0962	-1.33545	-0.77027	-0.81648
C1orf226	-0.47075	-0.47362	-0.70982	-1.30344	2.38363	1.47947	2.16991	1.38155	0.91548	1.09172	1.15609	1.06829	0.00906	0.46517	0.80612	0.96603
UHMK1	3.46654	3.79827	2.94071	3.81202	4.34893	2.42733	4.14682	2.91802	4.82708	4.23686	3.33754	4.5742	5.85594	5.23422	5.2467	5.41907
UAP1	6.00469	6.02314	5.67951	5.27949	4.34866	4.26549	4.67834	3.9903	6.89611	6.53972	6.89715	5.70315	5.31592	4.88882	4.68683	4.24512
DDR2	2.36423	2.1674	2.18235	2.85246	-2.31204	-2.06521	-2.21907	-2.02178	-2.44163	-2.2406	-2.51514	-2.11852	-2.57347	-2.43676	-3.05029	-3.13141
HSD17B7	0.66608	1.04577	0.58943	1.06741	1.96516	3.46232	1.93516	3.52276	1.89118	1.56917	1.52026	1.61428	2.29469	3.5328	2.03804	2.92468
RGS4	-2.47197	-2.69862	-3.0708	-2.17423	-3.2268	-3.10499	-3.32193	-3.32193	2.69596	3.32926	1.74786	2.79942	3.32302	2.95742	2.45371	2.82477
RGS5	-1.25637	-1.3447	-1.79224	-1.316	-1.91973	-2.66638	-1.29332	-2.08721	-1.62221	-0.77899	-1.52478	-0.79934	5.38821	6.0016	6.21124	5.98647
NUF2	4.19852	3.91423	3.91266	3.81644	3.29371	3.35472	3.66987	3.47385	4.1415	3.4458	3.15777	2.63869	4.73799	4.6902	3.88117	3.48913
PBX1	-2.5006	-2.86884	-2.45627	-2.18518	3.24021	2.1551	3.79891	2.09295	-3.26194	-3.08791	-3.02676	-2.71844	-3.32193	-3.24192	-3.17838	-2.70402
MGST3	3.44867	3.84271	3.95518	4.08739	3.46915	3.90674	3.78147	3.94239	3.35421	3.70534	3.32959	3.61543	3.74446	4.02149	2.78301	3.02535
ALDH9A1	4.90757	4.87563	4.89387	5.12277	4.67745	4.6347	4.94403	4.36923	5.85858	5.34251	5.66054	5.47556	5.16909	5.08052	4.83647	4.88031
TMCO1	4.864	4.97458	4.77878	5.0702	5.12116	5.03444	5.36553	4.92925	4.93783	4.77477	4.6813	4.61485	5.13237	5.05044	4.86073	4.75615
UCK2	4.55746	4.16139	3.93696	3.46398	4.45216	4.33835	4.17227	4.02727	5.46962	4.59129	5.24128	3.99114	5.36214	5.17524	5.10468	4.94985
MIR3658	8.39015	7.73616	7.51061	7.49433	7.26974	8.21515	7.58169	8.15623	8.57703	7.40794	8.12222	7.74321	9.66	9.28123	8.49191	8.04348
FAM78B	-2.80009	-2.4618	-2.5663	-2.87118	-2.86759	-3.12423	-3.32193	-2.71945	-0.53334	0.05449	-0.03981	-0.96264	-3.32193	-3.32193	-3.13456	-2.54897
POGK	2.61727	2.73512	2.49291	2.69313	3.54651	2.72293	3.89405	2.55593	3.27003	3.05525	3.1279	3.75527	3.96177	4.31686	4.60964	4.55675
TADA1	2.20331	1.99306	1.88743	2.35789	2.55972	2.29089	2.8656	2.22645	2.22249	2.08488	2.15108	2.02611	3.11048	3.17563	3.00499	2.72085
LINC01363	0.64854	1.36223	1.66726	1.06707	-3.01191	-3.32193	-3.32193	-3.32193	-3.32193	-3.32193	-3.32193	-3.32193	-3.32193	-3.32193	-3.32193	-3.32193
POU2F1	2.11508	1.83481	1.95909	1.84616	1.51598	1.37887	2.06957	1.74767	2.11695	0.57726	1.79433	1.53257	2.21745	0.86938	2.84951	1.55181
CREG1	5.59968	5.5859	5.45454	6.23241	5.09397	4.28424	4.60304	3.97506	4.61268	4.71393	4.03576	4.6879	4.03046	3.18839	2.85046	3.20621
RCSD1	1.02927	1.83118	0.75871	1.30137	-3.32193	-3.32193	-2.99608	-3.32193	-2.36096	-2.28835	-1.82218	-2.12863	-3.32193	-3.32193	-3.32193	-3.32193
MPZL1	4.89559	4.85726	5.05214	4.77415	5.05967	4.25249	5.13922	4.16602	4.71489	4.16251	4.58952	4.8006	5.45581	5.13176	5.39585	5.17006
ADCY10	-2.99671	-2.67337	-3.12057	-3.1083	0.5863	1.80741	1.21325	1.19875	-3.32193	-3.32193	-2.71419	-3.15862	-3.06119	-2.01137	-3.32193	-3.15409
MPC2	5.20194	5.53777	5.61662	5.54798	7.03519	6.80095	6.99377	6.78363	4.91396	5.00692	4.6613	4.24453	5.94137	6.33408	6.01581	5.84766
DCAF6	4.14065	4.3578	4.524	4.65136	5.71142	5.1031	5.78356	5.04155	4.35555	4.65407	4.35844	4.85336	5.08754	5.58942	5.47935	5.31277
GCSHP5	4.33429	3.98065	4.08832	4.30802	4.75152	4.78712	5.26322	4.93989	3.68805	3.79326	3.77127	3.61137	6.05769	6.08004	5.25338	5.17905
GPR161	1.06452	1.85433	2.08584	1.97532	2.41265	1.0012	2.40137	1.04661	2.02522	2.4632	2.32803	2.42327	2.2911	2.98875	3.74447	3.49414
TIPRL	4.5189	4.77166	4.55729	4.75642	4.2936	4.39129	4.75415	4.24081	4.64424	4.71029	4.48044	4.60004	4.9065	5.08132	4.50158	4.49588
SFT2D2	2.44719	2.79644	2.51221	2.76863	3.64819	3.30197	4.07669	3.22702	2.84941	2.43717	2.75168	3.52717	4.24879	4.12947	4.80603	4.79636
ANKRD36BP1	0.40223	0.85905	0.93282	0.26483	2.80583	-0.16169	3.75466	0.72832	2.03776	1.27131	1.55937	3.19494	3.99937	4.0004	4.55513	4.4611
TBX19	-0.81557	-0.52046	-0.78829	0.23218	-1.69957	0.12402	-1.32927	-0.21867	-2.52111	0.00423	-0.77347	-0.07807	-0.99962	0.46382	-0.37614	-0.10362
XCL1	-0.58261	-1.25627	-0.50355	-1.12542	0.6952	2.2303	0.75694	1.87934	-3.32193	-3.32193	-3.32193	-3.32193	-3.32193	-3.32193	-2.69707	-3.32193
ATP1B1	6.00608	5.76529	5.70656	5.6007	7.26036	6.0499	7.80795	6.09952	4.86872	3.80443	4.41826	3.7774	6.42504	5.98005	6.69132	5.8641
NME7	3.97128	3.88292	3.93791	3.75557	2.96542	3.28842	3.91666	3.28129	3.60418	3.17777	3.19871	2.71169	3.29866	3.15333	2.99385	2.40641
BLZF1	3.47931	3.71634	3.72972	4.06795	3.05715	2.43293	3.5816	1.96125	3.50049	3.29249	3.05824	3.76163	3.78874	3.05584	2.56071	2.56298
CCDC181	-2.20048	-2.41143	-2.77175	-2.00919	-1.48208	0.01622	-1.16001	-0.36614	-2.57067	-3.07225	-3.32193	-2.52035	0.58198	1.00862	0.77302	0.38364
SLC19A2	3.22328	2.93472	2.46865	2.93606	3.63555	2.70801	3.55622	2.68637	1.72014	1.27747	1.0351	1.27794	3.51674	3.15595	2.18933	2.46087
F5	-3.32193	-3.00764	-2.99204	-3.32193	2.64649	-0.24218	2.6929	-0.13214	-3.32193	-3.32193	-3.32193	-3.32193	-3.32193	-3.32193	-3.32193	-3.17682
C1orf112	2.60174	2.25008	2.39988	2.34813	2.50291	1.86819	2.98972	2.04887	2.13046	1.29748	1.78873	1.56592	3.11259	3.1658	3.06961	2.86477
METTL18	3.08087	2.40305	2.41798	3.14644	2.44005	2.24024	2.66535	2.18459	0.1751	0.92771	1.22765	1.10537	2.56519	1.92063	1.96481	1.3762
SCYL3	1.66827	1.47669	1.55192	1.98981	1.51038	1.46277	1.68505	1.70121	1.00033	0.78251	1.15443	1.54168	1.79315	1.75843	1.43003	1.44302
KIFAP3	2.23246	3.01431	2.88676	3.47279	1.97353	1.9977	2.89148	1.70975	2.54267	2.62969	2.53238	3.34138	3.10659	3.2766	2.95164	3.10263

GORAB	2.04677	2.34684	2.09333	2.75996	1.63879	1.7328	2.09311	1.95154	1.04309	1.15215	1.11572	1.28693	1.19642	1.27044	0.9606	0.85302
PRRX1	-0.13695	0.41793	-0.16537	1.00079	-3.32193	-3.07057	-3.32193	-3.16884	-3.32193	-3.32193	-3.1877	-3.19862	-2.94903	-2.35464	-2.39698	-2.45273
FMO3	-2.39861	-1.73101	-2.70742	-1.74638	-3.32193	-2.7827	-3.32193	-3.32193	-3.32193	-3.32193	-3.32193	-3.32193	-0.75725	0.38647	-0.03312	-0.48927
PFN1P1	5.35165	5.27093	5.06934	3.8071	3.31013	4.67328	2.64746	4.23787	5.25834	5.45873	5.07652	3.48955	2.31825	1.49641	1.98328	1.73246
VAMP4	1.15135	1.43315	1.53516	2.10338	0.7303	1.26111	1.56789	0.97153	0.90006	1.14926	1.21946	1.73872	1.69903	1.47385	1.65964	1.49363
EEF1AKNMT	4.8045	4.65706	4.42354	4.5228	4.82795	3.87163	4.73861	3.71696	4.22852	4.05866	4.49022	3.73004	4.74201	4.64311	4.74629	4.77669
DNM3	-3.32193	-3.21505	-3.10491	-2.98913	-3.24033	-2.9691	-2.92168	-3.20892	-3.10872	-2.98365	-3.13044	-2.84719	0.15623	2.05441	1.81323	1.81951
PIGC	2.12044	1.86836	2.38432	2.69501	2.97096	2.71984	3.3179	2.43774	2.57781	1.43664	2.17021	2.07925	3.16282	2.4634	3.17546	2.31338
SUCO	2.3847	2.69809	2.51752	3.03865	4.11682	3.61089	5.10878	3.52377	3.63983	3.75955	3.46185	4.45106	4.43589	4.84014	4.61285	4.94497
TNFSF18	-3.32193	-3.32193	-3.32193	-3.32193	-3.32193	-3.32193	-3.32193	-3.32193	-3.32193	-2.8649	-3.32193	-3.32193	3.54064	3.97816	0.03219	1.69182
PRDX6	6.90465	6.73117	6.5304	7.03235	6.3079	7.04996	6.23753	7.0651	6.87355	6.62015	6.61812	6.69155	7.25127	6.89941	6.52278	6.44198
KLHL20	2.05954	1.92845	1.77764	2.16963	2.00189	1.62683	2.16793	1.45808	2.5477	2.58345	2.41558	2.87373	2.67882	2.80008	2.12109	2.39594
CENPL	2.07802	1.88007	1.92075	1.86752	2.74571	1.27232	2.98166	1.33534	2.53384	1.93787	2.14387	1.83741	3.26718	3.26579	2.83024	2.60046
DARS2	4.41469	4.37425	4.36027	4.41779	5.18602	4.09739	5.54784	4.06098	4.72185	4.05961	4.34523	4.43298	4.82196	5.01596	4.74391	4.78505
GAS5-AS1	4.6653	4.33511	4.54957	3.41797	3.55876	5.10268	3.54686	5.23815	4.53033	5.242	4.45917	3.75041	5.26911	4.29043	2.70185	2.84673
GAS5	5.62741	5.22983	5.32956	5.11882	4.29208	5.08874	4.25508	5.20308	5.1398	5.83728	5.27078	5.12315	5.32993	4.6203	3.38497	3.21056
ZBTB37	-1.31098	-1.27478	-1.33056	-0.93556	-0.09713	-0.44027	-0.02056	0.16919	-0.22519	-0.58807	-0.92832	0.40572	0.82734	0.82248	0.91203	1.0864
SERPINC1	-2.79688	-2.33247	-3.32193	-2.68578	-0.24169	0.87262	-0.9721	1.51969	-3.32193	-2.81268	-2.78115	-3.32193	-2.29922	-2.56886	-3.32193	-2.12963
RC3H1	1.4311	1.59184	1.22478	1.85788	1.67511	0.63452	1.9001	1.07958	1.5852	1.45095	1.45833	2.76696	2.25898	2.42351	2.2378	2.65829
RABGAP1L	-0.42992	-0.11418	0.12407	0.25251	1.07445	0.49425	1.66443	0.39806	1.10736	0.88102	1.35694	1.7542	1.12991	0.59811	1.37799	0.74741
CACYBP	6.24679	6.17331	5.83778	6.05804	5.65854	5.57837	5.96042	5.71052	5.6495	5.08514	5.18893	4.82525	6.60232	6.54459	5.97458	5.82524
MRPS14	3.69994	3.50889	3.54734	3.5479	3.3454	3.47397	3.19798	3.36147	3.87839	3.47526	3.66846	3.14803	3.70993	3.03645	2.99715	2.44445
COP1	3.46944	3.68321	3.6019	3.9356	4.41903	4.11378	4.54466	3.91123	4.08862	3.93363	3.89842	3.89764	4.05728	3.90656	3.96148	3.91241
SEC16B	-1.42066	-1.62431	-1.19583	-0.72572	0.65081	1.32425	0.45086	0.90837	-1.81891	-1.01257	-1.38319	-0.78902	-1.45897	-1.22257	-1.59497	-1.09047
CRYZL2P-SEC16	-0.90098	-0.76407	-0.84337	-0.46439	1.17681	1.95716	1.21829	1.36967	0.26844	0.3276	0.37819	0.36927	0.43961	0.59839	0.25851	0.61314
CRYZL2P	0.44499	0.74243	0.54742	0.34217	1.60703	3.29161	1.94652	2.55683	1.79576	1.6386	1.71346	2.05846	2.27265	2.25112	1.63096	2.25618
RASAL2-AS1	1.6158	1.4658	1.82147	0.07928	1.48554	0.37746	0.73832	0.35101	2.28876	1.7671	2.49273	-1.17293	1.73378	1.37543	1.84444	1.56613
RASAL2	2.51996	2.51455	2.55505	2.36124	1.43458	0.76914	1.57355	0.94405	2.57507	2.53613	2.81878	2.63529	3.76505	3.40419	3.65827	3.32046
C1orf220	-1.75741	-2.61897	-1.21308	-2.27983	-0.06607	-0.71548	-0.01796	-0.44999	-1.61015	-2.69956	-2.41951	-2.47761	-2.25459	-0.4478	-0.99844	-1.32111
RALGPS2	1.672	1.57333	1.96235	1.88657	2.77802	1.70641	3.53909	1.73493	2.44809	2.1205	2.30835	2.61174	3.3952	2.62448	3.28511	2.44994
ANGPTL1	-3.32193	-2.7255	-3.32193	-2.95689	-0.68257	-0.51551	-0.10428	-0.4661	-3.32193	-3.32193	-3.01594	-2.80348	-1.75368	-1.37758	-1.98607	-1.84657
FAM20B	3.94908	3.84606	3.77254	3.44481	4.39902	3.27451	4.6911	3.21283	4.3077	3.72375	4.10501	4.10174	4.64904	4.45819	4.67031	4.46993
TOR3A	3.39537	2.94597	2.95031	3.14644	5.07223	4.17713	4.78189	4.14606	3.73551	3.31878	3.82797	2.74267	3.9656	4.23743	3.40073	3.94617
ABL2	3.60686	3.8078	3.47343	3.81967	2.02354	1.55642	2.02605	1.7299	4.24927	3.76534	3.22572	4.11126	4.08874	3.38437	3.76421	3.70742
SOAT1	4.53404	4.74948	4.41714	5.04426	4.94436	4.43575	5.36925	4.48101	3.92048	3.44079	3.66573	4.13997	4.4497	4.84658	4.43474	4.88149
TOR1AIP2	3.44807	3.35341	3.39299	3.23449	2.82121	2.21014	3.15203	2.20599	2.18873	1.95108	2.08702	2.47633	3.02391	2.16184	3.00395	2.42878
TOR1AIP1	2.71672	2.9177	2.68284	3.01415	3.18897	2.46444	3.63713	2.15043	3.04827	2.65084	2.86288	3.40999	3.5317	3.18259	3.58115	3.38442
CEP350	1.23403	1.34286	1.48564	1.82065	2.58509	1.2459	3.16352	1.61487	2.21275	2.19854	2.03467	3.10334	3.12332	2.89074	3.14169	3.33916
LHX4	-0.4339	-0.63004	0.50573	-0.73427	-0.38933	-0.44536	-0.82071	-0.46503	-1.03804	-1.24795	-0.79198	-1.49442	0.20583	0.09711	-0.64852	0.32151
ACBD6	3.39854	3.18752	3.27197	3.02403	2.99494	2.79973	2.78439	2.73333	3.38404	2.86063	3.30019	2.60046	3.71052	3.06331	3.65512	2.88352
XPR1	2.09027	2.12634	2.03816	2.33378	3.9332	2.94112	4.35928	2.82126	3.48876	3.05682	3.05153	4.05198	4.26679	4.41818	4.80515	4.88973
KIAA1614	-1.12721	-0.9954	-0.88382	-1.36472	-2.14501	-2.11497	-2.08917	-1.83088	0.32232	0.78438	0.76632	0.34384	-2.0052	-1.54097	-1.30305	-1.15469
KIAA1614-AS1	0.73381	0.5169	0.76242	0.24931	-1.10083	-1.4248	-0.99286	-1.04503	1.03004	1.31658	1.83257	1.26258	-0.36663	-0.62824	0.10797	-0.16983
STX6	2.99506	3.1444	2.85895	3.36183	3.29276	2.7655	3.32499	2.78028	3.7048	3.90077	3.08389	4.14812	3.97978	4.39405	3.94876	4.3832
MR1	1.00093	0.94597	1.01297	1.71184	1.26033	1.56273	1.48387	0.90883	1.09509	1.37735	1.35606	1.91672	0.57089	0.8096	1.06872	1.38944
IER5	6.19468	6.12047	6.16691	5.1042	3.78277	4.10336	3.44091	4.19431	5.55376	5.3962	5.3979	3.99835	3.27317	3.38457	2.58302	3.30243
GLUL	1.54523	1.34491	1.09496	1.35753	3.89994	4.17113	3.887	3.95062	3.0073	2.99792	3.183	3.20024	3.15251	3.32498	4.24998	3.92464
RNASEL	-0.72057	0.01469	-0.33906	0.08377	-3.04066	-3.14801	-3.0156	-3.1194	-0.60014	-0.08451	0.11703	0.91827	-0.03862	0.67234	0.41575	1.45745
RGS16	-0.86854	-1.01502	-1.06233	0.59736	-1.53757	-0.94251	-2.94508	-1.17683	-2.52001	-1.97605	-2.90912	-3.32193	-1.99817	-2.32298	-1.98474	-2.15311
NPL	1.02464	0.53433	0.57159	1.52449	0.40679	0.74615	0.85101	-0.04082	-1.35796	-2.08687	-2.12477	-1.6356	-0.46731	-1.10362	-1.17601	-1.54127

DHX9	5.94965	5.82026	5.62945	5.87518	5.62218	4.43236	6.16466	4.58151	5.76478	5.52103	6.036	6.30628	6.40626	6.34374	6.77424	6.54102
LAMC2	7.35365	7.46261	7.95384	7.63199	0.97007	-2.64416	2.12893	-2.13938	4.44455	4.83759	5.05222	5.3092	0.50548	-0.7369	-1.14855	1.00717
NMNAT2	1.37975	1.57012	1.67481	2.11739	-3.13541	-3.32193	-3.32193	-3.14709	2.8073	3.07288	2.79365	3.34239	-2.64925	-2.43784	-2.45217	-1.12217
SMG7-AS1	-1.70615	-1.97426	-1.53693	-2.21192	-0.76814	-1.59093	-0.92058	-1.42325	-0.1954	0.01011	-0.13915	-1.0248	-0.77281	-0.42521	-0.67648	0.28618
SMG7	3.53448	3.30338	3.24414	3.47439	3.50634	2.94219	3.70435	2.99447	3.60556	3.38396	3.57683	4.11175	4.17434	3.98968	4.34831	4.37655
NCF2	2.70369	3.49272	3.12176	3.72069	-1.34851	-2.97937	-1.01193	-2.61657	-3.32193	-3.32193	-2.25266	-2.31823	-2.8208	-2.62525	-2.73106	-2.72038
ARPC5	3.81882	2.67375	4.23687	2.98223	3.96382	1.59691	4.48797	1.38468	4.60896	2.46763	4.33576	2.16026	5.09249	2.2175	5.0841	2.12773
RGL1	0.90322	1.17064	0.88798	1.94455	-0.28497	-1.70328	0.05974	-1.96235	0.90192	0.39315	0.8938	1.00005	0.20722	0.77427	0.92742	1.56334
COLGALT2	-3.1732	-2.74899	-2.99159	-3.32193	-3.19464	-3.32193	-2.93888	-3.32193	0.1987	-0.12534	0.34478	0.38347	2.49093	1.9658	2.43521	2.33295
TSEN15	3.60553	3.52041	3.51462	3.81318	3.1815	3.4664	3.507	3.48175	4.11029	3.89097	4.04636	3.91054	5.1962	5.52838	5.35603	4.77516
C1orf21	1.42718	1.82622	1.84259	1.90446	-2.41789	-3.22226	-2.71002	-3.20537	2.01391	2.14567	1.77701	2.25531	-2.90776	-2.40844	-2.46554	-3.13534
EDEM3	2.60337	2.74112	2.69733	3.61654	3.76858	2.65859	4.35983	2.25829	2.26102	2.26853	2.74501	3.40508	3.51162	2.95076	3.42419	3
RNF2	3.08156	2.97129	3.15816	3.0112	2.86682	2.6023	3.35845	2.26049	3.74302	3.06184	3.22621	3.92445	3.99989	3.20454	3.85524	3.01389
TRMT1L	2.78904	2.86541	2.99037	3.46428	3.39395	2.28585	3.81221	2.32471	2.94625	2.78248	3.00327	3.66364	3.42933	3.23257	3.36313	3.26014
SWT1	0.60094	1.02426	0.97074	1.69682	0.82777	0.80348	0.96976	0.70101	0.27815	0.33691	0.45342	1.62126	1.26888	1.64793	1.26488	1.63593
IVNS1ABP	3.67536	3.75761	3.72164	4.13119	3.39705	3.34975	3.82578	3.19133	4.50364	4.85102	4.53663	4.75146	6.12624	6.39756	6.02329	5.93062
HMCN1	-3.20616	-2.96429	-3.18576	-3.0458	-2.74008	-3.09672	-2.84691	-3.25197	-3.12574	-1.95959	-2.29434	-0.5352	-2.01583	-2.07726	-1.72067	-1.25852
PRG4	1.64674	1.48243	1.44627	1.78284	1.07913	1.59683	0.77285	1.88963	1.23483	0.24263	0.30069	0.57119	2.27065	2.19546	1.97071	1.40744
TPR	3.81908	3.86812	3.86085	4.12584	4.04051	3.47644	4.4543	3.5632	4.63677	4.05007	4.23395	4.90206	5.24998	5.03118	5.76427	5.46958
ODR4	2.63173	2.58569	2.60999	3.1545	3.38442	3.48011	4.1201	3.50643	1.97518	2.05688	1.52774	2.50177	3.91962	3.38792	2.86877	2.71206
PTGS2	1.18906	2.08176	2.10624	4.19706	-1.62055	-2.94579	-1.99283	-3.08902	-1.41911	-0.6768	-1.67287	-0.00913	-3.32193	-3.32193	-3.32193	-3.32193
PLA2G4A	1.73962	2.21567	1.92977	3.27952	-1.41441	-1.53262	-0.35778	-2.9202	0.68362	0.73283	0.52842	1.46547	-2.28107	-1.41905	-1.70749	-0.55731
MIR4426	5.85801	5.86232	5.19328	6.16268	5.78233	5.74577	5.72785	5.50854	5.94659	6.47892	5.81386	5.7811	4.59862	5.64697	3.70467	4.08158
RGS2	-0.60948	-0.31018	-1.14896	-0.70323	0.65834	1.89978	1.35279	1.6092	1.8597	4.1106	2.16123	4.74677	2.83779	3.32821	2.0087	2.10153
UCHL5	4.29487	4.0894	3.89303	3.94738	3.93097	3.47901	4.41557	3.46576	3.7514	3.04956	3.17891	2.94476	4.38566	4.05703	3.73146	3.31753
GLRX2	4.8013	4.64268	4.41517	4.87973	4.77496	5.21923	5.4555	5.14934	4.35391	3.89232	4.03371	3.95477	5.0467	5.00222	4.02179	3.79892
CDC73	3.34335	3.2953	3.15599	3.44638	4.0701	2.80554	4.51013	2.62962	4.62602	4.53177	4.30375	4.81267	4.65708	4.54828	4.4303	4.25591
KCNT2	-3.32193	-3.32193	-3.14624	-3.32193	-1.40143	-1.07164	-0.73701	-1.96566	-2.99525	-2.01407	-2.39143	-1.91156	-1.06851	0.94571	-0.03404	1.61615
CFH	-3.32193	-3.32193	-3.32193	-3.15805	0.92051	0.15264	0.75124	-0.18111	0.3619	1.05986	0.80611	1.8927	-0.15398	1.42286	0.36937	2.56812
CFHR1	-3.32193	-3.32193	-3.32193	-3.32193	-0.5143	-0.73686	-2.3297	-1.06444	0.00484	-0.1787	0.16952	-0.65329	-0.88148	-0.96112	-2.31341	-0.16344
F13B	-3.32193	-3.32193	-3.32193	-3.32193	3.58092	-0.63376	3.69632	0.00617	-3.32193	-3.32193	-3.32193	-3.32193	-3.32193	-3.00313	-3.32193	-3.32193
ASPM	2.9205	2.75472	2.69334	2.37927	4.08776	2.12427	4.60734	2.5975	4.07226	3.25791	3.46528	3.43361	4.85531	4.8161	5.01895	4.87169
ZBTB41	0.20353	0.26162	0.49588	1.17078	1.57888	0.20662	2.61857	0.39871	1.87236	2.03375	1.53524	2.68362	2.67144	2.29834	2.8033	2.42313
DENND1B	-1.05261	-0.67155	-0.76735	-0.11448	0.32586	-0.1107	1.02641	-0.11243	0.36614	0.79759	0.53187	1.5431	1.45113	1.55381	1.60449	1.75266
C1orf53	1.09248	1.87355	1.32644	0.08697	0.99145	1.88599	1.2344	1.77562	2.68905	2.84888	2.68174	2.66943	2.09748	2.89489	1.99308	2.06717
LHX9	-3.21849	-3.09931	-3.0879	-3.32193	-3.27709	-3.32193	-3.32193	-3.32193	-0.65371	-0.75014	-1.46677	-0.60681	-3.163	-2.77184	-2.54824	-2.8755
NEK7	2.97346	2.67564	2.86624	2.8817	3.03894	1.42733	3.73709	1.49038	3.73769	3.53296	3.16954	4.08533	3.54923	2.88348	3.36454	2.54702
PTPRC	-3.32193	-3.32193	-3.32193	-2.7679	-3.32193	-3.32193	-3.32193	-3.32193	-2.91778	-3.32193	-3.32193	-3.32193	-2.41763	0.14541	-0.8131	-0.20247
NR5A2	-1.02866	-0.834	0.08538	-0.34562	2.9027	1.51672	3.21735	1.84997	-1.46371	-1.16316	-1.49299	-0.61085	3.43703	3.08336	4.41966	3.86874
LINC00862	-1.0255	-1.13039	-0.22904	-0.82871	0.27644	-0.01445	0.83919	-0.0402	-2.96103	-2.55014	-1.98858	-1.69606	-0.20711	-1.11191	-1.22822	-1.28557
ZNF281	4.13526	4.3659	4.3509	4.96738	4.04	4.04576	4.49232	4.30245	3.91311	4.16162	4.29616	4.78536	4.51921	4.55188	5.03952	5.02236
KIF14	3.13974	2.95854	2.81753	3.12605	3.13179	1.61102	3.73022	2.02523	3.55802	2.76889	2.68005	2.7403	4.5466	4.52435	4.27241	4.28851
DDX59	2.09911	1.90469	2.10999	2.49149	2.16119	2.01162	2.39339	2.08077	1.96132	1.77193	2.02045	1.88937	3.09545	3.13111	2.18355	2.46076
CAMSAP2	2.88658	3.08762	2.94971	3.65363	3.36187	2.52369	4.174	2.49045	2.99364	3.35793	3.24441	4.39483	4.60785	4.91809	5.29396	5.20529
INAVA	4.0371	4.00868	3.8086	4.22692	4.27462	3.64326	3.83661	3.70907	0.67802	2.61221	1.25545	3.54438	-0.0183	1.30619	0.67096	2.55206
KIF21B	2.47716	2.33955	1.89136	2.10208	0.50159	1.27803	0.10819	0.9941	1.9251	1.93608	1.3421	2.63884	-2.0284	-1.71573	-1.1401	-0.40634
ASCL5	-1.98147	-2.42667	-3.32193	-1.35786	-1.84184	1.51096	-1.5133	1.85137	-3.32193	-2.86838	-2.479	-2.25879	-3.32193	-2.94447	-3.32193	-3.32193
TMEM9	4.90475	4.95346	4.91356	4.94754	6.30514	7.34158	5.88085	7.31053	4.60942	4.90312	4.82884	4.4652	4.70591	5.21703	5.01578	5.19932
IGFN1	5.46065	5.31739	5.09165	4.89969	-2.88505	-1.58571	-3.15896	-1.86125	-1.92244	-1.04997	-1.27967	-1.59615	3.0653	3.94923	2.1769	3.08878
PKP1	-3.32193	-3.32193	-3.32193	-2.92632	-3.24733	-3.32193	-3.32193	-3.32193	-3.12627	-3.32193	-2.84856	-3.16041	-1.75734	-1.00016	-0.62415	-0.10592

LAD1	-2.79007	-2.73662	-1.81173	-2.43897	0.90321	-0.86059	0.57918	-2.70818	-2.29595	-3.32193	-2.56259	-3.32193	-1.86448	-1.86775	-1.67664	-1.75748
PHLDA3	4.88301	5.39104	5.54955	4.77057	-0.53903	-0.99968	-0.95578	-1.45498	6.00212	6.10907	6.36093	4.98563	3.8304	3.60263	4.75846	3.8827
CSRP1	4.29367	4.31089	4.28674	3.98327	1.88891	1.74961	2.09209	1.70803	4.00115	4.22927	3.30555	3.57307	3.27442	3.23194	3.597	3.31376
RPS10P7	-0.32227	0.28936	0.22152	0.03634	-2.13684	0.42937	-1.8027	0.36635	-1.42995	-0.00034	-1.28386	-0.65052	-1.10315	-0.31764	-0.50531	-0.23794
NAV1	2.11007	2.17849	2.40511	2.05899	-3.08784	-2.96624	-3.06662	-3.05221	3.67767	3.44091	3.67661	4.21453	0.93892	1.12284	1.90691	2.67453
IPO9-AS1	4.3102	4.20359	4.09261	3.71961	3.95368	1.17365	3.83385	1.4375	5.36601	4.90977	5.25141	5.28204	4.54195	4.83725	5.23548	5.39273
IPO9	3.33796	3.42952	3.3243	3.48557	3.709	3.12122	3.60374	3.24288	3.81447	3.54474	3.7708	3.87935	4.36165	4.67353	4.74709	4.91671
SHISA4	1.34757	1.86488	2.40969	2.68729	2.85522	3.95982	1.77375	3.5781	3.99659	4.39941	4.40533	3.27288	2.9692	3.59769	4.17748	4.46834
TIMM17A	4.76126	4.57626	4.33826	4.56651	5.27815	4.93291	5.51204	4.71114	4.95027	4.72536	4.87974	3.91407	6.02364	6.02118	5.27349	5.22406
RNPEP	4.66157	4.66333	4.69167	4.58556	5.27531	5.45329	5.06795	5.39587	6.21001	5.85986	6.01761	5.99882	5.69043	5.67991	5.82291	5.7509
ELF3-AS1	0.57517	0.3213	0.48536	0.27278	4.558	4.49722	4.21225	4.60214	2.63736	1.94055	2.53192	1.94286	1.82312	2.20514	1.75808	2.17818
ELF3	-1.2758	-0.68756	-0.80313	-0.6949	5.70381	6.28527	5.20565	6.10847	4.99104	4.64477	5.04364	3.54625	2.82461	4.76889	3.21754	3.91126
GPR37L1	-1.34182	-0.52486	-1.15545	-0.68844	-0.47309	0.59027	-1.01193	0.28804	-1.36749	-0.88085	-1.21923	-0.25653	0.51504	1.0289	0.3948	0.01181
ARL8A	4.34333	4.87279	4.64966	4.72748	4.69408	5.91015	4.53207	5.36732	4.76483	4.99518	4.9598	4.40247	5.6555	6.03956	5.88368	5.81162
UBE2T	5.72813	5.27149	4.90972	5.1165	5.05519	5.29642	4.89194	5.52752	5.10815	4.69434	4.6705	4.04205	6.76559	7.17363	5.64349	5.45873
PPP1R12B	-1.02775	-1.08279	-0.87529	-0.74279	-0.31751	-1.10469	-0.0908	-1.0293	-0.88623	-1.02697	-0.84184	-0.19799	0.42945	0.57984	0.50476	0.73754
KDM5B	2.73267	2.87581	3.09818	3.33262	2.3001	2.39839	2.94765	2.0988	3.34226	3.50017	3.76388	4.67262	3.50826	3.0896	4.52398	4.7985
PCAT6	0.94957	1.23341	2.63122	1.5667	1.79506	4.54701	0.5693	3.83894	0.69639	2.3633	0.93113	0.12702	0.68078	0.24999	0.02268	1.45317
RABIF	3.07111	3.3967	2.8658	3.19088	3.504	3.45716	3.14328	3.1924	3.11081	2.74884	1.97749	2.48035	4.73326	4.37118	3.5619	3.85485
KLHL12	3.00962	2.5335	2.76101	2.65681	3.48407	2.81041	3.19147	3.00946	3.54614	2.73853	3.16196	2.52997	4.09578	3.39298	3.24923	2.85662
ADIPOR1	5.14938	5.33126	5.30462	5.46078	5.83294	5.88945	5.833	5.71467	5.12315	4.98195	5.2478	5.281	5.53702	5.86015	5.83122	5.82096
CYB5R1	3.78472	3.76143	3.67786	3.9195	4.32395	4.24687	4.1727	4.15614	3.68693	3.84201	3.95138	3.36136	4.17323	4.14636	4.30933	4.21443
TMEM183A	4.46571	4.23392	4.05022	4.2905	4.81001	4.75789	4.86509	4.7086	4.86494	4.63474	4.62665	4.63061	5.38538	5.38649	5.44294	5.34419
PPFIA4	-0.70908	-0.33823	0.58681	-1.31185	-2.33297	-0.65848	-2.78807	-1.20807	-0.64605	0.47256	-0.24221	0.06689	-2.79739	-2.3229	-2.32744	-1.9222
ADORA1	-3.07681	-2.44896	-3.32193	-3.01884	-2.30582	-2.6788	-3.0919	-2.59006	2.70367	3.75988	2.91156	3.14449	-0.90173	-0.94554	-0.7924	-1.92881
CHI3L1	-0.90861	0.83757	0.61349	3.62611	-3.32193	-3.32193	-2.81081	-3.32193	-3.32193	-3.32193	-3.32193	-2.80411	-2.88169	-2.88304	-3.32193	-2.58492
BTG2	-0.26547	-0.72369	-0.8188	-0.36733	2.23358	1.65329	2.21598	1.34725	4.61531	2.88266	4.84925	3.73733	0.82873	0.28778	0.26539	-0.11381
PRELP	-3.13917	-2.63817	-3.32193	-3.32193	-3.09287	-3.32193	-3.32193	-3.32193	-1.32825	-2.20288	-1.1086	-2.30458	-3.17684	-3.32193	-3.14696	-3.32193
ATP2B4	1.71616	2.36322	2.77486	2.58405	0.36378	-0.69752	0.47435	-1.3775	4.81385	4.15192	4.6493	5.35441	3.30306	2.30712	4.37238	4.1084
ZC3H11A	3.20395	3.21975	3.37889	3.75789	3.12866	2.84683	3.59568	2.91668	3.34867	3.54798	3.77557	4.76253	4.77231	4.44036	4.50562	4.47926
ZBED6	3.23785	3.25239	3.41374	3.79217	3.16401	2.88006	3.63225	2.94825	3.3855	3.58603	3.81612	4.80285	4.80962	4.47984	4.55227	4.53656
SNRPE	6.62067	6.55109	6.49968	6.05248	6.17207	6.21657	6.10147	6.25157	6.66291	6.47418	6.37332	5.34453	7.1081	7.10037	6.09526	6.1405
SOX13	2.22695	2.47792	2.64875	2.27906	2.70075	2.37193	1.91126	2.62846	2.2755	3.31972	3.20704	3.44604	2.1382	2.67889	3.13394	3.43669
ETNK2	1.38764	2.15814	2.00976	1.98404	1.25396	1.60784	0.97306	1.31876	3.76721	3.95855	4.13105	3.85922	3.02182	3.35338	4.33386	4.16734
ERLNC1	-0.77969	0.57779	0.79343	1.12983	0.31229	0.64478	0.59977	-0.4038	2.77187	2.80993	2.84177	3.16778	2.3414	2.90141	3.47119	3.6255
KISS1	-0.40201	0.11494	0.18587	0.26967	-0.41335	2.28072	-0.71098	0.94322	2.37562	1.33791	2.24775	-0.46864	3.49169	0.92184	-0.6837	0.22435
GOLT1A	1.27674	2.05804	1.19245	2.19689	4.04133	5.99211	3.80762	5.7079	-1.56389	0.43482	-1.13962	-1.23842	1.12924	1.0886	0.27369	1.66307
PLEKHA6	2.33296	2.02867	1.87423	1.92334	3.79146	2.52814	3.42633	2.92524	-1.46432	-0.61417	-1.02981	-0.78208	1.71847	1.72634	1.52809	2.45877
PPP1R15B	4.26154	4.29271	4.10422	4.34919	4.81181	4.22965	5.16258	4.101	4.0197	3.90602	3.99627	4.78169	5.231	5.29778	5.02395	5.06481
PIK3C2B	0.6022	0.70206	0.79983	0.9264	2.437	1.70082	2.0789	1.8165	1.24558	1.74482	1.8858	2.15487	1.69842	2.26248	2.92176	3.17478
MDM4	0.63451	0.49942	0.89073	0.71352	0.79488	2.27303	1.11098	2.54083	1.09249	1.48147	1.57421	2.36354	2.25821	2.52557	2.27269	2.46332
NFASC	-2.87316	-2.82671	-2.63919	-2.3799	-3.00238	-3.12335	-3.13767	-3.20185	1.1728	1.28675	1.46322	1.27765	-3.32193	-3.28141	-3.22476	-3.22268
TMEM81	1.50516	0.73949	0.8685	1.15908	0.59507	1.81431	1.00261	1.86175	0.66903	0.83642	0.73932	0.45465	1.61689	1.69533	1.89375	1.81215
RBBP5	2.8235	2.88731	2.6168	2.95063	3.34367	2.49151	3.6891	2.41691	3.52735	3.09811	3.30264	3.50592	4.02023	3.72363	4.14839	3.95825
DSTYK	2.17957	2.01374	1.94033	2.48206	2.05367	1.62435	2.04044	1.79757	2.72285	2.58627	2.25876	3.1604	3.31692	3.31182	3.39185	3.78337
TMCC2	1.11358	1.17537	0.8513	1.13237	0.62381	0.2326	0.02778	-0.21348	1.73763	1.58243	1.47958	0.67537	1.82952	1.60477	2.41268	2.08749
NUAK2	3.95047	3.87598	3.53816	2.86301	3.58696	2.62436	3.80255	2.62362	5.12149	4.54807	4.81927	4.18502	6.27176	5.80699	5.89463	4.91383
SNRPGP10	6.15994	6.38095	6.05987	5.6982	5.90026	6.0217	5.27568	6.21646	6.12649	5.33442	5.70795	5.03564	5.20368	4.98209	4.05022	4.19198
BLACAT1	-1.47704	-0.61525	-1.19159	-0.68132	-3.32193	-3.32193	-3.32193	-3.32193	-0.85734	0.53589	-0.32517	0.92139	-3.32193	-3.32193	-3.32193	-3.32193
CDK18	-0.33448	0.55042	0.16736	-0.03663	1.11132	2.81997	0.8957	2.29599	-0.20761	1.24362	0.15345	0.47768	-2.88252	-3.08634	-3.17335	-2.90766

MFSD4A	-2.97927	-2.78452	-3.10929	-2.08697	-2.22527	-2.26241	-2.32122	-1.62946	-2.93058	-3.14655	-2.96835	-2.99522	-0.77827	0.18389	0.06633	-0.01522
ELK4	1.09266	1.46182	1.2134	1.77251	1.90426	1.55443	2.24186	1.61592	2.11356	2.03139	1.9291	2.98935	3.55002	3.80702	3.94082	3.65755
SLC45A3	3.70899	3.79834	3.58463	3.37418	2.89813	3.15328	1.80945	2.4159	2.66965	2.92221	2.77245	2.18647	1.86698	0.97506	1.70601	2.03192
RAB29	3.86936	3.89172	3.78808	4.56979	4.36854	4.11794	4.55014	3.93276	3.27763	3.24624	3.07484	3.51525	4.88261	5.02238	5.02204	4.99291
SLC41A1	2.98658	2.45199	2.41185	2.27711	3.35646	1.85522	2.89957	1.55885	4.24056	3.62298	4.00552	3.57775	3.82183	3.38021	3.57103	3.5723
RAB7B	-2.14346	-2.1965	-2.51592	-1.08273	-3.19836	-3.32193	-3.32193	-2.99949	-1.64264	-1.10323	-1.38246	-0.36091	-2.10801	-2.3319	-3.32193	-2.16303
FAM72A	3.68493	3.0961	3.085	2.85247	3.38441	3.02651	3.23082	2.929	3.39522	2.6581	2.81759	1.75996	4.13514	3.93987	3.45564	3.08214
SRGAP2	2.2326	2.1325	2.25732	2.3993	2.24199	1.54025	2.06226	1.25399	3.73112	3.61035	3.41221	4.11763	3.39067	3.36406	3.67992	4.04897
IKBKE	3.94422	4.15515	4.03554	4.05888	3.97176	2.25016	2.95664	2.1955	4.48832	5.03094	4.7084	5.26565	3.625	3.90025	3.13399	4.19853
C1orf147	2.08652	2.53453	2.00156	2.55983	1.7666	0.15166	0.78619	0.41488	2.24744	2.72216	2.48166	3.45244	1.1108	1.59431	0.95484	1.82082
RASSF5	1.42882	1.27659	1.10849	1.38628	0.4302	-1.08556	-0.50977	-1.00872	-1.24026	-0.36608	-2.00098	-0.69712	-1.91264	-1.0787	-3.04401	-1.6568
EIF2D	2.72802	2.29806	2.31951	2.27093	3.07631	3.42047	2.96665	3.17823	3.20438	3.19652	3.2648	3.24063	3.48336	3.23403	3.65185	3.30686
DYRK3	2.71741	2.75049	2.65827	2.53958	1.28948	0.85724	2.01192	0.85365	2.77339	2.37144	2.78364	2.64558	2.43039	2.27931	2.75662	2.66647
MAPKAPK2	4.68401	4.58255	4.59495	4.49622	4.27491	4.7658	4.15672	4.38964	4.47713	4.39294	4.4048	4.6207	5.4562	5.61522	5.91279	5.74696
IL24	4.16859	4.5229	2.91341	5.00594	-3.09967	-3.32193	-2.87189	-3.32193	2.0713	3.31217	2.11277	3.47311	-3.32193	-3.32193	-3.32193	-3.32193
FCMR	0.72558	0.36426	0.05638	0.13944	-3.32193	-3.32193	-3.32193	-3.32193	2.11314	1.40597	1.25957	0.79031	-2.71984	-3.32193	-2.61601	-3.32193
C1orf116	-1.97048	-2.04397	-2.20434	-1.59292	-2.54043	-3.12958	-2.37954	-2.20127	-2.91275	-2.25967	-2.41367	-3.32193	-2.10399	-1.21641	-1.33536	-1.36186
PFKFB2	0.87607	0.84117	0.65885	1.06278	1.75332	1.42116	2.14161	1.58784	1.3952	1.05265	1.20648	0.97732	2.45064	2.74313	2.73595	2.87176
YOD1	1.6863	1.70162	1.48191	2.0915	2.42142	1.90079	2.6191	2.22854	3.00237	2.42127	1.95798	2.83603	4.12381	4.13786	3.74486	4.01566
C4BPB	-0.46022	-0.983	-0.77203	-0.92653	-0.83445	-0.26651	-1.72474	0.25065	-2.67992	-3.02034	-2.73741	-3.02495	-2.22882	-2.10935	-3.02304	-2.36502
CD55	3.42244	3.56103	3.37915	3.70609	-2.31733	-2.0533	-1.76039	-2.10387	2.23805	2.07025	1.65389	2.34906	4.08413	3.27758	2.88614	2.8937
CR1L	-0.00397	-0.17335	0.41043	-0.12954	-3.32193	-2.67393	-3.32193	-2.90671	-3.32193	-2.97667	-2.95404	-2.70687	-3.32193	-3.32193	-3.32193	-3.32193
CD46	3.82237	3.50769	3.90679	4.1546	5.22905	3.81524	5.29009	3.68996	3.42764	2.80054	2.86093	3.14623	5.60476	4.05643	4.24853	3.3649
MIR29B2CHG	-2.1059	-2.0398	-1.93046	-2.00504	-1.99705	-1.6449	-2.19041	-1.37096	-3.17157	-2.97325	-3.12425	-2.92844	-2.90707	-2.24701	-2.65609	-2.64435
CD34	-3.20531	-2.96187	-3.05952	-3.17621	-3.12908	-2.50138	-3.01654	-3.05857	-0.96862	-0.80881	-0.52898	-0.50114	-3.32193	-3.32193	-3.32193	-3.32193
PLXNA2	1.01468	0.91754	0.49338	0.38806	2.53243	1.98781	2.43971	1.71449	-2.38348	-2.8571	-2.55631	-3.10759	-3.19993	-3.14319	-3.17457	-2.91077
CAMK1G	-0.90226	-1.12631	-1.55417	-1.34774	-3.15434	-2.95094	-2.97627	-3.32193	2.17513	3.04728	2.49271	3.25674	-3.32193	-3.32193	-3.32193	-3.32193
MIR4260	10.6364	10.5649	10.7282	10.0302	6.19779	3.41155	6.81447	1.51379	9.51152	9.5086	9.38997	8.93627	3.57261	3.67355	2.75186	4.09225
HSD11B1-AS1	3.12492	3.66689	3.72334	2.94485	-3.32193	-1.22088	-0.84778	-3.32193	0.30422	1.62344	0.53451	0.23846	-3.32193	-3.32193	-3.32193	-3.32193
HSD11B1	3.77002	4.2424	4.18668	4.52912	-3.32193	-3.32193	-3.32193	-3.32193	-1.12736	0.05636	-1.67779	-1.1831	-3.32193	-3.32193	-3.32193	-3.32193
TRAF3IP3	-0.83479	-0.69653	-0.68494	-0.5266	-2.72701	-1.93233	-2.44119	-1.9064	-0.94604	-0.92987	-1.6072	-0.57859	-1.24581	-0.31547	-0.56987	-0.6495
C1orf74	1.20399	1.27782	1.22519	1.349	-0.46134	-0.40857	-0.44588	-0.66173	0.71439	0.88096	0.71796	1.00047	0.95852	1.63268	1.47994	1.56659
IRF6	-3.32193	-3.32193	-2.86338	-3.05968	-1.11357	-2.60944	-1.34082	-2.51352	-0.02764	0.75156	0.13469	0.66175	-2.86656	-3.004	-2.94172	-3.32193
UTP25	2.27346	2.08658	1.91068	2.42212	1.77656	0.80627	2.11708	0.86162	2.1891	2.44296	2.30538	2.82035	2.63777	2.85508	2.25631	2.77498
SYT14	-3.24368	-3.15187	-3.22968	-3.04565	-3.32193	-3.32193	-3.24876	-3.32193	-3.06571	-2.55754	-3.02217	-2.10728	-1.07251	-0.64503	-0.78864	-0.91688
SERTAD4-AS1	3.25262	3.07544	3.30733	2.22854	-3.32193	-3.32193	-3.32193	-3.32193	2.50358	1.62373	1.41283	0.26212	2.83291	1.52401	3.1146	0.68593
SERTAD4	1.64333	1.26931	1.34411	1.15877	-3.32193	-3.32193	-3.32193	-3.32193	1.57106	-0.09686	0.5822	0.05946	1.57559	0.27289	1.84678	0.22249
HHAT	1.31273	0.97281	0.73514	0.10263	-1.12857	-0.58386	-1.60345	-0.95173	0.67289	0.28177	-0.19292	0.05896	-0.58282	-0.9699	0.0835	0.37573
KCNH1	-2.76161	-2.79195	-3.0741	-2.55726	-3.32193	-3.10787	-3.32193	-3.19221	-0.40969	-0.10253	-1.04357	-1.24319	-0.3327	0.62173	0.32821	0.1011
RCOR3	1.71548	1.73918	1.89157	2.14797	1.14167	1.57662	1.56198	2.11227	1.75461	1.75538	1.72299	2.34392	2.56679	2.21765	2.3214	2.08977
TRAF5	0.12339	0.50874	0.73942	0.98459	-0.02304	0.60192	0.15287	0.78817	0.36727	0.43574	0.26909	1.3111	2.2582	2.72259	2.18637	2.45447
LINC00467	0.52963	0.35058	0.62951	0.897	-1.33489	0.07405	-0.97942	-0.07263	1.11685	0.74986	1.24948	0.92828	1.2192	0.49327	0.53545	0.05175
SNX25P1	-0.82291	-1.69478	0.12731	-0.11661	1.59679	1.68032	1.69027	1.62692	-2.26417	-1.83921	-3.32193	-2.4095	2.37946	1.55247	1.76019	1.18874
SLC30A1	3.32347	3.48848	3.16495	4.05489	4.52922	2.75216	5.13906	2.83435	3.85671	3.88328	3.99399	4.57593	4.47781	4.20476	4.64195	4.69589
NEK2	3.98396	3.61909	3.57221	3.47874	4.52476	3.47955	4.54641	3.86746	4.89592	4.04183	4.16565	3.51894	5.63076	5.91397	5.41592	5.24816
LPGAT1	2.72648	2.64797	2.50064	2.67108	5.9097	4.72926	5.70009	4.91444	3.70134	3.28094	2.45711	4.0874	5.4581	4.92454	5.06498	5.30179
INTS7	2.40267	2.36969	2.20748	2.70077	3.0135	2.4883	3.55784	2.86709	2.08563	1.67815	1.85889	2.57196	4.21375	4.45062	3.94538	4.09342
DTL	4.11604	3.60074	3.3399	3.15207	3.36059	2.04911	3.37866	2.33303	4.34111	2.46857	3.13496	2.33741	5.01656	4.83141	4.75197	4.40332
RPL21P28	4.04353	3.68989	3.74695	2.05556	2.96563	4.16763	1.99993	3.94391	3.71966	4.52561	3.01617	3.4683	4.64533	3.98001	2.71006	2.16693
PPP2R5A	2.3481	2.33401	2.45542	2.3635	4.78472	4.22554	5.04021	4.09103	4.30696	4.04751	4.04584	3.96915	4.99946	5.18228	4.72607	4.87217

NENF	5.32456	5.27999	5.31984	4.90395	6.29327	7.07379	5.50462	6.97899	5.93638	5.87736	5.99045	5.36355	6.26647	5.98313	5.80819	5.71211
ATF3	0.58727	0.33842	0.63841	0.49529	1.84807	2.43884	2.2945	1.85553	3.02085	1.78786	2.12395	1.1338	1.86268	1.59663	0.91007	0.66087
BATF3	0.34813	0.6572	0.5077	-0.41493	2.81257	3.02006	2.69646	2.46209	3.10644	2.51096	2.74604	1.19956	2.88875	2.29975	2.84827	1.58658
NSL1	2.7809	2.52176	2.61965	2.75659	3.38926	3.34356	3.54834	3.22172	3.1854	2.85858	2.74913	2.78999	4.09366	3.9817	3.87231	3.54256
TATDN3	1.67529	1.76067	1.65923	2.43359	1.88171	2.32471	2.13226	2.14985	1.90449	1.8936	2.1334	1.92237	2.73315	2.7211	2.29062	2.06311
FLVCR1-DT	-0.8585	-0.31495	-0.65533	-0.72101	1.34464	2.08205	0.8923	2.21259	0.30701	-0.66164	-0.69031	-0.62829	-0.02277	0.49774	-0.97953	-0.44132
FLVCR1	2.08967	2.09253	1.93559	2.00316	5.28323	4.41576	4.88908	4.62836	1.09123	0.49856	-0.09509	0.56752	3.66037	3.4346	2.84281	3.38165
VASH2	-2.02789	-2.66635	-2.63739	-1.76562	0.5571	1.10223	0.96924	0.63493	-1.36611	-2.66512	-1.80679	-2.46612	1.16442	2.25128	1.96363	2.25087
ANGEL2	1.5805	1.65672	1.50705	2.1272	1.72175	0.91862	1.81769	1.46029	2.11369	2.30288	1.3585	2.76431	2.7118	3.04038	2.21479	2.6491
RPS6KC1	2.38284	2.62422	2.84808	3.24101	3.07344	2.61392	3.69105	2.44727	2.72247	2.94806	2.72975	3.48259	3.03111	3.17886	3.28342	3.16525
PROX1-AS1	-3.32193	-3.32193	-3.32193	-3.32193	-0.08058	-1.12525	-0.31465	-0.91427	-3.32193	-3.32193	-3.32193	-3.32193	-3.32193	-3.32193	-3.32193	-3.32193
PROX1	-3.32193	-3.32193	-3.18051	-3.32193	2.88735	1.47091	3.24494	1.74717	-2.8322	-3.32193	-3.19753	-3.0032	-3.22688	-3.13832	-3.32193	-2.37097
SMYD2	2.59388	3.00202	3.03336	2.96635	3.67451	3.79237	4.03693	3.36931	3.51238	4.06785	3.78304	3.83099	3.42905	3.84933	4.0719	3.72312
PTPN14	2.10158	2.3432	2.62162	2.56288	1.83274	0.47364	2.62483	1.10593	3.58375	4.28012	4.64418	4.87617	4.09948	4.81498	5.82433	4.93926
CENPF	4.30829	3.99212	3.93511	3.84449	4.36964	2.90897	4.9224	3.29585	5.35077	4.18425	4.57043	4.36452	5.85477	5.90795	6.45119	6.20424
KCNK2	-3.32193	-3.32193	-3.32193	-2.51313	-3.32193	-3.32193	-3.32193	-3.32193	-0.2947	-0.24042	-0.14887	-0.54933	-3.32193	-2.93246	-3.32193	-3.32193
KCTD3	3.57023	3.46465	3.16065	3.60476	4.23763	3.54721	4.39336	3.66085	4.45515	4.53855	4.07905	4.83924	4.67728	5.07966	4.55133	4.6238
GPATCH2	0.95991	1.0986	0.73245	1.48306	1.1209	0.48238	1.41086	0.58536	1.32198	1.35453	1.11519	1.40538	1.95061	1.75646	1.78894	2.00975
SPATA17	-2.54391	-2.1822	-2.2317	-1.55376	1.06586	-0.39127	1.56454	0.51794	-2.5629	-2.07188	-2.01106	-1.72128	-1.89974	-1.75979	-1.00554	-2.06188
SPATA17-AS1	-1.81202	-1.70176	-0.40206	-3.32193	3.73171	1.93988	4.52615	2.78943	-0.91706	-0.01309	-1.77877	-1.15085	-0.27955	-0.28407	0.7552	-0.28398
RRP15	2.98142	2.77418	2.35819	2.76008	2.4144	2.02215	2.96373	2.04965	2.37645	1.70973	2.25129	2.04686	3.51582	2.86212	3.28179	2.55914
TGFB2-AS1	2.93061	2.59527	2.55526	1.35763	-1.13584	-3.32193	-1.47341	-1.21269	5.38841	4.24116	5.30402	2.11336	5.97175	5.29892	6.74048	5.3681
TGFB2	3.84336	3.32341	3.52886	3.26462	-0.538	-1.41806	0.44975	-0.08574	5.2136	4.02038	4.93691	4.12909	6.02837	5.53336	6.60015	4.915
TGFB2-OT1	3.91663	3.57511	3.89285	3.76625	-1.8981	-0.45482	-0.08089	0.31574	4.8897	3.83172	4.61792	4.11045	6.18301	5.79714	6.68282	5.06229
LYPLAL1-DT	4.18719	4.60196	4.52902	4.37887	3.51557	3.36293	3.66767	2.8043	3.86335	3.74792	3.48609	2.60782	4.128	4.02986	4.09507	4.01883
LYPLAL1	3.48478	3.83354	3.60031	4.13272	2.59855	3.10311	2.85931	2.94959	2.93416	2.9056	2.58723	2.56186	3.98026	4.07295	3.32816	3.10918
ZC3H11B	-1.73101	-1.31773	-1.40297	-1.49799	-1.81005	-1.24414	-1.72023	-2.6453	-1.86021	-0.94215	-1.69678	0.18062	-1.88472	-1.63239	-1.48932	-1.22666
SLC30A10	-2.97821	-3.32193	-3.32193	-3.32193	1.51563	-1.92577	1.15993	-1.49013	-3.32193	-3.32193	-3.13373	-3.32193	-3.32193	-3.32193	-3.32193	-3.32193
BPNT1	4.12536	4.32367	4.14841	4.32131	4.7162	4.65863	5.00342	4.60274	3.53305	3.73583	3.80686	3.72437	5.026	5.27477	4.98366	4.99984
IARS2	5.21727	5.24946	5.14209	5.50336	6.24485	5.35235	6.65801	5.40032	5.71827	5.49373	5.50972	5.90866	6.76459	6.94853	7.00081	6.99683
RAB3GAP2	2.41608	2.57156	2.69057	3.11638	2.72956	2.29339	3.4774	2.39702	2.78166	2.51463	3.03	3.4785	4.04645	3.24538	4.21973	3.51901
MORF4L1P1	4.21412	3.97129	3.95457	3.95427	4.9099	4.09256	4.84769	3.95136	5.30265	5.04747	4.09969	4.75674	4.42483	4.66033	3.55429	3.65001
MARK1	-2.54256	-2.12879	-1.56645	-2.5366	-3.15624	-3.32193	-3.32193	-3.32193	0.84694	0.76778	0.99987	0.79068	-3.02975	-3.32193	-3.13696	-3.32193
C1orf115	-0.27284	0.13149	-0.27334	-0.34195	4.14933	3.22385	3.39246	3.70062	3.3797	3.20853	3.16689	2.80258	4.28282	4.70633	4.31945	4.45378
HLX-AS1	2.88875	2.61693	1.81955	1.01028	-3.32193	-3.32193	-3.32193	-3.32193	4.09466	4.1947	4.42769	2.9654	2.542	3.36784	3.40998	3.40506
HLX	2.30064	1.64708	1.36137	1.20065	-3.16298	-3.32193	-3.14816	-3.10396	2.81421	3.18662	3.35248	2.55696	1.53261	2.33498	2.17376	2.73775
DUSP10	5.07862	4.34826	4.94496	4.12511	0.74185	-0.09727	0.60132	-0.38598	3.80739	3.36764	4.29938	3.93307	2.52474	2.11819	2.44363	2.10789
LINC02474	3.2951	2.68109	3.22061	2.41576	-3.32193	-3.32193	-3.32193	-3.32193	-2.59038	-3.32193	-3.32193	-3.32193	-3.32193	-3.32193	-3.32193	-3.32193
LINC01705	3.24653	3.87483	3.30985	4.05849	-3.32193	-3.32193	-3.32193	-3.32193	-1.94053	-1.00142	-3.32193	-1.46659	-1.66026	-3.32193	-3.32193	-3.32193
HHIPL2	1.80655	2.64875	2.73764	3.63608	-1.88698	-1.66782	-2.25062	-2.05688	-2.2535	-0.68185	-1.34613	-0.65089	-2.86803	-3.32193	-3.0288	-3.02294
TAF1A	2.69014	2.47936	2.2048	2.5904	1.29013	0.95843	1.51233	1.05449	2.39858	2.18476	2.20136	2.005	2.31402	2.18166	1.56888	1.77508
TAF1A-AS1	1.09354	1.09938	0.75303	1.04233	0.07692	0.91525	0.67707	0.86309	0.61444	1.04359	0.94847	-0.05497	0.44149	0.95494	0.29193	1.1305
MIA3	3.4225	3.40488	3.4704	3.92644	3.28461	3.2298	3.68688	3.20641	3.35961	3.03459	3.384	4.03098	4.17076	3.32711	4.47983	4.15746
AIDA	4.58804	4.86208	4.73255	5.05441	4.73575	3.60539	5.02869	3.63256	5.14136	4.8692	4.66493	5.4306	6.0344	5.31311	5.88995	5.30409
BROX	3.56313	3.71115	3.82469	4.2559	3.37562	2.66087	4.11472	2.56572	3.33103	3.58526	3.38972	4.2677	4.03775	4.03048	4.61486	4.60631
DISP1	-0.98913	-1.35349	-0.8332	-0.43994	1.31531	1.18426	1.90608	0.68651	-1.5792	-0.913	-0.61542	-0.16343	-1.95363	-1.89507	-1.31151	-1.28772
CAPN8	-3.32193	-3.09816	-3.32193	-3.32193	2.88399	1.45677	3.4481	0.3079	-3.32193	-2.95647	-3.32193	-3.32193	-3.32193	-3.32193	-3.12946	-2.9526
TP53BP2	4.1535	4.54537	4.3606	4.28873	3.50341	2.71601	3.82289	2.48167	4.29722	4.01369	4.15501	4.36265	4.97781	4.69984	5.17511	4.97742
GTF2IP20	2.17598	2.06252	2.37293	2.10684	0.53793	1.65443	-0.03815	2.19422	-0.10929	0.12838	-0.09839	0.82257	0.76463	1.52467	-0.06176	1.31827
FBXO28	3.17592	3.51094	3.3657	3.8429	3.268	2.43914	3.96887	2.44512	3.35961	3.55333	3.02411	4.47372	4.31458	4.23307	4.24738	4.31828

DEGS1	5.93915	5.99465	6.19992	6.56214	5.86197	5.62547	6.29055	5.5672	5.59208	5.75705	5.20163	6.41084	7.2152	6.70246	6.41908	6.13231
NVL	2.65726	2.60236	2.48102	2.6896	2.75048	2.73073	3.06414	2.82405	3.05154	3.15147	2.95125	3.47533	3.37445	3.7756	3.40179	3.55782
CNIH4	4.54611	4.68928	4.6904	4.68475	4.78788	4.7675	4.80567	4.4731	4.46801	4.11438	4.17247	3.50002	5.23488	4.95196	5.1376	4.62832
WDR26	3.74251	3.97503	3.85674	4.4596	3.19989	3.31945	3.53025	3.34208	3.76513	3.94174	3.51962	4.57242	4.37804	4.20853	4.07396	4.23777
CNIH3	0.57095	1.05517	1.32455	1.56253	-1.06855	-1.43309	-1.04182	-1.72558	0.84415	2.54371	1.53358	3.14858	-1.69213	-1.30901	0.42393	1.02375
DNAH14	-0.08406	-0.18682	-0.35533	-0.02093	-0.05588	0.22884	0.05393	0.05964	0.05533	-0.30959	-0.34759	0.02728	0.47571	0.24239	-0.34956	-0.23452
LBR	4.47352	4.25224	4.05105	4.24498	5.62247	4.50174	5.93622	4.83882	5.59099	4.96181	4.98978	5.12951	5.82422	6.1385	5.60923	5.6999
ENAH	3.13888	3.09955	3.09332	3.10091	3.80894	2.82677	4.1088	2.78122	2.84439	2.84281	2.84747	3.10762	4.56457	4.85658	5.51586	5.12675
SRP9	6.8821	6.82112	6.77622	6.97698	6.60449	6.31169	7.22864	6.08744	7.08684	6.67441	6.70492	6.3839	8.34094	8.01111	7.60096	7.17776
EPHX1	4.51967	4.8069	4.71169	5.50763	5.33989	5.57561	4.81498	5.74958	2.98286	2.77485	2.95043	3.18221	5.3395	5.38778	6.08109	6.05413
TMEM63A	1.96289	1.83306	1.9123	1.95295	3.60869	3.37632	3.37273	3.10434	1.55803	1.7659	1.97878	2.1346	2.44826	2.53212	2.6941	3.00077
PYCR2	4.48411	4.0399	3.90765	3.78187	4.80035	5.23656	3.96598	5.27698	4.59953	3.92453	4.14297	3.24734	4.75167	4.5252	3.80804	3.81371
SDE2	3.4967	3.57437	3.14467	3.4359	3.67571	3.03455	4.06836	2.87689	3.35681	3.47923	2.86002	3.72299	4.19399	4.57389	4.2608	4.30893
LINC01703	-0.07278	-0.07576	-2.23789	0.2315	1.01945	1.93645	0.50957	1.79626	0.12292	0.7222	-0.17879	-0.45933	1.34709	1.46293	0.00369	1.47265
ACBD3	4.64522	4.90443	4.85851	5.22904	5.09223	4.38936	5.60928	4.11557	4.83749	5.12194	5.00747	5.29094	4.8566	5.10106	5.05017	5.07711
ACBD3-AS1	4.49734	4.76178	4.81323	5.15495	5.46643	3.71744	5.81498	3.36181	5.00622	5.24831	5.24111	5.71416	4.94951	4.93373	5.24984	5.14942
LIN9	2.32473	1.81242	1.753	2.22893	2.15571	1.34362	2.31513	1.63847	2.00707	1.52607	1.34683	1.53569	3.22125	2.95199	2.52484	2.3052
PARP1	5.20335	4.68589	4.52481	4.3902	5.43421	4.25732	5.29383	4.1964	5.92101	4.99402	5.64406	4.98079	5.85784	5.33461	5.86601	5.24937
ITPKB	-2.32716	-2.24176	-1.82135	-2.34025	-3.13221	-2.90554	-3.18054	-3.32193	1.59614	2.29822	2.27908	2.56926	2.50655	2.32431	3.37703	3.79366
PSEN2	4.47063	4.30997	4.39596	4.16054	4.25037	3.99199	4.09848	4.08769	3.22232	2.74898	3.15845	3.03887	3.81779	2.9246	3.64872	3.76602
COQ8A	3.04308	2.6934	2.41895	2.50891	2.66445	2.25089	1.75699	2.44664	2.98998	3.14633	3.21534	2.29925	2.57825	2.11601	2.26148	2.62327
CDC42BPA	1.46263	1.59122	1.59972	1.84498	2.96985	1.69995	3.33845	1.93509	2.84907	2.69582	2.62245	3.40686	4.73509	4.37462	4.95739	4.75002
ZNF678	-0.74606	-0.83842	-0.67574	-0.18393	0.29161	0.40436	0.98937	0.58293	0.72093	1.12562	-0.14775	1.54722	2.51944	3.19414	2.52616	3.20412
SNAP47	4.02529	4.00119	3.76245	3.80659	4.25708	4.25803	3.82598	4.17228	3.70817	3.73145	3.76704	3.63096	3.92153	3.95033	4.07913	4.10954
JMJD4	3.42842	3.48328	3.23778	3.24108	4.40071	4.62462	3.8213	4.57572	3.09178	3.26375	3.38165	3.0385	3.3688	3.34588	3.68667	3.66905
WNT9A	3.72619	4.06233	4.43987	3.93999	0.85595	-0.1674	1.67873	0.00952	-2.78154	-1.3857	-2.17474	-1.26888	-0.8782	-0.26473	-1.02366	0.66693
MIR3620	4.30368	3.73685	4.54709	1.93382	4.27002	3.70188	4.34163	4.81477	3.52599	4.45215	4.28073	4.22703	4.83681	4.7518	3.38097	3.41223
C1orf35	4.14727	4.15818	4.21265	3.71749	3.84497	4.47454	3.05425	4.69605	3.65314	3.53181	3.66224	2.63954	4.15029	4.49209	3.77489	3.90434
MRPL55	4.41788	4.24023	4.17712	3.96653	4.559	5.42896	4.03448	5.52085	4.29395	3.88194	3.91704	3.14382	4.78492	4.79823	3.97971	3.92977
GUK1	6.24081	6.11308	6.06994	5.70395	5.55567	6.29868	4.83268	5.91393	6.28219	6.43152	6.29074	5.63278	5.52604	5.57894	5.44845	5.59826
GJC2	0.39074	1.25786	1.35593	0.35547	-1.14339	-0.32247	-2.34449	-0.11541	-0.02205	0.34236	0.14231	-0.22692	-0.52206	-0.70553	-0.01162	0.19758
IBA57	0.19924	0.06519	-0.17982	-0.67271	1.38199	0.26074	1.13649	0.33949	0.98576	0.87635	1.25197	0.4887	1.54318	1.19018	2.05933	1.46118
OBSCN-AS1	-2.81034	-3.01291	-2.73289	-2.46916	-1.74256	-0.85765	-0.79398	-1.30849	-2.32956	-1.294	-1.74462	-0.38505	-1.58463	-0.26714	0.37715	-0.01013
OBSCN	-2.5186	-1.80516	-2.01115	-1.95577	-0.81982	-0.24114	-1.03462	-0.6367	-1.19082	0.12392	-0.57198	0.64208	-1.16333	-0.06268	0.3608	0.85593
TRIM11	3.50105	3.39239	3.23762	2.48912	3.11981	3.20757	2.52065	3.21145	3.13567	3.32643	3.33042	2.52966	3.33751	3.53511	3.52836	3.55637
TRIM17	-1.98208	-1.4341	-1.96864	-1.63972	-0.52578	-1.07099	-1.03517	0.01527	-1.30359	-1.1247	-0.97739	-0.35047	-1.55672	-1.07194	-1.00922	-0.51929
RNF187	5.74706	5.77419	5.8443	5.48186	6.09131	6.19888	5.21101	5.90785	6.76292	7.08697	6.96603	6.36359	5.43571	6.10283	6.18635	6.49805
DUSP5P1	-2.57226	-3.32193	-2.46811	-3.32193	-0.38532	-1.70517	-1.49646	-0.58353	0.071	0.74587	0.84329	1.3162	0.0108	2.47998	0.84116	1.06687
FTH1P2	6.32822	6.37842	6.5657	5.76343	2.00729	3.59685	2.05114	2.26461	3.03591	4.28813	3.18219	4.07782	2.26981	0.65127	2.50475	2.44125
RHOU	-3.32193	-3.32193	-3.04655	-3.0302	4.49037	3.64207	4.85793	3.42515	-1.1495	-0.78759	-0.65902	-0.68572	1.93629	0.13458	1.84457	1.41354
RAB4A	2.7316	2.51241	2.82223	2.75705	4.14214	3.9239	4.48984	3.97689	3.99165	3.58686	3.64808	3.93586	4.83286	4.54738	4.24069	4.01463
CCSAP	1.29544	0.75329	1.26601	0.56234	2.50424	1.39557	3.14485	0.95786	2.91589	1.92767	2.12924	2.08642	3.50487	2.71415	3.70658	2.79842
ACTA1	2.01632	2.37	-0.91449	-2.11358	-2.106	1.99429	-1.76691	2.50011	4.05082	1.67977	1.86769	1.61017	-3.32193	-1.93178	-1.74535	-0.60543
NUP133	3.02539	2.88051	2.91468	3.20995	3.58952	2.82949	4.03356	2.68851	3.84835	3.50628	3.69086	3.99134	4.51231	4.59427	4.8896	4.72773
ABCB10	2.88998	2.62738	2.73252	2.67982	4.67085	2.45033	4.27568	2.84652	3.74312	2.65303	3.08858	2.46683	4.41383	2.64052	3.8904	2.53472
TAF5L	2.41059	2.19211	2.16148	2.05286	2.82711	1.71992	3.00569	1.72076	3.00632	2.41072	2.71869	2.83934	3.89013	3.34259	3.71542	2.92388
URB2	2.63331	2.10759	1.94059	2.01831	3.50731	2.11662	3.80687	2.43074	2.51661	2.1707	2.34217	2.54243	3.84765	3.86371	3.68015	3.62408
PGBD5	2.76723	3.26332	3.2521	2.93698	0.73881	0.51889	-0.02264	0.69505	-3.32193	-3.32193	-3.2228	-3.14538	-1.18813	-1.69418	-1.22512	-2.13731
COG2	1.56191	1.50573	1.19261	1.64094	1.68797	1.71605	1.95431	1.81823	1.40968	1.60186	1.57601	2.34609	2.20944	2.36038	1.95613	2.16004
C1orf198	2.74778	2.59891	2.37228	2.38564	3.45889	2.364	3.01314	2.3679	3.18896	2.56084	2.57472	2.68476	4.7028	4.56801	4.91068	4.32718

TTC13	2.31167	2.56859	2.4516	2.51836	3.33983	3.51441	3.72316	3.3165	1.93612	1.81211	1.95589	2.59017	3.63218	3.10453	3.81801	3.12023
ARV1	3.60057	3.6243	3.49807	3.97101	3.76061	3.787	4.01279	3.76759	3.09503	2.85926	2.60611	3.31464	3.99559	3.66597	3.17199	2.98441
FAM89A	3.91125	3.63216	4.08742	3.80467	3.82353	3.92392	3.81567	3.66779	2.74298	2.23128	3.22794	1.78657	5.5345	4.41674	5.44484	3.984
MIR1182	5.9286	5.75446	6.19161	5.94754	6.27125	6.01834	6.4261	5.83796	5.39065	3.95709	5.46043	4.25269	7.91173	6.93691	8.07963	6.15287
TRIM67	-3.32193	-3.07483	-3.18627	-3.04678	-2.20665	-2.57937	-2.62464	-1.89823	-3.32193	-2.9203	-3.32193	-3.01546	-1.00663	-0.00502	-0.9185	-0.69022
C1orf131	1.78274	1.25413	1.56255	1.57433	2.25049	2.17218	2.31374	2.34959	1.81415	1.18314	1.76445	1.45256	2.59238	2.32908	1.9503	1.66823
GNPAT	3.90182	3.61615	3.9638	4.06553	4.53886	4.27357	4.80269	4.22648	4.63597	3.9891	4.40478	4.3514	5.4437	4.9216	5.54143	4.62701
EXOC8	1.98746	2.40397	2.18116	2.3216	2.5426	1.34849	2.68287	1.68405	2.3371	2.41257	2.03158	2.77646	3.47922	3.46483	3.34403	3.64047
SPRTN	2.01989	2.24677	2.01396	2.17402	2.52449	1.14574	2.53704	1.26162	2.33164	1.75249	1.89576	1.59222	3.01781	2.74335	2.83311	2.79661
EGLN1	3.96357	4.02356	4.15739	3.62591	4.48776	3.71321	4.68917	3.50885	5.1639	5.20423	5.03866	5.17081	4.23565	4.19419	4.52536	4.25588
TSNAX	3.24059	3.39406	3.34102	3.79532	2.83064	2.29739	3.53506	2.1959	2.7392	2.6553	2.80576	3.21653	4.07028	3.53653	3.5803	3.09122
TSNAX-DISC1	2.46238	2.4038	2.44651	2.46633	2.45768	1.39891	3.00045	0.61106	2.60537	2.98295	2.8965	3.19534	3.66267	3.49925	3.53598	3.44001
DISC1	-2.94266	-2.36218	-2.54587	-2.3314	-3.18159	-2.69225	-3.24314	-3.22226	-0.98444	0.42951	-0.16621	1.1211	0.14823	1.11161	0.82906	1.40751
SIPA1L2	0.22221	0.34967	0.34171	0.7895	1.18261	-0.52357	1.51554	-0.93555	0.55537	0.36194	-0.33193	1.49827	1.91396	2.52046	1.94235	3.74971
MAP10	0.39404	0.05864	0.43858	1.13091	-2.00001	-2.40146	-2.16082	-3.32193	0.99905	1.40833	1.29591	1.87083	1.53681	1.75447	1.47329	1.77288
NTPCR	1.37394	1.55078	1.42582	1.83969	1.08467	1.24904	0.89475	1.09228	2.1511	2.25645	2.35203	2.32748	1.52825	1.77921	1.47655	2.00968
PCNX2	0.36221	0.38336	0.51717	0.92167	-0.16465	-0.40886	-0.25255	-0.48449	0.51157	0.94535	1.09555	1.87511	1.60409	1.82663	2.47933	2.28057
MAP3K21	1.19925	0.76185	0.78758	0.14084	2.2546	0.92163	2.28608	0.91396	0.90906	0.40012	0.41031	0.05485	3.37642	3.27423	3.62573	2.94068
KCNK1	-0.27308	-0.24375	-0.66115	-0.98507	3.99297	0.10244	4.03553	0.31651	-3.32193	-3.32193	-3.32193	-3.32193	-2.73416	-2.73588	-2.82766	-3.04833
SLC35F3	3.21448	3.07778	2.82645	2.49053	0.53755	-0.48597	0.52388	-0.62379	2.78169	1.86279	2.39472	0.37571	-0.94709	-0.7538	-0.06172	-1.21131
COA6-AS1	5.55266	5.49975	5.24789	5.22561	5.97959	6.40451	5.40714	6.21785	4.91759	4.97582	4.8945	2.55044	5.9777	5.95061	5.42239	5.58238
COA6	5.82262	5.88787	5.66654	5.8871	5.76295	6.47264	5.58997	6.3456	4.90821	5.00862	4.70071	3.85789	6.10814	6.13402	5.13891	5.27959
TARBP1	1.89472	1.69047	2.06114	2.0876	3.31666	3.03339	3.53593	2.95936	2.11024	1.99479	1.8847	2.40019	2.84146	3.0395	2.47127	2.74261
IRF2BP2	5.67005	5.68889	6.11711	6.04604	5.50591	6.05265	5.52932	5.84775	5.84369	6.27272	6.06178	6.43011	5.12849	4.96176	5.53034	5.64322
LINC01348	0.63354	0.64965	1.36767	0.78404	-0.48116	-0.01438	-0.4265	0.00803	-3.32193	-1.94007	-2.52331	-2.57633	-3.32193	-3.07615	-3.32193	-3.02071
TOMM20	6.14767	6.67823	6.47634	6.72927	5.47752	5.64491	6.25109	5.63507	5.40616	6.65933	6.09118	6.84617	6.14515	7.51777	7.2081	7.35606
RBM34	4.75762	4.84293	4.71696	5.1991	4.27426	4.47295	4.54385	4.52683	4.42343	4.37682	4.15105	4.30483	5.23572	5.09421	4.42622	4.58448
ARID4B	3.86114	4.02386	3.98512	4.91995	3.49123	3.62927	3.92355	3.87751	3.64573	3.66352	3.57893	4.21299	4.28176	4.21761	4.22731	4.31333
GGPS1	2.92182	3.40564	3.41919	4.00664	3.37703	3.84981	3.784	3.72106	2.61254	3.09573	2.82083	3.40972	3.82897	4.18723	3.74486	3.88892
B3GALNT2	1.76874	1.41501	1.38715	1.50187	2.77409	1.11391	2.65832	1.4015	2.34588	1.3306	2.13772	1.53608	3.2712	2.04883	3.11287	2.39335
GNG4	-0.76469	-0.45596	-0.92793	-0.27644	1.81462	3.25238	1.66525	3.43517	3.09903	2.89611	2.97102	2.90371	3.27171	3.66008	4.16586	4.00273
LYST	0.03136	0.17682	0.47423	0.83059	0.16794	-0.75609	0.70085	-0.53887	0.51059	1.05418	1.17649	2.13836	3.02308	3.206	3.68714	3.30805
LDHAP2	-0.80398	-1.45278	-0.40843	-0.52115	-0.9023	-2.47661	-0.88551	-2.36797	-0.43224	0.21876	0.21553	0.19629	1.32622	0.86573	2.10863	0.96008
NID1	4.78607	4.61602	4.96319	4.68915	6.56868	5.73479	6.16353	5.78077	5.75916	4.91317	5.57564	5.40244	6.13966	5.1947	6.31256	5.85546
GPR137B	3.05338	3.56936	3.45043	4.00111	2.54919	3.25003	2.59216	3.05238	3.36966	3.5489	3.14972	3.70807	3.80505	4.60712	4.23093	4.69205
ERO1B	0.91127	1.27718	0.87365	1.69754	2.98118	2.89685	3.57584	2.83647	0.46352	1.28177	0.81943	1.98923	3.04523	3.47787	2.9112	3.23206
EDARADD	1.26845	1.29759	1.43953	0.09981	1.0123	2.21505	1.18571	1.66358	3.27786	3.05385	3.08086	3.26971	0.38512	0.67262	0.70423	1.09085
ENO1P1	2.38366	2.56363	2.55632	0.92848	1.69352	3.19008	2.07424	2.25467	4.16025	4.02547	3.87294	3.9119	0.75306	1.30426	1.37869	1.61456
LGALS8	4.11345	3.86057	3.95042	4.24711	3.05337	3.34504	3.47604	3.43867	3.42339	3.00006	3.374	3.93476	4.29966	3.6422	4.03029	3.55787
LGALS8-AS1	2.83419	2.80234	2.91586	2.52323	2.62893	1.63972	2.78911	1.5919	2.59551	2.30114	2.54572	2.03187	3.07833	2.11819	2.9407	2.6036
HEATR1	4.2708	4.13287	3.87191	4.19214	4.46512	3.60231	4.89464	3.70775	4.54403	4.13349	4.38359	4.78013	4.92141	4.71621	4.96806	4.94096
MTR	2.34712	2.01863	2.42949	2.30233	1.86908	0.9001	1.90678	0.93961	2.93411	1.99118	2.92467	2.66943	3.32277	2.62959	3.46787	2.70911
RPL35P1	3.48767	3.32026	3.32775	1.82475	1.76393	2.54258	0.35028	1.74132	2.0004	3.38072	2.15251	-0.15937	-0.11768	-0.75817	0.13928	-0.97313
RYR2	0.01164	-0.29435	-0.55787	-0.22588	-3.21871	-3.14251	-3.20889	-3.32193	-3.05531	-3.15034	-3.32193	-3.32193	-3.32193	-3.27358	-3.09945	-3.32193
ZP4	1.90267	2.23654	1.97383	2.18127	-3.32193	-3.32193	-3.32193	-3.32193	-0.03631	0.28604	0.78653	1.22062	-1.75333	-2.33674	-2.39703	-1.81765
LINC01139	2.69209	2.91163	2.92181	3.51687	-0.86155	1.29191	-0.00642	1.32332	2.44392	3.18717	2.77569	2.53518	2.82018	2.307	1.50996	1.63501
CHRM3	-1.34609	-1.24432	-0.95583	-0.60933	-3.20922	-3.23178	-3.32193	-3.21645	-3.32193	-3.32193	-3.32193	-3.32193	-3.25159	-3.05993	-3.32193	-2.4667
FMN2	-1.98756	-1.43569	-1.93624	-1.93367	-3.18175	-3.32193	-3.21792	-3.07035	2.07219	1.57559	1.8813	0.005	3.66945	3.23213	3.80667	2.82675
GREM2	-2.37288	-3.32193	-2.79486	-1.30242	-3.21217	-2.67619	-3.32193	-2.79311	-3.32193	-3.32193	-3.32193	-3.32193	-1.06157	1.65587	1.2038	1.30995
RGS7	-3.32193	-3.32193	-3.32193	-3.32193	-3.32193	-3.32193	-3.32193	-2.87992	-1.8616	-1.86572	-2.15268	-2.66982	2.80144	3.52205	1.29848	1.84806

FH	6.2034	6.00162	5.88253	6.18734	5.65946	5.88643	5.90033	6.01092	5.87696	5.23095	5.68295	5.40193	6.32717	6.46377	6.02002	5.82462
KMO	1.02277	1.71976	1.81457	2.22677	-0.56377	0.79616	-0.44699	0.52122	1.83156	2.60078	2.34755	3.41681	2.18532	3.09372	2.18555	2.76285
OPN3	2.35995	3.03345	3.05572	3.28151	1.97526	1.79857	2.30304	1.635	3.56682	4.25491	4.13782	4.35711	3.71959	4.38348	3.99936	4.38611
CHML	2.05744	2.20609	2.0429	2.06341	2.97013	1.49282	3.80941	1.75081	3.84046	3.86086	3.9252	4.16158	5.22109	4.7976	4.78044	4.75333
EXO1	3.98105	3.12897	3.21988	2.86681	3.16247	1.62099	3.16825	1.98459	3.81878	1.94865	2.59055	1.78288	4.49733	4.28146	4.19822	3.74613
LINC01347	-0.13884	-0.05616	-0.0562	0.06186	-1.06614	-0.76043	-1.06725	-0.12479	-2.20135	-1.21343	-1.20119	-1.23229	-1.26326	-0.7637	-0.60063	-0.17017
CEP170	2.86958	3.27382	3.08413	3.59717	2.00404	1.87673	2.67618	1.80074	3.2494	3.97574	4.06968	4.93874	4.09994	4.81352	5.08416	5.17738
SDCCAG8	2.17592	2.53102	2.44782	2.96261	0.82559	1.35748	0.95435	1.3964	2.58471	2.84832	2.93044	3.51032	2.57001	2.551	2.83135	2.57266
AKT3	1.99966	2.46101	2.67107	2.52515	-2.26642	-0.91414	-2.38268	-1.11878	3.22714	3.15624	3.34177	3.99398	4.11896	4.19818	4.74708	3.97814
ZBTB18	2.54165	2.73484	2.68535	2.79658	0.7684	1.52227	1.22912	1.6439	2.86907	3.89646	3.37167	3.79777	5.17602	6.67123	5.24591	5.04463
DESI2	3.52985	3.51726	3.32312	3.44725	3.83939	3.34847	4.07702	3.22375	3.74895	3.69432	3.02951	4.09678	5.20923	5.28099	4.71009	5.02407
COX20	4.06957	4.22421	4.24392	4.33507	4.13453	4.72758	4.47076	4.65695	4.27578	3.98416	3.74308	3.82762	5.25085	4.54878	4.66267	4.18538
EFCAB2	0.58274	0.57201	0.65467	0.76983	0.35994	1.10525	0.65352	0.8608	-1.84589	-1.82315	-1.02228	-1.0449	0.29071	-0.43329	-0.13721	-0.57351
KIF26B	-2.34377	-2.17812	-2.0562	-2.12501	-3.25736	-3.0375	-3.32193	-3.32193	0.14851	-1.02998	-0.52015	-1.37307	-1.22	-0.02419	0.7031	0.85101
SMYD3	2.24209	2.20618	2.31011	2.35355	1.67381	2.04962	1.75395	2.05662	4.0928	3.69179	4.14335	3.71914	2.51814	2.62117	2.50996	2.29369
TFB2M	4.747	4.61576	4.38529	4.266	5.10956	3.33325	5.22728	3.43827	3.89167	3.58087	4.06348	3.22907	4.49648	3.5206	4.03735	3.17545
CNST	2.18775	2.03601	1.87622	2.14884	2.34845	1.23428	3.0005	1.02355	2.19972	1.9414	2.27853	2.6178	2.49156	1.6216	2.53126	2.03671
SCCPDH	4.99751	4.95119	4.77388	5.25354	5.9684	5.78699	6.18026	5.65762	4.66068	4.67161	4.32305	4.76409	5.58509	5.44778	5.38995	5.22066
KIF28P	0.50697	0.70681	0.7356	0.79901	0.17454	1.50495	0.09214	1.5371	-1.20856	-1.18539	-1.51088	0.16705	0.94742	1.03014	0.81614	0.96186
LINC01341	-1.86077	-2.25617	-1.40754	-2.01417	-0.82796	0.54505	-1.22662	1.06536	-3.32193	-2.23302	-2.63897	-1.8126	-2.38405	-2.63639	-2.37335	-1.98801
AHCTF1	3.84114	3.65346	3.56107	3.97742	3.91492	3.02428	4.48359	3.17082	3.86468	3.31734	3.55938	3.99409	5.12343	4.68648	5.16051	4.78126
ZNF695	-2.82289	-2.55998	-2.74699	-2.48797	1.71618	1.65548	1.84445	1.48723	-3.32193	-3.32193	-3.32193	-3.32193	1.08647	1.18754	0.33797	0.09479
ZNF670-ZNF695	-1.10734	-1.13852	-1.59908	-1.41188	1.82104	1.7292	1.78818	1.26902	-1.38768	-1.48395	-2.14724	-2.07046	1.00136	1.18864	0.48333	0.53642
ZNF670	1.93983	1.51212	1.21002	1.88242	1.73986	0.93378	2.23701	1.24395	1.18373	0.67927	0.34406	0.31482	2.55918	2.72959	2.39656	2.23143
ZNF669	1.46753	1.80227	1.57493	1.81327	1.2912	1.02996	2.00541	1.16648	2.10155	2.03796	1.63454	2.04027	3.22867	3.89526	3.70585	3.84862
ZNF124	0.38157	-0.13608	-0.00626	0.04643	0.83648	0.80767	1.04034	1.14077	0.37664	0.15146	-0.20858	0.34799	1.65794	2.27746	1.26103	1.99641
MIR3916	4.81622	4.75355	4.83123	3.77084	4.70315	5.69649	4.58447	4.88565	3.95004	4.58409	4.36653	3.65805	5.69621	6.60623	5.33283	6.24486
ZNF496	2.20026	2.06854	2.01668	1.73483	1.95761	1.94693	1.65738	2.07422	3.26948	3.5613	3.50313	3.46571	3.25836	3.38543	4.01346	3.89874
NLRP3	2.35759	2.18584	2.19672	1.82359	-3.32193	-3.32193	-3.32193	-2.66002	3.45004	2.47081	3.33711	2.99225	-3.32193	-3.32193	-3.11307	-3.10878
TRIM58	-1.89722	-1.43395	-2.43025	-1.98906	0.01386	-1.76289	0.5339	-3.32193	-3.32193	-3.32193	-3.32193	-3.32193	-3.32193	-3.32193	-3.02354	-3.32193
LYPD8	-0.33298	0.15378	-0.18284	0.49974	-3.32193	-2.88813	-2.91718	-1.70684	-2.83313	-3.32193	-3.32193	-2.59265	-0.30989	0.10437	-0.271	-0.428
SH3BP5L	4.44294	4.22967	3.80611	3.34387	3.3228	3.07378	3.1782	3.00181	4.02454	3.85162	4.09372	3.30901	3.95367	4.00542	4.10182	4.14666
ZNF672	3.56337	3.49831	3.41865	2.97312	4.29594	4.45523	3.93929	4.17899	3.61232	3.65576	3.56755	3.58482	3.822	4.03068	4.22644	4.34521
ZNF692	3.04584	2.53558	2.94595	2.45586	3.40306	4.60682	2.00326	4.5987	1.3927	2.24726	2.25268	1.34166	2.51151	2.66918	1.71691	1.4239
PGBD2	0.45992	0.65929	0.49528	0.8831	0.90268	1.27576	0.43307	1.15307	0.27728	0.29565	0.18272	0.47482	1.46708	1.39998	0.89975	1.28933
RPL23AP25	2.15651	2.01831	2.09439	1.51146	0.6736	0.84386	-3.32193	1.9591	2.06755	1.87033	1.43552	2.08123	0.95498	0.49423	0.89567	1.04474
FAM110C	-3.0642	-3.32193	-3.02125	-3.32193	0.73268	0.0963	1.39934	-0.16653	-3.02638	-3.32193	-3.32193	-3.32193	-3.11624	-2.93746	-3.32193	-3.32193
SH3YL1	-1.79743	-1.88018	-1.78879	-1.60752	-0.92926	-0.9194	-1.15075	-0.50724	-0.94298	-0.38908	-0.36418	-0.04415	-1.03902	0.03974	-0.57612	-0.53497
ACP1	4.70949	4.5719	4.58191	4.74207	5.31947	5.51508	5.63005	5.35901	4.88611	4.90171	4.66232	4.68128	5.08539	4.88928	4.43769	4.30074
TMEM18	3.01815	2.86483	2.92799	3.00105	1.52801	1.52066	1.58559	1.96595	2.47842	1.97384	2.07468	1.89434	2.76091	2.10167	1.98148	1.55481
PXDN	-3.22574	-2.49504	-2.91406	-1.77852	-2.68376	-2.61938	-2.39517	-3.00461	6.35717	4.90551	6.42831	5.50258	-2.97702	-3.32193	-3.32193	-3.32193
EIPR1	3.12344	3.12818	2.93789	3.01932	3.07023	3.47684	2.93347	3.36434	3.24141	3.32581	3.4449	2.78464	2.83028	2.98285	2.8701	2.78799
TRAPPC12	3.5094	3.64977	3.7848	3.03374	3.12369	3.4905	2.30413	3.16135	3.40066	3.06406	3.63189	2.54757	2.5657	2.36964	2.84826	2.94568
ADI1	4.0187	3.93228	3.95233	4.37278	4.12684	4.03075	4.24445	4.10443	3.68223	2.59363	3.48617	2.85716	4.01771	3.35992	3.3751	3.28458
RNASEH1	4.68976	4.4629	4.52651	4.21201	4.6243	4.44577	4.67888	4.35952	4.18131	3.4857	3.97556	3.52385	4.03368	3.73291	3.86373	3.59401
RNASEH1-AS1	4.73724	4.37639	4.21688	3.97321	4.27363	4.22148	3.91542	4.27996	3.00452	3.03753	2.70112	2.13386	3.05951	3.50359	1.8102	2.75565
COLEC11	-3.32193	-2.61957	-2.10534	-2.8861	-0.9237	0.12256	-0.95105	0.21829	-3.32193	-3.32193	-2.9552	-2.70868	-1.01749	0.81354	-0.54198	-0.16707
DCDC2C	4.61706	4.70778	4.41022	4.57733	-2.95004	-2.88896	-3.32193	-3.32193	-3.32193	-3.32193	-3.32193	-3.32193	-2.69675	-3.32193	-3.32193	-3.32193
CMPK2	2.58915	2.66375	2.8037	3.47843	-2.21805	-2.87322	-1.89753	-2.42793	-3.04728	-2.71605	-2.86377	-2.72444	-3.32193	-3.32193	-3.09265	-3.32193
RSAD2	-1.19375	-0.64245	-0.21616	0.30455	-3.32193	-3.32193	-3.32193	-3.32193	-3.07507	-2.62657	-2.44881	-1.90279	-3.32193	-3.32193	-3.32193	-3.32193

RNF144A-AS1	-1.44549	-1.3224	-1.74747	-0.77391	-2.18076	-2.08888	-2.62285	-1.06687	-2.3434	-2.75336	-2.89329	-2.61424	-2.98664	-3.14516	-3.32193	-3.10414
RNF144A	-0.72837	-0.91417	-0.49246	-0.47275	1.09552	-0.38316	1.08037	-0.15421	0.79128	0.57284	0.6353	0.38389	-1.7502	-1.70878	-0.84191	-1.0406
ID2-AS1	-2.86065	-1.19001	-2.096	-1.56572	0.44793	0.6993	0.68906	0.95404	-3.32193	-0.80516	-1.42195	-0.91976	-1.45148	-1.70897	-2.54001	-2.24948
ID2	1.69272	2.44186	2.76844	4.11977	5.28805	5.91699	5.18477	6.13217	2.37106	3.4809	2.30731	3.43888	1.73392	0.91533	0.91157	0.62541
KIDINS220	2.05727	2.08198	1.99226	2.64641	2.99038	2.13785	3.39731	2.09436	2.51601	2.4174	2.21014	3.19637	2.83059	2.97701	3.57563	3.83487
MBOAT2	1.22693	1.65971	2.01811	2.42724	1.49186	1.27972	2.05357	1.06596	2.29242	1.9422	1.86983	2.74018	2.84891	2.60182	3.05663	2.88251
ASAP2	3.35445	3.31197	3.29173	3.40468	2.8203	0.94668	3.10023	1.14509	5.02878	4.90527	4.80721	5.15854	3.41826	3.07445	2.89561	2.93858
ITGB1BP1	4.47056	4.62352	4.42606	4.75217	3.793	3.69939	4.09314	3.59843	4.5929	4.32301	4.06097	4.50864	4.30435	4.16485	3.47633	3.55571
CPSF3	4.58542	4.715	4.42432	5.0231	3.95511	3.96834	4.21804	4.15248	4.47356	4.05494	3.94641	4.48751	3.63145	4.19606	3.70232	3.94227
IAH1	3.83006	3.91039	4.21035	4.2471	3.6017	4.02365	3.75125	4.01458	3.75234	4.13182	3.94196	4.24259	3.38591	3.56834	3.82758	3.5534
ADAM17	4.24883	4.2926	4.49755	4.68098	4.00395	3.58891	4.49373	3.71469	4.86933	4.82481	4.80778	5.54875	4.46398	3.96269	4.98295	4.72041
TAF1B	2.06587	2.25485	2.11467	2.53347	2.49145	1.29613	3.01228	1.80095	3.31234	3.22129	3.15442	3.64193	3.4246	3.28475	2.91092	2.97321
GRHL1	-0.81481	-1.08173	-1.27858	-0.66702	0.17376	-0.63768	0.19875	-0.76718	-2.39019	-2.96314	-2.46684	-2.30916	-0.58678	-0.29302	-0.89441	-0.94598
KLF11	2.09674	2.26536	2.57792	2.81972	1.19584	0.66693	1.50139	0.29455	2.50666	2.76615	2.43528	3.43858	1.92475	2.65655	3.15124	2.60279
CYS1	-3.32193	-2.9368	-3.32193	-3.32193	-2.13697	0.78761	-1.98462	0.54778	2.79585	3.79712	3.24426	2.64997	-1.40491	-0.52312	0.31639	0.15175
RRM2	5.84558	5.32718	5.05072	4.68182	5.62456	4.61198	5.75986	4.71341	6.91731	5.38209	5.78623	4.91473	6.15972	5.69892	5.36193	5.11169
NOL10	4.55841	4.38375	4.12405	4.22902	4.39194	4.03686	4.76106	4.15143	4.29352	3.68488	4.07169	4.03534	4.04798	3.29459	4.06569	3.6437
RN7SL832P	-0.15912	0.06013	-0.17553	0.34677	-0.55235	1.10811	-0.47624	0.99459	-1.9764	-1.49118	-2.07485	-2.14753	-1.05623	-0.86634	-2.14181	-1.47879
ATP6V1C2	6.63294	6.59393	6.56109	6.84971	7.01217	7.85978	7.07039	7.87669	6.69869	6.47758	6.2731	6.38751	5.70427	5.61366	5.31643	5.40354
KCNF1	-1.42953	-0.84507	-1.24688	-0.20014	4.023	5.17924	4.48015	4.83246	-0.80355	-0.47148	-0.75694	-1.14058	-1.16708	0.17923	0.69733	1.90292
ROCK2	2.56978	2.76685	2.88676	3.2504	3.92926	2.58539	4.86542	2.56425	4.13241	3.86897	4.20344	5.06129	3.90686	3.52065	4.17012	3.70464
E2F6	2.85923	2.70141	2.44843	2.91994	3.15672	3.04643	3.64568	2.82598	3.03107	2.68901	2.43773	2.87862	3.32583	3.45222	2.86727	2.7743
GREB1	-0.17649	-0.04868	-0.0884	-0.13846	-3.01159	-3.32193	-3.01808	-3.03028	-1.7162	-1.62584	-1.23372	-0.88772	-3.00468	-3.0057	-3.08334	-2.74644
LPIN1	1.44222	1.36049	1.51199	1.80872	0.56675	0.66774	0.46107	0.78795	1.88361	2.15094	2.11883	2.52539	2.07778	2.72637	2.5261	2.8443
TRIB2	-0.18746	-0.0963	0.19174	0.96931	-2.37036	-3.09414	-2.36036	-3.05745	1.33258	2.60564	1.69458	3.7573	-3.32193	-2.59051	-2.9181	-2.1074
NBAS	2.50738	2.52513	2.54447	2.65729	3.01812	2.20951	3.52149	2.16496	3.59664	3.40784	3.56935	4.39414	2.75542	2.4316	3.36143	3.48501
DDX1	5.89159	5.82676	5.68268	5.87246	5.92651	5.51324	6.3448	5.46116	6.11126	6.00586	6.17466	6.1275	5.26041	4.92531	5.05967	4.94886
FAM49A	3.56718	3.15548	3.11666	2.93269	-3.15713	-3.32193	-3.32193	-3.32193	-3.10087	-3.13622	-3.12333	-3.13917	-3.32193	-3.03219	-3.32193	-3.32193
VSNL1	-2.83139	-2.38905	-2.03545	-1.59922	2.49742	-0.91614	2.9621	-0.66694	-3.32193	-3.32193	-3.32193	-3.32193	-3.10951	-2.92562	-3.32193	-2.84146
SMC6	3.00857	2.82165	3.13976	3.34526	2.73324	1.94943	3.18536	2.09495	2.83473	1.96402	2.36676	2.51149	3.09669	1.8293	2.86558	1.83876
GEN1	1.11141	0.87835	1.00342	1.31854	1.43312	0.88879	1.83967	1.45732	0.65972	0.32388	0.21475	0.68859	1.44191	1.54727	0.89087	1.00141
KCNS3	-0.24528	-0.09746	-0.21165	-0.46225	-2.28436	-2.32012	-3.02165	-2.6532	1.10146	0.57181	-0.2435	-0.23112	-0.51043	-0.72949	-0.26246	-0.11644
RDH14	4.36053	4.46671	4.47836	4.69179	4.83463	4.61918	4.75423	4.75348	4.38579	4.45513	4.34801	4.10107	4.16316	4.28777	3.7247	3.6882
NT5C1B-RDH14	2.92076	2.65516	2.76859	2.70609	3.24063	2.86595	3.11416	2.64151	2.92006	2.95571	2.73211	2.78153	2.08992	2.22192	1.72477	1.79984
OSR1	-2.20455	-1.1695	-1.62081	-1.33905	-2.74014	-1.63616	-3.32193	-1.86839	1.30973	1.4372	2.293	1.93659	-0.45421	-0.50463	-0.54327	-0.92143
TTC32	1.79113	2.20724	2.37652	1.98114	2.63793	3.02866	3.02242	3.11314	0.59279	0.78277	-0.13657	-0.38298	1.40116	1.97962	0.37179	0.70084
WDR35	1.44085	1.25706	1.19805	1.44665	1.49221	0.93552	1.99721	0.82606	1.16437	1.41124	1.55183	1.9329	2.36931	2.19676	1.73319	2.01561
MATN3	-1.00501	-0.04977	0.10186	0.33646	0.45269	-0.79042	1.24915	-0.53089	-0.3856	-0.20408	-0.37625	-0.44002	1.2281	0.79263	1.06784	0.22502
LAPTM4A	6.92628	7.09935	7.11419	7.46286	7.45364	7.36691	7.77456	7.21046	7.29653	7.63027	7.35901	7.51585	6.95187	7.38325	6.96487	7.27579
PUM2	3.7324	3.86604	3.80072	4.23393	3.98803	3.16916	4.4135	3.39387	4.38643	4.25346	4.01862	4.97583	4.4695	4.38985	4.26889	4.29072
RHOB	4.64575	5.13487	5.38579	4.71859	6.01207	7.10285	6.4504	6.60403	5.31491	6.40036	6.7301	6.51049	5.65223	7.36411	7.06298	6.75223
HS1BP3	2.90072	2.96216	3.26462	2.75521	3.64474	3.5115	3.23767	3.1512	2.6923	2.98014	3.04861	2.6472	2.70412	2.51381	3.36344	2.97275
HS1BP3-IT1	-0.42127	-0.2759	-0.48804	-1.92524	1.45258	2.61262	1.6957	2.1401	0.20475	-0.03927	-0.37705	0.40737	-3.32193	-2.31489	-2.16165	-2.14413
GDF7	-3.32193	-3.32193	-3.1919	-3.32193	2.3564	2.37939	1.95973	2.02077	-1.47754	-1.07047	-0.98819	-1.11323	0.35476	1.882	1.68882	1.92228
LDAH	2.03273	1.97523	1.93868	2.24531	3.01408	2.44047	3.32244	2.27735	2.00262	1.75499	1.8262	2.56293	3.0173	2.31591	2.61691	2.06467
TDRD15	-0.69273	-0.279	-0.48501	0.80318	-2.51277	-2.0547	-2.36238	-2.08668	-3.32193	-3.32193	-3.32193	-3.32193	-3.32193	-3.32193	-3.15742	-3.32193
KLHL29	2.24089	2.3606	2.4282	1.32096	2.20963	-0.59956	1.38199	-0.65825	1.7092	1.49098	1.28597	0.32161	1.50897	1.7231	1.72802	1.97492
ATAD2B	0.20215	-0.29684	-0.00883	0.16076	1.05169	0.61918	1.73018	0.69788	0.48371	-0.26064	0.34761	0.67236	1.5528	0.95761	1.57605	1.05013
UBXN2A	2.12156	2.29573	2.32676	2.3253	2.25829	1.83905	2.78448	1.94704	2.47671	2.18173	2.24074	2.7685	3.13648	2.90425	2.87279	2.78917
MFSD2B	0.28598	0.94521	0.84713	1.17858	0.67835	1.53292	0.52464	0.96439	0.96332	2.22344	1.61876	1.01658	1.47785	2.22024	1.83035	1.68844

WDCP	1.90752	1.82453	1.46104	1.53682	2.09806	1.71462	2.20189	1.85894	2.72153	2.50676	2.56828	2.91087	3.04537	3.11824	2.1918	2.5912
FKBP1B	1.63609	2.28658	2.25111	2.7571	2.03904	3.11488	1.93176	2.67249	2.2242	3.68021	3.0276	2.67218	3.04577	3.78959	3.24298	3.19103
SF3B6	6.40785	6.49266	6.46785	6.68863	5.90806	6.05548	6.19639	5.9185	6.26487	6.6204	6.39912	6.00748	6.45907	6.32232	5.16764	5.14853
FAM228B	0.77429	1.17751	1.38202	1.70882	1.5071	1.62526	1.32314	1.42834	2.14757	2.11004	2.60935	2.20319	1.5337	1.53488	1.34589	1.36763
TP53I3	4.13113	4.26608	4.25025	4.16519	4.49718	4.76833	4.08124	4.53718	5.03727	5.09535	5.55069	4.84185	4.2996	4.15076	4.13219	4.43009
PFN4	-1.34378	-0.56182	0.19519	0.66404	0.58942	1.17312	0.08469	0.49698	-0.5187	0.73048	0.47958	-0.32782	-0.56535	-0.14683	-1.39334	-0.91503
ITSN2	3.00964	3.23549	3.20906	3.79339	3.32717	2.68662	4.1161	2.63867	3.04428	2.80559	2.94437	3.52148	2.49652	2.1986	2.6399	2.2168
NCOA1	1.16906	1.20148	1.25053	1.84165	2.31603	1.7692	2.61676	1.48266	2.18638	1.70675	2.01418	2.97997	1.76092	1.5535	2.61281	2.38503
PTRHD1	4.01913	4.33524	4.23682	4.37493	5.1492	5.52702	4.88458	5.32959	4.05057	4.57275	4.45624	3.93172	3.34856	3.88099	3.28168	3.27809
CENPO	4.23534	3.94315	3.86267	3.76496	5.2723	4.6578	5.04216	4.60233	4.67647	4.0275	4.24558	3.3942	4.5596	4.6635	4.69245	4.48128
ADCY3	4.07312	3.64165	3.57576	2.99573	4.97131	3.71634	4.46859	3.71464	4.73373	4.00442	4.47234	3.40345	4.40719	4.30613	4.8606	4.5487
DNAJC27	-0.36548	-0.53827	-0.97502	-0.64372	-0.15667	0.04942	-0.09341	-0.11919	0.56511	0.40521	0.22923	1.10698	1.30317	0.66599	0.10434	0.168
EFR3B	1.58941	1.38785	1.22108	0.46115	0.21186	-0.67563	0.20591	-0.80927	0.00417	-1.58557	-0.54679	-1.404	-0.50879	-1.46137	0.31084	0.38798
DNMT3A	1.30433	1.07056	1.16964	1.42872	2.06935	2.03859	1.86073	2.14027	2.20497	2.05644	2.52913	2.42006	0.90731	1.03964	2.51813	2.44948
DTNB	0.95271	0.53916	0.81546	0.48803	1.41644	1.79544	0.98963	1.47869	1.53734	1.14085	1.2397	1.18426	0.71377	1.44352	1.46725	2.12105
ASXL2	1.8897	2.03094	1.89335	2.34283	2.71688	1.19744	3.05404	1.00997	2.44934	2.01519	2.2115	2.93508	2.93539	2.71925	2.98513	3.01422
KIF3C	3.98269	4.16479	4.08764	4.05271	2.62513	2.00782	2.44971	1.92249	4.85432	4.4574	4.39453	4.17106	5.17163	5.24923	5.61149	5.84085
RAB10	5.90568	6.10356	6.05798	6.24238	7.29758	6.8775	7.836	6.79438	6.46597	6.77406	6.52866	6.65837	6.25347	6.56518	6.70997	6.68149
GAREM2	-1.06197	-1.09945	-1.80699	-1.91608	1.22447	1.5691	0.40036	1.30019	1.38202	1.50312	1.73205	1.04948	0.66404	0.79264	1.33372	1.46745
HADHA	6.08612	5.58391	6.01113	5.65645	6.66145	5.54843	6.85304	5.23625	6.54995	5.50541	6.41038	6.07636	6.04005	4.0643	6.46207	4.64052
HADHB	4.45622	4.50024	4.52902	4.79221	4.84469	4.88037	5.10407	4.8288	5.1199	4.91367	5.23537	5.33426	4.82705	4.38801	4.58761	4.28894
ADGRF3	-0.50177	-0.47401	-0.78882	-1.12927	2.17722	-0.82849	2.42467	-1.04523	0.11973	0.0319	0.4688	-0.08481	-0.40666	-0.07119	0.9069	1.12247
SELENOI	2.91317	2.91768	2.40704	2.85092	4.38103	3.10226	5.17167	3.09077	2.45968	2.65241	2.80167	3.14421	3.30081	3.56116	3.66658	3.55627
KCNK3	-2.23619	-0.66214	-1.23425	-1.2692	-3.17666	-3.32193	-3.01995	-2.79084	-2.66212	-1.60602	-2.39259	-1.38173	-3.32193	-3.06526	-3.32193	-3.00762
SLC35F6	4.45154	4.59226	4.33632	4.28798	5.43237	5.42207	5.29008	5.22163	4.56994	4.41628	4.61329	4.79011	4.41543	4.5631	4.88435	4.71614
CENPA	4.43472	4.08108	4.11679	3.48124	4.61579	3.72229	4.62141	3.62019	4.78782	3.90037	4.12543	3.09754	5.09448	4.7077	4.52864	3.89911
DPYSL5	-3.32193	-3.32193	-3.32193	-3.13491	-3.25627	-3.32193	-3.32193	-3.32193	-1.01434	-0.96489	-1.42436	-1.60534	-3.09322	-1.17675	-0.39696	-0.89255
MAPRE3	1.76484	2.2583	2.10435	2.28895	0.72534	2.12598	0.97383	1.83568	1.77072	1.82384	2.0267	2.17091	-0.25448	-0.45291	0.06017	0.35884
TMEM214	4.62619	4.60182	4.52609	4.59248	6.41875	6.11016	5.83649	5.96623	5.10831	4.90204	4.99919	4.93307	3.70907	3.29079	3.81505	3.84174
AGBL5	4.6857	4.7016	4.89771	4.62162	4.84928	5.10171	4.52362	4.87274	5.0251	5.09885	4.92242	4.04491	4.41123	4.36655	4.28423	4.0344
OST4	7.90141	7.96256	8.02852	7.6731	7.5191	8.36119	7.1728	8.16581	7.79967	8.05669	7.66178	6.19508	7.34326	7.25699	6.23523	6.31717
EMILIN1	-0.76067	-0.67066	-0.28601	-0.20526	0.41418	1.76176	-0.32999	1.58114	-1.25465	-0.50414	-0.5804	-1.1418	-2.37952	-1.35534	-1.623	-1.44262
KHK	2.75387	2.52736	2.20392	1.77819	4.51415	4.25374	3.65541	4.47528	1.53642	2.06504	1.6178	0.69135	1.53691	1.53194	2.25509	2.0477
CGREF1	1.72641	1.07437	1.23497	1.07957	3.80885	3.79843	2.95253	3.92018	0.40975	0.6279	0.47913	-0.47522	1.02997	1.07728	1.31325	0.89738
ABHD1	0.71562	0.25248	0.22947	-0.93449	0.76709	2.13355	0.43485	2.50149	-0.67558	-0.77366	-0.77047	-1.05473	0.88489	0.80637	-0.4656	-0.28672
PREB	4.97602	4.68131	4.62352	4.51239	6.17199	6.09258	5.68191	5.99507	4.91554	4.52856	4.74732	4.16695	4.74882	4.51713	4.46552	4.41821
SLC5A6	4.18215	3.86	3.70136	3.81218	6.16588	5.65317	5.84299	5.65363	4.73792	4.30875	4.6153	3.89767	4.50207	4.38424	4.55758	4.67697
ATRAID	4.99854	4.75613	5.20368	4.98823	6.81363	7.23486	6.46139	6.94252	5.35856	5.36228	5.50138	4.99939	4.90422	4.60566	4.47925	4.25411
CAD	4.99351	4.62624	4.53617	4.142	5.45472	4.36967	4.93113	4.62366	5.51646	4.65609	5.34227	4.42357	4.70546	4.35566	5.17581	4.97785
SLC30A3	2.689	2.73181	2.81494	2.67664	0.07575	0.71002	-1.18663	0.71739	0.38655	-0.59211	-0.44984	-1.20986	-2.30963	-2.63657	-2.00726	-2.73046
TRIM54	0.15169	0.1891	0.7051	-0.12769	-1.78186	-0.85139	-3.32193	-0.51217	-2.59704	-2.69755	-3.32193	-3.32193	-3.32193	-3.32193	-3.32193	-2.45636
UCN	2.10363	2.95796	3.01444	2.01569	1.77271	3.72552	0.63973	3.92554	0.54765	2.26513	1.60068	1.27125	-1.3007	-1.9861	-0.60111	-0.795
MPV17	4.19371	4.08185	4.12192	3.83224	3.65325	4.40742	2.97331	4.29493	3.49344	3.67278	3.46396	3.3795	3.13067	3.17287	2.84015	3.11943
GTF3C2	3.98409	3.62627	3.47753	3.4951	4.74971	3.9938	4.41558	4.01882	4.34841	3.87891	4.24627	3.91599	4.26523	3.78407	4.18158	4.11916
GTF3C2-AS1	2.87834	2.46189	2.53143	2.24438	4.10129	2.57576	3.72193	2.56497	3.64343	3.31091	3.77284	3.1572	3.50728	3.02938	3.43074	3.16897
EIF2B4	3.51165	3.46733	3.26865	3.49933	4.28474	4.31933	4.23513	4.29884	3.78465	3.37646	3.84507	3.44992	3.35341	3.26394	3.48709	3.28224
SNX17	5.50755	5.472	5.33702	5.34128	6.12963	6.10032	5.80879	6.01803	6.2385	6.09175	6.28585	5.63113	5.34295	5.47932	5.60798	5.56602
ZNF513	3.46311	3.5533	3.77691	3.36915	4.27824	5.44624	3.14746	5.31315	3.45064	3.79345	4.06679	2.64613	2.5251	2.87773	2.44001	2.68008
PPM1G	6.96881	6.78236	6.47998	6.34591	6.27213	6.27786	6.13468	6.38879	6.80573	6.04594	6.40673	5.76746	6.0857	6.24702	6.30343	6.16785
FTH1P3	2.57228	2.09647	2.995	0.83001	-0.16781	0.77604	0.42279	0.06821	-0.19699	1.01599	-0.61995	0.14781	-0.10017	-2.29517	-1.49913	0.18589

NRBP1	5.05519	5.26121	5.21711	5.42297	5.73994	5.85688	5.7422	5.77532	5.52602	5.33752	5.69501	5.52725	5.1734	5.30358	5.42524	5.47343
KRTCAP3	1.62631	1.46982	1.4444	1.72787	-0.49239	1.74158	0.1125	1.99349	1.34676	0.97602	1.03451	1.72201	0.29489	1.03088	-0.34629	0.53564
IFT172	1.78808	1.71518	1.83575	1.82869	0.85654	0.8629	0.69398	0.79681	2.31787	2.06969	2.29687	2.71637	1.48151	1.28916	1.46202	1.87583
FNDC4	1.5262	2.44946	2.21612	2.41683	4.21801	4.63718	3.68034	4.1347	4.00276	4.71937	4.5197	3.74704	2.49109	2.8028	2.67377	3.05587
GCKR	-1.63473	-1.20997	-1.58287	-0.29283	2.93627	3.84974	2.07794	3.69839	-2.42642	-1.78431	-1.8422	-2.36923	-3.32193	-2.85187	-3.01679	-3.32193
C2orf16	-2.76299	-2.92285	-2.73261	-2.39895	-0.91576	-1.33416	-0.60706	-1.26821	-1.94617	-1.16236	-1.29046	-0.54972	-2.52167	-2.27145	-2.42789	-1.38958
ZNF512	2.56647	2.57122	2.39042	2.82685	2.99721	2.30835	3.03352	2.44132	3.84359	3.90592	3.5807	4.53522	3.06718	2.49164	3.17199	2.85734
CCDC121	-1.05272	-0.688	-0.55225	-0.1643	1.10531	-0.0659	1.07888	0.18736	0.51829	-0.80169	-0.36125	-0.42128	0.92287	0.11097	-0.05918	0.18728
GPN1	3.9303	3.88496	3.75773	3.84344	4.0111	3.1569	4.04101	3.11699	4.21874	3.8202	4.11918	3.90574	3.91473	3.6162	3.85805	3.32004
SUPT7L	3.48112	3.43492	3.21132	3.73485	3.29706	3.59365	3.34441	3.74622	2.85536	2.64625	3.07801	3.31922	3.08271	2.82461	2.53124	2.35259
SLC4A1AP	3.82028	3.55795	3.31265	3.82333	3.62042	3.31338	3.65308	3.31315	3.77066	3.51788	3.68763	3.43815	3.39933	3.51515	3.19166	3.21611
MRPL33	5.96654	5.99372	5.56738	5.58993	5.14292	5.391	5.16873	5.41979	5.86561	6.07787	5.39585	5.5294	6.3485	6.42693	4.71129	4.88648
RBKS	1.19147	1.64519	1.43968	1.34059	1.20765	1.4773	0.8787	0.70304	1.98455	2.54585	1.8586	2.37134	1.92862	1.87184	1.1936	1.21083
BABAM2	4.14157	4.1154	3.96148	3.87646	2.84677	3.55294	2.9415	3.39178	4.74571	4.89501	4.5973	5.07743	3.65262	3.4913	3.08554	3.07529
PLB1	-2.2307	-1.54283	-2.48899	-1.78441	-2.8782	-2.9593	-2.62987	-3.03019	-2.59331	-1.92397	-2.41244	-1.51426	-2.86876	-2.40924	-2.69928	-2.31378
PPP1CB	5.35404	5.64191	5.29374	5.90343	5.22298	4.62588	5.5916	4.65061	5.81975	6.1907	4.99031	6.45914	5.24659	5.60104	5.40858	5.78646
SPDYA	4.08288	4.31712	3.93354	4.46767	4.22102	2.79682	4.58241	2.79451	4.79908	5.05084	3.93226	5.29825	4.175	4.45603	4.48654	4.82955
TRMT61B	3.54708	3.42422	3.19827	3.51022	3.33916	2.94397	3.64016	2.98412	2.58006	3.14938	3.06874	3.08303	2.92822	3.40071	2.61326	2.66958
WDR43	5.12529	4.81306	4.58697	4.85301	4.89327	4.13745	5.33352	4.26384	5.02306	4.86176	4.99153	5.06238	4.72134	4.33613	4.77083	4.16834
CLIP4	3.02604	3.05336	3.03579	3.14083	-0.24101	-1.24137	0.05811	-0.74018	2.9163	3.61672	2.6822	4.25851	2.59725	2.40109	2.02653	2.05394
YPEL5	4.3904	4.81434	5.08373	5.63198	3.5791	3.9851	4.13512	3.72697	4.31881	5.14606	4.63368	5.59837	3.9617	3.84188	3.99793	4.05642
LBH	-2.18575	-1.29569	-0.92746	-0.50444	-1.02182	-2.89317	-0.4055	-2.63427	-1.25745	-1.01138	-1.38208	-1.29245	-2.18005	0.1047	-0.23171	0.6958
LCLAT1	1.37775	0.95799	0.6261	1.29818	3.02152	2.20795	3.51646	2.10843	2.03858	1.51495	1.74022	2.26837	2.85808	2.96148	2.40463	2.26766
GALNT14	2.42443	1.54745	2.39345	1.79418	-2.47881	-3.32193	-2.76034	-3.05634	4.64094	4.19814	4.7129	3.93319	3.81823	3.25883	4.17482	3.45013
EHD3	-1.09447	-0.81157	-0.36895	-0.32658	0.99883	1.43653	0.86311	0.96483	3.58137	3.77364	4.00186	3.71127	1.45218	-0.28845	1.20523	0.86028
XDH	-0.86259	-1.07348	-0.96795	-0.85093	-2.85496	-2.71792	-3.15842	-2.77721	2.11402	1.73259	2.09375	2.65892	0.53568	-0.88475	-0.07867	0.23749
MEMO1	3.84832	3.84984	3.65407	3.97764	3.58305	3.68177	3.53499	3.75351	3.7961	3.91069	3.59493	3.79837	3.70112	3.71399	3.1836	3.16667
DPY30	5.36296	5.29035	5.15543	5.45834	5.35838	5.70795	5.45303	5.63234	5.51462	5.42033	5.20966	5.26714	5.41452	5.35674	4.76281	4.76819
SPAST	1.82947	1.72615	1.82354	2.13265	3.25117	2.04235	4.01977	2.06104	2.68954	2.40911	2.75433	3.37307	3.13003	2.20459	3.46905	2.96353
SLC30A6	2.8654	2.80231	2.60066	3.17737	4.08449	3.1451	4.71073	2.89807	2.85539	2.39507	2.43445	3.42427	3.52029	2.25737	2.92452	2.38126
NLRC4	-1.57528	-2.18319	-1.98717	-0.98411	-1.78088	-0.69041	-1.47441	-1.7189	-2.32501	-2.45319	-2.10089	-1.54108	-1.99423	-2.3197	-2.82732	-2.61973
YIPF4	2.6227	2.86073	2.81664	3.3207	3.17511	2.5745	3.38407	2.72022	2.49714	2.44873	2.27464	2.34142	2.42323	2.0205	2.54207	2.40619
BIRC6	2.35326	2.46248	2.28021	2.82195	3.34904	2.34766	3.8491	2.40551	2.9524	3.01607	3.02722	3.90324	3.2105	3.2038	3.31477	3.68728
BIRC6-AS1	0.52228	0.7864	0.50509	0.44904	2.41937	-0.59309	2.94254	-1.00773	1.42686	1.90719	2.12699	2.27805	2.41526	1.87339	2.37935	2.60325
TTC27	3.89641	3.50001	3.40714	3.63641	4.14178	3.2271	4.56884	2.98123	4.60223	4.07502	4.56545	4.59106	4.01222	3.29859	3.83242	3.51639
LTBP1	1.79391	2.42371	3.5475	3.20434	3.25878	3.3302	3.79694	3.49147	1.62236	1.62377	1.68014	3.18507	0.90081	0.51697	0.94103	2.46338
RASGRP3	-0.3254	-0.23076	-0.80741	0.31687	-1.41226	0.93653	-1.12536	1.03062	-1.84419	-2.52245	-2.16529	-1.77638	-1.31908	-1.36282	-2.18463	-1.47848
FAM98A	4.51167	4.53283	4.0465	4.46861	4.52028	3.88465	4.96721	3.8772	4.66354	4.35074	4.55337	4.65811	4.36013	4.02919	3.87983	4.13494
CRIM1-DT	1.61766	2.13232	1.3853	1.32728	1.77915	2.44892	0.36474	2.73503	3.54655	5.3136	3.6827	3.65119	3.79585	4.19449	2.18717	2.70377
CRIM1	3.92016	3.43329	3.53646	3.01173	3.66653	1.98417	3.64502	1.85304	6.51735	5.76908	6.446	5.16243	6.00869	4.64041	5.76555	4.39866
FEZ2	2.67116	2.98309	2.8248	3.09989	2.21061	1.57516	2.56355	1.67736	3.26269	3.47717	3.25205	3.89253	3.38126	3.31361	2.95613	2.84481
VIT	-3.32193	-3.32193	-3.32193	-3.32193	-3.32193	-3.04512	-3.32193	-3.32193	-2.3418	-1.46496	-1.99022	-1.00974	-1.60188	0.23613	-1.00155	0.51295
STRN	1.96491	1.77381	1.96805	2.17083	2.92167	1.38444	3.58209	1.21628	3.10383	2.54147	2.86159	3.57036	-0.09247	-0.0207	-0.2264	0.03668
HEATR5B	1.79764	2.1338	2.1818	2.60733	2.75958	2.59417	3.47482	2.55635	2.71944	3.09052	3.01878	3.8897	1.93343	2.42536	2.55562	2.789
GPATCH11	2.29359	2.47945	2.16022	3.35576	1.75	1.42403	2.58489	1.72201	2.46044	2.11844	2.34914	2.2681	2.89327	3.10235	2.26167	2.21314
EIF2AK2	3.03532	3.03441	2.76093	3.13628	3.12431	2.22516	3.80378	2.45036	3.68111	3.50517	3.60945	4.5305	3.6933	3.9061	4.01464	4.23648
SULT6B1	1.18097	1.48313	1.52397	1.2584	1.69355	1.69549	2.32794	1.4581	1.75193	1.05446	1.75562	1.43822	2.80393	2.26597	1.66715	1.5365
CEBPZOS	4.80409	4.95726	4.76383	5.25086	4.33488	5.00089	4.99955	5.02157	4.30054	4.27262	4.18231	5.0514	5.04664	4.68293	4.45894	4.51396
CEBPZ	5.73402	5.77437	5.52311	6.02785	5.4035	4.84337	6.03385	4.89284	4.99749	5.06649	4.87938	5.60878	5.22741	4.93556	4.89572	4.80324
NDUFAF7	3.27292	3.14285	3.01498	3.43037	2.97034	2.94646	3.31642	2.58892	2.70036	2.72205	2.62198	3.22184	2.79938	2.6377	2.13173	2.05865

PRKD3	2.64955	2.84471	2.84798	3.11279	2.36508	1.3981	2.98839	1.22887	3.39237	2.98633	3.21457	3.91479	3.17045	2.75188	3.28747	2.94634
QPCT	4.89932	5.12341	5.24487	5.74703	0.72009	-0.03565	0.63179	-0.50673	2.60998	3.52397	2.78133	4.40534	3.3281	4.13999	2.87613	4.26649
CDC42EP3	3.06932	3.01995	2.79843	2.89244	-3.14982	-3.0277	-2.9675	-3.0863	5.0153	4.73896	4.3992	4.7164	3.79473	3.59552	3.43332	3.51506
RMDN2	-0.60754	-0.68325	-0.51918	-0.20487	-0.57467	-1.6332	-0.13746	-1.34971	-0.746	-1.11468	-1.39043	-1.25538	-0.56122	-2.1122	-1.42761	-1.72736
CYP1B1	2.84758	2.29968	2.67787	4.29166	1.04799	-2.71603	1.70316	-3.11591	2.01619	1.95722	2.49435	2.90485	4.64103	2.79712	5.00221	4.13484
CYP1B1-AS1	0.79597	0.39677	0.65013	1.47714	0.01061	-3.04603	-0.2196	-3.0024	0.58998	0.81412	1.39307	1.06746	1.98026	0.33732	2.90748	2.02377
ATL2	3.85455	3.51875	3.51283	3.91892	4.6878	3.75488	5.16026	3.74077	3.44944	3.13563	2.93351	3.70826	4.21916	3.53754	3.76586	3.38239
RPLP0P6	5.15261	4.98198	5.21002	3.91595	4.64453	4.41426	4.14095	4.06552	6.10533	6.07657	6.07007	4.57616	3.51663	2.03598	3.56421	2.79236
HNRNPLL	2.93006	3.0233	3.12364	3.49387	2.94	2.31099	3.2636	2.02655	3.3631	3.31004	3.23866	3.47326	3.49034	3.36578	3.26612	3.37052
GALM	3.28993	3.40476	3.33897	3.64749	4.26599	3.99666	4.11185	4.13504	2.53378	2.19526	2.3133	2.3081	2.90457	2.96515	3.40475	3.03858
SRSF7	6.0571	5.67333	5.62458	5.52661	5.62884	5.43056	5.57878	5.55663	5.87116	5.06912	5.65558	4.60132	5.44034	5.25398	4.82237	4.51411
GEMIN6	3.27422	3.38228	2.8533	2.78345	2.89222	2.82041	2.85001	3.04802	2.41512	2.4877	2.39566	1.98679	2.80768	2.75589	1.57123	1.53413
DHX57	2.56137	2.50178	2.28653	2.80397	2.62388	2.46333	3.30987	2.40348	2.62983	2.38336	2.77743	3.46913	2.45912	2.25289	2.80441	2.93728
MORN2	4.25156	4.43447	4.11185	4.6033	3.14141	3.87899	3.69526	3.42296	3.21618	3.05837	3.0824	2.85205	4.08702	4.24287	3.13621	3.1313
ARHGEF33	-2.81187	-2.35694	-2.90512	-3.08502	-0.95938	-0.73631	-0.44731	-0.46964	-3.10244	-2.82729	-2.52928	-2.70268	-2.79284	-2.409	-2.97716	-2.9704
SOS1	1.93363	1.93512	2.13364	2.64768	3.32496	2.32092	3.93437	2.35691	2.81639	2.71805	2.76939	4.01725	2.90393	2.8088	3.23443	3.23966
SOS1-IT1	0.20434	0.57468	0.26287	0.59108	0.72321	0.62225	1.6608	1.29611	-0.05397	0.4365	-0.00344	0.34875	0.16123	0.76077	0.14346	0.9799
MAP4K3	2.35825	2.68528	2.96049	3.78095	3.32021	2.94342	4.11329	2.57113	3.06734	3.43231	3.09898	3.95998	2.89455	2.86397	2.61384	2.55887
MAP4K3-DT	0.89651	1.0813	1.15567	1.56012	0.54534	1.56171	1.16956	1.35893	-0.32934	0.33999	0.07611	0.82371	-0.03662	-0.12361	-0.20179	-0.75058
THUMPD2	2.04253	1.82405	1.76343	2.1807	1.71816	2.27547	2.17485	2.10484	2.06432	1.9771	1.92655	1.9674	2.02721	1.49869	0.50388	0.45199
SLC8A1-AS1	-0.32917	-0.18249	-0.2004	0.17493	-3.21386	-3.19417	-3.20359	-3.17277	-0.43699	-0.20377	-0.62096	-0.16809	-2.87968	-3.32193	-2.88925	-3.32193
SLC8A1	0.10246	-0.0028	-0.05284	-0.06847	-3.27918	-3.32193	-3.27502	-3.32193	0.73088	0.76071	0.38586	0.31943	-2.68527	-3.06678	-2.90183	-3.22553
PKDCC	-0.79944	-1.13572	-1.07454	-1.23612	2.7941	2.62241	1.73556	2.37952	0.1221	0.10092	0.16892	-1.13238	1.97319	1.88017	3.16561	2.13219
EML4	3.07244	2.88688	3.09034	3.22138	4.95692	4.91038	5.39341	4.56379	3.81883	3.11013	3.78459	3.84248	3.72762	2.37959	4.305	3.32145
COX7A2L	5.11931	5.26344	5.10444	5.4031	4.57324	4.8929	4.70129	4.73245	5.0087	5.161	4.88925	5.06981	4.52426	4.24918	3.992	3.78962
KCNG3	-1.23536	-2.22498	-2.03344	-1.59686	0.53076	-1.61826	0.19154	-1.27291	-0.74803	-1.20851	-1.26749	-1.22739	0.09752	-0.51032	-0.22477	-0.6557
MTA3	1.83288	2.03907	1.96269	2.25879	2.16464	2.34812	2.49735	2.46195	2.69745	2.85267	2.62926	3.33432	1.89777	1.64322	2.23097	2.06118
OXER1	-2.80009	-3.32193	-3.32193	-3.32193	-3.32193	-2.79525	-2.82954	-3.32193	0.08734	0.25407	0.54034	-0.47459	-2.89819	-2.30718	-2.82005	-2.434
HAAO	-2.96048	-3.32193	-2.9026	-1.6636	-3.00945	-2.95695	-3.32193	-2.57581	2.7902	2.88273	2.64715	2.11686	-1.84456	-2.09941	-1.14122	-1.03217
ZFP36L2	3.6231	3.61054	3.69897	3.27743	5.39441	4.15953	5.22274	4.2598	5.74227	5.38659	6.16853	4.67587	2.43662	3.03814	3.51697	3.23633
LINC01126	-3.32193	-1.95025	-1.90193	-2.19067	0.40225	-0.21293	-1.14526	-1.19595	-2.25419	-1.61113	-2.02011	-2.78887	-3.32193	-3.32193	-2.78569	-3.32193
THADA	1.54684	1.49254	1.22882	1.60506	2.18605	1.95814	2.58855	1.73396	1.72964	1.41597	1.84568	2.27022	1.63106	1.50786	1.41912	1.70889
PLEKHH2	-2.42039	-2.4068	-2.11473	-1.84002	-0.00744	1.37705	0.64696	1.74717	-1.12742	-1.25289	-1.53429	-1.01015	-1.28941	-0.44514	-1.16301	-0.0784
DYNC2LI1	1.29449	1.3303	1.57874	1.96264	1.46592	2.51287	2.11627	2.25024	0.99632	1.6833	1.50255	2.01268	1.42259	1.9506	0.95998	1.25079
LRPPRC	4.98829	4.85534	4.7522	5.08451	5.45814	4.48719	6.03657	4.58559	5.07106	5.23344	5.06273	5.796	5.36757	5.36156	5.25054	5.19636
PPM1B	2.94988	2.90125	2.75845	2.80387	3.49678	2.37962	3.6859	2.51788	3.5743	2.77693	2.76815	2.59839	3.47248	3.28245	3.52518	2.94935
SLC3A1	0.1487	0.57052	0.35706	0.88951	1.63854	1.3892	2.05055	1.3457	0.82279	1.00776	0.32819	2.06847	1.90097	2.50725	1.71017	2.0091
PREPL	1.75476	1.89197	1.58962	2.10234	2.85031	2.22398	3.21812	2.07772	2.25047	2.43695	1.69859	3.51534	3.01964	3.66376	2.91004	3.25092
CAMKMT	0.22598	-0.10558	-0.00213	-0.13584	0.95739	1.26331	1.21076	1.2846	0.03811	0.15398	0.61385	0.24816	0.1247	1.57742	1.09919	0.8991
LINC01833	-3.32193	-3.32193	-3.32193	-3.32193	-3.20406	-3.32193	-3.07448	-3.01344	-0.55116	-0.40129	-0.74093	0.17135	-1.27206	-0.34284	-1.16983	-0.86277
SIX3-AS1	-3.32193	-3.32193	-3.32193	-3.32193	-3.32193	-2.57195	-3.32193	-3.32193	1.54676	1.53405	0.98989	0.39456	-3.32193	-1.46119	-2.6049	-0.83491
SIX3	-3.32193	-3.32193	-3.32193	-3.32193	-2.20037	-2.93123	-3.32193	-2.87209	4.24907	4.94494	4.33003	4.58047	-2.53701	0.05177	-1.85611	-0.08844
SIX2	-2.88685	-2.48186	-2.44693	-3.32193	-2.7874	-3.32193	-3.32193	-3.32193	0.61537	0.0235	0.82969	-1.90596	-2.07172	-1.77428	-1.19742	-2.30012
SRBD1	1.74245	1.409	1.43631	1.69873	1.67964	0.80633	2.18182	0.84936	2.01858	1.63759	2.17965	1.87698	1.29289	0.65731	1.42496	0.4917
PRKCE	1.11636	0.87346	0.66434	0.68085	0.57591	-0.52699	0.64375	-0.50307	2.70467	2.97408	2.55326	2.88166	0.62494	0.56752	0.71426	0.5238
EPAS1	4.12486	4.53953	4.56822	4.77851	5.5484	5.09995	5.63337	4.83259	3.49739	4.21007	3.00302	4.96836	2.95	2.45329	2.98439	3.70424
ATP6V1E2	0.76665	0.61782	0.98528	0.83808	1.06803	1.41666	1.35312	1.49869	0.6805	0.08857	0.39781	0.40661	0.6953	0.53929	0.99533	0.65686
RHOQ	4.31546	4.6673	4.61504	4.78166	3.9948	4.37208	4.09873	4.30929	4.42403	4.32755	3.8523	5.16689	4.32787	4.27468	3.89171	4.18477
PIGF	2.77301	3.19057	3.09757	3.40202	3.12782	3.14291	3.33393	2.97636	3.36244	3.2723	2.96723	4.12776	3.16473	3.05155	2.72355	2.83374
CRIPT	3.61857	3.95501	3.62982	4.23913	3.12703	2.98431	3.30151	2.73558	3.79423	3.54142	3.27431	3.26445	3.15515	2.40081	3.09571	2.2548

SOCS5	1.26136	1.34481	1.51506	1.84935	2.55787	1.86953	3.46622	1.78903	4.26599	4.90464	4.83438	5.54204	3.48234	2.78409	3.75437	3.02934
LINC01119	-1.41793	-1.81254	-1.92986	-1.70028	-3.32193	-2.68541	-3.32193	-2.59738	-1.05131	-0.25072	-0.30355	-0.68973	-1.10392	-2.42946	-1.95958	-1.94022
MCFD2	4.63484	4.83205	5.01889	5.04316	5.20062	5.00373	5.96305	4.50809	5.39027	6.02131	6.47843	6.35168	4.67246	4.33155	5.43198	4.61692
TTC7A	3.389	3.2425	3.06065	2.65944	3.32937	2.72591	2.91329	2.62549	3.36695	3.15889	3.23481	2.55347	2.78816	2.15402	2.67614	2.28184
STPG4	-2.33961	-2.69196	-2.95571	-2.93466	-2.29626	-2.52298	-3.026	-2.21061	0.54681	0.89841	1.03252	0.12997	-2.5028	-2.50503	-2.77029	-3.32193
BCYRN1	8.47081	8.06827	7.69658	5.8434	6.88191	8.85512	7.20485	9.29846	7.93148	7.90893	7.61084	8.5591	6.11719	6.98902	4.32963	5.33062
EPCAM	-1.81165	-2.06898	-1.22257	-1.96814	5.01711	2.31025	5.65703	2.12135	-1.01758	-0.19681	-1.49272	-0.26845	-3.00998	-0.72478	-1.85465	-1.11777
MSH2	4.52346	4.51559	4.29075	4.73781	4.33951	2.99606	4.79332	3.01617	4.32604	3.88418	4.07872	3.79738	4.70489	4.913	4.68679	4.61719
MSH6	4.31497	4.33204	4.18324	4.56307	4.85839	3.55631	5.06889	3.74793	4.25223	3.69907	3.75321	4.27369	4.05929	4.07319	4.30125	4.32622
FBXO11	3.51658	3.62533	3.51694	4.05275	3.35999	3.53371	4.12673	3.67334	3.59698	3.61916	3.53065	4.47868	3.7677	3.795	3.82562	3.88755
FOXN2	1.85269	2.02622	1.6947	2.21427	3.25699	2.53915	3.74669	2.5918	2.69003	2.67381	2.04241	3.33255	2.5907	1.99675	1.79903	1.71292
PPP1R21	2.26929	2.24627	2.83651	3.14256	2.49476	2.9743	2.73259	3.14314	2.71332	2.8814	2.81212	3.70973	2.84649	3.07224	2.9177	3.05467
STON1	-1.93741	-1.56789	-1.41761	-1.3499	-2.18624	-1.53826	-2.51308	-1.66409	-1.48418	-0.7254	-1.47098	-0.00412	-2.04709	-2.09784	-0.98933	-0.55712
STON1-GTF2A1L	-2.6969	-3.05545	-1.882	-2.22373	-2.77539	-3.07979	-2.57789	-2.42669	-1.8993	-0.99749	-1.70856	-0.11644	-2.22917	-2.43707	-1.05995	-1.20049
ASB3	1.79233	2.15136	1.91783	2.74645	1.42214	2.01761	1.61698	1.73932	1.45165	1.90597	1.57758	1.99625	1.5115	1.94891	1.62117	1.76833
GPR75-ASB3	3.21528	3.5865	3.29362	4.1204	2.79032	3.39174	3.01573	3.08794	2.83118	3.29145	2.90444	3.38469	2.88277	3.34284	3.00301	3.21603
CHAC2	3.42415	3.04912	2.53795	2.95223	3.37926	1.75154	3.12271	2.25762	2.62713	1.68528	0.85135	-0.18482	3.93011	3.34911	1.51026	1.91434
ERLEC1	4.93178	4.87475	4.86383	5.51728	5.90895	5.92744	6.44381	5.70017	4.95449	4.71953	5.10202	5.03938	4.07058	3.74166	3.84166	3.66274
GPR75	0.57962	1.11062	0.57646	0.74421	0.23311	0.13981	0.65343	0.19852	-1.49036	-1.38426	-0.84639	-0.0444	-0.34013	0.53244	0.36872	0.94585
PSME4	3.41987	3.41937	3.43687	3.81534	4.35232	3.47491	4.96567	3.2953	3.3398	3.14649	3.29102	4.26975	3.60953	3.52509	4.0127	4.08298
ACYP2	-0.20737	0.42864	0.96518	0.62372	-0.29133	0.81545	0.02778	0.78064	-0.47815	0.17021	-0.11086	-0.12783	-1.13537	-0.56994	-1.12576	-0.65741
EML6	-2.02761	-1.49973	-1.34668	-0.90328	-0.97426	-1.158	-0.83131	-1.16635	-2.7367	-3.11324	-3.09888	-2.72736	-2.77237	-2.54458	-2.49778	-2.76365
CLHC1	0.31854	0.66498	0.60566	1.19107	-0.09016	0.52425	0.03205	0.46506	-1.15615	-0.45476	-0.26664	-0.94832	-0.24031	0.14489	-0.98745	-0.31102
MTIF2	4.89375	4.79853	4.51103	4.98086	4.64962	4.06028	5.05073	4.31135	4.19864	4.35023	4.03767	4.73899	4.15792	4.05333	3.9781	3.78353
PRORSD1P	-0.31422	0.01302	0.02548	-0.56084	-0.37145	0.13345	-0.60209	0.48379	-2.82693	-1.07848	-2.5315	-1.90555	-1.52164	-0.81776	-1.31263	-0.40943
CCDC88A	2.73702	2.6393	2.85495	3.84617	3.24324	3.21025	4.09205	3.43127	2.84039	2.67144	2.85604	3.72452	2.55079	2.45762	3.26861	2.94123
CFAP36	3.80111	4.05767	3.94922	4.37558	3.39119	4.03552	3.84418	3.86647	3.40966	3.81619	3.54365	3.92534	3.75036	3.80891	2.93498	3.10666
PPP4R3B	3.30667	3.12941	3.22216	3.6063	3.75471	2.8083	4.20466	2.89235	3.45103	3.21851	3.12277	3.9163	2.67074	2.61274	2.75985	2.79286
PNPT1	4.00167	3.70558	3.34558	3.79083	4.08678	4.04977	4.51215	4.10581	3.50856	3.20563	3.30565	3.42792	3.03577	2.96024	2.53274	2.01922
EFEMP1	-0.66908	-1.14627	-1.69493	-0.42864	-1.32371	-1.74632	-1.04695	-1.39747	1.87767	1.86091	1.77739	1.85192	0.77518	0.97499	1.33991	0.07836
CCDC85A	-3.32193	-3.32193	-3.0222	-3.00456	-3.20719	-3.32193	-3.32193	-3.32193	0.37932	1.65295	0.90829	0.27337	-3.11692	-2.07743	-2.86546	-3.07072
VRK2	3.2174	3.10687	3.29595	3.51202	3.83636	3.28037	4.54646	3.05448	3.34373	3.10601	3.24706	3.64776	3.4616	2.89494	3.19856	2.52483
FANCL	3.55438	3.49774	3.58423	3.89982	3.76284	3.91318	4.37397	3.76661	3.07734	2.92769	2.90691	3.61361	3.62661	3.01575	2.80989	2.24711
EIF3FP3	2.62635	2.70423	2.76314	2.23302	1.32066	1.7134	0.70869	1.87582	3.54184	3.22258	2.96339	3.81315	1.15546	1.47398	1.50044	1.38443
BCL11A	0.97963	0.69476	1.07666	0.49597	-3.32193	-3.19271	-3.32193	-3.32193	-1.16911	-0.49886	-0.73887	-0.25506	-3.22081	-3.22116	-3.32193	-3.19703
PAPOLG	0.8465	0.97453	0.86377	1.64139	1.08417	0.89053	1.7465	0.8862	1.1243	1.03255	1.11224	1.75342	0.80329	0.73189	0.80525	0.93924
REL	0.47458	0.31673	0.42415	0.87369	1.72036	0.68346	2.32518	0.78574	0.8917	0.8445	1.13764	1.89084	0.81879	0.57419	1.06233	1.04612
PUS10	-0.19934	-0.19392	-0.6972	0.2799	0.98746	0.7568	1.40958	0.56116	-0.32976	0.09546	-0.02515	0.4836	-0.02601	-0.16508	-0.75162	-0.29913
PEX13	2.94554	3.29043	3.09297	3.47137	3.56632	3.1693	4.00542	2.995	2.73963	2.9445	2.84008	3.3089	2.93769	2.93261	3.15002	3.35029
KIAA1841	1.31555	1.62091	1.80012	1.90817	2.03586	1.92663	2.51607	2.05459	0.65875	0.06463	0.14538	0.79801	1.66268	1.74838	1.46034	1.56657
C2orf74	4.13076	4.14527	4.11069	4.54332	2.54693	3.38671	2.77453	3.4841	3.60738	3.79006	3.60252	3.33691	1.84466	1.63197	0.85749	1.07019
AHSA2P	2.57111	2.51402	2.70428	3.2494	3.17609	4.26852	3.05651	4.1175	2.04071	2.33785	2.4187	3.341	2.46055	2.3449	2.26662	2.19021
USP34	1.97856	2.10131	2.14808	2.71839	3.19768	2.42348	3.81411	2.49863	2.98265	3.04095	3.10346	4.33323	3.37602	3.08353	3.65371	3.58155
XPO1	4.17562	3.96126	4.02208	4.34826	4.97042	3.63759	5.64826	3.80578	5.13138	4.33107	4.78281	4.86832	5.20717	4.56001	5.10501	4.26471
FAM161A	1.50277	1.74457	1.37075	2.17077	1.06751	1.12223	1.3995	1.31639	0.68846	0.79805	0.54841	1.14755	1.85578	2.40044	1.48275	1.6783
CCT4	7.36253	7.28577	7.11622	7.29909	7.45799	6.66419	7.69666	6.43096	7.22747	7.18512	7.1579	6.90414	7.31702	6.92085	7.0568	6.70591
COMMD1	3.1368	2.63651	3.18507	2.21618	3.32308	2.76392	2.88736	2.25389	3.9318	3.39062	3.78101	2.21796	3.10694	0.84562	2.92538	0.99986
RPSAP26	1.20134	0.9862	1.36835	0.03642	1.07159	1.56525	0.23322	1.56818	2.24071	3.5992	2.79405	1.36867	0.32052	0.65237	0.848	-0.12392
B3GNT2	3.6837	3.78995	4.02377	4.03873	4.19496	2.10316	4.66369	2.27592	3.31295	2.51047	3.09267	2.75167	3.47087	2.24419	3.05491	2.01595
TMEM17	0.66315	0.55875	0.54273	1.35852	0.92733	0.9379	0.97869	0.42036	-1.42557	-0.27259	-0.52872	-0.878	1.08313	0.50654	0.47732	0.37155

EHBP1	2.98872	2.94673	3.0991	3.44314	4.52129	4.1115	5.13277	4.14659	2.94401	2.42064	2.68244	3.23145	4.74491	4.5621	5.13928	4.9922
OTX1	0.48621	0.32604	0.33404	0.50357	1.03604	1.14454	0.87879	1.37971	1.53601	0.9232	1.25042	0.49491	0.62929	1.09989	1.12775	1.2558
WDPCP	-1.26412	-1.3368	-1.30572	-0.97799	-1.56523	-1.7374	-1.09196	-1.44801	-0.65233	-0.65778	-0.97241	-0.67847	-0.99212	-0.60899	-1.40787	-1.4339
MDH1	5.27962	5.2968	5.27303	5.65379	5.04278	5.36757	5.34104	5.31201	5.64639	5.55503	5.45594	5.30262	5.20646	5.28052	4.46548	4.30198
UGP2	3.69575	3.98594	4.12318	4.16302	4.19955	4.10164	4.79025	4.00162	4.49434	4.43942	4.24774	4.65679	4.20558	4.23943	4.05945	4.1259
VPS54	2.40974	2.41575	2.23437	2.71933	4.0912	4.23597	4.38772	4.29356	3.17204	3.321	2.8109	3.81805	3.07663	3.33581	2.95531	3.10595
PELI1	1.57469	1.76317	1.35161	1.98107	1.42709	1.97221	1.81031	1.97335	1.22078	1.49646	0.87556	1.89562	2.00222	3.30909	1.9262	2.57462
RPL23AP37	-0.30963	-0.44478	0.68469	-3.32193	-1.55571	-0.91919	-1.01193	-1.19663	0.65892	2.40061	-0.54414	1.60805	-3.32193	-1.64063	-3.32193	-3.32193
LGALSL	-0.36105	-0.84426	-0.22093	-1.09807	2.79208	2.8629	3.2358	2.29858	2.31915	1.72118	2.18232	1.70549	2.3061	1.08959	2.34994	1.30163
AFTPH	2.66551	2.88672	2.94883	3.34932	3.19163	3.19141	3.69781	3.28099	2.85151	2.936	2.71259	3.29784	2.84924	3.07212	2.52123	2.67005
SERTAD2	2.90165	2.86361	3.02087	2.64812	2.37453	1.75572	2.7477	1.93452	3.92988	3.58985	3.73607	3.9735	3.19557	3.18283	3.1498	2.83794
SLC1A4	2.30541	1.89844	2.08664	1.64231	2.69061	2.23694	2.71657	2.25786	2.18881	0.98309	1.20257	0.93595	2.89759	3.21089	3.52016	3.48409
CEP68	1.42394	1.17753	1.09537	1.44007	1.17861	1.40494	0.90514	1.51702	1.76806	1.65371	1.68447	1.65357	1.45641	1.4177	1.61169	1.77774
RAB1A	6.727	7.00706	6.83506	7.23205	6.89945	6.98014	7.43282	6.80204	6.82597	6.57488	6.73055	6.78074	6.02382	6.16703	5.79031	5.63327
SPRED2	3.54784	3.42602	3.41192	3.11345	4.02507	3.28583	3.85595	3.08445	4.40424	3.807	4.22337	4.06198	3.35362	2.58807	3.88689	3.26199
MEIS1	-0.92715	-0.95935	-1.22442	-0.30774	-1.37186	-1.14668	-1.50725	-1.3078	-0.72907	-1.11162	-1.09645	-0.878	-2.90654	-2.79257	-2.57544	-2.3439
ETAA1	1.57146	1.84739	1.96282	2.45848	2.60086	2.3561	3.4581	2.56055	1.87627	2.61149	2.34535	2.91656	1.98358	2.65642	2.43673	2.20762
C1D	2.03899	2.42074	2.3812	2.9914	2.63505	2.72427	3.75843	2.47639	2.03757	3.04818	2.89566	2.69361	2.51178	3.45666	2.71284	2.41826
WDR92	1.96549	1.556	1.73271	2.08115	1.98354	1.59654	2.00904	1.51652	2.29906	2.01607	2.34678	1.98553	1.9639	1.80794	1.52579	1.38221
PNO1	4.08188	3.41788	3.21964	3.36776	4.79438	3.60422	4.60734	3.65502	4.25505	3.46146	3.24506	3.07204	4.57664	3.27226	3.10092	2.80964
PPP3R1	4.58134	4.86736	4.8634	5.25687	4.29818	4.17529	5.34491	4.08589	4.40677	4.73603	4.85223	5.15325	4.82758	5.52876	5.56568	5.40658
CNRIP1	0.83541	1.54771	1.42845	1.38568	-3.32193	-3.32193	-3.32193	-3.32193	3.76299	3.97356	3.66154	3.51119	-1.97576	-1.35735	-1.60062	-1.6736
FBXO48	0.00585	-0.22879	0.30565	-0.18746	-0.3359	-0.55283	0.21568	0.04077	0.09927	-0.08106	-0.46521	0.42933	-0.26929	-0.37022	-0.71302	-0.4842
APLF	-0.07634	0.18622	0.33323	0.83918	-2.0397	-1.45817	-1.72226	-1.76577	-1.30954	-0.97403	-1.22841	-0.12872	-0.71002	-0.95432	-0.84435	-0.57086
ANTXR1	3.1568	3.18985	3.52526	3.50945	-3.00969	-3.18985	-3.08679	-3.16776	5.68974	5.42296	5.57212	5.59957	4.51249	3.90124	4.87933	4.13735
GFPT1	3.76909	3.92988	3.7257	4.17278	4.83272	4.75713	5.43607	4.52069	4.48961	4.10876	4.19809	4.77186	4.49921	4.49932	4.43884	4.24179
NFU1	3.97076	4.15894	4.13014	4.26542	4.01645	4.19221	4.28462	4.29559	4.39798	4.07554	4.16073	4.25715	4.18586	4.01591	3.61774	3.57586
AAK1	1.88619	1.83832	1.89766	2.0562	1.54063	1.05039	1.87777	1.35367	1.87241	1.60046	1.72602	2.45642	1.47701	1.18179	1.9685	1.74963
ANXA4	2.34104	2.39632	2.67061	2.9939	4.96363	5.35772	4.41323	5.42	3.08092	3.14162	3.10556	3.44033	3.15651	3.02494	2.81596	2.67191
GMCL1	2.46992	2.55764	2.45913	2.92687	4.22579	3.26573	4.15197	3.25305	2.76728	2.16812	2.24959	2.56436	2.98545	2.32523	2.57443	2.13021
SNRNP27	4.10078	4.08631	3.77827	4.47765	3.76284	3.79313	3.59378	3.89827	4.14943	4.02551	3.43408	3.92938	4.13361	4.51202	2.96707	3.30565
MXD1	2.12596	2.44609	1.9284	2.75469	1.76371	1.65054	2.41263	1.7151	1.7636	1.87601	1.07921	2.09051	1.99183	2.44925	1.39607	1.89275
PCBP1-AS1	5.08119	5.24529	4.78693	5.05095	4.69188	4.59971	4.03492	4.72594	4.77755	4.37763	3.80533	3.97382	3.74702	4.02411	3.04583	3.6114
LINC01816	-0.31575	0.36099	0.5743	-0.25152	1.60299	2.80222	0.49899	2.98348	1.18927	2.06002	0.74749	0.72769	-2.38196	-0.62343	-2.23831	-0.8426
C2orf42	1.6685	1.1762	1.77326	1.7853	2.54569	1.75952	2.65234	1.45668	2.2696	1.89066	2.26171	2.32339	2.57999	1.76754	2.59461	1.89126
TIA1	2.44801	2.37188	2.79641	3.34191	2.8257	3.19007	3.33787	3.31473	2.28657	2.01102	2.39831	2.82195	3.3667	2.87303	3.13618	2.65574
PCYOX1	3.63645	3.62564	3.67141	4.21157	5.84522	5.03557	6.31838	5.16941	4.51964	4.31565	4.35622	5.4302	4.8393	4.51533	5.31428	5.21153
SNRPG	6.47218	6.39747	6.31565	6.12183	6.26345	6.71872	6.17762	6.61346	6.24677	5.82368	5.91516	5.13192	6.16477	6.07114	4.93579	4.67963
FAM136A	5.27726	4.71676	4.66655	4.66879	5.56247	5.17113	5.74609	5.05762	5.06414	3.78176	4.72323	4.1646	5.53612	4.002	4.78248	3.76538
TGFA	0.21834	1.44111	1.04534	1.19618	-3.0598	-3.32193	-3.12536	-3.32193	1.89423	2.9364	1.00835	4.03757	-2.09071	-2.87173	-3.32193	-1.41622
ADD2	-1.15495	-1.58333	-2.0285	-2.01804	-3.08061	-3.07612	-3.32193	-3.32193	2.48976	1.96246	2.17897	1.6624	-3.32193	-3.32193	-3.32193	-3.32193
VAX2	-3.32193	-3.32193	-2.47261	-3.32193	-0.83701	0.17149	-0.45084	0.84005	1.86366	1.74952	2.00914	0.59571	-2.27913	-3.32193	-3.32193	-3.32193
ATP6V1B1-AS1	2.60795	1.3917	1.13679	1.22437	-1.07032	-1.41281	-1.49646	-0.58353	2.41344	1.48234	2.25062	1.11577	-3.32193	-1.94575	-2.59896	-3.32193
TEX261	3.7204	3.73444	3.41634	3.40745	5.3373	4.67358	5.27378	4.4853	4.50434	4.54274	4.81098	4.74238	3.89043	4.31244	4.682	4.71636
NAGK	3.07914	3.40328	3.55855	3.62257	3.16848	4.05149	3.20778	3.89631	3.26973	3.06262	3.32695	2.62187	2.84998	3.26244	2.93985	3.13801
MCEE	1.7023	1.82089	1.34176	1.74755	2.59137	3.54804	2.27162	3.24474	1.10792	1.9727	1.52228	0.82887	1.48322	1.47826	0.55468	-0.16354
MPHOSPH10	3.81943	3.65419	3.43087	4.09803	3.47299	3.82327	4.08338	3.97506	3.42713	3.44553	3.56698	3.73955	3.11248	3.00393	2.64078	2.76766
PAIP2B	-0.29174	-0.35881	-0.29062	-0.66535	0.36335	0.87747	0.23023	0.82864	0.04807	-0.07968	0.10187	-0.27491	-0.32695	-0.23001	-0.46302	0.12314
ZNF638	3.00705	3.037	3.02408	3.58869	3.48556	2.87221	4.06367	3.04789	3.41789	3.64749	3.52705	4.58071	3.07893	2.72749	3.13013	3.18752
DYSF	3.96538	4.55673	4.36483	3.8101	-2.88496	-2.36904	-2.89372	-2.59033	6.09069	5.89529	6.01699	5.18573	0.39846	1.24841	1.86525	2.80924

CYP26B1	0.86222	0.91773	1.09434	1.99561	1.70843	1.13537	1.70649	1.29639	-2.39636	-2.29008	-2.23633	-1.57889	-0.42936	0.99764	0.97213	1.48715
EXOC6B	0.97436	1.22015	1.26267	1.64905	2.50162	1.54807	2.84751	1.32658	2.47931	2.21054	2.27228	3.07619	1.91576	1.93112	2.49502	2.52043
SPR	5.09977	4.7036	4.44098	4.58885	6.6437	6.21175	5.96605	6.24572	5.91026	5.84155	5.79754	5.11482	4.91125	4.74808	4.69682	4.85357
SFXN5	1.87859	1.64748	1.64864	1.20192	1.8017	1.82943	1.11427	1.90889	1.91134	1.56993	1.76843	0.87868	1.26176	0.90767	1.24635	1.53307
RAB11FIP5	4.61541	4.65206	4.58921	3.98061	3.88724	2.83322	3.48754	2.84051	5.46962	5.21156	5.29555	4.95265	3.32914	3.20956	3.84054	4.05254
SMYD5	3.99908	3.48315	3.25805	3.1974	4.77642	4.62019	4.38628	4.44347	4.67746	4.3063	4.55646	3.98336	3.91332	3.69114	4.00737	3.9887
PRADC1	3.8577	3.8728	3.6756	3.39854	5.4269	5.66032	4.99176	5.53464	4.38806	3.63698	3.86933	2.56181	4.13767	4.35225	4.02399	4.11931
FBXO41	-2.75204	-2.59733	-2.66759	-2.42963	2.27869	2.53873	1.58201	2.37023	3.02311	3.4622	3.28168	3.074	-2.57081	-2.17783	-1.85859	-0.56338
ALMS1	1.37637	0.92883	1.37446	0.98718	1.15528	-0.05072	1.34968	0.2192	0.88438	0.15289	0.39895	0.86997	1.09316	0.35265	1.10148	0.69095
ALMS1-IT1	1.42141	0.55027	0.98295	1.89804	-0.59251	-0.77556	-1.06425	-0.17036	0.59734	-1.13542	-0.94411	-1.39837	-0.62527	-0.28828	-0.84375	-1.36747
NAT8	-3.32193	-3.32193	-3.32193	-3.32193	6.25083	5.56979	5.89767	5.4615	-2.35652	-3.32193	-3.32193	-2.49254	-3.32193	-3.32193	-3.32193	-3.32193
NAT8B	-3.32193	-3.32193	-3.32193	-3.32193	2.70489	1.35719	2.18617	1.58308	-3.32193	-3.32193	-3.32193	-3.32193	-3.32193	-3.32193	-3.32193	-3.32193
TPRKB	5.33099	5.30403	5.20158	4.98379	4.74926	4.77581	4.91565	4.79411	4.80036	4.46901	4.29432	3.41998	4.27632	4.15856	3.43979	3.35586
DUSP11	2.8775	2.96683	2.94034	3.17467	2.96196	2.81362	3.33281	2.95568	2.87367	2.87644	2.74942	3.0055	2.84513	3.03921	2.05529	1.92493
STAMBP	2.47626	2.63477	2.51749	2.96352	2.98294	2.78644	3.11974	2.77891	2.86468	2.69981	2.93964	3.14024	2.33246	2.2267	2.43656	2.13089
ACTG2	-3.05451	-3.32193	-1.77094	-2.30161	-3.09174	-3.32193	-2.67089	-3.32193	-3.32193	-3.06325	-3.32193	-3.32193	-0.91382	0.0315	0.74411	-0.22566
DGUOK	5.48028	5.01393	5.29111	5.00991	5.33555	5.71485	4.91761	5.29679	5.68823	4.86856	5.68778	4.35249	5.02548	3.71974	4.21621	2.63002
DGUOK-AS1	5.30339	4.85814	5.23764	4.95524	5.0554	5.55741	4.56793	5.21458	5.23839	4.14978	5.30755	4.00819	4.72573	3.60257	3.82263	2.01442
TET3	1.11652	0.38608	0.7937	0.53543	2.65652	1.14841	2.56877	1.57227	3.00453	2.25455	3.02377	2.81379	3.34817	2.72626	3.72552	3.52201
FNBP1P1	-1.58408	-0.78449	-1.597	-1.34074	0.0597	-1.01877	0.37736	0.0369	-0.63732	-0.054	0.47538	1.32101	0.33198	0.94341	0.88487	0.94351
BOLA3	4.06174	3.96207	3.71724	3.56119	3.73918	4.29596	3.56439	4.31909	3.85009	3.3407	3.48965	2.35397	3.9246	3.54072	2.41572	2.30675
BOLA3-AS1	-0.5302	-0.2996	-0.14853	0.35084	2.68402	2.86557	2.75196	3.0626	1.48821	2.00631	1.70329	1.79489	1.21492	1.88786	1.33584	1.86851
MOB1A	4.12052	3.49308	4.09753	3.69721	4.71563	3.10315	5.01127	2.81806	5.21373	3.73481	4.91888	4.11794	4.61675	2.59593	4.53848	2.34
MTHFD2	-2.1361	-1.66818	-1.99398	-1.24504	4.66831	4.79611	5.09826	4.53174	5.62515	4.18002	4.56834	3.76141	4.56817	3.93454	3.47063	2.7021
SLC4A5	-2.85011	-2.42012	-2.22043	-2.25235	-0.29567	1.01888	0.14021	0.82208	0.34382	-0.16374	-0.02671	-0.00217	-0.0877	-0.56965	-0.3868	-1.01045
DCTN1	4.81515	4.8102	4.7516	4.82485	5.09801	4.25006	5.08901	4.32425	5.67147	5.40328	5.65187	5.56102	4.50274	4.38351	5.18507	5.1319
DCTN1-AS1	0.71414	1.31485	1.02924	0.68936	2.78271	0.02114	2.94926	0.00231	3.12771	2.74971	3.01875	2.8042	2.46001	2.39862	3.60247	3.55688
C2orf81	0.60906	1.2617	0.37555	0.11292	0.12501	2.08397	-0.7765	2.05954	2.29848	3.00386	2.75077	1.87267	-0.06995	1.07225	0.70837	1.51422
WDR54	4.8349	4.76759	5.00112	4.28719	2.48182	3.27437	1.84626	2.90525	5.10909	5.31649	4.92357	4.33386	3.24422	3.32703	3.2209	3.44337
RTKN	0.17867	0.65723	0.80541	1.45572	5.44999	5.19135	4.99161	5.10328	3.95909	3.86714	4.09151	3.71771	3.45575	3.60948	3.87832	3.98073
INO80B	5.19422	5.49281	5.39324	5.25343	4.29852	5.15058	3.81215	5.1081	4.51105	5.51515	5.43492	4.50614	2.73373	3.7901	3.5165	3.90007
INO80B-WBP1	5.54317	5.91023	5.92631	5.87987	6.06352	6.74403	5.01233	6.4957	5.70213	6.28039	6.37065	5.24679	3.94674	4.68041	4.67187	4.83487
WBP1	4.06878	4.57009	4.70309	4.74766	5.5392	6.15643	4.35019	5.85501	4.91036	5.27492	5.47857	4.23581	3.21767	3.81417	3.89742	4.03647
MOGS	5.99206	5.91351	5.94548	5.72784	7.41058	7.18119	6.69262	6.87656	5.32056	5.30586	5.77531	4.68211	4.51172	3.98862	4.61754	4.58565
MRPL53	5.73096	5.53941	5.72376	5.72066	5.79386	6.44163	5.62077	6.38698	5.46815	5.40291	5.37701	5.05873	5.20656	5.00682	4.24457	4.2116
CCDC142	4.26526	4.17811	4.34233	4.33111	4.47325	4.85199	4.10574	4.8247	3.88581	3.87878	3.88699	3.59929	3.68667	3.45617	3.04809	3.03639
TTC31	1.90358	1.73642	1.92613	1.67657	2.62366	2.45724	2.07873	2.51087	2.54107	2.5687	2.55889	2.84636	2.77735	2.7798	2.85065	2.94721
LBX2	2.04559	2.95558	2.74846	2.78826	2.49031	3.53152	1.37108	3.09147	2.22317	3.04354	2.95852	2.73551	0.19347	0.57999	-0.1417	0.72548
LBX2-AS1	3.51838	4.13526	4.24671	4.53506	2.93444	4.30143	1.85243	4.25906	3.13766	3.45227	3.53086	2.97584	1.49231	1.84346	1.55804	1.90084
PCGF1	3.32045	3.04461	3.07451	2.87381	3.13772	3.53129	2.50944	3.78231	2.6812	2.98187	2.15144	2.49602	2.15318	2.42692	0.818	1.3334
DQX1	-2.98395	-2.94753	-3.32193	-2.90697	1.09038	0.5206	0.24395	0.627	-2.93584	-2.72822	-2.97316	-2.51394	-2.45225	-3.05161	-3.32193	-2.72239
AUP1	6.34479	6.16393	6.03767	5.87127	7.37351	7.65202	6.90745	7.50578	6.1173	5.98459	5.98241	5.76188	6.0445	5.79531	5.71762	5.725
HTRA2	5.85425	5.81053	5.76372	5.59362	6.10579	5.66532	5.80574	5.38689	5.32809	5.34254	5.25743	5.07628	4.59515	4.40993	4.77918	4.65168
LOXL3	3.56783	3.57531	3.47535	3.69433	1.95792	2.7209	1.48881	2.59078	2.39191	2.64573	2.50214	2.52761	1.25431	1.45335	1.15249	1.33931
DOK1	1.7477	2.22383	2.01813	2.00733	1.35279	1.69594	0.51662	1.69988	2.6103	3.17549	3.22585	2.62785	-1.47695	-0.33009	-0.71225	0.09597
M1AP	-2.4932	-2.95414	-2.63219	-2.59668	2.21344	1.58662	1.58393	1.47356	3.08061	2.99698	2.73684	2.74358	-3.32193	-3.32193	-3.32193	-3.32193
SEMA4F	0.81368	0.72494	0.79829	0.86	3.49769	2.54444	2.90004	2.48541	2.60141	2.92367	2.93852	2.62878	1.17501	1.32939	1.9576	2.13138
HK2	-1.12542	-0.99357	-0.19542	0.1815	-0.84529	0.8232	-0.54498	-0.46565	5.63083	5.46951	5.42644	5.09569	-2.18231	-1.55729	-1.7587	-1.3228
LINC01291	-1.54958	-1.20502	-1.29245	-0.81289	-3.01532	-3.32193	-3.32193	-3.32193	2.84946	2.98438	2.67277	3.10303	0.70383	-0.52524	-0.12621	-1.35776
LINC01293	-0.48792	-2.07328	-0.9003	-1.28783	-2.98809	-3.32193	-3.32193	-3.32193	4.51295	4.5035	4.32492	5.17754	2.8205	1.61689	1.51145	0.56168

POLE4	4.16095	4.31203	4.0862	4.2447	3.59862	3.89835	3.35583	3.7675	5.64047	5.22894	5.17673	3.88834	4.78977	4.57218	3.89518	4.00771
EVA1A	4.31052	4.36737	4.40444	4.61185	-1.19645	-1.48448	-1.2625	-1.43779	4.98914	5.1162	5.22496	5.20088	-2.79268	-3.0341	-2.97705	-3.32193
MRPL19	4.33198	4.11357	3.775	4.20103	4.37981	4.07307	4.68914	3.95502	4.45823	3.8429	4.15537	4.13048	4.26174	3.63063	3.79724	3.11709
GCFC2	3.27169	3.04915	2.59437	3.02993	3.96077	3.39175	4.0752	3.37201	3.41924	2.87396	3.23235	3.13925	3.17183	2.72656	2.68224	2.29136
CYCSP6	3.97972	3.42931	3.34304	3.77884	-2.25742	-3.32193	-1.55687	-1.30162	-0.51986	-0.24562	-1.44918	-0.76793	-3.32193	-3.32193	-3.32193	-3.32193
SUCLG1	4.39832	4.22318	4.23157	4.22734	4.30263	4.42031	4.43074	4.33285	4.6834	4.38763	4.49174	4.22695	4.18152	4.01422	3.72301	3.27994
DNAH6	-1.65182	-1.93704	-2.16831	-1.83006	-3.18762	-3.2405	-3.24659	-3.13718	-3.2287	-2.96928	-2.88154	-2.91412	-3.19762	-3.32193	-3.24491	-3.16866
TRABD2A	5.18647	5.2483	5.62376	4.18735	3.5702	4.56526	3.10699	4.2711	5.55368	5.43265	4.94009	3.19529	5.07916	5.20894	2.71806	2.49886
KCMF1	3.94872	3.90912	3.959	4.04038	3.79706	3.78958	4.38688	3.54234	4.0027	3.36069	3.68532	3.77042	3.3872	2.95125	3.67321	2.75162
TCF7L1	-0.03038	0.22121	0.24272	-0.08393	-0.4743	0.00959	-1.27983	0.27312	3.72478	3.3961	3.37733	2.34121	1.59471	1.73363	2.5463	2.1171
TGOLN2	4.73825	4.66218	4.92636	4.97453	6.68525	5.70035	6.97533	5.55517	5.59535	4.82698	5.57551	5.47974	5.13728	4.36642	5.23005	4.42142
RETSAT	3.65306	3.82062	3.7508	3.76855	5.43055	5.28369	5.44519	5.01346	4.58356	4.81022	4.71137	4.92221	3.86476	4.0013	4.36976	4.5177
ELMOD3	0.94734	0.73125	0.90563	0.74777	2.48517	1.61956	2.65025	1.40239	1.62998	1.51238	1.82113	1.6742	1.1058	1.15056	1.46691	1.67283
CAPG	6.95945	7.60492	7.89928	7.57416	0.5761	-0.44988	-0.18755	-1.38354	7.62785	8.11591	8.00771	7.23953	5.12893	5.60225	5.57017	5.5676
SH2D6	-2.47018	-2.39351	-2.1899	-1.72181	-0.26055	-0.92375	-0.63359	-0.9806	-2.05709	-1.42824	-2.44725	-2.66921	-3.32193	-2.48583	-3.32193	-3.06333
MAT2A	5.58119	5.46565	5.26227	5.65626	6.3348	5.67714	6.47912	5.43711	6.00486	6.09034	6.63056	6.27545	5.41677	5.59973	5.4325	5.31044
GGCX	3.6003	3.52402	3.43063	3.7395	4.78766	4.96517	4.98009	4.80279	4.04531	3.87626	4.26879	4.05079	3.30023	3.38524	3.43433	3.30231
VAMP8	5.21955	5.45681	5.2791	5.63754	7.15889	7.64446	7.05042	7.48562	3.93713	4.09032	3.92654	2.96886	4.65649	4.76612	3.9064	4.12018
VAMP5	6.20607	6.62653	6.41658	6.10617	1.91168	3.57108	1.55121	3.434	-3.32193	-0.8445	-2.33519	-1.83819	0.44583	1.16692	0.54315	1.42726
RNF181	6.17729	6.25337	6.22912	6.28541	5.71318	6.86683	5.2742	6.61154	6.30916	6.45149	6.28168	5.53844	5.13081	5.02585	4.65795	4.70664
TMEM150A	2.09223	2.36959	2.44608	2.76165	3.25546	4.10811	2.64574	4.03741	1.56434	2.44514	2.48776	2.61828	0.16595	-0.38498	0.55103	0.45725
USP39	4.67313	4.4873	4.46511	4.36651	4.87286	4.21152	4.58412	4.28448	4.9641	4.14819	4.6856	3.99993	4.42117	4.2551	4.47776	4.15602
C2orf68	3.04929	3.09237	3.17277	2.9967	3.65063	3.91213	3.38212	3.98464	3.27917	3.65717	3.33401	3.51146	2.12349	2.57588	2.64672	3.11506
ATOH8	0.74084	1.04557	0.47126	2.24613	-2.59859	-0.25816	-2.35559	0.47654	-1.44515	-0.48222	-0.69883	-0.45466	-2.18386	-1.60233	-1.3044	-0.65289
ST3GAL5	0.70621	0.87156	0.94114	1.32291	0.23221	0.68274	0.8013	0.65154	1.26293	1.93168	1.68877	3.06043	1.58983	2.18171	2.08608	2.72
POLR1A	2.94183	2.79426	2.49477	2.3951	4.08512	3.00938	4.03388	3.23688	4.11073	3.77164	4.19881	4.10616	3.45382	3.31802	4.20905	4.09147
PTCD3	2.79373	2.65261	2.61683	3.0198	3.21529	3.47672	3.72489	3.62774	2.62965	2.70506	2.79709	3.27887	2.95443	3.55284	2.80669	2.92106
IMMT	5.34341	5.42994	5.43343	5.59622	5.37033	5.57302	5.75945	5.46808	5.97822	5.47894	5.68241	6.15945	5.17454	5.20966	5.27608	5.22568
MRPL35	4.11006	3.96705	3.67574	3.97115	3.35869	3.38536	3.76249	3.21871	3.56763	3.53518	3.51587	3.67172	3.78038	4.05204	3.45119	3.54247
KDM3A	2.07615	2.55002	2.89594	3.03086	0.98446	1.39259	1.54731	1.26535	2.02573	3.149	2.58495	4.05301	1.46887	1.82535	2.21857	2.14106
CHMP3	3.20015	3.40107	3.30363	3.89845	4.16951	3.73029	4.29658	3.71682	3.94697	4.10389	4.11524	4.49882	4.28016	4.49701	4.35154	4.39631
RNF103-CHMP3	4.91918	4.97791	4.94823	5.28121	6.13817	5.28913	6.23815	5.02366	5.43282	5.76622	5.74399	5.73107	5.37874	5.77932	5.64224	5.87419
RNF103	2.13075	2.40722	2.45537	3.17761	3.73284	3.5645	4.20573	3.60442	2.10918	2.4843	2.1636	2.84936	2.20064	3.19744	2.81017	3.28363
RMND5A	3.68785	3.37408	3.25289	3.35783	3.42278	2.89435	3.99587	2.61765	3.92113	3.27674	3.66099	3.88605	3.72158	3.93127	4.30673	4.25897
RGPD1	1.35533	1.47217	1.29922	1.83154	-1.61976	-1.08217	-0.98739	-0.50337	-1.73013	-0.68456	-0.98875	-0.25283	-1.71957	-2.08757	-1.85836	-1.45708
PLGLB1	-0.23923	0.06609	-0.2971	0.43033	-1.97965	-0.18469	-1.72504	0.31816	-2.9656	-1.80621	-2.00106	-2.07645	-2.36279	-2.1096	-2.56459	-2.2002
LINC01943	4.67326	4.49457	4.53776	3.43222	3.2009	2.58624	3.01898	2.53486	5.69266	5.88905	5.38353	4.26339	4.73145	4.41245	3.29955	3.07355
CYTOR	3.94844	3.75322	3.76437	2.84549	2.36329	1.82834	2.28051	2.02854	4.90884	5.02317	4.55053	3.85926	3.99565	3.71044	2.58407	2.49124
PLGLB2	0.18014	0.41871	0.05416	0.94536	-1.4041	0.47314	-1.48548	0.69183	-3.32193	-1.5889	-2.04286	-2.11673	-1.93316	-1.15381	-2.33188	-2.09291
RGPD2	1.50062	1.61566	1.42182	2.01725	-1.53927	-0.48491	-1.21726	-0.35199	-1.65122	-0.58901	-0.9654	-0.31847	-1.475	-1.6246	-2.01325	-1.23946
MTATP8P2	2.23592	1.99322	2.69437	0.63702	0.79227	3.35054	-1.12455	3.63678	-0.00985	0.2768	0.75882	0.25424	-0.51043	-1.32216	-0.26246	0.29259
KRCC1	0.68286	1.39745	1.49954	2.83639	1.05266	3.05946	1.43488	3.10951	1.64905	2.93672	1.55759	3.15607	3.525	3.19459	2.53767	2.69919
FABP1	-3.32193	-3.32193	-2.86537	-3.32193	2.71054	4.07677	2.41958	3.66192	-3.32193	-3.32193	-3.32193	-3.32193	-3.32193	-3.32193	-3.32193	-3.32193
THNSL2	-2.53608	-2.69729	-2.54381	-2.07633	-2.82619	-3.15583	-2.78527	-3.12842	-0.90276	-0.03976	-0.41483	0.4298	-3.19159	-3.32193	-3.32193	-3.1613
EIF2AK3	1.28828	1.45686	1.47172	1.99804	3.41177	2.63759	4.15852	2.39159	2.01242	1.98919	1.72068	2.65564	1.99376	2.27458	2.22707	2.24105
EIF2AK3-DT	-1.92638	-2.4631	-1.4835	-1.94269	-1.09108	0.09902	-0.93547	0.18213	-2.28409	-1.7749	-1.99898	-2.29994	-2.67313	-1.4392	-1.96742	-2.88505
RPIA	4.25422	3.73323	3.60771	3.45532	3.68005	3.38694	3.54408	3.39097	4.74693	4.06565	4.54429	3.43428	4.28144	4.15943	3.3958	2.73498
IGKC	4.39398	3.96258	3.67337	4.09994	-3.32193	-3.32193	-3.32193	-3.32193	-3.32193	-3.32193	-3.32193	-3.32193	-2.20917	0.0381	-2.04881	-3.32193
LSP1P4	4.42131	4.43012	4.63542	4.66605	1.74688	2.00986	1.30442	2.28426	-0.15718	0.73712	0.26859	0.46007	-3.32193	-3.32193	-3.0088	-3.32193
GGT8P	4.70421	4.32746	4.74898	5.32425	-0.04778	-1.34006	-0.35681	-0.68836	-3.32193	-2.05482	-3.32193	-3.32193	-3.32193	-3.32193	-3.32193	-3.32193

MRPS5	4.72068	4.45334	4.34819	4.45	3.97139	4.48136	4.09896	4.50117	3.87458	3.51592	3.77165	3.30911	4.09167	3.93575	3.9903	3.58539
ZNF514	1.38194	1.40297	1.68598	1.76877	1.00796	0.68603	0.89106	0.65709	0.30889	0.85014	0.8756	1.33905	1.64406	2.08846	1.9938	2.02851
ZNF2	1.07074	0.70132	0.67416	1.00897	0.94952	1.20126	0.83678	0.76042	0.3493	1.03409	0.38651	1.33939	0.59941	1.25667	0.83792	1.29293
PROM2	-2.30466	-2.21798	-2.38841	-1.616	-3.17302	-2.84863	-3.15909	-2.63574	1.57849	1.93654	2.35407	2.02009	-3.05821	-3.18452	-2.6339	-2.15252
KCNIP3	-1.57054	-1.91032	-1.68825	-2.41776	-3.15117	-3.18481	-3.32193	-3.01789	3.14319	3.41227	3.56041	2.96239	-1.6257	-1.43656	-1.18784	-1.59523
FAHD2A	2.93525	2.65115	2.63294	2.38388	2.10272	2.86576	1.7761	2.77531	2.39931	2.47066	2.44642	2.30202	1.43731	1.93427	1.93162	1.70867
LINC00342	-0.72744	-0.58713	-0.25574	-0.59647	-0.64071	1.01316	-0.55402	1.63095	-0.40599	-1.08426	-0.91607	-0.59493	0.62289	0.0366	0.39734	0.29663
ANKRD36C	0.47969	0.29603	0.62015	2.10609	0.27625	1.73188	0.71177	2.20514	-0.03904	-0.49204	-0.61427	0.51611	1.20063	1.0057	0.72739	1.2124
FAHD2CP	0.79247	0.98003	0.99316	0.3678	0.79303	2.74425	0.78657	2.35159	1.16988	1.22542	0.77866	1.11295	0.98203	0.75313	0.50162	1.17563
GPAT2	-2.82661	-1.74064	-2.46686	-2.72059	-1.6422	0.01603	-1.60199	-0.23944	-1.95057	-2.03011	-1.89035	-2.12379	-1.531	-1.67492	-2.38642	-1.55704
DUSP2	-0.02359	-0.13352	-1.78403	-0.98363	-0.32281	0.56654	-1.07406	0.42471	-2.76912	-2.84957	-2.44826	-3.32193	1.35321	1.94978	1.41669	1.22313
STARD7	5.19665	5.14756	4.61554	5.00984	6.23055	5.01486	6.1664	5.18279	5.42418	4.98894	4.83459	4.84567	6.44603	6.47201	6.02907	6.22515
STARD7-AS1	3.64654	3.57204	3.33366	2.27946	5.2874	2.82414	4.54753	2.62105	4.49121	4.0056	3.93196	2.57465	4.15003	4.2227	4.47949	4.62275
TMEM127	3.96735	4.27327	4.20708	4.18682	5.09401	4.69395	5.07372	4.44271	3.95302	4.02243	4.03251	4.48932	4.18864	4.53098	4.97246	5.39218
CIAO1	5.78369	5.61358	5.33731	5.4893	5.19485	5.22191	5.02255	5.20829	4.24706	4.37604	4.38429	4.22632	4.92832	4.92033	4.65911	4.79773
ITPRIPL1	-3.32193	-3.32193	-3.32193	-3.04619	3.14464	2.9208	3.0104	2.88413	2.04061	1.65105	1.56998	1.31511	-3.32193	-3.32193	-3.32193	-3.10431
NCAPH	4.73176	4.15586	4.34712	3.64778	4.79038	3.87094	4.87476	3.74027	4.39151	2.95392	3.89628	2.68975	5.23928	4.87206	4.92879	4.24793
NEURL3	0.88018	1.69591	1.55357	1.71294	3.13522	1.95276	2.99849	1.32366	-2.26267	-1.12133	-2.60473	-2.0155	-0.82966	0.36617	-1.44583	-0.38213
ARID5A	3.66239	3.83022	3.98017	3.40953	2.45935	2.60054	1.40064	2.57339	1.4763	1.50036	1.52306	0.66536	2.25497	1.88866	2.17865	2.20288
KANSL3	3.29696	3.20959	3.30768	3.54397	3.44831	3.02093	3.21371	3.05349	2.73435	2.77598	2.82623	3.00447	3.26251	3.38076	3.83913	3.85882
FER1L5	-2.71914	-3.09996	-2.63088	-3.19293	-2.61763	-2.18534	-2.6866	-1.49181	-3.32193	-3.32193	-3.21524	-3.32193	-3.02071	-2.83148	-2.67492	-2.66343
LMAN2L	3.38836	2.9123	3.28887	3.07975	5.31128	4.10111	5.11597	3.79001	3.30605	2.56111	3.15138	2.66602	4.87619	3.79461	4.85588	4.00074
CNNM4	2.32499	2.12067	2.05401	1.86814	3.74775	2.51752	3.39262	2.69671	1.49642	1.83774	1.47374	1.63794	2.63739	2.67852	2.87353	2.7605
CNNM3	3.21597	2.89879	2.75321	2.58538	5.23725	4.16232	4.76302	4.53469	2.85799	2.73158	2.69835	2.51891	3.40113	3.30884	4.08724	4.21503
ANKRD23	3.72919	3.6045	3.12851	3.26271	4.14325	4.35752	3.69475	4.49507	2.46873	2.01639	2.18084	1.37	3.48086	3.16608	3.15144	2.9956
ANKRD39	3.27304	3.10413	2.71192	2.77532	2.92629	3.63321	2.21828	3.64992	1.70711	1.28979	1.35546	0.36418	2.75504	2.4533	1.97713	1.98736
SEMA4C	4.02775	4.31219	4.35964	4.29699	3.47895	4.3561	2.99703	4.24727	2.70759	3.35581	3.30081	2.90862	3.51065	4.18011	4.17828	4.77203
FAM178B	1.22227	0.46238	0.11853	-0.67778	-3.32193	-3.32193	-3.32193	-3.32193	-3.32193	-3.32193	-3.32193	-3.32193	-3.32193	-3.32193	-3.32193	-3.04258
FAHD2B	1.31659	1.38944	1.2297	0.78196	2.29408	2.83104	1.55108	2.52419	2.39264	2.01294	2.21117	1.90627	2.27147	1.9722	2.46678	2.20019
ANKRD36	1.26891	0.73811	1.2195	2.22936	0.93377	2.11147	0.9869	2.57598	-0.13305	-0.12189	-0.2235	0.56268	1.36453	1.31716	0.91142	0.82628
ANKRD36B	1.34568	0.8973	1.51435	2.20624	0.89706	2.03503	0.98777	2.47691	-0.24108	-0.03919	-0.01366	0.63792	1.37653	1.27353	0.61695	0.92669
COX5B	7.09418	6.97408	7.05751	6.91713	6.85548	7.55731	6.42913	7.41312	6.00043	6.26129	5.96014	5.41214	7.12208	7.05943	5.97941	5.93486
ACTR1B	4.45407	4.38848	4.56435	3.84088	4.78316	5.04498	4.02749	4.94219	4.2098	4.2956	4.13643	3.66021	3.1823	3.36493	3.16436	3.30284
C2orf92	-2.26919	-1.73218	-0.74896	-1.16283	-1.54214	-0.06338	-1.68161	-0.09267	-3.13445	-2.77548	-2.74207	-2.30858	-2.51603	-2.17687	-2.67124	-2.13192
TMEM131	2.34112	2.2679	2.48778	2.82058	3.23942	2.55813	3.81859	2.53997	2.38413	2.31834	2.20404	3.41821	3.48151	3.24891	3.54933	3.96488
HMGN1P36	5.09669	5.01147	4.73528	4.51008	4.988	4.69522	5.30318	4.46558	5.7944	4.58248	5.71264	4.52742	4.32882	2.72377	4.93946	3.28658
INPP4A	0.69462	0.88281	0.96182	1.09977	1.30283	0.89177	1.05121	0.6889	0.63255	0.8272	0.8874	1.24878	1.56048	1.85938	2.00596	2.16237
COA5	3.38131	3.56959	3.38233	3.75309	3.44735	3.92574	3.26728	3.8826	1.82049	2.47259	2.16953	1.86612	3.19447	3.38698	2.29448	2.43195
UNC50	3.55572	3.65092	3.64037	3.9805	4.77927	4.73702	5.00887	4.61122	2.68959	2.72483	2.92587	2.79964	3.77958	4.2195	3.04863	3.54196
MGAT4A	-2.23221	-3.32193	-2.42714	-2.38407	2.73291	2.46437	3.60578	1.73572	-2.96462	-3.01958	-2.90621	-2.77753	-2.85848	-2.85989	-2.0221	-3.01631
TSGA10	-0.45122	-0.71807	-0.59718	-0.22221	-0.81276	-1.1484	-0.49606	-1.23297	-1.8823	-1.82284	-1.75519	-1.22021	-0.27663	-0.92361	-0.75553	-0.80489
C2orf15	-1.08947	-1.07797	-0.40087	0.11198	0.93115	0.92246	1.02414	1.11637	-1.03802	-1.24793	-1.16843	-0.84709	-0.08855	0.39275	0.84425	0.75568
LIPT1	1.89137	1.34842	1.08223	2.09429	1.20971	1.59777	1.11521	1.33933	1.00004	0.95532	0.6615	0.77755	1.9393	1.34812	1.18806	0.51534
MITD1	3.46509	3.59445	3.63505	3.95493	2.6131	3.07244	2.57033	2.99451	2.12232	2.05333	1.87466	2.12287	2.63591	2.94217	1.58925	1.61977
MRPL30	5.01904	5.0051	4.68218	4.63898	4.70981	4.22516	5.06278	4.17263	4.48269	4.64954	4.2951	4.53627	5.12721	5.36318	4.81373	4.7684
LYG1	-1.38069	-1.56022	-1.50481	-0.29137	-0.57134	1.12568	-1.45352	1.76293	-3.32193	-1.71114	-2.01672	-1.43728	-1.40297	-0.61194	-0.49379	-0.33471
TXNDC9	4.54607	4.86837	4.48552	5.22221	3.95132	3.18157	4.18066	3.16732	3.90039	3.67851	3.42239	3.81808	5.0314	4.81316	3.85211	3.86226
EIF5B	6.44076	6.74331	6.46423	6.84743	5.35308	6.34393	6.15632	6.69515	4.73235	4.49709	4.81287	5.38228	5.42267	5.92039	5.75488	5.7248
REV1	1.67216	1.62939	1.69438	2.17842	1.93872	2.00685	2.26578	2.18771	1.26253	1.55671	1.30446	2.11546	2.58523	2.86597	2.43887	2.3258
AFF3	2.6279	2.42759	2.76691	2.35051	0.20574	-2.62942	0.02254	-2.8758	1.9463	1.90874	2.09508	2.7209	-2.71301	-2.62738	-2.71445	-2.40205

LONRF2	-2.44571	-2.4148	-2.98396	-2.38521	-1.10678	-1.69719	-1.12414	-2.0641	-2.60167	-1.7173	-2.25401	-0.99616	2.03932	2.2435	2.86389	2.89922
CHST10	-3.32193	-3.32193	-3.32193	-3.32193	2.66545	2.6732	2.73448	2.72272	2.13174	1.78768	2.10799	2.04173	2.98284	2.9728	3.23988	3.22236
PDCL3	4.57	4.79646	4.52667	4.84439	5.02415	4.9502	5.23907	5.07587	3.8369	3.63739	3.74942	3.33709	4.32371	4.72099	4.01854	4.32465
NPAS2	1.21301	1.03754	1.21604	0.95851	0.85809	0.16865	0.84283	-0.41437	1.84332	2.13422	2.02555	2.54235	0.44441	-0.14373	0.66812	1.20229
TBC1D8	3.15858	2.78447	3.30813	3.21417	4.33236	5.34703	4.33542	5.08265	0.79137	1.7714	0.94613	3.00559	4.08831	3.89725	4.19276	4.84297
TBC1D8-AS1	-1.51225	-1.53082	-2.24251	-1.74809	-0.33697	-0.51112	-0.37254	-0.12098	-1.2255	-2.17913	-1.47566	-2.40297	-1.02152	-0.54204	-1.12394	-0.55485
CNOT11	5.14892	5.08987	5.12458	4.68776	5.5582	5.34951	5.41509	5.25317	4.7588	4.12101	4.68034	4.04671	5.36672	5.13748	5.13785	4.85111
RNF149	4.89677	4.85944	4.66498	5.13877	4.52666	4.23087	4.55181	4.27362	3.0706	3.35785	2.97223	3.3465	4.52486	4.26628	4.14489	4.09927
SNORD89	4.80803	4.52508	4.554	4.43204	3.08494	3.382	2.82587	2.52815	0.75076	-0.40259	0.59507	1.4231	3.11	2.12158	2.21723	1.77479
CREG2	-1.60844	-1.26267	-0.93132	-1.52842	-2.09731	-3.32193	-2.83106	-3.14674	-2.61629	-0.97646	-2.67831	-1.86283	-3.32193	-3.32193	-3.32193	-3.32193
IL1R2	-2.73205	-2.04848	-2.94429	-2.92267	3.16522	3.51252	3.33232	2.97826	-2.6555	-2.02253	-2.98663	-2.18631	-3.32193	-3.32193	-3.32193	-3.32193
IL1R1	1.80152	2.0346	2.08886	3.12168	4.56306	3.30166	5.22189	2.93963	2.00822	1.7506	2.66004	3.1434	-2.8067	-2.80824	-2.81759	-2.80821
IL1R1-AS1	-0.1318	1.61568	1.45825	2.31418	3.82426	0.41417	5.24907	0.84124	2.33895	1.29339	1.50394	2.64695	-1.94802	-3.32193	-3.32193	-3.32193
IL18R1	-0.43077	-1.39529	-1.8944	-1.72465	2.12037	-0.76923	2.23064	-0.81973	-1.07997	-2.07373	-1.09109	-0.93634	-3.32193	-3.32193	-3.32193	-3.32193
SLC9A2	-2.98919	-1.58675	-2.93532	-1.754	2.40147	-1.01915	3.096	-1.40477	-2.64113	-3.32193	-3.32193	-3.15463	-3.32193	-3.18279	-3.32193	-3.32193
MFSD9	2.58507	2.42926	2.28675	2.50755	3.70874	2.89712	3.94444	3.04236	1.43451	0.66993	1.38068	0.88925	2.62215	1.85203	3.04644	2.44408
TMEM182	-0.14338	0.00573	-0.442	0.72186	0.38442	0.02534	0.52257	-0.21713	-1.1309	-0.78394	-1.21395	-1.00019	0.75911	0.82911	0.23155	0.44364
MRPS9	5.13409	4.95361	4.65603	4.81704	4.32673	4.69118	4.58324	4.5082	4.03144	3.73862	3.92339	3.87396	4.48678	4.52195	3.70738	3.63229
TGFBRAP1	2.95445	2.72857	2.35249	2.67762	3.53192	2.16275	3.5669	2.44209	2.82796	2.53497	2.54693	2.91169	3.55637	3.38009	3.74041	3.62902
C2orf49	2.13663	2.4079	2.0796	2.37744	2.30429	2.46327	2.85018	2.25862	1.92201	2.08004	2.05793	2.69789	2.00997	2.68439	2.02805	2.10751
FHL2	2.46552	2.61385	2.6304	2.05366	1.36033	0.25042	1.86493	-0.30307	4.11688	4.08916	4.18524	4.19549	3.23201	3.34457	3.03897	3.03033
NCK2	3.65369	3.93898	3.97754	3.46528	4.23243	4.11844	3.90234	3.97756	4.52924	4.27787	4.40475	4.09208	3.86581	4.04554	4.90034	4.75766
UXS1	3.88302	3.74116	3.58824	3.90013	3.78023	3.56055	3.92521	3.52802	2.89428	2.43197	2.85216	2.41068	3.39797	3.09957	2.95214	2.73017
RGPD3	0.18969	0.51862	0.02381	0.54999	0.14857	-0.27171	0.25796	-0.44483	0.61026	0.87704	0.3501	2.12073	-0.48075	0.19077	0.0423	0.36136
EEF1A1P12	2.77534	2.78566	3.07624	-0.03875	1.34988	1.7253	1.7584	0.91315	2.41354	3.97493	2.4373	2.54586	0.78764	0.66486	0.56199	1.19819
RGPD4	0.25548	0.3914	-0.13963	0.07089	0.17833	-0.42223	0.67649	-0.68099	0.55618	1.01264	0.40198	2.02108	-0.93549	-0.82186	-0.06737	0.43032
SULT1C2	2.57049	2.98366	3.01254	3.66395	-2.51815	0.68187	-2.64044	0.79713	-3.17474	-2.98015	-2.95771	-2.55571	-3.12685	-2.26393	-3.32193	-2.20271
SULT1C4	-2.97959	-3.32193	-3.32193	-3.32193	1.37347	4.05932	1.71142	4.19036	-3.32193	-3.32193	-2.96868	-3.32193	4.24664	5.42977	5.22124	4.72344
GCC2	1.94322	2.01029	2.15972	3.75132	3.42947	2.34162	4.47789	2.62794	2.03343	2.17558	2.14868	3.5333	2.47188	2.22926	2.73572	2.52239
GCC2-AS1	2.19165	1.96935	2.00458	3.01268	3.41986	2.71853	4.1669	2.99211	1.80208	1.97834	1.97226	2.78958	2.33527	2.45518	2.52537	2.02902
LIMS1	4.2484	4.17548	4.49933	4.35932	4.14834	2.8498	4.59632	2.82958	3.65833	3.04567	3.22011	3.44665	4.69472	3.87036	5.06949	3.67692
RANBP2	3.81628	3.99206	3.85157	4.28018	4.57058	3.19701	5.22727	3.43803	3.8553	3.6927	3.72859	4.77118	4.34343	4.20365	4.53717	4.7439
CCDC138	2.13698	1.52632	1.84403	1.85444	1.55196	0.59268	2.10845	0.91366	1.07577	-0.42347	0.24123	-0.17292	2.13788	0.92444	1.53598	0.85745
SH3RF3-AS1	0.3892	0.79017	1.25881	-0.76448	-3.32193	-3.32193	-3.32193	-3.32193	2.2363	2.30817	2.71505	1.0829	-3.32193	-2.22893	-3.32193	-3.32193
SH3RF3	2.36987	2.34165	2.67234	1.529	-3.24222	-3.32193	-3.32193	-3.32193	3.34678	2.93203	3.23853	2.59608	-3.32193	-3.04834	-2.9938	-2.5972
SOWAHC	3.82774	4.14584	4.30056	3.84441	4.5511	4.14362	4.65848	3.8452	4.23661	3.45467	3.85613	3.33865	3.49688	2.85387	3.35553	2.72364
RGPD5	2.41236	2.49966	2.62866	3.12649	2.05339	1.4805	2.54458	1.91321	2.71083	2.83551	2.46975	3.89153	2.52552	2.86576	2.53816	2.89662
LIMS3	2.83857	3.01982	2.89298	2.32517	1.26666	-0.93105	1.07839	-0.58657	2.15684	1.86219	1.89696	2.26989	1.60322	0.85137	2.49351	1.20936
GPAA1P1	1.74468	1.90657	2.51841	0.65384	-3.32193	-2.43997	-1.58447	-3.32193	1.88177	2.25172	1.82961	1.39447	0.35115	1.56725	0.5391	1.84725
ZBTB45P1	2.83517	2.6875	2.50013	1.90064	-1.26207	-1.24	-1.51959	-0.44612	3.09195	3.71766	2.89614	2.88298	3.42051	4.22821	4.56459	4.4214
LINC01123	2.08597	1.85448	1.71424	1.25678	-1.88863	-1.56425	-1.97763	-1.29274	1.92453	2.76081	1.85349	2.18383	2.51809	3.50969	3.66348	3.44061
MALL	-3.32193	-3.32193	-2.95418	-1.80782	-2.29279	-3.32193	-2.22178	-3.32193	-2.22471	-0.00289	-1.98666	0.80692	1.7907	2.16994	2.16952	2.34322
NPHP1	-0.76549	-1.33561	-0.93758	-0.75772	-1.86914	-1.64321	-1.4547	-1.11718	-1.44057	-1.25801	-1.13472	-0.72108	-0.18356	-0.38754	-1.18387	-1.33577
MTLN	2.50148	2.06261	1.77295	1.70171	3.1445	4.50779	2.78281	4.28531	3.77055	3.8557	3.61959	2.51906	4.67111	4.56157	3.67187	3.81339
LINC01106	2.20305	1.7644	2.0394	1.51347	-1.84561	-2.43546	-2.29008	-2.10091	1.58225	2.35563	1.43752	1.96082	2.56815	3.59843	3.58982	3.22585
ZBTB45P2	2.75294	2.47568	2.39643	1.63589	-1.31682	-1.48894	-1.5709	-0.93769	2.91159	3.58828	2.75904	2.81153	3.23772	4.18154	4.46425	4.35626
GPAA1P2	1.76587	2.24216	2.34797	2.07454	-3.32193	-2.79674	-2.17382	-3.32193	2.70295	3.09052	2.94555	2.65926	1.69169	2.12827	1.58623	2.32667
LIMS4	2.98974	3.17084	3.04258	2.50201	1.40245	-0.83379	1.19221	-0.5595	2.25228	1.98898	2.03548	2.3788	1.7187	0.92873	2.61073	1.32366
RGPD6	2.5061	2.57485	2.69835	3.19455	2.1414	1.55097	2.63275	1.99681	2.77494	2.92095	2.55466	3.97784	2.60197	2.94018	2.62368	2.98535
BUB1	4.19934	3.8933	3.75641	3.59542	4.47489	3.07483	4.92781	3.08256	4.71548	3.82728	4.0381	3.65843	4.56175	4.36178	4.61164	4.18136

BCL2L11	1.23254	0.84872	0.6704	1.38758	1.08737	0.34121	1.49026	0.33874	1.04592	0.57904	0.73973	2.22838	2.40499	2.09039	3.05933	3.27814
MIR4435-2HG	3.33604	2.85867	2.97581	2.18566	2.11075	1.70369	2.09061	1.8727	3.36449	3.33283	3.01157	2.95294	3.33224	3.24601	2.82965	2.00741
ANAPC1	2.79673	2.69154	2.43447	2.59637	3.98778	2.6116	4.32399	2.6928	3.57847	2.88213	3.25149	3.44535	3.33249	3.17233	3.51004	3.39623
MERTK	-2.91021	-1.63301	-2.33776	-1.07329	2.96746	2.80642	3.36083	2.63877	1.36517	1.06382	1.39604	1.54488	1.24908	1.16342	1.21599	1.56969
TMEM87B	2.81562	3.22725	3.54394	3.95049	3.56023	3.26459	4.49439	3.08367	3.41123	3.32138	3.34777	4.42369	3.51564	2.81071	3.36951	3.17078
FBLN7	-1.42906	-0.96993	-0.94523	-1.08202	-3.18414	-2.88057	-3.32193	-3.13247	-3.32193	-3.16647	-3.32193	-3.16896	-2.86803	-3.19482	-2.577	-2.14144
ZC3H8	1.69589	1.47475	1.13076	1.54625	0.52953	0.8671	1.10611	1.25932	0.00307	0.59082	0.19868	0.43142	1.54095	1.18043	0.58824	0.72177
ZC3H6	-0.49099	0.08334	0.13377	1.12669	-2.29811	-2.0612	-1.84684	-1.64841	-1.00738	-0.42127	-1.32292	0.47547	-1.9451	-1.57293	-1.53598	-1.2708
RGPD8	2.53963	2.67236	2.69957	3.07201	2.58088	1.59871	3.0487	1.46872	3.20039	3.39993	2.96246	4.34225	2.86195	3.15642	2.88674	3.32338
TTL	2.37197	2.67589	2.61767	2.74852	2.70251	1.67246	3.21879	1.64244	1.82166	1.99174	2.57331	2.56168	2.43265	2.59512	3.2503	2.61076
POLR1B	4.14146	3.75848	3.41244	3.63849	3.51971	2.61197	3.95061	2.58669	3.34124	2.90965	3.21288	3.18738	3.42942	2.5934	3.32434	2.75822
CHCHD5	3.06686	3.15857	3.12272	2.79806	3.35693	4.38921	2.34813	4.35462	3.41733	3.70503	3.74458	2.86073	2.32692	2.82355	2.32437	2.38899
SLC20A1	6.24291	5.86658	5.96511	6.57576	4.89923	4.49278	5.20458	4.57207	6.35696	5.89144	6.38146	7.00153	5.6442	5.03991	4.68688	5.3767
NT5DC4	1.32564	0.66404	1.39205	0.68468	0.95695	-0.68955	1.49018	-0.58191	3.2504	1.63754	2.80897	1.5721	1.95789	0.47572	1.55642	-0.08538
CKAP2L	3.4359	2.95009	3.07007	2.64994	2.8828	1.13091	3.2306	1.03256	5.18311	3.53971	4.58088	3.6249	3.9259	2.35475	3.39331	2.15028
IL1A	6.98417	7.18845	6.58348	7.53054	-1.58734	-2.24581	-2.30525	-2.11788	6.37517	5.77295	6.03033	6.21326	-2.2209	-1.48712	-2.89018	-2.88195
IL36G	0.47875	2.19593	1.02308	2.77502	-3.32193	-3.32193	-3.32193	-3.32193	-3.32193	-2.69682	-3.32193	-3.32193	-3.32193	-3.32193	-3.32193	-3.32193
IL36B	5.38229	5.60762	5.99125	6.36738	-3.0876	-2.81618	-2.84931	-3.32193	-2.75372	-3.32193	-3.32193	-3.32193	-2.91558	-3.32193	-2.4796	-2.83111
IL36RN	3.83655	4.51152	5.04512	4.9411	-3.18454	-3.32193	-3.32193	-3.32193	-3.32193	-2.78218	-3.00727	-3.32193	-3.07788	-3.32193	-2.78664	-2.77679
IL1RN	2.02037	3.71766	3.22256	4.17749	-2.46603	-3.01042	-2.0985	-2.43398	-3.32193	-3.32193	-3.32193	-2.78395	-2.68017	-3.07573	-2.57111	-2.7708
PSD4	1.72191	2.29912	2.1584	1.79085	-1.36083	-0.58583	-1.52317	-0.89026	-0.14345	1.01099	0.87074	1.22908	1.63601	1.02405	2.47156	2.66773
PAX8-AS1	3.71625	3.87074	3.94867	3.94248	-3.09328	-3.32193	-3.15095	-3.04244	-2.29018	-1.89448	-1.85272	-1.61818	-0.27735	-0.81989	-1.00306	-0.23487
PAX8	2.6622	2.64541	2.85444	2.49765	-2.54566	-1.45361	-2.41035	-1.51666	-1.95186	-1.13931	-1.12486	-1.40203	-0.01829	-0.53639	-0.11855	0.11444
CBWD2	2.9618	2.88432	2.66999	2.96598	2.09003	2.55713	2.1422	2.57271	2.49954	2.45523	2.3117	2.16119	2.90992	2.77701	2.26161	2.42685
WASH2P	3.42662	3.1642	3.33761	2.872	2.74829	3.63334	1.73678	3.70956	3.26991	3.6465	3.41227	3.0929	2.60934	2.78663	2.38138	2.68929
DDX11L2	1.89087	1.37433	1.58205	1.42927	-0.30875	-0.00274	-1.47751	0.03484	0.9792	0.85846	0.99847	0.50155	-1.47009	-1.65885	-1.25833	-3.32193
RPL23AP7	3.24941	3.43633	3.56189	2.9868	2.32042	2.44546	2.2828	2.12143	3.37873	3.05216	3.06598	2.33464	2.87361	2.47677	2	1.66032
RABL2A	0.21324	0.29977	0.53285	0.15197	0.70549	0.73755	0.56037	0.56837	0.93875	1.10795	1.46628	1.42437	0.21741	0.16159	0.69335	0.27424
SLC35F5	2.65598	2.63951	2.55278	2.95836	4.46423	3.42284	4.86622	3.25336	3.71436	3.8014	3.6235	4.45942	4.78437	4.72434	4.85618	4.6048
DDX18	4.61807	4.73797	4.47705	5.04696	4.27489	4.29575	4.99333	4.41992	4.43212	4.63499	4.68094	4.67119	4.58961	4.50924	4.63721	3.68082
HTR5BP	1.69353	1.87042	1.85266	1.98948	1.38033	0.99611	1.75517	0.25597	0.71762	1.30187	1.4012	1.44158	0.78926	1.17269	0.41801	0.48199
CCDC93	3.74307	3.71083	3.73854	3.95798	2.97102	2.6748	3.3589	2.63244	2.96062	2.99947	3.00995	3.60304	2.86191	3.19177	2.8812	2.85356
INSIG2	3.35596	4.19158	4.23029	4.19037	2.19572	2.55608	2.67602	2.12986	3.35298	3.7924	2.9811	3.35778	1.36643	2.38489	1.54297	1.8819
EN1	-2.09257	-2.94137	-2.61069	-2.08438	-3.1659	-2.97494	-3.32193	-2.92154	-0.34429	-0.32161	-0.09577	-1.84447	-3.32193	-2.28654	-2.72403	-2.98572
STEAP3	5.75933	6.07898	6.52148	6.09222	3.86589	3.19613	3.38818	3.3415	5.4915	5.65108	5.77066	5.84426	4.7857	4.01285	5.11405	5.20712
STEAP3-AS1	3.27425	3.63738	4.2491	3.52576	2.22609	0.50263	1.64003	-0.01555	3.65891	3.89641	4.04571	3.95089	2.90819	2.20745	3.25762	3.48961
C2orf76	1.91074	2.21977	2.39716	2.72665	2.38728	3.08137	2.80314	2.76106	1.4137	2.04012	1.43132	1.6733	2.01002	2.95814	1.84575	1.71381
DBI	6.41641	6.47658	6.26722	6.31482	6.22978	7.05043	5.9429	7.00611	6.10684	5.86238	5.6883	4.83665	6.30787	6.84543	5.2193	5.36402
TMEM37	-3.32193	-1.7857	-2.47205	-1.04475	2.368	3.84568	1.87647	3.48232	-1.95769	-1.12285	-1.86043	-0.04881	2.34692	2.60267	3.71823	3.1468
TMEM177	3.33863	2.66155	2.64305	2.40234	3.3559	3.51603	2.80453	3.54752	3.11107	2.5638	3.12707	1.96701	3.30015	3.02738	2.90992	2.66301
PTPN4	0.18658	0.33928	-0.29633	0.62653	1.17767	0.86554	1.91119	0.92339	0.5935	1.14006	0.68419	2.17428	0.81605	1.61122	1.11058	1.57006
EPB41L5	-0.06949	0.10588	0.50351	1.03774	3.41254	2.46459	3.77998	2.52095	1.04508	1.17704	1.08303	2.10754	1.25089	1.31193	1.91253	2.08041
TMEM185B	4.84248	4.84168	4.70699	4.66343	5.55027	4.87289	5.70173	4.56735	4.85867	4.59858	5.08184	4.89522	4.80025	4.8445	4.98995	4.96177
RALB	4.96585	5.20972	5.19857	5.24334	5.25193	4.81233	5.91513	4.31874	5.91171	5.83405	5.82943	6.33211	5.6102	5.8878	5.31217	5.60316
INHBB	-3.32193	-2.97496	-3.32193	-3.32193	4.41995	2.14895	4.62253	3.00418	0.32701	-1.0084	-0.99567	-1.81751	0.8082	0.80335	1.19982	0.53348
GLI2	-3.17694	-2.64989	-2.8617	-3.14119	-0.78477	-3.04241	-1.37261	-3.32193	2.22767	2.04509	2.81186	1.19215	2.90621	1.76958	3.68921	2.51414
TFCP2L1	-2.45156	-2.68251	-2.84431	-3.04801	-1.82169	-2.65644	-1.34467	-2.94842	-1.7267	-2.08464	-1.88057	-1.87454	-2.43955	-2.60255	-2.14239	-2.02641
CLASP1	2.52434	2.61583	2.63187	2.97723	2.79468	2.31758	3.16178	2.25118	3.05228	2.92658	2.91079	3.85728	2.45866	2.56204	3.23463	3.21884
NIFK-AS1	4.11438	4.06732	3.72072	4.07431	3.17863	3.5892	3.56077	3.63268	3.75141	3.82899	3.52473	3.99667	3.53463	3.47201	2.45254	2.26033
NIFK	5.35474	5.35518	5.03524	5.34555	4.57337	4.70462	4.84593	4.72627	5.10839	5.14547	4.99127	5.06373	4.74613	4.6386	3.66301	3.42591

TSN	4.13651	4.19418	3.85762	4.47386	5.24873	4.48155	5.66389	4.55701	4.94224	4.81965	4.78626	4.82604	4.6949	5.00349	4.47863	4.53165
YWHAZP2	1.62531	1.31799	0.69211	0.23472	0.35301	-0.91278	0.75892	-0.44612	1.43419	1.76117	1.45974	1.55082	0.24583	0.02213	0.60881	0.81933
GYPC	6.15769	5.95813	5.81394	5.19763	-3.18223	-3.00921	-3.32193	-2.96045	3.85938	4.75579	4.40293	4.04313	-0.43896	0.45501	0.70609	1.01126
BIN1	4.01756	3.99466	3.74808	3.8175	5.78491	5.01637	5.35799	5.03945	4.7352	5.03017	5.06438	4.65669	4.34843	4.69068	4.54728	5.10344
CYP27C1	-1.87399	-1.76626	-2.16589	-1.98442	-1.03209	-2.71388	-0.58944	-2.45625	0.60996	-0.44761	1.08611	0.39214	2.28265	1.17538	2.48398	1.38561
ERCC3	3.96245	3.82791	3.76208	3.91639	3.46038	3.34211	3.43153	3.52517	3.10366	2.85181	3.12347	3.24559	3.8549	3.7294	3.33086	3.40734
MAP3K2	2.37813	2.54169	2.69688	3.20318	2.93281	1.86624	3.57715	2.05064	2.13357	1.78356	2.22101	2.90759	3.50513	2.64125	3.70443	3.14299
PROC	-3.32193	-3.32193	-2.8693	-3.32193	1.99426	4.01259	1.69419	4.16161	-2.26207	-2.94346	-3.32193	-3.32193	-3.32193	-2.53241	-2.40279	-2.63745
IWS1	5.02199	4.7532	4.67318	4.97944	4.43884	4.26713	4.64248	4.31932	4.88002	4.15018	4.61649	4.59234	5.01429	4.90052	4.8333	4.77816
MYO7B	-1.56436	-0.11775	-0.17845	-0.51205	-0.34838	-1.24804	-0.67373	-1.59605	-2.93507	-2.89949	-2.3856	-2.9057	-3.32193	-2.97098	-2.90312	-3.20282
LIMS2	-2.19177	-0.6816	-0.27599	-0.21435	-1.09245	-1.90817	-1.38951	-1.92602	-2.27722	-1.64179	-1.78852	-2.23311	0.44458	0.18285	2.11297	0.90758
WDR33	3.14416	3.01263	3.07353	2.93861	3.32323	3.07026	3.3923	3.25306	3.21061	2.9972	3.27069	3.0732	3.40725	3.6648	3.40669	3.52125
SFT2D3	1.55916	2.00659	2.02273	2.12957	2.52088	3.02622	2.81241	3.20477	1.47562	1.86659	1.63863	2.07625	2.64844	3.18117	2.89156	2.84747
POLR2D	4.11127	4.02109	3.92896	3.68565	3.99438	3.76702	4.145	4.02818	4.16585	3.70238	4.00625	3.96485	4.86322	5.06561	4.72337	4.66617
RNU6-395P	4.76506	5.24426	4.96938	4.93558	4.96331	5.40248	5.36133	6.21187	5.16369	4.67343	4.96341	5.47648	3.96233	4.62514	3.95227	4.60843
AMMECR1L	3.19079	3.148	3.01023	3.3098	3.20121	2.78464	3.42444	3.05677	3.20977	3.53102	3.254	4.00575	4.14244	4.31662	4.01176	4.14652
SAP130	3.9986	4.04913	3.98055	4.16646	3.53636	3.22918	3.52745	3.14739	3.77556	3.30589	3.65982	3.86322	3.61007	3.47258	4.40921	4.34672
UGGT1	4.76563	4.69777	4.81334	4.4926	5.31145	5.33544	5.66033	5.36347	4.85335	4.21141	4.78891	5.209	4.92154	4.31976	5.4349	5.06902
HS6ST1	3.84992	3.51456	3.42782	2.63926	3.79219	3.06368	2.78479	3.13637	3.64232	2.91291	3.48427	2.13911	3.60277	3.38008	3.78525	3.82154
FAR2P1	-3.06827	-3.04007	-3.32193	-3.32193	-0.473	0.69241	-1.15234	0.41646	-3.32193	-3.32193	-3.32193	-3.32193	-3.32193	-3.32193	-3.32193	-3.32193
POTEF	-0.38868	-0.7958	-0.95716	-1.80478	0.34127	-0.2417	0.13529	0.36103	-0.5143	-0.46925	-1.17189	-2.28712	-3.12726	-2.95692	-3.08798	-2.87844
MED15P9	-3.32193	-3.32193	-2.82797	-3.32193	-0.21572	1.17658	-0.62842	1.37877	-2.83589	-2.5867	-2.88149	-2.33633	-1.23787	-1.66553	-2.11019	-1.43809
CCDC74B	-2.5633	-2.08375	-2.30428	-1.65127	-0.03772	1.31958	-0.58835	1.26557	-0.0521	0.20265	-0.00588	-0.04533	0.14114	0.84908	0.3663	0.90486
MZT2B	6.93493	6.97114	7.18634	6.10361	7.68453	8.50611	6.81227	8.48303	8.12612	8.34936	8.08677	6.51526	7.05488	7.14605	6.83915	6.72691
NOC2LP1	-0.9248	-0.71094	-1.72626	-2.54094	5.14839	4.14837	4.37026	4.10905	-1.91405	-0.89177	-2.6534	-2.26616	-3.32193	-3.03257	-2.97525	-2.68476
CCDC115	2.3409	2.09391	2.34597	2.58656	3.23392	3.19321	2.69146	3.18693	3.40537	3.36334	3.57531	2.76389	2.96067	3.0479	2.88529	2.72432
IMP4	5.2986	4.99911	4.64307	4.51651	5.69848	5.75921	5.32568	5.75656	5.21829	5.0348	5.26442	4.00621	5.28823	5.03917	4.83795	4.7174
PTPN18	0.56997	-0.30563	-0.059	-0.93176	3.39328	3.59698	2.46146	3.47242	3.76046	3.13067	3.46893	3.01152	1.4887	1.05151	1.65146	1.59073
FAR2P2	-3.32193	-3.32193	-3.32193	-3.32193	-1.38581	-0.70834	-1.1483	-0.54555	-1.43795	-1.39954	-1.83718	-1.1749	-3.32193	-3.32193	-3.32193	-3.32193
POTEI	-0.94357	-1.37599	-0.98317	-2.96689	-0.92384	-1.6283	-1.70693	-1.36151	-1.12133	-0.98579	-1.65509	-2.36373	-2.71836	-2.64036	-2.43902	-3.04016
POTEJ	1.0833	1.04054	1.31719	-1.06944	0.34394	0.19385	-0.56022	-0.57125	1.05195	1.26349	1.08415	0.51265	-0.60566	-0.57112	-0.06757	-1.12592
ARHGEF4	1.70154	2.27165	2.49054	2.09232	1.42801	-0.80264	0.94842	-0.60651	0.7785	1.74784	1.26965	2.10034	-2.3407	-2.56903	-2.38999	-1.95999
FAM168B	5.07838	5.07308	4.96793	4.96749	5.35331	4.50435	5.81671	4.54129	4.88326	5.18525	5.28399	5.35505	5.84752	6.63537	6.55809	6.56964
PLEKHB2	5.33608	5.36941	4.99311	5.61346	5.30985	4.68703	5.94273	4.47364	5.56657	5.27549	5.18226	5.53612	6.46087	6.5826	6.2468	6.52169
POTEE	0.86539	1.02332	1.21124	-1.68802	1.08996	0.77388	0.44529	0.21041	0.88217	1.08231	0.99912	0.31133	-0.5871	-0.65572	-0.07632	-1.06065
FAR2P4	-3.32193	-3.03275	-3.01828	-3.32193	0.64271	1.59404	-0.28657	1.25178	-3.32193	-3.07014	-3.32193	-3.32193	-3.32193	-3.32193	-3.32193	-3.32193
KLF2P4	-3.32193	-3.32193	-3.32193	-3.32193	1.15428	1.14661	-0.20287	1.80367	-3.32193	-2.63402	-3.32193	-3.32193	-3.32193	-3.32193	-3.32193	-3.32193
NOC2LP2	-0.09172	0.17715	-0.14548	-1.99309	2.93138	1.78675	1.78827	2.04834	-1.40188	-1.59514	-2.45232	-1.78967	-2.92931	-3.32193	-3.32193	-2.84726
MZT2A	4.74103	4.95236	5.07307	3.94002	5.08594	5.8281	4.28666	5.77056	6.03768	6.23995	6.02155	4.7165	5.1205	5.14464	4.97611	4.85357
TUBA3D	-0.55042	-0.63233	0.82266	-0.15672	-0.85257	-0.88376	-1.26067	-0.04914	-0.16755	-1.58134	-1.31516	0.02977	-0.42112	0.01427	-0.17088	-0.48167
CCDC74A	-1.99229	-1.61491	-1.32617	-0.57099	1.62202	2.33557	0.84415	1.87582	2.32927	2.82388	2.88561	2.48534	1.23432	2.2589	1.43341	1.89466
POTEKP	0.70514	0.3775	0.4496	-2.53199	0.09593	0.58619	-0.9126	0.9668	-1.19888	-0.80257	-0.23824	0.00472	-2.57466	-2.23312	-0.68091	-1.894
C2orf27A	0.71595	0.60961	0.23267	1.18677	-0.93261	-0.0645	-0.41626	-0.22519	0.62428	0.16456	-0.03308	0.38326	-0.03395	-0.11029	-1.595	-0.80526
GPR39	4.98221	4.85842	4.85994	4.64424	4.0779	3.50881	4.14597	3.81016	3.44456	3.81631	3.49881	3.8054	6.14448	5.70669	6.3477	5.79705
LYPD1	5.06333	4.99468	4.97259	4.69682	3.93126	3.63956	3.90378	4.00536	3.34498	3.79782	3.40601	3.65612	6.81676	6.28157	7.1214	6.43016
NCKAP5	-3.32193	-3.08022	-3.18934	-2.63	-3.32193	-3.32193	-3.32193	-3.32193	-2.67423	-2.10038	-2.50836	-1.93371	2.30758	2.65029	2.7808	2.73667
MGAT5	3.97504	3.59494	3.59503	3.40244	3.83236	2.29357	4.03116	2.06674	3.91317	3.41844	3.80253	3.79353	3.74162	3.33141	3.99405	3.82608
CCNT2-AS1	-0.52073	0.42703	0.15111	-0.64761	1.66852	0.99792	1.11763	0.86752	0.25155	-0.11649	-0.477	-0.42117	0.82409	1.35807	0.85599	0.59922
ACMSD	-1.90397	-2.36708	-1.45749	-2.55333	1.16495	0.86296	1.10771	0.81338	-1.3528	-2.46898	-1.87189	-2.98372	-0.53643	-0.25212	-1.34859	-0.46297
CCNT2	2.11741	2.07235	2.1843	2.80396	2.01635	2.15269	2.63111	2.36804	2.0122	2.31585	2.02064	2.94938	2.95104	3.05189	3.02314	3.06873

RAB3GAP1	3.39829	3.40595	3.25279	3.6575	3.73301	3.30074	4.18223	3.11043	4.11283	3.89078	3.93977	4.58207	4.02909	3.96102	4.01762	4.14993
ZRANB3	0.88404	1.10111	0.85185	0.90581	0.95656	0.56311	1.06144	0.36168	1.33051	0.90575	1.13127	1.04932	1.31118	1.0245	1.45099	1.20806
R3HDM1	2.86072	2.3986	2.25	2.36429	3.17025	2.25691	3.42744	2.03468	3.03935	2.16891	2.42578	2.97453	3.62356	2.53597	3.4841	2.75395
UBXN4	5.13757	5.18911	5.34619	5.92962	5.34341	5.05359	5.84574	5.05939	4.63468	4.37759	4.58251	5.15642	5.05834	4.43608	4.78723	4.38451
MCM6	4.17783	3.39629	3.68114	3.48072	5.34292	2.60941	5.27222	2.97795	5.20312	3.22787	4.34534	2.82496	5.66435	3.49966	5.55492	3.18196
DARS-AS1	2.17658	2.514	2.56012	1.89185	2.10664	0.88352	2.21594	0.47866	2.69184	2.91297	2.85332	2.66483	1.98944	2.06298	2.38816	2.17933
CXCR4	-3.32193	-1.02417	-0.96174	-1.14421	-2.65032	-2.88594	-1.92756	-3.32193	-0.01101	-0.03661	-1.23323	-0.0098	-2.69265	-1.32314	-2.32225	-2.57252
HNMT	0.33186	0.96343	0.31111	1.54711	2.98636	2.54642	3.09405	2.68427	-3.07135	-2.76407	-3.32193	-3.32193	3.12591	3.62404	2.20249	2.99977
SPOPL	1.76037	1.6022	1.37963	1.90131	2.52143	1.23671	3.30262	1.14554	2.90001	2.67686	2.40151	3.4974	3.50375	3.00443	2.79959	2.53088
LRP1B	-2.92267	-3.32193	-3.32193	-3.16489	-2.9983	-3.32193	-2.94607	-3.32193	-3.04482	-2.84137	-2.91019	-1.54878	-1.90126	-0.99488	-1.20144	0.15498
KYNU	4.27022	4.54278	4.63019	5.68132	2.82186	1.94808	3.41724	1.30801	0.43915	1.54772	0.48273	2.44319	0.62028	0.74557	-0.94058	0.8513
GTDC1	-0.12569	0.08349	-0.02474	0.35176	-0.35054	-0.38662	0.25049	-0.39906	0.47833	0.24931	0.15863	0.85953	0.6092	0.66404	0.66061	0.45715
ZEB2	0.31478	0.27429	0.45849	1.02185	-3.14304	-2.82773	-3.20152	-2.52335	1.79827	1.69114	1.91975	2.37865	-0.86762	-0.56175	-0.0315	-0.47032
ZEB2-AS1	-2.42057	-2.60002	-2.30178	-1.82322	-3.32193	-3.32193	-3.32193	-3.32193	0.65943	0.49676	0.08262	0.12065	-1.951	-2.57566	-2.97009	-2.05202
TEX41	-1.40229	-1.33993	-0.92292	-0.83142	-3.29069	-3.17889	-3.32193	-3.32193	-2.14681	-2.2222	-2.51662	-1.88355	-3.05661	-2.87607	-3.32193	-3.18364
ACVR2A	1.05887	0.81549	1.07732	1.73436	1.17435	1.02553	1.87321	1.00646	0.96512	0.9688	0.85611	1.41404	0.90943	0.72092	1.0421	1.43652
ORC4	2.56874	2.66538	2.54717	3.01219	2.64868	2.60328	3.22963	2.71172	2.37009	2.56708	2.23251	2.92168	3.26622	3.76305	3.32783	3.06751
MBD5	-0.78598	-0.73027	-0.64772	-0.01059	-0.70445	-0.42077	-0.32932	-0.33525	-1.22671	-1.00571	-1.0653	-0.78018	-0.19333	0.35779	0.43565	0.42183
EPC2	1.22561	1.74245	0.98393	2.16611	1.50587	0.8822	2.18639	0.87691	2.02705	1.86908	1.7831	2.8383	3.07976	2.93013	2.82967	2.88484
KIF5C	-2.99692	-3.0721	-2.64504	-3.17615	1.23043	1.35191	1.79279	1.38096	1.28094	0.05951	1.00148	0.45366	-2.48052	-2.71219	-1.30198	-2.91184
LYPD6B	-3.32193	-3.32193	-3.32193	-3.32193	2.57233	1.838	2.59826	1.89646	-1.66518	-0.5241	-0.81335	-0.12114	-3.32193	-3.32193	-3.32193	-3.05788
LYPD6	-3.11503	-3.09162	-3.07985	-3.06532	1.51792	0.77054	1.87569	0.59674	0.86452	0.47892	0.74975	0.70926	-3.15739	-2.64215	-2.19904	-2.64211
MMADHC	6.60701	6.9037	6.69474	7.23991	5.96205	5.69037	6.43463	5.54661	6.02125	6.15693	6.01863	6.15158	5.74205	5.87926	5.559	5.57488
RND3	6.15755	6.50423	6.52312	7.1676	1.80843	0.05501	2.47055	-0.3337	4.70901	5.10246	4.19808	5.67916	4.45	4.42689	4.41802	3.96052
RBM43	0.63034	0.90527	0.64338	1.2911	-0.52704	-0.51686	-0.31026	-0.96868	1.1892	1.50836	1.40037	2.2283	2.05226	2.01091	1.95241	2.0904
NMI	4.07651	4.05279	3.84743	4.31212	0.99594	1.86403	1.74313	2.06526	3.82013	3.68163	3.77453	4.48849	4.26676	4.47657	3.9257	4.0319
TNFAIP6	-2.01474	-2.24922	-3.32193	0.35497	-3.32193	-3.32193	-2.77713	-3.32193	-3.32193	-2.76221	-3.32193	-2.77008	3.23555	4.02612	2.78757	2.91357
RIF1	2.22651	2.50269	2.30122	2.83452	2.3897	1.26706	3.32053	1.58329	2.41527	2.66578	2.49714	3.41836	2.90375	3.69233	3.82505	3.72062
NEB	-2.48429	-2.49861	-2.53789	-2.42241	0.78577	-0.6575	0.35113	-0.38191	-2.79316	-3.18713	-3.07811	-2.73171	-2.17566	-2.12761	-2.39825	-2.02216
ARL5A	3.3208	3.15287	3.11156	3.46677	3.5626	2.78665	4.18422	2.61038	3.19221	2.91106	2.94073	3.33972	3.7235	3.10703	3.98592	3.46602
CACNB4	-3.26147	-3.18958	-3.25057	-3.2095	-3.29594	-3.26081	-3.29339	-3.28567	-2.81314	-2.29918	-3.03305	-2.58255	-3.01776	-2.96129	-3.1816	-2.88362
STAM2	2.0966	2.55646	2.57074	3.05246	2.52153	2.12699	3.27453	1.93215	2.63926	2.51326	2.43368	3.31348	3.24404	3.15177	2.82192	3.06917
FMNL2	3.72074	3.90685	4.111	4.46127	3.30804	3.20544	4.06683	2.96671	4.28961	4.30997	4.33035	5.33762	6.20067	5.63441	6.37836	6.09231
PRPF40A	4.38788	4.40986	4.35029	5.07721	4.61818	4.01241	5.25491	4.11852	4.7445	4.71125	4.52406	5.30981	5.09107	5.24763	5.06834	5.10314
ARL6IP6	3.44925	3.59589	3.91559	3.68759	3.56344	2.43175	3.2883	2.22517	4.24664	3.87484	3.61728	3.13388	4.04074	4.15763	3.29149	3.33571
LINC01876	-1.73681	-1.48766	-1.89286	-1.83459	-0.38177	-0.09253	0.43688	0.95261	-3.32193	-3.32193	-3.32193	-3.32193	-3.32193	-3.32193	-3.32193	-3.32193
NR4A2	-0.67061	-0.44062	-0.41697	-0.29292	4.56263	4.9365	5.09111	4.09288	-1.82661	-1.79256	-1.30753	-0.24831	-0.13234	-1.47539	-1.39522	-1.445
GPD2	3.73583	3.84368	3.39183	4.19691	3.96878	3.28559	4.36837	3.18059	3.97122	3.32123	3.12413	3.80764	4.38741	4.20552	4.39369	4.41439
GALNT5	-3.32193	-3.32193	-3.32193	-2.80479	0.44041	-2.77559	1.26651	-3.13873	-3.32193	-3.17167	-3.32193	-3.32193	-3.32193	-3.32193	-3.32193	-3.32193
FAM133DP	1.36234	2.133	1.71966	2.4563	0.42251	1.10038	1.2027	1.99066	-0.68451	0.95698	0.88387	1.15675	1.45407	0.09015	0.35569	0.70885
ACVR1	2.89022	2.89871	2.99663	3.4436	3.56146	3.39555	4.01515	3.22207	2.6386	2.89415	2.73313	3.58865	3.53862	4.1456	3.74984	4.0966
CCDC148	-1.29629	-2.2181	-1.44666	-2.29756	-2.11743	-1.60932	-1.11033	-1.76192	-1.67012	-2.06958	-2.13743	-1.58659	0.56531	0.88164	-0.31499	-0.28733
PKP4	3.39975	2.89429	3.0574	2.7604	4.04437	2.927	3.90835	3.24953	3.24723	2.30684	2.51858	2.34767	4.48323	3.82478	4.22263	3.69485
PKP4-AS1	0.38114	0.34662	0.1399	-0.23346	1.63067	0.39581	1.90715	0.08222	0.32406	-0.68412	-0.59686	-0.61898	1.95154	1.38206	1.91009	1.21273
TANC1	1.88674	1.81715	1.75718	2.26898	2.12389	1.05089	2.19165	1.25241	2.60029	2.45689	2.56769	3.15365	2.32961	2.11686	2.41614	2.58441
BTF3L4P2	3.78483	4.08479	3.65422	3.68288	3.23819	3.8006	2.10019	3.39303	4.71001	4.85142	4.83033	4.14972	3.20759	3.02767	3.64633	3.28267
WDSUB1	3.26668	3.36464	3.43038	4.08644	2.82657	2.71564	2.98886	2.90567	2.77835	2.62396	2.27649	2.84782	3.0184	2.59239	2.46319	2.01354
BAZ2B	0.10486	0.60793	0.54913	2.01582	0.5564	0.75788	1.15727	0.74983	-0.83499	-0.19833	-0.39005	0.77174	1.33899	1.40544	1.97605	2.17423
CD302	2.62659	2.64539	2.66682	3.01908	2.46455	3.61256	2.9827	3.37638	3.04384	2.90717	2.94784	4.01035	3.02811	2.97472	3.19753	3.17632
LY75-CD302	1.46005	1.48448	1.58342	2.15466	0.76923	1.74415	1.20505	1.65029	1.18364	1.15875	0.93149	1.88015	0.76849	0.86905	0.99173	1.03121

LY75	0.17903	0.5812	0.72876	1.53367	-3.21768	-3.08512	-3.32193	-3.32193	-3.32193	-2.81063	-2.779	-2.44514	-3.32193	-3.13577	-3.32193	-3.32193
PLA2R1	-3.05631	-2.89932	-2.39736	-2.13543	-2.49598	-3.11617	-2.32867	-2.69806	-0.73859	-0.52583	-0.55398	0.35995	-0.20405	-0.5234	0.2632	0.32092
ITGB6	-1.43988	-0.82135	-0.62229	-0.14968	-1.21139	-3.32193	-1.0366	-3.32193	-2.54831	-2.19939	-1.66653	-1.86981	1.96245	1.78063	2.16441	1.72462
RBMS1	3.63187	3.41695	3.29723	3.51844	1.98067	1.88821	2.36095	1.93326	3.76425	2.81953	2.65498	3.21421	4.58637	3.54109	3.98432	3.63553
MIR4785	5.51781	5.11712	5.65772	3.89373	4.53159	2.71143	4.08501	2.94248	7.06141	6.28571	6.36988	4.02025	6.23003	4.82367	6.90543	5.30472
TANK	2.70028	2.77513	2.86182	3.62329	3.04911	3.36816	3.63196	3.14618	2.25005	2.81739	2.61107	3.22212	3.8479	3.9399	3.39593	3.44708
LINC01806	2.41257	2.41702	1.94569	2.54336	-2.96878	-2.59052	-3.32193	-2.84855	0.30868	0.53991	-0.42921	0.60772	2.55608	1.37735	1.59948	1.45662
PSMD14	5.65765	5.76601	5.66281	5.93229	4.94873	4.91512	5.33555	4.8076	4.78377	4.41305	4.43956	4.18075	4.79252	4.87937	4.4326	4.29643
DPP4	2.42858	2.80106	2.88511	3.65075	-0.9221	-2.76613	-0.33236	-2.52519	1.55857	1.77901	1.42453	2.83806	-1.35353	-0.71782	-0.84012	-0.81417
FAP	-0.74004	0.54189	-0.1717	1.90751	-3.25557	-3.32193	-3.32193	-3.32193	-2.60463	-2.80846	-2.77671	-2.71282	-0.03367	-1.13697	-1.29741	-1.81676
IFIH1	2.9078	3.02192	2.89744	3.64869	1.91694	1.25556	2.61633	1.09674	2.9617	3.15001	3.34343	4.75534	2.49377	2.58515	2.05606	2.27312
GCA	-0.11074	0.10404	0.14249	0.93987	1.47554	2.08912	1.99826	2.03771	1.79624	2.36895	1.78967	2.49628	3.35606	4.3764	2.79823	3.42845
KCNH7	-3.11274	-3.32193	-3.32193	-3.32193	-2.18913	-3.32193	-2.53262	-3.07632	-3.08147	-3.32193	-2.75153	-3.1229	-0.59441	1.72401	0.21129	1.29876
FIGN	1.11164	0.55275	0.70133	0.88309	1.2067	0.63441	1.78132	0.80196	1.2982	0.51845	1.11788	0.8732	2.41387	1.85693	2.74974	2.1734
GRB14	0.97299	1.84981	1.53487	2.85889	3.04367	3.39585	3.22618	3.65699	-0.02262	0.52704	-0.21581	0.52924	2.54473	3.66638	3.20453	3.50015
COBLL1	-0.73334	-0.75657	-1.30363	-0.92201	0.63645	-0.42642	1.12099	-0.05111	-0.25975	-1.10169	-0.56555	-0.61589	-1.59914	-1.76618	-1.02168	-1.23016
SLC38A11	-3.32193	-3.32193	-3.32193	-3.32193	0.54555	-0.3099	0.94622	0.08999	-3.32193	-3.32193	-3.32193	-3.32193	-3.32193	-3.32193	-3.32193	-3.32193
SCN2A	-3.06307	-2.75835	-2.97524	-2.90964	-2.84735	-1.22478	-2.47799	-1.37933	-2.41452	-1.92006	-1.75242	-1.2845	-3.32193	-3.32193	-3.32193	-3.32193
GALNT3	-2.00542	-3.32193	-2.19937	-1.90364	-1.31305	-2.58222	-0.57341	-2.652	-1.22676	-2.47509	-2.72287	-2.7651	-3.14592	-3.32193	-3.11013	-2.75158
TTC21B	1.50527	1.52283	1.28808	1.63343	1.88023	1.15968	2.34983	1.30788	1.84101	1.66805	1.528	1.98385	2.05566	2.0811	1.7245	1.64818
TTC21B-AS1	-1.01615	-1.18263	-0.91227	-1.04983	0.71924	-2.16678	1.09704	-1.11754	0.37749	0.47232	0.76807	0.95668	0.4434	0.71329	0.459	0.42606
SCN1A-AS1	-3.32193	-3.32193	-3.32193	-3.08354	1.0073	0.2727	0.88003	0.60598	-2.74052	-2.56812	-2.52515	-1.54427	-0.37889	0.75603	0.47606	1.10811
SCN1A	-3.32193	-3.32193	-3.32193	-3.32193	-0.90434	-1.4029	-0.83069	-0.73274	-3.32193	-3.32193	-3.32193	-3.32193	-3.22183	-2.29473	-2.98545	-2.8337
SCN9A	-3.32193	-3.20549	-3.32193	-2.8592	-1.73519	-2.3333	-1.29489	-1.84611	-1.73599	-1.1864	-1.84462	-0.18293	1.07079	1.95908	1.83208	2.60356
XIRP2	-2.19021	-2.13914	-1.89246	-2.33829	-3.28589	-3.08234	-3.16982	-3.13077	-3.32193	-3.16506	-3.32193	-2.72914	-1.09351	-1.65053	-1.63589	-1.37783
B3GALT1	-0.04625	-0.2894	-0.02258	0.53244	-2.34328	-0.15033	-2.43182	-0.27776	-3.32193	-2.41003	-2.61587	-2.31398	-0.38001	1.52168	2.54383	1.97673
STK39	3.63425	3.70063	3.74344	3.71782	-0.19078	-0.19095	0.1235	-0.49694	4.06455	3.69056	3.75524	4.07637	5.5564	5.78309	5.90095	5.42532
CERS6	0.60933	0.64866	0.74381	0.35298	4.00702	3.21902	4.58989	2.99614	3.65061	3.10193	3.25705	3.8507	4.62517	4.26697	4.77193	4.7268
CERS6-AS1	0.80084	0.52282	0.61575	0.22733	3.45514	3.53988	4.15133	3.39658	3.22496	2.58051	2.67801	3.67344	4.2872	3.88266	4.45927	4.39128
NOSTRIN	-1.84961	-2.00351	-1.95631	-2.6803	1.6651	0.83968	2.25693	0.3742	-0.91662	-2.32318	-2.38141	-2.55555	-0.91953	-1.36357	-0.93349	-1.41809
SPC25	4.58517	4.21366	4.26521	3.7898	4.27673	3.56856	4.55305	3.65369	4.57985	3.10373	3.90194	2.33791	4.99871	4.92647	4.27648	4.04569
LRP2	-3.32193	-3.32193	-3.24257	-3.32193	1.35329	-0.24604	0.88484	-0.2549	-3.24403	-3.32193	-3.32193	-3.19694	-3.32193	-3.32193	-3.08035	-3.32193
BBS5	0.18795	0.07863	-0.50337	-0.74996	1.18299	1.83268	1.0837	1.83381	1.41906	1.6503	1.88065	1.29736	2.42535	2.53398	1.95962	1.90329
KLHL41	-2.19435	-2.10162	-2.2843	-2.86359	-0.20516	1.2487	0.98571	0.33653	-0.69786	-1.73717	-1.19244	-1.01989	-1.59042	-1.16207	-1.28241	-1.25847
FASTKD1	3.10004	2.94	2.69213	3.28088	2.42168	2.44878	2.88593	2.42436	1.65086	1.82658	1.33372	2.10943	2.76999	2.95165	1.98028	2.24135
PPIG	4.83922	4.73951	4.74583	5.12681	4.00439	4.60653	4.49176	4.89934	3.90932	3.57204	3.78605	4.03623	4.19804	3.88055	4.18049	3.79543
CCDC173	-1.40475	-2.20505	-2.16252	-1.86107	-1.39559	-0.31852	-0.54962	-0.47539	-1.93576	-1.90317	-2.26623	-1.59799	-0.40999	0.30805	0.03971	-1.18861
PHOSPHO2	1.46472	1.75635	2.04145	1.92118	2.17257	1.5852	1.92608	0.82784	0.98509	0.97157	0.58963	0.36368	2.36931	2.3869	2.3728	2.00694
KLHL23	3.34869	3.21524	2.78515	3.48056	3.27312	2.9907	3.76473	2.87226	2.23313	1.85542	1.53712	1.87418	4.04739	4.09707	3.97789	3.70455
SSB	6.28035	6.004	5.94577	6.12066	5.49108	5.95268	6.00101	6.07676	5.82576	5.28292	5.66399	5.60953	6.03669	5.71714	5.27941	4.95074
METTL5	6.14521	5.90028	5.91888	6.23723	5.56113	6.23376	6.05754	6.31098	5.70284	5.35783	5.46464	5.56068	6.12638	5.86326	5.28538	4.9308
UBR3	2.18348	2.28899	2.19449	2.56142	3.14179	2.0275	3.65759	2.11096	2.67048	2.82902	2.56086	3.46537	3.49469	3.49345	3.45435	3.4798
LINC01124	0.26285	1.16333	1.63083	1.29886	0.00443	-0.69212	-0.01486	-0.40721	-1.49134	-1.14075	-1.60879	-3.32193	-3.32193	-3.32193	-3.32193	-3.32193
SP5	3.15031	3.42145	3.25742	2.2368	3.25166	3.19631	2.61206	3.4343	2.36069	2.09694	1.598	0.43304	-3.32193	-3.32193	-3.32193	-3.32193
EIF2S2P4	3.84561	3.98715	3.80009	4.60263	2.55954	3.20025	2.92784	2.94465	2.83983	2.24475	2.52052	1.82355	2.22423	1.90024	1.23827	1.63313
ERICH2	0.00572	0.98862	0.56261	0.79097	-0.03279	2.18248	-0.28058	1.26542	-0.51371	-0.74152	-0.37914	0.53271	-3.32193	-3.32193	-3.32193	-3.32193
GAD1	0.79001	0.55975	0.19997	0.94021	1.40466	1.52612	1.04275	1.60144	0.75455	0.73278	0.43151	0.39404	-2.58731	-3.21959	-3.32193	-2.77648
GORASP2	4.77508	4.09853	4.60142	4.2225	5.26077	4.64432	5.50761	4.30038	5.03368	4.0817	4.86586	4.49824	5.21225	4.14916	5.21906	3.99611
TLK1	2.99057	2.78823	3.00694	3.24584	3.04904	1.99571	3.59064	1.97378	2.65234	2.09685	2.60099	2.63273	3.20611	2.35021	3.56936	2.6125
METTL8	1.67328	1.56247	1.29026	1.67042	1.90245	1.43802	1.87175	1.86279	1.58565	1.65008	0.99963	1.63648	3.05039	2.57914	2.11664	2.23302

DCAF17	1.47297	1.45985	1.43607	1.78229	2.26589	1.77419	2.97076	1.90803	1.81423	1.58167	1.67224	2.16404	2.52653	2.52743	2.42865	2.46222
CYBRD1	3.06386	3.29439	3.45573	4.10058	0.32499	2.11935	0.47268	2.00209	4.49942	4.56314	4.15823	5.21505	4.94189	4.95211	4.71455	4.78938
DYNC1I2	5.38568	5.45209	5.44939	6.00656	5.16275	5.45198	5.85287	5.53372	5.61645	5.69164	5.65228	5.92251	5.38413	5.41304	5.37223	5.31529
SLC25A12	2.85441	2.67112	2.25114	2.87593	2.09871	2.01689	2.18362	2.01679	3.26479	3.08708	2.23393	2.77846	3.66255	4.02979	2.65702	3.1946
HAT1	4.33671	4.20123	4.06781	4.30191	4.05569	3.25604	4.54924	3.28781	4.1242	3.53009	3.73623	3.21886	4.48873	4.6097	4.06074	3.88441
METAP1D	1.57553	1.15019	1.21297	1.05641	0.35903	1.02967	0.55997	1.17043	-1.39969	-0.27707	-0.45414	-0.29867	0.62455	-0.01896	-0.02013	0.15712
DLX1	-2.13687	-1.3736	-1.99481	-2.70277	-2.61607	-0.51254	-2.47985	-0.60935	0.74779	1.28313	1.38241	0.61245	-0.55197	0.37092	-0.3051	-0.24163
DLX2	1.38332	1.34167	0.77389	0.36469	0.12136	1.49641	0.16928	1.58043	0.52255	0.08074	0.36863	-0.42643	-0.48565	-0.37111	0.29011	-0.0869
ITGA6-AS1	3.66802	3.8086	3.94318	2.84717	4.49794	-0.40402	4.89226	-0.5146	5.64452	5.38043	6.19935	5.10982	4.7474	4.33105	5.89758	5.72747
PDK1	2.16532	2.44929	2.49298	2.15215	0.26923	0.87137	0.56322	0.44267	3.37071	3.52166	2.95813	3.9972	1.43425	1.08349	1.37263	0.75945
RAPGEF4	-2.59666	-2.17011	-2.72174	-2.07439	-1.35368	-1.54725	-0.95274	-1.8299	-2.85765	-2.28644	-3.02849	-2.03216	-3.0947	-2.99429	-2.82034	-3.17626
MAP3K20	2.69182	3.47212	3.20928	4.07039	2.47091	1.58781	2.98024	1.8688	2.84928	3.16037	2.5313	3.81507	4.37461	5.45403	4.91462	5.35743
MAP3K20-AS1	1.61012	1.99481	1.721	2.71348	1.71298	-0.2852	2.41342	-0.63269	1.69435	1.47276	1.21104	2.22802	3.25589	4.22798	3.49562	3.87836
CDCA7	1.51702	1.1543	1.35284	1.13228	3.25443	2.88375	3.26891	2.69657	3.04678	2.16819	2.44748	2.36449	4.73791	4.72608	4.06941	3.66136
SP3	2.89768	3.00041	3.25277	3.36028	3.18752	1.99739	3.70869	2.0301	3.47558	3.44658	3.47106	3.89914	3.93393	3.08261	4.28674	3.49927
OLA1	5.62318	5.59728	5.57963	5.74854	4.68201	4.44978	5.25234	4.45531	5.11466	4.96697	4.87192	5.53657	5.69884	4.93039	5.42807	4.82638
SP9	-2.93911	-1.88608	-1.5063	-1.98023	2.1661	2.95429	1.48272	3.09113	-1.25877	-0.54357	-1.50699	-0.56456	1.3651	1.85236	2.03698	2.62744
CIR1	4.42774	4.74419	4.62974	5.45611	3.03984	4.33488	3.51911	4.51606	3.45815	4.03396	3.81789	4.39455	2.98511	3.50544	3.31522	3.42212
SCRN3	2.61163	2.78887	2.63784	3.0456	1.74557	1.7952	2.31036	1.65619	2.68104	2.59065	2.20809	2.72208	2.94219	2.70451	2.58086	2.46681
GPR155	-0.10599	-0.48055	0.3059	0.3337	-1.54861	-0.56547	-1.43274	-0.98852	-0.94889	-0.64506	-0.93151	0.10495	-0.30009	-0.85644	-0.24476	-0.58763
WIPF1	1.58983	1.13826	1.16168	1.19621	-2.21328	-1.17093	-2.04115	-1.20427	1.75015	1.50202	2.24118	1.58688	1.64147	0.24213	2.79504	1.01897
CHRNA1	-0.03028	-0.05701	0.54953	0.86026	-3.22716	-2.91783	-3.32193	-3.0708	-2.37402	-2.93423	-2.32653	-1.75141	-2.86175	-2.15333	-2.63441	-2.49004
CHN1	3.35073	3.18147	3.01297	3.31826	-0.21331	1.1268	0.20077	1.40279	1.40092	1.49828	1.07422	1.70815	2.21728	2.39049	2.22537	2.08031
ATF2	2.93177	3.1588	3.26979	3.69059	3.35441	2.88219	4.08685	2.96138	3.59133	3.87403	3.5016	4.31316	3.76791	4.14514	3.69013	3.76262
ATP5MC3	5.90146	5.70812	5.55532	5.7402	5.68426	6.01039	5.43561	5.83493	5.38773	5.03205	5.22203	4.07513	5.59806	5.27041	4.6531	4.66751
LNPK	3.00992	3.07671	3.08859	3.29104	2.2924	1.69977	2.63213	1.86069	2.9102	2.87667	2.317	3.27292	3.38658	3.27167	3.0454	3.06087
HOXD13	0.35394	0.79357	0.41301	0.40569	-2.52636	-3.32193	-3.32193	-2.45465	0.74043	1.09632	1.62253	0.65818	-3.32193	-2.97656	-2.90963	-3.32193
HOXD11	4.3508	4.37453	4.46071	4.43911	-3.32193	-3.32193	-3.32193	-3.32193	-1.32451	-2.04764	-1.98611	-1.27289	-2.95661	-3.32193	-3.32193	-3.32193
HOXD10	4.53743	4.45992	4.64385	4.8579	-3.15528	-2.95288	-3.32193	-3.32193	-1.57957	-1.63303	-1.03426	-0.82462	-3.02813	-3.32193	-3.32193	-3.32193
HOXD-AS2	2.73807	2.51307	2.81747	2.88123	-1.70087	-3.32193	-1.98798	-2.60297	-3.32193	-3.32193	-3.32193	-3.32193	-3.32193	-3.32193	-3.32193	-3.32193
HOXD9	4.32649	4.41249	4.76255	4.08054	2.66847	0.30218	1.40976	-0.75231	-3.32193	-2.47358	-3.32193	-3.32193	-2.91577	-2.91703	-3.32193	-2.46591
HOXD8	-1.13841	-0.52195	-0.85029	0.45113	1.00415	-1.56155	0.19806	-1.39249	-3.32193	-3.32193	-3.32193	-3.32193	-3.32193	-3.32193	-3.32193	-3.32193
HOXD3	0.2555	0.11383	-0.10578	0.09291	-2.59918	-3.32193	-3.32193	-2.9883	-3.32193	-3.32193	-3.32193	-3.32193	-3.32193	-3.32193	-3.32193	-3.32193
HOXD4	1.31361	1.41068	1.23942	0.87974	-3.32193	-3.32193	-3.32193	-3.32193	-3.32193	-3.32193	-3.32193	-2.7345	-3.32193	-3.32193	-3.32193	-3.32193
HAGLR	1.29389	1.38629	1.3261	1.50936	-1.88965	-2.60204	-2.0038	-3.07061	-3.32193	-3.32193	-3.32193	-3.32193	-2.99921	-3.32193	-3.32193	-3.32193
MTX2	5.12165	4.52876	4.89827	4.88203	4.76387	4.25298	4.88101	4.11755	4.22493	3.55847	4.33547	3.07998	4.92663	4.13188	4.36755	3.1716
LINC01116	2.19645	2.52793	2.54207	2.31922	-2.86803	-2.1076	-3.32193	-2.72001	3.69504	3.68486	3.99754	3.71133	-2.8986	-3.32193	-3.32193	-3.32193
LINC01117	1.40804	1.73995	1.81563	0.44521	-3.32193	-3.32193	-3.32193	-3.32193	2.16609	2.38937	2.98579	1.70388	-3.32193	-3.32193	-3.32193	-3.32193
MIR4444-1	9.76763	9.61924	9.36609	9.17336	8.67756	7.86005	8.93547	7.58654	10.0722	8.99387	9.6802	8.61778	10.6176	10.3134	10.3081	9.72891
NFE2L2	4.40557	4.25762	4.18592	4.75703	6.0568	5.12161	6.41497	5.16794	4.69913	4.38742	4.24231	5.23013	4.65467	4.23235	4.00564	3.90903
DNAJC19P5	0.4093	0.81067	0.8843	0.72271	1.64386	2.86819	2.54387	2.64032	-0.57568	0.36086	-0.21206	0.3382	0.08689	0.52818	-0.81353	0.08232
AGPS	4.00516	3.90615	3.66275	4.09655	4.75193	3.51403	5.4772	3.55117	4.48961	4.23066	4.08889	4.51519	4.25042	4.40951	4.4305	4.11706
TTC30B	0.86359	1.42257	1.24251	1.731	1.30535	0.64627	1.3723	0.9719	1.62615	2.23894	2.13858	2.96552	1.43482	2.1952	1.71054	2.43246
TTC30A	1.31619	1.69358	1.83289	2.10362	0.97427	0.44445	1.67439	0.60538	1.6553	2.49797	2.46893	3.35188	0.90356	1.87551	2.12637	2.56826
PDE11A	-3.23321	-3.22272	-3.32193	-3.32193	-1.09225	-1.46228	-0.8472	-1.28879	-1.57386	-1.30116	-1.22258	-0.73927	-3.25197	-3.18571	-3.32193	-3.32193
RBM45	1.36858	1.36538	1.46067	1.88129	1.68142	1.26087	1.7336	1.637	1.805	1.96403	1.77721	1.66717	1.77283	1.79442	1.18173	1.44788
OSBPL6	1.73548	1.17728	1.24214	1.42014	0.09371	-1.09257	0.29008	-0.61142	1.78487	1.38328	1.05726	2.07027	0.63763	0.06239	-0.01408	-0.17676
PRKRA	2.99948	2.77902	2.98477	3.18582	3.99621	3.54456	4.22238	3.43982	3.71172	2.86737	3.20785	3.18861	4.33651	3.20181	3.91149	3.17269
PJVK	-1.78783	-2.04766	-1.62313	-0.78907	-0.30166	0.86817	-0.30767	0.81049	-2.2502	-0.84401	-1.22646	-1.43589	-2.52059	-0.94516	-2.39204	-1.40528
FKBP7	0.54669	0.39635	0.67145	1.27632	2.42368	3.23647	2.83925	2.71674	1.40503	2.00437	2.21479	2.16078	1.10642	2.00523	1.86259	2.08943

PLEKHA3	1.63326	1.06678	1.5257	1.62072	1.72564	0.7684	2.10635	0.28535	1.10638	-0.35803	0.78727	0.25558	1.83941	-0.5643	1.59881	-0.58716
TTN-AS1	-3.0286	-2.90248	-2.98038	-2.35938	-0.70021	-1.10552	-0.53684	-1.35655	-0.81767	-0.59585	-1.17605	-0.17836	-0.92449	-0.64442	-1.5247	-0.64599
TTN	-3.24854	-3.2599	-3.24592	-3.07169	-2.09315	-1.92374	-1.86696	-1.91449	-2.09881	-1.92384	-2.2881	-1.42384	-2.00742	-1.64161	-2.2671	-1.6629
SESTD1	0.75993	1.3322	1.56953	1.92737	2.2239	2.04894	3.13762	2.01903	1.36297	1.13557	1.23006	2.13267	1.34961	2.28312	2.16386	2.36698
CWC22	4.3045	4.18866	4.08289	4.37181	4.17462	3.8415	4.74644	3.88395	3.79567	3.75693	3.62234	4.24816	3.88999	3.60049	3.63808	3.04565
FTH1P20	5.41239	5.5397	5.67548	5.32086	1.39303	1.76278	0.63619	2.45694	2.01825	3.29228	2.21668	4.30029	0.51749	0.26975	0.66668	1.02498
UBE2E3	4.46473	4.15363	4.05446	4.40767	4.8168	3.92579	4.66087	3.95717	5.1987	4.67386	4.39451	4.57641	5.3584	4.85176	4.38797	4.59541
ITGA4	-3.06315	-3.03444	-3.02005	-2.5195	-3.20631	-2.83931	-3.32193	-2.76872	4.64729	4.54447	4.55489	4.92565	3.39716	3.75533	3.6673	3.72813
CERKL	-3.32193	-3.32193	-3.32193	-2.3018	-2.34486	-2.7427	-2.50619	-2.84948	2.60918	2.7502	2.45041	3.6807	2.23074	2.66228	2.03989	2.38792
NEUROD1	-3.32193	-3.32193	-3.32193	-2.94806	-3.32193	-3.32193	-3.32193	-2.68275	-3.32193	-3.02801	-3.00838	-3.32193	-0.79255	1.35122	-0.1081	1.30933
ITPRID2	2.68754	3.11654	3.10629	3.47676	3.99936	3.69844	5.06002	3.36085	4.07378	5.09003	5.00387	5.44241	6.2065	6.51807	6.89762	6.64457
PPP1R1C	-1.78301	-1.44466	-1.53703	-0.94059	-2.90267	-0.56052	-2.19367	-0.3239	0.67378	0.32028	-0.53059	0.12916	-0.57277	0.05965	-1.0617	-0.74677
DNAJC10	2.99258	3.28846	3.1696	3.74609	4.34276	4.18888	5.14063	3.88875	2.97512	2.91218	2.82705	3.87117	3.77614	3.83161	3.54082	3.73806
NCKAP1	3.30412	3.38935	3.41007	3.88677	4.48952	3.19586	5.14934	3.14193	3.73286	3.68425	3.57974	4.32241	4.75112	4.77903	4.63425	4.38992
KRT8P10	-1.2453	-1.31829	-2.13867	-1.72198	1.05785	-0.41357	1.26487	-0.10571	1.10928	1.79156	1.34756	0.83022	-2.4419	-1.20103	-1.97921	-0.94693
DUSP19	-0.6903	-0.08528	-0.20148	-0.0918	0.29651	0.00718	0.49279	0.4367	-0.04009	-0.46104	-0.39965	0.08137	0.02308	0.06581	-0.61852	-0.39185
NUP35	3.1136	2.56394	2.2506	2.44671	3.6597	2.57464	3.87883	2.56511	2.25097	1.68979	1.71347	1.03265	3.76183	3.7631	3.23604	2.95187
CACYBPP2	2.70276	2.78861	2.35554	2.52001	1.99615	2.61317	2.12899	2.11129	2.12361	1.62312	1.4377	1.54671	3.01464	2.51847	2.19566	1.87762
ZNF804A	-1.45581	-1.26712	-1.49553	-0.83855	-3.32193	-3.32193	-3.11486	-3.32193	1.34528	1.68931	1.67044	2.35054	-3.32193	-3.14679	-3.32193	-3.32193
ZC3H15	6.71843	6.40909	6.22963	6.76179	5.83864	5.68303	6.25452	5.85941	5.7648	5.52089	5.33319	5.77096	5.75166	5.35374	5.08176	4.86829
ITGAV	4.03524	4.40177	4.79783	5.37025	5.49664	3.4077	6.24718	3.41522	4.45055	4.70571	4.96479	5.87491	5.74501	5.43933	6.11835	5.70973
FAM171B	0.95486	1.07287	0.85161	1.37237	2.48963	1.35888	2.77395	1.3449	1.77133	1.49741	1.29046	2.08913	2.17795	1.73735	2.47415	2.71964
CALCRL	-3.32193	-3.32193	-3.12073	-3.10846	-3.32193	-2.21794	-2.88498	-2.78513	-2.10688	-1.76171	-1.82008	-1.32416	-0.74948	-0.35768	-0.45967	0.63656
TFPI	1.55373	1.68857	1.48149	2.25314	3.18881	5.28146	3.63096	5.2639	3.77291	4.37455	3.47102	4.79795	5.20603	5.0376	4.99347	4.68174
LINC01090	-2.74664	-3.32193	-3.32193	-3.32193	1.50937	1.79441	1.92776	1.69912	-2.6716	-2.36219	-3.32193	-2.37408	-3.32193	-2.50227	-3.32193	-2.75814
GULP1	1.25134	1.11938	1.19868	1.91549	2.25719	1.29379	3.05954	1.15504	2.74191	2.8094	2.65729	3.48209	2.87841	2.88028	3.05675	2.68458
COL3A1	2.83176	2.8528	2.86701	3.50086	-1.44939	-1.70189	-0.95783	-1.53968	3.81357	3.15407	4.45617	3.37929	3.65616	3.20721	4.74758	3.89085
MIR3606	5.72992	5.51508	5.50286	6.13101	1.69566	0.42823	1.26296	0.64642	6.80468	5.83597	7.77038	5.71848	6.48957	5.80422	7.45412	6.79055
COL5A2	-1.51683	-1.95314	-1.24732	-0.5965	2.78345	1.41468	2.65207	1.66458	3.6061	3.37438	3.50706	3.34711	6.85318	7.23045	6.89062	7.15307
WDR75	4.46596	4.18777	4.06398	4.57239	3.96635	3.98105	4.27947	3.98585	3.921	4.04424	3.77121	4.24865	4.68374	4.64522	3.87177	3.88132
SLC40A1	-1.37038	-1.46778	-2.27805	-1.18635	4.42976	3.15505	4.52283	3.30532	1.3075	0.92415	0.96852	1.01443	4.30651	4.47391	5.0214	4.79918
ASNSD1	5.01917	4.91945	4.68544	5.14861	4.55733	4.05445	4.96758	3.9957	4.48317	4.56755	4.13482	4.81563	5.40352	5.0771	4.5033	4.39859
ANKAR	-0.32029	-0.41399	-0.5673	0.09748	0.36176	0.40767	0.46817	0.21178	-0.39967	-0.46615	-0.48522	-0.71047	0.67762	0.26883	0.14246	-0.16939
OSGEPL1	1.36142	1.43374	1.48262	1.71019	1.96667	1.94301	2.41599	2.12137	1.57061	1.5966	0.97268	1.31152	2.10151	1.79285	1.6123	1.09037
OSGEPL1-AS1	0.00356	0.86551	0.57524	1.1048	1.43633	1.13662	2.16365	1.82166	0.20036	1.43868	-0.08711	0.85684	1.71439	1.05144	-0.34135	0.31602
ORMDL1	4.13678	4.17116	4.22021	4.52187	3.97483	4.48825	4.48268	4.23126	2.67599	2.61634	2.53807	2.52384	4.16227	3.85689	2.82439	2.7238
PMS1	3.00729	2.93336	2.87098	3.00724	3.04385	2.5426	3.5381	2.34373	2.44869	2.15379	2.00236	2.30145	3.68162	3.36271	2.88672	2.56186
C2orf88	-1.23941	-1.46806	-1.29814	-0.84307	1.36676	1.09847	1.56709	1.49804	-0.36413	-1.41675	-2.01464	-0.8845	-0.62704	-0.85041	-0.6356	-1.10081
HNRNPCP2	4.65072	4.19327	4.35416	3.18716	3.64203	3.97153	3.4426	3.82179	4.82067	4.90292	4.2679	4.27737	3.60619	3.39017	3.06051	3.17645
HIBCH	2.40217	2.41236	2.34887	2.87416	2.40552	2.35963	2.70242	2.29368	2.46506	2.34216	2.42046	2.23864	2.60693	2.69009	1.8387	1.9246
INPP1	2.90082	2.67887	2.72322	2.93412	4.11481	4.08804	4.38623	3.81635	2.88532	3.12973	2.97536	3.04722	2.43109	2.66223	1.23737	2.0346
MFSD6	1.61346	1.60034	1.53867	2.17075	3.13228	2.33466	3.58835	1.97889	0.54715	0.66824	0.30236	1.01973	2.82511	1.79301	3.48475	3.02677
NEMP2	1.35822	1.2126	1.32024	1.00588	0.51542	0.36886	0.79338	0.38214	2.96196	1.82032	2.58637	2.23717	1.36751	0.142	1.38476	0.4252
NAB1	4.07101	4.3007	4.19033	4.56665	4.30559	3.60395	4.77018	3.4692	3.98101	4.40516	4.12028	5.14894	3.1989	4.01168	3.31113	3.7237
GLS	4.14796	4.31238	4.45047	4.64591	3.69153	2.74842	4.24305	2.65962	3.60982	3.80332	3.74804	3.89671	5.48416	5.3729	4.84037	4.54789
STAT1	4.76992	4.82458	4.77357	5.36279	5.11579	4.23916	5.77602	4.08857	4.68898	4.64181	4.69684	5.75674	4.7047	4.96621	4.77929	4.93809
STAT4	-2.13396	-2.45808	-2.42244	-1.59712	-0.55282	0.49665	-0.75064	0.29804	-0.24651	-1.08987	-0.50354	-0.50243	-2.54344	-2.54558	-2.41753	-2.70404
MYO1B	3.82874	4.00904	3.91909	4.4609	3.96483	3.00782	4.78793	2.86496	5.55748	5.25282	5.39434	5.96133	4.29733	3.89435	4.35072	4.37937
NABP1	2.96274	3.19348	3.11739	3.56622	0.6271	1.33418	1.53712	1.62393	2.85639	3.08787	3.15278	3.0654	2.77735	2.4227	1.61986	1.74316
CAVIN2	-3.32193	-2.69941	-2.67161	-3.32193	1.07793	1.58109	1.11378	1.61975	5.95965	5.47977	5.58368	5.25285	0.81318	-0.02639	-0.89965	-2.20238

TMEFF2	-3.32193	-3.32193	-3.32193	-3.32193	-3.18521	-2.36099	-3.32193	-1.90492	-1.71767	-2.57597	-2.43548	-2.79209	1.13116	1.41031	0.45811	0.15885
SLC39A10	2.54163	2.10647	2.26876	2.39006	3.74521	2.55167	4.39772	2.41094	4.68746	4.65857	4.35014	4.90179	4.32331	3.92274	3.82979	3.57334
DNAH7	-2.47335	-1.99337	-2.60226	-1.54154	-3.00309	-2.58867	-2.69564	-2.60002	-2.77067	-2.16592	-2.1557	-1.26038	-2.54553	-1.28737	-1.72954	-0.91656
STK17B	3.43744	3.32041	3.42789	3.84147	1.55353	1.57246	2.30902	1.47487	3.4431	3.36173	3.26399	4.25026	2.55469	2.84598	2.69716	2.38146
HECW2	0.52526	0.88232	0.84999	1.09625	-1.22691	-1.18426	-0.9056	-0.87919	1.39138	1.51584	1.65399	2.27904	-2.15484	-1.05322	-0.99181	-0.4608
HECW2-AS1	1.3579	0.7416	1.77342	2.08607	-2.82599	-2.74825	-1.83482	-2.66709	0.58827	1.33442	0.72333	2.38623	-3.32193	-3.32193	-3.32193	-3.32193
CCDC150	0.65518	0.06298	0.21852	0.62942	0.45031	1.60106	0.61965	1.67303	-0.70742	-0.8468	-0.86675	-0.88707	0.22342	0.30161	-1.05101	-0.68024
GTF3C3	3.15982	3.19545	2.97066	3.47306	3.20708	3.18081	3.69234	2.89798	2.88024	3.17344	3.0888	3.72696	3.82729	3.91399	3.43163	3.61247
PGAP1	-0.41355	0.03079	-0.26023	0.53735	0.0569	-0.34241	0.70738	-0.15885	-0.14255	0.67933	0.05489	1.51108	1.82382	2.4147	1.78923	2.61312
ANKRD44	-0.14885	0.24487	0.20403	0.64559	-0.76962	-0.92373	0.2092	-1.39194	-0.97759	-0.84758	-0.38158	0.30288	2.14935	2.39487	2.88645	3.00432
NPM1P46	2.01718	1.08008	1.15444	1.30955	0.12535	0.24929	0.89438	-0.65836	0.98351	1.47621	0.48676	1.80832	-0.31665	-1.39528	-1.17573	-3.32193
COQ10B	4.39818	4.64745	4.48726	5.16071	3.94433	4.71094	4.68838	4.37055	3.46427	3.56228	3.27794	3.66937	4.74742	5.43343	3.94223	4.39207
HSPE1	8.12831	7.75406	7.61298	7.46902	7.69866	7.98174	7.63135	7.87574	6.85518	6.37709	6.75153	5.08762	7.81308	7.42389	6.77538	6.16701
HSPE1-MOB4	9.21126	8.89478	8.77867	8.67932	8.86389	8.98807	8.84425	8.84257	8.17434	7.71476	7.97914	6.62775	8.96653	8.67557	8.09199	7.60591
MOB4	4.10051	4.25244	4.00602	4.5177	3.81477	3.58171	4.24696	3.50586	4.14204	3.93134	3.63398	4.15175	4.62389	4.62537	4.22849	4.10148
RFTN2	-2.20901	-1.86764	-2.27126	-0.76288	-2.29153	-1.06239	-2.64114	-0.83386	-2.78534	-3.00095	-2.83492	-2.6327	-1.93262	-1.88117	-2.52273	-1.61285
MARS2	3.60677	3.48287	2.75916	2.98574	3.57188	2.72206	3.15491	2.50209	2.04755	1.85606	2.13347	1.72986	2.96601	2.85286	2.47857	2.53477
SATB2	0.5247	-0.08519	0.09081	0.37642	2.27879	1.53961	2.55539	1.56355	2.13125	1.32822	2.22376	1.78398	0.81027	0.02548	0.81844	0.31085
SATB2-AS1	-1.05294	-1.21324	-1.44453	-1.75015	0.78434	0.68683	0.01093	-0.01739	0.39057	-0.0681	0.62194	-0.71251	-1.22951	-0.79989	-0.59156	-0.42689
FTCDNL1	-0.05918	-0.3081	-0.88521	-1.50118	-1.14244	-1.59152	-1.248	-1.56225	-1.20711	-0.88312	-0.5274	-0.41105	-0.92519	-0.14331	-1.4203	-0.17932
C2orf69	2.99255	3.13735	2.98408	3.07458	3.97328	3.4237	4.49261	3.34126	3.95935	3.39715	3.41605	3.77414	4.68311	4.79474	4.56763	4.564
TYW5	0.54654	0.51393	0.40757	0.72209	1.27735	1.19098	1.53033	1.32759	0.39165	0.63655	0.38145	0.75809	1.42732	1.68834	1.05433	1.2112
MAIP1	3.80809	3.62411	3.66327	3.8357	4.73707	4.43956	4.86498	4.40522	4.39902	4.02872	4.1151	4.09121	4.87301	4.85492	4.73884	4.32965
SPATS2L	3.28794	3.06546	3.20587	3.04247	3.91632	3.0666	4.43045	2.99651	4.8461	4.18066	4.51207	4.7662	5.3784	5.09341	5.58203	5.18436
KCTD18	1.01898	1.23322	1.26367	1.64618	1.82118	1.58773	1.64911	1.73573	1.4461	2.5648	1.20688	2.89145	1.09007	2.10137	0.76391	2.19668
SGO2	2.37954	2.06327	2.44873	2.53468	3.0406	1.5689	3.83021	1.73081	3.55703	2.20129	2.88031	2.37028	4.11565	2.47588	3.56246	2.36272
AOX1	3.62491	3.50358	3.24291	3.08045	-2.59719	-2.81227	-1.89158	-1.90969	3.83377	2.72675	3.25762	2.3707	1.96254	0.29065	0.40817	-0.16707
CLK1	2.72579	2.76683	2.72062	3.13727	2.32276	3.66652	2.95177	3.72311	2.50685	3.49697	2.88325	3.27041	2.95318	3.14824	2.09451	2.09103
PPIL3	2.53599	3.00396	3.16458	3.41382	2.13699	2.98358	2.49038	2.83446	3.00457	3.20439	2.4323	3.39614	3.55632	3.68317	2.83156	3.12154
NIF3L1	3.70508	3.2796	3.6876	3.52998	4.09768	3.67693	4.27306	3.52278	3.54709	2.87559	3.17815	3.08983	4.63696	4.16636	3.82512	3.65707
ORC2	2.62106	2.51811	2.28532	2.5383	3.59001	3.52915	4.07883	3.8545	3.0413	2.54749	2.75896	3.34873	4.10608	3.91586	3.62105	3.52638
FAM126B	0.68038	0.77541	0.65869	1.17921	1.2225	1.27965	2.3991	1.75961	1.73659	1.65255	1.45212	2.6093	3.34116	3.31908	3.16288	3.13608
HNRNPA1P35	2.15448	1.62534	2.13641	2.05994	0.77294	0.85696	0.74831	0.42886	2.0124	2.204	0.95366	0.02408	0.23066	-0.5859	0.41268	0.16663
NDUFB3	5.34697	5.5256	5.47407	5.47331	5.88122	6.08383	6.02075	5.85627	5.86017	6.03811	5.84466	5.41925	6.83485	6.79087	5.38841	5.55667
CFLAR	1.22817	1.19686	1.25433	0.97504	2.68402	1.80141	2.96198	1.69723	3.26697	2.79562	3.19046	2.80884	3.30738	2.87252	3.13853	2.55969
IMPDH1P10	1.75343	0.70072	0.61458	0.23899	3.53345	1.96964	3.41562	1.09452	3.30307	2.46883	3.01392	2.634	3.52302	3.02763	3.40925	2.81261
CFLAR-AS1	0.87739	1.10445	1.32041	1.26657	2.06755	1.35751	2.34994	1.43219	2.75721	2.01335	2.58033	2.01988	2.98958	2.68855	2.28397	1.81054
CASP10	-0.48825	-0.3439	-0.27561	-0.26011	1.39225	1.35504	1.81291	1.40676	0.99121	1.11671	1.05576	1.69918	1.73337	2.12635	1.57387	2.27052
CASP8	2.54418	2.73178	2.25834	3.11257	3.25927	3.95073	3.7963	3.83259	3.4706	3.57791	3.70031	4.27652	3.54137	3.77536	3.29408	3.68569
TRAK2	2.52894	2.2254	2.27311	2.75628	3.24469	2.04956	3.54243	2.12235	3.75978	2.34377	3.20558	3.35516	4.67662	2.56252	4.47491	2.92014
STRADB	2.8548	2.82501	2.79393	3.26599	4.40925	4.35104	4.44169	4.40788	4.45756	3.85387	3.99746	4.56281	5.6666	5.27601	5.32219	5.10707
C2CD6	-3.32193	-3.32193	-3.32193	-2.95732	-2.17842	-1.02476	-2.35348	-0.767	-3.32193	-3.32193	-3.32193	-3.17402	-2.97976	-2.79135	-3.03805	-3.03236
TMEM237	1.24081	1.0342	1.23912	1.41279	2.59862	2.11706	2.61479	2.09654	3.14433	2.6225	2.66809	2.64343	4.97398	4.59325	4.62493	4.42516
ENO1P4	-1.30628	-1.76019	-0.58504	-0.89133	1.09172	0.78792	1.3188	-0.37276	2.60019	2.23636	2.31168	2.31146	3.768	3.74474	4.15926	4.2205
MPP4	-0.39213	-0.37282	-0.23966	-1.19012	-3.32193	-2.92278	-3.1239	-3.32193	2.03995	1.38288	1.54835	0.53088	2.46168	0.77558	1.30022	0.53961
ALS2	1.45333	1.45993	1.27499	1.55585	2.77381	2.25405	3.04419	2.26119	2.45813	2.34081	2.25366	2.84935	3.96397	3.82271	3.6935	3.82028
CDK15	-3.15023	-3.32193	-2.78668	-3.32193	-3.32193	-3.14844	-3.32193	-3.32193	1.65117	1.18998	0.98937	-0.10983	-2.19222	-3.18618	-3.01004	-3.32193
FZD7	2.4139	2.17053	2.46173	2.29982	2.45675	1.34492	1.7593	1.02689	3.98486	3.6136	3.78538	2.9919	3.1215	2.7697	3.58559	3.74911
SUMO1	5.84149	6.13308	5.83597	6.04587	6.02886	6.11227	6.33117	5.95016	6.5978	6.52234	6.29453	6.50535	6.73441	6.80057	6.37921	6.36613
NOP58	6.48	6.27996	5.9914	6.26049	5.90447	5.69322	6.23736	5.94037	5.6814	5.30564	5.32792	5.27945	6.15206	5.66276	5.48175	5.18574

BMPR2	1.28772	1.46325	1.49561	2.00687	2.28469	1.12246	2.59934	1.13897	3.36193	3.254	3.05233	4.70933	3.81816	3.6605	4.6171	4.49696
FAM117B	0.89788	0.97287	0.66901	0.82335	2.16807	1.52564	2.05463	1.10504	1.88238	2.17023	1.68532	2.23173	1.65876	1.212	2.50033	2.62604
ICA1L	-2.59153	-1.9648	-2.42074	-1.67746	-1.28271	-0.32914	-0.75589	-0.35955	-1.29888	-1.01315	-1.30951	-0.896	-0.41829	-0.29583	-0.5706	-0.08423
WDR12	2.89129	2.72485	2.36831	2.59049	3.49206	3.4079	3.66101	3.55187	2.9588	2.79579	2.6131	2.72995	3.77894	3.83704	2.93855	3.14557
CARF	-2.20535	-1.72861	-1.88708	-0.73891	-1.5707	-1.78181	-1.85639	-1.94154	-1.6467	-1.05496	-1.11064	-0.17819	-1.16814	-0.75854	-1.45012	-0.31903
NBEAL1	1.3877	1.39621	1.59442	1.12571	1.89729	0.89794	2.54215	0.93319	1.45641	1.79229	1.67718	1.78167	2.12392	2.12012	2.41558	2.56855
RPL12P16	6.06855	6.1235	6.32942	5.465	4.38885	4.96807	3.75314	4.7636	5.82782	6.23346	5.93755	4.98718	4.82293	4.19763	4.19007	4.22145
CYP20A1	3.14926	3.23392	3.2076	3.3532	3.17959	3.45714	3.474	3.57079	2.52476	2.35254	2.6941	3.16052	2.80162	2.70808	2.91389	2.73721
ABI2	2.28007	2.48056	2.51501	2.78285	1.80412	1.70409	2.20533	1.67541	2.94231	2.85865	2.69495	3.6292	3.3824	3.35367	3.38947	3.08162
RAPH1	2.31139	2.72281	2.71751	2.85531	1.91204	1.23107	2.61794	1.39689	2.66083	2.90374	2.72751	3.65302	2.97756	2.82736	3.1922	2.67228
PARD3B	-2.34239	-1.57803	-1.94665	-1.99426	0.59727	-0.89328	0.99858	-0.38295	-0.01988	0.45285	-0.03637	1.10306	-0.65983	0.24544	0.4696	-0.04673
NRP2	4.16549	4.11963	4.31262	4.41098	-0.82994	-2.08602	-0.73578	-2.64577	2.19519	1.29806	2.11034	1.9284	3.2032	1.74151	3.64609	3.1891
INO80D	0.15727	0.54362	0.10084	0.84589	0.97752	1.53101	1.39246	1.44384	0.26111	0.30467	0.50605	1.30158	1.75821	1.47776	1.73884	2.03918
NDUFS1	3.23166	3.34407	3.23056	3.40084	4.15693	3.51128	4.54858	3.51502	3.73331	3.57491	3.64382	3.88104	4.32296	4.40281	4.4157	4.4149
GCSHP3	2.50027	2.25661	2.93078	3.3396	3.12082	2.3554	3.60211	2.12091	1.63199	2.10803	2.55032	3.22041	2.82738	2.61899	3.58969	2.89933
EEF1B2	7.74372	7.49885	7.37705	7.32456	7.35905	7.38901	7.27805	7.30792	8.05492	7.98186	7.94992	7.7192	7.69696	7.26697	7.02749	6.86414
GPR1	-3.32193	-2.16519	-2.91654	-2.89355	-3.16319	-3.32193	-3.32193	-3.32193	-0.21093	-1.68378	-0.11601	-1.28089	-3.32193	-3.32193	-3.32193	-3.32193
ZDBF2	-2.78552	-2.7317	-2.62373	-1.99074	0.78726	0.80475	1.33984	0.73322	0.14187	0.65014	0.28653	2.26407	-0.62843	-0.53156	-0.5861	0.13248
ADAM23	-3.32193	-3.32193	-3.12885	-3.32193	2.07272	0.59017	1.77278	0.63417	-0.53515	-0.82055	-0.11025	0.07185	-3.32193	-3.32193	-3.32193	-3.32193
MDH1B	-2.51567	-2.96567	-3.32193	-2.61698	-0.9325	-1.99199	-0.94535	-1.33342	-1.15814	-0.72414	-0.89033	-0.68856	-1.27643	-0.1231	-0.47496	-1.10377
FASTKD2	2.51541	2.40648	2.02252	2.45502	2.88828	1.91799	3.25918	2.08598	2.78281	2.62228	2.72693	2.96975	3.86525	3.38341	3.20823	3.25271
KLF7	2.93072	3.23006	3.6415	3.49317	0.65386	-0.63883	1.18554	-0.58185	3.39533	3.41538	3.65935	3.94633	2.36595	2.21608	2.88683	2.43704
MYOSLID	4.71897	4.88496	5.15119	4.34209	-3.32193	-2.11809	-2.64231	-3.32193	4.21196	3.89064	4.3786	3.98232	-1.72736	-1.14481	-1.09151	-1.79227
CREB1	2.38706	2.59911	2.6273	2.92026	2.30758	1.5293	2.76951	1.4346	2.81636	2.91328	2.72414	3.37913	3.28387	3.10506	3.63643	3.37506
METTL21A	2.68881	2.59901	2.42991	2.60095	3.3388	3.08806	3.46111	3.12481	2.23008	1.84838	1.85307	1.28558	3.23983	2.49888	2.45542	2.37308
PPP1R14BP2	0.74263	0.55914	0.18328	-3.32193	1.75126	2.97755	0.31287	2.79931	-1.72174	1.29857	0.0078	-1.21283	-0.62643	-0.35424	-0.09779	-1.88586
CCNYL1	2.89481	2.79651	2.94466	3.04607	4.20534	3.50713	4.78761	3.50054	3.28397	2.54986	2.99538	3.49322	4.77244	4.21096	4.27684	3.99978
MIR4775	4.22704	4.6142	4.75871	4.20078	5.66226	4.39408	5.23925	4.30694	4.44302	4.00595	4.62973	3.3089	5.94753	5.31046	5.79255	5.0186
FZD5	1.90034	1.97488	1.89481	1.55266	6.21213	4.66959	6.02781	4.8282	1.2384	1.73308	1.34773	1.63984	1.60687	2.54705	2.93448	3.37969
PLEKHM3	0.23548	0.52959	0.0749	0.89107	0.51625	-0.46336	0.79289	-0.76025	0.39348	0.35605	0.18059	1.33388	0.06789	-0.38745	0.65351	0.54873
IDH1	4.17673	4.26288	4.25913	5.09234	6.4399	6.79874	6.75768	6.66569	5.2664	4.32159	4.97435	4.83896	5.48224	5.49487	5.79866	5.61751
IDH1-AS1	1.15889	0.37514	-0.31851	0.67432	2.27216	3.67798	1.6259	3.04863	0.91876	0.02019	0.76192	-1.19433	3.03143	3.80476	2.17379	2.4685
PIKFYVE	1.47495	1.55131	1.07848	2.39886	2.9181	2.28941	2.85392	2.54062	2.33306	2.60979	1.5278	3.21571	3.21378	3.76221	2.92814	3.68018
PTH2R	1.91059	1.71147	1.69678	1.83161	-3.32193	-3.08467	-3.10157	-3.32193	-3.32193	-3.32193	-3.32193	-3.32193	1.73429	2.28137	1.98745	2.33177
MAP2	-3.22817	-3.2171	-3.32193	-3.096	-2.89733	-3.22717	-3.07336	-3.10815	-2.2566	-1.47799	-1.83214	-0.05993	-1.73749	-0.8882	0.75688	0.57444
RPE	4.19914	4.28109	4.25002	4.40318	5.11258	4.54326	5.531	4.29088	4.77365	4.68521	4.80833	4.68552	5.33422	5.18028	5.02612	4.68918
KANSL1L	0.55688	0.60007	0.52886	1.0836	1.09368	1.25819	1.99044	1.50574	0.58731	0.69246	0.306	1.51813	1.61234	1.84654	1.42394	1.3722
LANCL1-AS1	-1.80474	-3.32193	-1.64101	-1.85557	0.09612	0.24207	0.48931	0.33675	-2.6979	-2.78691	-2.03095	-1.14212	-2.2555	-1.0006	-2.40429	-1.61188
LANCL1	2.67313	2.41217	2.31185	2.9945	5.6209	4.90981	5.97454	4.71874	4.50329	4.23618	4.34773	4.55633	4.87217	4.43338	4.61466	4.42673
ERBB4	-3.32193	-3.32193	-3.32193	-3.32193	-3.32193	-2.94179	-2.90619	-3.22307	-3.32193	-3.16487	-3.32193	-3.32193	1.0892	2.04824	2.05045	2.76389
IKZF2	-2.92463	-2.67175	-3.02872	-2.68008	-3.00883	-2.92079	-2.94793	-3.11924	-1.28787	-0.34957	-0.85402	0.44608	1.40511	1.47656	0.93367	0.97272
SPAG16	1.06537	1.39617	1.23232	1.38058	1.24091	1.09341	1.29361	0.79266	1.25306	1.19754	1.06774	0.94613	1.96066	1.93087	1.4247	1.41595
BARD1	1.68768	1.32069	1.26522	1.18913	2.13847	0.88449	2.67108	0.9109	2.6418	1.38764	1.75416	1.33654	3.33226	3.42296	3.15676	2.87098
RPL10P6	2.51437	1.97641	2.87355	-1.29299	1.85769	3.9215	0.76106	3.5852	2.21382	3.38617	1.39415	1.7395	1.47347	1.56456	0.58315	1.16578
ABCA12	-3.20766	-3.19427	-2.95126	-3.17912	-3.2243	-3.20644	-3.32193	-3.32193	-0.28884	0.35202	-0.4844	1.8545	0.84664	2.74038	2.22168	2.81921
ATIC	5.66622	5.52586	5.30195	5.62098	6.55732	6.53846	6.74319	6.50298	6.02245	5.50852	5.86016	5.94516	5.77704	5.70351	5.53292	5.51173
MREG	-1.848	-1.27384	-1.6868	-0.78361	0.74396	1.10717	0.56068	2.10393	2.80284	2.46904	2.63314	2.50731	-1.06482	-0.65451	-1.35487	-0.74741
PECR	1.75206	1.94508	1.79946	2.05812	4.50021	4.36411	4.18046	4.54638	2.7934	2.8904	2.69319	2.95486	2.61784	2.91184	2.48435	2.37676
TMEM169	-2.60667	-1.77552	-0.9842	-1.33255	1.02518	1.00244	0.79696	0.96897	-2.32581	-3.05135	-2.1018	-1.63004	0.12275	0.71051	0.66993	0.43571
LINC01963	1.97138	2.20495	1.43273	2.31801	0.87826	-0.3256	1.24995	-0.31513	3.08201	2.1463	2.56638	3.12505	2.42053	2.12214	3.41534	3.38813

SMARCAL1	2.64213	2.70763	2.64144	2.85571	3.12045	3.02034	2.99418	2.71235	3.12272	3.15683	3.32422	3.8148	3.33722	3.32014	3.75517	3.53576
IGFBP2	0.25198	0.60916	0.62914	0.43696	-1.49069	-2.10973	-1.82434	-2.49444	0.78869	1.02225	0.2987	-0.57546	2.41402	2.25231	3.0517	3.01077
IGFBP5	-3.15781	-3.32193	-3.12944	-0.32261	-2.77546	-3.32193	-3.32193	-3.12873	0.05417	0.73845	0.97058	0.58959	3.14868	2.70982	3.30243	3.05014
DIRC3	-0.44967	0.09899	0.3591	-0.58408	-3.32193	-3.32193	-3.32193	-3.32193	-3.06687	-3.10727	-3.09253	-2.14449	-3.32193	-2.98879	-3.10924	-3.10488
TNS1	2.28472	3.6865	3.99322	2.34186	-1.57035	0.25935	-1.58955	0.19132	-3.09106	-2.75642	-3.11445	-2.85995	-3.26675	-2.93148	-3.19096	-2.65946
AAMP	6.3199	6.10182	5.92052	5.8722	6.27472	6.22878	5.74303	6.0102	5.76782	5.7559	6.08269	5.23411	5.94804	5.96624	5.7094	5.86729
PNKD	4.70048	4.646	4.69825	4.60016	5.80482	5.77746	5.0727	5.69423	4.48043	4.84586	4.8154	4.44161	4.08859	4.43483	3.98529	4.41273
TMBIM1	4.88402	4.9331	4.93713	4.85153	6.74274	5.77827	6.65206	5.56452	5.38023	5.69378	5.67714	5.59923	5.70193	5.56283	5.9718	5.90872
MIR6513	5.98094	6.09078	6.0485	6.68187	8.95293	6.67055	9.21526	6.41279	7.31635	7.81885	7.89199	7.92967	8.78749	8.73184	9.30967	9.09497
CATIP	-0.87074	-0.97039	-0.97401	-0.53583	-0.0041	1.2256	-1.30986	0.74015	-2.54937	-1.57759	-2.28483	-2.34903	-3.10889	-2.6133	-2.84907	-2.84018
CATIP-AS1	1.20363	0.92482	1.08198	1.52672	2.03492	3.1877	0.49437	2.56962	-2.2082	0.09179	-0.95743	-1.78651	-2.48636	-1.27681	-1.77979	-1.75899
SLC11A1	-2.78858	-2.94207	-3.1766	-2.01854	-1.32142	0.26717	-1.09724	-0.81563	-3.04936	-2.493	-2.77265	-2.99492	-3.22421	-2.81706	-2.89794	-3.20118
CTDSP1	3.80128	3.85543	3.90128	3.7292	5.63983	5.81872	5.06391	5.64036	5.06436	5.41529	5.38798	5.2005	3.90644	4.30743	4.80671	5.03431
VIL1	0.6188	0.16307	0.58032	0.80107	6.46064	5.22571	6.38528	5.2685	0.96436	0.50533	0.49867	0.7245	2.204	1.86281	1.98755	1.63685
USP37	1.00946	0.76051	0.96411	1.11578	2.62547	1.68945	3.15829	1.8864	2.04613	1.39042	1.37916	1.95524	3.01874	2.72863	2.92557	2.6166
CNOT9	4.11255	3.91691	3.98013	4.05199	4.66762	4.26932	4.88374	4.17611	4.18294	3.81563	4.07225	4.22919	4.85576	5.18139	5.00069	5.15204
PLCD4	0.98438	0.59507	0.31507	0.52204	0.85435	0.65192	0.74894	0.76968	0.58424	0.27476	0.62191	1.00445	0.85599	0.09857	1.13526	1.24613
ZNF142	2.96781	2.96068	2.58882	2.69778	3.33976	1.90984	2.99313	2.10783	2.71984	2.48272	2.76352	2.58536	2.94453	2.78664	3.61871	3.46291
BCS1L	3.86042	3.59549	3.39536	3.35736	4.46836	4.01322	3.81767	4.10679	4.11661	3.63913	4.07885	3.0241	4.0641	3.70154	3.81222	3.72383
RNF25	4.29855	4.28447	4.22218	4.25078	4.08984	4.23634	3.93431	4.13953	3.91925	3.84522	4.16943	3.58223	4.16599	4.05167	3.85343	3.94404
STK36	1.90823	2.05665	2.20138	2.5559	2.33656	2.32802	1.9687	2.42231	1.21481	1.50334	1.94389	2.38594	1.70642	2.44063	2.13466	2.7193
TTLL4	3.30982	2.93105	2.90807	3.15124	3.69396	2.50207	3.61154	2.5931	3.25547	2.8043	3.02783	3.5583	3.63539	3.41684	3.80678	4.02962
CYP27A1	3.42861	3.97217	3.92637	4.56377	2.62703	3.63664	2.12106	3.90248	0.38923	0.83267	0.93044	1.44068	3.51257	3.58277	3.88324	3.68038
WNT10A	-0.90999	-0.29536	-0.98785	-0.69694	0.84046	-3.02329	0.66566	-2.46462	-3.32193	-2.4132	-2.55053	-2.12417	-3.32193	-3.32193	-3.03839	-3.03271
CDK5R2	-2.37454	-0.68732	-1.81954	-1.0272	0.2198	0.62225	-0.56123	0.58369	0.86913	1.20679	0.65504	0.77695	-3.00875	-3.32193	-3.32193	-3.32193
IHH	-2.85345	-3.32193	-2.38909	-2.34473	2.88745	2.67444	1.88015	3.65047	-2.78992	-2.0117	-3.32193	-2.87441	-2.94279	-3.32193	-3.32193	-3.32193
NHEJ1	1.97981	1.9777	2.00141	1.79577	2.97856	2.15916	3.0454	2.23336	2.38845	2.07601	2.19343	2.61699	3.34375	3.44057	3.5784	3.10059
SLC23A3	-0.50741	-1.54831	-1.04986	-0.90149	-1.04557	-0.84264	-1.73128	-0.6476	-2.51328	-1.1414	-1.12898	-1.09611	-1.36942	-1.37323	-1.52727	-1.00109
CNPPD1	4.74493	4.52248	4.56195	4.26825	5.50725	5.23924	4.98776	5.17481	5.39467	5.52551	5.59864	4.74333	5.27825	5.25912	5.54643	5.51264
RETREG2	4.80378	4.84162	4.79799	4.72144	5.95144	5.1855	5.45301	5.05179	5.56352	5.63008	5.65288	5.50064	5.32147	5.26331	5.768	5.81186
ZFAND2B	3.25379	2.84394	3.08635	2.56048	3.836	4.14441	3.3217	4.10188	2.62216	2.42414	2.62867	2.33953	3.05936	2.78603	2.81978	2.46453
ABCB6	1.38955	1.2178	1.22867	1.43172	0.63835	0.51268	0.14031	0.7773	2.6365	2.91613	2.84602	2.83583	2.80092	3.48902	3.34353	3.95915
ATG9A	4.599	4.43079	4.45066	3.91695	5.8847	5.36042	5.36623	5.2439	4.86657	4.75523	4.78204	4.87642	4.91196	5.14528	5.67611	5.99174
ANKZF1	4.31557	4.13721	4.31181	3.55542	2.92776	3.3675	2.40855	2.95956	3.09057	3.47192	3.12643	3.15991	2.84896	3.04766	2.56087	3.18367
GLB1L	1.61257	1.32229	1.39721	1.2268	2.1782	2.81242	2.35999	2.38265	0.21152	1.06808	0.98964	1.43829	1.24115	1.5912	1.50447	1.9458
STK16	3.49522	3.23779	3.34812	2.97472	3.63933	3.72012	3.12508	3.73658	2.70557	3.32982	3.03221	2.85974	2.66363	2.98858	2.60607	3.06539
TUBA4A	6.31324	5.9475	5.83678	5.46443	6.21888	5.9704	5.74165	5.88894	0.29624	-0.00138	-0.96035	0.07861	3.44523	3.03476	3.85313	2.97819
TUBA4B	1.18516	0.9774	0.75626	0.46944	1.74037	1.01247	0.33994	1.13127	-2.70123	-3.32193	-3.32193	-3.32193	-2.02948	-2.26313	-2.10482	-1.61846
DNAJB2	3.71309	3.68454	3.91467	3.16547	5.29216	4.73645	4.73267	4.53421	3.55814	3.80186	3.66144	3.14366	3.49534	3.06269	3.85693	2.85116
PTPRN	-1.75003	-1.41823	-1.84639	-1.33458	-1.54205	-1.37718	-1.81521	-2.1654	-2.41609	-1.42745	-2.49118	-1.94167	-3.21947	-3.32193	-3.32193	-3.32193
DNPEP	4.4171	4.25476	4.16121	4.24447	4.50471	4.27264	3.9509	4.3366	4.91235	4.81078	5.05612	4.6607	3.88439	3.81906	4.04612	4.25117
DES	-1.9574	-0.18806	-0.86827	0.37062	-1.44399	-0.79159	-1.57684	-0.86361	-2.52928	-1.98925	-2.33722	-3.32193	-2.52259	-2.52478	-3.32193	-2.93525
SPEG	1.47634	2.00205	2.29285	1.95	-1.43947	-0.58813	-1.80555	-0.65281	0.78823	1.68774	1.78427	1.01033	-2.38335	-1.48561	-1.39085	-0.36472
GMPPA	4.06751	4.09655	3.96694	3.92818	4.65457	5.06516	4.16946	5.0205	4.27834	4.13084	4.27357	3.7738	2.93314	2.79975	2.88676	2.97635
CHPF	5.78409	6.18993	6.06886	5.66545	7.27644	8.21546	6.32812	8.10704	6.042	6.53037	6.68564	5.95279	3.84107	4.20579	4.23025	5.30066
TMEM198	0.83666	1.94514	1.54482	1.08017	4.2282	4.99725	3.65274	4.92908	1.38931	2.32371	2.35647	0.63741	1.15231	2.22737	1.88476	3.13205
OBSL1	0.12865	2.26155	1.85626	1.92233	1.89063	4.28234	1.37156	4.2408	4.17293	5.1287	4.99158	4.56458	1.00762	2.60614	2.7753	3.8803
STK11IP	2.66821	2.63326	2.81966	2.6462	3.23221	3.38921	2.7394	3.21352	2.85707	3.31584	3.63048	3.14745	2.75318	3.40484	3.38307	3.94206
SLC4A3	-3.11722	-3.09405	-3.08239	-3.32193	-0.18943	0.16003	-1.32613	-0.27701	2.87377	2.99896	3.389	2.38695	0.23254	0.13706	1.15384	1.5345
EPHA4	-2.82524	-2.29486	-2.34097	-2.84777	-3.26031	-3.32193	-3.06877	-3.32193	1.58018	2.13094	1.78327	2.82844	-3.32193	-2.92049	-3.18693	-2.83542

SGPP2	-0.76472	-1.1029	-0.96174	-0.36679	-0.77557	-0.31172	-0.68688	-0.61389	-2.97662	-3.32193	-1.77197	-2.58931	-2.25595	-2.25864	-3.03254	-2.78183
FARSB	5.49479	5.28404	5.00637	5.20284	5.0156	4.92615	5.48563	5.03151	5.33533	4.61553	5.06656	4.84344	5.33696	5.10638	5.0174	4.60494
ACSL3	3.63302	3.74884	3.65645	4.01828	4.91532	5.38267	5.47243	5.27978	4.68404	4.18999	4.35756	4.37998	4.91283	5.55422	5.32065	5.45824
SCG2	-2.42947	-2.13192	-2.3115	-1.83562	-0.52694	1.87919	0.15379	1.68862	-0.91234	1.63238	-0.10786	3.64933	6.95249	6.97291	7.41049	7.61763
AP1S3	2.28772	2.33959	1.95514	1.65159	2.88188	1.61702	3.36892	2.04597	0.80119	1.14656	0.13052	0.19868	3.38106	3.70961	4.10924	3.80147
WDFY1	4.04	4.4462	4.22392	4.43009	4.63422	3.2463	5.18855	3.23598	4.06037	4.53138	4.21925	4.7615	4.17005	4.66714	4.5881	4.57285
MRPL44	4.74334	4.93307	4.46861	4.91913	4.90452	4.48616	5.19756	4.3676	4.71228	4.56889	4.72551	4.70201	5.31557	5.33542	4.98364	4.70103
SERPINE2	1.44297	2.12176	1.91507	3.43005	0.93576	-0.83921	1.55766	-1.21007	3.85069	4.32612	3.95674	5.07741	5.46764	5.89848	4.79285	5.54604
CUL3	3.84272	3.87459	3.66897	4.07769	3.77825	2.99476	4.30536	2.96695	3.72361	3.23799	3.38636	3.53347	4.08802	4.26464	4.24623	4.33239
DOCK10	2.98821	3.01489	3.06351	3.49501	-3.32193	-3.22308	-3.32193	-3.20633	2.07072	2.02515	2.41263	2.97695	2.64996	2.5987	3.09276	2.86941
IRS1	3.78913	3.86556	4.23253	3.54311	4.90301	2.30937	4.14154	2.32911	5.14916	5.11486	5.11982	4.49839	5.10572	4.66945	5.52585	5.40269
RHBDD1	1.7049	1.73112	1.60121	1.82851	2.41294	2.41534	2.46969	2.19902	1.74127	1.67864	1.59711	2.22934	2.24078	2.33413	2.31886	2.3058
COL4A4	-1.11777	-0.81961	-0.87293	-0.37087	-3.01954	-3.01402	-3.03547	-3.19325	-2.87336	-2.57285	-3.00722	-3.03142	0.52565	-0.34781	1.10058	0.75226
COL4A3	-1.71713	-1.82928	-1.72951	-1.71771	-3.17347	-3.2029	-3.01368	-2.93951	-2.94717	-3.20819	-3.20009	-3.10621	-1.80291	-2.14454	-1.88603	-2.15511
MFF	3.96205	3.90017	3.83829	4.16919	3.61918	3.63081	3.94726	3.62449	3.44187	3.38962	3.31765	3.89366	3.84861	3.64875	3.59413	3.21906
MIR5703	5.28193	5.68465	5.80545	5.39542	7.70368	5.29777	8.00141	4.05175	7.36121	6.79474	6.77104	2.01491	4.64805	4.8734	8.19245	7.62616
AGFG1	3.07922	2.90725	3.15209	3.24592	4.06418	3.01002	4.59706	2.72826	3.9194	3.5348	3.55924	4.44778	4.50067	4.09737	4.81979	4.43136
SLC19A3	-1.65081	-1.46774	-1.96979	-1.91337	1.96497	-0.20803	1.60884	-0.15707	0.20111	-0.53564	-0.17699	-0.49921	0.78258	0.02986	0.44928	-0.13588
CCL20	6.12625	6.43352	5.19615	6.42938	7.8573	5.86388	7.05078	5.63198	-0.99126	-0.37947	-2.18617	-0.30009	-0.44646	-0.74603	-3.32193	-3.32193
DAW1	-0.07891	-0.10219	-0.65985	-1.90495	-2.78634	-2.97989	-3.00346	-3.32193	-0.42291	-1.49342	-1.04759	-1.51105	-1.60806	-2.29843	-3.32193	-3.32193
PID1	2.37176	2.61674	2.3198	2.81986	-0.71425	-3.32193	-0.80141	-2.97209	2.47786	2.06818	2.16149	1.79419	-1.23329	-1.93185	-3.32193	-1.8359
DNER	5.05466	5.72878	5.75509	6.20508	-2.09525	-2.18671	-1.21292	-2.36708	6.0744	6.45362	6.14226	6.71905	1.76182	1.99835	1.26146	1.81016
FBXO36	-0.85597	-0.88461	-0.76253	-0.20106	-1.04588	0.04948	-1.41326	-0.7578	0.1751	0.09427	-0.71427	0.88997	0.4147	0.59734	-0.67592	-0.23659
SLC16A14	-3.11164	-1.76739	-2.11946	-2.64902	-3.32193	-2.76027	-3.32193	-2.8643	-0.47364	-0.85217	-1.84263	-1.27791	0.09921	2.08002	0.1811	1.40996
SP110	2.49562	2.66231	2.44825	3.30189	0.88201	1.10937	1.62597	0.88604	3.00196	3.01438	3.06698	3.98098	0.84844	1.31201	1.04579	1.68806
SP140	2.26708	2.2242	2.3803	2.67441	-1.49851	-1.0602	-1.03104	-0.30202	2.99469	3.21686	3.01942	3.73842	-0.4292	0.30995	-1.04403	-0.19529
SP140L	2.94418	3.04547	2.88573	3.30457	2.40707	2.57557	2.80703	2.77564	3.11939	3.11388	3.27243	3.59927	2.68434	3.0923	2.6258	2.89387
SP100	2.43844	2.41497	2.55907	2.93352	1.31024	0.82105	2.04231	0.43406	3.56728	3.84208	3.86184	4.56883	2.71942	2.5502	2.40684	2.30216
HMGB1P3	2.09011	2.41478	2.60186	3.4692	0.57653	0.70369	2.29922	1.03433	3.07855	3.67078	3.95434	4.74711	3.21408	3.14627	2.5245	2.80433
CAB39	4.41758	4.46209	3.80332	4.38557	4.219	2.40923	3.83849	2.89468	4.81714	5.25462	3.22344	5.13523	4.39201	4.90134	3.25014	4.43245
BANF1P3	3.02305	3.23961	3.75352	1.79848	4.24165	5.25865	4.44299	5.08975	2.94564	3.56762	3.67017	2.37134	0.97358	1.2758	1.8102	2.63086
ITM2C	5.75069	6.26246	6.42198	5.94582	7.21505	6.52035	6.58276	6.20101	6.15113	6.48524	6.31317	5.54996	4.94452	5.35134	5.63706	6.03815
C2orf72	1.68878	2.00482	2.14459	1.69126	7.25509	5.54037	6.72682	5.68902	-0.4491	0.80893	-1.00961	-0.21052	1.00753	1.06906	1.30261	1.14039
PSMD1	5.0478	5.00269	4.87176	5.364	6.34137	5.67733	6.75332	5.97102	6.083	5.51758	5.7427	5.84989	5.95432	5.92109	6.01059	6.04784
ARMC9	2.90823	3.03671	3.20888	3.57838	1.1161	0.75874	1.19514	0.67822	1.87095	2.41597	2.56898	2.84146	2.53814	2.01153	2.48132	2.29676
B3GNT7	-1.45365	-1.0318	-1.45108	-1.29013	2.40971	0.84049	2.20516	0.74549	1.27672	2.60822	2.01804	3.45729	-2.92192	-0.75272	-1.67042	1.72416
LINC00471	-1.82818	-2.08375	-0.73202	-2.50287	-0.19868	0.33685	-0.36193	0.62306	-0.44748	-1.58418	-0.58979	-2.66757	-0.21951	-0.14654	-0.56063	-0.96142
MIR1244-1	11.5413	11.2727	11.244	11.3573	10.1328	10.1852	10.7568	10.1785	11.5566	10.639	11.0066	10.6599	12.2135	10.9007	11.4387	10.2022
PDE6D	2.97475	3.00847	3.29915	3.54664	2.78431	2.93438	2.63244	3.00641	2.69912	2.83014	2.31629	2.31593	3.04808	3.40912	2.09086	2.6596
COPS7B	3.20717	2.92257	2.79731	2.66326	3.00408	2.8817	2.83962	2.57506	3.66974	3.02797	3.49994	2.69058	3.78684	3.40699	3.7392	3.48541
DIS3L2	1.65998	1.63074	1.68092	1.51444	2.1174	2.29793	2.10808	2.42007	2.03373	2.13887	2.34441	2.23108	1.87865	2.12056	2.27267	2.34401
TIGD1	0.85986	0.29668	0.51709	1.29364	1.38483	1.68211	1.64886	2.01388	1.0176	1.12519	0.50289	1.49508	2.22845	2.96086	2.16203	2.37243
EIF4E2	3.87019	4.1106	3.80609	4.14765	4.27823	4.16454	4.52254	3.97287	4.18091	3.81062	3.83051	3.84089	4.78128	4.78589	4.5268	4.67844
MIR5001	2.65741	2.63177	2.48978	3.44328	3.73171	3.74848	3.54738	2.71411	2.46278	3.50159	2.04266	2.58161	3.7524	4.98209	4.27546	4.55943
EFHD1	6.55518	6.35585	6.28502	6.19563	1.59266	1.31682	0.99927	0.89088	-1.33793	-1.75547	-2.48172	-2.18091	-0.74163	0.12773	0.72585	0.283
GIGYF2	2.58514	2.58395	2.53661	2.65207	3.62991	2.55124	3.92841	2.56755	3.111	2.94656	3.35868	3.44022	3.50965	3.07666	3.8452	3.59786
SNORC	1.40027	1.661	1.72575	1.43108	2.08872	2.75768	2.28112	2.70387	1.10379	1.19266	1.3296	2.06306	2.60915	2.24385	2.99547	2.84116
NGEF	-2.58759	-2.68163	-2.84362	-2.28826	2.48804	2.6219	1.84177	2.67247	-2.85132	-1.85734	-1.87901	-1.64299	-1.42294	-1.19516	-0.92225	-0.41963
ATG16L1	2.60432	2.61392	2.46687	2.54492	3.58687	2.90378	3.95485	2.73315	2.96862	2.37484	2.44845	2.51941	4.46995	4.16224	4.13841	4.30116
DGKD	3.09617	2.9188	2.8788	2.88164	2.69357	1.98657	2.6702	1.99384	2.59154	2.38521	2.43494	2.50583	3.63835	3.61729	4.34792	3.93697

USP40	2.41369	2.1837	2.41164	2.4472	2.80615	2.54995	2.94765	2.55203	2.38467	2.25265	2.29955	2.91956	2.38496	2.40307	2.70268	2.78041
UGT1A8	-3.32193	-2.53632	-2.50307	-3.32193	6.64879	5.20327	7.17714	5.00901	-2.51491	-3.32193	-3.32193	-3.32193	2.61763	4.11831	4.12113	3.43695
UGT1A10	-2.74499	-2.68792	-2.65972	-2.62531	6.35845	4.94125	6.85639	4.7612	-2.66978	-3.32193	-3.32193	-3.32193	2.31962	3.79686	3.8117	3.13499
UGT1A9	-3.32193	-2.53857	-2.5054	-3.32193	6.6436	5.1979	7.17236	5.00536	-2.51722	-3.32193	-3.32193	-3.32193	3.15924	4.5436	4.61913	3.98524
UGT1A7	-3.32193	-2.81796	-2.79454	-3.32193	5.85787	4.41436	6.38639	4.22051	-2.8029	-3.32193	-3.32193	-3.32193	1.85463	3.3326	3.35559	2.66319
UGT1A6	-3.32193	-2.90364	-2.88362	-3.072	5.61441	4.1885	6.11715	4.01406	-2.71552	-3.32193	-3.32193	-3.32193	1.62362	3.11465	3.13709	2.44664
UGT1A5	-3.32193	-2.52039	-2.48664	-3.32193	6.68856	5.24082	7.21716	5.04654	-2.49866	-3.32193	-3.32193	-3.32193	2.65467	4.15155	4.15345	3.47426
UGT1A4	-3.32193	-2.56421	-2.53185	-3.32193	6.64639	5.18383	7.15359	5.0008	-2.54338	-3.32193	-3.32193	-3.32193	2.59237	4.07419	4.08199	3.39929
UGT1A3	-3.32193	-2.52534	-2.49175	-3.32193	6.68047	5.23938	7.20971	5.04008	-2.50371	-3.32193	-3.32193	-3.32193	2.64321	4.13998	4.14698	3.46272
UGT1A1	-3.32193	-2.84682	-2.82451	-2.79719	6.60114	4.6844	6.95954	4.44069	-2.83248	-3.32193	-3.32193	-3.32193	1.88498	3.38338	3.40153	2.78284
HJURP	4.98943	4.54983	4.69871	4.05427	4.63165	3.50516	4.68135	3.84823	5.23146	4.54532	4.89472	3.82341	5.70155	5.50969	5.50898	5.26956
MSL3P1	3.22761	2.97505	3.19879	3.60204	2.23309	1.44255	2.33948	1.42949	3.59686	3.71982	3.72954	4.22776	4.73506	4.75856	4.33648	4.23663
TRPM8	-2.9494	-3.17107	-2.77065	-2.51965	-3.32193	-3.06059	-3.32193	-3.32193	-2.56926	-2.36068	-2.30947	-2.23978	-0.62983	-0.5671	-0.59385	0.40784
SH3BP4	3.95161	3.7632	3.78634	3.3053	-2.52302	-3.32193	-3.1823	-2.6017	4.76046	4.12283	3.42276	3.69479	4.84072	4.13251	4.91854	4.3272
AGAP1	2.36601	2.63075	2.82384	2.06777	3.2498	1.14396	3.06982	0.86301	3.27583	3.24622	3.20583	3.31837	2.9687	3.27796	3.78395	3.65241
GBX2	-3.32193	-3.32193	-3.32193	-3.32193	-1.04561	0.81635	-2.15087	0.46051	2.65445	1.45993	1.98371	-0.06172	2.43444	2.01859	1.85637	1.75346
ACKR3	0.71962	1.4657	1.97372	1.00663	-3.12535	-3.32193	-3.32193	-3.32193	-3.32193	-3.32193	-3.32193	-3.32193	-3.32193	-3.32193	-3.32193	-3.32193
COPS8	4.10158	4.07627	3.82324	4.25586	3.72172	3.42023	3.98343	3.38602	4.16213	4.20812	3.73392	4.25923	4.69949	5.02043	4.10232	4.13632
MLPH	3.71537	3.53179	3.86919	3.62115	5.10728	5.3876	5.17167	5.09714	4.69091	5.03297	4.96768	5.19971	4.81574	4.5311	4.78475	4.60655
RAB17	-2.46488	-3.32193	-2.665	-3.32193	0.82192	1.60252	0.1955	2.06085	-1.70147	-0.46217	-1.43848	-1.66493	-2.5041	-1.55353	-2.15882	-1.15724
LRRFIP1	4.17057	3.75045	4.14127	3.82222	3.65563	2.39765	4.18038	2.35575	4.7538	3.62279	4.14779	3.66811	4.509	2.77514	4.28735	2.91692
RAMP1	4.65507	4.53945	4.63449	4.17345	-2.70441	-1.78203	-3.32193	-1.62405	-3.32193	-1.8265	-1.75894	-3.32193	-3.32193	-3.32193	-3.32193	-3.32193
UBE2F	3.95775	4.24717	4.24609	4.20061	3.46916	3.51265	3.59493	3.36679	2.7485	3.03041	2.64748	3.13145	3.11286	3.10537	2.59986	2.7079
UBE2F-SCLY	5.14055	5.02801	5.03911	4.83582	5.04981	4.71868	4.67604	4.69817	4.36253	3.8771	3.89937	3.45844	4.56555	4.23247	3.98621	4.10164
SCLY	2.72732	2.26072	2.18876	1.84085	2.6301	2.11109	1.92076	2.38241	1.94566	0.79852	1.29706	-0.02299	2.15313	1.75219	1.55989	1.61492
ESPNL	-0.98483	-1.47417	-1.35284	-1.91892	0.17199	-0.89153	-0.87338	-0.73873	-1.81061	-1.06837	-1.43478	-1.77545	-3.32193	-3.16748	-3.32193	-2.96297
ERFE	1.25183	1.60923	1.51316	0.35554	1.63613	1.40509	0.54486	1.2157	1.74759	1.17509	1.6707	-0.75061	0.69152	1.11724	1.41811	1.20487
ILKAP	2.73108	2.58438	2.50328	2.26386	3.58128	3.42293	3.39877	3.40422	3.01425	2.82067	3.13402	2.65169	3.27557	3.40454	2.65111	2.55153
LINC02610	-3.32193	-3.32193	-3.32193	-3.32193	0.54709	0.56283	-1.00324	0.45486	-2.37235	-2.12803	-2.06893	-2.73085	-3.32193	-3.32193	-3.32193	-3.32193
HES6	1.67184	1.8677	1.41843	0.27056	4.98286	5.17006	3.90707	5.00425	1.77465	1.63819	2.07208	-0.22009	3.95163	3.9493	3.94624	3.67149
PER2	-1.52165	-1.30753	-1.6715	-1.37728	1.86667	1.25226	1.7866	1.57173	1.29939	0.68949	1.12502	0.64684	1.3094	0.81253	1.5043	1.36625
TRAF3IP1	2.74553	2.44849	2.7595	2.71176	3.53268	2.80043	3.6915	3.15014	2.90793	3.00771	2.80653	2.93012	3.1122	3.57096	3.18391	3.50087
ASB1	2.70268	2.81236	2.68169	2.52586	3.68258	2.71599	3.42455	2.61286	3.20456	3.44585	3.07234	3.35022	3.09206	3.67968	3.25701	3.70261
TWIST2	-2.15855	-2.06397	-2.0181	-0.18022	2.04242	1.81817	1.31715	1.2692	2.33565	2.17744	2.56619	2.10166	1.52827	2.16593	1.61319	1.66223
HDAC4	0.02477	0.35556	0.02121	0.20841	1.19198	0.15773	0.48051	0.38024	-0.09418	0.40716	-0.32613	0.29666	1.04926	1.39451	1.5603	1.83258
NDUFA10	3.13775	3.0817	3.02132	3.03601	3.63657	3.61029	3.63598	3.58865	3.84174	3.738	3.77982	3.83422	3.82526	3.92363	3.57523	3.59801
COPS9	5.25845	5.26709	5.26926	4.68435	5.21537	5.19769	4.86435	4.90377	5.43569	5.19606	5.13241	3.90369	6.26299	5.873	4.24498	4.63667
GPC1	2.97344	3.12703	3.57594	2.98805	5.01587	4.57874	4.54799	4.74366	4.44316	4.20857	4.91977	3.85316	3.32739	3.35413	4.99788	4.57009
ANKMY1	0.59774	0.7144	0.77427	0.21235	1.93113	1.5033	1.33206	1.4991	1.98815	2.21571	1.98038	1.431	1.16371	1.09961	1.66008	1.72273
DUSP28	0.32296	0.59379	0.22157	0.53329	1.64639	1.86963	1.52287	1.85173	1.31245	1.28728	0.78726	0.73737	0.87791	1.10984	0.71224	0.86021
RNPEPL1	3.52414	3.98504	3.93492	3.30946	4.94739	5.1028	4.29243	4.99546	5.33491	5.47661	5.35109	5.23566	3.36488	3.52235	4.62054	4.53522
CAPN10-DT	0.09782	-0.075	0.15243	-0.17817	0.17641	0.11222	-0.31052	-0.10344	-0.80184	-1.89758	-1.17885	-1.34262	-1.30751	-0.59853	-0.41816	-0.22198
CAPN10	2.84649	2.82407	2.76134	1.71612	3.78779	3.94953	3.19736	3.83209	2.4677	2.59647	2.66677	1.4992	2.56877	2.87079	2.5534	2.66961
GPR35	-2.27399	-1.63249	-1.65827	-1.77335	2.85038	2.56019	2.80238	2.25702	-1.76122	-2.01704	-1.86886	-1.72542	-2.87998	-2.76002	-2.41608	-1.76868
KIF1A	-3.23405	-2.88632	-2.51944	-3.10957	-1.56744	1.17981	-1.92053	1.33367	-3.22027	-3.08107	-3.23099	-3.15961	-2.29809	0.36898	-0.39664	0.88505
MAB21L4	-3.32193	-3.32193	-3.32193	-3.32193	1.66894	1.75864	1.18219	2.4137	-3.32193	-2.97711	-3.32193	-3.32193	-3.32193	-3.32193	-3.32193	-3.32193
SNED1	-0.55124	-1.00763	-1.08293	-1.11897	-0.32914	-0.13881	-0.84066	-0.07558	-1.47854	-0.7344	-1.09928	-0.53526	-0.88131	-0.62532	-1.32248	-1.06215
MTERF4	1.4825	1.16836	1.02412	1.09148	1.48706	1.68659	1.52112	1.77628	0.83135	1.22444	1.01249	1.1822	1.70897	1.8887	1.23376	1.52693
PASK	1.58397	1.03445	1.03185	0.84497	1.94866	1.25326	1.39439	1.58205	1.45987	0.51672	1.30893	0.35319	1.60489	1.94612	1.5876	1.51813
PPP1R7	4.52664	4.4826	4.56467	4.57979	4.14508	4.67963	4.30022	4.61846	4.87538	4.49988	4.70869	4.335	4.4847	4.48475	4.11655	3.98045

ANO7	1.11868	0.9641	0.54223	-0.37527	1.31303	1.1156	0.82086	0.87531	1.2127	1.29416	0.73732	0.74516	-0.61003	0.09506	0.38964	0.81754
FARP2	1.19977	1.25742	0.97762	1.23272	2.00222	1.31622	2.60084	1.7291	1.50028	2.33587	2.28212	2.95649	2.39837	3.46603	3.75797	3.71098
STK25	4.32815	4.3339	4.37906	4.09386	5.02012	4.71236	4.15077	4.57872	5.53741	5.33812	5.40273	4.55756	4.67734	4.64437	4.78528	4.89871
BOK-AS1	4.60652	4.70663	4.61782	2.55834	4.6417	2.41892	4.18901	2.13979	5.13071	5.07131	4.88189	3.27974	5.07742	4.65328	5.51888	5.47213
BOK	5.95741	5.88969	5.73097	4.59729	5.80562	4.87455	4.72805	4.56458	6.48351	6.49385	6.2707	5.31686	5.96621	5.70953	6.15312	6.332
THAP4	5.06896	4.41677	4.50741	3.7368	5.48555	4.40906	4.80979	4.0078	5.59445	4.66291	5.4692	4.02699	5.21033	3.73412	5.38626	3.76825
ATG4B	3.63636	3.34562	3.43396	3.26829	3.71261	4.26685	3.23412	4.38093	3.24655	3.12704	3.31254	2.85782	3.60015	3.61021	3.47626	3.34695
DTYMK	5.34567	4.92343	4.8573	4.2394	6.40368	5.55862	5.86906	5.55662	6.20313	5.3452	5.61982	4.0036	6.37947	6.15865	5.89792	5.58301
ING5	1.81537	1.70056	1.72234	1.47203	2.73851	3.04239	2.54801	3.1318	1.6357	1.53672	2.06188	1.50571	2.64159	2.66233	2.38235	2.38911
D2HGDH	0.81667	1.14549	0.90814	-0.09666	2.02324	2.2844	0.99555	2.50545	2.02313	2.69593	2.58356	1.49457	2.12106	2.38687	2.74961	2.71287
LINC01881	-0.50863	0.6263	0.0068	0.08956	1.02071	-0.19741	1.44691	0.07876	0.49376	0.38016	0.02292	0.91901	0.37467	0.36992	0.83871	0.83719
RPL23AP88	-0.70638	-1.77657	-0.49901	0.86906	2.74566	3.00914	2.03187	3.44315	-0.52259	-0.39708	0.42187	0.91278	0.52535	1.24408	-0.12645	0.3423
CNTN6	0.34082	0.37868	0.80337	1.08134	-3.32193	-3.32193	-3.32193	-3.32193	-3.32193	-3.32193	-3.32193	-3.32193	-3.32193	-3.32193	-3.32193	-3.13858
TRNT1	3.11767	3.013	2.85438	3.34339	1.66195	1.48645	2.10037	1.23785	1.35181	1.67463	1.84678	1.86134	2.34188	2.16381	1.49879	1.66031
CRBN	2.56559	2.51737	2.59943	3.0399	1.23586	0.98344	1.64849	1.0876	0.69661	1.30185	1.50614	1.88838	1.85278	1.39545	1.47128	0.83218
SUMF1	3.37136	3.17541	3.56135	3.59111	3.10694	2.4612	2.98282	2.18112	1.37379	1.66364	1.62472	2.01707	2.35623	1.29458	1.71208	1.46945
SETMAR	1.39175	1.67877	1.3352	1.65486	0.7084	0.31494	0.75206	0.42462	0.78125	-0.29801	0.80359	0.44173	1.14647	0.40376	1.0948	0.88059
ITPR1	2.42235	3.47134	3.93434	3.42354	1.25104	-0.0527	1.25495	0.01316	1.01713	0.91655	0.11788	1.19599	-1.31553	-2.20117	-1.45666	-1.55294
BHLHE40-AS1	3.76372	4.0899	4.52638	3.08784	2.44629	2.26535	2.51756	2.05072	2.9884	2.88206	1.90296	1.1455	1.35709	1.2064	1.91908	1.73708
BHLHE40	6.95152	7.18546	7.59072	6.64633	4.84677	6.05495	5.04564	5.40582	5.45078	5.45322	4.49051	4.69009	4.08278	3.88142	4.53227	4.39933
ARL8B	5.12299	5.23229	5.2499	5.58062	4.76308	4.49216	5.40878	4.23187	4.44429	4.23024	4.18759	4.61482	5.38103	5.31825	5.00517	5.1207
EDEM1	4.61227	4.61554	4.51321	4.78825	3.9837	3.69921	3.62052	3.40844	3.99506	4.07231	3.10143	4.29751	3.97858	3.51614	2.89229	3.31812
LMCD1	1.6247	1.28547	1.32015	0.61096	1.58626	0.50082	1.56394	0.59016	-0.61411	-2.11306	-0.77511	-2.07705	1.39179	0.61046	2.13801	0.6644
SSUH2	-2.42256	-2.96864	-2.65676	-2.15308	2.07935	3.05065	1.57747	2.70265	-3.32193	-3.01347	-3.32193	-3.32193	-3.32193	-2.49661	-3.01622	-3.32193
OXTR	0.01616	0.21587	0.28728	0.01667	2.48318	0.41605	3.14516	0.40819	7.00096	6.41481	7.42506	6.2533	6.2057	4.85518	6.44764	4.67354
RAD18	3.03996	2.6148	2.44868	2.1168	2.84409	1.55733	3.24469	1.59528	2.91729	1.81073	1.95905	2.15089	3.36893	2.92201	2.73507	2.28461
SRGAP3	-2.74453	-2.64528	-2.53213	-2.93165	-1.05452	-0.8465	-1.27258	-0.61878	-2.12839	-2.09256	-2.43462	-1.78334	-3.02925	-2.6654	-2.51955	-2.57314
THUMPD3-AS1	2.45099	2.24811	2.34957	2.70773	1.41613	1.62704	1.49218	1.98879	0.38696	0.52186	0.36916	0.97402	1.87414	1.52159	0.93111	0.98762
THUMPD3	4.03467	3.99819	3.98645	4.2158	3.33046	2.79298	3.44484	2.74942	3.2782	3.00424	3.13865	3.39355	3.59312	3.4957	3.50195	3.56283
SETD5	3.98753	4.06972	4.04088	4.40194	3.30194	4.03497	3.44961	4.23043	3.86857	4.00115	3.95131	4.58912	3.68852	4.03723	4.1551	4.3639
LHFPL4	-3.32193	-3.32193	-3.09229	-3.32193	-2.47064	-3.12374	-2.69545	-2.17619	2.82954	3.56399	3.36445	3.24198	-3.32193	-3.32193	-3.32193	-3.32193
MTMR14	3.79939	4.09518	3.91662	4.04654	3.56709	3.62703	3.43781	3.43569	3.62062	3.61156	3.61065	3.67815	2.83613	2.78545	3.37911	3.49975
BRPF1	3.89117	3.7021	3.66001	3.34899	2.90157	2.68864	2.33921	2.65206	2.54806	2.59879	2.77976	2.17192	2.83784	2.89631	3.19182	3.1597
OGG1	2.59375	2.49981	2.4006	2.51339	1.92417	1.85335	1.38145	1.84329	2.32091	2.33236	2.14636	1.67064	3.10103	3.02394	2.95486	2.97491
CAMK1	3.24605	3.49535	3.35157	3.29656	1.18219	1.5916	0.62131	1.84626	3.22038	3.46251	3.2023	2.69081	4.39393	4.2572	4.17198	4.16357
TADA3	4.74754	5.28943	5.37854	5.13198	4.01789	4.24685	3.812	4.0034	4.08171	4.79051	4.56473	4.47162	3.11181	4.00882	3.94777	4.21
ARPC4	6.30187	6.32093	6.32367	6.04514	5.14012	5.06732	5.15707	4.93174	5.22806	5.11079	5.29064	4.90862	5.05376	5.01005	5.39433	5.31986
ARPC4-TTLL3	5.44558	5.46519	5.55833	5.4039	4.6659	4.35193	4.83829	4.19867	4.6807	4.56533	4.81411	4.46464	4.60356	4.64087	4.85212	4.89025
TTLL3	-0.4413	0.03491	0.92663	0.56569	0.78398	1.70691	0.00238	1.98418	-0.85572	-0.22068	-0.06905	0.04251	1.46331	1.61246	0.95406	1.70664
RPUSD3	3.74344	3.36412	3.19276	3.20819	3.21659	3.17097	2.54688	3.13744	3.01415	3.00373	2.5688	2.28508	2.76481	2.96415	2.40109	2.81546
CIDEC	-2.83344	-2.39245	-1.37167	-2.72861	3.67987	4.19794	3.85863	3.4505	-3.32193	-2.84831	-3.32193	-2.85516	-3.32193	-2.92733	-3.32193	-3.32193
JAGN1	5.11146	5.05403	4.99739	5.07248	5.24758	5.36421	5.22148	5.2192	3.99967	4.4111	4.21396	4.24755	4.40992	4.6644	4.08292	4.20929
IL17RE	-2.3249	-1.71347	-1.95841	-2.44369	-2.28112	-1.73034	-2.62618	-3.062	1.06154	2.14452	1.45835	1.37078	-0.84914	-0.29932	-0.75815	0.39485
IL17RC	4.43335	4.27962	4.409	4.06513	3.11679	3.70745	2.2116	3.61819	3.34123	3.51413	3.85247	3.16426	2.83144	3.12889	3.75315	4.05303
CRELD1	1.22602	1.90424	1.99786	2.0516	2.80402	3.73895	2.341	3.46871	1.12263	2.72194	2.2956	2.80211	1.26391	1.91549	1.75509	2.08739
PRRT3	0.95042	1.10884	0.69359	1.16294	0.35604	0.92064	-0.35752	1.18872	0.84381	1.168	0.75882	0.53094	1.06438	0.93734	-0.19878	0.66043
PRRT3-AS1	2.29377	2.92969	1.88522	2.62381	2.13578	2.54872	1.29855	2.71477	2.95237	3.37	2.81561	2.25501	4.03039	3.38694	2.30082	2.46664
EMC3	4.35966	4.4345	4.23529	4.56748	4.38571	4.47301	4.42704	4.31647	3.15543	3.36714	3.09983	3.1986	3.48449	3.68912	2.99299	3.61681
EMC3-AS1	2.57319	2.12725	2.09059	2.2538	0.70179	1.10398	0.79787	1.58884	-0.11113	-1.82951	-1.09536	-0.22973	1.66804	1.69248	1.18569	1.5431
FANCD2	2.37458	1.9462	2.10181	1.51598	2.72324	1.64401	2.66523	2.06241	2.33412	1.20568	1.51311	1.13679	2.95274	3.10265	3.47514	3.27

FANCD2OS	2.53429	1.73026	2.1115	1.65558	2.52435	1.60411	2.45693	1.8894	2.11667	0.67877	1.47143	1.0295	2.45693	2.69919	2.97129	2.95862
BRK1	7.3138	7.45612	7.51416	7.40594	6.74803	6.60976	6.86486	6.29988	6.83959	6.90664	6.64571	6.31006	7.16791	7.02749	6.59868	6.74453
VHL	4.98445	5.05777	5.13639	4.28679	4.15489	4.52419	4.26209	4.3606	3.81366	4.27077	4.08006	4.54502	4.34378	4.57026	4.63193	4.85209
IRAK2	4.68263	5.05155	4.71634	5.29385	3.99564	2.43751	3.69537	2.41893	4.02345	5.08412	4.1392	5.98326	4.22704	4.39173	3.48339	4.38764
TATDN2	3.96761	3.70195	3.85367	3.74535	4.19117	2.96151	4.09207	3.12075	3.66448	3.24825	3.52709	3.71366	4.06534	3.78096	4.2915	4.10431
GHRL	-0.59065	-0.93773	-1.13166	0.06956	-1.70082	-0.28215	-2.05649	0.05551	-2.11326	-2.87556	-2.4908	-1.6717	-0.96458	-1.24163	-1.36881	-1.34534
GHRLOS	0.7542	-0.12952	-0.14301	1.03268	-0.73554	0.36932	-0.21474	0.58683	-1.95409	-1.46477	-1.38919	-0.42537	0.08261	-0.30795	-0.33547	-0.66456
SEC13	5.30967	5.28959	5.18185	5.0994	4.94053	5.37844	4.53233	5.09325	4.5641	3.98129	4.65691	3.97059	4.42045	4.29703	4.18989	4.153
HRH1	2.56258	2.62363	2.77214	3.06486	-3.08522	-3.13101	-2.84489	-3.32193	3.24327	3.21775	2.65312	3.308	2.50878	1.81978	2.04323	2.20371
ATG7	3.62422	4.01923	4.07014	4.08502	1.66333	1.72168	2.12596	1.23078	2.50757	2.58223	3.11828	3.34751	2.4978	2.20581	2.68188	2.45544
VGLL4	2.79781	3.13816	3.51856	3.06577	2.27155	2.17086	2.73505	1.52663	2.09223	2.66374	3.36615	3.1985	2.31675	2.27224	3.42628	2.61564
TAMM41	2.08383	1.82988	1.65865	1.82357	0.44663	0.21354	0.33524	0.76957	1.31641	0.94976	1.01928	0.79839	1.00631	0.93352	0.35117	0.43587
TIMP4	1.42604	1.36814	1.35161	2.08971	-0.12177	-3.32193	-0.67373	-1.90055	-3.32193	-3.32193	-3.32193	-3.32193	-3.32193	-3.32193	-3.32193	-3.32193
PPARG	4.10524	4.17444	4.1742	4.26896	3.08321	3.06388	3.34525	2.93584	1.26097	2.32779	1.92454	3.47273	2.91147	3.34736	2.96539	2.89492
TSEN2	2.08271	1.43181	1.42214	1.47499	1.84862	1.76367	1.94803	2.10524	0.28016	0.17646	0.25392	0.44294	1.81227	1.43187	0.87898	0.84586
MKRN2OS	0.04761	-0.4756	-0.85607	-0.91988	-1.43312	-0.51322	-1.40634	0.69077	-2.01176	-1.71503	-2.10857	-1.73151	-1.45027	-0.82685	-1.10586	-1.2133
MKRN2	5.04764	4.86126	4.85936	4.96663	3.35921	3.62452	3.55464	3.51659	3.63849	3.50426	3.55573	3.86465	4.30784	3.86663	3.78509	3.37367
RAF1	4.32407	4.22285	4.16182	4.35946	3.7616	3.8319	3.56265	3.98874	3.90019	3.50638	3.30225	3.75275	3.9895	4.04839	3.72221	3.88658
TMEM40	-1.088	-0.6483	-1.03739	-0.96411	-2.01123	-3.32193	-2.17116	-2.71945	4.11454	4.10282	4.10324	3.11295	5.48178	4.99273	4.0236	3.47709
CAND2	-1.43756	-1.20441	-1.37577	-1.18499	-2.18217	-2.42702	-1.81021	-2.09042	1.26637	1.35568	1.90301	1.27715	-1.58601	-1.49663	-1.85805	-1.35717
SNORA7A	5.48443	4.88534	4.73119	4.31008	3.81672	4.50346	3.71479	4.22461	3.4768	3.51184	3.68093	2.80347	4.42856	3.67134	3.42786	3.30858
IQSEC1	3.20709	3.44757	3.68926	3.37361	2.83801	2.30294	2.53483	2.56597	2.54958	2.82631	2.72662	3.00398	2.62521	2.93281	4.08419	3.43992
NUP210	5.16892	5.13236	5.15018	5.12658	4.84496	4.10677	3.99462	4.31065	2.92971	3.36826	2.41884	2.70699	4.65936	4.56993	5.06532	5.18313
HDAC11	1.87049	1.97945	2.11468	1.53679	3.46488	5.26887	2.87642	4.62918	2.02865	2.76321	2.30493	1.63661	1.15706	1.10675	2.04019	1.84775
FBLN2	-2.75257	-2.39202	-2.66818	0.33409	-0.58089	-1.23057	-0.89807	-1.23065	-2.86255	-2.93119	-2.90599	-2.50267	-3.32193	-3.32193	-3.1153	-3.32193
WNT7A	-3.32193	-3.32193	-3.32193	-3.32193	-1.6622	-3.32193	-0.76008	-3.32193	-0.66214	0.13358	-0.11207	-1.24687	-2.98213	-3.32193	-3.10553	-3.1011
CHCHD4	4.47654	4.12139	3.95525	3.68001	3.63825	2.88021	3.4014	2.94596	3.63463	2.483	3.10178	2.20971	3.91271	2.28888	3.19761	1.77984
TMEM43	5.29396	4.98558	5.29662	5.12548	4.83774	3.80345	4.83773	3.80508	4.90215	4.64818	5.13066	4.78111	4.76008	3.76928	4.70445	3.80833
XPC	3.2936	3.31068	3.07334	3.2092	3.10863	2.84761	2.6358	2.47115	4.12552	3.82156	4.34525	3.94524	2.57097	2.44399	2.71612	2.83599
LSM3	4.87051	4.9217	4.84827	4.60397	4.1569	4.15295	4.43146	3.98443	3.44087	3.77764	3.8803	3.19604	3.93592	4.35695	3.75512	3.71844
SLC6A6	4.08882	3.75413	4.05972	3.50199	2.9153	2.07932	2.79621	1.83924	4.96273	4.69114	4.95501	4.77676	4.15634	3.70741	4.71284	4.30281
GRIP2	-1.08245	-0.67178	-0.73007	-0.22023	-2.58587	-1.71464	-2.21212	-1.8435	-1.39271	-1.20508	-1.28214	-1.24498	-0.46564	-1.3945	-0.40612	-1.15042
CCDC174	3.31057	3.22272	3.12703	3.56864	1.86798	2.54535	1.7829	2.70885	1.80714	2.15595	2.09093	2.57921	2.05988	1.85192	1.59327	1.69195
FGD5-AS1	4.11131	4.29332	4.36337	4.60364	4.35641	3.75459	4.68598	3.7222	4.65172	4.47045	4.50832	4.89014	4.72675	5.17592	4.59884	4.83589
NR2C2	3.15772	2.91654	2.95638	3.09663	2.32975	2.16818	2.43166	2.2421	2.61564	2.20408	2.41424	2.72882	3.15727	3.16543	3.15803	3.25364
MRPS25	4.53411	4.28324	4.06351	4.17073	3.1822	3.32797	3.03122	3.56227	2.93702	2.25864	2.77634	2.35508	3.48878	3.30463	2.87592	2.87697
RBSN	2.22219	1.7417	1.89599	1.68575	2.05277	1.58625	2.10981	1.45093	2.78168	2.31207	2.56218	2.58009	2.60584	2.10408	2.91938	2.66536
CAPN7	2.74104	2.76039	2.82725	3.143	3.06269	1.96304	3.57634	2.30207	1.87242	2.36557	2.36211	2.65918	2.33711	2.5689	2.4209	2.44113
SH3BP5-AS1	3.70211	3.85694	3.71406	4.12627	-0.17187	0.20658	-0.6082	0.48981	2.11009	2.43995	1.98429	2.25444	1.33842	1.04661	1.04588	0.96314
SH3BP5	4.41525	4.52577	4.34705	4.74369	0.15635	0.20989	-0.04252	0.62549	2.90206	3.29268	2.82744	2.92617	2.00651	1.57038	1.73329	1.68439
METTL6	2.1918	2.40148	2.478	2.5084	1.80643	1.17384	2.31285	1.39858	1.49342	1.61975	1.39658	1.99125	2.25443	2.52394	1.98242	2.30394
EAF1	3.55025	3.64899	3.62773	3.91693	2.83805	2.03231	3.30963	1.91577	2.5894	2.08903	2.05572	2.57416	3.10191	2.87427	2.70371	2.8422
EAF1-AS1	2.40841	2.22539	2.40387	2.69917	1.97712	0.70087	2.40531	0.55314	1.71997	1.02848	1.19889	1.54921	2.16683	1.76343	1.90132	1.7258
COLQ	-1.71555	-1.69595	-1.29219	-0.6444	-2.84191	-2.23261	-3.09108	-2.79364	-2.80374	-2.39874	-2.34895	-1.76684	-3.32193	-2.95429	-3.08622	-2.87533
HACL1	3.66397	3.6574	3.41064	3.79604	3.61206	3.36421	3.79718	3.13791	2.83947	2.76297	2.94102	2.45172	3.1501	2.79536	2.71192	2.72688
BTD	2.24207	2.41808	2.51982	2.52624	3.47701	3.40131	3.4354	3.23206	2.61828	2.37623	2.76709	2.40176	2.16445	2.37492	2.10472	2.66371
ANKRD28	3.00649	2.97952	3.09144	3.25474	2.02597	1.01487	2.46701	1.25837	3.96022	3.6236	3.4709	4.13751	3.38513	3.22617	2.95038	2.9986
RN7SL4P	5.62347	5.24404	6.29157	1.46311	7.00169	8.41188	8.17076	8.10904	5.56054	6.48632	5.95345	7.54618	5.53199	5.48776	4.84475	4.7851
DPH3	3.84298	3.78867	3.93587	4.12342	3.41645	3.23891	3.82314	3.32565	2.71331	2.60101	2.90758	3.14024	3.48485	3.4122	3.24837	2.65737
OXNAD1	2.16773	2.11455	1.69505	2.12429	1.45087	0.98529	1.74198	1.06986	-0.0612	-0.1354	-0.34749	-0.53219	0.9967	1.07585	0.65174	0.5405

RFTN1	4.48095	4.42471	4.31127	4.44247	-2.50703	-2.11127	-2.17559	-1.67755	4.40205	4.69363	4.75136	4.94571	4.61503	4.71119	4.62049	5.43728
PLCL2	1.63989	1.59055	1.62478	1.90171	-3.23236	-3.32193	-3.13191	-3.32193	1.35293	1.36312	1.32544	1.90532	-3.32193	-3.32193	-3.32193	-2.94969
PLCL2-AS1	2.8253	2.88415	2.58811	3.25568	-3.32193	-3.32193	-3.32193	-3.32193	1.58532	2.06729	1.67911	2.58844	-3.32193	-3.32193	-3.32193	-3.32193
TBC1D5	2.18778	2.23725	1.76748	2.15572	1.56593	0.2035	0.41612	0.92143	2.19882	2.15598	0.60389	2.32477	2.45545	2.62275	1.23195	2.25432
SATB1	-0.35293	0.04125	0.23461	0.99456	-3.21927	-3.32193	-3.32193	-3.22588	-3.1398	-2.73473	-3.15853	-2.7969	-3.32193	-3.32193	-3.32193	-3.16752
EFHB	-2.51809	-2.44457	-2.40852	-2.05163	-2.61402	-2.86041	-2.89106	-3.32193	-2.59937	-2.39678	-2.08808	-2.05056	2.73508	2.96057	2.38579	1.51873
RAB5A	4.50181	4.85604	4.65365	5.01355	3.60734	3.51084	4.30291	3.43061	3.86231	3.77859	3.77557	4.78182	4.56379	4.5655	4.74013	4.18343
PP2D1	2.55661	2.82263	2.50487	3.24168	1.19017	1.37195	2.25714	1.12799	1.1438	1.14151	1.05365	2.55436	2.98776	2.83026	2.80169	2.17404
KAT2B	1.42011	1.44812	1.43025	1.82509	0.76813	-0.09589	1.38265	-0.02212	2.10273	2.0115	1.61072	2.54424	3.71319	3.01807	3.14078	2.71505
SGO1	3.28553	2.94499	3.03047	2.91612	2.52323	2.08436	2.63087	2.21963	2.46475	1.40143	1.66289	0.88679	2.72863	2.74416	2.56248	2.25977
SGO1-AS1	1.67028	1.23493	1.55611	1.28745	1.16476	0.66067	1.05207	0.70601	0.96599	-0.01685	0.37752	-0.86806	0.88665	0.95222	0.7081	0.76735
ZNF385D	-3.02571	-2.55343	-2.97708	-2.66616	-3.28743	-3.24112	-3.24717	-3.22732	-3.32193	-3.24476	-3.32193	-3.32193	-2.70306	-2.09887	-1.7308	-2.40379
HMGB1P5	6.22858	5.63613	5.51157	5.27525	5.40828	5.66714	5.19956	5.69899	6.54156	5.97923	5.73385	5.98062	5.38554	5.493	5.01709	5.21667
UBE2E2	3.87801	3.68611	3.8615	3.87812	3.28913	3.18016	3.43427	3.00414	3.06459	3.02617	2.88627	3.02596	3.06746	3.18486	2.73629	2.82503
UBE2E1	5.80668	6.15612	6.06185	6.459	3.86445	3.97479	4.47136	3.85643	4.52298	4.7757	4.62714	4.95419	4.8058	5.01017	4.12764	3.91223
NKIRAS1	3.29916	3.64569	3.37099	3.84548	2.23071	2.32931	3.02344	2.51979	0.83868	1.81818	1.50169	1.80307	1.95666	3.37991	2.48114	2.81005
NR1D2	3.00383	2.94273	2.9358	3.35061	0.68394	-0.44673	1.26475	-0.01708	1.80513	1.58176	1.5987	2.56753	2.10428	1.34845	2.26247	1.44134
THRB	-1.94114	-1.94319	-2.92496	-1.89565	2.13115	1.38122	2.50211	1.25673	0.08862	0.23167	0.12144	1.2567	-0.11429	1.27299	1.09324	1.65252
RARB	0.68842	0.43172	0.71547	1.28224	1.477	1.38108	1.59445	1.48373	1.88247	2.24977	2.08136	3.27983	-0.43163	-1.11861	-1.39363	-0.61454
TOP2B	4.82727	4.79411	4.75554	5.12198	4.77013	3.75754	5.25614	3.7445	5.33095	5.19394	5.15938	5.90467	4.92701	5.32938	5.42043	5.49505
MIR4442	5.70674	5.66696	6.04382	4.14145	7.30848	4.4824	7.35204	4.55598	7.70599	7.77504	7.75558	3.73094	5.97082	6.39572	7.14634	7.25463
NGLY1	2.72186	2.67301	2.57631	2.91627	2.51145	2.38081	2.73756	2.35998	2.70602	2.83376	2.34837	2.91975	2.46242	2.0668	1.7164	1.65415
OXSM	3.31693	3.23049	3.11785	3.42508	3.39165	2.79695	3.02558	2.73018	3.15085	3.1717	2.82493	2.76941	2.99116	2.69173	2.13495	2.46545
NEK10	-2.46595	-2.13747	-2.0933	-1.577	-1.7228	-1.92254	-1.82902	-1.77261	-1.57617	-1.22417	-1.25549	-0.95836	-1.23914	-1.55523	-1.84754	-0.82023
SLC4A7	2.79416	3.01293	2.46261	3.32687	3.56523	3.44145	4.62149	3.30344	2.61202	3.09894	2.26021	3.37728	3.9121	4.7224	3.73922	4.94863
CMC1	2.72667	2.65937	2.8098	3.11669	1.65263	1.89807	1.95674	1.93497	1.83155	1.7608	1.59584	2.03612	1.95176	1.51322	1.13015	0.7059
AZI2	3.08922	3.36187	3.31441	3.85932	2.64436	2.33453	3.05187	2.30501	2.72725	2.81861	2.53255	3.58235	2.70833	2.50167	2.23933	2.19705
RBMS3	-1.48133	-1.16243	-0.59608	-1.09345	-3.29623	-3.26148	-3.32193	-3.32193	-1.17025	-1.14891	-0.71652	-0.91789	-0.89526	-0.83002	0.64776	-1.47319
TGFBR2	3.7634	3.14116	4.23914	3.95929	6.50755	4.28671	7.0564	3.89121	6.16257	5.19556	6.56145	6.57476	7.20603	4.61817	7.62153	5.58449
STT3B	5.40619	5.29622	5.32068	5.68082	6.02569	5.15736	6.6064	5.1676	5.43385	5.458	5.39015	5.94816	5.54564	5.4925	5.62749	5.5837
OSBPL10	1.63553	1.39179	1.39286	1.25745	1.39172	-0.07125	1.7513	-0.19956	2.45178	2.16775	2.25275	2.55911	0.75194	0.16597	1.28253	0.67703
ZNF860	-1.28908	-0.78452	-0.47405	0.31153	-0.52283	-2.03331	0.01478	-2.4354	0.5679	0.56187	-0.43314	1.07507	-0.77475	-0.52664	-0.73792	-1.2346
GPD1L	1.64265	1.50552	1.02428	1.86798	2.20041	2.96892	3.22933	2.55266	1.91203	1.54644	1.53412	2.06199	2.7298	2.88609	2.50743	2.4866
CMTM8	0.21132	0.96719	0.05638	1.12856	5.469	6.03572	5.33173	6.12284	1.89204	2.36069	1.789	1.72082	2.69385	3.22307	2.00479	2.44748
CMTM7	3.75793	3.56696	3.19304	3.26813	3.30074	3.50526	3.6236	3.21406	3.29519	3.29818	3.12733	3.27967	4.79126	4.78613	4.71128	4.67932
CMTM6	4.0467	4.25025	4.17144	4.68364	4.82827	4.11525	5.45936	3.98274	4.19812	4.0547	3.88247	4.59843	5.18882	5.11219	4.7656	4.51027
DYNC1LI1	3.71691	3.59065	3.55288	3.78589	3.37842	3.09093	3.75716	3.06433	3.33896	2.72362	3.00822	3.15105	3.90244	3.31338	3.82313	3.30485
CNOT10	3.51389	3.53664	3.49273	3.73582	2.73489	2.66319	3.06474	2.49655	2.81635	2.44864	2.53376	3.21735	3.01435	2.80247	2.86225	2.76326
CNOT10-AS1	3.86689	4.09687	3.93482	4.00712	2.87084	2.45644	3.30659	1.95003	3.04103	2.70579	2.87705	3.83895	3.22713	3.44509	3.17069	3.50011
TRIM71	-3.32193	-3.18403	-2.52193	-2.90138	2.97757	2.08036	3.25942	1.70346	-3.32193	-3.32193	-3.32193	-3.32193	-3.32193	-3.32193	-3.32193	-3.32193
GLB1	4.60001	4.70873	4.80064	5.04499	4.81636	4.81789	4.63239	4.54519	3.95041	4.15646	4.11335	4.39273	4.34953	4.40806	4.38546	4.49111
TMPPE	0.10899	-0.27782	-0.50883	-1.74838	1.77938	-1.26335	1.30725	-1.23154	0.13862	-0.58615	0.39655	-0.56682	1.7176	1.08168	1.87524	1.77521
CRTAP	5.83778	5.84729	5.92838	5.61194	5.6861	5.77089	5.42462	5.56998	5.79497	5.57791	5.7364	5.71753	5.42941	4.89815	5.96351	5.63602
SUSD5	-3.32193	-2.71611	-3.07874	-3.32193	0.1467	-1.71928	0.72061	-1.27452	-0.90961	-0.7176	-0.82219	-0.18663	2.42799	2.0791	2.48652	2.67272
FBXL2	0.40637	0.83368	1.14506	0.76385	-0.24459	0.01617	0.01074	-0.06614	0.39222	0.6903	0.72476	1.45925	1.0969	0.73575	1.04455	0.90984
UBP1	3.62041	3.64356	3.57115	3.52783	3.25033	2.10061	3.33288	2.19933	3.51502	3.40359	3.44514	3.57627	3.5469	3.48133	3.30434	3.22976
CLASP2	1.48236	1.09679	1.24296	1.57494	2.2533	0.67918	2.70281	0.5984	1.75437	0.69191	1.21413	1.27318	2.38089	1.64117	2.23823	1.95947
PDCD6IP	4.34505	4.59613	4.61868	4.89372	4.04797	3.4302	4.59857	3.3171	4.26488	4.072	4.06482	4.76944	4.71498	4.75568	4.62334	4.49131
ARPP21	-3.32193	-3.32193	-3.32193	-3.32193	-3.32193	-3.32193	-3.32193	-3.32193	-3.32193	-3.32193	-3.32193	-3.32193	-1.19299	-0.67047	0.36679	-0.35303
STAC	0.21327	-0.01198	0.2733	-0.37963	-3.21762	-3.32193	-3.10185	-3.32193	2.75028	2.5662	2.10041	2.56669	3.66996	3.99918	4.30287	4.00487

TRANK1	0.54697	0.86588	0.78983	1.72611	-1.00541	-1.44697	-0.64841	-1.87069	2.05093	2.52392	2.23823	3.73105	-1.21142	0.29572	-0.27009	1.5687
EPM2AIP1	1.41643	1.748	1.63161	2.50653	1.3574	1.44202	1.74209	2.10162	0.59448	1.0503	1.29563	2.02996	1.25681	1.83471	1.67415	1.73735
MLH1	3.22852	3.23617	3.0577	3.35795	3.67402	3.21691	3.89416	3.34145	3.64832	3.44993	3.61002	3.55137	4.00525	4.28292	3.77997	4.09271
RPL29P11	3.43655	3.81635	4.01546	2.45334	2.39471	2.73103	2.6852	2.56473	3.42423	4.45974	3.44434	2.77178	1.43043	1.72323	1.70612	1.35786
LRRFIP2	2.35725	2.59495	2.39193	2.73421	1.99175	2.11003	2.49404	2.22626	2.73919	2.01347	2.34851	2.21458	2.95719	2.80477	2.85791	2.18726
UBE2FP1	1.60961	1.03479	1.47203	1.56045	1.3411	-0.03245	1.51361	0.62818	2.04837	1.4745	0.78141	0.6612	1.96669	1.26556	2.4778	0.36297
GOLGA4	3.87506	3.79202	3.71899	5.07033	4.23401	3.74746	4.90092	4.06417	4.04153	3.85945	3.73354	4.7862	4.70779	4.5612	5.22078	4.72
TCEA1P2	2.86707	2.772	2.84884	2.03608	2.41658	0.94026	2.83526	1.34787	4.12314	3.83042	3.50721	3.45708	4.42685	3.75931	4.56905	4.33963
C3orf35	-1.062	-1.88822	-1.55873	-2.13559	-2.6049	-0.54054	-3.01645	-0.51253	-2.95047	-2.53043	-2.71459	-2.54077	-0.13368	-1.6577	-2.53647	-1.52888
ITGA9	-2.96431	-2.61575	-2.7915	-2.54773	-2.5091	-1.14949	-2.98555	-1.63013	-3.03743	-3.19704	-2.27333	-2.62394	-2.87059	-2.95143	-2.03954	-2.60776
ITGA9-AS1	-2.76928	-2.3832	-2.45049	-2.01559	-1.58671	-0.07478	-1.98567	-0.0374	-2.97565	-3.02908	-2.75288	-2.79336	-2.31788	-2.32046	-1.53489	-1.88463
CTDSPL	2.12374	2.31749	2.25219	2.43509	2.781	3.58179	3.00668	3.47527	2.17644	2.1668	2.21722	2.6454	2.8421	3.15745	3.54791	3.5902
VILL	-3.15863	-3.13985	-3.32193	-2.94055	-2.01388	-1.1093	-2.02662	-1.60791	-0.83727	-0.5858	-0.30816	-0.73721	-3.32193	-3.32193	-3.16563	-2.76826
PLCD1	-0.19495	-0.23933	-0.03819	-0.1602	1.04521	2.15296	0.21161	1.78163	1.75836	2.04434	2.23941	2.1424	-0.39131	0.54823	1.66659	1.72807
DLEC1	0.9789	0.56471	0.64771	0.67115	0.99824	1.88562	0.75705	2.31365	0.64213	0.67715	0.62892	0.3389	0.57099	0.84242	0.67979	0.40739
ACAA1	2.67607	2.55734	2.42743	2.57862	3.35615	3.57483	3.00389	3.8651	2.68878	2.82002	2.90029	2.70934	2.41362	2.68784	2.77982	2.73211
MYD88	2.97868	3.21288	3.23457	3.41103	2.12287	2.0053	2.0212	1.76947	3.39894	2.68319	3.45204	3.25455	3.5559	2.89453	3.92446	3.37988
OXSR1	3.87675	3.96224	4.0957	3.9723	3.00001	2.1879	3.42937	2.13761	4.36719	3.84706	4.26386	4.3016	3.97395	3.51846	4.00599	3.40055
XYLB	1.55216	1.19203	1.30735	0.95141	1.707	1.05847	1.60518	1.42846	1.21954	0.84389	1.20917	1.45625	2.21078	1.92042	1.948	1.89875
ACVR2B-AS1	-1.0892	-2.56304	-2.02254	-1.42475	-1.41499	-1.517	-2.95699	-1.4871	-2.54218	-1.33155	-2.13433	-2.41354	-1.54974	-1.4549	-0.41951	0.16748
ACVR2B	0.10577	0.36073	0.38839	0.59114	0.6997	0.31195	0.89715	-0.01271	1.48187	1.82247	1.11313	2.72564	1.57021	2.26265	2.85971	3.49076
EXOG	0.81909	0.37946	0.79679	0.6617	0.56765	0.74635	0.84175	1.42247	-0.21763	0.17849	0.11795	0.31155	1.33424	1.51056	0.93808	1.12012
WDR48	2.50232	2.66877	2.74817	3.14626	2.11448	1.57623	2.87136	1.45971	2.76522	3.42284	3.29848	4.09	2.25861	2.87769	2.74001	2.85618
GORASP1	3.15515	2.94278	3.00925	2.73598	3.5461	2.91902	2.98701	2.76222	3.5636	3.15265	3.66031	3.01536	3.3406	3.05192	3.21406	2.96791
TTC21A	-0.79797	-0.91269	-0.33156	-0.51491	-1.33548	0.26776	-1.18533	-0.14298	-0.96779	-1.05829	-0.43664	-0.39348	-1.15686	-0.00045	-0.31363	0.09334
CSRNP1	3.46139	3.50439	3.49281	2.84858	3.41738	3.41244	3.11904	3.2024	3.43593	3.53066	3.65053	3.5379	2.97041	2.70251	2.89368	2.88395
SLC25A38	3.92774	3.67993	3.50909	3.90639	-3.32193	-3.32193	-2.90314	-3.32193	4.1366	3.84802	4.01215	3.8166	4.2206	3.68477	3.45759	3.36614
SNORA6	4.63061	3.46743	3.81818	3.77884	2.6351	2.08998	3.04548	2.89455	2.28673	3.00543	3.10757	3.29941	1.73178	2.87229	1.35256	3.10884
SNORA62	3.5567	3.84137	3.66908	3.95131	2.45575	2.64492	2.27353	2.61671	2.07977	2.5777	1.70176	1.98079	3.00866	3.62945	0.63501	1.05665
MYRIP	-3.32193	-3.32193	-3.32193	-3.32193	-3.32193	-3.32193	-3.32193	-3.32193	-1.19103	-0.36679	-0.96926	-0.31891	1.01124	2.02222	2.09233	1.50961
EIF1B-AS1	-2.95937	-2.75534	-3.09636	-3.08273	-2.9397	-2.5377	-2.98086	-3.32193	-2.4499	-1.64203	-1.849	-2.35996	-1.43989	-0.5805	-0.86228	-0.57441
EIF1B	4.76636	4.70682	4.61647	5.02402	4.27219	4.36603	3.87282	4.23518	4.1685	4.40852	3.43602	4.13191	4.23988	4.64971	3.2739	3.62992
ENTPD3-AS1	-0.30809	-0.73663	-0.19045	-0.74579	-0.36019	0.51105	-0.14018	0.55753	-0.21478	-0.09836	-0.41181	-0.86968	-0.44109	0.31713	-1.07284	-0.83542
ZNF619	-0.31305	-0.26304	-0.8674	-0.33176	-0.01323	-0.8573	0.2526	-0.18917	0.58396	0.77251	0.03731	1.26197	1.06595	1.36157	0.78672	1.25211
ZNF620	0.72168	0.27351	0.40436	0.19993	1.81682	1.18848	1.92952	1.66487	1.05885	1.19318	0.78912	1.56166	1.72295	1.95161	1.96124	1.75972
ZNF621	1.6541	1.68142	1.95838	1.64426	2.3049	1.58165	2.71765	2.07782	1.91103	2.32978	2.31321	3.25287	2.00306	2.44494	2.37859	2.58764
ULK4	0.1411	0.00055	-0.25028	-0.52347	0.89809	0.48553	1.06566	0.38593	0.06508	-0.0865	0.14027	0.60435	-0.55163	-0.69967	-1.3072	-0.59053
TRAK1	2.52145	2.25094	2.19387	2.06433	2.44778	1.97847	2.3807	2.03771	3.20832	2.79267	3.2764	2.91263	2.06812	1.48869	2.32414	1.99749
CCK	-3.32193	-3.32193	-3.32193	-3.32193	3.40132	2.52241	2.99144	1.50844	-3.32193	-3.32193	-3.32193	-3.32193	-3.32193	-3.32193	-3.32193	-3.32193
VIPR1	0.22911	-0.58581	-0.4198	-0.34011	2.39517	0.59781	1.91809	1.00019	-1.3466	-0.79493	0.06668	-0.1566	-2.91118	-3.32193	-3.18396	-3.32193
VIPR1-AS1	-0.48945	-0.99341	-0.98014	-1.31601	1.41958	-0.93572	1.28802	-0.47054	-1.86993	-1.79982	-0.74889	-0.85455	-3.01859	-3.32193	-3.32193	-3.32193
SEC22C	2.10585	1.95193	2.19347	2.43183	3.16799	2.46079	3.4876	2.21671	3.17536	2.4769	2.92208	2.98192	3.82097	3.07201	3.63509	3.20556
SS18L2	4.30975	4.29497	4.28088	4.36149	4.22018	4.48205	4.30352	4.23844	4.15002	3.8305	4.00173	3.50124	4.59561	4.60992	3.93946	3.73829
NKTR	1.95389	1.97807	2.08324	3.0268	1.27548	1.88711	1.57741	2.24912	1.22346	1.41685	1.19899	2.26597	2.06759	2.03088	1.78722	2.13124
ZBTB47	2.46443	2.74185	2.50926	2.90904	1.42718	1.58764	0.88254	1.89274	3.63083	3.84712	3.85759	4.02248	2.12484	2.76512	2.68486	3.34138
HIGD1A	6.30987	6.42653	6.31049	6.80282	6.60749	5.85227	7.09429	5.70087	6.14491	5.53156	5.87433	4.99758	6.63439	6.34783	5.65534	5.27821
KRBOX1	-2.71082	-3.32193	-1.27777	-2.10056	-1.09047	-1.0792	-0.75553	-1.64581	-2.38112	-2.99504	-2.97348	-2.73696	2.58709	2.63558	1.8276	1.40479
KRBOX1-AS1	-3.32193	-3.32193	-2.42527	-3.32193	-1.73312	-1.64101	-1.42822	-2.42263	-3.32193	-3.32193	-3.32193	-3.32193	1.56036	1.54138	1.07995	0.93362
POMGNT2	4.16448	3.98826	3.85856	3.68291	4.48464	4.28629	3.9789	4.24729	4.22735	4.117	4.28147	3.91139	3.08907	3.29228	3.4431	3.53569
SNRK	1.46316	1.34102	1.16795	1.63388	1.57543	0.26495	2.1363	0.49479	2.51961	1.88836	2.19177	2.72078	2.26403	1.39261	2.56222	2.32305

SNRK-AS1	1.42604	1.69423	1.35161	2.34033	0.89374	0.90972	2.05554	0.53995	2.66784	1.582	1.16609	2.35139	2.42482	1.46771	2.44234	2.18375
ANO10	3.72986	3.81815	3.78343	3.97474	3.41937	3.59251	3.58589	3.64751	4.16026	3.69998	3.90573	4.24464	3.20656	2.93797	3.57642	3.36253
ABHD5	3.50218	3.26748	2.76472	3.35492	2.54502	1.99421	2.88474	1.98936	3.03659	2.24016	2.36291	2.65379	2.79561	2.56232	2.83422	2.69493
TCAIM	0.67828	0.54108	0.65293	0.92069	1.52157	1.65609	2.1884	1.84169	0.82503	0.85077	0.81171	1.63427	1.76403	1.34835	1.41119	0.91421
C3orf86	-1.48258	-1.11478	-1.05335	-0.1301	1.17811	3.90931	0.78628	3.47777	-1.62245	-2.62451	-1.4457	-1.30206	-0.6093	-0.40703	0.27239	0.30136
ZNF445	0.14211	-0.06098	-0.1931	-0.20355	1.79251	1.43462	1.81266	1.79715	1.1081	1.07231	0.99708	1.69711	1.2517	1.57059	1.7464	2.01767
ZNF852	-0.19838	-0.85308	-0.6347	-0.82675	0.53575	0.69866	0.70141	0.22726	-0.65789	-0.70439	-1.00108	-0.85524	-0.03228	0.0254	0.06637	0.23337
ZKSCAN7	-3.07772	-3.32193	-3.32193	-3.32193	-3.21313	-2.6809	-3.32193	-2.79711	-0.70246	-0.36616	-0.79169	-0.42094	-0.38708	0.85867	-0.00278	0.89831
ZKSCAN7-AS1	-0.71168	-3.32193	-3.32193	-0.42548	-0.90586	-1.87786	-1.2593	-3.32193	1.30686	0.11736	0.75434	0.77695	0.97583	2.36427	0.91648	2.03078
MPRIPP1	-1.1318	-1.9435	-1.50102	-1.12067	-1.54731	-3.32193	-2.58618	-2.43621	0.59291	1.15734	-0.0204	-0.49805	-0.73095	-1.63244	-1.42594	-0.33008
ZNF197	1.01104	1.19436	1.16792	1.58292	-0.08515	0.37229	0.22723	0.7388	2.02127	1.95696	1.94209	2.68436	1.71799	1.97198	2.03836	2.01825
ZNF35	1.99141	1.88687	1.80065	2.19807	2.2188	1.89704	2.72882	1.80618	1.9862	1.83058	2.01975	2.17652	2.29798	2.45136	2.19533	2.14504
ZNF502	-3.32193	-3.32193	-3.32193	-3.32193	-3.32193	-3.32193	-3.32193	-3.32193	-3.32193	-3.32193	-3.32193	-3.32193	0.59634	1.4579	-0.05842	0.35633
ZNF501	-0.84544	-0.60607	-1.22018	-0.99732	-2.9027	-3.05806	-2.68422	-3.32193	-0.10376	0.25221	-0.14887	0.34105	0.80602	1.53837	1.22421	1.30054
KIAA1143	3.42301	3.33698	3.03784	3.32357	2.81525	2.22677	3.03322	2.47484	4.1911	4.20618	3.6134	4.82582	4.13313	3.97047	3.79055	3.93895
KIF15	2.60683	2.29176	2.20899	1.95677	2.07387	0.78109	2.29616	1.19116	3.17158	2.18028	2.41914	1.96884	2.94143	2.67923	2.98616	2.28457
TMEM42	2.23197	1.89452	2.25622	2.08146	1.4499	2.24752	0.41814	2.27071	2.50503	2.74981	2.9273	1.80216	1.87297	1.86796	1.45846	1.39835
MIR564	4.43077	4.48165	4.06428	4.5255	4.02063	4.95362	3.44492	4.43996	5.30189	5.55688	5.75451	3.36112	3.77439	4.07537	4.24938	4.28078
ZDHHC3	3.26094	3.20547	3.03073	3.18393	3.19575	2.89185	3.20942	2.56937	3.97728	3.77227	3.58472	3.95003	3.69927	4.03205	3.72971	4.12512
EXOSC7	3.72484	3.31393	3.35364	3.12504	2.99919	3.51842	2.95099	3.33274	3.78325	3.41468	3.87486	2.53553	3.46606	3.32815	3.01624	2.72165
CLEC3B	3.63128	3.65932	3.44857	3.69564	3.15378	3.63059	3.25414	3.03623	4.03091	3.3397	3.92925	2.59121	3.73402	3.64288	2.96565	2.64818
CDCP1	3.55351	3.8424	4.09706	3.32086	0.36785	-1.71306	0.98665	-2.22774	6.68162	6.74312	6.56414	7.7083	-1.94979	-2.06091	-2.30408	-1.53771
RPS24P8	4.07867	3.39252	3.83604	1.81236	1.72152	3.46354	1.66159	3.10374	3.25176	4.73999	3.44628	3.05937	1.77718	1.56733	1.31978	2.20843
TMEM158	7.82128	7.78463	8.62043	7.13941	-0.37202	-0.33548	-0.60071	0.28709	5.72232	5.36799	5.34301	4.25494	0.80185	-2.3201	0.21743	-0.54613
LARS2	3.35697	2.90651	2.9068	2.97408	3.28825	2.13023	3.22376	1.86522	3.54543	2.87384	3.71806	2.99739	2.72118	2.56818	3.10979	3.00121
LIMD1	2.34454	2.28405	2.52225	2.0154	3.64553	3.44447	3.73641	3.38051	3.70503	3.41881	3.60262	3.76289	3.40045	2.66424	4.03988	3.36397
LIMD1-AS1	3.54402	3.34735	3.71503	3.17019	4.18335	4.6551	4.61714	4.55628	4.16734	3.79027	3.98011	4.44645	4.21914	3.57932	4.66497	4.01401
SACM1L	2.51237	2.21721	2.34416	2.61753	2.93886	2.08814	3.58065	2.04632	2.59722	2.11595	2.68033	2.46836	2.99069	2.10805	2.81441	2.13614
SLC6A20	-3.1627	-3.32193	-3.32193	-3.32193	-3.32193	-3.32193	-3.17277	-3.13442	-3.32193	-3.16809	-3.15729	-3.32193	0.37585	1.62662	2.38234	2.20817
LZTFL1	1.34245	1.78699	1.76315	2.06413	-0.3213	-0.28767	0.66809	-0.86596	0.39528	1.31639	1.36664	2.14136	0.61069	0.79964	1.59571	0.40061
FYCO1	2.16073	1.78222	0.97136	1.35407	3.03182	1.07821	1.83905	1.76122	4.21164	3.7731	2.65137	3.48762	3.26225	2.88654	2.45458	3.07655
CCR1	-1.49098	-1.14921	-0.80753	-1.01556	-3.15753	-3.32193	-3.32193	-3.32193	-0.11119	-0.68808	0.09967	-0.18231	-3.32193	-3.32193	-3.32193	-3.32193
CCR5	-3.32193	-3.01481	-2.32076	-3.32193	-3.32193	-3.32193	-3.32193	-3.32193	-0.58844	-0.81407	-0.62729	-0.5972	-3.32193	-3.32193	-3.32193	-3.32193
CCRL2	3.2139	3.28503	3.00004	2.89955	-2.80517	-3.32193	-2.76283	-3.32193	0.99	0.89561	1.10351	1.45629	-3.32193	-3.32193	-3.32193	-3.32193
LINC02009	3.82484	4.47889	3.90028	4.51578	-2.90499	-3.32193	-3.32193	-3.32193	-2.9371	-3.32193	-2.97431	-3.00078	-3.32193	-3.32193	-3.32193	-3.32193
RTP3	-2.65515	-3.32193	-3.32193	-3.32193	-3.32193	-3.32193	-3.32193	-3.32193	0.74046	-0.25754	0.58484	-0.87415	-3.32193	-3.32193	-3.32193	-3.32193
ALS2CL	1.43567	1.65928	2.07786	0.60136	1.73945	1.39503	1.22632	0.81669	0.52077	1.3006	1.6117	0.738	-1.79598	-2.70354	-3.00527	-2.58295
TMIE	-0.71168	-1.56152	-1.50614	-2.26929	-0.13098	0.93124	-1.00815	0.33803	-2.74218	-0.56824	-1.00951	-2.46853	-3.32193	-3.32193	-3.32193	-3.32193
CCDC12	3.20571	2.89684	2.98439	2.81246	3.09977	3.7918	2.85437	3.80368	3.52415	3.36034	3.61986	1.99445	2.73856	3.014	2.81798	2.35571
NBEAL2	3.41308	3.68057	3.41074	3.45765	3.98497	3.36199	3.29854	3.46755	3.39237	3.671	3.61873	3.7926	2.97323	3.1436	3.60312	3.78973
SETD2	2.6593	2.32922	2.48054	2.65965	2.71569	2.1365	2.9775	1.97452	3.14053	2.77323	3.00959	3.53016	3.31469	3.15396	3.77028	3.67349
KIF9-AS1	-0.96582	-0.97409	-1.25451	-1.25134	-1.21841	-0.7843	-1.00186	-0.32673	-0.69967	-0.23665	-0.53336	-0.45224	-1.52169	-2.08273	-1.6813	-1.13399
KIF9	1.33033	1.40401	1.20194	1.24961	1.27587	1.07867	1.56594	0.82593	0.97522	1.41763	1.49634	1.35902	1.43212	1.38774	1.34748	1.60969
KLHL18	2.32831	1.91644	1.61106	1.88994	2.37467	1.45035	2.49783	1.12288	2.72254	2.13102	2.79503	2.27163	2.92709	2.7358	3.26989	3.04602
PTPN23	4.76044	4.7448	4.75344	4.01965	4.48282	4.30981	3.86059	4.4836	5.02432	4.75707	5.02462	4.37449	3.48298	3.63553	3.98612	4.31966
SCAP	4.41767	4.31352	4.27519	3.97234	5.11772	5.40236	4.59619	5.39334	5.05938	5.35695	5.6506	5.09627	3.13343	3.75119	4.45925	4.73294
ELP6	3.97933	3.60201	3.35476	3.42976	3.34889	3.48655	2.99893	3.45874	3.55179	3.60712	3.60998	3.1779	3.56184	3.69149	3.07771	3.27823
CSPG5	-1.07915	-1.39846	-0.05369	-1.43716	1.6794	0.91024	0.93257	0.80669	-0.04717	0.35928	-0.13563	-0.43367	0.50896	0.24172	1.24748	0.1901
SMARCC1	4.59093	4.38353	4.4098	4.49035	4.30458	2.73752	4.52145	2.83586	5.64123	4.88542	5.4262	5.33166	5.15686	4.80715	5.81028	5.52942
DHX30	4.92206	4.59733	4.28551	4.18627	4.79994	4.5115	4.26609	4.57132	4.8964	4.50862	4.79918	4.18054	4.20611	4.15422	4.24779	4.3101

CDC25A	3.94557	3.34048	2.45483	2.56817	3.87338	2.85751	3.82553	3.35542	3.75833	2.4254	2.68862	1.33942	4.72587	4.87919	4.33788	4.368
ZNF589	0.09758	0.02168	-0.26397	-0.40237	1.33909	0.7707	1.41787	0.2471	0.66625	0.26064	0.372	0.47786	0.11126	-0.15025	0.01815	-0.07319
FCF1P2	2.20985	2.37304	2.40726	1.64387	2.7474	2.4902	3.23571	2.41285	1.80911	2.23922	1.8269	2.78971	2.43803	2.59062	2.66174	2.21628
NME6	2.719	2.54712	2.28594	2.34066	3.09124	2.54612	2.93407	2.41315	2.80457	2.27177	2.47835	2.22379	2.907	2.87439	2.46077	2.2791
PLXNB1	-1.00374	-0.89282	-0.4482	-0.35011	4.11333	4.02942	3.41241	4.06813	-0.58772	-0.63051	-0.44208	-0.3439	2.05897	2.34303	2.80687	3.07757
CCDC51	6.82889	6.73392	6.6557	6.16375	5.94757	6.57895	5.33239	6.50529	6.46909	6.43762	6.24344	4.57972	6.00072	6.29344	4.79398	5.00275
TMA7	8.1634	7.99658	7.9585	7.39685	7.13992	8.06367	6.57851	7.98604	7.85951	7.81726	7.49719	5.85262	7.31502	7.49227	5.71345	5.89758
ATRIP	2.63985	2.51957	2.14808	1.91465	1.86787	2.18023	1.61741	2.37266	2.95046	2.33001	2.29971	2.11114	2.05558	2.26265	2.09941	2.05528
TREX1	4.31121	4.40448	4.44936	4.04368	-0.02887	0.42239	-0.80147	0.77384	-0.64208	0.05389	0.14764	-0.88615	0.63966	0.75261	0.70885	1.29434
SHISA5	5.28562	5.38245	5.2743	5.29489	5.82192	5.73471	5.43673	5.42196	6.17487	6.20433	6.33649	6.26095	5.37203	5.65345	5.86848	6.05531
PFKFB4	2.1108	2.79932	3.40316	2.65002	0.79977	1.92624	0.47825	1.47275	4.33662	4.95395	4.25134	5.30979	1.37244	1.42726	1.73446	1.60372
UCN2	3.76215	3.4724	4.43173	3.89267	-2.24275	-1.45432	-2.33763	-2.62299	-0.06977	1.7153	1.49284	2.89775	1.1062	-0.66241	0.26393	-0.12305
COL7A1	6.41455	6.26971	6.94323	6.07797	0.67591	0.70577	-0.25249	0.38851	2.36517	3.22984	3.46997	4.22714	2.48623	2.38469	3.32856	3.5051
MIR711	8.49829	8.13403	8.84879	8.03943	2.40723	2.39577	1.96826	2.88515	5.00672	5.0696	5.69871	5.86261	4.20953	3.77107	5.61029	6.01963
UQCRC1	6.91432	6.82945	6.85447	6.80038	6.36909	6.43667	6.10965	6.25298	7.3548	7.47853	7.2567	7.46047	6.11288	6.13912	6.45468	6.37261
SLC26A6	3.13873	3.38696	3.4623	3.27699	3.53577	3.8853	2.97945	3.96872	2.54073	3.37348	2.77865	3.82393	2.26984	2.63967	2.76988	3.04901
MIR6824	3.89562	3.64899	5.0094	3.45765	4.79857	4.57083	0.32186	4.25917	0.61537	3.5825	1.40731	3.04878	3.76679	3.54106	1.29419	3.73598
CELSR3	-0.16098	0.00607	0.41445	-0.15512	1.66005	1.64701	0.98927	1.63274	3.41244	4.40958	4.1205	4.5099	0.89678	2.20929	2.18396	2.86052
MIR4793	2.66554	2.03744	1.70958	0.29417	3.69842	3.44938	1.77857	4.00144	5.75543	7.01275	6.87611	6.69035	2.31968	3.88014	5.09462	5.38843
NCKIPSD	3.51799	3.50432	3.71124	3.18059	3.23998	3.08252	2.62788	2.70122	4.62245	4.76973	4.44778	4.71492	3.25753	3.31265	3.67066	3.52239
IP6K2	2.62576	2.81224	2.91123	3.08104	3.13038	3.42761	2.53127	3.3887	3.08018	3.20199	3.49563	3.02836	3.03142	3.04151	2.80548	2.67866
PRKAR2A	4.50486	4.29254	4.21641	4.41318	4.82447	3.96418	5.12359	4.09881	5.20542	4.62258	4.99285	5.11805	4.77703	4.43192	5.12135	4.92004
PRKAR2A-AS1	1.90528	2.05999	1.83762	0.78733	3.28946	0.60633	2.72069	0.32745	3.6976	3.29931	3.74647	2.20417	1.81355	1.68906	3.15633	2.91909
SLC25A20	2.12707	1.93956	2.08028	2.34846	3.82134	3.74448	3.71288	3.55955	3.37666	3.42098	3.72276	3.95126	3.09363	3.10913	2.41525	2.43607
ARIH2OS	2.4698	2.60797	2.28564	1.57613	3.21077	2.12837	3.43285	1.76246	2.96978	2.62958	2.89642	2.90936	3.18868	3.15931	3.26393	3.28818
ARIH2	4.07285	3.80374	3.62231	3.78276	4.0242	3.52886	4.17871	3.64134	3.97751	3.62401	3.73204	3.86249	4.21355	4.29928	3.94544	4.04609
P4HTM	0.42137	0.66182	0.91148	0.53244	1.02333	3.01245	0.36195	3.19359	1.38016	2.34639	1.78496	2.14831	-0.1717	0.73296	0.28124	0.64801
WDR6	5.13887	5.1006	5.13912	5.11066	5.17099	5.12993	4.64779	5.10815	5.15849	5.20939	5.35053	5.04517	4.99602	5.17138	5.15038	5.46302
DALRD3	4.8663	5.06738	5.09223	5.0613	5.16339	5.87654	4.40146	5.74826	5.44996	5.69459	5.68351	5.17107	4.51345	4.90127	4.67457	4.92109
NDUFAF3	5.53703	5.70526	5.5621	5.39108	6.04404	6.99521	5.31274	6.86507	6.02726	5.83171	5.90977	5.07527	4.97389	5.24169	4.56459	4.68247
IMPDH2	6.92664	6.45991	6.44748	5.96817	5.92049	5.42912	5.65512	5.54347	7.42465	7.15531	7.31409	7.03386	6.52143	6.38365	6.2175	5.81182
QRICH1	4.1131	3.90907	3.90995	4.00579	3.97241	3.72558	3.79635	3.55874	4.55624	4.18056	4.47294	4.26553	4.15401	3.99291	3.68946	3.74765
USP19	4.34572	4.23354	4.1639	4.30579	4.1578	3.63019	3.62488	3.67907	4.25429	4.34171	4.48403	4.38794	3.19878	3.42001	3.75022	4.0058
LAMB2	3.91886	4.32718	4.54048	4.65561	5.79603	5.88716	5.30704	5.72902	5.13964	5.71678	5.72829	6.12219	3.54877	4.02007	4.15047	4.53414
CCDC71	3.59502	3.83638	3.67812	3.62852	3.50261	3.96433	3.14496	3.72959	4.47149	4.64495	4.85758	4.67746	3.05793	3.07631	3.44858	3.01701
KLHDC8B	-0.8172	-0.20594	-0.20598	1.03723	3.47812	3.49823	3.29801	3.27531	3.29256	4.04777	3.56511	3.56835	3.13854	3.70207	4.07525	3.93847
CCDC36	-2.70113	-3.05747	-3.32193	-3.02764	-0.39159	-1.74009	-0.94819	-1.04767	-3.32193	-3.09192	-3.32193	-3.32193	-3.32193	-3.32193	-3.32193	-3.32193
C3orf62	1.50657	1.5477	1.62305	1.98092	0.18159	1.16081	0.06804	1.34246	0.95373	1.0956	1.36308	1.44856	0.82177	0.61829	0.17826	0.39143
MIR4271	2.55875	3.20183	3.05905	4.8014	2.4625	3.24332	2.14573	3.79473	2.04942	1.22284	3.44628	3.95178	2.21337	3.45304	-3.32193	2.5241
USP4	3.15565	3.0306	3.13283	3.41074	2.11512	1.9261	2.38522	1.90532	3.4918	3.3254	3.55773	3.79705	2.50565	2.57076	2.85014	2.71128
GPX1	8.41015	8.60845	8.62828	8.18321	6.91204	7.64263	6.26144	7.26697	8.28781	8.61241	8.763	7.98855	6.9573	7.36653	6.93428	7.25901
TCTA	3.58092	3.69059	3.71378	3.978	4.56332	4.08841	4.18522	4.02956	3.34796	3.71102	3.76788	3.49085	3.26239	2.92579	3.04377	2.95696
AMT	-0.52634	-0.73625	-0.70823	-1.18106	-1.06307	-0.32827	-2.06679	-0.52611	-1.44653	-1.18887	-0.76185	-0.97214	-0.74336	-1.84037	-1.53523	-1.56806
NICN1	2.4884	2.96403	2.65895	2.70544	2.16276	2.57707	1.67436	2.42312	2.96347	3.33861	3.12875	3.3714	2.2225	1.82962	2.37436	2.288
DAG1	4.84573	4.71618	4.93082	4.61548	5.96537	4.51907	5.79223	4.56853	5.31488	5.06768	5.22418	5.54149	5.79401	5.48467	6.3841	5.94166
BSN	-2.80539	-2.42737	-3.0954	-2.43798	-0.5118	-1.81713	-1.23123	-1.74114	-2.03172	-1.5767	-1.40545	-1.30819	-2.36819	-2.45679	-1.87014	-1.34342
APEH	4.60602	4.6248	4.48624	4.59511	5.65922	5.41009	5.28654	5.19603	5.76793	6.00597	6.05904	5.69497	4.93666	5.6854	5.52001	5.86711
MST1	-0.84172	-0.22782	-0.04674	-0.68888	2.67706	4.9186	1.73757	4.07703	-1.52109	-0.18408	-0.7606	-0.41265	-1.72111	-0.59923	-1.15263	-0.55427
RNF123	3.37017	3.36742	3.40657	3.25087	3.62402	3.6249	3.15551	3.55882	3.56182	3.45257	3.80361	3.41759	2.6968	2.79666	2.97995	3.13969
AMIGO3	2.17641	2.21263	2.22098	1.75978	2.98133	3.26927	2.34673	3.67901	1.88495	1.81789	2.05149	1.59965	1.32233	1.77551	1.65771	1.7689

GMPPB	3.9598	3.98646	3.87437	3.73758	4.26169	4.15407	3.74101	4.18486	3.62253	3.2262	3.49494	2.84669	3.11715	3.60192	3.32074	3.50543
IP6K1	3.80844	3.86367	3.74956	3.82526	3.78111	3.27988	3.7462	3.37308	3.90713	4.4998	4.51961	4.8548	3.51924	4.10371	4.42076	4.53788
INKA1	-1.07967	-0.22794	-0.02309	-0.80832	-1.49999	-1.3154	-1.4011	-0.87882	1.00601	0.97895	0.57682	0.12492	-2.65777	-2.20747	-2.04394	-1.65074
UBA7	2.38957	2.48069	2.74313	2.78912	-1.72229	-0.86538	-1.94676	-0.49548	1.51551	2.44233	2.72849	2.81987	-0.88503	-0.40891	-1.60713	-0.02444
MIR5193	4.87251	4.96664	5.21789	5.40419	-0.50469	1.57516	-3.32193	-0.07871	2.34081	3.63913	3.65988	4.48383	0.28174	1.99671	1.09724	1.52577
TRAIP	2.47168	1.49728	1.85695	1.09884	2.24697	1.82334	1.89491	2.14681	2.43517	1.31346	1.92449	1.10562	2.30594	2.68934	2.28361	2.43707
CAMKV	-3.32193	-3.32193	-3.01652	-3.32193	0.27166	-1.05201	0.48576	-0.86321	-3.02173	-3.32193	-3.32193	-2.51077	-3.1129	-2.37175	-3.32193	-3.06588
MST1R	-0.17916	0.6865	0.18007	0.12265	0.90294	-1.27311	1.08931	-1.1471	-3.09324	-2.43051	-2.38193	-2.55601	-3.32193	-2.37866	-3.13152	-2.21513
MON1A	2.35067	1.95138	1.59531	1.21832	2.2023	1.9346	1.57405	2.12338	2.55402	2.1025	2.31577	1.86361	1.88581	1.4151	1.65131	1.53984
RBM6	3.97299	3.69205	3.77208	3.77812	3.2518	3.62907	3.22447	3.7684	3.19665	2.89502	3.21972	2.90997	3.07216	3.13541	2.85074	3.0557
RBM5	2.72685	2.50989	2.92484	3.17174	2.23666	2.9073	2.28672	3.21821	2.22283	2.70266	2.81726	3.10102	2.57783	2.73604	2.39706	2.76216
RBM5-AS1	1.96636	1.83059	2.24607	2.2311	2.16983	0.84681	2.49586	1.06909	2.49379	2.80977	2.8199	2.92315	2.7957	2.66699	2.18188	2.62426
SEMA3F-AS1	3.07323	2.88103	3.39424	3.6353	2.46494	3.5255	2.23079	3.83279	2.22917	2.61228	3.00117	2.48256	1.93028	2.43311	1.99809	2.34553
SEMA3F	1.45284	2.32993	1.53643	1.98455	0.09745	0.71784	-0.51361	0.83453	-1.19603	-0.21778	-1.44788	-0.4638	-2.99771	-3.32193	-2.50004	-2.14847
SLC38A3	-2.48812	-1.8592	-2.0647	-2.16191	-2.73849	0.64471	-3.32193	0.60324	-1.82684	-2.675	-2.3125	-1.80883	-2.93626	-2.07449	-3.07492	-1.44527
SEMA3B	-1.1963	-0.03511	0.24367	-0.49104	1.14693	2.32125	0.4789	2.06939	-0.59281	0.7621	0.60577	0.51259	-0.27341	1.76479	1.8662	2.16111
LSMEM2	4.41598	3.5983	3.95865	3.48325	4.07432	3.74859	3.79406	3.63897	3.35677	2.2043	3.93321	1.6111	3.90255	2.81448	3.89052	2.22113
IFRD2	5.22661	4.40582	4.82973	3.9457	5.54951	4.53511	5.14592	4.44035	5.04467	4.0507	5.64335	3.3263	4.66346	3.70162	5.13479	3.75109
HYAL3	2.84258	3.24106	3.61342	2.94065	4.93135	4.86255	4.34842	4.3034	2.27949	2.88028	2.51705	2.421	2.24569	2.12695	2.31943	2.21779
NAA80	3.56141	3.77362	3.98723	3.24168	3.59235	3.86328	3.02608	3.54352	2.61346	2.95005	2.64077	1.88678	1.68366	1.91947	1.9483	2.00416
HYAL1	-0.63225	0.37405	-0.21206	-0.28718	4.24965	4.14269	3.62484	3.48314	-1.72917	-1.20244	-1.59493	-2.05319	-2.34323	-1.4312	-1.47293	-2.17846
HYAL2	3.65925	3.82069	3.78196	4.10752	4.58912	4.7013	4.20654	4.55447	3.46942	3.51879	3.95165	3.46721	2.78857	3.17068	3.53125	3.35734
TUSC2	5.48573	5.61194	5.59824	5.50149	5.80796	5.66444	5.52132	5.43636	5.20193	5.37619	5.33392	4.91604	5.07732	5.5488	5.09044	5.14519
RASSF1	3.10307	3.06667	3.35207	2.84858	3.83557	3.30757	3.57317	3.40285	2.63775	2.97126	2.34407	2.04802	2.96223	3.01408	2.22874	1.82978
RASSF1-AS1	0.35493	0.98154	1.13424	-0.7464	0.88941	1.75614	-0.11195	1.47901	-1.60653	0.69222	-0.97967	-0.09202	-0.20147	0.40825	0.17859	0.32246
ZMYND10	-2.20985	-1.98791	-0.96342	-1.17739	-1.67221	-0.81154	-2.14244	-1.3243	-0.45389	-0.03297	-0.08572	-0.74777	-0.77266	0.3027	0.29571	0.50838
NPRL2	3.26912	3.37929	3.05348	3.28873	3.21684	3.67785	3.11941	3.56367	3.08172	3.11586	3.13075	2.83257	2.36276	2.77383	2.45965	2.54257
CYB561D2	5.36763	5.29694	5.19367	5.34296	5.55505	5.70393	5.25807	5.67468	4.75486	4.91655	5.04172	4.26696	4.06806	4.3216	4.40602	4.53329
TMEM115	5.58186	5.54747	5.58684	5.46615	5.96748	5.86326	5.43291	5.90325	5.24949	5.40514	5.50247	4.97173	4.74006	4.75275	5.08445	5.32143
CACNA2D2	-2.86955	-2.56579	-2.79954	-1.58871	-1.62506	-0.54923	-1.32936	-0.78126	-2.13019	-1.91194	-2.30942	-2.28812	-2.03347	-2.42468	-2.05498	-1.96724
C3orf18	1.58079	2.33197	2.37731	2.26478	2.42958	2.44983	2.0172	2.22523	2.74042	3.79964	3.21855	2.57153	-0.76924	0.3507	0.2571	0.81198
HEMK1	0.82709	0.74722	0.76548	0.59191	0.60757	0.93646	-0.10787	0.55915	0.9929	1.58367	1.40129	0.89041	-0.57844	-0.21063	-0.64006	0.13508
CISH	-0.8882	-0.17809	-1.58282	-0.52668	-2.4436	-1.5328	-2.32769	-2.14301	-2.05183	-2.02091	-1.79218	-1.80034	-0.42995	1.30473	0.68483	1.1007
MAPKAPK3	3.95417	4.14352	3.87408	3.8118	4.33509	3.6517	4.15684	3.39315	4.41988	4.4702	4.56816	4.08326	3.73466	3.69037	4.17872	4.17042
DOCK3	-0.5271	-1.09496	-0.7271	-0.67581	0.46476	0.15423	0.04971	0.16706	-1.52575	-1.32484	-1.15531	-1.43861	-2.68446	-2.68629	-2.37257	-2.56314
MANF	6.27734	6.17128	6.11866	5.96315	5.30019	5.63159	5.03738	5.38604	6.16781	5.9698	6.43875	5.40878	5.20032	5.09213	4.79495	4.43953
RBM15B	4.49059	4.50969	4.60794	3.85792	4.63273	3.76742	4.09191	3.51986	5.46402	5.13121	5.6154	4.73461	4.1297	3.92242	5.01405	4.57076
DCAF1	3.07535	2.69992	2.85375	2.8642	3.43919	2.41266	3.57217	2.35822	3.89772	3.16734	3.61885	3.87555	3.35215	3.09559	3.58898	3.28762
RAD54L2	1.3192	1.85068	1.75586	1.60094	1.45706	1.59862	2.0024	1.89051	1.4303	1.71742	2.17952	2.61408	1.45223	2.12197	2.66854	2.57289
TEX264	3.51243	3.45598	3.63812	3.24869	4.12614	4.30042	3.38435	4.28496	3.42095	3.72446	3.70104	3.04426	2.91048	2.91969	3.20056	3.39407
RRP9	6.04561	5.71999	5.26458	5.01178	5.59552	5.74838	4.88403	5.59581	5.89062	5.64779	5.96025	4.35254	5.11051	4.91838	4.55368	4.53819
PARP3	2.57484	3.05509	3.11131	3.28845	1.53761	2.89074	1.15566	2.69344	3.61708	4.59104	4.46206	4.71472	1.84347	2.73988	2.3174	2.77092
GPR62	-0.83521	0.41417	-0.29791	-0.86991	-0.28256	0.74492	-1.2154	0.57568	0.54474	0.25468	0.54375	0.52931	0.27508	1.15656	-0.18793	0.35086
PCBP4	2.9717	3.7549	4.02554	3.80653	4.27031	4.43834	3.60348	4.09305	5.07238	5.21179	5.36772	4.20514	3.99194	4.2891	4.69765	4.6275
ABHD14B	5.33949	5.29905	5.41272	5.22689	3.8858	4.25107	3.33523	4.03267	5.15508	5.55395	5.45279	5.3459	3.85104	3.44507	3.78311	3.85323
ABHD14A	4.4422	4.53015	4.59167	4.26744	4.04421	4.68964	3.38656	4.61802	4.55501	4.81787	4.88338	4.16611	2.80811	2.87495	3.1416	3.1262
ABHD14A-ACY1	3.23393	3.25418	3.19499	3.4711	4.25585	5.18953	3.5403	5.07641	3.20681	3.76587	3.47167	3.4608	2.43977	2.8634	2.56203	2.97219
ACY1	2.75225	2.78576	2.65424	3.00168	3.89757	4.84525	3.16522	4.73105	2.54118	3.38332	2.9349	3.03624	2.22218	2.54851	2.32188	2.78191
DUSP7	2.84336	2.5867	2.50277	1.69593	1.95237	1.26685	1.22149	1.36064	3.68326	3.73456	3.78303	3.65004	3.74018	3.85436	3.72748	3.54009
POC1A	3.93199	3.38559	3.26821	2.92597	3.76198	2.8941	3.39423	2.92163	4.59627	3.56772	3.91917	2.99843	4.39906	4.43048	4.20956	3.90348

ALAS1	5.71829	5.72134	5.36004	5.7854	5.8389	5.25749	5.86733	5.08081	5.61822	5.11019	5.2866	5.54338	4.99514	4.94148	4.34274	4.57369
TLR9	-2.64237	-3.02903	-3.32193	-2.99634	-2.30609	-2.31198	-2.86313	-2.54321	-1.48835	-0.47626	-2.15423	-0.66125	-3.32193	-2.76644	-3.06918	-2.84538
TWF2	6.42714	6.76426	6.64986	6.1542	5.05889	5.77559	4.49447	5.36618	7.61658	7.65186	7.81063	7.22138	5.53302	5.69075	5.85066	6.18766
PPM1M	0.84419	1.30232	1.57718	1.85277	1.50194	1.89756	1.01956	1.98249	3.71263	4.30594	4.3685	4.20456	1.64653	1.7474	1.91565	2.53738
WDR82	4.41913	4.43578	4.26734	4.34398	4.50903	3.90261	4.7282	3.84475	5.56575	5.10091	5.55927	5.38619	5.15548	5.2383	5.44865	5.16775
GLYCTK	1.81802	1.95884	1.73762	1.10343	5.14403	5.50764	3.91684	5.40637	1.45145	2.26142	1.98888	1.50334	0.70815	1.42975	0.74329	1.51859
GLYCTK-AS1	1.58563	1.74697	1.62041	1.19127	4.50052	5.32949	3.08495	5.29236	0.98608	1.50792	1.46406	0.88402	0.20049	0.81159	-0.33547	0.88157
DNAH1	-1.70364	-1.48411	-1.12679	-1.09766	-0.39484	-0.2991	-1.33031	-0.2199	-1.96535	-1.13265	-1.17801	-0.59463	-0.71679	-2.28756	-1.67269	-0.97288
BAP1	4.54031	4.42069	4.29139	4.35686	4.86735	4.33317	4.50468	4.18275	5.3495	5.40646	5.47494	5.30486	4.71621	5.01316	5.3367	5.34497
PHF7	0.94187	1.10025	0.94837	0.71569	0.59429	0.51445	0.64444	0.58329	-0.37738	0.3029	-0.85281	-0.49034	-0.16072	0.67222	-0.83094	-0.32675
SEMA3G	-2.49458	-2.71641	-2.38272	-2.20214	-0.53112	-0.59128	-0.86727	-0.13516	-1.93221	-2.39888	-2.92041	-2.65534	-2.87406	-3.32193	-2.52336	-2.94088
TNNC1	-1.00378	-0.52148	-2.4842	-2.44313	0.45412	0.65815	0.77625	1.05372	0.14285	-0.70696	-0.78127	-1.79096	-1.96481	-0.02646	-2.13997	-2.60082
NISCH	3.02802	2.65315	2.94636	2.75995	2.95412	2.97307	2.2741	2.95011	3.4953	3.67017	3.7132	3.85401	2.83232	3.03358	2.90555	3.42274
STAB1	-0.1419	-0.14196	-0.53423	-0.63047	0.58884	1.45336	-0.12936	1.74879	1.01834	-0.37425	0.63262	-1.18278	1.34519	1.42877	0.59808	0.14875
NT5DC2	3.9227	3.93997	3.92607	3.45127	5.4622	5.30355	4.59984	5.31508	5.79394	4.92334	5.57962	4.28509	5.26183	5.2362	5.60117	5.35737
SMIM4	3.39409	3.3811	2.82063	2.9034	3.17172	3.59862	2.79289	3.56264	2.9977	2.79594	2.81399	2.29346	3.68794	3.71537	3.3702	3.39728
PBRM1	2.36647	2.32445	2.13603	2.93369	2.85723	1.73068	2.97305	1.84405	3.80689	3.17484	3.45747	4.11026	3.795	3.61175	3.91262	3.89992
GNL3	5.66578	5.18626	4.92022	5.08618	5.37042	5.42393	5.57192	5.39421	5.34864	5.37157	5.46287	5.06779	5.58173	4.96839	4.59809	3.87639
GLT8D1	3.54233	3.47041	3.95737	3.91855	3.84522	4.10021	4.02012	3.74143	3.50838	3.72427	4.10864	4.01132	3.50882	3.56357	3.10122	3.11585
SPCS1	4.80219	4.79197	4.8167	5.01041	4.64035	5.26053	4.70318	5.18892	4.51881	4.3524	4.29361	3.65455	4.19052	4.14699	3.35507	3.40969
NEK4	2.62857	2.4226	2.27304	2.43413	2.44993	1.94295	2.54494	2.04106	2.99576	2.64338	2.60304	3.26443	3.20537	3.24135	2.96324	2.8572
ITIH1	-3.32193	-3.32193	-3.32193	-3.32193	0.5568	1.17546	-0.50258	1.29665	-3.32193	-2.91115	-3.32193	-3.32193	-3.32193	-3.32193	-3.32193	-3.32193
MUSTN1	1.82448	1.437	1.51215	1.18425	0.46364	2.06656	0.38379	2.41128	-1.73683	0.6722	0.43073	-0.41013	0.72283	1.212	0.00527	0.76161
STIMATE	2.81455	2.498	2.52721	2.39144	2.99657	2.45832	3.00265	2.22508	3.00346	1.96378	2.82533	2.27827	2.94444	2.28354	3.3265	2.76305
SFMBT1	1.6982	1.08805	0.74102	1.27087	2.42417	1.0254	2.7792	1.11469	3.01695	1.98672	3.02103	2.60614	2.28621	2.18623	2.98311	2.27412
RFT1	3.10187	2.69975	2.67422	2.36525	3.29721	2.36786	3.18425	2.07762	3.29015	2.25215	3.37229	2.52547	2.60475	1.58664	2.88317	1.87208
PRKCD	4.75118	4.84948	4.87488	4.99128	3.17002	3.13557	2.90326	3.05471	4.90873	4.76121	4.61942	4.33977	3.83957	3.86304	3.84938	3.92588
DCP1A	3.02119	2.64203	2.92952	3.10556	2.08307	2.30113	2.60478	2.40953	2.88237	2.06343	3.01876	2.84016	2.90177	1.73277	2.76299	2.12997
CACNA1D	-3.32193	-3.32193	-3.32193	-3.19076	-2.39853	-2.19656	-1.93309	-1.62509	-3.32193	-3.32193	-3.32193	-3.32193	-2.79286	-3.01692	-2.9171	-3.07833
CHDH	-2.64363	-2.09084	-2.65054	-2.99702	2.67733	1.62002	2.19315	2.2084	-3.16322	-2.85113	-3.32193	-3.19121	0.78439	0.56022	0.78555	0.89319
IL17RB	-0.25005	0.2505	0.32206	0.60315	2.72228	1.93339	2.54771	2.08151	-0.14918	-0.63942	-0.00027	0.65748	2.05064	1.98875	1.83331	1.81372
ACTR8	2.97265	3.19575	2.91024	3.44245	2.95773	2.56765	3.5895	2.68068	3.20807	3.27098	3.33709	3.66612	2.86057	3.13785	3.33962	3.33403
SELENOK	5.1779	5.24038	5.13671	5.48436	4.24865	4.85917	4.04994	4.70886	4.38493	4.29254	4.23396	3.76826	4.58497	4.85001	3.61072	3.83119
CACNA2D3	-0.21008	-0.56009	0.21812	-0.09048	-3.32193	-3.32193	-3.32193	-3.0617	-1.97403	-2.13158	-2.18105	-2.24958	2.27091	2.74772	2.14788	1.76226
WNT5A	3.16953	3.59979	4.03093	4.70164	-2.98009	-3.04433	-3.32193	-3.00047	2.15112	2.0344	2.23864	3.14942	1.51699	2.77074	2.60894	2.79062
WNT5A-AS1	3.68127	3.82906	4.62891	4.0834	-2.10874	-3.32193	-3.32193	-3.32193	3.2175	3.34038	3.66022	2.71692	1.19467	2.75799	3.14467	3.47999
ERC2	-2.4108	-1.64557	-0.98372	-0.87667	-3.20676	-3.18584	-3.32193	-3.16312	-2.67246	-1.84038	-2.73049	-2.77228	-3.32193	-3.21575	-3.32193	-3.06982
CCDC66	1.48377	1.29282	1.27015	1.39915	0.61678	0.82225	0.80051	0.89003	0.47999	0.45201	0.47792	1.11917	0.88386	1.03443	0.53739	0.55881
ARHGEF3	1.69716	2.10911	1.83114	2.57393	2.2642	2.21967	2.80728	2.15958	1.39561	1.99748	1.76737	3.09347	1.48158	1.4331	2.07097	2.07056
SPATA12	0.00086	0.05468	-1.30456	-0.58109	-2.35386	-1.60981	-1.32109	-1.77951	-2.23678	-1.39919	-2.32222	-1.30431	-1.065	-0.6162	-1.53253	-0.28293
IL17RD	-1.00647	-0.27074	-0.10925	-1.03089	0.50632	0.22277	0.60117	-0.03119	-0.13376	0.85316	0.20309	1.20701	2.12251	2.48553	3.05845	3.27105
HESX1	-2.15599	-2.06128	-0.88347	-1.57552	-0.55414	0.68622	-0.43723	0.59015	-2.03167	-0.51522	-2.12754	-1.3409	-1.02984	-0.35908	-1.84619	-1.30957
APPL1	3.24154	3.23677	3.13225	3.5072	3.62385	2.37824	4.14854	2.44765	4.0945	3.69305	3.44668	4.41315	4.08909	3.81838	4.12365	3.88645
ASB14	1.62185	1.74903	1.4721	1.76737	2.00953	1.48286	2.4833	1.67116	2.03262	2.00483	1.70457	2.21107	2.38845	2.06578	2.51529	2.26611
DNAH12	-2.95196	-3.14402	-2.9691	-3.21919	1.22785	-0.49644	1.83407	-0.24458	-2.69558	-1.92415	-2.13804	-1.4943	-2.97164	-3.08002	-2.9668	-2.8972
PDE12	3.09431	3.13503	2.95921	2.75429	3.03264	1.9463	3.1316	2.05121	3.33662	2.99029	2.9917	3.2677	3.20267	3.09726	2.64851	2.97035
ARF4	6.9347	7.14048	7.03058	7.44955	6.50976	6.81843	7.0869	6.54328	6.62739	6.65344	6.59875	6.85771	6.37094	6.2843	5.82808	5.75975
DENND6A	2.41296	1.9665	2.27958	2.18753	2.60316	1.35931	2.91668	1.13082	2.85759	2.20121	2.92308	2.58189	3.00908	2.09955	3.68263	2.47743
DENND6A-AS1	1.89714	1.78679	1.64675	1.73553	2.82228	0.68622	3.35762	0.69745	2.59648	1.76117	2.8854	2.80167	3.3395	2.75657	3.90311	2.61686
SLMAP	2.8223	2.75757	2.57279	2.77664	2.61424	0.92386	2.68241	1.00387	3.18348	2.37699	2.7091	2.71927	2.91288	1.71458	2.46224	1.94195

FLNB-AS1	4.25394	4.45875	4.74414	4.12996	1.75674	0.75427	1.95952	1.02951	6.22346	5.5905	5.80527	5.76906	5.43455	4.61113	5.43741	5.01905
ABHD6	1.45326	1.54377	1.32668	2.1596	2.9594	2.59625	2.76588	2.54493	1.84555	1.89047	1.65383	2.06336	1.55053	1.66557	1.25807	1.77112
RPP14	1.74552	1.76895	1.6715	1.67304	2.55253	2.3276	2.7028	2.49696	2.19244	2.26066	2.14423	2.29457	2.33892	2.66866	2.27853	2.40616
HTD2	3.00863	2.91474	2.88556	2.77625	3.85687	3.22049	4.01514	3.32035	3.31244	3.32039	3.27792	3.30113	3.50439	3.9038	3.54965	3.59807
PXK	2.37924	2.01925	1.96178	2.59196	1.39187	1.08584	1.45344	0.75381	3.24055	3.00716	2.84861	3.99501	1.37134	1.05681	1.34248	0.79988
PDHB	3.30836	3.19655	3.06438	3.41863	4.36182	4.59924	4.37234	4.34315	4.94287	4.76383	4.73362	4.51853	4.10479	4.03784	3.74477	3.8194
KCTD6	-0.17753	-0.85126	-0.86367	0.16598	2.406	3.26909	2.70028	3.28221	1.22326	0.64491	0.89206	1.88645	0.77003	0.74832	0.78092	0.21602
ACOX2	-1.73159	-2.57586	-2.75971	-2.7295	2.32523	3.57217	1.44799	3.30158	-0.49857	0.0708	-0.89559	0.1008	-2.24952	-2.486	-2.351	-1.41592
C3orf67	-0.34095	0.28074	0.202	0.27356	-1.98055	-1.74088	-1.77816	-2.41219	-1.02564	-1.18322	-1.60123	-1.28185	0.03708	0.12859	-0.53997	-0.22941
FHIT	-1.13056	-0.95338	-1.08339	-0.72645	-1.01764	0.51332	-1.61513	-0.15569	-2.32541	-1.84228	-2.40588	-1.3306	-1.05862	0.26639	-0.19007	-0.80225
PTPRG	1.20604	1.19245	1.43411	1.5524	2.39564	0.47794	2.63944	0.88592	3.60651	3.55698	3.49415	4.12071	2.64322	2.80647	2.95488	2.91155
RPL10AP6	3.41877	2.89694	3.47249	2.29535	2.01554	2.39924	1.95527	2.29685	3.33087	4.46102	3.51664	3.37421	1.22538	1.22045	0.54973	0.68718
PTPRG-AS1	0.22685	-0.1301	-0.12336	-0.14192	1.27306	-0.76439	1.97637	0.19804	2.4106	1.92427	2.19805	3.13312	1.52226	1.73149	1.79854	1.52585
C3orf14	-3.32193	-3.06294	-3.32193	-3.32193	1.98341	1.32714	2.24125	2.09343	2.18895	1.92593	1.59552	1.39204	-3.32193	-3.32193	-3.32193	-3.32193
THOC7	4.93531	5.04275	4.85613	5.06335	4.29904	4.64871	4.91412	4.75804	5.54295	5.35024	4.96333	5.83845	5.14607	5.02534	4.3454	4.01327
ATXN7	1.18568	1.16766	1.19247	1.39012	1.45682	0.67556	1.80261	0.88063	2.26937	1.68206	2.10983	2.45908	1.6306	1.00175	1.94435	1.50247
PSMD6-AS2	-0.72352	-0.74524	-1.32371	-0.09272	-1.44057	0.03784	-0.36133	0.38158	-0.88689	-1.67434	-1.02081	-0.18623	-1.01126	-0.16909	-1.42841	-0.48043
PSMD6	3.90899	3.95082	3.65855	4.0509	3.24317	3.51017	3.43569	3.50487	3.96578	3.73419	3.95468	3.94779	3.58377	3.58267	3.0424	3.0361
PRICKLE2	-2.01531	-2.04829	-2.32365	-1.61753	-3.08459	-3.04353	-3.12359	-2.79768	-3.07884	-2.17267	-2.45973	-1.45629	1.09824	1.20296	2.15915	1.55589
PRICKLE2-AS1	-2.2552	-1.53227	-1.47643	-0.61536	-3.32193	-3.32193	-3.32193	-3.32193	-3.32193	-3.32193	-1.61351	-0.46864	2.16148	2.18384	3.58671	2.85943
PRICKLE2-AS3	-2.37121	-2.55808	-2.86945	-2.19918	-3.14298	-3.32193	-3.32193	-3.32193	-3.32193	-2.94359	-2.60451	-2.1982	-0.40025	-0.50055	1.19968	0.58094
ADAMTS9	2.95731	3.41451	3.30581	3.33622	-2.10338	-1.64194	-1.56739	-2.23538	-3.32193	-3.32193	-2.94217	-3.22581	-0.77615	0.13545	-0.12232	-0.12731
ADAMTS9-AS1	0.05406	0.32574	0.6001	0.39238	-3.01488	-3.16757	-3.17886	-3.32193	-3.32193	-3.32193	-3.32193	-3.32193	-2.16813	-2.48625	-2.20225	-1.75542
MAGI1	0.60956	0.62599	0.387	0.46304	2.48271	2.17386	2.49919	2.45437	1.79764	1.77194	1.81696	2.02154	1.06978	1.18855	1.67135	2.07574
SLC25A26	3.37416	3.24735	3.25969	2.66229	2.41383	2.03469	2.52594	2.16569	3.38296	3.69284	3.77097	3.60871	3.97946	3.97434	3.88564	4.34135
LRIG1	4.05984	3.97123	4.21131	3.09011	1.87282	1.14345	1.58254	1.67128	4.43047	4.70189	4.76055	4.37895	5.15429	5.2102	5.29993	5.77762
KBTBD8	0.35605	0.94183	-0.19002	1.00236	-0.12924	-1.59088	-0.06397	-0.91916	-0.03472	-0.44988	-0.9787	-0.47109	-0.93326	-1.84115	-1.27605	-1.09598
SUCLG2	3.50813	3.63129	3.7507	3.76297	4.56529	3.64597	4.88382	3.5138	5.35969	5.39817	5.24042	5.34603	4.27386	3.38851	4.11021	3.29371
SUCLG2-AS1	0.13529	-0.34091	0.01405	0.05258	-1.09041	-2.12403	-0.20989	-1.01217	-0.52476	-0.63701	-0.86041	0.16246	1.52982	0.69481	0.96893	0.80626
PSMC1P1	3.74289	3.60608	3.61943	3.69388	3.35239	3.17268	4.03083	2.85464	3.90678	3.39676	3.66077	2.99263	3.40632	2.65452	3.37476	2.33185
EOGT	1.40247	1.39551	1.58465	2.34027	2.80414	2.18974	3.36137	2.39264	2.79823	2.92172	3.11673	3.67897	2.83463	3.19003	2.99329	2.90808
TMF1	2.536	2.71362	2.57196	3.30117	3.76328	2.69838	4.286	2.78779	3.64827	3.99233	3.52157	4.56555	3.32246	3.59255	2.94517	3.32215
UBA3	3.81442	3.85853	3.95275	4.27654	3.5827	3.29405	3.88383	3.28132	4.05313	4.11324	3.86305	4.07198	4.63143	4.54748	3.88304	3.54874
ARL6IP5	4.93532	5.34031	5.30155	5.94391	4.9704	4.39294	5.70065	4.3567	5.55004	6.12162	5.64271	6.62337	6.27774	6.66469	6.04445	6.24601
FRMD4B	-0.78207	-1.33501	-1.24453	-1.08138	-0.51389	-0.8688	-0.29032	-0.83165	0.87445	0.66256	0.73194	1.28332	2.07283	2.1936	2.51245	2.83154
MITF	0.80807	0.94764	0.51545	0.2506	-0.54253	-1.46663	-0.11109	-1.65212	0.14188	-0.68911	-0.19882	-0.36754	2.61918	2.25543	2.95689	2.29321
FOXP1	2.365	2.3465	2.42477	2.47798	1.45483	1.77259	2.18402	2.05571	1.7038	0.8367	1.41488	1.70414	1.68648	0.88185	2.26566	1.8728
EIF4E3	-2.36882	-2.4674	-2.50003	-2.02659	-3.15544	-3.32193	-3.32193	-3.32193	-1.77352	-1.20664	-1.88067	-1.32071	-2.35184	-1.92684	-2.1601	-1.50245
RYBP	2.30157	2.06799	2.42997	2.56036	1.66244	0.95462	2.42534	0.77578	3.4098	2.84494	3.43416	3.73621	2.69963	1.57951	2.51288	2.03545
SHQ1	2.38286	1.78271	2.01836	1.94763	1.83388	0.51604	2.21783	-0.02175	2.5165	0.77774	2.26945	1.37523	2.11662	0.55503	1.96564	0.5602
GXYLT2	3.09148	2.90459	3.11695	2.9071	1.63751	-1.04338	2.15263	-0.26874	3.14699	2.96242	2.59499	2.70055	1.4347	1.30987	1.86246	1.8021
FTH1P23	3.81457	3.95079	4.25991	2.62218	-0.51599	1.16358	-1.46021	0.5288	0.92923	2.24078	0.77233	2.17458	-0.19452	-1.216	0.39237	0.26502
PPP4R2	3.14699	3.34536	3.14933	3.71633	3.23969	2.50224	3.75472	2.72604	4.07552	4.12868	3.69309	4.55324	3.98282	3.92561	3.50231	3.41836
EBLN2	-0.71672	0.37108	-0.11801	0.40648	-0.71209	0.4943	-0.42038	0.26257	-0.2307	0.11189	-0.46889	0.35972	-1.50022	-0.48712	-0.3109	-0.85963
HNRNPA3P6	4.25972	4.03381	3.75871	3.64027	2.15634	2.28042	2.5688	2.2844	3.94808	3.23256	3.57633	2.62536	3.04606	2.86133	2.33202	2.82816
ALG1L6P	2.51891	1.96069	2.2356	1.79107	2.76486	3.06729	1.93638	3.00288	3.21165	2.96372	3.86052	3.41686	1.41516	1.61231	2.00371	1.67694
FAM86DP	1.70834	1.51743	1.75327	1.72922	1.79601	2.16077	1.33202	1.97975	2.14699	2.50931	2.44462	2.84838	1.56105	1.41856	1.23139	1.43881
LINC00960	-3.00696	-3.32193	-2.42108	-1.97707	-3.05021	-2.33152	-3.32193	-3.32193	1.8203	2.18482	2.03334	2.19031	2.65378	2.35847	2.78214	2.41634
ZNF717	0.0214	0.47838	0.1423	0.36013	-1.77649	-0.57554	-1.50205	-0.64972	-0.64767	0.08205	-0.17788	0.35377	0.64983	0.56127	0.42247	0.46327
ROBO2	-3.32193	-3.32193	-3.24444	-3.2395	-1.00691	-0.72762	-0.34567	0.19793	-3.1048	-3.25854	-3.12689	-2.7943	-3.32193	-3.32193	-3.32193	-3.32193

ROBO1	1.72439	1.80586	1.92405	1.76827	5.97741	4.15383	6.19284	3.78842	2.20821	2.76179	3.19482	3.17266	0.03215	0.92138	3.56146	3.35324
LINC02027	-3.32193	-3.32193	-3.32193	-3.32193	1.24339	2.68254	0.39566	2.14953	-3.32193	-3.32193	-3.32193	-3.32193	-3.32193	-3.32193	-3.32193	-3.32193
GBE1	3.30757	3.55139	3.8279	3.705	3.25311	3.79957	3.8624	3.6712	5.22831	5.36471	4.72205	5.59626	4.11489	4.26905	4.01981	3.99662
VGLL3	0.17063	0.54941	0.12538	0.83808	-3.285	-3.32193	-3.16623	-3.32193	3.22646	3.06126	3.09569	3.63667	-2.6676	-2.19011	-1.74584	-2.9458
CHMP2B	3.47504	3.83425	3.50454	4.13551	4.01784	3.91957	4.79509	3.63968	4.40705	4.49859	4.13952	5.24257	3.5079	3.81819	2.8594	3.24612
CGGBP1	2.57236	2.81546	2.99076	3.28716	2.90884	3.06529	3.41413	2.98088	4.43228	4.45329	4.27531	5.11875	3.6859	3.93263	3.76507	3.7633
ZNF654	0.77622	1.15766	1.30522	1.69291	0.65686	-0.21222	1.31695	-0.06339	2.42342	2.44012	2.58219	3.55925	1.64548	1.18241	1.70782	1.20083
C3orf38	1.80402	2.1885	2.09663	2.75452	2.34088	1.66378	3.07943	1.75046	2.73041	3.17131	2.99692	3.64102	2.37985	2.85296	2.64061	2.78084
PROS1	1.19564	1.36639	1.30259	1.93224	4.80973	4.55054	5.30813	4.32678	2.38199	3.16805	2.81074	3.53138	4.46731	4.63099	4.51963	4.66143
ARL13B	1.3385	0.92864	1.56336	1.70512	2.3988	0.86318	2.6169	0.97577	2.25374	1.39085	1.51993	1.38687	2.55983	1.99792	2.27649	1.61915
DHFR2	0.20099	0.62267	0.03925	0.70944	0.47263	0.35503	0.69944	0.4729	0.80395	0.77094	0.67931	1.05243	0.79189	1.34755	0.6736	1.0812
NSUN3	0.08967	0.16389	-0.20603	0.57627	0.33807	0.10407	0.52314	-0.31397	0.54676	0.91052	0.48378	1.16436	1.05285	1.34521	0.59173	1.3785
ARMC10P1	1.71242	1.09607	1.2378	1.72623	0.81373	0.88269	0.04552	0.68845	0.33415	1.1943	0.29682	1.5557	1.2909	0.60145	0.48543	0.51476
HNRNPKP4	1.00282	1.48677	1.19488	0.66404	0.39275	0.75299	0.32186	0.22114	1.5675	1.45728	1.16758	0.5658	0.09103	-0.24618	0.29011	0.49722
MTRNR2L12	5.06471	5.0328	5.35567	4.05589	3.68806	6.91781	4.18542	6.8381	4.11984	5.57121	3.58256	6.57302	3.29834	3.90701	3.77961	5.12519
ARL6	0.57666	0.90396	0.68499	0.98914	0.16776	0.47165	0.50041	0.01177	0.98089	1.25745	0.65676	1.67286	1.02915	1.05217	0.07836	0.81217
CRYBG3	1.44008	1.48239	1.47271	1.78029	1.46	0.67865	2.18521	0.88631	2.81936	2.4016	2.38208	3.48039	2.27005	1.50517	2.38002	1.91569
RIOX2	2.89167	2.49069	2.50413	2.73353	2.88314	2.22457	2.91748	2.26187	3.62455	3.26415	3.33343	3.65797	3.14988	2.58712	2.72127	2.49782
CLDND1	3.69528	3.8755	3.86318	4.39508	3.45846	3.46404	3.75049	3.33844	3.7119	3.72063	3.11582	4.27642	3.57084	3.2595	2.1935	2.37328
CPOX	4.22077	4.33139	4.27707	4.18535	4.46902	3.64312	4.55081	3.54636	4.59788	4.55807	4.04481	4.12933	4.24221	4.26712	3.49022	3.88041
ST3GAL6-AS1	2.76996	3.20062	2.70826	2.17522	-3.32193	-3.32193	-3.32193	-3.32193	0.22668	-0.15358	-0.21257	-1.46659	-3.32193	-3.32193	-3.32193	-3.32193
ST3GAL6	2.44152	2.56862	2.30883	2.7355	-1.34536	-2.41614	-1.24264	-2.79544	2.35654	2.74283	2.44824	3.81225	3.80689	3.34336	3.99868	3.83318
LINC00973	5.01664	5.2868	5.24966	5.54678	-3.32193	-3.32193	-3.32193	-3.32193	1.58836	2.94938	0.75528	2.86579	3.19193	2.91297	0.71418	0.99243
COL8A1	2.75647	2.59784	2.80938	3.2674	-3.27225	-2.90656	-3.32193	-3.18689	-1.51741	-1.23054	-1.67277	-1.51351	2.46023	0.02073	2.08238	0.14575
CMSS1	3.105	2.49715	2.48781	2.28788	2.3036	1.2933	2.25166	1.16232	3.87053	2.33073	3.28974	1.56926	2.34118	-0.42073	1.16863	-0.97233
FILIP1L	-2.28501	-2.10274	-2.62321	-1.45179	-1.3164	-0.51827	-0.78926	-0.08243	1.60134	3.04701	2.507	3.66265	3.22969	3.46482	4.18099	3.59193
TBC1D23	2.04956	2.38056	2.43349	2.68104	2.455	1.6493	3.29494	1.76746	3.1953	3.77744	3.36755	4.1054	2.63645	2.64226	2.50528	2.44992
NIT2	2.36039	2.47351	2.37345	2.55449	2.17719	2.75295	2.07767	2.69097	2.67599	2.89599	2.68607	2.60605	2.46845	2.44619	1.67605	2.04348
TOMM70	4.22718	4.22219	4.04017	4.40337	4.23916	3.4589	4.86601	3.48818	4.87311	4.81648	4.83225	5.46737	4.6542	4.64972	4.56707	4.21742
LNP1	0.6635	0.29075	0.57538	0.53656	1.47618	1.6647	1.5373	1.04978	0.9599	1.045	0.88888	0.90042	0.80668	0.9092	0.98407	1.01405
TMEM45A	1.67893	2.13552	2.56151	1.61032	2.25104	2.27217	2.47015	2.01271	2.68025	3.00478	1.75143	2.70222	0.23057	-0.31069	-1.08868	-0.81943
TFG	4.58042	4.22993	4.72103	4.82635	4.46073	4.08141	4.96224	3.83383	5.31946	4.31555	5.18923	5.07492	5.41003	4.0286	4.65142	3.56541
ABI3BP	-2.13761	-2.29933	-2.44413	-1.93982	-3.27925	-3.12915	-2.77404	-2.90362	-1.24987	-0.66256	-0.99191	0.11798	1.5646	1.25055	1.14582	0.70018
SENP7	-0.63028	0.34631	-0.01259	0.81098	0.73698	0.44752	1.12706	0.70477	1.03311	1.77532	0.80466	2.34184	1.37862	2.11263	1.30066	1.64683
TRMT10C	4.23299	4.47555	4.09977	4.84789	4.09043	3.79259	4.70687	3.99916	4.36782	4.22736	4.07348	4.36991	4.01815	4.29831	3.60278	3.86213
PCNP	5.14683	5.3007	5.15665	5.33073	4.7777	4.45492	5.27359	4.18771	5.72477	5.47708	5.6127	5.73546	5.59453	5.12214	5.11445	4.66787
ZBTB11	2.20824	2.18648	2.225	2.69332	2.45953	1.51689	2.79254	1.56098	2.62246	2.6978	2.69726	3.22137	2.69572	2.26722	2.56445	2.4111
ZBTB11-AS1	2.08101	1.88309	1.85588	1.74435	2.73057	1.5936	2.64474	1.59831	2.58974	2.67868	2.87576	2.24501	1.90124	1.67459	2.06321	1.79436
CEP97	0.5024	0.29994	0.60878	0.76593	0.57625	-0.23831	1.32467	-0.044	1.12314	1.00875	1.36938	1.48759	1.56497	1.84115	1.73458	1.75845
NXPE3	-3.08343	-3.18327	-3.32193	-2.89927	1.60555	1.67756	2.1443	1.74413	3.08208	3.02405	3.13846	3.71833	1.96176	2.05014	1.97354	2.2152
NFKBIZ	3.02875	3.4429	2.98848	3.71314	3.10999	2.05492	3.07212	2.09102	4.30315	4.45607	4.28531	4.58633	2.49635	2.89527	1.78226	2.93464
LINC02085	-0.58291	-0.93035	-0.98999	-2.35446	-2.12929	-2.85454	-2.55093	-3.32193	1.08631	1.34757	0.51887	0.5133	-2.94735	-3.32193	-3.32193	-3.32193
ALCAM	4.066	3.69626	4.12662	3.7522	4.64171	3.70509	5.18021	3.47841	6.68956	5.69684	6.64255	5.78438	6.08208	4.59963	6.12095	4.41815
CBLB	-0.60563	0.02812	-0.25538	0.27366	-0.58826	-0.18105	-0.2509	-0.17224	1.3438	1.87135	1.82214	3.14053	-0.59998	-0.23939	-0.02555	0.62227
DUBR	-0.57339	0.33324	-0.10356	0.41643	-1.14349	-1.80304	-0.54449	-2.4128	0.97529	1.57658	1.45311	2.10964	0.27875	0.65012	0.43388	0.21715
BBX	2.83019	3.07085	3.20109	3.97492	1.95707	1.73302	2.71227	1.82187	3.39418	3.39367	3.76392	4.67411	2.79165	2.70878	3.51947	3.41675
CD47	3.79315	4.06989	4.13502	4.15555	2.50323	1.54243	2.67899	1.47989	3.72565	3.72772	3.55212	4.03967	3.97299	3.85075	3.94033	3.62202
IFT57	3.20305	2.89387	2.84898	3.12721	2.04481	2.44207	2.12147	2.19715	3.54178	3.73071	3.57879	3.93727	4.06434	4.17553	3.59017	3.67696
MYH15	-2.05156	-1.26182	-1.20217	-0.29846	-3.26141	-3.32193	-2.29636	-3.32193	-0.84353	-0.21974	-0.89193	0.13031	2.55699	2.65833	2.19483	3.81035
CIP2A	2.47451	2.40943	2.14971	2.38828	3.44502	1.99024	3.75497	2.63892	3.81236	2.68743	2.75214	2.66687	4.19209	3.99799	3.62458	3.49798

DZIP3	0.40547	0.15324	0.42191	0.98776	0.66405	0.37078	1.15715	1.04236	1.3698	1.75447	1.18143	2.40671	2.061	2.23175	1.96136	2.07628
LINC00488	2.45674	2.41138	2.37066	2.9119	-3.32193	-3.32193	-3.32193	-2.87163	-3.32193	-3.32193	-3.32193	-2.41434	-2.53629	-3.32193	-3.32193	-1.8348
NECTIN3	1.45186	1.43618	1.36077	1.89848	0.94731	0.3419	1.66419	0.15715	3.52456	4.10743	3.62157	4.78201	4.15207	4.34843	4.15561	3.9074
CD96	-2.57907	-3.11881	-3.32193	-3.09537	-3.32193	-3.32193	-3.32193	-3.32193	-3.32193	-3.32193	-3.13348	-3.14856	-0.0668	-0.49439	-0.86222	-0.04761
ZBED2	-2.31652	-2.23051	-3.32193	-2.13804	-3.32193	-3.32193	-3.32193	-3.32193	-2.20351	-3.32193	-3.32193	-3.32193	4.32694	4.00829	2.9507	2.20874
PLCXD2	-1.03155	-0.84623	-0.80762	-0.55926	-0.01078	-0.58126	0.35462	-0.41877	1.64426	1.36097	1.69411	1.60955	3.07271	2.92101	2.43767	2.48178
PHLDB2	1.33774	1.32143	1.28135	1.35591	-1.12394	-1.46841	-0.62735	-1.37713	4.66966	4.87956	4.70183	5.0861	3.112	2.58891	2.62765	2.66011
ABHD10	2.86151	2.68792	2.38221	2.77524	3.00973	2.73244	3.05753	2.8211	3.93544	3.59283	3.26721	3.39225	3.88091	3.93185	3.15473	3.37211
C3orf52	-2.99102	-3.12698	-3.32193	-2.46305	1.71401	1.02992	2.3211	0.84946	1.33757	1.0191	1.08159	0.7868	2.23727	2.68468	1.80092	1.90125
GCSAM	-3.32193	-3.32193	-3.32193	-3.32193	-0.9006	-1.51904	-0.45015	-1.2611	-2.18945	-1.95306	-1.67792	-2.20627	-0.8624	-0.25719	-0.54029	-0.83527
CD200	-3.32193	-3.32193	-2.93521	-3.32193	-3.32193	-3.32193	-3.00901	-3.32193	-3.32193	-3.32193	-3.32193	-3.32193	-0.81715	0.15422	0.85554	-0.49872
ATG3	3.45068	3.4356	3.13945	3.48682	3.17423	2.79582	3.48801	2.82354	4.27647	3.65021	3.96942	3.5268	3.87544	3.39778	3.83296	3.02567
SLC35A5	2.40098	2.46492	2.15018	2.58381	2.39748	1.45189	2.56608	1.76161	3.3042	3.26531	3.31098	3.52749	3.4059	3.60554	3.5898	3.54791
CCDC80	1.51355	1.61623	1.65673	1.90129	-2.7088	-2.10733	-2.32362	-2.55111	5.48796	5.25827	5.98237	4.75432	1.77654	1.41821	2.38851	1.73945
CD200R1	-1.74298	-2.09636	-1.14179	-0.18804	-2.81395	-2.0549	-3.32193	-2.36173	-2.5623	-2.09444	-2.03431	-2.10849	-3.32193	-3.32193	-3.32193	-3.32193
GTPBP8	1.70688	1.31655	1.37817	1.51889	1.70948	1.21107	1.86429	1.00439	2.99981	2.93558	2.84013	2.75569	2.34771	2.05996	1.66877	1.5513
NEPRO	2.53922	2.4218	2.48745	2.84923	2.30518	2.14992	2.73786	2.20357	3.23381	3.23272	3.19188	4.06748	2.88814	2.54541	2.55148	2.34484
BOC	0.27735	-0.02113	0.22421	-0.05422	-3.14863	-3.2163	-3.22414	-3.19844	-0.99504	-1.13604	-1.10218	-1.45984	-2.88559	-3.0881	-2.80551	-3.0351
CFAP44	-1.18853	-0.68109	-0.73754	-0.36259	-1.45	-0.85632	-1.3066	-0.92764	-1.88432	-1.27115	-1.7119	-0.67313	-1.94212	-1.10709	-2.09388	-0.96675
SPICE1	0.76797	0.64062	0.74394	0.87946	0.44998	0.02717	0.65804	-0.2055	0.38317	-0.09112	0.03049	0.4239	0.87221	0.6248	0.02073	0.49228
SIDT1	-2.57858	-2.91676	-2.67079	-2.75026	-0.91755	-2.41409	-0.08175	-2.77781	-3.32193	-3.19383	-3.18475	-3.32193	-3.32193	-3.32193	-3.19504	-3.32193
USF3	-0.52867	-0.67507	-0.79785	-0.38975	0.90359	-0.22772	1.21091	-0.12064	0.60255	0.16186	0.53213	1.02008	0.74851	-0.07224	0.9107	0.28588
NAA50	4.10836	4.22267	3.83659	4.14305	4.76842	3.80479	5.50592	3.7046	5.55891	5.20116	5.30291	4.97534	5.56115	5.74209	5.48378	5.54102
ATP6V1A	3.57297	3.74557	3.49167	4.17874	3.79118	3.33903	4.42593	3.16533	4.23753	4.08199	4.01756	4.52744	4.57032	4.73987	3.78288	4.07401
GRAMD1C	-3.06052	-3.03155	-3.01702	-3.15151	-1.45105	-1.31735	-0.78064	-1.57229	-0.03294	-0.01343	-0.03712	0.93905	0.46063	-0.04632	-0.5004	-0.47326
ZDHHC23	-2.50544	-2.63469	-2.72236	-2.16142	2.21536	1.29431	2.47781	1.41877	1.12786	1.18293	1.35361	2.16705	0.86449	1.14078	1.24699	1.28353
CCDC191	-1.24475	-0.87957	-0.68926	0.03393	-1.74224	-0.72949	-1.77962	-0.99892	-1.12896	-0.5296	-0.97325	0.57283	0.2912	0.11444	0.14545	0.09922
QTRT2	1.74723	1.64047	1.23908	1.40853	2.0104	1.13408	2.3432	1.20204	2.68952	1.62861	2.14161	1.82539	3.05682	2.34053	2.61067	1.93506
ZBTB20	-1.37741	-1.411	-1.57104	-0.42956	-0.18138	-0.09751	0.37111	0.58951	-0.3184	-0.39179	-0.07765	1.05224	1.03273	0.61665	2.0692	1.60836
MIR568	0.79406	0.95154	-3.32193	0.17804	1.33845	0.80903	2.63244	2.30992	0.99944	1.70097	1.80129	3.6429	3.45689	2.95477	4.6017	4.32418
BZW1P2	4.24689	4.7375	4.35553	4.96244	5.80241	4.84752	6.9371	4.48155	5.93948	5.60213	5.54612	4.36991	6.13296	6.17691	6.01149	5.7181
UPK1B	-3.32193	-3.32193	-3.32193	-3.32193	5.24214	4.09328	5.70986	3.92731	-0.89452	-1.74581	-1.84992	-2.91627	2.9487	3.49179	2.65087	2.75518
B4GALT4	1.77106	1.73591	1.9381	2.23496	3.58802	3.58823	3.99896	3.36189	3.48195	3.05884	3.09796	2.89172	2.77037	3.24852	2.38494	2.50481
B4GALT4-AS1	3.06606	2.77095	3.28308	2.79577	5.37661	4.23667	5.83201	3.47638	5.22544	4.58725	4.61391	4.28101	4.3924	4.75768	4.13219	3.99745
ARHGAP31	2.11314	2.11539	1.8898	2.06793	-1.26488	-2.7353	-0.88883	-2.19729	2.45764	2.23321	2.52906	2.92069	0.38425	0.18838	1.28531	1.20376
TMEM39A	2.17765	2.18882	2.15214	2.66595	3.15504	3.02918	3.1869	2.90853	2.73626	3.14681	2.77379	3.17633	2.39012	2.47331	2.03131	2.13389
POGLUT1	0.48684	0.81316	1.00327	1.24969	1.84879	2.02091	2.26481	2.15621	1.47618	1.13655	1.50094	1.66388	1.17569	0.86071	1.32877	0.64345
TIMMDC1	5.04159	5.0651	4.96134	5.54375	4.70305	4.61324	4.71947	4.48436	4.58644	4.45068	4.60689	4.62837	4.70183	4.79339	4.2286	4.35693
ADPRH	0.56465	0.54504	0.32836	0.62635	-2.08494	-1.88878	-2.48608	-1.73683	0.90985	0.57981	0.43761	1.06547	-0.58525	-0.10924	-0.78167	0.11728
POPDC2	0.34993	-0.29943	-0.43574	-0.51233	-0.3948	0.36458	-0.72806	0.16273	0.22652	0.02616	-0.06646	-1.43116	0.06351	0.22099	-1.20631	-1.08398
COX17	4.38767	4.29219	3.99776	4.10025	3.92023	5.0291	3.8589	5.00083	4.21267	3.99551	3.66093	2.99258	4.18017	4.2988	2.80559	2.47889
MAATS1	-1.38092	-1.2559	-1.15403	-1.26145	-3.19393	-2.49573	-2.82848	-2.98872	-1.78374	-1.78798	-1.34308	-1.14549	-1.23395	-0.13609	-1.55707	0.05373
GSK3B	2.93763	3.13291	2.98364	3.0615	3.04547	2.34182	3.42636	2.09169	4.37301	4.22229	4.37348	4.95534	4.30859	4.025	4.4965	4.63945
GPR156	-1.90752	-2.42447	-2.27756	-2.02586	-2.80059	-3.1462	-2.54159	-2.93814	-1.62873	-1.80939	-1.67876	-1.35297	-2.05579	-0.9158	-0.82417	-0.48881
LRRC58	3.48128	3.88256	3.70076	4.26431	3.63516	3.40535	4.58099	3.42193	3.71294	4.01614	3.91607	4.81605	3.90789	4.04021	4.65965	4.22214
FSTL1	4.55121	3.88103	4.67436	4.05147	-2.13024	-3.08334	-2.20943	-2.61293	6.54029	4.66911	5.94045	4.37132	5.5487	2.51337	5.58488	2.02058
MIR198	8.59118	7.85755	8.73381	8.04443	0.76124	-3.32193	2.25519	-3.32193	10.5398	8.50558	9.88487	8.32956	9.57199	6.37786	9.68574	6.07227
NDUFB4	6.20251	6.26804	6.30296	6.05056	5.76191	6.36591	5.69055	6.2598	6.22297	5.97855	6.12376	5.53398	6.11555	5.94375	5.19767	4.93414
HGD	-2.9883	-3.32193	-3.32193	-3.32193	5.20585	5.05966	5.06896	5.00443	-3.32193	-3.32193	-2.69988	-2.74342	-3.32193	-3.32193	-3.00184	-3.32193
RABL3	1.77842	1.85399	1.62472	1.7498	2.1071	1.00709	2.60907	0.89066	2.80633	2.69849	2.87987	3.03102	2.70037	2.52095	2.6879	2.56192

GTF2E1	2.99989	3.40529	3.11104	3.19249	2.48212	1.96181	2.81192	2.1644	3.38678	3.67591	3.85424	3.89537	3.28581	4.00905	3.60804	3.85859
POLQ	2.03138	1.53079	1.47519	1.50654	1.16739	0.28527	1.38117	1.12561	2.01593	0.88877	1.38663	0.89009	2.6307	2.69907	2.48196	2.60563
HCLS1	0.61586	0.81485	0.66031	0.51339	-3.23145	-3.11519	-3.32193	-2.14001	-3.32193	-2.6549	-2.61576	-2.95575	-3.32193	-3.15977	-3.32193	-3.12191
GOLGB1	2.79675	3.09973	3.10065	3.86304	3.44053	3.12394	3.58794	3.22628	3.63163	3.73426	3.50187	4.87988	3.00776	2.75019	3.34499	3.80239
IQCB1	3.38332	2.65532	2.77719	2.72053	2.70284	2.90428	2.84412	3.0381	2.8363	2.70189	2.43304	2.6939	3.42614	2.96164	1.66093	2.22363
EAF2	0.9895	1.78521	1.12296	1.37348	1.3863	1.90126	1.30682	1.46459	1.43262	0.84103	-0.27441	0.4298	1.0413	1.15202	-0.22843	-0.20054
SLC15A2	-2.51467	-1.44801	-2.40477	-1.71262	-2.81134	-1.72652	-3.16274	-1.17821	-3.12624	-3.15776	-3.32193	-2.45059	-2.6552	-1.96521	-2.54293	-2.43149
CCDC58	5.29994	5.13441	4.8348	4.90465	4.53887	4.9489	4.44905	4.80817	4.44288	4.21858	4.01534	3.20197	4.16991	3.82664	3.57782	3.31425
FAM162A	4.27766	4.47358	4.5616	4.06482	4.18739	4.91557	4.10548	4.75061	4.87667	5.6586	4.54687	4.95784	3.17967	2.97801	2.38362	2.53379
WDR5B	0.71712	1.42002	1.03751	2.23547	0.73843	0.85492	0.91967	0.93471	0.19255	0.90337	0.93773	1.40677	0.55874	0.99224	0.37314	0.44606
KPNA1	3.29115	3.44661	3.2546	3.77725	3.37756	2.48226	3.70727	2.56078	3.66634	3.19109	3.14457	3.84493	3.8353	3.83744	3.5234	3.70104
PARP9	1.28955	0.97199	0.9026	1.58732	0.69546	-0.73592	1.51392	-0.3554	1.9691	2.03059	2.38609	2.80699	1.44312	1.64304	1.47395	1.97769
DTX3L	3.46789	3.40135	3.3603	3.69748	2.01755	0.9798	2.82786	0.80885	3.91546	3.9188	4.1043	5.23613	3.11591	3.35892	3.54125	3.92244
PARP14	3.03262	2.97316	2.85607	3.30642	1.1526	0.32362	1.55778	0.02115	3.79663	3.72167	4.06914	4.95432	2.99416	3.16001	3.12564	3.44956
HSPBAP1	-0.54458	-0.46353	-0.56637	0.44587	-0.80304	-0.94679	-0.54244	-0.67432	0.3733	0.47989	0.1836	0.52652	0.76561	1.00474	0.25207	0.42275
LINC02035	0.36798	-0.1594	-0.16944	0.04319	-1.45461	-2.22001	-2.28042	-1.89659	0.10028	-0.51179	-1.34502	-0.1	0.43798	-0.23047	-0.13304	0.48555
PDIA5	3.99373	3.97132	4.19614	4.17719	4.25984	4.87704	4.31545	4.64513	4.91687	4.76232	4.89562	4.65241	2.94847	2.71471	2.95059	2.58526
MIR7110	4.55852	3.83603	4.19187	2.53515	3.27531	4.19764	3.76324	4.91434	3.66538	-0.04358	2.73211	1.81645	2.97768	-0.31384	-0.05645	1.85708
SEC22A	1.62769	1.69317	1.70092	1.96956	1.94624	1.42706	2.02259	1.17723	2.41745	2.22405	2.29543	2.41435	2.41028	1.98404	2.05983	1.60892
ADCY5	-3.32193	-3.32193	-3.0502	-3.17082	0.99341	-0.37308	0.31391	-0.10113	-2.0449	-1.50209	-2.19894	-1.78231	-3.22631	-3.05308	-3.09909	-3.09454
HACD2	3.23224	2.60279	2.73268	2.93083	5.03496	3.09293	5.53319	3.06707	4.93378	2.83211	4.30621	3.31994	4.3637	2.36453	3.78758	1.8801
MYLK-AS1	3.42119	3.29116	3.50577	3.37818	1.59752	1.85727	1.25802	1.72364	5.66214	5.57985	5.70362	6.19131	3.61597	3.87071	4.87959	4.11557
MYLK	2.28872	2.26423	2.41094	2.55071	-0.35378	-1.46609	-0.61633	-1.3839	4.91139	5.06206	5.08955	5.25502	3.00785	3.02619	3.89484	3.18311
CCDC14	1.72253	1.81725	1.88364	2.3088	1.10158	1.28432	1.4466	1.53009	1.43204	1.90916	1.43523	2.43665	2.46989	2.78097	1.62783	1.97783
KALRN	-2.8242	-2.51706	-2.48321	-2.79578	-0.81847	-1.94224	-0.95147	-2.03956	-1.95233	-1.98394	-2.24043	-1.196	-0.97239	-0.935	-0.6267	-1.60447
UMPS	3.4069	2.94654	2.90232	2.89585	3.24902	2.47685	3.34016	2.3727	2.96371	2.24223	2.66155	1.99391	3.32192	2.72926	2.77117	2.41318
ITGB5	3.73534	3.88103	4.44767	3.9237	6.97772	6.86507	6.75758	6.51722	5.15382	4.73412	5.04216	4.63737	5.78899	5.02834	6.34095	5.83781
MUC13	-2.40564	-3.32193	-2.71258	-3.32193	5.95029	4.97408	6.10344	4.94776	-2.991	-3.32193	-3.32193	-3.32193	-3.32193	-2.89292	-3.32193	-3.32193
HEG1	3.52863	3.33335	3.56873	3.39705	1.4957	0.96355	1.97533	0.87915	5.8384	5.3755	5.61223	5.65891	1.92145	1.85203	2.58631	2.57733
SLC12A8	4.01449	3.86512	3.5831	3.52587	-3.08678	-2.81458	-3.32193	-2.90994	0.95686	1.61398	0.76355	1.27392	0.13936	-0.03129	0.61603	0.24452
ZNF148	1.65355	1.7424	1.54899	2.2314	1.58001	0.73627	2.43192	0.82878	2.66565	2.60928	2.60864	3.40976	2.45022	2.10047	2.52488	2.39625
SNX4	3.55587	3.60392	3.82293	4.11702	4.27239	3.34363	4.99536	3.259	4.49687	4.44905	4.47321	4.75426	4.45099	4.25965	4.42561	4.02572
OSBPL11	2.87958	2.98844	2.82309	3.22255	2.79873	1.23073	3.16753	1.37239	3.19908	2.2239	2.75637	2.51989	2.95611	1.86466	2.70837	2.11397
LINC02614	-0.98991	-0.82153	-0.54096	-0.29841	-2.41017	-0.52599	-2.26802	-1.04707	-1.49569	-1.20584	-1.61299	-0.86749	-1.8804	-2.03506	-2.04042	-1.34918
FAM86JP	-0.99844	-0.33708	-0.26874	-0.81923	0.55971	1.04444	0.53776	0.89455	1.83748	1.53247	1.90251	1.01988	-0.36613	-0.78329	-1.57634	0.0029
ALG1L	0.02951	1.36741	0.79585	0.336	1.29046	1.78705	1.05422	1.66272	2.90381	2.81246	2.98564	2.24055	-0.56273	-0.42114	-0.48337	0.44129
SLC41A3	2.44812	2.27624	2.24637	2.07624	2.14087	2.07683	1.48159	2.0858	2.82643	2.64728	2.59451	2.23562	2.1904	2.21708	2.22498	2.16269
ALDH1L1	-2.9624	-2.99525	-3.14036	-3.12922	-2.85243	-2.89607	-3.04533	-2.70033	-2.84192	-2.41635	-2.82577	-2.70221	-2.10141	-1.38539	-1.52041	-0.7983
KLF15	-1.7821	-2.38262	-2.91827	-1.4142	1.35256	3.0502	0.02255	2.99574	-2.92494	-3.32193	-3.32193	-3.32193	-1.32593	-1.32978	-2.98829	-1.94585
ZXDC	2.0461	2.04882	2.13319	1.78355	2.01693	1.78179	1.57785	2.05846	2.10633	2.31109	2.29208	2.4752	1.37626	1.31989	1.64694	1.61099
CHST13	-0.18296	0.50051	-0.11662	-0.89396	3.62243	5.78295	2.45573	6.14867	1.33369	1.63787	1.61644	-0.11632	-1.71822	-1.18881	-0.7441	0.40897
TXNRD3	0.96685	0.34381	0.62732	1.0174	0.93557	1.17482	0.85392	1.18626	2.55773	2.19633	2.05414	2.16454	1.4579	1.45294	1.64705	1.76439
CHCHD6	3.1156	2.63609	2.72013	2.41904	2.8784	3.13597	2.25314	3.28128	3.93995	4.04804	3.89554	3.1079	2.79576	2.63133	2.62317	2.46664
PLXNA1	4.0453	4.02382	4.16689	3.54655	3.92844	2.97157	3.44144	3.04703	4.57033	5.44965	5.0606	6.10846	3.64458	3.55841	4.27524	4.79968
TPRA1	6.51542	6.20539	5.98665	6.0248	4.75212	4.5461	3.99777	4.63796	4.78373	5.15741	4.90118	4.19132	3.92112	3.73435	3.52297	3.97139
MIR6825	5.44658	4.65408	5.4359	4.16302	2.03401	3.72012	1.19871	3.81627	3.05332	3.65232	3.46777	-3.32193	3.06891	0.02571	1.79418	1.2601
MCM2	5.84533	5.67932	5.37496	4.96348	5.23657	3.58038	4.60861	3.72321	5.84364	4.78483	5.37097	3.73587	5.23396	5.62036	5.77307	5.56193
PODXL2	-2.04682	-0.6942	-1.06322	-0.86721	4.87097	5.45715	4.06781	5.6922	2.9968	3.29115	3.04061	2.38294	3.94469	4.23754	4.50954	4.51277
ABTB1	2.84923	3.04864	3.32827	3.04024	1.6894	2.17081	1.26858	1.47885	2.8686	3.82353	3.31599	3.50279	1.50542	1.83921	1.72487	2.71514
MGLL	5.02707	5.22546	4.90996	4.87489	-1.32124	-1.65516	-1.63345	-1.45402	5.9266	5.78012	5.44189	5.65218	5.4007	5.22167	4.88082	5.37819

RUVBL1	4.88452	4.66506	4.57761	4.45535	4.66228	3.88632	4.56315	3.75519	5.29942	4.75799	5.23868	4.40212	4.75587	4.4292	4.61542	4.40815
EEFSEC	3.83163	3.62455	3.75562	3.2646	3.33341	3.21301	2.53246	3.31289	4.23025	3.84928	4.05933	3.37463	2.39881	2.08039	3.171	2.32527
GATA2	1.38207	1.01046	0.60677	0.73125	1.95236	2.74024	1.4839	2.76276	2.55735	2.50822	2.41388	1.04514	-0.03992	-1.42759	-0.58854	-1.078
GATA2-AS1	-2.89974	-3.32193	-3.32193	-2.42793	2.81358	2.56289	2.02383	2.51612	1.90126	2.13568	0.94957	0.87994	-0.38491	-1.35137	-0.85099	-0.66474
RAB7A	7.47416	7.61257	7.66858	7.78773	6.26777	6.40025	6.79644	5.99713	6.50585	6.61223	7.15538	6.80867	6.22627	6.48126	7.09122	6.45198
FTH1P4	2.10783	1.30036	1.70451	1.03486	-1.32905	-0.92768	-1.46589	-1.90546	-0.41017	0.39838	-1.35557	-0.15474	-1.64429	-1.64783	-3.32193	-2.07728
ACAD9	3.9682	3.71652	3.90857	3.91334	3.45055	3.23318	3.21075	3.41707	4.20368	3.54274	3.90173	3.48807	3.64316	3.36562	3.54977	3.60408
KIAA1257	1.48168	1.2412	1.42116	1.26031	0.7397	0.74611	-0.36389	0.80136	1.58444	0.87519	1.01782	0.37438	-0.07629	0.09704	0.05544	0.40245
RAB43	4.251	4.39179	4.43539	4.03679	4.56222	4.62218	3.7762	4.36799	4.27964	4.65718	4.34576	4.24111	3.38447	3.18702	3.82676	4.29947
ISY1-RAB43	4.86379	4.89664	4.90872	4.65294	4.76221	4.81994	4.11053	4.60608	4.91716	5.16059	4.88662	4.75102	3.79332	3.80885	4.0197	4.44447
ISY1	4.2423	4.20874	4.09895	4.18058	2.91783	2.98278	2.66723	3.09409	4.21519	4.21159	4.00317	3.82032	2.92888	3.20369	2.61671	2.66466
CNBP	8.30243	8.28697	7.98894	8.62035	6.83922	6.51346	6.95699	6.45086	7.78498	7.68881	7.52664	7.49836	7.49603	7.21708	6.89107	6.65418
COPG1	5.7048	5.87254	5.78678	6.24038	5.67368	6.12171	5.64344	5.98247	6.20115	6.11356	6.30199	6.54529	4.59621	4.79411	5.0592	5.16413
HMCES	4.98755	4.90354	4.82521	5.02501	4.69138	4.44702	4.59415	4.55163	5.01006	4.20901	4.57052	4.14413	5.22645	5.1488	4.91205	4.96503
H1FX-AS1	4.45641	5.18169	5.23081	4.95474	3.8627	4.24319	3.62572	4.52288	4.58646	5.45937	5.24423	4.13495	3.38995	5.04274	4.7818	4.79033
RPL32P3	1.59081	1.16791	1.45076	1.82254	1.43582	1.55576	1.29184	1.9979	-0.28443	-0.0528	0.01493	0.29533	-0.47194	0.26669	-0.27947	0.21338
SNORA7B	2.98497	3.14957	3.12094	3.67669	2.2034	3.35921	2.26026	3.74226	1.43085	1.1723	1.27125	0.75529	-0.03672	0.88245	-0.65951	2.15674
EFCAB12	-3.05975	-2.3172	-2.04895	-1.67189	-0.8774	-0.80211	-1.11172	0.01803	-3.16382	-3.06834	-3.32193	-3.19171	-3.01836	-2.55634	-2.04274	-1.96815
MBD4	3.30487	3.11966	3.40512	3.64138	3.39385	3.20829	3.99503	3.0716	2.67586	2.25796	3.08493	2.9256	2.46867	2.23929	2.54616	1.62246
IFT122	1.08519	0.92525	0.9923	1.03003	1.07586	1.05581	1.14577	1.34103	2.2853	1.8799	2.00777	2.25949	1.54626	1.78186	2.2156	2.42812
PLXND1	2.36987	3.0333	2.97308	3.02842	2.94849	2.96118	2.67333	2.81539	1.42971	2.19796	1.72038	1.95523	2.69489	2.17748	3.63189	3.80561
TMCC1	1.07387	1.17007	1.13305	1.50804	2.28205	2.43001	2.69686	2.11324	1.27445	1.05751	1.39482	1.88173	1.92659	1.95656	2.07809	2.02555
TMCC1-AS1	-0.50558	-0.54764	-0.48082	-0.71633	-1.43074	-0.29883	-1.33008	-1.06072	-1.66816	-0.94089	-1.8682	-0.75299	0.57933	-0.15291	-0.37108	-0.43647
FAM86HP	-0.00747	-0.2575	0.29961	0.61436	-0.914	0.57267	-0.0131	0.90603	-2.56199	-0.83597	-0.47795	0.36471	-0.70347	-0.1654	-1.17874	-0.68051
PIK3R4	3.3856	3.49992	3.17442	3.8668	3.03877	1.90094	3.21157	2.04197	3.38825	2.84238	2.86959	3.4056	3.22872	3.49277	3.14159	3.45445
ATP2C1	3.91721	4.22422	4.15852	4.6375	3.7425	3.02622	4.31278	3.22157	4.32247	4.35699	4.2931	5.12731	4.14397	4.19809	4.28861	4.31229
ASTE1	2.38358	2.55964	2.67925	3.16855	1.59186	2.11641	1.83469	1.87013	2.35837	2.15954	2.27475	2.89995	2.47169	2.07094	2.19404	2.07407
NEK11	-1.15894	-0.84817	-1.41608	0.12169	-1.36103	-2.02011	-1.06249	-1.4119	1.40262	1.97431	1.4316	2.22929	0.51448	0.89917	0.69789	0.93386
NUDT16P1	-3.32193	-2.51164	-2.83887	-2.8122	-2.32338	0.28694	-1.3883	0.65748	2.34894	2.50278	2.0716	2.495	-2.98508	-3.32193	-3.32193	-3.32193
NUDT16	2.73047	2.33483	2.254	2.02596	1.83411	1.71936	1.66981	1.7013	3.08857	2.83552	3.27922	2.82412	2.35536	2.07729	2.39081	2.32891
MRPL3	6.00815	5.6492	5.48666	5.57699	5.65324	5.08882	5.98384	5.01037	6.19706	5.98871	5.8475	5.7513	5.80733	5.45633	5.34738	4.95073
DNAJC13	2.87261	2.83637	2.94964	3.41886	2.9751	1.71552	3.63364	1.68629	3.63038	3.39259	3.41654	4.37948	3.92622	3.64166	3.99667	3.69901
ACAD11	2.10869	1.97191	2.49626	2.0317	2.6944	2.70424	2.56615	2.54673	2.77763	3.06299	2.85633	2.67854	2.42267	2.74164	2.29453	2.49217
NPHP3-ACAD11	1.73052	1.84375	2.26609	2.40123	2.18246	2.44082	2.38026	2.32724	2.38385	2.63484	2.72314	3.02646	2.69201	2.81046	2.51663	2.7363
ACKR4	0.41059	-0.20674	0.34838	0.00749	0.35008	0.46191	0.2816	-0.19819	0.17876	0.98316	1.1054	1.44463	1.14618	0.80951	0.38538	0.45017
UBA5	2.71809	2.72968	2.88224	2.82562	2.75765	2.90954	3.17554	2.93709	3.24319	3.14894	3.12054	3.32098	2.69927	2.90887	2.53834	2.53387
NPHP3	0.96798	1.15005	1.51987	1.9285	1.03108	1.31866	1.62977	1.44697	1.65969	1.49344	1.93405	2.34135	1.95585	2.0102	1.87465	2.03194
NPHP3-AS1	-0.52429	-0.38051	-0.22068	-0.10989	-0.05948	-0.20521	-0.14284	-0.74749	0.37141	0.08614	0.49617	-0.10098	-0.22119	-0.04196	0.22117	0.46263
TMEM108	-2.86935	-3.05075	-3.32193	-3.32193	-3.11217	-2.68145	-2.99083	-2.9119	-0.16428	-0.74309	-0.38078	-0.99505	-3.32193	-3.32193	-3.32193	-3.32193
CDV3	5.97924	5.10058	5.77947	4.94765	5.3126	3.41736	6.05617	3.28381	7.37615	5.43816	7.1592	5.30468	6.29952	3.53967	5.88173	3.06272
TOPBP1	3.45634	3.34602	3.20654	3.55151	3.94483	3.01537	4.33742	3.20776	4.1868	3.67347	3.82086	4.17894	4.12668	4.45336	4.02345	4.0273
TF	-2.26023	-2.23869	-2.51335	-3.32193	7.01442	8.57172	6.73583	8.43272	-2.44017	-2.98606	-2.51377	-2.56728	-2.81001	-2.96246	-2.71872	-3.08699
SRPRB	4.30473	4.00385	3.59819	4.06743	5.52414	5.69229	5.57832	5.56157	4.45556	4.27273	4.37961	4.15726	4.23873	4.26658	3.95302	4.00405
RAB6B	-1.70466	-1.94579	-1.89737	-1.17581	1.90488	1.3469	1.48667	1.61679	2.36393	3.08325	1.77983	2.52929	-1.42125	-0.29274	-1.20698	-0.22304
SLCO2A1	-3.32193	-3.32193	-3.32193	-3.32193	-3.32193	-3.32193	-2.97227	-3.32193	-0.26141	0.60088	-0.69563	-0.76748	-3.32193	-3.32193	-3.13256	-3.32193
RYK	2.99636	2.98081	2.87439	2.98713	2.65948	2.40002	2.98942	2.02881	3.36168	3.85677	3.58854	3.88508	2.65442	2.89255	2.32426	2.52994
RPL39P5	5.83212	5.29612	4.95525	4.52434	3.31285	1.13569	3.58976	2.27164	7.11827	6.8982	6.75113	6.13411	6.15645	5.55223	4.89754	3.53238
AMOTL2	5.00902	4.30185	3.86081	2.96263	3.16309	-0.15212	2.92026	1.05283	7.12355	6.96701	6.99299	5.48126	5.15185	4.60519	4.42613	3.30875
ANAPC13	4.20621	4.04726	3.85627	4.21587	4.33978	4.43222	4.70833	4.39745	5.30897	5.37622	5.22049	5.18851	4.80278	4.92386	4.3477	4.41863
CEP63	1.50538	1.64274	1.41809	1.53077	1.32465	1.16413	1.5146	1.08803	2.43459	2.26907	2.03205	1.99681	1.66786	1.66833	1.55326	1.27559

EPHB1	-2.16661	-2.73607	-1.56518	-1.89419	-3.25513	-3.32193	-2.8195	-3.14215	0.10122	0.5523	-0.37633	0.40814	-3.32193	-3.32193	-3.32193	-3.17282
PPP2R3A	1.02229	1.01648	1.26983	1.54247	-0.40329	-0.59788	0.12317	-0.74971	2.54413	2.37041	2.20603	4.1619	0.85968	0.95026	1.5448	1.52773
MSL2	2.65532	2.45569	2.54123	2.55313	2.17177	1.31456	2.39938	1.35844	3.17092	3.08034	3.2701	3.75808	2.13626	2.35563	2.64767	2.51203
PCCB	2.78246	2.62573	2.50872	2.81421	3.54051	3.37035	3.5905	3.18717	3.61835	3.16403	3.52228	3.28558	3.33427	3.21008	3.58427	3.18104
STAG1	2.22955	2.34943	2.11458	2.59254	2.714	1.86017	3.47811	1.86451	3.31691	3.05369	3.16744	3.84336	3.16439	3.30844	3.38687	3.25265
SLC35G2	0.10785	0.37727	-0.23329	0.84096	2.33455	3.09087	2.78522	3.0944	2.19605	2.25661	2.02715	2.39669	1.94806	3.03029	1.08565	2.1468
NCK1-DT	2.36486	2.4661	2.3191	2.49919	1.05917	0.89316	-0.21682	0.7782	0.30608	0.44037	-0.26633	0.36089	1.54016	1.47045	0.93728	1.34373
NCK1	3.03207	3.20407	3.01675	3.78346	2.13403	1.79443	2.64661	1.65377	3.51451	3.59073	3.40972	3.59726	3.03132	2.97048	2.44052	2.22416
IL20RB	1.63824	1.7553	1.33157	2.40329	0.76508	0.75409	1.54217	0.91972	1.86334	1.99048	1.91602	1.67544	1.44932	1.36425	0.6213	0.53362
CLDN18	-2.47383	-3.02972	-2.0458	-1.61146	-1.56545	-0.27215	-2.1159	-1.67058	-3.02033	-3.32193	-3.05024	-2.50757	-2.61986	-2.92983	-1.30198	-1.76022
DZIP1L	1.78815	1.41835	1.81821	1.53435	0.57059	-0.0424	0.44751	0.40703	1.54859	1.39875	1.70992	1.46929	1.43471	1.19263	1.02772	1.38245
DBR1	2.79242	2.76528	2.88821	3.23767	2.5087	1.80821	2.56638	1.98408	3.05196	2.47793	2.81159	2.7912	3.02896	3.02727	3.14895	3.03161
ARMC8	1.5032	1.55989	1.53657	1.72403	1.74993	1.73774	2.30467	1.62333	2.50325	2.53787	2.38376	2.84751	2.91809	2.98428	2.51999	2.45106
MRAS	0.85368	0.78965	0.94125	1.40047	0.89989	0.95379	0.61742	1.01036	1.12663	1.11733	1.53188	1.65613	-0.79176	-1.60625	0.01158	-0.17568
ESYT3	-1.74823	-1.89638	-1.94762	-1.78763	-1.93686	-1.12111	-2.86878	-1.09922	-3.32193	-3.32193	-3.20835	-3.12041	-1.8707	-1.90698	-1.95161	-1.84962
CEP70	1.7882	1.89013	1.99644	2.06306	2.22617	2.07685	2.62782	2.03177	3.48056	3.43086	3.03148	3.77784	2.59849	2.85903	2.62891	2.64818
FAIM	1.38732	1.06319	1.22373	1.3529	1.80395	2.12396	2.44292	2.19808	1.88324	1.19355	1.64199	1.39486	2.1571	1.10654	1.48193	0.88627
PIK3CB	2.55676	2.67878	2.45938	3.07467	3.13962	2.21672	3.8308	1.98016	3.86592	3.382	3.37617	3.93923	3.76149	3.74231	3.45233	3.50828
EEF1A1P25	1.76887	1.90458	2.45163	-1.03904	0.34564	1.49832	-0.06237	1.19268	1.48835	2.62858	1.00269	0.46212	0.01694	-0.47063	0.53122	0.61793
FOXL2	0.59471	0.58841	1.08229	0.27312	-1.15104	-2.26808	-2.32667	-2.3589	-1.28889	-0.58976	-0.74629	-0.98974	1.52222	1.41982	1.7985	1.64978
FOXL2NB	-1.18481	-1.11275	-0.77794	-1.04049	-3.32193	-2.89578	-2.92439	-2.46712	-2.48186	-1.53195	-2.05386	-1.24398	-0.60127	-0.5768	-0.44855	-0.55546
MRPS22	4.00703	3.63241	3.78141	3.9008	2.97673	2.7167	3.23701	2.4421	4.13196	3.43396	4.11723	3.75168	3.25982	2.05793	2.73414	1.70767
COPB2	4.58893	4.7325	4.74087	5.12697	4.89651	4.79829	5.31376	4.77538	5.21218	5.2199	5.23159	5.51729	4.46762	4.51346	4.40266	4.47129
NMNAT3	-3.32193	-2.9972	-3.32193	-2.67317	-1.02441	-0.68272	-1.19798	-1.24813	-1.36754	-0.41388	-1.3116	-1.11265	0.95038	1.28392	0.91241	0.83989
CLSTN2	-2.81057	-2.94518	-3.32193	-3.05724	-1.20347	-3.32193	-0.99413	-3.32193	-0.73544	-1.15627	-0.99391	-1.22549	3.78454	4.58752	5.05533	4.54515
SLC25A36	-0.69269	-0.93634	-0.74765	-0.48892	1.82496	1.92144	2.61743	2.00024	3.30349	3.74102	3.24976	4.2551	2.02336	1.58168	1.46041	1.27905
PXYLP1	-2.65976	-2.71501	-2.50713	-2.46688	-1.29782	-1.57535	-1.34523	-1.40694	0.85287	1.45111	0.41241	2.67936	-0.11104	-0.26527	0.16048	-0.23398
ZBTB38	2.8138	2.82345	2.89032	3.10705	1.85876	0.23186	2.43841	-0.03657	4.08251	3.85512	3.72637	4.84779	3.15976	2.22692	3.29549	2.60714
RASA2	2.45612	2.25276	2.76002	2.58934	1.21881	0.81797	2.34496	0.60133	2.9452	1.94819	2.86875	2.63551	2.92364	1.55621	2.58147	1.09403
RNF7	4.53659	4.65097	4.52216	4.85758	4.03266	4.75783	4.20356	4.52783	4.60595	5.00603	4.70926	4.59441	4.78122	4.73287	4.0413	4.11536
ATP1B3	6.72145	6.61322	6.50524	6.69981	5.96292	6.08366	6.27577	5.97999	5.79002	5.35945	5.41289	4.96579	6.45961	6.43779	6.07292	6.25099
TFDP2	2.16895	1.98773	1.87301	1.91257	1.7725	0.90126	1.77347	0.64039	1.8704	1.61195	1.89359	2.10615	2.34279	1.72389	2.60391	2.38362
GK5	0.58919	0.20571	0.28605	0.49934	1.4184	0.94119	1.55403	0.90235	0.8354	-0.18152	0.19517	0.49278	2.789	2.32583	1.99142	1.68751
XRN1	1.09787	1.55356	1.31861	1.94608	0.72862	-0.22913	1.34292	0.07769	2.27599	2.61236	2.27222	3.46016	2.1518	2.33203	2.40962	2.36472
ATR	1.93606	1.97094	1.65704	2.31753	2.07463	0.73979	2.4709	0.83538	2.39557	1.81305	2.2109	2.5563	2.41039	2.16448	2.10578	2.16419
PLS1	2.27361	2.194	1.99029	2.38073	4.36088	2.71755	5.19796	2.56233	2.34365	1.75088	1.24056	1.60709	3.68999	4.05436	3.24671	3.04772
TRPC1	1.15859	1.53373	1.62719	2.17569	0.89216	0.73423	0.87354	0.77142	1.18641	2.67679	1.1967	3.14559	1.82503	2.5279	0.98585	1.85222
PCOLCE2	2.72515	2.4678	2.69326	2.66682	1.78787	2.82125	1.75207	2.83917	1.77323	1.71497	1.54531	1.39895	1.97962	2.19357	1.66276	1.74107
PAQR9	-1.14015	-0.54568	-1.74421	-0.87163	3.40705	2.79731	2.43762	2.8889	-0.5474	0.27859	-1.43442	0.05013	2.51845	3.26805	2.28725	2.80808
PAQR9-AS1	-0.55782	-0.04844	0.07959	0.29896	2.48988	2.0334	1.33643	1.90041	-0.29655	0.06764	-1.10866	-0.48244	1.14029	2.17402	1.32974	1.73151
U2SURP	3.76043	3.7268	3.80674	4.25548	3.19306	3.12864	3.93074	3.24777	3.43871	3.69193	3.83751	4.22872	3.60848	4.01236	3.73947	3.56281
CHST2	-3.32193	-3.32193	-3.32193	-3.04236	-2.07897	-3.09387	-2.14249	-2.83348	3.16664	3.46086	3.3761	3.08987	-1.87561	-2.02649	-2.02592	-1.45611
SLC9A9	-0.79899	0.24737	-0.28642	0.01574	-3.00932	-3.32193	-3.08649	-3.32193	-2.10508	-0.58279	-0.61965	0.23896	-1.69768	-1.04266	-1.22988	-1.33458
ST13P15	-0.59753	-0.45495	-0.10375	-1.85197	-0.65297	-0.92873	-1.46683	-1.51387	-0.97862	-0.13402	0.06238	0.97159	-2.69035	-1.92034	-0.99531	-0.96999
DIPK2A	2.91188	2.79877	2.24034	2.58747	2.55853	0.69238	1.67336	1.52403	3.07087	2.72083	1.5643	2.54243	2.45272	1.97104	1.12414	1.39287
RPL21P39	1.23243	0.7851	1.13361	-1.72331	0.07783	-0.13237	-0.51464	0.07809	0.04807	2.24748	0.94941	0.02004	-1.50728	-1.51096	-0.83586	-3.32193
PLSCR4	-1.81802	-0.14603	-0.96212	0.45927	-1.88068	-1.40177	-1.70997	-0.73146	0.50915	1.89903	-0.34969	2.09676	-2.42424	-1.60238	-3.32193	-1.66651
PLSCR1	3.76001	3.55357	3.43567	4.10536	2.10689	1.35559	2.69524	1.45286	3.98886	3.88678	3.95371	4.16739	2.86812	2.72973	1.9279	1.99699
ZIC4	-3.19683	-3.32193	-3.32193	-3.32193	-3.16435	-3.1955	-3.32193	-3.32193	-3.32193	-3.32193	-3.32193	-3.32193	-0.53739	-0.04429	-0.29014	0.21508
ZIC1	-3.16613	-3.32193	-3.32193	-3.12789	-1.06563	-1.70118	-1.0493	-1.42947	-3.32193	-3.32193	-3.16084	-3.32193	0.02883	-0.01421	0.06655	0.62917

AGTR1	-3.32193	-3.32193	-3.32193	-3.32193	4.8578	5.01732	4.66368	5.09619	1.79575	0.9479	0.06736	1.11979	-3.32193	-3.32193	-3.32193	-3.32193
UBQLN4P1	-0.26431	-0.36371	-1.12853	-1.22969	0.26078	-0.3271	-0.59257	-1.97841	0.5874	0.23932	0.52534	-0.48845	0.69532	0.19265	0.4161	0.83362
GYG1	3.36635	3.02482	3.26402	3.37813	3.76258	3.26956	4.00822	3.12497	4.49714	3.8268	4.12203	4.56403	4.3937	3.51351	3.65227	3.4755
HLTF	3.1827	3.19644	3.38527	3.59149	3.67993	2.35846	4.1618	2.37933	4.17199	3.36457	3.66517	3.91867	4.23524	2.99003	3.9781	2.87617
HLTF-AS1	2.36442	2.30097	2.49546	2.19715	4.13537	0.30396	4.48832	-1.6329	4.37512	3.60677	4.20985	3.64505	3.89704	2.91564	4.57578	3.8415
HPS3	3.44043	3.7075	3.97621	4.29151	3.3917	5.80621	3.88821	5.76479	3.30406	3.49334	2.96315	4.53595	3.89653	4.13035	3.84408	4.35326
CP	2.61625	2.99643	3.17154	3.68001	4.98652	6.82196	5.31608	6.49503	0.59415	0.92229	0.35516	2.05097	2.82621	2.56589	2.61416	3.69273
CPHL1P	-0.41303	-0.15656	0.4209	-0.0789	-2.37957	-1.4767	-2.52565	-1.30383	-3.32193	-2.85675	-3.05364	-2.3695	-3.32193	-2.77396	-3.32193	-3.06791
TM4SF18	1.89497	2.07073	1.93876	2.77251	2.53046	2.2868	3.26369	1.88922	3.29005	3.19646	3.1346	4.24002	7.01477	6.06404	6.78647	5.7841
TM4SF1-AS1	-3.32193	-3.32193	-2.33088	-2.28462	1.22066	0.96667	1.81717	1.47361	-0.19233	0.83846	0.48068	0.27071	0.19529	0.61513	-0.79198	-0.57892
TM4SF4	-2.53504	-2.82924	-3.32193	-3.32193	6.92085	6.80564	6.97224	6.58096	0.61663	0.74309	0.00244	0.56096	-1.00642	-0.25825	-2.06791	-0.66456
WWTR1	2.40933	2.761	0.94501	1.57364	1.68649	-0.22513	1.045	1.20508	2.8563	3.65927	2.27505	2.62717	4.82683	4.99134	4.41341	3.1206
WWTR1-AS1	2.47415	2.5906	1.07962	1.21461	1.82553	0.57215	-0.38089	0.79189	2.5913	3.68224	-0.35932	0.45624	3.13962	3.05191	0.73613	1.69708
COMMD2	2.11904	1.74533	1.7822	2.36116	2.47714	2.0755	3.15543	2.23716	2.42342	2.68987	2.22052	3.11138	3.56364	3.74434	3.33109	3.31383
ANKUB1	3.36618	2.99167	3.28167	2.67543	2.83322	1.60875	2.92502	0.99296	4.41107	3.61689	4.38632	3.24783	2.97594	2.0867	2.87269	2.08916
RNF13	3.27602	3.25431	3.22622	3.54723	4.0368	3.72994	4.56544	3.57704	3.70926	3.73309	3.65754	4.33912	3.3975	3.44854	3.58801	3.47538
PFN2	6.0853	5.62731	5.97959	5.71833	4.52723	4.31638	4.65435	4.01083	6.78695	6.08101	6.68564	6.4993	5.97582	4.81179	5.29186	4.00364
TMEM183B	3.64212	3.22763	3.12773	3.32221	3.76248	4.34159	3.78451	3.96801	3.76723	3.50833	3.57677	3.38834	4.48993	4.63582	4.53751	4.18284
TSC22D2	2.92436	2.93129	3.15577	2.25266	2.82807	1.52727	2.5335	1.34654	3.40285	2.77871	3.23523	2.09264	2.24487	1.91801	2.84067	2.10982
SERP1	5.94235	6.06787	5.79041	5.98255	5.58782	6.05115	5.76537	5.75678	5.41838	5.54318	5.1364	5.35859	5.18964	5.24351	4.91988	4.99312
EIF2A	5.32144	5.3675	5.28485	5.6267	4.30341	4.25661	4.9444	4.11326	4.94199	5.37116	4.72322	5.91877	4.80998	4.32737	4.19288	3.86451
SELENOT	4.95308	4.59572	4.68339	4.76966	4.34339	2.46663	4.54098	2.38194	4.66967	3.40321	4.30009	3.72591	5.0208	2.92637	4.17727	2.50755
ERICH6-AS1	-1.79738	-1.26532	-0.87638	-0.30251	0.98061	1.36012	0.20316	1.22186	0.37844	1.24027	1.16275	0.10921	-0.39269	0.3473	-0.45956	0.80611
SIAH2	4.33257	4.33429	4.22121	3.61679	4.57164	4.42891	3.94567	4.2404	5.27843	4.89512	4.79922	4.41458	3.95025	3.98997	3.9935	4.0427
SIAH2-AS1	4.78423	4.53676	4.75471	3.48653	5.03729	4.54906	4.08921	4.437	5.82171	5.13175	5.46664	4.67955	3.22307	3.45331	4.07919	4.09614
MED12L	-3.32193	-3.22562	-3.32193	-3.21405	-3.32193	-3.23489	-3.09267	-3.32193	-1.29546	-1.72906	-1.65914	-1.26934	-3.06765	-2.461	-3.08784	-2.75618
IGSF10	-3.32193	-3.21855	-3.21303	-3.099	-3.32193	-3.32193	-3.23545	-3.32193	-2.16434	-1.94592	-2.89668	-0.80956	-2.37087	-0.99142	-1.31007	-0.51325
AADACP1	-1.59315	-0.90789	-1.82214	-0.44801	-2.33427	-3.32193	-1.36566	-3.32193	0.17793	1.37419	0.6586	2.13044	2.98679	1.595	1.25302	1.52888
AADAC	-2.06136	-1.27455	-1.04134	-1.57795	1.31681	-0.58957	1.5688	-0.61389	-2.26417	-0.45767	-0.78086	-0.23468	0.11667	0.73283	-0.47004	-0.97522
SUCNR1	-3.32193	-3.32193	-3.32193	-3.32193	-3.21245	-3.32193	-3.32193	-3.32193	-3.32193	-3.32193	-3.32193	-2.71035	0.80185	1.62534	-0.62279	1.51272
MBNL1	2.70783	2.36439	2.6542	2.86779	2.7868	1.1203	3.48217	1.2912	3.95907	2.83346	3.61896	3.5071	4.32445	2.30986	4.23105	2.54965
MBNL1-AS1	-1.34133	-1.56439	-1.29621	-1.76893	0.42911	-2.1899	0.16756	-1.89849	1.0892	0.42097	1.31086	0.42248	0.98816	0.117	1.55372	0.37427
P2RY1	-3.32193	-3.32193	-3.32193	-3.32193	-3.18743	-2.89013	-3.04127	-2.56995	0.91968	0.61441	0.4215	0.48305	-3.32193	-3.08363	-3.17159	-3.16844
RAP2B	3.75009	3.92048	4.04007	3.62216	2.67084	2.12944	2.72764	1.74412	4.95349	4.91448	5.07594	4.67842	3.52928	2.96303	3.78011	3.48555
ARHGEF26	1.11395	0.72709	0.65455	0.75586	1.36744	0.74207	1.17922	1.11675	1.63582	0.13137	0.54817	-0.10305	2.01166	1.91671	1.8787	1.87726
DHX36	2.68741	2.90813	2.50376	3.47393	2.27238	1.68725	2.81165	1.98663	2.86119	2.77072	2.76529	3.42268	3.16901	3.33769	3.03988	3.13722
MME-AS1	5.17369	5.60291	5.62011	5.52765	-3.32193	-1.78814	-2.41507	-1.6305	5.93599	6.82357	5.82061	7.52387	2.52467	1.41199	0.91828	1.6826
PLCH1	-0.43756	-0.99779	-0.83271	-0.82518	0.75701	0.34547	1.25862	0.01968	-1.92414	-2.08535	-2.16296	-1.2585	-0.15712	0.28088	0.56976	0.64863
C3orf33	0.11367	0.2158	0.18155	0.42185	2.06807	2.28699	2.48534	2.41528	0.90729	1.20756	0.96061	1.18403	1.98058	2.32529	1.35917	1.88152
SLC33A1	2.60071	2.95011	2.89253	3.45542	3.06147	2.95038	3.25918	3.02933	2.2098	2.43969	2.19424	2.96418	2.22621	2.58271	2.12317	2.32005
GMPS	4.49614	4.4066	4.23347	4.29332	3.75529	3.22587	3.98447	3.15382	4.61463	3.68497	4.04175	4.09529	4.11761	3.86627	4.10896	3.77392
SETP14	3.13955	3.58188	3.33712	4.14733	1.58848	1.17459	2.34458	1.34031	2.21624	1.81932	2.14684	2.38985	1.34353	1.89097	1.83999	1.68852
KCNAB1	-0.91478	-1.17377	-1.73078	-0.99892	-1.65983	-0.52383	-1.26122	-0.3454	-2.19434	-1.92228	-1.98541	-1.76964	-1.94492	-2.40796	-1.87287	-1.74196
SSR3	6.95702	6.97658	6.64926	6.39516	5.99456	6.08902	6.4745	5.83188	6.62293	6.06642	6.25583	6.42679	6.3512	5.6552	5.65312	5.24832
TIPARP	3.41448	3.57124	3.62105	4.09285	2.89773	1.89608	3.80508	1.63183	4.62066	4.37624	4.7592	5.49252	4.89914	3.85943	4.46336	4.25283
LINC00886	-1.45804	-1.25466	-0.56195	-1.12377	-1.55012	-2.82559	-1.88579	-2.75335	-2.36279	-2.65522	-2.28502	-2.49816	-1.37199	-2.76066	-1.58791	-1.95158
PA2G4P4	0.68726	0.35522	0.15222	-0.39	0.49639	0.97003	0.47209	0.7329	0.9499	0.31001	0.61207	-0.59898	-0.37944	-0.70703	-1.07053	-1.04555
CCNL1	3.34301	3.3839	3.62406	4.11515	2.65805	3.22919	3.21659	3.30329	1.91809	2.19665	2.20002	2.95953	2.70539	2.77975	1.752	2.03561
VEPH1	0.46355	0.63112	-0.1302	0.6599	-3.25114	-2.87918	-2.79371	-3.13182	1.56707	1.42262	1.17573	1.69747	-0.45234	-1.11865	-1.47377	-1.69895
PTX3	5.00473	4.68844	4.24156	4.8995	-2.89989	-2.83188	-3.32193	-2.76039	5.32522	5.2906	4.97033	4.483	3.83773	2.66586	1.67531	1.83779

SHOX2	-2.16442	-1.78619	-2.13555	-1.15337	-1.36781	-0.74242	-1.60051	-0.54471	1.39029	2.20932	2.31518	2.39581	-3.01037	-3.01137	-3.32193	-2.39535
RSRC1	2.71733	2.43149	2.40901	2.98756	2.55696	2.78204	2.83522	2.56731	2.75415	2.59411	2.5173	3.32913	2.61179	2.8417	2.92898	2.75083
MLF1	-3.32193	-3.32193	-2.99122	-2.97197	1.85919	1.81965	2.33131	1.90573	2.44186	2.18447	1.57702	1.89851	-3.32193	-3.32193	-3.32193	-3.32193
GFM1	3.17306	3.01541	2.83071	3.31624	3.35847	2.61987	3.77312	2.54695	2.75106	2.24497	2.51975	2.42726	3.30081	3.24863	2.81405	2.99835
LXN	1.24524	1.69051	1.24003	1.47037	3.03336	1.05842	3.40611	0.76199	2.94817	2.73736	2.6678	2.31962	2.62391	2.99192	2.51517	2.6769
MFSD1	4.53159	4.53887	4.5016	5.07353	3.38508	3.00704	4.09261	2.9632	2.67697	2.52282	2.4588	3.00054	2.82365	2.87374	2.30698	2.77527
IQCJ-SCHIP1	2.58848	2.53363	2.48689	2.62449	2.48412	1.01592	2.51175	0.50493	2.55337	2.4766	2.51055	2.4915	2.95235	2.91871	2.8261	2.54536
SCHIP1	1.26574	1.22943	1.13274	1.60162	0.80491	-0.18427	1.21843	-0.55725	0.84739	0.83496	0.97966	1.39568	1.71079	1.69171	1.21388	0.76971
IL12A-AS1	-0.15284	-0.63683	-1.62448	-2.27762	-2.70264	-3.32193	-3.32193	-3.32193	-1.43831	-1.7232	-1.38333	-2.64535	-2.21507	-3.10254	-3.32193	-3.32193
IL12A	2.22201	1.34143	0.74636	0.66801	-1.84821	-1.97786	-2.54771	-3.32193	0.37183	0.21899	0.26729	-1.11821	-0.05235	-0.95514	-1.65317	-1.47438
IFT80	2.36501	1.88509	2.16851	2.1335	1.88999	1.06384	2.10785	1.15242	2.77119	1.88292	2.04756	1.85081	2.75901	2.67029	3.01493	2.69034
SMC4	5.26326	4.98088	5.04868	5.38369	3.91741	3.53793	4.46084	4.17963	5.08159	4.12403	4.3875	4.38134	5.26626	5.06203	4.9898	4.70985
TRIM59	3.30728	3.02523	3.08447	3.13673	2.15796	1.98594	2.79767	2.68663	3.3234	2.54782	2.8304	2.77107	3.82715	3.59071	3.37164	3.12412
KPNA4	3.9218	4.08184	4.01427	4.41225	3.52159	2.6635	4.29807	2.70348	3.34526	3.15019	3.10843	3.89614	4.1448	3.94663	4.05516	3.7742
KRT8P12	0.79106	0.50512	1.28438	0.6191	2.20438	1.96168	2.43529	2.20466	1.4794	0.7098	1.36428	1.02923	2.01813	2.82479	2.91783	2.81885
ARL14	-1.1207	-0.98873	-1.67497	-0.35577	1.97121	0.45785	2.37706	0.67637	-2.06231	-0.68842	-1.52062	0.16796	-3.32193	-1.79944	-3.32193	-3.32193
PPM1L	-2.11439	-1.75651	-1.81258	-1.5068	0.62847	-0.59707	1.21984	-0.49787	-1.94647	-2.44082	-2.25284	-2.36158	-2.52887	-2.05194	-1.81324	-2.02305
B3GALNT1	3.10708	3.12461	2.95002	3.49176	1.1858	1.13821	1.86121	1.37504	2.10618	1.85785	2.08805	2.1232	2.75647	2.70729	2.30337	2.51881
NMD3	4.1623	4.24059	3.91954	4.27181	3.97061	2.68567	4.29217	2.81539	3.74848	3.81004	2.93478	3.82307	4.21487	3.85272	3.36622	3.5547
SPTSSB	-3.32193	-3.32193	-3.32193	-2.98665	1.69697	0.37224	1.91667	1.06806	-3.32193	-3.32193	-3.32193	-3.32193	-3.32193	-3.32193	-3.32193	-3.32193
LINC02067	5.06217	5.11176	4.98679	4.64975	4.53332	4.14322	4.62933	3.69839	5.55648	5.70414	5.52783	3.8636	3.76402	3.64565	3.60706	3.52282
RPL23AP42	7.69624	7.79589	7.67041	7.28647	7.14574	6.82941	7.23832	6.36143	8.20676	8.38209	8.17031	6.66285	6.40575	6.26481	6.24062	6.20139
LINC01322	-3.32193	-3.32193	-3.32193	-2.71145	-1.19525	-2.14199	-0.18608	-2.74052	1.82522	1.88014	1.35834	1.32939	-3.32193	-3.32193	-3.32193	-3.32193
BCHE	-2.9979	-2.67549	-3.32193	-2.13821	-1.28575	-1.84566	-1.47968	-2.00138	1.88765	1.75627	1.36642	2.23494	-2.65122	-2.19793	-2.53844	-2.16571
LINC01326	1.62346	1.2676	2.31367	3.00289	-3.32193	-3.32193	-3.32193	-3.32193	-3.32193	-3.32193	-3.32193	-3.32193	-3.32193	-3.32193	-3.32193	-3.32193
PDCD10	4.51116	4.56965	4.73356	4.77527	3.35121	2.85605	3.91206	2.67485	3.94373	3.90905	3.95809	4.0385	4.10583	3.72829	3.47494	3.22505
SERPINI1	-1.24819	-1.36508	-2.22378	-0.77043	0.89416	1.26554	1.82601	1.13734	-2.51773	-2.15968	-1.90912	-2.17323	-3.32193	-2.73588	-2.63227	-3.32193
GOLIM4	4.17959	4.01817	3.88767	4.38321	4.10924	3.36686	4.38737	3.34647	3.86806	3.45986	3.878	3.42914	3.45763	3.36213	3.61854	3.92991
MECOM	0.05361	0.22872	-0.17157	0.49888	-3.32193	-3.32193	-3.32193	-3.32193	0.57283	0.19427	0.62263	0.64427	-3.05843	-2.37198	-2.52897	-2.44849
RPL22P1	8.81762	8.85442	8.83228	8.67166	7.58862	8.6283	7.30763	8.79767	7.80937	7.86318	7.63196	7.09827	8.44669	8.08276	7.00675	7.04839
TERC	-0.56952	-0.77828	-3.32193	-1.11338	0.18368	-0.90199	0.38428	-0.70863	2.14912	1.373	1.54779	-0.12658	-0.17342	-0.17799	-3.32193	-3.32193
ACTRT3	1.34408	1.80277	1.25881	1.8938	1.96867	1.58314	2.46465	1.14919	0.49039	0.33015	0.76894	0.07601	0.89445	1.43228	1.16578	1.51479
MYNN	1.43329	1.52713	1.77472	1.90385	2.75856	2.45472	3.12478	2.41136	1.20424	1.65825	1.38497	1.65811	1.8359	1.53705	1.31754	1.24701
SEC62	4.55682	4.83419	4.72839	5.27409	5.68012	6.4481	6.51784	6.56042	3.36714	3.79618	3.63008	4.4313	3.12187	3.37979	3.6888	3.38359
GPR160	1.52884	1.62748	1.53858	1.38155	1.36568	1.72405	1.76508	1.55429	2.00928	2.23778	1.65442	2.27894	0.30106	0.28028	0.61304	0.02493
PHC3	1.18256	1.25151	0.8269	1.42061	2.2272	1.45368	2.34966	1.68258	1.0367	1.36825	0.56376	2.12921	1.82345	2.12123	1.49559	2.02845
PRKCI	3.31412	3.47139	3.57939	3.79231	4.23841	2.75204	4.86655	2.78572	3.39154	2.9154	3.14417	3.27213	3.75126	2.79402	3.91601	2.86208
SKIL	2.43717	2.86524	2.83873	3.75581	3.59235	2.80887	4.66535	2.86798	2.80206	3.04702	3.22288	4.45364	2.94823	2.88959	3.39466	3.50101
CLDN11	-2.0497	-2.21402	-2.6351	-2.34038	-2.85463	-2.84656	-3.1583	-3.32193	-2.64545	-2.15796	-2.27486	-2.43142	1.10183	1.50184	1.02924	1.3425
RPL22L1	3.84611	3.64345	3.38095	3.1544	4.27702	4.81603	4.71667	4.8457	4.92556	5.89083	5.4063	4.95881	3.31543	2.88114	1.84972	1.23067
EIF5A2	1.94882	1.71253	1.60538	2.29275	3.6226	3.0834	4.42529	3.19034	2.94431	2.22126	2.18788	2.92569	4.01603	2.95379	3.24692	2.60857
RNU1-70P	-3.32193	-3.32193	-3.32193	-3.32193	4.03387	2.65999	3.91833	3.09478	-3.32193	-3.32193	-3.32193	-0.86609	-0.24896	-1.09101	0.93155	0.03371
SLC2A2	-3.32193	-2.99431	-2.7007	-3.32193	5.45472	3.43012	5.74337	3.47189	-2.28205	-3.0362	-2.05183	-3.32193	-1.44648	-0.92812	-1.09339	-1.73943
TNIK	0.20954	0.72558	0.67769	1.52705	3.21853	2.09761	3.58396	2.10151	3.08278	3.33777	3.00094	3.95425	0.84058	1.76787	2.25098	3.00959
PLD1	0.50772	0.99281	0.99113	1.72253	2.4346	2.54204	2.95435	2.55203	1.46042	1.91775	1.68426	2.88882	0.68985	0.85625	0.76232	1.2071
FNDC3B	3.84037	3.9958	4.21297	4.44135	4.86481	4.12663	5.58285	3.90764	4.05129	3.95862	4.2796	4.97137	3.97748	3.77431	4.50914	4.3266
TNFSF10	-0.70372	-0.51049	-0.39236	0.64799	1.86023	0.98212	1.82452	1.47746	-1.58303	-1.1688	-0.88935	0.04565	0.18007	0.26188	0.32441	0.44875
NCEH1	4.12006	3.966	3.73909	4.36602	6.17779	4.84598	7.49556	4.78327	5.74802	4.84036	5.43107	5.13455	6.56077	6.0959	5.90555	5.38968
ECT2	3.85338	3.55961	3.68273	3.62325	5.6786	3.79841	6.61121	3.74367	4.86562	4.122	4.2747	3.84696	5.96747	5.84196	5.68785	5.02701
TBL1XR1	2.70814	3.13281	2.79635	3.39392	1.98265	1.43136	2.56692	1.3819	2.54353	3.13029	2.67153	3.93662	2.77711	3.61665	2.90845	3.1663

TBL1XR1-AS1	2.03354	1.7917	1.8243	2.03941	1.75572	-1.09814	2.08811	-1.23819	2.84184	2.93128	2.85734	3.59233	2.68465	3.30435	3.02857	2.88046
LINC02015	2.45211	2.97087	2.98791	4.08932	1.37295	0.59468	1.84629	0.50685	-3.32193	-3.32193	-3.32193	-3.32193	-1.82569	-0.93401	-2.3318	-0.31165
KCNMB2-AS1	-0.26126	0.848	0.03475	0.68388	-1.86022	-1.08546	-2.12956	-1.65126	-3.32193	-1.85187	-3.32193	-2.80077	-2.03606	-0.91608	-3.32193	-1.27395
ZMAT3	0.75968	1.10435	0.62235	1.64003	0.72369	0.84166	1.31082	0.96834	2.62957	2.90576	2.67839	4.0877	1.60285	1.86244	1.64464	1.89823
PIK3CA	1.38699	1.77138	1.69869	2.33789	0.20697	0.22317	0.91529	-0.02501	1.67205	2.09863	1.74264	2.88641	1.94627	1.89456	2.34599	2.16028
KCNMB3	-0.27565	-0.05371	0.11514	0.71244	-1.91487	-0.20245	-1.13603	-0.28689	-0.70745	0.13779	-0.32475	0.72134	0.19263	0.287	0.17833	0.24869
LRRFIP1P1	-0.27636	0.19813	0.31764	1.34647	-0.31393	0.28455	-0.69878	0.50104	0.62852	1.04684	0.64865	1.35439	0.39861	0.78117	0.16704	0.8093
ZNF639	2.44982	2.45549	2.30825	2.78049	0.92762	0.81467	1.3083	0.77824	1.99752	2.10463	1.73272	2.35014	2.15465	2.09995	2	1.38826
MFN1	3.22302	3.24837	3.2267	3.68334	2.18549	1.54548	2.87979	1.25911	2.55705	2.88496	2.87201	3.30842	3.60705	3.56399	3.53528	3.31889
GNB4	-1.97453	-1.63089	-1.75607	-1.07087	2.94395	1.78506	3.43291	1.55947	3.93506	3.52411	3.66223	3.99595	-0.52208	0.00616	-0.45011	-0.40216
ACTL6A	4.61551	4.21823	4.23381	4.33262	3.05898	2.43745	2.96129	2.46616	4.49105	3.62995	3.84222	3.13898	4.91152	4.67366	4.18159	4.12028
MRPL47	5.97948	5.79563	5.67737	5.84259	3.62604	4.04279	3.84045	3.95702	4.6697	4.45265	4.33421	4.15129	5.33941	4.97521	4.39451	3.98695
NDUFB5	3.60833	3.98305	4.01374	4.33338	1.87608	1.66029	2.38432	1.58165	2.98012	2.86585	3.06077	3.08047	3.73834	3.41931	3.3696	3.08058
USP13	2.31966	2.00053	2.004	2.5843	1.23717	0.76484	1.74924	0.45286	2.80837	1.77995	2.52093	2.51011	3.15671	2.72642	3.33126	2.63069
TTC14	1.50199	1.72294	1.63811	2.24264	1.35888	0.77668	2.10368	0.56155	0.58738	1.01663	1.05896	1.96357	2.08159	1.47802	1.57763	1.47811
CCDC39	-0.04781	0.34031	0.23318	0.59772	0.17385	-0.83934	0.64957	-1.41151	-0.67217	-0.32145	-0.24838	0.23934	0.62855	0.15793	0.26036	0.27764
FXR1	4.3364	4.44496	4.26709	4.57103	2.23251	2.04603	2.83307	2.10581	4.35027	4.49314	4.34082	4.98556	4.39636	4.42943	4.62762	4.38831
DNAJC19	3.52431	3.2917	3.48576	3.36334	1.90304	1.99032	1.88599	1.948	2.98161	2.60639	2.79459	2.60184	2.90875	2.40702	2.29101	1.82484
ATP11B	2.111	2.64336	2.65518	2.96995	3.2213	2.66786	4.08197	2.39498	2.48125	3.03092	2.75749	3.66145	2.07018	2.82316	2.88446	3.03927
DCUN1D1	2.25076	2.54524	1.86455	2.88126	3.14695	2.28576	3.20752	2.4124	2.33454	2.47117	1.0948	2.85194	2.99092	2.98846	2.05994	2.85238
MCCC1	2.66282	2.64824	2.46631	2.93559	2.90239	3.15507	2.83289	3.19507	2.61675	2.41901	2.54374	2.54554	2.61084	2.66438	2.63343	2.67244
MCCC1-AS1	2.47988	2.95036	2.63921	3.31641	2.20921	3.95294	2.21588	3.86596	1.81551	2.07345	2.03343	1.43125	2.10853	2.77228	2.24775	2.72995
LAMP3	-0.41343	-0.73164	-0.56002	-1.122	0.40771	-0.39965	0.69492	-0.45608	1.44579	0.49982	0.47013	0.55478	0.14252	-0.57438	-0.47116	-0.13012
MCF2L2	-0.96774	-0.81066	-0.85852	-0.14394	-2.13158	-1.69042	-1.26186	-2.01095	-1.51454	-1.31468	-2.10217	-0.41991	0.34222	0.41283	0.02671	0.39121
B3GNT5	1.74883	2.15215	1.70113	2.30061	0.28887	0.11544	1.23371	-0.35765	1.04379	1.47753	0.63409	2.75157	3.07147	3.13644	2.70639	3.46373
LINC00888	1.60884	2.4037	1.86321	2.454	2.40524	3.252	3.00381	3.40959	1.12058	2.45243	1.35186	2.198	2.3076	3.12814	1.97268	3.10935
KLHL24	1.96176	2.63528	2.48978	3.36376	0.85678	0.6252	1.14988	0.76621	0.81414	1.8427	0.7163	2.3959	1.51728	1.4322	1.85632	2.24508
YEATS2	2.5623	2.58978	2.73637	2.49466	2.52079	2.14608	2.70336	1.96223	3.5007	3.32315	3.39994	3.74185	2.76898	2.79013	3.13396	3.18412
YEATS2-AS1	1.4383	1.35056	1.54153	1.6301	1.51658	0.79188	1.82989	0.88732	2.356	2.37914	2.40228	2.87192	2.01464	1.80306	2.01498	2.12448
MAP6D1	0.95395	0.66286	0.40263	0.26133	0.68325	0.67721	0.24255	0.67364	2.02544	1.24419	1.28525	0.46811	-0.26488	-0.31384	0.76568	-0.65585
PARL	4.67389	4.45647	4.34844	4.5097	4.89334	5.24741	4.90528	5.13523	4.60901	4.41178	4.12393	4.10675	4.382	4.71733	4.43284	4.34367
ABCC5	2.16859	1.75448	1.91564	2.15393	3.08423	2.74376	3.29756	2.63972	1.43407	1.11141	1.3795	1.64568	2.23176	2.56168	2.44276	2.87622
EEF1A1P8	-0.00772	0.14297	0.37007	-0.75117	-0.68396	0.08557	-0.81159	-2.58634	-1.02924	0.68685	0.11735	0.07609	-2.41417	-1.86459	-2.70476	-2.6937
EIF2B5	3.30548	2.95778	2.66463	2.98831	3.82187	3.97579	3.74444	4.13226	3.14296	3.03071	3.27835	2.91805	3.69007	3.7284	3.2294	3.27058
DVL3	4.55136	4.60219	4.76961	4.41089	5.25933	5.14276	4.67028	5.23735	4.40458	4.4786	4.64458	4.20284	4.31387	4.36416	4.77118	4.79811
ABCF3	4.41247	4.50368	4.42405	4.58478	4.55479	4.6419	4.39407	4.62893	3.54514	3.45876	3.86628	3.51684	3.71114	3.67876	3.7721	3.90498
VWA5B2	-3.13867	-2.63656	-2.60658	-3.32193	0.07714	1.17874	-0.50046	1.26531	-2.61727	-2.20057	-2.67921	-3.32193	-2.52052	-2.43761	-1.89907	-1.81071
ALG3	5.38994	5.03749	4.93292	4.78028	6.26528	6.89848	5.70474	6.89298	4.36793	4.03669	3.99573	4.07722	3.90375	3.26263	3.41224	3.68827
ECE2	2.20946	1.47804	1.26395	1.53424	3.25823	3.41136	2.55381	3.14558	1.47763	0.8857	1.13644	0.51105	1.57827	0.53613	1.1979	0.74167
CAMK2N2	0.98585	2.29188	1.98115	2.47695	3.58872	4.50367	2.53393	4.53258	2.51854	2.15089	2.0406	0.1717	0.98185	2.06781	2.55262	2.44319
FAM131A	3.06222	3.15603	3.23944	2.40695	2.38271	2.45903	1.59172	2.62802	1.19112	0.86981	1.22843	0.49648	1.73223	1.82169	1.3641	1.4671
CLCN2	0.70473	0.78669	1.07493	0.59833	2.42007	2.21383	1.79329	2.43777	-0.93405	-0.33333	-0.43153	-0.77738	0.86339	0.96217	0.62595	0.92224
POLR2H	5.46711	5.27837	5.31753	5.052	5.26757	5.85471	4.8694	5.8313	4.33229	4.48329	4.34993	3.82092	4.42205	4.50399	3.53827	3.58411
THPO	-3.32193	-3.32193	-3.32193	-2.72582	3.26065	3.71937	2.3052	3.68643	-3.32193	-3.32193	-3.32193	-3.32193	-3.32193	-3.32193	-3.32193	-2.1933
CHRD	-3.16199	-3.14358	-3.32193	-3.32193	2.88061	4.32295	1.53682	3.92486	-3.32193	-2.67552	-3.00821	-3.03235	-2.8705	-2.77833	-3.16886	-1.60464
EPHB3	-1.97637	-2.09203	-2.83056	-3.03956	1.07048	0.4294	0.41239	0.46806	-2.32533	-2.00795	-2.54824	-2.22672	-2.979	-3.32193	-2.91375	-2.90591
MAGEF1	3.96905	4.63883	4.19455	4.77343	4.87256	5.63088	4.94221	5.59021	3.55275	3.72066	3.88217	3.54326	3.98379	4.96385	4.31421	4.41613
VPS8	1.01201	1.37623	1.23453	2.0013	1.84712	1.64526	2.13918	1.76718	0.78862	1.26058	0.49652	2.26905	1.62007	1.69017	1.50842	1.7479
EHHADH-AS1	-0.92137	-0.33296	-1.19084	-1.32129	-0.50242	0.08632	0.10649	-1.18644	-0.49807	-0.84015	-0.75474	0.34729	0.48954	0.18838	-0.27496	-0.09641
EHHADH	0.66557	0.89591	0.05527	0.87203	1.93509	1.24529	1.91401	1.429	1.55618	0.7992	1.33865	1.44887	2.12308	1.54749	2.31374	1.85961

MAP3K13	1.3968	1.87765	1.66958	2.1426	1.63971	2.37438	2.03571	2.17539	0.84873	1.03606	0.41756	1.23973	1.64386	1.90789	1.10741	1.39288
RPL4P4	5.29531	5.47697	5.21702	4.9437	3.7047	4.3882	3.24277	3.81225	4.7403	5.41644	4.73944	4.39337	2.95819	2.35098	2.82435	2.52675
TMEM41A	2.27666	2.41031	2.12226	2.47789	4.08903	4.05107	4.10366	4.17947	1.28789	1.04262	0.89714	1.58231	2.76682	3.37015	2.12059	2.86615
LIPH	0.2305	0.07177	0.02088	1.13206	4.45909	4.148	5.24298	3.57858	-0.95504	-0.21328	-1.6686	0.84455	1.61586	2.8271	1.92722	3.4709
SENP2	2.86777	2.82269	2.70615	3.00727	4.24002	3.616	4.28709	3.58627	2.75903	2.18168	1.97245	2.46706	3.23199	2.89282	3.1141	2.87931
IGF2BP2	4.80386	4.39734	4.8383	4.44255	4.9142	4.55564	4.95734	4.59325	5.62666	4.20931	5.19004	4.78841	5.58297	3.28697	5.75898	3.72774
IGF2BP2-AS1	-0.85064	-1.84394	-1.33579	-0.39616	-3.18899	-0.51986	-3.32193	-0.05139	-3.32193	-3.03605	-2.76526	-2.60266	-2.15435	-2.54822	-3.03862	-2.5861
TRA2B	4.41691	4.29143	4.16672	4.27221	4.07767	3.24915	4.24777	3.18286	4.34	3.67512	4.12252	3.1683	4.26111	4.28523	3.9907	3.80343
NMRAL2P	1.3174	0.21503	0.9667	1.57082	-2.31947	-2.39913	-1.77118	-3.32193	-3.32193	-2.9604	-3.32193	-3.32193	-0.17356	-0.8337	-1.89336	-1.87345
ETV5	3.26065	3.25685	3.37205	3.42248	2.59786	2.26504	2.77719	1.68637	2.91308	2.36277	2.53836	3.2892	3.85267	3.64931	4.23443	4.22712
DGKG	1.53235	1.69703	1.6814	1.90012	-0.18333	-1.17427	-0.23055	-1.76904	0.35063	0.30063	0.17036	0.57273	0.28547	0.09195	1.055	1.19348
CRYGS	0.55691	0.71276	0.59517	1.00941	0.51924	1.48523	0.72185	1.6141	0.60972	0.10329	0.33197	0.68261	1.47789	1.35079	0.59686	0.48832
TBCCD1	1.26433	1.5431	1.39965	1.70717	2.24101	1.83062	2.35649	1.98176	2.10855	1.87213	1.65353	2.10165	2.54402	2.50771	2.05988	1.91463
DNAJB11	4.81211	4.75755	4.49273	4.89207	5.37743	5.7363	5.62497	5.65869	4.2531	3.79057	4.20885	3.79446	4.09742	4.23925	3.74048	3.87936
AHSG	-2.91067	-2.86752	-3.32193	-3.32193	1.20123	6.03486	1.37512	5.65072	-2.5008	-3.32193	-3.32193	-2.62115	-3.32193	-3.32193	-3.32193	-2.91933
KNG1	-3.32193	-3.32193	-3.32193	-3.32193	1.77512	3.38749	1.35619	4.10061	-3.01018	-3.32193	-3.32193	-3.32193	-3.32193	-3.32193	-3.32193	-3.05588
EIF4A2	5.72647	5.47761	5.58048	5.83825	5.27005	5.37653	5.76152	5.60636	4.90639	5.44799	5.02035	5.38587	5.03592	4.45498	3.649	3.52027
SNORD2	5.08085	4.15177	4.12298	3.10765	5.33012	2.79152	5.90562	3.02276	4.20195	3.82787	4.47505	4.68323	3.73532	3.73021	2.9293	3.15077
SNORA63B	3.98867	3.32412	3.05711	3.85182	3.72588	2.69595	4.70131	3.06274	2.17013	2.47928	2.41396	2.28848	3.69519	2.70121	0.38392	2.32944
MIR1248	3.95196	2.33081	4.38671	3.59886	4.25314	2.90746	4.6267	3.39924	1.40773	1.54804	2.6248	2.49899	3.12172	2.53996	-3.32193	2.54006
SNORA81	3.94119	2.17138	3.81222	3.06291	3.74771	2.74691	4.4909	2.87981	1.089	1.60558	2.6834	2.08184	2.64355	1.8089	0.43176	2.38017
SNORA63	5.5407	4.57639	4.90944	4.7453	4.6042	4.3859	5.44155	4.29877	2.92937	3.28372	3.81857	3.41825	3.35528	3.14092	2.01062	1.2601
SNORA4	4.17768	4.38758	4.2293	4.64093	4.44863	4.68305	5.39568	4.38107	2.73057	2.7242	2.70239	3.42803	3.00556	3.57942	2.13608	1.97886
RFC4	4.31173	4.06903	4.01856	3.91388	3.98037	3.54834	4.25001	3.74815	3.30053	2.58937	2.94338	2.02753	3.77537	3.95273	3.20143	3.01088
RPS20P14	3.08882	2.87958	3.33072	1.00369	1.14138	3.06748	0.38674	2.15679	2.61216	3.47486	2.26005	2.85862	1.30134	-0.08699	1.24134	1.87836
ST6GAL1	-2.03397	-1.44794	-1.93917	-1.01115	-1.78137	-2.42855	-1.50088	-2.55119	1.74979	1.31505	2.08645	1.59388	-0.29053	-0.3427	0.40351	0.77032
RPL39L	2.72607	2.75632	2.88489	2.47339	3.95352	3.58722	3.70236	3.15313	5.4232	4.95535	4.37373	3.3553	4.03481	3.72127	3.43237	3.4914
RTP4	-3.32193	-2.23709	-1.33699	-0.33363	-3.32193	-3.32193	-3.32193	-3.32193	-0.55529	-0.04858	0.85388	0.7738	-3.32193	-2.49025	-3.32193	-3.32193
BCL6	1.79584	1.75964	1.98394	1.9738	2.09572	3.22097	1.77851	3.36867	2.92697	2.68699	2.81126	2.60812	1.58392	0.82646	1.23034	1.92074
LPP-AS2	-0.63427	-1.00846	-0.36393	-0.28378	0.32856	0.35966	0.42728	0.44609	1.15011	0.52439	0.86367	1.32147	0.27336	0.7416	0.90054	1.0647
LPP	1.37426	1.64588	1.6354	2.21148	1.27405	1.21718	1.68107	1.44604	2.49107	1.96182	2.3117	3.04262	3.64482	2.37953	4.64594	3.5565
TP63	-3.32193	-3.32193	-3.0038	-3.14376	-3.32193	-3.32193	-3.32193	-3.32193	-3.32193	-3.18389	-3.32193	-2.84184	-2.11707	-0.98164	-1.79146	-0.67033
P3H2	3.29987	3.71415	3.18945	3.07393	1.74254	2.00219	2.03803	2.22098	3.08774	3.01998	3.0515	2.69006	4.8035	4.44804	4.52342	4.07748
CLDN1	3.68666	4.46274	4.06978	4.25077	7.77195	5.9217	8.40309	6.02334	2.73359	4.08373	3.36856	4.39712	5.76015	6.03706	6.85564	5.71524
CLDN16	-3.32193	-2.75025	-2.72428	-2.07871	0.04147	-1.4627	0.15182	-1.70787	-2.3168	-2.44574	-2.57933	-2.30277	0.32649	0.97089	-0.17482	-0.0808
IL1RAP	2.01017	2.13869	1.80938	2.40963	1.37755	0.78464	1.01742	1.37402	2.29973	2.67525	0.62289	3.3829	2.8325	3.44952	1.46835	2.70963
UTS2B	1.87505	2.11476	2.13714	1.47099	3.80777	2.61662	3.47109	1.87681	3.04515	2.89309	2.86884	2.62248	2.93827	2.96261	3.73996	3.36597
CCDC50	3.63684	3.70707	3.79901	3.91465	4.64916	4.02778	5.09582	3.79428	3.74665	3.82397	3.57445	4.55331	4.3106	4.45675	4.70073	4.27806
FGF12	-3.32193	-3.32193	-2.99996	-2.70571	2.2238	0.58271	2.33194	0.51642	0.8617	1.36895	1.09306	2.03963	0.22211	1.26199	1.38885	0.78829
MB21D2	2.70692	2.80323	2.79753	2.24044	2.91231	1.89195	3.42015	1.435	3.85636	3.44325	3.78187	3.02488	2.8251	2.87058	3.3315	2.17954
OPA1	3.15251	3.06521	2.83633	3.36053	4.07136	3.27036	4.51364	3.34228	4.13944	3.84251	3.64396	4.51952	4.21925	4.38925	4.0936	4.23072
OPA1-AS1	2.63001	2.68031	2.18275	2.54028	4.5715	1.77218	5.21997	1.33034	4.4153	3.96443	3.66883	4.89995	4.49894	4.71752	4.60011	4.74247
HES1	0.71652	0.61954	0.33273	1.77657	4.49976	5.41436	4.37809	5.74412	2.10434	1.31933	1.33199	2.81925	1.56574	2.32391	2.41864	2.97946
ATP13A3	3.94866	4.33079	3.99752	4.71811	2.87967	1.40047	3.58953	1.55201	3.57765	3.88489	3.19972	4.71232	4.44859	4.44583	3.60118	4.09002
LINC00884	1.13642	1.29583	1.40539	1.56078	-0.56637	-1.66729	-0.20324	-2.36011	-1.08778	-0.70779	-1.52433	-1.22654	-2.29305	-1.80283	-1.98761	-0.75515
TMEM44-AS1	2.42526	2.74784	2.7025	2.61859	1.63441	1.87831	0.54556	2.31245	1.28567	0.98017	0.40214	0.90711	1.94459	2.26619	0.8653	1.98478
TMEM44	2.88754	3.12962	3.447	3.24275	0.73834	1.05559	0.35096	1.41707	2.96239	3.3769	3.27644	3.27051	2.40213	1.95329	2.40569	2.64633
LSG1	4.44403	4.28276	4.09144	4.45151	2.77664	2.11592	3.00167	2.01683	3.96208	3.49026	4.02121	3.99699	3.73479	3.61442	3.49584	3.57928
FAM43A	3.46651	3.95453	4.06415	2.78583	0.61107	-1.64274	0.08215	-1.63449	3.83221	3.66033	4.44599	3.29365	-1.76102	-1.76442	-1.04024	-2.17479
XXYLT1	2.85139	2.84846	2.92859	2.8733	3.57559	2.74124	3.10292	3.12973	2.26402	2.30531	2.76002	2.22775	1.99619	2.13054	2.49781	2.31407

ACAP2	1.88024	1.95795	1.85788	2.39111	1.35061	0.10054	2.13506	-0.19972	2.7232	2.34635	2.66529	3.34104	3.38762	2.56292	3.03373	2.55068
PPP1R2	3.71549	3.81795	3.74135	3.89296	5.03131	4.64337	5.57678	4.46052	3.59934	3.50177	3.21346	3.85906	3.35807	3.34124	3.54942	3.1476
MUC20-OT1	2.12728	2.29318	2.38698	2.29901	1.17721	1.32127	0.871	1.54954	0.40612	0.66758	0.76467	0.94005	1.19996	0.93679	0.6425	0.83407
SDHAP2	4.73709	4.8321	5.04846	4.58758	3.85278	3.56384	3.43519	3.66612	2.55354	2.84176	2.98826	2.87649	3.49986	3.13086	2.83512	2.85118
MIR570	2.51119	1.8845	1.9604	3.20018	2.85522	3.03384	3.75955	4.32806	3.81255	3.72427	3.74007	3.20197	3.8597	3.14478	2.80225	2.83336
TNK2	3.43344	3.56538	3.89433	2.71156	3.01838	3.64792	1.81756	3.94386	2.6157	3.10372	2.92373	2.18841	1.72615	2.04228	2.36831	2.71951
TNK2-AS1	2.03531	1.64799	2.19365	0.8715	2.81783	2.47665	1.37218	2.8194	1.73467	1.80411	2.24534	0.97119	0.94679	1.63516	2.25297	2.06462
SDHAP1	3.71754	3.67564	3.85221	3.85274	3.6771	3.57798	3.19766	3.52331	1.52167	2.11056	2.37393	2.064	2.68047	2.38729	2.12606	2.32241
SLC51A	0.97249	0.94624	1.03255	1.40843	1.99267	1.07138	2.35705	0.7986	1.49928	1.41868	1.29541	2.2833	1.75444	2.00119	1.64981	1.90645
PCYT1A	3.4266	3.44756	3.35971	3.68619	4.3068	3.64989	4.48382	3.61055	3.95535	3.95015	4.00117	4.48896	3.89066	3.98576	3.73619	4.08813
TCTEX1D2	2.07419	2.84021	2.20211	2.42451	3.05326	2.93686	2.81127	3.28264	2.87582	3.26157	2.74024	2.83328	3.5858	3.42612	2.91338	2.44603
TM4SF19-TCTEX	7.64797	7.86792	7.59361	8.12197	3.40096	3.99023	3.50751	3.7035	3.86841	4.20943	3.53726	4.31789	4.33325	4.00008	3.5206	3.61799
TM4SF19-AS1	6.48825	6.76156	6.47631	6.96794	-0.12362	2.13694	0.54268	1.17688	1.30167	1.8773	0.87301	2.45247	1.34941	0.70637	0.67058	1.51891
TM4SF19	7.11785	7.35596	7.15582	7.60445	0.59069	2.77306	1.23317	1.88644	2.24961	2.71444	1.65461	3.10183	2.2261	1.47508	1.16744	2.15293
UBXN7	2.32223	2.76321	2.86768	2.9931	2.31478	1.6966	2.71537	1.88009	2.80637	2.9404	2.75354	3.52809	3.30285	3.48708	3.72525	3.66035
UBXN7-AS1	2.56827	2.99978	2.9039	1.96622	3.55012	2.12736	3.59835	0.82706	3.74649	3.90972	3.30594	3.42951	3.2793	3.84634	4.03715	4.06852
RNF168	3.08325	3.14812	3.49978	3.44043	3.1702	2.84567	3.42256	2.92462	2.64963	1.97127	2.36101	2.4101	2.47465	2.15783	3.08007	2.58317
WDR53	3.54534	3.59958	3.48587	3.8077	3.09695	3.06986	3.13221	2.86629	2.84368	2.09247	2.12117	1.92027	2.68422	2.35716	1.71155	2.4245
FBXO45	3.59542	3.88121	3.35609	3.71705	2.91471	2.16113	2.95185	2.34751	3.08889	2.71693	1.92083	2.52385	2.99182	3.22438	2.4932	2.89248
NRROS	0.59794	1.73683	0.9268	1.55002	-3.32193	-3.32193	-3.32193	-2.61312	-0.67342	-0.07596	-0.67817	-0.35354	1.71193	2.14071	0.83238	2.97381
PIGX	2.54677	2.58439	2.65635	3.25277	3.56965	3.46505	3.87134	3.30442	2.64342	2.52928	2.40821	2.59874	3.39334	3.37503	3.1964	3.45753
CEP19	2.0177	2.02071	2.0968	2.88762	1.89693	1.95575	2.69723	1.67972	0.72902	1.66006	1.34906	2.20565	1.93713	1.88988	1.80749	1.78152
PAK2	4.14887	4.26955	4.18559	4.46814	4.38705	3.7105	5.16574	3.66875	4.84892	4.63553	4.61485	5.55541	5.19115	4.9412	5.48607	4.993
SENP5	2.48105	2.50076	2.49948	2.53857	2.79315	2.63779	3.43703	2.61732	2.48245	2.10506	2.50518	3.09248	2.65665	2.14232	3.05036	2.71124
NCBP2	4.48896	4.66844	4.43081	4.77616	4.32266	4.78966	4.75574	4.68854	4.13455	4.08398	3.91183	4.16164	4.27952	4.54646	3.57925	3.89247
NCBP2-AS1	4.14067	4.55493	4.55436	4.39372	4.83522	4.53224	5.16219	4.04664	4.5169	4.50708	4.36162	4.02979	4.52585	4.69896	4.0855	4.57404
PIGZ	-2.17884	-2.0853	-1.26885	-2.38429	0.11833	1.55328	-0.36428	1.22799	1.10496	1.36212	1.3096	1.45158	1.40945	0.78467	1.30855	1.29791
MELTF	1.75426	2.11593	2.72411	1.79648	2.55318	3.31535	1.9691	3.21816	5.15304	5.264	5.45008	4.27955	1.23008	0.71792	2.25679	1.84853
MELTF-AS1	2.28388	2.40619	3.29091	1.17026	3.39879	4.70832	1.82158	4.77678	5.73029	5.90592	6.07709	3.87128	1.72377	1.37855	2.55829	2.5241
DLG1	2.78534	2.77205	2.94598	3.42771	2.84704	2.41953	3.28311	2.6008	5.17523	5.18079	5.31575	5.99164	3.59619	3.55774	3.28973	3.52197
DLG1-AS1	-0.04319	0.35924	0.8773	0.39462	0.6045	-0.51004	0.9231	-1.05452	3.41922	3.30214	3.88882	3.62114	1.26134	1.16732	1.19354	1.8608
BDH1	2.67844	2.4819	2.51077	2.37131	-1.5466	-1.22709	-2.04049	-0.72158	-2.48047	-2.66674	-2.98319	-2.91821	-3.05872	-2.18667	-2.8298	-2.38525
RUBCN	2.07658	1.9608	2.05545	2.05695	2.5136	2.66054	2.7946	2.57854	2.82288	2.50993	2.8525	2.85798	3.12544	2.83336	3.44929	3.06301
MIR922	4.58823	4.11331	4.43751	5.08614	4.56697	4.91324	5.23514	5.19256	5.32893	4.89078	5.03809	4.86627	5.44752	5.17006	5.91834	5.33296
FYTTD1	2.85933	2.98866	2.68877	3.15622	3.691	2.88839	4.15917	2.85668	3.79982	4.02591	3.50731	4.59769	3.49418	3.69063	3.17898	3.37543
LRCH3	1.91773	2.12415	2.30814	2.33286	2.18809	2.50156	2.67536	2.54074	2.6162	2.46108	2.67613	3.15693	2.39692	2.30916	2.49279	2.32591
IQCG	3.27261	3.23995	2.95688	3.18889	-0.48798	-0.45412	-0.97963	-0.27105	0.89705	1.14591	1.0857	0.93459	0.02757	-0.22788	-0.10035	-0.01194
LMLN	0.9288	1.28994	1.21506	1.7667	0.79449	0.53124	1.55279	0.31847	1.34428	1.38162	1.58268	2.76641	2.29037	2.27912	2.33473	2.46918
LMLN-AS1	2.08837	1.72878	2.11809	2.64298	2.31187	1.4362	2.84484	-0.3561	2.79165	2.83638	2.72836	4.26608	3.21708	3.21199	3.27354	3.92523
FAM157A	2.6642	2.51765	2.68725	2.41968	2.05915	3.11526	0.97794	3.68312	-1.49637	-0.4476	-0.82399	-0.57631	-0.68256	-0.57248	-1.10238	-0.13328
ZNF595	2.78002	2.50065	2.47784	2.54957	2.75584	2.63351	2.82775	2.48948	0.88225	0.9462	0.45148	0.99236	1.38954	1.22261	1.639	1.46336
ZNF718	1.05585	0.85876	0.63353	0.80048	-0.48128	-1.88097	-0.00401	-1.83993	0.05936	-0.01372	-0.54429	0.52609	1.62414	1.96195	1.83709	1.69824
ZNF876P	-1.9101	-1.96953	-2.11299	-2.55738	-0.25052	0.68815	-0.07004	0.24661	-2.9195	-2.98062	-3.32193	-2.48424	-1.38874	-2.12253	-1.6831	-1.5433
ZNF732	0.53092	-0.89714	-0.1574	-1.12841	-1.37489	-1.66797	-1.21641	-2.1438	-2.45582	-3.32193	-2.87131	-3.32193	1.777	1.76029	1.72683	1.71254
ZNF141	0.48248	0.15848	0.20978	0.31365	0.65517	-0.3033	0.79878	-0.14631	1.11978	0.73425	0.57118	1.09536	1.21597	0.97597	1.08771	1.23558
ABCA11P	1.47016	1.0692	1.22715	1.59204	1.40059	1.62356	1.96977	1.81063	1.70875	1.97671	1.87711	1.87189	2.75424	2.96515	2.45721	2.23723
ZNF721	1.48982	1.28972	1.37261	1.77058	1.98495	1.96149	2.7017	2.22636	1.9202	1.84878	1.69848	2.3267	2.70551	2.65339	2.53161	2.32053
PIGG	1.98847	1.98619	2.08536	2.20052	3.11066	2.98585	3.27033	3.04308	2.36947	1.9907	2.24992	2.3725	2.63094	2.66562	2.68374	2.8137
PDE6B	-2.93766	-3.32193	-3.32193	-2.49772	-1.76401	-0.99668	-3.12981	-1.1109	-0.54511	-0.09117	-0.24641	-0.20637	-3.32193	-2.75975	-3.12571	-3.32193
ATP5ME	6.69885	6.35469	6.27382	5.20122	6.9939	8.2604	5.71658	8.28358	6.26987	6.46071	6.11471	3.85038	6.57464	6.35906	4.7748	5.33868

MYL5	4.52105	4.24328	4.15777	3.28538	4.85933	6.10627	3.58671	6.15348	4.07948	4.36919	4.02777	2.15645	4.3789	4.19577	2.68577	3.25513
SLC49A3	0.41454	0.363	-0.46201	0.72803	0.84437	1.75048	-0.29972	1.47943	-0.55631	1.09628	0.72321	0.40535	-1.50241	-0.96447	-1.29236	-0.39812
PCGF3	2.86829	2.88703	2.95938	3.09511	3.50228	3.55416	3.45541	3.37426	3.0512	2.76616	2.71708	3.18862	3.57789	3.55587	3.77426	4.17171
CPLX1	-2.60651	-2.87804	-2.85698	-3.32193	3.93157	5.17743	2.99093	5.32313	-0.44565	0.35181	0.94771	-0.26833	-1.06736	-1.41264	-1.29884	-0.40015
GAK	3.7747	3.89458	3.81546	3.63035	3.70357	4.16744	3.2621	4.05109	3.15349	3.22032	3.31881	3.12791	2.59692	2.82867	2.93811	3.25784
TMEM175	2.06012	2.2883	2.31406	2.19823	2.52809	3.78767	1.45391	3.89513	2.09028	2.29056	2.31679	1.9245	1.5622	1.40156	1.32965	1.75791
DGKQ	2.72848	3.00089	2.95937	2.22453	4.11414	4.28692	3.2438	4.20563	2.10199	2.83133	2.72671	1.93757	1.98322	2.35685	2.94837	3.07914
SLC26A1	-0.24943	0.81878	0.90898	0.2576	5.70502	4.9315	4.98369	5.05934	-0.4532	0.66159	0.99391	0.84536	-0.68028	-0.28673	0.61602	0.95219
IDUA	1.12176	1.58327	2.13705	1.45701	4.4747	4.83422	3.847	4.86341	1.88204	2.37551	2.14456	2.35658	-0.43746	0.47246	0.61016	1.08815
FGFRL1	4.82553	4.86307	4.65853	4.61713	5.22267	4.98022	4.60538	4.78217	3.15968	3.26263	2.85175	3.17165	3.16352	3.32551	4.16952	4.40754
RNF212	-3.32193	-3.09035	-2.87031	-3.32193	-3.32193	-3.32193	-3.12683	-3.32193	2.7937	2.95867	2.99541	3.10438	-3.32193	-3.32193	-3.12267	-3.32193
SPON2	-2.99063	-2.80126	-3.11664	-2.74775	-1.59843	0.9754	-1.71397	0.78854	-1.99983	-1.86643	-1.79989	-1.88201	3.36923	3.82539	5.10932	5.38386
CTBP1-AS	4.4135	4.52981	4.48015	3.81951	5.12878	5.54804	4.34272	5.55509	4.94113	4.98748	5.3908	4.20084	4.09792	4.36582	4.83639	4.64209
CTBP1	4.11276	4.20455	4.16404	3.60247	5.03172	5.30241	4.35977	5.31079	4.76802	4.77946	5.2172	4.18318	4.06889	4.38972	4.71833	4.62979
CTBP1-DT	0.48559	0.30597	0.78461	0.02095	2.33466	1.4108	1.72079	1.11491	2.04479	1.46498	2.21869	1.22606	1.04222	0.47909	1.77252	0.70511
MAEA	2.80195	2.54706	2.7249	2.33346	3.35036	2.85142	3.10372	2.82124	3.32363	2.76967	3.2412	2.739	3.26482	2.5124	3.03499	2.73926
UVSSA	0.0495	0.48892	0.55575	0.23981	1.96319	1.92843	1.81256	2.22188	0.17569	0.33085	0.09559	0.68827	0.26141	0.43533	0.64685	0.63686
FAM53A	0.67929	0.32286	0.44158	0.09864	0.64328	1.27141	-0.38359	1.68442	-1.58166	-0.70838	-0.46214	-1.96459	-0.3693	0.10018	0.56654	-0.65287
SLBP	5.29325	5.09579	5.04852	5.07097	5.51527	4.84024	5.59615	4.98946	5.44637	4.69195	4.94188	4.40843	5.96634	6.03082	5.08455	4.79707
TMEM129	3.40396	3.60552	3.77863	3.16232	4.5926	4.68639	3.65803	4.47592	3.21716	3.80675	4.01324	2.93868	1.83997	2.54555	2.83151	2.92248
TACC3	4.86224	4.18335	4.2788	3.41498	4.72203	4.29526	4.2845	4.66907	4.99541	3.7661	4.37364	3.19207	4.98783	4.72817	4.88373	4.6531
FGFR3	-1.24773	-1.36464	-0.89569	-1.46001	5.95073	6.05339	4.93679	6.24228	-1.13739	-1.58973	-1.32415	-1.74726	-1.02936	-1.36487	-0.23257	-0.59064
LETM1	3.93921	3.60532	3.29053	3.05324	5.09295	4.314	4.76864	4.51916	3.93554	3.44626	3.72091	3.20415	4.00595	3.94853	4.56046	4.46161
NSD2	3.37005	2.92463	3.15481	2.48014	3.51	2.84457	3.56706	3.12768	3.91812	3.27815	3.81282	3.10704	3.61662	3.71207	4.19635	3.84729
NELFA	3.79255	3.29523	3.46438	2.68319	3.60179	3.92719	2.85129	3.9526	3.91962	3.63899	4.04605	2.37372	2.97744	2.62382	3.13172	2.80816
MIR943	6.45319	6.02172	5.99281	5.31136	6.68922	6.6951	5.73549	6.61919	6.86078	6.53071	6.8311	4.55959	5.7835	5.17769	5.65967	5.54944
C4orf48	5.77768	5.84623	6.06281	5.59041	5.49855	7.68965	5.07785	7.53723	6.17199	6.19258	5.76278	4.69218	6.24134	6.33375	5.51712	5.76871
NAT8L	1.85982	1.97928	2.02787	1.30437	2.21302	3.37551	1.9407	3.63178	1.42302	2.73384	2.35008	1.6532	2.26671	3.32441	4.06044	3.94365
POLN	0.04942	0.08272	0.27206	0.09007	0.73337	-0.30145	1.04338	0.18926	0.30259	0.25741	0.36414	-0.94539	0.3664	0.52004	0.19673	0.01761
HAUS3	1.57935	1.5166	1.56678	1.59709	1.99807	1.38377	2.50506	1.71688	1.26935	1.18332	1.32224	0.95812	1.93564	1.98787	1.82769	1.63266
MXD4	1.73202	2.47387	2.79754	2.49894	3.27618	3.727	2.64822	3.43976	2.86712	4.00813	4.14659	4.04874	1.78708	1.57738	3.06936	3.56162
MIR4800	1.99299	1.18764	2.23187	3.62611	4.1552	3.78999	4.4485	3.67728	4.35035	4.95289	5.05595	6.42008	3.31912	4.06707	4.5959	5.1346
ZFYVE28	-1.51458	-0.42922	-0.43416	-0.55578	-0.12253	0.51506	-0.24257	0.41938	-1.94701	0.39302	-1.44483	0.73746	-1.26571	-1.67096	-1.85158	-0.78573
RNF4	3.60196	3.38912	3.33147	3.18842	4.2955	3.55417	4.3133	3.2589	4.14027	3.54104	3.9392	3.5344	4.42306	4.13005	4.56656	4.09874
FAM193A	2.31434	2.28372	2.38952	2.3815	2.69605	2.73123	2.63723	3.1115	2.79592	2.7495	2.8991	3.19048	2.65015	2.35468	3.08589	3.01976
TNIP2	5.45251	5.40349	5.32124	4.70441	5.2052	5.43562	4.96091	5.18148	5.23585	5.12981	5.10575	4.49581	4.66079	4.46402	4.67289	4.60585
SH3BP2	2.26992	2.15885	2.28272	1.86762	-1.41081	-0.01795	-2.02782	-0.49815	1.28951	1.90644	1.70491	1.90297	-1.96179	-1.73924	-1.49557	-1.21504
ADD1	3.09801	3.14304	3.19161	3.04937	4.51085	3.8478	4.44784	3.85488	4.53437	4.27511	4.45061	4.6434	4.35095	4.25269	4.75526	4.56724
MFSD10	4.77329	4.84694	5.18803	4.06119	6.26955	6.79974	5.33688	6.6831	5.73226	5.67078	5.79858	4.77839	4.50074	4.54386	4.69371	4.76386
NOP14-AS1	2.99539	2.8076	2.93597	2.51703	3.40727	3.05718	3.54837	3.38065	3.11079	2.78064	2.90642	2.74104	3.22422	2.9706	3.14969	2.94687
NOP14	4.69108	4.35836	4.41501	3.91209	5.1119	4.55864	5.24334	4.84412	4.97908	4.43101	4.7258	4.54019	5.05012	4.6229	5.00462	4.81802
GRK4	0.59246	0.57941	0.16394	0.3219	0.64442	0.71425	0.2225	0.87466	0.03432	0.20677	-0.11471	-0.48935	0.32749	0.18186	0.23302	0.42711
HTT	2.70058	2.70594	2.70294	2.67363	3.78726	2.96443	3.71811	3.00881	2.85424	2.83156	2.85965	3.49123	3.17665	3.38737	4.33911	4.30829
MSANTD1	-2.23085	-2.37881	-2.5308	-1.6415	-2.79946	-2.44917	-2.95707	-1.53768	-3.01289	-2.94646	-2.81016	-2.74946	-3.21023	-3.10727	-3.32193	-2.05657
RGS12	0.033	-0.02668	0.21374	0.18279	1.04285	0.86075	0.77934	1.35339	0.88452	0.25671	0.52847	0.41562	1.48526	1.10434	1.93865	2.17464
LRPAP1	4.41339	4.14602	4.23732	4.16376	5.72299	6.31039	5.22603	6.1488	4.54383	4.15364	4.55739	3.80897	3.50716	2.71799	3.39533	3.3064
LINC02600	-3.32193	-3.32193	-3.32193	-3.32193	0.50435	-0.68907	0.53093	-0.03324	-3.05966	-2.58937	-3.08598	-3.32193	-3.32193	-3.32193	-3.32193	-3.09866
ADRA2C	-3.32193	-3.32193	-3.32193	-3.32193	5.81655	4.09879	4.78115	4.04026	2.37638	1.36342	2.6719	-0.30564	-3.32193	-3.32193	-0.88341	-3.32193
FAM86EP	0.0535	0.38176	-0.29651	-0.21584	1.82467	1.72495	1.2835	1.60779	0.63541	0.09611	0.59546	0.18545	0.83128	0.51064	0.61191	0.73882
TMEM128	2.44938	2.09171	2.417	2.79185	2.9431	2.92586	2.66816	2.93481	2.40425	2.0974	2.18383	1.55483	3.03318	2.42409	1.74078	1.29547

LYAR	5.72462	5.3298	5.0044	5.19559	4.76057	5.02013	4.91872	5.41729	4.20949	3.39564	3.86714	2.95351	4.31249	3.97245	3.0881	3.16729
ZBTB49	0.88457	0.76393	0.03279	0.94961	0.54373	1.2354	1.22146	1.46054	-0.52845	-0.37044	-0.14071	0.72554	0.42411	0.6681	0.38461	0.29549
STX18	2.60121	2.94735	2.6493	2.74742	2.87681	3.07046	2.88149	2.69788	2.23846	2.25945	2.45502	2.34131	2.559	2.60333	2.60966	2.32619
STX18-AS1	-2.22177	-1.43042	-1.61774	-1.30463	-2.22873	-1.43503	-2.27393	-1.90901	-2.524	-2.63241	-2.98472	-2.6417	-1.84056	-1.52275	-0.77264	-1.42336
MSX1	5.1258	5.27826	5.14056	4.66036	-0.70583	-0.12922	-2.07892	-0.78841	2.49363	2.72921	2.58888	1.31886	0.35153	0.17483	-0.52658	0.31409
STK32B	-3.32193	-2.91349	-2.71964	-2.38071	-3.23526	-2.79368	-3.32193	-3.32193	3.94177	3.96914	3.74465	4.28765	2.03866	2.75887	2.28096	2.43463
EVC2	0.9706	1.39508	0.9899	1.33271	1.21029	1.40584	1.05143	1.71535	1.27818	1.81143	1.30118	1.90023	-2.90929	-3.32193	-3.13986	-3.32193
EVC	3.6325	3.42154	3.32264	3.08087	3.68168	1.33695	3.07919	1.4513	3.8792	4.11291	4.34192	4.3254	1.64143	1.07644	1.87425	1.96882
CRMP1	2.2962	1.86019	1.91809	2.28147	1.80821	0.78	1.50442	1.54688	1.97898	2.10097	2.14912	2.43142	0.78115	0.12228	0.84196	0.83016
WFS1	4.90057	4.55426	5.29496	4.517	5.45906	4.89548	4.92941	4.59424	3.99564	2.74777	3.76029	2.22687	3.5029	2.5274	4.47251	3.03666
PPP2R2C	-3.20506	-2.96114	-3.18447	-3.1759	2.21315	1.10268	1.8405	0.961	1.28349	1.23003	0.51466	1.21148	-3.22958	-3.32193	-3.21015	-3.10199
MAN2B2	0.73233	0.85078	1.56174	1.21677	4.55806	3.68296	4.11301	3.46263	3.85968	3.39869	4.15391	3.55367	2.04673	1.19264	2.76104	2.15091
MRFAP1	7.04532	6.94607	6.72091	6.99047	7.62199	7.46224	7.86329	7.31883	7.06656	6.65561	6.76333	6.60376	7.42298	7.48627	7.28182	7.35316
LINC02482	2.05177	1.47693	1.04334	1.52912	1.915	2.64071	2.19015	2.47736	1.5256	2.18069	0.85974	1.97662	2.44125	2.43619	1.2526	1.57125
S100P	0.01367	0.95421	0.61984	1.43431	5.36456	7.26313	5.11611	6.51417	-2.55233	-3.32193	-2.14799	-2.66675	-3.32193	-3.32193	-2.663	-2.65135
MRFAP1L1	4.57355	4.63198	4.51312	4.71465	4.75725	5.10149	4.95757	5.26547	3.6645	4.06317	3.87098	3.7798	4.52699	4.82743	4.03401	4.07433
BLOC1S4	3.29918	3.44288	3.43168	3.56002	4.26001	5.01883	3.93435	4.70462	3.42706	4.00195	3.6243	3.11341	3.74133	4.15564	3.53633	4.00228
KIAA0232	1.39981	1.58035	1.49893	1.74456	2.9881	1.86976	3.64612	1.99975	1.65369	1.28307	1.14995	2.08045	3.35579	3.23202	3.34006	3.3886
TBC1D14	3.2234	3.08793	2.9699	2.97367	4.02105	3.45795	3.95349	3.32553	3.872	3.74276	3.92219	3.77286	4.01398	3.98164	4.29159	4.04505
CCDC96	1.30772	1.20665	1.20081	0.94667	1.12488	0.89827	0.95329	0.31943	0.9351	0.31654	0.87208	-0.84314	1.11409	0.87853	1.63794	0.82063
TADA2B	2.12418	2.28715	2.27617	2.00663	3.27014	3.07055	3.48546	2.7783	2.42317	2.65685	2.83381	2.49598	2.61634	2.77056	3.43116	2.96052
GRPEL1	4.66927	4.46884	4.20969	4.27484	4.14098	3.84388	4.19466	4.04038	4.05503	3.56946	3.67636	3.42481	4.42224	4.12617	3.39172	3.41565
SORCS2	-0.07742	0.25026	0.56832	-0.14158	-3.32193	-1.57337	-3.16529	-2.11079	0.22783	1.29729	0.79006	1.2527	-3.32193	-3.1898	-3.32193	-3.15856
AFAP1-AS1	5.02761	4.66423	4.34746	4.27959	-0.76135	-2.06745	-0.47718	-2.00324	3.14337	3.34425	3.67497	3.36456	1.85109	1.40604	2.39455	2.20957
AFAP1	3.17629	3.04448	3.16343	3.04129	0.94324	-0.46291	1.29046	-0.15162	5.07085	5.40921	5.4697	6.43215	4.40282	3.84694	4.47048	4.3339
ABLIM2	-1.65236	-0.87188	-1.10527	-0.81167	-1.32299	-0.69221	-1.90146	-0.88214	-2.94533	-2.52089	-3.32193	-2.87313	-2.47061	-2.7362	-3.32193	-3.15174
SH3TC1	-0.36948	0.10917	-0.41309	0.03552	2.35415	3.06729	1.82656	2.95282	0.15502	1.9481	1.23526	1.94251	-0.72471	0.47779	0.00343	1.86078
HTRA3	0.85281	1.06594	1.0659	1.33297	-2.9459	-2.36204	-2.79958	-2.24341	-2.26079	-1.34753	-2.53425	-1.54077	-2.5292	-3.08017	-2.78961	-2.22411
ACOX3	2.06225	2.13122	2.07455	2.29714	3.08877	2.74646	2.69726	2.99072	2.05587	1.94698	2.0695	2.25334	2.60312	2.16837	2.72513	2.5726
TRMT44	0.94768	0.38599	0.25725	0.54327	1.38837	1.50744	1.30625	1.49209	0.50568	0.65333	0.90271	0.7248	0.88564	0.82437	0.99271	0.86852
GPR78	-3.19388	-3.04889	-3.17142	-2.76741	-2.30408	-2.51396	-2.63804	-2.68782	-1.27626	-0.39155	-0.69149	-1.39001	-3.32193	-3.22101	-3.32193	-3.32193
CPZ	-3.02896	-2.73174	-3.32193	-2.43228	-1.52024	-1.94699	-1.89895	-2.09602	-0.21932	0.80744	0.41816	-0.23944	-3.32193	-3.08833	-3.32193	-3.32193
SLC2A9	-3.32193	-3.32193	-3.32193	-3.12263	0.10676	1.40646	0.11878	0.78159	-3.32193	-3.32193	-3.32193	-3.16975	-3.32193	-3.32193	-3.32193	-3.32193
ZNF518B	-0.04649	-0.16676	0.05093	0.14572	2.0803	0.75728	2.658	0.99112	-3.32193	-3.32193	-3.32193	-3.22773	-3.32193	-3.32193	-3.32193	-3.32193
RNPS1P1	0.84068	0.6073	1.20387	-0.43367	0.40144	0.85569	-0.61612	-1.7316	1.1135	0.21393	0.66804	-0.28396	-0.27128	-0.27581	-0.14052	-0.96432
HSP90AB2P	0.15416	-0.7549	0.37835	-1.49125	-0.69864	-1.29621	-0.88585	-0.99558	-0.05397	1.06059	-0.26116	0.16878	-1.35503	-1.35886	-0.94617	-0.46208
RAB28	1.88726	1.99385	1.60378	2.1594	2.93624	2.82694	3.06784	2.57542	2.75732	2.48352	2.53193	2.73535	3.52557	3.31041	2.63355	2.43824
NKX3-2	-3.32193	-2.37108	-2.91238	-2.88919	-0.8699	-0.13218	-1.37891	-0.22093	1.81311	1.87986	2.25666	0.8328	-0.79193	-0.79619	-0.44435	-0.41704
LINC01096	-3.32193	-3.32193	-3.32193	-3.32193	-2.48816	-2.62521	-1.87861	-2.02289	1.20946	-0.0172	0.95951	0.04452	-1.19519	-2.03624	-0.9704	-3.32193
BOD1L1	2.97386	3.18885	3.0915	4.20004	2.46256	2.69327	2.82741	3.07338	2.57087	2.39055	2.45115	3.37705	2.96668	3.06004	3.33739	3.46537
LINC01085	-2.94341	-3.32193	-3.32193	-3.32193	-3.32193	-3.32193	-2.68031	-3.32193	0.15222	0.227	-0.40994	0.30757	-2.76604	-2.36826	-2.66853	-2.65696
LINC00504	-1.74647	-1.23496	-0.85943	-1.04513	-1.61514	-3.11526	-1.00539	-3.32193	-3.32193	-3.32193	-3.32193	-2.95588	-3.32193	-3.15983	-3.12603	-3.32193
CPEB2	1.47606	1.77129	2.18041	2.64229	2.49775	1.12261	3.26738	1.24108	1.90989	1.96961	1.87721	2.76867	4.37831	3.77073	4.54223	4.01073
CC2D2A	0.34876	-0.2053	-0.11708	-0.40171	-0.50079	0.15195	0.007	0.04898	1.77942	1.29956	1.55755	1.9605	1.54692	1.01416	1.22095	1.22682
FBXL5	3.48756	3.4158	3.55112	3.72847	3.45165	3.13186	3.86746	2.89291	4.06438	4.02844	3.94614	4.35876	4.67293	4.41265	4.06953	3.73695
FAM200B	2.24007	2.40825	2.19717	2.40236	1.74137	2.09986	2.26075	2.14139	2.22331	2.26067	1.97169	1.73242	3.27886	3.05977	2.24662	2.3099
CD38	-3.32193	-2.72899	-3.32193	-2.66959	-3.32193	-3.32193	-3.17699	-2.97786	-1.01507	0.17155	-0.47067	0.63132	-3.32193	-3.32193	-3.32193	-2.79391
FGFBP2	-3.32193	-3.32193	-3.32193	-3.32193	1.39588	0.68406	2.51648	1.16456	-2.53809	-0.092	-0.86185	-1.85422	-3.32193	-2.75176	-3.32193	-3.32193
PROM1	-3.32193	-3.32193	-3.15369	-2.28323	2.185	1.15851	2.80326	1.49542	-3.32193	-2.94117	-3.03942	-3.06137	-3.20851	-3.32193	-3.05967	-3.32193
TAPT1	-0.72344	0.21062	-0.0627	0.98364	1.19245	1.87921	2.33401	1.9798	-0.46929	0.56242	0.04915	0.64732	-0.18977	1.28992	1.05548	0.99738

TAPT1-AS1	-0.65811	-0.27728	-1.0019	-0.74553	-0.19192	-0.92683	-0.45545	-0.31902	-1.92548	-1.06125	-1.71931	-1.55783	-1.74634	-0.40004	-0.65205	-0.25133
LDB2	0.62759	0.52722	0.36195	0.26905	-3.23921	-2.43997	-3.32193	-1.91454	-3.32193	-3.32193	-3.32193	-3.32193	-3.32193	-3.32193	-3.32193	-3.32193
QDPR	3.27658	3.65227	3.34619	3.59059	4.50575	5.01663	4.21695	5.052	3.51407	3.79942	3.65359	3.47768	3.21461	3.90251	3.03825	3.82661
LAP3	4.05191	3.83798	3.58831	4.01801	5.10045	4.15759	5.14313	3.90236	4.7943	4.32418	4.77366	4.30539	4.94255	4.75787	4.52636	4.57726
MED28	1.59733	1.78219	1.58349	1.7041	2.31365	2.62612	2.51946	2.51901	2.13627	2.38726	2.49437	2.73094	2.5613	3.22866	2.76986	2.79381
FAM184B	-0.59384	-0.08633	-0.38365	-0.4418	-0.63808	0.40115	-0.70324	0.63207	-0.97512	-0.82783	-0.45135	0.12282	0.11434	0.23832	0.79389	0.86046
DCAF16	3.50649	3.31848	3.23528	2.7113	3.03627	3.11342	3.24591	3.15038	2.46787	2.31603	2.63464	2.53947	3.72026	3.48165	3.4525	3.43636
NCAPG	3.8642	3.7166	3.72635	3.31838	4.77445	3.65556	5.36778	3.91537	4.61688	3.6711	3.80776	3.43166	4.61124	4.43108	4.42609	4.1004
LCORL	1.32358	1.15212	1.12798	1.16127	1.33617	1.50106	2.0264	1.69491	1.48014	0.90151	0.87737	1.27352	1.62064	1.51015	1.5669	1.11742
SLIT2	-1.90067	-2.09207	-1.93816	-1.59061	-3.22718	-3.32193	-3.32193	-3.32193	0.94852	0.70115	1.1064	1.34018	-3.23432	-3.32193	-3.32193	-3.32193
PACRGL	1.52199	1.2957	1.35779	1.88612	1.35655	0.6768	1.44536	0.53685	1.18288	1.31873	1.13342	1.24047	2.0129	1.85447	1.68897	1.20024
KCNIP4	-1.70738	-1.8979	-2.79166	-1.38085	-1.66671	-1.11987	-1.68971	-2.24135	-1.55768	-1.92852	-1.96937	-1.8483	0.31681	-0.37111	0.11693	-0.39859
ADGRA3	1.99147	1.69423	1.69651	1.91666	3.23441	2.46712	3.5827	2.59882	2.00391	2.08218	2.33009	2.34237	2.91853	2.83748	3.1322	3.24665
CDC42P6	2.09662	2.43116	2.46664	2.17715	2.3766	1.7526	3.00429	1.65308	2.77287	3.63733	3.12999	2.60966	1.07428	1.06938	2.03141	2.1503
PPARGC1A	-1.54472	-1.52104	-2.13997	-1.54218	0.10219	-0.01749	0.5717	-0.03694	-3.10038	-3.22587	-3.32193	-3.32193	-3.32193	-3.32193	-3.22679	-3.32193
DHX15	4.36467	4.19019	4.06224	4.28174	4.88561	3.52508	5.21386	3.62894	4.74792	4.1182	4.33595	4.07612	5.21169	5.29186	4.74027	4.72691
SOD3	0.73837	1.39947	1.25706	0.47423	-3.11416	-1.34006	-3.32193	-0.90563	-3.32193	-3.32193	-3.32193	-3.32193	2.43685	2.96111	2.30457	3.36664
CCDC149	-2.75051	-2.69388	-2.85325	-2.63173	-1.553	-0.45287	-1.48575	-0.40524	-1.89182	-1.36219	-2.25787	-1.38042	1.07396	1.30516	0.77275	1.84547
SEPSECS	-0.23917	-0.06995	0.1027	0.6435	1.10227	0.99187	0.90967	0.97555	0.09744	0.28315	-0.69887	0.70911	0.33462	0.83341	0.3258	0.42183
SEPSECS-AS1	-3.08538	-3.05892	-2.81383	-2.57796	-0.70237	-0.60285	-1.15438	0.01615	-2.15675	-1.70758	-2.37866	-2.0885	-0.18168	-0.63836	-1.02546	-1.40279
PI4K2B	2.19301	2.51985	2.86174	3.14051	3.3176	1.97633	3.87692	1.7028	3.25233	2.77932	2.99248	2.93445	3.08731	1.93128	3.26666	2.05834
ZCCHC4	1.84501	1.67289	1.65582	2.0463	1.74793	0.98011	2.0008	1.34124	1.30938	0.49541	1.0134	1.15294	1.77836	1.49192	0.87548	0.8094
ANAPC4	1.58986	1.4535	1.31052	2.008	2.295	2.111	2.53215	2.33683	1.42015	1.2123	1.4873	1.80207	2.09984	2.32174	1.62581	1.83044
SEL1L3	3.04364	3.36801	3.33545	3.92826	3.11449	0.65758	3.23211	0.68152	-0.39113	0.06954	-0.72598	1.04608	2.2486	2.4262	2.84885	3.03576
SMIM20	3.44425	3.45444	3.45123	3.42445	3.80783	3.67318	3.60222	3.26396	3.096	3.18696	2.93194	2.71836	3.77388	3.43953	3.27222	2.90237
RBPJ	3.02423	3.77255	3.7973	4.17122	1.93591	1.69125	2.7759	1.90359	1.70005	2.51228	2.29429	3.54074	1.80625	2.32317	3.10782	3.23369
TBC1D19	0.15969	0.28235	0.22779	0.98775	0.20897	-0.34824	0.63754	-0.18512	0.41653	0.88247	0.58957	1.58134	1.27472	1.30112	1.28123	2.33985
STIM2	1.51775	1.60302	1.28824	2.0502	3.00612	2.66603	3.62491	2.60228	0.59473	0.67344	1.01445	1.49887	0.95556	1.25627	0.67945	1.7976
PCDH7	-3.14979	-2.88301	-3.02891	-2.41129	-3.32193	-3.23234	-3.32193	-3.32193	-0.52168	0.14017	-0.23738	0.78392	-3.18537	-3.18583	-3.00929	-2.68363
LINC02506	3.04297	2.47492	2.66983	2.81582	2.93442	2.83916	3.214	3.09193	2.75209	2.99117	2.16399	2.29616	-2.45136	-0.56106	-0.98903	0.04079
ARAP2	-1.27842	-1.7896	-1.70294	-1.41148	0.31089	-1.24462	1.27504	-1.15487	-0.58599	-1.21354	-1.2178	-0.54352	-3.32193	-3.32193	-3.32193	-3.32193
C4orf19	-1.0225	0.44142	-0.15735	0.21553	1.26102	-0.58515	0.94918	-0.38361	-2.11859	-1.93175	-2.66153	-2.70723	-2.79685	-2.11257	-1.79959	-2.25587
RELL1	1.55102	1.98105	1.8833	1.27192	4.18301	2.04627	4.37512	1.85142	2.78893	2.2599	2.14925	2.48012	3.25266	3.03946	2.8361	2.91264
PGM2	4.4945	4.10416	3.83287	3.95126	4.17531	2.75084	4.33675	2.6103	4.61209	3.85259	3.80431	3.74511	4.41116	3.16176	3.44929	2.71403
TBC1D1	2.98387	2.76416	2.80782	2.53753	3.21502	1.93302	3.4103	1.72205	4.00639	2.89239	3.67739	3.361	3.72839	2.59213	4.36301	3.02237
KLF3-AS1	-0.0715	-0.3821	0.01974	-0.8197	2.55757	1.03534	1.96155	0.48505	1.13218	0.88826	0.80568	0.54176	0.96387	0.87267	1.80976	1.9083
KLF3	2.31638	2.30506	2.23702	2.45101	4.45713	3.25112	4.54544	3.20222	3.58386	3.41494	3.22012	3.86791	4.1261	4.02448	4.14026	4.07611
TLR1	-1.71138	-2.49952	-1.54244	-0.91024	-1.90415	-1.47572	-1.49168	-1.03873	-2.47738	-1.59491	-1.76112	-0.35585	-0.35147	0.95877	0.5583	1.25582
TLR6	0.30066	0.54285	0.14479	0.74398	-0.85378	-1.23423	0.32472	-1.8183	-0.40025	0.88641	0.083	1.99165	1.98505	2.48063	2.73214	3.39801
FAM114A1	2.58002	3.06699	3.15631	3.67265	4.45798	3.78591	4.68683	3.40845	4.27234	4.10955	4.22896	4.9857	4.67185	4.29941	4.91456	4.61632
TMEM156	4.85497	4.71223	4.90669	5.03887	3.96378	2.94411	4.50421	2.15698	2.13614	2.13559	2.05252	2.936	3.33507	2.49408	0.94271	1.62565
KLHL5	3.89444	3.95361	4.06844	4.56328	5.16337	3.18593	5.54064	3.26472	4.07153	3.86701	4.09111	4.75512	4.62286	3.89774	4.77067	4.35909
WDR19	0.98594	1.2376	1.59565	1.98724	1.40715	1.94146	1.44409	1.44453	0.4006	1.37666	1.04071	1.86423	1.63931	1.95965	1.62748	1.77058
RFC1	2.91657	2.84377	2.94907	3.54575	3.81473	2.76137	4.35148	3.02699	3.50651	3.26491	3.41011	3.76805	3.67665	3.7957	4.15297	3.71489
KLB	-2.72696	-2.80623	-2.50971	-3.32193	3.72948	3.06751	3.88044	3.13202	-3.12239	-2.86864	-2.98349	-3.00933	-1.83675	-0.84105	-1.70313	-0.32669
LIAS	3.06816	3.04971	2.96099	2.52207	3.56436	3.15273	4.00804	2.94614	3.15167	3.4585	3.03774	3.52808	3.54729	3.4954	2.87879	2.7124
UGDH	3.62992	3.61172	3.78019	4.13654	6.72496	4.0623	6.77637	4.09801	3.65413	3.15494	3.30731	3.70406	6.31992	4.96738	5.51399	4.67925
UGDH-AS1	-1.76153	-1.2773	-0.33491	-1.39122	0.59209	-0.53266	0.2531	-1.10672	-1.61447	-0.02009	-0.69135	-0.12282	-0.6813	0.07932	-0.1558	-0.17121
SMIM14	0.36672	1.22792	1.49697	2.50673	1.64205	1.37772	2.00848	1.13845	0.01826	0.83305	0.17478	1.55889	1.91917	2.13156	2.82802	2.63931
UBE2K	4.64665	4.85214	4.49662	4.90812	4.86091	4.13911	5.32656	4.24142	4.73082	4.13583	3.73151	4.28232	5.18435	5.1686	4.55519	4.79081

PDS5A	3.34043	3.34447	3.10538	3.48169	4.13302	2.26699	4.10802	2.6262	3.81844	3.3712	3.09413	4.06986	4.43418	3.96661	4.0518	4.07628
N4BP2	-0.45144	0.31336	0.39549	0.79393	1.84221	0.94048	2.91312	0.96972	0.60574	0.9852	0.65963	2.2121	0.87268	1.37912	1.64561	1.89509
RHOH	-0.78202	-0.50142	-1.03028	-0.01504	-2.83747	-2.54581	-2.41344	-2.55767	-3.32193	-3.32193	-3.32193	-3.32193	-3.32193	-3.32193	-3.32193	-3.32193
RBM47	0.21761	0.84196	0.81337	1.53508	4.33462	3.17868	4.60689	3.12106	-0.77177	-0.32056	-0.49073	0.67662	0.9846	3.30536	2.6909	3.4054
APBB2	1.22677	1.37545	1.57254	1.45378	0.99484	0.42399	1.35635	0.13424	3.73733	3.27636	3.8755	4.3442	2.96024	1.90235	3.47376	2.3648
UCHL1	-2.89958	-2.85545	-1.72994	-2.80657	2.97921	6.65515	2.96028	6.33199	6.47138	6.29912	6.2531	6.20942	1.66182	1.93243	1.01137	1.35391
LIMCH1	-0.57427	-0.4313	-0.80075	-0.43355	0.18245	-1.0288	0.27163	-1.80102	3.8639	4.28764	3.38619	4.3332	0.94057	-0.2676	0.37166	-0.09428
TMEM33	4.36493	4.42609	4.13543	4.66422	4.21671	3.73945	4.90528	3.63757	2.52886	2.35435	2.47869	3.01163	3.37662	3.61346	3.61779	3.70913
SLC30A9	2.16094	2.43432	2.61344	2.74506	3.74601	2.63066	4.20003	2.54833	3.12151	3.29062	2.95742	3.53823	3.35152	3.31638	3.60785	3.29692
ATP8A1	-2.68555	-2.76934	-2.74407	-2.88902	-0.51975	-1.44904	-0.02946	-1.364	-3.10642	-3.2286	-3.04052	-2.53727	-3.10422	-2.97653	-3.22949	-2.83056
YIPF7	-0.13073	-0.46468	-0.05562	-1.43359	1.04513	-1.31122	1.43155	-1.71943	0.31552	-0.85183	-0.66163	-2.46463	0.38664	-0.73391	0.65203	0.2463
GUF1	2.69159	2.77559	2.57602	3.09172	3.26672	2.0961	3.81975	2.19433	2.57374	2.1707	2.26104	2.80838	3.00567	2.56939	2.81848	2.59327
GNPDA2	1.69951	1.77143	1.5505	2.40048	2.20579	1.39191	2.58181	1.4234	1.96265	1.63904	1.02441	1.8141	2.72924	2.51979	1.66812	1.78991
RAC1P2	4.09793	4.01778	4.62731	3.28473	3.22073	3.11075	3.12656	3.01288	3.66908	3.87836	3.45038	3.28653	2.30178	2.20149	2.70021	2.00874
COX7B2	4.1299	4.03561	4.17811	4.26012	3.36043	2.86704	3.99095	2.18941	-3.32193	-3.32193	-3.32193	-2.38706	-3.32193	-3.32193	-3.32193	-3.32193
COMMD8	3.04096	3.12636	2.91561	3.73381	4.19804	3.82185	4.63202	3.54252	4.08819	4.03702	3.77264	3.83021	4.28167	4.37763	3.68433	3.87594
ATP10D	0.49131	0.10216	0.06573	1.1795	2.65424	1.15577	2.7968	0.93207	1.80647	1.30874	1.65466	2.44829	0.22495	-0.52841	-0.77653	0.16022
NFXL1	2.24976	1.88521	2.03515	2.44715	2.75232	2.11606	3.16251	2.19137	1.80739	2.15042	1.66273	2.67827	1.75768	1.38581	1.16884	1.00624
NIPAL1	-0.54847	-0.52086	-1.0463	-0.71867	1.10398	-0.80549	1.48555	-0.86921	1.22131	1.6903	1.16136	2.11857	2.01909	2.52371	1.41344	2.23119
TXK	-1.44126	-1.6179	-1.94955	-1.01429	-2.43095	-2.23103	-2.62062	-2.10198	0.5111	1.29252	0.38313	1.40215	-0.38557	-0.49258	-1.04644	-0.84141
TEC	1.26965	1.4297	1.12185	1.80887	1.21199	0.81156	1.56477	1.08118	0.936	1.03559	0.69265	1.67149	1.59069	1.57847	1.12284	1.38867
SLAIN2	3.30955	3.51555	3.30263	3.45392	3.74721	2.63745	3.96981	2.60533	3.62984	3.45466	3.38231	3.95889	3.48303	3.45978	3.35493	3.39258
SLC10A4	-3.32193	-3.32193	-2.72387	-3.32193	5.41904	4.50594	4.50873	4.40198	1.73357	0.48143	1.43572	-0.9685	-3.32193	-3.32193	-3.32193	-3.32193
FRYL	0.85894	0.51468	0.92399	0.95982	1.62304	-0.16696	2.44081	-0.15516	2.0439	0.86597	1.79822	1.85299	2.66541	0.83301	2.82648	1.18975
OCIAD1	3.74449	4.03877	3.79804	4.49886	4.99017	5.04265	5.55519	5.04862	3.30996	3.84527	3.4968	4.12952	4.68967	4.93299	3.91761	4.2657
OCIAD1-AS1	0.89317	2.33081	1.96965	2.18165	2.07201	2.08599	3.05337	2.31525	-1.18303	-0.07817	1.15303	2.09243	2.48716	2.35892	2.43639	1.56466
OCIAD2	5.81749	5.84011	5.94767	5.74965	0.73679	2.55666	0.6935	2.11712	4.84448	5.06724	4.76051	3.7254	5.18551	5.05468	4.61539	4.98923
ANKRD20A17P	0.74467	-0.40549	0.42698	0.65384	-0.28855	0.06215	0.25026	0.50462	-2.57493	-3.32193	-3.32193	-1.91072	-3.32193	-1.82513	-3.32193	-2.22504
DCUN1D4	1.92404	1.67113	1.80262	2.20482	2.69421	2.71661	3.48875	2.56048	2.50097	2.16272	2.62117	2.93101	3.84538	3.32314	3.90761	3.3423
SGCB	2.26308	2.64568	2.92448	3.22259	1.29387	1.24109	1.68302	1.05094	3.14748	3.22942	2.69466	3.68749	-3.32193	-2.98069	-3.32193	-3.32193
SPATA18	-2.61855	-2.2808	-2.51922	-1.84443	-3.32193	-3.32193	-3.32193	-3.32193	0.99916	1.41134	1.46068	2.35144	-3.32193	-3.32193	-3.32193	-3.32193
USP46	1.41556	1.02454	1.34116	1.70198	1.44014	0.49695	2.02893	0.37949	1.58668	0.74666	1.52009	1.31779	2.54969	1.76067	2.5547	1.54593
USP46-AS1	0.36168	0.51597	0.89122	0.04949	1.18283	-0.11458	0.92397	0.41488	-1.48099	-0.42911	-1.04813	0.11488	1.13005	0.68087	-0.30925	0.0559
DANCR	5.76404	5.05604	5.00409	4.77456	5.6937	6.04594	5.50986	6.10982	3.61123	3.36265	3.11275	2.75815	5.54351	4.74277	4.26065	4.18042
SNORA26	2.82738	1.56371	1.6391	2.87339	2.05406	3.83295	2.72945	4.06579	-0.25248	0.95632	-3.32193	-3.32193	2.03108	2.71281	0.94271	0.42398
ERVMER34-1	-2.85649	-3.04255	-3.02852	-2.75571	0.36567	-0.10837	0.60555	-0.53805	-0.4748	-2.25057	-2.33204	-2.87733	0.97396	0.22647	0.636	0.06686
SCFD2	1.37047	1.51724	1.19791	1.21094	2.91477	2.78144	2.81041	2.61555	2.62326	2.13755	2.71422	2.23883	1.74218	2.07343	1.90791	2.47134
FIP1L1	3.36708	3.23812	3.07109	3.24603	2.98434	3.07487	3.13155	3.22308	3.20285	2.91131	3.14716	3.03684	3.43899	3.68122	3.15099	3.34368
LNX1	1.67398	1.40942	1.408	1.85817	0.80461	1.49961	1.02582	1.88587	1.0161	0.6811	0.85866	0.65805	1.33429	1.69266	1.218	1.20747
CHIC2	4.15581	4.14413	4.45064	4.2634	2.52409	2.68056	2.78992	2.61434	4.39069	4.25721	3.96297	3.34317	3.80592	3.47074	3.39246	3.43908
PDGFRA	-2.03771	-1.15491	-2.1061	-0.11163	-3.01498	-3.00938	-3.1217	-3.07132	-2.96794	-3.02244	-3.20741	-3.02702	-2.73714	-3.15263	-3.02512	-2.20598
KIT	-3.32193	-3.32193	-2.74095	-3.32193	-3.16036	-3.13155	-3.32193	-3.32193	0.28563	2.11249	1.2705	2.24961	-3.32193	-3.32193	-3.32193	-3.32193
SRD5A3	1.54077	2.26022	2.67412	1.98442	3.69147	3.27075	3.48497	3.11833	2.26746	2.05201	2.26642	2.32018	1.86756	1.27295	1.80177	1.61562
SRD5A3-AS1	-0.05988	0.52286	0.83102	0.26767	2.17773	1.36995	2.03939	1.14792	0.65261	0.36973	0.59035	0.41047	0.24686	-0.1328	0.00981	-0.16091
TMEM165	4.0705	4.21709	4.4824	4.36746	4.6224	4.13283	4.82258	3.94161	3.2282	3.38062	3.40437	3.04736	4.03668	3.86189	3.5145	3.45536
CLOCK	1.05628	1.30018	1.34872	1.67751	2.17166	0.83289	2.66295	1.02198	1.15352	0.67382	0.64416	1.46063	2.53653	2.05446	2.23235	2.0688
EXOC1	2.33203	2.5527	2.73747	3.29449	2.09748	1.53027	2.7173	1.87045	2.93388	2.95536	2.91229	3.6196	2.78901	2.76	2.75531	2.86984
CEP135	0.57759	0.34433	0.54815	0.50125	0.59241	-0.61259	1.09596	-0.1845	1.0374	0.60464	0.90873	1.4406	2.53438	2.29998	2.02838	1.89885
AASDH	0.58575	0.67574	0.52954	0.85219	0.81918	0.06243	1.08654	0.71996	0.72309	1.3654	0.60085	1.62664	1.57885	1.67144	0.74525	1.29849
PPAT	3.12413	2.73696	2.4883	2.8035	3.07397	1.92899	3.75486	1.8868	3.10859	1.76656	2.65054	2.21575	4.18224	2.82009	3.6134	2.22693

PAICS	5.7345	5.31774	5.24309	4.77439	4.98214	4.86088	5.25651	4.93684	5.59242	5.27167	5.00652	5.31823	5.90664	5.83766	5.53395	5.31718
SRP72	4.68251	4.36803	4.56496	4.73968	5.0699	4.38773	5.58192	3.96772	5.0907	4.47043	4.97542	5.05714	5.23026	3.03573	5.45596	3.87072
HOPX	-3.32193	-3.32193	-3.32193	-3.15024	-3.32193	-3.32193	-3.32193	-3.32193	-0.36795	0.27514	-0.38892	-0.89824	-3.32193	-3.32193	-3.32193	-3.32193
REST	2.09779	2.35911	2.49869	3.17747	2.46505	1.79969	3.21192	1.88608	2.77536	2.3863	2.91319	3.48382	3.12214	2.69998	3.54993	3.40118
NOA1	4.03919	4.12482	4.05588	3.54377	3.7042	3.02139	2.97144	3.1023	4.53038	5.00434	4.82419	4.73103	3.44489	3.34222	3.52524	3.44048
POLR2B	4.44199	4.52611	4.31841	4.82828	4.63556	4.08535	5.15947	4.11466	4.47288	4.46047	4.35635	4.70648	5.89212	5.96943	5.33928	5.32899
IGFBP7	4.00944	4.44992	4.39986	4.56409	2.75718	3.91677	3.205	4.19561	4.19978	5.33038	4.53991	5.07243	8.05766	8.00711	8.04869	7.75394
IGFBP7-AS1	1.89258	2.6341	2.85479	2.23797	-1.18119	1.02803	-1.69816	1.6677	3.19742	4.43049	3.58755	3.94156	6.73337	6.75126	7.17333	6.78204
EPHA5	-3.21233	-3.19947	-3.19296	-3.32193	-2.1368	-3.01227	-1.70701	-3.19247	-2.68922	-2.70577	-2.33555	-3.02976	1.19472	1.0551	2.07539	1.99909
EPHA5-AS1	-3.32193	-2.5628	-3.32193	-3.32193	-1.36504	-2.15357	-0.83468	-1.64322	-3.32193	-1.33112	-1.0485	-3.32193	2.83929	2.16436	3.11119	3.20023
CENPC	0.29441	0.16545	0.45459	0.33377	0.5893	-0.20871	0.8337	0.31824	1.06355	0.3483	0.52093	0.63433	1.32613	0.9635	0.78024	0.51382
UBA6	2.60917	3.02892	3.16794	3.2495	2.66634	2.20039	3.53866	2.04028	2.88741	3.45945	3.2516	4.19587	3.3781	3.43299	3.34572	3.22783
UBA6-AS1	0.34415	0.59101	0.79312	0.52171	0.69556	1.09826	0.97912	0.77075	0.56115	1.08888	1.30168	1.57388	0.73085	0.26018	0.46047	-0.17144
YTHDC1	3.64959	3.62229	3.52546	4.0247	3.27937	2.96138	3.69341	3.21148	3.54653	3.42389	3.53206	3.89063	3.89838	3.91897	3.69201	3.78549
MT2P1	3.63218	3.98265	3.66236	3.02342	-3.32193	-0.91919	-3.32193	-3.32193	4.44563	5.65544	4.10724	5.28907	0.51504	1.4587	1.33886	-0.96051
UGT2B10	-2.18064	-3.32193	-3.32193	-3.32193	0.48266	0.25969	0.83858	-0.1318	-3.32193	-3.32193	-3.32193	-3.32193	-3.32193	-3.32193	-3.32193	-3.32193
UGT2A3	-3.32193	-3.32193	-3.32193	-3.32193	2.75317	1.29733	3.07118	1.63913	-3.32193	-3.32193	-3.32193	-2.75577	-3.32193	-3.32193	-3.32193	-3.32193
UGT2B7	1.08886	1.8205	2.21995	2.67558	0.66751	1.79473	0.84777	1.88599	-0.78782	-0.52213	-0.57892	-0.2924	1.46712	1.64648	0.74374	1.12347
UGT2B11	-2.90868	-3.32193	-3.32193	-3.32193	1.52357	0.71598	2.37224	0.40375	-3.32193	-3.32193	-3.32193	-3.32193	-3.32193	-2.98974	-3.32193	-2.91738
UGT2B4	-3.32193	-2.89152	-3.32193	-3.32193	0.84541	1.09449	1.08654	0.71996	-3.32193	-3.32193	-3.32193	-3.32193	-3.32193	-3.32193	-3.32193	-3.32193
SULT1B1	-3.32193	-3.32193	-3.32193	-3.32193	-2.5049	-2.92136	-1.57189	-2.73507	-3.32193	-3.32193	-3.32193	-3.32193	-1.74478	0.52034	-1.20948	-1.32702
UTP3	4.90577	5.11621	4.7656	5.06064	4.3864	4.30074	4.6307	4.26999	4.0064	3.76196	3.99315	4.19996	4.96712	4.78672	4.25675	4.39222
RUFY3	1.78528	1.83393	1.94005	2.26988	2.12709	2.07158	2.1078	2.04493	1.31097	1.67276	1.70851	2.01712	2.09817	2.32913	2.45679	2.59475
GRSF1	4.3941	4.35037	4.28668	4.37435	4.37795	3.85437	4.6648	3.87977	4.57695	4.49858	4.34787	4.6484	4.73296	4.53111	4.34258	4.31738
MOB1B	1.59405	1.63185	1.56688	2.18682	2.11009	1.43484	2.91483	1.41672	2.4997	2.33604	2.39298	3.00003	3.52261	3.35812	3.34979	3.08732
DCK	2.46871	2.48721	2.33038	2.74169	3.6093	2.3668	4.08406	2.55544	3.39609	3.07485	3.03517	2.78065	4.10348	4.53563	3.69844	3.379
SLC4A4	-2.11037	-1.90965	-2.02238	-1.96728	3.49712	1.26004	3.95988	1.40768	2.16601	1.02508	2.11013	1.89134	0.94365	0.79137	1.1557	1.03014
NPFFR2	-1.59814	-1.63681	-1.2799	-0.31102	-0.72971	-2.32341	-0.10596	-1.02526	-3.32193	-2.92439	-2.57205	-2.36909	-1.28877	-0.11737	-1.27189	-1.77281
ADAMTS3	-1.99136	-1.76445	-2.1316	-1.42189	-3.32193	-3.17213	-3.32193	-3.32193	-0.48127	0.13983	0.13452	0.39016	0.50848	0.63506	0.44413	1.12459
COX18	1.55436	1.74812	1.40983	1.447	2.00785	1.98489	2.1475	1.8717	0.67816	1.02987	1.10536	1.41301	1.35989	1.76935	2.17255	2.14795
ANKRD17	3.3256	3.41044	3.34754	4.00717	3.80367	3.07743	4.18273	3.27536	3.76948	3.53345	3.69295	4.43587	4.10746	3.94346	4.3592	4.50651
ALB	-3.02347	-3.32193	-2.97451	-3.32193	6.76134	4.74413	6.81889	5.48611	-2.98035	-3.32193	-3.32193	-3.32193	-3.32193	-1.83954	-2.79701	-1.35269
AFP	-2.35654	-2.09759	-2.96338	-2.64271	3.96768	5.92203	4.16594	6.29575	-0.8095	-1.60102	-1.63471	-2.23188	-1.46913	0.1667	-2.38875	-0.7101
RASSF6	-3.32193	-3.32193	-3.32193	-3.32193	0.58171	-0.20241	0.98469	0.60385	-3.32193	-2.67646	-2.87441	-2.79209	-2.38321	0.3126	-1.78616	0.08311
CXCL6	-3.32193	-3.32193	-3.32193	-3.32193	1.36531	0.92397	0.91997	0.62083	4.5489	4.93927	4.23906	4.3233	1.13026	4.91046	-0.0254	2.38814
CXCL5	9.26287	10.2118	9.25749	10.333	6.38358	4.54157	6.97544	4.76346	-2.27485	-2.94911	-1.5005	-2.21117	1.17386	2.12865	-0.89447	0.49881
CXCL3	5.63811	6.17503	5.02987	5.6551	1.92554	0.25854	1.6187	0.67594	3.58639	3.79994	3.38217	3.15701	0.58252	0.75156	-2.09805	-1.42254
CXCL2	6.34854	6.82311	5.37637	6.61104	3.55687	3.13491	3.28681	3.02892	5.37007	5.06948	4.94548	5.00365	3.41133	3.14152	0.30592	0.77885
MTHFD2L	1.58538	1.19619	1.04065	1.28388	0.43884	0.40353	0.59382	0.4233	0.15139	0.323	-0.29977	0.13303	2.05736	1.5459	0.99581	1.26221
EPGN	-3.32193	-2.35075	-2.73036	-3.32193	-2.53379	-2.8033	-2.83715	-3.32193	0.31478	-0.17806	0.79903	0.48328	-2.09495	-2.3205	-3.32193	-2.81856
EREG	-2.57451	-2.93816	-2.75389	-3.32193	5.18999	3.73085	6.68717	2.74157	5.34252	5.13738	5.94056	6.57553	6.53869	4.66656	6.12636	4.89606
AREG	-3.32193	-3.32193	-2.68229	-3.32193	6.99671	6.96465	7.43935	6.32253	1.01145	1.24529	0.23059	1.82281	4.33948	3.36742	2.83982	2.84349
BTC	-2.68852	-2.6271	-3.32193	-2.55991	-1.43744	-2.24151	-1.04127	-2.11325	-1.36467	0.64315	-0.92064	1.4673	1.04606	1.25133	1.46347	1.71093
PARM1	-2.37703	-3.09167	-3.32193	-3.06536	5.11982	4.0306	5.58653	3.59609	-1.01667	-1.13033	-1.4285	-1.24623	2.97351	2.91187	2.85289	3.14182
RCHY1	1.50127	1.83856	1.48704	2.19918	1.15305	1.28145	2.01814	1.31639	1.44606	1.94935	1.75795	2.22422	1.14514	2.17945	1.5073	1.52046
THAP6	0.83829	1.131	0.79783	1.26877	1.0172	0.31625	1.69191	0.57136	0.92404	0.96989	0.94677	1.30106	1.46561	1.38753	0.95627	1.1192
ODAPH	0.21164	0.36458	0.17583	0.0759	-2.92035	-3.32193	-2.88608	-3.32193	-0.1672	-1.82529	-0.91436	-1.66692	-3.32193	-3.32193	-2.87751	-2.86908
CDKL2	-3.32193	-3.32193	-3.00789	-3.32193	-3.20134	-2.82104	-3.32193	-3.32193	0.39708	0.53206	0.19151	1.04642	-2.91961	-3.1075	-3.32193	-3.05852
G3BP2	3.44977	3.7883	3.88833	3.87993	4.87505	3.76003	5.69992	3.39718	4.18868	4.09099	4.7625	4.72045	4.84865	5.02415	5.97863	5.2892
USO1	4.05548	4.3368	4.32199	4.85766	5.0943	4.49332	5.66684	4.27315	4.47511	4.28747	4.61743	5.21943	4.5261	3.97351	4.42517	4.06666

NAAA	1.6694	1.34598	1.3527	1.92275	-0.78464	-1.68417	-1.12835	-0.96002	1.56202	0.52881	0.81465	0.87386	1.94993	0.26374	1.10032	0.54483
SDAD1	3.64961	3.81239	3.61068	4.2682	3.67269	3.65511	4.41338	3.46218	3.56778	3.36522	3.57469	4.03489	4.25303	4.19967	4.21629	3.97061
NUP54	3.21871	2.80406	2.92029	3.21567	3.31607	2.21407	3.76755	2.41945	3.54759	2.96716	3.00669	3.17759	3.90631	3.19905	3.10755	2.33374
SCARB2	2.68262	2.67731	2.95849	3.25132	3.95892	3.57074	4.43649	3.35581	2.49123	2.77089	2.56556	3.66927	3.43188	3.43725	3.7386	3.8047
FAM47E	-2.91429	-3.07919	-3.18875	-3.18043	-2.0669	-2.38998	-2.07612	-1.58634	-3.07125	-3.21262	-3.32193	-3.2144	-3.14829	-2.99437	-3.11295	-2.92289
FAM47E-STBD1	1.60624	1.96921	2.22226	2.36372	3.87776	3.70056	4.36397	3.4185	3.33082	2.88651	2.98403	3.01182	3.11931	2.39449	3.31154	2.74661
STBD1	1.61959	2.01723	2.22359	2.37038	3.9766	3.76708	4.36197	3.43147	3.37677	2.97611	3.05392	2.99529	3.151	2.41786	3.3408	2.82743
SHROOM3	0.54838	0.33927	0.58256	0.08473	2.43039	-0.16307	2.20961	-0.20981	0.34745	-0.66902	-0.23188	-0.89937	1.87466	1.24481	2.84252	1.88197
SOWAHB	-3.01039	-3.32193	-3.32193	-3.32193	3.56078	0.94692	2.27046	0.95217	-2.23742	-2.77117	-3.00035	-1.82216	-3.32193	-2.86112	-2.77571	-2.76569
CCNI	5.62625	5.7656	5.78545	6.18127	5.11947	4.84038	5.63615	4.71189	6.04346	5.72451	5.7376	6.94967	5.67211	5.13399	6.48623	5.63169
CCNG2	1.10201	1.89321	2.16792	2.42897	-0.8509	0.64153	0.00263	-0.24919	2.63459	3.39894	2.64766	3.5067	1.03792	2.05759	1.92206	2.54295
CNOT6L	1.03361	1.30537	0.94547	1.28866	1.39321	0.66248	2.17512	0.72006	1.77918	1.35213	1.4516	2.40809	2.72622	2.64711	3.05096	2.67962
MRPL1	3.89525	4.00144	3.97422	4.6761	4.08286	3.99382	4.51377	3.95358	3.80555	3.52856	3.858	3.84178	4.16238	3.8177	3.76963	3.16792
FRAS1	-3.05329	-3.07853	-3.18837	-2.62584	3.71462	1.98303	3.759	1.85799	-3.19078	-3.26608	-3.32193	-3.16305	2.13029	2.33533	3.1129	3.28438
SERBP1P5	2.08413	2.13033	1.53747	1.17441	1.91998	0.6432	1.89501	1.24292	2.42511	2.53689	1.7518	1.37406	1.24204	0.17124	1.03214	0.25124
ANXA3	-3.11842	-3.32193	-3.08377	-3.32193	3.10534	1.02148	4.0546	0.12573	-1.95185	-2.11104	-2.05142	-2.12496	5.9391	5.50972	5.41802	4.36289
BMP2K	1.79	1.90216	1.94726	2.23694	1.48536	0.61277	1.77268	0.61399	1.58728	0.94859	0.76712	1.7474	3.23169	2.61719	2.7835	2.4198
PAQR3	2.38886	2.66217	2.71306	2.97404	1.66185	0.71698	2.09835	0.89258	1.64085	1.20695	0.96161	1.70305	3.04313	3.03546	2.65841	2.63094
LINC01088	1.16157	1.97737	1.19315	1.85083	-2.21416	-1.07946	-2.53502	-0.51296	-3.32193	-3.32193	-3.05739	-3.32193	-3.32193	-2.50776	-3.07645	-3.32193
NAA11	3.40829	3.43398	3.22644	3.37879	3.1888	2.53464	2.86027	2.71358	-3.32193	-3.32193	-2.946	-3.32193	-3.32193	-3.32193	-3.32193	-3.32193
ANTXR2	2.02067	2.19177	2.36523	2.30082	1.7245	0.44036	1.97054	0.77372	3.16532	2.59475	2.91179	2.17972	3.60884	2.54367	3.41239	3.20265
PRDM8	-2.31116	-1.78652	-1.83459	-1.57817	-3.12823	-3.09414	-3.32193	-3.32193	1.36598	1.7565	1.42953	1.4404	2.39013	3.02541	2.44945	3.36253
FGF5	0.93475	0.75109	0.58017	0.52554	-3.24475	-3.14455	-3.32193	-3.32193	2.67172	2.44041	2.52861	2.84356	6.04799	5.31314	5.63164	5.12363
BMP3	-3.32193	-3.11788	-3.32193	-2.89782	-3.32193	-3.32193	-3.32193	-3.32193	-3.11113	-3.32193	-3.32193	-3.32193	1.98158	2.8481	4.22467	2.88323
HNRNPDL	5.70168	5.4565	5.53062	5.58864	4.94791	5.39874	5.20472	5.46376	5.40058	5.17989	5.37927	4.8587	5.58799	5.47286	5.07234	4.65317
ENOPH1	4.86362	4.66157	4.43448	4.63649	4.6051	4.6975	4.90903	4.57043	4.59169	4.2012	4.12193	3.79772	5.68243	5.85709	4.87827	4.8971
TMEM150C	-3.32193	-3.01473	-2.73606	-3.32193	-2.70017	-3.04244	-3.32193	-2.73415	1.06387	0.62993	1.0351	1.47803	1.5107	1.18812	2.86112	1.98482
SCD5	-0.72184	-0.983	-0.80765	-1.03482	-2.70125	-2.53005	-3.32193	-3.32193	4.07305	3.40325	3.91695	3.61317	4.65148	4.76645	5.38583	5.03713
SEC31A	3.87499	4.1098	4.06178	4.61821	4.11302	3.91558	4.47109	3.87541	4.35618	4.33917	4.4829	5.40089	4.27116	4.07907	4.48275	4.38973
THAP9-AS1	2.82935	2.76476	2.79019	3.01784	1.72618	1.84717	2.21132	2.0898	1.56162	1.85732	1.79266	1.72804	3.16637	3.28472	2.50542	2.10532
THAP9	-0.32423	-0.38912	-0.71451	-0.3612	-0.00309	-0.41097	0.72708	0.13953	-0.89614	-0.50498	-1.26702	-0.15479	0.62826	0.99139	-0.01526	0.6495
LIN54	1.44074	1.31102	1.45903	1.57572	1.79504	1.15172	2.45247	1.35339	2.04456	1.15261	1.25868	1.51666	2.59845	2.3916	2.55603	2.19418
COPS4	2.92353	2.78474	2.72198	2.92615	3.62183	2.37997	3.94656	2.21108	3.60192	3.5414	3.6799	3.7204	3.77859	3.652	3.83719	3.36682
PLAC8	-0.04966	-1.04587	-0.06023	-1.397	3.0701	2.59134	3.36067	2.02755	5.42685	4.97234	5.08342	4.41253	2.04959	1.14215	0.34839	-0.6206
COQ2	2.90964	3.16785	2.94309	2.98933	3.57569	3.15342	3.74904	3.2953	3.71242	3.35294	3.38904	3.28214	3.48427	3.57218	3.27646	2.86948
HPSE	-1.75012	-0.93127	-0.86786	-1.07837	1.47203	0.19808	1.1787	-0.50193	1.21203	1.00168	1.10008	0.4948	1.18689	1.03611	1.3318	0.59625
HELQ	1.11728	0.93095	0.61947	1.16183	1.03152	0.62024	1.00872	0.87997	1.09822	1.24956	1.15192	1.69193	1.5198	1.67822	1.18606	1.40588
MRPS18C	3.38564	3.54852	3.1455	3.36858	3.17875	3.04614	3.02233	2.77687	3.21366	3.12572	2.77429	3.02307	3.64911	3.4489	2.79757	2.71014
ABRAXAS1	2.7584	2.90982	2.57921	2.76603	2.59306	2.28172	2.47237	2.06872	2.75668	2.69177	2.42004	2.88455	3.1745	2.94698	2.50919	2.46839
GPAT3	3.35585	3.18469	3.06561	3.89489	4.76138	3.83666	4.97334	3.63818	3.539	3.63488	3.0135	4.6516	4.96766	4.73348	3.93871	4.46547
NKX6-1	-3.32193	-3.32193	-3.32193	-2.92414	-2.80869	-2.99507	-3.32193	-3.32193	-3.32193	-3.32193	-3.32193	-3.32193	0.79523	1.112	0.62359	0.35395
CDS1	-2.29062	-1.75987	-1.36378	-1.07895	2.01903	0.88085	2.58378	1.13094	-2.46681	-1.25581	-3.32193	-0.87484	0.03821	2.35376	1.53963	2.44124
WDFY3	0.66651	0.62952	0.79163	0.8223	2.06458	1.06202	2.47579	1.02765	1.74127	1.64529	1.6346	2.55883	2.48847	2.32995	3.04752	3.04375
WDFY3-AS1	0.60108	-3.32193	0.28576	-0.5224	2.89814	-0.44088	3.95696	-0.23619	2.57425	1.57741	2.22231	3.37818	3.38681	3.01581	4.11977	4.00696
WDFY3-AS2	-1.79746	-2.33421	-2.19373	-2.04599	-1.94665	-1.55486	-1.86151	-1.62937	-1.46681	-1.27335	-1.96838	-1.17144	-1.57667	-1.38363	-1.72223	-2.17072
ARHGAP24	0.59767	0.65636	0.38873	0.96971	-3.20641	-2.47887	-3.07921	-2.46026	1.61637	1.6367	1.94547	1.82476	2.49466	2.33827	2.72312	2.43199
MAPK10	-2.44445	-2.49312	-2.66005	-2.2815	-2.95041	-3.14713	-2.88178	-3.17367	-1.81584	-1.52514	-1.36419	-1.79559	-0.97371	-1.12207	-0.67465	-0.91651
PTPN13	-3.20859	-3.1953	-3.06654	-3.18026	0.36417	0.65162	1.21891	0.32978	2.0388	2.25031	2.73957	2.98086	1.60966	1.87694	3.23409	2.353
C4orf36	0.73212	0.91473	0.42092	0.6834	-0.72111	-0.09873	-0.56198	-0.84832	-0.34973	0.14834	-0.18758	0.12598	-0.05091	-0.4955	-0.79069	-0.83677
AFF1	1.9269	1.96889	1.97746	1.8169	2.35736	1.51416	2.61374	1.29162	2.12758	1.78536	2.2955	2.38993	3.17353	2.22416	3.07724	2.90464

TECRP1	4.66615	4.32677	4.58909	3.60994	3.57016	5.91246	2.57551	6.2405	4.1651	3.83059	3.45162	2.74708	2.57903	2.97463	2.25171	2.42658
KLHL8	1.58098	1.39778	1.18622	1.87076	1.4589	0.92041	2.04585	0.85373	1.59435	1.01681	1.03286	1.19602	2.30117	1.72097	2.04631	1.68271
GAPDHP60	1.28692	0.58573	0.57159	0.35799	-0.59254	1.39161	-2.53274	0.89744	1.82592	2.74827	1.8048	0.183	-1.53769	-0.49632	-2.0061	-2.50537
HSD17B11	3.49667	3.42293	3.81859	4.15089	6.53648	5.52225	7.07804	5.26976	4.36372	4.1541	3.86417	4.43186	5.31119	4.55848	4.92645	4.42625
NUDT9	3.19657	3.31063	3.11237	3.27811	4.53002	4.43071	4.78467	4.5401	2.68855	2.50355	2.44044	2.65902	3.68871	4.10967	3.58963	3.85712
HSP90AB3P	2.68299	2.56385	2.3957	1.09017	1.7939	1.52638	1.50275	1.40539	2.23518	2.92756	2.04719	2.00994	0.38206	0.84862	0.64738	1.13177
SPP1	5.11448	5.11237	5.4264	6.37883	2.17981	2.79275	2.30395	1.91039	5.61116	5.70621	5.66025	5.49551	8.75291	9.19527	9.22948	9.41089
PKD2	1.7244	1.56168	1.83264	2.03901	2.54239	1.82944	2.62325	1.68086	2.90059	3.03312	3.01517	3.55532	3.34066	3.00022	2.82952	3.3521
ABCG2	1.06014	1.06662	0.57002	1.41503	5.48228	3.64184	5.64548	3.6867	-0.56308	-0.12314	-0.1853	0.56664	-2.5125	-2.86258	-3.32193	-3.32193
PPM1K	1.04188	0.93782	0.71052	0.98432	0.93926	1.16306	0.6048	1.54048	-0.28286	-0.12636	-0.80154	0.02688	-1.1947	-1.69994	-1.92733	-1.70613
HERC6	0.67855	0.46277	0.31361	1.27327	0.92451	-0.94363	1.42019	-0.47098	0.57141	0.47708	0.77397	1.71717	-3.17793	-2.44482	-2.60861	-2.71504
HERC5	2.64514	2.72796	2.36047	3.39543	0.88195	0.36229	1.5193	0.56091	2.11769	2.0316	1.79657	3.09237	-1.98226	-2.40365	-2.76962	-2.92376
PYURF	5.50394	5.39753	5.34708	5.27643	5.29596	5.00708	5.47035	4.74544	5.55752	5.38102	5.37749	4.68507	5.93057	5.16556	5.03897	4.55633
HERC3	3.01958	2.91571	2.90513	2.77891	2.92562	2.44951	2.99317	2.27395	3.44814	3.19097	2.9876	2.7813	3.41079	2.68943	2.58196	2.1598
PIGY	6.0962	6.13652	5.89292	6.13101	5.8229	5.29112	6.11981	5.09307	6.19511	5.57576	5.60398	5.51957	6.64978	5.97546	5.5924	5.05561
NAP1L5	1.82547	2.05432	2.02205	2.58734	0.09182	-0.08126	-0.66514	0.88753	-3.32193	-3.32193	-3.32193	-3.32193	0.95144	2.09647	1.33707	1.74983
FAM13A-AS1	-0.58231	0.11781	0.70406	-0.01347	-0.85429	0.23728	-0.66636	0.34108	-0.51439	-0.23999	-0.78255	0.54008	-1.28601	-1.49312	-1.33736	-1.31372
FAM13A	0.11992	0.60773	1.03437	0.68263	-0.40831	0.12652	-0.13391	0.07042	-0.4311	0.21997	-0.30074	0.85983	-1.37607	-1.39719	-1.35263	-0.82564
TIGD2	1.51113	1.31204	1.50786	1.78667	1.37286	0.13764	1.52102	0.64474	2.07802	1.14864	1.70002	1.64107	0.89357	0.05889	0.37603	-0.18947
GPRIN3	-3.25011	-3.24155	-3.15721	-2.919	2.62476	1.59506	2.61861	1.68733	-3.23877	-3.18647	-3.17689	-2.75254	-3.32193	-3.32193	-3.32193	-3.32193
SNCA	-3.32193	-3.32193	-3.32193	-3.32193	-2.66673	0.32587	-2.69764	0.79313	-3.32193	-3.32193	-3.32193	-3.07911	-3.32193	-3.32193	-3.32193	-3.32193
TMSB4XP8	9.16887	9.23551	9.39598	8.8348	8.00266	8.43444	7.63023	7.82731	8.32579	9.11354	8.38719	8.95119	8.63099	8.00203	7.79345	7.5463
SMARCAD1	2.94443	3.10435	2.81413	3.67053	2.58121	2.11312	3.32905	2.21195	2.33467	2.18072	2.15563	3.20928	3.56686	3.66142	3.48744	3.66507
PDLIM5	3.14421	3.36178	3.29166	3.72846	3.00859	2.18385	3.41191	2.18163	3.99146	3.93397	3.09282	4.44546	4.06192	3.91353	3.73224	3.80221
BMPR1B	-0.15322	0.12918	-0.4904	0.2468	-2.98724	-3.32193	-3.16517	-2.95177	-2.95921	-2.65032	-2.61097	-2.76979	-2.75472	-2.7564	-2.88663	-3.01161
LINC02267	-0.15074	0.14621	-0.35279	-0.67652	-1.53588	-1.03318	-2.64567	-0.15189	-3.32193	-2.9336	-2.58758	-3.32193	-2.73515	-2.73687	-2.38273	-2.62134
RAP1GDS1	2.26104	2.15978	2.41177	2.38684	1.79862	1.55367	2.49646	1.44412	2.86495	2.54256	2.82712	3.14778	3.74243	3.39224	3.47572	3.24118
TSPAN5	1.98349	2.0995	2.63826	2.08428	0.06954	-0.11436	0.3449	-0.54805	3.2804	3.46059	3.29375	3.6333	4.40691	4.45585	4.57326	4.94549
EIF4E	2.53728	2.47076	2.40326	2.84608	2.83289	2.41029	3.28559	2.49702	2.77538	2.40544	2.40008	2.0082	3.52062	3.48255	2.77344	2.68159
TBCAP3	1.84101	1.89905	1.46067	0.05948	2.45629	0.89575	2.44376	2.08482	2.81547	1.97746	1.27457	-0.30854	2.31385	2.78589	1	2.07956
METAP1	3.7633	3.46734	3.51673	3.56469	3.98482	3.27326	4.20169	3.31048	4.47026	4.14097	4.03622	4.59316	5.17655	5.11433	4.47497	4.4577
ADH5	4.84048	5.13629	5.05256	5.5649	5.17813	5.52647	5.28	5.51731	4.96561	5.22546	4.614	5.01712	5.34245	5.8837	4.99463	5.18329
ADH4	-3.32193	-3.32193	-3.32193	-2.8371	0.88509	3.46892	1.62429	2.7732	-3.32193	-3.32193	-3.32193	-2.94348	-3.32193	-3.32193	-3.32193	-3.32193
ADH6	-3.32193	-2.84076	-2.81822	-3.32193	2.96614	3.2338	3.01502	3.25383	-2.63044	-2.57328	-2.69146	-2.44544	-2.56423	-2.56633	-3.09743	-2.42626
ADH1A	-3.32193	-3.32193	-3.32193	-3.32193	0.78272	-2.89161	0.5336	-1.92396	-3.32193	-3.32193	-3.32193	-3.32193	-3.32193	-3.32193	-2.33315	-3.32193
ADH1C	-3.32193	-3.32193	-3.32193	-3.32193	2.51991	-1.19261	1.75351	-0.08357	-3.32193	-3.32193	-3.32193	-2.48105	-3.32193	-3.32193	-3.32193	-2.8291
TRMT10A	-0.30588	0.30324	-0.08941	0.08692	0.70314	0.16535	1.33081	0.47085	-0.85405	-0.01636	-0.83904	-0.14163	0.70255	0.64763	-0.36426	0.11885
MTTP	-2.42455	-2.60339	-2.18944	-2.69477	4.84371	3.45408	4.87699	3.71868	-2.09728	-2.15341	-2.51892	-2.16701	-2.08823	-2.09119	-2.82437	-2.67924
LAMTOR3	2.87137	3.02489	3.10557	3.76237	2.32818	2.93337	3.11628	2.67112	2.21628	2.32354	2.17437	2.61036	3.07317	3.03314	2.57446	2.15545
DNAJB14	0.99073	1.08289	1.14813	1.65497	1.87734	1.0773	2.8316	1.04425	1.63578	1.74929	1.80217	2.68841	2.34861	2.11272	2.34817	2.07048
PPP3CA	3.59337	3.81798	3.78528	4.03685	3.2721	2.86698	4.11888	2.99321	4.46	4.95131	5.00395	5.43215	3.92533	4.39609	5.79942	5.44094
BANK1	-3.32193	-3.32193	-3.32193	-3.32193	-1.9088	-2.72654	-1.25716	-2.94287	-3.32193	-3.32193	-3.32193	-3.32193	-0.9012	-0.33956	-0.9046	-1.24979
SLC39A8	2.47239	2.37338	2.18762	2.60585	1.97091	0.46847	2.21731	0.42157	2.34201	2.05847	1.58061	2.11986	2.51616	1.94459	1.96218	2.04698
NFKB1	3.4369	3.31572	3.26863	3.21595	3.52159	2.37624	3.65722	2.21539	4.21261	3.95253	4.28849	4.27685	3.31664	3.19587	3.32372	3.19063
MANBA	1.96658	1.75405	2.23238	2.54229	2.57822	2.90577	3.05317	2.95587	0.43406	1.17348	1.18444	2.08781	1.88603	1.71423	1.97427	2.05886
UBE2D3	5.03585	5.30501	5.06942	5.40397	4.6974	4.4517	4.95498	4.38558	5.08567	5.03516	4.74598	5.1229	5.18269	5.22637	4.6888	4.87728
CISD2	4.08829	4.05371	4.12539	4.26525	5.07498	4.84425	5.50539	4.58505	3.93789	3.43403	3.58178	3.27733	5.30437	4.76666	4.81381	4.15792
SLC9B1	2.70917	2.7781	2.86109	2.98186	4.27709	3.56757	4.46274	3.20422	3.01794	2.53147	2.60137	1.52007	3.98057	3.6099	3.7751	3.33242
SLC9B2	1.93587	1.98476	1.23788	2.22533	3.23808	2.62631	3.56911	2.64376	1.09168	0.6942	0.3916	0.68349	2.98724	2.40834	2.1606	1.80011
BDH2	1.95806	1.94841	1.09658	2.52294	1.34801	2.76137	1.01958	2.92157	0.1199	0.66195	-0.57693	0.22118	2.24947	2.61294	0.68827	1.58998

CENPE	3.45692	3.26038	3.17287	3.72959	2.88171	1.81013	3.64169	2.21879	2.87216	2.2364	2.17584	2.57687	3.33334	2.84321	2.97308	2.59135
TET2	-0.43299	-0.74405	-0.08498	-0.11913	0.15632	-0.78315	0.58346	-0.79511	0.27697	0.03171	0.21296	1.15149	1.09832	0.721	1.52821	1.18562
PPA2	3.19724	3.20992	3.21828	3.3894	3.07295	2.90728	3.42383	2.8038	2.89281	2.86875	2.69867	2.57657	4.22932	4.48292	3.82202	3.67184
EEF1A1P9	3.77407	3.27414	3.88036	1.0591	2.15756	2.38034	1.67384	1.60261	3.05641	4.65589	2.9259	3.75558	1.90654	1.13934	1.81929	2.01543
ARHGEF38	1.59873	1.75521	1.06327	1.43212	0.58118	0.30908	1.16426	0.60887	0.53111	0.2702	0.70276	0.22387	1.74725	2.0898	1.97907	1.81872
INTS12	3.23583	3.31261	2.96197	3.08274	2.57161	2.21749	2.99007	2.29534	2.39569	2.36481	2.40013	2.32447	3.35638	3.68354	3.64577	3.40531
GSTCD	1.94414	1.86497	1.70267	1.83627	2.38723	0.56919	2.67178	0.99401	1.3908	1.05212	0.89729	1.27853	3.06989	3.85495	2.82622	3.05407
NPNT	-3.32193	-3.12985	-3.32193	-3.10759	3.85997	2.43589	4.44529	2.41184	-3.32193	-3.15541	-3.32193	-3.15807	1.7948	3.3745	3.12516	3.1033
TBCK	0.86804	1.01483	1.17613	0.99052	0.98815	0.39467	1.15407	0.6435	0.46752	0.91442	0.73368	1.45865	1.27505	0.98628	0.98587	1.29367
AIMP1	4.92682	4.74129	4.60633	5.04373	4.15456	4.22709	4.55066	4.21087	3.80518	3.62978	3.71122	3.63135	4.52654	4.02057	3.72648	3.72341
PAPSS1	4.64793	4.97307	4.73138	5.31442	3.35358	3.15369	3.5564	3.42892	4.3008	4.07024	4.10101	4.55181	3.60233	3.83415	3.75501	3.82896
SGMS2	1.64113	1.22421	1.21159	1.32176	2.91925	0.27502	3.49297	0.07747	2.38952	0.78022	1.57792	1.47456	2.80666	0.61947	2.53048	0.49512
CYP2U1	-0.06942	0.19305	-0.53898	0.36666	0.10929	-0.47817	0.21126	-0.77453	1.01709	1.20013	0.68128	1.48104	1.91859	1.73557	1.74224	1.73567
HADH	2.08474	1.78122	1.881	1.75493	1.5036	1.70287	1.36492	1.91312	2.06452	1.57505	1.56586	1.64413	1.61977	2.18476	1.75183	1.80592
LEF1	-3.32193	-3.10471	-3.32193	-3.07977	1.96794	2.31873	2.00196	2.07567	-3.32193	-2.96668	-3.12033	-3.32193	-3.32193	-3.32193	-3.32193	-3.32193
LEF1-AS1	-3.32193	-3.32193	-3.32193	-3.32193	0.46211	-0.07357	0.1911	-0.83223	-3.32193	-3.32193	-3.32193	-3.32193	-3.32193	-3.32193	-3.32193	-3.32193
RPL34	7.74852	7.9132	7.89272	7.53786	6.26391	6.86018	6.50601	6.58992	6.48063	7.21242	7.0044	6.71498	6.52909	6.75139	6.06359	5.76579
OSTC	6.24489	6.44021	6.50097	6.89753	6.53272	6.98265	6.92713	6.88041	5.84849	6.04052	5.72805	5.76146	6.19154	5.98093	5.33068	5.46518
ETNPPL	-3.32193	-2.94865	-3.32193	-2.9082	3.43652	1.68359	3.34259	1.32741	-3.32193	-3.32193	-3.32193	-3.32193	-3.32193	-3.32193	-3.32193	-3.32193
SEC24B-AS1	-0.57631	-2.0902	-1.1334	0.06754	-1.7792	-1.42154	-2.96537	-1.66956	-2.55789	-2.41974	-2.9311	-2.22503	-1.47859	-1.07354	-1.37047	-1.34701
SEC24B	3.53732	3.64405	3.64625	4.00852	3.05494	2.19555	3.62241	2.17694	3.31007	3.32529	3.21199	4.31318	3.7014	3.64232	3.86613	3.47816
MCUB	4.17264	4.19371	4.15704	3.93229	3.49628	3.25736	3.58557	3.01313	3.41269	3.14596	2.44455	3.25857	4.00843	3.66365	3.35328	3.08206
CASP6	3.95963	4.07237	3.96113	4.39761	3.87672	3.5818	3.97478	3.41884	3.69093	2.98536	3.1426	3.00937	4.18241	4.09807	3.78567	3.95756
PLA2G12A	2.77397	2.77927	2.6082	2.97085	3.47767	3.10862	3.56357	2.89978	1.88331	1.81614	1.85056	2.08324	2.20557	1.92481	2.23464	2.06036
CFI	-3.32193	-3.32193	-3.32193	-3.32193	2.06456	0.84885	1.71294	1.55331	-3.32193	-3.06717	-3.32193	-3.32193	-3.11165	-2.48862	-2.66779	-2.07815
GAR1	6.24587	6.18797	5.90812	5.58602	4.27202	4.52818	4.72101	4.69028	4.38014	3.96774	4.25698	3.95338	5.27773	5.26706	4.37712	4.18013
ELOVL6	3.39845	3.03457	3.13455	3.01119	1.51146	2.86039	1.49078	2.74496	2.68756	1.61547	2.17228	2.15897	3.1989	3.02012	2.82715	2.56123
PITX2	-3.13558	-2.37442	-2.74657	-2.55959	-2.88753	-2.96712	-2.99148	-2.91268	0.54819	0.25125	0.84777	-0.22077	-0.66821	-1.00508	-0.93996	-0.9144
FAM241A	1.97694	1.78127	1.41803	1.63461	0.65771	0.78421	1.40466	0.99098	1.36911	1.04202	0.6857	1.03035	2.26502	2.33391	1.01059	1.35564
AP1AR	4.32995	3.93323	4.29203	4.23632	3.84597	2.28157	4.36672	2.07073	2.58345	2.10962	2.59766	2.15645	3.46776	1.67635	2.83939	1.14482
TIFA	3.71777	3.79431	3.6658	3.7369	1.50083	1.72801	1.78998	1.25136	2.45163	2.42366	1.94326	2.6706	2.23034	2.46765	2.19012	2.00741
ALPK1	0.84312	0.91159	0.90732	0.94739	-0.8927	-0.24311	-0.76642	-0.6625	0.81082	0.97839	0.73606	1.21456	0.64179	1.07425	0.63687	1.12238
ZGRF1	0.87916	0.58223	0.60808	0.47673	-0.33054	-0.13556	-0.08375	0.15587	-0.26299	-0.9227	-0.44236	-0.69599	0.98813	1.16979	0.45404	0.58284
LARP7	4.62738	4.68383	4.68146	5.46694	3.10178	4.06886	3.79378	4.25144	2.94083	3.36337	2.91145	3.8011	3.75835	4.06346	3.46986	3.48689
RPL7AP30	1.54971	1.75836	1.88007	0.53999	0.01581	1.46557	-0.84927	0.57691	1.09761	1.39976	1.01373	0.03467	0.12832	-0.66018	-0.60265	-1.37539
ANK2	-1.45781	-1.56063	-1.59034	-1.33754	-1.49399	-1.36402	-1.27212	-1.67692	-0.45664	-0.76439	-0.44359	-0.34677	0.48668	1.27136	1.73486	1.6422
CAMK2D	1.108	1.4533	1.37022	2.04217	3.28501	2.05306	3.77898	2.04422	1.93995	2.4209	2.11663	3.26198	4.56186	4.93445	4.81134	5.00362
ARSJ	1.38052	1.1032	1.10757	1.31031	0.13651	-1.47701	0.60587	-1.66537	1.05726	1.87309	0.76013	1.92718	3.47407	4.31663	4.32816	3.59987
UGT8	-3.32193	-3.32193	-3.32193	-3.32193	-2.55514	-2.90618	-2.16269	-2.84383	-0.23661	-0.64044	-0.44796	-0.43057	-3.32193	-3.32193	-3.32193	-3.32193
TRAM1L1	-0.65039	-0.59992	-1.42387	-0.77517	0.43788	0.58152	0.31726	0.80135	0.38298	0.08661	0.88652	0.8262	0.73372	1.62989	0.7123	1.52129
SNHG8	6.25963	6.25115	5.97864	5.375	5.37221	5.8473	4.62912	5.59415	4.54003	5.33045	4.90584	3.76252	5.54252	4.84066	3.5633	3.29024
SNORA24	4.74204	3.81399	4.02181	3.839	3.19508	4.26818	2.4932	4.63015	1.115	2.39005	1.91934	-3.32193	-0.82585	1.73875	-0.58716	-3.32193
PRSS12	-1.42999	-1.0127	-1.48611	-0.87577	1.42501	1.39822	1.56554	0.84243	2.45658	2.15907	2.34641	2.3791	-2.81114	-2.07353	-2.04468	-1.80989
CEP170P1	0.63703	0.85535	0.58802	0.39781	-0.36683	-0.70552	0.37888	-1.38931	1.33288	1.99982	2.16978	3.64374	1.41052	2.16517	2.56845	2.98969
METTL14	2.38193	2.55176	2.52474	2.94946	1.95621	1.8339	2.42161	1.84708	2.08895	2.35531	2.10647	2.62749	2.06823	2.27541	1.95588	1.97857
SEC24D	3.1642	3.30873	3.34439	3.89296	3.07159	2.5506	3.01736	2.32043	3.24011	3.26847	2.97388	3.68449	2.63909	2.40365	2.35184	2.37431
SYNPO2	-2.58759	-2.32687	-2.98613	-2.44397	-3.28843	-3.32193	-3.24931	-3.32193	-2.49705	-2.20209	-2.34085	-1.36458	-0.96693	0.02181	0.34729	-0.216
USP53	2.03589	2.64276	2.74456	3.41084	0.95797	0.04609	1.52437	0.3583	0.31026	0.1565	-0.64781	1.23478	1.44382	0.96389	0.95729	0.99643
C4orf3	5.47262	6.17917	6.43169	6.01529	4.46113	4.97539	4.54922	4.83069	5.20986	5.80472	5.01489	5.20233	4.58103	4.79176	3.7441	4.19099
GTF2IP12	0.85778	0.67492	0.68982	0.53259	-0.53512	-0.6456	-1.02029	-0.17695	-0.57568	0.06675	0.14169	0.70492	-0.39648	-0.48694	-0.74713	-0.05462

PDE5A	-1.40394	-1.15985	-1.1583	-1.39346	-0.98664	-1.11638	-1.23073	-1.12387	0.01958	-0.01721	0.39761	0.46423	0.0028	0.7057	-0.2596	-0.29896
MAD2L1	4.44883	3.83032	4.02862	3.50679	3.61218	2.01141	3.6528	2.13357	3.42506	2.6578	2.64491	1.41145	3.97198	3.60512	3.32759	2.51097
PRDM5	-0.24791	0.21634	-0.32983	-0.0672	-2.29403	-1.91644	-2.22307	-1.4282	-0.37142	-0.2529	-0.28966	0.01758	-3.32193	-3.23289	-3.32193	-3.32193
NDNF	-3.32193	-3.32193	-3.32193	-3.32193	-3.19404	-3.32193	-3.32193	-3.32193	-2.14021	-0.37055	-2.57493	0.28368	-3.32193	-3.32193	-3.32193	-3.32193
TNIP3	3.02381	2.58384	2.65495	3.08284	-3.08932	-3.32193	-3.06822	-3.00579	0.94079	-0.16086	0.7476	0.93807	-3.32193	-3.32193	-3.32193	-3.32193
EXOSC9	4.73824	4.41342	4.18222	4.40806	3.55799	3.30661	3.91293	3.44844	3.80366	3.11219	3.21971	2.74889	4.14396	4.10366	3.61017	3.00468
CCNA2	5.94412	5.43779	5.36495	5.13841	5.54621	4.27555	5.72433	4.3568	6.18598	4.71183	5.2931	4.31475	6.27664	6.07002	6.29152	5.5102
BBS7	2.64886	2.40472	2.6038	2.60053	2.07354	1.39781	2.2527	1.08836	2.0016	1.79207	1.53463	1.94985	2.76256	2.68616	2.10069	2.04643
KIAA1109	1.03747	1.22817	1.32116	1.88541	1.5788	0.5544	2.27468	0.70379	0.86296	1.21595	1.12805	2.67826	2.32653	2.44032	2.73224	3.11405
CETN4P	-1.83108	-1.34544	-1.90599	-0.01616	-1.9783	-1.6271	-2.36668	-1.36911	-3.32193	-2.66021	-3.32193	-2.85578	-1.71366	-1.2183	-2.21597	-2.48579
BBS12	1.57144	1.99404	1.5022	2.59408	-0.78533	-0.78548	-0.14857	0.13121	-0.04777	1.2168	0.61263	1.91301	0.87675	1.04232	1.01549	1.33773
FGF2	6.1151	6.42999	5.60667	6.43308	-0.83084	-0.37373	-0.13182	-0.10291	4.71508	4.95305	4.869	5.21924	5.23217	5.49933	4.81584	4.81269
NUDT6	3.89513	4.12983	3.45884	4.59674	-0.3398	-0.19449	-0.46091	0.0149	2.48602	2.76542	2.35779	3.36968	3.29893	3.56991	2.49636	2.54167
SPATA5	2.51322	2.53704	2.38953	2.13773	0.93401	-0.34907	1.17135	0.07237	1.4348	0.5826	0.50196	0.66081	1.94036	1.86143	1.94702	1.35685
SPRY1	-0.42221	0.33915	0.19419	-0.81163	2.54574	2.79725	2.52324	2.98247	0.77924	1.61586	0.69741	1.83665	1.30925	2.25646	2.09412	3.24836
LINC01091	-3.05944	-3.32193	-3.32193	-3.15078	-1.48005	-1.38665	-1.6281	-0.86003	-3.32193	-3.32193	-3.32193	-3.32193	-2.92944	-2.31293	-2.9593	-2.08126
ANKRD50	1.56535	1.64214	1.62778	2.09695	1.41755	-0.09113	1.76352	-0.1222	3.06494	2.77943	3.00317	3.52297	4.02557	3.30397	3.76809	3.63612
FAT4	-3.01618	-2.75934	-2.63016	-2.75945	-1.98628	-2.58356	-1.90258	-2.53115	2.28845	2.34887	2.57039	2.81135	2.12219	2.05582	2.06173	2.27114
INTU	-1.01121	-1.47288	-1.06573	-0.78146	-0.90251	-0.83875	-0.54328	-0.83736	-1.5985	-1.32867	-1.4141	-0.39695	0.14133	0.69242	0.07688	0.74378
HSPA4L	1.89264	1.65233	0.88645	1.2691	2.57189	1.12419	2.74457	1.32591	2.69278	1.78073	1.46297	2.47281	3.37664	3.01098	2.4155	2.46532
PLK4	2.43727	1.85516	2.07143	1.59897	2.77638	1.41884	2.98316	1.69182	2.78306	1.98451	1.8034	1.5232	3.03066	2.717	2.21615	1.68227
MFSD8	0.85896	0.71432	0.95484	1.2124	1.79693	1.4666	2.26169	1.73327	0.55347	0.71144	0.58914	1.37714	1.55522	1.41635	0.90028	0.98577
ABHD18	0.71584	1.0609	1.20779	1.25963	0.3015	0.50358	0.46834	1.06421	1.32446	0.55784	0.81541	1.09782	1.75641	0.88398	0.43908	0.50438
LARP1B	1.45389	1.37125	1.21235	1.89167	2.35087	2.17284	2.66208	2.42232	1.17953	0.8294	0.66126	1.40274	2.51491	2.09161	1.46198	1.07532
FOSL1P1	0.19691	-0.43337	-0.86058	0.08483	-2.83244	1.48163	-3.32193	2.46732	-0.61534	-0.43047	-1.75117	-1.43246	-2.00562	-2.0087	-1.82819	-1.80776
PGRMC2	3.67813	3.58284	4.08366	3.63536	4.14856	3.83433	4.6475	3.54029	5.21159	4.66601	5.04823	5.29081	5.34747	4.15757	5.10211	3.41416
LINC02615	-1.57172	-0.72702	-0.42629	-0.86865	-0.16297	1.6597	-0.52323	1.93041	1.16565	1.87934	1.26428	2.23091	0.72747	1.71115	0.82395	0.4035
JADE1	3.06999	2.88005	2.76297	2.75807	2.73488	2.13682	3.01639	2.59836	3.61529	2.89955	3.15783	3.08165	3.99385	3.57853	4.26907	3.43993
SCLT1	2.07026	1.88065	1.72934	1.74897	1.53136	1.26849	1.87711	1.67577	2.43542	1.60091	1.82857	1.93275	2.74405	2.00589	2.52972	1.7211
C4orf33	-0.23051	0.38355	-0.33153	0.12651	0.723	0.47136	1.18189	0.4471	1.04794	0.56195	0.74002	1.02138	0.94126	1.16473	0.50629	0.36473
LINC02377	-1.49515	-3.32193	-0.72976	-1.66936	1.6949	1.41989	1.67412	1.33569	1.30383	1.25865	1.25527	0.96456	-3.32193	-3.32193	-3.32193	-3.32193
PES1P1	-0.46215	-1.14797	-1.68101	-0.85854	1.35015	0.71095	1.40362	0.34467	-3.32193	-3.32193	-3.32193	-3.32193	-3.32193	-3.32193	-3.32193	-3.32193
PCDH18	-3.16349	-3.32193	-3.32193	-2.65638	-3.32193	-3.32193	-3.17351	-3.32193	-1.23845	-1.67724	-0.97466	-0.62099	-3.32193	-3.08163	-3.32193	-3.02731
SLC7A11-AS1	-0.75805	-0.81961	-1.22319	-0.71574	-0.19138	-1.49518	0.47869	-1.76227	-1.45971	-1.64993	-1.27509	-0.94904	0.08039	0.14151	-0.66841	0.35632
SLC7A11	2.75074	2.03601	1.75272	2.61446	2.29226	1.99603	3.0404	1.82602	1.48221	0.81603	0.75176	1.80199	2.05872	1.79629	0.78972	1.82673
NOCT	4.55087	4.51864	4.31149	3.37723	3.23975	3.57466	3.13417	3.68019	4.14122	3.83765	3.73301	3.56005	2.88929	2.77625	3.13755	2.78144
ELF2	2.07909	2.22007	2.11868	2.39565	1.38442	1.04389	1.71467	0.86827	1.94949	1.43587	1.76817	2.01623	2.33655	1.74363	2.12687	1.88797
PPP1R14BP3	5.39088	5.74786	5.6879	4.5643	4.36584	5.81641	4.33088	5.5793	5.52093	5.81005	5.49469	2.4741	2.90273	3.02376	2.44057	2.82338
MGARP	-1.86834	-3.32193	-3.32193	-3.32193	-1.09155	-0.21448	-1.0627	-0.47298	1.37452	0.73553	1.0154	0.44765	5.10938	5.21032	4.67561	3.9316
NDUFC1	5.26327	5.22825	5.11215	5.07546	4.89155	5.85681	4.99887	5.68549	4.70493	4.48952	4.39358	4.01206	5.55923	5.62242	4.79784	4.50417
NAA15	4.56819	4.50369	4.22525	4.85863	3.90358	3.03065	4.72665	3.14635	3.77696	3.07029	3.40674	3.7989	4.32932	3.76485	4.44164	3.70634
RAB33B	0.82137	1.49574	1.18662	1.69479	1.36629	0.81092	1.81936	0.73168	0.07167	0.80918	0.16788	1.44326	1.43295	1.70358	1.44534	1.3788
SETD7	3.25601	2.91513	2.93215	3.06449	2.861	1.38368	2.96629	1.29177	2.18906	1.1073	1.49633	1.79407	3.70798	2.4545	3.59719	2.78954
MGST2	3.63713	3.69067	3.52171	3.87726	4.70431	5.12298	4.60435	4.97814	-0.46649	-0.4441	-1.14005	-0.2125	2.68368	2.40027	1.96193	2.40297
MAML3	0.92075	0.49	1.19527	0.74754	1.96109	0.58388	2.08158	0.28572	-1.33677	-1.48941	-1.20916	-1.03435	2.2126	0.49027	2.71067	1.79494
SCOC	3.78142	3.59194	3.89288	4.01896	2.95445	1.82419	3.7596	1.55124	2.97347	1.37689	2.39878	1.30628	4.15052	1.07192	2.98502	0.87407
SCOC-AS1	-0.77827	-0.28117	-0.65712	-0.98124	-0.93237	-0.1297	-1.68768	0.48783	-2.51568	-2.15703	-2.58448	-1.41594	-1.50514	-0.85591	-1.40842	-2.61835
CLGN	4.54574	4.67122	4.50727	5.35497	2.89874	3.9219	3.35395	3.93299	-0.13957	-0.09283	-0.76482	0.09044	-0.97614	-0.3619	-1.06145	-0.66004
ELMOD2	1.51208	2.10208	2.0244	2.58474	2.74103	2.4809	3.49334	2.33487	1.02364	1.8797	1.26613	2.2884	2.2568	2.96816	2.37507	2.6723
TBC1D9	3.67363	4.14312	3.68197	4.54147	3.26406	2.11889	3.54501	1.83182	3.06346	2.7089	2.62525	3.15987	2.34204	3.4322	2.79714	3.37293

ZNF330	4.87717	4.70994	4.55818	4.9401	2.48492	3.41718	3.08239	3.38375	3.87276	3.37023	3.55668	3.34998	3.6816	3.64928	2.78217	2.49952
LINC02432	-3.32193	-3.32193	-3.32193	-3.32193	-2.22251	-0.63471	-2.6624	-0.58601	1.85292	1.6841	1.61295	1.41698	-3.32193	-3.32193	-3.32193	-3.32193
IL15	0.20983	0.2992	0.34464	0.60507	-2.85995	-2.94396	-2.31195	-2.65542	-0.7526	-0.59557	-0.73402	-0.26857	-2.06344	-1.49666	-2.05043	-1.9761
INPP4B	1.14844	1.18051	0.75293	1.00271	-3.25625	-3.32193	-3.18131	-3.32193	2.30205	2.41266	2.25389	3.07344	1.76347	0.87479	1.72887	1.52476
USP38	2.66659	3.02958	2.62787	3.06679	1.71696	1.01055	2.17902	1.10492	2.03452	1.95856	1.90487	2.52599	2.06749	2.48764	1.96933	2.39901
GAB1	0.09277	0.08975	0.57558	0.91935	-0.18149	-0.70882	0.51045	-0.79992	0.23725	0.14518	0.35156	1.41111	1.88256	1.87284	2.23003	2.1289
SMARCA5	4.46488	4.56452	4.50397	4.78631	4.21477	3.28243	4.80103	3.22971	4.41244	4.06114	4.30147	4.75693	4.35897	4.57508	4.75963	4.59203
SMARCA5-AS1	4.96851	4.86023	4.85304	3.93932	5.43919	2.52271	5.13168	1.85466	5.65562	5.23417	5.5977	4.40596	4.42369	4.51071	5.58055	5.52181
GUSBP5	-2.48971	-3.32193	-2.7737	-2.7441	-3.32193	-3.32193	-3.32193	-3.32193	1.0983	1.11737	0.25268	-0.53144	-3.32193	-2.93831	-2.51862	-2.85644
HHIP-AS1	-3.32193	-3.32193	-2.19687	-1.50755	-3.04489	-3.32193	-3.32193	-3.32193	-3.32193	-3.32193	-3.32193	-3.32193	2.933	3.04817	2.46609	2.50946
HHIP	-3.23205	-3.32193	-3.1174	-3.20936	-3.32193	-2.98854	-3.23786	-3.21564	-3.32193	-3.32193	-3.32193	-3.32193	1.0973	1.16844	1.6213	1.41107
ANAPC10	2.90638	2.45784	2.45639	2.40601	2.3711	1.4065	2.96459	1.19176	2.38049	1.79755	2.20111	1.98409	3.15061	2.87217	2.58476	2.18221
ABCE1	5.45712	4.99924	4.91656	5.27005	4.43842	3.73761	5.15054	3.84873	4.67084	3.8634	4.26755	4.38857	5.65431	5.16439	4.98872	4.3808
OTUD4	4.0661	3.98241	3.82561	4.18815	3.36136	2.31369	3.9111	2.66191	4.04649	3.6597	3.74049	4.37514	4.67728	4.66002	4.42532	4.54387
SMAD1	1.37754	1.4112	1.33828	1.69493	0.97947	-0.23227	1.17922	-0.5264	1.28568	0.82196	1.36097	1.05636	2.31845	2.4007	2.48654	2.4889
SMAD1-AS1	3.33687	2.97958	3.35496	2.93296	3.29735	1.34888	3.19172	0.08351	3.35443	3.00636	3.62085	3.4134	4.0583	4.11741	4.54611	3.83507
MMAA	-0.93069	-0.3361	-0.78815	-0.18687	-0.50348	-0.40701	-0.83032	-0.56239	-1.18187	-0.81619	-0.68119	-0.12873	-1.46846	-0.68763	-0.73237	-0.552
ZNF827	1.80192	1.85287	1.9894	2.62133	-1.17148	-2.59826	-1.38271	-1.42018	1.49426	1.62798	1.91845	2.69356	1.63536	1.93416	2.34794	2.43986
LSM6	3.62805	3.4043	3.40193	2.96649	2.38108	2.02007	2.16906	2.19175	3.3747	2.86208	3.28082	0.63957	2.01787	2.33741	1.76901	1.38268
SLC10A7	0.52079	0.20814	0.359	0.75053	0.92164	0.7113	1.65771	0.95311	1.03996	0.61596	0.71282	1.6186	-0.2733	0.08461	-0.17337	-0.14534
TTC29	-2.77018	-3.32193	-2.45176	-2.65463	-3.32193	-3.32193	-3.32193	-3.32193	-2.08698	-0.86453	-0.50789	0.29415	-2.13455	-3.0809	-3.03222	-3.02643
EDNRA	-3.32193	-3.32193	-3.32193	-3.32193	-3.32193	-3.32193	-3.32193	-3.32193	-1.1825	-1.52602	-1.78729	-0.81414	-3.32193	-3.32193	-3.32193	-2.70262
TMEM184C	3.26609	3.40358	3.27519	3.73375	3.68947	2.42291	4.02997	2.17873	2.92331	2.57167	2.75951	3.34645	3.19309	2.94709	3.34993	3.22874
PRMT9	1.69084	1.93663	1.64153	2.26456	1.6331	1.19334	1.95808	1.57101	0.95652	1.15243	1.27537	0.93538	1.21659	1.32016	1.17452	1.11967
ARHGAP10	0.96637	0.81543	1.01643	0.51557	0.51874	0.03743	0.93965	-0.14158	2.5344	2.13486	2.57682	2.59519	2.51823	2.49311	3.00738	2.82441
NR3C2	-0.59106	-0.3241	-0.57649	-0.79952	-0.032	-0.55221	0.32347	-1.19983	-2.16561	-1.95129	-2.34115	-2.24222	-3.07819	-3.19535	-3.32193	-2.77741
DCLK2	-0.93994	-0.32234	-0.69191	-1.25472	1.15409	0.33171	0.89655	1.19192	0.83543	1.00078	0.81418	0.57505	-0.55461	1.14911	0.58461	0.48444
LRBA	1.42427	1.77407	1.68825	2.12844	0.5329	0.46183	0.97339	0.47109	2.21355	2.4192	2.26997	3.25441	2.46811	2.92519	3.20367	3.53113
SNORD73A	5.90212	6.00964	5.89513	6.23778	5.11201	5.44497	5.61884	6.53002	2.49865	4.01967	4.12244	3.889	5.22949	5.22436	3.52357	3.93053
FAM160A1	0.68769	0.61417	0.4906	0.32448	-0.08605	-2.24213	-0.50994	-2.67898	-0.26594	-0.77204	-1.40686	-1.17425	1.89228	1.51803	2.10662	2.45685
GATB	2.39749	2.1318	2.16086	2.24796	1.03458	1.21987	1.03364	1.11437	0.94	1.31078	1.09619	1.18764	1.2036	1.23703	1.05591	1.06391
FBXW7	1.98655	2.1574	2.03716	2.44193	1.77383	1.90546	1.86767	2.20314	1.52165	1.33496	1.34843	1.44999	1.68479	1.561	1.26607	1.59595
FBXW7-AS1	2.80425	2.51605	2.70632	2.10906	2.89811	1.3958	3.26943	1.36822	1.7016	2.24101	2.07262	2.68462	2.99016	2.92447	2.78086	2.58601
MIR4453HG	-0.23404	-0.04228	-0.10852	-0.17817	-0.04691	0.12958	0.3045	-0.01107	-1.43191	-1.739	-1.66931	-2.01653	-0.41779	0.41352	-0.52949	-0.13942
TMEM154	-1.76489	-0.74614	-1.20659	-0.73963	-3.28146	-2.90118	-3.23443	-3.32193	-3.32193	-3.32193	-3.22514	-3.32193	-2.17988	-0.86333	-2.39949	-1.46809
TIGD4	-2.14825	-2.28126	-2.00703	-2.4798	-1.16265	-0.26862	-1.05593	-0.23979	-3.32193	-2.38967	-2.33953	-1.69738	-1.75256	-0.82195	-1.3228	-0.91882
ARFIP1	3.01781	3.3535	3.23008	3.88417	3.01228	2.86537	3.84349	2.51111	2.67271	2.97248	2.75049	3.31591	2.94327	3.29389	3.08766	3.00549
FHDC1	-1.52612	-2.37715	-1.51031	-1.2279	-0.01461	2.30574	0.2748	1.81983	-0.75489	-1.46958	-2.1717	-2.31878	1.95992	1.66835	1.2988	1.19073
TRIM2	-0.35203	0.30667	0.30062	0.97144	0.01835	0.37732	0.50802	0.00157	1.04418	1.698	1.2287	2.31587	1.42251	2.06197	2.40299	2.71742
ANXA2P1	-0.73004	0.05761	0.2537	-0.07939	-1.25028	-1.89172	-1.96313	-1.73995	0.96423	1.93274	0.57456	0.82258	-0.40235	-1.46708	-0.59913	-1.53377
MND1	3.93584	3.28213	3.39312	2.80185	2.87628	2.56476	3.002	2.59122	2.61592	1.91276	1.40823	1.16512	3.60074	3.03556	2.45798	1.56419
TMEM131L	2.14862	1.81565	1.84599	1.99738	1.25032	0.90114	1.75972	1.16161	1.68008	1.30828	1.59346	1.8148	2.14648	2.21102	2.78254	2.89632
TLR2	1.33386	1.68309	1.88377	2.54564	-3.11159	-3.32193	-3.09235	-3.03529	-3.32193	-3.32193	-3.32193	-3.32193	-1.86921	-2.42235	-2.28028	-1.8348
PLRG1	4.47678	4.47259	4.48365	4.62905	4.30288	3.93253	4.76044	3.87857	3.82938	3.87926	3.78105	4.05908	3.94995	4.14304	3.5764	3.38136
LRAT	-3.32193	-3.32193	-3.15638	-3.32193	-2.71494	-2.6236	-2.85391	-2.87174	-2.64703	-2.94685	-3.32193	-2.57254	0.34016	0.91585	0.8676	0.33551
MAP9	-2.98521	-3.18658	-3.04975	-2.90848	1.35613	1.869	2.12754	2.11141	1.32411	1.54292	1.11146	2.53572	-3.32193	-3.32193	-3.32193	-3.32193
GUCY1B1	-1.90655	-2.5549	-2.52224	-2.98496	-3.32193	-3.04606	-3.32193	-3.32193	1.46714	0.5721	0.68822	1.33922	-3.32193	-3.10446	-3.32193	-3.32193
TDO2	-3.32193	-2.90945	-3.32193	-2.86538	-3.2343	-2.78865	-2.82329	-2.16646	0.01192	1.95146	1.67833	3.85206	-3.32193	-3.32193	-3.32193	-3.32193
CTSO	-1.81647	-1.34046	-0.81883	-1.00574	-2.6823	-3.32193	-3.00549	-2.62149	1.8282	1.4245	2.81568	3.40747	-1.44385	-3.32193	-2.99902	-2.99264
PDGFC	2.02458	2.43178	2.35914	2.81856	1.78555	0.87952	2.32085	1.25954	4.09052	4.18731	3.81041	4.58433	1.67732	2.46653	2.73512	2.54639

GLRB	0.97257	1.02799	0.97142	1.17167	0.67457	0.59124	1.01926	0.59879	1.93717	2.0366	1.50731	1.73689	0.86606	1.82093	0.75297	-0.06801
FAM198B-AS1	-3.32193	-3.32193	-2.91472	-2.41985	-3.32193	-3.32193	-3.32193	-3.32193	-1.25167	0.28078	0.01794	0.40761	-1.52284	-1.43985	-1.05565	-1.79567
TMEM144	1.89493	2.13673	2.01414	2.34843	1.64015	1.93623	2.20132	1.68528	0.63623	0.48916	0.03147	0.5073	-1.62744	-1.66058	-2.08139	-1.96754
C4orf46	3.6149	3.24263	3.09683	3.04774	1.80829	1.06943	2.10275	1.35057	2.64786	1.80361	1.54781	1.59424	2.5495	2.64152	1.52669	1.61569
ETFDH	3.70659	3.7463	3.71996	4.10221	3.2409	3.38879	3.57129	3.38897	2.20717	2.08667	2.27029	2.61885	1.77783	2.31266	1.25848	1.82328
PPID	6.01545	6.02064	5.84616	6.09096	4.19859	4.12583	4.77691	4.17431	3.77519	3.5562	3.66604	3.76752	3.80092	3.94577	2.79739	2.9234
FNIP2	1.9066	2.37122	2.37898	3.31392	1.49443	0.54343	2.08166	0.68117	1.19624	0.60926	1.34138	1.50338	0.89711	1.40473	1.20733	1.49462
RAPGEF2	1.60175	1.68391	1.94599	2.07217	2.32224	0.66933	2.99662	0.64248	1.95321	1.40587	1.9069	2.44149	0.37892	0.21391	1.38184	0.99927
NAF1	1.83617	1.5669	1.40245	1.12337	2.17912	1.07552	2.13069	1.26542	2.28824	1.82378	1.77021	1.52095	1.4558	1.54106	1.34203	1.00437
NPY1R	-2.86633	-3.32193	-3.32193	-3.01812	-3.01558	-3.07372	-3.09134	-2.79417	-0.97126	-1.30622	-1.68232	-0.75425	-2.79948	-1.94653	-2.40632	-2.14028
TMA16	3.29585	3.29579	3.06539	3.32207	2.57181	2.90568	3.07225	2.9038	2.97366	3.04976	2.98265	2.75258	1.76067	1.66442	1.34869	0.70552
NACA3P	1.31929	0.77668	0.73193	0.38913	-0.84216	1.33454	-2.23923	0.73728	0.43612	1.87985	0.8774	0.85052	-1.073	-1.8009	-3.32193	-0.81725
TMEM192	2.43632	2.29705	2.111	2.52462	2.2166	2.82073	2.85555	2.7104	1.34488	1.44936	1.1956	1.67649	1.18462	0.87918	1.1923	1.25861
KLHL2	2.75591	2.62105	2.70829	2.95739	2.65866	2.37486	3.24964	1.81117	2.27886	2.00854	2.19698	1.86923	1.66806	1.2442	1.1156	0.64805
MSMO1	3.51753	3.77769	3.26401	4.16352	5.215	6.47778	5.73965	6.56983	4.47153	4.11563	4.09713	4.11467	4.6959	5.59599	4.23693	4.7034
CPE	-0.05012	-0.80794	-0.82043	-0.66665	3.72541	2.91227	3.67103	2.67897	3.21884	3.65522	3.27722	3.65665	-2.6289	-2.45855	-2.73599	-1.04671
DDX60	0.53135	1.32217	1.40453	2.23751	-0.74155	-2.46293	-0.02902	-1.77861	1.75463	1.95638	2.06131	3.11562	0.63252	0.80863	0.62114	1.45841
DDX60L	0.7998	1.21061	1.25816	2.06015	-2.15371	-2.99055	-2.22949	-2.33118	1.78223	2.54244	2.38203	4.40142	1.27554	1.68708	1.20581	2.14332
PALLD	4.50798	4.0837	4.64705	4.37258	4.57328	2.02864	4.66103	1.98247	2.45379	0.6326	1.59334	0.68334	2.87069	0.3427	2.96486	0.85987
CBR4	2.67192	2.61701	2.77941	3.04754	2.89168	2.2206	3.08868	2.07685	1.92209	1.89436	1.61259	1.96391	1.37783	1.53513	1.17674	1.29669
SH3RF1	2.29756	2.23277	2.23589	2.18347	1.98637	0.67172	2.58181	0.13915	2.95446	2.74713	2.88577	3.09172	2.40901	2.48947	3.32879	2.67866
NEK1	1.33316	1.43315	1.29712	2.01397	0.80776	-0.48612	1.2485	-0.28238	1.20432	1.16026	1.12477	1.52202	0.41092	0.42979	0.13612	0.25576
CLCN3	2.97948	3.0538	3.15671	3.67001	3.57882	2.78274	4.08527	2.61158	3.03691	2.94507	2.90188	3.65842	2.75424	2.7824	3.04692	3.07969
HPF1	4.08889	4.14999	4.28077	4.40947	3.79416	3.77682	4.25318	3.59179	4.30476	4.05496	3.88999	4.11826	3.07749	3.36602	2.83144	2.27705
MFAP3L	-2.33883	-2.55842	-2.92623	-2.48631	0.59128	-1.18069	0.4867	-0.8795	-0.58286	-0.51592	-0.90965	-0.8075	-2.39267	-2.75128	-2.13822	-2.63798
AADAT	0.46021	-0.60993	-0.60997	0.28391	1.33433	1.49127	1.4716	1.70427	1.79042	0.98765	1.52508	1.23022	0.63058	0.422	-0.11983	-0.01559
GALNTL6	-3.09794	-3.07277	-3.18509	-3.32193	-1.38927	-1.10438	-1.44804	-1.55758	-3.32193	-3.32193	-3.32193	-3.32193	-3.32193	-3.14419	-3.32193	-3.32193
GALNT7	3.26253	3.05485	3.26247	3.81039	3.94598	2.94298	4.78721	2.91382	3.84905	3.67445	3.83622	4.34054	-2.21664	-2.88575	-1.76469	-2.03846
HMGB2	6.34206	6.06668	6.20733	5.56179	4.75512	4.88309	4.89264	5.22846	7.17076	6.15965	6.56615	4.68141	5.27699	5.55786	4.4766	3.96875
SAP30	5.44464	5.41502	5.57099	4.97034	4.16427	4.44616	4.23804	4.17576	4.75998	4.44351	3.76787	3.31699	3.45561	3.2283	3.06213	2.48756
MORF4	-0.03028	-0.29034	-1.78891	-3.32193	-1.18617	-0.77376	-2.00451	-0.57687	-0.60506	1.17513	-1.21354	0.21515	-2.62043	-0.73896	-1.98492	-1.58207
FBXO8	2.46297	2.82907	2.57464	3.13455	3.23299	2.80111	3.73058	2.75891	2.74015	2.60074	2.41578	2.58287	1.67549	2.05433	1.35187	1.27381
CEP44	0.91433	0.83638	0.7644	1.209	0.94876	0.26336	1.17864	0.29405	0.73854	0.31477	0.28221	0.24271	0.22117	0.39484	0.26462	0.04897
HPGD	-2.96494	-3.32193	-3.32193	-2.88426	1.92458	-0.43699	2.75344	-0.81733	-1.67986	-1.59897	-2.95362	-1.94515	-1.91617	-1.98044	-2.09529	-2.16303
WDR17	-2.6471	-2.78959	-2.76508	-2.51218	-0.06981	-0.6605	0.02654	-0.39199	-3.32193	-3.32193	-3.32193	-3.32193	-3.32193	-3.21411	-3.32193	-3.32193
SPCS3	4.93629	5.11464	5.14651	5.20404	4.42371	4.37216	5.1805	3.92166	3.72831	3.85543	4.35655	4.45925	3.48996	3.58849	3.35344	3.21936
VEGFC	3.90506	4.505	4.70956	4.89021	-3.32193	-3.32193	-3.32193	-2.85574	3.78462	3.89856	4.17849	4.74711	-3.32193	-3.32193	-3.32193	-3.32193
NEIL3	2.76865	2.05751	2.23235	2.03792	2.63591	0.90828	2.71449	1.64438	2.81787	2.08678	1.93453	1.32879	3.65226	3.55967	3.14809	2.72836
AGA	2.037	2.10465	2.30645	2.66143	2.77925	2.93341	2.51251	2.77174	2.367	2.42971	2.00265	2.53444	2.83262	3.07638	2.80113	2.66841
DCTD	4.41165	4.5468	4.38899	4.46734	3.52032	3.42809	3.47448	3.43386	4.56092	4.50237	4.19454	4.69443	3.46325	3.27749	2.63563	2.8701
WWC2-AS2	-0.43449	-1.00275	-0.53599	-2.09144	-0.70514	-1.50789	-1.3267	-0.21564	0.80983	0.71612	0.58454	-0.72366	-1.76135	-1.30296	-0.979	-1.40523
WWC2	2.11653	1.77096	1.5968	1.73745	1.8344	0.17822	1.92879	0.49165	3.76806	3.16734	3.10622	3.68451	2.81495	2.54269	2.32189	1.99952
CLDN22	0.34795	0.06355	-0.01358	0.62529	-1.29865	-0.92783	-0.79292	-0.06132	0.64894	1.00992	0.00487	1.25129	0.74708	0.50035	-0.69718	-1.05445
CDKN2AIP	3.06006	3.18203	2.97731	3.20375	1.76261	0.80455	1.84564	1.0468	2.25151	2.32157	2.11447	2.19229	1.77122	1.87471	1.08339	1.26356
ING2	4.35058	4.3274	4.57072	4.2631	2.51525	2.42835	2.80928	2.27073	3.44025	2.94674	3.01904	3.13227	1.89698	0.97779	1.99902	1.04626
RWDD4	3.83258	3.51058	3.51867	4.08498	2.09348	2.10833	2.43236	2.21902	2.86432	2.57949	2.05478	3.26529	2.66004	2.75632	1.81423	1.75447
TRAPPC11	2.36436	2.23524	2.23646	2.92244	1.53558	0.78709	1.99804	0.93922	2.4528	2.24047	2.37275	3.07705	1.92031	1.9153	1.84347	1.81158
STOX2	-3.32193	-3.32193	-3.32193	-3.20213	-0.52652	-0.75547	-0.28027	-0.41001	-2.54162	-2.64816	-2.96105	-2.12005	-3.32193	-2.47144	-2.53926	-2.90546
IRF2	4.19828	4.27098	4.26244	4.51507	2.6183	2.33226	2.48805	2.23018	4.47184	4.19613	4.30463	4.45868	2.7682	2.75068	2.05883	2.47109
CASP3	3.96109	4.38086	4.07982	4.89761	2.65981	2.19102	3.20107	2.29522	3.68467	4.04832	3.75441	3.8721	4.65568	4.26318	3.61368	3.85752

PRIMPOL	2.37231	2.53586	2.54215	2.40705	0.70525	0.53852	1.30443	0.54967	2.15291	1.64494	1.97006	1.57175	1.98952	1.59435	1.36518	1.27365
CENPU	3.56661	3.22695	3.14561	3.3153	1.61259	1.42095	2.07778	1.74464	4.00971	2.53034	2.93392	2.75542	4.46266	3.69189	3.5226	3.0544
ACSL1	3.50168	3.55562	3.62097	4.52967	1.59034	0.08616	2.12237	-0.26101	1.56406	-0.13627	0.78652	0.83675	2.54782	0.40541	2.51721	0.53595
SLC25A4	3.50229	3.27579	2.9752	3.29407	1.62634	2.01312	1.51192	1.78031	1.99049	1.34548	1.35418	1.33928	3.08917	2.99182	2.68208	2.4289
CFAP97	2.34534	2.22246	2.18053	2.36489	1.85532	0.71232	2.51554	0.6177	2.82699	2.90927	2.68969	3.43236	2.34987	2.28878	2.4684	2.02135
SNX25	2.91522	2.83174	2.89698	2.93576	1.75563	1.5534	1.70343	1.30154	2.81016	2.73798	2.71391	2.51258	2.60039	1.88563	2.17416	1.77963
LRP2BP	-0.49082	-0.32496	-0.36843	-0.0312	-2.33258	-1.11246	-2.56573	-0.82605	-1.75577	-1.4973	-1.70107	-1.43475	-1.66752	-1.16573	-2.05319	-1.8098
ANKRD37	2.90114	3.29633	3.69663	3.31263	1.06562	3.58038	0.94501	2.85205	2.5096	3.49046	2.4975	2.29013	0.70653	0.82156	0.44755	0.31602
UFSP2	3.41645	3.51197	3.76094	3.79445	1.92974	3.36266	2.11109	2.92635	3.25395	3.64768	3.24293	3.46637	2.12547	2.11319	2.338	1.75195
C4orf47	0.49241	0.3914	0.46356	1.34408	-2.18015	-0.99389	-2.27879	-2.0883	-0.16407	0.50072	0.21345	0.23542	-1.84659	-2.79232	-3.32193	-1.63584
PDLIM3	-2.72664	-2.50276	-2.05172	-2.19619	-1.67433	-2.89509	-1.86345	-2.73095	-3.1858	-3.32193	-3.2	-3.20996	-1.67385	-1.3496	-1.2341	-1.2099
SORBS2	-3.07635	-3.32193	-3.24474	-2.82629	-2.39114	-3.13199	-2.49049	-3.17091	0.81625	1.03593	1.30408	1.73466	-0.2367	0.59335	0.84153	0.41782
TLR3	-0.46734	-0.88768	-1.27049	-0.43219	-3.18699	-3.32193	-3.32193	-3.13633	0.96457	1.19002	1.25329	2.7764	-0.07397	1.1664	0.36758	1.02673
FAM149A	-3.32193	-3.32193	-3.32193	-3.32193	-1.40374	-1.37432	-2.34236	-1.1398	-0.61431	-1.08035	-0.71672	-0.5604	0.61662	2.01713	1.72037	1.8414
RPSAP70	-3.32193	-3.32193	-3.32193	-3.32193	-1.05437	-0.27476	-1.5082	0.76778	-0.94994	-0.18509	-0.41235	-0.52263	1.27641	2.97888	2.41457	2.59954
CYP4V2	1.46419	1.50379	1.62802	1.45287	-0.77597	-0.38811	-1.47974	-0.43425	-0.18131	-0.18705	-0.7767	0.90925	-1.2223	-1.0872	-1.42658	-0.7138
KLKB1	0.3592	-0.53535	0.17317	0.21627	-1.06034	-1.34994	-1.53395	-0.6633	-1.67187	-1.98813	-2.08232	0.13307	-2.30079	-1.81272	-2.73517	-1.71224
F11	-3.02243	-2.9897	-3.32193	-2.95316	0.77143	-0.01973	1.10158	0.61065	-3.32193	-3.32193	-3.32193	-3.32193	-3.32193	-3.32193	-3.32193	-3.32193
F11-AS1	0.65563	0.30613	0.03607	-0.26819	-0.11539	0.31111	-0.01295	0.3832	-3.32193	-3.32193	-3.32193	-3.32193	-3.32193	-3.32193	-3.32193	-3.32193
FAT1	2.96796	2.64377	2.47567	2.28648	5.04462	2.35494	5.66279	2.54472	4.28837	3.62273	4.08055	4.50606	4.67863	3.9143	5.28365	4.83243
TRIML2	1.12871	1.14636	2.01766	0.92519	-3.03961	-2.72302	-3.01447	-3.32193	-2.94811	-2.74588	-3.32193	-1.7711	2.41766	2.01901	1.33886	1.86831
LINC02434	1.18789	0.826	0.67265	2.05389	0.44986	2.31274	0.22664	1.69429	-3.32193	-2.24778	-3.32193	-3.32193	-3.32193	-1.12667	-3.32193	-1.62705
LINC01060	-0.27376	-0.16981	-1.07915	-0.37113	-3.32193	-3.32193	-3.32193	-3.32193	-3.32193	-3.32193	-3.32193	-3.32193	1.03976	1.06127	0.33009	0.60333
FRG1	5.05903	5.14834	5.17106	5.36244	2.31212	3.14313	3.00655	3.28185	4.0243	4.09318	4.36709	4.19632	2.38661	3.05292	2.80779	2.58524
RNA5SP174	2.87481	3.96375	3.97127	2.088	-1.39843	-3.32193	-3.32193	-0.18064	2.2287	3.06095	3.36744	1.94154	-3.32193	-3.32193	-3.32193	-3.32193
DUX4L9	-2.163	-3.32193	-3.32193	-3.32193	-2.98643	-3.32193	-3.32193	-3.32193	1.21493	1.53302	1.47621	0.69549	-3.32193	-3.32193	-3.32193	-3.32193
AGGF1P1	-3.32193	-3.32193	-3.32193	-2.76353	-3.32193	-2.85879	-3.32193	-3.32193	0.35048	-0.34748	-0.25685	-0.18306	-2.65597	-2.20485	-1.37346	-1.49106
RPL23AP84	-1.20245	-1.79715	-3.32193	0.45096	2.699	2.91481	1.54123	3.35705	-1.03254	-1.93625	0.18862	0.88304	0.19954	1.21399	-0.15442	0.1102
PLEKHG4B	-1.48728	-1.49263	-0.75949	-1.16446	-2.47968	-2.01474	-2.64476	-1.98478	0.09716	0.37027	0.38811	0.45043	-1.84559	-1.31989	-0.74732	-0.87819
CCDC127	1.55196	1.57652	1.53882	1.83766	2.22134	1.89052	2.38694	1.76637	1.51228	1.68561	1.52429	2.087	1.46992	1.73774	1.70926	1.8811
SDHA	4.70685	4.68862	4.66513	4.7564	4.62499	4.34478	4.61298	4.22064	4.97539	4.65153	4.78244	4.46563	4.4564	4.80126	4.63979	4.65889
PDCD6	3.43759	3.8705	3.91682	4.14546	3.16649	3.81455	3.48691	3.64031	2.2869	3.67639	3.49566	3.29104	1.96044	3.72556	2.50419	2.70014
AHRR	2.82269	3.18	3.52065	3.87257	1.9115	2.24178	2.18701	1.95428	2.51211	3.44111	3.32583	3.22455	1.89186	2.61929	2.43253	2.70743
EXOC3-AS1	2.07278	2.26737	2.46428	2.84196	1.57242	2.02279	0.91541	2.16519	-0.6383	1.0567	0.63265	0.42094	0.07125	0.9147	1.24639	1.635
EXOC3	4.14226	4.4837	4.54126	4.50211	3.24652	3.29787	3.06522	3.36921	3.5766	3.61439	3.70505	3.95958	2.83091	2.99617	3.06342	3.19572
SLC9A3-AS1	4.71773	5.75984	6.22827	5.7484	3.79456	3.59286	2.17381	3.39035	0.99081	2.42525	2.04854	1.34806	-0.41474	0.02208	-0.91426	1.001
SLC9A3	4.36245	5.58846	6.01773	5.37633	3.45899	3.00925	1.94432	2.58599	0.80999	2.35105	1.93615	1.02144	-0.8971	-0.84379	-0.60133	0.43497
CEP72	3.8459	4.01565	3.63422	3.78242	3.00048	2.11305	2.49707	2.73033	2.44668	2.0955	2.18265	1.59287	1.68927	1.69908	1.86591	1.89661
TPPP	0.02156	0.03675	-0.26378	-0.68818	1.93435	1.06606	1.70663	1.31693	-0.39755	-0.4962	-0.88739	-0.95622	0.97667	1.12605	2.55964	2.2103
ZDHHC11B	-1.85521	-2.39599	-2.22441	-2.8315	0.4579	1.68388	0.12155	2.12209	-2.51824	-1.97352	-1.8225	-1.90391	-2.86052	-2.73628	-2.77665	-1.33162
ZDHHC11	-0.46376	-0.92693	-0.79567	-0.62245	0.83427	1.24396	0.21758	1.51009	-0.88593	-0.44405	-0.33313	-0.48651	-1.86303	-1.65361	-2.2027	-1.38377
BRD9	3.7325	3.50905	3.51297	3.65395	2.96027	3.46095	2.90291	3.60119	3.01484	2.73889	2.97322	2.80312	2.85723	2.7581	2.99091	2.8219
TRIP13	5.37884	4.8567	4.79773	4.44176	4.44094	3.72173	4.24523	3.85196	5.11434	3.45809	3.70748	3.30086	5.02918	4.43488	4.40641	3.60945
NKD2	0.05876	0.31893	1.0315	0.02425	0.59007	1.3024	-0.85725	1.39385	-1.96382	-1.47628	-2.68281	-2.50219	-0.93922	-1.50632	-0.76609	-1.36298
SLC12A7	1.8745	2.24606	1.82454	2.45165	6.14613	5.12258	5.57419	5.17353	1.38827	2.28987	1.82906	2.38943	3.55016	3.65467	4.51554	4.76343
SLC6A19	-3.32193	-3.32193	-3.32193	-3.07618	1.92783	2.02544	1.59682	2.7695	-3.32193	-3.32193	-3.32193	-3.32193	-3.32193	-3.32193	-3.13233	-3.32193
TERT	4.65648	4.39818	4.407	3.75418	1.70638	1.59645	0.47192	2.35603	-1.21009	-1.76336	-0.99823	-1.77956	-0.08463	-0.28297	-0.70441	0.35769
CLPTM1L	5.51469	5.40647	5.33586	5.3088	5.31737	5.359	5.31262	5.27595	5.25017	5.01646	5.40193	4.93328	4.29968	4.38793	4.61064	4.56672
LPCAT1	4.18921	4.33877	4.15486	4.2967	3.81493	3.20337	4.04041	3.22558	4.44802	4.54861	4.40915	4.35471	4.43654	4.32999	4.65684	4.47786
SDHAP3	-1.0087	-1.33258	-1.57706	-1.20387	1.64841	1.86616	1.13743	1.55244	-0.48345	0.24447	0.74545	0.14221	1.53562	1.81105	1.12741	1.52429

MRPL36	5.7958	5.73382	5.43643	5.2312	4.61346	4.67809	3.84195	4.70736	5.52709	5.33123	4.80518	4.12201	4.78779	4.48799	3.78254	3.98698
NDUFS6	7.6728	7.53847	7.46369	6.52568	6.48294	6.88854	5.84594	6.87078	6.98792	6.38376	6.64554	5.25094	6.18429	6.11715	5.49478	5.54735
LINC01019	1.02921	1.2389	0.79772	0.88422	-3.32193	-3.32193	-3.32193	-2.95616	-3.32193	-3.32193	-3.32193	-3.32193	-3.32193	-3.32193	-3.32193	-3.32193
ADAMTS16	1.98264	1.9974	1.99047	1.30625	-3.24302	-3.32193	-3.32193	-3.11098	1.72267	1.88742	1.9781	1.97361	-3.32193	-3.32193	-3.15023	-3.32193
ICE1	3.23697	3.25338	3.2541	3.54505	2.63936	1.77342	3.09707	1.50276	3.52927	3.51938	3.32654	4.4171	3.32803	3.3724	3.60066	3.69748
MED10	5.75089	5.68613	5.63641	5.74337	4.14879	4.64735	4.64572	4.46584	4.19649	4.30493	4.29918	3.75324	4.77594	4.71879	3.73379	3.6261
UBE2QL1	0.14637	0.14919	0.54908	-0.48846	-3.21577	-3.32193	-3.32193	-3.32193	-3.32193	-3.32193	-3.32193	-3.32193	-2.96388	-2.55493	-3.09339	-3.32193
NSUN2	5.64872	5.46112	5.19925	5.3447	4.56202	3.74078	4.7623	3.71447	4.97017	4.43863	4.87594	4.63661	5.11954	5.05738	4.64371	4.59939
SRD5A1	2.07192	1.98197	2.1344	2.3371	1.5834	0.91071	1.70272	0.61903	2.54341	2.1867	2.16253	2.34702	2.31112	1.49827	2.76232	1.91892
TENT4A	3.52556	3.14877	3.35759	2.99719	2.28123	2.20493	1.65707	2.41632	3.58175	3.11246	2.72627	2.99898	2.92444	2.78936	2.14204	2.25952
MTRR	3.37792	3.21461	2.83731	3.17413	3.01423	2.65146	3.17656	2.24655	2.68187	2.79762	2.30029	2.81111	2.57701	2.3339	1.4451	1.81835
FASTKD3	2.8569	2.49027	2.59019	3.02233	1.77516	1.85427	2.08177	1.86956	1.67557	1.65017	1.93158	2.06857	2.10899	2.03844	1.5748	1.37403
MIR4458HG	1.13277	1.20691	1.03534	1.49041	0.111	1.25019	0.00233	0.89982	-0.20212	0.2469	0.01144	-0.12984	0.01558	0.28341	-0.83367	-0.72212
SEMA5A	-1.71167	-2.66558	-1.82407	-1.80448	-3.10347	-3.32193	-3.32193	-3.32193	-2.51808	-1.52504	-1.50405	-0.86229	-3.32193	-3.32193	-3.32193	-3.23432
SNHG18	-2.83876	-2.40125	-2.36395	-1.73276	-3.32193	-3.32193	-3.32193	-3.32193	-0.03129	0.83823	-0.56289	-0.10522	-3.32193	-3.32193	-3.32193	-3.32193
CMBL	3.25293	3.72571	3.42625	4.22263	3.45919	3.02683	3.79435	2.98761	4.08585	5.27897	5.15805	5.84426	3.02979	4.34956	4.25161	4.70462
ROPN1L	0.05619	0.08251	0.33789	0.91031	-2.5348	-1.87435	-2.47599	-2.72924	0.31259	-0.05143	0.04157	-0.20196	-0.90232	-0.81903	-1.92617	-0.54884
ANKRD33B	2.98448	2.96555	2.53029	1.80947	2.0366	-0.63164	2.51618	-0.0189	4.23132	4.3529	4.03393	3.66731	2.86857	2.86962	3.19518	2.34207
DAP	5.21489	5.41124	5.11831	5.13384	4.75496	4.95368	4.55115	4.65557	6.24142	6.02357	5.95052	5.77741	4.53618	4.31941	4.33363	4.4641
LINC01194	1.27678	1.62283	1.11374	1.57539	0.37627	0.92862	-0.1311	0.86936	-3.32193	-3.32193	-3.32193	-3.32193	-3.32193	-3.32193	-3.32193	-3.32193
DNAH5	-1.6588	-1.68269	-1.33716	-1.09321	-2.41095	-1.11406	-2.0546	-1.72874	-2.85381	-2.13799	-2.13997	-2.04151	-0.28039	-0.53868	0.03778	-0.04562
OTULINL	-0.94588	-1.57687	-1.52174	-1.89783	1.30488	-0.60256	1.66739	-1.04334	1.60649	0.59886	0.93782	0.54051	-2.00435	-2.6485	-1.82682	-2.86584
EEF1A1P13	5.02025	4.60709	5.01517	4.0289	3.69359	4.02304	3.45954	3.44181	4.59668	5.92657	4.37606	4.03693	2.86376	2.33026	2.83631	3.11451
OTULIN	2.02624	2.13019	2.04785	1.88083	2.31361	1.26042	2.65113	1.21974	2.04685	1.55634	1.81783	2.11939	2.3518	2.10872	2.17683	2.25097
ANKH	1.80155	1.73601	1.69209	2.04015	2.69736	2.17636	2.43321	1.82762	1.70641	1.84493	1.675	2.67452	-0.01478	0.09045	0.21053	0.57935
FBXL7	-1.09618	-1.43754	-1.69726	-1.04837	-3.22714	-2.60239	-2.51766	-3.32193	-0.03873	-0.05374	-0.45516	-0.35457	-2.99939	-2.62229	-2.07466	-2.25688
ZNF622	6.45177	6.51357	6.20152	6.14216	4.72712	4.4002	4.71128	4.28518	5.59908	5.66054	5.67964	5.67686	4.61724	4.83397	4.91409	5.17826
RETREG1	1.03837	1.19728	1.07922	2.16206	-2.53819	-2.72227	-2.76044	-2.83221	-0.3023	-0.46876	-1.38981	-0.48993	-0.01185	-1.21229	-0.29734	-0.91298
MYO10	-2.40404	-2.36898	-2.42967	-2.74547	-2.98888	-2.90272	-3.11322	-3.14277	4.60072	4.39479	4.39009	4.77419	4.09191	3.99908	5.02865	5.01387
RPS26P28	2.44694	1.63407	2.27976	-0.08217	1.35766	3.50775	2.09207	2.20462	2.66029	3.35161	2.23902	1.61351	-0.03858	1.2408	-3.32193	-0.40943
FTH1P10	5.7926	5.74628	6.08796	5.46736	1.65449	2.91174	1.18978	2.42246	2.30748	3.49645	2.44892	3.98583	0.99713	0.73425	0.18372	1.34664
CDH18	0.75127	0.65252	0.4734	1.26397	-3.22712	-2.91769	-3.32193	-3.0707	2.15376	2.04979	2.24454	2.1344	-3.32193	-3.32193	-3.32193	-3.32193
LINC02241	2.29141	2.25055	2.10474	2.25705	-3.09401	-2.46207	-2.86129	-3.32193	-3.32193	-3.32193	-3.32193	-3.32193	-3.32193	-3.32193	-3.32193	-3.32193
GUSBP1	0.95792	0.74181	0.45506	0.813	-0.24298	0.14797	-0.96495	0.44451	-0.57329	0.22761	-0.43324	-0.00395	-1.01313	-0.63973	-1.00691	-1.38364
HSPD1P1	3.95638	3.03127	3.0722	2.66544	2.94501	3.62409	3.43339	3.49545	2.77451	2.87603	2.71698	2.37093	3.18877	3.19479	3.02294	3.0233
PRDM9	1.4608	1.16639	1.45268	0.54419	-3.01585	-2.86239	-3.09154	-2.5891	-3.32193	-3.32193	-3.32193	-3.32193	-3.32193	-3.32193	-3.32193	-3.32193
C5orf17	1.24422	1.87046	1.87522	1.90858	0.13335	-0.30056	-0.08949	-0.09309	-3.32193	-3.32193	-3.32193	-3.32193	-3.32193	-3.32193	-3.32193	-3.32193
LINC02228	0.72935	1.42677	0.78883	1.50638	0.8879	0.17558	1.01697	-0.07973	-0.00248	1.51406	0.35818	2.93711	0.41153	0.48782	0.03594	1.26206
LINC02211	0.88263	1.56719	0.84992	1.67722	1.06724	0.54095	1.14401	0.34645	0.13047	1.61266	0.4554	2.92599	0.52686	0.61997	0.09916	1.39009
MSNP1	-0.86747	-0.72987	-0.44015	-1.61583	-2.3573	-2.39128	-3.32193	-3.32193	-0.18231	0.12941	0.43655	-0.2825	-1.85972	-2.55672	-1.88293	-0.67865
CDH9	-3.32193	-3.32193	-3.32193	-3.01962	-2.92829	-0.55898	-3.0925	-0.31683	-3.32193	-3.32193	-3.32193	-3.32193	-1.86986	-0.6589	-1.52772	-0.90919
PURPL	-0.11437	0.60402	0.73855	1.4526	-3.00011	-2.40171	-2.07514	-1.68984	1.35739	2.14153	2.31513	3.20481	-1.16574	-0.42842	-0.79205	-0.10065
LINC02103	-3.32193	-3.32193	-3.32193	-3.32193	-3.32193	-3.32193	-3.32193	-3.32193	-3.32193	-3.32193	-3.32193	-3.32193	2.74991	2.64149	1.86476	1.83818
LINC02109	-0.68116	-0.19534	-0.34795	0.27903	-2.05611	0.34526	-0.89606	0.46308	-2.6076	-2.98191	-2.95958	-3.32193	-3.32193	-3.32193	-3.32193	-3.32193
CDH6	-3.32193	-3.32193	-3.0317	-3.10992	-2.43278	-3.23326	-2.20392	-3.21817	-3.32193	-2.39072	-2.98874	-1.00514	-1.04954	0.40977	2.35001	1.50018
DROSHA	3.94607	3.88002	3.60207	3.90913	3.08268	2.51553	3.17027	2.43714	3.93999	3.44918	3.60286	3.85074	3.48313	3.54115	3.83515	3.7239
C5orf22	3.01449	2.99869	3.08696	3.52819	2.2657	1.76196	2.62301	1.59489	3.19878	2.54608	2.99328	3.38572	3.31289	2.77364	2.71482	2.37383
PDZD2	0.77199	0.53379	0.68772	0.86098	-3.04605	-2.83512	-2.9168	-2.01427	-0.90901	-1.07948	-0.58769	-0.63121	-2.28663	-2.55877	-2.04622	-1.79166
GOLPH3	5.38028	5.55804	5.42695	5.87814	4.90787	4.52403	5.50509	4.41819	5.03777	5.03936	5.13653	5.5801	4.80858	5.22365	5.42057	5.2678
MTMR12	3.21442	3.03675	2.75123	3.13642	2.67431	1.61912	2.96986	1.58339	3.8787	3.37452	3.3616	3.81477	3.85009	3.72016	3.83848	3.8859

ZFR	4.10677	4.11585	4.08935	4.49203	4.27153	2.77065	4.73715	2.74532	4.97054	4.8761	4.77035	5.51704	4.44745	4.35509	4.57002	4.56996
SUB1	5.12869	5.42634	5.10881	5.70392	3.16052	3.79813	3.62771	3.75122	4.15114	4.25968	3.9259	4.44836	4.0456	4.30519	3.22996	3.4218
NPR3	-2.54962	-2.10505	-2.17686	-1.29387	-3.32193	-3.32193	-3.32193	-3.32193	-2.31774	-2.33195	-2.74762	-2.64692	-0.69505	-0.49827	-1.14241	-0.64979
ADAMTS12	3.48805	3.31776	3.29207	3.54391	-3.2278	-3.21056	-3.21881	-3.32193	-1.37329	-1.70529	-0.94521	-0.93959	-3.32193	-2.68255	-2.70795	-2.93254
AMACR	1.49936	1.79373	1.62474	2.39621	1.14541	0.97473	1.14496	0.90651	2.29598	2.26849	2.20798	2.67375	1.16127	1.68065	0.50304	1.56033
C1QTNF3-AMAC	1.90666	2.13739	2.03607	2.83578	1.49301	1.42747	1.50051	1.33711	2.57267	2.59059	2.45346	3.12735	1.65736	2.13163	0.92314	1.98144
RAI14	5.22289	5.38554	5.5287	5.75869	2.12226	1.02104	2.67068	1.25925	5.33505	5.28668	5.21314	6.12435	4.27917	3.43971	3.82909	3.67922
RAD1	3.76932	3.63774	3.27918	3.29097	2.84175	2.06035	3.09561	2.14123	3.19639	2.71366	2.66687	2.27861	3.49561	3.67173	3.15644	3.05984
BRIX1	4.75009	4.57572	4.30126	4.46957	3.26579	2.85928	3.6276	2.84279	3.60857	3.1387	3.3009	3.09468	3.91044	3.51616	3.06202	2.52427
DNAJC21	3.14415	2.74978	3.19963	3.32774	2.47556	1.56136	2.75877	1.42024	3.81058	2.5883	3.44586	2.99263	3.08376	1.7003	3.08502	1.81359
PRLR	-2.15624	-2.1869	-2.14392	-1.62487	-2.94147	-2.817	-3.1006	-2.8867	-2.50877	-2.83684	-2.57802	-2.579	-3.32193	-3.32193	-3.32193	-3.24089
SPEF2	-2.09146	-1.99353	-2.0444	-1.44575	-2.39699	-2.49175	-2.29876	-2.3008	-1.55493	-0.72396	-0.93394	-0.22309	-1.50469	-1.34814	-1.56176	-1.50062
IL7R	1.77817	3.28049	1.90947	3.57907	-3.32193	-3.32193	-3.32193	-3.32193	1.09725	2.85565	0.60371	3.0857	-3.18398	-3.05895	-3.32193	-3.32193
LMBRD2	0.77643	1.28563	1.16358	1.75747	1.19661	-0.39286	1.97708	-0.20531	0.64861	1.24263	0.69429	1.6945	0.4474	1.37661	0.48781	1.23175
SKP2	3.96293	3.54018	3.36912	3.39132	3.29134	2.3302	3.14436	2.53052	4.66264	3.23326	4.19119	3.59739	3.77071	3.41249	3.30332	2.80507
NADK2	2.69045	2.46961	2.72523	3.02907	3.19992	2.14442	3.35461	2.13411	2.60488	1.97458	2.26025	2.10507	2.87659	1.35373	2.19849	1.34152
SLC1A3	2.37617	1.83841	2.16617	2.25581	-1.1952	-3.32193	-1.2386	-3.12363	-3.32193	-3.32193	-3.32193	-3.32193	-3.32193	-3.32193	-3.32193	-3.32193
NIPBL	2.28388	2.59898	2.63365	3.27844	2.20702	1.47866	2.73113	1.69871	2.88634	2.97334	2.63589	3.8914	2.61757	2.66625	3.01089	3.11535
CPLANE1	0.31349	0.84534	0.82332	1.3219	0.64182	-0.13065	1.40308	0.38602	0.36876	0.17981	0.4584	1.49339	1.56869	1.17894	2.08704	1.96179
NUP155	4.23485	4.07683	3.8669	3.58317	3.6507	2.31076	3.92642	2.37825	3.89844	3.02951	3.56964	3.37015	3.87644	4.00941	4.27929	4.04062
WDR70	3.02857	3.02357	3.16715	3.31136	0.93173	1.13477	1.31486	1.28355	2.27941	2.4316	2.71479	2.95638	1.08496	1.66184	1.36983	1.55612
GDNF-AS1	0.14563	0.45476	1.22926	0.19156	-3.32193	-3.32193	-3.32193	-3.32193	-2.45612	-2.2263	-2.46247	-2.40029	-3.23571	-3.15491	-3.32193	-3.116
GDNF	1.87186	2.46541	3.23864	1.84208	-3.32193	-3.32193	-3.09598	-3.32193	-0.62389	-0.99182	-1.46027	-0.1929	-2.67191	-2.43383	-2.89236	-1.85074
EGFLAM	0.44466	0.32956	0.51933	0.68374	-3.32193	-2.97235	-3.32193	-3.32193	-2.92664	-2.84451	-2.33407	-2.99185	-3.32193	-3.32193	-3.32193	-2.98321
LIFR	-1.06062	-1.29199	-0.67343	-0.50869	-0.48038	-2.53319	-0.17181	-2.10609	1.37945	1.27586	0.49359	1.81183	-0.92857	-1.49273	-0.67889	-1.57259
OSMR-AS1	0.34717	0.45881	0.53124	0.03528	-2.25919	-2.24402	-2.89766	-1.02304	-2.13467	-1.14181	-1.78729	-2.06559	-0.76659	-1.17428	-1.26977	-0.65258
OSMR	4.64722	4.8688	4.86636	4.94494	2.8161	2.75031	3.0031	2.63808	4.70765	4.64997	3.5539	5.29639	4.27347	4.00479	3.80445	4.30436
RICTOR	2.00024	2.44426	2.55953	3.27621	0.95718	0.10932	1.73524	0.27557	1.81644	1.88152	1.48227	3.05769	2.33487	1.9736	2.24743	2.1821
FYB1	1.5165	1.96825	2.21792	3.3234	-3.17388	-3.14734	-3.16002	-3.32193	-3.12294	-0.98599	-1.88179	0.85818	-2.73852	-1.19199	-2.34241	-1.68466
C9	0.99539	1.6645	1.47489	1.67253	-1.16403	-0.78925	0.20869	-0.9809	2.49396	2.08198	2.73094	1.97805	1.485	1.9929	2.42577	1.96292
DAB2	2.51906	2.77143	2.75204	3.41287	-0.72733	0.98637	0.21516	0.53778	3.20633	2.95455	3.45094	3.58481	2.63374	2.99684	3.13586	2.70241
LINC02104	-3.32193	-3.32193	-1.97992	-3.32193	-3.32193	-3.32193	-3.32193	-3.32193	-1.31756	1.06977	-0.37496	-1.0939	2.19105	3.97054	1.49648	1.96098
TTC33	0.67983	0.85706	0.57383	1.51496	1.26898	-0.33959	1.88359	0.02897	1.37833	1.63013	1.4419	1.74742	1.36633	1.83714	1.37774	1.34644
PTGER4	0.68594	0.59218	0.30739	1.00334	-0.90853	-2.48	-0.89176	-1.80368	2.4408	1.79211	1.45352	1.87437	3.22595	3.04486	1.46282	2.15692
PRKAA1	3.15737	3.35063	3.23523	3.68862	3.22904	2.37339	3.96024	2.29171	3.60348	3.16908	3.45501	4.07553	4.08341	3.34648	3.93838	3.31045
CARD6	1.68041	1.89681	1.65614	2.72812	-1.26982	-1.31024	-1.33483	-1.1984	-2.03769	-2.57301	-2.53025	-1.90371	0.65898	0.1801	0.70741	0.38428
C7	-3.32193	-3.09939	-2.88673	-3.07389	-3.32193	-3.32193	-3.32193	-3.32193	-0.61548	-0.51082	-0.64661	-1.05785	-3.32193	-3.32193	-3.32193	-3.32193
PLCXD3	-3.32193	-3.32193	-3.32193	-3.32193	-3.32193	-3.32193	-3.32193	-3.32193	-3.1627	-1.97205	-2.82011	-1.52516	1.61592	0.44545	1.9259	1.02145
OXCT1	3.35107	3.2774	3.22864	3.4515	-2.05009	-1.70927	-1.62344	-2.25117	3.61761	3.64822	3.85578	3.78855	2.7804	3.7259	3.11881	3.08395
OXCT1-AS1	0.57592	-0.21871	0.1105	-0.06788	-2.60956	-3.32193	-3.32193	-3.32193	1.04677	0.93518	1.28458	0.96551	-0.41931	0.8016	0.58445	0.57893
C5orf51	3.26297	3.54655	3.54447	3.8616	2.8166	2.49794	3.42607	2.0959	2.83322	2.54148	2.61625	3.89234	3.44523	3.3399	3.23405	3.08521
FBXO4	1.14506	1.08622	1.36238	1.2872	0.5953	0.56549	0.26985	0.90798	2.56235	2.38273	1.89837	2.07077	0.01123	0.40731	-0.38873	0.07711
GHR	-3.32193	-3.32193	-3.32193	-3.32193	-3.18477	-3.32193	-3.32193	-3.32193	-0.43157	-1.56588	-1.0314	-0.53545	-3.32193	-3.32193	-3.16864	-3.32193
CCDC152	1.92972	2.09218	1.94558	2.42073	3.33781	3.81734	3.75576	3.44481	-3.32193	-1.38243	-3.32193	-0.70212	3.17784	3.73238	3.13101	3.27837
SELENOP	-0.32936	-0.10934	-0.33077	0.11349	3.29121	3.54873	3.60427	3.18959	-3.10954	-1.68723	-3.13118	-0.8895	2.88982	3.51249	2.89423	3.11468
ANXA2R	1.26873	0.86073	1.31573	1.26277	0.2358	1.18309	0.29094	0.84986	-0.04452	0.76528	-0.43393	0.34686	0.79155	1.22315	0.42465	0.75459
ZNF131	2.29899	2.24923	2.20239	2.40294	1.96096	1.60818	2.13342	1.87859	1.84632	1.94254	1.66375	2.13835	2.22328	1.97625	1.73473	1.44554
NIM1K	-0.38681	-0.46227	-0.44383	-0.41551	-2.07935	-2.42095	-2.47334	-2.13582	-0.74045	-0.90433	-1.06589	-0.86968	-2.58031	-2.44452	-2.63106	-2.28884
HMGCS1	3.43903	3.59314	3.31838	3.98289	4.22753	5.00042	4.80593	5.17418	4.29791	4.07557	4.12846	4.07944	5.1068	6.63322	5.38974	5.86894
CCL28	1.66488	1.88031	2.30964	1.60663	0.8106	1.20345	1.14654	1.05609	-0.51823	-0.42434	-0.7427	0.14839	-0.42444	-0.08358	-0.31417	-0.17323

TMEM267	2.20854	2.14756	1.53364	2.35786	2.4748	1.64686	2.62743	2.22036	1.65838	1.46167	0.63338	1.04058	3.12845	2.6721	1.1755	1.82927
C5orf34	2.18063	1.76476	1.94715	1.64441	0.93077	1.09275	1.23982	1.18698	1.03385	0.4517	0.49408	0.34813	2.59864	2.97582	1.76457	1.73106
EEF1A1P19	3.56527	3.08465	3.77323	1.40721	2.54208	2.66583	2.49995	1.66568	3.28703	4.83172	3.3036	3.25145	0.86066	0.89033	1.68251	1.82889
PAIP1	5.19039	5.18379	5.29679	5.54193	4.93239	4.67293	5.18663	4.44963	4.95976	4.72291	4.82591	4.86449	5.38815	5.14005	5.34001	4.9243
NNT-AS1	1.65221	2.255	1.94823	2.77196	1.34176	0.84274	1.72419	1.21202	1.15386	1.54071	1.44088	1.93068	1.54518	2.3426	1.77014	2.33614
NNT	2.25083	2.70103	2.54127	3.3789	3.66735	2.79359	3.97881	2.8182	3.63715	3.83577	3.72206	4.4196	3.68377	4.01728	4.10683	4.15913
FGF10-AS1	3.10826	3.41042	3.2359	3.46142	-3.32193	-3.32193	-2.85464	-3.32193	-3.32193	-3.32193	-3.32193	-3.32193	-3.32193	-3.32193	-3.32193	-3.32193
MRPS30	4.54077	4.16157	4.01402	4.15967	2.95579	2.36412	3.14744	2.17177	3.36001	2.86394	3.33558	2.92554	3.25119	2.48036	3.21535	2.15694
EMB	1.06563	0.62448	0.6975	0.68674	-0.68868	-0.29248	0.0137	-0.44977	0.06189	0.27554	0.20826	0.89799	-3.32193	-3.32193	-2.96363	-3.32193
PARP8	1.99634	2.05154	2.06284	2.66792	0.60031	0.86755	0.63046	0.79412	1.81106	2.04747	1.85198	2.83416	-2.87537	-2.34814	-3.12359	-1.91214
ISL1	-2.46761	-3.32193	-3.32193	-1.71796	-0.84298	0.34575	-1.50892	0.8461	1.30974	1.47236	1.13246	1.46209	-3.32193	-3.32193	-3.32193	-3.32193
ITGA1	0.22155	0.8048	1.02605	2.22345	1.98992	1.58063	2.45752	1.6904	0.87934	1.38258	0.98826	2.80234	0.74439	1.28869	1.04241	1.47307
PELO	4.73922	4.73554	4.58315	4.72567	3.55357	3.24803	3.65843	3.08841	3.77473	3.84632	3.78843	3.85601	3.14146	3.33467	3.09898	3.09237
ITGA2	4.67703	5.36651	5.43965	6.29566	2.57911	1.85275	4.38034	1.5934	3.02511	4.29906	4.33812	6.48093	4.48986	3.83338	4.69066	5.32948
MOCS2	3.70769	3.91804	3.51174	4.2974	2.83526	2.99044	2.9347	3.10075	3.17492	2.97874	2.63545	3.40318	3.41306	3.30724	2.29165	2.61146
FST	1.68292	2.62609	1.51415	3.07314	5.98777	7.50291	6.62682	7.20069	0.89711	0.36149	-0.52333	-0.04789	-3.32193	-2.82815	-3.32193	-2.99413
NDUFS4	5.31818	5.30407	5.44793	5.55221	4.89776	5.51122	5.16806	5.28958	5.62719	5.70833	5.47035	4.96322	5.23803	5.13265	4.87464	4.68914
ARL15	0.40754	0.75106	1.06055	1.13101	1.4491	1.81994	2.12261	1.44662	1.88269	1.44441	1.51235	2.31704	-0.23	0.61926	0.30088	0.0806
SNX18	2.91955	3.18305	3.16779	3.05616	3.57967	2.32725	3.31381	2.33331	3.11373	3.48001	3.44197	3.26631	1.71791	1.37079	1.95861	1.70195
ESM1	2.67963	2.91968	2.60186	1.69	-2.63348	-3.32193	-2.97844	-3.32193	-2.58253	-2.24413	-2.64686	-3.32193	-1.52211	-2.78625	-2.45414	-2.96462
GPX8	3.67595	3.5947	3.7352	4.16451	1.83181	2.28919	2.64664	1.96149	4.1839	3.4381	4.05709	4.07374	4.47158	3.59674	3.85479	3.13329
MCIDAS	1.97199	1.92895	1.81964	1.34287	1.19609	1.54331	0.18828	1.54866	1.73802	1.19113	1.46107	0.33476	1.65684	1.63849	0.87692	0.90677
CCNO	3.81184	3.51911	3.5964	3.29998	2.90647	4.19526	1.77567	3.72406	2.68557	2.46458	2.43001	1.83071	2.23413	2.20833	1.68397	2.02766
DHX29	3.67318	3.37662	3.20918	4.13317	4.06893	2.99224	4.49702	3.09152	4.06982	3.97884	3.74215	4.49955	3.84237	3.46833	3.42754	3.42697
MTREX	4.87872	4.70941	4.6982	4.91943	4.14392	3.78357	4.80834	3.77299	4.54255	4.1015	4.41156	4.64955	4.1938	3.91157	4.14349	3.76371
PLPP1	4.46734	4.42844	4.6067	4.57978	4.66269	4.90285	4.98584	4.96308	3.9619	3.61783	3.94248	3.76977	3.77317	3.65902	3.76051	3.54023
SLC38A9	2.42233	2.257	2.38609	2.87802	1.70679	1.29161	2.42973	1.66723	1.25722	1.27751	0.90841	1.84897	1.55884	1.658	1.72606	1.6853
IL31RA	2.18387	1.81828	2.34003	2.09599	-1.50588	-3.09359	0.21503	-2.63918	2.95771	2.99104	3.40933	3.84739	2.95733	2.61141	2.87227	2.73788
IL6ST	5.11428	5.43754	5.20134	5.49482	4.63765	2.60786	5.42355	2.90714	5.11127	5.52387	4.96378	6.46565	5.38077	5.06005	5.43651	5.46401
ANKRD55	-1.37114	-2.4133	-1.18599	-1.59847	-3.03351	-3.32193	-3.32193	-2.3736	1.81639	1.2084	0.86973	1.05371	-0.57454	-1.62719	-1.62652	-1.30368
RPL26P19	5.07756	5.10883	5.02852	4.40103	3.17879	4.0671	3.20119	3.64787	4.82361	5.91689	4.74955	4.69873	2.9253	2.78199	2.59006	2.35464
MAP3K1	1.53421	1.51904	1.46331	1.48128	2.20913	-0.06752	2.60872	0.36111	2.25601	2.02743	1.97238	2.86209	2.57409	2.68102	3.25402	2.92586
SETD9	2.07811	2.11821	1.68722	2.02697	1.97452	1.58234	2.14703	1.69274	1.96108	2.08439	1.76212	2.03194	1.54892	1.691	1.04823	0.68583
MIER3	1.90676	1.97798	1.93264	2.50497	2.40887	1.47635	3.1979	1.53763	2.66226	2.20767	2.55136	3.06354	3.07639	2.79577	3.27488	2.61952
GPBP1	4.02797	3.91207	4.0588	4.25657	3.2856	2.39647	4.09148	2.3763	3.53427	3.51508	3.58808	3.87409	3.62325	3.35723	4.00852	3.2332
PDE4D	0.42453	0.74944	0.79502	1.17431	1.9078	0.73869	2.30774	0.85107	-1.54204	-1.41368	-1.52904	-0.65089	1.67058	1.99092	2.53667	2.09912
DEPDC1B	2.75877	2.13848	2.02221	1.93837	3.27351	2.40482	3.42754	2.55994	3.47532	2.80372	2.85603	1.86302	3.88885	3.81687	3.13156	2.89589
ELOVL7	1.59323	2.10033	1.52252	2.8625	3.26753	2.0461	3.94722	2.77354	1.14368	1.30538	1.14302	2.25667	4.01239	4.32478	3.17093	2.75919
ERCC8	1.42976	1.26332	1.29414	1.57066	1.16051	0.88635	1.46493	0.66497	1.32248	1.03247	1.40845	1.35564	1.91669	1.66674	1.66578	1.22312
GNL3LP1	0.68851	-0.24762	-0.37414	0.23069	-0.64007	-0.37959	-0.57085	-0.59542	0.50363	0.01583	0.97052	0.38857	-0.31627	0.314	0.62594	0.09499
NDUFAF2	6.47832	6.1863	5.92735	6.00157	4.84394	5.73924	4.94831	5.71353	5.40641	5.3607	5.24071	4.23554	5.73311	5.3937	4.68226	4.61192
SMIM15	5.12362	5.29199	5.02378	5.51564	4.54552	4.2421	5.2796	4.14878	4.75747	4.60417	4.6411	4.75456	4.45314	4.6746	4.53985	4.30467
SMIM15-AS1	5.01239	4.98766	4.92979	4.51546	5.06507	3.82754	5.31733	3.39744	5.13422	5.04883	5.10187	4.25859	4.06628	4.4608	5.00972	4.75634
LINC02057	2.17632	2.38659	2.2099	1.55444	-2.13754	-3.32193	-1.69065	-3.32193	0.30102	0.32866	0.72434	-0.18244	0.94066	-1.14916	-0.42715	-2.53026
ZSWIM6	3.68892	3.39939	3.79074	3.35535	0.97345	-0.13115	1.3125	0.29199	3.28434	2.51229	3.49208	2.69597	2.73912	1.2307	2.50163	1.41935
KIF2A	3.87765	3.76197	3.87579	3.99121	3.28373	1.8016	4.15948	1.57587	4.44582	3.95661	4.25146	4.73129	4.47068	3.41571	4.8205	3.75211
DIMT1	4.16384	4.05789	3.89404	4.26637	3.37432	3.33899	3.79368	3.33023	3.92063	3.46175	3.84264	3.88093	4.15702	3.79877	3.93105	3.23656
IPO11	2.813	2.38672	2.4013	2.56912	2.68583	1.139	3.31448	1.00714	3.07098	1.90794	2.61967	2.51125	3.05752	2.07798	2.65112	1.86228
CKS1BP3	4.01314	3.65502	3.40261	2.91482	4.02089	3.96918	3.82388	3.29831	4.7616	3.83899	3.58178	3.68126	4.36628	4.04098	3.50535	3.27608
SREK1IP1	3.04897	3.29576	3.22644	3.42581	2.2529	2.63978	2.84481	2.8463	2.45895	2.24102	2.19882	2.78336	2.59974	2.36408	2.30367	1.94043

CWC27	3.95786	4.06441	4.01383	4.34327	3.05862	3.06467	3.57484	3.19899	3.82892	3.69455	3.67865	3.86193	3.3407	3.36312	3.44337	3.07089
ADAMTS6	3.01298	2.97797	2.65372	2.02855	-1.32153	-0.77234	-0.69937	-0.15621	2.62667	2.34717	2.02164	2.13276	1.13339	-0.38688	0.06046	0.08901
CENPK	2.75695	2.67421	2.65656	2.67159	1.90842	0.82721	2.35714	1.47303	2.29013	1.42552	1.75774	1.15891	3.15136	3.12783	2.16417	1.65016
PPWD1	3.5446	3.50234	3.41158	3.72247	2.84877	2.67698	3.21532	2.58848	3.30638	3.50524	3.38187	3.72356	2.78186	3.00725	2.46331	2.64528
TRIM23	1.925	2.44998	2.09421	3.15133	1.82468	0.92272	2.42623	1.33324	2.3641	2.92387	2.2274	3.06968	2.06979	2.16953	2.06522	2.31284
TRAPPC13	2.1137	2.15782	2.18939	2.85131	1.50838	1.34367	2.07768	1.18518	0.85991	1.09661	1.22378	1.50319	1.60064	1.57916	1.28315	0.8886
SHLD3	2.26381	2.27677	2.04484	2.41633	1.53744	1.09606	2.16658	0.9154	1.41806	1.25483	1.01725	0.83657	1.48204	1.55487	1.24108	1.31669
SGTB	3.1077	3.08421	3.04024	3.25447	1.78857	1.35465	2.47442	0.92502	2.85933	1.90021	2.54126	2.13779	3.34931	1.76519	3.18967	1.49281
NLN	2.66575	2.51339	2.38261	2.89327	2.45346	1.63378	2.79266	1.56893	3.4747	3.13676	3.0331	3.41203	3.55278	3.42668	3.81315	3.54948
ERBIN	3.81778	3.86497	3.76942	4.28766	3.0096	2.18992	3.86107	2.0897	5.42296	4.96779	5.14301	5.1162	4.21385	3.56952	4.10948	3.58752
SREK1	2.63663	2.56254	2.66008	3.42146	1.60417	1.99297	2.10508	2.31873	2.25366	2.11174	2.31475	2.56184	2.12881	2.16497	2.27044	2.16907
MAST4	1.66574	1.99786	2.02811	2.07932	-1.48073	-2.43007	-1.3721	-2.22953	-1.55746	-1.47355	-0.92318	-0.87245	-1.2266	-1.07187	-0.81199	-0.05604
PIK3R1	1.71637	1.81985	1.72354	1.86387	0.68641	0.1065	1.29967	0.06088	1.80461	1.60749	1.77525	2.67663	0.63875	1.09559	1.29091	1.86882
SLC30A5	4.10054	4.08987	4.01967	4.53322	4.45123	3.76832	4.85466	3.66044	3.59064	3.28963	3.45918	3.72132	3.16887	2.95553	3.24456	2.84482
CCNB1	6.97815	6.49629	6.55443	6.29823	6.37137	5.40715	6.50798	5.49048	7.0559	5.89678	6.67731	5.48443	6.74713	6.39748	6.42707	5.78819
CENPH	4.05892	4.14132	4.04711	3.91836	2.32793	3.09805	2.70589	2.89987	2.4874	2.6938	2.86823	2.46205	2.13132	3.55296	2.82121	2.21924
MRPS36	4.7676	4.89824	4.71712	5.17605	4.10844	4.81004	4.32442	4.80606	3.89149	3.99979	3.91425	3.30188	4.37056	4.41449	3.46539	3.44514
CDK7	5.2211	5.09632	5.13396	5.3133	3.66283	3.35983	4.00204	3.13941	4.21303	4.25935	4.07659	3.91487	4.60053	3.92567	3.84725	3.22947
CCDC125	1.75193	1.58465	1.4187	1.88537	1.57523	1.23003	1.95991	1.15688	0.66894	0.64384	0.37416	1.14118	1.36512	1.18708	1.10784	1.18717
AK6	5.65517	5.62343	5.47989	5.79032	5.18953	4.85213	5.6781	4.87519	5.50245	5.13755	5.1679	4.33231	5.35924	4.92569	4.76973	4.53953
TAF9	6.06264	6.05654	5.83766	6.21263	5.34463	5.12411	5.69799	5.03428	6.14311	5.6923	5.61716	5.63828	6.02361	5.52038	5.32544	5.05538
RAD17	4.06894	4.11413	4.03119	4.24743	3.76943	3.15563	4.38533	3.16645	4.14851	3.99255	3.85435	3.83673	4.04596	3.96078	3.77925	3.43888
MARVELD2	-0.44649	-0.27022	-0.02574	0.13774	2.86006	1.3958	3.249	1.80424	-0.65715	-1.35728	-1.27968	-0.9007	1.31236	0.89258	2.00115	0.83632
OCLN	2.01344	1.87539	2.05879	2.22183	3.66541	2.39003	4.1626	2.67371	1.4552	0.92088	0.52986	1.4679	2.72881	2.45803	2.90228	1.82339
GTF2H2C	4.22331	3.93253	3.79983	4.0136	3.03525	2.68034	3.29618	2.90878	3.11187	2.64555	2.95636	2.84074	3.22734	3.12094	2.69658	2.51163
GUSBP3	0.82929	1.73395	1.20125	2.68939	2.14527	1.36877	1.07888	2.60484	1.493	1.55072	2.08968	2.26665	2.86358	3.11355	1.95368	3.25812
CDH12P2	-3.32193	-1.34989	-0.47974	1.61309	-0.02196	2.55905	0.6403	2.91353	0.93838	-1.50986	0.53507	2.40187	-0.12634	1.45578	1.063	2.22491
SERF1B	3.92215	3.71367	3.4842	3.51145	2.6507	3.07168	2.32632	3.03716	3.67636	3.68433	3.55431	2.71963	3.9171	4.08402	2.88956	3.43617
SMN2	5.91317	5.76564	5.74257	5.6462	3.50788	3.3957	3.90626	2.96826	4.66603	4.43961	4.60718	4.2783	4.46671	3.85067	3.94785	3.17065
CDH12P3	-3.32193	-1.339	-0.46717	0.8161	-0.00884	2.5734	0.11413	2.80412	0.70402	-0.71929	0.54866	2.27464	-0.11333	1.46985	0.43929	2.15853
GTF2H2B	4.60326	4.3204	4.24638	4.46525	3.42114	2.95088	3.74322	3.14887	3.49246	3.03349	3.33659	3.22997	3.53498	3.57297	2.9584	2.89673
CDH12P1	-3.32193	-1.339	-0.46717	1.62721	-0.00884	2.5734	0.65396	2.92793	0.95221	-1.49941	0.79517	2.41619	-0.11333	1.46985	1.0769	2.2392
SERF1A	3.80656	3.59845	3.36689	3.39767	2.51072	2.96197	2.13766	2.91587	3.55384	3.56197	3.42582	2.51081	3.81881	3.96449	2.78444	3.3568
SMN1	6.05387	5.90448	5.86375	5.84281	3.74918	3.82401	4.14686	3.50679	4.86502	4.59797	4.8382	4.68294	4.74209	4.13824	4.17733	3.3948
NAIP	-0.55797	-0.12817	-0.08788	0.60531	-1.90878	-0.33478	-1.7466	0.17454	-0.92483	-0.40931	-0.62291	0.47676	-0.65087	-0.04018	-0.37728	0.22263
GTF2H2	3.71029	3.40633	3.23669	3.55512	2.06495	1.5909	2.36977	1.88193	2.55102	2.03956	2.40062	2.35147	2.56994	2.49333	1.97317	1.79842
OCLNP1	1.67184	1.10616	0.78661	1.35222	3.06224	1.3374	2.83631	1.96595	0.64291	0.61626	-0.10644	0.95762	0.69806	0.98099	1.1907	0.40816
GUSBP9	1.3174	2.32839	2.27005	2.61817	1.77824	2.55529	1.29689	3.23666	0.84737	2.2368	2.1717	2.78728	2.54259	1.05043	1.81798	1.89968
BDP1	2.00246	2.03307	1.81996	2.88204	2.58372	1.01761	3.07462	1.23215	2.20542	2.21713	1.88796	2.93755	2.63497	2.65913	2.73172	2.91501
MCCC2	4.29653	4.16891	3.88951	4.29527	3.86393	3.88962	4.23771	3.87123	3.61001	3.61969	3.59918	4.30511	4.24364	4.62469	4.08937	4.26523
MAP1B	0.20265	0.22537	0.39593	0.68254	-1.70756	-0.28504	-1.01221	-0.50094	5.2777	4.54513	4.90508	5.49847	-3.18931	-2.80416	-3.01779	-2.87853
MRPS27	4.37474	4.04114	3.94034	4.13634	3.46071	2.84047	3.5963	2.85712	4.27907	4.24945	4.34882	4.8379	4.1872	4.06825	4.00093	3.61814
PTCD2	0.93098	0.83395	0.78592	1.06314	0.06977	-0.18891	0.31978	-0.29291	0.64795	0.73907	0.9198	1.20067	0.71439	0.3394	0.57378	0.08781
LINC02056	5.23545	5.07235	5.29343	4.91476	-3.32193	-3.32193	-3.32193	-3.32193	-2.24958	-1.8212	-3.32193	-3.32193	-3.32193	-3.32193	-3.32193	-3.32193
TNPO1	4.97502	4.97322	4.92935	5.37423	3.9912	2.64738	4.66068	2.79422	5.06404	4.69459	4.67556	5.27209	5.49342	5.02373	5.02979	4.84117
FCHO2	2.28181	2.3193	2.2486	2.95898	1.87029	0.93044	2.95863	0.55685	2.67516	2.28571	2.56074	3.22443	2.70723	2.00215	2.06202	1.91035
TMEM171	5.59653	5.90348	5.48885	5.70202	-1.87944	-3.32193	-3.32193	-2.64261	3.58862	2.80212	3.30667	1.76864	1.72619	-0.30318	1.85758	0.56711
FOXD1	-2.19524	-2.58708	-2.05758	-1.6254	-1.61618	-1.03234	-1.03897	-0.67139	8.07304	7.43992	8.22499	5.42495	2.42901	2.52004	2.50215	2.52594
FOXD1-AS1	-1.82088	-1.71098	-1.65808	-3.32193	-1.58544	-1.81059	-0.707	-0.90121	8.20142	7.41903	8.05092	7.29912	3.79356	3.74299	3.40407	3.17496
ANKRA2	2.32299	2.36324	2.69985	3.37067	1.36698	1.81695	1.5792	1.59275	1.65694	2.47519	1.875	2.66756	1.64083	1.68779	1.07483	1.17937

UTP15	3.17808	3.05788	2.69408	3.21353	2.47233	1.9116	2.83536	1.96239	2.34913	2.2051	1.95994	2.50771	2.54426	2.50063	1.93654	1.8901
ARHGEF28	1.78435	1.92956	1.74164	1.54755	1.70752	0.51759	2.10219	0.40445	2.01834	1.93493	1.65198	1.89463	4.24901	4.30999	4.12805	4.26583
ENC1	3.61618	3.40783	3.46676	3.1507	-0.87694	-2.01536	-0.40458	-2.24942	5.05287	4.40116	5.19465	4.78823	4.12296	3.62955	4.98691	4.49059
HEXB	6.13574	6.10361	6.35107	6.27799	6.05781	6.0242	6.02548	5.88887	5.95637	5.69518	5.98234	5.84074	5.07466	4.84546	5.11079	4.83743
GFM2	5.08338	4.99445	4.88483	5.07637	4.29285	4.34422	4.44724	4.3318	4.94962	4.91565	4.82852	4.68831	4.5756	4.41905	3.84455	3.9917
NSA2	5.46012	5.28749	5.1389	5.43509	4.25491	4.36648	4.63078	4.22096	5.09239	5.05472	5.0053	4.9341	4.81375	4.03137	4.31267	3.91104
FAM169A	0.73231	0.66515	0.30953	1.10881	2.81525	1.97108	3.44673	1.8795	2.20908	2.12982	1.94504	2.69873	2.1225	2.17503	2.04958	2.15023
RNU6-1330P	-0.25297	1.77339	-0.03549	3.08714	3.95255	3.59187	5.05391	3.15246	3.60032	4.04385	2.63826	4.98334	4.07838	3.74108	4.3118	3.86061
GCNT4	-3.13322	-3.32193	-3.10091	-3.08754	2.17679	1.84958	3.22871	1.74031	-2.34039	-2.46713	-1.47164	-1.21362	1.19506	2.16871	2.31317	1.70049
HMGCR	2.94816	3.32332	3.01598	3.79967	4.67807	4.76531	5.49638	4.92602	4.17088	4.18389	4.11318	4.34987	5.4134	6.21974	6.22052	6.00068
POLK	1.77226	2.05541	1.98834	2.65495	1.23376	0.37626	1.93799	0.35173	2.11131	2.17966	2.05421	2.90261	2.25134	1.81296	1.89276	1.64135
POC5	3.21472	3.04622	2.80876	3.23578	0.95269	0.99201	1.25714	1.37288	1.90179	1.68957	2.03119	1.92281	2.13842	2.36754	1.26274	1.33128
HMGN2P4	1.56164	1.72289	2.36962	2.9662	-1.16469	-0.47817	-1.058	0.84481	2.20126	1.86227	1.14422	-0.5578	1.01083	0.45645	-0.18778	0.9177
IQGAP2	-2.1351	-1.07484	-1.53827	-0.2451	3.96858	1.42826	4.49428	1.52003	-3.166	-2.96121	-3.05476	-2.23988	-3.21501	-3.21537	-3.19264	-3.06897
F2RL2	-1.61555	-2.3103	-3.32193	-1.60566	2.22221	-0.15046	2.57857	0.26756	-0.50409	-0.78545	-1.00403	0.17624	-2.70655	-2.16047	-2.60095	-2.24853
F2R	2.409	2.60347	3.21081	3.33975	3.11719	2.44006	4.21958	1.56591	3.84462	5.02462	4.15682	5.4409	2.88706	3.21396	3.40809	4.12758
F2RL1	4.2866	3.90136	4.62937	4.99988	5.66746	4.3501	6.76578	3.38695	-0.02262	0.18568	-1.2305	-0.62768	1.02306	0.0877	1.35331	1.2961
AGGF1	3.22736	3.41578	3.45999	3.88975	3.13305	2.46924	3.53465	2.46676	3.66783	3.94543	3.71203	4.59913	3.09806	3.50936	3.47856	3.48879
ZBED3	-0.73596	-1.39276	-1.61772	-2.087	1.43886	0.60549	0.84929	0.94719	0.97792	0.82044	0.98266	0.72021	1.34269	0.53471	2.18518	1.07029
ZBED3-AS1	-1.92586	-2.67709	-2.29266	-3.10972	-0.82673	-1.91599	-1.22406	-1.92013	-1.19899	-1.14872	-1.23416	-1.42007	-0.61993	-1.34385	-0.39755	-0.73161
PDE8B	-1.67871	-1.67455	-1.00797	-0.78655	-2.2572	-1.22527	-1.7969	-1.39908	-0.85021	-1.10022	-1.64389	-0.59604	-1.20844	-0.45758	-0.95407	-0.73282
WDR41	2.03705	1.90718	1.955	2.11373	2.26179	1.88461	2.6719	1.58416	2.95629	2.83363	2.45186	3.1056	3.21729	3.40143	2.91471	3.13836
RPL7P23	3.24045	3.14506	3.40091	1.81155	2.1164	2.60592	2.21977	2.46921	2.85305	4.21994	2.68936	3.16423	2.08788	1.77627	1.91468	2.34146
TBCA	6.47295	6.58818	6.49481	6.63876	5.13824	5.71354	5.48862	5.79973	5.72214	5.94378	5.72648	5.33631	5.55961	5.72629	4.31625	4.30851
ACTBP2	3.57508	3.98613	4.32166	0.81367	1.64305	1.72872	-1.0378	1.6887	3.64979	3.02926	4.19824	0.11471	-0.41557	-1.43536	-0.83164	0.60056
AP3B1	3.62423	3.7758	3.9326	4.1202	3.58005	2.84929	4.21131	2.75598	4.23996	3.75726	4.05008	4.59489	4.02044	4.13925	4.18541	3.93737
SCAMP1-AS1	1.99926	1.80852	1.94324	2.10768	0.49158	1.01917	0.43644	0.80607	0.44207	1.42727	0.17879	0.17178	1.2674	1.81343	0.01394	0.5799
SCAMP1	3.74418	4.02143	4.16123	4.17631	3.20706	1.99575	4.10171	1.5727	4.11637	4.11824	4.07098	4.72784	3.89049	3.55777	4.38417	3.85829
LHFPL2	3.76499	4.37735	4.43509	4.76071	3.06781	2.23985	3.83744	2.13911	2.82789	3.35511	3.27879	4.22384	3.23955	3.71933	4.32376	4.34508
ARSB	3.01211	2.60837	3.09233	2.94577	3.63354	3.37303	3.78398	2.89128	2.90278	1.07671	2.59956	1.51252	2.36675	0.9029	2.7655	1.43977
DMGDH	0.43896	0.88043	0.96723	1.34648	1.1345	0.76393	1.24802	0.68797	-2.89603	-3.1298	-3.32193	-2.81592	-0.27041	0.04832	-0.23873	0.35847
BHMT2	6.56214	6.40698	6.32191	6.60357	5.44205	5.77281	5.51782	5.56997	-2.6739	-3.01718	-2.73183	-2.77354	4.16417	3.91867	4.01509	3.86511
BHMT	-0.94606	-1.42545	-1.02345	-0.54647	-0.88831	-1.2026	-1.15206	-1.57306	-3.32193	-3.32193	-3.32193	-3.32193	-1.62317	-1.78892	-2.61554	-1.66553
JMY	1.36292	1.51368	1.2982	1.8349	1.22322	0.19084	1.35148	0.43909	1.63835	1.90696	1.90181	2.66104	1.17542	1.09104	1.47498	1.64333
HOMER1	2.6478	2.61593	2.33074	2.75254	2.35731	1.76663	2.60972	1.54601	1.19171	1.36107	1.02403	1.26033	1.33945	1.01421	1.11839	1.1802
TENT2	3.13421	3.2204	3.22985	3.69609	2.26596	2.15689	2.86424	2.13438	3.30661	3.27895	3.28521	3.89342	3.08572	3.26361	3.0705	2.84982
MTX3	1.75894	1.1315	1.56434	1.53468	1.36602	0.87561	1.93539	0.8407	0.75196	0.8645	0.69007	1.22076	2.20679	1.51752	1.287	1.32281
SERINC5	1.36829	1.34455	1.08902	1.86487	3.34005	1.60194	3.49681	1.83306	1.98729	2.35854	2.06456	2.85339	1.26302	0.99031	1.46654	1.82484
HNRNPA1P12	1.66401	1.78825	2.10767	2.13161	-1.06703	0.11086	-0.67184	-1.36521	0.17966	1.28472	0.43825	0.77214	-1.06002	-0.72651	-0.64442	-1.26795
ZFYVE16	1.76751	1.82812	2.04243	2.8194	2.55602	1.1388	3.2168	1.38571	1.94068	1.94421	1.70036	2.74956	2.6399	2.28811	2.32402	2.20794
FAM151B	-0.56882	0.03512	-0.80289	-0.51711	-0.36116	-0.35571	-0.05562	-0.19761	-0.08699	-0.01351	-0.69615	0.09799	-0.38657	-0.06273	-0.06186	-0.18515
DHFR	5.88737	5.53028	5.45187	5.51482	4.08776	3.16691	4.19554	3.45503	5.60449	4.39203	4.75086	4.23957	6.06101	5.48813	5.64866	5.01923
MTRNR2L2	3.02305	4.33097	3.67611	0.29417	1.81142	5.06492	2.34945	5.25921	1.68293	4.03954	1.92471	3.35733	2.67582	2.67075	2.78948	4.61328
MSH3	3.154	3.30809	3.27901	3.68185	2.12991	1.5605	2.7866	1.91297	3.54961	3.31063	3.33948	4.0853	2.53784	2.61082	2.68034	2.66094
RASGRF2	-1.7944	-2.02144	-1.6689	-1.52765	-2.83667	-2.59162	-2.31187	-2.18055	-2.90324	-2.61838	-2.86238	-2.5103	0.10548	-1.15038	0.40471	-0.01382
CKMT2-AS1	1.50468	1.74508	1.49696	2.25302	1.07816	0.43577	0.61941	0.41782	-0.13141	0.00812	-0.2426	-0.25362	0.77606	0.7311	0.3158	0.45156
ZCCHC9	3.49077	3.56568	3.71963	4.01027	3.33957	3.18824	3.455	3.27214	2.66995	2.54518	2.78784	2.56015	1.9791	1.15517	1.2087	0.81108
ACOT12	-3.32193	-3.32193	-3.32193	-3.32193	1.17499	-0.86378	1.32253	-0.58518	-2.5243	-3.32193	-3.32193	-3.32193	-1.86696	-3.00243	-3.32193	-1.65777
SSBP2	-1.31245	-1.18548	-0.80435	-0.60888	-0.99103	-1.54205	-1.22623	-1.96897	-0.02999	0.45081	-0.16666	0.89569	0.78853	0.59657	0.53227	0.48071
PPIAP11	4.09612	4.07778	4.14011	2.34154	2.81834	4.41073	2.77815	3.70266	4.05041	4.47663	3.37495	3.00176	2.85865	2.25398	1.89037	2.84135

ATP6AP1L	1.55096	1.85923	1.58736	1.9802	-2.18916	-1.86383	-2.46862	-1.83716	-2.51182	-1.31447	-1.96206	-0.91569	-2.37267	-1.80837	-2.03278	-1.95389
TMEM167A	4.68025	4.8961	4.63725	5.18398	4.56396	4.16407	5.02106	4.15066	4.37829	4.74829	3.85139	4.7697	4.75002	5.74053	4.21096	5.41013
XRCC4	2.57498	2.59866	2.38397	2.67751	1.47142	1.10614	2.30626	1.31049	2.00381	2.41412	2.18482	2.52705	1.57805	2.82701	1.78683	1.98156
VCAN	-2.28868	-2.20109	-2.28352	-1.43107	-3.08583	-3.32193	-2.7015	-3.32193	-0.9784	-1.15781	-1.01233	0.68983	5.20488	5.64774	6.34518	6.37066
VCAN-AS1	-3.32193	-1.74343	-2.2874	-3.32193	-2.8616	-3.32193	-2.45319	-3.32193	-0.70292	-0.06888	-0.43101	1.71721	6.14038	6.71762	7.10933	7.07308
EDIL3	-0.80236	-0.66347	-0.49628	0.98424	-3.24134	-3.137	-2.61592	-3.32193	0.31928	1.9276	0.8887	3.33651	5.16956	5.46991	6.18316	5.94694
COX7C	8.02086	8.1094	8.01766	7.79194	7.15901	7.88473	6.52859	7.83486	7.25786	7.62935	7.40887	6.55655	6.92654	6.88327	5.74122	5.73204
LINC01949	-3.32193	-3.32193	-3.32193	-3.32193	-0.83478	-0.45401	-0.64712	0.04723	-3.32193	-3.32193	-3.32193	-3.32193	-3.32193	-3.32193	-3.32193	-3.32193
RASA1	2.92151	3.01054	3.22676	3.4395	2.99551	1.94471	3.45181	1.87344	4.42729	4.50196	4.7512	5.45586	4.29113	4.01553	4.72094	4.15532
CCNH	4.52881	4.46002	4.15693	4.48593	2.51508	2.59557	2.95687	2.57619	3.90192	3.77474	3.39758	3.81972	3.75072	3.44509	2.81414	2.61202
TMEM161B	1.69487	1.65099	1.7677	1.97756	1.32818	0.94351	1.95945	1.00313	1.09735	1.5965	0.98289	1.87967	1.27795	1.64615	0.49821	0.68455
TMEM161B-AS1	1.00416	0.35328	0.97398	0.65885	-0.16078	0.87375	-0.26116	0.59847	-0.49475	-0.93927	-0.44628	-1.38921	-0.25211	-0.37934	-1.32022	-0.66904
MEF2C	-2.32889	-2.79122	-2.69194	-1.90671	-3.24321	-3.32193	-3.23565	-2.84409	-2.11738	-1.60295	-1.47429	-0.73521	-3.32193	-3.32193	-3.32193	-3.14706
CETN3	3.71904	3.15487	3.71756	3.28579	2.36797	1.87275	2.80339	2.02948	2.71743	1.39509	2.57293	0.53629	2.6964	0.76513	1.48329	-0.1445
MBLAC2	0.77695	0.96868	0.75494	1.49062	-0.05097	-0.36321	-0.14543	0.03848	1.10454	1.78623	1.01896	1.85315	0.8761	1.51296	0.36514	0.83294
POLR3G	3.64138	2.91352	2.50649	2.79202	2.57631	2.28796	2.92919	2.33951	4.64477	4.12099	4.09799	4.30196	4.20448	2.60878	2.3651	1.75662
LYSMD3	2.87251	3.37247	3.28549	3.84624	3.13033	2.68604	3.96346	2.49781	3.02361	3.86806	3.19687	4.09555	2.1844	2.7064	2.56065	2.41456
ADGRV1	-2.10102	-1.50467	-1.46998	-1.65624	-1.47699	-2.35753	-1.0046	-2.37483	-1.80234	-1.14613	-1.55599	-1.87909	-2.55544	-2.37221	-2.08415	-2.28455
LUCAT1	3.63467	4.10169	4.73345	4.2242	0.94669	1.59376	1.38938	1.02791	0.61258	1.86026	0.5698	2.04559	2.05726	1.11467	1.14918	1.63194
ARRDC3	3.09011	4.84485	5.17559	5.42334	1.25789	2.16146	2.12855	1.84747	2.40399	4.94703	2.69772	5.83677	3.93496	4.54124	4.94755	5.19994
NR2F1-AS1	1.03588	1.84268	1.39886	1.44738	-2.8528	-2.63497	-3.19252	-2.26799	0.26117	0.5239	0.10966	0.52988	0.77123	1.79034	2.02976	2.07742
NR2F1	1.98594	2.38455	2.48381	2.82715	-1.61025	-0.0884	-2.17974	0.43565	1.99524	2.5229	1.83353	2.75975	3.28597	4.18698	5.01068	4.98333
FAM172A	0.84709	1.49273	1.40961	1.85457	0.39564	0.22372	0.98972	0.18603	2.16804	2.12606	2.12599	2.50004	1.91421	1.77964	2.0523	1.75952
MIR2277	3.65742	3.09258	2.81165	3.78616	3.04473	0.83799	2.47325	2.02518	4.48612	4.55075	5.2532	4.06298	4.09582	4.47574	4.63227	3.62246
NPM1P27	6.12441	5.83252	5.54313	5.52787	4.7392	4.3723	4.64751	4.13435	5.57611	5.36834	5.38717	4.84906	4.93082	3.71076	4.6648	3.64504
KIAA0825	-2.34383	-1.73231	-1.7639	-0.80866	-3.19092	-3.32193	-2.89089	-3.19927	-2.56171	-2.21687	-2.49585	-1.64724	-2.5065	-2.3291	-2.17729	-1.82617
SLF1	2.33242	2.06969	2.17883	2.88131	0.99285	0.36788	1.66231	0.74872	1.6268	1.00445	1.04143	1.03976	1.96336	2.0956	1.43238	1.37755
MCTP1	1.71561	1.75338	1.47369	2.1702	-2.41695	-2.29436	-2.35193	-2.02864	1.06005	0.95967	0.72795	1.32426	2.20684	1.94815	1.59928	1.68145
TTC37	3.59334	3.60244	3.61435	4.04388	3.596	2.88794	4.22043	2.89217	4.20383	4.68113	4.19475	5.17053	3.59244	3.66773	3.37368	3.3464
ARSK	1.94189	1.86946	1.61478	2.28375	1.63358	0.81171	1.9864	1.16339	2.27708	2.26346	1.95037	2.43861	2.03662	2.03159	1.69748	1.85252
RFESD	-0.79761	-0.65863	-0.5927	-0.55498	-1.55244	-0.94743	-1.60389	-1.1633	-1.59213	-0.76635	-1.15383	-1.25233	-1.5554	-0.94643	-2.10938	-1.62401
SPATA9	-0.06504	-0.47434	-0.07329	0.23941	-1.14629	-0.94692	-1.03923	-0.83713	-1.36277	-0.336	-0.8426	-1.10024	-0.96866	-0.46385	-1.45954	-1.15114
RHOBTB3	4.29131	4.08778	4.1728	4.66431	1.7443	1.9839	1.87168	2.14962	3.81576	3.86258	3.57799	4.53697	3.99887	3.99251	3.48309	3.63876
GLRX	3.98676	4.46372	4.25572	4.67229	2.50241	3.86485	2.72026	3.31177	3.24978	3.96045	3.0211	3.54277	2.47168	2.03016	1.38124	1.91745
ELL2	4.83264	5.0242	5.25197	5.21681	3.51087	3.29777	4.29537	2.96523	4.77443	4.96341	4.8645	5.25524	3.1691	2.05067	2.48826	2.05723
PCSK1	-0.93206	-0.34115	-0.43234	0.29417	-1.04368	-2.10112	-0.30426	-1.96269	-3.32193	-3.32193	-3.32193	-3.13462	-3.32193	-2.89701	-3.32193	-3.32193
CAST	4.53414	4.66784	4.86221	5.10044	3.24678	2.53103	3.66479	2.32674	5.28846	4.47374	4.81248	5.63266	4.54811	2.84824	4.53029	3.47809
ERAP1	4.47418	4.45412	4.6515	4.994	3.22461	2.287	3.55725	2.13336	4.57753	4.32766	4.71421	5.47709	3.64185	2.56522	3.65829	2.92645
ERAP2	4.51375	4.59109	4.6382	5.1723	1.7823	1.49427	2.31912	1.73178	4.99315	5.16458	5.56141	6.41873	2.8337	2.82364	3.21493	3.17458
LNPEP	1.81828	2.06153	1.95475	2.36125	1.70534	1.00137	2.3157	1.32926	1.61103	2.41969	2.12457	3.60961	2.66023	3.03216	2.64832	3.27873
LIX1-AS1	1.54281	1.72534	1.46815	2.08847	1.85985	0.2021	2.28327	-0.70169	2.44182	2.75206	2.88932	1.44691	1.64068	1.50439	1.4349	1.7988
RIOK2	3.02762	3.05885	2.63223	3.35006	2.08805	1.75717	2.60045	1.54533	3.07823	3.27849	2.99839	3.55428	2.75075	2.87907	2.01172	2.47994
RGMB	3.71484	3.93914	3.31743	3.18404	1.40578	1.30809	1.23809	1.51983	4.39186	4.54091	4.57841	4.50595	4.66895	5.9345	4.1695	5.42108
RGMB-AS1	1.89107	2.56918	1.90617	1.15818	-0.82608	0.09876	-1.97406	-0.80443	3.07923	3.37588	3.59757	1.93934	3.45875	4.57303	3.12603	4.48094
CHD1	3.43406	3.44298	3.23451	3.76546	2.80097	2.0644	3.53886	2.36467	3.34376	3.0222	3.16334	3.76327	3.61226	3.62015	3.77269	3.67554
FAM174A	4.35682	4.30644	4.56112	4.50188	3.72817	3.79691	3.78781	3.28518	2.82946	2.94691	2.92505	2.08978	2.21775	0.55503	2.11729	0.85727
ST8SIA4	0.18349	0.25741	0.24532	1.39328	-3.32193	-3.32193	-3.32193	-3.32193	0.52873	1.39782	0.0832	2.26904	0.41425	0.03569	-1.08686	0.27239
LINC00491	0.57684	0.604	0.80623	1.40884	0.58201	-0.10253	0.81001	0.00405	-3.32193	-3.32193	-3.32193	-3.32193	-3.32193	-3.32193	-3.32193	-3.32193
PAM	3.55046	3.91722	4.38106	3.88946	3.55345	3.18367	4.0063	2.81121	4.36231	4.43339	4.28308	4.96918	4.19332	3.66	4.30513	3.55493
GIN1	0.83902	0.67787	0.48256	1.15833	-0.20998	-0.4755	0.33419	-0.43413	0.18326	0.45482	-0.05118	0.92184	0.41775	0.63111	0.38392	-0.07218

PPIP5K2	1.81556	2.02672	1.73168	2.60668	1.50436	0.37297	1.80994	0.39914	1.86873	1.91312	1.34719	2.442	2.83765	2.51517	1.97354	1.98844
C5orf30	3.03802	2.95722	2.87607	3.14279	2.37067	2.02884	2.88147	1.95508	2.22539	2.40081	2.46157	2.75274	2.49529	2.56252	2.21021	1.65843
NUDT12	1.36203	1.75653	1.67704	2.51672	1.60084	1.23927	2.16776	1.2856	-0.6327	0.17112	0.30977	0.61914	1.5276	1.06964	1.27753	0.83943
EFNA5	2.30194	2.4042	2.40808	2.87098	2.03549	2.17092	2.439	2.22676	2.05844	2.09972	2.02703	2.82366	2.93718	3.25627	3.66271	4.33011
FBXL17	1.00065	1.77554	1.53368	1.67222	1.3507	0.64003	1.8461	0.91362	1.26204	2.09203	1.7677	2.09272	0.06063	0.57959	0.85962	1.178
FER	0.33031	0.74337	0.58311	0.78181	0.44257	-0.4689	1.02268	0.1153	1.71401	1.6931	1.59333	2.71213	2.0627	2.1789	2.31682	2.21575
PJA2	3.83116	4.49346	4.48058	5.06573	3.95102	2.9967	4.71187	2.7327	4.40457	5.16329	4.70507	5.92029	3.60025	4.33066	4.56807	4.62942
MAN2A1	1.16022	1.49602	1.20802	1.62543	3.64268	2.33002	3.95161	2.56389	4.08269	3.91949	3.5852	4.25076	4.37124	4.27199	3.85484	4.0992
SLC25A46	2.81239	3.16105	3.07289	3.42094	2.55147	2.01475	3.0839	1.83883	3.28214	3.22254	3.11944	3.49726	2.88402	2.61351	2.76376	2.60843
WDR36	2.84317	2.70177	2.56092	2.70583	2.64342	1.972	3.27304	1.74654	3.61037	3.56445	3.34343	3.86046	3.54981	3.18928	2.56484	2.61696
CAMK4	0.11371	0.19642	0.06387	0.43699	-3.22285	-3.24275	-3.17897	-3.22923	2.09648	1.67037	1.99639	2.37824	-2.03357	-2.67463	-2.3519	-2.25913
STARD4	1.8017	2.30538	2.1225	2.78122	1.57636	2.02227	2.00465	1.93937	3.38298	3.62589	2.86508	4.18249	3.09065	3.587	2.30732	2.66055
STARD4-AS1	-0.96488	-0.12407	-0.07901	0.8343	0.56322	0.64071	0.95201	0.49342	2.07234	3.05607	2.57331	4.38055	0.6653	1.57827	1.01137	1.62804
NREP	-1.0518	-0.53999	-0.28826	0.67858	0.6784	0.60439	1.00628	0.52076	1.95883	3.32528	3.05869	4.77716	0.26369	1.51817	0.86564	1.66276
EPB41L4A	-0.46592	-0.64842	-0.91984	-0.28763	-1.77575	-0.75205	-1.89522	-0.72366	-1.8432	-1.27249	-1.28925	-1.14719	1.75415	2.45962	2.98443	2.42458
EPB41L4A-AS1	3.07987	2.85035	2.79632	2.60338	1.77478	2.55403	1.53343	2.55325	2.34024	3.76981	3.32624	3.00771	2.44031	2.70751	2.58892	2.27987
EPB41L4A-DT	-3.32193	-3.32193	-2.10947	-3.32193	-3.32193	-3.32193	-3.32193	-3.32193	-2.60277	-3.32193	-3.32193	-3.32193	0.84693	1.90521	2.64555	2.25274
APC	0.87533	1.1426	0.91175	1.62159	1.64796	0.34856	2.35778	0.32216	2.5124	2.17884	2.40323	3.39036	1.83604	1.51676	2.32431	2.31873
SRP19	5.00113	5.12581	4.97997	5.27579	3.48233	4.16689	3.87359	3.97498	3.5246	3.33702	3.5175	3.45398	3.76041	3.59366	2.81988	2.68619
REEP5	5.79661	5.55499	5.65411	5.96952	5.36993	5.76797	5.54911	5.6454	5.31586	5.01588	4.99459	5.76517	5.602	5.96592	5.46286	5.71497
ZRSR2P1	1.89472	1.54189	1.56459	2.16745	0.21901	0.29362	-0.25816	0.56506	0.2369	-0.07406	0.14954	0.54926	-0.21219	-0.04521	-0.22945	0.30075
DCP2	3.05669	2.8444	3.10139	3.19663	1.7652	1.19179	2.74799	0.96742	3.81365	2.9171	3.63007	3.63523	3.9443	3.54752	4.18901	3.77943
MCC	-0.38748	-0.29802	-0.48182	0.165	-2.97023	-3.22025	-3.2278	-2.89581	-1.5418	-1.32382	-1.83873	-0.56676	-2.43539	-2.67686	-3.05066	-2.8864
YTHDC2	1.87433	1.87813	1.69841	2.24978	2.23788	1.13536	2.70712	1.61025	1.9304	1.87867	1.81534	2.55967	1.99441	2.27135	1.92582	2.05222
TRIM36	1.88481	2.31429	2.34019	2.70645	-1.79223	-1.29306	-1.97673	-1.16061	0.7975	-0.0634	-0.14832	0.7036	2.10014	1.98022	1.89701	2.05362
PGGT1B	1.72602	1.80092	1.79169	2.37348	1.09794	1.18119	1.8788	1.2478	1.43271	0.85037	1.29205	1.46526	1.58787	0.99736	1.73013	1.07681
CCDC112	3.83136	3.61242	3.90697	4.18918	1.31918	1.43328	2.15033	1.95942	2.68001	2.2455	2.44072	2.70987	2.31094	1.40169	2.36994	0.95772
FEM1C	2.38156	2.77402	2.94273	3.33242	2.02405	0.96233	2.82031	1.04834	3.24317	3.4338	3.07418	4.07246	2.66954	2.51998	2.96027	2.74748
TMED7-TICAM2	4.51574	4.5468	4.69894	4.70411	5.07992	2.88421	5.53995	2.16442	4.88626	4.62129	5.00667	5.23336	3.64146	3.23455	3.75668	3.20825
TICAM2	2.28127	2.45172	2.75722	3.0293	1.0291	0.95488	2.07353	0.09426	2.29601	1.76515	2.2952	2.24778	0.59048	0.67277	1.37284	0.65158
TMED7	5.98645	6.12393	6.16379	6.51229	6.00726	5.01268	6.78717	4.70761	5.88072	5.76999	5.88484	6.45754	5.25195	4.65605	4.9437	4.45566
CDO1	-3.32193	-3.32193	-3.32193	-3.32193	1.3445	1.19329	1.27304	0.96756	-3.32193	-3.32193	-3.32193	-3.32193	-2.9683	-2.44982	-2.9013	-2.89325
ATG12	2.92785	3.50483	3.24379	3.63254	2.38654	2.39883	2.60668	2.57478	2.25061	2.93447	1.80042	2.81497	2.71325	2.92957	1.64449	2.21208
AP3S1	5.46111	5.61055	5.6738	6.01121	4.71703	4.53543	5.19268	4.42943	5.25164	5.14531	5.00471	5.32171	4.71698	4.27555	4.00582	3.6613
LVRN	0.00081	0.26626	-0.10258	0.00092	-0.87027	-0.63497	-0.02284	-0.23245	-2.97495	-3.02849	-3.32193	-3.17023	-3.32193	-3.32193	-3.32193	-3.32193
ARL14EPL	-0.58273	-0.73542	-0.67015	-0.38558	-2.72104	-3.32193	-3.32193	-3.32193	-1.79319	-0.62721	-1.50618	0.60627	-3.32193	-3.32193	-3.32193	-3.32193
COMMD10	3.36534	3.19943	3.13666	3.09253	2.25765	2.17848	2.95317	1.78003	3.23259	2.53861	2.55473	2.36356	3.91445	3.13015	3.15566	2.36207
SEMA6A	-2.38013	-2.54322	-2.37843	-2.47005	3.72536	3.42826	3.62059	3.50869	-2.10938	-2.16515	-2.06231	-1.6366	-2.79187	-2.79346	-3.13889	-2.39692
SEMA6A-AS1	-2.45559	-2.37799	-2.34003	-3.32193	3.68986	2.82014	2.48032	3.01695	-2.35354	-2.70925	-2.22647	-1.82596	-3.04106	-1.88188	-3.32193	-2.70327
SEMA6A-AS2	-3.32193	-3.32193	-3.32193	-3.32193	2.83669	-0.7651	3.03851	-0.56798	-3.32193	-1.96989	-3.32193	-1.9848	-3.32193	-3.32193	-3.32193	-0.80742
DTWD2	0.05848	0.84647	0.54026	0.66491	0.81256	-0.39053	1.33139	-0.64422	1.0246	0.82344	1.21972	1.6886	1.56451	0.88386	1.80838	1.11566
PTMAP2	5.98804	6.08813	5.74217	5.57714	4.11579	3.4461	4.08216	3.1459	5.28537	4.59285	5.00287	3.53296	4.66609	3.07684	4.05678	2.86397
MIR1244-2	8.83203	8.31664	8.25339	7.98692	7.06488	7.40912	7.57756	7.31964	8.89353	8.27626	8.35482	7.8692	8.80933	7.35791	7.99241	7.20958
DMXL1	0.97567	1.17956	1.2378	1.96196	1.31489	0.08127	2.10241	0.19653	1.36342	1.32606	1.38057	2.12599	1.8056	1.60653	1.98033	1.78873
TNFAIP8	1.57179	1.77196	1.93348	2.3492	-0.30149	-1.17256	0.69445	-1.39926	0.63963	0.21567	-0.02883	0.54382	1.27896	1.28902	1.03348	0.58279
HSD17B4	4.39199	4.27593	4.39469	4.80499	3.69051	3.60408	3.94295	3.38256	3.88622	3.51123	3.92335	4.07174	3.12934	3.08849	3.28151	3.29392
PRR16	-1.71415	-1.77824	-1.54536	-0.26454	-3.32193	-3.32193	-3.32193	-3.32193	1.50454	1.50165	1.97031	1.20173	2.88223	1.94737	2.06116	0.81993
SRFBP1	2.96511	3.078	3.52834	3.42135	2.49334	1.01222	3.25619	0.81696	5.18838	5.62763	4.97019	5.18599	2.92326	1.37451	2.77415	1.71776
LOX	1.68713	3.28196	4.02929	3.47761	-1.51631	-1.01128	-1.32052	-1.82082	6.34755	7.0595	6.03245	7.03053	2.12928	1.72735	1.89537	2.05967
SNX2	4.75643	4.72099	4.67105	4.96422	4.12763	3.81015	4.41296	3.65279	4.97371	4.98789	4.91956	4.91539	4.0736	3.98918	3.85158	3.74987

SNX24	3.5141	3.2625	3.65058	3.57774	3.44853	2.77905	3.71144	2.54764	3.66437	3.19285	3.44067	3.65744	3.74689	2.79926	2.85572	2.16832
PPIC	4.17031	4.46639	4.46605	4.9679	4.68422	4.99176	4.88196	4.89085	4.65848	4.60417	4.69865	4.71682	3.94779	4.14697	3.23639	3.36687
CEP120	1.83175	1.57358	1.86879	2.25056	1.41315	0.65712	2.07536	0.65841	2.54453	2.11297	2.20576	2.85892	2.36721	2.47028	2.05715	2.10063
KRT8P33	-0.12325	-0.37073	0.00666	-2.07275	0.98278	0.71727	0.56032	0.54825	1.89282	1.91899	2.09798	0.72346	-0.06706	0.11005	0.19089	-0.54483
CSNK1G3	3.20103	3.28828	3.35771	3.71491	2.86508	1.9334	3.41057	1.73593	3.92787	3.88156	3.1841	4.26527	3.6652	3.4947	3.15671	3.29198
ZNF608	-3.03003	-2.73371	-2.98203	-2.22948	0.94755	0.78335	1.55914	0.79937	-2.84582	-2.80341	-2.89062	-2.81079	1.79622	2.17637	3.10789	3.12287
GRAMD2B	0.99276	0.61491	0.92767	1.46335	0.01952	1.00785	0.67054	1.0775	2.75744	2.70394	3.20491	3.24058	2.62459	1.99073	2.99071	2.05263
ALDH7A1	2.48047	2.24612	2.31211	2.46598	2.38341	2.9156	2.52613	2.95498	3.36683	2.90625	3.09395	2.91368	2.56472	2.74303	2.84069	2.67259
PHAX	4.67905	4.68795	4.54391	4.87486	3.98662	3.41607	4.44772	3.29331	4.45053	4.08999	4.23761	4.16205	4.37718	4.09845	4.44079	4.04714
LMNB1	5.83264	5.42937	5.41345	4.89427	4.76077	3.60063	4.74852	3.86877	6.85877	5.44516	6.22523	4.69269	5.6647	5.69529	5.62281	5.15731
C5orf63	-1.47767	-1.40102	-1.39083	-1.22754	-1.37994	-0.5831	-1.27804	-0.38152	-0.16737	-0.48573	-0.19829	-0.23954	-0.86722	-0.64544	-1.48338	-1.02987
MEGF10	-3.09254	-3.18874	-3.18169	-3.32193	-3.32193	-3.32193	-3.32193	-3.32193	-3.05851	-2.26521	-2.88144	-1.56964	-0.39296	0.23421	1.1307	1.37241
PRRC1	2.91639	3.0858	3.05782	3.45749	3.27754	3.07972	3.70402	3.11014	3.59256	3.62779	3.67133	4.22222	3.20946	3.47367	3.44294	3.50956
LINC01184	1.79157	2.13275	1.98364	2.09366	1.38906	2.06733	1.78236	2.06993	0.68461	0.84029	0.54179	0.63872	1.24304	1.56499	1.2724	1.40834
SLC12A2	1.59033	1.40527	1.49808	1.42317	6.11917	3.7635	6.48496	3.59358	1.54032	0.31493	1.40098	0.95253	1.2375	1.62156	2.84969	2.32619
FBN2	-1.052	-0.97345	-0.66758	-0.55823	-2.7616	-3.32193	-2.85277	-3.13301	4.78317	3.84721	4.66106	4.65894	-3.32193	-3.32193	-3.16837	-3.32193
SLC27A6	-3.32193	-3.0007	-2.9848	-2.67946	-3.0725	-3.32193	-2.82132	-3.32193	1.4915	0.59848	1.46992	1.15539	-2.89095	-3.32193	-3.32193	-3.32193
ISOC1	4.3662	4.75233	4.46854	4.88478	4.46507	4.68847	4.94294	4.65674	3.29555	3.71582	4.23198	3.80538	2.26342	3.64728	2.86411	2.96181
CHSY3	-2.33248	-1.63841	-2.37037	-1.86691	-2.99872	-2.83801	-2.68789	-2.55364	0.96759	1.30778	1.44007	0.33157	-3.32193	-3.32193	-3.32193	-3.32193
HINT1	7.6985	7.88603	7.78725	7.80338	6.93483	7.06238	6.77453	6.92423	7.12481	7.29543	6.87401	6.41905	7.13398	7.08893	5.95665	5.98904
LYRM7	3.15558	3.10042	2.51901	3.073	1.62494	1.63914	2.06872	1.87228	2.50289	2.16089	2.10109	2.81307	2.82166	2.4315	2.31528	2.26978
CDC42SE2	3.10273	3.24566	3.15002	3.6562	3.39333	3.2348	3.97031	3.01778	3.70971	3.62643	3.14657	4.31636	4.26886	4.80007	4.10315	4.44547
RAPGEF6	1.05748	0.97925	0.8301	1.1233	1.33551	-0.19117	1.92803	0.04173	1.92205	1.64491	1.55947	2.35233	2.35693	1.80216	1.98424	1.59485
FNIP1	2.81324	2.92647	3.11944	3.38208	2.43629	1.57779	3.0548	1.67437	2.44628	2.61834	2.68646	3.51826	3.6228	3.03859	3.87974	3.39683
ACSL6	-3.01325	-3.0579	-2.43603	-3.11956	-1.64218	-3.01018	-1.96645	-2.67345	-2.82016	-2.83384	-2.93354	-2.43588	-3.25605	-3.25627	-3.24203	-3.01998
CSF2	6.51755	6.32822	4.72817	5.19777	-2.44405	-1.73976	-2.38042	-1.14595	5.59228	5.65825	4.86869	5.04475	-1.97415	-3.32193	-3.32193	-3.32193
P4HA2-AS1	5.18069	5.47024	6.10235	5.39313	3.72915	4.29629	3.80757	4.10971	5.33321	5.22789	4.93423	5.54158	3.29442	2.69036	3.11553	2.39109
P4HA2	4.54597	4.78716	5.40111	4.54498	3.86636	3.23038	3.87825	2.76767	5.34346	5.16665	5.18529	5.1548	2.93452	2.12481	3.23527	2.33969
PDLIM4	0.03415	-0.45861	-0.14151	-1.6672	-2.82311	-3.32193	-3.32193	-2.95545	4.32982	4.71256	4.46034	4.07531	-3.32193	-3.32193	-3.32193	-3.32193
SLC22A4	3.09761	3.41041	3.00649	3.63036	-0.32378	-0.89459	-0.7082	-1.06562	2.46909	3.09831	2.65343	3.8191	1.28089	1.2351	1.23909	1.92263
MIR3936HG	1.74841	1.96767	1.65876	1.94037	1.10678	0.58572	1.0899	0.98115	1.28489	1.8096	1.50068	2.42743	1.19665	1.34411	1.22066	1.54104
SLC22A5	0.22425	0.24259	0.14618	0.11196	1.35704	0.90093	1.26647	1.06014	1.38092	1.35833	1.19406	1.61768	1.015	1.56177	0.98784	1.42981
IRF1	3.3478	3.07406	3.26165	3.35943	1.20023	1.12617	1.08117	1.04604	5.20944	5.02351	5.77639	5.27441	2.4985	2.56905	2.60994	2.39949
RAD50	3.95952	4.18573	4.08275	5.01963	3.11018	2.85591	3.64093	3.3397	3.57282	3.62615	3.58577	4.74667	3.27826	3.34711	3.77505	3.90969
TH2LCRR	4.74461	4.93362	4.82907	5.5999	3.40038	3.6115	3.86551	4.31555	3.96554	3.82317	3.84602	4.65232	3.68573	3.56923	4.2649	3.94897
KIF3A	2.44663	2.37279	2.51841	3.25193	1.48563	1.5028	1.88387	1.56209	1.98778	2.18624	1.65946	2.83508	2.66446	2.96863	2.16875	2.68151
CCNI2	-0.88413	-0.44685	0.36989	-0.41368	-3.32193	-3.32193	-3.32193	-2.65846	-1.27288	-1.78911	-1.30355	-2.37277	-2.49885	-2.8539	-1.22403	-2.03672
SOWAHA	-2.53523	-2.69658	-2.95864	-2.93773	0.8264	-0.76423	0.53975	-1.47899	-1.89663	-2.55705	-1.41338	-3.32193	-1.31124	-2.67479	-0.25418	-1.67906
SHROOM1	3.2943	3.43845	3.35488	3.0611	3.53877	4.01023	2.71683	4.1065	3.17627	3.06985	3.43484	2.32499	1.65084	1.33698	2.28131	1.65541
GDF9	5.74078	5.96345	5.91958	5.70546	4.92592	5.23636	4.66788	5.18542	4.81712	4.83141	4.74692	2.92068	5.28348	5.27284	4.22833	4.24398
UQCRQ	7.69088	7.8574	7.74428	7.5597	6.64442	7.27181	6.25801	7.24783	6.6605	6.74383	6.52995	5.63615	6.9414	6.90191	5.685	5.82654
LEAP2	-0.6982	-0.90332	-1.30095	-0.86741	0.63488	-0.02772	0.1297	0.95785	-1.19233	-1.07878	-1.90426	-1.81829	-0.50069	-0.61214	-1.40622	-1.95757
AFF4	2.88945	3.18078	3.24558	3.69427	3.32235	1.86496	3.84163	1.91104	3.55274	3.32349	3.222	4.28071	4.4331	3.76656	4.77367	4.45126
ZCCHC10	2.94154	2.99957	2.87935	3.55328	2.64251	2.87517	3.00993	2.88497	2.78002	2.49998	2.54059	2.63119	3.49105	3.29035	2.57971	2.4004
HSPA4	6.75832	6.76912	6.55038	6.99577	6.36424	5.98442	6.85013	5.93221	6.16573	5.76527	6.07823	6.46499	6.24806	6.12635	6.35086	6.32666
C5orf15	5.28549	5.19261	5.4083	6.03014	4.88314	4.12112	5.16489	4.22788	5.32164	5.07684	5.17729	5.46924	5.56214	5.13735	4.52261	4.13848
TCF7	-0.04167	0.22005	0.15606	0.59625	0.83483	0.3356	-0.81616	0.54367	-1.93585	-1.59704	-2.47475	-1.9797	-1.49909	-1.8333	-2.15084	-1.21369
SKP1	4.07977	4.09174	4.12469	4.54888	3.25381	2.83003	3.51097	2.49119	4.04089	3.93615	3.7898	4.34536	4.1601	3.91247	2.99943	2.99562
PPP2CA	5.58009	5.46253	5.31045	5.63778	4.64742	4.5348	5.23596	4.42261	5.60834	5.198	5.28911	5.50279	5.35947	5.4921	5.22012	5.04669
MIR3661	9.06421	8.76405	8.76785	8.6185	8.94491	7.53646	9.41746	7.24822	9.73224	9.27221	9.40617	8.48388	9.21481	9.37785	9.30791	9.17198

CDKL3	-0.21482	0.01703	0.20506	0.21121	-1.09972	-0.48183	-0.74258	-0.278	-2.40713	-0.26109	-1.01593	-0.46242	0.02139	-0.13198	-1.54627	-0.68899
UBE2B	4.62628	4.82359	4.71892	5.16412	3.75609	4.04273	4.24531	3.81731	4.11849	4.07944	3.53993	3.9752	3.99066	4.16652	3.15806	3.31469
CDKN2AIPNL	4.92403	4.67997	4.60467	4.24399	4.6894	4.54376	4.58587	4.34911	5.65293	5.62347	5.19804	5.05596	5.03728	5.45026	4.59835	4.74093
LINC01843	-3.32193	-3.32193	-3.32193	-3.32193	2.51795	1.36795	1.60643	1.42497	0.64789	1.13476	-0.408	-0.66266	0.50409	0.1179	-1.20194	-0.62739
JADE2	1.99342	2.05557	1.91732	2.0659	2.78548	1.68454	2.31121	1.69614	4.49509	3.9426	4.40983	4.19365	3.29488	3.28162	3.73379	3.87626
SAR1B	2.63285	2.47067	2.27963	2.82373	3.53462	3.37256	3.893	3.37591	2.83339	2.51388	2.6086	2.99743	3.00859	2.91007	2.88354	2.64354
SEC24A	2.81357	2.9556	2.88078	3.36331	3.60589	2.35717	4.24593	2.17718	3.76872	3.79507	3.59991	4.72189	3.23213	2.12622	3.02079	2.39623
CAMLG	4.73271	4.80466	4.74208	4.8154	4.10533	3.83471	4.07046	4.02029	5.15052	5.68805	5.18479	5.25161	4.59479	4.45259	4.14697	4.08867
DDX46	4.99681	4.93598	4.89155	5.40077	4.2145	3.77137	4.66115	4.18095	4.61972	3.88011	4.41418	4.24068	4.8136	4.54984	4.76439	4.42148
C5orf24	3.00398	3.19514	3.29363	3.8118	2.46016	1.63557	3.12623	1.65769	3.49273	3.36378	3.18888	4.14328	4.46444	3.83523	4.11529	3.84808
TXNDC15	2.76707	3.0657	3.00831	3.10819	3.66064	4.62859	3.85454	4.53769	3.41807	3.6466	3.83814	4.18119	3.2445	3.41466	3.41734	3.3785
PCBD2	0.11756	-0.01918	-0.50912	-0.05774	0.19337	0.01187	-0.03072	0.07396	0.35901	0.16709	-0.25155	0.67626	0.78909	0.24057	-0.31713	0.01195
MTCYBP18	-0.27353	0.04674	-0.25404	-1.46465	-1.29024	0.44423	-1.35495	1.49504	-0.77826	-0.7347	-3.32193	-0.5333	-1.928	-3.32193	-0.3321	1.2207
MTND6P4	3.37283	3.04914	3.12618	1.54372	2.79072	5.80793	2.81543	5.99866	2.25284	3.19102	2.14166	3.41566	1.7491	2.28058	2.17011	3.1852
MTND5P11	1.39348	1.05038	1.4535	0.24053	0.31735	3.02001	-0.43498	2.97153	0.13189	0.01245	-0.66096	1.90859	-0.57679	-0.44361	-0.25826	0.71944
MTND4P12	3.79795	3.42332	3.84026	2.00483	3.47396	7.15378	3.44432	7.33326	4.28967	4.85058	3.62096	4.65915	1.86097	1.80237	2.2412	3.14493
PITX1	4.55247	4.37455	4.37412	3.86623	2.07774	0.54929	1.16732	0.41764	4.31172	4.5245	4.79151	3.55219	2.44659	2.67385	3.44982	3.29069
C5orf66	4.0973	3.97525	3.99509	4.03261	3.39493	3.28578	3.47006	3.0801	3.84648	3.37193	3.77883	3.21976	3.3445	3.25931	3.13793	3.20699
TGFBI	7.33597	7.1787	7.64613	7.08681	5.35104	6.07336	5.41665	6.11316	2.44269	3.34237	2.53941	4.12072	5.46356	4.29924	5.85455	4.91887
SMAD5	2.91022	2.77558	2.7325	2.947	2.27116	1.62549	2.99506	1.55537	3.4326	3.67544	3.51692	4.16129	2.96251	3.52369	3.58309	3.29489
SPOCK1	2.15893	3.70295	3.88649	3.60806	-2.56602	-3.15057	-1.92681	-3.12235	0.24784	1.45114	0.99967	2.09966	1.25503	1.98406	1.99366	2.72518
KLHL3	-3.32193	-2.87045	-3.32193	-3.00236	-2.11596	-1.02747	-2.38328	-0.32036	-3.32193	-3.2336	-3.32193	-3.07557	-2.72458	-2.34292	-2.51447	-1.90984
HNRNPA0	5.36927	5.20701	5.21853	4.59266	5.24316	5.05088	4.83721	5.18238	5.39095	5.21236	5.37658	4.49098	4.35724	4.85164	4.47883	4.58832
PKD2L2	0.07144	0.05114	0.38453	0.76581	0.35957	-0.89303	0.72856	-0.60933	-0.72716	-0.27963	-0.61497	0.53014	0.53203	0.03491	-0.34963	-0.54375
FAM13B	1.79204	1.93972	2.03959	2.79709	2.2191	0.83418	2.94646	1.12971	1.15925	1.59128	1.42565	2.82185	2.37595	1.88918	1.47945	1.70465
BRD8	3.35373	3.07603	3.02248	3.44132	3.05406	2.74986	2.83239	2.91236	3.20615	3.09137	3.12354	3.35459	2.73705	2.6454	2.35217	2.6007
KIF20A	4.6497	4.04219	4.03635	3.46485	4.60592	3.74238	4.79858	3.76349	5.52655	3.90144	4.80896	3.81166	4.7275	4.46776	4.87587	4.15074
CDC23	3.51657	3.42733	3.34412	3.40917	3.97167	3.02156	4.27301	2.99566	3.93898	3.63799	3.94358	4.03972	4.13845	4.09508	3.81681	3.64374
CDC25C	2.71025	2.06636	2.04037	1.79756	3.0755	1.97721	3.09031	2.02461	3.28569	2.153	2.60014	1.72751	2.77081	2.33092	2.33945	2.16183
FAM53C	3.24461	3.06653	3.02609	3.09332	3.8527	2.71502	3.38069	2.834	3.29062	3.26778	3.25077	3.2482	3.11917	3.29674	3.31732	3.52181
KDM3B	3.04443	2.9762	3.0207	3.25579	4.10677	2.57411	4.02504	2.75614	4.44854	4.22241	4.01418	4.70228	3.89149	4.08447	4.1397	4.50612
REEP2	-0.00279	0.37148	0.47296	0.84847	0.14238	1.13932	-0.31127	1.4554	1.39357	1.41966	1.05873	0.77662	3.12871	2.85595	3.89743	3.74247
EGR1	-0.29114	0.46151	-0.74147	0.13637	3.14739	3.78437	3.31252	2.68365	2.66413	3.44363	2.62582	2.50597	1.78749	1.97191	2.63598	2.55892
ETF1	6.00262	6.00851	5.8083	6.22007	5.99929	4.92634	6.54306	4.7498	6.08705	5.35503	5.65313	5.90505	6.15831	5.63645	5.95365	5.53316
SNORD63B	3.96555	3.78545	3.98756	5.13307	3.923	4.17283	2.87669	5.40259	0.47379	1.72573	1.26133	1.13892	2.98506	3.39044	2.1158	-3.32193
SIL1	3.97765	3.71967	3.69859	3.56079	5.15487	5.66203	4.96443	5.55472	3.88405	3.96054	4.24355	3.7907	3.0533	2.50432	3.24564	3.13832
SNHG4	3.91942	3.22837	3.53079	2.27232	3.10531	2.6997	3.03337	3.0935	3.08479	3.15718	3.403	3.43248	2.45071	1.84469	2.1022	1.34376
MATR3	4.76979	4.63351	4.52878	5.00244	4.53576	3.59087	5.05629	3.61537	5.42343	5.47804	5.16182	5.96739	5.20177	5.00303	5.01109	4.89362
PAIP2	5.67236	5.88719	5.8834	6.19551	4.80574	4.71509	5.27117	4.42019	5.60221	5.41972	5.53718	5.59059	4.69059	4.75374	4.52804	4.175
SLC23A1	-0.96598	-1.53628	-1.87704	-0.62014	2.42189	2.84896	1.91233	3.14285	-2.67733	-2.76875	-1.73305	-1.02766	-3.07117	-3.32193	-2.37678	-2.54845
PROB1	-0.03083	-0.20867	0.45898	-1.443	0.43819	0.81115	-0.85566	1.4012	-0.31381	1.17794	0.89382	-0.26431	-1.8465	-1.17451	0.11604	-0.08489
SPATA24	1.86608	2.44733	2.44516	2.24442	1.8052	2.18013	1.79477	2.11001	1.66541	2.11334	1.60324	1.57151	1.4993	1.41489	1.00568	1.7799
DNAJC18	1.03321	1.73567	1.34916	1.76621	0.42504	0.51418	0.66229	0.73331	1.77882	2.18065	2.00781	2.75876	0.19894	0.69561	0.70296	1.16628
ECSCR	4.5028	4.46934	4.48534	4.70255	-2.35444	-3.32193	-3.32193	-1.4439	0.73892	0.98555	0.97407	0.9622	-2.20095	-3.32193	-3.32193	-3.32193
UBE2D2	5.83674	5.72497	5.77716	5.4869	5.35548	4.97837	5.23448	4.90341	5.80575	5.57931	5.31972	5.10542	5.52013	5.33427	5.43829	4.98287
CXXC5	3.74395	4.3998	4.48971	4.80619	5.04112	4.13242	4.60208	3.98595	2.42477	3.54354	2.83894	3.83904	-2.76264	-2.14669	-2.09137	-1.5737
CXXC5-AS1	-0.14073	-1.99439	-0.79068	-0.3599	2.27546	0.90149	2.83414	0.28678	-1.96382	0.01143	0.28066	0.91472	-3.32193	-3.32193	-2.13022	-1.01803
NRG2	-1.79146	-0.93237	-0.86897	-0.54527	1.46178	0.62383	0.51634	0.65119	-1.06126	-0.16041	-1.25092	-0.35168	-3.32193	-3.32193	-3.10291	-3.32193
MALINC1	-1.11687	-0.44759	-0.78273	-0.5181	-0.44891	-0.8459	0.09632	-0.65096	-1.95795	-0.84422	-0.87778	-0.80925	-2.15995	-1.52846	-1.66442	-1.74559
PURA	2.59216	2.6484	2.67488	3.14411	1.61836	1.23906	2.14095	1.46026	2.16206	1.59041	2.37132	2.26236	1.64107	0.75798	1.98387	1.23272

IGIP	-0.32957	-0.50513	0.28308	0.33534	-0.06206	0.16501	0.58849	0.1563	-0.68556	-0.01835	0.28219	1.09936	-0.28469	-0.94203	-0.1635	-0.40149
CYSTM1	5.14598	5.09371	5.19649	4.97881	6.04163	6.22567	5.78414	5.72086	4.95533	4.64591	4.83325	4.32442	5.7415	5.51016	5.40333	4.97113
PFDN1	5.79632	5.53993	5.40912	5.34631	4.8804	5.10221	4.51616	5.09336	5.88198	5.63632	5.67041	5.41234	5.979	6.00701	5.63103	5.30945
HBEGF	5.66127	5.37273	4.9764	4.87371	0.71368	0.9215	1.42895	0.73114	3.29934	3.04156	3.43067	3.17746	5.15865	4.53396	4.31749	2.91233
ANKHD1	4.0032	4.07539	4.01513	4.5455	3.94613	3.66903	4.10997	3.97128	4.07994	3.87307	3.99094	4.77104	3.87663	3.81346	4.26738	4.31761
ANKHD1-EIF4EB	4.03858	4.08439	4.0397	4.56455	3.93108	3.69616	4.07942	3.94261	4.09028	3.87711	4.00858	4.76745	3.86577	3.80132	4.2549	4.3068
SRA1	4.18227	4.25026	4.1434	4.21672	4.57069	4.94529	4.2689	4.75547	4.80149	4.36425	4.61803	3.95861	3.96726	4.09911	3.98875	4.03393
EIF4EBP3	4.12077	3.80399	4.13189	4.17851	2.70925	3.90464	2.23667	3.51795	3.55082	3.45033	3.8018	4.00644	2.6662	2.68099	3.47837	2.93789
APBB3	1.94612	1.62065	2.36447	2.1053	2.89354	3.14878	1.97821	2.98987	1.63557	2.10162	2.34047	1.69422	1.17612	1.03798	0.98759	1.38069
SLC35A4	4.43753	4.36195	4.23761	4.25523	5.66119	4.99699	5.35805	4.89131	5.6411	5.28951	5.51559	5.16261	5.16854	5.55958	5.68103	5.92592
CD14	0.41636	1.52172	0.64395	2.20778	-0.35547	1.48773	0.0845	-0.34878	4.30795	4.63586	4.51774	4.56694	0.4061	0.99664	-0.38097	1.12977
NDUFA2	6.33982	6.45707	6.42511	6.3202	6.46014	6.86078	5.83697	6.85008	6.12183	6.28278	5.937	4.70098	5.63775	5.66539	4.46993	4.73988
TMCO6	2.18029	1.67107	2.05994	1.90191	2.87726	2.85759	1.9034	2.86194	2.3858	2.33248	2.23716	1.99523	1.54237	1.83193	0.47584	1.23219
IK	6.61264	6.59067	6.48978	6.70755	6.03522	6.1191	5.89586	6.02338	6.29795	5.94142	6.24728	5.34971	5.26614	5.029	4.90134	4.74257
MIR3655	9.17489	9.11715	8.80625	8.49241	8.57947	7.59223	8.75542	7.17678	9.14007	8.46584	9.24896	7.84912	7.43452	7.06267	8.04149	7.70782
WDR55	4.02052	3.89289	4.00735	3.90421	4.49936	3.90855	4.33331	3.94366	3.8473	3.42474	4.03564	3.47441	3.66365	3.39941	3.35579	3.18428
DND1	4.31309	4.08574	4.25268	3.5255	4.87201	4.66823	4.55067	4.81302	4.34188	4.20728	4.45556	3.70854	3.99073	3.69316	3.76843	3.81918
HARS2	4.0737	4.23152	4.22623	4.55647	4.20007	3.80338	4.21348	3.95031	3.64557	4.07329	4.17231	4.11325	3.89805	3.77048	3.88098	3.67692
ZMAT2	5.45954	5.43374	5.22093	5.6284	5.53989	5.09152	5.44889	5.17262	5.69011	5.62569	5.48771	5.38126	5.72475	5.73871	5.2971	5.47134
PCDHA1	-2.68514	-3.32193	-2.45664	-2.41459	-2.50154	-2.44103	-2.29628	-2.00873	-3.10626	-2.36859	-2.31767	-2.84217	-2.69584	-1.39007	-1.12788	-0.8014
PCDHA7	-2.47029	-2.64204	-2.75967	-1.56923	-2.89787	-3.32193	-3.15179	-2.61003	-3.32193	-2.49344	-3.13427	-2.99512	-1.34321	0.08818	-0.40943	0.9304
PCDHA9	-2.93441	-3.09181	-2.9728	-2.65882	-3.18685	-3.32193	-3.22175	-3.19545	-3.32193	-3.03168	-3.32193	-3.22024	-3.00996	-1.77381	-1.77979	-0.85974
PCDHA10	-2.99435	-2.83594	-2.8758	-2.72275	-3.29102	-3.32193	-3.25486	-3.237	-3.32193	-3.18663	-3.17707	-2.85034	-3.10811	-2.35467	-2.70331	-1.64883
PCDHB2	-2.6764	-2.14128	-3.03168	-3.01454	-2.60901	-3.32193	-3.32193	-2.58104	3.6586	3.42837	3.95003	3.78236	3.30526	3.31301	3.95671	4.00796
PCDHB3	-3.02171	-3.32193	-2.45625	-2.95228	-3.06311	-2.55497	-3.32193	-2.97126	1.00657	0.61133	0.74989	1.39065	1.24209	1.13409	2.17546	2.3474
PCDHB4	-3.32193	-3.32193	-3.32193	-3.32193	-3.32193	-3.32193	-3.32193	-3.32193	-1.67915	-0.91469	-0.72624	-1.29418	0.18048	0.04899	0.44278	0.81827
PCDHB5	-2.57072	-1.04866	-2.69937	-2.4247	-2.02867	-1.81499	-2.81081	-1.92949	3.91986	3.80971	4.32785	3.95355	3.79982	4.47119	4.93879	5.19781
PCDHB6	-1.90863	-2.70515	-2.2337	-2.18444	-3.18321	-3.32193	-2.79222	-1.61653	4.55771	4.39223	4.31416	4.35033	3.62862	3.76212	3.58633	3.86269
PCDHB17P	-3.32193	-2.96114	-2.94353	-3.32193	-3.32193	-2.99316	-3.32193	-3.32193	1.20588	1.18427	1.31402	1.56349	0.45335	1.39473	1.58506	1.45586
PCDHB7	-3.32193	-3.32193	-3.32193	-3.32193	-3.32193	-3.32193	-3.32193	-3.32193	0.4541	0.68772	1.01574	0.96827	1.97952	1.96264	2.87632	3.26229
PCDHB8	-3.32193	-3.32193	-2.58108	-3.32193	-3.32193	-3.32193	-3.32193	-2.90303	0.63104	1.29221	1.20211	1.57203	4.11623	4.45883	5.22992	5.47461
PCDHB16	-3.32193	-2.0628	-1.91335	-3.06381	-3.14328	-3.32193	-3.12675	-2.86862	-0.68239	-0.09226	0.45426	0.36926	2.17034	2.71842	3.1802	3.5963
PCDHB9	-3.08829	-3.32193	-2.81957	-3.32193	-3.32193	-3.32193	-3.32193	-2.44508	0.19169	0.92362	1.23766	0.84786	0.58952	1.52895	1.76112	2.66548
PCDHB10	-3.32193	-2.98304	-1.75811	-2.94586	-3.05844	-2.75923	-3.32193	-2.96512	0.63532	1.28868	1.58886	1.62028	2.13952	3.09327	3.31034	3.78633
PCDHB11	-3.07719	-2.45006	-2.41418	-3.0193	-3.32193	-3.32193	-3.32193	-3.03517	0.5819	1.26291	1.54368	1.86407	0.47412	0.92906	1.95508	2.05009
PCDHB12	-3.05855	-2.39652	-3.01475	-3.32193	-3.32193	-2.63709	-3.32193	-3.32193	1.27981	1.46744	1.90757	1.55978	1.40162	1.33541	2.25276	2.58562
PCDHB13	-3.32193	-2.42614	-2.87476	-3.32193	-3.32193	-2.4939	-3.32193	-2.87324	2.32248	1.99074	2.5329	2.48421	2.65243	2.65012	3.81584	3.66684
PCDHB14	-3.10687	-2.14368	-2.85632	-2.83046	-3.13727	-2.59934	-3.32193	-3.06951	1.67043	1.44347	1.81989	1.48407	0.81754	1.16729	2.38153	2.36934
PCDHB18P	-2.53258	-3.32193	-3.32193	-2.38127	-3.18036	-3.32193	-3.32193	-2.95596	-1.18897	-1.58166	-0.55974	-0.25765	-0.94782	-0.62241	-0.24863	0.03412
PCDHB19P	-3.32193	-3.32193	-2.65206	-2.61734	-3.32193	-3.32193	-3.32193	-3.32193	-0.68548	-0.72491	-0.37383	-0.86457	-1.27714	-0.58945	0.44572	0.72911
PCDHB15	-2.85056	-1.56258	-1.70835	-1.02841	-3.20815	-3.0645	-3.32193	-2.77635	-1.27087	-1.02546	-1.55562	-1.15841	0.07533	0.69897	0.87498	1.20538
TAF7	5.11813	5.15293	5.03653	5.4223	3.11811	2.07758	3.76153	2.21498	5.97304	6.04338	5.98317	6.06729	5.67653	5.84282	5.43403	5.57226
PCDHGA1	1.75055	1.97004	1.79407	2.05075	-2.56884	-2.61921	-2.8915	-2.10229	3.43577	3.24748	3.49675	3.6155	4.27546	4.46964	5.38571	5.37317
PCDHGA2	1.72128	1.95234	1.781	2.0123	-2.40936	-2.55621	-2.80493	-2.16127	3.51902	3.36394	3.57022	3.73296	4.35567	4.53568	5.47754	5.44556
PCDHGA3	2.08895	2.32568	2.13114	2.37399	-2.41211	-2.34696	-2.79013	-1.8869	3.67089	3.51055	3.69983	3.8545	4.68164	4.85869	5.81112	5.80744
PCDHGB1	2.43322	2.52552	2.45786	2.61708	-2.20157	-2.45638	-2.78056	-1.86741	3.87318	3.72511	3.95699	4.06991	4.67468	4.89185	5.82339	5.80949
PCDHGA4	2.09116	2.31217	2.14033	2.37639	-2.41363	-2.23514	-2.52183	-1.88894	3.66557	3.50539	3.68003	3.87071	4.77466	4.96545	5.895	5.87295
PCDHGB2	2.22939	2.4175	2.2987	2.48696	-2.20091	-2.45583	-2.63698	-1.76823	3.74398	3.61615	3.77489	3.91462	4.62982	4.84476	5.76154	5.7785
PCDHGA5	1.87734	2.09714	1.9095	2.15639	-2.38088	-2.56971	-2.8582	-1.93777	3.40541	3.26162	3.43303	3.61059	4.34596	4.58081	5.47777	5.48094
PCDHGB3	2.13453	2.36062	2.17836	2.42011	-2.4489	-2.45419	-2.77904	-1.86433	3.66372	3.50249	3.69058	3.85332	4.58509	4.81548	5.73679	5.73633

PCDHGA6	1.44349	1.67067	1.47176	1.70971	-2.54927	-2.47904	-2.6901	-1.86221	2.95045	2.80684	2.99041	3.16122	3.90884	4.13578	5.01	5.01436
PCDHGA7	1.93956	2.16002	2.0069	2.26248	-2.34451	-2.31532	-2.83722	-1.98485	3.46669	3.30396	3.48838	3.63395	4.39391	4.60745	5.53268	5.51234
PCDHGB4	2.04381	2.41202	2.32567	2.68745	-1.21982	-1.61151	-1.13077	-1.39325	3.26843	3.152	3.3146	3.4732	4.65896	4.76879	5.65509	5.68474
PCDHGA8	1.44658	1.76512	1.60945	1.96802	-2.35738	-2.26033	-2.65697	-2.00452	2.80641	2.6504	2.82204	2.97224	3.96875	4.07621	4.96859	4.94582
PCDHGB5	2.24968	2.44186	2.32459	2.49934	-0.41059	-1.47866	-0.6158	-1.52539	4.10665	3.93804	4.12815	4.32843	5.03735	5.26792	6.17726	6.20451
PCDHGA9	2.14759	2.34749	2.18688	2.42803	-2.29256	-2.21313	-2.77828	-1.58528	3.70237	3.52083	3.7024	3.85264	4.59517	4.81855	5.72822	5.72924
PCDHGB6	1.6683	1.85576	1.6802	1.93631	-2.37823	-2.21287	-2.70453	-1.22879	3.1846	3.05297	3.20793	3.40768	4.092	4.32027	5.23342	5.21892
PCDHGA10	1.89601	2.10749	1.90848	2.26452	-2.03566	-2.54898	-2.417	-1.76343	3.20143	3.01573	3.2151	3.35534	4.09954	4.32132	5.24424	5.2256
PCDHGB7	2.06858	2.29467	2.12998	2.37031	-2.18786	-2.35986	-2.79821	-1.90347	3.70625	3.6232	3.74681	4.10289	4.51871	4.73264	5.65835	5.63909
PCDHGA11	1.86825	2.06709	1.88363	2.1255	-1.91913	-2.08945	-2.00273	-1.70647	3.37245	3.20782	3.3998	3.54432	4.29156	4.50082	5.43352	5.41434
PCDHGA12	2.10669	2.33833	2.15509	2.40295	-2.16344	-2.338	-2.51268	-1.87542	3.64205	3.47309	3.66376	3.80159	4.56152	4.77438	5.70622	5.68222
PCDHGC3	2.20633	2.43942	2.29494	2.48111	-2.34307	-2.31385	-2.58481	-1.88928	3.87756	3.61657	3.84038	4.0658	4.57066	4.79905	5.79152	5.69801
PCDHGC4	2.06093	2.28205	2.10929	2.34584	-2.47776	-2.48292	-2.79892	-1.90492	3.59556	3.42867	3.61346	3.7598	4.52101	4.72635	5.66368	5.64162
PCDHGC5	2.0725	2.36322	2.15338	2.37991	-2.47981	-2.48496	-2.80033	-1.90782	3.59097	3.42408	3.61267	3.75834	4.51486	4.72041	5.65736	5.63876
DIAPH1	4.65074	4.48231	4.30808	4.41243	6.20777	4.53828	6.10417	4.64741	5.36436	4.54986	4.57387	4.78334	5.06305	4.97086	5.19153	5.23534
HDAC3	3.60685	3.38226	3.50813	3.30371	4.10407	3.52617	3.88039	3.48838	4.68244	4.70345	4.603	4.23265	3.40292	3.71342	3.41205	3.43679
RELL2	1.97335	2.17933	2.72622	2.257	4.42763	5.03058	2.97418	5.13885	2.97896	3.47415	3.04359	2.70789	2.26538	3.51339	2.6209	2.85175
FCHSD1	1.21642	1.47631	1.90062	1.77546	2.77767	3.38543	1.77245	3.33656	3.35487	3.5001	3.57882	2.93047	1.61522	2.17618	2.15574	2.27475
ARAP3	2.26451	1.95907	2.11645	2.03257	-2.8646	-3.32193	-3.32193	-3.32193	3.4814	3.64954	3.61806	3.90375	-2.48492	-1.7569	-1.96735	-1.09756
PCDH1	-2.48287	-1.93054	-1.88181	-2.32491	1.58672	0.9814	0.7512	1.01686	-1.41471	-0.60416	-1.53463	0.67419	-2.72259	-2.82567	-2.07047	-1.65236
DELE1	2.18296	2.1633	2.33395	2.15131	3.58556	3.20114	3.22481	2.81053	3.04883	3.45342	3.41733	3.5893	2.81929	2.32177	2.51718	2.20837
RNF14	3.28526	3.68779	3.5321	3.97624	2.29237	1.71304	2.58448	1.92681	3.53892	3.50531	3.20052	3.91548	3.73779	3.97931	3.58875	3.71487
GNPDA1	4.95601	4.70814	4.58974	4.96428	4.68449	4.76666	4.87866	4.67785	4.2308	3.83118	3.91068	3.93318	5.37185	5.26651	5.1744	5.13227
NDFIP1	4.10191	4.28695	4.21008	4.61069	4.73306	4.6023	5.30459	4.40608	3.92982	3.91724	3.81086	4.29228	4.72322	4.62001	4.49657	4.54518
SPRY4	2.33925	2.62113	2.36593	2.70646	2.45582	3.24118	3.07168	3.11727	1.91897	3.00692	2.41142	3.97119	3.27758	3.28615	3.44081	4.59319
FGF1	0.56856	0.03533	0.81225	-1.26619	-3.32193	-3.32193	-3.32193	-3.32193	1.38626	0.49086	1.24552	-0.18264	1.74935	-1.8172	0.70591	-2.03753
ARHGAP26	2.27303	2.04281	2.10743	1.97253	2.43423	1.41644	2.66001	1.4805	0.32082	0.87394	0.32258	1.51135	1.91351	2.69651	2.91233	3.24663
NR3C1	2.83084	3.11551	3.42579	3.64656	3.68821	1.89823	4.48223	2.35394	4.12191	4.4712	4.33442	5.20099	3.34998	3.28161	4.04936	3.45293
RPL7P21	1.12288	0.91334	0.87783	0.03671	1.9205	1.36263	2.4793	1.18942	0.85185	1.66191	1.08804	0.45895	1.14181	1.47069	1.05921	0.9177
YIPF5	4.33706	4.77381	4.59836	5.70156	5.00771	4.43718	5.50884	4.23892	4.06174	4.34496	3.94631	4.78836	3.9824	4.24229	3.47404	3.74216
KCTD16	-3.1732	-3.156	-3.14733	-3.13658	-3.28904	-3.32193	-3.2506	-3.32193	-3.15043	-3.11145	-3.32193	-3.18057	-1.86562	-0.34619	-0.85985	-1.27711
PRELID2	0.96218	1.13139	0.91004	0.87318	0.595	-0.10135	0.99057	-0.06082	1.72496	1.52846	0.99979	1.33507	1.86871	1.52816	1.47938	1.25262
SH3RF2	0.09202	0.1372	0.52104	0.33926	2.1723	0.37135	1.85045	0.73987	1.87484	2.01536	1.85767	1.8881	1.34857	1.20371	1.67552	1.62514
RBM27	2.94235	3.08454	2.74066	3.44213	3.38442	1.83651	3.8792	1.76464	3.76208	3.52644	3.58114	4.25492	3.21824	3.05425	3.39352	2.98513
TCERG1	3.73331	3.51303	3.46909	3.50525	3.6672	3.01411	3.86618	3.10012	3.6842	3.2103	3.80307	3.48147	3.89315	3.59949	3.88364	3.28892
DPYSL3	3.05706	3.48566	3.43181	3.5036	-2.48828	-3.17197	-2.24306	-3.14707	3.25656	3.92955	4.08856	4.1451	4.26405	6.02462	6.46076	6.33226
SPINK1	0.38531	1.90662	1.86444	1.87769	-3.03184	-2.27899	-2.7471	-3.32193	-3.32193	-3.32193	-3.32193	-3.32193	-2.82538	-2.82688	-3.32193	-3.32193
C5orf46	-3.32193	-3.32193	-3.32193	-3.32193	-3.32193	-3.32193	-3.32193	-3.32193	-3.32193	-3.32193	-3.32193	-3.32193	3.68735	2.5372	1.57871	1.13106
SPINK5	-2.40676	-1.84189	-2.28672	-1.53113	-3.32193	-3.32193	-3.10115	-2.81459	-2.45508	-1.0206	-2.38262	-0.64969	-1.76462	-0.77588	-1.72276	-0.90996
FBXO38	1.99911	2.12589	2.18214	2.71105	2.1365	1.96891	2.6022	1.85775	2.13841	2.31436	2.28814	2.97313	2.1402	2.21936	2.22889	2.15678
ADRB2	2.44426	2.22636	1.26106	1.80173	-2.85418	-2.78008	-2.81519	-2.70253	2.6212	2.42234	2.32978	1.63836	1.69754	1.45395	0.31111	0.86064
SH3TC2	-0.72384	-0.79905	-0.97307	-0.57131	-3.2301	-3.24859	-3.25409	-3.32193	0.11536	-0.36505	-0.22869	0.17854	0.90919	-0.24314	0.21873	0.53001
SH3TC2-DT	-3.32193	-2.83243	-2.80957	-2.38936	-3.32193	-3.32193	-3.32193	-3.32193	-1.69355	-1.96482	-2.00501	-2.72569	0.36088	-1.82308	-1.97355	-1.60697
ABLIM3	3.8553	3.90945	3.97761	3.55444	2.47535	2.93517	2.42338	2.38149	4.54531	4.72193	4.59383	5.07317	0.12836	1.14066	0.7213	1.93806
AFAP1L1	3.17159	3.5077	3.33785	3.16044	-1.17749	0.95978	-0.87562	-0.14928	4.30334	4.07387	3.85367	4.15794	4.20478	3.5502	3.87438	3.9558
GRPEL2	3.66595	3.5253	3.27063	3.57771	3.13708	2.60089	3.39824	2.84012	2.89315	2.75253	2.30973	3.40001	2.86443	3.08358	2.231	2.36148
GRPEL2-AS1	4.14672	3.78513	3.38107	3.0291	4.36807	1.77683	4.09444	1.51726	3.98622	3.42469	3.82121	3.75426	3.71591	3.55261	3.33322	3.62957
PCYOX1L	-1.491	-0.83826	-1.15694	-1.24168	-1.25002	-0.25669	-1.6135	-0.09453	1.22931	1.68703	1.4592	1.44287	0.73372	0.87739	0.93132	0.92377
CSNK1A1	5.1298	4.88901	4.99125	5.14192	4.73181	3.77297	5.01739	3.66007	5.89674	5.22737	5.34425	5.59267	5.17542	4.70756	4.96233	4.67021
ARHGEF37	-1.29997	-0.92847	-1.00803	-0.83631	-2.47004	-1.39576	-2.57314	-1.10744	-0.93342	-0.97806	-1.36985	-0.36371	-1.0683	-0.70817	-0.23179	0.09405

PPARGC1B	1.13851	0.46899	0.28691	0.23809	-0.45072	-1.73259	-0.59492	-1.17208	0.31136	-0.38128	0.20691	-0.81581	0.77684	0.4924	0.62038	0.59483
SLC26A2	3.15412	3.3721	2.97197	3.44228	2.27323	0.90222	2.6447	0.72713	2.9409	2.92364	2.89773	3.82413	3.77244	2.74493	3.47524	3.21809
TIGD6	1.75636	1.80658	2.05053	1.64135	1.88541	0.94598	1.92615	0.65281	1.89387	2.02998	1.76226	2.22243	1.70404	1.45292	2.06636	2.03532
HMGXB3	5.12862	4.92108	5.02048	5.07347	4.48708	4.43883	4.56877	4.45186	3.85552	3.85673	4.1373	4.30321	3.77318	3.87467	4.12081	4.21742
CSF1R	1.23886	1.57289	1.11815	1.68682	-2.66573	-2.7789	-3.13208	-2.14913	-2.55593	-1.52699	-1.65053	-0.54449	-2.88457	-3.1615	-2.95712	-2.40949
RPL7P1	5.15516	5.29583	5.07484	4.5269	4.32199	4.42122	4.81891	4.04752	4.96056	5.68922	5.00784	5.0313	4.44844	4.04296	3.92391	4.08158
PDGFRB	-2.05068	-2.25256	-1.33527	-1.26622	-2.64349	-1.07756	-3.05527	-0.88959	1.17726	1.20297	1.26192	0.96001	-2.99357	-2.728	-2.93074	-2.70782
CAMK2A	-3.15047	-2.96208	-2.9445	-2.61065	-3.32193	-2.40112	-3.32193	-3.12017	-2.65585	-0.89407	-0.65288	0.22003	-2.84118	-2.84263	-3.32193	-3.00427
ARSI	-1.7862	-2.27514	-2.2344	-2.18517	-0.12541	0.17913	-0.2665	-0.71715	4.55131	4.53607	5.33317	4.34422	1.44499	1.65055	1.61798	2.6785
TCOF1	5.88014	5.12949	5.08538	4.38828	4.71726	3.69347	4.34814	3.91524	5.30507	4.12322	5.00428	4.1789	4.47628	4.05758	4.83107	4.38113
CD74	3.05565	2.95108	2.6172	2.95073	-0.1075	1.0847	-1.4226	0.5197	6.01189	7.56198	7.06619	7.54901	-3.0698	-2.22316	-3.32193	-3.32193
NDST1-AS1	2.73075	2.47106	3.01713	1.83235	4.08992	1.2902	3.98634	0.68266	5.66803	5.36392	5.774	4.89192	3.99655	4.11994	5.4659	5.56793
SYNPO	3.01609	3.37982	3.33164	2.20122	3.77766	1.38357	3.03945	1.39437	4.97508	5.01103	5.30554	4.3407	1.13639	0.08777	1.2261	1.62082
RBM22	4.56651	4.38848	4.31203	4.70102	3.72874	2.89893	3.96452	2.85291	3.98156	3.86096	4.02407	4.43519	3.76084	3.83072	3.54106	3.81716
DCTN4	3.51663	3.80886	3.54923	4.23178	3.0312	2.36127	3.59571	2.42362	3.74257	4.00284	3.99432	4.93487	3.13101	3.52425	3.71431	3.52671
SMIM3	3.89501	3.20977	3.41508	3.54794	2.37924	1.70624	2.13328	1.74243	2.34776	1.68393	2.21754	1.61624	2.43107	1.56456	1.68436	0.67264
ZNF300	3.04379	2.58139	2.85797	3.35768	0.38196	1.30221	0.7026	1.50668	0.77879	1.11767	0.67324	1.46055	1.66	2.20327	1.02282	1.69697
GPX3	4.81614	4.88735	4.97041	5.28448	3.23998	3.49684	2.99622	3.24313	1.36813	1.82004	2.15273	3.50866	0.41175	1.07811	0.31954	0.57417
CCDC69	3.79089	3.77701	3.42548	4.28011	0.66483	-0.62133	-0.06482	-0.38537	1.31322	1.38857	-0.16969	1.33994	-1.54139	-2.17564	-3.12229	-2.63777
GM2A	5.4006	5.71786	5.65347	5.73012	5.12646	5.16493	5.16569	4.86491	6.35675	5.70134	6.19686	6.26914	3.91004	3.59351	4.22843	4.40622
SLC36A1	1.00899	1.02463	1.55847	1.42373	2.14744	1.59598	2.99598	1.55101	1.7304	2.02784	3.12367	2.51027	0.06971	1.06194	2.30478	1.8192
FAT2	-2.35033	-2.69963	-2.55999	-1.97133	-3.1379	-3.10539	-3.32193	-2.61262	-2.93204	-2.45504	-2.32515	-2.38758	-3.32193	-3.09948	-3.18187	-3.11241
CLMAT3	0.02999	-0.36605	0.39729	0.33648	-2.51275	-1.1261	-1.33065	-1.89684	7.84534	7.81837	8.07072	7.35615	4.06138	3.88862	4.28635	4.11969
ATOX1	4.76214	4.51181	4.37188	4.25556	2.79212	3.45999	2.38689	3.49908	4.04076	3.85082	3.87269	3.40665	2.92331	3.02866	1.76964	1.83577
G3BP1	5.15823	4.85316	4.65681	4.77829	4.33	2.93591	4.55634	2.9275	5.81347	5.04185	5.39204	5.26371	4.662	4.37864	4.44069	4.24894
FAM114A2	1.97068	1.94654	1.66794	1.94807	2.54049	2.03764	2.70775	1.92465	2.44616	2.10362	2.18142	2.37684	1.39378	1.02524	1.08895	0.48178
MFAP3	1.69691	1.71425	2.19245	2.14213	3.20922	1.10228	3.99356	0.96298	2.35266	2.17129	2.67244	2.9535	3.00318	2.53028	3.00866	2.6471
GALNT10	3.14529	3.01806	3.36293	3.17973	3.64875	1.99825	3.89174	1.81969	3.82652	3.74704	4.33192	4.2035	4.38921	4.0569	4.96504	4.15328
SAP30L-AS1	0.52421	0.46578	1.10541	1.3972	-0.31826	-0.89507	0.02636	-0.23619	-0.34702	-1.45141	-0.57605	-0.68422	0.65993	-0.8343	-1.07169	0.08431
SAP30L	3.58397	3.77122	3.91521	3.35595	3.20555	3.00408	3.05016	2.71155	3.06467	3.01977	3.0697	2.95552	2.15011	2.34678	2.50958	2.67836
FAXDC2	0.75745	0.89613	1.25035	0.80037	0.87529	-0.09441	0.54026	-0.46131	0.95326	0.67108	0.98655	0.78228	0.17267	0.39664	0.63874	0.72431
CNOT8	4.34642	4.4536	4.55725	4.37972	3.48497	3.30217	3.5904	3.05272	4.03545	3.8821	3.92583	3.97224	3.66078	3.80794	3.28875	3.6846
GEMIN5	3.32977	2.85591	2.43765	2.91107	3.55795	2.13343	3.62319	2.26302	3.78818	3.28376	3.58784	3.73997	3.9605	3.57436	3.89994	3.67319
MRPL22	3.99198	3.78948	3.72553	3.6618	3.47193	3.85423	3.63828	3.81621	3.5373	3.23594	3.41666	2.61041	3.0744	3.01697	2.40364	2.32295
SGCD	-3.32193	-3.32193	-3.32193	-3.1942	-3.32193	-3.32193	-3.32193	-3.32193	-3.09484	-2.96258	-2.70674	-3.04865	-1.47988	-0.12251	-1.0027	-0.10455
HAVCR1	-2.87745	-2.46596	-2.13137	-1.82521	4.78615	3.81479	5.09527	3.47631	4.85573	4.38828	4.57503	4.8868	7.07129	7.241	7.19519	6.45169
HAVCR2	0.64985	0.49089	0.3611	0.41825	-0.7944	0.097	-0.18305	-0.35941	-0.3883	-1.26955	-0.77072	-0.92819	-1.19871	-1.31252	-0.7215	-2.06267
MED7	2.2678	2.30584	2.42529	2.55708	2.15957	2.38358	2.58357	2.10928	2.52791	2.28651	2.32566	2.79594	2.77442	2.6644	2.57921	2.23329
CYFIP2	-2.05608	-1.29681	-1.68869	-1.71158	1.43788	1.03629	1.14064	1.12699	3.46233	3.27523	3.71872	4.00841	2.06464	2.16363	3.19027	3.02323
ADAM19	4.83693	4.88316	5.15555	5.19126	2.42755	2.11	2.1336	1.70105	6.10118	6.19917	6.79653	7.26995	2.95525	1.40921	2.99419	3.38844
NIPAL4	-0.16312	0.87887	0.28024	1.63394	-3.21313	-3.32193	-3.32193	-3.32193	0.47125	0.34918	0.59827	1.14773	4.38693	2.93171	4.62027	3.64607
THG1L	2.77078	2.98028	2.96532	2.9174	2.13395	2.15777	2.31027	2.06037	3.47021	4.00817	3.68474	4.05686	3.00556	3.21445	2.90882	2.7663
LSM11	1.47294	1.62769	1.66558	1.2935	1.69556	0.54469	1.88249	0.36934	1.46371	1.90645	1.81527	1.82906	2.34941	2.75949	2.97969	2.75958
CLINT1	4.9219	4.77923	4.74716	5.19602	5.1723	3.80863	5.64937	3.74965	4.44928	4.13459	4.52614	4.73299	4.13923	3.74938	3.84343	3.51984
EBF1	-1.99567	-1.44259	-1.56434	-0.64564	-2.23757	-1.55075	-2.20247	-1.32611	-3.32193	-3.32193	-3.32193	-3.32193	-3.32193	-3.32193	-3.32193	-3.32193
RNF145	5.44512	5.46486	5.33039	5.39947	4.12005	3.78564	4.76474	3.86246	5.80885	5.77897	5.84417	6.24558	5.34654	5.23074	4.84311	4.82027
UBLCP1	4.28991	4.4706	4.65832	5.01761	3.17675	2.40337	4.02826	2.35106	3.78876	3.03748	4.05005	3.92287	3.64718	2.39564	3.3863	1.81749
ADRA1B	-2.03256	-0.29825	-0.4147	-0.99683	-2.63186	-1.86132	-2.2664	-2.56738	-0.89846	-0.05373	-0.94644	-0.35006	5.86881	5.54765	6.69293	6.11594
TTC1	6.48036	6.43226	6.1987	6.50136	5.02316	5.09051	5.14674	5.02827	5.48952	5.32582	5.36649	5.40575	5.05991	5.00929	4.55928	4.55037
PWWP2A	2.51181	2.57879	2.79413	2.78402	1.55607	1.10378	1.80496	0.83789	1.94169	1.96967	2.05344	2.20461	2.12092	1.49921	1.81809	1.35978

FABP6	6.74932	6.95806	7.23366	5.94754	-0.71377	0.72295	-2.62381	0.17758	-1.58854	-2.12915	-2.56428	-1.50292	-2.71561	-2.71738	-2.1372	-3.32193
CCNJL	4.08432	4.28034	4.15117	3.98581	4.35752	4.81089	4.42937	4.70285	3.08104	3.71731	3.35544	3.47842	2.97823	3.6196	3.8652	4.40229
ZBED8	0.78122	1.16627	1.20194	1.09983	1.20711	0.60585	1.27988	0.77362	0.3471	0.6636	0.01019	0.83619	1.98786	1.19889	1.12257	1.54716
SLU7	4.49309	4.761	4.6363	5.30825	3.12325	3.24378	3.54717	3.19027	3.48016	3.77689	3.67675	4.14372	3.0873	3.34342	2.96054	3.50488
PTTG1	5.96717	5.53349	5.4533	5.24668	5.5163	5.29848	5.22021	5.18846	6.1249	5.39616	5.58824	4.48985	5.86482	5.72496	4.67863	4.2064
MIR3142HG	2.60174	2.41264	2.50956	3.08548	1.07418	0.44553	0.79677	0.03242	0.98688	1.50189	1.63459	1.81615	-2.10703	-0.93984	-2.91917	-1.11381
GABRG2	-2.2394	-1.92427	-1.87541	-1.35565	-3.14629	-2.89705	-3.19119	-3.10638	-3.26622	-3.32193	-3.22408	-2.95569	-3.24733	-3.32193	-3.32193	-3.32193
CCNG1	5.03736	4.96308	5.40369	5.51919	5.09643	4.01067	5.72812	3.65457	6.22855	5.46967	6.13108	6.42443	6.597	4.81771	6.88235	5.24491
NUDCD2	3.34391	3.1717	3.26762	3.34266	2.71791	2.51241	2.68481	2.3907	3.00048	2.71622	2.59104	2.31183	2.52686	2.6874	2.00413	1.95434
HMMR	4.77144	4.33283	4.44177	4.26622	4.1156	2.73967	4.72661	2.782	4.44508	3.38983	3.52591	3.3364	3.40452	2.83172	2.92464	2.23824
HMMR-AS1	4.48035	3.76255	4.01305	4.05165	3.70605	2.20472	4.17323	2.50962	3.99429	3.18483	3.12985	2.94697	2.64684	2.38627	2.24665	1.52103
MAT2B	3.6845	3.77523	3.65632	4.39856	3.63386	2.72633	4.0814	2.88929	4.1222	4.01724	4.04835	4.17831	3.81463	3.93015	3.18844	3.40122
TENM2	-2.09821	-2.19537	-2.32246	-2.54806	-3.08423	-3.22288	-3.23025	-3.20609	0.25674	-0.09901	0.06568	-0.40928	2.61322	3.05077	3.70691	2.39077
WWC1	4.02328	3.9057	3.56615	3.23503	4.372	2.38744	4.40862	2.69725	5.5545	4.54572	5.03034	3.63782	2.36143	2.66619	2.59681	2.07953
FBLL1	-2.72648	-3.32193	-2.17746	-1.48195	0.13514	1.72374	-1.56291	1.85789	-2.64937	-2.744	-2.70905	-3.32193	0.54693	2.48653	1.91022	2.33235
PANK3	3.41163	3.40459	3.30028	3.67975	3.79299	2.49771	4.45392	2.3651	3.9581	3.36265	3.69155	3.9238	4.6486	4.43241	4.69499	4.40479
SLC2A3P1	3.13178	2.90389	3.24468	2.93785	4.0485	1.07107	4.63312	-0.01351	4.35704	3.71126	4.15635	4.20204	4.6066	4.59899	4.9106	4.64677
RPL10P9	4.60919	4.50173	4.49066	3.54159	6.15972	7.90772	5.35305	7.91443	5.06728	5.81997	4.8149	4.08598	3.29421	2.87437	3.00486	2.66072
SLIT3	-1.78342	-1.7004	-1.22146	-0.96509	-3.2252	-3.24465	-3.18232	-3.06588	2.83104	3.56182	3.18386	3.48654	0.49469	0.8912	2.03787	1.57726
SPDL1	3.8098	3.45769	3.58672	3.56461	3.15902	1.90565	3.54303	2.24423	4.60238	4.01143	4.21309	3.95869	4.71462	4.2516	4.10384	3.33918
DOCK2	-1.68622	-1.14745	-1.34936	-1.57505	-3.18143	-3.15617	-3.16824	-3.32193	2.11809	1.83676	2.16308	2.357	0.12511	-0.03763	0.87212	-0.38842
INSYN2B	1.34065	1.15084	0.23032	0.31435	-3.32193	-3.32193	-3.32193	-2.93477	2.04644	2.50843	2.24428	2.8212	1.26981	0.57914	0.49522	0.49901
FOXI1	-3.32193	-3.32193	-3.32193	-3.32193	-3.32193	-3.32193	-3.32193	-3.32193	-3.32193	-3.32193	-3.32193	-3.32193	-0.12773	0.85068	-0.05087	-0.07137
LCP2	0.69723	0.63699	0.14111	0.94555	-3.32193	-3.32193	-3.32193	-3.32193	-3.1526	-3.32193	-3.03271	-3.18237	-3.32193	-3.32193	-3.32193	-3.32193
KCNIP1	-3.02138	-3.32193	-2.97212	-3.32193	-3.32193	-3.32193	-3.32193	-3.32193	-3.32193	-3.32193	-3.32193	-3.32193	0.4733	-0.28067	0.9852	-0.40458
RANBP17	-3.32193	-3.01352	-3.20575	-3.32193	-0.98328	-1.17295	-0.65478	-0.98821	-1.59786	-1.2724	-1.45811	-1.15546	-3.32193	-3.24434	-3.32193	-3.32193
TLX3	-3.32193	-3.32193	-3.32193	-3.32193	0.77541	1.5289	-0.27254	1.46399	-3.32193	-3.32193	-3.32193	-3.32193	-3.32193	-3.32193	-3.32193	-3.32193
FGF18	0.05368	-0.31867	0.70297	0.51805	-1.21108	-0.37387	-2.02377	-0.24519	-2.3895	-3.32193	-2.83152	-3.32193	0.26565	-0.84269	-1.32738	-0.8426
FBXW11	3.98419	4.0015	3.88977	3.97597	4.21627	2.9445	4.68171	2.77332	4.52692	4.20932	4.32156	4.66696	4.19434	4.21561	4.29803	4.19575
STK10	4.4987	4.56289	4.79107	4.22803	2.24534	1.63529	2.24308	1.42302	4.91667	4.90935	4.95469	4.99778	1.79098	2.03875	2.50178	2.99486
UBTD2	4.42755	4.79337	4.66789	5.18351	3.57566	2.91469	4.17588	2.89817	3.95546	3.63091	3.83793	4.21202	3.66456	3.62553	4.20651	4.04685
SH3PXD2B	2.29352	2.97608	2.39651	3.45017	2.77964	1.386	2.36328	1.36472	4.09856	4.28375	3.98148	4.52229	1.51556	1.99591	3.20561	3.70675
NEURL1B	-3.32193	-2.8295	-2.66884	-2.77838	-0.04818	-0.88963	-0.85962	0.16384	2.43892	1.84602	2.09347	0.73907	1.0221	1.97421	2.42841	1.9223
DUSP1	5.43142	5.02632	5.09253	4.40334	6.73112	5.71189	6.19927	5.32248	5.526	5.24309	4.41987	3.82812	5.99195	4.17592	5.23441	4.12359
ERGIC1	4.20945	4.32329	4.32893	3.71682	5.95122	4.93821	5.47252	4.4919	4.86417	4.96557	4.81572	4.61351	3.60183	3.50876	4.05295	3.98191
RPL26L1	6.09826	5.98672	5.89298	5.96709	5.45641	5.5323	5.6657	5.6661	5.45833	5.42794	5.41911	4.96051	5.39569	5.44328	4.70802	4.46126
ATP6V0E1	7.14226	7.35157	7.29073	7.60218	7.00945	7.24485	7.1745	6.96779	6.68475	6.71624	6.76537	6.60764	7.05022	7.01291	6.68086	6.75913
CREBRF	-0.15326	0.24824	0.40349	1.36216	-0.60255	-0.70045	-0.14439	-0.9667	0.7038	1.53179	0.72383	2.38367	0.20796	-0.36228	-0.06918	0.36892
BNIP1	3.14004	2.64994	2.56153	2.59524	2.69216	3.06441	2.55092	3.06136	2.94099	3.08819	2.98358	2.78969	2.81814	2.75587	2.29228	2.17241
NKX2-5	-2.24337	-3.32193	-3.32193	-3.32193	-2.92706	-2.00059	-2.89328	-2.79501	3.24668	3.06899	3.20762	2.3375	-3.32193	-3.32193	-3.32193	-3.32193
STC2	7.04102	6.68578	7.04774	5.45327	3.41907	4.72399	3.58414	4.2529	4.50726	4.88784	4.354	4.19013	7.27941	5.97116	7.05283	5.90556
BOD1	4.49574	4.87616	5.01409	4.98895	4.37478	4.4615	4.81812	4.16514	3.74651	5.27315	5.0149	5.11688	3.92556	5.19882	5.31314	4.98819
CPEB4	1.60726	2.37291	2.0266	2.79868	2.2624	2.21343	3.20796	2.1595	0.50321	0.92017	0.4271	2.15077	2.82232	3.46329	3.39263	3.92325
LINC01411	1.19245	1.77569	1.98969	2.26855	-3.32193	-3.0511	-3.32193	-3.32193	-3.32193	-3.06256	-3.32193	-3.32193	-3.32193	-3.32193	-3.32193	-3.32193
MSX2	-2.89765	-1.97993	-3.32193	-2.80428	-0.22587	-2.0693	-0.8709	-1.9287	0.27829	-0.16606	0.69868	-0.41842	-3.32193	-3.32193	-3.32193	-3.32193
SFXN1	4.25498	4.05589	4.13239	4.12423	4.08368	3.6288	4.66004	3.49718	4.61049	3.36875	4.32565	4.24565	4.64439	3.92739	4.68207	3.95548
CPLX2	-3.32193	-3.32193	-3.14621	-3.13541	0.77501	-1.3681	-0.00281	-1.10749	-3.32193	-3.32193	-3.32193	-3.32193	-3.32193	-3.32193	-3.0484	-3.32193
THOC3	5.22107	4.85854	4.68911	4.72109	4.24219	3.5556	4.07942	3.54545	4.62095	4.21631	4.37203	3.65135	4.69667	4.82792	4.01554	4.12152
SIMC1	-3.13265	-2.25051	-2.73862	-2.27796	1.50534	0.98932	1.017	0.93928	1.09556	1.53527	1.16957	1.4497	1.4636	1.9463	1.75103	1.84346
KIAA1191	5.24784	5.32291	5.15857	5.44876	5.37215	5.13742	5.49165	4.90929	5.4981	5.4891	5.27697	5.4718	4.78668	5.25238	4.83787	5.12319

ARL10	1.93552	1.29222	0.94823	0.8492	1.3542	1.14352	1.43887	0.90481	2.9458	3.50504	3.41505	4.4498	2.11404	2.16281	2.48454	2.3496
NOP16	6.31095	5.91203	5.7941	5.65211	5.49018	5.7594	5.32534	5.58684	5.31301	5.12106	5.33674	4.21199	5.25922	4.88078	4.3133	4.06114
HIGD2A	7.79602	7.6496	7.7039	7.53751	6.99221	7.48674	6.61359	7.29415	7.18803	7.08272	7.1214	6.27875	6.52694	6.34475	5.75222	5.67586
CLTB	6.63309	6.58516	6.36218	5.95956	6.73213	6.29027	6.72038	6.18963	7.04139	6.49757	6.77509	5.29997	5.75529	5.65467	5.75668	5.44492
FAF2	3.93415	3.94659	4.10914	4.25101	4.66897	3.95607	5.06033	3.78558	4.22545	3.82305	4.387	4.51021	4.13446	3.73314	4.1394	4.06621
RNF44	4.12371	4.0887	4.01495	3.73234	3.8448	2.88608	3.16514	2.87607	3.52308	3.77816	3.39011	3.22381	2.45189	2.63511	2.53806	3.01829
CDHR2	-0.90139	-0.50905	-0.78238	-0.66362	2.56669	1.80301	2.31519	1.38964	-0.49967	-0.15574	0.00293	-0.24654	-1.46795	-0.825	-0.16849	-0.1862
GPRIN1	2.97103	3.23405	3.18179	2.89319	3.52319	2.35411	2.95207	2.45176	3.79443	3.41854	3.81322	3.17446	1.95182	2.963	3.67664	3.62415
SNCB	-0.92799	-1.30764	-0.34455	-1.3472	-0.04263	0.73529	-0.4861	0.69475	1.43742	0.76915	1.32471	-1.09919	-1.31076	-1.31463	-1.6365	-0.58566
TSPAN17	3.77167	3.85455	3.69133	3.61537	4.37501	3.38993	3.8927	3.2097	4.52595	4.36612	4.17973	4.10784	4.13851	3.9263	4.37672	4.52285
UIMC1	3.2604	2.92597	2.67062	2.87095	3.03373	2.36931	2.984	2.43772	3.18351	2.87751	2.88661	3.07677	3.01445	2.6959	2.63547	2.37359
ZNF346	1.94229	1.07568	1.34092	1.47316	2.04389	1.04214	1.54288	1.15547	2.43655	1.9639	2.38211	2.45149	2.1057	1.39734	2.00779	1.50978
FGFR4	2.57684	2.87456	2.62622	2.61015	7.7342	7.45174	6.76244	7.51574	4.0785	4.43063	4.66623	3.8893	4.1973	5.30317	5.631	5.78122
NSD1	2.3777	2.2829	2.19415	2.46774	3.2834	1.8729	3.45927	2.03253	3.63903	3.41571	3.52941	4.21218	3.10115	2.93593	3.61632	3.76248
RAB24	4.20487	4.14151	4.22249	4.26105	4.80582	5.1975	4.43335	5.06002	3.40392	4.00095	3.83516	3.72376	3.48551	3.74319	2.95961	2.70455
MXD3	6.2178	6.06312	5.89976	5.74247	6.34943	6.4204	5.80922	6.28132	5.74861	5.33405	5.70261	4.71125	5.30777	5.05222	4.89462	4.61608
PRELID1	8.2204	8.15525	7.97793	7.54459	8.65971	8.22462	8.08908	8.01664	8.02509	7.58115	8.02522	6.49374	7.22116	6.65239	7.12767	6.67148
LMAN2	6.84078	6.86536	6.97312	6.81487	7.81039	8.13216	7.56371	7.97038	6.90798	7.00927	7.0667	6.39941	5.39212	5.75017	5.85509	5.76313
RGS14	3.03464	3.25584	3.19485	3.21509	5.00483	5.49742	4.18489	5.49743	1.44015	2.23038	1.59557	0.92759	1.86354	2.32614	2.50129	2.33539
F12	-2.96546	-3.32193	-3.32193	-2.27823	5.73507	4.38746	4.26886	4.45963	2.20038	2.68966	2.3894	1.4492	-2.41247	-3.32193	-3.32193	-3.32193
GRK6	5.05982	4.86612	4.83062	4.26982	6.03863	4.91347	5.29924	4.90528	5.21563	4.60778	4.86769	4.08844	4.71657	4.33947	5.28274	4.95682
PRR7-AS1	2.29009	1.73395	1.27763	0.82407	-0.01247	0.56947	-1.18205	1.07586	0.215	0.03417	0.35803	0.76589	0.09103	-0.65682	-0.17767	-0.01374
PRR7	4.66766	4.52719	4.38955	3.66751	3.94746	4.94848	3.21706	4.64394	5.73747	5.93381	5.85223	4.79458	3.37768	3.48699	3.71206	3.34261
DBN1	6.10809	6.39137	6.48263	6.30229	5.48835	5.46457	5.22355	5.37017	5.94992	6.19178	6.24721	5.93309	5.52755	5.73738	6.54505	6.69585
PDLIM7	6.34152	6.70884	7.01379	6.51425	4.24571	4.94642	3.91731	4.63083	4.95182	5.28941	5.58851	5.09346	4.99496	5.24019	5.86901	5.51069
DOK3	1.16423	1.56314	1.52837	0.58717	0.12617	0.82906	-0.20691	1.30705	-0.9667	0.53664	0.18141	0.02246	-1.10236	0.0249	-0.22914	-0.39626
DDX41	5.1491	5.13697	5.12872	4.7819	4.74563	4.7806	4.31271	4.71766	5.46961	5.71294	5.29414	5.08956	4.31557	4.35915	4.57607	4.52201
FAM193B	3.75763	3.74071	4.20022	3.40139	2.92858	3.94278	1.81562	4.22319	2.47799	2.45681	2.93017	2.1573	1.47606	1.91627	1.8676	1.82048
TMED9	7.45891	7.46937	7.58004	7.332	7.91318	8.20415	7.64286	7.97789	7.13047	7.05666	7.04696	6.34901	5.99535	5.83445	5.6629	5.74954
B4GALT7	3.95767	4.06805	4.03821	3.56954	3.20978	3.9304	2.36686	3.97335	2.92038	3.26893	3.54269	2.63405	1.59683	1.72661	1.66361	1.81885
N4BP3	1.48863	2.35868	2.81898	0.00349	1.41963	0.68711	1.62798	-0.35248	0.31325	0.41244	-0.05957	-0.47962	-1.12023	-0.3445	-0.35383	-0.17659
RMND5B	5.13595	4.82332	4.68431	4.35627	4.40785	4.47259	3.97936	4.36778	4.64752	4.17002	4.36418	3.52091	4.18434	4.07869	3.80181	3.62004
NHP2	7.96342	7.63003	7.44	6.89682	6.92057	6.91549	6.55374	6.75082	7.34521	6.69817	7.14758	5.22964	7.02373	6.71126	6.71247	6.38912
HNRNPAB	8.50757	8.26469	8.00171	7.9617	8.01639	7.37258	8.06166	7.35123	7.62066	6.24505	7.14786	5.72464	7.42093	7.16334	7.24627	6.67414
PHYKPL	3.81802	4.07153	3.80494	4.33406	2.94028	3.43089	3.12332	3.6019	2.58884	2.27199	2.54872	2.68314	2.82151	2.68186	2.19264	1.81042
CLK4	1.26407	1.29431	1.35615	2.05076	0.96737	1.13714	1.73833	1.12783	0.29525	1.08488	0.35276	1.64477	0.1543	0.50869	-0.3709	0.06524
RN7SKP70	1.70058	1.5816	1.80444	2.87397	1.8589	1.58286	1.83806	1.4983	-3.32193	1.88368	2.08867	1.74759	-0.00366	1.35572	0.55303	0.28442
ZNF354A	2.37379	2.25335	2.63533	2.60966	1.47618	1.18923	1.54678	1.17163	1.48952	1.76758	2.11386	2.16369	1.42999	0.95135	1.44822	0.81019
ZNF354B	1.61335	1.68384	1.68437	1.93968	1.74514	1.61892	1.49773	2.03136	0.77552	0.71082	0.59937	0.86915	1.66935	1.54163	1.50926	1.1111
ZNF879	-1.47457	-0.54943	-1.38023	-0.10056	-0.12827	-0.20433	0.58429	-0.34111	-3.32193	-3.32193	-3.32193	-3.32193	-0.17961	0.17607	0.02289	0.18114
ZNF354C	1.09543	0.8532	0.77764	0.9065	-1.98683	-2.41879	-1.67016	-1.49045	-2.58477	-2.24708	-2.7901	-2.82836	-3.16829	-3.32193	-2.69173	-2.81612
ADAMTS2	-1.31327	-0.64592	-0.62606	-1.02038	2.00349	-0.10883	1.54735	-0.71835	-3.32193	-3.32193	-3.32193	-3.32193	-3.32193	-3.32193	-3.32193	-3.32193
RUFY1	3.21177	3.04435	3.23777	3.46248	3.50715	3.0141	3.65129	3.15258	2.9872	2.95372	3.18658	3.51123	2.96812	2.78783	3.22714	2.94212
HNRNPH1	5.58379	5.23021	5.40462	5.43633	5.37906	4.96801	5.58818	5.03386	5.26741	4.33908	5.16655	4.70039	5.70739	4.88757	5.64477	4.85745
MAML1	3.80441	3.55521	3.73499	3.49774	3.58284	2.45632	3.26636	2.61537	4.15155	3.54536	3.84007	3.82566	3.38762	2.67608	3.68872	3.27958
MIR1229	5.49482	4.8648	4.74157	5.90059	4.7974	4.89088	4.3991	4.74724	2.98998	2.94347	3.04555	2.12607	3.53054	3.411	1.16853	0.26022
TBC1D9B	4.17962	3.88675	4.15735	3.48235	5.06876	3.79442	4.62467	3.61181	5.05019	4.70499	5.06516	4.58811	3.70214	3.02924	4.32466	3.55722
RNF130	1.72601	1.68636	1.96109	1.90788	2.77356	2.43622	2.8798	2.33517	2.44179	2.57961	2.68274	3.00648	1.39372	1.54411	1.72571	1.6188
MAPK9	2.98979	3.06331	3.12346	3.22592	3.13353	2.39061	3.3431	2.3533	2.89607	2.31878	2.53413	2.91216	2.74118	2.5217	2.6396	2.34288
GFPT2	5.58444	5.07492	5.63714	5.5111	-0.84911	-0.75512	-0.7735	-0.92997	1.947	1.264	2.26089	2.61679	-0.74639	-1.98793	-1.58843	-2.04332

CNOT6	3.38498	3.21815	2.99533	3.42249	3.43502	2.21769	3.5092	2.42498	4.04765	3.15859	3.0107	3.71681	4.15216	3.66606	3.50763	3.33041
MGAT1	4.86082	4.68543	4.93477	4.2588	5.46481	4.41122	4.82528	4.02363	4.5538	4.03267	4.92714	3.73761	3.46274	2.31858	3.63337	3.05618
HEIH	3.02391	3.5223	3.33894	3.29669	-0.06194	-1.56702	-1.01542	-1.62809	4.05239	4.5587	4.4595	3.4014	2.96208	3.51507	3.33666	3.21959
LINC00847	2.26733	2.57521	2.58136	2.7421	-1.15401	-2.3513	-1.85099	-2.23177	3.40845	3.76074	3.65772	2.99249	2.70486	2.96916	2.60435	2.71896
ZFP62	1.73819	1.75678	1.74089	1.73186	1.99268	1.42234	2.10829	1.76521	2.04247	2.13083	1.77152	2.74233	1.8211	1.89661	1.90869	2.19958
TRIM7	-1.18949	-1.72827	-0.82718	-1.73561	2.25177	1.85956	1.91936	1.83168	2.62602	2.3177	2.2095	1.54473	2.13494	1.62709	1.77921	1.34834
TRIM41	2.55511	2.35653	2.69222	2.27184	3.1247	2.32112	2.49996	2.25684	2.09945	1.74674	2.52088	1.16578	1.48904	1.04205	1.58757	1.04131
SNORD96A	6.51517	6.09733	6.28692	5.95678	5.73036	6.43493	5.16449	6.29261	5.44273	4.57191	5.45492	5.14938	5.73354	5.68605	4.7709	5.07276
SNORD95	4.28563	3.69049	4.76255	2.86873	4.42146	3.67739	3.27627	3.77351	3.01077	3.84878	4.41837	2.14665	-3.32193	1.47142	2.15667	2.18752
TRIM52	1.28798	1.261	1.55114	1.9881	1.22672	1.70356	1.26749	1.75608	-0.20112	-0.01507	0.25154	0.4628	0.69566	0.91224	0.52986	0.70937
TRIM52-AS1	3.01109	3.1219	3.01171	2.70106	2.51364	3.00551	2.36867	3.20461	2.69493	2.91647	2.31835	2.31152	3.27263	3.64205	2.86934	2.82045
RPL23AP45	-1.21198	-1.80522	-0.53655	-0.09324	2.68681	2.90259	1.67627	3.3165	-1.04238	-1.94388	0.17732	0.74169	0.18824	1.28111	-0.16542	0.09897
DUSP22	2.50312	2.79027	3.08073	2.69502	3.11282	3.35859	3.12106	2.89811	1.83137	2.03146	2.2867	1.98878	1.99727	2.53622	2.54773	2.74276
EXOC2	3.751	3.33373	3.81629	3.71114	3.5834	2.59757	3.96062	2.29222	4.67958	3.90969	4.7556	4.70689	3.71098	2.85057	4.13529	3.10119
FOXQ1	3.75253	3.97445	4.50834	3.88988	3.63125	0.4409	3.41597	0.13683	-0.11955	0.22604	-1.54059	-1.3704	-1.68656	-2.98577	-3.32193	-3.32193
FOXF2	-0.24255	0.32783	0.39973	0.38913	-2.78373	-3.32193	-3.32193	-2.81391	4.22206	3.6621	4.0884	2.11734	-3.32193	-3.32193	-3.32193	-3.32193
MIR6720	2.85353	3.42852	3.38139	4.32879	-3.32193	-3.32193	-3.32193	0.05931	6.45206	5.76345	6.20824	3.5061	-3.32193	-3.32193	-3.32193	-3.32193
FOXC1	2.44296	2.72758	2.40581	1.70904	3.22402	2.75187	2.58615	2.4241	2.82485	2.8728	3.24792	2.16521	1.71826	1.67195	2.20506	1.95649
GMDS	3.15671	2.92001	2.75818	2.97501	4.57678	5.15816	4.35048	4.92934	2.99658	2.62496	2.93135	2.42363	2.02494	2.45718	2.22107	1.98324
GMDS-DT	-2.32099	-1.68247	-1.53856	-1.15371	-1.25088	-0.68025	-1.72059	-0.66103	-2.6918	-1.9488	-2.43111	-2.63111	-1.7446	-1.30949	-1.69868	-1.22558
WRNIP1	5.30731	5.20043	5.20308	4.99167	4.73754	4.48573	4.42472	4.37955	5.37163	5.18092	5.27942	4.75137	4.81703	4.6818	4.48079	4.32662
SERPINB1	4.025	4.42234	4.65944	5.02216	4.67051	4.58902	5.15559	4.33056	4.75086	4.65859	4.57914	4.98117	3.81078	3.81172	3.58532	3.45211
SERPINB9P1	-1.25513	-0.94992	-1.26919	-1.73061	-0.49497	0.82063	-0.52398	0.30813	-1.29001	0.51882	-0.11086	0.64288	-0.54994	-0.31211	-0.60321	-1.28189
SERPINB9	0.63341	0.58127	0.25592	0.63352	2.90305	2.37514	3.0405	2.23126	3.69349	2.46834	1.97334	3.39702	2.0413	0.3693	1.36916	0.41389
SERPINB6	4.71303	4.75754	4.91269	4.82473	4.69237	4.95947	4.85074	4.8091	5.16157	5.10876	5.05247	5.43025	4.5073	4.34097	4.28877	4.39126
LINC01011	-0.44412	-0.35518	-0.28696	-0.6831	-0.21391	1.40073	-0.11291	0.8801	-1.24835	-0.60141	-0.91857	-1.36618	-0.50297	-0.50738	-1.35857	-0.84155
NQO2	3.71448	3.70831	3.62474	3.97948	4.37924	4.9157	4.04251	4.85741	3.1017	2.96091	3.00793	3.54982	1.89097	2.26523	1.55589	1.79146
HTATSF1P2	4.22287	4.3598	4.48511	4.30887	1.57196	2.1944	1.32829	1.85244	4.46709	4.55248	5.02643	5.12298	3.05148	2.887	2.22647	2.18304
RIPK1	3.23832	3.35761	3.37866	3.90593	3.73813	3.06235	3.90987	3.18759	3.79209	3.19203	3.69654	4.16665	3.58659	2.89647	3.55632	3.42641
BPHL	1.46398	1.71372	2.10425	2.47463	2.2815	2.93517	1.90588	3.13558	2.45541	2.38613	2.69706	2.74283	2.53122	2.47811	2.95248	2.71083
TUBB2A	6.98524	7.26107	7.27694	6.6843	7.48794	8.30558	6.88749	8.31474	8.59801	8.08142	8.59366	7.29391	6.96027	7.40673	7.38414	6.99039
TUBB2BP1	4.4253	4.93094	4.80579	3.23826	5.60449	6.18061	4.0504	6.07468	6.31926	5.81494	6.5851	3.78499	4.31656	4.58669	4.80759	4.37216
TUBB2B	5.92523	6.24369	6.19109	5.49551	6.72591	7.82041	5.7929	7.87137	8.27117	8.03197	7.97814	7.04028	5.62974	6.21155	6.10142	5.97246
PSMG4	2.84793	2.98059	2.97254	2.45155	3.01484	3.52499	2.64964	3.44761	1.42249	2.25601	2.2278	1.10779	0.96788	1.73198	1.77168	1.52464
SLC22A23	2.85324	3.18385	3.32337	2.75034	4.58258	4.07821	4.04348	3.92453	-1.45956	-1.47096	-1.3955	-1.45561	-1.44811	-1.53481	-1.44816	-0.35669
PXDC1	5.3423	5.3128	5.39694	4.77539	4.75061	4.4248	5.13071	4.07498	4.64626	4.88136	4.78116	4.25128	4.57539	4.69963	4.63111	3.97951
FAM50B	0.83772	0.70755	0.69863	0.39738	1.21285	2.45996	0.37844	2.18384	4.56563	4.75403	4.59169	4.01533	1.76746	2.44126	2.0585	2.39909
PRPF4B	3.42571	3.20688	3.34509	3.86286	3.61033	3.45465	4.31743	3.33234	3.65147	3.6115	3.73084	4.15257	3.99713	4.46006	4.20689	4.02254
C6orf201	4.5202	4.50618	4.56535	4.48464	4.71257	4.79629	5.15966	4.62893	5.90966	5.55881	5.7386	5.28395	5.18843	5.34235	5.43036	5.21299
ECI2	4.66226	4.68514	4.70177	4.61428	4.7309	5.17071	5.02738	5.0006	5.89816	5.62007	5.71507	5.39967	5.26836	5.39745	5.4279	5.24394
CDYL	3.92838	3.64615	3.83438	3.85166	2.94254	2.31728	3.31922	2.06168	4.45166	4.0532	4.29027	4.27248	3.7163	3.5476	3.70262	3.33571
RPP40	3.98946	3.7463	3.34709	3.85121	2.83579	2.43814	3.28353	2.72092	3.61338	2.57879	3.15551	3.12517	3.38126	2.23301	2.74617	2.24256
PPP1R3G	0.63704	0.34673	0.75937	-0.0914	-2.70144	-0.70902	-2.40673	-1.18894	2.28688	2.61818	2.30742	1.72628	-2.32822	-3.00354	-2.28134	-2.26504
LYRM4	2.84164	2.57704	2.52784	2.6916	2.06971	2.2986	1.63215	2.07574	2.38423	2.28427	2.14021	2.0069	2.35319	2.1847	2.11684	1.83387
FARS2	3.43243	3.42415	3.28113	3.25704	2.53065	1.95297	2.25819	1.82999	3.42861	3.84414	3.69965	3.56127	2.65697	3.24104	3.05221	3.31585
NRN1	0.92622	1.15892	0.84669	0.86833	-2.54966	-3.32193	-3.32193	-2.4795	-3.32193	-3.32193	-2.89393	-3.32193	-3.32193	-2.29264	-1.94814	-3.32193
RREB1	2.20671	2.29022	2.16539	2.01468	3.65686	2.52664	3.47968	2.68706	2.85434	2.6942	3.04766	2.82301	1.55253	1.52309	2.54752	2.66095
SSR1	4.78293	4.77867	4.73299	5.09851	5.40183	5.58026	5.88125	5.33729	4.66865	4.22775	4.54979	5.01438	4.49872	4.18398	4.4645	4.34916
RIOK1	3.98665	3.80594	3.39573	4.13211	3.60608	3.70545	3.92666	3.8638	3.64085	3.03831	3.31985	3.38746	3.977	3.83289	3.20334	3.16475
DSP	-0.97836	-0.84311	-0.92091	-0.82123	5.96399	5.0966	6.3176	5.13763	3.28058	3.24152	2.66208	3.75708	2.28786	2.2321	3.57	3.51016

SNRNP48	2.10069	2.03049	2.18865	2.04	2.05888	1.86248	2.5106	2.01951	2.30854	1.95721	2.2992	2.38365	2.55384	2.08483	2.10099	1.9644
BMP6	-0.60305	-1.07089	-0.2729	-1.26121	3.3823	4.268	3.6917	3.03017	-2.34116	-3.32193	-3.32193	-2.70904	1.28704	0.19674	0.1543	-0.21284
BLOC1S5	2.20746	2.19134	2.11895	2.39842	2.85981	2.39518	3.04084	2.46732	2.89151	2.74737	2.20514	2.68381	3.3173	3.34889	2.95321	2.65866
EEF1E1-BLOC1S	6.43807	6.24976	5.86212	6.22118	6.1585	5.98288	6.42686	5.79784	6.00267	5.83562	5.74968	5.08805	6.81914	6.76356	5.75324	5.68041
EEF1E1	5.26337	5.05563	4.67726	4.99789	4.68247	4.91612	4.98947	4.85442	4.58444	4.40351	4.36397	3.63131	5.50208	5.50975	4.19514	4.20137
SLC35B3	2.90861	3.03773	3.32138	3.65199	3.99187	4.0033	4.36052	3.87781	3.26911	3.30831	3.27709	3.61108	3.73974	3.50171	3.23157	3.14634
OFCC1	-2.34466	-2.14017	-2.50216	-2.0429	-1.81025	-0.37838	-1.16284	-0.88447	-2.6776	-2.93084	-3.32193	-1.89773	-2.85782	-2.31595	-3.32193	-2.61685
TFAP2A	2.10061	2.17516	2.33672	2.31042	1.49903	1.34823	1.72175	1.23293	4.71125	4.47915	4.72884	4.12938	3.34572	2.85504	3.48765	3.13586
TFAP2A-AS1	1.01902	1.17783	0.96175	1.20191	0.29855	1.32864	-0.19974	1.38387	3.33966	2.9297	2.95428	1.65256	1.92653	1.85167	2.16332	2.09185
GCNT2	2.79579	2.77214	2.41136	2.97944	2.80882	2.66959	3.36984	2.63587	2.65425	2.72803	2.25951	2.86506	3.11194	3.38841	3.01292	3.55003
C6orf52	0.89215	0.11827	0.43101	0.72393	-0.37487	0.4252	-1.22495	0.05635	1.16547	0.77279	0.7967	-0.38488	1.76619	2.05092	1.24077	0.27954
PAK1IP1	5.3748	5.16448	4.911	5.36253	5.11577	5.1283	5.61593	5.23028	4.93177	4.82646	4.84008	4.76556	5.44541	5.34193	4.61299	3.86954
TMEM14C	7.70753	7.72328	7.77622	7.82172	7.21812	7.29937	7.36976	7.08246	7.60938	7.6056	7.4544	7.41908	8.02708	8.01171	7.29457	7.17117
TMEM14B	5.77884	5.81767	5.66624	5.63604	4.92336	5.05805	4.94738	4.83741	6.1309	5.89836	5.73018	5.46047	6.15448	5.98365	5.30367	5.15198
MAK	0.43173	0.46261	0.94685	1.03386	-0.77697	-0.9239	-0.58701	-0.21	-0.31392	-0.74693	-1.17639	-1.46931	-0.09773	-0.26678	-0.75886	-0.91558
SYCP2L	-3.32193	-3.32193	-3.32193	-3.32193	0.1784	1.37605	0.44539	1.43805	-3.32193	-3.32193	-2.83047	-3.07618	0.73583	0.57683	0.07565	0.56206
ELOVL2	0.12472	0.98135	0.32219	0.75986	6.07577	4.77243	6.39948	4.90842	3.95563	3.35999	3.31273	3.52286	4.13951	4.31554	3.95474	3.96349
ELOVL2-AS1	-3.32193	-3.32193	-1.86872	-2.62827	0.7472	-1.16448	0.8019	-0.22401	-1.18092	-1.57435	-1.30767	-2.06441	-1.44146	-0.94356	-0.50023	-1.0535
SMIM13	3.88926	3.74066	3.55946	3.94863	3.80597	3.48515	4.19045	3.15093	3.92292	3.73363	3.65369	3.9033	4.61451	4.58395	3.98866	4.00848
NEDD9	-1.16928	-1.14181	-0.36849	-1.31236	1.66766	2.62474	1.80977	2.08232	-2.98706	-2.53745	-2.43147	-2.37832	2.61958	2.34405	3.08832	2.27217
TMEM170B	0.2905	0.41065	0.06441	0.77984	1.14334	0.71284	1.65121	0.9002	0.57697	1.34167	-0.13977	1.75786	1.82053	2.73491	2.36983	2.71922
ADTRP	3.56389	3.60024	3.59269	3.65411	-3.21166	-3.07207	-3.32193	-3.32193	-1.75807	-0.90829	-1.97149	-1.19478	-2.20283	-2.79789	-3.32193	-2.87302
HIVEP1	1.72348	1.91899	1.99797	2.15252	0.77841	-0.54353	0.53876	-0.22309	1.49558	1.15057	1.09974	1.88963	1.62271	1.55137	1.7221	1.84147
EDN1	2.84815	3.5125	2.33052	2.36746	5.45485	1.07529	5.72842	2.76019	6.06166	4.99911	5.03763	4.06278	5.88822	5.73515	4.28201	4.01407
RPL15P3	6.39703	6.41088	6.54988	5.2606	4.64516	5.30011	4.63446	4.69586	5.4677	6.26925	5.29719	5.15024	4.77022	4.48313	4.67373	4.55453
PHACTR1	2.1524	2.25325	1.92723	2.41711	-2.52625	-1.34256	-2.39158	-0.46093	1.23617	0.50926	1.04273	0.37387	-1.40766	-2.64823	-2.23451	-1.75563
TBC1D7	3.77472	4.35334	4.21555	5.07954	1.96809	2.42387	2.11837	2.36751	1.79016	1.12029	1.31769	1.00424	1.41756	1.33541	0.68597	0.9585
GFOD1	1.05852	0.77057	0.76592	0.45887	-1.29173	-2.41027	-0.79945	-2.01825	-0.55108	-1.65693	-0.72713	-2.25041	0.69531	0.14214	0.2239	-0.3062
GFOD1-AS1	3.00867	2.94658	2.81226	1.60934	0.57529	-3.32193	1.19037	-1.40335	1.86094	0.00245	1.99086	-3.32193	2.48395	2.16343	2.44801	2.07256
SIRT5	1.6013	1.68073	1.7743	2.07404	0.90143	1.44836	0.88595	1.33681	0.96922	1.46904	1.01974	1.84556	1.66218	1.7757	1.26323	1.74306
NOL7	7.10386	6.8607	6.81264	6.9398	6.5058	6.49076	6.63105	6.13925	6.59686	6.19487	6.46441	6.29876	6.71981	6.41796	6.43226	5.92919
RANBP9	4.92084	4.68721	5.03042	5.17183	4.33349	3.41601	4.53307	3.36139	4.51744	3.57908	4.32121	3.89456	4.68962	3.28782	4.41791	3.30057
MCUR1	3.97686	4.19551	3.95779	4.20124	3.87516	3.76961	4.15687	3.58898	3.59214	3.09411	3.26367	3.35998	4.01808	3.86731	3.99372	3.63131
RNF182	0.16955	-0.13708	0.20992	1.02884	-3.32193	-3.32193	-3.32193	-3.32193	3.54323	3.77374	3.10304	4.3073	6.20424	6.43449	5.77267	5.84471
CD83	3.91922	3.44256	3.30182	3.34355	2.52654	2.25707	2.83362	2.06151	3.72557	3.05023	3.56078	2.79002	4.41623	4.4062	3.78972	4.14708
JARID2	4.82502	4.79125	4.98211	4.91267	2.50276	2.22221	2.79267	2.30733	3.40376	4.01241	4.21201	4.72908	1.81008	2.27025	2.97021	3.14382
DTNBP1	3.9241	3.7381	3.71976	3.81401	1.98786	2.42515	2.02295	2.50985	3.38889	3.13837	3.40751	2.99259	1.54056	1.93064	1.61451	1.436
MYLIP	1.01378	-0.56826	-0.32686	0.60537	1.5753	1.52023	1.34277	1.94247	2.00941	2.25953	1.02513	2.07028	3.40664	4.77542	3.75886	4.06015
GMPR	2.70773	2.31574	2.1986	2.32666	-3.12321	-2.05511	-3.32193	-2.45231	2.53579	2.17826	1.48628	2.36275	-1.9244	-2.69629	-2.10286	-1.71976
ATXN1	1.725	1.845	2.08913	2.09685	2.28045	2.25948	2.54349	2.00093	2.45096	2.4335	2.80443	3.26586	1.62804	1.79639	2.50871	2.88656
RBM24	2.50813	2.75538	2.38573	2.27827	3.22722	2.18367	3.52636	2.30787	3.57136	2.95406	2.98562	2.82059	4.35819	4.7818	3.79837	3.74232
CAP2	3.34355	3.47168	3.49174	4.03071	3.91381	2.91527	4.19583	2.82373	5.13335	4.62226	5.10565	5.15476	4.91452	4.77803	4.79746	4.0918
RPL7P26	0.71722	0.18878	0.39997	-3.32193	-1.01903	0.98063	-2.35047	-0.06339	-0.29005	1.42262	-1.25189	-0.76635	-0.0188	-1.24668	0.11472	-0.31627
FAM8A1	1.72366	2.33755	1.72445	2.37589	5.30778	4.6085	5.22555	4.26348	2.76817	3.79469	2.90866	3.81289	3.02402	3.55567	3.12938	3.64251
NUP153	4.81023	4.79444	4.71096	4.58894	4.6435	3.0022	4.94807	2.96919	5.13925	4.52584	4.86561	4.95621	5.30989	5.08022	5.42212	5.27043
KIF13A	2.28434	2.18994	2.35979	2.49321	3.51837	2.50561	4.03374	2.31094	4.13132	3.79525	4.12222	4.50272	3.24623	3.39245	4.01354	3.84276
NHLRC1	0.46197	0.7225	1.30588	1.10331	1.63501	2.00346	1.74628	2.18109	2.97916	2.49224	2.74774	2.35265	1.50173	1.39695	1.32555	1.5003
TPMT	4.36763	4.28051	4.0128	4.25115	4.1024	3.95358	4.52285	3.67273	3.65612	3.05095	3.39763	3.31852	4.26986	3.40526	3.7317	2.90324
KDM1B	3.04287	3.14089	2.60482	3.43064	2.76097	1.48453	2.815	1.53231	3.61503	3.27942	2.64999	3.78727	3.70649	3.69111	3.09323	3.4392
DEK	5.85205	5.6981	5.72161	6.04232	5.7779	4.3116	6.25205	4.46968	6.7114	5.97799	5.86095	5.92447	6.64705	6.38225	6.34191	5.67253

RNF144B	3.27775	3.28971	3.59189	3.65051	-1.52114	-1.82642	-1.34379	-1.9404	1.0001	0.85321	0.58331	1.57829	1.95292	0.98908	1.36579	1.37186
ID4	-1.10712	-0.68897	-2.19971	-1.07038	4.67779	5.67487	4.20624	5.47266	-3.32193	-2.92217	-3.32193	-3.32193	-3.32193	-3.32193	-3.32193	-3.32193
MBOAT1	2.68174	2.19134	2.12236	2.53103	3.21232	0.92097	3.45456	0.10792	2.04365	1.07206	1.28123	1.28552	3.15576	2.91907	3.43267	4.24575
E2F3	3.70989	3.59823	3.58641	4.07658	3.24439	2.87445	3.90679	3.05711	3.47098	3.13918	3.54643	4.0192	4.22525	4.11073	4.28649	4.17174
CDKAL1	2.28973	2.1365	1.92461	2.13071	2.25616	2.2128	2.4552	1.97501	2.34459	1.92282	1.96667	2.43934	2.8417	3.32387	3.28941	3.59274
SOX4	4.67829	5.82594	6.0756	6.31671	3.7084	4.9824	4.22835	5.18325	2.61168	3.39723	3.44611	4.33476	5.46788	5.84648	7.19665	7.27439
CASC15	-3.01904	-3.06292	-2.15492	-1.70633	-0.37297	1.82	0.28402	1.54954	-3.13826	-2.35298	-2.81247	-2.03533	0.116	0.35193	0.28121	0.74736
DCDC2	-3.1136	-2.89033	-2.86977	-2.84454	4.28325	3.59881	4.54951	3.65004	3.57402	3.59683	3.463	2.28001	4.0148	3.60771	3.71884	2.88299
KAAG1	-3.32193	-3.32193	-3.32193	-3.32193	4.50379	3.69293	4.49082	3.52634	3.77375	3.85848	3.77139	1.69648	2.56256	2.5575	2.13404	1.93608
MRS2	3.52933	2.88704	3.2322	3.33111	4.69106	3.74583	5.03446	3.79263	3.59109	2.90259	3.45556	3.32054	5.51243	5.49828	5.91991	4.99204
GPLD1	0.86379	0.66683	0.69073	1.23312	2.19417	1.42457	2.38144	1.53417	1.45466	1.00132	1.25598	1.47373	3.14995	3.02991	3.43638	2.54675
ALDH5A1	0.61621	0.83759	0.39494	0.95924	2.44981	1.76345	2.22535	2.03325	1.96512	2.04374	1.9356	2.73088	2.89965	2.97524	2.93815	3.1672
KIAA0319	0.72026	0.65047	0.73475	1.11292	1.76744	1.36943	1.76257	1.43296	-3.151	-3.04847	-3.32193	-3.18103	1.54752	2.28979	2.17697	2.48321
TDP2	4.32695	4.33602	4.31973	4.76981	4.97241	5.37504	5.54575	5.07879	4.44945	4.16382	4.2822	4.46043	4.63686	4.44123	4.18621	4.11083
ACOT13	3.80381	4.12655	3.90077	4.59052	4.34408	5.01503	4.45805	5.00811	3.29442	3.608	3.30053	3.59364	3.26402	3.97602	3.01977	3.44268
C6orf62	5.79497	6.04699	6.00803	6.41292	5.94091	5.39339	6.37458	5.21338	5.79061	6.33866	6.03826	6.58059	5.73565	6.45976	6.26403	6.58153
GMNN	5.25376	4.98293	4.98146	4.87104	5.04769	4.32899	5.12109	4.6049	5.21263	4.31019	4.57317	3.48335	5.58971	5.59191	4.79612	4.61711
PPIAP29	7.03067	6.57881	6.7211	5.39638	5.16602	7.16983	4.79857	6.6196	6.42886	7.10154	6.08947	6.92245	4.6414	4.23252	4.10242	4.63461
CARMIL1	1.76511	1.51979	1.77689	2.00708	3.33235	1.25413	3.66979	1.42207	2.42428	2.39275	2.38089	3.08521	3.72848	4.21443	4.48183	4.26254
SCGN	-3.32193	-3.32193	-3.32193	-3.32193	2.74541	-0.44341	2.87477	-0.46815	-3.32193	-3.32193	-3.32193	-3.32193	-2.45755	-0.40715	-0.73696	-1.29819
SLC17A4	-3.32193	-3.32193	-3.32193	-3.32193	0.38115	-2.57562	-0.10662	-2.32464	-3.32193	-3.32193	-3.32193	-3.32193	-3.14402	-3.32193	-3.32193	-3.32193
SLC17A1	-3.32193	-3.32193	-3.32193	-3.32193	2.05791	-0.48548	1.98671	-1.61788	-3.32193	-3.32193	-3.32193	-3.32193	-0.65415	0.42754	0.49066	-0.78164
SLC17A3	-3.32193	-3.32193	-2.99328	-3.32193	1.53927	-1.08566	1.54699	-1.12157	-3.32193	-3.32193	-3.32193	-3.32193	-1.18981	0.4482	-0.32012	0.17965
TRIM38	2.15493	2.20959	2.19033	3.0444	-1.50583	-1.65859	-1.32135	-2.07523	1.96878	2.17907	2.42724	3.33476	2.19423	2.6461	2.47184	2.82458
HFE	1.5242	1.56322	1.26348	1.98816	3.28505	3.41471	3.17162	3.63576	2.33906	2.36484	2.25131	2.36271	2.04564	2.02556	1.69902	2.51905
BTN3A2	3.50217	3.44944	3.17282	3.80729	2.17441	3.1066	1.92345	3.14035	4.21315	4.85942	4.69824	5.3459	1.15879	2.0569	1.39255	2.49543
BTN2A2	2.70155	2.50995	2.60031	2.66282	1.93929	1.83633	1.39237	1.6855	1.87474	2.56691	1.91915	2.34466	2.51515	3.3131	2.28099	2.95589
BTN3A1	2.14973	1.87994	2.1569	2.33505	2.48763	1.8112	2.16617	1.79838	3.52975	3.42564	4.06645	3.8088	1.77456	2.01543	1.71341	2.27439
BTN2A3P	1.08768	0.92109	1.14617	1.21932	0.40399	0.46204	-0.07421	0.53417	0.64951	0.99946	0.28264	1.16927	1.14452	1.35942	0.86456	1.16325
BTN3A3	1.94291	1.76333	1.85857	2.36153	0.22171	0.54515	0.3966	0.69362	2.10745	2.71841	2.93195	3.44363	0.77566	1.18508	1.46393	1.76824
BTN2A1	4.48925	4.19703	4.43863	4.42775	4.56343	3.90276	4.30494	3.90395	3.66965	3.73993	3.62149	3.53868	4.52329	4.41158	4.1321	4.16509
BTN1A1	-2.97927	-3.32193	-3.32193	-3.32193	-0.50747	1.03676	0.29409	-0.05747	-2.93058	-2.72067	-3.32193	-2.72901	-1.59496	-1.42637	-1.59787	-0.26442
HCG11	-3.32193	-3.32193	-3.10603	-2.89537	1.53071	1.9072	2.08622	2.14109	0.56355	1.56772	0.83166	1.92873	-3.32193	-3.1761	-3.32193	-2.83763
HMGN4	5.14457	5.30324	5.23105	5.2916	5.80365	5.40442	6.14766	5.06624	6.82704	6.8485	6.607	6.58188	6.48368	6.61848	5.966	5.70701
ABT1	4.58319	4.37901	4.09005	3.90538	4.9058	4.84458	4.3412	4.74592	4.96399	4.71926	4.7583	3.96749	4.6263	4.97506	4.12206	4.27134
ZNF322	1.90737	1.93982	1.98963	2.23553	2.74422	2.07929	3.41139	1.77441	2.76657	3.18969	2.92021	3.48688	2.96645	3.22242	3.40627	3.27523
GUSBP2	-0.76811	-0.29501	-0.66023	-0.30465	0.8631	0.51215	0.94038	1.01198	0.44704	0.00496	0.04507	-0.06999	0.28174	0.67084	0.13991	0.42591
PRSS16	-2.30258	-2.74273	-1.96334	-1.47675	2.60302	2.95775	2.55227	2.86583	-2.30528	-0.71474	-1.30767	-0.51857	-2.21799	-1.06506	-0.90445	-0.91724
ZNF204P	-1.76477	-1.49657	-3.32193	-2.45997	2.30466	3.37567	2.60509	3.2179	-2.86107	-2.92991	-2.90463	-2.38018	-3.32193	-3.32193	-3.32193	-3.32193
ZNF391	-3.10711	-3.32193	-3.32193	-3.32193	0.80748	1.06811	0.93614	0.97926	-1.26099	-0.71096	-1.15431	0.25807	1.30142	1.50834	1.40305	1.6649
ZNF184	1.31414	0.80427	0.85456	1.41868	2.12613	1.31488	2.41174	1.31906	2.42198	2.31219	1.94858	2.53098	2.40069	2.33589	2.7641	2.54233
ZNF165	1.20797	1.32075	0.88408	1.00471	0.51576	1.72374	0.91682	1.72172	-0.6069	-0.50667	-1.05412	-0.0395	-0.69096	0.21901	-0.19478	0.32225
ZSCAN12P1	-1.39201	-1.95614	-1.90794	-2.19589	-0.84287	0.55912	-0.17302	-0.18451	-1.42578	-1.43051	-2.34341	-1.44844	-0.98952	-2.02176	-1.27566	-0.43456
ZSCAN16-AS1	1.89688	1.71441	2.12134	2.16794	2.32833	3.16806	1.86802	2.99146	1.15515	1.78921	1.02354	0.73528	1.48472	1.94614	1.70121	1.87641
ZSCAN16	2.51362	2.92182	2.73934	3.12632	2.16778	2.82224	2.02941	3.01684	1.04964	2.32279	1.06896	1.79893	2.76229	3.42971	2.93221	3.21447
ZKSCAN8	1.78002	1.78216	1.74735	1.94222	1.71976	1.40694	2.00605	1.44549	2.09148	2.39892	2.41098	3.58666	2.51961	2.78984	2.8851	3.2374
TOB2P1	0.51606	1.19873	0.82247	0.66131	2.08865	2.9731	2.27709	3.01844	2.29861	2.50663	1.66897	2.89584	-1.29878	0.99519	-0.51956	0.02318
ZSCAN9	1.93687	1.56972	1.95318	2.09702	1.37849	2.13038	1.67332	2.42148	1.34193	1.41435	1.71809	1.76406	2.52653	2.65856	2.22033	2.18142
ZKSCAN4	2.0764	2.17039	2.05835	2.9813	1.40044	1.52377	1.58393	1.66476	1.97689	2.58322	2.05741	2.60384	1.58157	2.3577	1.94261	2.33427
ZSCAN26	1.53851	1.78918	1.43404	1.82405	1.97124	2.07732	2.09571	2.28807	2.26483	2.53363	2.17422	3.16272	2.64069	3.07817	2.50676	2.73531

PGBD1	2.22701	2.33506	2.32021	2.97728	2.2109	2.28813	2.52494	2.57684	2.45514	1.72723	1.92935	2.2911	3.24718	3.36486	2.99353	3.31766
ZSCAN31	-2.25429	-0.71399	-1.03052	-0.16712	-0.1877	-0.72124	-0.09997	-0.03613	-2.02566	-1.06657	-1.6123	-0.55879	-0.43862	0.04111	0.09985	0.18775
ZKSCAN3	0.41601	0.62225	0.35318	1.1796	0.98964	0.65515	1.02156	1.08467	0.07066	0.95557	0.90822	1.49568	1.3918	1.91313	1.68793	1.94052
ZSCAN12	0.15955	0.0936	-0.22939	0.20413	0.58272	0.55673	0.89024	0.74772	0.5806	0.77158	0.61721	1.31229	1.40327	1.60068	0.91075	0.9815
ZSCAN23	-3.32193	-3.32193	-3.32193	-3.32193	-0.55145	-1.37023	-0.88539	-1.10303	-2.45453	-1.51144	-1.75672	-0.5041	-3.32193	-3.32193	-3.32193	-3.32193
ZBED9	-1.67475	-1.55928	-1.86951	-0.53783	1.34767	0.98029	1.79019	0.48515	-3.32193	-3.32193	-3.32193	-3.32193	-0.63736	-0.15036	-0.62794	-0.14196
TRIM27	4.78576	4.75719	4.57539	4.68327	4.48202	4.48133	4.29041	4.22756	3.93593	3.97732	4.01418	3.95116	3.67288	3.54093	3.53214	3.64771
HCG15	-1.99763	-0.93773	-2.4028	-0.79913	1.22392	0.39683	1.39353	0.52782	-2.41569	0.06042	-1.58327	-0.577	0.06031	0.34955	0.55016	0.34964
ZNF311	-2.74783	-1.5276	-1.36026	-0.9108	-3.17947	-3.32193	-3.32193	-3.32193	-3.32193	-3.32193	-3.32193	-3.32193	-0.61	0.33761	-0.20102	0.59167
OR2I1P	-0.94473	-1.23534	-1.29161	-0.08916	3.21779	0.84533	3.05721	0.93106	0.76336	2.03198	2.30467	3.51101	-3.32193	-2.78417	-2.5644	-3.32193
UBD	1.01992	0.76791	0.69832	2.08619	5.21905	2.89985	5.06569	3.11725	2.24719	3.61902	3.79261	4.5391	-3.32193	-1.63061	-1.18165	-3.32193
GABBR1	0.22909	0.54359	1.27688	1.1635	2.63094	2.67388	2.38971	2.35429	0.67005	1.72573	1.87545	2.15727	-0.40878	0.62754	-0.08948	0.63928
SUMO2P1	3.00454	3.66403	3.51497	3.28685	2.28055	2.49215	2.2301	2.21742	3.7823	3.86729	3.32317	2.97675	2.4469	1.01436	2.66948	0.97585
HLA-F	3.87107	3.67124	4.03615	3.63727	3.6232	3.93537	2.94643	3.78795	4.09379	4.66204	4.5853	4.49412	1.25252	1.46736	0.75457	1.13304
HLA-F-AS1	0.86963	1.09952	1.0414	0.81935	0.43736	0.89925	-0.36569	0.37054	-0.29978	-0.39095	-0.78343	-0.55427	-0.6476	-0.41042	-0.92808	-0.59546
RPL23AP1	2.42573	2.23995	2.70979	2.71119	1.13718	1.70731	0.41198	1.779	1.57193	1.48608	1.10329	0.86262	0.17984	0.00064	-1.95833	-0.14557
MICE	1.75999	2.13475	1.93929	1.61536	2.02149	2.41207	1.07293	1.40771	0.15527	-0.5518	-0.46311	-1.25856	-0.00956	0.19825	0.32517	-0.84107
HLA-V	0.7803	0.81691	1.30509	-0.05107	-2.95335	-2.82033	-2.50005	-2.59758	-0.23641	0.45306	-0.137	-0.63089	-3.32193	-3.17526	-3.32193	-3.32193
HCG4	2.40883	2.57246	3.13101	1.4598	-2.32855	-1.95872	-1.3571	-1.81099	2.09021	2.79164	2.20523	1.49966	-3.32193	-2.63748	-3.32193	-3.32193
HLA-G	0.55061	0.24738	0.54203	-0.6088	2.93258	3.72093	2.77383	3.59582	4.58167	5.29112	5.15855	4.95533	1.64846	2.04292	1.50182	1.80482
HCG4P8	0.17575	0.10808	-0.40206	-0.73724	-0.89632	0.28809	-1.09042	-0.80848	1.08759	1.75866	1.53078	1.13892	-3.32193	-2.62751	-3.32193	-1.59138
HLA-H	7.06008	7.12506	7.45622	6.70013	5.72797	6.29593	5.20626	5.93462	7.72659	8.43278	8.15606	7.85947	4.11716	4.42952	4.22961	4.57307
HCG4B	2.9295	3.26207	3.18587	2.38987	0.17774	0.17555	-0.26701	0.39063	1.63416	2.70471	2.28887	4.10233	-1.8157	-3.32193	-3.32193	-1.98363
HLA-K	3.87214	3.83368	4.20728	3.27116	0.87436	1.36495	0.35324	0.67834	4.16614	5.127	4.57134	5.34647	-1.5885	-1.59209	-2.04881	-1.11391
HLA-U	6.15048	6.09758	6.53974	5.28837	-1.13786	-0.93818	-3.32193	0.12778	0.63696	0.38639	1.83111	-0.16628	2.58116	3.75009	3.63808	2.43124
HLA-W	-1.31476	-1.61718	-0.52223	-1.49754	-0.70597	-1.71996	-0.40021	-0.51735	2.18469	3.06194	2.46536	3.90563	-3.32193	-2.48534	-3.32193	-2.33472
ZNRD1ASP	0.32771	0.07543	0.19323	0.02318	0.98649	1.10684	0.55722	0.83964	2.146	2.50633	2.45954	1.19264	0.61854	0.39996	0.0181	-0.02136
HLA-J	1.78667	1.50113	2.28459	0.85745	3.37596	3.31897	2.86494	2.79305	4.96377	5.39433	5.34891	3.7451	1.47876	1.37336	1.61829	1.59408
ZNRD1	2.91859	2.5226	2.52586	2.47765	2.96715	3.59303	2.57999	3.41054	2.82819	3.04485	2.88582	2.14665	3.18974	2.84366	2.15012	1.95403
PPP1R11	4.75708	4.80228	4.57689	4.79393	4.9697	5.11678	4.88269	4.68199	5.06508	5.12133	5.02971	5.1029	4.82383	4.83206	4.38861	4.64145
RNF39	2.3983	2.42008	2.25347	1.74081	2.30923	3.22476	1.94254	2.82544	2.13551	2.48441	1.97339	1.11627	2.24201	2.28654	0.85846	1.70427
TRIM31	-3.32193	-1.52937	-2.98838	-1.51685	2.43944	2.91813	2.21713	3.05307	-3.32193	-3.32193	-3.02627	-2.81983	-3.32193	-3.32193	-3.32193	-3.32193
TRIM31-AS1	-3.32193	-3.32193	-3.32193	-3.32193	2.98281	2.73877	2.95489	2.62136	-3.32193	-3.32193	-3.32193	-3.32193	-3.32193	-3.32193	-3.32193	-3.32193
TRIM10	-3.32193	-3.32193	-3.32193	-3.32193	0.641	-0.82439	0.67032	-0.57191	-3.32193	-3.04566	-3.32193	-3.32193	-3.32193	-3.32193	-3.32193	-3.32193
TRIM15	-3.32193	-2.89285	-3.32193	-3.32193	3.87263	3.09686	3.62218	2.94446	-3.32193	-3.32193	-3.32193	-2.95171	-3.32193	-3.32193	-2.94938	-2.94214
TRIM26	4.54184	4.41654	4.31084	4.24907	5.3262	4.6928	4.43851	4.99186	4.76022	4.2305	4.47355	4.0361	4.2261	4.22205	4.08421	4.54681
HCG17	1.10106	-0.14655	0.39116	1.02596	0.69599	-1.70474	0.86243	0.85019	-2.19193	-1.01619	-2.27977	-0.77426	1.82567	1.31889	2.07053	1.43001
HLA-L	1.16541	0.929	0.90342	1.07804	1.25538	1.29396	0.78131	0.98412	1.67682	1.9609	1.72819	1.14517	0.47745	0.56142	0.29946	0.03111
HCG18	2.03502	2.03544	1.95072	2.30135	2.04645	1.29343	2.08874	1.38978	1.30964	1.23125	1.39201	1.36699	1.60206	1.29319	1.62112	1.36209
TRIM39	2.99284	2.86673	2.8896	2.98002	3.24181	2.76229	3.13611	2.70534	2.58812	2.34381	2.74864	2.3096	2.61893	2.72753	3.0046	2.95137
TRIM39-RPP21	5.50451	5.41032	5.33974	4.86127	5.2484	5.75697	4.48908	5.74697	5.2472	4.97242	5.21628	3.7491	4.76402	4.30714	4.45129	4.20418
RPP21	5.45562	5.34984	5.28871	4.67813	5.07461	5.78317	3.94835	5.7645	5.12162	4.86455	5.05492	3.10021	4.5239	3.86656	4.00164	3.57167
RANP1	2.73489	1.89257	1.54836	0.81191	1.99955	2.15038	1.7818	2.07459	2.94892	2.7587	2.53137	1.60337	0.90658	0.2831	1.24884	0.04368
HLA-E	7.05067	7.10869	7.14498	7.00498	6.16251	6.20961	5.67091	5.94196	7.39676	7.99086	7.75566	7.94297	5.57098	5.0904	5.08426	5.47158
GNL1	3.93607	3.9724	4.00183	3.88177	4.34138	4.40364	3.95038	4.23557	4.39729	4.48977	4.57029	3.92889	2.71279	3.36498	3.27015	3.65039
PRR3	2.16002	2.45743	2.17777	2.57506	1.78632	2.70389	1.69939	2.98293	2.5595	2.88899	3.09616	2.97291	1.90697	3.00712	2.23395	2.64949
ABCF1	6.19139	6.07331	5.97398	6.39264	6.2503	5.62968	6.23491	5.71289	6.32447	5.59694	6.19052	5.77883	5.80102	5.62863	6.2092	5.91327
PPP1R10	4.12786	4.04031	3.95185	3.78029	4.6535	4.55542	4.32644	4.71979	4.76683	4.28135	4.72929	4.18596	4.11832	3.75439	4.2596	4.16116
MRPS18B	6.54369	6.38327	6.21854	6.22452	5.54203	5.68456	5.22339	5.7439	6.0701	5.95093	5.95167	5.94566	6.02365	6.05587	5.82516	5.70627
ATAT1	1.8938	1.92286	1.92282	2.25161	1.34163	2.10457	0.74846	2.00437	1.83408	1.33901	1.51085	1.49412	1.83425	1.75418	1.84367	1.93227

C6orf136	4.30582	3.93715	3.82434	3.6489	3.77467	4.29736	2.98786	4.38172	3.11458	3.17066	2.80918	2.32138	3.55526	3.77481	2.68537	2.96131
DHX16	5.05284	4.89988	4.86511	4.75436	4.98553	4.36997	4.85631	4.358	4.84225	4.79853	5.28007	4.54134	4.15044	4.91633	4.84674	4.90301
PPP1R18	6.553	6.55971	6.67279	6.41907	4.01003	4.23272	3.87646	3.85317	6.2317	6.42365	6.18118	6.2221	6.3337	6.14818	6.48393	6.5092
NRM	4.58327	4.60262	4.83145	4.40794	4.10853	4.49	3.41373	4.89299	4.93048	4.77618	4.55657	3.95408	4.50253	5.15592	4.85514	5.28084
MDC1	4.33943	3.86547	3.8026	3.3275	3.75653	2.45452	3.3779	2.96636	3.80793	2.9258	3.22381	2.82558	3.83012	3.73262	4.1633	4.33086
MDC1-AS1	5.58663	5.14541	5.09227	4.48567	5.13041	3.69665	5.09439	4.37427	4.89152	4.13279	4.38601	4.12492	5.24476	5.12551	5.43211	5.64022
FLOT1	5.51881	5.66964	5.79996	5.48786	6.72484	7.00863	6.12927	6.9467	6.52552	6.25634	6.29058	5.41095	5.44731	5.72752	4.91628	4.8783
IER3-AS1	10.1531	10.4592	10.0432	10.6208	6.88265	8.56002	7.69821	8.12665	9.01374	9.44963	8.77415	10.1861	8.13523	8.20226	7.45134	7.51157
LINC00243	-0.4987	-0.59563	0.26446	0.74808	-3.32193	-3.32193	-3.32193	-3.32193	-2.15418	-2.29747	-2.24399	-3.32193	-1.62568	-2.24039	-2.89828	-2.06162
DDR1	2.38694	2.683	2.71174	2.85437	5.078	3.93307	4.49568	3.80678	5.20475	5.88845	5.58641	5.57994	4.24567	4.36341	5.35385	5.58044
GTF2H4	4.50845	4.36268	4.24598	4.17365	4.02998	4.71952	3.439	4.56071	4.59558	4.82661	4.79584	4.43174	4.59904	5.10746	4.80094	4.87889
VARS2	3.52601	3.51245	3.41381	3.23109	4.22946	4.17657	3.50585	4.18637	4.15422	4.43715	4.55767	4.09817	3.54337	4.06014	3.98111	4.31826
PSORS1C1	0.14653	0.71106	0.98319	0.54187	-2.52182	-2.4624	-2.41333	-2.75726	1.05786	1.31905	1.78734	1.31033	-1.30395	-1.69149	-1.90708	-1.31714
CDSN	0.80096	1.44805	1.72538	1.14508	-2.72104	-3.32193	-2.43312	-3.32193	1.61795	1.93526	2.28947	1.68424	-1.28724	-1.56743	-1.46863	-0.79874
CCHCR1	2.99257	2.77576	2.91618	2.73331	3.50474	3.29802	2.92998	3.37477	3.57449	2.87285	3.26553	2.35609	3.96952	3.9622	4.23209	4.0401
TCF19	4.92575	4.69509	4.74506	4.04389	4.32531	2.95456	3.95023	3.4396	5.17712	3.7402	4.34197	2.94308	4.63404	4.53719	4.57469	4.19178
POU5F1	-1.55132	-1.83402	-0.16205	-3.32193	0.96736	0.36988	0.37936	0.67642	1.40837	0.97136	1.06965	1.19214	2.3013	2.29625	2.24223	2.53639
PSORS1C3	-1.46689	-3.32193	-1.97111	-3.32193	0.6788	-0.3928	0.10749	-1.96764	0.69715	-0.45198	0.99868	1.22239	2.44144	2.68584	2.72199	2.78015
HCG27	-0.71541	-0.64898	0.30471	-0.22432	-0.74671	0.35694	-0.9455	0.67429	-0.46101	-0.75912	-0.99066	-1.09229	-1.68444	-1.00762	-1.88083	-1.24666
RPL3P2	1.76141	1.51181	1.6587	-0.12903	0.38471	0.42155	-0.08452	0.49006	1.63208	2.32618	1.3254	1.57266	0.27733	-0.72826	0.19804	0.3743
MIR6891	7.73973	7.77667	8.02307	7.97061	3.42398	1.79706	2.98001	1.62191	6.3841	7.07998	7.44902	6.89204	3.48731	3.71473	3.49248	2.89328
MICA	3.42885	3.04931	3.23663	3.18474	5.19318	5.4693	5.00189	5.42907	4.42627	4.21285	4.08022	3.517	5.41386	5.71034	5.23446	5.22367
HCP5	0.81321	0.4903	0.44358	1.17273	-0.31512	-0.07365	-0.51404	-0.73672	0.21872	1.22768	0.82001	1.55437	-0.29172	-0.87813	-1.38321	-0.90388
MICB	4.51559	4.41503	4.17335	4.43299	4.69551	4.16035	4.54222	4.07554	5.2375	4.29863	4.61449	4.42659	6.23187	6.28518	5.53665	5.52044
PPIAP9	4.74147	5.40111	4.42746	5.85654	-3.32193	-3.32193	-3.32193	-3.32193	-3.32193	-1.99837	-3.32193	-3.32193	-1.10071	-1.10476	-3.32193	-3.32193
MCCD1	-1.9294	-1.42024	-2.349	-1.29413	-1.54959	-1.91954	-2.5075	-1.3585	-2.36243	-0.62466	-0.72466	-1.29275	1.65814	1.53179	-0.82227	-0.0404
ATP6V1G2-DDX3	6.75416	6.45554	6.41781	6.4362	6.30283	6.23006	6.29185	6.22464	7.51088	6.82371	7.46956	6.95369	6.64341	6.66212	6.54795	6.53943
DDX39B	5.53086	5.24625	5.31405	5.31612	5.05652	5.10115	4.99423	5.20796	6.00341	5.40223	6.00364	5.5803	5.38857	5.47238	5.2155	5.3249
SNORD84	3.81048	4.48777	4.82741	4.65664	5.00975	2.83639	4.65407	2.84819	4.21764	4.94505	5.00245	4.50708	6.01795	4.88834	5.6837	5.84272
ATP6V1G2	2.91585	3.10778	2.98864	2.63576	3.08971	2.39327	2.53669	2.1927	3.02245	3.6807	3.34675	2.21965	1.86395	2.1247	2.74987	2.19539
NFKBIL1	4.96173	4.89598	4.98314	4.62429	4.67152	4.80619	4.03468	4.5355	4.58402	5.17941	4.84339	4.29432	3.44631	3.58325	4.00102	3.827
LTB	1.92258	1.68764	1.31965	1.40771	4.81699	3.68136	4.42492	3.49643	0.35097	0.39935	-0.01386	-0.1284	4.06153	2.62724	2.12038	2.8976
APOM	3.55359	3.90218	3.84152	3.26343	6.53561	8.08525	5.98101	7.73228	4.93231	4.6274	5.05858	4.45039	4.66109	4.47128	5.01476	4.92145
C6orf47	4.19529	4.56571	4.55034	4.56885	4.77971	4.47241	4.3923	4.48089	3.89357	3.91344	4.08957	3.87738	4.27871	4.26443	4.40037	4.42973
C6orf47-AS1	4.35841	4.86907	4.64188	4.84699	5.1362	4.88181	4.90813	4.85324	4.05204	4.11577	4.33417	4.39852	4.81751	4.94057	4.73526	4.89228
GPANK1	5.13612	5.33087	5.04969	4.93644	4.47467	4.7256	4.13882	4.46803	4.69634	4.69935	4.76898	5.0457	3.53334	3.79776	3.67413	3.62489
CSNK2B	6.65175	6.65131	6.61893	6.66702	5.68914	6.64118	5.39374	6.64019	6.25918	6.02327	6.1445	6.28476	5.55247	5.61517	4.98782	5.02729
LY6G5B	4.05005	3.62231	3.9776	3.65277	2.67977	3.59228	1.76488	4.17058	2.50227	1.95526	2.595	1.71368	2.63656	2.6033	1.86587	2.24725
ABHD16A	3.00092	3.14174	2.99674	3.26063	4.78502	5.24232	4.16976	5.06742	3.51041	3.7993	4.0323	3.82444	3.02724	3.85061	3.2238	3.84961
DDAH2	5.06053	5.83916	6.13122	5.79133	5.91339	6.45184	5.35369	6.19965	5.30967	5.74869	5.72889	5.27695	5.70153	6.27554	6.43984	6.17687
MSH5	1.96704	1.53344	2.06545	1.47084	2.06235	3.0739	1.06158	3.35036	1.60981	1.13811	1.28356	1.36653	1.52594	1.46112	1.24508	1.37329
MSH5-SAPCD1	2.81483	2.14879	2.72894	2.21533	3.20295	4.3612	2.67405	4.43412	2.20497	1.75362	1.95143	1.92469	2.00769	2.0846	1.76271	2.0054
SAPCD1	2.81327	1.87999	2.3726	1.7537	3.38282	4.70631	3.31001	4.64393	0.63088	0.3261	1.00764	-0.48537	1.48262	1.49768	0.3129	1.42123
SAPCD1-AS1	2.7368	2.66353	2.90301	2.43651	5.50109	6.38678	5.46791	6.18961	0.89624	0.7853	1.69568	0.46132	2.06651	2.1917	1.13066	1.95558
VWA7	-2.00383	-1.55873	-0.78534	-0.53012	4.82116	4.42029	4.00995	4.11303	-3.00234	-1.61576	-2.79385	-2.17563	-1.73427	-1.00089	-2.46217	-0.68857
LSM2	5.90479	5.49415	5.41335	5.42452	5.69638	5.71989	5.43581	5.7016	5.9464	5.41138	5.71647	5.35348	6.1568	6.12864	5.44342	5.32857
HSPA1L	1.03739	1.81731	1.37512	1.07223	2.25231	2.12324	2.12816	2.03263	0.87782	0.78075	0.92709	1.00634	-0.17019	0.67842	0.76724	0.36834
SNORD48	5.70724	5.49241	5.88276	4.08235	5.7733	5.85119	5.08997	6.28519	3.96397	3.82097	4.03839	3.02633	6.07797	5.09508	3.54574	3.25812
SNORD52	4.97162	4.19397	5.50425	4.8014	4.40972	5.55301	4.57824	5.85717	2.45572	3.17572	3.97271	2.16755	4.03872	4.38533	2.4931	2.78295
NEU1	5.608	5.51689	5.67441	5.78193	6.16949	7.13368	6.21945	6.95598	3.56442	4.07707	4.06957	4.13009	4.51954	4.80053	4.52434	5.13449

EHMT2	4.90069	4.77088	4.81468	4.72498	4.41737	4.09221	3.48569	4.36219	5.06575	5.12673	5.19028	4.62876	3.57939	3.66783	4.51101	4.5099
EHMT2-AS1	6.45108	6.41522	6.37807	6.61198	5.69233	5.57427	5.04108	5.72287	6.22562	6.37446	6.4637	5.86518	4.64621	4.94484	5.57228	5.81103
C2	1.79855	2.22136	2.51448	2.96324	0.32095	0.61509	-0.71224	0.30322	-0.05339	0.22404	0.4319	0.25074	-1.21928	-0.92461	-0.78124	-0.56788
ZBTB12	2.76674	2.78307	3.07668	2.00687	2.78363	2.63467	1.62065	2.87525	2.76294	3.56986	3.32888	3.11996	1.74108	2.25863	2.91261	2.85793
CFB	3.21883	4.18688	4.07265	5.59964	0.75679	2.69853	-0.11032	2.37302	-2.14322	-1.49568	-2.03027	-0.39104	-2.2549	-2.17627	-2.40374	-2.2786
NELFE	5.94784	5.79427	5.69788	5.51018	5.74347	6.12213	5.27852	6.13428	6.15988	5.87937	5.79402	5.52675	5.452	5.4608	5.31693	5.37674
SKIV2L	5.08585	5.19825	4.98602	5.02527	4.50441	4.91336	3.82901	4.81933	4.29766	4.43414	4.73005	4.5668	3.88709	3.94758	4.19604	4.39171
DXO	4.35172	4.11778	4.10194	4.09984	4.1274	5.05811	3.4572	4.91715	2.96393	3.36286	3.51107	2.89699	2.73612	2.88671	2.74957	2.79974
STK19	3.11079	3.1665	3.0984	2.79475	3.3522	4.19212	2.8644	3.88834	2.43724	2.92514	2.96845	2.25979	2.78917	2.85914	2.35155	2.51893
C4A	-0.48358	-0.55327	-0.36273	-0.12422	-1.11366	1.27109	-1.77884	0.8228	-0.25125	0.51821	0.17458	0.08648	-1.24705	-0.66475	-0.90836	0.2057
C4A-AS1	-0.6928	-0.73944	-0.89192	0.11385	-1.21622	1.73173	-2.89346	1.20362	-0.91427	0.27194	-0.27025	-0.8454	-1.2541	-1.25802	-1.38473	-0.90556
TNXA	-1.05641	0.27661	0.0669	-0.37136	-2.75126	-0.02093	-3.32193	-0.63588	-2.7932	-0.08219	-1.26671	-1.36284	-2.00477	-2.64876	-2.25789	-2.52014
STK19B	2.74867	2.98991	1.44077	2.61742	2.43601	4.04122	2.80053	3.46434	-0.06628	2.61585	2.10149	1.69524	2.75015	2.86365	1.85274	0.619
C4B	-0.68149	-0.73238	-0.42262	-0.22217	-1.20585	1.28559	-1.87264	0.77281	-0.44639	0.34189	0.05351	-0.00067	-1.15095	-0.69114	-0.81453	0.27133
C4B-AS1	-0.6928	-0.73944	-0.39907	0.44649	-0.97404	1.95721	-2.89346	1.36039	-0.59991	0.5435	-0.14253	-0.59528	-0.75294	-1.59354	-1.25729	-0.55972
TNXB	-2.06432	-1.04473	-1.25906	-1.10861	-2.79279	-1.22944	-2.96778	-2.01322	-1.49788	-0.14434	-1.21972	-0.55796	-2.3703	-2.52547	-1.65209	-1.76652
ATF6B	5.51521	5.22575	5.46483	5.14278	5.20233	5.57649	4.34971	5.28978	5.61333	5.51347	5.83223	5.07604	3.80616	3.76813	3.77239	4.19459
FKBPL	3.75354	3.66276	3.51707	3.75527	3.45051	3.78771	2.78383	3.81072	3.82633	3.11589	3.53323	2.68831	3.08891	3.34273	2.81593	2.86945
PRRT1	1.67648	1.82155	2.03286	1.46848	2.39924	2.1704	1.6984	1.63755	1.73036	2.37674	2.18685	1.72004	0.86919	1.4111	1.207	1.55777
PPT2	4.61271	4.64152	4.70713	4.60256	4.7348	5.06107	4.16118	4.75703	4.0485	4.57903	4.55851	4.36605	3.46203	3.66343	3.3936	3.65765
PPT2-EGFL8	5.14797	5.17967	5.13811	4.91447	5.71006	5.65079	5.18021	5.43603	4.7821	4.87579	5.02287	4.78786	4.08181	4.34127	4.58187	4.85165
EGFL8	3.61013	3.60069	3.42825	3.07017	3.01989	4.61299	2.64068	4.79979	1.42301	2.0448	2.17546	2.77932	2.61627	3.15122	2.18227	2.59069
AGPAT1	5.62429	5.66111	5.59593	5.39621	6.26201	6.15822	5.75677	5.95197	5.35125	5.28443	5.48136	5.35224	4.57665	4.80558	5.25168	5.51339
MIR6721	8.65581	8.75039	8.60766	8.238	9.56216	8.85989	8.75582	8.46096	8.6563	8.56783	8.70991	7.93871	7.06917	7.22042	7.97361	8.23095
RNF5	6.82807	7.07427	6.79868	6.97755	7.22744	7.24584	6.78618	6.93114	6.71026	6.87505	6.76678	6.34289	6.49457	7.02712	6.54221	6.69954
AGER	1.18098	1.07942	1.43932	0.99052	1.14452	2.84098	0.28234	2.90263	0.87764	1.48671	1.20569	1.00928	1.00206	2.03434	0.88608	1.80376
PBX2	4.60024	4.5148	4.75505	4.5949	5.15858	5.09387	4.717	5.06635	4.72992	5.00885	5.25445	4.91069	4.78755	5.31346	4.89715	4.9319
GPSM3	4.35072	5.12585	4.96329	5.13276	-0.74224	1.37696	-0.38781	0.06492	1.80987	3.80178	2.6154	3.49204	1.72857	2.76536	1.0965	3.01915
NOTCH4	-1.78049	-1.79754	-1.89011	-1.42904	-2.76278	-2.91921	-3.03155	-2.36068	-2.60137	-2.50001	-2.52092	-2.85999	-2.32337	-1.44513	-3.21623	-1.80353
HLA-DRA	-3.32193	-2.57298	-2.5409	-3.32193	-3.32193	-1.81646	-3.32193	-3.32193	2.58982	4.95561	3.28884	4.93109	-3.32193	-3.32193	-3.32193	-3.32193
HLA-DRB5	-3.32193	-2.56374	-3.32193	-3.32193	-1.96037	-3.32193	-3.32193	-3.32193	2.57797	3.53893	2.72436	3.1754	-3.32193	-3.32193	-3.32193	-3.32193
HLA-DRB6	-3.32193	-3.32193	-3.32193	-3.32193	-3.32193	-3.32193	-3.32193	-3.32193	-0.02262	1.49015	0.57975	1.31379	-3.32193	-3.32193	-3.32193	-3.32193
HLA-DRB1	-2.61572	-2.04835	-2.00213	-2.47617	-1.93831	-2.60955	-3.32193	-1.99871	4.36314	5.20481	4.54779	5.02182	-3.32193	-2.74374	-2.18059	-3.32193
TAP2	5.27787	4.8265	4.50591	4.28057	4.40655	3.70933	4.0259	3.63827	5.54064	4.78784	5.542	4.76577	4.7225	4.4496	4.26035	4.21831
PSMB8	5.73079	5.57181	5.35869	5.61732	0.44916	2.5193	0.19828	2.60463	6.47588	6.48006	6.82435	6.21741	4.07569	4.70623	3.90559	4.49359
PSMB8-AS1	4.786	4.80076	4.6832	5.00403	3.62434	3.52138	3.20707	3.81818	5.18944	5.22147	5.52769	5.2972	4.27269	4.39442	3.97012	4.01193
PSMB9	5.04525	4.92395	4.95369	4.8845	0.80933	-0.34064	0.35517	-0.0418	5.82499	5.47377	6.08003	4.92474	3.04866	3.55046	3.02794	2.76158
TAP1	4.91934	4.95872	4.95095	5.05813	4.82874	3.61304	4.56554	3.69654	6.19122	6.16399	6.56007	6.13217	4.89585	5.02637	4.90123	5.01323
HLA-DMB	-3.09946	-3.07444	-3.06187	-3.04635	1.82719	2.27912	0.62982	2.30992	-1.21942	0.50422	-1.22375	0.23241	0.21166	1.88958	0.88832	1.14741
HLA-DMA	0.83557	0.74963	0.41546	1.05097	2.05154	3.7982	1.60632	3.76717	2.70226	3.48988	3.00009	3.56442	-0.84709	-0.33392	0.00937	-0.291
BRD2	5.08849	4.86088	4.86355	4.9361	5.43812	5.11861	5.27124	5.32589	5.39159	5.42706	5.57501	5.41105	4.84086	5.03528	5.11564	4.91457
HLA-DOA	-3.06679	-3.32193	-3.32193	-3.32193	-3.20802	-1.75832	-3.32193	-1.7033	-3.32193	-0.48279	-2.32048	-0.20052	-3.32193	-3.32193	-3.32193	-3.32193
HLA-DPA1	-2.83128	-1.99107	-2.75643	-2.89872	-3.09291	1.33917	-2.85923	1.40791	1.67439	3.04907	1.92855	3.08695	-3.17686	-3.32193	-3.32193	-3.14335
HLA-DPB1	-2.96795	-3.32193	-3.32193	-2.88785	-1.85125	2.03297	-2.49109	2.09338	1.15918	1.68282	1.02154	0.77237	-3.32193	-3.32193	-3.32193	-2.97555
COL11A2	-3.02291	-2.84919	-2.16631	-2.66066	-2.23459	-1.74698	-2.80596	-1.71102	-0.71639	-0.32273	0.00949	-0.49144	-3.08262	-2.46672	-1.64439	-2.16095
RXRB	3.55359	3.33947	3.3047	3.41584	3.88983	3.78161	3.15333	3.62316	4.48098	4.53733	4.61031	4.12457	3.96306	3.65755	3.8584	3.8361
SLC39A7	6.31707	6.16428	6.22353	6.12309	7.7249	7.74017	7.44734	7.50599	5.86195	5.75141	6.0358	5.53741	6.30076	6.24911	6.16808	6.17376
HSD17B8	2.99768	3.17424	2.69159	2.46951	2.28844	3.55583	1.43211	3.36028	3.64424	4.14371	3.88016	3.48005	1.43094	1.55859	1.64958	2.14844
RING1	5.34844	5.12859	5.36701	5.069	5.27362	5.96712	4.55896	5.9327	5.565	5.63773	5.88175	5.03564	5.04921	4.94298	5.24661	5.35793
HCG25	4.27093	3.98975	3.95964	3.98074	4.33311	4.94875	3.83627	4.93556	4.34033	3.91448	4.31313	4.37269	4.0028	4.17719	4.10851	4.1843

VPS52	3.59028	3.33927	3.28362	3.43364	4.24127	4.09662	4.06761	3.98401	4.17076	3.81192	4.19191	4.17317	3.88534	4.05155	4.05069	4.15061
B3GALT4	-0.16712	1.236	1.37727	1.09608	1.69952	2.8267	1.13158	2.64422	1.58981	1.27437	1.31547	1.11178	-0.58257	0.20709	-0.28035	1.02065
WDR46	5.84751	5.62162	5.48797	5.27987	5.31441	5.43641	4.92399	5.47069	5.5318	5.49815	5.85268	5.01213	4.58095	4.87352	4.75766	4.68386
PFDN6	5.83824	5.79825	5.69715	5.6742	4.98185	5.64859	4.76081	5.57338	4.99004	4.90352	5.07226	3.28427	5.13777	5.08983	4.68764	4.49455
MIR6834	4.40462	4.20029	5.23115	4.36899	2.96936	4.13242	2.45059	3.52314	3.33966	2.7154	3.00735	0.93862	3.06303	2.77228	1.91045	1.53861
RGL2	3.57619	3.8141	4.17477	3.99235	3.62762	3.7014	3.13141	3.31016	3.97589	4.43239	4.57014	4.23249	4.46245	4.04535	4.78382	4.19876
ZBTB22	2.70073	2.66149	2.90982	2.85742	3.3961	3.73058	2.09403	3.41383	3.32492	4.05208	3.90815	3.05295	3.09172	3.51434	3.20582	3.62894
DAXX	5.14119	5.23756	5.06705	5.1987	5.11616	4.90117	4.78219	4.94113	5.85461	5.53021	5.66042	5.36161	5.324	5.90995	5.31897	5.45023
LYPLA2P1	0.7322	1.57469	0.63526	-0.937	0.06652	0.53899	-1.27834	0.21545	0.72718	1.36356	0.96222	0.6599	-0.67999	-0.90171	-2.27603	-0.63464
KIFC1	5.26505	4.85963	4.89264	3.7217	5.39502	4.45708	4.78803	4.57826	5.99125	5.09301	5.58002	3.87393	5.16191	5.45066	5.20537	5.09016
PHF1	3.58921	3.81782	4.06794	3.91248	4.25356	5.08794	3.5415	4.94256	4.41098	4.58577	4.50774	4.2217	4.16771	4.44965	4.23977	4.3161
CUTA	6.92706	6.84233	7.04701	6.67013	6.79653	7.78188	6.3886	7.73184	7.55959	7.1514	7.19097	6.49673	7.0611	6.9202	6.918	6.8884
SYNGAP1	1.72839	1.99015	1.84344	1.82153	2.13607	2.17239	1.73928	2.0925	2.15233	2.22661	2.25958	2.2785	2.0591	2.33979	2.20685	2.55391
SYNGAP1-AS1	1.42782	1.47706	2.04025	1.24118	1.96651	2.54642	1.48387	2.27502	1.46652	2.32185	2.36662	2.05704	1.57916	2.64253	1.36355	2.03078
ZBTB9	3.16321	3.33632	3.00574	3.11802	3.5326	2.95503	3.26941	2.65234	3.60435	3.21143	3.40012	3.35827	3.44799	3.25518	3.63998	3.65698
GGNBP1	2.27389	2.64814	2.54028	2.4184	3.84693	3.19857	3.21993	2.62258	3.36897	3.60967	3.85752	3.01137	3.00829	3.41531	3.38179	3.55896
BAK1	4.64048	4.74632	4.73672	4.60458	5.61579	5.54361	5.33455	5.16114	5.06088	5.26558	5.54303	4.787	5.20042	5.4828	5.29378	5.60037
ITPR3	5.24111	5.32073	5.45933	5.12612	4.90688	4.05357	4.8982	3.55159	5.17185	5.23194	4.86455	5.57808	4.16911	4.97383	5.13681	6.15031
UQCC2	5.19301	5.15014	5.15273	5.12994	4.37086	5.35876	4.05721	5.24559	4.5812	4.37417	4.2541	3.98677	4.39076	4.49218	4.00833	4.52281
IP6K3	-1.40095	-0.4019	-0.16427	1.33768	-3.32193	-2.9638	-3.32193	-3.32193	-3.32193	-1.33788	-0.98802	-1.35622	-1.86428	-2.25999	-2.98157	-2.97488
LEMD2	3.85635	3.88945	4.0856	3.31172	5.50172	5.37254	4.93054	5.28469	4.27382	4.70038	4.58297	4.06225	3.72397	4.04251	4.08826	4.16879
MIR6835	5.86565	5.61767	5.48019	5.14203	4.97806	4.10983	4.7691	4.23657	5.45276	5.87208	5.33273	5.87604	4.3924	4.0993	4.74038	4.77181
SMIM29	4.07749	4.18854	4.37621	3.9238	3.13187	4.79733	2.81872	4.47885	4.79614	5.31179	5.15354	4.17709	3.36868	4.01714	3.44241	3.76803
RPL35P2	4.08668	3.95034	4.05004	3.48108	2.39941	3.70824	1.62931	2.50962	2.71403	3.75503	2.70559	3.38365	3.52233	3.93437	3.08227	3.70444
NUDT3	3.52662	3.24817	3.18806	3.26541	3.83625	3.81426	4.07775	3.8364	4.08859	3.70272	4.00015	4.19311	3.96235	4.18729	4.44048	4.39742
SPDEF	0.64135	1.16293	0.14905	1.23411	-1.22698	-0.18611	-0.8515	-1.04144	-3.32193	-1.57944	-2.81597	-1.97844	-3.32193	-1.95303	-2.84981	-1.74732
SNRPC	6.98802	6.65712	6.47029	6.57115	6.42458	6.90823	6.22417	6.87736	6.99084	6.70626	6.76765	6.12786	7.19957	7.30418	6.28381	6.26057
UHRF1BP1	2.09861	1.7975	1.72725	1.91786	3.32729	2.28563	3.58717	2.19874	3.05217	2.59567	2.81851	3.23565	3.68257	3.5981	3.96173	3.91555
RNY3P15	2.03585	-3.32193	2.75178	3.35061	3.45603	3.29393	4.67206	1.96595	3.60767	2.24164	3.74007	5.12894	4.88565	5.25554	6.10544	5.23186
TAF11	4.27834	4.14018	3.91785	4.29525	4.68475	4.69909	5.03328	4.97344	4.53045	4.37191	4.4715	4.61751	5.40182	5.38349	4.62772	4.67818
ANKS1A	2.15812	1.94131	2.20729	2.08721	3.17861	1.9924	2.93291	2.00826	3.00846	3.01907	3.0894	3.12077	2.82197	3.00054	3.62727	3.6624
SCUBE3	-1.41764	-0.92598	-1.32194	-0.26436	-0.95251	-0.41364	-0.77947	0.70577	-2.08987	-1.87324	-1.80687	-1.08902	-0.9391	-0.8123	-1.38814	-0.06706
ZNF76	1.81014	1.53468	1.72678	1.69872	1.64377	2.21132	0.80187	2.4802	2.32288	1.99292	2.32278	1.50134	1.99853	1.55479	1.63806	1.4025
DEF6	-1.53965	-2.07756	-2.87695	-1.97714	0.81379	1.64233	0.46569	1.99317	0.08008	0.31832	0.54443	0.73217	-2.75845	-2.35679	-2.41621	-2.94608
PPARD	4.30069	4.3634	4.37223	3.47993	4.14881	5.10348	3.68297	5.02391	4.64713	4.34225	4.20241	3.85912	2.95481	3.08067	3.55106	3.38528
FANCE	2.83183	2.43852	2.50597	2.07203	3.30681	2.45335	2.65163	2.65092	3.45671	2.93187	3.23062	2.42543	2.58596	2.73278	2.75981	2.13749
MIR7111	5.78334	4.80362	5.60373	4.15303	3.88263	4.71464	3.02673	5.06329	4.8539	5.55077	5.02165	5.81516	3.23104	3.22594	1.10992	1.14003
TEAD3	3.5094	3.25363	3.73244	2.93109	4.3047	4.54617	3.75387	4.58748	3.27737	3.39476	3.67468	3.1007	2.26633	2.95196	3.24274	3.1821
FKBP5	1.12998	0.59435	0.25108	0.06975	2.93643	1.99411	3.0196	2.04621	1.33294	-0.07086	0.25934	-0.26219	3.457	3.51369	3.50413	3.71133
LHFPL5	3.70871	3.68904	3.52313	3.73364	2.56882	2.76095	3.39881	2.8371	2.6727	2.05983	2.24587	2.39738	3.36825	3.02528	3.19916	2.69357
SRPK1	4.98975	4.82383	4.84815	4.89968	4.6455	3.83443	5.22268	3.75134	4.68369	3.98915	4.33802	4.6383	5.05352	4.62053	4.96877	4.5933
MAPK14	3.25846	3.09715	3.33998	2.99451	3.79201	2.64218	4.15806	2.01535	3.56442	2.83086	3.78302	3.01621	3.87582	3.08131	4.19895	3.09899
MAPK13	3.36782	3.67364	3.65037	3.55833	-2.38383	0.75864	-2.73379	-0.457	3.95585	3.97255	3.70573	4.33546	-2.43843	-2.04031	-1.86233	-1.46159
BRPF3	2.84338	2.61973	2.909	2.9254	3.89471	2.71003	3.85093	2.29747	3.77188	2.69665	3.87434	3.8621	3.28331	2.23523	3.71867	3.17526
PNPLA1	1.68745	2.0625	1.60393	1.89408	-3.32193	-3.32193	-3.32193	-2.63619	-3.32193	-3.32193	-3.32193	-3.32193	-3.32193	-3.32193	-3.00667	-2.51548
KCTD20	3.39569	3.31174	2.93187	3.717	5.17456	4.26909	5.3741	4.19254	5.38198	5.28548	4.92708	5.90906	5.85164	5.83084	5.72371	5.84661
STK38	2.88605	2.93118	2.97077	3.50251	4.10268	3.81989	4.53702	4.02324	3.94135	3.34578	3.84118	4.50537	5.11268	4.98169	5.39228	5.12246
SRSF3	5.96406	5.69371	5.71516	5.71847	5.629	4.99622	5.8598	5.04675	6.0779	5.39591	5.89872	5.17897	6.0095	5.75457	5.78118	5.33612
CDKN1A	5.47092	6.41493	5.94627	6.53819	3.53458	3.62906	3.83597	2.73721	7.72629	7.84285	8.47637	7.89118	4.9327	4.98932	4.06751	5.61894
CPNE5	0.98254	0.85317	0.36863	0.57826	-2.66859	-3.15333	-3.02456	-3.32193	-1.88353	-0.99427	-1.98611	-1.58872	-2.66535	-3.19006	-3.32193	-3.15888

PPIL1	5.06253	4.43063	4.4953	4.23076	5.17334	4.06219	5.2722	3.89136	5.05288	3.47232	4.85886	3.67152	5.98922	4.00901	5.48561	3.90254
C6orf89	3.17382	3.1335	3.23575	3.4319	3.76641	3.38379	3.94103	3.15643	4.20265	4.36277	4.24174	4.80873	3.87737	4.20098	4.0615	4.28304
MTCH1	6.64719	6.6698	6.70439	6.52106	6.83678	6.43218	6.53716	6.23372	7.43144	7.34383	7.33671	6.9517	7.0452	7.17175	7.42909	7.22768
COX6A1P2	4.3945	4.1949	4.50267	4.01192	3.93382	5.3659	3.18601	5.17735	3.94569	4.48062	4.0799	2.38626	2.05435	1.76754	1.79418	1.11477
PIM1	3.96459	4.17292	4.23161	3.39232	3.30081	2.83017	2.97508	2.5904	6.21318	6.20672	5.83232	4.84273	4.22074	4.46199	4.30842	3.80117
TMEM217	0.33643	0.03281	0.35142	0.74424	1.31996	0.84122	1.7692	-0.03369	1.39942	0.97042	1.23996	0.47543	1.06996	0.9207	1.92944	1.61397
TBC1D22B	2.21055	2.00911	1.76272	2.09093	2.89176	2.44607	3.07887	2.42749	3.3409	3.07431	3.34095	3.17409	3.55173	3.28434	3.48378	3.06516
RNF8	2.12866	2.00305	2.0404	1.794	2.28024	1.91683	2.57842	1.58122	2.61755	2.18607	2.33349	2.40694	3.05839	2.90501	2.75474	2.65474
CMTR1	3.45423	3.32818	3.20993	3.00586	4.24697	3.63629	4.0276	3.61233	4.22049	4.1244	4.21897	4.1997	3.75082	3.69542	4.13226	4.13208
CCDC167	5.25138	4.91052	4.86127	4.46607	6.48823	7.02983	5.63587	6.76576	5.6755	5.40246	5.55028	3.98093	6.07228	5.87841	5.47613	5.54968
MDGA1	-0.38191	0.38917	0.48155	0.66432	-2.61943	-2.79336	-3.05378	-2.78043	-0.44245	0.583	-0.00662	0.6676	-0.96736	0.58158	0.43674	1.1115
ZFAND3	4.31798	4.60517	4.56834	4.409	3.78106	4.39373	4.13023	4.21219	5.01687	5.65877	5.39347	6.14112	4.52351	5.34079	5.42588	5.36184
BTBD9	-0.22291	0.3747	0.49694	0.69641	1.25164	0.73408	1.13027	0.77944	2.26909	2.19563	2.18894	2.76641	1.1359	1.58652	1.6534	2.42151
GLO1	6.34007	6.11449	5.98001	6.19393	6.43952	6.23972	6.75117	6.03662	7.23429	6.77769	6.69072	7.00823	7.43374	7.35604	7.03693	6.90635
DNAH8	-2.58192	-2.51275	-2.5805	-2.65679	-2.63214	-2.02359	-2.45953	-1.6132	-3.32193	-3.32193	-3.32193	-3.32193	-3.15693	-2.53585	-2.79288	-3.11914
SAYSD1	-0.29261	0.11477	-0.39565	-0.14574	2.37987	2.47842	2.2118	2.63019	1.91464	1.77664	2.05941	1.64085	2.43872	2.44469	2.66267	2.67045
ANKRD18EP	-2.01485	-2.32704	-2.5575	-1.80527	0.23515	-0.86982	0.78575	-2.05714	-0.62503	-1.09032	-0.9223	0.38077	1.84332	1.91957	1.65103	2.03558
DAAM2	-1.83791	-1.97337	-2.18011	-1.51632	-2.88235	-3.08104	-3.24343	-3.12972	-2.68308	-2.89258	-2.62538	-2.62173	-2.36685	-3.01878	-2.8361	-2.55507
MOCS1	3.24645	3.08485	2.75033	3.08557	1.53444	1.33722	0.9413	0.99365	1.13245	0.99631	0.75277	1.06408	0.67127	0.64499	1.15958	1.48205
UNC5CL	-2.74181	-2.68449	-2.01974	-3.32193	1.88957	2.18715	2.27376	1.8254	-2.42429	-0.7926	-2.30363	-1.68429	-0.25709	1.0091	1.43252	1.80956
OARD1	2.23408	2.28716	2.40255	2.59288	2.63796	2.80595	2.92749	2.77917	1.73222	2.21164	1.66968	1.9429	1.96463	2.36275	1.74858	1.98363
TSPO2	-1.021	-3.32193	-3.32193	-1.74499	0.82222	1.16637	0.64757	1.49035	-3.32193	-3.32193	-3.32193	-3.32193	-2.63002	0.11258	-0.51457	-0.83662
NFYA	3.4508	3.5186	3.77946	3.88306	3.90047	3.20181	4.25049	3.19141	3.36493	3.06821	3.56951	3.53446	4.25566	3.89425	4.83523	4.11855
ADCY10P1	-0.8727	-0.59635	-0.40228	0.25368	-0.79449	0.27161	-1.18208	0.21568	-1.79303	-1.63659	-1.6256	-1.16479	-0.79436	-0.53327	-0.11082	-0.64183
FOXP4-AS1	1.6283	2.00621	1.24139	1.55205	-1.14269	-0.08901	-1.58772	-1.0135	-0.19644	-0.78394	-0.69784	-0.80427	0.78869	1.20509	-1.26069	-0.01159
FOXP4	5.11758	4.66599	5.37812	4.08172	4.28446	3.27074	3.47732	3.16305	5.19471	3.73962	5.36912	3.35391	4.15053	2.19153	5.21249	3.46592
MDFI	-2.93499	-3.32193	-2.87362	-3.32193	-0.0952	0.7538	-0.05674	-0.70784	-1.37314	-1.44626	-1.9471	-2.20657	-3.01043	-3.32193	-2.41035	-2.9432
TFEB	-0.56246	0.03621	-0.07924	0.35571	-0.41927	1.06129	-1.04452	0.80799	1.46854	2.51226	1.95321	2.28714	-0.72266	-0.91843	-0.87284	0.07327
FRS3	1.81626	1.93303	1.99742	1.47196	1.2808	2.11381	0.08848	2.4322	1.97062	2.56399	2.61359	1.52624	0.70645	0.84473	0.55988	1.04338
PRICKLE4	1.61196	2.5333	2.72361	2.51064	3.09021	3.73755	1.99166	3.5167	1.23279	2.52813	2.32841	1.90191	1.50078	2.05887	2.15986	2.244
TOMM6	8.91246	8.62537	8.53555	8.48513	8.52382	9.07692	8.19846	8.95927	8.2554	8.1849	8.22576	7.39367	8.79334	8.70021	8.08104	8.07428
USP49	0.39533	0.41832	0.27664	0.84447	-0.55106	-0.99315	-0.5625	-0.46294	0.31986	0.46624	0.46571	1.11656	1.09232	1.16442	1.27531	1.26452
MED20	2.89526	2.62892	2.9025	2.4542	2.99885	2.24614	2.88189	2.28907	4.30176	3.94458	4.1664	3.91156	4.1353	3.85227	4.02751	3.84357
BYSL	5.28193	5.0239	4.60616	4.43791	5.03038	4.62496	4.8885	4.78083	5.07249	4.71339	4.96422	4.08926	5.21445	5.267	5.01954	4.88984
CCND3	5.11877	5.53523	5.1918	5.02188	3.20094	2.97016	2.96885	2.93387	4.74064	4.65472	4.56001	4.01044	2.48591	4.28168	4.04984	4.22208
TAF8	2.13345	2.07344	1.88146	2.08271	1.49521	1.64008	1.65556	1.67431	1.95573	2.08495	2.279	2.388	1.47748	1.77991	1.70487	1.62917
C6orf132	-2.39126	-1.72064	-2.09423	-2.54376	-2.57907	-0.90188	-2.92446	-1.10857	2.89147	2.4381	2.97167	1.42644	-3.32193	-2.21794	-2.34132	-2.32567
GUCA1B	0.19659	-1.27554	0.01257	-0.16445	-1.5695	0.29639	-2.59867	0.33476	-0.77023	-0.99006	-1.36233	-0.27375	0.38058	0.92324	0.44886	0.40587
MRPS10	6.13282	6.20625	6.00847	6.45258	5.25793	4.97164	5.94551	4.66107	5.2472	4.8915	5.03703	5.24807	6.17277	5.90891	5.88249	5.41278
TRERF1	2.57581	2.18951	2.40512	2.19717	1.28462	-0.13303	1.46519	0.17218	3.27833	2.94817	3.38495	3.11506	1.99449	1.9809	2.54136	2.3876
UBR2	3.0908	3.10009	3.12823	3.52695	3.14225	2.64424	3.81317	2.69841	2.91252	3.03302	2.98914	3.99296	3.21877	2.86989	3.36711	3.47817
TBCC	4.88698	4.93851	4.73725	4.63793	4.40537	4.54295	4.1027	4.52756	4.65016	4.45068	4.89254	4.19829	4.59353	4.66289	4.39297	4.47085
BICRAL	0.91125	1.01596	0.80961	1.46631	1.3785	0.26408	1.60055	0.27045	0.49072	0.65671	0.21094	1.81962	1.21498	1.28535	1.48353	1.54713
RPL7L1	5.5097	5.45234	5.23546	5.25804	5.14656	4.81693	5.57541	4.75958	4.91564	5.07883	5.11191	5.4281	5.65646	5.81216	5.62687	5.62132
C6orf226	2.40661	2.71596	2.60802	2.61703	3.16951	4.3118	3.21522	3.83944	3.20977	3.43442	3.14392	2.33105	3.40113	4.27929	2.90365	3.10549
CNPY3	6.46319	6.50097	6.49714	6.16379	6.11553	6.76645	5.41374	6.70425	6.52355	6.38235	6.51036	5.63627	5.51644	5.56837	5.73592	5.98053
RPL24P4	3.70096	3.86634	3.98415	2.31824	2.3313	3.58846	1.95821	3.21868	3.85369	5.35083	3.8116	3.45093	2.28276	2.23425	1.92456	2.45866
GNMT	-0.41197	-0.26647	-0.34706	-0.62508	0.11629	0.26065	-0.13453	0.93234	1.43764	1.95233	1.23268	1.43728	-0.71199	-1.01268	-0.46971	-0.58811
PEX6	5.44764	5.63281	5.41342	5.2474	5.5202	5.07831	4.60803	5.18492	5.43582	5.90871	5.8678	5.46924	2.91076	3.13909	3.4379	3.92272
PPP2R5D	5.59037	5.36129	5.43906	5.38903	5.35262	4.92304	4.79234	4.8752	5.96183	5.18014	5.58397	5.07334	6.06716	5.51514	6.0709	5.78365

MEA1	7.5295	7.60942	7.54848	7.20341	6.66575	6.97299	6.03331	6.89415	7.23557	6.91296	6.93328	5.74624	6.86644	6.58937	6.63385	6.56721
KLHDC3	6.29639	6.28102	6.15822	6.23956	6.09334	6.12891	5.59579	6.03706	6.78802	6.77415	6.69652	6.44135	6.56289	6.98874	7.06001	7.44054
RRP36	5.49221	5.54378	5.5115	5.56968	5.88133	5.66357	5.87832	5.62115	5.50883	5.34699	5.61755	5.42431	5.5168	5.85941	5.97754	5.75837
CUL7	2.66922	3.15246	3.31967	3.5627	3.97562	4.55437	3.54979	4.13492	4.00731	4.79354	4.70442	5.19893	2.70191	3.03672	3.52547	3.86994
KLC4	2.44108	2.69821	2.89927	3.05208	3.35957	3.30227	2.89847	3.04969	2.59191	3.18002	3.09838	3.34818	1.56549	1.65432	2.39782	2.52357
MRPL2	6.07556	5.90122	5.68211	5.58231	4.29656	4.95306	3.87864	4.86001	5.09933	4.80961	5.03473	4.14248	4.54111	4.22595	3.95237	3.6518
PTK7	-1.1995	-0.75554	-0.29822	0.16427	-1.44753	0.97153	-1.5872	0.6059	2.59173	2.86602	2.97786	2.73273	1.26162	2.121	2.32257	2.76325
SRF	5.11819	4.83192	4.85938	4.18538	5.33701	4.38311	4.94353	4.31902	5.10645	4.67635	5.36858	4.14546	4.99769	5.03568	5.74993	5.28662
CUL9	1.66565	1.6126	1.87679	1.6131	0.90127	1.23944	0.03211	1.10403	2.05313	2.53749	2.60843	2.89975	0.92346	1.58877	1.54528	2.21343
DNPH1	7.28662	7.27826	7.3697	6.5193	6.96777	7.25466	6.0582	7.11369	6.07697	6.22075	6.46092	4.52336	6.06783	5.97425	6.1291	5.95617
ZNF318	2.75656	3.11086	3.24028	3.39702	3.06743	1.8697	2.89312	2.18342	3.00325	2.65734	2.68749	3.20733	2.78388	2.18568	2.86359	2.76115
ABCC10	3.24797	3.42598	3.36033	3.35009	3.22793	3.16251	2.53391	3.37553	2.73366	2.79929	2.99602	2.72229	2.39516	2.64648	2.98379	3.5748
DLK2	1.07943	1.97011	1.82954	1.81468	2.02865	2.4698	1.03139	2.6818	3.9173	4.14341	3.77904	3.14613	2.34378	2.39184	2.53606	2.73062
TJAP1	3.42471	2.79413	3.11538	2.72814	3.00475	3.36747	2.56418	3.43231	2.79185	2.74304	2.96879	2.73073	2.77355	2.31104	2.53458	2.23123
LRRC73	-0.03178	0.65862	1.0362	0.37967	0.32196	0.90756	-0.87822	1.26707	-1.6773	0.89862	-0.09857	0.09936	-1.37081	0.24547	0.36495	0.64531
POLR1C	4.69329	4.49532	4.36637	4.39332	4.40267	4.55638	4.56381	4.64695	3.82429	3.84851	4.15798	3.56726	4.60656	4.06993	3.87803	3.71411
YIPF3	5.54214	5.89082	5.82887	6.04688	6.31384	6.88013	6.15656	6.76274	5.49893	5.90068	5.73241	5.86305	4.85193	5.29915	5.1737	5.60066
XPO5	4.07219	3.80334	3.76301	3.69401	4.10336	3.32837	4.27146	3.50247	4.33647	3.50684	3.97486	4.22072	4.75671	4.3152	4.76014	4.35982
POLH	2.47487	2.40389	2.73801	2.13421	2.25529	1.59744	2.33116	1.80323	4.442	3.33628	4.15873	3.95236	3.9927	3.32577	3.76123	3.04467
POLH-AS1	1.82759	1.40696	1.57493	0.98578	0.48389	1.16358	-0.03189	1.33592	1.97351	1.28895	1.09108	1.51086	2.50818	2.70617	2.02396	2.67204
GTPBP2	4.9767	4.73474	5.16244	4.82932	4.13345	4.80558	3.5243	4.38697	4.07376	4.03977	4.35481	3.74953	4.40454	4.2819	3.96016	3.96604
MAD2L1BP	4.14459	4.30691	4.03875	4.32252	4.56892	4.13661	4.47374	3.86157	4.23744	4.29618	4.08685	4.05105	4.49477	4.66849	3.6904	3.96815
RSPH9	3.84575	3.63807	3.41337	3.46577	3.80803	4.37845	3.45273	4.29338	3.9791	3.80546	3.96287	2.97715	3.40177	3.56332	3.00761	3.13174
MRPS18A	5.74726	5.62711	5.36725	5.37888	5.73096	5.97138	5.31547	5.85736	5.9127	5.76153	5.84914	4.91295	5.11754	5.23677	4.95304	5.0882
VEGFA	5.40414	5.25763	5.84394	5.25301	3.07008	3.60223	2.50013	3.64204	2.87559	3.88323	2.98388	4.0605	3.38981	3.41456	2.93511	2.94871
C6orf223	-0.70094	-0.42479	0.17998	-0.46328	-2.90335	-2.47286	-2.68514	-2.19869	-3.0229	-2.66822	-2.8257	-2.36648	-2.3723	-2.06366	-2.85898	-1.50895
MRPL14	7.61197	7.53101	7.40041	6.76966	7.0802	7.18493	6.43247	7.05356	7.16831	6.89239	7.19458	5.32452	6.94519	6.89174	6.10091	6.02104
TMEM63B	4.13499	4.1	4.17087	4.17979	5.479	5.28569	5.27751	5.52408	4.43488	4.86995	4.96126	5.07981	3.69156	4.56043	4.58171	5.20821
CAPN11	-2.41523	-1.34173	-2.29595	-2.2486	-3.07612	-1.13995	-2.3071	-2.29305	-1.21608	-0.6253	-1.34187	-0.60001	-3.32193	-3.09454	-2.44602	-3.32193
SLC29A1	5.69733	5.37413	5.12008	4.75004	6.6186	5.76595	6.12081	5.57036	6.40999	6.15908	6.20215	5.96847	5.94649	6.20612	6.11358	6.8131
SLC35B2	6.23926	6.19311	6.50653	6.17319	7.37358	7.06676	6.77102	6.89738	6.19321	6.03697	6.35991	5.7023	5.92601	5.85849	6.11234	6.15622
MIR4647	8.05486	8.2629	8.18432	8.34274	8.35788	9.52311	7.65205	9.69454	7.12856	6.83583	7.08509	6.185	7.70288	7.29341	7.36609	7.44542
NFKBIE	4.8169	5.13894	5.05293	4.78972	5.04097	4.41836	4.54827	4.31315	4.97671	5.35091	5.32546	4.82818	3.49999	4.16325	3.63419	3.86753
TMEM151B	-0.25274	0.08059	-0.37008	-0.7745	0.51439	-0.79996	0.11377	-0.4626	0.19044	0.06484	0.35417	-0.77299	-0.02927	-0.38475	-0.15062	0.53186
AARS2	3.80222	3.56995	3.3712	3.10483	4.09226	3.39827	3.52499	3.52417	3.54154	3.51109	4.0301	3.25024	3.15141	3.13015	3.44984	3.65667
CDC5L	4.21346	4.24014	4.17521	4.67232	3.80918	2.8958	4.27705	3.14377	4.39902	4.2576	4.17795	4.51916	4.19186	4.35445	4.24771	4.22099
SUPT3H	2.31736	2.41591	2.27799	2.41003	0.89896	1.96647	1.3883	1.52335	2.13526	2.31435	2.28025	2.69105	2.19334	2.14565	2.14247	1.61547
RUNX2	3.44758	3.23662	3.31214	2.78521	-3.26502	-3.18996	-3.32193	-2.67721	3.51218	3.37417	3.57373	3.58609	-1.44535	-1.84944	-1.61645	-2.30972
ENPP4	2.94661	3.22535	2.93211	3.90222	3.17582	2.16566	4.08221	2.06649	0.22256	0.56936	-0.24582	1.17084	1.24777	1.45649	0.50829	0.60098
ENPP5	-3.32193	-2.82767	-2.80462	-1.93874	-0.54055	0.45399	-0.07474	0.43899	-3.04505	-3.08863	-3.07272	-2.29668	-3.32193	-3.32193	-3.32193	-3.32193
CYP39A1	-1.32419	-1.62589	-0.96076	0.26578	-1.73067	-3.32193	-1.15423	-2.64977	-2.95458	-3.32193	-3.32193	-3.01566	-2.84592	-1.4241	-2.19038	-1.10413
SLC25A27	0.32956	0.00115	-0.00741	0.96163	-2.39422	-0.91706	-2.10655	-1.10475	-1.91799	-2.388	-2.01907	-1.53054	-2.24499	-0.57513	-1.95592	-0.9584
ADGRF5	-2.76922	-2.59512	-2.6868	-2.79385	-3.25213	-3.32193	-3.17274	-3.32193	-2.69653	-2.39567	-2.75281	-1.68865	2.64466	3.36897	2.72652	3.56032
ADGRF1	-3.32193	-3.32193	-3.32193	-3.32193	-3.21124	-3.32193	-3.24002	-3.32193	-2.95345	-3.15753	-3.2313	-2.94714	-2.19976	-2.52878	-2.11118	-1.17782
TNFRSF21	4.42155	4.48028	4.19864	5.218	6.64881	5.70305	6.86889	5.25671	5.02109	5.08712	4.69038	5.46813	6.55281	6.48608	7.38882	7.93994
CD2AP	3.74554	3.49499	3.70024	3.72146	4.6394	2.71175	5.478	2.33236	4.53467	3.49784	4.61135	4.26751	5.15725	2.41714	5.4682	2.93552
ADGRF4	0.87929	0.92467	1.23302	2.11051	-2.34223	-2.77317	-0.75785	-2.69483	-2.47545	-3.03421	-2.76199	-2.42051	-3.32193	-3.32193	-3.0368	-3.32193
PTCHD4	-2.9436	-2.90343	-3.32193	-3.07187	-3.23288	-3.32193	-3.32193	-2.54519	0.79744	0.81762	1.2807	1.4485	-2.88646	-3.32193	-2.95874	-3.12495
CENPQ	4.53939	4.22874	4.18107	4.40133	2.80826	2.63527	3.66006	2.95801	4.26338	3.39339	3.22225	2.92878	4.82139	4.85686	3.90496	3.57818
C6orf141	1.41179	1.55801	1.71811	2.751	-2.0921	-1.3452	-2.01246	-2.36441	-3.32193	-2.72822	-3.32193	-2.32118	-0.79668	0.50477	-2.14547	0.44606

FTH1P5	2.85208	2.83449	3.51144	1.1746	-0.63413	0.14782	-3.32193	-1.18849	-1.91306	1.50917	0.17973	-0.64384	-2.24082	-2.24353	-0.63519	-0.95201
PKHD1	-3.32193	-3.32193	-3.24786	-3.32193	-3.32193	-2.92718	-3.32193	-3.10946	-2.72084	-1.95091	-2.37985	-1.59141	-0.05298	0.71782	1.75414	1.13196
MCM3	5.94224	5.56109	5.41612	5.27728	6.05965	5.01275	5.5424	4.9985	6.24798	5.18802	5.55247	4.68032	6.12548	5.74145	5.9241	5.46647
PAQR8	-2.93572	-2.72091	-3.32193	-3.32193	1.72833	2.84004	1.62953	2.71013	0.77022	2.5826	1.71361	3.30197	1.90888	2.65947	3.22887	3.66967
EFHC1	0.52421	0.48821	0.66745	0.77906	-0.03126	0.35268	0.05215	-0.25333	1.31536	1.64543	1.73952	1.65314	1.52939	1.55484	1.99808	1.54179
TRAM2	4.4006	4.37631	4.38389	3.95579	3.61285	2.48198	4.02241	2.3423	6.00321	6.20413	6.30971	6.25635	5.73197	5.38708	6.58411	5.85444
TRAM2-AS1	0.43279	1.27896	1.11007	1.55961	1.44794	1.17473	1.54451	1.04119	0.98401	1.45712	0.99622	1.97948	1.15413	1.6204	1.64472	1.71504
TMEM14A	4.68279	4.83552	4.57463	5.03528	6.71608	6.29011	6.48129	6.3376	5.28624	5.51074	3.90034	4.3934	5.43261	5.78154	3.42049	4.54761
GSTA1	-3.32193	-3.32193	-3.32193	-3.32193	0.85951	0.96214	0.30746	1.18535	-3.32193	-3.32193	-3.32193	-3.32193	-3.32193	-1.84492	-0.89886	-3.32193
GSTA4	-2.54483	-1.73573	-2.43791	-1.83553	4.24319	4.80306	4.02574	4.62989	0.91343	1.54676	0.98933	1.28341	2.31968	2.2056	1.5329	1.86864
RN7SK	5.50128	5.77639	5.8542	4.72174	2.3289	2.71359	4.20418	3.14154	0.56091	0.0182	-3.32193	1.36036	4.92202	5.21114	3.45206	3.80268
ICK	1.30359	0.70573	0.94234	0.91421	2.39679	0.90132	2.36362	0.86838	2.56632	2.28775	2.18415	3.27683	2.70516	1.78629	2.60376	2.12815
FBXO9	2.70421	2.65766	2.55527	2.89156	3.02551	2.94182	2.8389	2.95723	2.89263	2.73633	2.47147	3.00392	3.15554	3.24834	2.37878	2.48817
ELOVL5	5.64589	5.63182	5.26462	5.6177	6.11642	5.30342	6.25592	5.44162	6.38655	6.10675	5.48822	6.40419	6.97047	7.41725	6.76697	7.08118
GCLC	3.06083	2.97663	2.9171	3.61406	4.30062	3.32367	4.91598	3.1805	2.11855	1.33012	1.93513	1.86674	2.87337	2.18906	2.66314	2.37883
LRRC1	0.80594	0.48397	0.57479	0.95658	4.54821	3.52674	4.80896	3.42129	2.26232	1.3304	2.06066	1.68936	-0.37615	-1.37871	-0.24904	-0.82626
HMGCLL1	-3.32193	-3.32193	-3.32193	-3.05395	-3.22611	-3.32193	-3.32193	-3.32193	-3.32193	-3.32193	-3.32193	-3.32193	-0.28272	0.7681	-0.12314	0.19082
BMP5	-3.32193	-3.32193	-3.32193	-3.32193	-3.20635	-3.32193	-3.32193	-3.32193	0.11313	0.37079	0.07887	0.65147	4.20352	3.92607	5.20213	3.97622
COL21A1	-3.32193	-3.32193	-3.32193	-3.32193	-1.73576	0.54958	-1.76379	0.30413	-3.32193	-2.81304	-3.32193	-2.29371	1.5363	2.64688	1.40842	2.45199
BEND6	-1.76392	-1.33572	-1.27705	0.13673	-2.19777	-2.05632	-2.29536	-2.3283	-0.66028	-0.30429	-0.03257	0.2544	-1.38493	-1.27081	-1.80769	-0.24219
KIAA1586	2.73671	2.72257	2.42341	2.89585	1.79556	1.41926	2.4364	1.1621	1.96391	2.1246	1.82354	2.61141	3.4169	3.46878	3.2465	3.10468
ZNF451	2.10219	2.4061	2.20444	2.74696	1.62376	1.13023	2.34176	1.64402	0.89843	1.37506	1.07257	2.02221	1.90768	2.17797	1.97206	2.00945
ZNF451-AS1	2.77037	2.72261	2.55971	3.01593	2.22993	1.5637	2.40197	1.93934	1.6276	1.76003	1.33317	1.61456	2.25501	2.43225	2.0594	2.36494
BAG2	4.36068	3.95079	3.63756	4.16507	3.63983	3.05924	3.81619	3.33928	3.06824	2.51477	2.20068	2.39067	4.83356	4.58734	3.88489	3.87428
RAB23	2.44809	2.58227	2.71745	3.09311	3.08709	2.18917	3.6838	2.63441	1.99367	1.63609	1.73626	2.09329	3.5774	3.73172	3.36653	3.23534
PRIM2	4.14794	4.03025	4.10444	4.11743	2.81034	2.14992	2.88672	1.87977	2.93333	2.02182	2.52804	2.22887	3.64119	3.64809	3.01549	2.75221
LINC00680	2.05375	1.78443	1.66266	1.64893	1.20747	2.04173	1.25923	1.86572	1.15507	1.77604	1.34325	1.29527	1.57899	1.57401	1.2456	1.06334
FKBP1C	2.20854	1.90349	1.95795	1.0169	0.6808	1.12913	0.26441	0.64299	2.98682	3.53235	3.07907	3.07738	1.6693	1.0133	1.05033	1.7492
LGSN	-2.58517	-3.1208	-3.11041	-2.90345	-0.71439	0.37782	-0.62722	0.59543	-2.93253	-3.32193	-3.32193	-3.32193	-3.32193	-3.17913	-3.32193	-3.14552
PTP4A1	4.43814	4.54597	4.29662	4.70223	6.8455	6.09681	7.47035	6.15406	5.05899	4.4073	4.53796	5.28988	5.69254	6.00707	5.08268	5.75587
PHF3	3.42619	3.42652	3.44864	3.71462	3.86467	3.06167	4.45384	3.08694	3.77928	3.88724	3.74355	4.84259	3.36871	2.99873	3.92138	3.93425
EYS	-2.65193	-2.46075	-2.42519	-2.00443	-1.62595	0.80384	-1.06575	0.06541	-2.70982	-2.63146	-2.81207	-2.15202	-2.13143	-1.98496	-2.01396	-1.80414
SCAT8	-1.94267	-2.64465	-1.0606	-0.98762	1.11301	3.56868	1.97694	3.25698	-3.32193	-3.32193	-3.32193	-2.73118	-1.3484	-1.07393	-3.32193	0.31728
ADGRB3	-3.32193	-3.32193	-3.32193	-3.32193	-3.32193	-3.32193	-3.32193	-3.32193	-2.81908	-3.32193	-3.32193	-3.32193	-0.59482	-0.16244	-1.4188	-1.07874
LMBRD1	3.0931	3.3168	3.40446	3.86596	5.22967	4.57595	5.97279	4.50692	2.60209	3.13636	2.59088	3.69656	2.92944	2.71308	3.14751	3.07456
NPM1P37	2.09547	3.30272	2.14189	4.10866	-2.23011	-2.39757	-3.32193	-1.68389	-3.32193	-2.42949	-1.41129	-3.32193	-2.06408	-2.56216	-2.4361	-3.32193
FAM135A	0.59194	0.5434	0.25594	0.92925	2.46617	1.52557	3.43209	1.36505	0.28221	0.70816	0.23657	1.68385	3.32433	3.08542	3.1441	3.44442
SDHAF4	2.27589	2.15562	2.62435	1.88991	1.62094	2.50039	1.93468	2.35057	1.07364	1.34638	0.87252	0.35069	3.17818	2.81604	1.71827	1.67951
SMAP1	2.82293	3.62611	3.71321	4.10588	3.41516	4.02844	4.41557	4.03811	2.55263	3.4688	3.11756	4.49773	2.18804	3.13631	3.55854	3.47644
B3GAT2	1.68222	2.38458	2.52215	2.82771	2.2972	2.97187	3.32013	2.87333	1.35599	2.26892	1.91612	3.34383	1.13301	2.09471	2.50197	2.3492
OGFRL1	2.94599	2.93646	2.79431	3.2107	1.93843	1.71832	2.58283	1.58331	3.4296	3.39489	3.13256	3.82242	5.35797	4.94541	4.60195	4.43837
LINC00472	1.68293	2.03478	1.91024	1.59983	-2.95768	-3.32193	-3.32193	-2.97924	0.54843	1.64222	0.79464	0.87259	0.96665	1.03793	0.64477	0.67433
KCNQ5	0.25946	-0.07163	-0.01933	-0.75675	-3.32193	-3.32193	-3.32193	-3.32193	0.60231	-0.39138	0.42509	-0.72402	-3.32193	-3.32193	-3.32193	-3.32193
KHDC1	-1.80923	-1.8261	-1.91765	-2.41318	0.88095	1.12748	0.95471	1.33624	1.04673	0.14447	0.50591	0.23411	-3.32193	-3.32193	-3.03363	-3.02787
OOEP	-3.32193	-3.32193	-3.32193	-3.32193	3.58059	4.63922	3.66033	4.33888	-2.63044	-3.32193	-2.69146	-3.32193	-3.32193	-3.32193	-3.32193	-3.32193
OOEP-AS1	-3.32193	-3.32193	-3.32193	-3.32193	1.8995	3.87536	2.05945	3.04868	-3.32193	-3.32193	-3.32193	-3.32193	-3.32193	-3.32193	-3.32193	-3.32193
RPL39P3	9.99154	10.2276	9.93273	9.81999	9.15473	9.35985	8.24043	9.30127	9.954	10.223	9.90265	7.88791	8.50835	8.21533	7.98186	7.96376
DDX43	-1.63847	-2.15756	-2.91314	-2.28832	3.75975	3.87429	4.22959	4.12632	-2.6058	-3.32193	-2.95855	-1.69109	-3.32193	-3.32193	-2.98395	-2.69933
CGAS	4.65456	4.49398	4.18076	4.22172	0.61161	1.17185	1.17764	1.11517	2.95282	2.12131	2.10402	2.31827	2.42049	2.36733	1.68749	1.61881
MTO1	1.5599	1.26863	1.2495	1.49108	1.97796	1.46066	2.26463	1.57311	0.54615	0.15136	0.55788	0.63855	1.53638	1.41132	1.16899	0.9963

SLC17A5	3.79295	3.92665	4.09522	4.42594	5.55489	5.59674	6.12084	5.63335	2.50003	2.7494	2.19663	3.4134	4.04723	3.81627	3.5731	3.57671
CD109	2.6994	3.20737	3.78775	4.15829	5.13648	3.53864	5.70173	3.35826	3.59349	3.86032	2.99448	4.56428	4.87424	4.6239	4.12893	4.82087
COL12A1	0.0893	-0.43027	-0.02319	0.14381	-3.01428	-3.15105	-3.09033	-3.1229	2.862	2.49433	2.30764	1.92455	-2.70286	-1.99721	-1.59273	-3.15667
COX7A2	6.43354	6.49873	6.57877	6.49304	7.14747	7.64234	7.02477	7.44147	6.09479	6.10247	5.99098	5.30904	6.09861	6.13224	4.89097	5.09535
TMEM30A	4.91225	4.72647	4.84796	5.22029	5.48122	4.61385	5.95215	4.53273	4.73824	4.34577	4.3533	4.74641	5.09587	4.73672	4.80892	4.66314
FILIP1	-1.73487	-2.01906	-2.09408	-1.69421	-3.17003	-2.98355	-2.91552	-2.71951	-2.10978	-2.73293	-2.69744	-3.10132	-3.22665	-3.05399	-3.20663	-2.36925
SENP6	2.40463	2.30636	2.15385	2.68978	2.79402	2.28633	3.36277	2.44518	2.0819	2.27716	2.46754	2.88568	2.27693	2.90884	2.91081	2.72455
MYO6	2.56609	2.66835	2.57815	3.18415	4.2547	4.6528	4.81151	4.64772	3.03059	2.78624	2.88818	3.49366	3.35805	3.90525	3.634	4.10763
HTR1B	-3.32193	-3.32193	-2.78094	-3.32193	3.88228	0.46605	3.83385	0.20234	-3.32193	-3.32193	-3.32193	-3.32193	-0.33648	0.56464	-0.08421	0.77523
IRAK1BP1	0.4497	0.05043	0.18432	0.79648	0.85856	0.63844	1.30604	0.87818	0.48933	0.10095	0.35987	0.99463	1.5468	1.15107	1.39772	1.18214
PHIP	3.19999	3.10205	2.90887	3.85232	2.93856	3.20832	3.58577	3.78243	2.1538	1.73813	2.02643	2.63525	3.08687	3.17893	3.54807	3.58074
HMGN3	5.82824	5.58867	5.36697	5.86224	6.20352	7.00634	6.10649	6.92426	5.32039	4.70489	4.79146	3.45733	6.08192	5.90473	4.90985	4.80532
HMGN3-AS1	3.24069	2.85915	2.63632	2.99075	3.8276	4.23462	3.45865	3.95284	2.7926	2.26297	2.45324	0.2617	3.04122	2.84291	2.25654	2.43191
LCA5	-0.60038	-0.85518	-1.29214	-0.1974	-1.24962	-1.62737	-1.2244	-1.52428	-0.66791	-0.43442	-0.73985	-0.18901	0.49054	0.33125	0.34245	0.92851
SH3BGRL2	-0.23344	-1.1338	-1.0726	-0.49279	3.91008	1.9552	3.81256	2.15855	-2.49368	-3.32193	-3.32193	-3.32193	1.12283	0.66393	1.31544	0.56908
ELOVL4	-3.32193	-3.32193	-3.32193	-3.32193	0.67385	1.40806	1.16568	1.21117	-0.3546	0.01478	-0.49839	0.15813	-0.28707	0.25752	0.00185	0.22725
GAPDHP63	1.89624	1.17802	1.72065	-0.76984	-1.46648	1.08819	-1.66279	0.14845	0.69863	2.25768	0.82919	1.05154	-2.17944	-2.18225	-1.34249	-1.61866
TTK	3.56634	3.27623	3.50655	3.49159	3.80294	2.91081	4.36046	2.88318	3.38178	2.69473	2.71404	2.6299	3.94708	3.37217	3.48767	3.11294
BCKDHB	2.17728	2.30958	2.41073	2.69551	2.09419	2.5133	2.41466	2.45263	1.59699	1.9655	1.96049	2.73955	2.53369	2.21387	2.15978	2.11211
TENT5A	1.79052	2.14322	2.73274	2.98221	4.20908	4.81727	4.82569	4.98569	3.59309	3.74592	3.95495	4.93706	-1.31705	-1.15095	-1.13601	-1.11133
LINC02542	0.76987	0.66327	0.63615	0.72205	-0.78289	-1.05333	-0.66931	0.29045	-3.32193	-2.388	-2.3378	-3.32193	0.08626	0.58537	2.08635	1.08887
IBTK	3.43123	3.69422	3.80135	4.38417	4.94266	4.45934	5.74573	4.44185	3.02076	3.21033	3.00994	4.06699	3.82953	4.32654	3.50981	4.12012
TPBG	5.24605	5.93325	6.66578	5.76318	4.12555	3.52928	3.84825	3.19621	2.36283	2.18412	2.28531	2.11464	-1.90329	-2.22376	-1.17314	0.24617
UBE3D	1.06397	0.61403	0.73766	1.28952	0.98294	1.24516	1.07415	1.06513	-0.05792	-0.03229	-0.12447	0.33595	0.19666	0.2157	0.02224	-0.05184
DOP1A	2.14627	2.29662	2.25073	2.81255	2.68921	2.56938	3.38436	2.57162	1.79777	1.79396	1.7371	2.56098	2.04239	1.80311	1.95492	2.17822
PGM3	3.95378	4.2294	4.05719	4.69079	4.67922	4.67773	5.33654	4.55974	3.63358	3.51549	3.49436	4.04003	3.72399	3.05778	3.45284	3.21084
RWDD2A	2.93464	2.71039	2.6358	3.08307	2.46322	2.84062	2.21036	2.92981	1.40823	1.76481	0.58097	1.74094	0.68737	0.66792	-0.23093	0.34858
ME1	2.78491	2.55156	2.84637	3.12635	5.10318	4.49593	5.77653	4.21815	3.46538	2.98861	3.22192	3.84761	4.84424	3.71878	5.09194	3.83564
PRSS35	-2.92349	-3.32193	-3.32193	-2.83491	-2.83108	-2.12884	-2.64944	-2.2277	-2.86804	-2.63178	-2.91103	-2.39242	1.27352	2.91157	2.03868	2.60664
SNAP91	-2.76928	-3.32193	-2.82139	-2.19859	-3.12195	-2.76421	-2.914	-3.13442	-2.17088	-0.95359	-2.02878	-0.91204	-0.07369	0.65602	0.56707	0.51838
RIPPLY2	-0.01021	-0.2316	-0.47974	-0.75347	0.87206	1.5185	0.48786	1.87279	-0.45981	-0.608	-0.60192	-1.32263	-2.20694	-1.46833	-2.15631	-2.13874
CYB5R4	1.97315	1.96916	1.80233	2.18713	1.89246	0.94437	2.46696	1.01163	2.11194	1.91018	1.48732	2.16208	1.49241	1.97435	1.38249	1.19734
CEP162	0.91569	1.08478	1.19135	1.81502	0.78729	0.46031	1.5778	0.39418	0.36354	-0.18556	0.47771	1.07225	0.85645	0.37335	1.41791	0.44264
TBX18	-3.19543	-3.32193	-3.17323	-3.32193	2.39211	1.67151	2.41324	1.84756	-3.32193	-3.32193	-3.32193	-3.20174	3.7561	3.50593	3.67165	3.01226
LINC02535	1.60123	0.90141	1.07889	0.69648	1.49992	-0.28947	0.89417	-0.70304	0.92214	0.6409	0.62268	0.64515	4.13696	3.66232	2.80124	2.30811
NT5E	5.20867	5.81238	6.13345	6.1064	4.85785	5.32388	5.42612	5.10664	6.09386	6.44721	6.11682	7.56681	6.5979	6.12105	6.0335	6.56355
SNX14	2.60294	2.8054	2.89487	3.17346	4.57723	3.83045	5.12729	3.56308	3.71589	3.79143	3.53711	4.31167	4.03285	3.74537	3.84052	3.531
SYNCRIP	5.7452	5.69294	5.39235	5.73213	5.29118	4.5239	5.61595	4.73128	5.22984	4.68237	4.54251	4.97413	5.36505	5.35576	4.98805	4.93191
SNHG5	6.67543	6.58409	6.5765	5.98153	4.81891	4.96953	4.72629	5.02728	3.41389	3.99617	3.61396	3.16582	4.14967	3.56455	2.69267	2.83206
SNORD50B	5.43766	4.56954	5.48417	4.07862	3.83053	4.34187	5.00824	4.92748	2.3937	1.72573	1.8261	2.10579	4.32756	3.88888	0.21235	-3.32193
CGA	-3.32193	-3.32193	-3.32193	-3.32193	0.93991	6.02619	1.02493	5.22672	-3.32193	-3.32193	-2.88681	-3.32193	-3.32193	-3.32193	-3.32193	-3.32193
ZNF292	1.81923	2.09328	2.01051	2.98441	1.58738	1.97509	2.1974	2.2977	2.25484	2.70343	1.97595	3.75612	1.59576	1.88563	2.02833	2.36404
GJB7	0.57875	0.73476	0.59943	0.36152	0.00847	0.21899	0.24905	-0.0806	0.03647	0.08386	-0.36286	-0.8408	-0.42249	-1.0011	-1.23164	-1.03343
SMIM8	0.11727	0.31619	0.07912	0.52992	0.05397	0.31968	0.33259	0.47223	-0.14516	-0.13186	-0.57827	-0.07495	-0.25726	-0.32296	-0.46047	-0.75282
C6orf163	-0.77496	-1.02131	-0.95884	-0.97803	-2.47389	-1.65438	-2.99861	-0.37299	-2.36705	-2.49126	-2.96716	-2.50197	-1.89612	-1.50596	-2.72369	-1.26833
CFAP206	-2.12728	-1.77159	-1.30261	-1.42981	-1.73355	-1.16002	-1.83411	-1.21039	-2.00107	-2.82185	-1.53623	-0.57734	-1.72762	-0.17113	-0.61318	-1.06694
SLC35A1	2.68805	3.0478	3.09401	3.30423	4.25253	4.42419	4.50914	4.36194	2.44323	2.31595	2.31583	2.45607	2.84898	2.97791	2.23513	2.45776
RARS2	4.51605	4.76784	4.55974	4.83153	4.31493	4.57729	4.7427	4.41162	3.83939	3.98216	3.85227	3.91412	3.83708	4.20777	3.75418	3.99657
ORC3	4.19366	4.04336	3.96609	4.36856	3.30381	2.86167	3.41222	2.75088	3.31545	2.83998	2.77745	3.48917	3.77415	3.25949	2.89758	2.88127
AKIRIN2	6.23021	6.11482	6.04972	5.87578	4.59541	5.01999	4.53742	4.92351	4.80217	4.4839	4.70056	4.33602	4.34318	4.6877	4.61419	4.80467

CNR1	-2.4308	-2.2541	-1.79569	-1.18746	-2.41006	-0.97893	-2.07853	-1.06626	-2.96148	-3.16136	-3.32193	-3.16393	-2.58248	-2.66947	-3.0196	-2.75968
ACTBP8	0.36827	-0.3083	-0.10491	1.09345	-2.40165	1.48092	-1.75558	1.70751	-3.32193	-3.32193	-3.32193	-3.32193	-3.32193	-3.32193	-3.32193	-3.32193
RNGTT	2.56313	2.42777	2.27265	2.61329	2.88133	1.76055	3.29881	1.78345	2.56044	1.8486	2.03807	2.013	3.2573	2.84034	2.7018	2.57771
PNRC1	4.5255	4.80395	5.16477	5.33082	4.38769	4.45596	4.48234	4.23952	4.08617	4.38103	4.49602	4.70964	3.45579	2.75315	3.84948	3.5755
SRSF12	-2.43187	-2.35277	-2.63799	-3.32193	2.04856	2.3661	2.07374	2.22247	-2.47921	-1.22056	-1.38514	-1.35403	1.89649	2.58532	1.86863	1.94372
PM20D2	2.5614	2.51258	2.13641	2.18038	4.75484	3.07334	4.89195	3.30798	3.18985	2.7907	2.445	2.4897	4.47148	5.02767	4.66188	4.44956
UBE2J1	4.31281	4.24829	4.07744	4.43237	4.75529	4.55316	5.14614	4.42263	4.329	4.08638	3.82717	4.59979	4.99763	5.22108	4.73316	5.11553
RRAGD	4.33569	4.74271	4.32623	4.63379	3.19832	3.34652	3.6535	3.39378	2.96047	3.45421	2.51389	3.77683	2.84738	4.29416	3.43609	4.17981
ANKRD6	-1.10963	-1.42689	-1.17714	-0.75131	0.90228	0.08541	1.19107	0.47057	0.47396	1.33178	0.85488	1.49157	0.58654	1.56499	0.98345	1.73077
LYRM2	2.48534	2.50605	2.47683	3.24335	2.88449	2.53285	3.37975	2.65801	1.89826	1.9192	1.85978	2.32436	3.23323	3.1554	2.88417	2.75296
MDN1	2.14076	1.65685	1.51758	1.85535	2.87688	1.96378	3.10202	1.97674	2.50638	1.85614	2.32356	2.51213	3.3083	2.74738	3.58479	3.55942
CASP8AP2	1.86134	1.82085	1.9936	2.92947	1.96797	1.0353	2.33807	1.40161	0.95065	0.72865	0.77714	1.25529	2.47945	2.01074	1.82623	1.72963
BACH2	-1.74529	-1.24995	-0.90992	-0.75977	-3.32193	-3.32193	-3.32193	-3.32193	-2.77504	-1.74254	-1.87549	-0.94055	-3.23496	-3.32193	-3.11849	-3.32193
MAP3K7	2.78391	2.68982	2.79332	3.04402	3.08459	2.80406	3.6404	2.75047	2.76357	2.76117	2.88715	3.48766	2.72874	2.81759	3.08354	2.69417
LINC02531	1.5102	1.91553	2.20058	1.69566	1.1088	0.21243	0.85628	0.04827	-3.32193	-3.32193	-3.32193	-3.32193	-3.32193	-3.32193	-3.32193	-3.32193
EPHA7	-3.32193	-3.32193	-3.32193	-3.32193	1.28347	-1.09431	1.08477	-0.37975	-3.32193	-3.32193	-3.32193	-3.32193	-2.59737	-2.53005	-2.18929	-2.38516
MANEA	1.23841	1.43463	1.09665	1.59827	3.42975	1.63014	4.1133	1.3837	1.51871	0.01293	0.80162	1.76767	2.05516	0.12809	1.60543	0.10877
UFL1	2.35332	2.50224	2.54421	3.32306	3.86524	3.50521	4.227	3.41349	2.7735	2.10586	2.33907	3.17497	2.67112	2.20945	2.31177	1.94025
GPR63	-0.51493	-1.46019	-1.59194	-2.21606	-0.02835	-0.93818	0.00103	-0.53667	-1.15648	-1.67227	-2.00816	-2.08328	-1.98145	-2.10575	-2.23755	-2.40297
NDUFAF4	3.43224	3.1167	2.74946	2.94468	3.6859	3.82658	4.04691	3.72861	3.01003	2.51798	2.55892	2.64845	3.44258	2.86861	2.19934	2.29301
MMS22L	0.42918	0.1366	-0.13593	0.18147	1.12913	-0.42558	1.56506	-0.1443	0.56006	-0.60749	-0.01041	-0.33624	1.51005	0.84468	1.37614	0.82227
POU3F2	-2.47765	-2.70309	-3.07284	-2.83482	-2.97683	-3.32193	-3.32193	-3.07193	0.25207	-0.03749	0.28756	0.38561	-3.32193	-3.32193	-3.32193	-3.32193
FBXL4	0.56289	1.24839	0.68436	1.81922	1.56341	1.25224	1.96132	1.40497	1.82175	2.09376	1.70073	3.12022	1.55569	1.7528	1.35921	1.59961
FAXC	-3.32193	-3.32193	-3.32193	-3.32193	0.98521	0.08708	0.75198	0.27285	-0.97146	-0.98764	-1.38584	-1.45411	-0.11438	0.69796	0.19163	0.47758
COQ3	3.07111	2.68555	2.5457	2.69688	3.99326	4.23906	4.31297	3.93503	3.07947	2.34458	2.57176	1.2758	3.17385	3.18862	2.5214	2.37596
PNISR	2.8953	2.85129	3.00773	3.5227	2.88733	3.94257	3.17273	4.10821	1.64963	1.79647	2.04137	2.2459	2.21129	2.2793	1.93177	1.73381
USP45	0.48688	0.40345	0.35201	0.61919	0.76276	0.47153	1.00552	0.86386	0.31374	0.35679	0.04088	0.66036	1.30534	0.77436	0.71109	0.28626
TSTD3	1.33362	1.49395	1.52198	1.91323	2.0861	2.36665	2.1837	2.17444	0.7606	1.08592	0.68202	1.30506	1.75987	1.20433	1.56072	0.60136
CCNC	3.22063	3.32591	3.2422	3.75769	3.87746	3.71589	4.87121	3.53369	2.18897	3.33201	3.03121	3.29459	3.04304	3.4523	3.87284	3.30178
ASCC3	1.8373	1.72917	1.46316	1.9697	2.9259	1.47979	3.6375	1.67471	3.20845	2.63502	2.72188	3.34752	3.12511	3.17716	3.16182	3.37641
HACE1	-0.14156	0.00757	-0.02057	0.32889	0.84387	0.00059	1.33079	0.09666	0.48419	0.4562	0.06855	0.61693	0.2219	1.04791	0.54953	0.64095
LIN28B-AS1	-2.97554	-2.93833	-3.32193	-3.32193	0.0636	0.15729	0.18722	0.29831	-3.32193	-3.32193	-3.32193	-3.32193	-3.32193	-3.32193	-3.32193	-3.32193
LIN28B	-3.14649	-3.12641	-3.11629	-3.32193	4.92359	2.43513	5.26255	2.45094	-3.11991	-3.32193	-3.32193	-3.32193	-3.32193	-3.32193	-3.32193	-3.32193
BVES	-3.32193	-3.32193	-3.32193	-3.32193	2.23522	1.35557	2.6552	1.34016	-3.32193	-3.32193	-3.32193	-3.32193	-3.32193	-3.32193	-3.32193	-3.32193
POPDC3	-3.32193	-3.32193	-3.32193	-3.32193	3.6904	3.65233	3.97962	3.46519	3.94928	4.0883	3.97283	4.67162	-0.94279	-2.60454	-2.48368	-2.46969
PREP	3.87432	3.84903	3.62302	3.85042	3.90474	3.69711	4.08323	3.4843	4.78686	4.58572	4.63012	4.71417	4.00327	4.21007	4.23962	4.16166
ATG5	1.88256	2.04488	1.74414	2.57114	3.19025	2.84465	3.605	2.63397	3.10483	3.05435	2.57494	3.07869	3.44926	3.5521	2.5873	2.74782
PRDM1	-1.70293	-1.53034	-1.41773	-1.53059	-3.24913	-3.1543	-3.32193	-3.32193	3.60838	3.02251	3.36104	3.53104	-0.3275	-0.82081	0.09175	0.24142
CRYBG1	-0.00322	0.12547	-0.00694	0.67105	-0.16623	-1.68131	-0.12491	-1.94246	0.09121	-1.09564	-0.63819	-0.56815	0.65719	0.00754	0.44058	1.23631
RTN4IP1	2.35969	1.97794	2.30577	1.98967	3.52626	3.35571	3.68201	3.3172	1.97778	1.33712	2.15643	0.94587	2.44879	3.41389	3.1512	2.79276
QRSL1	1.71103	1.49353	1.60922	1.87089	3.1263	2.53213	3.56128	2.30499	1.44037	1.70948	1.60828	1.90421	2.53553	3.01589	2.69144	2.72858
LINC02532	-3.32193	-3.32193	-3.32193	-3.32193	1.93172	1.98487	1.92771	2.42359	-3.32193	-3.32193	-3.32193	-3.32193	-2.18716	-0.99051	-2.90367	-1.94112
CD24	-0.61854	-0.31617	0.71709	1.19087	7.71597	7.59054	8.34501	7.35921	2.62266	2.31657	2.38964	2.54825	7.88006	8.68026	8.16672	8.23245
BEND3	0.75599	0.1457	-0.01836	-0.12564	2.3956	1.01162	2.0108	1.12346	1.14441	0.20233	0.95157	0.1097	1.75351	1.43539	1.90742	1.298
PDSS2	1.29424	0.95728	0.6558	1.1675	3.36589	1.79822	3.09305	2.00115	1.23381	0.99076	0.24537	1.22042	1.87267	1.33506	1.08375	1.27202
SOBP	-2.78621	-2.40871	-2.27547	-2.81021	2.64921	2.22667	2.45223	2.57445	0.12839	-0.31363	0.32529	-0.43553	-3.32193	-3.08864	-3.17484	-3.32193
SEC63	3.45709	3.53782	3.5664	3.8188	5.17663	5.21127	5.60772	5.20548	3.76218	3.50597	3.40928	3.99483	4.05297	3.86294	4.02044	3.90493
RPL3P7	0.32499	0.23649	0.62428	-1.24005	-0.83048	0.18387	-1.32601	-0.49548	0.28273	1.72727	-0.15835	0.17888	-1.3183	-1.73717	-1.09902	-1.27769
OSTM1	3.66263	3.91972	3.55102	4.39245	5.41617	4.88836	5.76655	4.83356	3.6634	3.66756	2.93535	3.80735	4.52962	4.5116	3.92716	4.22418
SNX3	6.38708	6.67931	6.70775	6.79997	7.1942	7.18907	7.52394	6.82669	6.87705	6.97743	6.54163	7.02764	7.03976	7.27417	6.80027	6.70449

AFG1L	-1.09728	-0.80032	-1.05633	-0.88734	0.09412	-0.26251	0.95214	-0.08744	-1.25057	-0.88138	-0.89516	-0.6442	-0.62845	-0.21755	-0.86978	-0.60885
FOXO3	2.67867	2.75872	2.64545	3.03046	2.39545	1.6688	2.27174	1.4679	3.34483	3.51981	3.0359	4.14883	2.48257	2.6801	2.93784	3.43717
ARMC2	-1.28472	-1.15698	-0.69143	-1.02353	-0.28404	-0.69668	-0.59395	-0.8465	-0.57107	-1.14499	-0.85196	-0.40001	-0.7352	-0.27397	-0.80906	-0.78298
SESN1	0.07698	0.02656	0.14469	0.73887	1.17806	1.21531	1.79367	1.02064	1.49206	1.65068	2.16362	2.39055	-0.01377	0.80283	0.70824	0.64542
CEP57L1	0.6304	0.60604	0.54406	0.75208	0.8856	0.96674	1.68705	0.84281	0.75266	0.25551	0.62061	0.84236	1.4873	1.26206	1.14291	0.66329
CCDC162P	-3.21421	-3.20157	-2.77676	-2.95056	-1.6119	-1.64631	-1.38759	-1.9616	-2.97674	-2.94452	-3.10705	-3.12409	-2.58378	-2.75233	-2.29608	-3.02686
CD164	4.20051	4.09891	4.37729	4.76291	5.88194	5.37436	6.60141	4.99063	4.80938	4.38275	4.71145	5.23298	5.60801	4.50339	5.29665	4.50278
PPIL6	0.18804	0.24934	-0.25376	0.05686	1.69971	1.14988	1.49372	0.92012	0.31909	-0.09682	-0.11191	-0.11876	0.44566	0.35198	0.97719	0.89362
SMPD2	3.39826	3.29112	3.15565	3.10659	4.42896	4.27118	4.02566	4.25021	2.63037	2.57806	2.48777	2.12904	3.18055	3.02896	3.4582	3.36861
MICAL1	2.40074	2.87716	3.24725	3.21388	3.49257	4.80005	3.02127	4.18743	2.88511	3.50105	3.70157	3.55939	2.33523	2.70738	2.86665	3.45736
ZBTB24	1.34875	1.35745	1.12602	1.57335	1.99354	1.17709	2.5708	1.35545	1.68799	1.54627	1.76848	2.01177	2.2104	1.78586	1.97325	1.70326
AK9	-0.91482	-0.7997	-1.07908	-0.06819	-1.92571	-1.25632	-2.08123	-1.56626	-2.15154	-1.70124	-2.35443	-1.24951	-1.71442	-1.93238	-1.54727	-1.79832
FIG4	2.10157	2.64615	2.31883	3.15106	2.30604	2.72469	2.79016	2.04783	2.53633	2.57405	2.30299	3.20078	2.6269	2.23928	2.83483	2.31957
WASF1	2.20151	2.34204	2.57693	2.72723	2.29657	2.69573	2.63489	2.61623	2.94673	2.59048	2.56571	3.25649	2.92559	3.72273	2.84179	3.13189
CDC40	2.19444	2.25506	2.00446	2.45635	2.6018	2.00231	3.31216	1.9876	2.61073	2.36029	2.63503	2.72977	2.57022	2.1375	2.44527	2.06215
DDO	1.66027	1.88714	1.58125	2.0996	-1.59019	-1.09415	-1.42624	-0.79403	-1.05535	0.16216	0.2566	0.42603	-2.04257	-1.88541	-2.89123	-2.88302
CDK19	0.61315	0.7295	1.15265	1.31041	1.70635	0.79406	2.29452	0.9982	1.28452	1.39908	1.81696	2.37218	1.98071	1.53911	3.21235	1.95262
AMD1	3.77221	3.72148	3.51991	3.76372	4.6051	3.88168	5.34658	3.85778	3.68781	3.00814	3.43117	3.40482	4.89077	4.8606	4.70329	4.52718
GTF3C6	5.3721	5.50757	5.22217	5.65856	6.5769	7.398	6.93205	7.48045	6.20109	5.35234	5.682	5.77199	6.79285	6.83008	6.00137	6.06688
RPF2	4.01865	3.7518	3.49007	3.8889	4.46176	4.68652	4.99085	4.58154	3.38236	2.85535	3.02795	2.95494	4.42797	3.67264	3.66912	2.94286
SLC16A10	-1.03865	-1.37021	-1.75446	-1.61397	1.48324	-0.15827	1.11738	0.32205	-2.13004	-2.70574	-2.73615	-2.98632	-0.67838	-0.86903	-0.84942	-0.57151
MFSD4B	1.42883	1.6845	1.20595	0.96957	3.36637	2.99569	3.8625	3.34639	0.78253	0.61542	1.14089	1.99638	1.44508	1.32186	1.75847	1.35244
REV3L	0.1036	-0.02416	0.21194	0.81226	1.35721	0.11801	1.91584	0.17978	1.81547	0.8517	1.73228	2.3993	2.12673	1.21633	2.19497	1.66272
TRAF3IP2-AS1	-1.5497	-0.59863	-0.89204	-1.1017	-0.91342	-0.64981	-0.71043	-0.56773	-1.07581	-0.70721	-1.32079	-1.14829	-0.503	-0.67106	-1.66532	-1.79285
TRAF3IP2	1.96416	1.87289	1.64006	2.11977	-0.41719	-0.59331	-0.34843	-0.53187	1.91674	1.26039	1.75003	1.44153	0.80868	0.07355	0.99979	0.38075
FYN	0.73154	0.91585	0.64527	0.70804	-1.63313	-2.43668	-2.41135	-1.93275	2.36846	2.45486	2.58278	2.71	-3.10273	-3.00548	-3.18444	-3.32193
TUBE1	0.47625	0.39155	-0.02402	0.15273	1.38672	1.54827	1.40351	1.59728	-0.1486	-0.16126	-0.17778	0.09861	0.47564	0.55859	-0.66352	-0.4757
FAM229B	1.37997	0.74482	0.66217	0.99124	1.42192	1.87951	0.96477	1.27694	1.71929	1.87226	1.76049	1.14541	2.70219	2.63761	1.65973	1.85377
LAMA4	-0.14462	-0.10804	0.10713	0.36182	-3.24202	-3.13849	-3.07368	-3.32193	-3.32193	-3.32193	-3.32193	-3.32193	-1.03291	-0.74358	-0.56457	1.06293
HDAC2	3.27095	3.16304	3.22463	3.46686	3.48392	2.98942	4.04276	2.9213	3.27687	3.05143	3.18695	3.05632	3.56069	3.40681	3.95633	3.27017
HDAC2-AS2	-2.75508	-2.69881	-2.38662	-3.00807	-2.74606	-2.95236	-2.20553	-2.89597	-1.73028	-2.18006	-1.89155	-2.53115	-1.26284	-2.23302	-1.86453	-1.79652
HS3ST5	-3.32193	-2.81145	-3.32193	-3.32193	-0.63004	-1.60187	-0.79431	-1.26914	-2.10849	-2.53162	-2.33676	-2.54194	-2.79139	-3.12327	-2.87714	-3.32193
FRK	-2.55303	-3.06186	-3.13416	-2.58509	2.37853	1.40302	3.27216	1.16485	0.69044	0.25313	0.71152	1.02131	2.23703	2.56573	3.1609	3.544
NT5DC1	1.58444	1.81582	1.67	2.09389	2.5044	2.5575	3.00888	2.23957	2.03094	1.4058	1.78412	1.84117	2.88832	1.9154	2.36708	1.89394
TSPYL4	0.8693	0.98231	1.08662	1.30299	1.56682	1.1392	1.52259	1.06184	1.95489	1.58522	1.60545	1.73931	2.44706	3.67284	2.60267	3.51864
DSE	2.57936	2.7852	2.92156	3.13391	2.16821	1.22122	1.93166	0.95009	2.33125	2.32605	2.32941	2.78782	2.60904	2.40403	2.98129	2.91354
TSPYL1	3.90796	3.87662	3.77336	4.09818	4.99336	4.00654	4.9677	3.92323	2.6266	2.72804	2.88288	2.45881	4.67408	4.61635	5.09632	5.28266
TRAPPC3L	-1.39021	-1.15983	-2.58466	-1.25209	-3.01262	-3.32193	-3.32193	-3.32193	-0.12338	-0.17262	0.04712	0.69702	-2.10578	-3.03534	-2.9785	-1.91687
CALHM5	-1.53153	-0.97267	-1.15933	-0.55466	-3.22785	-3.21062	-3.32193	-3.19185	0.43721	0.75793	0.71801	1.493	-1.52043	-2.46992	-2.3331	-1.40095
RWDD1	3.19166	3.39342	3.13726	3.57358	3.72953	4.20266	4.03294	4.33112	3.05549	3.35865	3.08896	3.44895	3.21551	3.15171	2.76212	2.65326
ZUP1	1.98449	2.00108	1.86043	2.55866	3.05799	3.08087	3.52033	3.12425	1.68923	1.67789	1.4667	1.76405	1.83984	2.24205	1.45965	1.69456
KPNA5	-0.71348	-1.17348	-0.77789	-0.15505	0.65828	0.7012	1.54154	0.82677	0.12129	-0.1736	0.10007	-0.1329	1.54199	0.18918	1.03815	-0.16905
RFX6	-3.07142	-3.32193	-3.32193	-3.32193	0.1036	2.96126	0.36278	3.05085	-3.32193	-3.32193	-3.32193	-3.32193	-3.32193	-3.32193	-3.08187	-3.32193
ROS1	1.38369	1.48832	1.13426	2.0412	-3.26263	-3.18457	-3.19467	-3.32193	-3.32193	-3.07032	-3.32193	-3.19277	-3.32193	-3.32193	-3.32193	-3.18914
DCBLD1	1.92996	2.01245	2.10671	2.7918	2.52861	2.21585	3.00079	2.39446	2.71864	2.64551	3.13389	3.22947	3.34709	3.3483	3.6882	4.01838
GOPC	2.20513	2.83587	2.73475	3.31694	3.12988	3.16749	4.08267	3.19468	2.46441	3.61789	3.68388	3.95763	2.38875	3.54831	3.25983	3.45504
NUS1	3.8236	3.73022	3.71343	4.09888	4.95405	4.48345	5.61552	4.18456	4.22334	3.80329	4.24506	4.41136	4.79416	3.49262	4.70712	3.71755
CEP85L	-0.65935	-0.49893	-0.48962	0.01611	-0.18959	-0.46993	0.66482	-0.58672	-0.04749	0.17409	0.25839	1.04232	0.03612	0.20179	-0.05672	-0.05216
MCM9	0.05361	0.17854	-0.03559	-0.04347	1.08266	0.45921	1.23233	0.42339	0.47743	-0.20653	0.05573	-0.28924	0.99128	0.67644	0.84951	0.63659
ASF1A	3.57782	3.70148	3.49551	3.95558	3.82425	3.98872	4.04494	4.02124	2.84012	2.69885	2.34572	2.98806	3.93041	4.07291	3.25108	3.39801

FAM184A	-1.60331	-1.84219	-1.98037	-1.18592	0.87881	1.01704	1.47888	1.13567	-1.49516	-1.55575	-1.56766	-1.28054	-2.10362	-0.98742	-1.78478	-1.62776
MAN1A1	3.27062	3.48567	3.26017	4.27117	6.91992	6.27422	7.23678	6.12295	2.86372	2.78169	2.29118	3.19207	-3.32193	-3.00965	-3.12307	-3.32193
TBC1D32	-1.12244	-1.0271	-0.72379	-0.67928	0.16418	-0.25508	0.52767	-0.10775	-0.48716	-0.47959	-0.50457	0.33373	-0.57391	-0.304	-1.00967	-0.38005
GJA1	2.04886	2.19366	2.46452	2.79481	7.58726	6.18554	8.04428	5.55369	-2.9467	-2.74385	-2.70889	-2.17702	5.9797	5.36024	5.3084	5.42525
HSF2	2.429	2.44674	2.40778	2.71248	3.00531	2.73628	3.35921	2.94196	1.82331	2.38521	1.84055	2.78817	3.15724	3.36805	2.7811	2.73534
SERINC1	4.99176	5.45406	5.48824	6.12991	6.47836	5.80369	7.39168	5.70965	5.54585	5.80792	5.49197	6.44057	5.90204	5.82647	5.94288	6.14651
PKIB	-2.23059	-2.50515	-1.66963	-1.8821	3.95649	4.18475	4.91251	4.03562	-2.98524	-3.32193	-3.32193	-3.32193	2.89083	2.51891	2.72693	2.61982
SMPDL3A	0.12594	-0.69155	-0.71579	0.82246	3.26724	3.2561	3.66486	3.25867	0.71034	0.45884	0.82958	0.91484	0.67926	0.78654	0.74612	1.09174
CLVS2	-3.32193	-3.21645	-3.32193	-3.32193	-0.7805	-3.05292	0.01198	-3.01027	-3.32193	-3.32193	-3.32193	-3.32193	-3.32193	-3.32193	-3.32193	-3.32193
RNF217-AS1	0.98333	0.09276	0.28933	-0.63605	1.31703	0.0808	-0.19833	-0.08963	1.91952	1.6953	2.05293	-0.17855	-0.01392	0.51686	0.9933	1.64379
RNF217	0.63934	0.29252	0.29826	0.24008	1.0715	0.6876	1.16174	0.91953	1.72773	1.75938	1.80892	2.12833	1.94996	2.12037	2.02985	2.73702
TPD52L1	1.23779	1.35305	0.81442	1.80969	-1.89293	-1.39987	-2.85564	-2.17434	-2.44894	-2.07123	-2.13903	-2.14601	2.58886	2.72973	2.8871	2.55921
HDDC2	2.84896	2.76283	2.45207	2.83568	3.15205	3.75139	3.22917	3.82248	3.52538	3.05284	3.03758	2.96301	3.80839	3.73913	3.55504	3.31143
NCOA7	0.61276	0.69153	0.39077	1.16509	1.76393	0.9756	2.31411	1.06694	0.391	0.706	0.32369	1.59746	3.69864	3.09115	2.90152	2.83225
HINT3	2.66211	2.98313	2.7568	3.56526	3.36666	2.6724	3.72187	2.67072	-0.21389	0.39999	-0.65803	0.32709	3.93468	4.3616	3.524	4.01085
RNA5SP216	4.20154	4.71724	4.71503	5.34784	4.37399	5.05636	4.15136	4.87626	0.64789	0.94515	-0.408	-0.51833	4.94246	5.40808	4.73376	5.13744
TRMT11	2.73323	2.54397	2.26816	2.92659	3.21381	3.17882	3.5951	2.90929	1.54452	1.55476	1.65474	1.47532	2.99386	2.84242	1.44197	2.07407
CENPW	5.72827	5.55453	5.4799	5.04464	5.42809	5.80143	5.40231	5.71975	5.55004	4.56858	4.96574	3.62251	5.90655	5.83939	5.14293	4.84201
RNF146	2.3876	2.65721	2.73396	3.29155	2.56699	2.81444	2.90958	2.8595	1.55031	2.39001	2.2831	2.82888	2.79628	2.99559	2.85592	3.00814
ECHDC1	3.36224	3.32572	3.26876	3.63666	3.86172	3.75697	4.28495	3.66305	3.73369	3.34253	3.65412	3.59992	4.47543	4.13572	4.00897	3.67642
YWHAZP4	-0.03361	0.27185	0.01307	0.42812	-0.3249	-1.20921	-0.97341	-0.41134	0.81267	1.92591	0.7569	0.4298	-0.91756	-0.92174	0.3234	0.35246
KIAA0408	-0.30652	-0.41365	-0.36935	0.47256	-3.32193	-3.32193	-3.18414	-3.32193	0.97043	1.2558	1.16055	2.07097	1.06586	2.27361	1.68991	2.23249
SOGA3	0.63557	0.70554	0.62823	1.05024	-2.85615	-3.32193	-3.32193	-3.32193	2.62523	2.67389	3.00347	2.64968	1.97746	2.83436	3.0513	3.49026
C6orf58	-0.71959	0.37368	-0.94153	1.48005	-3.32193	-3.32193	-3.32193	-3.32193	-0.02262	1.66979	0.13546	1.89012	0.49717	1.96404	-1.03667	1.15614
PTPRK	3.07264	3.0153	2.92191	3.27176	4.06952	2.63101	4.43889	2.67988	2.82915	3.2142	3.50199	4.37449	3.23104	3.73129	4.23755	4.18932
LAMA2	-2.93505	-2.71994	-2.53221	-2.27389	-3.1871	-3.11343	-3.12842	-3.32193	-1.75331	-2.14532	-1.94728	-1.61723	-0.7516	-1.34333	-1.03188	-1.26833
ARHGAP18	2.42464	2.24404	2.13757	2.14	1.31847	0.47021	2.18581	0.1821	4.39769	4.03765	4.27306	4.47689	5.31538	4.79607	5.28491	4.54195
L3MBTL3	-0.35278	-0.92704	-0.86359	0.05514	0.3265	0.02977	0.66698	0.24278	1.75023	1.15213	1.66282	1.72009	2.1076	1.66943	1.57023	2.02499
SAMD3	-3.32193	-3.15047	-3.32193	-3.13045	-3.25463	-3.1666	-2.81624	-2.97999	-0.91249	-0.33301	-0.76321	-0.3157	-3.32193	-3.32193	-3.32193	-3.17173
TMEM200A	-3.32193	-2.81012	-3.12051	-2.60946	-1.71738	-2.21706	-1.29716	-2.28424	4.63885	4.73929	4.46233	4.89707	-3.18564	-3.32193	-3.32193	-3.32193
SMLR1	-3.32193	-3.32193	-2.87193	-3.32193	3.82041	3.27654	3.44443	3.79501	-3.32193	-3.32193	-3.32193	-2.95132	-3.32193	-2.75401	-2.94899	-3.32193
EPB41L2	3.70769	3.54612	3.59711	3.35875	4.38318	2.77015	4.52043	2.70147	4.52429	3.76435	4.33017	3.97524	4.86642	4.68878	4.90648	4.16992
AKAP7	-0.34215	0.32177	-0.30137	0.05174	1.17662	0.56084	1.60941	0.798	-0.11835	-0.32493	-0.2341	0.59125	1.06533	1.45081	0.99187	0.66209
MED23	0.74944	1.10886	1.01638	1.83322	2.23624	1.93209	2.52833	2.00833	1.46117	1.90501	1.53537	2.54782	2.26566	2.0883	2.08028	2.49789
OR2A4	-2.40893	-2.00778	-3.32193	-0.60532	0.74415	1.54207	0.96675	0.69848	-2.30307	-2.1347	-1.60528	-0.73163	-1.40854	-0.69677	-0.62604	0.06738
CTAGE9	-2.90776	-2.86435	-2.19637	-1.69016	0.83236	1.37181	1.53285	1.4594	-1.15886	-0.95217	-0.54454	-0.02524	1.05651	1.2378	1.02619	1.49867
ENPP1	0.49577	-0.24175	-0.1197	0.30341	2.19877	1.43898	2.81464	1.27869	0.84543	0.53058	0.49434	1.26691	1.66858	1.43715	1.9886	1.60427
MOXD1	-1.51757	-1.91822	-1.96384	-0.87764	0.85803	-0.30112	0.63305	-0.32623	5.03594	4.87135	5.02306	5.2221	1.60989	1.20739	1.02287	0.70584
STX7	1.34434	1.46082	1.50213	1.94666	1.94237	1.87104	2.75281	1.56643	1.91109	1.94299	1.88612	2.35479	2.0079	1.86325	2.04263	1.36069
VNN1	2.10365	2.83585	1.93737	3.47367	1.66311	-0.22106	2.36778	-0.58924	1.83039	2.84481	2.3047	3.63985	-2.65537	-0.67422	-3.01325	-2.52987
VNN2	-0.96389	-0.82831	-1.39831	0.66762	0.53808	0.55674	1.25322	0.36965	-3.32193	-3.32193	-3.32193	-2.44355	-3.32193	-3.02315	-3.32193	-3.32193
SLC18B1	2.73845	2.37768	2.29738	2.69469	4.5784	3.87232	4.8698	3.71491	2.43705	1.63273	2.18305	1.77041	2.49772	1.95162	2.5285	1.64938
SNORD100	4.73615	4.37378	4.52902	4.76409	4.35646	5.40712	4.02725	5.4132	-3.32193	0.11736	1.14784	1.58703	3.018	1.71755	0.10434	0.13299
SNORA33	4.6539	4.23746	3.96906	4.2998	3.70391	4.89131	4.91615	5.93471	1.52396	2.40089	0.96747	2.21031	3.61597	2.82595	0.31034	0.33938
EYA4	-3.32193	-2.34273	-2.54144	-2.11339	-2.87033	-1.57238	-2.53362	-1.25463	0.37526	0.01385	-0.15535	1.07165	-3.32193	-3.22761	-3.32193	-3.32193
TARID	-3.18611	-3.17033	-3.32193	-2.86369	-0.04088	4.16159	0.03065	3.64376	-2.2193	-2.22275	-2.45948	-1.89727	-2.85028	-2.62905	-3.32193	-2.85169
TCF21	-3.32193	-3.32193	-3.32193	-3.04063	1.02934	5.3648	1.04125	4.92235	-2.64866	-3.10228	-2.88541	-3.32193	-2.98027	-2.98136	-3.1043	-3.32193
TBPL1	2.27158	2.01492	2.02137	2.57352	1.82407	2.01988	2.31019	1.92621	2.05796	1.64611	1.61719	2.01519	2.3286	2.63133	1.9775	1.91933
SLC2A12	-0.77009	-1.66902	-1.36546	-0.62545	-1.74087	-0.84519	-1.35159	-1.43991	0.25134	-0.37703	-0.10817	1.10997	0.54682	0.45907	1.95654	1.50038
SGK1	0.89176	1.38932	0.8791	1.45813	2.08072	-0.97603	2.24718	-0.73964	4.11565	4.0109	4.46382	4.29538	3.98572	3.82079	4.04864	3.98996

HBS1L	2.54449	2.69271	2.64126	2.7524	3.35642	2.45613	3.95643	2.41455	2.87299	2.3438	2.5824	2.61021	3.00979	2.80282	3.13956	2.7556
MYB	-1.89137	-1.31719	-1.73278	-1.75533	-1.86764	-2.42884	-1.74743	-2.31593	-1.92012	-2.5724	-2.52961	-2.70296	0.14705	0.79278	-0.05386	0.65567
AHI1	0.54122	-0.08835	0.0567	1.13323	1.75019	1.64148	2.21537	1.57746	0.90468	1.10179	1.04129	2.26792	1.97369	1.65784	1.59849	1.4056
PDE7B	-2.71585	-2.52905	-2.93315	-1.7479	-0.85055	0.24145	-0.65268	0.71105	-3.32193	-3.32193	-3.32193	-2.24218	0.02904	1.00392	-0.5443	0.44471
MTFR2	2.58308	2.06659	1.98754	1.90272	2.90941	2.51225	3.06688	3.01576	2.32114	1.4311	1.24145	0.92741	3.15547	3.38362	2.41478	2.22504
BCLAF1	3.72153	3.58578	3.41305	4.02478	3.99549	2.9367	4.64626	3.16356	4.34772	3.92943	4.11736	4.51721	4.58568	4.33282	4.75626	4.4115
MAP7	-3.32193	-3.32193	-2.90227	-2.87859	0.01109	-2.12245	0.00636	-2.19342	-0.59269	-0.37099	-0.33324	0.35068	-1.44259	-0.90564	0.05473	-0.3819
MAP3K5	-1.93793	-1.39875	-1.61968	-1.81219	-2.41873	-3.12284	-2.45824	-3.32193	1.94845	1.61529	1.45317	1.92242	2.91263	2.58658	2.90532	2.40565
PEX7	0.21087	0.99921	0.58788	0.81164	1.88464	2.17142	1.86917	1.79516	2.28355	1.89914	2.17803	1.39289	1.69949	1.8611	1.26409	1.55971
IFNGR1	3.17078	2.99291	3.34843	3.64216	4.28049	3.5927	4.86691	3.5077	3.6664	3.48799	3.84714	3.63956	3.28876	2.47083	2.90271	1.91929
TNFAIP3	4.263	4.79959	4.7697	5.19402	3.34987	2.12841	3.71249	1.76891	4.18528	4.82635	4.16029	5.01571	1.23333	1.46618	0.82573	1.58928
PERP	4.88523	5.22426	5.36864	5.64524	6.82919	6.3927	7.33637	6.03659	4.19628	3.90674	3.62725	4.28774	6.73968	6.32231	6.93005	6.94844
ARFGEF3	-1.38944	-0.939	-1.04932	-1.28794	1.98376	1.57275	2.2846	1.4494	-0.71461	-0.13616	-1.79009	-0.1422	-0.6176	-0.78449	-0.30886	-0.3733
HEBP2	3.20722	3.22282	3.50568	3.35511	3.3723	3.85693	3.24612	3.75463	2.17244	2.13037	1.91839	1.82898	2.05557	1.7504	1.92844	2.04094
NHSL1	0.96771	0.77777	0.91707	0.72948	2.76416	3.07878	2.91509	2.92967	-0.03319	-0.15799	-0.06578	0.82786	3.20577	2.31001	3.43952	4.05743
CCDC28A	3.36418	3.40878	3.67016	3.80201	2.7871	2.8126	2.92589	2.65234	2.81641	2.48865	3.07431	3.07873	2.29317	1.86311	1.67706	1.32906
REPS1	2.53366	2.27644	2.58007	2.38751	2.22421	1.70608	2.62965	1.90074	2.15685	2.28857	2.66216	2.65688	1.89858	1.85885	2.55206	2.10839
ABRACL	7.10708	7.20423	7.02806	7.61632	4.57155	5.42399	5.1732	5.21641	4.79911	4.467	4.57805	4.67843	5.72513	5.63607	5.07698	5.1346
HECA	1.92791	2.40972	2.25334	2.65034	0.77791	0.26813	1.03463	-0.22366	2.39458	2.50735	2.23181	2.77323	2.31435	2.76365	2.38805	2.75224
CITED2	5.32719	6.25786	6.36585	6.02992	4.42732	6.11124	4.14603	5.54345	7.0278	7.21759	7.41266	6.10251	5.68115	5.65221	5.594	5.5603
VTA1	3.08207	3.31098	3.18223	3.70771	3.53966	3.19289	4.07381	3.16146	3.56401	3.55784	3.32003	3.85668	3.56951	3.75477	3.38074	3.53522
ADGRG6	0.54169	0.27544	0.20494	-0.13694	5.16287	4.13935	5.99528	4.24813	4.22089	4.427	4.21826	4.85143	4.74004	4.9211	4.89383	4.81553
HIVEP2	2.39469	2.56824	2.65086	2.51897	2.41089	1.42931	2.80961	1.29313	2.82634	2.54204	2.65927	3.58025	1.79483	1.73926	2.41238	2.29699
AIG1	3.1619	3.17077	3.40601	3.46523	3.55104	4.06331	3.54655	4.03526	2.45901	2.34019	1.86457	2.64142	2.40462	2.36332	1.87656	2.09752
ADAT2	0.11683	-0.0199	-0.43293	-0.25602	0.70877	1.21423	0.98408	0.95592	-0.1647	-0.92231	0.02697	-0.91406	0.53348	0.11039	0.26909	-0.93722
PEX3	3.14848	2.7911	2.79241	3.39922	3.31686	3.21445	3.79613	3.20137	2.86516	2.74452	2.83182	2.6611	3.12881	3.36426	1.95173	2.55939
VDAC1P8	2.91357	3.03693	2.97961	3.352	5.27965	4.63792	5.56326	4.38388	3.54971	3.0664	3.40868	2.8887	3.43641	3.45943	3.36462	3.45138
FUCA2	5.59847	5.69435	5.66203	5.98044	7.87558	7.66639	8.06396	7.4559	6.19111	5.76949	5.91736	5.84552	6.0082	5.99081	5.83645	5.99484
PHACTR2	0.10433	-0.03354	0.09312	0.28374	2.49387	1.08441	3.22289	1.21962	0.88922	0.09336	1.01419	1.02417	1.6227	1.14796	1.89447	1.2838
LTV1	5.49115	5.22159	4.90303	5.31778	5.63347	5.59966	5.93386	5.68171	4.19137	3.908	4.30249	3.7786	5.24759	4.56191	4.60478	3.65615
PLAGL1	-3.32193	-3.32193	-3.17728	-2.78638	0.31233	1.01864	0.4729	0.96556	-3.32193	-2.99136	-3.32193	-3.32193	-3.22467	-3.04875	-3.09545	-3.20175
HYMAI	-3.32193	-3.32193	-3.32193	-2.65702	-0.51304	-0.87445	-0.33525	-1.5219	-3.32193	-3.32193	-3.32193	-3.32193	-3.32193	-3.32193	-3.32193	-3.32193
SF3B5	7.56678	7.38182	7.31937	7.06139	7.55297	7.99174	7.0344	7.58134	6.92702	6.88171	7.02282	5.83576	7.44875	7.28281	6.81352	6.77479
STX11	3.29449	3.18598	3.10398	3.07773	4.0707	3.90019	3.91007	3.10158	0.75919	0.15097	-0.09711	-0.49163	2.6883	2.81805	2.55362	2.15866
UTRN	1.64826	1.83898	2.0957	2.02087	4.12068	2.45382	4.775	2.54429	2.87574	2.4106	2.88971	3.18635	3.86268	3.37981	4.95917	4.44722
EPM2A	-1.90641	-1.82374	-2.16182	-1.81503	-1.98698	-2.11612	-2.10589	-1.4826	-2.14568	-2.18049	-2.49219	-2.35956	-1.97993	-1.0276	-1.74172	-1.15242
FBXO30	2.49775	2.41184	2.11624	2.86567	2.17276	1.30913	2.58942	1.4507	1.53795	1.29336	1.23204	2.03338	2.45572	2.08955	2.23452	2.17967
SHPRH	-0.26034	-0.35549	-0.21565	0.30897	0.43728	-0.16494	0.73908	0.04496	-0.35791	-0.6656	-0.64855	0.42563	0.86144	0.48389	0.01679	0.49105
RAB32	4.90121	5.43265	5.83077	5.32017	6.59035	6.04153	6.593	5.66589	5.78016	5.40841	5.51781	4.8275	5.88276	5.96415	6.04928	5.5986
STXBP5-AS1	-0.32693	-0.11084	0.12999	0.17234	2.15333	-0.62528	0.9136	-0.95835	0.52177	0.41426	0.62721	-0.28249	-1.46196	-1.88178	-1.05244	-1.31329
STXBP5	1.16051	1.2002	1.41878	1.77033	3.48413	2.02658	3.92249	2.00073	1.78997	1.56072	1.73226	2.69781	1.55405	0.95605	1.04837	0.75387
SAMD5	0.99363	1.19635	0.75346	0.74956	-3.24858	-3.32193	-3.32193	-2.95218	-1.42969	-1.0982	-1.21503	-1.01211	-3.32193	-3.32193	-3.01801	-2.35202
SASH1	2.221	2.41625	2.18774	2.40024	1.94186	0.53113	2.02589	0.61654	2.67959	2.8138	2.70205	3.07715	3.65361	2.82292	3.14806	3.61426
UST	2.63372	2.67808	2.30929	2.50113	-2.87706	-3.32193	-2.75993	-3.32193	2.52961	2.68873	2.73438	3.11203	-2.47432	-3.14206	-1.9302	-2.10291
UST-AS1	1.65243	1.12042	0.7184	1.43683	-2.77432	-3.32193	-2.3114	-3.32193	0.69259	1.20239	1.48674	2.07789	-3.32193	-3.32193	-3.32193	-3.32193
TAB2	3.40585	3.74481	3.83308	4.16338	3.34775	2.39077	3.94004	2.35256	3.92177	3.88575	3.74246	4.82803	4.32486	4.01966	4.4649	4.03404
ZC3H12D	-1.6483	-0.75719	-1.09319	-1.71916	0.71556	0.59476	0.34804	0.76903	-3.32193	-3.03232	-2.54247	-2.91286	-3.19719	-3.19762	-3.32193	-3.02917
PPIL4	4.10438	4.00901	4.06568	4.38868	3.42975	3.68396	4.01296	3.58036	3.64504	3.67486	3.59723	4.04247	3.96237	4.10854	3.67966	3.47882
GINM1	4.72479	4.90018	5.1221	5.36762	5.25417	5.51487	5.52833	5.35176	4.66117	4.7207	4.20537	5.03116	5.39222	5.46044	5.01661	5.09433
RPS18P9	2.62621	2.48333	2.23187	2.65004	1.76393	3.16522	1.70037	3.02276	1.77987	1.59863	0.88585	1.32147	1.45608	1.14233	1.42083	1.89619

KATNA1	3.28556	3.18915	3.33053	3.33286	3.31119	3.3112	3.43346	3.1209	3.54953	3.32985	3.24349	3.45976	3.35652	3.60278	2.80792	2.63314
LATS1	1.50073	1.45983	1.44206	1.7111	2.23715	1.23367	2.77736	1.20176	1.60074	1.07057	1.34854	2.29063	2.9809	2.2214	2.59283	2.4404
NUP43	4.57567	4.14658	4.32313	3.90476	4.65029	4.12675	5.0701	4.20392	4.01254	3.3115	3.64901	3.64205	4.32339	4.07435	4.07492	3.73071
PCMT1	6.03319	6.1201	6.20559	6.29962	6.21845	5.72782	6.57112	5.42287	5.04048	4.66322	4.63224	4.26464	5.67319	5.7281	5.47072	5.20658
LRP11	4.27094	4.50562	4.55319	4.38988	5.48327	5.16039	5.42428	5.23724	3.94318	4.02793	3.88198	3.87843	3.66725	3.62255	3.74266	3.69506
RAET1E	2.2868	2.36339	2.32999	1.90803	-3.17172	-3.32193	-2.18061	-2.93534	-0.6426	0.06588	-0.84944	-0.10044	-3.32193	-3.32193	-2.74338	-3.32193
RAET1E-AS1	2.78641	3.15108	2.79476	3.19676	-0.90006	-1.4763	-0.7884	-0.21271	-0.0077	0.12384	0.04301	1.26942	-3.32193	-2.32089	-1.5355	-2.1508
RAET1G	0.25919	1.1443	0.8243	1.76413	-0.82159	-1.45785	-1.76116	-1.08091	-0.62955	-0.61263	-0.64147	0.25009	-2.82708	-2.82857	-2.32387	-1.98333
ULBP2	2.535	1.73499	2.28429	2.66588	0.32011	0.63622	0.68141	-0.11847	1.60524	1.34544	1.70435	0.88387	0.62512	-0.37018	-2.2525	-1.02031
ULBP1	-2.74272	-2.06718	-2.65718	-3.32193	-0.8596	0.86653	-0.42351	-0.02708	1.84914	0.91569	0.95785	0.17676	-1.67636	-1.59338	-2.04989	-2.18098
ULBP3	-3.32193	-3.32193	-3.32193	-3.32193	-1.32098	-2.47855	-0.88908	-3.32193	2.45607	2.09297	2.9541	1.51368	-3.32193	-3.32193	-3.32193	-3.32193
MTHFD1L	4.28991	3.79988	3.90753	3.38705	3.91717	3.3849	4.00911	3.15784	5.01494	4.03491	4.61776	4.19491	4.06618	3.06032	3.98612	2.78724
ZBTB2	3.57958	3.30076	3.15992	3.4479	3.14175	2.71158	3.37095	2.81983	3.26568	2.84731	3.03882	3.18595	3.9046	3.48008	3.63251	3.57462
RMND1	4.09654	4.09564	4.03676	4.31004	3.20789	3.21101	3.54858	3.19598	2.98064	2.76929	2.83741	3.47339	3.27163	2.36605	2.69381	2.34456
ARMT1	3.86063	4.22519	3.93544	4.57522	4.44329	3.43895	5.0775	3.43841	3.13824	3.42574	2.83111	3.43309	4.07915	4.13354	3.33466	3.77556
SYNE1	-1.73456	-1.98176	-2.18596	-1.85444	-1.64529	-1.59956	-1.14467	-1.58006	0.41767	0.5035	0.64401	1.19725	2.01299	2.55597	2.47208	2.4182
SYNE1-AS1	0.71706	-0.80445	-1.20908	-1.85113	-0.29464	-0.07186	-0.17456	-1.20469	1.5227	0.24207	1.09455	1.46518	2.87908	3.59001	3.55554	3.72772
MYCT1	-2.49945	-1.47895	-1.54469	-1.4792	-3.32193	-3.32193	-3.32193	-3.32193	-2.64795	-2.33061	-3.32193	-2.02506	-0.35424	-0.73723	-1.75807	-0.41522
FBXO5	3.24413	2.71747	2.93215	2.70963	2.90108	1.74637	3.26693	1.83513	3.00145	1.86933	2.31221	1.61169	3.73659	3.41184	3.22653	2.83879
MTRF1L	3.6756	4.22125	4.01947	4.39312	3.55532	3.55824	4.42944	3.48041	2.51498	2.85021	2.737	3.10824	2.92582	3.95932	3.61342	3.81245
RGS17	-2.94924	-2.49657	-3.32193	-2.86553	-1.36612	-0.33024	-0.74839	-0.23796	0.03574	0.40299	-0.2119	1.45208	-1.52981	-1.81561	-2.43887	-1.2639
CNKSR3	-0.04782	-0.15872	-0.41864	-0.33067	0.69001	0.23529	0.56637	0.377	0.44723	0.51877	0.09177	-0.31677	0.6473	1.57797	1.37941	0.91129
SCAF8	3.40307	3.18014	3.17121	3.4763	3.47241	2.92911	3.85344	2.92181	3.37213	3.14833	3.39438	3.92362	3.81421	3.36806	3.92226	3.69501
TIAM2	1.44256	1.31881	1.29404	1.52092	0.89808	1.21308	1.33624	0.96283	1.28631	1.01507	1.0174	1.80234	1.60507	1.5257	1.74751	1.66317
TFB1M	3.25493	3.44117	3.36689	3.56631	1.92547	2.66079	1.92867	2.1763	2.785	2.87752	2.46897	2.78172	2.41211	2.48901	2.05745	2.07884
NOX3	-3.32193	-3.32193	-3.32193	-3.32193	-3.32193	-3.32193	-3.32193	-3.32193	-3.32193	-3.32193	-3.32193	-3.32193	3.38711	2.68252	2.50801	1.90106
ARID1B	0.76513	0.79835	0.83359	0.85609	2.00758	0.81993	1.76422	0.91308	1.36143	1.1238	1.47074	1.56103	0.91414	0.59674	1.62385	1.33073
TMEM242	1.86514	1.66735	2.01439	1.96258	2.19046	2.42546	2.69955	2.12886	1.47547	1.56978	1.62191	1.44049	2.39108	2.36792	1.30886	1.81758
ZDHHC14	0.27458	0.21098	0.10518	0.0107	2.5731	1.83658	2.20616	2.19349	-0.56501	0.40578	0.08617	0.21232	-0.06957	0.49315	0.85791	0.87709
SNX9	4.54733	4.72457	5.0189	5.38504	4.86766	4.41422	5.31014	4.18082	4.11418	3.88156	4.12281	4.39102	4.8416	4.24245	4.8568	4.3168
SYNJ2	3.92741	3.72193	3.85467	4.018	3.25205	1.51473	3.19777	1.88983	2.16917	2.52936	2.28358	3.21984	3.57142	3.31816	3.91481	4.29924
SERAC1	0.3571	0.53109	0.42603	0.98344	1.46654	0.9688	1.92549	1.17222	-0.00765	-0.16349	-0.44557	0.47878	1.13097	1.18357	0.78951	0.72555
GTF2H5	2.75491	2.74468	2.79108	2.81316	2.97519	3.29318	2.94885	3.20846	1.98947	2.16408	2.09786	1.25795	2.92017	2.52023	2.18818	2.06002
TULP4	1.59093	1.59525	1.6502	1.6205	1.63215	0.81621	1.94949	0.90563	1.52027	1.21637	1.57197	2.00822	2.28135	1.53422	2.89192	2.63308
TMEM181	3.19263	2.94412	3.08339	3.59227	4.23368	3.76665	4.66269	3.74864	2.60292	2.54463	2.77134	3.341	3.54245	2.77254	3.05201	2.96873
TATDN2P2	-0.32676	-0.46167	-0.65066	-1.01119	2.18344	-0.7526	1.92986	-0.10562	-0.98487	-0.72662	-0.22011	-0.27375	1.29225	1.1874	1.67619	1.4792
DYNLT1	4.83009	4.63198	5.37852	4.88767	4.54529	4.81488	4.65011	4.13737	3.74821	3.23868	3.8022	2.56394	4.50971	3.08446	3.85527	2.49104
SYTL3	1.78659	1.8437	2.15374	1.75775	-1.82566	-1.14479	-1.32124	-1.49408	0.09177	-0.15019	0.0999	0.34333	-0.33487	-1.2018	-1.49057	-0.62324
MIR3918	4.13958	4.0841	4.31281	3.89232	-0.30603	-0.08348	0.72943	-3.32193	0.09765	2.30114	1.83111	1.3065	2.41424	1.03873	0.76005	2.31799
EZR	6.31078	6.19509	6.21053	6.06738	7.2424	6.63958	7.69945	6.39116	7.4411	6.74912	6.94856	6.66601	6.81775	6.75275	6.86782	6.67167
EZR-AS1	6.73718	6.71805	6.77933	6.19465	8.54073	6.39844	9.1741	5.72383	8.49732	7.75949	7.92133	7.27974	7.61235	7.32519	8.10322	7.84398
C6orf99	-3.32193	-3.32193	-3.32193	-3.32193	1.39649	0.72507	0.96749	0.35853	2.8352	1.39322	1.67299	-0.06412	-2.54976	-3.32193	-1.47879	-3.32193
RSPH3	0.06536	0.92024	1.05383	1.1215	1.23002	1.56487	1.45367	1.19843	0.99818	1.45155	1.33707	2.12478	0.27749	0.25055	0.94833	1.10755
WTAP	4.48961	4.76579	4.80898	5.74397	3.88146	3.63411	4.23946	3.76622	3.81644	3.71336	3.82303	4.05812	4.10119	3.89811	4.06283	4.06538
ACAT2	4.2739	4.12478	3.84674	3.95481	5.43035	6.01445	5.42247	6.09921	5.3516	4.07595	4.62606	3.79287	5.5472	5.94658	5.62231	5.32716
MRPL18	6.77764	6.57377	6.47529	6.42566	6.37707	6.7521	6.31768	6.74483	5.83081	5.25554	5.4827	4.85508	6.22986	6.04191	5.54584	5.28701
SLC22A1	-0.50585	-0.5882	-1.12679	0.26523	-2.22394	-1.89055	-1.96199	-0.78804	-3.32193	-1.93317	-2.89137	-1.49579	-1.36108	-1.27068	-3.32193	-0.68005
SLC22A3	1.91177	2.28911	2.1233	2.21288	0.21822	-0.49534	0.34311	-0.88587	-0.70849	-0.00845	-1.36522	-0.2023	1.74452	3.69269	3.19064	3.35858
MAP3K4	2.41447	2.21764	2.22397	2.29083	2.27282	1.64402	2.44247	1.72381	2.23432	1.8248	2.21498	2.16731	2.49082	2.40689	3.16921	2.78377
AGPAT4	1.73191	1.84672	2.05587	2.35208	-1.50697	-0.73425	-0.93969	-0.77534	-0.21543	0.65673	0.48745	1.21628	0.85259	1.88471	1.93605	2.18801

AGPAT4-IT1	0.62896	1.00131	1.17267	2.07776	-2.88786	-1.90434	-3.32193	-2.33417	-1.77523	-0.69416	-0.92106	-0.81063	-1.23866	0.0947	-0.37563	-0.07869
CAHM	0.07427	-1.03241	-1.71197	-0.62872	0.47258	1.49312	-0.27282	1.36326	-1.7304	-2.44593	-1.12364	-3.32193	-0.63736	0.46964	-0.39288	-0.7198
QKI	2.61758	2.70266	2.82004	2.69606	2.75207	1.19047	3.20972	1.0875	3.08994	2.60535	3.12963	3.15567	3.61035	2.928	4.32	2.83912
PDE10A	-3.07271	-3.13151	-3.21831	-3.10943	-2.58436	-1.84716	-2.36682	-2.28868	1.31558	1.57481	1.14806	2.03498	-3.32193	-3.25282	-3.23785	-3.32193
LINC00602	-3.32193	-3.32193	-3.32193	-3.32193	2.88651	3.31907	3.12024	2.24767	-2.799	-1.49941	-2.84752	-2.54574	-2.19906	-2.20184	-2.54147	-2.25132
SFT2D1	4.48887	4.65637	4.49342	4.53247	4.4311	4.98645	4.54224	4.84646	3.43622	3.64028	3.65363	3.19825	4.05936	4.28409	3.52794	3.55023
MPC1	3.24607	3.00808	3.09516	3.51653	3.97333	4.47116	3.92496	4.55499	2.78008	2.30479	2.52823	2.20825	3.32918	3.16298	2.51295	2.57986
RPS6KA2	0.37381	0.69759	0.98112	0.46854	-2.92149	-2.85643	-3.32193	-3.32193	-3.17209	-3.32193	-3.18766	-3.08496	-2.869	-2.10593	-2.09024	-2.31422
RAMACL	4.4466	4.6269	4.38401	5.10517	4.63082	4.87581	5.32218	4.91114	4.44369	4.64657	4.2614	3.65197	4.70243	5.01239	3.77673	4.17568
RNASET2	2.89369	2.79937	3.05603	2.65345	3.02407	4.30462	2.82108	3.91887	2.41703	2.96284	2.82262	2.71145	1.51629	1.11173	1.91998	1.81914
FGFR1OP	1.77949	1.60514	1.54622	1.88503	2.1105	1.60911	2.32427	1.69699	0.90568	0.52896	0.48485	0.88162	1.12812	1.55469	0.39743	0.72146
CCR6	-2.87059	-3.32193	-3.03791	-3.32193	3.65482	1.8518	4.20634	1.64907	-3.32193	-3.32193	-3.32193	-3.09037	-3.32193	-3.32193	-3.32193	-2.87997
AFDN-DT	-2.33224	-2.24713	-1.28129	-1.91092	1.4455	1.17598	1.16801	1.35738	-2.85524	-0.96603	-2.57279	-1.17577	-2.72276	-2.7245	-3.32193	-1.9416
AFDN	2.50098	2.57387	2.79546	3.23999	4.11805	3.09264	4.40601	3.32543	2.41342	2.48645	2.4846	3.42774	2.75248	2.69819	3.42706	3.0962
FRMD1	-0.40154	-0.39172	0.03054	-0.67863	-2.3451	-2.69684	-2.41414	-2.75782	-3.32193	-3.14649	-3.32193	-3.14929	-3.32193	-3.32193	-3.32193	-3.32193
DACT2	-3.32193	-3.32193	-3.32193	-3.32193	5.26601	5.24184	4.63446	5.32485	-3.04261	-3.32193	-3.32193	-3.09022	-2.80395	-3.12851	-3.32193	-1.43822
LINC01615	2.85652	3.56513	4.1332	2.82209	-3.32193	-2.51911	-1.41527	-2.4144	6.11033	6.77974	6.61539	6.21673	-0.83263	-0.83686	-1.68542	-3.32193
THBS2	-2.17575	-2.00119	-1.80159	-1.00514	-3.05157	-3.32193	-3.32193	-2.95611	-0.65315	0.9273	0.37185	1.20684	6.49821	6.62227	7.74203	7.74623
WDR27	1.3446	1.31588	1.80442	1.50842	0.94685	1.8204	0.44985	1.57271	0.07189	1.01016	0.60464	1.22262	1.09082	1.74541	1.10238	1.58767
C6orf120	3.63678	3.98285	4.06032	3.88702	4.89181	5.05826	5.17904	4.75827	4.28908	5.23338	5.05839	5.34727	4.0437	4.9455	4.71701	5.04748
PHF10	3.94363	3.5602	3.79259	3.69133	3.99708	4.03373	4.35727	3.84253	4.57106	4.12301	3.942	4.62622	4.87032	4.15937	4.2072	3.79086
TCTE3	-0.41462	-0.26916	-1.15153	-1.19828	0.25344	1.17091	0.0538	1.07296	-2.5674	-2.67116	-1.53559	-2.68004	-1.01851	-0.61324	-0.93438	-0.83272
ERMARD	1.60381	1.2477	1.41898	0.88882	1.85302	1.86561	1.74634	2.21367	1.49383	1.58732	1.64439	1.65361	1.03228	0.9968	0.70836	0.92561
DLL1	-0.61409	-1.33266	-1.37698	-1.10639	-1.98128	-2.53137	-2.39723	-1.60418	0.71197	1.34252	0.5319	0.55427	-1.53523	-1.53889	-0.83806	-0.44635
FAM120B	2.22341	2.35845	2.28499	2.68295	2.11856	1.99229	2.24802	2.03432	2.51001	2.52932	2.53132	3.16151	2.28101	2.33463	2.26891	2.74585
PSMB1	6.56173	6.44326	6.49725	6.69297	6.19891	6.53169	6.42683	6.36507	6.16669	5.94963	6.22632	6.13914	6.10829	5.81461	5.65686	5.1062
TBP	3.70683	3.67409	3.79333	3.79047	3.1239	3.21848	3.52843	3.2953	2.69658	2.73428	3.06267	3.1474	3.21017	3.71043	3.02782	3.00717
PDCD2	3.62764	3.72999	3.56239	3.51308	3.74086	3.54797	3.67396	3.50965	3.42155	3.42387	3.42657	3.2559	3.26611	2.59474	2.44151	2.34877
RPL23AP47	-1.89581	-1.78899	-0.51526	-0.43638	2.29308	2.4748	1.10259	2.87272	-1.02259	-1.92852	0.20001	0.28645	0.50773	0.36221	-0.42595	-0.75159
FAM20C	7.05792	7.66135	7.91328	7.20582	4.4564	3.9248	3.69129	4.23004	5.38159	6.4648	5.96835	6.21829	2.95811	2.63514	3.98365	4.00328
PDGFA	4.36009	3.73791	4.67283	3.74294	2.29868	1.91358	2.11222	1.81752	3.12564	2.3641	3.9684	2.82809	-0.29027	-1.15105	0.59478	-0.06257
PRKAR1B	4.4727	4.6148	4.46927	3.87083	3.58466	3.22122	2.95651	3.55264	4.07837	4.52235	4.32636	3.49482	3.57525	3.41822	4.22841	4.08302
DNAAF5	4.78328	4.67519	4.62083	4.16563	4.15477	3.39098	3.43011	3.42906	4.79477	4.64173	4.71902	4.22478	3.92809	4.16885	4.33261	4.40578
SUN1	4.71623	4.56557	4.81862	4.68089	5.06935	4.59758	4.92313	4.51325	4.05295	4.0944	4.05851	4.04349	4.99546	4.78581	5.03559	4.84298
GET4	4.87522	4.79067	4.86413	4.75159	5.04658	5.08167	4.63589	4.99746	3.67415	3.71973	3.81014	3.16503	4.36384	4.46677	4.98177	4.76903
ADAP1	-0.06098	0.73641	0.79478	0.46201	-0.25873	0.10652	-1.37245	-0.03601	-0.14884	0.39216	-0.32398	-0.61633	-0.18317	0.55037	1.73139	1.58625
COX19	2.06187	2.18534	2.4041	2.11869	1.7835	2.25478	1.65942	2.33069	1.59934	1.92607	1.72516	1.67506	2.42392	2.62467	2.4244	2.53237
C7orf50	5.5088	5.35555	5.30257	4.69559	4.80149	4.84449	4.12504	4.68393	4.79297	4.85445	4.76798	3.56633	5.01662	5.24452	5.04723	5.3359
GPR146	0.4617	0.83317	1.26227	1.46805	-0.81249	-1.42213	-1.4146	-0.97432	1.35342	1.39735	0.63252	0.82897	-0.64664	-1.04853	-0.40243	0.26607
GPER1	-0.48163	0.27087	0.08893	0.92949	-0.58804	-0.80221	-0.61673	-0.36914	-2.22033	-1.78521	-1.71663	-1.22733	-0.51752	-0.40706	0.57371	1.36588
ZFAND2A	3.67712	4.1115	3.77449	4.06006	4.0671	4.65416	4.11654	4.52141	3.35399	3.87782	3.01816	3.32695	3.87327	3.88641	2.65645	3.14286
MICALL2	2.09684	1.72315	2.33611	1.92623	1.79789	2.95075	0.89002	2.17829	0.66852	1.24377	1.42339	1.38095	0.68947	1.29683	1.0837	1.39791
MAFK	5.88979	6.16227	5.83565	5.01238	6.15141	6.73599	5.67924	6.70314	3.95229	4.14244	3.2085	3.00693	4.52015	5.16089	3.99012	4.39444
TMEM184A	3.38977	3.65028	3.36981	3.2368	5.15108	5.13991	4.63494	5.11173	0.98842	1.19903	0.44016	0.7993	2.01225	2.85939	1.7139	1.81384
PSMG3	4.69588	4.6737	4.52315	4.39199	5.26972	5.69897	4.99827	5.34022	3.9645	3.81902	4.05228	2.89143	4.10637	4.70921	3.9809	4.02259
PSMG3-AS1	-1.59056	-1.9187	-0.82879	-0.64474	0.34145	0.58046	0.03336	0.55134	-0.58246	0.2573	0.06884	0.23477	-0.72001	-0.59403	-1.22944	0.16986
ELFN1	-0.93266	-0.63544	-0.62145	-1.49485	-3.09363	-3.32193	-3.32193	-3.32193	0.67951	-0.09392	-0.58947	-2.09574	-3.11012	-3.32193	-3.32193	-2.33286
ELFN1-AS1	3.12636	2.80123	2.35627	2.66476	-3.32193	-3.32193	-3.32193	-1.80249	-3.32193	-3.32193	-3.32193	-3.32193	-3.32193	-3.32193	-3.32193	-3.32193
MAD1L1	4.97034	5.03616	4.96558	4.30969	2.89974	2.38185	2.48054	2.56613	3.57486	2.88305	3.30214	2.54585	2.65876	2.10939	3.08643	2.85059
MRM2	4.53406	4.36877	4.40951	4.21506	4.40134	3.50608	4.31653	3.33175	4.09393	3.29868	4.03967	3.14946	3.97777	3.42127	4.24568	2.94845

NUDT1	4.62991	4.29732	4.34401	3.85045	4.64742	4.90419	3.92848	4.91341	5.01527	4.41308	4.55895	2.85522	4.33651	4.49033	4.0834	3.96236
SNX8	5.59345	5.37996	5.29577	4.50718	5.10881	4.12245	4.51976	4.11451	5.12647	4.64408	4.96021	3.69025	4.73047	4.55931	5.30069	4.99081
CHST12	2.81509	3.12487	3.10022	2.48356	2.38039	2.2792	1.75343	2.3072	1.45591	1.95375	2.19457	1.59092	1.073	0.83301	1.60736	1.39186
LFNG	1.87381	2.44006	2.49612	3.1114	0.76626	1.84385	-0.10994	0.56306	4.18215	3.78504	4.88397	3.112	1.28118	2.35513	2.85882	3.55799
BRAT1	4.92218	4.73931	4.71872	3.95212	4.32475	4.38807	3.19833	4.46941	4.28872	4.3985	4.59692	3.57456	3.38024	3.22834	3.97097	4.06267
IQCE	2.69868	2.75213	2.85102	2.6849	3.37935	2.20994	3.2599	2.20968	2.68187	2.89655	3.25678	3.1223	2.40739	2.61183	3.16504	3.37671
AMZ1	-0.12157	0.79918	1.18718	0.4412	-2.55197	-1.96186	-2.82283	-1.81433	-3.32193	-3.1756	-2.77723	-2.61736	-2.35551	-2.89361	-2.81323	-2.34208
GNA12	5.29783	5.36335	5.369	5.17988	4.57377	4.1781	4.50675	3.68661	5.39461	5.33039	5.38174	5.65503	4.61418	4.6026	5.15185	5.15102
SDK1	-3.32193	-3.32193	-3.21594	-3.32193	-3.24516	-3.32193	-3.32193	-3.11646	-0.17575	0.08642	-0.35865	-0.04057	-2.99764	-2.99868	-2.68697	-3.32193
FOXK1	4.08495	4.12641	4.15473	3.42708	3.52917	2.40334	3.40325	2.49613	4.05388	4.75764	4.62679	4.68792	3.25487	3.65308	4.65762	4.94726
AP5Z1	4.96082	5.02893	5.03488	4.77456	3.84187	4.42294	3.08762	4.45632	3.06282	3.74721	3.71241	3.0624	2.96939	3.39621	3.35384	3.74662
RADIL	-3.32193	-2.98008	-3.13149	-3.32193	-0.12107	0.2581	-1.36188	-0.01082	1.45647	0.92847	1.46095	-0.98138	-0.77292	-1.29747	-0.00232	-0.91574
RNF216P1	2.55116	2.15306	2.24463	2.19566	2.96442	2.64441	3.32011	2.61225	2.64526	2.68472	2.73689	3.15376	2.99622	3.23304	3.22327	3.10808
RBAK-RBAKDN	3.05529	2.75349	3.10474	2.68958	3.99714	2.908	4.43612	2.74417	3.54965	3.80126	3.70632	4.23474	3.94507	4.33211	4.15626	4.26553
RBAK	1.92683	1.98367	2.1354	2.71711	2.10301	1.94653	2.64904	2.18371	2.22511	2.51698	2.23548	3.48812	2.24986	2.60002	2.67389	2.82281
ZNF890P	1.54347	0.72655	1.18292	1.12897	-0.06802	0.12946	-0.83963	0.28219	-1.10919	-0.12243	-0.33223	-0.71715	-1.53519	-0.60366	-1.0471	-0.68214
WIPI2	4.63181	4.77647	4.68408	4.94034	3.36411	3.18921	3.4735	3.05058	3.84802	3.90992	4.02222	4.084	3.57112	3.77676	3.89166	4.04453
SLC29A4	-0.88696	-0.48474	0.04764	-0.40055	3.94683	5.28858	2.24421	5.55882	2.79625	3.64713	2.79964	3.11423	1.46895	1.52275	3.15152	3.13643
TNRC18	4.95755	4.73243	5.03369	3.74367	4.62956	4.04032	3.65589	4.09579	5.67661	5.15009	5.91313	4.53163	3.33584	2.87073	4.90238	4.6061
FBXL18	4.01457	4.25584	4.0826	3.39212	3.3813	1.54714	2.80561	1.55107	3.68151	3.41829	3.63157	3.13838	2.76895	2.70976	3.37601	3.75777
FSCN1	7.7638	7.97376	8.39613	7.13373	3.08312	3.65508	2.83286	2.86314	6.82998	6.58196	6.79597	6.00069	6.71367	6.35241	7.88564	7.48287
RNF216	3.74347	3.52633	3.44481	3.47518	3.4512	3.54809	3.35963	3.62778	4.61839	4.25012	4.44182	4.35994	3.66235	3.78758	4.07531	4.13879
ZNF815P	2.28911	2.21445	2.23065	2.20526	1.15932	1.05459	0.9451	1.15404	0.05733	1.0361	1.08797	0.2853	0.0716	0.50178	0.33207	0.53501
CCZ1	4.9735	5.01881	4.73379	5.16663	4.11024	3.80339	3.92127	4.20177	3.9726	3.73681	3.32037	4.04793	3.76066	3.80306	2.62849	3.29689
RSPH10B	3.96037	3.86082	3.66633	4.28157	2.65209	2.91639	2.13104	3.54291	2.47418	2.11834	1.67434	2.34921	2.48341	2.56964	0.85335	1.53162
PMS2	2.94699	2.98819	2.93021	3.05996	2.64121	2.25254	3.11724	2.1578	2.7094	2.62869	2.80825	3.24548	3.25613	3.34364	3.31959	3.46081
AIMP2	5.97671	5.69378	5.45362	5.60631	5.10238	5.11434	4.97614	5.26572	5.32383	4.99186	5.37135	4.82586	5.62811	5.61911	5.2766	5.14962
EIF2AK1	5.74086	5.70453	5.60119	5.88418	5.53481	5.29984	5.67155	5.22718	5.26715	5.14637	5.16636	5.42179	5.64583	5.78934	5.83061	5.51219
ANKRD61	0.41671	0.50257	0.57516	-0.63125	-0.21681	-0.46539	1.15864	0.50782	-1.12393	-0.65323	-1.25218	-0.97219	0.49164	0.58283	0.70726	1.06238
USP42	2.51299	2.65441	2.62266	2.59869	2.1892	2.21704	2.31207	2.29768	2.61377	2.49164	2.83998	3.04918	2.3394	2.50518	3.00573	3.07406
CYTH3	3.43168	3.22098	3.35056	3.11317	2.65477	1.80374	2.55465	1.49461	3.89598	3.59962	3.60212	3.72786	4.34153	3.89242	4.27062	3.60745
FAM220A	3.95955	4.09474	4.06713	4.09055	3.68462	2.776	3.5762	2.70859	4.16625	4.34247	4.05456	3.91568	4.14516	4.08304	4.38697	4.01761
DAGLB	4.35761	4.26866	4.18579	4.08335	4.6312	3.61756	4.72824	3.69087	4.85568	4.57702	4.76471	4.45163	4.52499	4.16098	4.59843	4.41975
KDELR2	6.38688	6.37312	6.40268	6.50632	7.20381	6.70916	7.31873	6.52386	6.60268	6.23273	6.29806	6.61115	6.51983	6.24202	6.17567	6.04453
ZDHHC4	3.44635	3.6057	3.61161	3.85034	4.18719	3.90817	3.56457	3.7977	4.07156	3.95631	3.50833	4.07331	2.99026	3.05188	2.62831	3.07112
C7orf26	3.79684	3.50744	3.3569	3.17105	4.06507	3.39015	3.64408	3.57817	3.95823	3.53202	3.55718	3.38636	4.13173	3.70821	4.03542	4.24845
ZNF853	-2.64055	-3.32193	-3.32193	-3.32193	-2.43081	-1.16531	-2.8618	-1.05008	-2.76828	0.01719	-1.2769	-0.18096	-2.76324	-0.17558	0.20996	0.7236
ZNF316	4.85877	4.89128	4.90641	3.91544	4.21308	4.33777	3.67148	4.67968	4.27661	4.41882	4.52521	3.58518	3.5229	3.93658	4.55612	4.57497
ZNF12	2.25077	2.49204	2.39383	2.89951	2.48888	2.09181	2.99105	1.92395	2.73854	3.5519	3.00963	3.9285	2.62043	3.20415	3.09582	2.97002
PMS2CL	3.22402	3.04082	2.93766	2.90703	2.45614	2.77753	2.24238	3.19646	2.84198	3.08297	2.93055	3.00513	2.54136	2.51675	2.4049	2.63945
RSPH10B2	4.04325	3.84017	3.80423	4.32488	2.43531	2.93458	2.11691	3.36046	2.47915	2.25628	1.75942	2.32424	2.06782	2.20629	0.46107	1.1133
CCZ1B	4.98171	4.9363	4.75869	5.11987	3.82208	3.5818	3.64361	3.84887	3.7801	3.54725	3.15248	3.82677	3.71772	3.75747	2.57638	3.28212
C1GALT1	2.72969	2.80455	2.59957	2.92967	3.42469	2.41089	3.89591	2.76934	2.62213	2.40522	2.35812	3.12679	4.32504	4.01996	3.74529	3.57641
COL28A1	-2.88708	-2.98482	-2.81934	-2.79178	-2.89047	-2.88293	-3.17208	-2.81767	-3.13759	-2.2306	-2.75061	-2.49158	-1.64174	-0.96605	-2.09332	-1.10109
MIOS	2.15266	2.04793	1.88823	1.88927	2.49641	1.33974	2.87796	1.28533	2.35004	2.1384	2.20621	2.47155	2.92056	2.626	2.48731	2.14422
RPA3	4.5406	4.47608	4.47468	4.39558	3.782	3.60489	3.46156	3.47943	3.93662	3.6585	3.58687	2.67425	3.9141	4.18506	3.03847	3.02465
UMAD1	2.3286	2.44976	2.514	2.82749	2.77548	2.33821	2.59163	2.04788	2.57694	2.27126	1.71936	2.50314	2.62314	2.59127	1.64232	2.0274
GLCCI1	0.01876	0.03369	-0.00755	0.45051	0.43758	0.47292	0.97266	0.34082	0.48252	0.32451	0.61952	0.14342	0.8656	0.99419	1.14369	2.75132
ICA1	-2.24415	-2.40313	-1.99771	-2.05754	-0.98747	0.45152	-1.1822	0.44843	-0.25534	0.17021	0.24482	1.24581	0.76866	0.59662	0.05564	0.82572
NDUFA4	5.13285	5.11747	4.98983	5.33367	5.81698	5.70966	5.51074	5.58014	5.35738	5.53481	5.16193	4.78107	5.65206	5.75556	4.56486	4.66451

PHF14	3.1476	3.27723	3.11595	3.90556	3.21329	3.19903	3.61395	3.40967	3.00587	3.08705	3.00798	3.62726	2.26487	2.66994	2.64994	2.68684
TMEM106B	1.09199	1.4698	1.38207	2.33712	2.04415	1.3378	2.78267	1.19963	1.37024	2.14504	1.35848	3.11408	3.09287	3.45555	2.81257	3.0356
VWDE	-2.75794	-2.97886	-2.67419	-3.32193	-3.32193	-3.32193	-3.32193	-3.32193	-3.13416	-3.32193	-2.62645	-2.89958	-0.21949	-0.33858	-0.60064	-0.3861
SCIN	-3.22867	-3.21766	-3.21209	-2.99667	-3.20413	-2.637	-3.15244	-2.83948	-1.84416	-3.32193	-2.56342	-3.32193	-2.44457	-2.05444	-2.30754	-2.91505
ARL4A	2.94199	3.19397	3.33525	3.74004	3.35163	2.74176	3.94857	2.62434	2.92576	2.38048	2.52786	2.35636	3.93191	4.2851	3.66554	3.4384
ETV1	-0.23342	0.37384	-0.16222	1.03288	-2.20725	-1.7974	-1.48943	-1.4391	1.70972	2.12601	1.57754	3.33852	3.11462	3.75167	3.55854	4.18273
AGMO	-3.32193	-3.32193	-3.32193	-3.32193	2.77505	2.27063	3.0031	2.1947	-1.14529	-1.35069	-0.80918	-0.02619	-2.30967	-1.98841	-1.52327	-1.19153
MEOX2	-3.32193	-3.32193	-3.32193	-3.32193	-0.0834	-1.51001	-0.38363	-0.25412	-3.32193	-2.94412	-3.32193	-3.32193	-2.34164	-1.18677	-3.32193	-1.9915
SOSTDC1	-3.32193	-3.32193	-3.32193	-3.32193	-3.14141	-3.32193	-2.95126	-2.8643	-3.32193	-3.32193	-3.32193	-2.4016	-0.02162	1.02535	0.40981	0.8105
ANKMY2	2.82556	2.95629	2.92727	3.3736	2.51717	2.27178	2.77121	2.45135	3.61329	4.16656	3.49621	4.47373	3.35928	3.96712	3.59016	3.59635
BZW2	5.10488	4.70732	4.49649	4.68396	3.79595	3.78136	4.13493	3.64414	5.39161	4.80403	5.15211	5.06576	5.45455	5.21761	4.96165	4.91515
TSPAN13	1.44218	2.5407	1.69694	3.06734	6.0125	6.00078	6.60731	5.77847	2.82312	2.61439	2.11428	2.83726	2.97032	4.12318	2.96284	4.45479
AGR2	-3.32193	-3.32193	-3.32193	-3.32193	6.51902	5.36642	7.06463	4.62994	-3.32193	-3.32193	-3.32193	-3.32193	-3.32193	-2.81004	-2.71699	-1.80414
AHR	2.0012	1.82355	1.96714	2.37556	4.37265	3.22827	4.96274	2.93079	3.67391	3.91966	3.7008	5.41367	5.00056	4.248	5.62134	5.46283
SNX13	0.90878	0.97649	0.7527	1.80801	1.79281	0.71704	2.30212	0.62763	1.34464	1.42963	1.22527	2.08891	2.00657	2.13238	1.79742	2.08495
HDAC9	1.29142	1.31115	1.55117	1.53929	-2.9286	-2.0878	-1.92356	-2.743	-0.08665	0.13846	0.01127	0.97604	2.6695	2.95375	2.70206	2.73314
TWIST1	1.28021	1.9526	1.70751	1.45977	-2.7501	-3.32193	-2.54565	-3.32193	4.06143	4.22424	4.32709	2.97838	3.68268	3.32109	3.49694	3.49524
TWISTNB	3.64028	3.29577	3.16297	3.90253	3.0512	2.50977	3.78118	2.66352	3.97232	3.45911	3.73942	3.78611	4.19329	3.55911	4.12383	3.42617
RPL21P75	5.74872	5.5654	5.41761	5.41613	5.06192	4.93752	5.19173	4.59583	5.92605	6.0835	5.9155	6.01543	4.99202	4.4042	3.43818	2.83924
MACC1	-3.01714	-3.32193	-2.86608	-2.84068	-3.27467	-3.32193	-3.32193	-3.32193	0.36161	0.86708	0.3071	1.39361	-3.23592	-3.32193	-3.32193	-3.11649
ITGB8	-2.93539	-2.257	-2.63784	-0.97917	-3.14498	-3.32193	-3.25456	-3.32193	-0.95271	0.41096	0.09869	1.68555	-2.44027	-0.75707	-0.98336	-0.94405
SP8	-3.04842	-3.32193	-3.32193	-3.32193	1.7149	-0.48991	1.27426	-0.80656	-3.00852	-3.32193	-3.32193	-3.32193	-3.32193	-3.32193	-3.32193	-3.32193
RPL23P8	3.45684	4.0341	3.11991	2.42362	0.03446	1.76562	-0.71833	1.1743	0.65576	3.02568	1.23526	0.71998	0.73353	0.59987	-0.69107	-0.30786
SP4	1.05625	1.03023	0.96971	0.84591	0.40784	-0.45525	0.40078	-0.3563	0.96518	0.32482	0.04068	0.72937	1.45586	1.57374	1.25948	1.4001
DNAH11	1.62688	1.38418	1.14736	1.39471	-1.18454	-0.528	-1.48148	-0.57507	1.44773	0.86517	0.694	0.97278	0.29279	0.40821	-0.07761	-0.47355
CDCA7L	4.50424	4.19686	3.9065	3.94443	2.27779	2.07792	1.93898	2.0153	5.16349	4.5683	4.44944	4.37702	3.63203	3.81703	3.52409	3.40904
RAPGEF5	-1.36634	-1.93481	-1.66475	-1.92918	2.4464	1.32044	2.7723	1.73759	-0.6704	-0.61001	-1.06886	-0.47871	-2.08007	-2.54822	-2.31287	-2.79229
STEAP1B	-2.09514	-2.51271	-2.19031	-1.49888	2.01011	1.40118	2.17332	1.49077	4.05447	3.64488	3.87241	3.79572	1.44645	1.0802	0.49753	0.52687
EEF1A1P6	8.12255	7.85785	8.12654	7.34114	7.04696	7.00398	7.45024	6.55684	7.77511	8.59422	7.60778	7.87698	6.41573	5.73457	6.205	5.94254
IL6	6.98402	7.17718	6.47833	7.37902	-2.24113	-2.85802	-2.55614	-1.63001	6.08722	6.28302	5.96689	6.02156	1.44123	1.30564	-0.7336	-0.46931
TOMM7	6.33259	6.21774	6.19164	5.51247	6.03378	5.94244	5.45665	5.90284	6.06775	6.42789	6.15154	5.29197	6.52492	6.21029	5.05649	5.11702
SNHG26	2.08276	1.56159	2.0926	1.89443	-0.16733	-1.12515	0.0533	-0.13889	0.96672	1.17962	1.40069	1.24404	0.96469	0.4031	0.77215	0.03223
FAM126A	2.33211	2.44553	2.2378	2.87525	2.17907	1.61375	2.86793	1.46869	2.70787	2.85986	2.52789	3.22432	4.08927	4.16468	3.50315	3.62089
KLHL7-DT	0.17528	-0.07495	-0.42371	0.27282	-1.08064	-0.9201	0.12291	-1.12019	-2.55327	-1.58418	-3.32193	-1.8774	-1.2109	-1.91371	-1.59422	-1.85081
KLHL7	2.69391	2.82833	2.74922	3.26693	2.52804	1.76483	2.9172	1.7285	2.46625	2.60006	2.30331	2.83992	3.17368	3.39947	3.26162	3.33023
GPNMB	3.13827	3.49496	3.47744	4.39852	-2.1374	-1.51322	-1.76309	-2.06195	-2.38927	-2.1478	-2.08932	-1.05005	-0.76116	-0.13006	-0.77297	-0.87548
MALSU1	4.72471	4.67643	4.70475	4.81701	4.13853	3.8505	4.35968	3.96876	4.54174	4.08977	4.02136	3.9788	5.39625	4.81353	4.78642	4.21777
IGF2BP3	3.45788	3.22063	3.57651	3.66345	3.64353	1.85313	3.6273	2.35053	4.57164	3.48622	3.33822	3.71929	4.91091	3.57925	4.19349	3.42055
RPS2P32	-0.90226	-0.18233	-1.28896	-2.25264	1.79191	2.95487	1.63462	2.70406	1.44865	1.85755	2.14617	2.07868	-2.08112	-2.57413	-1.20994	-1.18562
TRA2A	4.58401	4.43498	4.58511	4.52219	3.88385	3.3329	4.13358	3.53928	4.44499	3.92723	4.40487	4.42254	4.69497	4.3698	4.4043	4.10284
CCDC126	1.58867	2.20637	1.79375	3.21577	1.61007	1.23553	2.34466	1.07195	0.22319	0.12546	0.04065	0.40361	1.67813	1.64979	1.05864	1.30344
FAM221A	1.44491	1.589	1.47907	1.74762	-0.13095	0.46849	-0.02332	0.21968	-2.43696	-2.55432	-2.91766	-2.84206	0.35857	0.115	-0.17638	-0.30245
MPP6	1.92396	1.77373	1.50993	1.59344	1.31345	-0.31553	1.47674	0.14405	1.41212	0.82986	0.32185	1.06674	2.18651	1.40836	1.20579	0.88451
GSDME	3.85038	4.4219	4.48692	5.00162	-2.37616	-1.35454	-2.67117	-1.39538	2.90532	2.81555	3.15996	3.11423	0.72365	0.97017	1.01121	1.31737
OSBPL3	2.56232	2.80827	2.98987	2.70553	2.26675	1.54076	2.80649	1.60423	2.41538	2.28323	2.49029	3.27139	3.17758	3.07725	2.9053	2.80749
CYCS	6.73538	6.44452	6.07679	6.0885	6.00926	5.72139	5.88914	5.5556	6.0625	5.13109	5.07653	3.71059	6.80604	6.52792	5.4738	5.40007
C7orf31	0.45872	0.61382	0.90559	1.24647	0.64192	0.55605	0.99139	0.92933	1.02001	1.03598	1.17522	1.9408	1.74354	1.3401	1.8306	1.33794
NFE2L3	5.03143	4.85632	4.85746	4.50741	2.67264	1.56685	2.73276	1.57991	6.41128	6.32788	6.49791	6.27875	4.1758	3.4322	3.84103	3.26315
CBX3	6.06124	6.11294	6.08529	6.18224	5.54948	4.98879	5.78954	5.01537	6.03663	6.0009	5.68312	5.74516	6.34685	6.46707	5.64786	5.58832
SNX10	2.97151	3.5409	3.08574	4.61617	2.0523	1.94387	2.90948	1.83337	0.80006	1.27432	0.62276	1.13186	-0.40207	0.56341	0.03813	-0.42301

SKAP2	2.9644	3.26781	3.3139	3.88893	4.08992	3.42213	4.54331	3.42663	3.37569	3.5952	3.02763	4.32098	2.46452	2.78306	2.34872	2.75587
HOXA1	1.92552	1.43698	1.3576	1.5058	3.22995	3.59225	3.35464	3.60535	-2.88331	-2.4079	-2.35846	-1.86844	-2.54083	-1.90489	-2.65863	-2.9453
HOTAIRM1	4.37852	3.97855	4.37087	4.11476	3.99875	5.42828	3.93145	5.0331	-2.64113	-3.32193	-2.2689	-2.74486	-2.17181	-3.32193	-2.74147	-3.32193
HOXA2	-0.07016	0.66766	0.92264	1.53119	-0.16618	0.76235	-0.47604	1.3875	-1.81853	-1.98713	-1.46722	-1.43675	-3.00304	-2.01221	-2.94179	-3.32193
HOXA3	1.88941	2.4704	2.65853	2.63709	3.36299	3.91978	3.07809	4.17514	-0.06044	1.02095	0.90599	0.8685	-2.05166	-0.39345	-1.34907	-0.63204
HOXA-AS2	-0.11559	-0.1388	0.23817	0.68058	2.65917	2.81542	2.48216	3.00018	-0.38417	0.21771	-0.19025	0.21269	-3.02295	-1.60186	-1.89766	-1.60179
HOXA4	-2.40467	-2.32389	-2.71187	-2.67961	3.39697	4.21771	3.11837	4.32724	2.71256	2.90081	2.40614	2.21676	-3.32193	-2.0666	-3.32193	-3.32193
HOXA-AS3	-1.96532	-1.94015	-2.49779	-2.91224	0.5612	0.71251	0.35354	0.40291	1.93897	1.98205	1.96338	1.77788	-3.32193	-2.63557	-3.00189	-2.31084
HOXA5	1.32755	0.29008	0.94967	1.22559	3.39343	4.27513	3.2704	4.58513	1.98476	2.01647	2.06962	2.6569	-1.51729	-0.18303	-1.86966	0.37627
HOXA6	0.09272	0.15947	-0.42663	-1.98568	2.4827	1.73836	2.03663	1.17323	2.40467	2.44844	2.96273	2.29561	-3.32193	-1.79287	-2.21537	-0.80711
HOXA7	-2.91931	-2.03195	-2.5041	-1.1024	-1.92107	-2.33904	-1.83501	-2.85425	3.122	3.03048	3.17587	2.86828	-2.99747	-3.32193	-3.32193	-2.14781
HOXA10-AS	2.04805	1.96054	1.84891	2.30959	1.1714	2.51054	1.3521	2.53426	3.5706	3.3995	3.28886	3.27262	0.02721	0.86523	-1.12531	0.20564
HOXA10	1.55944	1.78111	1.50001	1.96568	0.9892	2.14351	1.18224	2.30189	3.48192	3.17693	3.15122	3.24611	-0.34209	0.40686	-0.73761	-0.03129
HOXA11	-3.32193	-3.32193	-3.32193	-3.32193	-0.91965	-2.30726	-1.16673	-2.18415	2.15074	1.35924	2.06266	0.8402	-0.67318	-1.17981	-0.63032	-1.75156
HOXA11-AS	-3.32193	-3.32193	-3.32193	-3.32193	-1.08039	-1.15291	-1.0815	-2.3589	0.6919	0.80078	0.87693	-0.06253	-2.02379	-1.43435	-1.49806	-1.8275
HOXA13	-1.05439	-1.23301	-0.5216	-0.98694	1.56151	-0.75213	1.91648	-1.49775	1.32901	1.26036	0.99783	1.19183	-2.03824	-0.98558	-1.01924	-1.56377
EVX1	-3.32193	-3.32193	-2.9723	-3.32193	0.46256	-2.05637	0.34113	-2.68908	-3.32193	-3.32193	-3.01183	-3.32193	-3.32193	-3.32193	-3.32193	-3.02811
EIF4HP1	2.01936	2.68707	2.25835	0.8571	2.30625	0.85866	2.03816	0.52978	3.68229	3.35492	2.94619	2.88413	1.99793	2.20092	2.0324	2.10076
HIBADH	4.94974	4.92599	5.22137	5.55462	4.51654	4.12344	4.65921	3.99959	4.74932	4.26098	4.60657	4.69562	4.77197	4.32193	4.34735	3.57321
TAX1BP1	4.58431	4.79687	4.83656	5.32373	4.4648	3.76579	5.19859	3.59527	4.50337	4.70012	4.39508	5.47164	4.62217	4.67158	4.35005	4.25103
JAZF1	1.32959	1.24812	1.7531	1.53747	-2.06255	-1.42166	-1.11895	-1.60107	1.63557	1.55995	0.26305	1.71573	-0.07854	0.31664	0.22541	-0.07731
JAZF1-AS1	-2.22566	-1.32902	-0.69708	-1.66432	-3.32193	-3.32193	-3.32193	-3.32193	-0.31208	-0.84363	-1.49237	-1.83751	-2.79009	-1.92759	-2.02992	-1.81051
CREB5	-0.87452	-1.07977	-1.16307	-1.01607	-2.50442	-2.90391	-2.86528	-3.21213	-2.62752	-1.74304	-2.34798	-1.04557	1.80567	1.88011	2.29052	1.87766
CPVL	4.67364	4.59689	4.7488	5.20957	-3.06205	-3.32193	-3.32193	-3.32193	-3.32193	-3.03024	-3.32193	-3.03471	-3.32193	-2.53484	-3.32193	-1.82925
CHN2	0.59715	0.59837	0.84764	0.94125	-3.19392	-3.12374	-3.05419	-3.32193	-3.32193	-3.32193	-3.217	-3.32193	-3.16605	-3.09475	-3.32193	-3.22288
ZNRF2P2	0.40599	0.8234	1.58619	-0.88407	2.47579	1.63595	2.03637	1.19253	2.60896	2.52435	2.32042	1.84152	1.76145	1.50193	1.74763	2.38495
DPY19L2P3	0.1152	-0.03109	-0.12088	-1.24549	1.15468	0.26838	0.77775	0.10359	1.3651	1.19818	1.06983	0.67947	0.5965	0.3233	0.62548	1.18112
WIPF3	-3.32193	-3.32193	-3.32193	-3.32193	-1.53714	0.02663	-2.11157	-0.40644	-3.32193	-3.32193	-3.32193	-3.00854	-2.83546	-3.05914	-2.74787	-2.73743
SCRN1	4.32358	4.73474	4.49001	4.81098	4.82925	4.34493	5.57612	4.08764	6.03603	6.24451	6.58946	6.7548	4.78372	5.46899	5.93798	5.70841
FKBP14	2.51358	2.4415	2.57351	2.83708	3.72943	3.6897	4.46299	3.32646	2.33028	2.72072	2.69724	3.08733	4.31771	4.09072	3.92821	3.9316
PLEKHA8	1.59543	1.18084	1.83251	1.78476	2.3985	1.34176	2.83268	1.41057	2.32874	1.09202	1.6725	2.07725	2.78138	1.90711	2.96662	2.42374
MTURN	2.35784	2.07298	2.22059	1.83387	0.21377	0.82364	0.3761	0.61105	0.6452	1.20872	0.67958	1.44001	1.24567	0.83094	1.38243	1.4253
ZNRF2	2.7428	2.7491	2.79523	2.7948	4.1526	2.88409	4.08715	2.60747	2.97038	2.61212	2.59424	2.2383	2.62153	1.32752	2.22377	2.01238
NOD1	1.90367	1.48291	1.66628	1.82111	0.96429	0.77471	0.86463	0.51247	1.64577	0.97493	1.8673	1.20214	1.31577	1.01355	1.55375	1.26232
GGCT	6.14194	5.9908	5.82374	5.88107	5.37015	5.65722	5.63599	5.57858	5.11802	4.68036	4.80949	4.47938	6.29799	6.28445	5.35187	5.16807
GARS-DT	-0.22829	-0.36032	0.00701	-0.21857	-0.73474	0.15685	-0.72561	0.1751	-1.81284	-1.55148	-1.53503	-0.93538	-0.47483	-0.21349	-0.80008	-0.16033
INMT-MINDY4	0.66191	0.5169	0.08107	0.36815	0.3288	-1.05452	0.54436	-0.8658	0.65281	1.15762	1.44583	1.73072	1.10292	1.32091	0.71699	0.74665
MINDY4	-0.5302	-0.68389	-0.96123	-0.68763	-0.8293	-1.88285	-0.6591	-1.67153	-0.5607	-0.04817	0.20432	0.49963	-0.13248	0.07007	-0.50215	-0.41421
AQP1	-2.39306	-1.72317	-1.67049	-0.51666	-2.0354	-2.07663	-1.59076	-2.46895	-2.9855	-2.59629	-2.76795	-1.67027	0.80422	1.58142	3.55711	3.09505
PDE1C	-2.84364	-2.67368	-3.05939	-2.81062	-3.2547	-3.16676	-3.32193	-3.22865	3.72676	3.4177	3.21679	2.57817	3.99914	3.68147	3.957	3.15838
LSM5	3.53285	3.39681	3.12527	3.25104	2.401	3.37377	2.81453	3.55393	3.07528	2.9762	2.42431	2.80133	4.56159	4.5164	3.35771	3.44766
AVL9	2.16553	2.4813	2.32837	2.7108	2.54499	2.37839	3.19961	2.41234	2.88856	3.04882	2.70807	3.59526	3.56645	3.92722	3.74325	3.91274
DPY19L1P1	1.48171	1.461	1.55372	2.26944	1.46246	1.69872	2.45686	1.66014	1.56158	1.6459	1.51529	2.78219	2.6898	2.81972	2.66161	2.71209
ZNRF2P1	4.1247	4.27582	3.8911	2.49293	3.92643	2.5718	3.52951	2.09343	4.84867	4.85743	5.01007	3.09936	3.15125	2.76585	4.40435	3.71079
LINC00997	0.75183	0.53587	0.10165	0.55587	0.33175	1.12166	0.58146	1.08643	0.76759	0.42709	0.82475	1.45502	0.56439	-0.30432	1.6565	0.66761
DPY19L1P2	1.03626	-0.7958	0.85466	0.77078	-0.10694	-0.5139	-0.61212	-0.98979	-0.33979	1.35265	0.20719	2.09807	1.06595	1.65347	1.08719	1.69841
KBTBD2	3.65144	3.68387	3.68676	4.16977	3.16087	3.14291	3.79104	3.09438	3.64704	3.35683	3.24781	3.80713	4.11603	4.11396	3.97829	3.99323
RP9P	3.67509	3.59403	3.55803	3.47352	3.58402	4.07525	3.44206	4.09649	3.08285	2.80611	2.6359	2.23908	3.70033	3.34519	3.62705	3.10591
FKBP9	4.4517	4.37556	4.72075	4.57452	6.1359	5.24562	5.94426	5.23115	5.22926	4.8763	4.87311	5.20118	4.5205	3.99282	4.76342	4.62111
NT5C3A	4.10695	3.98064	3.74912	4.05739	3.68463	3.39868	4.35084	3.60928	2.52508	2.19473	2.17614	2.05843	4.44901	4.31152	3.66929	3.37843

RP9	3.45643	3.32391	3.44026	3.55661	2.96548	3.815	3.00707	4.00912	2.53696	2.6166	2.97307	2.75864	3.28906	3.65321	3.37404	3.26263
BBS9	0.51399	0.59088	0.79299	1.2105	1.02628	1.00003	1.42462	0.64183	1.42872	1.61345	1.35946	2.17951	1.0584	0.98386	1.43061	1.42204
BMPER	-2.59155	-1.84924	-2.9298	-1.66053	-3.24334	-3.32193	-3.32193	-3.32193	2.6946	2.42284	2.88326	1.83243	0.89183	-1.80986	-1.27013	-1.53784
DPY19L1	3.42049	3.59724	3.27043	4.06493	3.93793	3.32006	4.62322	3.21341	4.15409	4.48878	4.07698	5.49179	5.8191	6.3612	6.25252	6.37848
DPY19L2P1	-2.86218	-2.44028	-2.90674	-2.76241	-3.32193	-3.32193	-3.32193	-3.16929	-0.05655	-0.61728	-0.28761	-0.39935	-1.29081	-1.19456	-1.51119	-1.82051
HERPUD2	3.22915	3.45181	3.56059	3.60926	3.55801	3.06067	3.47289	2.95949	4.0295	3.99038	3.68161	4.15402	4.03533	3.77289	3.96772	4.06492
EEPD1	2.27968	1.76084	1.73333	1.55089	3.05454	2.95563	2.94633	2.7704	0.61648	0.34879	0.4618	-0.381	1.6774	2.121	2.33722	2.80551
KIAA0895	2.17942	2.26304	2.53239	2.29873	3.19206	1.7155	3.4967	1.54237	3.54327	2.6313	2.88783	2.31538	4.56936	4.93549	4.72851	4.53675
ANLN	5.95122	5.68555	5.99307	5.23389	5.54664	3.55851	6.12389	3.79103	6.70623	5.35602	5.73605	5.11929	7.3855	7.01958	6.98577	6.40678
AOAH	0.95254	0.91603	0.648	0.99866	-3.32193	-3.32193	-3.32193	-3.32193	-1.97591	-2.27501	-2.38364	-1.42468	-2.42187	-3.0922	-3.0456	-2.26611
ELMO1	2.34008	2.05966	1.96879	2.28115	-2.85844	0.34774	-2.44794	-0.23041	-3.32193	-3.2133	-3.32193	-3.32193	-3.14935	-2.99627	-3.21433	-3.32193
ELMO1-AS1	2.86842	2.47106	2.41261	2.68014	-3.32193	-3.32193	-1.41728	-3.32193	-3.32193	-3.32193	-3.32193	-3.32193	-3.32193	-2.21928	-3.32193	-3.32193
EPDR1	4.4395	4.64624	4.34527	4.89199	2.28579	3.37881	2.4429	3.42879	4.70524	4.91665	4.48478	5.14769	5.95338	6.8849	5.79089	6.19834
SFRP4	-2.80545	-2.34644	-3.32193	-2.6958	-3.32193	-2.80065	-3.32193	-2.72548	-3.32193	-3.32193	-2.78993	-3.05405	-1.3709	1.39893	0.69656	0.06372
STARD3NL	3.94295	4.01508	3.85601	4.26539	3.93187	4.0065	4.34537	3.85322	3.97674	4.21217	3.83623	4.15245	4.1564	4.97702	4.28623	4.45
AMPH	2.39239	2.82817	2.42481	2.57073	-3.32193	-3.32193	-3.32193	-3.32193	-3.32193	-3.32193	-3.32193	-3.32193	1.37413	2.03905	1.72597	1.68603
VPS41	2.8626	3.09459	3.25061	3.42127	3.26854	2.09577	3.74602	2.14379	2.59551	3.06023	2.67047	3.77614	3.74888	4.08152	4.06375	4.15375
YAE1	3.38714	3.44401	3.20584	3.87093	2.56998	2.98544	2.82078	3.11903	2.15523	2.32866	1.75965	2.41714	2.80476	2.80457	1.79691	1.62184
RALA	5.47391	6.07851	5.92096	5.99682	4.15888	4.26293	4.74764	4.23762	5.0914	5.11059	5.02342	5.31647	4.9964	5.12645	4.85456	4.64277
LINC00265	0.65079	0.60998	0.94869	0.82133	0.4761	1.27359	-0.16691	1.37258	0.96606	1.08283	1.16498	1.0095	-1.49918	-0.18771	-0.38445	-0.60115
RWDD4P2	3.49393	3.10603	3.23407	3.5558	1.94375	2.3555	2.20282	1.52978	2.77187	2.32726	1.41714	2.05354	2.93733	2.7596	0.74795	1.67542
CDK13	3.06436	3.43587	3.42172	3.33391	3.42063	2.42936	3.12773	2.65734	3.14027	3.25495	3.19781	3.39006	2.90332	3.15189	3.2967	3.58837
MPLKIP	3.68064	3.58156	3.44203	3.53443	2.4592	2.68815	2.34187	2.83158	2.43455	2.80543	2.31184	1.36488	3.21941	3.23141	2.65627	2.42347
SUGCT	-0.3106	-0.84331	-0.62789	-0.47937	-0.42258	-0.11405	-0.17449	-0.30251	1.1477	1.15176	1.28438	1.12828	2.71585	2.46144	2.22182	1.9989
INHBA-AS1	1.74517	1.25311	1.32787	1.8297	-3.32193	-3.32193	-3.32193	-3.32193	-3.32193	-2.56422	-3.32193	-2.82882	-2.57908	-2.23891	-3.32193	-2.05999
C7orf25	3.65504	3.67346	3.5068	4.00789	2.84675	2.41757	2.66571	2.60134	2.75237	2.27013	2.31514	2.4749	2.88405	2.41224	2.40021	1.88404
PSMA2	5.63217	5.64122	5.59233	5.70728	5.25463	5.24224	5.43997	5.1082	5.47836	5.37176	5.33165	4.4419	5.49938	5.35949	4.75779	4.61216
MRPL32	4.83382	4.64648	4.52495	4.69087	4.48851	4.56974	4.57027	4.6465	4.31535	4.49764	4.4084	4.09497	4.14856	3.94896	3.4321	3.27179
HECW1	-1.56074	-1.46597	-1.71733	-0.74147	-2.98892	-2.96471	-2.98921	-3.14279	-2.60297	-2.80719	-3.02293	-3.11022	-3.32193	-3.32193	-3.32193	-3.03867
LUARIS	-2.70621	-2.64613	-2.61648	-1.8997	-3.16742	-3.32193	-3.0018	-2.92514	0.60702	-0.33466	0.39307	-0.26583	-3.32193	-3.32193	-3.32193	-3.32193
STK17A	3.45282	3.35273	3.3637	3.75693	3.98528	2.3235	3.93533	2.64683	4.4891	5.13238	4.7367	5.85618	5.24509	5.44789	5.4264	5.83075
COA1	3.0245	2.88966	2.93024	2.82195	3.20592	2.9844	2.89464	2.88033	2.3263	2.4762	2.05688	1.94383	3.19678	3.09547	2.3388	2.54153
BLVRA	4.62099	4.92964	5.04093	5.24095	5.73604	6.09414	5.84769	5.88402	5.08264	5.079	5.15553	4.92423	5.45311	5.65648	5.45564	5.55635
URGCP-MRPS24	8.68507	8.48017	8.22828	7.92194	7.83954	7.84726	7.35307	7.77618	7.89517	7.68573	7.8035	6.73261	7.33109	7.0446	6.82935	6.7968
MRPS24	6.96439	6.75865	6.50936	6.19116	6.0358	6.11274	5.43154	6.03404	6.12598	5.92173	6.03987	4.89891	5.53987	5.25884	4.94409	4.93511
URGCP	3.70436	3.55302	3.52094	3.66399	3.8417	3.35079	3.74967	3.5702	2.94348	2.90468	3.00119	3.16518	2.93068	2.69336	3.56035	3.30723
UBE2D4	2.75903	2.49225	2.29259	2.26251	2.12177	2.16316	2.03984	1.95615	1.96684	1.21609	1.6879	1.74964	2.47436	1.66558	2.03334	1.70745
POLR2J4	2.48217	2.282	2.1895	1.89025	1.87952	1.97958	1.53655	1.77709	1.67126	1.10569	1.36565	1.08225	1.91511	1.23213	1.29355	1.26901
SPDYE1	0.98194	0.32305	0.53975	0.40115	-0.07987	0.99702	-0.20796	1.45862	-2.30638	-0.93294	-0.69906	-0.54946	-0.38525	-0.15119	-0.37454	0.02138
RASA4CP	-0.20855	1.4323	1.55274	1.35836	1.1826	2.63544	0.66221	2.03762	0.82223	2.08176	0.88315	1.42001	-1.32258	-1.22221	-0.03368	0.41407
LINC00957	-0.19221	1.94052	1.91604	1.73381	1.03643	2.64492	0.62977	2.04612	1.24459	2.50216	1.16456	1.91637	-0.41053	-1.05574	0.66025	0.87755
DBNL	3.52601	3.80918	3.76163	3.59636	3.79628	3.46157	3.43753	3.32205	4.4099	4.15533	4.52154	3.77079	3.58529	3.50511	4.0517	3.84588
PGAM2	-0.44627	2.09341	1.86418	2.48224	2.31751	2.25394	1.35135	2.36811	1.50432	2.34419	2.58748	2.36886	0.66897	2.25277	2.11662	2.23131
POLM	4.1548	4.56847	4.34233	3.88283	2.45928	2.85157	1.55663	3.26067	2.62099	2.92076	3.07591	2.5498	1.31323	1.70471	1.28009	2.13891
MIR6838	6.94776	7.08146	6.8252	6.05712	4.85509	5.29777	2.97492	5.85288	5.15141	4.59431	5.11093	4.85822	3.45388	2.7206	3.00707	4.34959
AEBP1	-1.51204	-0.59383	-0.64804	0.12257	-2.18612	-1.9525	-2.79724	-2.86501	-1.05189	0.17259	0.05814	-0.38045	-2.63168	-1.94922	-2.51644	-1.60261
POLD2	6.33591	5.88418	5.76285	5.15724	6.17031	6.07333	5.35891	5.95264	6.79527	6.18092	6.60307	5.27389	6.2283	5.70488	6.63849	6.21333
YKT6	6.3495	6.39944	6.29381	6.08746	5.97486	5.78109	5.78434	5.63316	5.80734	5.59249	5.79819	5.45824	6.00414	6.05398	6.4218	6.41237
CAMK2B	-2.95974	-2.80887	-3.02868	-3.01139	-3.26479	-3.18945	-3.32193	-2.57445	-2.90865	-2.7773	-2.4833	-2.97649	0.49734	-0.04571	0.60964	0.52279
NUDCD3	3.34392	3.55092	3.36222	3.31091	3.12343	2.57868	2.95967	2.67977	3.91603	3.89447	4.11505	3.75269	3.68733	4.35286	4.4767	4.60985

DDX56	5.44055	5.16436	4.9038	5.07048	5.26584	5.15588	5.03784	5.35806	4.68197	4.47339	4.90705	4.47025	5.48465	5.66075	5.58297	5.44106
TMED4	5.30428	5.17801	5.11863	5.07409	5.85418	5.56946	5.33037	5.42252	4.90091	4.92514	4.48767	4.9175	5.37266	4.81016	4.88899	4.947
OGDH	4.64428	4.64846	4.66621	4.79608	4.89774	4.48293	4.7138	4.42414	5.62128	5.3133	5.60297	5.22155	4.89279	5.0933	5.28589	5.60708
ZMIZ2	5.65312	5.46895	5.73972	5.09127	3.43575	3.05144	2.58334	3.27945	5.42172	5.24385	5.46188	5.28682	4.23196	3.54218	4.61196	4.16704
PURB	3.74866	3.77827	3.88468	3.48899	3.46611	2.35574	3.92277	2.22353	2.96334	1.88998	3.28607	2.61348	3.63182	2.68555	4.16681	2.99101
MIR4657	5.50766	5.80692	5.96707	5.74713	5.074	2.59033	6.09689	3.13894	4.67987	4.09129	4.19409	2.81581	5.44939	5.15127	5.73413	4.84201
MYO1G	0.38062	1.70982	2.24277	0.98522	-2.81703	-1.92557	-3.09857	-2.35097	-3.32193	-3.09175	-3.32193	-3.20419	-2.88753	-3.22429	-3.32193	-3.0892
SNHG15	3.76471	3.44579	3.47806	2.98019	2.99988	3.46624	2.6086	3.62063	1.71851	1.81004	2.12262	0.51754	2.62496	2.7785	0.9627	1.08028
SNORA9	3.87664	3.64899	3.06973	2.39416	2.96031	1.86396	2.60687	2.09237	0.54259	1.54279	1.33232	2.62883	1.50742	2.35359	1.52893	2.03078
CCM2	3.15181	3.3546	3.43631	3.19556	3.3857	3.32053	3.06736	3.33007	3.06268	2.79671	2.66426	2.65196	3.84012	3.97722	3.925	4.03327
NACAD	-0.97031	0.47851	0.49468	0.07924	-2.83421	-2.60967	-3.12364	-2.86199	-1.72825	-0.69547	-1.28391	-0.82656	-1.52567	-1.09032	-0.47819	0.31017
TBRG4	5.02917	4.87714	4.60918	4.60857	5.25174	5.29553	4.81518	5.39399	4.75957	4.79782	4.72759	4.3141	4.98026	4.92445	4.87237	4.74296
SNORA5A	2.9316	3.09612	2.48271	2.16577	3.84471	2.70966	3.58426	2.80509	2.26682	2.5072	1.88754	2.48304	1.8992	2.46615	3.24804	1.23931
SNORA5C	3.94447	2.54169	3.24735	2.70851	3.95405	2.39371	3.61618	3.14724	1.05344	2.3267	3.1543	1.16877	3.09298	2.84315	2.46173	2.8653
GTF2IP13	1.606	1.52274	1.38358	0.64163	-0.40758	0.07968	-1.21929	0.74174	-0.21845	-0.03459	-0.40824	-0.48944	-0.93337	-0.51868	-0.22954	0.06413
IGFBP1	3.45233	3.70753	3.81373	3.12011	6.22494	10.3461	6.21102	9.49711	3.86418	3.02038	2.31149	2.37134	3.75767	0.17156	0.95452	1.53354
IGFBP3	1.1631	3.16071	4.30694	4.13498	2.17416	4.21372	1.72313	3.05299	7.02064	6.72436	6.1658	6.17864	6.55865	5.29623	5.65682	5.59792
TNS3	3.34005	3.49567	3.73963	3.21371	4.03742	1.56573	3.87205	1.86892	4.714	5.04857	5.27206	5.33704	5.88129	5.33355	6.6469	5.83164
C7orf65	-2.47405	-3.32193	-2.14339	-2.57936	-3.16717	-3.32193	-3.32193	-3.32193	-1.52316	-1.20348	-1.52045	-0.6648	-0.41983	-1.07511	-1.49593	-0.81521
HUS1	2.23513	2.28565	2.22612	2.26746	2.69163	2.35502	3.07173	2.42219	2.17182	1.91574	1.61555	2.23186	2.81033	2.91476	2.31572	2.34664
LINC00525	-0.53958	0.07485	0.24315	0.99774	-0.80672	-2.10714	-0.76647	-1.28343	-0.66301	-0.72743	-0.36443	0.66574	0.03036	0.45941	-0.89803	-0.31961
PKD1L1	-3.32193	-2.77927	-2.85141	-2.72393	-2.89369	-3.00368	-2.93918	-3.06665	-0.60376	0.10163	0.42131	0.60347	-2.30759	-2.79121	-2.93153	-2.92398
C7orf69	-3.32193	-3.32193	-3.32193	-3.32193	-3.32193	-3.32193	-3.32193	-2.22264	3.63011	4.92545	4.72081	5.43806	-3.32193	-2.00019	-2.38302	-2.36783
SUN3	3.89169	3.94391	4.08142	3.97389	-2.10024	-1.83019	-2.44492	-1.44815	0.25557	-0.33757	-0.32859	-0.14063	3.88501	3.03836	2.5336	2.23007
UPP1	6.2979	5.92769	5.96248	5.21962	4.5073	4.89116	4.52379	4.41942	4.31675	4.20143	3.83552	4.22116	5.26101	5.2403	5.16312	5.3224
ABCA13	-1.07598	-1.19112	-1.5118	-1.6399	-3.1796	-3.20785	-3.32193	-3.25374	-2.29299	-3.21294	-2.85792	-3.16395	-3.23276	-3.32193	-3.32193	-3.21168
FIGNL1	2.05479	1.87373	2.09239	2.12513	2.80631	1.08847	3.22109	1.47369	3.5006	2.77916	2.98661	2.73289	3.39558	3.29818	3.1566	2.94448
DDC	-3.0029	-3.32193	-3.32193	-2.92969	3.57334	0.7237	3.42313	1.05119	-3.32193	-3.32193	-3.32193	-3.32193	-2.66048	-2.85059	-2.76358	-2.34662
DDC-AS1	-3.32193	-3.32193	-3.32193	-3.32193	1.48087	-1.00677	1.04125	-1.27975	-3.32193	-3.32193	-3.32193	-3.32193	-3.32193	-3.32193	-3.32193	-3.32193
GRB10	3.15445	3.77475	4.13858	3.95787	2.99505	1.03503	3.56964	0.35499	1.88107	1.82967	2.06737	2.4264	2.91601	3.1124	4.0536	4.02616
COBL	-3.17374	-3.1566	-2.85258	-3.13725	2.59357	0.72997	2.69751	0.66549	-3.32193	-3.32193	-3.32193	-3.32193	-3.32193	-3.32193	-3.32193	-3.32193
LINC01446	0.45394	0.3787	0.27316	-0.1273	0.86545	-0.06509	1.03888	-0.4424	-1.28377	-1.48287	-0.77442	-1.41682	-3.32193	-3.32193	-3.32193	-3.32193
RAC1P9	2.2184	1.46817	1.42234	0.67776	2.07734	0.58619	2.44878	0.80607	-0.33933	0.0767	0.79978	0.05446	-3.32193	-3.32193	-3.32193	-3.32193
HAUS6P3	0.44571	0.06254	-0.55337	1.03362	-0.194	-0.18483	-0.17572	-0.5485	-0.97862	-1.19088	-3.32193	-0.66266	-1.64519	-1.64872	-0.50955	-0.96999
SEC61G	6.90606	7.29997	7.38522	7.11875	6.7614	7.4362	6.93751	7.19414	6.54381	6.82188	6.31837	6.74347	6.41778	6.54303	5.17731	5.13304
EGFR	4.21021	4.36138	4.17902	3.99864	5.00728	2.94994	5.35351	3.17445	4.78402	4.97721	4.92427	5.47823	5.37397	4.78072	5.63601	5.14525
EGFR-AS1	1.83083	1.38661	1.47873	1.34721	3.226	0.38131	3.52826	-0.11645	2.85545	2.94013	2.66594	2.91083	2.9661	2.35313	3.32615	2.85854
LANCL2	2.55631	2.56254	2.43882	2.72533	2.60894	1.94951	2.73244	1.97325	1.89486	1.41864	1.97805	1.75477	2.87956	2.7559	3.01494	2.88026
VOPP1	5.48387	5.50855	5.42532	5.26864	4.76683	4.18094	4.74133	3.91548	5.27794	4.64335	4.77742	4.45799	5.732	5.55207	5.76455	5.64628
FKBP9P1	0.46396	0.08035	0.61906	-0.85377	0.72573	1.14432	0.48432	0.28831	0.46291	0.59148	-0.28494	0.94847	-1.20395	-1.15067	-1.32193	-0.53248
PSPHP1	4.79467	5.29095	5.35636	5.78876	5.82795	6.56234	6.55761	6.48515	4.88165	4.88923	4.45757	4.55445	-3.32193	-3.32193	-1.38116	-3.32193
ZNF713	-1.85462	-1.88999	-1.76526	-1.43219	-0.8491	-0.83934	-1.03399	-0.90274	-1.71235	-2.02392	-1.57738	-1.46219	-0.63657	-1.07706	0.15201	0.18075
NIPSNAP2	2.77474	3.08245	2.98964	3.11809	3.38189	2.76409	3.61058	2.74407	4.22729	4.04866	3.74907	4.3948	4.44784	4.18058	4.17508	3.7989
MRPS17	4.16722	3.8544	3.61327	3.45831	4.41631	4.47239	4.34582	4.49216	3.90854	3.86346	3.85545	3.50843	4.81939	4.78519	3.81443	3.53685
PSPH	4.24665	3.98534	4.00715	3.67801	5.70313	5.07025	5.60906	5.29539	3.37693	2.75247	2.71422	2.81271	4.35651	4.33423	3.62768	3.56517
SUMF2	5.14395	4.92556	5.27961	5.36181	6.22174	6.27291	5.86256	6.08102	5.68181	5.69744	5.8062	5.33676	4.96238	4.52721	4.9857	4.93679
PHKG1	3.41109	2.96849	3.46632	3.57871	3.71634	4.87175	3.53463	4.8585	3.24895	3.13273	3.09945	3.40902	3.47342	2.98353	2.76135	2.68581
NUPR2	1.48693	2.23966	1.65522	1.43282	2.67499	2.77969	2.42929	2.52714	0.92241	1.50107	0.51939	1.81412	-3.32193	-3.32193	-3.32193	-3.32193
TNRC18P3	-1.87277	-2.12352	-0.62882	-2.87399	-2.42862	-1.2489	-3.32193	-1.41722	-0.99341	-0.33045	-0.29147	-0.97109	-3.32193	-2.78558	-1.77224	-2.05453
ZNF716	-3.32193	-3.32193	-2.88486	-3.32193	0.06356	-1.31478	0.34592	-0.8318	-3.32193	-3.32193	-3.32193	-3.1309	-3.32193	-3.32193	-3.32193	-3.12565

GABPAP	-0.45483	-1.77238	-1.42959	-1.1163	-0.40292	-1.86981	0.41013	-3.32193	-0.05159	-0.49998	-0.65827	0.74289	-0.14063	-0.5546	-1.05133	-1.02626
ZNF727	0.70083	0.42782	0.25735	0.6405	-0.80801	-2.7687	0.38754	-2.2518	-3.32193	-3.32193	-3.32193	-3.32193	1.51211	1.77392	1.61209	1.92561
ZNF736	1.02747	0.78259	0.61969	0.52937	0.88333	0.94596	0.99575	0.73965	-3.32193	-3.32193	-3.32193	-3.32193	2.01275	2.42938	2.23485	2.52175
TRIM60P18	0.59074	0.65344	0.62634	0.81281	-0.52048	1.88805	0.81755	2.00076	-3.32193	-3.32193	-3.32193	-3.32193	-0.13959	1.88184	0.84829	0.80446
HNRNPCP7	-1.88088	-3.32193	-2.31043	-2.26353	-0.55056	0.29539	-0.4336	1.79331	-1.32536	-2.45247	-3.32193	-1.92907	0.14769	0.59061	0.11443	0.24849
ZNF680	0.666	0.61797	1.00678	1.53922	1.46842	1.4984	2.24974	1.88854	0.28017	0.41426	-0.09725	0.43434	2.52611	2.92294	2.36943	2.18929
ZNF107	1.181	0.52401	0.7029	0.52673	0.84292	0.97279	1.30866	1.44176	1.31803	0.57513	0.79826	1.26374	2.33857	2.02714	2.21475	2.1423
BNIP3P11	1.11625	0.96845	0.92335	-0.04374	-0.42658	0.43768	0.00109	0.11609	1.57737	0.51534	0.96828	0.35182	1.308	1.82524	1.51801	1.33428
ZNF138	2.11137	1.75298	1.64741	2.33641	1.60297	2.37678	2.04136	2.63733	1.19163	1.47432	1.11063	1.87288	2.61769	2.58734	2.31847	2.07695
ZNF273	-0.04375	-0.38738	-0.12294	-0.18217	-0.95661	-0.97671	-0.71129	-0.65864	-0.23322	-0.46831	-1.17191	-0.35787	1.08198	1.83726	0.8583	1.15523
ZNF117	-0.82466	-0.57696	-0.03319	0.91615	-1.92463	-0.9685	-2.26242	-0.98329	-1.76572	-1.04388	-1.55362	0.45027	-0.39644	0.51207	-0.27271	0.69878
ERV3-1	0.80398	0.94735	1.63941	1.9603	-0.33676	0.52423	-0.6472	0.63516	-0.66081	-0.34517	-1.14702	0.25559	-0.49108	0.42773	0.14329	1.29356
CCT6P3	1.58046	1.40903	1.65504	1.96315	0.58096	1.52734	1.00257	2.26598	-0.7219	-0.41671	-0.56855	0.33765	0.82798	0.86044	-0.32298	0.33259
INTS4P1	0.04842	0.02094	0.21361	0.72735	0.24962	-0.42848	0.54034	-0.74557	0.5938	0.32184	0.87145	0.85963	-0.34903	-0.56905	0.14498	0.1983
ZNF92	3.28975	3.37419	3.14051	4.39009	1.54383	1.20916	2.10015	1.56806	2.03815	1.9922	1.74452	3.12274	2.61877	3.13663	2.96369	3.10382
INTS4P2	0.23431	0.18334	0.30865	0.09836	0.19518	-0.4702	0.43923	0.02068	0.98305	0.48274	1.06427	1.26053	-0.70848	-1.02883	0.21951	0.00045
CCT6P1	2.98326	2.76062	3.17306	3.2637	1.71273	2.27676	1.6886	2.6533	1.39995	1.40842	0.75597	1.76736	2.78076	3.00161	1.81418	2.12537
VKORC1L1	4.52064	3.97216	4.07921	3.84305	4.52593	3.40721	4.96498	3.19546	4.37897	2.88904	4.01031	3.06511	5.1827	3.75194	5.42829	3.80521
GUSB	5.31964	5.29134	5.42663	5.16528	5.3788	5.69812	4.88882	5.74691	4.82862	4.91601	4.85506	4.65234	3.49177	3.84768	3.83044	3.98617
ASL	4.83888	4.85124	4.65013	4.79018	5.19949	6.38597	4.75084	5.98913	4.99135	5.35587	5.21402	4.56145	3.30755	3.74137	3.47463	3.73891
CRCP	4.3798	4.2902	4.0503	3.71545	4.32717	4.21969	4.62159	4.09611	4.10645	3.86611	4.03027	3.7906	4.71475	4.82475	4.43139	4.45297
TPST1	4.45568	4.66878	4.62061	5.02327	3.10174	3.36387	3.20311	3.13973	3.93328	4.08235	3.89592	4.03553	2.89293	3.31238	3.15817	3.0856
LINC00174	0.36671	0.18367	0.55446	0.59952	-1.14791	0.66534	-1.49318	1.21388	-1.56335	-0.97517	-1.09472	-0.9949	-1.85349	-1.14501	-1.18649	-0.6735
RPL35P5	3.08585	2.69083	2.65827	0.96616	0.9788	2.18707	-0.54077	1.22986	1.81289	2.42199	1.65178	0.91814	-0.75389	-0.40546	-0.15027	0.168
KCTD7	0.70368	0.74638	0.97951	0.6802	1.93725	0.7982	1.97475	0.66786	0.7739	0.72527	1.03205	0.89559	2.4534	1.8056	2.34645	1.74149
RABGEF1	4.26569	4.26343	4.42971	4.49156	3.29025	3.62337	3.65632	3.63246	3.65123	3.33053	3.51442	3.82708	4.12087	3.68603	3.61925	3.49209
GTF2IRD1P1	3.11222	3.07687	3.26518	3.72719	1.20463	2.48048	1.61906	2.5268	2.3601	2.02951	2.16909	2.85275	2.89678	2.17338	2.56202	2.09437
LINC02604	1.9595	1.54105	1.83746	2.0308	-0.39409	1.26501	-0.77537	2.01564	-1.69089	-1.15844	-1.30845	-0.98205	-1.3205	-1.09002	-1.39612	-0.72202
TMEM248	5.20224	5.17677	5.13194	5.29514	5.21359	5.00725	5.23177	4.87123	5.07522	4.98208	4.66907	5.34464	5.12817	5.42576	4.84224	5.21704
SBDS	6.93162	6.99035	6.86315	6.92612	5.86819	5.22449	6.67056	5.19414	6.12927	5.65898	5.78785	5.77984	6.68644	5.88283	5.65817	4.85169
TYW1	4.60179	4.62838	4.52384	4.48968	4.63918	3.87029	5.08568	3.87253	4.16834	4.00272	4.22511	4.09145	4.01789	4.06472	4.21252	3.91199
SPDYE21P	1.05019	0.20583	-0.38251	-2.19447	-0.43885	-0.06797	-1.46274	1.73859	-2.25784	-0.44717	-0.1881	-0.01498	-0.88593	-1.34724	-0.64926	-0.11365
PMS2P4	2.81881	2.58906	2.90174	2.7361	2.82081	2.38074	2.6598	2.31525	2.55759	2.88211	2.45559	1.83615	3.43651	3.45416	3.03722	3.48221
STAG3L4	2.47553	2.14373	2.23131	2.74016	2.13204	2.97267	1.86155	3.02617	1.94537	2.01793	1.59714	1.91736	3.31767	3.38462	2.39378	2.55683
AUTS2	-2.94571	-3.32193	-3.19993	-3.07332	-3.15004	-3.02806	-3.13409	-3.32193	-2.98881	-3.32193	-3.32193	-3.32193	-1.20886	-1.08275	-0.22803	-0.33913
CALN1	-2.15982	-2.06531	-1.77361	-1.38372	-0.19737	0.24765	0.07484	-0.5966	-3.32193	-3.32193	-3.32193	-3.32193	-3.32193	-3.32193	-3.32193	-3.21692
TYW1B	0.61289	0.79162	0.26385	1.03896	1.29709	1.58029	1.96437	1.45158	-0.19213	-0.22085	-0.58295	-0.16746	-0.2404	-0.60408	-1.1081	-0.76565
SBDSP1	4.42782	4.20008	4.22134	4.38526	4.29869	3.94145	4.76096	3.52348	3.65804	3.49503	3.38098	3.28097	5.26958	5.05334	4.01151	3.92008
POM121	4.83765	4.67349	4.58608	4.2885	4.51412	4.04039	4.16767	4.05997	4.80232	4.63245	4.70678	4.60929	4.30007	4.20661	4.62258	4.65534
NSUN5P2	4.73572	4.64908	4.66717	4.46245	4.22142	5.02965	3.65062	4.98673	3.26688	3.22326	3.42535	3.74215	3.81732	3.71737	3.61003	3.54286
TRIM74	-0.79873	-0.53074	-0.13195	-1.00105	-0.03814	1.15952	-0.75512	0.56619	-1.29818	-0.49303	-0.40375	-0.32148	-1.52281	-1.9173	-1.99354	-1.70862
STAG3L3	4.15719	3.84995	3.92738	3.90647	3.95883	4.20702	3.70295	4.58581	3.03917	3.06641	3.05829	2.92809	3.8396	3.30155	3.62308	3.75108
PMS2P7	4.37316	4.11385	3.99985	4.02745	4.27935	4.58882	4.38701	4.40895	3.63889	3.53404	3.19015	3.6555	4.41643	4.06159	4.04592	4.49139
SPDYE8P	1.18754	1.62239	1.30937	2.15002	1.22643	2.15998	1.78026	2.6442	0.84851	0.79953	0.45305	1.11396	0.15438	0.69065	0.70207	1.23749
SPDYE11	1.15884	1.43199	1.30607	2.08308	0.97587	1.84702	1.36535	2.32734	0.63334	0.48975	0.47852	0.90711	0.16198	0.60528	0.39703	0.98422
SPDYE9P	1.42226	1.66592	1.6296	2.20789	1.17503	1.95599	1.52454	2.40547	0.85892	0.5567	0.49478	0.99769	0.50591	0.97836	0.6854	1.00085
PMS2P6	4.89884	4.60841	4.56499	4.83427	4.21548	4.7526	3.91642	4.68445	3.67875	3.75351	3.33131	4.11289	4.70786	4.65467	3.97774	4.41469
SPDYE10P	1.9023	2.07802	2.28144	2.69734	1.63999	2.31308	1.87091	2.77791	1.42089	1.05051	0.94902	1.3581	1.16633	1.55582	1.17007	1.3608
GTF2IP4	5.72624	5.82165	5.69472	6.28367	5.98544	6.42311	6.26684	6.51799	5.69399	5.72456	5.49581	6.59989	6.24278	6.56118	6.81842	7.13138
GTF2IRD2P1	-1.54798	-2.16192	-1.47143	-1.03268	-2.23707	-1.84716	-3.05248	-1.96019	-1.84169	-0.81935	-0.84739	0.75272	-1.41215	-1.79134	-1.50618	-1.39941

POM121B	3.81566	3.73011	3.49384	2.68767	3.65212	2.60763	2.70879	2.55837	4.70041	4.73563	4.68393	4.79192	3.31155	3.1006	4.05367	4.04034
NSUN5	5.23218	5.03277	4.84582	4.52006	4.26358	4.84727	3.74928	4.80481	4.37784	4.16898	4.4409	3.82067	4.33173	4.40966	4.4932	4.09538
FZD9	-3.32193	-2.86502	-2.84344	-2.81699	3.49029	3.77185	2.3906	3.85913	-2.85115	-1.14523	-0.78229	-1.64256	-0.06157	0.0467	0.23584	1.30148
BAZ1B	4.72607	4.6063	4.68978	5.12567	5.05037	4.22087	5.30274	4.40143	5.27626	4.56355	4.84363	5.28233	5.63281	5.58648	5.99356	5.91871
BCL7B	4.13093	4.01435	4.0697	3.87827	4.70244	4.70895	4.53301	4.64475	4.33959	4.25516	4.35962	4.15197	4.18806	4.30282	4.15709	3.94582
TBL2	4.25152	4.08306	4.07462	4.11831	4.83497	4.36163	4.59216	4.20238	3.60328	3.36338	3.41305	2.91352	4.18173	3.42356	4.25746	3.75413
MLXIPL	-2.60406	-2.26153	-2.3549	-1.3032	1.98586	3.28027	0.27906	3.29292	-3.32193	-2.76975	-2.58388	-2.77753	-3.15036	-1.9237	-2.9348	-1.95849
VPS37D	-1.56209	0.22835	-0.36107	-0.63866	2.50457	3.49162	1.4716	3.01881	3.26946	4.39208	3.62216	2.51373	2.59591	3.05267	3.65383	4.20574
DNAJC30	3.82001	3.54017	3.56882	3.34957	3.2057	3.66312	2.57528	3.56544	3.281	3.63785	3.65408	3.33312	3.2547	3.02605	3.06348	3.24136
BUD23	4.65808	4.57934	4.34817	4.23437	4.93652	5.60644	4.62672	5.56135	4.61429	4.32784	4.34135	3.53183	4.64421	4.98561	4.59823	5.03361
STX1A	3.17166	3.31614	3.78075	3.65184	1.84395	3.5355	1.76371	3.4058	1.99424	2.92952	2.16922	2.93186	3.58938	3.77269	3.37759	4.27178
ABHD11-AS1	2.57579	2.4232	2.31073	2.18271	0.99428	2.04623	1.12385	2.6079	1.86517	2.43656	2.41208	1.78	1.36731	1.68334	1.35758	1.03207
ABHD11	4.21033	3.88655	3.87199	3.91878	2.83918	3.59269	2.13894	3.65095	3.69468	3.19998	3.61509	3.12871	3.02186	2.30561	2.34634	2.44021
CLDN3	3.17876	3.68011	3.05793	2.67948	5.1014	5.9253	4.37053	5.65911	1.80691	3.59502	1.78441	2.51145	-0.3805	0.89498	1.56072	2.05053
CLDN4	5.29594	5.76164	6.02636	5.36937	0.77598	-0.81784	0.95121	-1.14118	-2.35159	-0.79209	-1.88367	-1.07234	-3.32193	-3.32193	-3.1108	-2.75318
METTL27	1.50961	1.79914	2.03771	1.60153	0.43995	0.93826	-0.16963	1.27804	1.71957	2.37704	2.21498	1.94726	-3.32193	-2.82374	-3.32193	-1.7073
ELN	-1.67325	-1.1642	-0.82669	2.55238	-2.83333	-3.15849	-3.32193	-3.13149	-2.56534	-0.84269	-1.81426	-2.48417	-3.19371	-2.5221	-2.8999	-2.21241
ELN-AS1	1.52199	2.46037	2.6604	4.22504	-2.15896	-3.32193	-3.32193	-3.32193	-0.07724	2.68494	1.33232	0.56852	-3.32193	-0.35234	-0.94937	0.30909
LIMK1	5.41488	5.09498	5.55358	4.8812	2.97989	3.66515	2.43034	3.46823	5.41385	5.01029	5.85148	4.98042	3.67148	3.29017	4.90792	4.45459
LAT2	0.93365	0.99819	1.09984	0.12649	-1.38517	-0.69441	-1.77953	-0.30244	4.33394	3.40453	4.58598	3.28034	0.8895	-0.35873	0.61798	0.72227
RFC2	5.55558	5.38081	5.30011	5.026	4.74099	4.18028	4.78441	4.2647	5.39244	4.41895	4.67347	4.08505	5.77968	5.64622	5.61858	5.29351
CLIP2	3.79077	4.36505	4.27479	3.79101	4.93363	3.50092	4.45535	3.47959	4.45069	5.38833	4.8627	5.08345	2.40918	3.09282	4.50847	4.44363
GTF2IRD1	3.35789	3.12655	3.49915	3.27869	1.68692	1.93793	1.03638	1.85725	2.79007	2.46892	3.03423	2.56282	2.09536	2.20272	2.95665	2.64214
GTF2I	4.51054	4.54439	4.50263	4.92956	5.04131	5.12299	5.32474	5.22237	4.80194	4.73494	4.73321	5.43614	5.32747	5.43928	5.76951	5.96063
GTF2IRD2	-0.21624	-0.58762	0.03661	0.40055	-0.93362	-0.59656	-1.64058	-0.39529	-0.94825	-0.17917	-0.47848	0.68769	0.18781	-0.26821	-0.24126	-0.06975
STAG3L2	3.99504	3.76383	3.67385	3.55758	3.8323	3.74227	3.50686	4.29006	3.14405	3.05712	2.7813	2.92281	3.25312	2.82665	3.08563	3.37317
SPDYE12P	1.5942	1.60295	1.73118	1.59589	1.81566	2.53596	1.9125	3.02183	1.21608	0.44563	1.12805	1.36802	0.93648	0.44076	1.14692	1.23842
CASTOR2	2.01043	2.06708	2.38598	1.82799	3.8689	2.93395	3.67245	2.90348	2.11636	2.88809	2.28631	3.42415	1.47332	3.01814	3.80916	4.30936
RCC1L	4.81081	4.7306	4.51111	4.39221	5.32782	5.48049	4.88702	5.32777	4.76613	4.56231	4.78441	3.91238	4.51964	4.52178	4.68803	4.61281
GTF2IRD2B	0.84538	0.88818	1.25444	1.07298	0.61863	0.94576	0.046	1.08278	1.13715	1.36772	1.14541	1.63063	1.4413	1.31152	1.70555	1.63434
GTF2IP1	4.77249	4.82708	4.74004	5.2912	5.08727	5.52374	5.34945	5.58821	4.81421	4.78262	4.60846	5.61022	5.30483	5.56048	5.8144	6.09678
SPDYE13P	1.49735	1.60758	1.83055	2.01039	1.00006	2.17794	1.1883	2.63947	0.93265	-0.08303	0.27296	0.73761	-0.65889	0.76077	0.96875	0.69472
PMS2P10	2.74298	2.21875	2.42183	3.07466	2.17521	3.16145	2.37579	2.84586	2.92937	2.69132	2.52334	2.79756	1.75643	2.8135	1.85251	2.33636
SPDYE14P	1.84539	1.75926	2.08366	2.31816	1.45765	2.54296	1.5019	3.28659	1.26586	0.45908	0.81787	1.33155	0.59018	0.72058	0.99499	1.47095
SPDYE15P	1.7418	1.47681	2.05177	2.14125	1.43875	2.53546	1.49045	3.14301	1.19117	0.41684	0.68136	1.08647	0.38298	0.70753	0.9818	1.33131
PMS2P2	3.07475	2.70263	2.76218	2.79948	2.81262	3.28833	2.94084	3.32226	3.27756	3.14698	2.76555	2.98682	2.36931	2.36427	2.25221	2.4161
STAG3L1	4.23115	4.02158	4.06162	4.09463	4.12229	4.47556	3.72381	4.88716	3.2001	3.44068	3.34479	3.31808	3.51405	3.04095	3.2712	3.43378
TRIM73	-0.37339	-0.02926	0.33453	-1.14097	0.58227	1.26733	-0.07493	0.9987	-0.57879	-0.29429	0.11924	-0.57634	-0.7526	-0.58149	-1.34356	-1.1794
NSUN5P1	2.78498	2.71137	3.0987	2.5929	3.2142	3.72427	2.17059	3.39166	1.37198	1.80696	2.23327	2.02943	1.82986	2.24378	1.60175	1.70359
POM121C	4.78457	4.64618	4.48999	4.29865	4.50368	3.71505	4.08877	3.78318	4.85924	4.68809	4.80203	4.72714	3.9984	3.92732	4.49337	4.59402
SPDYE5	-0.32146	-0.17466	0.20269	0.01931	-0.21201	0.78946	-0.40803	1.61855	-1.05936	-0.69503	-1.35783	0.1025	-1.10242	-0.51187	-1.73422	-0.57892
PMS2P3	2.75514	2.63306	2.76733	2.06515	2.21492	2.65067	1.96989	2.445	2.02503	2.40608	1.91757	1.66332	2.26838	2.20964	1.57277	1.86881
HIP1	1.60758	1.8413	1.42105	2.08665	2.26017	1.25027	1.78661	1.30538	3.32989	3.58641	2.30201	4.29409	1.21039	1.64383	2.08348	2.37883
RHBDD2	4.73945	4.75722	4.91093	5.11516	5.19058	5.93271	4.70755	5.63566	4.89791	4.7931	4.76769	5.01711	4.26718	5.08872	5.08394	5.56356
POR	4.33639	4.38899	4.29367	4.06519	5.9382	6.66408	5.27447	6.77929	4.20875	3.91297	4.3316	3.84085	4.00691	3.58678	4.45913	4.31035
TMEM120A	3.48238	3.79992	4.02337	4.0303	5.96827	6.57932	5.45692	6.52496	4.57389	4.91976	4.6311	4.48953	4.32503	4.73588	5.01547	5.08772
STYXL1	3.82224	3.77333	3.98485	4.0551	3.21715	3.88033	3.1975	3.76757	2.89527	3.70634	3.28301	3.82544	3.98008	3.65053	3.66326	3.84463
MDH2	6.51845	6.35707	6.44626	6.12117	6.54116	6.7725	6.02027	6.53437	7.03448	6.60718	6.94779	5.87335	6.05741	5.81363	5.89052	5.81255
GTF2IP7	0.95553	1.22259	0.80175	0.85829	3.57218	3.02651	4.39893	3.2419	-2.88846	-2.95371	-3.32193	-2.66962	-1.06505	-0.8698	-2.66588	0.31911
SRRM3	-1.56858	-1.17719	-2.09676	-2.22402	0.70052	0.60003	-0.16723	0.5157	1.87586	2.21098	1.71453	1.17103	-1.22658	-1.32295	-0.34006	-0.01071

SSC4D	0.73926	1.34553	0.92141	1.03305	0.71674	1.42697	-0.11657	1.5602	0.59271	1.04587	0.43822	0.37918	-0.69512	-0.55577	-1.01657	-0.71199
ZP3	3.22629	3.33728	2.96695	2.87083	1.77625	3.44319	0.69636	3.30578	2.82278	2.50239	2.1922	2.20914	-0.55202	-0.9328	-1.13709	-0.94816
DTX2	4.59911	4.71278	4.3822	4.34132	2.50168	3.11954	2.29252	2.95135	4.77158	4.19713	4.61176	4.00185	2.12332	1.71561	2.46546	2.52685
FDPSP2	0.39104	1.39231	0.79918	0.49645	-0.53961	-0.03679	0.09651	-0.479	0.14499	-0.54784	-1.1249	0.22196	-0.63872	-1.19192	0.50766	-0.53314
UPK3B	0.30792	0.35497	0.46361	1.23073	-1.37703	0.32253	-2.97443	0.05243	-0.30008	-0.08303	-0.22491	0.0572	-2.56853	-3.02589	-3.32193	-2.43113
SPDYE16	-0.97604	-0.72117	-0.97876	-1.42922	-2.0775	-0.84592	-2.83521	-0.97418	-2.7373	-2.15614	-2.40307	-1.92684	-2.90318	-2.3171	-2.45733	-2.01846
POMZP3	5.00768	5.04879	4.76687	4.85292	4.20276	4.78399	3.54853	4.41148	5.1052	4.3282	4.81228	4.3436	3.04185	2.67431	2.89266	3.06379
DTX2P1-UPK3BF	3.2268	3.15255	3.09173	2.84345	1.58947	2.12785	1.47564	2.09265	2.70647	2.38943	2.66025	2.01129	1.19537	1.01324	1.37461	1.5074
FDPSP7	0.43489	0.80981	0.88343	0.28154	-0.52659	-0.24381	0.08717	-0.0353	-0.06505	-0.74408	-0.84246	0.5123	-0.83263	-1.87595	-0.44864	-0.91281
DTX2P1	4.04828	4.06093	3.77293	3.19531	2.13731	2.68775	1.64004	2.48113	3.68496	3.22616	3.7167	2.1604	1.7234	0.8234	1.84559	1.7427
UPK3BP1	3.05899	2.68534	2.80003	2.85243	0.5459	-0.19952	0.46569	1.58581	0.21837	1.00824	2.07277	0.4864	0.72503	0.36772	-0.8977	-2.00695
PMS2P11	3.3752	3.15417	3.27968	3.32195	1.77849	2.24736	1.08366	1.9256	2.06568	2.37435	1.60478	1.34224	1.71276	1.75365	0.33554	2.11182
SPDYE17	1.98759	2.03744	1.94778	2.16336	1.39278	2.53915	1.21448	3.19818	1.19477	0.52422	0.62065	1.44946	0.50602	0.72033	0.99474	1.58586
PMS2P9	3.06845	3.009	3.50438	3.54907	1.34037	1.25434	1.1441	2.01559	1.91785	1.65616	1.61648	1.16542	1.2285	1.36207	-0.5659	2.0765
SPDYE18	1.5033	1.37235	1.62171	0.49197	-0.45828	1.18098	-0.77849	1.02444	-0.70016	-0.5773	-0.08575	-1.39483	-1.36016	-0.69125	-1.38727	-0.28398
FAM185BP	1.8354	2.12376	2.45776	2.24871	1.02399	0.70872	0.50118	-0.19501	0.79738	0.43757	0.29033	0.17325	-0.0752	-0.36411	0.42428	0.56086
CCDC146	-1.96172	-1.34675	-0.90716	0.43619	-3.12292	-2.1633	-2.5993	-2.62473	-0.87489	-0.28271	-1.17545	0.16799	-2.35479	-2.08353	-2.58639	-2.19131
GSAP	0.86069	0.76288	1.02922	1.46826	-1.14702	-0.3424	-0.99299	-0.27201	-0.18154	0.09019	-0.07615	1.06097	0.03627	-0.58767	-0.69282	-0.66632
PTPN12	5.31399	5.59854	5.48189	5.99932	3.9921	3.74088	4.82593	3.78795	5.09061	5.15972	5.06589	5.91454	5.44101	5.28035	4.98427	5.11797
APTR	1.99299	2.0519	1.53013	2.21761	1.68025	2.33954	1.39173	2.17223	0.12358	0.71818	-0.27543	-0.10362	1.85236	1.87067	0.42543	1.12215
RSBN1L	3.69359	3.69358	3.57789	3.89262	2.36329	3.06817	2.78581	3.53653	2.89457	2.74086	2.92559	3.39292	2.53285	2.62538	2.64132	2.8214
TMEM60	4.19441	4.5346	4.70464	4.99102	4.17627	5.17908	4.35723	5.36626	4.05994	4.20082	3.70796	3.60678	5.13861	5.44333	4.40452	4.5128
PHTF2	1.15541	1.21201	0.90767	1.29028	3.21061	2.11418	3.28209	2.54963	2.95967	2.68429	1.7934	3.07281	3.95954	4.10713	2.98013	3.56753
MAGI2	-2.21293	-2.56428	-1.94121	-1.93461	-2.72521	-2.34325	-2.64618	-2.24292	-2.00702	-1.54743	-2.1523	-1.59498	-1.92182	-1.32275	-1.47043	-1.22061
MAGI2-AS3	1.42783	1.72889	1.45512	2.29398	-2.03145	-2.69898	-2.11484	-2.6124	0.9085	1.03611	0.06698	1.46705	2.7711	3.08415	1.65392	2.20021
GNAI1	2.87187	3.09248	2.99757	3.51527	1.46112	-0.65519	2.0184	-0.53794	3.84648	3.78772	3.69937	4.00055	3.90399	3.15071	3.7316	3.36607
SEMA3C	3.3788	4.3577	3.93386	4.82507	3.08271	-0.03452	4.25998	0.54096	4.57666	5.82974	4.99427	6.70672	5.5946	6.13654	6.02006	5.63384
HGF	-3.32193	-3.32193	-3.32193	-3.17121	0.19038	-0.70944	0.40209	-0.69249	-3.32193	-3.32193	-3.32193	-3.32193	-3.32193	-3.32193	-3.32193	-3.32193
CACNA2D1	-0.93764	-1.09727	-0.61909	-0.54117	0.98314	0.47403	1.95871	0.33084	0.70657	0.61231	0.96138	1.67209	-3.32193	-3.32193	-3.32193	-3.32193
PCLO	-0.50638	-0.22113	-0.98274	-0.42118	-0.0802	-1.09823	0.05755	-0.70709	-0.83275	-1.18347	-0.87192	0.1548	-3.32193	-3.28421	-3.27593	-3.27493
SEMA3E	-2.66651	-2.99058	-2.69428	-2.66129	-3.18745	-2.77088	-3.32193	-2.97302	-0.62566	-0.8216	-0.76487	0.58075	3.62485	4.15062	3.78705	3.84342
SEMA3A	0.31376	0.31429	-0.17631	0.77411	-3.0882	-3.32193	-3.02101	-3.18638	2.70085	3.77452	2.43815	3.91612	4.46231	4.72146	4.99922	5.34686
SEMA3D	-3.18933	-3.32193	-3.0255	-3.15646	-2.59582	-1.00268	-3.19778	-0.96519	1.99787	2.577	2.10386	2.46493	-1.3711	-0.1942	-0.63871	-0.76485
KIAA1324L	-1.87751	-1.82373	-1.61434	-1.19079	1.97317	1.4592	2.37039	1.32207	-3.16985	-3.32193	-3.32193	-3.32193	-3.21769	-3.32193	-3.32193	-3.32193
DMTF1	1.86223	1.9812	2.20509	2.52866	1.7821	2.40366	2.20179	2.48778	1.34645	1.58917	1.63475	2.37036	2.44011	2.41953	1.79403	1.98862
TMEM243	3.5222	3.3075	3.34133	3.35392	2.59586	2.73839	2.5614	2.51503	2.6754	2.72702	2.61981	2.48823	2.99331	2.05534	2.26663	1.5369
TP53TG1	0.823	-0.07167	-0.28797	-0.20723	2.44591	3.58371	1.55223	3.53108	2.45398	2.81763	3.10234	1.73832	2.36516	2.04519	1.92377	2.0349
CROT	1.40099	1.27915	1.01479	1.60962	2.43194	1.85383	2.79466	2.3678	2.23332	1.8846	1.71421	2.27164	2.96454	2.94129	2.32088	2.78651
ABCB1	-2.83544	-3.32193	-3.32193	-3.32193	1.86722	-0.26909	2.31942	-0.38124	-2.15648	-2.85026	-2.82068	-2.73035	4.55368	6.19024	5.05284	4.72096
SLC25A40	1.51071	1.52479	1.59147	1.91956	2.10126	1.13776	2.89589	1.57954	2.13058	1.60688	1.89672	2.13774	2.64407	2.99457	2.43523	1.99658
DBF4	3.33022	2.99787	2.99362	3.08776	2.7723	2.31113	3.37283	2.61888	3.40754	2.0186	2.54515	2.4579	3.93835	3.36167	3.45699	2.72853
ADAM22	-0.18025	-0.45719	-0.12967	-0.22605	0.57681	-0.07536	0.8179	-0.30166	1.05276	0.75089	0.66859	1.59126	-2.36066	-2.2566	-2.34235	-2.37236
SRI	3.70402	3.75762	3.90356	3.65917	4.26061	4.67823	4.3884	4.52483	5.56583	5.56638	5.4875	5.46848	4.25876	4.18498	3.70119	3.83545
STEAP1	4.87665	5.01188	4.74457	5.46909	4.80585	5.07717	5.14424	5.09528	3.95213	3.59939	3.62304	3.8213	-1.32431	-2.00499	-2.38684	-3.32193
STEAP2	1.81182	1.76493	1.39045	2.65925	1.69398	1.8634	2.30637	1.96054	0.5725	0.39051	0.10824	1.6111	-3.13348	-3.04864	-3.32193	-3.32193
CFAP69	-0.02443	0.12607	0.0365	0.62976	-1.26051	-0.98976	-1.05738	-0.60922	-1.10397	-0.73807	-0.79819	0.02543	-2.18602	-1.7747	-2.12199	-1.52026
GTPBP10	1.97454	2.12889	1.71011	2.01212	0.88245	0.96329	1.31501	1.03283	1.75171	1.84786	1.35481	2.50326	1.76703	1.96445	1.37025	1.55823
CLDN12	4.25529	4.46646	4.22958	4.67197	3.34737	3.34946	3.90439	3.04405	3.13511	3.06545	3.1503	3.61833	3.64622	3.719	3.10571	3.2314
CDK14	2.42335	2.41855	2.26028	2.69131	1.4314	0.16465	1.25547	-0.11539	-2.71077	-2.59387	-2.766	-2.42631	1.68295	1.96653	2.00759	1.91673
FZD1	0.76984	1.50581	1.38351	1.35753	2.25041	0.85838	1.804	0.90806	0.78199	0.33437	0.2026	0.69742	1.98539	2.51846	3.19979	3.20061

MTERF1	2.11694	2.12943	1.76154	2.49314	1.28631	1.2886	2.06149	1.54241	1.17535	1.17504	1.2241	1.565	1.6707	1.89927	0.8212	1.3401
AKAP9	2.676	2.91524	2.79112	4.32092	2.46973	2.9588	3.19076	3.38565	2.10966	2.47134	2.15634	3.48322	2.62925	2.6195	3.1554	3.48982
CYP51A1	3.9799	4.13851	3.77948	4.18469	6.10059	6.28706	6.50194	6.22597	5.24409	4.69034	4.52187	4.72023	6.42362	6.79109	6.32295	6.25649
LRRD1	3.08306	3.25323	2.86252	3.32384	5.17858	5.42352	5.60787	5.36214	4.29675	3.71378	3.58421	3.80216	5.54015	5.91936	5.41743	5.32583
CYP51A1-AS1	-1.81742	-1.04998	-1.51968	-2.32625	-0.09536	-0.85951	0.40599	-0.81534	-1.00982	-0.91521	-1.32521	-1.42004	0.37031	1.23501	0.74737	0.86084
KRIT1	2.70712	2.64711	2.71412	3.28296	2.86037	2.58661	3.01358	2.60444	2.5718	2.64995	2.6571	3.11676	3.53802	3.44896	3.01836	3.21609
ANKIB1	3.64618	3.82401	4.06487	4.41393	3.85299	2.91876	4.36275	2.83133	3.76423	3.75534	3.77723	4.74282	4.1255	4.05107	4.40651	4.43792
TMBIM7P	0.59431	0.63599	0.49471	0.15066	2.22134	0.60914	1.29119	-0.44526	0.64291	1.20442	0.12003	-0.16063	0.84914	1.08579	1.25807	1.22088
GATAD1	3.28614	3.4983	3.55924	4.19866	3.46187	3.57401	3.64729	3.72691	2.24821	2.6341	2.00968	3.03713	3.05285	3.28897	2.98942	2.86022
PEX1	1.92155	1.84641	1.53012	2.21529	2.37452	2.09728	2.63251	1.97604	1.59973	1.94742	1.38386	2.42945	1.96194	1.62633	1.90463	1.97484
RBM48	2.48783	2.43914	2.41072	3.06746	0.62883	1.21246	1.67607	1.5773	1.0488	1.13589	1.11681	1.83389	2.05338	2.00899	1.38922	1.57089
FAM133B	3.82672	3.76245	3.7631	4.27153	2.79046	3.46222	3.2725	3.92625	2.74659	3.07946	2.87296	3.2276	3.41056	3.57778	3.01577	2.92003
CDK6	3.51016	3.2779	3.29901	3.23668	3.03102	1.54095	3.84505	1.55515	4.58274	4.12153	4.46225	5.57181	5.87154	5.35472	6.03202	5.48867
SAMD9	1.24837	1.60194	1.5901	2.2508	0.90638	-0.59745	1.90539	-0.74116	3.33653	3.66665	3.47878	4.91884	0.48028	0.85968	0.69006	1.72908
SAMD9L	0.99474	1.38011	1.39985	2.41835	-2.31018	-3.32193	-1.37969	-2.72021	1.6424	2.01577	1.88544	3.38017	-3.32193	-3.32193	-3.32193	-2.81137
VPS50	1.86651	1.76499	1.70528	2.37533	1.72429	1.0592	2.45806	0.94556	1.04664	1.40979	1.21919	2.13617	2.03123	2.16049	2.01804	2.05718
GNGT1	5.84455	6.41873	6.16743	7.39234	0.12597	-0.26673	0.19317	0.20873	-3.32193	-2.14054	-3.32193	-1.96683	4.39665	3.352	1.96258	2.00492
GNG11	5.58002	6.12317	5.98768	6.636	-0.00645	0.69197	-0.63168	0.2483	2.39717	2.75776	2.40897	2.55584	5.98893	5.73793	4.87658	5.29266
BET1	3.79401	4.13195	3.79794	4.71997	2.57256	3.03139	2.31439	2.89802	2.2868	2.65315	1.5151	2.06369	3.09556	3.12906	1.51292	2.13801
CASD1	1.32197	1.56517	1.64861	2.28052	1.81071	1.9676	2.67692	1.79184	1.68478	2.15211	1.51646	2.83212	3.17632	3.14335	2.93	3.15513
SGCE	3.74591	3.82344	4.15295	4.27593	5.07895	4.48184	5.14563	4.69114	4.48406	4.78524	4.24618	4.75302	4.98891	5.19338	4.84583	4.7555
PPP1R9A	-0.53197	-0.34472	-0.44433	-0.22493	1.47565	0.80463	2.10332	1.0324	-0.07482	0.83561	-0.65846	1.94895	2.62374	2.99903	3.2012	3.28262
PON3	-3.06841	-3.32193	-3.32193	-3.00866	4.92959	4.85326	4.94318	4.60546	-1.44238	-1.14568	-1.06454	-1.35332	4.2367	3.83511	3.99024	4.07232
PON2	3.99399	3.73747	3.88662	4.36978	7.53064	7.10223	7.85993	6.89835	4.25482	4.31229	4.20867	4.67168	7.5934	7.1763	7.01939	7.12409
ASB4	-3.32193	-3.32193	-3.32193	-3.32193	0.87921	-0.67058	1.11164	-0.94073	-3.32193	-2.59939	-3.32193	-3.32193	-1.51098	-2.03077	-1.42062	-0.67506
PDK4	-1.55451	-1.04235	-1.72037	-0.18356	3.59767	4.31314	4.29118	3.82316	-0.94557	0.56032	-1.84727	0.84259	1.56192	3.60959	2.62458	3.65378
DYNC1I1	1.99668	1.78924	1.678	1.91369	-3.22141	-2.89586	-3.10955	-2.83221	-0.85146	-0.92693	-0.41266	-0.73862	-3.32193	-3.32193	-3.32193	-3.32193
SLC25A13	5.2314	5.29691	4.98074	5.45483	5.20313	4.56066	5.65765	4.72385	4.71101	4.06719	4.36617	4.5656	5.41378	5.45986	5.45553	5.52396
SEM1	4.28987	4.12111	4.11848	3.84202	3.57939	4.51881	3.17241	4.53444	3.91986	3.92771	3.90532	1.97896	3.32665	3.55759	2.27887	2.56411
SDHAF3	3.77413	3.71697	3.53588	3.57617	3.41645	3.65202	3.69667	3.11067	3.14967	2.5409	2.55957	1.82797	3.68096	3.32073	2.86297	2.70332
ASNS	2.66773	1.87906	2.14197	1.24199	4.213	5.68793	4.58623	5.35037	4.28385	3.00225	3.43083	2.7312	2.39855	1.67226	1.71964	1.20796
OR7E7P	-1.9806	-3.32193	-3.32193	-3.32193	-0.93394	-0.63759	-0.7343	-0.62748	-0.65549	0.44065	-0.44304	0.41788	0.67993	1.60101	0.07297	1.59397
OR7E38P	4.83913	4.74937	4.87831	4.50332	4.76408	4.76059	3.96523	4.59608	4.95682	4.60963	4.50008	4.0538	3.65184	3.66783	2.71582	2.96148
LMTK2	3.37516	3.25203	3.23058	2.95307	4.34069	3.56007	4.42116	3.47903	2.70186	2.54207	2.57724	3.03404	3.08231	3.12843	3.37024	3.49672
BHLHA15	0.88803	0.88488	1.92112	0.65384	3.26309	4.0377	2.62821	3.36175	0.42604	-0.40259	0.38851	-2.37932	-1.98628	-1.98938	-2.37439	-0.79949
TECPR1	2.97224	3.45515	3.29068	3.12143	2.8445	3.99612	2.18871	4.08941	3.32922	3.58245	3.70575	3.27205	1.94702	2.54446	2.94357	3.21561
BRI3	6.67772	6.85666	7.0563	6.64473	4.97862	6.02399	4.91907	5.91395	4.7611	5.21469	4.91655	5.0287	5.45307	5.62966	5.46011	5.51318
BAIAP2L1	3.67421	3.84505	3.90043	3.84333	5.27076	5.25516	5.53118	5.09806	3.40229	3.61851	3.82538	4.24385	4.48568	4.71433	4.46143	4.5972
RPS3AP26	5.03859	5.14252	5.14096	4.63484	3.39022	4.10564	3.63111	3.30347	4.40309	5.25147	4.03614	3.80625	3.70617	3.77622	2.72717	2.73751
NPTX2	-3.32193	-2.95586	-3.32193	-3.32193	1.37398	1.72258	0.99829	1.8063	-3.32193	-3.32193	-3.32193	-3.007	-2.63953	-2.4711	-3.00498	-1.85838
TRRAP	3.58308	3.43422	3.55054	3.45016	4.05839	2.95465	3.94716	2.92877	3.81183	3.5646	3.75426	4.1807	4.09724	3.94144	4.69012	4.83481
SMURF1	4.44155	4.42276	4.62294	4.5566	4.29363	3.25211	4.58187	3.20698	3.95238	3.8867	4.31603	4.75704	4.88283	4.55463	5.78821	5.19352
KPNA7	0.66426	1.15121	1.54848	0.16655	2.9096	2.14457	3.0292	2.14883	-0.56646	-0.79274	-0.1643	1.10254	-0.25117	0.52072	0.51768	0.74901
MYH16	3.0841	3.87767	3.739	3.61815	-1.3107	-1.74144	-0.77982	-1.14792	1.45742	2.37262	2.48725	2.54008	0.81409	0.19797	2.22352	2.83534
ARPC1A	7.1019	7.24112	7.27256	7.2633	6.38081	6.3306	6.21344	6.20374	7.58921	7.21721	7.34402	7.35492	7.25945	6.85235	6.80769	6.83363
PDAP1	8.07973	7.86808	7.94072	7.71608	6.99751	7.15368	6.74205	6.91584	7.45974	6.96943	7.32928	6.17817	7.2833	6.75553	7.09015	6.73709
BUD31	6.00105	6.00544	5.90266	5.92133	5.16849	5.89399	5.24451	5.82668	5.50088	5.11748	5.19813	5.02179	5.80236	5.77252	5.24053	5.12938
PTCD1	5.5373	5.45034	5.35692	5.21716	4.95424	5.28707	4.69327	5.32074	4.8721	4.48153	4.66623	4.19881	5.69287	5.62413	4.49691	4.59993
ATP5MF-PTCD1	7.08223	6.93408	6.78886	6.54667	6.24371	6.8581	5.6238	6.85777	6.46062	6.09199	6.34148	5.24234	6.93581	6.88043	5.84571	5.93645
CPSF4	4.72229	4.50514	4.46757	3.88832	3.69071	3.81528	3.13084	3.82748	4.50367	4.03155	4.38087	3.45802	4.24242	4.00213	3.82302	3.64835

ATP5MF	7.32376	7.20215	7.06785	6.75868	6.07965	7.24799	5.46301	7.23224	6.53252	6.23715	6.44242	5.09948	7.25288	7.18179	5.79622	5.88979
ZNF789	1.52968	1.27513	1.2111	1.55787	0.93739	1.31925	0.67444	1.49629	-0.09399	0.05754	-0.43904	-0.33353	1.37457	1.35436	0.79992	1.15744
ZNF394	3.97371	3.86196	3.59246	3.98021	2.58413	3.00056	2.59267	3.0922	3.20744	2.95313	3.07499	3.025	3.06606	3.17857	3.08629	3.22141
ZKSCAN5	2.35721	2.60159	2.50004	2.83546	2.34799	2.09065	2.64359	2.20061	2.73109	2.73867	2.8352	3.19587	2.3276	2.67645	2.56066	2.89797
FAM200A	2.06518	2.31424	2.39066	2.73619	1.54928	1.53681	1.83444	2.02687	1.57951	1.77947	1.35396	2.25846	2.63965	2.80538	1.7885	2.46879
ZNF655	3.32644	3.27981	3.32741	3.31202	2.42897	2.70423	2.82478	2.56116	3.50237	3.3696	3.51975	3.55436	3.87474	3.74635	3.63484	3.30477
ZSCAN25	2.30006	1.99735	2.02828	2.00806	2.11844	1.69174	2.0004	1.81654	2.19819	2.10749	2.45211	2.2437	2.21496	2.34943	2.15905	2.34651
CYP3A5	-1.93551	-1.8304	-2.35385	-0.89785	-0.13921	0.31325	-0.2106	1.09069	-2.09526	-1.96768	-1.90385	-1.33959	0.15281	1.06299	0.30323	0.74443
TRIM4	2.77819	2.9558	3.22487	3.32711	2.7114	2.75323	2.88799	2.61816	2.8666	3.47672	3.21043	3.68949	2.74977	2.98711	2.7057	2.93675
ZKSCAN1	3.04887	3.26514	3.31027	3.79463	2.93029	3.16094	3.07614	3.36809	2.96224	3.36623	3.21751	4.19964	3.35625	3.21274	3.79272	4.09024
ZSCAN21	2.11879	2.07429	1.92415	2.29493	2.61049	3.29532	2.46377	3.33122	2.06664	2.84085	2.63784	2.83062	2.8199	2.78551	2.86834	2.86484
ZNF3	3.14917	3.05988	3.08022	3.42	2.66337	3.04355	2.60497	3.43364	2.45212	2.74319	2.59199	2.97187	2.83817	3.32307	3.21845	3.43989
COPS6	6.9157	6.76693	6.74172	6.45239	5.96717	6.31176	5.60581	6.11943	7.04049	6.25715	6.85643	5.78377	6.53386	6.16032	6.44169	6.05242
MIR25	5.4438	5.10109	4.30767	4.75099	4.72199	5.39706	3.38536	6.18252	0.23139	1.87337	2.54669	1.44769	3.93554	4.53485	1.45756	3.44888
MIR93	3.87306	3.5943	2.54765	3.62611	3.06014	3.78999	0.00803	4.73552	1.23593	0.05035	1.63925	-3.32193	2.94494	1.95854	0.03739	1.55592
AP4M1	5.25175	5.08544	5.33448	4.81308	4.41797	5.08399	3.66107	5.06315	5.11602	4.6493	5.1778	4.09588	4.13745	3.94071	4.50245	4.31998
TAF6	5.75406	5.63239	5.85689	5.21891	5.28747	5.59408	4.603	5.52381	5.71039	5.11686	5.60651	4.57815	4.97313	4.82965	5.50063	5.12714
CNPY4	3.1404	3.47007	3.51489	3.54831	3.11717	3.80275	2.88529	3.85024	2.52349	3.119	3.22625	2.46216	1.98442	2.61975	2.44075	2.64407
MBLAC1	2.00103	2.55258	2.42428	1.19082	2.01598	2.75347	1.67342	2.44551	1.36134	1.255	1.23379	0.34579	2.00327	1.77259	1.59664	1.81678
LAMTOR4	5.63837	5.51068	5.59557	5.51566	5.75272	6.63819	5.26935	6.54114	5.46079	5.47128	5.37521	4.22666	5.66827	5.56736	4.77356	4.93246
MIR4658	7.13951	7.12391	6.65157	7.4997	6.77658	7.5299	6.30804	7.49557	6.47452	6.52688	6.79992	6.22512	5.55065	5.60198	5.54967	6.63144
GAL3ST4	-2.77898	-2.49874	-2.97633	-3.32193	-1.91617	-1.09048	-2.00413	-1.53712	-3.32193	-2.58992	-2.76281	-3.32193	-1.752	-1.44406	-1.02139	-0.55705
GPC2	-2.04737	-1.71402	-2.18377	-2.30486	-0.31099	1.46723	-0.80305	1.67208	-1.91612	-1.96349	-2.28618	-2.85266	0.00774	1.10939	0.63395	1.16146
STAG3	-0.98018	-1.41876	-0.78071	-1.21409	0.69671	1.28177	0.17602	1.61245	-1.68867	-1.33472	-1.46478	-1.41768	-1.30536	-0.58919	-0.59691	-0.05173
CASTOR3	0.57469	0.60029	0.62095	0.84273	2.17699	2.69191	1.82515	2.91567	-0.31228	0.07692	-0.24391	0.86857	-0.39009	0.51995	0.36655	1.37488
PVRIG	0.47605	0.26025	0.78415	0.30548	1.56443	1.02719	0.38406	1.62191	-0.9736	-0.3603	-1.54309	-1.80883	-1.09615	0.64691	0.1485	0.89223
SPDYE3	1.48823	1.37201	1.11771	1.12905	1.74654	2.05175	1.39252	1.87791	1.43228	0.86482	0.97705	1.38477	1.24288	0.82535	1.71147	0.71376
PMS2P1	4.31341	4.10401	4.07018	4.12874	4.28591	4.11715	3.95947	4.24553	4.15418	3.71615	3.83872	3.43627	4.21481	4.34193	3.82986	3.98997
STAG3L5P	2.62334	2.11274	2.28914	1.56815	3.07027	3.12888	2.67318	3.08102	1.67293	2.0369	2.47941	2.35382	2.38337	2.87547	1.44161	2.53588
STAG3L5P-PVRI	4.22416	3.54521	3.9584	3.92817	4.05222	5.27343	3.04011	5.24268	2.87461	3.17057	3.5702	3.05883	3.28155	3.82447	2.18485	3.08671
PILRB	3.84393	3.17019	3.57346	3.53279	3.35037	4.63282	2.03894	4.72144	2.29882	2.53284	2.9082	2.1042	2.54822	3.04924	1.56265	2.32606
PVRIG2P	4.50066	3.69224	4.283	3.57548	4.3569	5.48258	2.78973	5.4215	3.13271	3.49475	3.88101	2.91607	3.26487	3.80496	2.45403	3.13316
MIR6840	4.77759	3.88978	4.96261	4.99273	4.5715	5.50508	1.66043	6.10872	2.94928	3.89311	4.64461	2.40092	3.87367	4.58457	3.07884	3.11002
PILRA	2.84404	3.39536	3.20501	4.04536	1.66573	3.46127	0.48556	3.54802	0.95891	1.00197	1.10037	0.71411	1.38725	1.41535	-0.69631	-0.2258
ZCWPW1	-0.01615	-0.5615	-0.2854	0.25408	1.24186	0.79233	1.09126	1.2518	-0.41017	-0.09762	0.22181	-0.76649	0.83668	0.60813	1.07721	1.1962
MEPCE	4.92365	5.00185	4.73967	4.88511	5.33169	4.66983	4.54983	4.73222	4.70208	4.43372	4.95487	4.10099	4.47819	4.23324	4.64732	4.85027
PPP1R35	7.1085	7.12603	7.24798	6.34703	6.35153	7.70648	5.2936	7.42537	6.00721	6.31482	5.84986	3.98588	5.07482	5.28518	4.5203	4.54779
TSC22D4	5.45097	5.85611	5.69981	5.49315	4.05263	5.07676	3.42855	4.8641	4.33503	5.14302	5.01556	4.5145	4.2395	4.76147	5.12458	5.61618
NYAP1	-0.50224	0.63927	0.46681	0.65572	0.152	2.02175	-0.72484	1.80653	0.35882	2.4933	1.55947	0.45637	-1.13267	-0.46369	-0.69761	0.38281
AGFG2	2.78079	2.76106	2.48685	2.45982	4.76816	5.05891	4.01628	5.00227	2.97993	2.08858	2.55289	1.33205	3.40299	2.97529	3.86798	3.9113
IRS3P	0.71632	1.60604	1.0778	1.92636	-0.53579	1.33234	-1.12007	2.229	-3.32193	-2.51957	-3.32193	-3.32193	-3.32193	-2.18147	-2.52568	-1.99669
SAP25	2.94573	3.1414	3.23888	1.68074	0.31023	1.87253	-1.2752	2.75505	0.78649	0.6377	1.34078	-0.93217	0.08915	-0.58326	-0.33275	-0.211
LRCH4	4.10187	4.09975	4.22265	3.80277	2.8711	3.35031	1.98034	3.27976	2.88668	2.47547	3.14342	1.71586	2.69302	1.97041	2.99409	1.99156
FBXO24	-1.69748	-2.49053	-1.64664	-2.65798	-0.83125	-0.31814	-2.26512	-0.02767	-1.54731	-0.87194	-0.84789	-1.85963	-1.92573	-2.14251	-1.45326	-1.52088
PCOLCE-AS1	-2.21914	-1.93776	-2.08333	-1.35932	-1.10293	-0.13246	-2.09596	0.21154	-1.44692	-0.33699	-0.65513	-1.53617	-2.39659	-2.39903	-2.25446	-1.89971
PCOLCE	2.47237	3.03815	2.8071	2.58036	1.38429	3.05279	-0.18535	2.94101	1.80613	2.34469	2.10811	1.44612	-0.91491	0.21851	0.3264	0.73585
MOSPD3	4.00568	4.73045	4.59888	4.51852	3.75149	4.46628	2.56908	4.28648	4.74743	5.26583	4.91164	3.55828	3.24346	4.12728	3.89419	4.27856
GNB2	6.93257	6.87601	7.13097	6.61686	6.5903	6.46784	5.56656	6.33594	7.76614	7.46332	7.77247	6.75081	6.83561	6.56014	7.1191	6.65285
GIGYF1	4.55192	4.20632	4.58508	4.01213	4.12298	4.42185	3.45459	4.65913	3.64515	4.07035	4.47551	3.91042	4.35569	4.68836	5.33362	5.47684
POP7	7.56123	7.16011	6.88736	6.23588	6.68543	6.9671	5.91761	6.81738	6.46142	5.721	6.07814	4.01662	6.64449	6.66747	5.92363	5.96047

EPHB4	3.11491	2.97643	3.01569	2.65114	4.85697	4.11527	4.23954	3.92654	5.06762	4.55817	5.19065	4.21721	4.28319	4.01624	5.33557	5.17925
SLC12A9	2.26409	2.40889	2.61523	2.02354	3.19266	3.31162	2.376	3.40791	4.02937	3.48639	4.1778	2.82079	2.96181	2.93376	3.57963	3.37434
TRIP6	6.82442	6.66154	6.82626	6.19615	3.02409	1.87054	2.54593	2.03082	6.90299	6.68669	7.09137	5.51031	6.52492	6.55371	6.58523	6.02236
MIR6875	5.06679	5.48755	5.40065	4.45107	0.94952	-3.32193	-3.32193	0.46688	3.91986	5.34479	4.97118	2.85862	3.46968	3.66928	3.63808	3.09008
SRRT	6.16445	5.75446	5.65852	5.57576	5.69771	5.42275	5.27587	5.66474	6.67182	5.78833	6.34895	5.39649	5.30252	5.33404	5.32823	5.39387
UFSP1	2.08065	1.88203	1.81384	1.74254	2.66786	3.45583	1.65762	3.33768	0.86468	1.6349	1.33441	-0.01967	0.90333	0.39682	0.97081	1.93588
ACHE	-1.35392	-0.27964	-0.63895	-1.01769	1.11002	2.24096	0.07238	2.19482	0.82242	1.59868	1.63012	1.03724	-0.64884	2.00488	0.39122	1.74329
MUC3A	-3.32193	-3.21546	-3.32193	-3.32193	1.28783	0.10136	0.73164	0.3888	-1.3169	-0.85199	-1.55495	-1.8643	-3.10734	-2.86473	-3.32193	-2.76992
MUC12	-3.13806	-3.05479	-2.60461	-3.02469	0.37504	-3.02439	-0.05475	-2.86016	-1.61505	-1.75338	-1.7738	-2.56728	-2.92198	-2.84782	-3.09169	-2.75015
TRIM56	4.57548	4.57159	4.46797	4.38364	3.72748	3.75943	3.49385	4.0437	3.91588	3.94603	4.17137	3.88926	3.23188	3.45651	4.05028	4.26881
AP1S1	6.73755	6.88753	6.90485	7.01924	5.90343	5.83941	5.69844	5.39943	6.72743	6.85416	6.90998	6.01009	6.66672	6.82478	6.3869	6.55456
VGF	0.47677	3.75853	2.66058	3.93378	1.6819	6.48261	1.81943	5.81314	-0.32081	1.26139	-0.14749	0.62493	0.53525	3.04543	0.84723	3.69561
MOGAT3	-2.28647	-3.32193	-2.82158	-2.79412	4.77923	4.70814	4.41045	4.78498	-2.82958	-3.32193	-3.32193	-3.32193	-3.32193	-3.32193	-3.32193	-3.32193
PLOD3	6.56461	6.52147	6.61255	6.24065	6.45535	6.88612	5.84927	6.94078	5.78879	5.64267	6.03906	5.12876	4.67303	4.17466	4.83058	4.78024
ZNHIT1	5.43961	5.44025	5.40155	5.30654	4.27656	5.18085	3.94281	4.90553	4.7293	4.76397	4.69642	4.3625	4.48413	4.45967	4.46343	4.59671
CLDN15	2.10834	1.89273	2.14487	1.68517	2.54376	3.31808	1.44859	3.60804	1.06745	1.74828	1.56683	0.93032	1.54416	2.01336	1.32635	1.35666
FIS1	6.06493	6.10982	6.09232	5.9311	5.49121	6.18848	4.92458	5.99591	5.36143	5.58641	5.38856	4.62367	5.08347	4.98301	4.40564	4.6264
IFT22	3.71127	3.85581	3.75448	4.03892	2.89822	2.85234	2.876	2.41353	3.28864	3.34118	3.25944	3.57573	3.74444	4.08426	3.85445	3.93503
CUX1	2.80518	3.19687	3.2901	2.98476	2.65585	2.84673	2.24206	2.80566	3.04794	3.61085	3.5178	3.80201	2.11268	2.49873	3.25833	3.47445
SH2B2	2.28855	3.1783	3.26123	2.47665	2.79291	3.96683	1.549	4.06945	2.85082	3.65472	3.07556	2.26961	1.47405	1.68971	2.52531	2.99043
SPDYE6	3.50979	3.39315	3.63097	2.79431	2.83923	3.85655	2.36608	4.73799	-0.43404	0.55779	1.13228	1.41528	1.70674	2.30361	2.31152	2.86122
PRKRIP1	4.38031	4.35335	4.3131	4.09304	3.91909	4.52943	3.39883	4.47437	3.52645	3.64766	3.6883	3.18554	3.38057	3.51441	3.31672	3.3275
ORAI2	4.02462	4.32793	4.46081	4.60425	1.99927	2.68381	2.13105	2.26728	3.49051	3.54352	4.11437	3.85068	3.48266	3.23074	4.58972	4.23143
ALKBH4	4.39786	4.41365	4.30643	3.7675	3.53318	4.0047	2.69176	3.74801	3.39482	3.63237	3.88851	2.69967	2.73872	2.76735	2.84461	2.99908
LRWD1	5.26532	5.26334	5.17197	4.73532	4.06112	4.29706	3.39281	4.1748	3.93622	3.65714	4.13879	2.98585	3.75381	3.69959	3.943	3.77866
MIR5090	7.45461	7.64346	7.6298	6.86444	7.26728	6.19046	6.71334	6.18714	6.9939	6.74845	7.17814	5.79437	6.97727	6.77964	7.72156	7.47824
POLR2J	7.54291	7.30256	7.29203	6.90063	6.31555	7.19136	5.79695	7.10199	6.11147	6.14114	6.07695	4.5812	6.36689	6.36331	5.76273	5.68874
RASA4B	2.91466	4.47729	4.31024	4.70556	2.39194	4.06756	1.76892	3.415	1.90814	4.1084	3.18907	3.74595	0.57962	1.23464	2.07669	2.33035
POLR2J3	4.48911	4.23185	4.39845	4.15085	3.97668	4.84728	3.2459	4.9652	2.36917	2.39345	2.38081	1.64625	3.69202	3.23904	3.78081	3.57267
SPDYE2	3.49823	3.02328	3.39014	2.7073	2.91858	3.91623	2.44614	4.71192	0.08115	0.52251	1.10875	1.59062	1.82106	2.48472	2.39227	3.00578
RASA4	2.30818	3.8427	3.68115	4.18191	1.80545	3.56158	1.26074	2.92985	1.38574	3.52784	2.57847	3.25219	0.08893	0.75082	1.56675	1.84706
UPK3BL1	5.71892	5.35279	5.6052	5.14119	5.64627	6.65188	4.47115	6.7218	3.52706	3.28509	3.45206	2.89659	5.49766	4.57575	5.51614	5.11435
SPDYE2B	3.40947	3.26328	3.44995	2.64322	2.77508	3.88329	2.24801	4.70454	-0.90134	0.36957	0.69005	1.48477	1.78466	2.41524	2.26923	2.96584
POLR2J2	4.49188	4.2285	4.48595	4.3577	4.41696	4.76524	3.85639	4.649	2.07264	2.55154	2.48281	1.57823	4.28134	3.63491	4.1443	3.89895
RASA4DP	2.98776	4.73437	4.61844	5.02057	2.77303	4.37267	2.15394	3.44345	2.22948	4.0888	3.40066	3.32783	0.6972	1.05013	2.18578	2.2743
FAM185A	2.17254	2.14056	2.23756	2.05553	2.34379	2.01414	2.30203	2.27373	0.69655	0.80361	0.85502	1.17432	1.82016	2.02645	1.74775	1.89062
FBXL13	1.64158	1.83862	1.72726	2.01248	-1.14067	-1.27515	-0.76632	-1.16684	2.55659	2.85425	2.58887	3.32079	0.79743	0.82986	1.26929	0.84824
LRRC17	-0.27645	-0.35084	-0.16669	0.17113	-2.08561	-0.67064	-1.78762	-1.28384	0.72413	1.38899	0.85906	0.85276	-0.97342	-0.79633	-0.28543	-1.09476
NFE4	3.06379	2.9406	3.08568	3.30362	-3.32193	-2.35502	-3.32193	-3.32193	4.55651	4.7306	4.47284	4.84961	-2.86791	-3.32193	-3.32193	-2.37977
ARMC10	4.21367	4.13894	4.10138	4.33692	4.34763	3.85378	4.55233	3.6199	3.72016	3.71769	3.02505	3.74778	4.75188	4.21365	4.30787	4.09731
NAPEPLD	1.01401	0.82422	0.79041	1.61093	1.88279	1.03643	2.31806	1.23049	0.71294	0.66425	0.46052	1.48811	2.50613	2.73089	2.31347	2.69395
DPY19L2P2	-0.09767	-0.26233	-0.1935	0.20605	-0.27476	-1.14696	-0.29676	-0.18846	0.1406	0.71583	-0.21145	0.46363	0.59041	1.49842	0.02332	0.84532
PMPCB	4.67756	4.76254	4.73287	4.94297	4.61548	4.7015	4.95473	4.67958	4.50194	4.42781	4.47572	4.56694	4.64457	4.55352	4.37034	4.12699
DNAJC2	4.69091	4.59855	4.30913	5.13535	4.12015	4.36475	4.36349	4.68975	3.39662	3.23325	3.17463	3.76269	3.88849	3.94087	3.24848	3.04977
PSMC2	6.70729	6.80947	6.74818	7.08838	6.28958	6.18098	6.6388	6.27127	5.99459	5.61499	5.8835	5.52435	5.58997	5.32137	5.29921	4.8994
ORC5	3.52357	3.48084	3.17602	3.40924	3.88944	3.60575	4.35942	3.59961	2.99951	2.8428	2.78225	2.83062	3.76236	3.28747	3.20683	2.85977
EIF4BP6	2.8714	3.2094	2.98122	2.76549	2.20976	1.22171	2.18229	1.01362	4.31036	4.5098	4.30133	4.38605	2.3285	1.40712	2.22669	2.08517
LINC01004	-0.01145	0.84357	-0.41429	0.94051	-0.3229	0.92899	-0.10049	0.72315	-3.32193	-2.10566	-3.32193	-1.0282	-0.9157	0.01532	-0.53605	-1.44275
KMT2E-AS1	3.6729	3.67406	3.61097	3.77383	3.95478	4.04608	3.86489	4.14237	3.69711	3.45686	3.37415	1.72201	3.56451	3.76557	4.61854	4.81728
KMT2E	2.94812	3.21152	3.25141	4.21971	3.15703	3.07276	3.46266	3.40739	3.56048	3.54166	3.38507	4.48911	3.76865	3.91999	4.28258	4.21483

SRPK2	4.1685	4.19291	4.28554	4.88812	3.58981	3.39336	4.03091	3.81215	3.71187	3.3476	3.488	4.03788	3.83807	3.77271	4.13219	3.81749
PUS7	3.75245	3.13902	2.96645	3.12449	3.55135	2.90379	3.79848	3.27562	3.15957	2.36783	2.89317	2.97673	4.13025	3.25018	3.27858	2.94927
RINT1	4.16487	4.04627	3.93497	4.45597	4.24332	3.64742	4.76319	3.60557	3.54851	3.31457	3.48656	3.70306	3.59029	3.12429	3.27339	2.96888
EFCAB10	3.4592	3.41636	3.28772	4.05261	2.7655	3.24878	3.23292	3.10206	2.30132	1.84144	2.21827	2.69887	2.44729	1.93988	1.60018	1.31016
YBX1P2	4.74552	5.51153	4.78284	6.14193	0.40144	1.69688	0.82206	1.69678	2.59932	3.06018	2.15251	2.07874	-0.92673	0.45796	0.59389	1.29293
ATXN7L1	-0.28551	0.32791	0.10496	0.30325	-0.25306	-0.339	-0.30859	-0.08042	0.09481	0.54833	0.22181	0.9197	0.38089	0.33496	0.3451	0.09471
CDHR3	-1.15058	-1.43276	-1.7631	-1.46325	0.14357	0.05821	0.05723	0.15834	-0.41658	-0.11449	-0.91293	-0.55347	0.94669	1.33306	0.32209	0.36318
SYPL1	6.18338	5.95097	5.93449	6.36804	6.09118	4.88464	6.46487	5.01335	5.53095	4.98407	4.76384	5.32514	6.42832	5.56215	5.41794	4.90825
CCDC71L	4.91974	6.55214	5.88535	6.3557	-0.18626	1.15858	-1.17666	0.91737	4.23063	5.44522	5.08959	5.11603	1.52253	2.96123	2.56819	3.2875
LINC02577	1.26349	1.44973	1.5249	1.75021	-3.32193	-3.32193	-3.32193	-3.32193	-0.08851	-0.14732	-0.23603	0.26416	-1.3517	-2.89743	-3.32193	-3.04236
PIK3CG	-3.14448	-2.95031	-3.11396	-2.91002	-3.09942	-3.32193	-3.00655	-3.32193	0.65596	1.0836	0.98886	1.62769	-3.32193	-3.32193	-3.32193	-3.14852
PRKAR2B	0.32233	-0.10914	0.02241	0.7556	-2.8555	-2.69414	-2.73376	-2.13118	2.93397	2.26215	2.71395	2.88613	-1.444	-0.62768	-0.37844	-1.2663
HBP1	2.98793	3.16362	3.54625	4.06971	3.3133	2.97442	3.76132	2.67665	3.37433	3.58944	3.45942	4.39465	3.60102	2.82243	3.56813	3.30261
COG5	2.99364	3.09191	3.25025	3.64708	3.35633	3.02584	3.8383	2.78955	3.33544	3.266	3.06046	3.93448	3.66056	3.09606	3.55869	3.3067
DUS4L	2.20004	1.94403	2.02001	2.10341	1.67116	1.43133	2.01054	1.54469	0.81445	0.93835	0.4863	1.22993	1.98434	1.74532	1.47711	1.35882
BCAP29	3.31039	3.68861	3.69832	4.20016	3.78659	3.35008	4.46789	3.28695	3.45518	3.08775	2.881	3.72705	4.15329	3.49965	3.50835	3.31651
WBP1LP2	-0.06575	1.01458	0.95803	0.35399	1.48971	1.92431	1.47814	0.65989	1.74596	-0.36788	1.13397	1.25425	0.75194	-0.16941	0.85417	-0.21943
CBLL1	3.42416	3.43883	3.17121	3.57482	2.63602	2.44367	2.97915	2.61974	2.98516	2.8008	2.38391	3.42741	3.35919	3.46078	3.36558	3.54082
DLD	5.29194	5.39921	5.09066	5.78707	5.4483	4.9196	5.79222	5.12687	5.15847	5.29153	4.70968	5.455	5.64559	5.87213	5.35666	5.60447
NRCAM	1.12074	1.03263	1.11437	2.37363	4.82471	0.14493	5.28435	0.32107	-3.32193	-3.32193	-3.32193	-3.32193	-3.12227	-2.94809	-3.32193	-2.30981
PNPLA8	3.78873	4.38399	4.04305	4.98715	3.22624	2.98085	3.84442	2.81184	2.64973	2.67174	2.34856	3.07617	3.05611	3.42436	3.07739	3.34321
RPL7P32	2.19713	1.65271	1.72825	-1.46045	-0.15606	1.7526	0.34311	0.8774	1.26533	3.00842	0.36595	1.05509	1.09972	0.40219	-0.00524	-0.12911
THAP5	2.76465	2.56796	2.77927	3.4096	2.90466	2.55421	3.50947	2.72524	1.90184	2.09211	2.00209	2.47065	2.84727	2.33517	3.17744	2.58695
DNAJB9	3.63033	4.10202	3.86345	5.02671	5.14178	5.50045	5.80535	5.25929	3.14481	3.48282	2.85068	4.12302	4.19852	4.41032	3.45491	4.01538
IMMP2L	0.50559	0.40692	0.7342	0.99696	0.8252	1.5279	1.02343	1.29543	0.90458	1.4271	1.28306	0.83388	0.9263	1.46693	0.42036	0.95825
DOCK4	1.34287	1.61451	1.47443	1.92521	1.90101	1.38854	2.1651	1.51682	3.10637	3.14525	3.30478	3.94833	4.89294	4.87982	4.57375	5.30357
ZNF277	2.95546	3.22421	3.12318	3.67449	2.77786	3.05837	3.16088	3.22065	3.31054	4.0121	3.29199	4.6349	3.03901	3.30463	2.48574	2.99767
IFRD1	3.98664	3.68355	3.69269	4.08661	3.56166	3.91293	4.06011	3.80525	2.00106	2.15935	1.72216	2.23803	3.80817	3.7769	2.64715	2.64361
LSMEM1	-0.34247	-0.08896	-0.83351	0.30334	-0.3696	1.13023	0.0708	1.67738	-2.89106	-1.72682	-2.3729	-0.52263	-1.22292	-0.23789	-2.04133	-0.74482
TMEM168	1.32064	1.02046	0.93894	0.97287	2.8627	2.03722	3.25293	2.12834	1.71953	1.35196	1.51372	1.46445	2.78957	2.06509	2.06922	1.82712
BMT2	0.9406	1.04111	1.39679	1.9236	1.87211	1.76539	2.28675	1.40227	2.94238	3.16678	2.40267	3.84577	2.65217	2.84235	2.55681	3.04671
GPR85	-0.27916	-0.66652	-0.97068	-0.46868	-3.32193	-3.32193	-3.32193	-3.15224	0.00377	-0.40022	-0.9936	-0.1114	-0.67542	0.32774	-0.41267	0.48599
SMIM30	4.94874	5.20384	4.84118	5.8892	3.59435	4.8799	4.19584	4.7562	4.50769	4.74966	4.149	4.74825	5.69476	5.75596	4.35762	4.86697
MDFIC	2.85846	2.48288	2.64676	2.85326	-2.92818	-3.12103	-2.97043	-2.71094	4.0567	2.77705	3.28458	2.49047	4.00168	2.14975	3.31032	2.00291
TES	4.44905	4.43928	4.71634	4.44636	4.50388	2.93618	5.23043	2.47425	3.44796	3.10112	2.80228	3.69966	5.09693	5.32874	4.9259	4.54769
CAV2	3.88992	4.11844	4.51191	4.14436	2.46486	0.75377	3.09091	0.37802	5.1525	4.91775	5.10691	5.22857	6.0275	5.5437	5.87523	4.62927
CAPZA2	3.75134	4.04339	4.02064	4.37044	4.96961	3.89364	5.67527	3.75024	4.10616	4.34911	3.94375	4.4727	4.61518	5.04755	4.45772	4.29864
ST7-AS1	1.5734	1.02634	1.16462	1.79551	3.04115	1.73994	3.56973	1.55305	2.00579	1.48239	1.56155	0.97789	1.82543	1.96966	2.2534	2.16038
ST7	2.26207	1.94375	2.2146	2.37067	3.10223	2.89614	3.51015	2.8764	2.32116	1.30127	1.9869	1.88378	2.61032	2.52193	2.47287	2.4366
ST7-OT4	-0.32335	-0.84219	-1.16763	-0.79056	-0.35748	-0.53716	-0.26144	0.14392	-0.88836	-0.93953	-1.11351	-1.7694	-0.96907	-0.48589	-1.30056	-0.30265
ST7-AS2	-0.00919	0.10117	0.59611	0.93498	1.761	0.5277	1.77036	0.21775	0.87282	0.40853	0.2216	0.17233	1.06643	1.26558	1.11663	1.48312
WNT2	-3.32193	-3.32193	-3.32193	-3.32193	-3.03578	-3.32193	-3.32193	-3.32193	-3.32193	-3.32193	-3.32193	-3.32193	0.04372	2.54749	-1.01583	-0.18224
CFTR	-3.32193	-3.32193	-3.32193	-3.32193	-0.80551	0.29166	-1.26977	0.44072	-3.32193	-3.32193	-3.32193	-3.32193	-3.32193	-3.32193	-3.32193	-3.32193
LSM8	3.33605	3.24131	3.27278	3.49447	2.92602	3.58385	3.12453	3.57144	2.38627	2.38792	2.28995	2.17987	3.55117	3.71421	2.3605	2.56799
LINC02476	1.18883	1.53405	1.39846	2.42158	-1.27201	1.36697	-1.65293	1.75999	-3.32193	-3.32193	-3.32193	-2.24677	-3.32193	-2.63694	-2.86605	-3.32193
TSPAN12	2.35716	2.23386	1.6337	2.45465	3.62795	3.17742	3.66732	3.01363	0.01536	-0.26309	-1.90452	-0.00626	-3.08249	-3.32193	-3.32193	-3.02929
ING3	1.99999	1.72588	1.88656	1.94795	0.9605	0.86054	1.48313	0.60063	0.56965	0.64533	0.29358	0.72442	1.15212	0.76856	0.70918	0.2503
CPED1	1.07349	0.79856	0.28897	0.64372	-2.39715	-2.54548	-2.11007	-2.64472	-1.85922	-2.27337	-1.79672	-2.42708	1.5407	-0.14871	-0.30104	-0.00794
FAM3C	5.31107	5.38886	5.40576	5.9219	5.48432	5.43639	6.18462	5.09925	5.04557	5.23297	4.74745	6.06215	5.88096	5.5437	4.98413	5.39906
AASS	2.72802	2.46146	2.61552	2.48148	1.3657	1.51192	1.49271	1.32934	2.43966	1.60137	1.8973	2.04914	2.00728	1.64251	1.89353	1.86667

CADPS2	1.5654	1.82489	1.69675	2.16392	1.13199	1.80136	1.40881	1.50951	1.78755	1.49075	1.80959	2.2571	-3.32193	-3.19047	-3.32193	-3.32193
RPS26P31	2.66554	-0.23282	1.82166	-3.32193	1.41002	2.95731	0.82002	2.56946	1.56148	3.41115	2.00993	1.88527	-0.67999	-0.0432	0.21991	-1.65453
IQUB	-1.95432	-1.09062	-0.66542	-0.62446	-2.63014	-2.58571	-3.00477	-2.62008	-2.93659	-2.2223	-2.69363	-1.51579	-2.82346	-2.23045	-3.32193	-2.72347
NDUFA5	3.43074	3.46912	3.31295	3.80165	2.46833	2.8395	2.92345	2.83353	2.66991	2.73969	2.52099	2.70062	3.15208	3.30885	2.2903	2.46074
WASL	3.78868	3.56152	3.86916	4.02425	4.33341	2.68579	5.1045	2.68895	3.72021	3.19299	3.6014	3.94912	4.79028	3.47921	4.66273	3.55421
GPR37	-2.49759	-2.2153	-1.30627	-1.13051	-0.56004	-1.19205	-0.51556	-0.55516	1.83349	2.76843	1.87432	2.47391	0.85662	1.61738	1.52191	1.72642
POT1	2.02203	2.23993	2.23259	2.71804	2.1241	1.44439	2.50526	1.47346	1.90929	2.34702	1.69416	2.69503	2.55437	2.91034	2.49286	2.67368
ZNF800	2.34594	2.56181	2.6274	2.86888	2.41081	0.99742	2.85366	1.41444	2.1672	2.27926	2.29749	2.82227	2.03527	1.5785	2.4454	2.07669
GCC1	4.19077	3.85907	3.96574	3.98343	4.37861	4.2948	4.2763	4.25769	4.11448	3.90566	4.13557	3.83126	4.0956	4.18602	4.10079	4.252
ARF5	6.36529	6.42082	6.5427	6.35757	6.57927	7.25754	6.06044	7.10234	6.47904	6.80247	6.73935	6.2336	5.72633	6.23192	5.61855	5.73692
FSCN3	4.0034	3.72742	3.96403	3.99291	3.90409	4.73153	3.40961	4.74941	3.8607	3.94693	3.84891	3.55486	3.56891	3.96828	2.9593	3.24114
SND1-IT1	0.01035	-0.08516	-0.45395	0.64263	-2.83797	0.06883	-2.20555	0.97114	-2.02652	-2.64087	-2.91695	-2.40379	-3.32193	-3.00671	-1.694	-1.5386
RBM28	2.72164	2.30046	2.12419	2.26286	2.61956	2.77426	2.86715	2.75743	2.25343	1.5417	2.31468	1.78388	2.68159	2.37899	2.46038	2.18859
IMPDH1	6.053	5.79881	5.62391	5.17443	5.43197	5.31262	4.89411	5.13087	6.09359	5.65055	5.89776	4.81393	5.77067	5.64191	6.52747	6.38105
HILPDA	4.55895	5.31261	5.86297	4.713	5.03455	6.31048	5.03511	5.88399	4.82923	6.08591	4.19813	4.88239	2.92415	3.01622	2.75666	2.63467
METTL2B	4.76885	4.6129	4.61635	4.37762	4.12539	3.50969	4.39083	3.77567	3.06823	2.99117	3.20747	3.44529	3.65281	3.45567	3.57988	3.15897
CICP14	2.34241	2.59995	2.54972	2.12687	2.63903	2.33353	1.77176	3.08402	1.64981	2.46573	2.17454	2.40537	1.4818	1.85842	2.19387	2.71357
FAM71F2	-1.26169	-0.30094	-0.49333	0.63311	-3.1685	-2.98036	-3.32193	-2.14751	-3.11584	-2.40223	-2.9729	-2.32057	-2.72025	-2.29955	-2.73263	-1.48264
OPN1SW	3.22907	3.7327	3.68392	4.17541	2.68162	4.16414	2.93272	3.9476	0.58428	1.81323	1.66951	2.3221	0.40109	3.14311	1.71266	2.17719
CCDC136	-0.18832	0.35898	0.75697	0.63847	-1.80371	-1.30633	-1.79927	-2.0938	-0.54973	-0.60746	-1.02976	-0.88915	-1.85273	-2.65521	-1.61034	-1.25763
FLNC-AS1	5.46766	5.67847	5.47612	4.77834	-1.34069	-3.32193	-1.47714	-1.91518	8.41679	7.50712	8.21694	6.71968	1.92533	2.40666	3.40219	2.78125
KCP	4.60611	4.84799	4.74042	4.44845	4.20593	4.63413	3.89535	4.36215	3.39962	3.45484	3.73025	1.56976	3.3328	3.32136	3.47595	3.33437
ATP6V1F	8.90434	9.01226	8.8953	8.64728	8.22024	9.02969	7.7436	8.83489	7.66081	7.64767	7.81639	6.16425	7.69629	7.57626	7.36154	7.22645
ATP6V1FNB	-2.18874	-1.62928	-1.38543	-1.85848	-0.78213	-0.42752	-0.64741	-0.68856	-2.06662	-0.88591	-1.61416	-1.31583	-2.49075	-2.62497	-2.10202	-2.20782
IRF5	-0.43706	-0.00577	-0.53853	0.17653	1.03127	1.45241	-0.5573	1.36104	1.08459	0.99915	0.5459	0.49749	-0.84003	0.22197	0.28434	0.23061
TNPO3	4.86278	4.7115	4.64468	4.71883	5.2486	4.69359	5.59023	4.733	5.13724	4.55261	4.5798	5.21733	5.44009	5.51194	5.60028	5.67723
TPI1P2	-2.21466	-1.31357	-1.82446	0.3944	-0.0497	-0.72317	-0.56771	-1.10117	-2.09432	-0.38102	-0.15644	-0.03583	-1.55146	-0.861	-1.81762	-0.29787
TSPAN33	-2.43699	-2.92492	-2.097	-3.32193	4.80385	4.118	4.33632	3.63063	2.92833	2.02623	2.7204	1.55386	2.65327	2.05444	3.71607	3.11067
SMO	-1.36567	-1.38497	-1.67443	-1.82716	4.78235	4.40825	3.89774	4.47378	4.23167	4.4243	4.61658	3.92154	3.90361	4.1871	5.33163	5.2729
AHCYL2	1.41015	1.48176	1.62755	1.85949	2.50839	1.59349	2.86878	1.38054	1.4837	1.69244	1.6383	2.35035	2.20287	2.12958	2.36947	2.53021
STRIP2	2.78984	2.9036	2.7815	3.21529	1.65096	0.43864	1.52309	0.52141	0.839	0.06007	0.17149	-0.23229	1.68467	0.79862	1.0565	1.06893
SMKR1	1.27776	0.76482	0.6873	1.50856	1.83648	1.74853	1.63684	1.83933	0.3023	0.41064	0.24799	-0.58056	1.71033	1.45112	1.60187	1.33048
NRF1	2.59138	2.37559	2.21639	2.14499	2.42813	1.5809	2.27559	2.13643	2.85827	1.95375	2.38037	1.84638	2.92653	2.39782	2.79953	2.63557
RNA5SP244	4.93501	5.28501	4.8902	4.7194	4.5989	4.66237	4.54759	5.15143	5.15141	4.07087	4.61515	3.65325	4.87853	4.61135	5.05226	4.68914
UBE2H	5.09007	5.25362	5.28795	5.40931	4.02521	3.71429	4.62015	3.32296	5.32493	5.50575	5.53452	6.12556	5.7167	5.78309	5.99788	6.04449
ZC3HC1	4.1461	3.9453	3.90712	4.08369	3.75833	3.79562	3.64006	3.56212	3.52547	3.18158	3.69122	3.3597	3.93982	4.25816	3.84041	3.99066
KLHDC10	2.67668	2.91314	2.98504	3.29802	1.16055	0.13147	1.52548	0.06756	2.72803	3.03822	2.75824	3.7854	3.77208	3.95489	4.24025	4.0485
TMEM209	3.65821	3.67433	3.60821	3.72629	3.22016	2.57776	3.59461	2.45746	2.84211	1.79303	2.23122	2.69369	3.85662	3.30041	3.61045	3.40217
CPA4	-0.58066	0.77118	0.78758	-0.24703	-2.90712	-3.06099	-2.13209	-2.55666	2.77058	1.64405	2.57296	0.50572	2.35278	2.1024	2.94855	1.24923
CEP41	1.75687	1.2764	1.4356	1.69002	0.75669	0.01683	0.89853	0.35094	0.97569	0.18436	0.22468	0.60117	2.43383	2.13934	2.44273	2.06782
MESTIT1	-3.07282	-2.4374	-3.03117	-3.32193	-1.36528	-0.79488	-1.60514	-1.19676	0.0287	1.20105	0.45321	-0.14559	-0.69716	1.28648	0.2602	0.56502
MEST	-0.34532	0.20589	-0.79455	0.36153	1.24523	2.82055	1.43963	2.87889	3.40597	4.12839	3.56727	3.8108	2.29824	4.22728	3.01229	3.49863
COPG2	3.29197	3.21028	3.43102	3.77978	1.41809	2.09457	1.66929	2.19627	3.89718	4.03012	3.94552	4.62761	3.70836	4.40952	3.96052	4.17548
TSGA13	-0.28368	-0.64414	0.05127	-0.41392	-0.21968	-2.29186	-0.6322	-2.16752	1.04041	1.44217	1.5594	1.45322	0.36099	0.8823	1.87792	1.7451
LINC00513	0.01795	0.82464	-0.25208	0.09771	1.6504	3.66963	2.40609	3.09666	-1.39926	-0.24982	-2.32549	-1.42207	0.20605	1.48512	0.08805	0.34676
LINC-PINT	1.93742	1.82929	2.12984	1.88841	0.29195	0.77935	-0.02545	0.74358	-0.12153	-0.03131	-0.4674	-0.07798	-0.37081	-0.56273	-1.22351	-0.3752
MKLN1	2.30259	2.60882	2.49403	2.98848	2.71603	2.43005	3.44566	2.32694	2.7088	2.89643	2.69475	3.64031	3.56128	3.31629	3.58557	3.81675
MKLN1-AS	1.61964	1.65449	1.59099	2.07518	2.10457	0.96332	2.83144	0.32887	1.44625	2.03458	1.69557	1.26513	1.86333	0.9651	1.8563	2.08969
PODXL	6.92197	7.26486	6.85439	6.816	1.42213	1.28024	0.74921	1.64228	4.71816	5.36764	3.74655	5.80554	0.24852	-0.36091	-0.88371	-0.0109
EEF1B2P6	1.92232	1.89696	1.63494	-0.03875	1.07594	1.93779	0.47691	1.57555	1.16655	2.50087	1.62605	1.26053	0.86123	0.44398	-0.02792	0.42

PLXNA4	-3.32193	-3.17603	-2.90296	-3.32193	-3.1574	-3.32193	-2.98222	-3.32193	-2.8513	-2.73424	-2.74556	-1.72628	-3.21856	-3.32193	-3.08187	-3.02174
CHCHD3	5.58927	5.52178	5.39315	5.08979	5.5903	5.10876	5.83162	4.75212	5.53904	5.04158	5.29185	4.89645	5.12884	5.33048	5.08078	4.80191
MIR3654	10.3054	10.5801	10.462	10.1448	9.85668	9.94016	9.70705	9.67547	10.7675	10.6243	10.9334	10.1455	9.59869	9.37118	10.2668	10.1448
EXOC4	3.20435	3.34461	3.43484	3.66519	3.02076	2.06588	3.34433	1.74305	3.68386	3.60748	3.66051	4.41356	3.54613	3.42975	4.34148	4.08257
SLC35B4	3.34567	3.12137	3.28906	3.41934	3.0846	2.75383	3.68812	2.69216	2.35762	2.11857	2.47576	2.91879	3.20572	3.08801	3.74644	3.58502
AKR1B1	4.91603	4.74826	4.87986	5.41766	3.45672	3.07362	2.83728	2.66217	5.43165	5.18489	5.13136	4.74613	6.08634	6.19348	5.60916	5.97985
AKR1B10	0.68982	0.20031	0.20722	1.2984	2.90861	-0.28644	2.90577	-0.00024	-3.32193	-3.32193	-2.77496	-3.32193	-1.27307	-0.95209	0.45907	0.48836
BPGM	3.50838	4.06228	3.82286	4.70861	3.64064	3.48484	3.74072	3.46515	3.1325	2.80075	2.90271	3.11247	3.33089	3.77376	2.95895	3.50411
CALD1	4.23593	4.21286	4.65325	5.0417	4.9451	4.32415	5.31286	4.64764	3.43729	2.7026	3.6847	2.83015	4.33856	3.87839	4.83879	3.53841
AGBL3	-1.05176	-1.61479	-1.14451	-1.15641	-0.41583	-0.10948	-0.58828	-0.24234	-2.1619	-1.97917	-2.10756	-1.63701	-0.2083	0.51264	-1.13627	-0.13846
CYREN	3.58873	3.59305	3.5457	3.45469	4.57745	4.29576	4.51418	4.14358	3.20232	3.1012	3.07221	3.07453	3.42627	3.85495	3.63835	3.63039
TMEM140	3.74284	3.83399	3.84113	3.96381	4.85376	4.59822	4.67959	4.30275	3.45703	3.58972	3.40568	3.72308	3.53125	4.17336	3.80775	4.10373
WDR91	1.77926	1.8048	1.91013	2.02265	2.12691	2.38919	2.01842	2.30334	0.59057	0.3264	1.00437	0.94833	1.41162	1.8859	1.73055	1.94647
CNOT4	2.73414	2.71088	2.61899	3.04147	1.86643	1.43954	2.56745	1.52755	2.10066	1.73985	1.96435	2.74227	2.08834	1.96034	2.33766	2.23901
NUP205	4.86504	4.46286	4.53298	4.46616	4.69404	3.34811	5.09062	3.43761	4.4228	3.44825	4.12679	3.87976	4.9295	4.60561	5.20373	4.83041
STMP1	4.13542	3.92001	4.35126	4.54549	3.06303	3.44517	3.48664	3.34414	2.98433	2.30529	2.77303	2.66069	3.78297	3.14569	3.13794	2.69501
SLC13A4	-1.22016	-1.51466	-0.78134	-1.09935	-2.86761	-1.90812	-2.87667	-1.75732	-2.23409	-2.86407	-2.31967	-1.34464	-2.44655	-2.28048	-2.52316	-1.93985
MTPN	6.46737	6.74038	6.53156	6.99417	5.93433	4.98246	6.62644	4.74303	6.53751	6.28052	6.164	6.64565	6.60133	6.66945	6.62379	6.57167
LUZP6	8.11034	8.18471	8.13515	8.92515	6.60728	6.83898	7.79669	6.79007	7.27177	7.01666	6.80534	7.96295	7.95491	8.08685	7.70449	7.55091
DGKI	0.84967	1.04324	0.79254	1.03356	-3.32193	-2.94031	-3.32193	-3.08546	-3.32193	-3.12734	-3.24919	-3.07185	-3.21329	-3.32193	-3.32193	-2.5718
CREB3L2	3.5381	3.33229	3.31103	3.5885	4.15625	3.46447	4.06843	3.25292	4.77751	4.36865	4.26738	4.67157	2.90618	2.74072	3.33874	3.35791
TRIM24	2.15672	2.43009	2.50663	2.52128	3.46464	3.12976	3.49237	3.2247	2.89642	2.63278	2.95655	3.15366	3.28563	3.39667	3.93023	4.00207
ATP6V0A4	-1.68207	-1.87432	-0.90759	-1.10681	-3.32193	-3.32193	-3.08364	-3.32193	-0.0743	-1.08604	-0.25594	-1.04985	2.26836	2.48851	2.5973	3.06368
TMEM213	0.77615	0.52542	0.20255	0.46726	1.90172	1.29148	1.71856	0.63939	-0.47686	-1.22923	-0.28927	-1.09886	0.66967	0.13023	1.29605	1.14317
KIAA1549	2.09279	1.57922	1.97554	1.90097	4.06536	2.50803	4.14948	2.31843	1.41283	0.75696	1.6028	1.5855	2.6563	1.35457	3.50713	2.7933
ZC3HAV1L	-2.407	-3.32193	-3.32193	-3.32193	3.40237	2.68072	3.60005	2.42384	4.35244	4.3123	4.0096	4.88577	3.16663	3.16154	2.82266	3.18376
ZC3HAV1	3.35893	3.78205	3.60791	4.08196	3.60076	3.37731	3.92314	3.51089	3.18664	2.92902	3.35947	4.31721	3.90973	3.84529	3.97626	3.86556
TTC26	2.02113	1.82086	1.6736	1.95706	1.50071	0.84424	1.87954	0.69085	1.81917	1.48275	1.91957	1.83733	2.75298	2.76858	2.30226	2.0672
UBN2	0.42327	0.74754	0.83011	1.34119	1.02229	0.53653	1.3774	0.82175	0.05858	0.2418	0.49578	1.2379	1.55152	1.47351	1.74673	2.1095
FMC1	4.40141	4.5356	4.34853	4.12584	3.92775	4.48513	3.62814	4.37961	3.32837	3.25971	3.56861	2.74719	4.58007	4.25572	3.8812	3.95014
LUC7L2	3.8606	3.97388	3.85051	4.00144	4.55403	3.93848	4.715	4.05321	4.30197	3.96777	4.38194	4.09854	4.25285	4.77765	4.70913	4.63521
CLEC2L	3.2662	2.56703	2.29367	2.12597	-2.59754	-2.37213	-2.66806	-2.53065	-3.32193	-3.32193	-3.32193	-3.32193	-3.32193	-3.01187	-3.32193	-3.32193
HIPK2	3.53658	3.04936	3.21254	2.96649	-2.89709	-3.11673	-2.96328	-2.94377	3.89679	3.15616	3.21934	3.39816	5.45083	3.61068	5.42288	4.80033
TBXAS1	-0.21156	-0.39523	-0.46817	-1.01429	-3.32193	-3.32193	-3.01894	-3.32193	-1.94311	-2.75356	-2.20615	-2.27367	1.52867	0.87157	0.41253	1.08939
PARP12	4.00714	4.05378	4.00551	3.87384	3.04165	2.81853	3.27311	2.68491	3.05241	3.09066	3.31288	3.77519	2.31495	2.2701	2.02617	2.3163
KDM7A	1.04182	2.05177	1.98951	2.13115	0.22603	0.05704	1.23797	-0.05671	0.13083	0.15817	-0.08301	1.41941	-0.08212	-0.96806	-0.02902	0.27307
KDM7A-DT	0.76375	2.09512	2.51145	2.11776	1.05693	1.39987	0.22081	1.0281	-0.50129	-0.65613	-1.89481	-1.40267	-1.29842	-2.13744	-1.96763	-1.94833
SLC37A3	1.89858	1.81665	1.91256	2.20946	3.29973	2.72403	3.59302	2.70996	1.99467	1.85885	1.66694	2.20473	2.43462	2.68711	2.51597	2.73697
MKRN1	4.29222	4.48407	4.33856	4.61216	4.42237	4.20092	4.60546	3.92597	5.01632	4.96474	4.93585	5.04693	4.82696	5.07299	5.09548	5.42786
DENND2A	3.59881	3.74055	3.96874	2.82473	2.74308	0.73988	2.10693	0.74674	4.15657	4.5124	4.3296	4.25938	4.73677	4.48254	5.48382	4.81161
ADCK2	4.08678	4.2388	4.28399	3.63346	5.98046	4.62298	5.20876	4.58652	4.57237	4.66129	4.4766	3.55072	3.94876	4.27962	4.52478	4.77886
NDUFB2	5.15022	5.17176	5.14041	5.00805	5.88946	5.68459	5.26671	5.56707	5.35388	5.2351	5.11255	3.988	5.53637	5.3747	4.64834	4.74169
NDUFB2-AS1	5.83622	5.8706	5.87626	5.72015	6.66714	6.19547	6.1274	5.94365	6.14842	5.87822	5.87392	4.54599	6.32818	6.20146	5.54897	5.6011
BRAF	1.2192	1.32688	1.35761	1.74695	2.3864	1.42518	2.75518	1.56304	1.69099	1.66517	1.76212	2.7209	2.46119	2.23608	2.60122	2.5627
MRPS33	3.40421	3.54292	3.31801	3.70496	3.65573	3.6686	3.49034	3.74014	2.92774	3.33813	3.13402	2.86255	4.38698	4.29567	3.34407	3.29297
TMEM178B	-1.27513	-1.43529	-1.41073	-1.12473	2.98941	1.43663	3.4056	1.23942	2.04014	2.43179	2.23806	3.53472	3.80558	3.91311	3.96413	3.81273
AGK	2.61683	2.31707	2.21735	2.61612	2.36311	2.55218	2.42715	2.64004	3.00168	2.48504	2.76142	2.74346	3.72395	3.4462	2.95229	2.5195
WEE2-AS1	-1.00378	-0.68484	-1.18071	-0.94501	-2.36535	-1.43933	-2.15809	-1.46017	-2.12829	-1.04556	-2.07295	-0.88643	-0.95327	-0.86365	-1.12185	-1.56591
SSBP1	5.85017	5.75662	5.56124	5.56381	5.30059	5.71001	5.49817	5.54308	5.10548	4.87274	4.98683	4.26318	5.18786	5.14212	4.68766	4.59133
MGAM	-3.32193	-3.10555	-3.32193	-2.9737	-3.07864	-3.32193	-2.57962	-3.32193	-1.85751	-2.0648	-1.65948	-2.82623	-3.32193	-3.16807	-3.32193	-3.22385

KEL	2.01328	2.06551	2.14929	2.05171	-3.20553	-3.05886	-3.32193	-3.32193	-3.32193	-3.32193	-3.05296	-3.32193	-3.32193	-3.32193	-3.32193	-3.06726
GSTK1	4.60581	4.53585	4.4422	4.64986	4.77991	5.27444	4.3689	4.82985	4.307	4.4592	4.56213	4.27919	4.10668	4.01342	3.75193	3.88122
TMEM139	0.42338	0.73366	1.23834	0.92296	-1.33758	-1.536	-1.89148	-2.53946	-1.68452	-1.58418	-1.96135	-2.03813	1.99608	2.46855	2.00055	1.93431
CASP2	2.09128	1.92335	2.00344	2.01649	2.40576	1.38119	2.40192	1.76211	2.48846	1.52183	1.84347	2.20303	3.48533	2.98254	3.39281	3.15712
ZYX	7.13193	7.12334	7.42213	6.28984	6.62211	6.02796	6.26169	5.70584	7.18537	6.95283	7.51369	6.50784	6.80868	5.73207	7.33477	6.37041
EPHA1	5.28951	5.2464	5.52802	4.4541	4.4477	4.14765	3.81819	3.86126	4.92538	4.38608	5.25688	4.19037	4.46639	3.32122	4.92797	3.93264
CTAGE15	-2.39591	-2.57909	-1.25194	-1.99141	1.60563	1.51617	2.49692	1.80319	-0.68252	-1.25764	-0.53429	-0.15789	1.3204	1.81528	1.83424	1.94389
TCAF1P1	3.40617	3.38979	3.43441	3.33121	4.22897	1.62685	4.00915	1.43595	3.66794	3.98423	4.12322	4.09394	3.83723	4.19915	4.89143	5.01661
TCAF2	0.40525	1.19912	1.38005	-0.14705	-3.0542	-2.02543	-2.7877	-2.5447	2.14571	2.24531	1.48454	1.53566	-1.61845	-2.23487	-0.81132	-1.348
TCAF2C	0.00507	1.15681	0.82943	-1.05829	-3.14828	-2.63575	-2.96451	-2.26906	2.49342	2.48257	1.41054	1.61791	-1.5737	-3.32193	-0.56685	-1.05267
CTAGE6	-2.40142	-2.58378	-1.03558	-1.61968	1.75423	1.78235	2.3107	2.07242	-0.46866	-0.76649	-0.68018	0.04402	1.25386	1.58683	1.75166	2.07855
TCAF2P1	0.76799	1.92833	1.42364	-0.2715	-2.82782	-2.34199	-2.94426	-2.22169	2.85886	2.99131	2.17534	2.00734	-1.06877	-2.32119	-0.1103	-0.59267
TCAF1	3.95529	4.11972	4.11349	4.57821	3.99256	3.54725	4.23376	3.88494	3.41872	3.62391	3.79298	4.30615	4.32875	4.68252	4.8731	4.90975
CTAGE4	-1.32231	-1.89808	-0.43534	-0.66654	2.59949	2.98049	3.25304	3.11448	0.05821	0.0763	0.72539	1.50042	2.58426	2.57354	2.80743	3.03162
ARHGEF35	2.26097	2.39509	2.30492	2.12835	5.08103	4.40012	5.26394	4.28009	3.00745	3.07776	3.03031	2.59265	3.43028	3.6423	3.98722	4.24879
OR2A42	-1.79434	-1.604	-1.40027	-1.48389	-2.47415	-1.86501	-3.12472	-1.32689	-2.5315	-1.54748	-2.22573	-1.03868	-1.48131	-1.69785	-1.27014	-0.55237
OR2A20P	1.60051	2.17946	1.73946	2.20451	0.68495	2.12366	0.71686	2.1549	2.26004	2.48717	2.35113	3.05927	1.82272	2.32846	1.5874	1.99023
OR2A7	-1.66022	-2.17502	-1.88412	-0.43948	1.37666	1.74215	1.56633	1.16659	-1.90141	-1.86835	-1.07823	-0.11943	-0.65101	-0.36645	-0.34252	0.69008
CTAGE8	-1.32231	-2.31487	-0.58108	-0.95332	2.59341	2.81631	3.08236	3.02248	0.23953	-0.09136	0.67012	1.34764	2.41027	2.55641	2.68431	2.92447
ARHGEF34P	1.29053	1.6582	1.35329	1.33419	4.91109	3.55283	4.866	3.3935	2.84528	2.44201	2.5953	2.78029	3.67565	3.44827	4.65617	4.76146
OR2A9P	1.59957	2.03353	1.68421	2.05374	1.0397	2.24262	1.03837	2.30483	2.19776	2.53521	2.26975	2.83906	1.84004	2.34334	1.65176	2.12663
OR2A1-AS1	2.22257	2.63784	2.73833	2.34816	3.61577	3.04995	3.84575	3.12171	2.74997	2.92185	2.9547	3.25019	3.38212	3.59552	3.88953	4.01463
OR2A1	-1.99538	-2.51271	-1.56401	-1.68259	-2.4878	-1.98581	-2.92927	-1.18837	-2.6581	-1.68944	-2.42052	-0.963	-1.18018	-1.57587	-1.42679	-0.60836
ARHGEF5	2.20443	2.42906	2.2941	2.42972	4.98987	3.85578	4.98146	3.69687	3.25913	2.8737	3.17871	3.18452	3.88388	3.62603	4.78382	4.77604
TPK1	-0.81382	-0.74829	-0.84307	-0.39301	-0.56925	-0.54454	0.12021	-1.11762	-1.62411	-0.85692	-1.64814	-0.97249	1.46348	1.8689	1.68528	0.81538
EEF1A1P10	0.40968	0.62426	-0.16197	0.7231	-1.67381	-2.67619	-1.69676	-2.10269	-1.03089	-0.42254	-1.16148	-2.70904	-1.01832	-3.32193	-2.70548	-1.65311
CUL1	5.38747	5.27688	5.26996	5.54065	5.95121	5.38435	6.27361	5.21124	5.14327	4.79024	5.03062	5.42575	5.46187	5.58955	5.51387	5.34172
EZH2	3.76996	3.74125	3.55723	3.95266	3.61657	3.46823	3.91145	3.47025	3.86232	2.63776	3.30436	2.73489	4.39661	4.27351	4.13084	3.6757
ZNF786	2.16658	1.83274	1.77274	1.8134	2.75292	2.24916	2.43119	2.54324	2.09239	1.94574	2.30517	2.28351	2.77953	2.72277	2.84611	3.11557
ZNF425	-1.68732	-0.92883	-0.8654	-0.19985	-1.07253	-1.04961	-1.66136	-0.46209	-1.75758	0.73378	-0.0733	0.81254	-0.46063	-0.60229	0.42874	0.54179
ZNF398	1.38854	1.02311	1.37641	1.34995	2.61702	1.6386	2.42993	1.94255	2.13287	1.94165	2.34794	2.47098	2.17587	1.98596	2.70653	2.73489
ZNF282	4.25229	4.37918	4.30917	3.69609	4.67395	4.15986	4.26406	4.28306	4.68579	4.97573	4.98728	4.60813	4.2161	4.13512	4.86706	4.7996
ZNF212	3.21273	3.10211	3.1036	2.87498	2.63019	2.49162	2.52265	2.34763	2.78748	2.60825	3.1111	2.46223	2.53465	2.59015	2.82501	2.80299
ZNF783	0.04917	-0.72473	0.32001	-0.55072	2.07608	2.40056	1.35985	2.06221	0.21362	1.24485	1.63245	0.85319	0.97251	1.13081	1.33556	1.83522
ZNF777	3.72851	3.4994	3.49897	2.69921	4.11778	4.04084	3.47572	4.08047	4.3396	4.14513	4.5378	3.6257	3.56736	3.17617	4.1718	3.9772
ZNF746	2.49184	2.35912	2.57148	1.92632	3.32202	2.65835	2.68341	2.78787	3.12386	2.82308	3.46858	2.22053	2.39915	1.41931	2.61553	2.21661
ZNF767P	0.11982	-0.01417	0.08756	-0.14258	1.22832	1.485	0.36937	1.44522	-0.64462	-0.17873	0.24144	0.10026	0.45739	0.51493	0.3791	0.3018
KRBA1	0.92846	1.07563	1.21567	0.77865	2.30693	2.3544	1.19249	2.53802	1.9833	2.5483	2.17441	1.94994	0.553	0.98048	1.32307	1.6688
ZNF467	-2.43313	-1.05185	-2.31549	-0.23011	3.33381	5.29566	2.21962	5.35717	1.00993	3.83867	2.46233	4.22672	-0.70954	0.9222	1.53188	2.78969
SSPO	-3.32193	-3.32193	-3.32193	-3.32193	-1.10534	0.25232	-1.81086	0.05474	-3.26165	-2.74126	-2.98145	-2.55983	-3.28103	-3.24153	-3.22419	-2.91879
ZNF862	-2.12725	-2.29806	-1.65224	-1.53922	-1.72106	-0.82258	-1.92531	-1.22258	-2.00104	-0.58167	-1.60428	-0.66109	-0.69125	-0.14409	-1.03871	0.31153
ATP6V0E2-AS1	2.54367	2.64178	2.26118	2.1814	3.80343	4.57007	2.86758	4.71384	3.00398	2.9943	2.64536	2.11284	3.28456	3.27673	3.28346	3.11839
ATP6V0E2	3.6254	3.82568	3.31365	3.20279	5.47854	5.55298	4.8608	5.56359	4.88547	4.94168	4.4187	4.31232	4.68049	4.53431	4.76827	4.72981
ACTR3C	3.18756	3.28816	3.25351	3.25991	2.34049	1.36628	2.14327	1.25482	-3.13334	-3.16378	-3.1527	-2.89788	0.39417	1.14419	1.19591	1.3964
LRRC61	6.41285	6.48493	6.36516	6.37247	5.13385	5.27923	4.46157	5.16907	-2.59101	-2.45726	-2.95012	-2.97822	3.197	4.19799	3.77904	4.26777
ZBED6CL	4.54596	4.71677	4.72431	5.38508	3.94127	3.69402	3.96685	3.47454	-3.32193	-3.32193	-3.32193	-2.99901	1.3006	2.01631	1.98748	2.63693
RARRES2	0.8455	0.50088	0.57346	0.28933	0.59171	-0.40815	0.00735	-1.36466	-3.32193	-3.32193	-3.32193	-3.32193	-0.57834	-1.0635	1.68906	0.31426
REPIN1	5.39585	5.20486	5.12555	4.44115	6.98231	6.38546	5.87741	6.46891	5.39614	5.36848	5.46033	4.5521	5.81573	5.98041	5.80689	6.14909
ZNF775	3.10868	3.59893	3.71912	2.82451	3.43443	3.7887	2.22859	3.72055	3.48321	3.75145	3.60109	2.53783	2.09796	2.53365	2.65852	2.54006
LINC00996	0.61351	0.66415	0.84328	1.81962	-3.32193	-3.32193	-3.32193	-3.32193	-3.32193	-1.87638	-3.32193	-2.57535	-3.32193	-3.32193	-3.32193	-3.32193

GIMAP2	1.28196	0.7825	0.78439	1.69172	-3.32193	-3.32193	-3.32193	-2.41087	1.39954	1.47338	1.64567	1.73463	-0.49634	-0.23244	-2.05083	-0.72345
NOS3	-1.23129	-0.01067	-0.14691	-0.36811	-1.68776	-1.45943	-2.09624	-1.13081	0.30634	1.06967	1.10629	-0.12701	-3.05376	-2.72845	-2.51717	-1.91378
ATG9B	-1.44549	-0.51511	-0.72845	-0.61728	-1.63587	-1.0206	-2.22866	-1.36259	-0.93382	-0.27571	-0.2263	-0.86516	-3.07063	-3.07145	-2.77223	-1.91098
ABCB8	3.05401	3.12922	3.21246	3.06518	3.83975	4.01006	3.28014	3.90126	3.00945	2.62677	3.13719	2.4528	2.68784	2.98076	3.3008	3.58021
ASIC3	-0.12178	0.07177	0.49052	-0.23661	1.57473	2.93872	0.58232	3.14856	-0.22591	-0.5163	0.09155	-0.78106	-1.13916	-0.47928	-1.33418	-0.96178
CDK5	4.31177	4.54692	4.48635	4.84865	4.47626	4.81752	4.30578	4.77963	3.49136	3.60578	3.69011	3.42535	2.94008	3.36855	3.21029	3.32181
FASTK	4.3313	4.28307	4.47792	4.02193	5.93952	6.41752	4.85715	6.33483	5.11454	5.1792	5.35189	4.39119	4.52951	4.51135	4.84533	4.78914
TMUB1	6.03639	6.11774	6.06036	5.46045	7.89204	8.48656	6.81158	8.4285	6.53306	6.43421	6.49706	5.21723	5.25967	5.52359	5.95343	5.99181
AGAP3	4.5891	4.42726	4.41584	3.72438	3.42971	3.75348	2.63395	3.72927	3.57487	3.39881	3.71008	2.78855	2.82102	2.89795	3.20079	3.37729
ABCF2	5.11327	4.66694	4.28468	4.48205	5.26379	4.55417	5.23304	4.56157	5.15598	4.47308	4.95716	4.95493	5.29564	4.94176	5.0195	4.9425
CHPF2	5.24764	5.29095	5.35336	5.36054	6.06787	6.10731	5.72723	5.94926	4.77327	5.05563	5.1938	5.14606	4.34446	4.30504	4.53839	5.24162
MIR671	8.05789	8.09407	7.98941	8.212	7.95834	9.17325	7.35236	9.18716	6.55667	6.92926	6.93953	6.85115	6.18497	6.28806	6.44936	6.99081
SMARCD3	1.55431	1.82876	1.61075	1.70781	2.41971	3.23489	1.55055	3.38838	1.40666	2.16717	1.61814	1.89669	1.87489	1.85136	1.88456	2.22053
NUB1	3.82923	3.85885	3.96168	4.11285	3.94508	3.37199	4.29922	3.09792	4.06656	4.03989	4.30655	4.38196	3.78403	3.45726	3.58666	3.39998
WDR86	1.50815	1.22668	1.58993	1.8239	1.92928	1.43306	1.90865	1.22469	2.11719	2.24083	2.37011	2.27214	0.78562	0.16191	0.64697	0.69334
RHEB	6.35975	6.20278	6.19615	6.41401	5.55466	5.49493	5.95687	5.32952	5.74937	5.24459	5.32415	4.66492	6.46074	5.92005	5.69816	4.94699
PRKAG2	2.66738	2.55516	2.51272	2.57138	2.10138	2.6486	2.58521	2.57579	3.9831	3.77753	3.70783	3.79909	4.62228	4.31817	3.71578	2.90575
PRKAG2-AS1	1.24426	2.07713	1.80721	1.2477	-0.78135	1.04035	-1.07778	0.45417	-1.28533	-2.12845	-1.16598	-1.50203	-1.4683	-1.96417	-3.32193	-3.32193
GALNT11	1.664	1.67244	1.49921	1.98638	2.90825	2.40425	3.13013	2.47328	1.58678	2.09673	2.39942	2.63285	1.95693	2.82577	2.30502	2.615
KMT2C	1.40839	1.62481	1.6716	2.02156	1.65973	0.67989	2.1611	1.07108	1.65734	1.80002	1.94764	2.87703	2.62104	2.51391	2.87777	2.8385
LINC01003	1.74668	1.5915	1.88621	1.97543	1.76869	2.67183	1.49087	2.04597	0.16713	1.03678	0.90887	-0.23315	1.54916	1.82156	1.43532	1.67418
XRCC2	3.21409	2.37792	2.39848	1.80396	3.17932	1.78539	3.48435	2.67292	2.28611	0.85742	1.65461	0.56525	3.7542	3.06	3.59163	2.43463
ACTR3B	1.37105	1.28845	0.99473	1.02537	1.50102	1.98398	1.17934	2.01375	-0.68004	-0.86971	-0.30868	-0.64971	0.10979	0.71265	-0.4113	0.00755
LINC01287	1.25854	1.03464	1.25949	1.60894	-2.58927	-3.32193	-3.32193	-3.32193	-3.32193	-3.32193	-3.0226	-3.32193	-2.89031	-3.32193	-3.32193	-3.32193
DPP6	-1.82129	-1.59495	-1.1666	-2.21432	-3.28731	-3.24085	-3.24691	-3.32193	-3.32193	-3.32193	-3.32193	-3.32193	-3.32193	-3.32193	-3.24525	-3.32193
PAXIP1-AS2	2.07838	2.0504	1.82042	2.25645	2.43831	2.59379	2.796	2.64291	1.83109	1.79582	1.9778	2.67443	2.22009	2.22137	1.77139	2.00788
PAXIP1	1.43279	1.13476	1.1792	1.36121	2.28245	1.39996	2.33011	1.63502	1.86618	1.64472	1.87849	2.17561	2.03258	2.12428	2.08208	1.71413
PAXIP1-AS1	1.18102	0.78389	1.30096	1.18364	2.37829	2.76833	2.06292	3.1797	0.23839	0.75904	-0.01309	-0.41068	2.29203	2.77417	2.14915	2.19065
INSIG1	3.95372	3.98222	4.01094	3.84335	5.49679	5.76286	4.83605	5.48834	7.22527	6.04083	6.31904	5.24703	4.7885	4.43822	5.55697	4.06001
EN2	-3.32193	-3.32193	-3.32193	-3.32193	0.63817	-0.61102	-0.51974	-1.14089	0.73792	-0.02653	0.13047	-0.5006	-2.88024	-2.88159	-2.79934	-2.78968
RBM33	2.03812	1.85167	1.88712	1.8802	2.8627	2.07423	2.90863	2.51517	2.83205	2.68265	2.70679	2.95379	2.7966	2.73566	3.1588	3.04228
SHH	0.25554	1.29371	1.29536	0.80987	-1.54759	-2.62323	-1.36736	-2.38371	-3.32193	-3.32193	-3.10831	-3.32193	1.39752	2.06655	1.757	3.18429
LINC01006	0.89696	1.73572	1.80183	1.45665	0.42115	0.5322	-0.06043	0.62652	1.47791	2.83257	2.18974	1.85862	1.79683	2.98094	2.22646	2.8147
RNF32	0.42539	1.00952	0.90075	0.92085	1.38887	1.1727	1.07416	1.2515	0.37525	1.06086	0.45333	0.53268	1.06297	1.70502	1.31634	1.93562
LMBR1	2.31778	2.56266	2.53862	2.81699	3.61683	2.45363	4.1644	2.38938	2.62662	2.58992	2.48279	3.28803	3.19609	2.98224	3.39163	3.33949
NOM1	3.10278	2.83956	2.77431	2.65009	2.60116	2.50642	2.62195	2.97306	3.07331	2.71532	3.25337	2.67198	3.20948	3.64751	3.42201	3.38642
MNX1	-3.32193	-3.32193	-3.32193	-3.32193	3.1206	4.56142	2.08918	4.76487	4.20937	3.95313	4.30243	2.30749	0.48781	1.46101	1.18208	0.76908
MNX1-AS2	-3.32193	-3.32193	-3.32193	-3.32193	0.50794	2.4183	1.27768	3.12745	0.09064	-1.73644	1.02488	-1.01901	-0.41445	-1.93238	-1.01084	-0.136
MNX1-AS1	-3.32193	-3.32193	-3.32193	-3.32193	2.3333	2.54323	1.30227	2.23609	2.2569	2.83705	2.50103	1.19599	0.5867	1.41187	0.96401	1.62433
UBE3C	4.0654	3.81688	3.85606	4.03386	5.0111	3.8732	5.26942	3.80394	4.91821	4.40301	4.82458	4.9207	5.15084	4.858	5.15246	4.88345
DNAJB6	4.51795	4.4207	4.16035	4.3083	3.89863	3.39054	4.01089	3.55865	5.04927	4.75446	4.55884	4.56644	4.47484	4.2908	4.00466	3.77873
PTPRN2	-2.23506	-1.93115	-2.50553	-1.72391	-3.05337	-2.91623	-3.12042	-3.32193	0.56685	0.81307	0.53357	0.14373	-3.32193	-3.32193	-3.11613	-3.1119
NCAPG2	3.74596	3.27223	3.46909	3.26395	4.01573	2.64213	4.21283	2.82541	4.4557	3.34283	3.86647	3.32	4.85997	4.96707	4.8546	4.50656
ESYT2	3.68976	4.08951	4.19484	4.22658	5.23921	4.33541	5.68078	4.16329	3.9792	4.47044	4.41616	4.97147	4.78782	5.26496	5.58047	5.22841
WDR60	2.10907	2.46872	2.75142	3.66445	2.34305	2.49965	2.41325	2.97595	1.97429	2.59415	2.28914	3.20753	2.42774	2.65066	2.99441	3.07114
LINC00689	-3.32193	-3.32193	-3.32193	-3.32193	0.21635	-2.24201	0.3001	-1.63594	-3.32193	-3.32193	-3.32193	-3.32193	-3.14205	-2.90965	-3.20968	-3.20729
VIPR2	-3.32193	-3.32193	-2.88666	-3.32193	-0.46413	-1.49224	-0.80669	-1.45338	-3.32193	-3.32193	-3.32193	-3.32193	-3.0199	-2.77192	-3.13049	-2.53517
PLCXD1	2.78286	2.9784	2.8947	2.1596	5.98155	5.99674	5.82741	5.82113	4.14826	4.91594	4.63156	5.0343	-0.9644	0.69321	0.86674	1.481
GTPBP6	5.70693	5.55659	5.33508	4.86081	5.63418	6.04141	4.7839	6.06337	4.89752	4.72855	5.00122	3.8049	4.56877	4.57976	4.83416	4.62986
LINC00685	3.71768	4.2189	4.21534	5.26039	2.73254	4.24675	1.86972	5.26648	0.65576	-1.15289	1.54488	0.99347	-2.04654	0.45841	0.57051	1.36594

PPP2R3B	3.00833	2.83485	2.84573	2.04309	1.78475	2.73056	0.79858	2.7627	1.758	1.23763	1.53183	0.21536	1.73291	2.12017	2.10166	2.07324
CRLF2	2.61439	2.98205	3.05111	2.16577	-0.28356	-1.76022	-0.34957	-2.77668	-1.83893	-1.62858	-3.32193	-2.02081	-2.17787	-3.32193	-3.32193	-2.51159
CSF2RA	3.31741	3.35352	3.18631	3.58749	1.63794	0.94372	1.64454	1.13548	3.03195	2.94511	2.63432	2.94982	2.10136	1.47166	1.42897	1.51033
IL3RA	1.29874	2.31919	1.75704	3.74955	-0.78115	0.4698	-1.33437	1.27579	-0.00455	1.44679	0.50234	1.82462	-2.54696	-0.92914	-2.12029	-1.28616
LINC00106	2.89563	2.74883	3.4558	3.22776	2.1695	2.50264	0.95281	3.16803	-0.49503	0.80196	0.07134	0.12997	0.96255	1.99603	0.78844	1.26476
ASMTL-AS1	4.33322	3.98421	4.26659	4.21111	4.07596	5.72317	2.86061	5.63055	3.70868	3.79607	3.93895	3.74225	2.73521	2.94092	2.74528	2.8349
ASMTL	4.20913	4.07651	4.06187	3.87927	4.5984	5.79546	3.91071	5.63533	4.38834	4.62842	4.54557	4.55582	3.1328	2.77754	3.44907	3.28954
P2RY8	1.43875	1.54262	0.60677	1.12924	-3.32193	-3.08806	-3.10473	-3.32193	-2.46652	-2.90435	-2.87766	-2.32914	-3.13751	-3.32193	-3.32193	-3.32193
AKAP17A	5.67001	5.49092	5.72212	5.05659	4.98766	6.23748	4.30211	6.32476	4.4238	4.31857	4.61625	3.89427	3.91178	3.85572	4.02537	4.15333
DHRSX	2.39698	2.52483	2.6135	2.32939	4.20822	4.13163	3.68798	4.13278	3.77156	3.44246	3.5339	3.35644	2.97453	2.4389	3.21263	3.0314
ZBED1	3.86085	4.04409	3.92439	3.87256	4.90313	4.34806	4.47196	4.37682	5.22275	4.61541	5.10821	4.52748	3.34534	3.467	3.93343	4.14184
CD99P1	0.14967	0.302	0.49001	0.76986	-1.0972	-0.20311	-1.061	0.07181	-0.34809	-0.72357	-0.43789	-0.71436	-0.70497	-1.09428	-1.07265	-1.43978
CD99	6.90538	7.3562	7.56039	7.33625	7.57377	7.82295	7.31469	7.66451	6.97157	6.9487	6.93333	6.10181	6.00698	6.02123	6.2072	6.13458
GYG2	1.2844	1.29839	1.12963	1.64576	4.7545	4.58212	4.49553	4.63232	0.01897	0.38748	0.06474	-0.77812	2.61011	3.03374	3.48671	3.24596
ARSD	0.63905	1.24345	1.43446	1.70651	1.12243	1.63869	1.30464	1.54106	1.54753	1.82753	2.17826	2.57807	2.69475	2.20395	3.41721	3.50296
ARSD-AS1	1.25261	1.67622	2.23878	2.58098	1.11112	2.23755	1.83289	2.20949	1.67606	1.91406	2.38504	3.70973	3.84119	3.20673	4.22619	4.49581
PRKX	-2.34936	-2.36208	-2.32367	-2.78035	2.43784	1.87325	2.8223	1.88967	3.26632	2.48497	3.1933	2.8466	3.45122	2.70554	3.91969	3.23941
RNU6-146P	-3.32193	-3.32193	-3.32193	-3.32193	3.99735	3.14062	4.63921	2.12886	4.27143	2.81347	4.53469	4.49707	5.03359	4.38243	5.4141	4.40181
NLGN4X	1.71425	2.02871	2.11307	2.00385	3.75916	3.05758	4.0643	2.82972	-3.32193	-3.18366	-3.1739	-3.1859	-3.32193	-2.52882	-3.18497	-2.93748
VCX3A	3.55119	3.75437	3.56513	3.62179	2.07308	3.17182	2.01141	3.31242	-0.85378	-2.51278	-1.54618	-2.52329	-3.32193	-3.32193	-3.32193	-3.32193
PUDP	2.532	2.75583	2.31501	2.40484	4.80173	4.25727	4.70224	4.03975	4.6644	4.228	4.28464	4.04207	4.83026	4.84638	4.74479	4.70131
STS	-0.52472	-0.43203	-0.97451	-0.06428	1.17206	0.03786	1.15183	-0.0536	-0.36239	0.34556	-0.27327	0.84192	-1.19868	-0.56481	-0.46398	0.16618
VCX	2.85518	2.5354	2.71138	3.36677	1.1935	3.15006	1.4741	3.24222	-2.43043	-3.32193	-1.98611	-2.06202	-3.32193	-3.32193	-3.32193	-3.32193
PNPLA4	-1.26614	-2.14191	-1.07673	-1.88448	-0.11432	1.07507	-0.22105	0.86151	2.58468	2.06659	2.92607	1.85919	1.60925	0.7065	2.41492	0.65509
VCX2	3.0178	2.94278	3.09787	3.1693	-1.72632	1.01841	-0.15197	1.49488	-3.32193	-3.32193	-1.74999	-2.39383	-3.32193	-3.32193	-3.32193	-3.32193
VCX3B	3.12977	3.36892	3.16072	3.55752	2.37228	3.3002	1.90216	3.66337	-1.59802	-2.13676	-2.56981	-3.32193	-3.32193	-3.32193	-3.32193	-3.32193
ANOS1	-3.17288	-3.32193	-3.14696	-3.32193	-3.25674	-3.32193	-3.18234	-3.32193	0.26378	-0.2249	-0.08067	0.08345	0.4366	-0.3279	0.25228	0.01922
TBL1X	1.47494	0.83015	1.32234	0.8745	5.05876	3.40774	5.33447	3.44798	4.51531	3.60098	4.22986	4.06598	3.57791	2.73385	4.33687	2.91351
GPR143	-2.62203	-3.32193	-2.86789	-2.48339	-1.87637	-0.65152	-1.58583	-0.01276	-1.4981	-0.33567	-1.13366	-0.87848	-3.00627	-3.32193	-2.94556	-3.32193
SHROOM2	-2.76242	-2.6099	-2.90318	-3.15892	0.80064	-0.39397	0.10153	-0.42975	3.18383	2.98573	3.4511	3.16008	-3.32193	-2.94801	-2.78356	-3.19434
WWC3	2.68674	2.83612	2.90308	2.38117	3.12172	2.61417	2.75922	2.75078	3.21035	4.13625	3.85107	4.4192	2.13722	3.01131	3.02525	3.49008
CLCN4	1.3091	1.36539	1.17214	1.50693	3.18612	2.36489	3.49202	2.46607	1.70012	1.55244	1.42317	2.05423	2.4359	2.20375	2.55164	2.85375
MID1	2.46661	2.26445	2.43092	2.66453	4.7981	5.06236	5.31481	4.94337	3.70352	3.45865	3.69241	4.49599	4.15921	4.09588	4.35478	4.05809
HCCS	5.32981	5.25374	4.87182	5.2884	5.93338	5.24313	6.0911	5.09835	5.38633	4.76807	5.04066	4.64163	5.65562	5.63248	5.16194	5.03115
ARHGAP6	-2.84299	-2.68744	-3.01949	-2.86483	-3.04809	-3.18505	-3.32193	-3.01841	-3.02465	-3.19111	-3.32193	-3.32193	-2.43578	-1.46893	-1.38863	-1.59715
MSL3	1.73891	1.79882	1.84079	2.21878	2.45115	2.27722	2.88194	1.86895	2.65711	2.28384	2.53442	2.76183	3.78302	4.10008	3.70438	3.35172
FRMPD4	-2.97184	-2.82521	-2.91554	-2.37742	-3.32193	-3.32193	-3.32193	-3.32193	-3.32193	-3.32193	-3.32193	-3.32193	1.29419	0.14749	-0.89697	-0.0358
PRPS2	5.09858	4.92853	4.83987	4.83749	4.361	3.48129	4.49898	3.25886	5.10485	4.23657	4.76614	4.51785	6.58928	5.63462	6.55914	5.49272
FAM9C	-3.14243	-3.32193	-2.76524	-3.32193	2.32546	1.62631	2.66289	1.72888	-3.32193	-3.32193	-3.32193	-3.32193	-3.32193	-3.32193	-3.32193	-3.32193
LINC02154	4.00012	4.58773	4.78762	4.85716	-1.78323	-1.72931	-2.37289	-1.13374	5.41373	6.24607	5.84085	6.57397	4.17503	3.4486	3.40841	3.0735
LINC01203	-3.32193	-3.32193	-2.32389	-1.67792	0.35589	3.67597	0.1654	3.03736	-3.32193	-3.32193	-3.32193	-3.32193	-3.32193	-3.32193	-3.32193	-3.32193
GPX1P1	2.15526	3.38543	2.69437	2.15877	1.28623	3.06823	1.00919	2.73341	2.56213	3.2269	2.32452	1.40581	6.0795	6.60406	6.2416	6.35028
TCEANC	-2.193	-2.20708	-1.68533	-1.70825	-0.74225	-0.61033	-0.61269	-0.51456	-1.4792	-0.99826	-1.19053	-0.3939	-0.94772	-0.82805	-1.13879	-0.9041
RAB9A	4.27338	4.43093	4.46382	4.82366	4.39878	4.57585	4.89841	4.59448	3.35576	3.52525	3.32851	3.98201	4.44837	4.34716	4.09028	3.79363
TRAPPC2	1.32305	1.7266	1.82083	1.90993	0.76811	1.6174	1.49395	2.05348	0.12397	0.88944	1.01589	1.83583	1.54481	2.46124	1.70255	1.81169
OFD1	2.14952	1.88924	2.14987	2.59202	3.19376	3.99457	3.5551	3.95782	2.23948	2.60319	2.57922	3.47019	2.62988	2.52558	2.52228	2.44106
GEMIN8	1.34107	1.79831	1.53954	1.96327	2.8693	3.46069	2.83862	3.31664	2.1309	2.35099	2.50395	2.50715	2.45761	2.37125	2.21873	2.17172
FANCB	1.51199	0.93765	1.22082	0.18416	2.08825	0.69001	2.3255	0.35303	1.5109	0.13879	0.45987	-0.26478	2.65177	2.11146	1.91253	1.28228
MOSPD2	1.4871	1.28751	1.68035	1.92868	2.4888	2.08218	3.36039	1.69317	2.11328	1.5452	1.98788	2.39269	3.28595	2.29877	2.85602	1.82979
ASB9	2.08354	1.90675	1.75215	1.79015	3.80956	3.15384	3.77418	3.12657	1.52954	2.02113	1.84983	1.81764	3.5866	2.70832	3.18087	2.4805

PIGA	1.61311	1.75701	1.69691	2.04075	2.67793	2.22679	3.43971	2.01025	2.2745	1.9461	1.46384	2.27771	2.62971	2.76958	1.99287	2.22193
PIR	3.55055	3.31952	3.27653	3.7102	5.84636	5.71904	5.67576	5.70929	2.6418	2.45749	2.53536	2.76709	4.59165	4.32527	4.14894	4.01797
CLTRN	-2.33375	-3.32193	-3.32193	-2.62514	-1.60045	-1.15811	-2.0726	-2.20424	-3.32193	-2.76192	-3.32193	-2.05963	3.00676	4.17235	3.13542	2.45303
CA5BP1	3.93305	3.58895	3.42615	3.15138	4.45266	5.13277	4.11536	5.20204	3.83344	3.11679	3.52954	2.93431	2.51817	2.85652	2.72035	2.69651
CA5B	0.70521	0.73495	0.74472	0.73462	1.25019	0.90178	1.48748	1.12452	0.87695	0.73429	0.45834	0.98738	0.96754	0.91992	1.54922	1.52283
INE2	0.37967	0.33584	0.55958	0.5961	-0.75512	0.72188	0.03957	0.82768	-0.90711	-0.78777	-0.6044	0.35371	-0.18656	-0.36043	0.84821	-0.07593
ZRSR2	3.50432	3.48188	3.35584	3.91205	2.98043	4.2035	3.14079	4.31633	2.48958	2.68748	2.96409	2.77201	2.58116	2.48882	2.11953	2.3698
AP1S2	2.73561	3.05429	3.03217	3.64438	1.91924	1.65027	2.50353	1.82271	2.41242	2.26597	1.31911	3.12828	4.77937	4.58175	3.95261	4.03574
GRPR	-0.68258	0.06811	0.31498	0.28383	-3.32193	-2.82951	-3.32193	-3.32193	-3.32193	-3.32193	-3.32193	-2.85578	1.58492	2.19894	0.90075	1.731
MAGEB17	1.77576	1.99758	2.30784	1.62297	-1.92311	-2.34066	-1.83712	-2.22026	-3.32193	-3.32193	-3.32193	-3.32193	-2.51058	-1.79565	-3.32193	-2.76598
CTPS2	1.84529	1.69169	1.75308	2.09117	4.12503	3.82484	4.46233	3.57629	2.84257	2.63512	2.58379	2.93695	3.63915	3.40912	3.95404	4.08808
SYAP1	3.99473	4.08157	3.86351	4.18872	5.66037	6.35013	5.96187	6.12579	4.49041	4.06225	4.2087	4.63921	4.54645	4.41551	4.56396	4.42277
TXLNG	3.54716	3.52439	3.2943	3.84812	4.26061	3.83248	4.72946	3.8399	3.14043	3.19294	3.01221	3.6521	4.26508	4.48229	4.09423	3.93213
RBBP7	5.11319	4.97864	4.99014	5.16753	5.42587	5.01335	5.51141	5.01545	5.5561	4.90533	5.03495	4.66742	5.66683	5.52482	5.23271	5.14098
REPS2	-0.39836	-0.69899	0.08303	-0.44942	-2.53237	-3.32193	-2.25923	-3.1038	-0.73104	-1.2148	-0.86823	-1.00928	-2.02831	-2.7543	-2.15539	-2.70305
NHS	0.53709	0.7527	0.85529	0.92012	-3.02688	-3.11485	-3.32193	-3.19656	0.36612	0.98325	0.36299	1.49608	-2.54155	-2.70249	-1.78848	-1.92209
SCML1	2.22047	1.38065	1.56576	1.98183	3.55176	3.51173	3.8624	3.43708	0.48181	-0.19092	-0.05446	-0.23524	3.63485	2.8476	2.32776	1.76256
SCML2	0.96095	0.44816	0.33283	0.04608	3.16131	1.83701	3.62102	1.97952	2.8532	0.65805	2.08783	0.77443	4.60305	2.97103	3.93354	2.51659
CDKL5	-2.10895	-1.76677	-1.96486	-1.76075	0.19698	-0.702	1.26535	-0.81008	0.38944	0.19856	0.4995	0.88709	-0.03505	-0.4579	0.8066	0.34094
PHKA2-AS1	4.04063	3.60683	3.8417	3.67155	5.23262	6.50069	4.56703	6.44052	3.02702	2.84433	3.02505	2.95867	3.26985	3.44816	3.4719	3.1398
PHKA2	2.30937	2.09028	2.12664	1.86297	4.13733	4.5928	3.65638	4.49845	1.82255	1.75097	2.04749	2.10321	2.22836	2.28023	2.59302	2.49905
ADGRG2	-1.6026	-1.4846	-1.83089	-1.7712	-2.43914	-2.62755	-2.60642	-2.38908	-3.08599	-2.9492	-3.212	-3.22101	-2.64498	-2.44594	-2.35949	-2.34404
PDHA1	5.40554	5.1593	5.13151	5.33624	5.70788	6.11727	5.70903	6.10703	5.5185	5.1969	5.40488	5.08597	5.69288	5.82207	5.42172	5.378
MAP3K15	1.60335	1.48758	1.73001	1.8045	2.32797	2.83883	2.64657	2.9852	1.81376	1.64227	1.70465	2.48339	2.58031	2.92005	2.48663	2.55814
SH3KBP1	5.05192	5.15535	5.1682	5.65567	3.54415	3.45789	3.89141	3.14342	5.93593	5.81329	5.94544	6.49402	4.1353	3.52282	4.3868	4.06649
BCLAF3	-0.15135	-0.03182	0.58087	0.78534	0.45214	0.82626	0.8713	0.76027	0.50297	0.99943	0.55535	1.80102	0.85765	0.8072	0.86857	1.21981
MAP7D2	-1.38167	-0.32782	0.65154	-0.64983	5.13323	2.64403	5.04781	3.06874	-3.05151	-2.89749	-2.87043	-2.90372	3.44392	3.19889	2.92306	3.0234
EIF1AX	5.36988	5.2657	4.99731	5.30035	6.01335	5.4297	6.6496	5.50433	5.65222	5.10156	5.2321	5.21834	6.399	6.13477	6.07011	5.78586
RPS6KA3	3.23817	3.44679	3.30916	3.67373	6.17886	5.41785	6.78559	5.27771	3.60726	3.62204	3.13118	4.23967	4.54276	5.18407	4.51354	5.11899
CNKSR2	-3.32193	-3.32193	-3.14762	-3.32193	-1.18223	-1.75013	-1.50244	-2.49527	-0.63984	-0.50174	-1.83822	-0.4603	1.96512	1.72554	2.16941	1.57909
MBTPS2	2.0169	2.01519	1.86522	2.1597	4.40639	2.91055	4.67479	3.02594	2.918	2.95344	2.47221	3.3925	3.75317	3.52976	3.58734	3.50031
YY2	1.35808	1.53443	1.5449	1.25562	1.46972	1.3247	1.41347	2.03834	1.3974	1.02758	0.80127	1.07943	0.85446	0.3714	1.3254	0.42592
PHEX	-1.1831	-1.22604	-2.12264	-1.04786	-1.52352	-1.25295	-1.82844	-0.47037	-1.33369	-1.00407	-0.85363	0.27071	-0.18279	1.42298	0.58925	1.29222
DDX53	-1.24879	-0.12634	-0.84666	-0.17959	-2.38964	-2.26453	-2.32326	-1.05421	-3.32193	-3.32193	-3.32193	-3.32193	-3.09845	-2.09813	-2.46056	-3.32193
FAM3C2	4.58696	4.49093	4.51844	4.95572	5.04076	4.58211	5.76998	4.24352	4.75444	4.77373	4.56759	5.67512	4.30326	4.36195	3.94882	4.51022
PRDX4	5.87269	5.79576	5.86045	5.44909	8.0054	8.1797	7.72946	7.76969	6.63807	6.488	6.33472	5.68093	6.67968	6.33965	6.24996	6.05939
ACOT9	4.02237	3.93307	4.23654	3.93944	4.86442	4.67917	5.24094	4.60199	4.90393	4.34242	4.92627	4.6782	5.22892	4.75849	5.07023	4.49053
SAT1	4.51956	4.78188	4.79025	5.65599	6.00161	8.80187	6.89244	8.21357	4.43215	5.67758	4.972	5.81652	6.04366	6.72717	4.48206	6.10761
APOO	4.48739	4.41369	4.23104	4.44035	4.39718	4.89955	4.31227	4.79077	3.52191	3.08695	3.09422	3.00199	4.22737	4.22026	3.17188	3.41818
RPL9P7	6.0342	6.09038	5.95251	5.72261	4.76297	5.3891	4.50515	5.37992	5.64467	6.20767	5.66777	4.82724	6.31973	6.05834	5.12311	4.99831
CXorf58	1.75093	1.80487	1.80379	1.58552	2.7499	2.39974	2.34135	1.95918	1.34308	1.03134	1.01526	0.07797	2.40918	2.18199	1.24557	1.71674
KLHL15	1.46493	1.55256	1.06612	2.0517	3.32287	2.50591	3.84798	2.64437	2.28549	2.47641	1.98029	3.01591	2.85114	3.42172	2.83859	3.33782
ZFX	1.57805	1.60866	1.41361	2.17397	3.23282	2.44827	3.71182	2.80424	2.18974	1.87554	2.018	2.9164	2.54578	2.05756	2.83663	2.51482
PDK3	2.58586	2.73589	2.26553	2.73992	3.29445	3.46573	3.47877	3.09192	2.99998	2.64091	2.21247	2.85854	2.17698	2.10567	2.51326	1.95079
POLA1	2.7734	2.72015	2.36056	2.62334	3.70353	2.30525	4.01887	2.51698	3.61749	2.66105	2.97563	2.87698	3.57682	3.59702	3.79217	3.48761
EEF1B2P3	3.8141	3.4622	3.50542	2.84418	3.04747	3.29604	2.55338	2.57401	3.95742	4.20351	3.79243	2.92368	2.13903	2.16308	1.84694	2.08176
PPP4R3C	0.49956	0.95329	0.64926	1.49696	-2.81491	-1.14919	-2.37667	-1.34068	-3.32193	-3.32193	-3.32193	-3.32193	-3.32193	-3.32193	-3.32193	-3.32193
DCAF8L2	-1.21273	-0.58246	-0.962	-1.14446	-3.21881	-3.08757	-3.32193	-2.62373	1.83487	2.18301	1.91401	2.04795	-3.32193	-3.13774	-3.09967	-3.32193
IL1RAPL1	1.55353	2.09757	2.18174	2.06587	-3.32193	-3.32193	-3.32193	-3.32193	1.93122	3.19202	2.45162	3.85421	1.19417	2.43794	1.47863	2.72791
MAGEB2	6.7463	6.82252	6.91687	7.30896	-0.18772	-0.0331	0.85592	0.99431	-2.68008	-2.77117	-2.73756	-2.77893	-3.32193	-3.32193	-3.32193	-2.76569

NR0B1	3.42486	3.37038	3.64823	2.3948	1.90721	-1.24954	1.00529	-1.50874	-3.32193	-3.32193	-3.32193	-3.32193	-0.61704	-0.74895	-1.72826	-0.54697
FTLP2	2.26417	2.07883	2.71004	-0.24767	3.02738	4.36524	3.1055	3.51907	2.2367	3.05679	2.85936	3.10438	1.33246	0.44597	1.22987	1.56993
GK	3.55563	3.87428	3.52266	4.53778	3.96234	4.03927	4.17415	3.6501	0.83462	0.44031	0.22059	0.19337	2.3059	1.87679	1.37234	1.44074
GK-AS1	4.61004	4.71849	4.58316	5.34289	4.66194	5.25515	4.95592	4.71881	1.29619	1.26259	0.66179	-0.16628	2.80974	2.10032	1.90397	1.93469
TAB3	3.11037	2.89528	2.96955	3.46034	2.00006	1.6034	2.62526	1.7173	1.77463	1.64992	2.01234	2.82862	2.03697	1.75063	2.49223	2.16142
TAB3-AS1	1.56123	1.15922	1.23377	2.94453	0.98809	1.0156	1.86726	-0.22841	0.95686	-1.30232	0.11703	-1.32081	1.50078	1.05544	1.64362	1.80757
TAB3-AS2	4.05131	3.99199	3.86642	4.21371	3.90696	2.71143	4.63868	1.39458	4.19598	3.82749	4.00631	5.3004	4.18714	3.786	4.40345	4.25056
DMD	0.74859	0.85525	0.76118	1.26643	-3.04791	-2.8641	-3.11623	-3.18753	0.12736	0.42517	0.57151	1.18339	-2.52322	-2.23692	-2.28067	-2.53873
TBCAP1	1.55981	2.50953	2.58616	1.76379	0.59572	1.45402	1.67883	1.91453	1.36901	2.29464	1.60905	0.53607	0.82659	0.96555	-0.85409	-1.59241
TMEM47	-2.66718	-1.66713	-2.39044	-0.55632	-2.11971	-2.66137	-1.75354	-1.71342	0.91091	1.54821	0.94455	2.07096	2.98717	3.86164	3.98362	3.55326
PRRG1	2.02451	2.07587	1.81641	2.6551	1.64841	1.3145	2.27031	1.39199	3.45809	3.32804	3.20882	3.77443	3.57248	3.7882	2.95862	3.24233
LANCL3	-3.32193	-2.99565	-3.32193	-3.19081	-3.32193	-3.32193	-3.32193	-2.97832	-0.94205	-1.58831	-1.33394	-1.84365	-3.32193	-3.32193	-3.32193	-3.32193
XK	0.0555	-0.50966	-0.3749	-0.39839	1.08391	1.19259	1.41171	0.99807	-0.43214	-0.56696	-1.08726	-1.14139	-0.1426	0.26975	-0.35252	-0.27331
DYNLT3	2.98988	3.72511	3.91993	4.35894	3.392	3.8783	4.62266	3.68681	2.48367	2.82786	3.0966	3.81471	3.0707	3.34209	3.65149	2.8012
SYTL5	-3.32193	-3.32193	-3.32193	-3.32193	1.14546	1.88884	2.36472	1.8037	-3.32193	-3.11252	-3.32193	-3.32193	-3.32193	-3.15013	-3.32193	-3.32193
SRPX	-0.18246	-0.48646	-1.23811	-0.8789	2.3249	2.27474	2.56891	2.17426	2.64953	2.75847	2.61567	3.0176	3.32843	3.42513	3.04737	3.85121
RPGR	1.9484	1.74682	1.84853	2.49958	1.55029	1.39327	2.00537	1.44545	0.93057	0.70463	0.56996	1.17829	1.43096	1.60159	0.62477	1.39735
OTC	-3.32193	-3.32193	-3.32193	-3.32193	2.40007	-0.63353	2.20947	-1.15377	-3.32193	-3.32193	-3.32193	-3.32193	-3.32193	-3.32193	-3.32193	-3.32193
TSPAN7	-3.00051	-2.96567	-2.19587	-2.36207	5.74366	5.6977	5.66975	5.77154	-3.32193	-3.32193	-2.99019	-3.32193	-2.65605	-2.65796	-1.78284	-3.32193
MID1IP1	5.21111	5.10983	5.07681	4.7747	5.10367	5.37322	4.56142	5.03719	5.63187	5.47673	5.58053	4.70995	4.36591	4.48545	4.14379	4.30773
MID1IP1-AS1	3.2623	3.1915	3.34004	3.12087	2.64076	2.02337	1.48412	2.01013	3.34724	4.17105	3.85068	2.62436	3.21995	3.83591	3.06646	3.34648
GAPDHP1	4.2139	4.13825	4.5333	2.75046	3.08126	4.91462	2.71978	4.18548	5.56152	6.23626	5.09847	4.65178	1.43378	0.78886	1.77312	2.05989
BCOR	2.58822	2.46575	2.48247	2.59731	3.63933	2.37522	3.60075	2.31091	4.12585	3.86413	3.76593	4.32448	2.92345	2.82328	3.12588	3.1325
ATP6AP2	4.15563	4.42473	4.37838	4.96937	4.55973	4.82418	4.98713	4.80867	4.17605	4.38384	4.18576	4.63076	4.47755	4.60645	3.65297	3.91848
CXorf38	2.08692	1.87775	2.04158	2.16273	1.94794	2.17475	1.99865	1.77828	3.81945	3.47861	3.69959	3.7011	2.27474	2.69745	2.19399	2.09121
MED14	2.98416	2.99563	3.02618	3.38259	3.12593	2.61398	3.56764	2.42942	3.85117	3.39571	3.61256	4.33634	4.09501	3.98175	4.11505	3.76709
MED14OS	1.11747	1.27679	1.40824	1.11664	2.4838	1.01306	2.53983	-0.97064	3.20501	2.68255	3.14646	2.85047	3.24652	3.55411	4.04303	3.61096
MKRN4P	-1.98233	-2.22064	-3.32193	-0.70056	2.76938	1.87178	2.97523	1.7816	-3.32193	-2.33344	-1.95131	-2.34559	-2.1849	-3.32193	-3.32193	-2.73893
RPS2P55	4.04759	3.94406	4.34353	2.37966	3.22617	3.41622	2.20555	3.09897	4.34546	5.0695	3.82918	2.54829	2.6389	2.09946	3.39313	2.6887
USP9X	4.07362	4.2445	4.18514	4.64697	4.71418	3.91386	5.17144	3.92297	4.83799	4.77329	4.764	5.82453	4.91758	5.20531	5.55542	5.85196
CASK	1.56203	1.75545	1.72845	1.98953	3.17898	2.12534	3.66502	1.9359	4.13647	4.19015	4.43064	4.86916	2.52011	3.36651	3.47887	3.41411
CASK-AS1	3.16856	3.35741	3.2841	3.95513	3.79295	3.40785	4.81218	3.34168	4.28865	4.35369	4.62121	5.63857	4.02458	4.87742	4.59267	4.46027
IMPDH1P4	1.08664	1.49866	1.21276	0.76279	-1.99761	-3.32193	-2.3625	-3.32193	-2.65464	-1.34839	-2.71394	-3.32193	-3.32193	-3.32193	-3.32193	-3.32193
MAOA	2.75893	3.06848	2.73412	3.0786	2.32577	3.48186	2.80329	3.23288	1.08651	0.95698	1.28785	1.23225	-1.37341	-1.08119	-1.44187	-0.96852
MAOB	-3.32193	-3.32193	-3.32193	-3.32193	-0.55325	-1.78586	-0.14897	-1.62809	-3.32193	-2.98979	-3.32193	-3.32193	0.17705	2.66089	3.80919	3.45578
EFHC2	-3.01877	-2.2864	-2.44921	-1.85141	-1.89522	-1.91321	-1.65164	-2.68384	-2.26034	-1.34688	-2.53388	-0.26613	-3.07924	-1.82344	-2.24038	-1.60736
FUNDC1	3.87135	4.15755	3.53628	4.4236	4.46176	4.66659	4.72847	4.64248	3.94324	4.10591	3.65862	4.02198	4.02396	4.32658	3.19492	3.49216
RPSAP61	0.21507	-1.00907	0.19818	-0.87206	-1.19139	0.22959	-3.32193	-1.26736	1.10767	1.81131	1.01652	0.44041	-2.56275	-2.56486	-0.93793	-2.42459
KDM6A	2.11405	2.06618	2.13388	2.45776	2.48876	1.59114	2.85277	1.25625	2.52637	2.20738	2.56622	2.92655	2.62191	2.11882	2.41169	2.30076
LINC01204	0.79728	0.99131	0.80173	0.68597	-3.32193	-3.32193	-3.32193	-3.32193	-3.09356	-3.13001	-3.32193	-2.96606	-2.89241	-3.02284	-3.32193	-2.53956
MIR222HG	2.33928	1.93441	2.33696	2.51825	0.19097	0.90698	0.05168	0.7579	0.14296	0.16501	0.75516	0.30568	1.23189	1.22696	-2.27629	-1.42681
MIR221	3.67809	4.42506	4.87561	4.88231	1.63067	2.13102	-3.32193	2.5788	1.35634	2.06428	2.57225	1.78424	2.49252	2.33831	-3.32193	0.56274
LINC02595	2.91663	2.24671	1.71702	2.54795	1.81888	1.69549	1.75883	2.64482	0.8351	0.9231	-0.06249	0.11238	0.91347	2.20908	0.36245	0.9394
LINC01186	3.27001	2.09583	2.3066	1.75992	2.2105	0.89138	2.09297	1.20912	2.19138	1.17119	1.27014	-0.31258	2.70279	0.72119	1.15748	-1.11102
GAPDHP65	3.273	3.02735	3.56062	0.45008	1.39119	3.56078	-0.41864	3.03123	3.76035	4.53834	3.26464	3.08929	0.09363	-1.31787	-0.53687	0.20667
KRBOX4	2.25454	1.89359	1.88485	2.37549	1.70497	2.30387	1.70714	2.48848	1.98323	2.06598	1.82949	2.24584	2.86828	2.79759	2.02673	2.2976
ZNF674	-0.03528	-0.36942	-0.05927	0.19217	-0.04965	0.42197	0.34247	0.82112	0.4492	0.26056	0.26512	0.34967	1.19277	0.86319	1.14693	0.30513
ZNF674-AS1	1.95319	1.56103	1.18154	1.91751	1.1746	1.41569	1.38414	1.44414	0.57456	0.19467	0.61503	0.3022	1.62507	1.71283	0.8573	0.60547
CHST7	4.0483	4.15352	3.82369	3.83471	0.8241	2.76962	0.41613	2.65778	4.66015	4.66156	4.90648	3.88336	5.23294	5.4406	6.15162	6.41954
SLC9A7	2.3628	2.05477	2.18132	2.33381	2.69458	1.71857	3.05486	1.74722	4.0342	4.15694	4.45109	5.89518	4.41108	3.83155	4.88996	4.87094

RP2	3.27677	3.61356	3.51595	4.02497	4.17187	2.81838	5.25477	3.02836	3.78279	2.99846	3.03885	3.6215	4.45759	4.36483	3.8146	4.00292
JADE3	1.74344	1.66419	1.61884	1.73263	3.22539	2.03103	3.59038	2.10602	2.91572	2.35792	2.51177	2.80513	3.36333	2.43178	3.41984	2.91564
RGN	-2.68761	-2.93247	-3.32193	-2.28937	2.81356	3.37477	2.40763	3.58747	-3.32193	-3.32193	-3.32193	-2.71455	2.30604	2.445	2.37508	2.61716
NDUFB11	6.76482	6.65607	6.68933	6.5599	7.0479	8.05021	6.58645	7.79248	7.40206	7.09033	7.29713	6.60029	6.94093	6.85927	6.76268	6.86335
RBM10	5.30578	5.00491	5.01145	4.51251	5.10084	5.17059	4.41525	5.129	5.86889	5.33069	5.84423	5.18458	4.95028	4.86026	5.326	5.23972
INE1	0.73158	1.20937	1.44737	1.53573	0.93644	1.41911	0.77393	2.34538	-0.79256	0.36508	0.4607	0.42839	-0.42775	0.46703	0.98689	1.36992
CDK16	5.3424	5.17707	5.19723	4.78963	5.64438	5.61135	4.94141	5.62554	6.15078	5.64159	5.76616	4.94106	5.12017	5.5588	5.577	5.54462
USP11	4.25229	4.6236	4.5156	4.70575	4.08775	4.84046	3.70773	4.75826	5.33948	5.05229	5.22354	5.05711	3.90113	4.68606	4.89802	5.0508
ZNF41	1.32253	1.38563	1.09581	1.53893	1.34431	0.51464	0.71777	0.76687	0.68579	1.10391	0.21262	1.74204	1.95884	1.89609	1.17184	1.78924
LINC01560	1.46648	1.56093	1.00573	1.7588	0.57942	1.07318	1.04672	1.77201	-0.28635	-0.1902	-0.43115	0.28176	0.92256	1.56211	0.95105	1.72406
NUS1P1	1.66896	1.70511	1.49083	1.63195	3.48054	1.72374	3.5154	0.32342	2.23349	2.03144	2.75585	3.59841	2.2172	0.55959	2.71569	1.16787
ARAF	4.55351	4.49345	4.45439	4.22906	5.23028	4.81892	4.70182	4.60488	5.49642	5.10424	5.41463	4.84705	4.78318	4.56789	5.2856	5.06064
SYN1	0.15748	0.11112	-0.28326	-0.004	-0.13497	0.89973	-0.22061	1.02307	-1.13906	-0.02742	-0.88426	-0.1693	-0.83301	-0.35542	-0.63743	-0.52596
TIMP1	6.88884	7.66804	7.85036	8.22243	6.68811	7.83822	6.46887	7.1608	7.58804	7.8796	7.74964	7.44602	6.09593	6.08495	5.57024	5.9537
CFP	1.48965	2.19598	2.23765	2.51957	-2.95074	-2.14731	-3.14691	-2.45662	-2.75456	-1.94404	-2.54314	-2.48525	-2.26362	-1.99419	-1.88099	-2.00971
ELK1	5.35621	4.8096	5.10369	4.47537	5.13656	4.30565	4.52878	3.89781	5.29302	4.32903	5.37282	3.66451	4.60669	3.11114	4.78196	3.59593
UXT	5.59031	5.6935	5.59486	5.37442	5.65853	6.15478	5.51342	5.99258	5.55412	5.62679	5.47694	5.12291	5.44582	5.58098	4.83169	5.04454
UXT-AS1	4.42288	4.68359	4.68653	4.22349	4.79108	4.87305	4.66262	4.68216	4.55906	4.54545	4.49704	2.91246	4.18924	4.29779	3.85204	4.12951
ZNF81	-0.55825	-0.09583	-0.49437	-0.16956	0.1722	-0.1681	0.28153	0.28123	-0.116	-0.04588	-0.00702	0.94862	0.61648	1.11549	0.40047	1.3949
ZNF182	1.28499	1.06448	0.79956	1.62166	1.38333	1.52523	1.58898	1.53861	1.35879	1.79043	1.21353	2.46875	2.07694	2.09491	1.96387	2.35764
ZNF630	-1.26292	-0.53024	-1.23388	-0.0298	-0.30689	-1.17935	-0.57448	-0.79303	0.40823	0.68401	0.17374	0.66095	-0.55868	-0.59863	0.06684	0.09541
SSX1	7.80753	7.48724	7.46053	7.56366	-1.84808	-1.31373	-1.62925	-0.58002	-3.32193	-3.32193	-3.32193	-3.32193	-3.32193	-3.32193	-3.32193	-3.32193
SSX4	-0.5843	-1.38522	-2.5176	-3.32193	0.87898	1.1799	0.75506	0.98056	-1.64532	-3.32193	-3.32193	-3.32193	-3.32193	-3.32193	-3.32193	-3.32193
SSX4B	-0.72744	-3.32193	-1.62637	-3.32193	0.92036	1.05278	0.59578	0.92471	-1.64532	-3.32193	-3.32193	-3.32193	-3.32193	-3.32193	-3.32193	-3.32193
SLC38A5	-2.08713	-0.73902	-0.11302	-1.6453	-2.27402	-2.6561	-2.03276	-2.77601	-1.43559	-1.13842	-2.83588	-1.72916	-3.32193	-3.11912	-3.32193	-2.86001
FTSJ1	5.25563	5.168	5.26988	4.51871	4.73463	4.86649	4.4475	4.73951	6.14709	5.45374	5.84733	5.00092	5.03697	5.02063	4.89208	4.81148
PORCN	3.35837	3.71995	3.95051	3.32079	2.58247	3.2766	1.22505	3.24977	4.78682	4.41124	4.55406	4.06739	0.74426	0.85627	0.51494	1.81596
EBP	5.41806	5.40874	5.10652	5.08659	6.15457	7.08262	5.49183	7.22668	5.69637	4.59557	4.78666	3.81329	5.9362	6.35647	6.01353	6.24785
TBC1D25	2.60677	2.87181	3.0956	2.96826	3.42999	3.20535	3.06502	3.12311	3.81684	3.72598	3.95796	3.60984	3.29554	3.54571	3.86032	3.9329
RBM3	6.31592	6.20666	6.38408	6.47985	6.08284	4.9667	6.33938	5.09569	6.48795	6.05084	6.10388	6.09989	7.41431	6.81176	7.11776	6.64497
WDR13	3.73241	4.06047	3.95588	3.80158	4.04015	4.93859	3.36969	4.87292	3.95542	4.25082	4.41013	3.68346	3.26326	3.4119	3.78007	3.79151
WAS	-1.75838	-0.33072	-1.59202	-0.9672	-0.21523	0.60138	-1.98774	1.10758	-2.50818	-1.16566	-2.08883	-1.08677	-1.98153	-0.90893	-2.37061	-2.61158
SUV39H1	3.89221	3.38128	3.39628	2.58626	4.45835	3.58846	3.9483	3.86511	4.44245	3.07223	3.61481	2.3137	4.19927	3.757	4.3924	3.70382
HDAC6	1.42977	1.12649	1.41675	1.34858	3.18391	3.82563	2.49888	3.70241	2.82445	2.98035	2.99014	2.64824	2.41479	2.97071	2.98705	3.14344
PCSK1N	-1.00406	-0.43612	-0.49992	-2.54049	8.25852	10.1544	6.70209	10.3286	6.52306	7.31245	6.84042	4.10985	2.95846	3.74796	3.44153	4.16489
TIMM17B	5.86002	5.65112	5.60675	5.27804	6.50264	7.1161	5.59433	6.95266	5.83473	5.73514	5.68485	4.53718	5.58973	5.57604	5.66269	5.76099
PQBP1	6.32112	5.91617	5.81861	5.58287	6.1151	6.5746	5.60967	6.66855	6.02653	5.64973	6.00529	4.7071	6.15493	5.54861	6.1625	5.79445
SLC35A2	3.95787	4.09428	4.03587	3.84645	5.64223	5.68354	5.3716	5.30611	3.8585	4.16398	4.17033	3.75056	4.06398	4.52509	3.90109	4.20501
PIM2	2.37516	2.76985	2.16863	2.6818	4.01305	4.37984	3.45112	4.13637	4.46316	3.92069	3.85072	3.41315	4.06219	4.0437	3.05993	3.13682
OTUD5	5.41574	5.37412	5.53548	5.22342	4.76448	4.90935	4.37944	4.56163	5.73385	5.65586	5.93562	5.41218	5.102	4.5313	5.58623	5.16688
KCND1	0.54919	1.74643	1.69384	2.22416	-1.12782	-0.55413	-2.07563	-0.7162	1.06247	2.57005	1.49956	2.78741	-0.86187	0.21935	-0.43826	1.6261
GRIPAP1	3.37713	3.27439	3.16526	3.42125	3.41958	3.49474	3.05437	3.54904	3.46536	3.25087	3.69048	3.58955	3.32212	3.54758	3.25479	3.4803
TFE3	5.12109	5.07032	5.24107	4.92867	5.14167	4.93399	4.72538	4.78332	5.99917	6.04618	6.17438	5.74354	5.46114	5.55786	5.82242	5.83348
CCDC120	1.38417	1.35451	1.62007	1.03768	2.36848	2.39226	1.36701	2.31549	1.51001	2.04437	2.21745	1.47718	1.33566	1.54864	2.07399	1.93787
PRAF2	5.17351	5.5303	5.94718	6.179	4.12553	5.21942	3.57411	4.57709	5.24136	5.2976	5.34757	4.92817	3.77132	3.12439	4.14105	3.55444
WDR45	4.80524	5.16875	5.29626	5.56516	4.53275	5.23409	4.23645	4.74425	4.37161	4.75957	4.73418	4.51004	3.64146	3.58518	3.69722	4.01551
GPKOW	5.1046	4.90228	4.90571	4.71444	5.22625	5.09214	5.00553	4.81961	5.47345	5.00096	5.48619	4.64498	4.93741	4.69913	4.99437	4.69874
MAGIX	-0.40836	-0.41854	-0.8622	-0.27051	1.59328	2.95743	0.78003	2.90554	-1.08634	-0.70625	-1.16374	-0.99024	-1.61612	-0.77004	-1.46924	-0.65769
PLP2	8.25136	8.58586	8.74919	8.70438	7.69741	8.48149	7.7136	8.07894	7.62898	7.12153	7.28946	7.30071	8.03889	7.78597	7.96465	7.79933
PRICKLE3	3.85651	3.88225	3.92183	3.43782	3.86786	4.42455	3.14283	4.53199	4.43633	4.51426	4.50173	4.25409	2.41552	2.27001	1.93929	2.42338

SYP	-0.91161	-0.81676	-1.32288	-1.19041	3.20587	3.85651	2.96276	4.03899	0.04842	1.61856	0.84471	0.77262	0.12571	2.0303	0.47822	1.6352
SYP-AS1	-3.32193	-3.32193	-3.32193	-1.11547	4.0085	3.39565	4.17081	2.95686	-1.20793	1.82866	2.13813	0.40244	0.28905	2.74314	1.69798	2.47245
HSPB1P2	1.48584	1.71849	1.72233	0.43949	-0.38822	1.93988	-1.00562	0.97069	0.50772	0.68508	0.35393	-1.43632	-1.63163	-0.18976	-0.3611	-2.06663
CCDC22	5.02602	4.88071	4.69291	4.61797	4.25623	4.5231	3.8375	4.27791	4.36774	3.90429	4.20498	3.64713	3.62955	3.47624	3.81353	3.94131
FOXP3	1.83182	1.32332	1.2999	1.48649	0.83278	1.68382	0.00459	1.6319	0.20979	0.0469	-0.07327	-0.67088	0.73293	0.86129	-0.27257	0.31559
PPP1R3F	0.06837	0.43564	0.26509	0.40153	-0.28841	-0.63189	-0.90504	0.44458	0.21325	1.4617	0.79717	0.66087	0.10599	0.68702	0.34706	0.79367
GAGE10	-2.03351	-3.32193	-0.35175	-3.32193	-3.32193	-1.35212	-2.09136	-0.70192	1.56101	3.21432	2.63859	-2.07851	-3.32193	-3.32193	-1.41325	-3.32193
GAGE12J	-0.29106	-1.24454	0.70456	-1.11338	-1.30528	0.88017	-0.6537	-0.70863	2.14912	4.03773	3.49136	2.24001	-3.32193	-3.32193	-1.41913	-3.32193
GAGE13	-0.03117	-1.22187	0.87569	0.03466	-0.74289	1.01776	-0.62869	0.39969	2.28273	4.0051	3.40154	2.38858	-3.32193	-3.32193	-1.39736	-3.32193
GAGE2E	-0.56116	-3.32193	-0.35019	-1.54321	-0.12356	1.28488	0.62119	-1.60356	2.39194	2.80142	3.0144	0.64207	-3.32193	-3.32193	-3.32193	-3.32193
GAGE12C	-0.88413	-3.32193	-1.15537	-3.32193	0.43396	-0.24381	1.11239	-0.32073	0.3766	1.97613	2.12822	-1.39887	-3.32193	-3.32193	-3.32193	-3.32193
GAGE12D	-0.88413	-3.32193	-1.15537	-3.32193	0.6216	-0.00953	1.20133	-0.32073	0.71426	2.1707	2.36025	-1.39887	-3.32193	-3.32193	-3.32193	-3.32193
GAGE12F	-0.53771	-3.32193	-0.6036	-2.00766	0.07087	1.00376	0.50508	-3.32193	1.97564	3.06875	3.09057	0.52584	-3.32193	-3.32193	-3.32193	-3.32193
GAGE12E	-0.52073	-3.32193	-1.14035	-3.32193	0.8058	0.20957	1.21978	-0.30383	0.73238	2.47746	2.46247	-0.58647	-3.32193	-3.32193	-3.32193	-3.32193
GAGE12G	-0.88413	-3.32193	-0.68171	-1.80634	-0.46301	1.22252	0.41924	-3.32193	2.1861	3.0347	3.006	0.58123	-3.32193	-3.32193	-3.32193	-3.32193
GAGE12H	-0.88413	-3.32193	-1.15537	-3.32193	1.63332	0.66814	1.11239	-0.32073	0.55528	1.97613	2.27187	-1.39887	-3.32193	-3.32193	-3.32193	-3.32193
GAGE1	-0.37455	-1.79396	-0.15947	-0.50357	-2.1701	-1.26831	-1.84143	-1.17718	-0.66768	0.91095	0.71367	-1.03977	-3.07359	-3.32193	-3.32193	-3.32193
GAGE2A	1.73719	-0.39604	1.13076	1.43183	0.55917	1.12478	0.55787	0.40473	0.17011	1.74851	2.02118	-0.32782	-3.32193	-3.32193	-3.32193	-3.32193
VDAC1P2	0.87567	-0.12048	0.36729	-0.56418	-0.55282	-0.83239	-0.92644	-1.65651	0.07751	1.30877	0.41106	0.18741	-1.66841	-2.54558	-0.49363	-0.65602
PAGE1	5.0192	4.5893	4.39085	4.66912	1.05845	0.06215	1.44074	0.70219	-2.08523	-1.62111	-3.32193	-2.24715	-1.41773	-3.32193	-3.32193	-3.32193
USP27X-AS1	-2.88168	-1.94227	-3.32193	-1.26767	0.62268	0.95607	0.37753	0.26724	-1.2115	-1.41397	-1.81183	-1.08281	-2.06023	-1.76125	-1.29824	-1.69463
USP27X	-0.24958	0.48305	0.52068	0.41685	1.16376	1.52985	1.32625	1.43792	1.73947	0.92675	1.83606	1.62247	1.27651	0.24816	1.88543	0.682
CLCN5	0.8028	0.79284	0.57926	1.18158	3.19987	2.71502	3.16934	3.0841	1.12524	0.32866	0.39114	0.88443	3.18059	3.22123	2.85571	3.40584
CCNB3	0.78384	0.09286	-0.07382	-0.71957	2.26466	1.71908	2.46958	1.63425	-0.54825	-0.44106	-0.65468	-0.06324	-1.05209	-1.09017	-2.07692	-1.06451
SHROOM4	-0.07589	-0.32241	-0.57883	-0.20416	-3.32193	-3.32193	-3.32193	-3.32193	-0.01374	-0.45377	0.05866	0.07607	-3.32193	-3.32193	-3.32193	-3.32193
NUDT10	-1.456	-1.33324	-1.84134	-2.35548	-2.47202	-2.50301	-1.85541	-2.78644	-0.57568	0.06675	-0.54129	-1.01733	-3.32193	-3.32193	-3.32193	-3.32193
NUDT11	-1.75999	-1.35034	-2.85149	-1.91963	0.91293	0.53504	0.87296	0.1458	3.32884	2.96707	3.05169	3.20655	-3.32193	-3.32193	-3.32193	-3.32193
GSPT2	-1.5175	-1.28317	-1.33884	-0.85056	3.64917	3.11598	4.18166	2.98332	3.25299	3.67964	3.57902	4.01526	3.10177	3.62509	3.46231	3.8506
MAGED1	4.05538	4.52312	4.5723	5.08295	6.17534	6.52857	5.95217	6.30156	6.21845	6.4008	6.63146	6.76765	4.5357	4.95456	5.10138	5.53854
MAGED4B	-2.92821	-1.95186	-2.11962	-1.95208	3.79693	4.35302	3.2482	4.06405	3.03505	3.8939	3.91813	4.0409	3.39375	3.57958	4.54425	4.85367
MAGED4	-2.91836	-1.9267	-2.0964	-1.92692	3.83608	4.39139	3.29525	4.1014	3.07538	3.93444	3.95868	4.07756	3.4325	3.62301	4.58396	4.87976
XAGE1A	9.11271	8.98906	9.2838	8.62715	6.12345	7.147	5.96034	6.81593	-1.19453	-2.55742	-1.99918	-0.96525	-3.32193	-3.32193	-3.32193	-3.32193
XAGE1B	8.71329	8.59524	8.88464	8.23543	5.73534	6.75986	5.55712	6.42542	-1.4933	-2.70907	-2.2262	-1.27857	-3.32193	-3.32193	-3.32193	-3.32193
SSX2	6.50621	6.47984	6.19007	6.35761	5.89327	6.78992	5.6178	7.1405	-2.65533	-3.32193	-3.32193	-3.32193	-3.32193	-3.32193	-2.75397	-3.32193
SSX2B	5.94174	5.92848	5.62518	5.79209	5.31896	6.20767	5.06384	6.53767	-2.83644	-3.32193	-3.32193	-3.32193	-3.32193	-3.32193	-2.91202	-3.32193
FAM156B	3.30068	3.147	3.34329	3.22941	3.60026	4.44248	2.87477	4.49548	2.08756	2.09265	2.30323	1.77351	3.02934	3.218	2.26985	2.50894
FAM156A	2.78669	2.61461	2.80771	2.68508	3.06101	3.8969	2.3472	3.92878	1.60179	1.58468	1.80012	1.2924	2.5049	2.69222	1.77841	1.97387
TSPYL2	2.80122	2.42805	2.49035	2.84974	3.02279	4.57635	2.81859	4.71972	2.0459	1.57965	1.90923	1.48101	2.65102	2.92946	2.5994	3.39981
KANTR	0.34794	0.2907	0.11347	0.69595	-0.17308	1.17506	0.04842	1.61218	-0.78179	-0.7247	-0.75134	-0.54472	0.3447	0.44718	0.07787	0.40134
ACTG1P10	-1.36989	-0.99266	0.26873	-0.63641	0.25907	0.06049	-0.6537	0.63262	-0.73635	-0.7755	-0.52603	-0.63487	0.64255	-0.00589	0.08241	1.26901
KDM5C	4.92218	4.89609	4.84379	4.54611	5.24113	5.03379	4.89374	4.88347	5.07937	5.09437	5.22331	5.02422	4.53454	4.49118	5.05116	5.27048
IQSEC2	-0.68584	-0.28884	-0.16235	0.58435	-0.57458	-1.71892	-1.05352	-1.44254	1.64693	1.90789	1.92709	1.83025	-0.70022	-0.89002	-0.14616	0.20516
SMC1A	4.04417	3.7937	3.58934	3.77684	5.02416	3.49505	4.90258	3.77373	5.09778	4.19292	4.39638	4.06941	5.17941	5.21121	5.23952	5.40173
RIBC1	-1.19394	-1.46615	-0.91567	-1.22649	-0.52552	-1.18168	-0.96722	-0.48024	-2.18072	-2.15183	-1.33827	-1.8779	-1.54043	-0.67504	-0.57899	-0.13643
HSD17B10	7.19323	7.07543	7.04488	6.86348	7.41411	8.12453	7.11437	7.94003	7.10056	7.18341	7.58374	6.58068	7.09548	7.33282	6.99885	6.9777
PHF8	2.04282	1.78788	1.73251	1.49975	2.65479	2.2553	2.64518	2.08301	2.36276	2.12669	2.65703	2.47298	2.75206	2.70448	3.24217	3.22755
FAM120C	1.1882	1.38662	1.30576	1.36912	0.43824	0.18207	0.11074	0.23952	0.08916	0.55523	0.46389	1.09216	0.15809	0.62853	0.38324	0.68378
WNK3	-3.05534	-3.32193	-3.32193	-2.72506	-1.63827	-0.75512	-1.50389	-1.0888	-3.1112	-2.91464	-3.2242	-2.78692	0.12582	0.32953	0.29149	0.65888
TSR2	5.73402	5.28328	5.2987	4.99871	5.87099	5.72776	5.47705	5.78719	5.6649	5.16634	5.70738	4.06964	5.78233	5.56611	5.58117	5.34292
FGD1	3.98222	3.90255	3.81342	3.65953	3.42705	3.48375	2.48302	3.72562	3.66175	3.75223	3.66899	3.20234	3.73632	4.1155	4.29661	4.59873

GNL3L	5.01997	4.80702	4.22573	3.71766	4.98351	4.32289	5.09755	4.4306	5.05322	4.23732	4.9983	4.70143	5.24813	4.86963	5.58015	5.28786
MAGED2	4.60177	5.19043	5.48013	5.88801	6.48727	7.06819	6.41798	6.76265	5.77396	6.24667	6.1986	6.70608	5.36219	5.4518	5.58763	5.60085
TRO	-2.89251	-2.49147	-2.56956	-2.53163	-2.65384	-1.07736	-2.6996	-1.52528	1.86107	2.86987	2.6026	3.63132	-3.32193	-3.08262	-2.58901	-3.16777
APEX2	4.75143	4.4598	4.42201	4.12857	4.34008	4.28139	3.59026	4.1441	4.60043	3.85255	4.48058	3.6752	4.1345	3.65001	3.5901	3.35797
PAGE2B	2.89684	2.01858	2.91374	1.15429	1.12463	5.04733	0.13783	4.70802	-3.32193	-2.01383	-3.32193	-3.32193	-3.32193	-3.32193	-3.32193	-3.32193
PAGE2	6.7659	6.72871	6.86285	6.8189	4.01855	7.00353	3.80935	6.78148	-3.32193	-3.32193	-3.32193	-2.24257	-3.32193	-3.32193	-3.32193	-3.32193
FAM104B	2.36803	2.24374	2.33068	2.08947	2.1547	2.84733	2.0561	2.86854	2.52798	2.55583	2.24274	1.82568	3.32933	2.9427	2.05739	2.14861
PAGE5	6.82042	6.65553	6.77313	6.57852	2.36666	2.4278	1.54974	2.57032	-3.32193	-2.31668	-3.32193	-2.32898	-3.32193	-3.32193	-3.32193	-3.32193
MAGEH1	2.43526	3.00445	2.83672	3.64362	4.47948	4.75726	4.43005	4.45594	3.67238	3.78324	3.82563	3.75345	3.71341	4.22551	3.23663	3.70611
USP51	-2.55263	-0.90274	-2.02298	-0.11623	-1.87817	-1.07726	-1.75017	-1.13259	0.08693	1.00146	0.16564	1.6112	-1.57971	-0.34484	-0.80895	0.26292
FOXR2	3.13819	2.89282	2.40701	3.05141	-3.09401	-2.82886	-2.86129	-3.32193	-3.32193	-3.32193	-2.81863	-3.32193	-3.32193	-3.32193	-3.32193	-3.32193
RRAGB	1.55981	1.33888	1.8916	2.60987	2.16312	2.77974	2.36494	2.6552	1.49296	2.3423	2.18753	2.7818	3.39815	3.58437	3.49521	3.5878
UBQLN2	3.5502	3.56542	3.36384	3.7806	3.99829	3.22891	4.373	3.035	4.25889	4.09617	4.29569	4.69848	4.30061	4.44338	4.79769	5.06425
NBDY	3.61885	3.88726	4.04693	4.28967	1.96498	4.31232	2.09058	3.76188	4.98658	5.09451	4.9409	5.03248	4.58288	4.76642	3.76558	4.01055
SPIN3	0.16862	-0.10412	-0.15037	0.37152	0.06595	0.44174	-0.10815	0.27168	-0.7342	-0.47611	-0.69502	0.0797	0.01391	0.05779	-0.69145	-0.18552
SPIN2B	3.56292	3.49902	3.43398	3.69701	3.52686	3.89946	3.42543	3.83036	2.31796	2.70824	2.91794	2.42769	3.27678	3.61859	2.86279	3.08911
SPIN2A	1.54636	1.44537	1.23862	1.81775	1.37779	1.91337	1.35474	1.87922	0.29178	0.79248	1.05376	0.6804	1.04077	1.26716	0.46723	0.87206
FAAH2	-1.77495	-1.66323	-3.32193	-0.65789	2.1408	2.91884	2.24148	2.68024	-3.32193	-3.32193	-3.32193	-2.53213	-1.83293	-1.55611	-2.5278	-0.29912
ZXDB	0.99725	1.50297	1.60069	1.05531	2.31031	0.63339	1.96277	0.84118	2.84224	3.09063	2.72396	2.82844	2.14485	2.38949	2.53485	2.5406
ZXDA	0.25636	0.31464	0.02373	-0.0271	1.68594	0.7016	1.0143	0.48724	1.47296	2.03674	1.6371	2.08143	0.96192	1.58589	1.29872	1.94041
LINC01278	1.96201	2.03606	2.05826	2.18721	1.54152	2.61536	1.66284	2.40074	1.63797	1.67688	1.58445	2.29564	1.96113	1.8911	1.78956	2.13687
SPIN4	1.4135	1.38843	1.40385	1.64265	1.95569	0.86031	2.54077	1.06735	2.47409	1.83677	2.09075	2.49114	3.32123	3.54799	3.06063	2.96158
SPIN4-AS1	0.79884	0.61487	-0.91136	0.77396	1.44932	0.65411	1.87629	-0.90673	2.18495	1.54148	1.45538	1.96336	3.41442	3.35019	2.62948	3.22421
ARHGEF9	-1.19049	-1.11851	-0.94161	-0.83018	0.56434	0.66135	0.63899	0.55993	0.6723	0.97785	0.84718	1.16509	1.07654	1.90075	1.43241	1.89488
AMER1	0.12802	-0.3953	-0.60951	-0.58073	1.99159	0.47706	1.75975	0.55258	2.18765	1.46892	2.26432	2.12766	2.83074	2.59667	2.98228	3.09598
MTMR8	-1.75981	-0.81175	-0.66928	-1.14502	0.46428	-0.41743	0.73333	-0.82483	0.48243	0.05876	0.98443	-0.1626	1.17871	0.24964	0.37696	0.54718
ZC4H2	1.32224	1.51689	1.05172	1.6288	1.61793	2.21277	1.42807	2.23936	2.43337	2.78049	2.29355	2.65889	2.84316	3.35322	2.70111	3.17256
ZC3H12B	-2.00751	-2.68377	-2.45631	-1.92856	-0.77551	-0.53248	-0.29951	-0.09362	-2.66553	-3.06953	-2.72406	-2.9636	-1.55317	-1.59004	-1.00716	-0.9819
TLE1P1	-0.67328	-3.32193	0.98063	-1.90863	0.68796	0.97467	0.27141	1.76183	1.2615	0.90989	0.40984	0.43207	-0.47515	0.52329	0.44156	-0.70285
LAS1L	4.37306	4.03317	3.85587	3.75229	4.36831	4.21218	4.30763	4.1795	4.69722	4.02112	4.42569	3.89054	4.37796	4.24423	4.21686	4.16678
HEPH	-3.32193	-3.15525	-2.99018	-2.97088	0.24976	-3.03429	0.41402	-3.14589	-3.32193	-3.32193	-3.32193	-3.32193	-3.32193	-3.32193	-3.17896	-3.32193
EDA2R	-3.32193	-1.88695	-2.30212	-1.77779	-3.07802	-3.32193	-2.83152	-3.32193	3.21336	2.98945	3.44103	3.85497	-3.09556	-2.57593	-2.82206	-2.81275
AR	-0.74136	-0.69376	-0.72779	-0.07201	-3.24934	-3.32193	-3.16693	-3.32193	0.86635	1.41467	0.89214	2.04064	1.21285	0.70652	1.50477	1.58169
OPHN1	-0.25824	-0.06192	-0.31406	0.00782	1.36118	0.73539	1.90233	0.87958	2.65484	2.08119	2.15397	2.3381	0.08562	0.05049	0.50131	0.72805
YIPF6	3.17714	3.27609	3.08483	3.77325	4.35252	4.10394	4.79326	4.09082	2.81176	3.25746	2.52604	3.76392	2.89884	3.15666	2.65058	2.91889
STARD8	3.45103	3.43361	3.55621	3.16746	0.97683	0.63619	0.61712	0.46351	3.15074	2.60695	2.81927	2.25888	-0.36166	-0.7599	-0.15211	-0.08181
SERBP1P1	1.54107	0.92776	1.39863	1.14508	1.08237	0.53899	1.37748	0.3715	1.87869	1.55523	1.29665	0.58054	0.95203	-0.09591	0.46215	0.27497
EFNB1	4.55866	4.60968	4.4013	3.55077	3.96774	3.4553	3.51398	3.38711	3.0848	3.08763	3.39341	2.41071	1.49335	1.38109	1.66142	2.12887
PJA1	3.68074	3.66725	3.49194	3.63115	3.71926	2.86294	3.8728	2.67323	3.66231	3.52419	3.68285	3.37407	2.03525	2.18007	2.48325	2.5446
FAM155B	-3.32193	-3.32193	-3.32193	-3.32193	2.98543	2.61417	2.24477	2.50838	2.11313	2.0126	2.27151	1.53862	-1.99128	-1.48738	-1.9095	-1.18534
EDA	-2.58611	-2.23775	-2.48338	-2.44228	-2.29612	-2.57974	-2.29683	-2.28268	0.31864	0.54402	0.23857	0.32925	-3.20174	-2.6385	-2.924	-2.80304
IGBP1	5.17613	5.11382	5.12165	5.4062	4.94136	5.1903	4.99844	5.11087	5.93305	6.22381	5.98449	6.67644	4.54335	4.62829	4.43228	4.71897
IGBP1-AS2	5.05453	5.06835	5.08644	5.46034	4.9334	5.30057	5.00072	5.20903	5.89149	6.05484	5.94705	6.64893	4.62262	4.84202	4.44405	4.57712
RAB41	-1.36314	-1.03241	-0.23013	-0.40335	-0.02486	1.09999	0.90982	1.1114	-0.50614	-0.60636	-1.12364	-0.30103	-0.36103	-0.54365	-0.50129	-1.1906
PDZD11	5.74232	5.87519	5.94376	6.10324	6.06903	6.31239	6.0039	6.01004	6.4345	6.17716	6.27505	5.96284	5.09918	5.15696	5.17444	4.92527
KIF4A	4.54012	4.06044	4.1396	3.67667	5.11316	3.93702	5.46004	3.96324	5.29594	4.29025	4.54225	4.16234	4.23768	4.22948	4.65492	4.37398
DLG3	0.59923	0.77426	0.9476	1.33526	-0.61111	-0.97097	-0.59207	-1.38667	-0.16236	0.46312	0.38902	0.38957	-0.70933	0.28685	0.73826	0.90071
TEX11	0.81368	0.21351	0.70264	0.49356	-2.01933	-3.02001	-1.8787	-2.69181	-2.47165	-2.79183	-3.32193	-2.41654	-3.32193	-3.32193	-3.32193	-3.02953
RPS23P8	5.9199	5.77461	5.81006	4.83807	3.55908	4.68827	3.55279	4.12462	4.74903	5.38645	4.61259	5.16977	4.01167	3.49817	3.03275	2.29858
SNX12	4.83052	4.78624	4.65864	4.55745	5.97934	5.55649	6.04408	5.17109	6.14163	5.85827	6.09956	5.77293	5.50288	5.5074	5.65983	5.73388

FOXO4	1.98193	2.02421	1.82419	1.71523	2.55243	2.45821	2.10549	2.22635	0.76253	1.49262	0.90512	1.29157	-0.98362	-0.31641	0.12282	0.87642
IL2RG	2.86766	3.92175	3.84386	3.95481	0.83507	-2.04598	0.69815	-1.90383	-2.33601	-2.97578	-3.32193	-3.32193	-2.10668	-1.74872	-2.70185	-2.97209
MED12	2.88268	2.82867	2.88151	3.11283	3.80503	3.46723	3.63383	3.48733	3.52314	3.83409	3.82878	4.5421	2.00222	2.22579	2.78373	3.30633
NLGN3	-3.32193	-3.03806	-2.77689	-2.74744	-1.91831	-0.81065	-2.69663	-0.83426	-0.748	-0.33781	-0.93933	0.19772	-1.82553	-2.39029	-1.4781	-1.38234
GJB1	-3.32193	-2.2047	-2.8248	-3.32193	6.95539	6.40456	6.09039	6.47481	-3.32193	-2.90524	-3.32193	-2.91137	-2.46104	-3.32193	-3.32193	-3.32193
ZMYM3	2.66181	2.76573	2.77412	2.82749	3.77936	3.43896	3.07873	3.6064	3.97635	4.21843	3.89038	4.49488	2.83102	3.08915	3.39722	3.79459
TAF1	1.18886	1.1646	1.03971	1.62163	2.02719	1.35838	2.02475	1.43353	2.41054	2.6543	2.61329	3.23504	3.09759	3.57687	3.17825	3.47948
OGT	3.45526	3.21412	3.68099	3.47819	3.72447	4.07097	3.89761	3.52153	3.14464	3.5076	3.67541	4.30515	3.93027	3.85917	3.58156	3.52852
GCNA	-1.04153	-0.97433	-1.39849	-0.22158	-2.19535	-1.02891	-1.6435	-0.59758	-1.00398	-0.64976	-0.71148	0.20989	0.65781	0.14264	-2.0395	-0.22939
CXorf49B	-3.32193	-3.32193	-3.32193	-3.32193	0.51879	-0.00904	-0.56151	-0.08755	-3.32193	-3.32193	-3.32193	-3.32193	-3.32193	-3.32193	-3.32193	-3.32193
NHSL2	-3.26004	-3.25264	-3.11305	-3.24423	-1.23812	-1.80521	-1.10732	-2.15119	-1.3015	-0.84554	-0.81677	0.08958	-1.80874	-1.39369	-0.92447	-0.88739
RPS26P11	1.68811	1.63407	1.70958	0.29417	0.19145	2.87101	0.19017	2.5035	0.93707	2.1379	1.73748	1.27839	-0.03858	0.57802	-3.32193	-0.40943
RTL5	-3.32193	-3.32193	-3.32193	-3.05287	1.82727	1.92391	2.10912	1.70835	1.25345	1.16702	1.41902	1.41742	-0.46708	-0.19538	0.81482	0.95487
PIN4	4.14951	4.17134	3.90254	3.90695	3.16729	4.00929	2.7542	3.96883	3.62735	3.84914	3.6073	2.16482	2.91517	2.79549	1.76846	2.03673
ERCC6L	2.61565	2.17998	2.15752	2.04222	3.26559	1.69099	3.87208	1.79217	3.49351	2.30841	2.93616	2.08661	3.63745	3.29344	3.67243	3.13099
CITED1	-0.09256	0.18828	-1.10251	0.77771	0.05162	0.33255	0.00803	0.20039	-2.3039	-1.88847	-2.7791	-2.81803	-2.89381	-2.5662	-1.19489	-1.66581
HDAC8	2.64308	2.55222	2.51701	2.76618	2.32261	2.86055	2.79813	2.61037	2.26686	2.02339	2.47009	2.18876	2.56596	2.66961	2.48454	2.65107
PHKA1	2.17776	1.98863	1.81447	2.07714	3.81655	3.25231	4.0997	3.14593	2.55286	2.6197	2.40361	3.0436	3.79482	3.72872	3.69335	3.99885
NAP1L2	0.18248	0.6498	0.0021	1.1209	1.71932	1.30424	2.09637	1.56554	1.28388	1.22068	0.97105	1.69822	-1.46479	0.67523	-1.5887	0.3652
CHIC1	0.43586	0.63809	0.05772	1.12142	2.09449	1.65689	2.86454	1.45106	2.20924	2.51583	1.98492	3.42853	4.12876	4.8528	3.92598	4.18649
JPX	1.32779	1.2748	1.17861	1.76671	0.75089	1.73043	0.91035	1.63676	0.68157	0.86583	0.81117	1.1377	1.44083	1.14073	1.01329	0.71539
FTX	0.63932	0.63746	0.59505	1.17408	0.15225	1.68504	0.38454	1.91514	-0.23356	0.12145	0.06029	0.58484	0.64347	0.62081	0.26652	0.26389
RAB11FIP1P1	1.11786	0.05568	1.16321	1.49809	0.75712	2.93942	1.26453	3.41724	-1.49007	-0.01162	-0.11818	0.85761	0.30891	0.38237	-0.22266	1.0547
SLC16A2	5.26586	5.66068	5.2741	5.08641	3.83428	3.50021	3.41577	3.4324	6.30502	6.08453	6.01851	6.37534	4.94604	4.60528	5.34747	5.90826
RLIM	3.08925	3.33054	2.9437	3.29873	3.2095	2.42563	3.59498	2.49329	3.89763	4.01114	3.46202	4.81263	4.10345	4.57655	4.28757	4.77601
ABCB7	3.12492	2.94423	2.99903	3.07831	3.76389	3.30777	3.96023	3.41519	3.96826	4.04259	3.95452	4.46749	3.65401	3.79387	3.47285	3.76449
UPRT	2.87833	3.08008	3.08149	3.26868	2.81847	2.96243	3.06309	2.84618	3.30796	3.39026	3.18484	3.31395	2.71304	3.08867	2.3158	2.8808
TTC3P1	-2.30683	-2.22026	-1.90873	-0.90232	-0.54495	-1.11615	-0.15488	-0.35269	2.15274	1.95545	1.92679	3.30573	0.36723	1.68192	1.71388	2.68418
PBDC1	4.97463	4.66755	4.66476	4.59491	4.2796	4.35654	4.47104	4.13953	5.00445	4.27467	4.94616	4.07774	4.04025	3.72973	3.60066	3.57965
MAGEE1	0.63589	0.62281	0.4505	1.03094	1.77928	1.30913	1.67511	1.05906	2.08756	1.75624	2.11452	2.29387	2.60403	2.61871	2.55005	2.90019
ATRX	2.45524	2.62214	2.67431	3.97303	2.76395	2.89258	3.45346	3.37075	3.26961	3.32178	3.0948	4.39129	3.60839	3.51612	3.98444	4.01999
MAGT1	4.5886	3.90238	4.58181	4.05173	6.07237	4.67265	6.67754	4.09853	5.31159	3.75238	5.16399	4.06706	5.68927	2.56849	5.71459	2.7849
COX7B	5.08327	4.83162	4.86934	5.12237	4.99678	5.83898	4.96773	5.74698	4.89063	4.88394	4.64305	4.44323	5.84361	5.85554	4.51657	4.34959
ATP7A	0.06541	0.3384	0.52287	0.97158	2.07453	1.26864	2.88352	1.05705	1.48398	1.45944	1.57754	3.29223	2.33762	2.12034	2.58058	3.17244
PGAM4	3.60562	3.53619	3.33287	1.41224	1.57864	2.35791	1.3574	1.64004	4.37413	4.5231	3.39298	5.19942	2.32994	2.57548	2.56359	2.66387
TAF9B	3.25201	3.59146	3.38924	3.99706	3.29742	2.91661	3.58044	2.97685	3.61891	3.83645	3.15624	4.61536	4.45334	4.33949	4.12627	4.14885
RTL3	-0.88991	-1.33615	-1.27748	-2.01157	-3.32193	-3.32193	-3.32193	-3.32193	0.29191	-0.65063	-0.49969	-1.0321	-2.77671	-3.32193	-3.32193	-3.32193
LPAR4	-1.32489	-1.39656	-2.19662	-1.38359	-3.04481	-2.99761	-3.02008	-3.32193	-2.95479	-2.75552	-3.32193	-2.04805	0.45374	0.56739	0.00652	-0.31193
ITM2A	-2.85892	-3.32193	-3.32193	-3.32193	-1.48544	-1.98256	-1.52592	-2.78579	2.34207	3.47034	3.13149	3.81665	-1.56847	1.52445	-0.16031	0.13363
CHMP1B2P	-2.58996	-3.15376	-3.14497	-2.44668	-0.95357	-0.0459	-0.43933	-0.68025	-2.60808	-3.17629	-2.89719	-2.62013	-3.32193	-3.32193	-3.32193	-3.32193
BRWD3	0.59095	1.02518	0.6175	1.42942	2.51778	1.71257	3.02931	1.75069	2.90926	3.01171	2.84295	4.12113	3.16757	3.66191	3.18977	3.75369
VDAC1P1	1.80173	1.35921	1.24916	0.18885	0.82492	0.74255	0.98514	1.04227	1.40773	2.02557	1.35903	0.73271	-0.73237	-0.17476	-3.32193	-1.44244
HMGN5	2.40712	2.55312	1.96563	2.53045	3.14046	4.89974	3.40941	5.12075	2.41348	2.19903	1.46586	2.02111	1.77486	2.81175	1.10764	1.96096
SH3BGRL	3.03884	3.44803	3.26875	3.87219	3.86574	3.9281	4.31767	3.44862	5.21492	5.21832	5.08383	6.37782	5.3296	5.0199	4.76618	4.58685
RPS6KA6	-3.1959	-2.93525	-2.80329	-3.02271	-0.36128	-0.53386	0.35153	-0.19942	0.92891	1.22941	0.87974	1.96953	-0.01916	0.50421	0.09415	0.75334
HDX	-1.32404	-0.44199	-0.9488	-0.22439	-2.73738	-1.43326	-2.1218	-1.78199	0.13008	0.39699	0.23208	1.37504	0.0425	0.65001	0.46067	0.80634
APOOL	2.18798	2.36608	2.1365	2.20752	2.92132	2.39975	3.31103	2.49941	2.744	2.67756	2.04524	3.25717	3.4645	2.83806	3.75716	3.26595
SATL1	1.3365	1.41624	1.21777	1.91285	1.36786	1.78669	1.93762	2.32662	1.4012	1.18528	0.34232	1.76687	1.80685	1.71076	2.13918	1.8614
ZNF711	-2.77073	-2.41856	-3.07856	-2.33716	1.22629	1.57035	1.72859	1.57427	1.86851	2.07995	1.95451	2.70606	0.28438	1.52239	1.01797	1.60232
POF1B	-3.32193	-3.32193	-3.32193	-3.00789	3.7576	2.01926	4.65957	1.93045	-2.78805	-2.86616	-3.32193	-2.69167	-0.63645	-0.20603	-0.16676	0.62992

CHM	1.76714	1.81392	1.83055	2.17995	2.30297	1.17074	3.04946	1.27331	1.97444	2.12697	1.8242	2.67954	3.28745	2.65107	2.55512	2.60911
KLHL4	-0.08862	0.63447	0.92727	1.3929	-3.09546	-2.83174	-2.23739	-3.10946	-1.9711	-0.4534	-0.95853	0.53918	4.4314	4.75815	4.76569	4.78015
RPSAP15	5.15445	5.03027	5.07181	3.61778	4.88086	4.24534	4.86021	3.61062	6.65971	7.11898	6.82015	6.59744	1.51307	1.23375	1.22505	2.11182
EIF4A1P10	4.2522	3.65955	4.14718	2.89379	2.60016	3.11816	2.05356	2.88515	4.19189	4.51553	4.17668	3.93425	0.96355	0.58023	0.58647	1.02208
ST13P18	-0.50518	-0.71565	-0.00678	-1.37256	-0.63123	-0.49154	-1.68212	-0.28791	0.67202	1.32471	0.92646	0.04452	-0.66008	-1.34018	-0.28363	-1.34011
NAP1L3	-0.36508	-0.52663	-0.68354	-0.06807	-1.11334	-0.7238	-1.13012	-0.94408	1.71333	1.78511	1.82486	2.00116	-2.56387	-0.7806	-2.23572	-0.99482
FAM133A	2.64962	2.95462	2.75184	3.3151	1.66937	2.15062	1.87065	2.63597	1.49651	1.4511	1.16364	1.64379	-2.54006	-2.14919	-3.32193	-3.32193
DIAPH2	1.70907	1.79702	1.42242	2.09266	1.32125	1.00455	1.94959	0.74468	2.19161	2.12019	1.94668	3.12196	2.82336	2.52264	3.11151	2.70047
DIAPH2-AS1	-1.09006	-0.85536	-0.52009	-0.01291	-1.67055	-1.30093	-0.89095	-1.2521	-0.38799	-0.24213	-1.04982	0.31862	0.41971	-1.26926	1.12828	-0.12023
TSPAN6	3.30586	3.42515	3.49204	4.08336	3.79049	3.66838	4.19719	3.42845	3.95761	4.14907	3.9712	4.55926	4.36553	4.40657	4.26801	4.483
SRPX2	1.47168	1.47979	1.64768	1.82897	-0.97002	-1.43322	-0.76243	-1.17644	1.49827	1.6974	1.80212	2.24189	0.64719	0.4775	0.03255	0.96074
SYTL4	0.97842	1.21924	0.9776	1.45315	0.75965	-0.74536	0.93665	-0.42229	-0.45099	-0.27677	-0.87889	0.12502	-0.55388	-0.36403	-0.64681	0.13652
CSTF2	5.88115	5.52188	5.41724	5.42538	4.81732	4.1146	4.64065	4.06805	5.17272	4.38823	4.85411	4.21226	5.30667	5.11346	4.90449	5.0215
NOX1	-1.00302	-1.06221	-1.22695	-0.72824	-0.91563	-0.82258	-0.10355	-1.49203	-2.54504	-2.94794	-1.94921	-2.20842	-1.36398	-0.91261	-2.41196	-2.18579
TRMT2B	3.69409	3.62838	3.44239	3.36407	3.59477	2.99919	3.65401	3.03239	3.3155	2.86019	3.092	3.2505	3.71648	3.29672	3.71671	3.70735
CENPI	2.63806	2.66668	2.69685	2.18404	3.31444	1.65579	3.76162	1.98799	3.3423	2.34717	2.72924	2.44537	4.01146	4.10955	3.70758	3.59774
TIMM8A	3.91053	3.4769	3.60067	3.18885	3.71858	2.79396	3.87282	2.56774	3.05332	2.74512	3.00924	1.68099	4.00732	3.82822	3.02377	3.03986
GLA	5.19356	5.08639	4.78053	5.38399	6.28356	6.06241	6.58574	5.7588	4.0806	3.77897	3.88163	4.02783	5.43123	5.23252	4.8248	4.99877
HNRNPH2	5.56704	5.65019	5.51875	6.08786	5.69707	4.76982	6.0885	4.69651	5.95767	6.21008	6.12506	6.1637	6.06851	6.56903	5.97576	6.35345
ARMCX4	-2.82938	-2.91459	-2.82294	-2.72385	-3.09194	-3.23953	-3.17329	-2.82127	-1.63048	-1.57588	-0.827	-1.31079	-3.02596	-3.13779	-3.32193	-2.78207
ARMCX1	4.0431	4.52401	4.27992	5.05649	-0.83169	-2.01971	-0.49599	-1.47915	4.59955	5.30617	5.25328	5.63186	-2.22703	-1.2818	-3.32193	-3.32193
ARMCX7P	2.94753	3.07406	3.05823	3.10704	3.56607	4.39006	3.36804	4.30176	4.17884	4.43805	4.3729	4.68261	-3.32193	-3.32193	-3.32193	-3.32193
ARMCX6	2.73297	2.7015	2.62258	2.76382	3.55182	3.99623	3.32558	3.80436	4.02371	4.48226	4.42304	4.45971	-3.32193	-3.32193	-3.32193	-3.32193
ARMCX3	3.51055	3.59464	3.89028	4.36468	3.93271	3.41802	4.52578	3.12494	3.91275	4.26194	4.1794	4.51224	-2.21927	-2.22202	-3.08951	-2.70274
ARMCX3-AS1	3.44237	3.38007	3.59808	3.47216	4.81169	3.45811	5.07937	2.70163	4.78304	4.93048	5.01169	4.35903	-1.52211	-3.32193	-3.32193	-3.32193
ARMCX2	-1.87303	-1.44618	-1.71332	-1.32087	-2.97083	-3.03665	-3.05666	-3.32193	3.43066	3.48462	3.71321	3.80516	-3.32193	-3.32193	-3.32193	-3.04569
ZMAT1	-3.32193	-3.02108	-3.32193	-2.71636	-3.25999	-2.62007	-3.18913	-2.63105	-2.10739	-0.97605	-1.82673	-0.50003	-3.20967	-3.00938	-3.18624	-3.05698
BEX5	-2.02312	-0.97215	-0.90916	-0.8343	-0.35441	-0.38763	-0.48727	-0.0463	1.61839	2.83604	1.49718	1.80387	-0.57347	-0.24991	-0.94828	-0.29956
NXF2	3.16632	2.94198	2.86427	3.08124	-1.68489	-1.58017	-1.08607	-1.13735	-3.32193	-2.92202	-2.8963	-3.32193	-3.32193	-2.99012	-2.92548	-2.91784
NXF2B	2.46928	2.25834	2.13838	2.43136	-2.08101	-2.04685	-1.46508	-1.66753	-3.32193	-3.06674	-3.04947	-3.32193	-3.32193	-3.11199	-3.06906	-2.84517
ARMCX5	1.35026	0.87841	0.90973	1.23537	2.22575	1.15391	2.5637	1.38891	2.3073	1.66136	2.21764	2.19993	2.73058	1.93109	2.32302	1.97259
ARMCX5-GPRAS	1.25719	0.59822	0.87166	1.22696	2.17816	1.12703	2.34213	1.20997	2.92989	2.68918	2.71486	2.78912	3.04683	2.88908	2.74731	2.9603
GPRASP1	-2.25034	-1.99343	-2.6619	-1.40289	-1.93693	-1.74645	-2.30157	-0.89752	-1.00457	-0.69105	-1.05755	-0.39311	-0.37458	0.17897	-0.42891	0.14206
GPRASP2	1.44168	1.22363	0.85739	1.49125	0.58632	-0.10604	0.01618	-0.32789	3.4285	2.8738	3.32644	3.21999	2.95102	2.15185	3.19238	2.74257
BHLHB9	-1.4452	-1.3221	-1.32214	-0.62052	0.54792	-0.22818	1.05831	-0.14719	1.65866	1.73466	1.71852	2.37671	1.23812	1.72514	1.49879	2.26542
LINC00630	0.19788	-0.39144	-0.40729	0.1181	0.6595	-0.69041	0.6223	-0.49139	-0.12232	-0.57978	0.01028	0.22002	0.7871	0.8903	0.53103	0.79476
BEX1	1.77555	2.57236	1.71926	2.49993	-1.17346	-0.12436	-0.9455	-2.27339	-3.32193	-0.69029	-3.32193	-3.32193	-3.32193	-2.55534	-2.42846	-2.4138
BEX4	-2.65796	-3.32193	-3.32193	-3.32193	-1.52197	-0.8041	-0.89855	-0.95155	5.47	5.89344	5.95361	5.75805	0.80594	1.48116	1.18659	2.30677
TCEAL8	5.16165	5.71608	5.58815	6.34412	-1.06602	-1.77277	-1.33488	-0.66934	5.92034	6.47618	5.92979	6.49173	5.12845	5.49248	4.47069	4.73483
BEX2	2.62567	2.85024	2.26041	2.9876	2.4838	3.5415	2.55846	3.5506	1.30921	2.93983	2.36716	2.36953	2.80252	3.80841	2.362	3.25812
TCEAL9	6.72856	7.0518	7.25013	7.75488	6.9029	7.274	7.3635	7.35562	6.40664	6.46186	6.40693	5.58857	6.80294	6.88324	6.16687	6.15036
BEX3	7.04012	7.43087	7.3371	7.917	7.71313	8.68651	7.66641	8.6781	8.4738	8.67065	8.57548	7.99605	7.86146	8.26481	7.54846	7.80839
RAB40A	-2.08796	-1.98986	-1.82075	-1.44132	-2.06213	-0.86462	-3.08277	-1.60057	-1.72705	-0.68608	-1.32137	-1.21891	-1.42511	-1.68453	-1.55747	-1.79336
TCEAL4	5.73441	5.68676	5.64122	5.82071	4.91061	6.04534	5.20774	6.14457	5.89572	5.86754	6.0438	5.2948	5.09577	5.07011	4.90543	4.97463
TCEAL3	4.36262	4.47796	4.5259	4.52425	2.56565	4.06	2.92824	3.98543	5.32268	5.34992	5.31703	4.39284	3.89455	4.08534	3.50483	3.75556
TCEAL1	2.69933	3.15528	3.20571	4.12169	3.96477	4.45914	4.31851	4.57884	3.87627	4.66528	4.36294	4.90683	3.43339	3.9814	2.52183	3.10601
MORF4L2-AS1	0.40905	0.75437	0.36752	0.80233	-0.33048	-0.10843	1.0073	0.42126	1.20514	1.70365	1.48441	1.69561	1.33303	1.85375	1.60804	1.4014
RAB9B	-3.05041	-2.55871	-3.32193	-2.28937	-3.32193	-2.81732	-3.32193	-3.32193	-0.84341	-0.18645	-0.12789	0.90054	-2.34126	-0.81404	-2.06858	-1.17289
TMSB15B-AS1	-1.57002	-1.21011	-0.94115	-2.00306	-3.32193	-3.32193	-3.32193	-3.32193	-0.62038	0.66381	-0.06101	1.15875	-0.50854	-1.09015	-1.18728	-0.68978
TMSB15B	-2.23528	-2.0475	-1.90821	-2.04771	-3.32193	-2.96122	-3.32193	-3.32193	1.18374	1.28089	0.96209	0.69432	-0.80284	-0.80708	-0.91818	-0.96789

SLC25A53	-0.07341	0.05901	0.28088	-0.70065	0.12059	-0.40841	-0.14501	-0.50047	-0.16688	-0.36236	-0.06272	-0.69913	-0.74313	-0.74741	-0.41595	-0.43097
FAM199X	2.76832	3.05527	3.12008	3.23706	4.0941	2.5132	4.54346	2.32257	3.84674	3.951	3.84227	3.99601	3.36236	3.3934	3.52078	3.12484
RADX	-1.42711	-1.66474	-1.78852	-1.5468	2.33087	1.36399	2.3209	1.59901	0.19029	-0.36114	-1.45373	-0.811	1.01217	1.14396	-0.23756	0.57433
RNF128	-1.0456	-1.39895	-1.34114	-0.40238	6.05016	5.53395	6.52271	5.33986	-3.32193	-2.79973	-2.20334	-1.66941	3.2836	3.08198	3.40507	3.57369
TBC1D8B	-1.22876	-1.31292	-1.63244	-0.9359	1.47212	0.91983	1.7916	0.69188	1.12993	0.73745	0.99038	1.35731	0.29409	-0.48994	0.26238	-0.48663
MORC4	0.01445	0.38791	0.07956	-0.16346	3.33321	2.15106	3.29328	2.41394	3.62608	3.1153	2.65863	3.43041	5.82594	5.01076	5.5653	5.47549
CLDN2	-3.32193	-3.32193	-2.9775	-3.32193	2.03628	-0.14333	2.01091	-0.04949	-2.9833	-2.59211	-2.36355	-1.66308	3.6836	4.46339	5.18027	5.12258
RBM41	1.54171	1.31295	1.49972	2.59884	2.1553	1.81611	2.8927	1.58741	1.50361	2.03097	2.02385	2.87969	2.65511	2.84808	3.03795	3.01477
NUP62CL	-0.2374	-0.08948	-0.43393	-0.35435	1.89504	1.8602	2.06156	1.49541	-1.35876	-2.83561	-2.80526	-2.48344	2.78116	2.91912	1.71156	2.57087
FRMPD3	-3.32193	-3.32193	-3.1839	-3.04219	-3.32193	-3.32193	-3.21209	-3.32193	-1.41188	-2.33594	-2.41278	-2.34807	-2.70731	-2.648	-2.40351	-2.51926
PRPS1	5.41909	5.09027	4.73094	4.8907	5.96671	5.48014	6.08008	5.28123	6.61105	5.66997	6.30286	5.45159	6.64153	6.41212	5.89743	5.84804
TSC22D3	-1.75919	-0.70892	-1.04141	-0.4397	3.38208	2.45523	2.87788	2.46047	1.97216	3.41737	2.40487	3.74992	0.53836	0.3443	0.22994	0.53683
NCBP2L	-3.32193	-3.32193	-3.32193	-3.32193	2.44442	0.99077	1.67868	0.83777	-2.42055	-1.36591	-1.97318	-0.58589	-2.4132	-2.2071	-1.84575	-2.25642
MID2	0.89082	0.71987	0.76828	0.7769	-0.5676	0.15512	-0.45088	0.07564	2.46415	1.87834	2.20702	2.92738	2.20652	1.38077	2.39072	1.97802
VSIG1	-3.32193	-3.32193	-3.00699	-2.98855	1.9586	1.40058	2.30564	0.77948	-1.77851	-1.84315	-2.27466	-1.49909	-3.32193	-3.32193	-3.32193	-3.05775
PSMD10	4.61644	4.63591	4.65909	5.10459	4.85133	4.99333	5.15863	4.82429	5.04154	5.07471	4.9526	4.58718	5.15912	5.61567	4.97866	5.09447
ATG4A	1.96043	2.53218	2.13872	3.04161	2.70714	2.77171	3.40186	2.30023	1.61297	1.51676	2.01008	2.35823	1.9869	2.04113	1.71801	1.38403
COL4A6	-3.20607	-3.07376	-3.06115	-2.81378	-3.27158	-3.09655	-3.11266	-3.18514	1.01054	2.21897	1.71324	2.53391	-0.18519	0.92717	0.20637	1.62977
COL4A5	-2.33859	-2.39802	-2.36063	-1.96028	-1.04462	-1.33513	-1.05474	-1.41628	1.05814	2.10328	1.87582	3.02227	2.45022	2.11191	2.20523	3.15765
NXT2	2.22799	1.74033	2.17357	1.95356	3.06757	1.6425	3.77563	1.49136	2.64468	2.161	2.66568	2.94713	2.67149	2.141	2.72895	2.05447
KCNE5	-1.26486	-2.19576	-1.51585	-1.73915	-0.50595	1.35082	-1.53151	1.41655	-0.76132	-0.49471	-0.51372	-1.51561	-1.70654	-0.39953	-1.7311	-0.47992
ACSL4	4.40516	4.45789	4.45293	4.96964	5.86266	5.33694	6.44783	5.20594	5.6774	5.16516	5.27809	5.86474	5.742	5.18793	5.95961	5.23483
TMEM164	3.33428	3.30245	3.05294	3.37014	3.71849	2.30092	3.46166	2.33631	4.09764	4.15114	4.14265	4.66444	4.04393	3.97438	3.84076	4.61959
AMMECR1	2.3343	1.95235	1.97238	2.09258	2.1022	1.21157	2.60445	1.26006	3.04278	2.69479	3.39426	3.64625	3.42337	3.29385	3.72873	3.15929
CHRDL1	-3.06131	-3.32193	-3.01793	-3.32193	-2.57975	-2.18371	-3.07727	-2.36441	5.41142	5.84718	5.61281	6.11484	-3.32193	-3.32193	-3.32193	-3.32193
PAK3	-2.28371	-2.73038	-2.54512	-2.43042	0.59422	-2.50556	0.24113	-2.0396	-1.46414	-0.84365	-1.33541	-0.60707	-3.08695	-3.08772	-3.04029	-2.79517
EIF4BP7	1.74565	1.64481	1.8273	1.1425	1.69025	0.90657	1.04125	0.22559	3.29322	3.76976	3.56705	3.0527	0.74525	-0.58185	1.3518	0.82458
ALG13	1.51995	1.79158	1.63671	2.22743	2.02621	1.76824	2.35023	1.70842	1.80465	2.0304	1.90797	2.63884	2.82231	2.90679	1.97715	2.15299
AMOT	-2.97272	-2.72518	-3.03943	-3.16457	-1.39918	-2.37859	-1.93291	-3.03842	-0.8189	-0.44654	-1.12232	-0.59399	2.41356	3.07073	3.2178	3.5744
IL13RA2	-0.4729	0.16081	-0.85623	-0.50929	-3.32193	-3.32193	-2.79031	-2.22738	-3.32193	-1.81547	-2.32993	-2.08468	4.29974	1.67477	1.25012	2.00458
LRCH2	-1.16355	-0.21766	-0.14856	0.85558	-3.22854	-3.10879	-3.32193	-3.32193	-3.32193	-3.32193	-3.32193	-3.12121	-3.32193	-3.32193	-3.32193	-3.11571
PLS3	5.21179	5.43009	5.46088	5.62817	6.24968	4.91621	7.03551	5.0872	5.98583	6.80554	6.58196	7.06614	5.51549	6.13443	6.59312	6.08324
DANT2	-2.2379	-2.44378	-0.15828	0.18498	-2.3575	-0.95682	-2.559	-0.38452	-1.87002	-0.22437	-1.59058	-1.13955	-2.9518	-3.32193	-2.54553	-1.65026
SLC6A14	-1.70816	-1.16195	-1.62696	-1.38913	-2.25575	-0.46396	-1.6973	-0.57379	-3.32193	-3.32193	-3.32193	-3.32193	-3.13984	-2.27067	-3.32193	-2.90495
CT83	4.26452	3.99967	3.68392	3.4126	2.3653	4.09932	2.02362	4.13907	-3.32193	-3.32193	-3.32193	-3.32193	-3.32193	-3.32193	-3.32193	-3.32193
KLHL13	-2.16969	-2.6226	-2.71151	-1.56267	1.14712	-0.59537	1.48409	-0.66936	-2.39265	-2.3477	-2.89388	-2.52487	-1.02377	0.21227	-0.84608	-0.14781
WDR44	2.71064	3.18872	3.18137	3.75067	3.01536	2.20767	3.80874	2.01948	3.18856	3.22401	3.19685	4.04037	2.71866	2.6685	2.59943	2.52599
DOCK11	1.38814	1.86844	2.09974	2.4578	1.10435	-0.36367	2.04663	-0.29651	0.11083	0.08086	-0.70276	0.53257	1.41156	1.07124	1.97701	1.56123
IL13RA1	4.39558	4.54304	4.62683	5.02061	5.91864	5.07467	6.39084	5.02846	4.42195	4.49235	4.34155	5.52015	4.5046	4.05834	4.61707	4.9366
LINC01285	-2.05462	-0.40679	0.19893	-0.34895	-1.79677	-1.45714	-2.23391	-2.03828	-3.32193	-3.06632	-3.32193	-3.32193	-2.48406	-0.6947	-1.59814	-1.41646
LONRF3	2.19983	2.16917	1.90328	1.91116	3.35486	2.14744	3.74635	1.5634	-3.32193	-3.07679	-3.32193	-2.02527	1.91509	1.00297	2.15247	1.99267
PGRMC1	7.66568	7.68784	7.68307	7.60266	6.82809	6.17377	6.82808	5.9239	6.91225	6.69116	6.5383	6.50667	5.9337	6.02194	5.98878	5.92884
SLC25A43	3.18895	3.28097	3.34982	3.39384	4.12986	3.11941	4.10579	2.89713	3.9842	3.68188	3.70676	4.19649	3.38479	2.70023	2.94715	2.42658
SLC25A5-AS1	5.51827	5.25708	5.27685	4.95771	5.56871	4.18594	5.85587	3.88436	5.89829	5.41235	5.3721	6.07233	5.27665	5.13291	5.11268	4.84126
CXorf56	4.69882	4.62332	4.42276	4.12242	3.86671	3.07618	4.05894	3.19977	4.00087	3.83056	4.15891	3.40136	3.3718	3.02764	3.55942	2.99942
UBE2A	4.41661	4.39931	4.39682	4.70179	4.34418	4.05392	4.638	3.91764	4.55824	3.96126	4.16059	4.54775	4.35987	4.21811	3.68557	3.63256
NKRF	3.66007	3.47079	3.21857	3.64175	2.94456	2.33307	3.52796	2.58547	3.0689	2.83728	3.00209	3.31726	2.56465	2.71396	2.47973	2.45707
SOWAHD	-0.51438	-0.17494	0.14688	0.5117	-0.873	-1.30983	-2.76926	-0.65445	4.12763	2.91193	3.78955	0.60617	-2.2007	-1.96493	-3.32193	-2.74855
UPF3B	4.2926	4.13067	4.07658	4.64934	3.9205	4.08413	4.26958	4.73261	4.06448	3.33047	3.50427	3.26011	3.33709	2.64502	2.32376	1.85412
RNF113A	5.43611	5.41693	5.12495	5.19936	4.17822	4.89981	4.12946	4.76482	4.82972	4.86495	4.90357	4.52999	3.27384	3.11319	2.66527	3.28219

NDUFA1	7.40695	7.47175	7.42117	6.99142	7.50605	8.02633	7.10549	7.87268	7.32223	7.4885	7.22363	5.95767	7.10914	7.01151	5.68015	5.80134
NKAP	2.84771	2.70054	2.76007	2.90638	2.94322	3.08067	2.9498	2.80965	2.78653	2.34678	2.6791	2.83907	1.28324	1.09866	1.40775	1.1065
RHOXF1P3	3.07822	3.14521	3.1625	2.85233	3.64664	3.28879	3.13074	2.78944	3.3028	2.84123	3.31478	2.68714	1.55957	1.21699	2.03489	1.62275
NKAPP1	0.05581	-0.7044	-0.51603	-0.11866	-0.73387	-1.64749	-0.6963	-0.9968	-0.32197	-1.23381	-1.32393	-1.25258	-1.61504	-1.31881	-2.07103	-0.87137
EEF1A1P30	2.14029	2.07286	1.08934	1.58775	-2.73569	-1.84155	-3.32193	-1.68687	-3.32193	-2.22549	-3.32193	-2.68083	-3.32193	-3.32193	-2.23311	-3.32193
ZBTB33	2.59585	2.69921	2.60186	3.16217	3.32128	1.80613	3.9242	1.67479	3.52699	3.70858	3.52674	4.4967	2.68965	2.4638	2.46242	2.56467
TMEM255A	0.68598	0.72944	1.83018	2.23164	-3.09434	-2.63469	-3.32193	-3.01243	-3.01878	-3.32193	-3.32193	-3.07011	-3.32193	-3.32193	-3.06846	-2.84411
LAMP2	5.15801	5.19042	5.51753	5.93124	5.82652	5.38124	6.4687	5.20691	5.74805	5.80545	5.46359	6.67331	5.366	5.3163	5.34089	5.35991
CUL4B	3.51559	3.6808	3.79261	4.32288	4.43326	3.46726	5.19565	3.28542	4.92142	5.0039	4.84812	5.66736	3.59009	2.99099	3.81504	3.22713
MCTS1	4.24822	4.42433	4.35658	4.74209	4.19428	4.39686	4.79604	4.30641	4.39612	4.39367	4.13169	4.30817	3.84753	3.9034	3.09365	2.93644
C1GALT1C1	4.39029	4.79804	4.77888	5.37839	5.42244	5.14572	5.91965	4.92626	4.36944	4.32608	4.17928	4.54265	4.13262	3.68888	3.12675	3.2827
CT47A6	1.85911	1.73179	0.45954	1.41491	-3.32193	-3.32193	-3.32193	-3.32193	-3.32193	-3.32193	-3.32193	-3.32193	-3.32193	-3.32193	-3.32193	-3.32193
GLUD2	0.49318	0.71148	0.9315	0.00565	5.37772	3.53468	5.596	3.16187	1.39674	1.63322	1.00201	0.97035	0.39997	0.92479	1.27406	1.39044
HSPA8P1	0.1028	-2.76097	-0.73305	-2.27297	-1.12138	-1.67092	-0.52938	-2.31672	-0.75594	0.61263	-1.46125	-0.28189	-1.32719	-1.4563	-1.23989	-1.21572
THOC2	3.90108	3.90867	3.9126	4.45435	4.24362	4.45887	4.87795	4.66236	3.92168	3.97993	4.03597	4.72655	3.81078	4.31873	4.04847	4.12867
FERP1	3.90463	3.99264	3.92349	4.22932	3.41385	3.61724	4.15569	3.43425	3.59036	3.79484	3.78528	4.47734	3.65513	4.47616	4.40086	4.3056
XIAP	3.83949	4.31797	4.23309	4.79502	3.6476	3.20627	3.811	3.47856	3.02267	3.15222	2.48916	3.6965	3.82655	3.79865	3.70576	3.96272
STAG2	3.7715	4.20757	4.0915	4.57697	3.9448	2.83109	5.05059	2.75784	3.80457	4.75897	4.53837	5.5383	3.83119	5.05576	5.01321	5.11452
TENM1	-0.23058	0.0267	0.2082	0.00591	-2.96448	-2.48113	-2.84348	-2.48406	-2.96599	-2.66169	-2.5161	-2.14869	-3.32193	-3.32193	-3.24133	-3.08781
TEX13C	0.54175	0.41254	0.94316	0.62505	-1.7343	-2.62944	-1.81173	-1.92651	-3.32193	-3.32193	-3.32193	-3.32193	-3.32193	-3.32193	-3.32193	-3.32193
MTCO1P53	-1.20723	-1.51768	-2.11065	-3.32193	0.95538	4.27866	0.04654	4.03068	-3.32193	-2.27182	-3.32193	-2.28448	-3.32193	-3.32193	-3.32193	-3.32193
SMARCA1	3.97439	4.48557	4.62487	5.23533	6.39978	5.65294	6.7464	5.6046	5.49879	5.8202	5.55739	6.4905	4.42946	4.89336	4.85914	5.12897
OCRL	3.13574	2.98061	2.84085	3.33912	4.35732	3.32177	4.15997	3.50609	4.07067	3.75391	3.08565	4.28562	4.11291	4.02567	3.37265	3.48551
APLN	2.2624	2.27992	2.15729	-0.01956	-2.4558	-2.52739	-3.02792	-2.95677	4.66862	4.45199	3.15058	1.03655	4.25191	2.61596	2.37475	1.7988
XPNPEP2	-3.32193	-3.32193	-3.32193	-2.96586	0.13525	1.72514	0.23431	1.6597	-3.32193	-3.32193	-3.32193	-3.32193	-3.32193	-3.32193	-3.04488	-3.32193
ZDHHC9	3.45684	3.47237	3.48558	3.64051	5.41446	4.78002	5.59878	4.65275	3.81824	4.07538	3.94526	4.54813	3.98947	4.14375	4.59423	4.59408
UTP14A	4.79735	4.4796	4.21146	4.44278	4.27738	3.1621	4.56749	3.37218	4.80965	4.7352	4.85204	4.745	4.25537	3.90245	4.22932	3.8542
BCORL1	2.56889	2.81553	2.85621	3.02036	3.02769	1.97717	2.7037	1.89727	3.26806	3.4819	3.45544	3.95794	2.44312	2.63394	3.63306	3.9032
ELF4	3.44248	3.28208	3.23745	2.94151	4.01377	3.0936	3.94407	2.93144	6.13608	4.86175	5.7457	5.06148	4.14648	3.45301	4.55148	4.12415
AIFM1	5.33323	5.13957	4.9988	5.32265	5.69145	5.50123	5.77196	5.43711	5.50107	4.95318	5.17074	4.98754	5.36413	5.2353	5.2013	5.27521
ZNF280C	0.84124	0.95773	0.66736	0.62081	-0.07938	-0.63962	0.3118	-0.29133	1.29607	1.15824	1.10254	2.10153	1.71164	1.64163	1.39315	1.26575
SLC25A14	2.57412	2.40113	2.2182	2.70368	1.90184	2.41028	2.38634	2.29765	1.63139	2.05457	1.82656	2.07311	1.81353	2.07299	0.97936	1.06246
RBMX2	3.89116	4.10733	4.03372	4.00974	3.39968	3.80657	3.67585	4.05876	3.7471	4.15107	4.05814	4.09454	2.80393	3.13472	2.80149	2.67668
ENOX2	1.59465	1.12203	1.00896	1.25685	1.71771	0.05001	1.67731	0.09869	1.18366	-0.45472	0.38133	0.26358	1.15799	-1.01026	-0.02448	-1.13923
IGSF1	-1.33208	-1.7482	-1.18753	-1.30151	0.47535	-0.20438	0.26229	-0.38139	-3.32193	-3.32193	-3.16614	-3.32193	-3.32193	-3.32193	-3.17776	-3.17473
FIRRE	-0.07701	-0.51445	-0.63036	-0.12888	-2.10097	-1.19923	-1.87789	-1.64715	-2.42885	-1.94346	-1.98404	-1.21072	-3.32193	-3.32193	-3.32193	-3.32193
STK26	3.98718	3.79613	3.36048	4.05607	3.52358	1.87036	3.99783	1.94229	4.40513	4.06576	3.54576	3.99244	4.46887	4.42545	3.5886	3.69728
RAP2C	3.38762	3.42498	3.3807	3.76451	4.08588	3.04542	4.38505	2.799	4.52586	3.99693	4.36871	4.10122	3.83389	3.10253	3.82858	3.01146
RAP2C-AS1	0.0312	0.24364	0.2847	0.36901	1.61119	-0.51625	1.66595	-0.74656	1.7399	1.16202	1.76426	1.72438	0.8906	0.27945	1.2535	0.75278
MBNL3	-2.72836	-2.66999	-3.02783	-2.67834	-1.43361	-3.23199	-1.78568	-3.11861	-3.219	-3.15496	-3.32193	-3.08199	-3.32193	-3.12155	-3.32193	-3.15307
HS6ST2	-2.3726	-2.41829	-2.12862	-1.66445	-1.43369	0.36993	-1.53024	0.47701	-3.32193	-3.32193	-3.32193	-3.32193	-2.75027	-1.76709	-1.63392	-1.75223
TFDP3	1.42604	1.53607	0.88862	1.96007	-3.32193	-3.32193	-3.32193	-3.32193	-3.32193	-3.32193	-3.32193	-3.32193	-3.32193	-3.32193	-3.32193	-3.32193
GPC4	-1.51308	-0.75691	-0.92584	-1.01429	-1.64699	-2.61455	-1.64796	-2.24012	-3.32193	-3.32193	-3.32193	-3.32193	1.34454	3.2908	3.44643	3.56777
GPC3	-2.95872	-3.32193	-2.57489	-3.32193	5.55708	6.6591	5.29066	6.82945	-3.32193	-2.68768	-3.32193	-3.32193	-3.02999	-1.62823	-2.07908	-1.3983
PHF6	2.83571	2.98036	2.72581	3.13191	2.68908	2.04384	3.39637	2.01792	3.3192	3.29424	3.10021	3.78506	2.71261	2.8199	2.61738	2.42806
HPRT1	6.43472	6.49617	6.31662	6.45054	4.88215	5.22135	5.14875	5.29307	6.42313	6.05745	5.84871	6.21196	5.87699	5.87185	5.10868	4.9724
MIR503HG	-2.37703	-1.10967	-1.91926	-3.32193	0.91654	2.1073	-0.79858	1.81181	3.46123	3.5146	4.82628	2.68123	2.371	2.58144	1.77061	1.41834
PLAC1	-1.38251	-1.42355	-2.75643	-1.71527	-3.09291	-1.37075	-2.22814	-1.04264	-0.80884	0.10639	-0.29801	-1.43907	-0.44479	1.08663	-1.97314	-0.52919
FAM122B	3.71214	3.60946	3.53915	3.79401	3.24916	3.41979	2.96627	3.07409	4.03431	3.70966	3.79531	3.38885	3.35334	3.72513	3.28657	3.31532
FAM122C	2.04133	1.63927	1.762	0.94752	1.72443	1.20795	0.21048	0.86618	2.46021	2.23552	2.44194	0.47069	0.51681	0.67879	1.10384	0.93499

MOSPD1	3.99997	4.26178	4.24572	4.8561	2.8401	3.12992	3.87751	3.13124	2.5275	3.11458	2.07647	3.31423	3.70415	3.77022	2.87925	3.36754
SMIM10	3.63564	3.77729	3.73656	4.08235	2.29484	2.68593	2.71407	2.83423	3.87437	4.41905	3.79898	3.87871	-3.32193	-3.32193	-3.32193	-3.32193
RTL8B	0.52685	1.22395	1.89947	-0.66209	3.72747	5.13715	2.98752	4.87315	5.45225	5.04661	5.38983	4.49926	-2.18412	-1.69277	-2.02152	-1.4672
RTL8C	7.07841	7.44258	7.73813	7.11612	7.00047	8.12431	5.95409	7.89218	7.97392	8.08254	8.10758	7.18865	6.08545	6.42073	6.58643	6.55539
RTL8A	5.70398	5.80246	5.95994	5.60015	5.7573	7.12592	5.14945	6.90598	6.50595	6.53526	6.58987	6.01092	5.44147	5.7873	5.39172	5.41715
SMIM10L2B	-1.49325	-1.78108	-2.09308	-1.37191	0.58208	1.46799	-0.20488	0.91653	-2.91093	-2.25583	-1.45648	-2.97844	-2.09979	-0.82403	-0.41549	-0.23931
CT55	3.78786	3.532	3.22301	3.50554	-0.7964	-1.26137	-0.83622	-2.27511	-3.32193	-3.32193	-3.32193	-3.32193	-1.85972	-3.32193	-3.32193	-3.32193
ZNF75D	-1.17967	-0.56731	-0.34429	0.06806	0.69407	0.33604	0.61755	0.42809	-0.20536	0.49167	0.01133	0.99148	-0.19139	0.76494	0.04899	0.45017
ZNF449	0.12456	-0.15065	0.13825	0.5643	0.62381	-0.04598	0.82525	0.44818	0.75788	1.30627	0.83954	1.87242	0.81075	1.03924	0.75265	0.74614
SMIM10L2A	-3.32193	-3.32193	-3.32193	-3.32193	-0.2452	-0.18407	-1.11981	-0.93342	-1.70374	-1.66818	-1.73769	-2.55351	-3.32193	-3.32193	-3.32193	-3.32193
INTS6L	-2.8043	-2.43616	-1.77721	-2.82763	0.82215	1.75396	1.30268	1.59745	0.58946	0.61904	0.42301	1.29433	-3.32193	-3.32193	-3.18047	-3.32193
CT45A1	3.19836	2.58906	2.80119	2.86249	4.37836	3.72767	4.65828	3.5782	-3.32193	-3.32193	-3.32193	-3.32193	-3.32193	-3.32193	-3.32193	-3.32193
CT45A3	4.71109	4.02787	4.26773	4.14943	6.25602	4.95376	6.69592	4.84994	-3.32193	-3.32193	-3.32193	-3.32193	-3.32193	-3.32193	-3.32193	-3.32193
CT45A5	4.00092	3.43691	3.55362	3.69236	5.54329	4.62264	5.82995	4.5384	-3.32193	-3.32193	-3.32193	-3.32193	-3.32193	-3.32193	-3.32193	-3.32193
CT45A6	4.27935	3.54872	3.77098	3.87252	5.76843	4.88186	6.09663	4.79774	-3.32193	-3.32193	-3.32193	-3.32193	-3.32193	-3.32193	-3.32193	-3.32193
CT45A2	2.39787	2.04355	1.62902	1.87189	5.37751	4.24266	5.57435	4.0402	-3.32193	-3.32193	-3.32193	-3.32193	-3.32193	-3.32193	-3.32193	-3.32193
CT45A7	4.19971	3.4378	3.66928	3.82159	5.69458	4.74174	6.06984	4.71572	-3.32193	-3.32193	-3.32193	-3.32193	-3.32193	-3.32193	-3.32193	-3.32193
CT45A8	2.66092	2.39917	1.93831	2.02762	5.71371	4.6236	5.85583	4.38811	-3.32193	-3.32193	-3.32193	-3.32193	-3.32193	-3.32193	-3.32193	-3.32193
CT45A9	2.65811	2.39637	1.93553	2.02483	5.72603	4.63434	5.86189	4.38527	-3.32193	-3.32193	-3.32193	-3.32193	-3.32193	-3.32193	-3.32193	-3.32193
CT45A10	3.13195	3.00348	1.9694	2.43959	5.61248	4.48747	5.81313	4.72087	-3.32193	-3.32193	-3.32193	-3.32193	-3.32193	-3.32193	-3.32193	-3.32193
SAGE1	-2.7125	-2.65291	-3.32193	-3.32193	3.45429	4.15794	3.5368	3.69467	-3.32193	-3.32193	-3.32193	-3.00097	-3.32193	-3.32193	-2.99892	-3.32193
MMGT1	3.3225	3.3525	3.08961	3.53689	4.01337	2.28877	4.59059	1.94626	3.70974	3.16826	3.26409	4.26249	3.97114	3.45626	3.75919	3.68765
SLC9A6	2.21668	2.28952	2.21064	2.59616	3.26539	2.45311	3.86544	2.4986	2.50492	2.67735	2.58245	3.0783	2.44712	2.89486	2.9961	3.23746
FHL1	3.45734	3.67102	3.97909	3.91317	-0.67195	-0.43212	-0.77789	-0.68185	3.19233	3.27008	2.97992	3.36564	2.68636	2.36214	2.17745	2.19246
MAP7D3	3.38782	2.9846	3.0136	3.57101	1.11831	1.53515	1.58057	1.48745	3.80488	3.08533	3.39524	3.26072	2.54779	1.29727	1.7353	1.13084
HTATSF1	4.93268	4.83094	4.80954	5.26669	4.84879	4.21854	5.19833	4.26556	5.34047	4.76737	4.96106	5.06144	4.35051	4.47512	3.81158	4.26332
ARHGEF6	-0.11987	0.3072	0.47113	1.10822	-3.32193	-3.1559	-3.16799	-3.32193	-2.44122	-2.55815	-2.73711	-3.16572	-3.32193	-3.07299	-3.16466	-3.32193
RBMX	5.28583	5.06147	4.94127	4.92834	4.92761	4.26235	5.22096	4.4445	5.55132	4.75478	5.2408	5.15429	5.65212	5.32317	5.82789	5.20809
FGF13	1.05762	0.85946	0.68056	0.99298	-3.29912	-3.2682	-3.27225	-3.32193	-2.67385	-2.95511	-2.97467	-2.67188	-3.19998	-3.32193	-3.32193	-3.21995
ATP11C	1.89742	1.9233	1.87781	2.18412	2.11654	0.37534	1.92491	0.89318	3.06099	2.87272	2.14765	3.48868	2.81991	2.49718	2.0362	2.56765
HNRNPA3P3	2.5103	3.16791	2.88328	3.46517	-1.1588	-0.61677	-1.25641	-1.23529	0.68483	0.69058	-0.07619	-0.42062	-0.65569	0.28572	-0.18105	-0.65994
LINC00632	-1.1995	-0.49785	-0.92152	-0.35104	-3.03604	-2.11755	-3.32193	-2.38032	-0.18481	-0.9352	-0.33105	-1.17644	2.33697	2.62357	0.6805	0.85773
CDR1	-3.32193	-3.32193	-3.32193	-2.4785	-3.32193	-3.32193	-3.32193	-3.32193	-0.99353	-0.77253	-0.93325	0.82387	0.01711	0.69835	-0.33854	-0.54938
SPANXB1	10.6004	10.5096	10.8844	10.2582	0.01958	-0.73229	-0.47819	0.11655	-1.77422	-0.43771	-1.17507	-1.27315	0.48176	-1.03638	-0.45011	-0.14011
LDOC1	2.63826	2.78922	3.29585	2.12327	3.77411	5.34189	3.05938	4.95648	6.17559	6.96534	6.82151	6.52695	-1.85666	-1.64675	-3.32193	-2.25369
SPANXC	4.24424	3.74208	4.78978	3.03589	-1.88711	-3.32193	-1.79987	-3.32193	-3.32193	-3.32193	-3.32193	-3.32193	1.62536	0.25808	-1.77856	-0.17979
SPANXA2-OT1	0.52102	0.6292	0.3783	0.73901	-3.32193	-3.32193	-3.32193	-3.32193	-2.2722	-2.79196	-2.35568	-2.79948	-2.53845	-1.83817	-2.79636	-2.0907
SPANXA1	7.28331	7.25519	7.71935	7.05329	-3.32193	-2.22727	-3.32193	-3.32193	-3.32193	-2.26321	-3.32193	-2.27594	6.5772	5.55486	4.53997	4.69253
SPANXA2	7.31622	7.24443	7.71935	7.01426	-2.75924	-2.22727	-2.28741	-3.32193	-3.32193	-2.26321	-3.32193	-2.27594	6.57986	5.54134	4.58578	4.70471
SPANXD	5.07345	5.14899	5.37471	4.99319	-2.7531	-3.32193	-3.32193	-3.32193	-3.32193	-3.32193	-3.32193	-3.32193	2.34842	1.93778	0.79058	0.51868
MAGEC1	4.88042	4.63259	4.44522	4.52929	-2.01934	-2.01731	-2.43572	-2.12209	-3.32193	-3.08893	-3.32193	-3.32193	-2.80875	-3.32193	-2.8921	-2.8839
MAGEC2	6.98141	7.04933	6.96231	7.38006	1.72761	1.77007	2.0107	2.04745	0.13334	-0.29654	-0.20546	-0.25311	-0.92263	0.10269	-1.62903	-1.44896
SPANXN4	1.83876	1.8968	1.29507	1.76216	-3.32193	-3.32193	-3.32193	-3.32193	-1.36334	-2.18609	-1.04119	-1.57586	-3.32193	-3.32193	-3.32193	-3.32193
SLITRK4	-2.80054	-3.06437	-3.05134	-2.49867	-2.40547	1.40758	-2.52994	1.71337	-3.32193	-3.20562	-3.32193	-3.32193	-3.32193	-2.82848	-2.90778	-3.0955
FMR1	2.81323	2.68612	2.80005	3.07844	2.32496	1.63036	3.06433	1.61827	3.49449	2.65875	2.97294	3.26838	3.72264	3.46389	3.48406	3.1446
FTH1P8	6.21117	6.24199	6.21604	5.62848	2.15729	3.25783	2.44783	2.7149	3.33688	3.95737	2.86699	4.68497	0.99473	1.71483	1.64498	1.80285
IDS	3.30342	3.15886	3.60438	3.37436	3.37528	2.27683	3.90758	1.5854	3.4106	3.07798	3.34404	3.7221	3.28083	1.10695	2.96854	1.73809
LINC00893	-0.42265	-0.45711	-0.34218	-0.41032	0.27103	0.71889	-0.99415	0.10209	-0.5655	-0.34872	-0.55633	-0.71223	-2.08946	-1.72866	-2.0355	-0.88545
CXorf40A	2.8985	2.55423	2.59389	2.55519	2.39847	2.22927	2.11069	2.27011	2.11852	2.61304	2.23984	1.86778	1.04997	1.51635	0.84577	1.55682
HSFX3	1.71936	1.28878	0.82629	1.23137	0.75765	1.40346	0.90825	1.44846	-0.42985	0.14609	-0.7158	0.52487	-1.77391	0.14611	-2.20259	-0.51622

MAGEA9B	0.30013	0.54638	0.42706	0.51206	0.19748	-0.76075	-0.22726	0.01868	-3.32193	-3.32193	-3.32193	-3.32193	-3.32193	-3.32193	-3.32193	-1.9562
HSFX2	-0.58081	-1.25466	0.06083	0.47717	-0.23686	0.86762	-1.45256	1.2214	-1.45656	0.12024	-0.9649	0.02337	-1.4451	-1.00152	-1.05889	-1.40571
TMEM185A	2.1871	2.23074	2.14209	2.32215	2.38724	2.01447	2.68897	2.15347	2.5751	2.67744	2.59428	2.82279	2.06712	2.39734	2.48969	2.70416
MAGEA11	1.51352	1.02562	1.23609	1.41854	-2.42961	-2.39147	-2.81791	-2.98118	-3.32193	-3.32193	-3.02088	-3.32193	-3.32193	-3.32193	-3.32193	-3.32193
TMEM185AP1	0.30705	1.12042	0.96528	1.48114	1.18481	-0.05541	1.65616	0.69745	1.54044	2.07456	1.8616	2.49183	1.28605	0.51761	0.24576	0.70122
HSFX1	-0.70372	-1.44905	0.11591	0.26071	-0.31248	0.54514	-1.21038	0.80399	-1.41219	0.07471	-1.22544	0.20098	-1.4006	-1.18604	-1.06647	-0.47929
MAGEA9	0.32792	0.66784	0.45124	1.0317	0.39776	-0.5943	-0.21959	0.09341	-3.32193	-3.32193	-3.32193	-3.32193	-3.32193	-3.32193	-3.32193	-1.90504
CXorf40B	4.02213	4.06743	3.77571	3.79544	4.29056	3.85668	4.09896	3.8307	3.93972	4.07076	3.83105	3.25438	3.12153	3.44547	2.42143	3.00161
HSFX4	2.41794	2.22083	1.28083	2.31682	1.85112	2.20377	1.98362	1.78767	0.48056	0.80196	0.47531	1.26258	-0.20039	0.35269	-2.15092	0.46401
LINC00894	-1.48291	-1.46688	-1.35535	-1.46028	-1.22289	-0.49795	-1.41511	-0.07891	-2.22338	-2.2756	-1.98514	-1.92737	-1.72766	-1.68593	-2.36771	-1.36781
XRCC6P2	0.37041	-0.20926	-0.22265	-1.06968	-0.12957	-0.05683	0.18118	-1.3076	0.30895	0.99241	0.31884	-0.27426	-1.44625	-0.6619	-1.23325	-0.84358
MAMLD1	1.85486	1.67792	1.32626	1.35065	-0.21177	-1.81405	-0.68302	-1.46038	3.12358	2.84239	2.44464	3.23059	2.69335	3.33427	3.25207	3.44864
MTM1	1.45819	1.82738	1.47446	2.11125	2.72078	1.62741	3.46361	1.76978	1.6265	1.9914	1.67044	2.47972	1.81006	1.99721	1.24458	1.56882
MTMR1	2.55614	2.52666	2.08063	2.09365	2.49741	1.64531	2.81155	1.71488	4.21095	3.78356	4.09814	4.25565	3.07513	3.11222	3.06587	3.00102
CD99L2	3.06297	3.23464	3.43824	3.65122	2.86721	3.26188	2.52554	2.89172	2.97269	3.51755	3.58022	3.56029	2.98731	2.98031	3.90365	3.79583
HMGB3	4.67434	4.49597	4.54379	4.63646	3.83377	4.04253	4.04754	3.94596	4.84795	3.7217	4.02085	3.08146	5.65416	6.10266	5.41451	5.6735
VMA21	4.31097	4.35102	4.13254	4.34841	3.78292	3.46012	4.51201	3.23625	4.05857	3.73002	3.77412	4.76034	5.14758	4.98605	4.39232	4.27369
PASD1	4.04917	3.91528	3.57192	3.75314	-2.19049	-2.88432	-1.60383	-2.15164	-3.32193	-3.32193	-3.32193	-3.32193	-3.32193	-3.32193	-3.32193	-3.09418
MAGEA4-AS1	-3.32193	-2.69132	-3.32193	-3.32193	4.00842	4.23772	3.39164	3.96988	-3.32193	-3.32193	-3.32193	-3.32193	-3.32193	-3.32193	-3.32193	-3.32193
MAGEA4	-2.9978	-2.9627	-2.00451	-3.32193	8.73814	7.85782	8.56508	7.60446	-2.65689	-3.0082	-2.71604	-2.75866	-3.32193	-3.32193	-3.32193	-3.32193
GABRE	-1.58138	-1.78428	-1.33443	-1.03699	-2.22995	-1.64194	-2.18054	-1.26213	-3.32193	-3.32193	-3.32193	-3.32193	-3.32193	-3.15064	-3.21479	-3.32193
MAGEA10	-2.97969	-2.64297	-2.92448	-2.90188	4.23993	4.27998	4.55095	4.31976	-3.32193	-3.32193	-3.32193	-3.32193	-3.32193	-3.32193	-2.99354	-3.32193
GABRA3	0.57361	0.88374	1.34149	1.37866	1.13165	0.62391	1.42851	0.31594	-0.46606	-0.7811	-0.37496	-0.55839	-2.80084	-3.12722	-3.32193	-3.32193
MAGEA3	7.09169	7.13955	6.94139	7.11507	7.66807	7.30946	7.66227	7.15213	-0.50337	-0.22865	-0.45494	-1.0882	-3.32193	-3.32193	-2.82171	-2.81239
CSAG2	3.44907	3.2496	3.26439	3.47731	5.09928	6.01802	4.85122	5.75181	-3.32193	-3.32193	-3.32193	-3.32193	-3.32193	-3.32193	-3.32193	-3.32193
MAGEA2B	3.87266	3.78696	3.85682	3.74078	6.92235	5.82202	6.77659	5.648	-2.51875	-2.62771	-2.3249	-3.32193	-3.32193	-3.32193	-2.93683	-3.32193
CSAG1	4.86637	4.69302	4.73464	4.74135	5.93818	6.48488	5.59686	6.33835	-1.91471	-2.56887	-2.01593	-3.32193	-3.32193	-3.32193	-3.32193	-3.32193
MAGEA12	4.56726	4.67613	4.57056	4.68744	6.20444	5.87234	6.22537	5.81153	-2.77172	-2.21387	-3.32193	-2.50853	-3.32193	-3.32193	-2.85583	-3.32193
CSAG4	3.05725	2.88146	3.04609	2.36768	5.06039	4.12576	4.78363	4.05365	-3.32193	-3.32193	-3.32193	-3.32193	-3.32193	-3.32193	-2.33536	-3.32193
MAGEA2	3.93559	3.871	3.90233	3.81477	6.99905	5.8832	6.84773	5.7135	-2.47864	-2.59178	-2.27814	-3.32193	-3.32193	-3.32193	-3.32193	-3.32193
CSAG3	2.80318	2.4566	2.5953	2.74507	4.53422	5.4502	4.38571	5.23611	-3.32193	-3.32193	-3.32193	-2.57401	-3.32193	-3.32193	-3.32193	-3.32193
MAGEA6	6.82652	6.89038	6.67339	6.84234	7.26425	6.46643	7.1584	6.14383	-2.34485	-1.24681	-0.79906	-0.61505	-3.32193	-2.91571	-2.83883	-3.32193
CETN2	4.81959	4.83161	4.86984	5.26748	4.19439	4.71091	4.45987	4.57872	5.1955	5.25183	5.14655	5.46522	5.51022	6.02289	4.95959	5.3793
NSDHL	4.50408	4.66005	4.25742	4.57937	5.14866	5.50556	5.20001	5.30393	5.26065	4.83019	4.96891	4.12003	5.58275	6.05692	5.72495	6.06113
ZNF185	2.16058	2.61975	2.88367	2.42039	0.43675	-0.68539	0.16834	-0.83544	5.67347	5.04552	5.37632	4.83721	1.15239	0.36012	1.63358	1.03683
PNMA3	-3.32193	-3.32193	-3.32193	-3.32193	-1.35763	-0.83493	-2.08739	-0.58535	-3.32193	-2.83716	-3.04154	-3.32193	-2.91653	-0.62235	-2.4813	-0.34544
PNMA6A	-1.5314	-0.70685	-1.69842	-1.4496	2.08179	1.85473	1.65536	1.90137	1.41838	1.88412	1.64199	0.84693	2.16238	2.87594	2.702	3.40018
PNMA6B	-2.12435	-1.35707	-2.50125	-1.53557	1.9395	1.44133	1.49403	1.01662	1.89084	2.13861	2.01901	1.24403	1.69667	2.39229	2.12458	3.03189
MAGEA1	5.00645	5.14789	4.94753	5.22939	0.23813	-0.7255	-0.01495	-0.91085	-1.09144	-1.64912	-1.07053	-2.42621	5.71359	6.10732	5.71992	5.79119
ZNF275	0.50177	0.36518	-0.0274	0.55811	1.37108	0.66428	1.2822	1.06777	1.87105	1.58772	0.88635	1.9756	2.06572	1.56849	1.56943	1.81741
ZFP92	-3.14928	-3.32193	-2.78405	-3.1072	-2.46603	-3.14748	-2.44956	-2.94074	1.56887	1.50894	1.84926	1.78676	1.18345	1.32859	1.95412	2.07259
TREX2	3.98911	3.2334	3.02863	2.3652	4.04671	3.87166	3.40054	3.85284	4.41273	3.52855	4.01352	2.4142	3.93818	4.12795	3.82333	3.7237
HAUS7	3.45101	2.70276	2.49525	1.84059	3.51799	3.35687	2.84844	3.37612	3.85712	2.96261	3.46489	1.87918	3.41177	3.61518	3.30257	3.21429
BGN	-2.35905	-3.32193	-3.32193	-0.60124	-2.97852	-2.60841	-3.32193	-3.32193	-2.52655	-1.82082	-2.33403	-1.55659	5.37745	6.28176	6.31374	7.26376
CCNQ	3.88898	3.5769	3.47963	3.01533	4.59821	4.80948	4.44573	4.59487	4.43539	3.82372	4.46483	3.84271	4.5157	3.77117	3.89503	3.59641
DUSP9	-0.60129	-0.80918	-1.10671	-1.85479	4.60284	5.61147	3.27077	6.14292	0.14721	0.42241	-0.75543	-0.86099	0.49998	0.44981	1.03464	0.34845
PNCK	-1.30436	-0.20092	0.19855	-0.69054	0.00927	-0.17446	-0.06822	-0.44489	-1.13796	-0.44983	-1.06182	-0.90627	-0.64962	-0.65396	0.35498	0.74585
SLC6A8	2.70241	3.70891	4.14516	2.98933	3.46642	4.13033	2.85636	3.98175	2.7609	2.95676	2.18447	2.30969	2.62303	2.99823	4.0446	4.16285
ABCD1	3.04918	3.67778	3.59061	3.59847	4.879	5.19602	3.91412	5.28479	3.32337	3.93261	3.74982	3.43621	1.09741	1.64156	2.16795	2.64501
PLXNB3	1.03523	1.93721	2.00378	1.67365	-2.91127	-1.48367	-2.53639	-1.56859	2.3637	3.44604	2.48741	2.89729	-1.87012	-0.71276	-2.01128	0.27897

SRPK3	0.63789	1.02011	1.13308	0.92357	-0.80471	-0.30174	-1.89979	-0.28635	1.18133	2.01014	0.94888	1.43592	-2.49898	-0.90449	-1.79868	-0.37606
IDH3G	5.68379	5.63424	5.61675	5.30563	5.20823	5.64407	4.75225	5.46711	5.91878	5.8177	6.02291	5.40687	5.51932	5.55786	5.53464	5.35243
SSR4	5.03199	5.05718	5.03267	4.91126	6.02899	7.55541	5.46234	7.28378	5.45646	5.59341	5.49001	4.84845	5.46335	5.21074	5.30404	5.41411
PDZD4	-3.32193	-3.05808	-2.61225	-3.02832	-0.8479	-0.85747	-1.81819	-0.66284	3.01809	3.61728	2.68578	2.25639	-2.33608	-1.75928	-1.63528	-1.86526
AVPR2	-2.064	-0.7188	-1.70806	-1.45998	-3.12312	-3.32193	-3.32193	-2.82238	0.59957	2.67957	1.9441	1.8463	-3.32193	-3.32193	-3.32193	-2.90025
ARHGAP4	3.97834	3.90925	3.8938	3.67354	4.12901	4.37368	3.7713	4.27599	5.98371	6.6333	6.58677	5.89005	4.45744	4.05345	3.88369	3.756
NAA10	5.01903	4.89748	4.80949	4.43355	4.92666	5.55146	4.41107	5.59512	5.19856	4.87039	4.9562	4.24307	5.13202	4.75188	4.45381	4.37516
RENBP	1.91069	3.09927	2.67869	3.4096	-1.85586	-0.57298	-1.52892	0.39142	-2.7973	-2.87426	-2.84594	-2.04053	-2.19603	-2.65374	-2.87807	-2.24838
HCFC1	5.04258	4.91224	4.84778	4.11754	5.67719	4.64532	4.8948	4.68025	5.58863	5.2232	5.68369	5.01655	4.65809	4.9276	5.37201	5.62745
TMEM187	1.52777	2.18177	1.84346	1.89359	1.88665	2.02013	0.87349	1.88693	2.95285	3.22618	2.51088	2.67164	0.95081	1.53735	1.09843	1.95571
MECP2	1.88109	1.70229	1.88739	1.69458	2.3692	1.77933	2.3808	1.73852	2.71136	2.11159	2.59049	2.38841	2.83895	2.15953	3.39083	2.74466
TKTL1	-0.13921	0.07677	-0.61217	-0.59138	-3.32193	-3.32193	-3.32193	-3.32193	-3.32193	-3.32193	-3.32193	-3.057	-3.32193	-3.10037	-3.32193	-2.6237
EMD	5.28619	5.37353	5.46029	4.88468	5.47418	5.67543	5.00088	5.64923	6.46001	5.98007	6.0833	5.3108	6.04419	6.02117	5.76043	5.37352
SNORA70	6.57795	5.78001	6.06281	6.13952	6.08699	6.91197	5.44519	7.59704	4.78126	5.13035	5.8986	5.29794	5.62039	5.43242	3.0589	4.03297
DNASE1L1	2.77931	3.35989	3.54561	3.40405	5.70026	5.54442	5.32723	5.1572	4.41935	4.67686	4.59001	4.29202	2.15634	2.67989	2.52848	3.31173
TAZ	3.15571	2.80758	3.01879	2.47999	3.90552	4.68915	3.03335	4.73733	3.21292	3.20404	3.15458	2.33799	3.04485	3.31744	3.39103	3.5777
ATP6AP1	5.58005	5.6553	5.91965	5.68751	6.85581	7.21724	6.51786	7.1883	5.6747	5.50404	5.79984	5.35439	5.66624	5.70534	6.03557	6.31455
GDI1	4.99446	5.16656	5.34399	5.14897	4.77077	5.22649	4.60802	4.90907	4.7756	4.9882	5.13589	4.65531	5.0189	5.1474	5.09012	4.81686
FAM50A	6.20815	6.34592	6.39398	6.09878	6.49017	7.18445	6.28997	7.12621	6.0603	5.99359	6.04848	5.47007	6.05205	6.22817	6.27273	6.20445
MIR6858	4.12729	4.08766	4.9848	3.1496	3.51291	5.18617	0.24036	4.6381	1.48178	-3.32193	2.2928	-3.32193	2.87973	1.49203	1.20911	1.23931
PLXNA3	1.12947	2.301	2.74664	2.20209	2.49633	2.4385	1.8926	2.33196	1.76438	3.1383	2.55947	3.31499	2.03863	2.25461	2.75232	3.21279
LAGE3	6.90879	6.90778	7.01191	6.74088	7.36524	8.09872	6.63986	7.8237	6.62327	6.46254	6.48352	4.94131	6.26411	6.43641	6.21335	6.12625
UBL4A	5.51392	5.46444	5.44946	5.13569	6.20422	5.68174	5.7336	5.4482	5.56373	5.52537	5.58601	5.21272	5.09596	5.33926	5.04845	5.1127
SLC10A3	4.54508	4.76548	4.82035	4.52306	5.9733	6.29401	5.33404	6.10929	4.78037	4.89772	5.0731	4.2198	5.11275	5.16296	5.27488	5.33555
FAM3A	3.76755	3.88706	3.8822	3.35178	5.09729	5.82915	4.32567	5.65803	3.84781	4.18012	4.21474	3.6352	3.52383	3.61842	3.9317	4.05747
IKBKG	4.99555	5.03485	5.16066	4.78586	5.60717	6.12537	5.32148	5.59268	5.46587	5.15557	5.46474	4.63383	5.59524	5.90607	6.41425	6.62492
CTAG1A	2.86605	2.3994	2.59125	1.30336	2.20616	3.14922	2.27567	2.78373	-3.32193	-3.32193	-3.32193	-2.52206	-3.32193	-3.32193	-3.32193	-3.32193
CTAG1B	3.2136	2.80646	2.93639	1.65232	2.52302	3.44256	2.61473	3.10598	-3.32193	-3.32193	-3.32193	-2.52514	-3.32193	-3.32193	-3.32193	-3.32193
IKBKGP1	5.47377	5.09926	5.35368	5.03517	5.12918	6.2016	4.35687	5.91668	5.47547	5.2363	5.6526	4.70152	5.1823	5.55007	5.51346	5.729
CTAG2	8.32337	8.29173	8.19605	7.35136	7.86845	8.63084	6.86558	8.48123	-0.8514	-1.99575	-2.46619	-2.01049	-3.32193	-3.32193	-3.32193	-3.32193
GAB3	-2.01616	-2.21314	-1.27252	-1.54963	-1.32153	-1.33028	-1.52553	-1.33036	-2.43005	-2.04716	-2.38456	-0.98644	-3.16445	-2.89457	-2.23485	-2.03704
DKC1	5.73807	5.2786	4.95374	5.19031	5.63028	5.31377	5.87202	5.51954	5.61961	5.03766	5.42844	4.63432	6.09983	5.80033	5.46576	5.11606
SNORA56	2.1082	1.79761	0.60934	1.96258	3.77005	4.81158	3.7023	4.80779	0.58366	1.84035	0.97804	-3.32193	2.26707	1.76029	2.68226	2.26213
MPP1	3.51889	3.14304	3.25403	3.05436	3.67737	3.46034	3.91103	3.36163	3.39217	2.60356	2.95581	2.61513	3.18818	2.50027	3.3201	2.5252
F8	-0.37633	0.58994	0.28638	-0.07962	0.2226	-0.99955	0.1312	-0.71509	0.93505	0.96621	0.74248	1.06934	0.44948	0.43128	0.81392	0.63987
F8A1	7.29255	7.73073	7.78603	6.65486	5.73308	6.27816	4.68532	6.20806	6.8183	6.80159	6.79271	5.49135	5.72469	5.18541	5.66915	5.34655
MIR1184-1	9.07539	9.31153	9.43471	8.87407	6.8325	7.47933	6.01115	7.03975	7.82654	7.91893	7.73389	7.41814	6.82937	6.07508	6.8407	6.43545
FUNDC2	1.67982	1.80337	1.67142	1.96483	2.27381	2.10764	2.44235	2.0349	3.00353	2.77204	2.66686	2.43775	2.53152	2.38018	2.18662	2.1827
MTCP1	3.02623	2.94016	3.04427	3.19284	2.8457	3.30586	2.50406	3.23632	2.40313	2.7242	2.24034	2.39037	3.31064	3.0472	2.90882	2.69674
CMC4	3.94302	3.84438	3.96108	4.09038	3.70921	4.1113	3.37978	4.05789	3.29728	3.6383	3.11566	3.27937	4.2103	3.92389	3.80888	3.63265
BRCC3	3.3188	3.217	3.12764	3.3891	3.94585	2.76195	4.16955	2.76586	4.11109	3.62416	3.48119	4.13402	4.71057	4.25568	4.13849	4.0975
VBP1	5.67084	5.67227	5.39141	5.89077	5.72453	5.36873	6.15925	5.13318	6.01464	5.60646	5.59659	5.51217	6.35428	6.15309	5.65972	5.32449
CLIC2	2.79329	3.15433	2.70993	3.62611	-0.76741	-0.28912	-0.54222	-0.23854	-2.61237	-1.95391	-2.43494	-1.19499	-3.04204	-2.00385	-2.98746	-2.70522
F8A2	7.4328	7.92768	8.01987	6.27348	6.36435	6.84542	5.35745	6.67837	7.45145	7.40113	7.44067	5.85761	5.97168	5.4204	6.22492	5.83218
MIR1184-2	8.2154	8.11059	8.49743	7.69996	7.72921	8.25393	7.27833	7.7824	8.64434	8.59113	8.598	8.06018	8.0286	7.17522	7.94458	7.52728
F8A3	6.86523	7.33581	7.41593	5.73591	5.82794	6.35845	4.899	6.22558	6.97031	6.92568	6.9378	5.74749	5.46848	4.90012	5.66134	5.27832
MIR1184-3	8.20776	8.06865	8.46367	7.67921	7.70869	8.20439	7.27833	7.79457	8.66068	8.5393	8.61603	8.03179	8.02179	7.16295	7.94458	7.52728
TMLHE-AS1	0.09427	-1.57314	-0.62548	-0.43977	-0.53624	-0.00989	-0.38171	0.48634	-0.76376	-0.13402	-0.49393	1.24676	0.36335	0.65785	-0.28116	0.73112
TMLHE	1.31873	1.75062	1.48342	1.98992	2.78643	2.23323	2.96739	2.33422	2.82162	3.24908	2.80488	3.78604	2.87273	3.33112	3.09195	3.60062
VAMP7	4.97594	5.27576	5.0545	5.6157	4.45135	4.35271	5.20795	4.21055	4.75081	5.21823	4.9028	5.69178	4.48523	5.13089	4.99355	4.87252

WASH6P	3.45378	3.1119	3.30687	2.7799	1.95983	2.91833	0.84341	3.20716	2.75316	3.056	2.99449	2.2868	2.1393	2.29717	2.14286	2.31355
RPL23AP53	0.69363	1.15629	1.38774	1.31867	1.7321	0.13382	1.77755	0.53959	1.92743	1.90994	1.51135	1.60824	3.1724	2.97424	2.48671	2.93578
ZNF596	-1.42942	-1.02479	-0.83874	-0.44221	-1.27675	-1.63469	-0.90482	-1.46911	-0.60917	-0.39552	-0.81507	0.49606	-0.36203	-0.07843	-0.53113	0.55982
FBXO25	0.36453	0.44004	0.42007	0.43363	0.85283	0.42413	1.29642	0.54189	1.32514	1.67283	1.01865	2.13016	1.43268	1.61151	1.49617	1.65957
TDRP	2.2514	2.05332	2.14614	2.33213	2.74332	2.37359	3.2271	2.29379	2.57113	2.92966	2.51856	2.72608	3.80744	4.25165	3.6636	3.85604
ERICH1	1.26195	1.25335	1.21516	1.80107	0.5814	1.07655	0.71536	0.96407	1.54125	1.54416	1.39483	1.06841	2.03379	2.69851	1.86803	2.11386
CLN8	2.48688	2.36625	2.35225	2.08869	2.02719	1.51312	1.77568	1.9765	2.12852	2.37724	2.35473	2.43633	3.1083	2.94781	3.13549	3.3364
ARHGEF10	-3.32193	-3.32193	-3.20397	-3.32193	-0.05234	-0.37903	-0.04995	0.13855	3.06188	1.88721	2.82086	2.36127	2.55597	1.3992	2.59765	1.30983
KBTBD11	-3.32193	-3.32193	-3.13665	-3.32193	-1.55339	-2.76828	-1.71246	-2.15961	0.55714	0.55768	0.68685	0.83885	-2.14011	-1.78779	-0.71788	-1.08166
CSMD1	-0.4283	-0.3245	-0.23089	-0.02147	0.058	-0.66979	0.11089	-1.04411	-3.32193	-3.32193	-3.32193	-3.32193	-3.32193	-3.32193	-3.32193	-3.26466
MCPH1	0.98678	1.04305	0.70776	1.08044	0.68466	-0.31035	1.3312	0.02296	1.19362	0.90198	1.18883	1.78953	1.80839	1.80595	1.80887	1.62073
ANGPT2	-1.0648	-0.79651	-1.14284	-0.79277	-1.76312	-2.13665	-1.88315	-2.16728	-1.34761	-1.59083	-1.09577	-1.3073	-0.96343	-0.6315	-1.11738	-0.89973
MCPH1-AS1	0.56162	0.61348	0.68647	1.36557	-0.87917	-1.59136	-0.62809	-1.67972	-0.35626	-0.09636	0.30453	-0.11831	0.65734	1.16876	0.77027	0.97444
AGPAT5	3.60636	3.56047	3.48176	3.69414	3.75024	2.92065	4.16082	2.68399	3.96029	4.17722	3.36161	4.21632	5.14232	5.4116	4.57756	4.95346
XKR5	-3.32193	-3.09154	-3.32193	-3.32193	-3.32193	-3.32193	-3.32193	-3.32193	-0.32314	-0.62058	-0.15352	-1.29649	-3.32193	-3.32193	-3.32193	-3.11961
FAM86B3P	0.8899	0.63123	0.9776	0.22192	-0.78449	-0.19523	-1.12965	-0.54032	-0.87678	-0.83384	-0.59658	-0.74042	0.91487	0.83148	0.72421	0.45972
ALG1L13P	1.70689	1.84467	2.05896	1.38145	-0.12019	0.59454	-0.73537	0.46039	-1.07683	-0.19129	0.06293	-0.60095	1.66334	2.07153	2.23538	1.59987
PRAG1	1.45575	1.7521	2.13543	1.3547	3.25861	2.04138	2.62566	2.71295	4.34853	4.32198	4.76472	4.20664	2.75132	2.75947	3.40826	4.35551
CLDN23	1.65146	1.7627	1.82121	2.02684	4.08221	2.95789	3.48402	3.19618	1.89472	1.81393	0.88144	1.04785	0.73971	0.91664	0.5184	1.77285
MFHAS1	2.83059	2.74284	2.59885	2.56261	3.53225	1.46277	3.17233	1.68926	3.45661	3.2471	3.00567	2.91863	4.56706	4.76333	4.82961	5.32786
ERI1	2.18936	2.10505	2.26142	2.16628	2.38722	0.98577	3.0056	1.15399	2.93448	2.37166	2.38544	2.39888	4.59821	4.05487	3.94881	3.61292
PPP1R3B	2.05588	2.42318	2.87953	2.16235	1.83811	1.60792	2.2413	1.26389	3.71263	4.52815	3.69776	5.02489	3.19261	3.6665	4.31713	3.88746
TNKS	0.95069	1.15758	1.37958	1.40144	1.19019	0.06475	1.33381	0.15722	2.51642	2.66834	2.77614	3.61372	2.55631	2.33261	3.15828	3.03924
MSRA	-1.26184	-1.34731	-1.28879	-0.39755	-1.13166	-1.61594	-1.158	-0.93949	2.09142	1.79934	1.83103	2.15341	1.96827	1.47431	1.31367	1.90286
RP1L1	-2.98602	-3.0635	-2.9317	-3.17095	-3.32193	-3.32193	-3.32193	-3.17926	-1.98639	-1.6479	-2.46213	-2.10436	-3.32193	-3.22672	-2.8189	-3.20386
SOX7	-2.03033	-1.78782	-2.42839	-2.38536	-2.93514	-3.00694	-2.78534	-2.03788	4.12397	4.63039	4.05096	4.13577	-1.45525	-0.90698	-2.77515	-2.05108
PINX1	2.26094	1.89825	1.83263	1.41738	1.98356	1.19751	2.15035	1.36458	5.01873	5.3725	4.94409	4.25965	3.74995	3.34937	2.83979	2.57916
XKR6	-2.64748	-2.583	-2.05161	-2.51262	0.42932	0.10996	-0.23272	0.21823	0.04992	0.64662	0.39985	0.31511	-3.32193	-3.32193	-3.32193	-3.32193
MTMR9	0.46285	0.62725	0.37969	1.08018	1.56032	0.86413	2.09491	0.89649	2.17517	2.031	1.92199	2.67458	2.96613	3.04645	2.799	3.08975
FAM167A	0.84501	1.14509	1.15006	0.74574	-1.11965	-1.17459	-1.31373	-1.48689	5.59551	5.03625	5.41579	4.45263	-3.00971	-2.44014	-3.12378	-2.94234
BLK	-3.32193	-3.32193	-3.32193	-3.32193	-3.32193	-3.32193	-3.32193	-3.32193	0.317	0.95509	1.35656	0.40654	-3.32193	-3.32193	-2.93395	-3.11068
GATA4	-2.88964	-2.65454	-2.62517	-3.32193	4.79211	4.02644	4.14784	4.27967	3.2573	3.00912	3.10217	2.18208	2.01971	1.24102	2.24888	2.23869
C8orf49	-3.32193	-3.32193	-2.0579	-3.32193	0.12194	0.3743	0.39485	0.76114	-2.77754	-1.78515	-1.22825	-0.37155	-1.80906	-1.8124	-0.67315	-0.93419
NEIL2	3.08976	2.64561	2.5036	2.2846	1.93731	1.91928	1.98239	1.71385	3.6258	3.62355	3.73392	3.4657	4.38568	4.57824	4.4404	4.52211
FDFT1	4.20914	4.31302	3.90525	4.22417	4.43081	4.93909	4.36555	4.82713	5.71921	5.15351	5.20254	4.68609	5.43826	6.14418	5.50409	5.58149
ALG1L11P	0.99797	0.33514	-0.97522	-0.90116	0.17055	1.01306	-1.93792	-0.48314	1.81067	1.91031	2.24653	2.00865	2.27177	2.41977	2.1846	2.54164
FAM86B1	0.77725	-0.28552	0.13677	-0.77058	-0.39205	0.59381	-0.87544	-0.10641	1.7491	2.05917	2.07815	1.8388	1.82709	2.02348	1.98262	2.19915
FAM86B2	1.58384	0.52527	1.21494	-0.16977	0.02426	0.18146	-0.54922	-0.13456	2.4285	3.01777	2.93243	2.3832	2.47897	2.68763	2.67639	3.1108
LONRF1	1.02758	0.92053	1.10708	1.17268	2.12869	2.02202	2.26756	1.8767	2.13763	1.78952	2.066	1.59071	3.12264	2.89586	3.23566	2.7052
TRMT9B	-3.32193	-3.32193	-3.32193	-3.22864	-1.52619	-2.56023	-1.40894	-2.27237	-2.08738	-1.6237	-1.77671	-1.53204	-2.4839	-1.59374	-1.61117	-1.09971
DLC1	2.34495	2.29084	1.87369	1.78837	1.55034	-0.18615	1.87029	-0.01817	4.70156	4.44021	4.73135	4.35174	4.88259	4.10673	4.8589	4.2227
C8orf48	0.72911	1.25752	1.21855	1.59244	-0.90667	-1.23164	-0.61817	-1.49193	0.83644	1.28137	1.41115	1.06059	2.86699	3.13409	2.22155	2.0298
TUSC3	3.67426	3.85308	3.99285	4.31766	4.00314	3.85286	4.30306	3.44814	4.44002	4.44642	4.2666	4.32118	-1.26424	-0.4304	-0.42956	-0.32107
MICU3	-0.2783	-0.04396	0.39806	0.94813	-1.45488	-1.42434	-0.76712	-0.28366	-3.32193	-3.32193	-3.32193	-3.32193	0.35036	1.48365	0.3858	0.94503
ZDHHC2	2.00236	2.28856	2.45751	2.64804	1.85945	1.36181	1.92203	1.19725	3.04568	3.32847	2.42346	3.79449	3.08985	3.24118	3.04294	3.28188
CNOT7	2.31602	2.60557	2.36925	2.67071	2.98707	2.53627	3.69857	2.54506	2.83598	3.59236	3.31615	4.31232	3.51117	4.75264	4.45447	4.54867
VPS37A	2.97391	3.0156	3.03176	2.91139	3.33482	3.27292	3.78336	3.03499	3.00699	3.02739	2.85964	3.42339	3.79543	3.76116	3.87059	3.62742
MTMR7	-1.22686	-1.09757	-0.89927	-1.11567	-1.59232	-0.47317	-0.84115	-0.9388	-1.01093	-1.01609	-1.61704	-0.49913	-0.65719	-0.7363	-0.62868	-0.78831
SLC7A2	1.51546	1.16921	0.86512	1.33874	2.27656	2.86925	2.98503	2.87352	-0.20987	-0.39076	0.38994	-0.0505	0.76544	1.91797	2.46171	2.92638
PDGFRL	2.46272	2.07805	2.37197	2.07307	3.2015	4.42635	3.41762	4.20596	2.39722	3.12709	2.44362	2.5983	2.9205	3.96278	3.9292	4.16655

MTUS1	2.65326	1.99296	2.46078	2.21029	4.5645	4.10152	4.99828	4.06398	-2.39488	-2.15437	-2.58255	-1.61409	2.25487	2.28011	3.19688	2.79542
FGL1	-3.32193	-2.71048	-3.32193	-2.64962	4.25115	4.22417	4.34277	4.61056	-2.97337	-3.32193	-3.32193	-3.32193	-1.27532	0.19684	-0.98833	0.90049
PCM1	2.54156	2.62452	2.57285	2.9417	4.14664	3.18352	4.78129	3.32456	3.71865	3.42519	3.30525	4.40858	4.3707	4.61214	4.78181	4.89459
ASAH1	3.09228	3.3099	3.35626	3.94939	3.98857	4.52526	4.48465	4.34083	1.9748	2.26342	1.93257	2.79432	3.82739	3.9506	3.83165	3.96661
NAT1	0.45832	0.04951	-0.32811	0.89751	0.57941	0.8553	0.48901	0.69174	0.94297	0.30329	0.5056	0.38701	1.26493	1.00896	0.44686	0.80704
PSD3	0.17049	0.10225	0.40153	0.64336	-0.50975	-2.12687	-0.35663	-1.73579	2.7795	2.58343	2.95667	3.40135	2.32311	2.23017	2.68856	3.16299
SH2D4A	3.20939	2.9175	2.72136	3.02761	3.23835	1.9644	3.28922	2.1432	4.67992	3.70649	4.09747	4.2076	4.5169	4.06619	4.20527	4.3102
CSGALNACT1	-0.20642	-0.24917	-0.22669	0.38527	4.89316	3.8926	5.745	3.21555	-1.36619	-1.42284	-1.06254	-0.65126	2.19589	1.79111	2.14548	2.68757
INTS10	2.69434	2.27213	2.4152	2.71402	3.40658	3.2042	3.54342	3.11448	3.13009	2.8444	3.08419	3.38503	3.46247	3.22399	2.79704	3.03249
ATP6V1B2	4.1821	4.49414	4.25188	4.72381	3.45355	3.10328	4.0522	2.88041	4.16636	4.11567	4.01964	4.16424	4.60943	4.89219	4.65072	4.89821
XPO7	3.03155	3.04354	2.86261	3.32987	3.76773	2.83171	4.12462	2.87087	4.17005	3.8315	4.08128	4.87912	4.91849	4.96693	5.40891	5.19654
DMTN	-1.78158	-0.67616	-0.8039	-0.8941	-0.36223	2.97023	-0.27084	2.34889	0.49666	0.85331	0.53417	0.32357	4.38904	4.12749	5.05379	4.82407
FAM160B2	2.07586	2.01283	1.98748	1.67318	2.67253	3.06129	1.76155	3.13904	3.23371	3.42872	3.4718	3.35884	2.9617	3.036	3.33355	3.52931
NUDT18	1.57187	1.87649	1.89259	0.87159	2.79732	2.78503	1.81733	2.70344	3.43792	3.95288	3.80932	2.50403	1.58909	2.06051	2.49262	2.3756
HR	-3.08717	-2.06588	-2.92793	-2.48941	-1.89505	-2.54399	-2.8238	-2.52646	-2.62931	-1.67575	-3.0794	-2.20247	-3.04971	-2.45191	-2.65244	-1.92235
REEP4	5.0148	4.17416	4.55291	3.52543	5.60756	4.94402	5.02744	5.01089	5.81949	3.93166	5.12546	2.94446	5.40378	4.06211	5.56438	3.75878
BMP1	2.36132	2.59056	3.23523	2.82043	2.87059	2.64502	2.64784	2.54735	4.04738	4.38795	4.53974	4.46023	2.73722	3.04725	4.31233	4.30651
PHYHIP	-2.00638	-2.81827	-1.6416	-1.38854	-3.11018	-3.32193	-3.09082	-3.32193	-0.90867	0.19024	0.11546	-0.05206	-2.30709	-1.94441	-1.02639	-0.76025
POLR3D	3.07264	3.06341	2.80377	2.89412	2.61248	2.82917	2.67825	2.84616	3.22168	3.74254	3.62661	3.91658	3.99092	4.32973	4.16205	4.06436
PIWIL2	-0.83948	-1.01791	-1.55714	-0.59464	-0.55876	-0.72533	-0.69786	-0.59552	-1.51052	-1.32441	-2.81664	-2.1333	-2.35731	-1.84117	-2.99115	-1.74882
SLC39A14	5.07116	5.08133	5.24521	5.29727	6.89839	5.46733	6.9849	5.37072	4.69101	4.44567	4.59497	4.90122	5.61369	5.21896	5.44228	5.32306
PPP3CC	3.23932	3.51077	3.27007	3.92989	2.84014	2.74517	3.38831	2.59804	2.73008	2.78983	2.3639	2.51962	3.74615	3.66463	3.35361	3.16716
SORBS3	4.07049	4.27073	4.27264	3.31946	2.86492	3.37085	1.85011	3.43759	6.25167	5.90488	6.17116	4.67255	5.06086	4.96867	5.93508	5.59576
PDLIM2	1.2776	1.62037	1.7446	0.45771	0.95305	1.04522	0.42202	0.58586	3.9201	3.77793	3.87102	2.08193	2.89107	2.05296	2.60445	2.11464
C8orf58	1.7249	1.92713	2.59268	1.31764	2.0581	2.84744	1.69826	2.67573	5.38895	5.52192	5.49389	4.7857	2.95307	2.04209	2.52963	2.0261
CCAR2	4.84848	4.58203	4.68908	4.35919	5.02649	4.6304	4.55495	4.59624	5.57023	5.23005	5.71173	5.16986	5.92536	5.60989	6.23176	5.91479
BIN3	2.31431	2.17766	2.20381	1.9775	1.90558	2.28486	1.51937	2.06648	2.60937	2.80019	2.5858	2.45243	2.608	2.45702	2.39207	2.46259
PEBP4	-0.67671	-2.59469	-0.61376	-2.01482	-0.2876	-1.85133	-0.40709	-3.32193	1.594	1.08902	1.40103	-0.38771	1.04775	-0.3514	2.15348	0.2422
RHOBTB2	1.82546	1.58464	1.87866	1.11752	1.73165	1.71028	1.62982	1.43051	3.2958	2.61279	3.1395	2.69706	3.49939	2.36856	3.65408	2.88482
TNFRSF10B	4.99386	4.62388	4.83457	4.79083	5.17077	5.06385	5.42891	4.74236	6.34062	5.95686	6.37604	6.33388	6.67571	5.84086	6.27085	6.00339
TNFRSF10C	-0.41414	-0.05465	0.24595	0.19547	0.14348	2.32268	0.39434	1.48946	1.56191	2.39519	2.22148	2.25726	-1.08739	-1.1642	0.00478	-0.44465
TNFRSF10D	3.01915	2.77534	3.01411	2.93215	2.00132	2.46832	1.67445	2.23025	4.57833	4.95543	4.43736	4.78727	4.72684	4.04337	3.39932	3.82203
TNFRSF10A-AS1	3.22649	3.46471	3.0327	2.84304	3.71589	3.64871	4.39413	3.86708	3.34414	3.31747	2.70866	3.90689	5.22582	5.70132	4.69625	5.33228
TNFRSF10A	4.09952	3.97686	3.57399	3.78092	4.85558	3.95139	5.01278	3.80946	3.99969	4.24748	3.68685	4.397	5.52053	5.72647	5.23419	5.69961
CHMP7	3.87163	4.09465	3.82499	3.81699	3.72009	3.32505	3.6631	3.20434	4.3638	4.49174	4.54689	4.4279	4.58135	5.17573	5.03618	5.22268
R3HCC1	3.94081	3.79827	3.77978	3.27415	3.91189	3.87679	3.48557	3.90519	4.56469	4.84579	4.66574	4.14084	4.07763	4.48243	4.34951	4.30517
ENTPD4	2.79596	2.58367	3.24167	2.71445	3.33824	2.74145	3.6892	2.34081	6.85903	6.78952	6.56936	4.8597	4.66171	3.58243	4.99762	4.40384
SLC25A37	5.6635	5.90688	5.95824	6.09941	1.26189	2.1889	1.15974	2.17239	4.65043	5.91338	5.4872	6.78914	3.65803	3.88195	3.7311	4.41315
NKX3-1	2.93905	3.03023	2.64528	2.96311	-1.46592	-2.35137	-1.97122	-2.67629	3.1975	3.07917	2.68191	3.57126	2.50029	1.83337	1.92317	2.62446
STC1	6.39228	6.74952	6.70955	6.14187	-2.75957	-2.67355	-2.55771	-2.09853	5.50917	6.00266	4.25041	6.81325	7.57939	5.63406	6.18495	7.06194
NEFL	-0.00517	0.09207	-0.43986	0.10194	-3.02511	-3.32193	-3.09869	-3.32193	-3.32193	-3.32193	-3.32193	-3.32193	-3.32193	-3.32193	-3.32193	-3.32193
DOCK5	1.756	1.85837	2.09656	1.88412	1.97722	0.91816	2.35242	0.64464	3.44874	2.95037	3.13172	3.36356	2.85588	1.87664	2.50961	2.61402
GNRH1	-0.77466	-0.87579	0.31968	-0.49073	-0.96702	1.21833	-0.96815	0.87097	-1.49007	-1.18508	-2.38638	-0.38061	0.183	0.5271	-0.62881	-0.60209
KCTD9	2.8303	2.59042	3.55888	2.87631	3.13399	1.99461	4.09802	1.64748	3.24574	2.56209	3.70809	2.69201	4.02953	2.61516	4.29475	2.40635
CDCA2	3.23485	3.09562	3.36616	2.80623	3.54292	2.31596	3.97181	2.33928	4.60242	3.37894	3.87238	3.05787	4.96356	4.54853	5.02761	4.48712
PPP2R2A	3.01317	3.1348	3.11204	3.05259	2.62885	2.10113	3.12461	2.15905	3.64642	3.27677	3.33011	3.75547	4.10245	3.93606	3.82247	3.63532
SDAD1P1	-0.64847	-0.45157	-0.27887	0.31283	-1.80122	-1.4268	-2.38609	-1.8651	2.92127	3.15683	2.28163	3.54439	0.46832	0.46355	0.83262	0.63747
BNIP3L	4.58896	4.9925	5.25981	4.98319	1.62872	2.60744	1.99478	1.91268	6.72594	6.95111	6.24442	7.31153	5.40776	5.64658	5.64763	5.61896
PNMA2	2.0907	2.87062	2.96287	2.92912	-2.49975	-2.43914	-3.32193	-3.10102	4.11581	4.38957	3.96409	4.80357	3.71412	4.20579	3.94867	3.81449
DPYSL2	3.58234	4.07201	4.14439	4.13556	-0.7241	-2.26936	-0.67664	-1.55596	6.03131	5.89162	5.69968	5.96243	5.44732	5.65162	5.99141	5.94955

TRIM35	2.15811	1.95422	1.79974	2.02521	2.93573	1.9379	2.83304	1.7414	2.83532	2.61194	2.88531	2.70678	3.24799	3.18328	3.63102	3.52677
PTK2B	-2.95064	-2.04244	-2.89141	-2.08402	1.15805	1.18662	0.94595	0.98074	-0.5808	-0.546	-0.36022	-0.45269	-0.4419	-0.76418	-0.61904	-0.1954
EPHX2	-2.89215	-3.32193	-3.32193	-3.32193	2.39866	3.14921	2.09716	2.88865	0.17452	0.65296	0.65437	1.01758	1.43826	0.98751	1.39759	1.8835
SCARA3	1.39081	1.28329	1.25159	1.67214	3.33872	0.65946	3.07094	-0.14956	0.33771	0.55965	-0.22106	0.14259	6.3526	6.68393	7.40651	7.30792
CCDC25	3.20808	2.93305	3.04684	3.22216	3.38203	2.68026	3.76777	2.62377	3.67453	2.98453	3.60787	3.07596	3.63208	2.78797	3.64229	2.53918
ESCO2	1.39023	0.85155	0.96845	0.4626	1.68951	-0.73229	2.2678	-0.79911	1.87793	-0.20138	0.91449	0.07107	3.2356	0.96655	2.79113	0.5444
PBK	4.03975	3.5618	3.56339	3.59606	4.65134	3.70483	5.04119	3.76275	5.51104	4.68067	4.75419	3.70576	6.48281	6.66079	5.61801	5.44805
SCARA5	-3.32193	-3.32193	-3.05011	-3.32193	2.50442	2.05989	2.00735	1.85506	-3.32193	-3.32193	-3.32193	-3.32193	1.68354	3.01157	2.57641	4.15318
NUGGC	-3.06366	-3.32193	-3.02063	-3.32193	-0.71814	0.07232	-0.94098	0.2297	-3.02577	-3.07213	-2.83016	-2.86597	-2.77522	-2.77684	-3.0744	-2.09594
ELP3	2.63015	2.5333	2.22399	2.52893	3.19198	3.12653	3.36668	2.86316	3.34469	3.32595	3.34396	3.77567	3.85066	4.04734	4.01412	4.21988
ZNF395	3.18634	3.1711	3.51072	2.76582	3.01864	2.67145	2.33418	2.63773	5.09883	5.15111	4.88209	5.31065	3.53661	2.85026	4.19756	3.81413
FBXO16	2.8505	2.75388	3.13818	2.31017	2.81169	2.67411	2.41046	2.37566	4.73733	4.7792	4.60344	4.61227	3.25244	2.53345	3.91818	3.62771
FZD3	0.20501	-0.07907	-0.34569	0.12142	0.83442	0.03147	0.89147	0.35437	-1.22713	-1.32713	-1.99066	-0.49742	2.3282	1.97365	1.94139	2.13976
EXTL3	2.84048	2.61728	2.418	2.33226	3.88225	3.37997	3.83395	3.37264	3.77541	3.50375	3.68204	3.77045	3.88748	4.27654	4.79996	5.18971
INTS9	1.59046	1.56528	1.2552	1.6398	1.29003	1.41128	1.21131	1.50981	1.99303	1.78943	2.35497	2.0374	2.01064	2.59487	2.75104	2.63568
HMBOX1	-0.25662	0.04588	-0.43915	0.71015	0.54246	0.80255	0.30536	0.8901	0.01593	0.37388	0.61363	0.82757	0.81471	1.24736	1.51548	1.46472
KIF13B	1.82797	1.8715	2.02276	2.06433	4.08337	2.90569	4.30475	2.8831	2.45272	2.68505	2.49075	3.14838	2.58727	2.72943	3.81665	3.96532
DUSP4	3.85751	3.68618	3.65745	3.75703	4.39314	3.39115	4.48684	2.62473	2.2477	3.30403	2.40191	4.3752	3.90258	3.09996	3.44531	3.81396
SARAF	4.94034	5.18824	5.24039	5.76111	6.25567	5.95629	6.69128	5.63916	5.56617	5.75798	5.49119	6.22322	6.35346	6.61964	6.47164	6.7294
LEPROTL1	3.61194	3.8189	3.5148	4.14451	4.40138	4.50346	4.87285	4.32608	3.41287	3.47828	3.4459	3.66157	4.26229	4.62704	4.33191	4.15168
DCTN6	3.83401	3.86685	3.68313	4.25273	4.30774	4.46539	4.70187	4.2078	4.11547	4.07457	4.05958	3.83079	4.6252	4.24117	4.00432	3.54436
TUBBP1	2.46532	2.68948	2.81289	0.5549	1.16526	0.89835	0.92946	0.87859	2.48046	2.49752	1.93067	1.25077	0.75568	0.92874	1.68089	1.02331
RBPMS-AS1	3.86938	3.7618	3.97506	3.55215	6.31761	6.93464	6.26564	6.52138	5.3138	5.32125	5.29412	4.74435	5.47558	5.716	6.6239	6.1363
RBPMS	2.61612	2.53027	2.68058	2.47594	4.71582	5.70413	4.64965	5.41741	3.67774	3.76643	3.71837	3.75879	4.24685	4.46598	4.9805	4.49056
GTF2E2	4.55571	4.19863	4.20311	4.34338	4.2422	4.44905	4.67587	4.11416	4.33831	3.85137	4.13541	3.78898	5.44508	5.32617	5.19351	5.01834
GSR	5.08147	4.85678	4.36718	4.78889	6.51974	5.02825	6.02692	5.57063	4.41901	4.10317	2.75099	4.3837	6.53603	6.53759	5.31365	6.15866
UBXN8	1.47402	1.6965	1.37108	2.18479	2.92605	3.16655	3.45346	3.00358	1.51004	1.70661	1.51055	1.78206	2.78748	2.87791	2.35654	2.6152
PPP2CB	4.12865	4.11001	4.19396	4.35865	4.44625	3.9968	5.20996	3.83242	4.46297	4.02456	4.15066	4.13599	5.90498	5.88976	5.60978	5.26324
TEX15	0.53029	0.24248	-0.13284	0.08091	1.37761	-0.11897	2.14512	-0.25826	-3.21444	-3.32193	-3.32193	-3.32193	1.25935	0.9319	1.50827	0.85949
PURG	-1.70928	-1.23988	-1.54023	-1.19177	-2.2268	-2.44822	-2.49944	-2.16878	-0.85065	-0.69898	-0.24088	-0.06118	0.61827	2.2701	2.63942	2.18864
WRN	1.75643	1.62487	1.51508	2.0837	1.98387	1.64266	2.31052	1.47027	2.48754	1.8771	2.07381	2.32865	3.50542	3.5686	3.4855	3.21439
NRG1	0.76245	1.13709	1.62622	1.82118	-3.16308	-3.04953	-2.99331	-3.00639	5.2479	4.88716	5.01765	5.22901	3.94864	4.17087	3.51553	3.81013
FUT10	-0.2623	-0.60076	-0.43504	0.15534	1.76991	0.30313	1.88968	0.24708	0.86542	0.8008	0.81858	1.53382	2.43107	2.00832	2.04204	2.42102
TTI2	2.78309	2.51404	2.07592	2.46129	2.72151	2.28039	2.94515	2.28288	3.15088	2.53802	2.86758	2.94123	4.07706	3.54605	3.59123	3.21766
MAK16	3.18091	2.67212	2.54355	2.76389	3.12626	2.76293	3.49982	2.67066	3.29536	2.58857	3.06106	3.13248	4.64565	3.81534	3.91067	3.15621
RNF122	1.17665	1.22554	1.3288	0.49699	0.72493	1.38984	0.50448	1.72777	2.54769	1.98047	1.82509	1.45363	1.65979	1.97351	3.09581	3.23034
VENTXP5	1.80938	1.88631	1.50019	1.08594	-0.92873	-0.13602	-3.32193	-1.12866	-3.32193	-3.32193	-3.32193	-3.32193	-3.32193	-3.32193	-3.32193	-3.32193
UNC5D	-2.17145	-1.76957	-1.75864	-0.71957	-3.27862	-3.32193	-3.22839	-3.20377	-3.32193	-3.32193	-3.32193	-3.32193	-3.32193	-3.32193	-3.32193	-3.32193
LSM12P1	4.55231	4.36069	4.39604	4.44372	4.08997	4.23141	4.51687	4.02418	4.97262	4.87023	5.03475	4.99471	4.55909	4.57906	5.19558	4.51721
LINC01605	3.70059	3.1927	3.42394	3.00325	-3.32193	-3.32193	-3.32193	-3.32193	4.62633	4.19717	4.9094	2.90617	-3.32193	-3.32193	-3.32193	-3.32193
ZNF703	3.93133	3.87955	3.82347	2.84396	2.36984	2.0945	1.17838	1.826	6.72265	5.94694	6.98217	4.66726	-1.29107	-1.57698	-0.82458	-1.04555
ERLIN2	2.36734	2.134	2.15779	2.32995	3.96627	3.2306	4.00476	3.17392	3.27215	2.85617	3.38743	3.10649	3.60702	2.93141	3.50279	2.71508
PLPBP	2.53177	2.3877	2.3244	2.51146	4.22396	3.92026	4.50947	3.62045	4.47287	3.91845	3.89947	4.40734	4.49313	4.36595	4.11324	4.27382
ADGRA2	1.01688	0.83013	0.73348	0.37109	0.98983	1.4093	0.5805	1.48863	2.27881	2.26803	2.5485	1.27098	1.39599	1.09327	0.92184	0.96766
BRF2	2.53115	2.06058	1.95031	1.76614	2.97724	3.00072	2.70148	3.01681	2.43165	2.55478	2.47939	2.05381	3.21172	3.13634	2.86695	2.9244
RAB11FIP1	0.98792	1.04238	0.66383	1.06333	4.086	3.18422	4.24161	3.178	2.68956	2.49814	2.10268	2.58868	2.9086	3.2211	3.5719	3.58054
EIF4EBP1	9.19491	9.03898	9.00755	8.29727	6.77307	7.96533	5.89846	7.72027	8.35197	7.85331	7.96403	6.03378	7.92869	7.13588	7.38801	7.25729
ASH2L	2.80079	2.47728	2.92286	3.11067	3.01158	2.2908	3.01438	2.12571	3.61477	3.06946	3.41003	3.05167	3.3021	2.69749	3.19614	2.76877
LSM1	4.9588	5.09037	4.90544	5.31726	5.02913	5.2528	5.42086	5.29315	4.31112	4.47811	4.27203	3.99128	5.26772	5.64706	4.16907	4.5487
BAG4	3.00146	3.13964	2.77195	3.33679	3.14615	2.59055	3.51649	2.74075	3.26467	3.32834	3.11994	3.60179	3.5114	3.71585	3.81355	4.02455

DDHD2	2.28445	1.83374	2.3816	2.33661	2.5145	1.81339	3.00821	1.79814	2.51024	1.84873	1.98268	2.17203	4.03979	3.29709	3.86168	3.08968
PLPP5	3.36961	3.422	3.25692	3.77513	4.34694	5.10915	3.90917	4.89315	2.28462	2.13343	2.42033	2.07296	3.15458	3.02675	2.58666	2.60462
NSD3	2.45384	2.24594	2.44397	2.52594	2.76454	1.72704	2.95549	1.77773	2.9527	2.20921	2.69567	2.93538	3.53175	2.47561	3.75483	3.13427
LETM2	2.05733	1.81538	1.95971	1.8518	-1.79239	-0.93361	-1.25178	-1.31484	0.21707	-0.32602	-0.00836	-0.28272	0.4677	1.13804	0.74465	0.65457
FGFR1	3.20083	2.55493	2.78498	2.47183	2.47889	2.01973	2.04543	1.87844	4.00383	3.7646	3.88714	3.19662	5.96762	5.74317	6.99108	6.29603
RPS20P22	-1.1414	-0.39414	-1.69296	-0.68881	-1.99968	-1.12901	-1.21826	0.0151	-2.56963	-1.61204	-2.63485	-0.60332	1.29442	2.76796	2.2529	3.70372
RNF5P1	3.17169	3.50999	3.41374	3.16841	1.27475	2.24798	0.8829	1.58791	1.18383	1.65493	1.31165	0.67696	0.42071	0.53244	0.80461	0.71618
TACC1	3.26268	3.14623	3.08015	3.34962	3.01548	2.81029	3.58831	3.08079	3.6288	3.17804	3.14127	3.84176	4.42374	4.42702	4.59417	4.53467
PLEKHA2	2.7964	2.6197	3.00934	2.26577	0.80902	0.66793	1.26439	0.37493	5.6886	4.78158	5.44875	4.99827	4.48745	3.15895	4.85633	3.3361
TM2D2	3.38872	3.76134	3.56478	3.7716	4.72054	4.38431	4.98867	4.32307	4.1491	4.16389	4.32629	3.95373	4.64854	5.0436	4.96697	5.04593
ADAM32	-3.21455	-2.98828	-2.87178	-2.7488	-2.9856	-2.62147	-3.12752	-2.87025	-3.08359	-2.86505	-2.75604	-2.52904	-2.4844	-1.74149	-2.86893	-2.2823
TCIM	-2.14825	-1.79614	-1.00938	0.2103	5.74216	7.09515	6.24913	6.21217	-3.32193	-0.98944	-1.47314	-1.95043	5.76667	4.96244	4.86179	4.67297
SFRP1	-2.22356	-2.52959	-1.97424	-1.08796	-1.76683	-2.00962	-2.50567	-1.67446	7.89927	7.82553	8.05927	7.39118	4.28252	2.95858	3.84364	3.84015
GOLGA7	5.02404	5.26999	5.12002	5.52231	4.80457	4.87501	4.87835	4.62799	5.27213	5.15563	4.76745	5.2293	5.83589	5.93895	5.58204	5.71287
GINS4	4.10519	3.38873	3.47252	2.912	3.58888	3.11857	3.47196	3.41344	4.19565	2.58951	3.36338	2.10643	4.65146	4.05979	4.15294	3.77739
GPAT4	4.88582	4.83387	4.83635	4.8831	4.52759	4.44926	4.72043	4.55142	4.13839	3.92888	4.13834	4.1733	4.77751	4.9785	4.96864	5.2484
ANK1	2.2501	2.35316	2.10356	1.41063	-3.19552	-3.22073	-3.22825	-3.32193	-2.906	-1.60515	-2.24005	-1.13832	2.85924	3.62746	4.71802	5.22705
KAT6A	1.72914	1.77802	1.70721	2.02726	2.69413	1.15402	3.00064	1.23802	3.20099	2.86764	3.0344	3.6691	3.60055	3.54453	4.16439	4.13829
AP3M2	1.26314	0.24203	0.8709	0.66274	1.62933	1.20983	1.77102	1.09073	1.29712	1.22768	1.38457	1.48959	2.57565	1.81452	1.74283	1.42361
IKBKB	1.62599	1.56078	1.66096	1.80217	2.50727	2.56859	2.28987	2.6226	3.12235	3.19016	3.08782	3.14606	2.64847	2.91418	2.65253	2.89346
POLB	2.524	2.13277	2.18077	2.61194	2.9483	2.47164	3.45996	2.41641	2.80259	2.46297	2.3869	2.21211	3.25293	2.57125	2.776	2.17267
RPL5P23	0.23018	-0.31739	-0.66736	-0.16789	-1.17862	0.3508	-1.90548	0.34467	-0.27313	1.33012	-1.40398	-0.71646	-0.76036	-0.32553	-1.88503	-1.86505
VDAC3	6.02208	5.85128	5.8107	5.9798	5.98953	5.54591	6.0127	5.4097	5.94937	5.20419	5.38889	4.92319	6.39061	5.91503	6.01185	5.60834
SLC20A2	3.20043	2.87593	2.74808	2.79303	4.86915	4.00361	4.9524	3.90968	2.04297	1.77115	1.89057	1.58626	4.01019	3.52062	3.98484	3.49088
SMIM19	1.49426	1.51051	1.57486	1.53711	2.66664	2.3092	2.84261	2.18931	2.07545	2.35584	1.59165	1.84841	2.28307	2.40959	2.56752	2.12967
THAP1	2.26216	2.27539	2.46345	2.98185	2.46527	1.90054	2.89279	1.81231	2.39686	2.329	2.1086	2.61042	2.75348	3.16336	2.76164	2.6302
RNF170	1.42718	1.44276	1.77638	2.1229	2.68385	1.76204	3.07714	1.90447	1.30389	1.87661	1.44844	2.21555	2.88957	2.67894	2.5398	2.70694
HOOK3	2.24223	2.33211	2.48202	2.3095	2.1653	1.9298	2.937	2.1168	2.72145	2.55369	2.68288	3.55359	3.93083	3.73098	4.32736	4.10222
FNTA	3.21072	3.26361	3.28914	3.58739	3.20229	3.02856	3.58511	2.78345	2.76686	3.09412	2.879	3.1855	3.55147	3.02678	2.93085	2.80879
POMK	1.87028	1.43241	1.1734	0.12668	3.59834	0.79116	3.54585	1.63229	2.89793	2.87124	2.61817	3.49963	3.72106	4.44139	4.53817	5.22903
HGSNAT	2.07209	2.31901	2.59767	3.01165	3.99038	3.88385	3.99433	3.93201	2.4533	2.61284	2.56262	3.32628	2.99024	2.42695	3.53614	3.57463
ASNSP1	-1.91998	-1.97916	-1.61249	-2.29562	-0.73707	-0.02071	-0.83656	0.1498	-3.32193	-3.32193	-3.32193	-3.32193	-3.0416	-3.32193	-3.32193	-3.32193
RPL10AP2	1.87192	1.39549	1.14334	-0.23605	-0.40505	0.73104	-1.63689	-0.07871	0.8117	2.36042	1.13089	1.843	-0.60421	-0.09887	-3.32193	-2.21218
SPIDR	2.01594	1.77909	1.95433	1.89401	2.23477	1.99392	2.19422	2.19976	2.59652	2.42895	2.71191	2.63021	3.02549	3.22804	3.09653	3.0093
CEBPD	4.34122	4.71686	4.47664	4.45107	4.94874	5.81243	3.83154	5.81184	6.75599	7.32151	7.34486	5.91486	4.36786	5.86598	4.5255	4.90885
MCM4	5.79825	5.48271	5.33104	4.79979	5.05782	3.66058	5.0688	4.14784	5.96605	4.58483	5.20951	4.47967	5.82363	5.55937	5.49188	5.09855
UBE2V2	4.08502	4.294	3.89819	4.6259	3.47797	4.01861	4.02758	3.92656	3.66263	3.80161	3.31944	3.66639	4.57351	4.67478	3.78736	3.79147
RPL29P19	2.87874	1.3572	2.16914	2.3216	-2.5424	-3.32193	-3.32193	-3.32193	2.0767	2.00171	2.50922	2.03516	-0.16313	1.30497	0.29585	-1.21994
SNAI2	2.12464	1.25847	1.94662	2.56646	-2.64546	-2.88253	-3.32193	-3.32193	5.4026	4.64722	5.6387	4.84344	-2.45195	-2.45428	-3.32193	-3.32193
PXDNL	-1.13412	-0.90941	-1.26543	-1.54302	-3.32193	-3.32193	-3.13504	-2.88633	1.4333	0.26326	0.66804	-0.00147	2.31482	1.77873	1.76391	1.19117
PCMTD1	1.28821	2.04291	1.96978	2.60739	1.14767	1.58687	1.84488	1.23661	1.57064	2.5989	1.93432	3.5601	2.60955	2.94858	2.56272	3.18884
ST18	-1.84731	-2.02781	-2.09462	-1.45233	-3.32193	-3.32193	-3.32193	-3.32193	-3.32193	-3.32193	-3.32193	-3.32193	-3.32193	-3.09746	-3.32193	-3.24796
RB1CC1	4.16233	4.57583	4.48478	5.77767	4.09542	3.69719	4.94889	3.67309	3.50518	4.2162	3.64454	5.05697	3.79676	3.93437	4.03726	4.26873
ATP6V1H	4.21454	4.40846	4.15554	4.72845	3.21254	3.41629	4.0391	3.56708	3.66692	3.39572	3.46759	4.09218	3.99566	4.57931	4.25866	4.53751
RGS20	-1.44434	-2.2573	-2.03487	-1.533	0.89982	-0.17351	1.17386	0.22656	2.77709	2.86287	2.94615	2.12574	3.29496	2.13411	1.91375	1.33355
TCEA1	4.90953	4.8903	4.88603	5.02926	4.08961	3.69691	4.85536	3.50449	5.69364	5.38136	5.24781	5.70583	6.80998	5.96796	6.5508	5.84119
LYPLA1	4.04458	4.20587	4.12723	4.40047	4.67199	3.2293	5.31957	3.06006	5.10215	4.68761	4.92926	5.19893	6.51814	5.83734	6.10536	5.77394
MRPL15	6.13912	6.20608	5.77504	6.10415	6.04008	5.43263	6.08066	5.44053	6.64943	6.72855	6.62144	6.66674	8.01389	7.57986	7.28727	7.27268
RP1	-1.44291	-1.14632	-0.75432	-0.81709	-3.25374	-3.32193	-3.32193	-3.32193	-3.32193	-3.32193	-3.23918	-3.03969	-3.25892	-3.32193	-3.32193	-3.32193
TMEM68	1.11361	1.13289	1.05933	1.60798	1.64057	1.32599	2.20011	1.29108	0.5433	0.26284	0.6614	0.695	1.79136	1.55615	0.94377	0.58082

TGS1	3.44258	3.19751	2.89138	3.63312	3.45113	2.27075	3.66483	2.72545	3.45799	2.9011	3.06536	3.46148	3.73517	3.61677	3.41835	3.5946
LYN	2.58744	2.56483	1.39358	2.4887	3.61106	2.00734	3.11866	2.42455	3.79833	2.90794	2.03239	3.18291	3.14878	2.71699	1.65218	2.04999
SNORD54	5.26029	4.77631	4.27151	4.64154	3.16278	4.4824	4.02851	4.6381	2.04942	1.78705	1.88754	2.74177	4.03872	3.5675	1.20911	3.00234
PLAG1	-0.31564	0.16755	-0.01139	0.8688	-2.06571	-1.94181	-1.81108	-1.38518	0.68592	1.36971	0.24857	2.23057	-0.14035	0.96817	0.24841	1.4591
CHCHD7	2.88561	2.98932	2.71327	2.59999	2.09784	2.07274	1.85591	2.01791	2.24624	2.24224	2.27572	1.86774	2.38132	2.25353	1.4206	1.59975
IMPAD1	5.05236	5.24316	5.26704	5.16655	4.63819	3.99841	5.17331	3.61255	4.65475	4.29739	4.31152	4.87196	4.84379	4.86418	5.00562	5.05103
LINC01602	-3.32193	-2.82312	-2.79989	-2.77145	-2.92952	-3.32193	-3.32193	-3.32193	-0.06566	0.21983	-0.34701	0.43927	-2.95645	-2.9576	-3.32193	-3.32193
FAM110B	-0.97642	-0.55307	-0.97358	-0.52565	-3.22879	-3.32193	-2.79617	-3.32193	-1.16242	-0.77471	-0.89118	-0.58722	-3.0046	-1.25606	-0.42544	-0.45755
UBXN2B	2.42882	2.32573	2.59373	2.77865	2.1981	1.27192	2.54246	1.21814	2.20759	1.74976	2.09649	2.91185	3.71967	2.86448	3.34725	2.74509
SDCBP	6.34349	6.80651	6.90494	7.44168	5.15272	4.59947	6.08806	4.20135	5.33801	5.64624	5.14171	5.99665	6.92379	7.17471	6.39474	6.77677
NSMAF	4.2657	4.05468	4.52131	4.46463	2.37574	1.8583	2.62378	1.65747	3.58808	2.71865	3.24043	3.16467	4.25665	3.64979	4.02061	3.4846
TOX	-1.38905	-2.14226	-0.87524	-1.47035	-3.21117	-3.32193	-3.08881	-3.32193	-3.32193	-3.32193	-3.06537	-3.32193	2.62045	2.99298	2.51008	2.28301
CA8	-0.62093	-0.10704	-0.10968	-0.02767	-3.09419	-3.32193	-3.32193	-3.32193	-3.32193	-3.32193	-2.96797	-3.14915	-3.04658	-3.32193	-3.14799	-3.32193
RAB2A	5.03371	5.20344	5.22082	5.49016	5.229	5.21123	5.7902	4.94767	5.38901	5.34968	5.17188	5.4172	5.57063	5.85711	5.63495	5.60623
CHD7	2.97543	2.72231	2.60221	2.80861	2.26162	1.93698	2.73116	2.37971	2.85169	2.39041	2.71738	3.10733	3.04111	3.26476	3.64949	3.77734
CLVS1	1.83364	2.13777	2.39547	2.94121	1.07169	2.53338	2.38815	2.25774	0.24613	0.04452	0.26112	0.97162	2.29611	1.9276	1.83104	1.47189
NPM1P6	3.32061	3.03582	2.90491	2.4885	0.78852	1.53252	1.12393	1.6171	2.28226	2.41723	1.52068	2.14016	0.98902	0.00616	0.2702	-0.0446
KRT8P3	1.26495	1.32467	1.89415	0.78477	3.0473	2.82499	3.32905	2.70807	3.98655	4.4286	4.05135	3.84482	-0.29803	0.3924	-0.12309	-0.09493
GGH	5.62629	5.72888	5.80138	6.09002	5.77855	5.61804	6.07534	5.55982	5.00934	4.58992	4.60425	4.21356	5.13443	5.1887	4.64006	4.44501
TTPA	-2.59968	-2.53176	-1.97736	-1.92113	2.15705	0.02817	2.2448	0.37614	-0.50596	-1.79604	-1.19579	-1.97716	-1.39606	-0.78127	-0.82302	-1.95168
YTHDF3-AS1	0.7836	0.74609	1.18717	1.0075	0.91871	1.97277	0.49744	1.72867	-1.06814	0.54108	-0.59969	-0.12063	1.0925	1.64974	0.30479	0.44978
YTHDF3	3.57745	3.98991	3.96636	4.53403	3.86467	2.53711	4.72482	2.29517	3.71936	3.54958	4.20291	4.58428	4.27045	3.5363	4.94297	4.32344
ARMC1	4.46046	4.27052	4.05337	4.52072	4.10213	2.81298	4.31012	3.06301	4.5146	3.94206	3.82317	4.41465	5.17217	4.78773	4.7352	4.44163
MTFR1	3.97204	3.83687	3.60138	3.9491	3.89833	3.02119	4.32698	3.17857	3.86013	3.04698	3.26837	3.45574	4.87927	4.73359	4.52702	4.48128
PDE7A	2.22922	2.43062	2.53518	2.96463	1.8167	1.8173	2.40879	2.00586	1.3252	0.8668	1.32418	1.4624	2.58796	2.49792	2.73482	2.46641
TRIM55	-2.14591	-2.05068	-3.32193	-1.37256	1.6451	-0.71456	1.95819	-0.9449	-1.85892	-2.34253	-1.96255	-1.66692	5.46235	5.17259	4.73226	4.58018
CRH	-1.73121	-1.38729	-2.64755	-2.13926	8.29423	9.37963	7.88828	8.50742	-2.65775	-1.35468	-3.32193	-1.56312	-3.32193	-1.41558	-2.7561	-2.33518
RRS1	6.74895	6.71329	6.4613	6.26894	6.50974	6.25307	6.48229	6.40478	4.67442	4.87798	5.5088	4.48801	4.93419	5.56046	5.26013	4.90584
ADHFE1	4.14166	4.20386	3.95404	3.99633	3.69791	3.79163	3.9789	4.10092	1.90017	2.22351	2.7028	1.98739	2.60864	3.24102	2.79431	2.45749
VXN	-2.0977	-2.28637	-2.61418	-2.19703	-1.51575	-1.38184	-1.68015	-1.70546	1.43801	1.03264	1.23661	0.88611	-0.88979	0.1118	-0.74281	-1.82334
MYBL1	1.92558	1.62497	1.52055	1.33497	1.86856	0.34566	2.02612	0.68235	4.02244	3.58369	3.13328	3.49182	3.90937	3.90506	2.74026	2.38589
VCPIP1	2.51815	2.69962	2.59396	3.19947	2.18144	1.18805	2.77254	0.99578	2.40829	2.62818	2.60703	3.25892	2.51767	2.91005	2.9411	3.22471
C8orf44	2.42414	2.63702	2.43893	2.41714	0.34609	1.73404	0.82977	1.86304	-0.22385	0.7256	0.04375	1.04816	1.06376	1.31872	1.09834	1.24344
SGK3	1.71866	1.84013	1.28804	2.38897	2.25887	1.8273	2.84629	1.8058	1.33222	0.31757	0.74793	0.74849	2.47137	2.82981	2.23681	2.33414
MCMDC2	4.29149	4.38528	4.50578	3.84122	2.48112	3.10586	2.40577	3.15447	2.82772	2.78827	2.57498	1.71561	3.24311	3.14311	1.38901	1.61732
SNHG6	7.98712	8.14121	8.21234	7.78971	6.26965	6.90056	6.0754	6.98597	6.43065	6.58507	6.2611	5.6879	6.37659	6.2842	5.03486	5.22702
SNORD87	6.47653	5.36489	6.23876	6.2698	5.3255	5.94998	5.48769	6.60879	2.84708	3.34921	2.87324	2.91461	3.27271	3.62685	2.56757	0.8496
PPP1R42	4.22704	4.39279	4.30724	4.67416	3.60022	3.87903	4.10411	3.80822	3.31662	3.78007	3.61885	3.47933	4.19913	4.52789	3.93379	3.87534
COPS5	3.97949	4.09017	4.07662	4.24833	3.52708	3.50689	3.85963	3.5211	3.21847	3.68452	3.57829	3.49444	3.91718	4.23805	3.92492	3.88486
CSPP1	3.46434	3.61223	3.38589	4.16171	1.95994	2.85234	2.6023	3.33685	2.23723	2.27997	2.42761	3.04072	2.86405	2.93503	2.52341	2.53861
ARFGEF1	2.59772	2.65474	2.63155	3.12195	2.80233	1.52815	3.44585	1.5253	3.32881	3.25612	3.47532	3.92208	3.89578	3.82524	4.17235	3.97443
NCOA2	0.875	1.03298	1.23639	1.96039	2.25254	0.9497	2.69448	1.01087	2.05591	1.67373	1.92176	3.0577	2.48401	2.49451	3.2999	3.3016
TRAM1	6.07693	6.14642	6.32542	6.75599	6.42268	5.21058	7.15073	4.90908	6.03784	5.67054	5.8602	6.30287	6.62575	6.02095	6.6843	5.82277
LACTB2-AS1	1.79112	1.84819	1.67241	1.86456	1.24737	0.63621	1.12015	0.02099	0.54333	-0.85421	0.10254	-0.49918	1.33399	1.40973	0.90113	0.24363
LACTB2	3.9759	4.01033	3.99607	4.43047	3.2591	2.97486	3.31173	2.78417	2.30996	1.18297	2.09211	2.00883	3.79288	3.66824	2.93004	2.41051
EYA1	-0.45303	-0.24917	-0.23961	0.0929	-3.32193	-3.32193	-3.13273	-3.32193	-3.08989	-3.32193	-3.32193	-3.12997	-3.32193	-3.32193	-3.32193	-3.32193
MSC-AS1	3.99959	4.05881	4.35736	4.15225	-3.178	-3.32193	-2.8934	-3.12419	-3.32193	-3.32193	-3.32193	-3.32193	0.08122	-0.06177	0.68649	0.19985
MSC	7.53312	7.66985	7.86255	7.51187	-2.62239	-2.52056	-2.30146	-1.65091	-2.43138	-2.88449	-2.85673	-3.32193	2.43923	1.97901	2.84476	2.41502
TRPA1	0.08608	0.02183	0.52702	0.5683	-3.32193	-3.32193	-3.32193	-3.32193	-3.32193	-3.32193	-3.32193	-3.32193	-2.35588	-2.76117	-1.49152	-2.11625
TERF1	2.17092	2.09845	1.87221	2.36715	2.05564	1.9448	2.68185	1.9883	1.92524	1.95282	1.77147	2.49795	2.4715	2.37972	2.23982	2.20515

RDH10	2.30951	1.89456	1.7948	2.22803	4.29195	4.49761	4.26626	4.10337	1.7609	1.71314	1.79475	1.92818	3.93635	5.03469	3.92428	5.15897
RDH10-AS1	-3.32193	-2.567	-2.53473	-2.49549	-2.23287	-1.70628	-2.35624	-1.54429	-3.32193	-2.84291	-3.0451	-1.97225	-2.60835	-0.79038	-1.86012	-0.84089
STAU2	2.3415	2.4004	2.43664	2.93058	2.25972	1.57008	2.87701	1.29844	2.20214	2.08761	1.91892	2.82104	3.12809	3.21897	3.39246	3.26603
UBE2W	1.85168	2.58115	2.24579	2.87641	1.47496	1.33674	2.40717	1.27228	1.34563	2.16619	1.41894	2.67928	2.26777	3.02472	2.61068	2.60465
ELOC	5.64782	5.71429	5.59816	5.70341	4.37874	4.81695	4.74857	4.53528	4.48702	4.21648	4.24936	3.71021	5.14091	5.01934	4.18325	4.02904
TMEM70	4.8895	4.77376	4.63039	4.79861	4.74209	4.13098	5.01845	3.97004	4.05729	3.07164	3.83068	2.83429	4.23718	3.5536	3.32048	2.73947
RPS20P21	2.20961	2.22388	2.82169	1.93109	1.37773	1.38768	1.61211	1.33468	0.95686	0.49485	0.98203	0.22963	1.26574	0.59751	1.03162	1.7707
LY96	3.93693	4.11449	3.94482	4.54495	-1.92736	0.83237	-2.18868	-1.307	-3.32193	-0.48099	-2.10497	0.3919	-2.3207	-2.32327	-1.53888	-3.32193
JPH1	1.55407	0.84101	1.1784	0.81236	-3.2172	-3.32193	-2.90946	-3.32193	-1.70811	-3.09425	-2.8706	-3.09782	-3.32193	-3.32193	-3.32193	-3.32193
GDAP1	2.58943	2.62354	2.38638	2.89738	1.94186	1.80284	2.31644	1.89621	2.71782	2.99761	2.62568	3.20687	2.13969	3.3087	2.27394	2.79518
CRISPLD1	0.97243	1.03974	0.69419	1.88816	-3.03781	-3.32193	-2.47606	-3.05505	-3.32193	-3.10192	-3.32193	-3.32193	-3.32193	-3.32193	-3.10394	-3.32193
CASC9	2.85063	3.56909	3.30748	4.26228	0.11718	0.34711	-0.02138	0.79028	-3.32193	-3.32193	-3.32193	-3.32193	1.67622	1.59471	0.89584	1.80471
HNF4G	-0.48668	-0.90234	-0.88887	-0.12743	2.38118	0.66524	2.85401	0.5113	-1.46278	-0.81814	-0.61691	-0.39917	-1.50213	-2.22797	-1.5939	-2.04792
LINC01111	-3.32193	-3.32193	-3.32193	-3.32193	-3.32193	-3.32193	-3.32193	-3.32193	-3.32193	-2.78584	-3.32193	-3.32193	1.43079	2.16088	0.33172	-0.53102
ZFHX4-AS1	-3.32193	-2.49493	-3.32193	-3.32193	-3.32193	-2.55876	-3.32193	-3.32193	-2.83571	-2.58644	-2.54426	-2.33601	0.87086	-0.91417	0.53573	-1.31179
ZFHX4	0.24308	-0.03298	-0.10109	0.40509	-2.7946	-2.99987	-2.96906	-2.88568	1.00667	0.55976	0.9135	1.45202	2.85283	2.29868	3.25938	2.29821
PEX2	2.64117	2.88957	2.73749	2.8905	2.6391	2.39617	3.10188	2.20614	3.07919	3.38921	3.13569	3.01453	3.54696	3.7588	2.78037	3.22528
PKIA	1.53514	2.29699	1.9206	2.54025	-1.68457	-0.87116	-1.35062	-1.08244	1.65987	1.91632	1.59349	2.83803	-2.4225	-2.66673	-2.55382	-2.39727
ZC2HC1A	1.99937	2.05034	2.05191	2.69467	0.86912	0.99684	1.59853	0.59747	1.89721	2.30249	1.67629	3.15362	2.30531	2.68864	2.84584	2.69924
IL7	1.91985	1.93502	1.70999	2.67737	0.11301	-0.1737	0.65416	-1.53561	1.79812	2.20197	1.64801	2.51002	1.03835	1.67359	1.93121	1.89423
LINC02605	-1.19432	-1.86474	-1.53246	-0.17561	-3.32193	-3.32193	-3.32193	-3.32193	-1.83245	-1.62161	-1.15488	-0.75758	-3.32193	-2.83164	-3.32193	-3.32193
HEY1	2.36258	2.43556	2.23537	2.58041	-1.16244	-0.6029	-1.12455	-1.00038	-1.29677	-1.8097	-1.74172	-1.51287	-1.89263	-1.39142	-2.38302	-1.58061
MRPS28	5.09714	5.11149	5.02179	5.19187	4.79331	4.88105	4.79963	4.82515	4.7421	4.70089	4.69966	3.89922	5.05001	5.23952	4.1203	4.11397
TPD52	4.08594	4.03115	4.11145	3.80994	3.7799	2.66705	4.16474	2.52442	3.29865	2.46078	2.19614	2.41008	4.05929	2.81567	4.37924	2.51779
ZBTB10	1.94313	2.22094	2.33121	2.32768	2.03934	0.74386	2.31583	0.71824	1.78525	1.5606	1.58692	2.01931	2.05628	1.9793	2.8129	2.69836
RPSAP47	4.93148	4.52087	4.61688	2.70031	4.29188	4.30143	3.67191	3.52796	6.3063	7.25327	6.49343	7.68473	-0.96907	0.16791	0.02561	-0.35415
ZNF704	0.17518	0.27338	0.5093	0.84589	-2.81878	-2.52948	-3.18896	-2.80535	-3.08274	-3.25172	-3.24664	-3.32193	-1.86665	-1.06047	-0.86433	-0.17256
PAG1	2.18359	2.82176	2.69628	2.57901	-2.35336	-2.22498	-2.38073	-1.90244	1.36266	1.7245	0.92167	2.34654	1.70274	2.62866	2.75741	2.38184
FABP5	5.21456	4.65906	4.37055	4.36267	5.60097	5.42184	5.32831	5.35091	6.47096	5.80239	6.03897	5.22639	2.91375	1.60701	1.06894	1.3258
FTH1P11	5.8006	5.80908	6.16034	5.1919	2.05933	3.0879	2.01594	1.9206	2.58355	3.71583	2.67231	3.50037	0.51016	1.45364	1.33382	1.36414
IMPA1	3.84086	3.97661	3.37951	4.48427	3.63007	3.28395	4.02189	3.33636	3.28567	3.27747	2.49942	3.48232	3.83172	4.32445	2.70796	3.23647
SLC10A5	-2.94095	-2.5748	-1.66734	-2.85566	-0.43092	-1.30196	-0.12324	-1.50787	-2.28357	-2.20706	-3.32193	-1.08173	-1.13555	-1.67882	-1.47515	-1.45229
ZFAND1	3.93147	3.89536	3.63047	4.04772	2.85786	2.41989	2.95426	2.65523	3.11133	3.66786	2.17229	3.17624	2.95101	2.28916	1.42486	1.49259
CHMP4C	1.94237	2.00772	1.96134	2.33217	3.06472	2.86614	4.00162	2.18662	2.00576	2.22413	2.47822	2.69769	1.19105	1.68572	1.82978	1.70749
SNX16	1.39251	1.56235	1.65611	2.29373	1.06194	0.64514	1.9704	0.73284	1.73504	1.85714	1.75652	2.04332	2.28137	1.80896	1.62748	0.94384
HNRNPA1P4	3.23913	2.16307	2.6279	1.3575	0.58036	1.97839	0.94166	1.20033	2.60086	3.13941	1.67092	2.22471	-0.7795	-0.02686	1.35073	0.68055
LINC01419	-0.83845	0.44361	-0.6347	0.40481	4.46857	4.86269	4.46322	4.90499	-2.65008	-3.32193	-3.32193	-3.32193	-3.32193	-2.83822	-2.74935	-3.32193
LRRCC1	-1.81665	-1.87838	-2.02779	-1.22146	1.18085	0.65244	1.42702	0.5706	1.29341	0.96126	0.36797	1.38574	0.97138	1.23194	0.78569	0.67386
E2F5	3.14071	3.10082	3.02133	3.24859	3.55379	3.36912	3.46758	3.56008	2.8825	3.26023	2.54861	3.14975	4.12216	3.95059	2.77083	3.12923
CA13	-1.97461	-2.36179	-2.10119	-1.7614	-1.61447	-1.88714	-1.46551	-1.90514	0.72276	-0.58074	-0.35184	-0.03145	1.92019	1.29009	0.59213	0.59569
CA3	3.27434	3.61388	4.18043	2.64388	-2.54501	-3.32193	-3.32193	-3.32193	-3.32193	-3.32193	-3.32193	-3.32193	-3.32193	-3.32193	-3.32193	-3.32193
CA3-AS1	2.50003	2.66383	3.1738	1.57724	-0.05087	0.43141	-0.56417	-0.39413	-3.32193	-3.32193	-3.32193	-3.32193	-3.32193	-3.32193	-3.32193	-3.32193
CA2	-1.8222	-1.53034	-0.25794	-0.61306	5.67711	5.408	6.03829	5.16709	-2.43451	-2.88627	-3.32193	-3.32193	-2.03977	-3.32193	-3.32193	-3.32193
ATP6V0D2	-1.48258	-0.82865	-1.30231	0.01638	-1.64829	0.37766	-1.77325	-2.16699	-3.32193	-3.00157	-2.47286	-3.00643	-3.05649	-2.46982	-2.74433	-2.51056
WWP1	3.02642	3.13348	2.99115	3.82653	4.2424	3.2137	4.81084	3.18187	3.9666	3.73527	4.03616	4.70983	4.65121	4.57782	4.96281	4.4881
RMDN1	3.07088	3.3837	3.2937	3.73023	2.44721	2.22424	2.53854	2.51713	3.20782	3.2736	2.96948	3.47906	3.31057	3.24144	2.90478	2.72379
CPNE3	3.84481	3.97519	3.90031	4.2966	4.43801	2.96379	5.04106	2.79557	4.41724	4.37499	4.40671	5.03287	5.18844	4.80037	5.21977	4.94222
DCAF4L2	-2.37079	-1.8194	-2.68695	-2.19871	3.51945	3.53774	3.74275	3.65099	-2.9757	-3.32193	-3.32193	-3.32193	-3.32193	-3.32193	-3.32193	-3.32193
MMP16	-1.34121	-1.31474	-1.42187	-0.43656	-2.6208	-3.23138	-2.64567	-3.21599	1.19955	1.69244	1.47568	3.09143	-3.32193	-3.32193	-3.32193	-3.23444
RIPK2	4.62649	5.02197	4.6454	5.06261	4.70561	4.09536	4.88726	3.80325	3.61198	3.84422	3.31145	4.2431	4.71474	5.20688	5.28072	4.96358

OSGIN2	3.3803	3.53152	3.40989	4.19949	4.20725	3.32058	4.62419	3.36672	2.64335	2.6303	1.84956	3.2948	5.07872	5.69366	5.06273	5.49028
NBN	3.96648	4.1309	3.74023	4.7505	4.04353	3.03465	4.6595	2.93974	3.72098	3.46648	3.50068	4.29933	4.89343	5.12479	4.70265	4.71867
DECR1	3.03559	3.12832	2.9504	3.6379	4.30813	4.80633	4.43189	4.7773	3.39127	3.6802	3.51706	3.66299	4.3774	4.44502	4.29606	4.42785
CALB1	-2.69558	-2.7484	-1.76993	-2.82441	5.00914	3.32108	5.62631	3.26191	-3.32193	-3.17808	-3.32193	-3.32193	-2.99361	-3.32193	-3.32193	-3.17644
TMEM64	2.28484	1.09296	1.807	0.52135	4.11035	1.94728	4.4436	1.37002	4.05228	2.24904	3.93838	2.30032	5.15498	3.33502	5.65693	3.21517
NECAB1	-3.00899	-2.08349	-2.80544	-1.7429	-0.30426	0.26559	0.72148	-0.11852	1.28509	0.67351	1.04955	0.69208	1.3332	0.62025	0.96362	-0.5333
C8orf88	-2.42032	-1.34989	-0.47974	-0.89331	3.0948	2.37601	3.76043	1.76734	4.40532	3.34281	3.93535	3.31846	4.33369	3.11088	3.55912	1.76725
PIP4P2	2.91983	2.73006	2.61541	3.74004	2.18955	2.75036	2.83143	2.43272	2.78594	2.8729	2.11169	3.02061	3.38297	3.56535	2.6244	3.00533
OTUD6B-AS1	1.54384	1.70502	1.48976	2.17069	1.89759	1.62784	2.38846	1.28397	1.20596	1.53101	1.17261	1.60922	2.7436	3.24901	2.03401	2.15331
OTUD6B	3.31828	3.03641	2.73685	3.08343	2.67411	1.69478	3.25615	1.71369	2.63798	2.37846	2.36573	2.38532	3.49324	3.09677	2.71896	2.35294
LRRC69	-0.9112	-0.7745	-0.39226	-0.63256	-1.99421	-0.64558	-0.92491	-0.44547	-2.94875	-2.52724	-2.11063	-1.77296	-0.52099	-0.36947	-0.8347	-0.20375
TRIQK	0.85463	1.27442	1.30201	1.64655	1.54535	1.0754	1.88278	1.07341	0.98428	1.12525	0.51325	1.84861	1.50587	1.23428	1.30413	0.82136
FAM92A	2.49072	2.3099	2.45963	2.73266	1.38099	1.30334	1.77739	1.47184	2.7596	2.58694	2.35165	2.94345	3.14672	2.48364	2.89668	2.21427
RBM12B	2.7269	2.3003	1.99513	2.13609	2.70032	1.79425	2.89008	1.94275	3.18586	3.15421	2.98176	2.79652	2.93857	2.51874	3.03608	2.78875
RBM12B-AS1	2.05	1.78034	1.23448	1.13789	2.56193	1.09957	2.93436	-0.93208	2.45626	2.6263	2.39815	2.87009	2.70595	2.27259	1.76163	2.64322
TMEM67	1.17952	1.06225	1.11455	1.79085	0.50743	0.11838	0.92766	-0.04468	0.98889	1.37037	1.07044	1.85536	1.53846	1.62872	1.19116	1.20427
PDP1	4.48553	4.86967	4.75473	5.17622	2.05836	1.43562	2.80029	1.73518	4.58458	5.12829	4.59738	5.34659	4.71859	4.19793	4.51311	4.43789
RPL34P18	1.66422	1.82583	2.47368	1.88676	-0.24313	1.46469	0.95744	1.80323	1.53766	2.18261	0.93883	0.4298	-0.34548	0.17235	-0.45805	-3.32193
CDH17	-2.83866	-3.32193	-2.76473	-2.99866	4.00856	-0.11287	4.48329	-1.29467	-3.32193	-3.06872	-3.32193	-3.32193	-3.32193	-3.32193	-3.07102	-3.06594
GEM	4.49888	4.39162	4.35145	4.43772	1.42047	0.93183	2.26476	-0.26519	2.21135	2.61355	1.56273	2.08408	2.96205	2.67111	2.09408	2.82935
RAD54B	2.05694	1.68493	1.69263	1.69376	2.31869	1.2831	2.83236	1.23801	1.68135	1.30248	1.48215	1.38213	3.45727	3.63675	2.74049	2.82189
FSBP	2.35215	1.95532	2.09312	2.0934	2.58752	1.71594	3.17672	1.70339	1.80149	1.51968	1.72088	1.82228	3.8276	3.97244	3.01668	3.00686
VIRMA	3.2841	3.45227	3.1855	3.49679	2.8825	2.14175	3.41867	2.04249	3.47754	3.50808	3.3714	4.29345	3.50184	3.58661	4.00978	3.94562
DPY19L4	2.50576	2.68609	2.82452	3.24661	3.18556	2.18701	3.73313	2.11009	2.41516	2.51374	2.26253	3.02725	3.54486	3.22696	3.20269	3.31769
INTS8	3.44288	3.27328	3.2618	3.67582	3.62335	3.14225	3.9334	3.12333	3.21691	2.60065	3.1016	3.2222	3.86573	3.69804	3.57789	3.33412
CCNE2	2.2669	2.16298	2.28948	2.76705	1.91583	1.87551	2.47344	2.14563	1.86767	0.94204	1.39143	0.98121	3.81987	3.60041	2.81623	2.42516
NDUFAF6	1.90098	1.60278	1.37713	1.86119	1.29971	1.01664	1.39684	0.93787	0.817	0.60911	0.62607	1.13871	2.36445	2.36735	1.71414	1.49534
TP53INP1	0.23906	0.86308	0.41269	1.62587	1.12367	1.27657	1.08179	1.85318	1.44492	2.17962	1.49554	2.63954	2.34214	2.89812	3.03459	3.15275
PLEKHF2	1.84337	1.51054	1.86657	2.01269	2.23014	1.03644	2.89607	1.18751	2.7778	2.43199	2.7358	2.9447	3.34477	2.89139	3.28005	2.60588
C8orf37	0.68987	0.57028	0.87773	0.62291	1.05474	0.50392	1.31629	0.51504	0.53896	0.66135	0.15426	0.81157	1.4372	1.24583	1.15941	0.73767
GDF6	-3.32193	-3.32193	-3.32193	-2.7089	-3.19884	-3.32193	-3.06401	-3.32193	-2.34007	-2.17572	-2.26105	-3.06034	0.20345	0.51198	1.62444	0.55691
UQCRB	5.03942	5.12033	5.06489	5.13197	4.31846	4.88035	4.33554	4.80872	4.57163	4.70597	4.43192	3.5719	4.96259	4.85361	3.9683	3.76913
MTERF3	3.89959	3.85936	3.62679	4.01333	3.37145	3.13268	3.26202	3.33557	3.58153	3.35021	3.18868	3.12432	4.43108	4.20957	3.518	3.18579
PTDSS1	5.0246	4.65788	4.44758	4.40529	5.24574	4.03717	5.25351	3.95398	4.7026	4.18736	4.51557	4.78302	5.15407	4.69347	5.17191	4.79871
SDC2	-2.43011	-1.68385	-1.724	-2.26534	2.41667	3.0651	2.50031	3.11567	-1.94839	-2.21427	-2.70674	-1.47583	5.11675	5.51203	5.12363	5.05864
CPQ	-1.0539	-1.22572	-0.43763	-0.71276	1.91127	2.53544	1.97637	2.3637	-0.31368	0.48434	0.07527	1.12813	3.24139	3.56801	3.43528	3.22428
TSPYL5	-3.32193	-3.06616	-3.32193	-3.03722	-3.12432	-3.32193	-3.32193	-3.32193	3.20158	3.3644	3.23685	3.60187	-3.32193	-3.32193	-3.32193	-3.32193
MTDH	6.14894	5.98641	5.91262	6.47128	5.91592	5.13817	6.23546	5.28266	5.62248	5.42807	5.51226	5.72351	5.75428	5.26585	5.7562	5.48928
LAPTM4B	6.76245	6.4433	6.47387	6.60135	6.91343	6.0348	7.07463	5.7365	6.36429	5.73888	5.98698	5.7288	6.8912	6.49798	7.01994	6.74168
MATN2	-1.90013	-0.70097	-0.82575	-0.49883	0.49309	-0.00844	0.34522	0.53088	4.85603	4.54215	4.73329	4.4694	1.76078	2.28435	2.79501	1.39151
SNORA72	3.15698	2.42748	2.9565	3.9016	0.88596	2.73103	3.15078	3.40464	-0.35212	0.30329	0.39857	0.82552	2.36931	1.9153	0.29011	0.31911
ERICH5	-1.14375	-0.63455	-0.94978	0.06466	0.55228	0.3318	0.96441	0.69617	-2.30432	-2.13608	-2.77936	-1.34936	1.42738	1.61297	2.0785	1.92553
RIDA	4.50683	4.28551	4.00016	4.51865	3.18635	4.0769	3.42405	4.27528	3.40297	3.5955	3.4898	3.34313	4.30828	4.46344	3.51119	3.54845
POP1	2.75239	2.75721	2.11304	2.98455	2.76231	1.60573	2.73763	1.85589	2.93522	2.56441	2.83309	2.47484	3.50664	3.71138	3.55115	3.49726
NIPAL2	0.06019	0.41048	0.02808	0.84592	0.55749	-0.02479	0.38222	-0.13943	-0.16893	0.20912	-0.31539	1.16476	-1.9883	-2.80305	-2.19027	-1.59963
STK3	0.5224	0.29486	0.43414	0.39827	1.02321	-0.20998	1.85966	-0.19208	1.80823	0.45394	1.43291	0.53939	2.63648	1.09114	2.49069	0.3669
OSR2	0.24616	-0.14762	-0.12669	-0.37934	1.65991	1.37336	1.39068	1.40707	1.69623	1.30772	1.39206	-0.03078	1.61708	1.59245	2.1942	2.55199
VPS13B	0.07288	0.19189	0.2499	0.49869	1.00633	-0.06029	1.3473	0.07776	1.93704	1.80661	2.01237	2.72654	1.98525	2.21908	2.64451	2.92316
COX6C	5.38515	5.42635	5.50413	5.5081	4.91145	5.82564	4.89159	5.91233	4.68857	5.20022	4.94522	4.78915	5.88506	6.22946	4.64905	4.72563
FBXO43	-0.11209	0.00605	-0.55225	-0.57669	0.38957	-0.06757	0.44945	0.44572	-0.73108	-1.3106	-1.03878	-2.37233	1.17316	1.57707	1.437	0.62113

POLR2K	6.00234	5.89643	5.66151	6.29239	4.72543	4.99886	4.91671	4.83456	4.50918	4.24098	4.33378	3.85853	5.73987	5.58739	4.90397	4.27468
SPAG1	2.7708	2.32521	2.53893	2.45125	3.22541	1.88809	3.7837	1.90779	2.49374	1.59295	2.07706	2.393	3.38466	2.4259	3.04701	1.96003
RNF19A	2.35945	2.50105	2.67731	2.91777	2.96161	2.13972	3.82685	2.01758	2.95022	2.73651	2.74968	3.4966	3.52641	3.59272	3.6307	3.44127
ANKRD46	1.00502	1.21115	0.85301	1.52492	1.38349	1.16595	2.04722	1.30987	1.31445	1.91261	1.78372	2.32703	1.74378	2.32883	2.35872	2.16947
MIR7705	9.92121	10.245	10.0119	10.0762	7.94176	9.05767	9.25608	9.36821	9.3491	9.2788	8.91441	10.7735	10.6149	9.93994	9.40663	9.01768
RPS26P6	4.87362	4.45141	4.853	3.60957	5.04297	5.23868	5.35414	5.19797	5.32272	5.5139	4.93532	4.78582	5.18061	4.83179	3.61319	3.06529
ZNF706	4.44344	4.27277	4.26424	4.3859	3.42826	3.95925	3.6468	3.5939	3.47335	3.51959	3.43313	3.26218	3.76165	3.75567	3.04417	3.07008
NCALD	-3.18901	-3.03902	-3.16574	-3.32193	-1.06768	-1.11705	-1.0105	-0.75246	-3.02991	-3.07567	-2.8366	-2.60786	-2.7091	-2.33687	-1.86214	-1.18756
RRM2B	1.8747	2.27088	1.36145	2.83005	2.94354	1.59153	3.23426	1.51705	4.14915	4.22307	3.30903	4.43896	3.6471	3.57615	2.688	3.39266
UBR5-AS1	1.7354	1.61317	1.24157	2.09099	-0.20121	1.18007	0.19652	1.6225	0.80505	0.27773	0.74025	1.46237	1.81069	1.34713	0.65888	0.71697
UBR5	3.07005	3.17008	3.2354	3.61502	3.28363	2.24906	3.88641	2.23691	3.75001	3.60506	3.7894	4.50702	4.06765	4.12741	4.42439	4.49965
KLF10	3.94281	3.81019	3.79753	3.96512	3.33696	1.82788	3.80914	1.96534	5.01064	4.96569	4.61492	5.18663	4.56893	4.1147	4.06695	3.76451
GASAL1	-0.89177	0.41327	0.01444	0.31371	2.53835	1.85709	2.86203	1.73572	1.52322	0.77716	0.85217	0.79783	-0.23065	-0.30983	-1.69178	-0.59462
AZIN1	5.0274	5.01328	4.69482	4.94164	4.29974	3.18585	4.85549	3.24935	5.28545	4.73566	4.69066	4.86645	6.05601	5.9841	5.60016	5.28695
AZIN1-AS1	-0.12158	-0.11224	-0.32776	-0.39042	-0.89669	-1.33162	-1.75601	-0.79917	-1.88415	-1.38245	-0.68449	-1.74119	-1.11396	-1.0188	-0.88579	-0.48413
ATP6V1C1	4.85355	4.95251	4.77181	5.4207	4.05563	3.12272	4.79499	3.13898	3.77112	4.02737	3.8471	4.44154	4.83032	5.45552	5.17363	5.41881
BAALC-AS1	-1.39494	-1.27033	-0.68556	-0.78548	-3.32193	-3.32193	-3.32193	-3.32193	-2.55804	-2.85092	-2.45046	-2.35929	-1.71842	-1.07384	-0.83984	-1.14676
FZD6	3.73675	3.57124	3.82009	4.11549	-1.32184	-3.32193	-0.61599	-2.22738	2.61216	2.69792	2.21081	2.8028	4.9098	4.87199	4.90929	4.55011
CTHRC1	4.19903	4.55739	5.05686	5.79267	-1.55098	-2.10437	-1.71805	-1.69941	3.85044	3.90859	3.80717	3.54329	-2.56942	-3.32193	-3.32193	-3.32193
SLC25A32	4.74068	4.58137	4.15097	4.58765	3.87821	2.55506	4.14939	2.6645	4.00219	3.38169	3.29007	3.57771	5.10066	4.35717	3.91517	3.66467
DCAF13	4.44849	4.27441	4.00835	4.40232	3.44766	3.12362	3.77703	3.00499	3.52669	3.37511	3.40677	2.92341	3.79302	3.43243	3.04332	2.96619
RIMS2	-3.02567	-2.99326	-3.32193	-3.03999	-3.03762	-3.32193	-3.17607	-3.32193	1.08429	-0.3257	0.14223	-0.04425	-1.02068	-2.27391	-2.41846	-3.24385
LRP12	3.21607	3.09083	2.71184	3.68898	1.66816	0.41795	1.22514	0.89965	1.9084	1.88067	0.43293	1.94961	1.30055	1.23613	-0.04158	0.63818
ZFPM2	-1.06092	-1.4832	-1.42661	-0.58408	-2.85563	-2.73251	-3.1934	-2.11652	-1.40175	-1.97702	-1.43883	-1.27829	-1.72104	-1.84048	-0.919	-2.41371
ZFPM2-AS1	1.25073	1.04837	1.00811	1.75542	-0.75748	-0.74557	-0.84085	-0.38191	0.88978	1.32258	1.04397	1.93496	0.8726	1.3429	1.18679	0.29796
OXR1	1.56099	1.21868	1.09712	1.15798	1.76017	0.19156	2.16684	0.47612	2.08898	2.08554	1.68348	1.81109	2.91095	2.91899	2.30946	2.31889
TAGLN2P1	1.23126	1.3071	1.8886	0.52135	0.41725	-0.14865	-1.08849	-0.80646	2.23589	2.72299	1.57355	1.74799	-0.27739	-0.96337	0.4461	0.17915
ANGPT1	0.2627	0.63628	0.4616	1.18382	-0.48391	-0.74807	0.28451	-0.95897	-0.98565	0.86547	-0.15064	1.68584	-0.35399	0.94033	0.05048	0.57719
EMC2	3.35039	3.26187	2.5755	3.36231	3.35229	2.3915	2.79899	3.17566	2.91079	3.05563	1.26097	2.15669	3.9829	3.66253	1.9858	2.35272
TMEM74	-2.46649	-1.99198	-2.13391	-2.08179	-0.49975	-2.08084	-0.55816	-2.60671	0.01342	-0.36521	-0.04941	-0.77127	-1.89392	-2.61549	-2.72249	-2.11012
NUDCD1	4.66076	4.62007	4.46314	4.54484	3.73927	2.62374	4.13981	2.51996	4.53649	4.45396	4.56685	3.83674	4.40932	4.66315	4.41053	3.95803
ENY2	4.64111	4.70705	4.52159	4.76083	3.95283	4.21622	3.9573	4.16307	4.27982	4.2638	4.25108	3.28598	4.32001	4.41372	3.80241	3.845
EBAG9	2.84063	2.87144	2.45169	2.98164	2.55478	2.38891	2.88402	2.23707	2.33644	2.18671	1.94743	2.56961	2.46845	2.8134	2.37097	2.39064
SYBU	-0.85456	-1.74452	-2.28824	-2.06196	-0.54246	-1.57945	-0.41544	-1.57105	0.03525	-0.63619	-0.67451	-1.38525	-3.09156	-3.09232	-2.61443	-3.04019
KCNV1	-3.32193	-3.15223	-3.32193	-3.32193	-2.81089	-3.02929	-3.04977	-3.32193	-0.3255	-0.23334	-0.80506	-1.12518	-3.32193	-3.32193	-3.32193	-3.32193
LINC01608	1.80977	1.27311	1.2769	1.28996	2.06227	2.69273	2.75432	2.69673	-3.32193	-3.32193	-3.32193	-3.32193	-3.32193	-3.32193	-3.32193	-3.32193
CSMD3	-2.57411	-2.40395	-2.33384	-2.04148	-3.32193	-3.32193	-3.27198	-3.32193	-2.05672	-1.82652	-1.54636	-1.51286	-3.32193	-3.32193	-3.32193	-3.32193
TRPS1	0.00613	0.46723	0.21784	0.8543	-2.70287	-2.88431	-3.1445	-2.99986	0.05242	0.69678	0.51489	2.27838	0.84552	1.60855	1.69547	2.1294
EIF3H	5.97773	5.93234	5.828	5.98301	4.43034	4.64696	4.82419	4.6074	5.84311	5.77289	5.74036	6.17143	5.78227	5.65762	5.51399	5.33187
UTP23	3.61612	3.38543	3.38741	3.81101	3.26899	2.00526	3.88661	2.1338	4.00052	3.44079	3.63322	3.48481	3.94316	3.75786	4.13826	3.73596
RAD21	5.34468	5.29988	5.38945	5.62057	5.10806	4.28277	5.67162	4.14258	5.99046	5.35207	5.60551	5.61004	6.03492	5.93397	6.21475	5.68197
RAD21-AS1	0.37518	-0.50072	0.20433	0.55076	0.98979	-1.42166	1.17797	-1.81753	1.52742	1.02587	1.63229	1.2667	3.78696	3.25774	4.07041	3.38192
MIR3610	-3.32193	-3.32193	-3.32193	-3.32193	-0.85847	-3.32193	1.05993	-3.32193	-3.32193	2.38574	1.7673	-3.32193	8.31791	7.66846	8.50982	7.60847
SLC30A8	-2.32999	-2.68507	-2.30396	-2.48989	-1.76496	-0.01841	-2.21505	0.35179	-3.32193	-3.32193	-3.32193	-3.32193	-3.32193	-3.32193	-3.32193	-3.32193
MED30	3.95556	3.45003	3.63502	3.51028	2.8472	2.86656	1.97216	3.11939	3.42628	3.42686	1.98278	1.95642	2.77735	3.27066	0.8872	1.17406
EXT1	4.57337	4.56594	4.78264	4.46651	4.3205	3.07113	4.45738	3.3476	6.23425	6.14047	6.12935	6.17728	4.23023	4.50306	4.27446	4.70455
SAMD12	-0.6412	-0.81163	-0.97049	-0.87211	-1.47198	-1.71654	-0.969	-1.59194	-1.04153	-0.394	-0.92666	-0.37639	-0.34069	0.28799	-0.2329	0.47363
TNFRSF11B	-1.13005	-0.20032	-1.76047	0.50284	-3.16282	-3.32193	-2.99279	-3.32193	0.03622	0.53621	-0.25248	0.565	-3.04088	-2.8073	-2.71385	-3.32193
COLEC10	-1.40031	-1.69595	-1.91497	-2.65591	-3.06205	-3.32193	-2.80207	-3.32193	4.29684	4.90649	4.58084	4.60737	-3.32193	-3.32193	-3.32193	-3.32193
MAL2	-3.32193	-2.72935	-2.97929	-2.66997	-0.90929	-2.56803	-0.4059	-1.93523	-1.95564	-2.25775	-2.55367	-1.48435	-3.32193	-2.70848	-3.32193	-3.32193

ENPP2	-1.20262	-1.47828	-1.47543	-1.20127	-2.95019	-3.32193	-3.17441	-3.32193	0.14469	-0.34085	-0.44759	-0.40345	-3.19712	-3.19755	-2.59012	-3.32193
TAF2	2.94686	3.13126	2.86884	3.50321	3.01346	1.98966	3.59503	1.91842	3.05288	2.93014	2.97703	3.61452	3.63831	4.22432	3.88444	3.80313
DSCC1	3.8462	3.37025	3.01494	3.09279	3.70226	2.58706	3.87068	2.96267	3.18759	1.54482	2.39459	1.13203	4.33214	4.16049	3.46726	3.17516
DEPTOR	-1.70274	-1.3255	-1.86071	-2.00306	-0.01076	2.79152	-0.44321	2.51932	-1.70634	-1.00113	-1.81612	-0.72476	0.16395	1.74765	1.46285	2.84339
MRPL13	3.61274	3.5421	3.39512	3.63391	3.51698	3.51543	3.7867	3.50477	3.40923	3.28354	3.28772	2.89566	4.39553	4.55405	3.56592	3.51785
MTBP	1.17204	1.04753	0.7287	0.83413	1.46074	0.02036	1.72716	0.44779	0.86614	0.26978	0.53797	0.78742	1.80864	1.45496	0.87615	0.50489
SNTB1	1.10271	1.0444	1.0633	1.12207	3.81079	3.3064	3.71066	3.33455	2.02543	1.87648	2.04953	2.48723	-1.78961	-0.60659	-1.29507	-0.34968
HAS2	-1.40412	-0.38591	-0.72563	-0.36192	-3.32193	-3.32193	-3.09184	-3.32193	-0.97403	-0.96911	-0.83513	-0.22588	1.23223	1.27979	2.59084	2.856
HAS2-AS1	-2.12254	-1.76604	-1.97943	-2.25769	-3.32193	-3.32193	-3.32193	-3.32193	-1.99602	-1.20634	-2.7882	-1.37047	-0.42809	0.10875	1.21906	1.27465
ZHX2	2.60264	2.1082	2.56795	2.0463	3.22451	2.67575	3.03216	2.44461	1.52292	1.3198	1.50834	1.80136	2.89455	1.65883	2.82621	1.8676
DERL1	4.08571	4.29271	4.24744	4.30566	4.42621	4.17415	4.89182	3.89059	3.2229	3.15106	3.53349	3.34308	3.7322	3.87996	4.26759	4.03098
TBC1D31	2.96129	2.77773	2.78923	2.77313	2.99738	2.03262	3.18087	1.71071	2.17667	2.004	2.24788	1.49422	2.64879	2.65666	2.69714	2.5748
FAM83A	2.66201	3.40145	3.91992	1.79315	-3.17872	-2.62494	-3.32193	-3.12515	0.29654	0.06737	-0.29911	-0.50296	-3.06793	-3.1898	-3.01788	-2.64432
FAM83A-AS1	1.00102	2.039	2.04525	-0.15182	-3.32193	-3.32193	-3.32193	-3.32193	-3.32193	-3.32193	-2.18851	-1.92212	-3.32193	-3.32193	-3.32193	-3.32193
C8orf76	3.94683	3.94058	3.95356	3.887	3.94097	3.39687	3.96397	3.078	3.54293	2.91893	3.35554	2.62159	3.95459	2.9881	3.49872	2.64158
ZHX1-C8orf76	4.75727	4.74376	4.82143	4.76755	5.01417	4.17824	5.08046	3.94742	4.6837	4.29182	4.55224	3.94729	4.94701	4.11211	4.86695	4.06471
ZHX1	1.92441	2.26031	2.04876	2.96945	2.41999	1.62297	2.97442	2.1509	2.42208	2.59714	2.56571	3.47133	2.70136	3.15657	3.3114	3.09957
ATAD2	4.23432	3.86086	3.59855	3.82172	4.74423	3.38264	5.15011	3.61493	4.47517	3.16914	3.30478	3.06332	5.5558	5.61997	5.03739	4.88262
WDYHV1	3.20177	3.44452	3.20163	3.37769	2.59381	2.41319	2.58799	2.90767	2.99465	2.64227	2.25587	2.62616	4.13606	4.26493	3.24023	3.51057
FBXO32	5.50621	5.83072	5.56715	6.2303	-1.96574	-2.81152	-1.70967	-3.00039	1.17585	2.35036	1.68235	3.87538	-0.79363	0.02518	-0.23188	0.31857
KLHL38	2.56916	3.15224	3.69494	2.89068	-3.09998	-3.32193	-3.32193	-3.32193	-2.78075	-3.32193	-3.32193	-3.32193	-3.32193	-3.32193	-3.32193	-3.32193
ANXA13	-1.16273	-0.11408	-1.27207	-0.9661	-0.88446	-2.45426	-1.1151	-1.89014	-3.32193	-3.32193	-3.32193	-3.32193	-2.92192	-0.29275	-0.9205	-0.89487
FAM91A1	4.35334	4.37197	4.15397	4.69333	4.08511	2.71961	4.80502	2.61793	3.80419	3.36667	3.39494	3.8967	4.76708	4.07807	4.34755	3.88614
FER1L6	-3.14782	-2.95688	-2.93908	-2.9172	0.05235	-2.98923	0.20613	-3.11709	-3.32193	-3.32193	-3.14194	-3.32193	-3.32193	-3.18426	-3.15526	-3.32193
TMEM65	2.02853	2.95616	2.72852	3.04808	2.06149	1.3312	2.66504	1.34103	0.87068	1.99849	1.11497	1.8166	1.89124	3.49818	3.01823	3.52459
TRMT12	2.91805	2.96319	2.31942	3.06702	2.90605	2.31653	3.2458	2.27866	1.82555	1.80873	2.21815	1.81103	3.04237	2.89834	3.10227	2.9209
RNF139-AS1	-1.7857	-2.31923	-1.87745	-1.44174	-1.65115	-1.42952	-1.64781	-1.73968	-3.32193	-3.04027	-3.16385	-2.35569	-2.05817	-1.0733	-2.00337	-1.7546
RNF139	3.69478	3.82775	3.59949	3.98632	4.09128	3.59037	4.60144	3.46029	3.5477	3.77131	3.62165	4.34674	4.19346	4.50012	4.37231	4.44518
TATDN1	5.3954	5.51866	5.4479	5.3658	4.69764	4.51805	4.86474	4.64185	4.44086	4.18022	4.38068	3.73635	4.79787	4.64191	4.70127	4.78175
NDUFB9	7.329	7.27433	7.20544	7.07812	6.58801	6.82495	6.43997	6.97987	6.0978	5.70821	5.96551	5.3591	6.57397	6.34868	6.04497	6.29442
MTSS1	2.8033	3.15641	3.20028	3.09505	2.48948	2.69094	2.63746	2.64169	2.53831	2.93454	2.88026	3.41668	-1.26966	-0.22984	-0.69468	-0.58099
ZNF572	-0.74259	-0.72408	-0.53618	0.12069	-1.8305	-1.77751	-1.9723	-1.01561	-1.6422	-0.15228	-0.75877	0.34281	-0.52676	0.55482	-0.2021	0.70163
SQLE	4.14091	4.29327	3.92402	4.34896	5.09605	5.38311	5.38604	5.40405	4.57038	4.38609	4.24511	4.19391	5.78874	6.40782	6.23922	6.38903
WASHC5	3.60335	3.41184	3.57535	3.67551	3.95028	2.58223	4.31854	2.50502	4.31607	4.17092	4.20968	4.87387	4.79739	4.81256	4.8356	4.95951
WASHC5-AS1	4.04397	3.97106	3.96417	4.20636	3.7571	2.89773	4.20862	3.3455	4.41336	3.94483	4.12888	5.21241	4.76759	4.86311	4.61256	4.8058
NSMCE2	3.8109	3.98169	4.03773	4.30411	3.44042	3.00418	3.8201	2.81645	3.32091	3.61892	3.42558	3.32032	4.02698	4.23085	4.01159	4.11851
TRIB1	3.7399	4.01458	3.73566	3.90395	2.52099	2.40491	2.99293	1.93566	2.28524	2.65618	2.31525	3.53212	5.16805	6.16973	6.15255	6.26938
CASC19	-1.08789	-1.3146	-1.70361	-1.74404	-0.33434	0.51815	0.25439	0.87775	-3.26448	-3.32193	-3.32193	-3.32193	0.57349	-0.32974	-1.297	-0.50708
PRNCR1	-1.56563	-1.50123	-1.50281	-1.59531	-2.24217	-2.33169	-2.30428	-2.3637	-1.85096	-2.01734	-1.50351	-1.61944	-2.12663	-2.32214	-1.86424	-1.84409
CASC8	-1.74257	-1.62962	-1.41571	-2.81771	-3.13195	-2.90502	-3.32193	-1.95643	-3.32193	-3.32193	-3.32193	-3.32193	1.45846	2.16924	-1.95345	-1.13374
MYC	4.2794	3.19939	2.75462	1.67879	5.19503	4.27586	4.57034	4.36579	7.03954	6.70279	6.80262	4.90269	6.1226	5.61879	5.83201	5.0023
PVT1	1.96376	1.81783	1.13252	1.20899	0.04281	0.50007	-0.06299	0.84588	2.50271	2.27543	2.28874	1.60729	2.74119	2.40464	1.54567	1.79258
MIR1204	3.22761	2.98205	1.5083	1.59679	1.35607	1.85346	2.46112	1.51379	5.07731	4.33675	4.27055	3.84558	5.07374	4.71832	3.85576	5.18085
RNU4-25P	3.03539	2.21567	1.72169	1.40922	0.4749	0.85272	-1.26887	0.38303	2.26507	3.04175	2.67633	2.29232	2.95727	3.0472	0.09292	2.23579
FAM49B	2.83534	2.6985	3.09555	3.15519	2.18375	1.16328	3.06187	1.26591	2.1463	2.04404	3.00904	2.26703	3.13571	2.82095	3.94368	2.3176
ASAP1	3.78407	3.51885	3.49319	3.61501	2.91159	1.55272	3.07995	1.5463	5.76905	5.45443	5.24568	5.70814	4.38316	4.16683	4.24391	4.15897
ADCY8	-2.29223	-2.79745	-2.77324	-1.83238	-3.03365	-3.32193	-3.32193	-3.32193	-2.94045	-3.32193	-3.13939	-3.32193	-1.41348	-2.10699	-0.83665	-1.49771
EFR3A	3.67519	3.46971	3.57599	4.30627	4.0554	2.5544	4.72459	2.2198	3.8949	3.55363	3.67681	4.64164	5.02447	4.91567	4.88631	4.89897
KCNQ3	0.2163	-0.32963	-0.22401	-0.48991	-3.32193	-3.32193	-3.32193	-3.32193	-1.70426	-2.23484	-2.13824	-2.37266	-3.32193	-3.18992	-3.0883	-3.23801
LRRC6	0.63486	1.11744	1.21265	1.63128	-3.01472	-2.43641	-2.11783	-2.58675	-0.35484	-0.27335	-0.23024	1.09718	-2.25541	-1.30282	-2.98081	-1.78167

PHF20L1	1.58016	1.56896	1.54177	2.38408	1.10502	0.65302	1.3128	0.98242	1.51744	1.54577	1.02204	2.03139	1.88988	1.65054	1.62246	1.69462
SLA	-2.2393	-2.47516	-2.64206	-1.54054	-3.32193	-3.32193	-3.32193	-3.32193	-1.97958	-2.40968	-1.8418	-2.49758	-3.32193	-3.32193	-3.32193	-3.18551
ST13P6	0.02229	0.06845	0.24452	-0.15172	-0.04778	-0.3003	-0.28077	-0.94663	1.48619	0.73294	1.14784	0.6497	-0.88522	-0.88942	-0.50395	-0.22429
ST3GAL1	5.0408	5.58203	5.2587	5.97603	1.3826	1.06745	1.66107	0.50784	1.69679	1.73654	1.14357	1.97022	0.94486	0.77423	0.84623	1.99536
ZFAT	1.03801	1.10799	1.01552	0.84426	0.821	0.23349	0.41931	-0.23991	1.18216	1.14997	0.82478	1.1906	0.46925	0.39895	1.11978	1.33648
ZFAT-AS1	0.23205	0.38518	0.10999	0.43521	0.51998	-0.54586	0.49733	-1.75332	1.08239	1.06528	-0.06455	1.54636	0.54579	0.72119	1.20764	1.37875
KHDRBS3	-0.13925	-0.03407	-0.00951	-0.66047	0.71404	-0.11089	0.57638	-0.47527	0.9221	0.75211	0.21442	-0.12495	-0.91856	-1.34245	-1.0675	-1.46263
LINC02055	1.09517	1.19476	0.79162	1.20706	-1.64719	0.11898	-0.99543	0.37183	-3.32193	-3.32193	-2.94661	-3.32193	-3.32193	-3.32193	-3.32193	-3.32193
KCNK9	-3.32193	-3.32193	-3.32193	-3.03478	-2.94747	-3.32193	-3.10428	-3.04992	-3.05557	-3.09762	-3.32193	-3.32193	-1.53115	-0.66405	-0.48146	-0.32558
TRAPPC9	0.22293	1.08747	1.13641	1.12641	2.23474	1.84054	2.21375	1.78646	2.19392	2.42269	2.42361	3.0362	1.67927	2.11563	3.26385	3.19547
PEG13	-2.45662	-2.63051	-2.02311	-2.06907	-3.16316	-2.34425	-3.32193	-1.84071	-3.32193	-2.98378	-3.32193	-2.98889	-2.5137	-2.00187	-0.65266	-0.51165
CHRAC1	4.82823	4.55181	4.39362	4.23714	4.10895	3.16844	4.27243	2.91432	4.03698	2.85182	3.63511	3.41481	5.04991	3.81805	4.83725	3.58041
AGO2	2.58425	2.48593	2.58085	2.4004	3.16457	1.93062	3.18374	1.69195	3.11215	2.95824	3.05946	3.59842	4.90192	4.88507	5.64249	5.37078
ERICD	-1.28636	-1.86794	-1.81796	-1.17655	-0.14184	-0.24407	-0.21835	-0.03556	-2.78549	-1.6251	-2.83506	-2.25018	0.09949	1.31591	1.54181	2.02515
PTK2	3.44158	3.49273	3.51788	3.88142	3.00668	2.37515	3.61243	2.32303	3.67469	3.7447	3.87176	4.45404	4.59831	4.6679	5.08132	5.07789
DENND3	1.82112	1.95702	2.05494	1.98805	0.09244	-0.26391	0.01949	-0.15335	1.85008	1.70395	2.02042	2.06956	2.80328	3.06848	2.74456	3.00728
SLC45A4	-3.32193	-3.32193	-3.17551	-3.16641	2.47124	2.62131	2.53758	2.44944	-0.14087	0.77269	0.45447	0.85557	-3.32193	-3.2238	-2.72118	-2.98398
PTP4A3	-1.79167	-1.6806	-1.49135	-2.08179	-1.45992	0.67821	-2.31671	-0.14854	0.05755	1.8423	1.00024	1.52984	-2.14047	-1.19448	-1.59159	0.03054
TSNARE1	2.1837	1.57989	2.14979	1.49583	-0.40353	0.08953	-1.02444	-0.16191	2.04757	2.12027	2.17793	1.59864	1.5329	0.92693	1.80174	1.8474
ADGRB1	-3.32193	-3.16046	-3	-3.14153	-2.9784	-2.80924	-3.18641	-3.15137	-1.25546	-0.16232	-0.46791	-0.6894	-3.32193	-3.32193	-3.32193	-3.32193
ARC	0.0842	0.12465	0.53868	-0.40517	-2.79504	-2.2867	-3.00901	-2.37639	-1.21164	-1.62061	-2.26865	-3.00463	-3.32193	-1.72469	-2.74131	-2.14171
JRK	1.07815	0.76393	0.78352	0.13053	1.59352	0.68659	1.39809	1.20364	2.35648	2.38921	2.67343	2.18556	2.03618	2.55151	2.88723	3.03337
PSCA	0.31568	-0.08928	0.12557	-1.20608	-2.23664	-2.40339	-2.82529	-1.09997	2.84026	2.65486	2.88689	1.18278	1.66425	1.47536	0.79842	1.19596
LY6K	-1.68571	-1.87771	-1.61224	-2.53455	-2.82926	-2.20901	-3.32193	-2.38688	6.24894	5.57131	5.81138	4.4181	2.48831	1.67691	0.7744	1.15862
LNCOC1	-2.07981	-2.38116	-1.84713	-2.72059	-2.77208	-2.45139	-2.61062	-2.12076	5.22304	4.57929	4.80997	3.62135	1.33816	0.41842	-0.54236	-0.2184
THEM6	3.97723	3.63771	3.48621	2.64563	4.39672	4.03431	3.32714	4.02914	3.53444	3.67693	3.17341	2.43896	4.20559	4.70157	4.36767	4.80943
LYNX1-SLURP2	-3.32193	-3.32193	-3.32193	-3.32193	0.136	-1.13072	-0.29781	-0.60062	0.61843	1.3437	1.50637	-0.73299	-0.83487	0.08934	0.74142	1.54177
LYNX1	-3.32193	-3.32193	-3.32193	-3.0648	0.15158	-0.4639	-0.53229	0.02727	0.63696	1.12854	1.25665	0.47432	-0.66035	0.80967	1.35846	2.4671
LY6E-DT	-2.63998	-2.23077	-1.90482	-1.84683	-1.79481	-1.37659	-2.11018	-1.90125	0.20616	0.60213	0.34319	0.10284	-3.11053	-2.92742	-2.3499	-2.65316
LY6E	4.7102	4.94735	4.8653	4.81353	4.92019	5.16661	4.12913	4.7821	6.91396	6.88303	6.79424	6.08015	4.27115	3.93476	4.31993	4.40507
C8orf31	-2.27934	-3.32193	-2.92804	-2.90562	-1.34888	0.26116	-1.87229	0.50965	-1.4066	-0.3877	-1.04603	-0.50866	-3.32193	-3.32193	-3.32193	-3.32193
ZFP41	2.2064	2.61197	2.51821	1.76109	2.35027	1.7771	1.91437	2.09359	1.43887	1.90271	1.67583	1.41473	1.795	2.71414	2.86064	3.48585
GLI4	3.57357	3.99234	4.4671	4.0948	3.6073	3.90081	2.25357	4.01209	2.1971	2.87227	2.91012	2.40564	1.91825	1.53331	1.48057	1.81518
MINCR	2.22803	2.10973	2.3798	2.48776	1.54363	2.91232	1.14672	2.83395	-0.20008	0.62127	0.31557	-0.09253	2.94798	2.58683	0.3929	0.82465
ZNF696	2.91236	2.89089	2.58257	2.27432	2.92777	2.79205	2.41593	2.92181	2.12095	2.40215	2.54386	1.40551	2.4582	2.6395	2.75469	2.25754
TOP1MT	3.45851	3.22301	3.13414	2.83652	3.13244	3.76283	2.60763	3.53356	3.47907	3.55512	3.60535	3.08094	3.11943	2.97113	3.35063	3.54045
RHPN1-AS1	-0.87536	-0.97493	-1.36721	-0.71115	0.54367	0.61538	0.25747	0.3821	-2.05227	-0.62976	0.03171	-1.04809	-0.32657	0.5443	0.41823	1.02498
RHPN1	3.42255	3.37186	3.52009	2.62819	2.93457	4.58081	0.86324	4.04983	0.15873	2.22479	1.50415	0.93845	1.07809	2.17928	1.12305	2.00113
ZC3H3	4.00496	4.15931	4.01069	3.71137	3.5636	3.61797	2.96149	3.76913	4.16934	3.88198	4.04808	3.5009	3.90015	3.98656	4.7755	4.67903
GSDMD	4.3801	4.31614	4.22926	4.19779	3.11052	4.0033	2.37398	3.83435	4.69868	4.81398	5.102	4.30867	3.96768	4.20046	4.31782	4.34817
MROH6	-1.26213	-0.16733	-0.46238	-0.5143	1.65921	1.73213	0.24455	2.09338	0.76242	0.07148	0.58925	-0.2903	-0.00491	0.68878	-0.85235	0.91605
NAPRT	4.24257	4.62231	4.5844	4.59055	2.52687	3.67717	0.88061	3.70288	0.79973	0.49833	1.26178	-0.62236	4.0139	4.44654	4.14506	4.73028
TIGD5	3.30409	3.70012	3.69248	2.60848	3.40519	3.27061	2.15102	3.6905	3.05622	3.41127	3.3919	2.25352	1.63783	1.91674	2.32025	2.51973
PYCR3	3.8842	3.5184	3.34514	2.77934	3.90357	3.51053	2.86612	3.38655	3.1106	3.04736	3.49224	1.99218	3.51598	3.27795	3.68837	3.54889
TSTA3	4.24952	4.38405	4.39183	4.18133	5.00439	5.59669	4.41697	5.41231	4.77993	4.49969	4.96242	3.60241	5.19094	5.00147	5.30612	5.18342
ZNF623	2.24416	2.2351	2.352	2.49899	2.26728	1.62101	2.30921	1.99111	2.26947	2.466	2.51175	3.01977	2.86421	3.08494	3.32234	3.38319
ZNF707	2.55825	2.48377	2.3707	2.15428	1.66145	1.28196	1.42026	1.32619	1.43792	1.10984	1.47576	0.7093	1.55336	1.78325	2.02097	2.17543
MAPK15	-3.32193	-3.32193	-2.64227	-3.32193	-0.19002	-0.58876	-0.68609	-0.63548	-2.94875	-1.64201	-1.17782	-1.77296	-2.83864	-2.64858	-2.53331	-2.51993
FAM83H	0.21036	1.05111	1.28795	1.10085	5.94252	4.83691	5.34442	4.92691	0.92826	2.13815	2.21738	1.87915	2.0546	1.60223	2.91033	2.91096
MIR4664	-3.32193	-3.32193	1.42766	0.56556	7.24267	5.3179	5.67578	5.33648	1.4012	4.60551	4.35638	4.06009	0.8582	1.81284	1.12917	2.12663

IQANK1	-2.49092	-2.71353	-2.68623	-1.95831	-0.27267	-1.1632	-0.29949	-0.3826	-2.1408	-2.39491	-2.12591	-2.65224	-2.63466	-1.70491	-1.03886	-1.05871
SCRIB	6.68957	6.10397	6.66629	4.80063	5.89024	5.04547	4.80457	5.16151	6.23135	4.86335	6.64016	3.7704	5.34502	4.32137	6.6091	4.91308
MIR937	6.34558	5.69375	6.71258	5.19207	7.42789	4.68082	6.91364	4.80779	7.52921	5.97172	7.59711	5.33307	6.85861	6.15491	8.51685	6.71289
PUF60	6.04674	5.90896	5.80932	5.50933	5.50882	4.98429	5.11771	4.98508	5.92302	5.59345	5.97444	5.11421	6.15709	6.08753	6.28789	6.06819
NRBP2	2.61379	2.28065	3.21817	2.37127	3.04968	2.7264	2.22972	1.95168	2.87155	2.39302	3.27572	2.21489	3.54936	1.88955	3.6697	1.80072
EPPK1	-1.81448	-2.41811	-1.57523	-3.00588	1.3846	-0.3921	1.52596	-0.2151	-2.2715	-0.79323	-0.39935	-1.77047	0.91233	2.16492	4.40424	4.47855
PARP10	4.65676	4.9029	4.79585	4.0866	2.88635	2.65635	2.42214	2.68931	4.75813	5.49009	5.75223	4.85451	3.37994	3.64531	4.26482	4.61205
GRINA	7.19818	7.36043	7.39377	7.21452	6.61713	6.95061	6.14504	6.94195	6.27007	6.61271	6.38631	6.62393	6.90282	7.02557	7.27572	7.60605
SMPD5	-0.0409	-0.15068	0.7222	0.32298	0.92699	2.22306	-0.69432	1.7455	-0.10573	0.31811	0.00487	-0.29696	-0.53528	-0.99349	-0.95374	-0.01497
OPLAH	2.36126	2.65149	2.90969	2.48023	3.29809	3.90462	1.82775	3.54732	2.70565	3.0969	3.17335	2.44309	1.99231	1.26298	2.01008	2.22537
EXOSC4	6.398	6.44953	6.18398	6.03622	5.55751	5.42402	4.98943	5.32395	5.33735	5.10693	5.23432	3.82975	5.56217	5.44313	5.53583	5.33503
MIR6847	4.49802	4.59021	4.59016	4.4195	3.47085	3.89574	3.08739	4.74724	3.20977	1.74587	2.5671	2.12607	2.42955	3.2867	2.13608	1.1987
GPAA1	6.70803	6.67327	6.70014	6.41508	6.65524	6.73695	5.77699	6.78742	6.49951	6.03657	6.33754	5.44094	5.81907	5.90914	6.3407	6.52961
CYC1	8.22663	8.12065	8.05393	7.6282	7.42364	6.90895	6.48312	6.83534	7.58352	7.02238	7.45245	5.94674	7.63916	7.18648	7.51988	7.15781
SHARPIN	5.50519	5.69255	5.83524	5.12592	5.91587	6.04676	4.88469	5.99293	5.77828	5.61837	5.88775	4.60074	5.22981	5.1984	5.71498	5.5898
MAF1	6.77283	6.61073	6.64522	5.90965	6.80155	6.27579	5.85819	5.90746	6.87869	6.42244	6.877	5.47028	6.5907	6.02733	7.27092	6.79338
HGH1	4.40969	4.20612	3.89427	3.35502	5.33861	4.75989	4.64804	4.78094	3.85742	3.90885	4.03187	3.1257	4.5639	4.74983	5.18429	5.06847
TSSK5P	0.85711	0.20975	1.01875	-0.91024	3.23877	0.87212	2.75052	1.60955	1.31447	2.26404	1.93474	1.84324	2.61669	3.46664	3.49731	4.07762
MROH1	3.85023	3.9833	4.09393	3.76812	2.151	2.38527	1.31349	2.24223	2.25046	2.64711	2.5777	2.70134	2.23633	2.51552	2.7174	3.06701
BOP1	6.54789	6.1505	6.023	4.96891	6.13381	6.01987	5.24496	6.17673	6.87058	6.49582	6.96587	5.19575	6.56617	6.32676	7.00315	6.75491
MIR7112	5.16676	5.7106	5.54787	0.68468	3.55625	4.18656	0.28043	3.68726	3.29502	3.53779	2.91105	2.21031	3.50147	1.93675	2.53606	3.55485
SCX	4.43948	4.96084	5.26608	3.43166	1.47125	3.6809	-0.40991	3.9047	0.00437	0.6073	0.77369	-0.02357	0.46273	-0.38247	-0.24855	0.62338
HSF1	5.46479	5.35195	5.32386	4.58546	4.72539	4.87807	4.0511	4.90192	5.54095	5.28936	5.43504	4.87883	5.56633	5.46005	5.87292	5.80113
DGAT1	4.73997	4.44339	4.4654	4.10238	4.73633	5.17153	3.61303	5.135	4.29449	4.15822	4.29005	3.88692	4.26452	4.09207	4.39459	4.45161
MIR6848	6.80843	6.07255	6.22829	6.80831	6.77896	7.63315	6.26089	7.10066	5.89197	5.9814	6.41101	5.80239	6.02783	6.07919	6.10659	6.49032
TMEM249	0.57498	0.58859	0.69855	-0.16904	-0.33374	0.33246	-1.47621	0.46568	-1.24108	-0.73093	0.32386	-1.46007	0.14671	-0.00727	-0.2693	-1.18762
SLC52A2	7.60246	7.69483	7.60963	6.90862	7.54265	7.68491	6.58821	7.6673	6.34317	6.17197	6.52637	4.79363	6.25246	6.05944	6.6761	6.63208
FBXL6	6.18751	6.35298	6.29878	5.35769	6.34708	6.21064	5.48099	6.15687	5.14718	5.10826	5.45333	3.62919	4.89327	4.96312	5.30976	5.29416
ADCK5	2.94749	3.04142	3.34007	3.26911	2.69672	3.23281	1.65004	3.42924	2.46691	2.46769	2.65907	1.86471	2.17402	2.5252	2.58935	2.66506
CPSF1	5.41452	5.42565	5.36451	4.80398	5.50828	5.47338	4.49076	5.45114	5.89299	5.67743	5.95642	5.36675	5.11759	5.40521	5.75497	6.01681
MIR939	8.32199	8.11014	8.17693	8.18806	7.70269	8.2408	6.89931	8.53761	7.9392	7.27002	7.74705	8.1884	7.54658	7.48305	7.84883	7.3701
SLC39A4	0.39861	1.1675	0.75653	0.54863	3.2185	4.64292	2.15752	4.56156	1.90251	1.89275	1.60377	-0.01197	-0.19565	0.38271	-0.20825	0.06771
VPS28	5.0165	5.02767	5.3835	4.64107	4.50565	5.23495	3.38687	5.1039	5.16814	5.33095	5.50763	4.5308	4.31079	4.21367	4.18176	4.12949
TONSL	4.15759	3.60195	3.64837	2.85542	3.79456	3.21433	2.59444	3.62395	3.47402	2.71648	3.2896	2.08362	3.35025	3.65914	3.60489	3.96911
TONSL-AS1	4.57623	4.1708	4.28059	3.17438	4.54587	3.47258	2.91605	4.06244	3.95297	2.92962	3.98911	0.56096	3.88094	4.36639	4.46517	4.69689
CYHR1	3.33059	3.48419	3.83752	3.24646	2.98741	3.43348	2.02419	3.28806	2.68538	2.98324	3.08396	2.4667	2.62377	2.64749	3.23228	3.40408
KIFC2	3.81016	4.47734	5.07996	4.08923	4.58166	5.20592	2.94329	4.66819	2.46903	3.37847	3.06705	2.46211	3.03944	3.86752	3.7185	3.98678
FOXH1	2.36576	2.61375	3.30329	2.8761	2.6347	3.61638	1.28951	3.37709	0.65784	1.7481	1.61127	1.18602	2.08881	2.72494	2.09567	2.46373
PPP1R16A	5.56834	5.5743	5.70988	4.48047	4.54581	5.36426	3.18038	5.29268	4.42258	4.84442	4.8228	3.80059	4.27674	4.85715	4.62375	5.12713
GPT	-1.38982	-0.96887	-0.99897	-1.25169	1.35153	3.34354	0.32955	3.43542	-1.60326	-1.32658	-1.71685	-1.55525	-1.29028	-0.88984	-1.54801	-1.12892
MFSD3	5.33843	5.74339	5.68357	5.20405	5.658	6.56418	4.55258	6.51252	3.66328	4.88839	4.48932	3.26071	3.03649	3.88328	3.6988	4.33688
RECQL4	5.71428	5.31209	5.50398	4.55362	5.35807	5.12166	4.02494	5.3314	5.06763	4.34175	4.84982	3.05884	5.0674	5.28342	5.48919	5.39144
LRRC14	3.21458	3.34159	3.57571	2.98916	3.66766	3.32539	2.88225	3.51075	3.75265	3.83398	3.78995	3.47969	3.34875	3.61296	3.94605	3.84116
LRRC24	3.84585	3.94842	4.30875	3.7994	4.67719	4.81413	3.71611	4.9611	4.67841	5.07189	4.86487	4.34784	3.96119	4.2537	4.55053	4.35369
C8orf82	4.36155	4.79209	5.00807	3.93986	4.86444	4.92007	3.88534	4.89647	4.20455	4.65611	4.58966	3.24268	4.23273	3.97324	4.88785	4.67182
ARHGAP39	3.58409	3.86151	3.88118	2.80639	2.69284	2.97932	1.66357	3.27426	3.55407	3.65114	4.02552	3.14001	3.31317	3.6202	4.16534	3.96013
ZNF251	1.86845	1.69577	1.96955	1.71977	1.86545	2.04809	1.47731	2.17344	1.99658	2.45001	2.08784	2.73113	2.00977	1.78614	1.59722	1.53154
ZNF34	0.91179	0.70396	0.74199	1.17672	0.41279	0.33961	0.27327	0.18729	0.88424	0.83519	1.08835	0.66908	1.59835	1.69407	1.7376	1.73905
MIR6850	8.27868	8.50882	8.34735	6.98856	7.51793	7.18453	6.87869	6.38081	7.41086	7.99603	7.59882	4.86036	6.79106	6.54633	6.45224	7.13343
ZNF517	0.8797	1.29092	1.46868	0.84654	1.34158	1.47292	0.48193	1.96025	-0.15393	1.00249	1.01699	0.71247	0.26266	0.85769	0.96842	0.8691

ZNF7	1.13461	1.40419	1.34878	1.58154	1.35	1.08387	1.21629	1.46147	1.13938	1.55688	1.52606	1.4638	2.15458	2.20215	1.74237	2.09951
COMMD5	4.47489	4.76797	4.51864	4.54908	4.21621	4.69779	4.02178	4.42367	4.00245	4.43641	4.38802	3.89538	4.60909	5.32267	4.66539	4.90743
ZNF250	-0.05132	-0.346	0.10414	-0.35994	-0.57027	-0.18666	-0.45359	-0.48432	0.28198	-0.1625	0.4049	0.53616	1.22635	1.37589	0.9733	1.15103
ZNF16	0.18574	0.2198	0.11533	0.10145	0.32403	-0.18579	0.16824	0.25444	0.83847	0.96924	1.10918	1.29882	0.97766	1.1028	0.91289	0.96434
ZNF252P	0.29676	0.39932	0.45044	1.10861	1.32178	0.09668	1.86078	0.49757	1.20725	1.32496	1.24874	2.02899	2.37795	2.15637	2.13523	2.21578
TMED10P1	-0.80096	-3.32193	-0.94026	-0.86577	1.11322	-1.55806	1.13849	-0.31352	0.42177	0.02019	0.65352	1.00691	2.09671	1.61352	1.65742	1.46442
ZNF252P-AS1	-2.03561	-2.09173	-2.22663	-2.38962	-0.69084	-1.35478	-0.37402	-1.48705	-0.94851	-0.71594	-1.15894	-0.48623	-0.26396	-0.02919	-0.26403	0.01835
C8orf33	4.47416	4.02863	3.79706	4.09565	4.42358	3.41545	4.52924	3.52644	3.99186	3.8843	4.01063	3.54689	5.54984	5.64688	5.12114	5.10922
WASHC1	3.88321	3.67076	3.76424	3.34795	3.45669	4.36371	2.48846	4.60602	3.5428	4.23423	4.08362	3.63495	2.76328	3.01058	2.68417	2.60159
FOXD4	-1.1397	-1.85455	-1.23087	-0.42422	-2.90621	-2.83905	-2.52782	-2.36938	-0.1895	0.6236	0.16124	0.09638	-2.37777	-3.32193	-1.79741	-2.50033
CBWD1	2.64154	2.50485	2.34767	2.54401	1.06595	1.5644	1.16162	1.5367	1.68687	1.74794	1.55435	1.30924	2.19664	2.15329	1.4212	1.56539
DOCK8	-2.84255	-2.35996	-2.09904	-2.60847	-1.87739	-1.33631	-1.68858	-1.15763	-3.32193	-3.32193	-3.24014	-3.32193	-3.32193	-3.32193	-3.10622	-3.17151
KANK1	2.20279	2.89195	3.50663	2.28456	2.14255	1.93728	2.0346	1.99416	1.46816	0.96647	0.89179	1.03301	-0.2077	-0.38361	-0.15761	-0.07054
SMARCA2	2.59937	2.48055	2.39843	2.71482	0.14878	-0.85845	0.21861	-0.68673	2.20838	1.76568	1.95408	2.03154	1.21834	1.04594	1.56618	1.79936
VLDLR-AS1	-0.03292	0.40753	1.24804	-0.0019	-2.28302	-2.44462	-2.496	-2.33311	1.58773	1.64028	1.88144	2.11164	2.14795	2.77803	2.91246	2.78694
VLDLR	1.50293	2.08459	2.41094	1.96267	-1.7655	-0.43715	-1.39872	-0.79972	2.17458	2.08513	2.18889	2.882	3.13829	3.76145	3.57464	3.36376
PUM3	5.69391	5.33936	5.01198	5.44524	3.07325	2.60208	3.5925	2.65683	4.25266	3.81938	4.05689	4.02673	4.64831	4.37093	3.61516	3.30635
RFX3	-1.07327	-0.51342	-0.67374	-0.46827	-1.65113	-1.97388	-0.75398	-2.32785	-0.94339	-0.4411	-0.74491	0.36998	-0.06688	-0.4991	0.65338	0.41594
GLIS3	1.32422	1.22091	1.28623	1.23566	0.03424	-1.30215	0.10811	-1.28107	1.89896	1.46874	1.53774	1.9206	1.83464	2.09335	2.7763	2.4679
GLIS3-AS1	0.66024	1.08242	0.681	0.22389	-0.00267	-2.27293	0.3611	-1.50827	1.60869	0.8745	1.85009	0.68131	1.96738	1.45491	2.93952	2.14855
SLC1A1	1.00177	1.14635	1.10224	1.04302	-0.39674	-1.66244	-0.07147	-2.21459	-3.32193	-3.32193	-3.32193	-3.07822	1.43331	1.25598	2.37251	1.29224
SPATA6L	-0.11652	0.34664	0.08656	0.55676	-0.63973	-0.81335	-0.80487	-0.41097	-2.09043	-1.49744	-1.46537	-0.69022	-0.7677	0.08814	-0.65852	-0.08358
PLPP6	1.70644	2.04412	2.03484	2.43959	1.50178	1.42519	1.43747	1.05342	1.24341	1.63215	1.7084	1.98139	1.36648	2.32135	2.04516	2.76318
CDC37L1	2.81511	2.78077	2.59949	3.10186	2.22728	2.22967	2.61722	1.99293	2.27848	2.21675	2.20786	2.53636	3.07551	3.33235	2.65786	2.54463
AK3	4.28339	4.37545	4.2503	4.72203	2.52093	2.12218	3.10709	2.29215	3.73564	3.55901	3.58815	3.85406	4.52492	4.18772	4.44425	4.15563
RCL1	3.47394	2.98031	2.74628	2.9964	0.78139	0.90198	1.27521	0.66836	1.82614	1.05026	1.78162	1.39566	2.75492	1.9183	1.65139	1.43589
HNRNPA1P41	1.98081	2.26109	2.21965	2.63775	-2.27538	-0.71938	-1.96503	-0.52108	-0.35756	-0.42434	-1.16309	-1.56553	-1.75359	-2.11669	-3.32193	-2.46104
JAK2	0.99959	1.37796	1.17688	2.07479	-0.58578	-0.54574	-0.081	-0.9054	1.16008	1.48798	1.54978	2.09548	1.55491	1.46011	1.55302	1.77161
INSL6	-2.56657	-3.32193	-3.32193	-3.32193	2.02651	2.8047	2.22033	2.32488	-3.32193	-3.32193	-3.32193	-3.32193	-3.32193	-3.32193	-3.32193	-3.32193
RLN2	-3.32193	-3.32193	-3.32193	-3.32193	-3.32193	-3.32193	-3.32193	-2.03231	-2.05183	-2.65689	-3.32193	-3.32193	2.23725	3.08596	0.92404	1.6698
PLGRKT	3.97225	4.48093	4.57355	4.90808	1.38784	2.01506	1.36442	1.70192	3.13768	3.23631	3.01259	2.42573	3.33893	3.56411	2.90595	2.69864
CD274	2.34166	2.41623	1.98995	1.68903	-2.40918	-1.7808	-2.16199	-1.52622	0.09255	-0.47508	-0.58298	-0.57104	2.60048	2.50835	0.98255	1.26581
PDCD1LG2	2.70029	2.80487	3.0126	2.72339	-3.32193	-3.32193	-3.32193	-3.32193	2.06403	1.96252	2.72842	2.60982	2.3901	1.03349	0.81606	1.24718
RIC1	1.98462	1.99407	2.09563	2.46989	1.36013	0.50113	2.02569	0.16453	1.8255	1.6267	1.94593	2.36299	3.00865	2.58055	3.02176	2.67488
ERMP1	3.67243	3.54962	3.75587	3.81026	3.88887	3.0632	4.52114	2.99832	2.43842	2.18532	2.07928	2.63169	3.56056	4.07488	3.92787	5.20154
KIAA2026	1.87669	2.3593	2.36934	3.18893	0.16676	0.90404	0.80276	1.3275	1.206	1.3895	1.16305	2.59408	1.52003	2.02064	1.8571	2.38913
MIR4665	3.31097	3.73685	2.82445	3.64411	2.5708	2.81827	2.89459	2.57109	3.52599	3.25906	3.62209	3.08441	3.54163	3.71564	4.6682	5.11328
RANBP6	3.49747	3.71372	3.54304	3.91721	1.62517	1.26994	2.35807	1.44282	2.61896	2.45223	2.41831	2.78469	3.31962	3.36908	3.30951	3.69385
UHRF2	2.44679	2.29653	2.55464	2.68297	0.87291	0.14369	1.12054	0.56304	1.71112	1.93918	1.87442	2.39986	2.81289	2.82159	2.63133	2.58554
GLDC	0.79269	0.5406	0.20063	0.47857	3.68384	2.46372	3.50862	3.12482	-0.58824	-0.21442	-1.23855	-0.77196	3.48467	3.49353	3.16641	3.91294
RNF2P1	1.12087	1.3792	0.798	1.27988	1.53937	1.13603	1.02622	1.97773	-0.00985	0.18574	-1.56143	-0.27147	2.44835	2.18239	0.43	0.60861
KDM4C	1.65648	1.75454	1.80473	1.89012	0.53354	0.06019	0.86902	0.18564	1.15855	1.43442	1.5185	1.63256	1.17362	1.01797	0.87179	0.832
RPL4P5	2.2209	1.60198	2.19148	-0.08217	0.5801	1.63849	-0.16026	1.58198	1.94377	3.14393	2.17336	1.9441	0.2582	-1.78624	0.40001	0.06998
DMAC1	4.68727	4.41653	4.40301	4.15655	1.52046	2.23378	1.33049	2.07837	3.15068	3.05875	3.19089	2.2876	4.35395	3.4838	3.11619	3.03189
PTPRD	-2.96798	-3.21354	-2.91113	-2.55467	-3.27995	-3.22394	-3.32193	-3.20732	1.88265	0.91307	1.33731	2.38778	2.78015	2.39374	2.34415	2.57884
PTPRD-AS1	-3.32193	-3.32193	-3.32193	-3.32193	-3.32193	-3.32193	-3.32193	-3.32193	-1.00609	-1.83624	-0.85901	-1.34003	0.03764	-0.00167	-0.14847	-0.56277
RPS26P3	2.56038	1.51276	2.27976	-1.39008	1.06288	3.69522	1.68826	2.96213	1.42885	2.7119	2.37374	1.39895	1.01575	1.34335	-1.67608	0.24878
RN7SL5P	4.55783	4.41058	5.14293	-0.49319	6.36732	8.03565	7.11084	7.83747	5.27061	5.75375	5.70592	5.0085	4.53662	4.28841	3.96036	4.05452
LURAP1L-AS1	1.92265	2.3548	1.9626	2.55173	-3.32193	-3.32193	-3.32193	-1.16293	-2.2317	-3.32193	-3.32193	-1.41022	-0.34943	-1.06148	-1.80853	-2.35999
LURAP1L	2.81079	3.11097	3.23506	4.30517	2.27692	2.9621	2.82781	2.5291	0.58145	1.37427	0.71912	1.72659	3.67385	3.47219	3.21884	3.51953

MPDZ	1.23769	1.06605	1.32549	1.44614	0.93354	0.10372	1.24401	0.14695	2.7009	2.41095	2.72502	3.01242	3.01964	2.67904	2.90211	3.02184
NFIB	1.42767	1.65556	1.44846	2.52335	-0.21897	0.06585	0.35669	0.64229	0.38009	0.7308	0.66007	1.52805	2.02595	2.57113	2.96299	2.48548
ZDHHC21	0.5364	0.60713	0.64452	1.27378	-0.16762	-1.07514	0.72468	-0.73826	-0.30716	-0.18753	-0.16284	0.72537	0.79781	-0.16715	0.76867	-0.3092
LDHAP4	3.90243	3.8495	4.06833	2.7604	0.78105	-0.6206	-0.27593	-0.41989	4.19134	4.20733	3.87595	3.42172	1.90901	0.32591	1.65269	1.57717
TTC39B	-0.89253	-0.72111	-0.95945	-0.28224	0.05979	-0.96752	0.23885	-0.61706	-0.42876	-0.35756	-0.86816	0.25201	0.01643	0.06752	-0.02006	0.55079
SNAPC3	2.9589	2.92237	3.02149	3.05972	2.04408	1.54481	2.17703	1.29153	3.06584	2.63283	2.23851	2.71887	3.38323	2.72823	2.83138	2.29411
PSIP1	4.04927	3.95363	4.03827	4.30551	2.50871	2.26872	2.71252	2.37506	4.25406	3.58478	3.8824	3.43549	4.06198	3.19243	4.28628	3.31763
FTH1P12	3.62697	3.00089	3.81942	1.72278	-1.32513	1.25015	-0.67594	-0.09283	-0.40564	2.19608	0.16534	0.97779	-0.19684	-3.32193	0.6593	-1.41537
CCDC171	-2.55428	-2.4832	-2.50915	-2.28767	-2.44159	-2.72113	-2.18952	-2.78016	-1.69971	-2.33256	-1.74433	-1.49706	-1.91537	-1.86519	-2.21191	-1.5435
BNC2	1.00322	0.82952	0.93524	1.13152	-3.26116	-3.24988	-3.32193	-3.32193	0.33322	0.15191	0.5135	0.45169	1.30854	1.12942	1.64935	1.85571
CNTLN	1.19006	0.87355	0.89674	1.46755	-0.12609	-0.64604	0.13131	-0.21883	0.83821	0.28229	0.30292	1.12529	1.85048	1.64535	1.5021	1.40037
ADAMTSL1	2.05035	1.78124	2.20557	1.81105	-3.32193	-3.32193	-3.32193	-3.32193	-1.38027	-2.91167	-2.16052	-3.17425	-3.32193	-3.32193	-3.32193	-3.32193
RRAGA	6.41914	6.8896	7.12411	7.18726	4.55581	4.81	4.6196	4.38127	6.10306	6.14464	5.87512	6.2274	5.57482	5.81414	5.62482	5.81008
HAUS6	3.77205	3.79866	3.8715	3.81585	1.69116	0.46069	2.00328	0.74082	2.35491	2.10391	2.08602	2.51631	3.35852	2.88049	3.21025	2.74168
PLIN2	5.39821	6.34098	6.92007	6.92439	3.18722	3.7672	3.39013	3.27551	4.33731	5.40469	4.19341	5.9786	4.71204	4.40498	4.29448	4.32373
DENND4C	2.12434	2.67032	2.63189	3.40267	1.36958	0.2994	2.15088	0.33476	1.54921	2.19136	1.72225	3.37941	2.0127	1.96735	2.08814	2.58449
ACER2	-0.53628	-0.72258	-0.96902	-0.1019	0.17759	-1.04188	0.22434	-1.91598	1.37807	0.65206	0.40214	0.71823	2.20477	1.94972	2.28328	2.63818
MLLT3	0.65107	0.86814	1.06404	0.97687	-0.1281	-0.85281	0.25844	-1.61211	1.76855	1.54461	1.76701	2.50012	-3.32193	-3.21822	-3.32193	-3.32193
FOCAD	2.43019	2.44543	2.43178	2.86111	0.64298	0.23759	1.03494	0.0541	2.19424	1.87658	2.03479	2.62875	-3.32193	-3.32193	-3.32193	-3.32193
KLHL9	1.85073	2.31671	2.30657	2.67747	1.63311	1.00738	2.57879	0.79315	2.21237	2.99827	3.03252	3.56543	-3.32193	-3.32193	-3.1459	-3.32193
IFNWP19	1.64003	2.84394	2.16551	2.62262	-3.32193	-3.32193	-3.32193	-3.32193	-3.32193	-3.32193	-3.32193	-3.32193	-3.32193	-3.32193	-3.32193	-3.32193
MIR31HG	2.32087	3.60214	3.04366	3.19762	-3.32193	-3.32193	-3.32193	-3.32193	-3.32193	-3.32193	-3.32193	-3.32193	-3.32193	-3.32193	-3.32193	-3.32193
MTAP	3.17233	3.48118	3.27789	3.71044	0.42738	0.62679	1.06518	0.35725	-3.32193	-3.32193	-3.32193	-3.32193	-3.32193	-3.32193	-3.32193	-3.32193
CDKN2A	5.80645	5.96813	6.21183	5.73572	4.54701	5.07654	4.21013	4.54363	-3.04884	-3.09187	-2.86622	-2.72749	-3.32193	-3.32193	-3.32193	-3.32193
CDKN2B-AS1	-0.66035	-0.39634	-0.25164	-0.24797	-2.01024	-0.0123	-1.06723	-0.89705	-3.32193	-3.32193	-3.32193	-3.32193	-3.32193	-3.32193	-3.32193	-3.32193
CDKN2B	1.3921	2.48979	2.5329	3.9433	0.6208	1.52837	0.96841	0.67932	-3.32193	-3.32193	-3.32193	-3.32193	-3.32193	-3.32193	-3.32193	-3.32193
DMRTA1	1.1941	1.4478	1.03441	1.82713	-1.117	-0.2641	-0.60082	-0.45424	-3.32193	-3.32193	-3.32193	-3.32193	-3.32193	-3.32193	-3.32193	-3.32193
TUSC1	5.0095	5.34188	5.30332	4.42885	3.20288	3.10259	2.51438	3.19256	4.41511	4.59525	4.67849	2.99788	-3.32193	-3.32193	-3.32193	-3.32193
CAAP1	3.40684	3.37301	3.2287	3.77142	2.8628	2.99327	3.32237	2.79286	2.48732	2.38958	2.1358	2.60426	3.88028	4.05342	3.35114	3.46679
PLAA	4.64665	4.74914	4.74511	5.02456	3.59322	3.37327	4.0778	3.26146	3.71088	3.45796	3.58067	4.03438	3.586	3.74931	3.46396	3.44829
IFT74	2.86238	2.79571	3.14035	3.21889	2.3087	1.59727	2.76443	1.29869	3.07255	2.31126	2.8325	2.9563	2.42294	1.73422	2.86845	1.97538
LRRC19	-2.12638	-2.03018	-2.72781	-1.23183	-2.4564	-2.11741	-2.18152	-0.59352	-3.32193	-3.32193	-3.32193	-3.32193	-1.50342	-1.04062	-2.45697	-0.77922
MOB3B	0.21696	0.34224	0.06643	0.18507	-3.25634	-3.03333	-3.18151	-3.32193	0.53537	0.26096	0.23605	0.81647	-2.02413	-2.43059	-2.61941	-1.40407
C9orf72	2.16718	2.56558	2.15976	3.03802	-0.1531	0.72542	0.50039	0.61489	-0.52436	-0.33757	-0.89517	0.01477	0.89943	0.93282	-0.084	0.44562
SLC4A1APP1	0.92974	0.62952	0.22549	-0.76547	0.0438	1.74794	-0.08559	2.40971	-0.51576	0.28556	-1.4457	0.10803	-3.32193	-1.31499	-3.32193	-1.06662
ACO1	4.34025	4.18151	4.18472	4.40422	4.23865	3.91831	4.35998	3.92274	5.07576	4.71872	4.80589	5.15094	5.20287	4.38394	4.7913	4.498
DDX58	2.38817	2.97905	3.1483	3.80141	1.52178	1.28895	2.46612	0.77544	3.36986	3.409	3.38451	4.75805	3.68403	3.78469	3.7905	3.58927
TOPORS	3.2441	3.43762	3.36898	4.05077	2.96452	3.06561	3.40372	3.19465	2.54736	2.9202	2.82068	3.68542	3.2955	3.25732	3.37443	3.44048
SMIM27	1.11289	0.96868	1.17875	1.01857	1.11872	1.18429	0.86783	1.18419	0.50967	0.68936	0.72958	-0.09671	1.30659	0.95986	1.06347	1.13545
NDUFB6	5.81066	5.88803	5.88083	6.05887	5.20406	5.71603	5.45243	5.52742	5.28899	5.22961	5.09181	4.40942	5.44625	5.34832	4.55356	4.30733
ASS1P12	0.80289	0.03273	-0.23552	-0.28866	0.70598	-0.56579	0.35712	-0.36381	-3.32193	-2.63491	-2.59487	-2.1849	-1.52378	-0.16804	-1.54977	-0.75192
APTX	3.68266	3.41004	3.48416	3.72922	3.05826	3.05693	2.99594	3.09661	2.63114	2.57686	2.55379	2.53345	3.55266	3.70621	2.89029	3.00283
DNAJA1	7.37095	7.31411	7.08068	7.47459	6.60771	6.15349	7.13151	6.13194	6.05716	6.07355	6.23774	6.28169	6.86137	7.08992	7.07711	6.85674
SMU1	3.71158	3.63992	3.53164	3.77526	3.20656	2.99004	3.45201	2.95611	3.4368	3.25823	3.22954	3.40123	3.8835	3.87421	3.68552	3.67179
B4GALT1	4.97741	4.93466	5.18226	4.78806	5.10605	4.4544	5.40317	3.89404	4.9039	5.04074	5.86026	5.87992	3.97677	3.83586	5.19251	4.7683
B4GALT1-AS1	4.15192	4.48009	4.58418	2.79852	5.2175	2.74612	4.52103	1.55931	4.87356	5.15068	6.09568	4.09018	2.79745	2.79238	4.91886	4.56858
BAG1	4.02646	3.93877	3.47053	3.89669	2.92393	2.51725	2.62139	2.64622	2.89659	2.89185	2.70543	2.2288	1.95698	2.35121	1.59094	2.04858
CHMP5	5.94194	6.19982	6.07771	6.64805	4.49965	4.50602	4.65179	4.43903	4.63145	4.869	4.5093	4.60453	4.97583	4.92087	3.86888	3.86928
NFX1	2.97136	2.81462	2.74415	3.08107	2.51188	2.4338	2.78906	2.71896	2.47979	2.10053	2.41747	2.74392	2.90555	2.45275	3.01865	2.45786
AQP7	-3.12312	-2.9087	-2.88888	-3.32193	-0.21882	0.58901	-1.21426	0.52163	-3.32193	-3.32193	-3.32193	-3.32193	-2.89191	-3.32193	-3.13155	-3.32193

AQP3	-2.10448	-0.9531	-1.96007	-1.44341	2.20611	4.2797	2.4316	4.21775	-0.83096	0.40806	-0.50613	0.14422	-1.8014	0.51026	-1.22096	1.04677
NOL6	4.84142	4.45966	3.89479	3.81608	5.23072	4.82853	4.70762	4.87885	3.7963	3.646	4.09849	3.28058	3.84903	3.97581	4.02881	3.99321
SUGT1P1	0.64843	0.94867	-1.77019	0.41685	0.57043	0.50472	-0.21593	0.88394	1.88789	2.2681	1.86675	2.04224	3.23648	3.39742	2.05224	2.86001
ANKRD18B	2.30354	2.09237	2.0856	2.36042	1.94837	2.8156	2.34267	3.73591	1.53768	1.15959	1.82042	2.66524	2.61869	3.17096	2.91923	2.90875
SNX18P7	1.12288	0.68619	0.48873	0.41615	2.82939	0.27762	2.56524	1.27857	2.125	1.15152	1.76214	2.50122	3.28852	3.99444	3.84163	3.28705
CYP4F26P	0.6168	0.48797	0.5605	0.06749	2.44976	1.96195	1.74398	2.93558	0.94478	0.85711	1.32438	1.65141	3.02176	3.48331	3.35991	3.48856
ANXA2P2	4.78893	5.00569	5.34254	4.52716	3.75952	3.73687	3.88775	3.15036	6.13414	6.58495	6.06317	5.44135	3.19549	2.93426	3.49668	3.13548
PTENP1	-0.43257	-0.73361	-0.89648	-0.82148	-0.59746	-0.20779	-0.59505	-1.02116	-0.24297	0.47747	0.06441	1.17265	-0.66072	-0.03005	-0.06944	0.21587
PRSS3	2.64541	2.60123	3.00033	2.49436	-2.64903	-0.48791	-2.8005	-1.90818	-3.32193	-3.32193	-2.75323	-3.32193	-3.32193	-3.32193	-3.32193	-2.78075
UBE2R2-AS1	2.18219	2.59076	2.9587	2.88143	-0.97832	0.94056	-2.23805	1.06866	-3.32193	-3.32193	-2.15671	-3.32193	-3.32193	-2.36853	-2.22076	-2.20382
UBE2R2	4.85857	5.02934	5.10612	5.15138	5.10556	5.36908	5.31272	5.44608	5.16135	5.26415	5.29081	5.5246	4.44199	4.92745	5.31059	5.06286
UBAP2	3.06193	2.95171	2.79491	2.91732	3.24247	3.50506	3.24382	3.57023	2.94854	2.3008	2.59527	2.75509	3.24373	3.24993	3.62427	3.5506
DCAF12	3.37212	3.10648	2.96491	3.17786	3.87075	3.02886	3.77938	2.93924	3.69645	2.45607	2.68636	2.83914	4.17726	3.04069	3.76232	2.90383
UBAP1	4.46643	4.23735	4.3807	4.38914	4.6548	4.68478	4.57948	4.55527	4.2779	4.06215	4.00474	4.37873	4.20971	4.14346	4.10503	4.2339
KIF24	2.06656	1.49253	1.66651	1.20498	1.60957	1.11147	1.76417	1.49838	1.13055	0.3048	0.76544	0.3517	1.47903	1.63878	1.59109	1.96527
NUDT2	5.22875	4.84731	5.00788	4.81618	3.61019	4.12103	3.09153	4.31734	4.15437	4.31771	4.08771	3.96052	4.49174	4.00449	3.57967	3.31668
MYORG	1.03776	1.46918	1.01848	0.99921	5.45044	3.97842	5.12548	4.06114	1.59395	1.37383	1.29275	0.95604	2.22965	2.51965	3.51144	3.62393
FAM219A	4.2879	4.18985	4.33151	3.59938	3.21914	3.36864	2.67424	3.20995	3.90309	3.6561	3.89068	3.04122	2.43441	2.32713	2.74054	2.32953
ENHO	-0.9149	-0.42646	-1.18468	-1.83082	4.16117	3.58424	2.88717	3.82551	-0.54871	-1.4086	-1.33195	-0.9771	-1.62246	-2.23793	-2.56931	-0.32195
CNTFR	-2.87022	-2.82352	-3.32193	-3.32193	1.60195	-1.08398	0.13124	-0.58104	-3.32193	-3.32193	-3.32193	-3.32193	-3.32193	-2.42584	-3.32193	-3.32193
RPP25L	4.21561	3.93139	4.17273	4.04608	4.70372	5.40931	3.72638	5.17441	3.46007	4.18535	4.09659	2.86974	3.81515	3.52724	2.93209	2.82886
DCTN3	4.5281	4.62078	4.45979	4.38004	3.7384	4.23225	3.44486	4.17149	4.59244	4.34704	4.27675	3.6133	4.01022	4.11439	3.60714	3.64312
SIGMAR1	6.65269	6.12757	5.9431	5.53225	7.16407	7.52737	6.42305	7.43355	6.24435	5.53654	5.71706	4.62403	6.35953	6.15489	6.3816	6.41078
GALT	2.45655	2.35078	2.11618	2.37606	3.00643	4.16381	2.19263	4.24747	2.18335	2.61158	2.53863	2.11789	1.69239	2.27591	1.41823	2.06705
IL11RA	-1.16726	-1.53636	-0.64788	-0.42988	1.91889	2.67852	0.83129	2.67084	1.72708	1.77795	1.96445	0.89308	1.04587	1.31219	1.59203	1.4271
CCL27	-2.49821	-2.42336	-1.10508	-1.35255	0.35301	1.18888	0.32186	1.80259	-1.4403	-1.33283	-1.94605	-0.71089	-0.63736	-0.78377	-0.02599	-1.31994
DNAJB5	1.60935	2.24587	2.00075	2.1723	0.84879	2.07589	0.25641	1.72786	1.68198	1.86068	2.14615	1.62527	0.71184	2.1869	1.40787	1.76185
FANCG	4.67395	4.23303	4.20109	3.76277	4.25055	4.42104	3.55172	4.80568	4.1435	3.33068	3.66077	2.676	3.77549	4.00689	3.53126	3.56255
PIGO	2.57712	2.54293	2.46153	2.47953	4.48745	4.1449	4.08858	4.04021	2.7986	2.63811	2.93975	2.55249	2.89817	2.76159	3.3113	3.26897
STOML2	7.10993	6.84664	6.79625	6.58602	7.4043	7.60747	7.22464	7.35416	6.40034	5.86293	6.43556	5.79457	6.48089	6.20825	6.32978	5.74561
FAM214B	2.83508	3.39578	3.54393	3.69564	2.48623	3.68599	2.62795	3.13036	2.40437	2.81724	2.84558	2.97673	3.42299	3.39863	3.42296	3.8991
UNC13B	1.395	1.00082	0.82209	1.10485	2.78197	2.81693	2.8195	2.59598	2.02961	1.02439	1.83551	1.73104	1.83805	1.01976	2.39326	1.87331
RUSC2	5.20281	5.12524	4.76498	3.91665	3.40443	3.78371	2.72303	3.7639	4.36882	3.68371	4.17896	3.19398	3.54711	2.96322	3.56047	3.34348
FAM166B	1.34054	1.5009	1.25012	0.61188	-0.84561	1.07164	-2.71025	0.83356	-0.67571	-1.65201	-0.97084	-1.93854	0.51553	-0.39139	-0.41868	-0.29046
TESK1	3.53408	3.99293	4.1152	3.6997	4.64999	5.65398	3.76655	5.73633	3.53464	3.4465	3.44945	2.74492	2.5168	2.61918	3.3615	3.43319
CD72	1.75607	2.02219	2.08909	1.88287	2.06312	3.8174	1.4833	3.81618	1.1231	0.77697	0.56288	0.65773	0.38199	0.27428	0.70655	0.95173
RMRP	-0.04574	0.10452	0.55814	2.48125	2.32761	2.34417	3.7956	3.12288	0.53253	1.78705	1.63229	1.01561	4.72344	4.05338	3.98733	3.23665
CCDC107	4.74724	4.68114	4.88417	4.51931	5.49735	6.31944	5.11519	6.11517	4.22107	4.01854	3.95185	3.1332	4.65229	4.03737	3.8804	3.51307
ARHGEF39	3.32776	3.51664	3.89917	2.88459	4.11748	4.76785	3.76613	4.66162	2.68853	2.90623	2.36105	1.78294	2.82429	2.59928	2.24223	1.93771
CA9	4.63908	5.86706	6.54063	3.77729	0.89272	5.86706	-0.68833	5.08447	3.11022	4.85603	1.83447	3.19784	-2.53267	-3.32193	-3.32193	-2.39058
TPM2	2.65407	2.70365	3.15263	2.8467	7.00566	7.39197	6.66192	7.24894	6.98493	6.74655	6.91969	6.37792	0.42977	1.47497	0.69575	0.65853
MIR6852	7.71832	8.10137	8.43878	7.80903	8.70605	7.5903	8.96129	6.89467	9.43692	8.85763	9.41939	9.17661	8.24848	7.82554	9.28099	8.83913
CREB3	5.48278	5.49353	5.22688	5.57821	6.689	7.26361	6.47746	6.99253	5.26496	5.22885	5.31216	5.20281	5.59439	5.41612	5.68429	5.52571
GBA2	3.58995	3.34826	3.45095	3.73759	4.45126	5.17298	4.05977	5.07287	3.10891	3.35231	3.21049	3.31469	5.12624	5.37637	4.54248	5.57278
RGP1	3.12732	3.45719	3.2723	3.07789	4.56403	4.19253	4.45601	4.06779	3.02009	3.46852	3.4284	3.59373	4.10987	4.43909	4.44977	5.11473
MSMP	3.82806	4.09981	3.97781	4.24969	5.68154	4.80317	5.69839	4.48455	4.27483	4.45135	4.53534	4.98638	5.8043	6.17154	6.08716	7.14143
NPR2	-0.46892	-0.92075	-0.73795	-0.72023	-0.85725	1.03499	-1.54088	1.47251	-0.65099	-0.39903	-0.59583	-0.24739	0.94478	0.73775	1.01236	0.7547
SPAG8	0.05787	-0.70828	-0.4981	0.05083	-2.10033	-0.1871	-2.70623	0.43808	-1.91207	-2.45415	-0.96149	-1.78981	-0.60287	-0.11473	-1.92502	-1.4026
HINT2	4.17416	4.10697	4.21463	4.20697	3.61988	5.31746	2.62054	5.33275	3.19222	2.94948	2.71051	1.8365	3.52025	3.97114	3.09533	3.29867
TMEM8B	0.663	0.78835	1.06047	0.7281	0.41511	1.74746	-0.29549	1.53378	0.30616	0.46169	0.08124	-0.07519	0.43581	1.06271	0.94867	1.33368

HRCT1	1.5653	1.51142	2.18658	0.80748	0.00306	-0.11089	-0.21325	-0.54999	-1.05936	-0.45356	0.15781	-2.49453	2.11245	2.77825	2.96754	2.3569
YBX1P10	3.47055	3.90889	3.62759	3.09443	1.9203	2.34766	1.6874	1.95873	3.26757	3.83801	3.32128	3.05752	2.13849	1.78893	2.59168	2.82605
RECK	0.74778	0.35803	0.71042	0.91294	0.86638	0.98716	1.1039	0.67496	1.56091	0.88415	1.32618	1.68521	2.55917	1.78558	2.55724	1.4295
GLIPR2	1.42831	2.15793	2.79331	2.53022	0.02448	-0.07758	-0.34427	-0.70442	-1.85112	-1.34382	-1.95508	-1.77265	2.65302	2.99876	3.4422	2.58507
CLTA	7.62217	7.561	7.57008	7.56138	7.00609	7.08793	7.04567	6.85678	6.45087	6.18937	6.38712	6.08995	6.8583	6.69778	6.52026	6.242
GNE	2.61746	2.41058	2.29813	2.37805	3.12999	2.66928	3.39731	2.31353	2.85111	1.54345	2.54229	2.00331	3.23824	1.70653	3.75425	2.13772
RNF38	2.20209	2.08064	2.00893	2.34944	2.85539	1.80236	3.14116	2.01448	2.996	1.80812	2.27555	2.59873	3.67131	2.93939	3.96802	2.80942
MELK	4.79994	4.3872	4.58246	4.29187	5.15308	4.34584	5.28231	4.37805	4.57821	3.14601	3.79742	2.88621	5.52766	5.0914	4.91193	4.25103
EBLN3P	2.71998	2.78922	2.72156	3.16532	3.39059	2.45585	3.57702	2.63757	1.87823	2.09727	1.91493	2.35509	2.46493	1.89039	2.49951	1.61702
ZCCHC7	3.13276	3.24207	3.05308	3.25909	2.63273	2.81537	2.77249	2.93766	2.38677	2.43945	2.16692	2.50236	1.15859	1.12384	0.98169	0.24055
GRHPR	3.92205	3.80361	3.80294	3.37542	4.49811	4.40008	3.64408	4.49069	4.77498	4.6545	4.78802	3.77108	4.13423	3.83829	4.09525	3.82437
ZBTB5	1.14633	0.74972	1.00557	1.04914	3.35977	2.08918	3.67255	2.10756	2.32013	1.2015	2.33694	1.45398	3.06901	2.06536	3.24071	2.15514
POLR1E	4.51738	3.95394	3.80077	3.28879	4.47572	3.4841	4.54148	3.72457	4.47859	3.97377	4.06553	4.00303	4.44705	3.71681	4.00793	3.2717
FBXO10	1.85606	1.51598	1.953	1.77255	2.72896	2.50776	2.74068	2.45212	2.06754	1.17568	2.5322	1.51005	2.34721	1.5353	3.18708	2.02349
TOMM5	5.72576	5.53399	5.04676	5.05537	5.84988	6.18245	5.45412	6.29005	4.71522	4.50019	3.94395	3.03502	5.47198	5.4625	4.12815	4.36703
TRMT10B	1.77858	1.50363	1.48282	2.02347	1.56744	2.54163	1.73793	2.86448	0.79643	0.37986	0.78835	1.03638	2.04647	1.60333	1.20017	1.67442
EXOSC3	5.03938	4.99218	4.72965	4.69482	4.62064	4.49696	4.6658	4.60381	3.94011	3.55286	3.74969	3.01924	3.86411	3.88858	3.66388	3.25859
DCAF10	1.63122	2.04306	2.14413	2.4952	2.64259	2.40415	2.70197	2.59343	2.05592	2.45312	1.92673	3.22948	2.14324	2.35892	1.93582	2.4395
PAICSP1	-0.20876	1.12155	0.68802	1.18504	-0.31436	0.47466	-0.9526	0.47456	-2.01011	-1.29519	-0.83019	-0.93457	-1.15286	0.06473	-1.82285	-0.46925
SLC25A51	2.34503	2.73657	2.79662	2.80002	2.74796	2.65326	2.79615	2.38097	2.24714	2.24752	1.83008	2.58807	2.51158	2.7324	2.31794	2.71986
TMX2P1	2.6664	2.77972	2.83047	2.62971	3.28352	3.07256	3.41136	3.36037	2.80336	2.9068	2.66581	3.27213	3.16119	2.77829	3.34305	3.16655
SHB	5.4166	5.62186	5.90531	4.53574	6.82408	5.6343	6.05994	5.22688	5.46232	5.15807	5.08573	3.86407	5.21716	4.82235	5.65449	5.16978
ALDH1B1	4.24737	3.90784	3.75243	3.31388	5.15638	4.64798	4.73203	4.49453	3.02411	2.60355	3.00458	1.84722	4.57083	4.59924	5.6133	4.75032
IGFBPL1	-3.32193	-3.32193	-2.44124	-3.32193	0.76413	0.68499	1.15123	1.14499	-1.91553	-0.78759	-1.09604	-0.80791	0.21035	2.95412	3.85525	3.61194
FAM95C	-1.75056	-2.5873	-2.76884	-2.00372	-2.51152	-0.39989	-2.68763	-0.01271	-1.32931	-1.00201	-0.7606	0.37446	-2.93351	-2.26707	-2.10915	-1.3635
SNX18P3	-2.83897	-1.84421	-2.05101	-1.73325	-2.19625	-1.73215	-2.52095	-1.79003	-0.71697	-0.41159	0.00886	0.96121	-0.08769	0.58181	0.3948	0.42398
ANKRD18A	-0.74273	-1.59956	-1.9655	-0.98145	-2.07858	-0.20179	-2.07939	-0.09609	-0.63403	-0.19065	-0.17073	1.23122	-1.76161	-0.90468	-1.02547	-0.95964
FAM201A	-1.54752	-1.42767	-1.90341	-1.71905	-1.83684	-0.8131	-2.51187	-0.56511	-0.35835	0.87298	0.14167	0.1553	-2.35969	-1.87176	-1.51291	-1.57238
CNTNAP3	-3.20887	-3.32193	-3.32193	-3.32193	-1.16422	0.23841	-1.01254	0.43375	1.06335	1.87528	1.46027	3.03131	4.83771	4.5249	5.1883	5.25505
ZNF658B	-2.18774	-2.40038	-1.67875	-2.31784	-1.72261	-1.73042	-1.54931	-1.9115	-0.46388	0.82442	-0.47604	1.42577	1.46887	2.19947	1.0416	2.38168
GLIDR	-0.08373	0.12173	-0.79087	0.24869	-2.31291	-0.78065	-1.63244	-0.24872	-0.136	0.7734	0.12157	0.79431	0.63331	1.39725	0.53429	1.51404
FGF7P3	2.31179	2.5897	2.47	2.26502	2.23137	1.62671	1.69627	1.56861	1.53655	1.68388	1.35007	-0.26406	3.0205	2.43935	1.88869	2.55288
ANKRD20A2	-2.48588	-2.79523	-3.32193	-3.00255	-2.99989	-2.47887	-3.32193	-1.92453	-1.41971	-1.3117	-1.53948	-1.02689	2.29667	1.73688	1.74883	1.74689
SNX18P8	-3.32193	-3.32193	-3.32193	-3.32193	-3.32193	-3.32193	-3.32193	-3.32193	-3.32193	-3.32193	-1.81612	-2.44079	1.78212	1.25556	0.70141	1.86907
FAM95B1	-3.1871	-3.17144	-3.16352	-3.32193	-3.15225	-2.94661	-3.32193	-3.16293	-2.01147	-1.53284	-2.54764	-2.37435	3.15916	2.21008	2.57629	2.78791
IGKV1OR9-2	-1.39521	1.38729	0.13203	0.21553	-3.32193	-3.32193	-3.32193	-3.32193	-3.32193	-3.32193	-3.32193	-1.4516	5.02623	4.35342	6.23401	5.55015
FRG1HP	3.27013	3.46219	3.35337	3.85967	1.42241	2.05812	1.26459	1.92398	2.06644	2.53168	1.88617	2.45151	3.10727	3.34935	2.59252	2.82758
PGM5P2	1.87461	2.34243	1.72433	2.00332	-0.77898	-0.44593	-0.54493	-0.02059	-0.62873	0.58356	-0.13268	0.32417	2.02199	2.63689	1.79418	2.11968
CBWD6	3.15211	2.97328	2.68783	2.78795	1.74999	2.48458	1.91859	2.40493	1.6094	1.90726	1.50517	1.13919	3.58212	3.53192	2.51018	2.86358
CNTNAP3B	-3.32193	-3.22986	-3.32193	-3.12252	1.65849	2.14833	1.68869	2.01125	-0.91618	-0.42396	-0.92762	0.89429	4.14544	3.93735	4.48525	4.70559
ATP5F1AP1	-3.32193	-3.32193	-3.32193	-3.32193	-2.3233	-3.32193	-3.32193	-1.69132	-3.32193	-3.32193	-3.32193	-2.24199	0.26865	0.02473	1.51026	0.6167
ANKRD20A7P	-3.32193	-3.32193	-3.32193	-3.32193	-1.09334	0.38156	-1.11988	1.13962	-2.86385	-2.37326	-3.32193	-1.67415	-0.18308	-1.14761	-1.66716	-0.97746
CNTNAP3C	-2.78673	-3.32193	-3.32193	-3.32193	0.38045	0.83207	0.08987	0.89756	2.62199	3.46697	3.12999	3.8948	5.96548	5.4385	6.35764	6.20685
FAM27C	1.2436	0.72733	0.52948	0.27903	2.72862	2.87993	1.99315	3.16054	3.508	3.29567	2.93927	1.92702	3.63672	3.46172	2.26045	2.25465
FGF7P6	-0.51535	0.20592	-0.30332	-0.53031	-1.21033	-1.1757	-1.2641	-0.74628	-1.31103	-0.45701	-0.98533	-1.63293	-0.15754	0.35659	-1.00505	-0.08994
LINC01410	-0.25005	-0.19396	0.13591	0.09426	-1.70769	0.00583	-1.78203	0.10078	0.26148	0.86746	0.36841	0.80476	0.37946	0.56176	0.52758	1.0141
RNA5SP283	3.64292	4.25799	3.34267	2.33344	1.37459	3.7321	0.3979	3.89132	4.6506	4.82788	4.64954	2.33522	3.13524	3.36214	4.90637	4.30963
BMS1P9	0.09041	1.30004	-0.69086	1.34233	-0.1809	-0.66198	-0.97341	-0.26855	-3.32193	-1.19013	-3.32193	-1.20907	-0.00991	-0.96929	0.03001	-2.42988
AQP7P1	-0.01269	-0.09911	0.41343	0.2929	-3.32193	-1.0726	-3.32193	-1.85374	-1.47801	-1.37156	-1.29422	-1.14549	0.19828	0.73587	0.12794	0.98871
BMS1P10	2.94909	2.76319	3.19072	2.94687	0.31938	0.58619	0.26259	0.6632	-0.99213	-0.51169	-0.4226	-0.2817	1.21775	1.24611	1.05993	0.89392

DUX4L50	-1.657	-3.32193	-1.4852	0.55613	-1.81399	1.26589	-0.9848	1.4234	0.30515	0.98849	1.00837	0.52381	0.05024	2.30159	1.9762	2.9889
FRG1JP	3.21258	2.81231	3.44165	3.93116	0.75978	1.19627	1.08624	1.62041	-1.03624	-0.02288	-0.50529	-0.16765	1.17327	0.2706	-0.60572	-0.76584
ANKRD20A4	-3.09285	-3.32193	-3.05424	-2.80139	-3.03592	-1.86778	-3.32193	-2.16611	-2.05877	-2.00375	-1.94095	-1.6065	1.9441	1.2854	1.28279	1.10354
SNX18P9	-3.32193	-3.32193	-3.32193	-3.32193	-3.32193	-3.32193	-3.32193	-3.32193	-3.32193	-3.32193	-3.32193	-1.85606	1.93121	1.27613	0.9735	2.24061
CYP4F25P	-3.32193	-3.32193	-3.32193	-3.32193	-3.32193	-3.32193	-3.32193	-1.84931	-1.16174	-0.28696	-1.9736	-0.93105	4.08218	3.23589	3.48736	3.72341
BMS1P11	1.91964	1.78521	1.43158	1.34233	0.32724	0.60283	0.31736	1.4916	-0.49083	-0.53123	-0.44234	0.4298	1.73514	1.01212	0.95757	0.55512
IGKV1OR-2	-3.32193	1.06429	-1.46311	-0.59974	-3.32193	-3.32193	-3.32193	-3.32193	-3.32193	-3.32193	-3.32193	-1.68865	4.08332	3.5482	5.45	4.9051
BMS1P12	1.56474	0.96409	0.94362	0.70183	1.07561	1.34783	1.17308	1.50886	-1.04889	-0.93228	-0.15653	0.19175	1.63892	0.9913	0.65022	0.57978
CBWD4P	3.49035	3.01431	2.91459	3.44229	2.72067	4.22262	2.86579	3.96374	3.01584	3.42308	2.28902	2.86574	4.05517	4.13605	3.24638	3.90848
CBWD5	2.72475	2.61966	2.26613	2.47673	1.84345	2.61658	1.97279	2.52394	1.87886	2.11975	1.63176	1.26215	3.45055	3.50664	2.54251	2.95467
BMS1P13	0.95728	0.56369	0.72984	0.51451	0.79809	0.83568	0.98753	1.1284	-1.30113	-0.71929	-0.63242	-0.95544	1.36365	0.9623	0.43929	0.46854
SNX18P4	-3.32193	-3.32193	-3.32193	-3.32193	-3.32193	-3.32193	-3.32193	-3.32193	-3.32193	-3.32193	-2.35149	-2.41274	1.7192	1.54581	0.68098	1.74575
ANKRD20A3	-2.63758	-3.02669	-3.32193	-2.99376	-3.14702	-2.82712	-3.32193	-2.44044	-2.05227	-2.41276	-2.61813	-1.64127	1.07932	0.58284	0.41823	0.10747
ZNF658	-1.19582	-1.6627	-0.62018	-1.10733	-1.50706	-1.47694	-1.49695	-1.88071	0.07039	0.81025	-0.26801	1.60032	2.53604	3.13661	2.43135	3.2643
CNTNAP3P2	-3.32193	-3.32193	-3.32193	-3.32193	0.74058	0.94798	0.49922	0.97446	0.23241	0.67135	-0.06827	1.26651	5.23794	4.82745	5.71144	5.76549
FAM27E3	-0.89302	-1.9211	-1.36363	-1.53107	-0.07648	0.41306	-0.85001	0.51886	0.70402	0.1809	0.73519	-0.6121	1.80614	1.4256	1.22205	0.69727
FAM27B	2.65407	1.91869	1.64237	0.02854	3.62356	3.99473	2.85651	4.22773	4.64896	4.35609	4.02956	3.06871	5.23959	4.7462	3.24686	3.76682
ANKRD20A1	-3.05929	-2.78764	-3.32193	-2.99762	-2.90055	-2.46791	-3.32193	-1.78591	-1.4929	-1.36285	-1.52195	-0.80285	2.53065	1.79361	1.78078	1.46961
CBWD3	2.98409	2.84651	2.54407	2.68144	1.66405	2.3596	1.72211	2.33506	1.47141	1.77789	1.38916	0.95127	3.47912	3.39663	2.39862	2.73687
FAM122A	1.99453	1.9237	1.59179	1.80062	1.39446	1.51678	1.3076	1.60688	1.69389	1.3152	1.56782	1.55113	1.90876	2.10368	2.08623	1.90384
FXN	2.98008	2.40245	2.28887	2.29294	2.14338	1.45592	1.99809	1.36813	2.75064	1.57411	2.63239	1.4079	2.91211	1.50679	2.03873	1.42063
TJP2	4.52457	4.20087	3.98172	3.84929	3.96824	2.1748	3.85868	2.66772	3.6097	3.43441	2.67529	3.17699	4.25762	4.08182	3.7607	4.37861
APBA1	-3.13752	-3.11648	-3.32193	-3.20282	-1.88452	-3.05075	-1.84587	-2.91663	-3.10968	-3.06223	-3.13131	-2.9902	0.79886	1.23343	2.05718	1.15639
PTAR1	1.1454	1.0894	1.02641	1.54541	2.32303	0.84128	2.94058	0.76261	1.87057	1.49132	1.71795	2.53883	4.25953	3.49934	4.31209	3.65073
MAMDC2-AS1	-3.32193	-3.32193	-3.32193	-3.32193	-3.32193	-3.01309	-3.21966	-3.32193	-0.91611	-0.7718	-0.76328	-0.55232	-2.52296	-1.55811	-1.79665	-1.59773
MAMDC2	-3.32193	-2.57511	-2.5431	-2.72886	-3.32193	-3.05456	-3.07342	-3.32193	3.29142	3.13108	3.51185	4.00539	0.42906	1.88138	1.75213	1.34304
SMC5-AS1	-2.13243	-2.75714	-3.32193	-2.7	-2.53513	-3.32193	-2.83805	-2.31076	1.22218	1.29072	1.8455	1.59163	-0.09522	0.58467	0.57125	0.67787
RPL24P8	2.76835	2.53536	2.06132	2.33939	1.41846	2.784	1.65847	2.25522	3.01677	3.28265	2.91903	1.9356	1.01732	0.59074	0.25617	0.28511
SMC5	2.41969	2.36914	2.22825	2.52825	2.79468	1.23772	3.3516	1.411	2.78011	1.99651	2.47283	2.58439	4.25761	3.11633	3.93395	2.75308
KLF9	1.99435	1.93997	2.47372	2.26732	2.28226	1.33695	2.38508	1.00625	0.94891	0.54541	1.29956	1.22166	0.58322	-0.27601	0.36566	-0.01047
CEMIP2	2.44951	2.6365	2.22231	3.31376	6.03155	4.92775	6.78796	4.96319	4.28552	4.61883	4.46724	5.36388	4.85396	5.95513	5.56872	6.19088
ABHD17B	2.98004	3.01403	2.93566	3.49296	2.94851	2.08306	3.34868	1.89487	2.99933	2.80315	2.98154	3.16621	3.54713	3.40094	3.66918	3.64253
C9orf85	1.63789	1.98322	2.06564	2.02849	0.12992	0.79997	0.85156	0.6645	1.01642	2.01541	1.25073	2.0493	1.93702	1.84172	1.18617	1.46741
HSPB1P1	3.81208	3.97755	4.05503	2.23042	1.27361	3.91838	0.59929	2.79729	2.76458	2.99881	2.99126	1.42581	1.16197	0.3599	-3.32193	-0.53176
GDA	-1.41526	-2.49696	-2.4625	-2.32795	2.49949	1.08768	2.91682	0.83334	-3.32193	-3.32193	-3.32193	-3.32193	-3.32193	-3.32193	-3.19264	-3.32193
ZFAND5	5.12085	5.30693	5.34869	5.80341	4.80812	4.51499	5.29445	4.42624	5.38447	5.46217	5.70681	6.18648	5.44401	5.67501	5.30984	5.80277
C9orf40	3.92367	3.82153	4.13464	3.65111	2.64332	2.65647	2.62549	2.80789	3.38021	2.67166	2.95799	2.02995	4.15297	4.21006	3.87934	3.67736
CARNMT1	2.55862	2.57937	2.43974	3.07575	2.9937	2.48494	3.53843	2.57219	2.89499	3.17715	3.06769	3.62543	3.47249	3.43795	2.78643	3.01224
NMRK1	0.21944	0.56058	0.67697	1.36977	1.31989	2.26613	1.25507	1.7122	0.31465	1.5399	1.15176	1.6794	0.97972	1.59249	0.8221	1.53717
OSTF1	5.22064	5.34288	5.62398	5.42244	4.73551	4.16096	5.05636	3.48902	5.2961	4.35544	5.09742	4.32294	4.65933	2.5577	4.16711	2.33292
PCSK5	-3.32193	-3.32193	-2.95513	-3.21475	-0.34937	-3.23546	-0.29907	-3.22074	-3.22296	-3.32193	-3.32193	-3.24071	-3.25449	-3.32193	-3.24015	-3.23839
RFK	3.37984	3.92165	3.28228	3.8809	4.18592	4.04215	5.14179	3.96792	1.69657	2.53472	2.47355	2.83222	3.28003	4.71136	3.79894	3.95908
RPSAP9	2.87859	2.62811	3.01311	1.39928	2.99446	2.82398	3.14233	1.98052	4.53364	5.21659	4.12797	3.42028	0.25768	0.33329	-0.23274	0.27356
GCNT1	-3.01139	-3.32193	-3.32193	-3.32193	-3.1818	-3.15661	-2.78749	-2.80743	1.23956	0.20636	0.8111	0.1983	-1.89249	-2.11253	-1.06385	-2.45774
VPS13A-AS1	-0.86611	-0.09045	-0.30639	0.06199	2.98402	-0.22434	3.28268	0.22305	0.57586	0.62483	0.57941	1.32769	1.35629	1.89486	1.98365	2.63904
VPS13A	1.1313	1.31841	1.13376	1.77124	2.48464	2.35287	2.85925	2.07197	0.53505	0.42774	0.45413	1.65	2.17044	2.37599	2.01848	2.34809
GNAQ	2.59078	2.86556	2.76249	3.22533	2.90956	2.56082	3.27078	2.57932	4.01178	3.97398	3.79475	4.62813	4.24266	4.42661	4.4571	4.44604
CEP78	2.00324	1.67028	1.66656	1.67131	1.9152	0.92484	2.06622	1.41111	1.91701	0.90506	1.50815	1.15514	2.56246	1.85935	2.49803	2.02793
PSAT1	5.9216	5.36971	5.18121	5.0133	5.8079	6.31491	6.19329	6.28353	-3.32193	-2.89749	-2.87043	-2.90372	7.05637	6.83313	6.29278	6.22964
CHCHD2P9	3.04784	2.98777	3.48418	1.16905	2.12593	3.35799	2.79318	2.52815	2.54528	3.40654	2.22783	0.29802	1.15928	0.51466	0.48543	1.90845

TLE4	-0.8742	-1.01832	-0.89429	-0.49342	-0.30649	-0.35748	0.14897	-0.80195	2.93509	2.86858	3.59116	3.34048	4.32382	3.52499	4.57973	3.58348
TLE1	4.62088	4.68495	4.58586	4.54512	4.36826	3.6654	4.34139	3.61976	4.72923	4.50318	4.78486	4.15717	3.86305	4.54561	4.01665	4.15065
RASEF	0.74487	0.85056	0.41227	1.31451	-1.27816	-3.16098	-0.6827	-2.96841	-3.32193	-3.02901	-3.15724	-3.03349	0.41634	0.65643	0.92165	0.69154
FRMD3	0.41415	0.72942	0.48979	1.01326	-0.66994	-1.44942	-0.99393	-2.1323	-2.96784	-3.02236	-3.0024	-3.02694	-3.32193	-2.769	-3.02504	-3.16259
IDNK	1.16302	-0.0523	0.74101	1.0317	1.17782	2.05878	0.8618	2.16236	-0.32872	-0.31945	-0.22857	0.52455	2.82211	2.20444	2.99709	2.42245
UBQLN1	4.86225	4.88207	4.70709	4.99117	4.54335	3.58194	4.9978	3.41389	4.82226	4.39702	4.43737	4.87663	5.77574	5.8808	5.73504	5.65335
GKAP1	1.36692	1.25888	1.43871	1.90741	1.281	1.94858	1.59175	2.18601	-0.10912	0.27117	-0.29795	0.93071	1.84353	2.13775	1.31552	1.84583
KIF27	-0.03537	-0.02725	0.29513	0.84519	-0.41947	-0.52407	-0.26375	0.21365	-0.80118	-0.40502	-0.77371	0.51726	1.24565	0.93753	1.15005	1.44512
C9orf64	2.48256	2.75947	2.5346	2.72993	4.02227	3.18133	4.00014	3.18553	3.64726	3.42132	3.10182	3.24934	2.99368	4.11873	3.21798	3.61867
RMI1	2.28695	2.09526	2.14603	2.44997	2.15741	0.84844	2.46805	1.40777	2.45995	1.80452	1.53454	1.74496	3.67513	3.41354	2.87722	2.82726
AGTPBP1	3.42261	3.60874	3.72112	4.09933	1.20312	0.92247	1.85614	0.75591	2.70696	2.72592	2.32191	3.4364	3.46524	3.22676	3.38779	2.83256
NAA35	3.27366	3.21154	3.13126	3.48372	3.46297	3.02102	4.2114	2.82217	3.42015	3.47476	3.49487	4.12918	4.0163	4.10485	3.93187	3.98233
GOLM1	5.01821	4.75364	4.57675	4.79492	4.05991	4.16906	4.35277	3.73976	5.54957	5.13515	4.71032	5.30456	5.79074	5.89652	5.42352	6.08978
ISCA1	3.98046	3.80806	3.83204	4.17007	3.74552	3.96314	3.99496	3.69633	2.79786	2.43772	2.62273	2.98743	4.76656	4.30461	3.54019	3.71915
TUT7	1.90501	2.35796	2.4271	3.11444	2.28599	1.91153	3.08991	1.49835	1.73472	1.95294	1.86249	2.93289	2.27811	2.28648	2.37663	2.91274
CDC20P1	-0.47213	-0.32753	-0.25913	-1.46884	-1.29461	-1.96048	-2.75343	-1.81286	2.5835	1.19871	1.86301	1.29539	-2.17393	-2.83248	-2.74275	-2.31484
DAPK1	1.89381	1.74288	1.82699	2.14503	3.22353	2.62531	3.10428	2.64291	1.28067	0.74818	1.10371	1.25089	3.06704	3.53889	3.96875	3.73027
CTSL	7.50279	7.64853	7.75528	8.35914	6.63079	7.22382	6.68754	7.02912	5.44516	5.46064	5.58438	5.30719	5.69862	4.90487	4.26572	4.63487
SPATA31C1	-3.32193	-3.32193	-3.32193	-3.32193	-0.73088	-0.4338	-0.98717	-0.36967	-3.32193	-3.32193	-3.32193	-3.32193	-3.32193	-3.32193	-3.32193	-3.32193
CDK20	0.59398	0.93364	0.48592	0.48564	0.71083	1.25595	0.36636	0.78911	-0.04935	0.66881	0.77777	0.32912	0.96936	1.77478	1.41244	1.45679
RPS10P3	2.52506	2.43034	2.76569	1.05891	2.27426	2.82566	1.2015	2.46445	2.61503	4.10597	2.9541	2.09433	1.21133	1.60573	2.20164	2.92838
SPATA31C2	-3.32193	-3.32193	-3.32193	-3.32193	0.76303	0.21361	0.3665	0.71349	-3.32193	-3.32193	-3.32193	-3.32193	-3.32193	-3.32193	-3.32193	-3.32193
SPIN1	3.42986	3.68125	3.52298	3.86546	3.41024	2.67982	3.92428	2.7347	4.30196	4.35707	4.15407	4.89535	5.35387	5.72269	5.28725	5.21659
NXNL2	-1.49713	-0.73837	-1.45969	-0.51081	-0.41907	-1.24694	-0.46793	-1.06486	-0.78525	-0.10861	-0.60009	-1.31727	-1.86621	-1.74428	-1.54476	-1.18061
S1PR3	2.42632	2.54887	2.81402	2.83139	-2.51204	-2.70102	-2.32692	-3.17729	1.74695	1.09407	1.72081	1.28484	3.26644	2.16605	2.97958	2.57885
SHC3	1.81069	2.49245	2.2919	2.49463	-3.32193	-3.32193	-3.32193	-3.19367	-3.19646	-3.21709	-3.32193	-2.86722	2.22481	2.22543	2.24549	2.96427
CKS2	8.0006	7.71518	7.70146	7.04921	7.52525	7.60115	7.71258	7.53276	7.36181	6.62925	6.91765	4.53263	8.64933	8.33583	7.9884	7.46382
SECISBP2	2.45579	2.29615	2.39072	2.84177	1.54509	1.74272	1.84479	1.50939	1.48687	1.55441	1.57331	2.01305	2.54998	2.29199	2.66731	2.4191
SEMA4D	2.94631	2.94097	3.1152	2.61675	-0.74703	-1.28511	-0.8222	-1.32234	0.13782	0.0867	0.13663	0.36143	-0.06069	0.09355	0.41118	0.44038
GADD45G	1.14633	1.18556	0.82315	1.34808	-3.32193	-3.32193	-2.25428	-3.32193	-0.97302	-3.32193	-1.82392	-2.68358	-1.81617	-1.60319	-3.32193	-2.2204
LINC01508	-0.35775	-1.22673	-1.16665	-0.91741	-2.15946	-3.32193	-0.85298	-3.32193	-3.32193	-2.76745	-3.32193	-3.32193	4.1907	4.27027	3.36568	3.02899
DIRAS2	-3.32193	-3.32193	-3.32193	-3.32193	-3.22386	-3.09856	-3.32193	-3.32193	-3.32193	-3.32193	-3.32193	-3.32193	1.60666	2.95256	1.38642	1.49331
AUH	3.86839	3.66851	4.077	4.1149	2.52707	2.0466	2.79368	2.05219	2.18102	2.33302	2.48117	2.49553	2.37557	2.13525	3.24574	2.3114
NFIL3	4.68844	4.54152	5.11947	5.04208	3.5567	4.46218	3.85303	4.07725	4.51194	4.60674	4.10613	5.08047	4.44411	4.39583	4.24306	4.35861
ROR2	0.30323	0.96838	0.73519	1.32664	-2.96209	-2.97738	-3.32193	-3.10946	2.79776	2.39814	2.44941	2.20228	-3.17852	-3.32193	-3.14898	-2.84628
SPTLC1	4.18741	4.22855	4.19763	4.54363	4.56296	3.95643	4.88991	3.84634	4.27324	4.34586	4.29086	4.9351	4.91818	4.83734	4.58272	4.59574
NOL8	2.9104	2.79144	2.83638	3.41862	2.3666	2.2078	2.98291	2.43747	1.98674	1.97703	2.12155	2.55835	2.97848	2.53248	3.02488	2.57006
CENPP	2.59724	2.57996	2.35691	2.69654	1.02039	1.44784	1.12338	1.76106	1.19675	0.55369	0.51591	0.65299	1.62	1.83359	1.10457	1.10904
IPPK	3.77889	3.7981	3.56648	3.79184	2.07788	2.41699	2.15749	2.65958	2.15889	1.61136	1.40731	1.7491	2.92998	3.00084	2.2296	2.56212
BICD2	3.93238	3.81221	3.83571	3.80296	3.59402	1.93966	3.63857	2.06816	4.19088	3.71101	4.08222	3.83285	4.67253	4.08126	4.99698	4.42789
ANKRD19P	-0.80076	-1.57142	-1.79985	-0.45071	-0.37501	0.22046	-0.94922	0.56391	-1.81771	-1.3049	-1.22638	-0.19437	-1.80747	-1.44511	-2.14738	-1.48599
EEF1DP2	-0.42572	-1.28628	-1.22695	0.78839	0.0544	0.90198	-1.48271	1.2628	-0.65501	-0.47102	-0.06134	0.81418	-1.36398	-1.12233	-3.32193	-1.83811
ZNF484	0.2366	0.15795	-0.08316	0.66236	0.6525	0.17856	0.32219	-0.43203	0.25895	0.52284	0.18546	1.2847	1.05381	1.1304	1.63057	1.61762
SUSD3	1.54929	1.1623	0.99483	1.36872	-2.71599	-3.32193	-3.32193	-3.32193	-3.32193	-3.32193	-3.32193	-3.32193	-3.32193	-3.32193	-3.32193	-2.18485
CARD19	4.5187	3.91506	5.02283	3.48931	3.27269	2.53734	2.68375	2.09245	3.89594	2.62023	4.07944	1.6908	4.22422	2.72872	3.74157	2.29812
NINJ1	5.63495	5.98703	6.17574	5.74822	4.47782	4.90694	3.50392	4.82287	3.31901	4.36195	3.54827	4.07269	3.59254	4.18573	3.99699	4.50253
WNK2	-1.50281	-1.74656	-1.51125	-2.0526	0.95097	0.08033	0.19276	0.156	-1.20114	-1.88833	-1.18067	-2.44519	0.22506	0.28157	1.72258	1.61137
C9orf129	1.58109	0.93485	0.8898	0.26752	1.44776	0.67445	1.19991	1.07028	1.75844	1.46415	1.56391	2.37867	1.17649	0.55094	1.41601	1.7577
FAM120AOS	4.4194	4.32004	4.31576	3.80918	4.76885	3.69565	4.49525	3.69651	4.33945	4.08174	4.27113	3.7718	4.35746	4.16316	4.59943	4.36019
FAM120A	5.15244	4.99887	4.89605	5.01857	5.05765	3.83396	5.06554	3.79576	5.098	4.62405	4.81832	5.00579	5.38146	4.99467	5.2141	5.02574

PHF2	2.45404	2.2275	2.49765	2.29883	2.54775	2.24791	2.32318	2.14071	3.22742	2.971	3.06815	3.09239	3.58899	3.7424	4.02266	3.85145
PTPDC1	1.23374	1.02626	1.44584	1.38857	0.73052	-0.18522	1.046	0.28782	0.50883	-0.71919	0.2817	-0.24727	2.11372	1.3207	1.37873	0.90109
ZNF169	-0.63653	-0.36648	-0.32954	-0.24912	-0.84889	-0.9555	-1.31647	-0.89811	-0.83446	-0.55535	-0.66119	-0.95017	-0.42633	-0.52282	-1.10633	-0.54066
MFSD14B	5.56464	5.57042	5.45885	5.8075	5.18658	4.34271	5.45718	4.22765	4.98429	4.70851	4.81513	4.94651	5.77848	5.89317	5.28813	5.36208
PCAT7	-3.32193	-3.32193	-3.32193	-3.32193	0.91383	1.3374	0.55509	0.53427	1.56929	2.05335	1.55188	1.83856	1.02344	2.00137	1.76991	1.74562
FBP2	-3.32193	-3.32193	-3.32193	-3.32193	0.08382	0.08692	0.13653	0.02799	0.09464	1.11409	0.3455	0.22801	-0.16476	0.62221	0.60364	1.09174
FANCC	1.0411	0.48518	0.64225	0.42333	2.43869	1.58382	2.56673	1.56257	1.06015	0.3298	0.84122	0.12065	2.63892	2.49595	2.7582	2.27978
PTCH1	-0.66976	-0.64203	-0.79431	-1.10117	0.36299	-0.56271	0.079	-0.04694	0.76436	0.0416	0.0768	-0.0853	0.65712	0.98066	0.9123	1.22458
LINC00476	-0.84544	-0.26106	-0.41687	0.00422	-0.2795	-0.64717	-0.70984	-0.76436	-1.19731	-1.01096	-0.88519	-0.75998	0.12373	0.36511	0.52277	-0.07934
ERCC6L2	0.09377	0.38222	0.42601	0.8203	0.67783	-1.0169	0.91958	-0.87337	0.43818	0.45672	0.36548	1.19477	1.66267	1.19096	1.67135	1.26583
EIF4BP3	2.63809	2.5587	2.31348	1.96834	1.15074	0.70223	0.92704	1.10155	3.60803	3.63676	3.69161	4.40994	1.20293	0.797	1.07225	1.15632
SLC35D2	4.20318	4.42576	4.47115	4.46695	6.29495	5.7132	6.4168	5.48577	4.55854	4.42872	4.85641	4.80326	5.62061	5.14619	6.01527	5.60318
ZNF367	2.9957	2.60811	2.45861	1.91898	3.43472	1.95902	3.72836	2.27341	3.68842	2.14769	2.95457	1.10993	5.06048	5.67287	5.14908	4.60785
HABP4	3.46286	3.11237	3.24569	3.06791	4.4691	4.49334	4.33842	4.60114	3.20115	2.62111	3.04736	2.09498	5.0374	4.9475	5.38005	4.41492
CDC14B	2.20954	1.91214	1.93932	2.20893	2.13461	2.14171	2.49741	2.30573	1.59194	1.31159	1.52707	1.70384	3.25989	3.00921	3.29329	2.90385
PRXL2C	3.1346	3.00163	3.36828	3.31909	2.27666	2.41727	2.724	2.27184	3.06161	2.56662	2.86699	3.53595	4.26041	4.03441	4.26136	3.6441
ZNF510	0.50985	0.49509	0.85457	0.89067	-0.2131	-0.31758	0.14184	-0.29129	1.00325	0.61194	0.89938	1.4772	1.91306	2.15343	2.37323	1.84995
ZNF782	-0.03922	-0.47001	-0.12714	0.38768	0.34435	-0.49167	-0.03245	0.21231	0.05224	-0.73225	-0.26777	-0.35015	0.25324	0.1834	0.38118	0.11529
MFSD14C	2.80232	3.06465	2.93843	2.85496	1.97355	1.07196	1.80138	1.73501	1.60809	1.40782	1.44773	1.36064	2.86089	2.67928	2.4584	2.75372
NUTM2G	-0.44254	-0.60482	-0.64064	-0.27329	0.51086	0.40652	0.52147	0.99326	-2.21437	-1.98117	-2.30102	-2.51172	-0.53213	0.04359	0.19333	0.14835
CTSV	-1.82805	-1.71843	-2.2017	-2.44705	3.9556	3.78038	4.2074	3.47235	1.69949	0.94951	1.48689	0.41868	2.96778	2.48323	2.62893	2.04567
FAM220CP	-1.0783	-2.28751	-2.24709	-2.19824	0.53469	1.88297	-0.68251	1.91297	-3.32193	-3.32193	-3.32193	-3.32193	-3.32193	-2.02435	-1.84508	-1.10604
ANKRD18CP	-3.32193	-1.4616	-2.16216	-0.83574	-0.91217	-0.30226	-0.8316	-0.24561	-3.32193	-2.73477	-2.97732	-3.32193	-1.9256	-1.37843	-2.51789	-1.84731
ZNF322P1	1.10719	1.22259	1.46565	1.74054	2.48731	1.16472	3.0443	0.02326	2.65381	3.12177	2.85115	3.38483	2.46769	2.96669	3.2445	3.22829
SUGT1P4-STRA6	0.52819	0.12244	0.52766	0.61311	1.36011	1.54595	1.14671	1.38913	0.31689	-0.42063	-0.29177	-0.25918	0.96825	0.56083	-0.28493	0.5162
STRA6LP	0.00757	-0.48987	0.04312	-0.08944	0.67462	0.94075	0.28674	0.64699	0.29666	-0.13633	0.2026	0.1548	0.93467	0.1766	-0.03696	0.46175
SUGT1P4	3.22761	3.8482	4.16517	3.1496	4.45896	4.62618	4.16521	4.4689	2.90796	2.766	2.2928	0.26081	4.03872	3.8193	2.86569	3.43817
CCDC180	-1.57445	-2.2047	-1.73039	-1.90984	-1.36307	-1.19045	-1.6904	-1.27567	-1.01409	-1.3555	-0.88086	-0.95503	-0.47682	-1.10836	-1.14964	-0.79533
TDRD7	2.60042	2.94148	3.15226	3.41227	2.18535	1.18888	2.68685	1.75249	2.78821	2.59922	2.46846	3.48381	3.08553	3.07084	2.80641	3.06007
TMOD1	1.99754	1.99994	2.37392	2.83896	1.03337	1.73894	1.38978	1.67222	0.90769	0.43444	0.55192	2.11318	1.46282	2.31774	1.4843	1.74317
TSTD2	1.82161	2.05754	2.13367	2.45154	1.82803	1.47754	1.84883	1.42056	1.53341	1.25112	1.43307	2.17258	2.18924	2.84495	2.27948	2.64965
NCBP1	3.79196	3.58518	3.3957	3.55093	3.54371	2.47131	3.81477	2.40363	3.67066	2.895	3.3279	3.80854	4.41916	4.22646	4.02689	4.08867
XPA	3.80175	3.59409	3.72165	3.93865	2.8842	3.03407	2.96478	2.93626	2.87475	3.13697	2.89242	2.80818	2.8209	2.6203	2.97485	2.75541
FOXE1	-3.32193	-3.32193	-3.32193	-3.32193	3.21831	2.7723	2.84679	2.8939	3.86416	3.48692	3.95396	3.44012	-3.08849	-2.88896	-2.13022	-1.98199
TRMO	1.53025	1.43909	1.68101	1.67826	1.03207	0.92018	1.51495	0.63189	0.38071	0.08618	0.50582	0.22384	1.64985	1.96554	1.55507	1.77219
ANP32B	7.7754	7.48863	7.34717	7.53004	6.47241	6.41761	6.57141	6.45405	7.43192	6.6587	7.07449	6.53057	7.69346	7.59291	7.54436	7.26134
NANS	4.7303	4.68623	4.46273	4.60794	3.70247	4.28913	3.53914	3.94876	3.73695	3.30186	3.76129	3.1975	3.73532	3.80454	4.09948	3.86591
TRIM14	4.05843	3.88616	3.69056	3.95318	3.33009	3.52444	3.27066	3.35439	4.00607	3.62457	4.36309	4.03135	3.88297	3.78467	4.58092	4.03684
CORO2A	1.15794	1.83143	2.25223	2.32728	2.32049	1.27482	1.54261	0.99219	0.1708	-0.05289	0.38897	0.17578	-0.66251	-1.6324	-0.23148	-0.60732
TBC1D2	4.45017	4.55199	4.44672	4.24981	1.61542	1.97718	1.37824	1.67112	4.81815	4.70171	4.84365	3.19134	1.42053	-0.33772	0.17204	0.34802
GABBR2	-3.32193	-3.166	-3.15781	-3.32193	-2.67903	-3.32193	-2.76158	-3.00937	-0.50873	-2.01687	-0.85467	-1.63608	-2.92284	-3.10933	-3.0659	-3.32193
ANKS6	-3.32193	-3.18642	-3.17926	-3.17038	3.13885	2.26083	2.71946	2.20745	2.46609	2.33368	2.38325	2.32642	2.4365	2.29917	3.00739	2.83723
GALNT12	-0.47689	-0.09993	0.31398	-0.46463	2.62606	2.76106	2.41748	2.42423	1.54511	1.71064	1.74631	2.06386	-1.58817	-1.86869	-0.03802	-0.32163
COL15A1	-1.48743	-1.88781	-1.35759	-0.6662	-3.32193	-2.45352	-3.17281	-3.32193	-0.98053	-2.02186	-1.95957	-1.07094	-3.19576	-3.32193	-3.32193	-3.32193
TGFBR1	3.1538	3.12594	3.55436	3.69231	3.02558	1.79532	3.66997	1.78453	3.8012	3.31509	3.52149	4.03636	3.54329	2.65867	4.08387	3.28047
ALG2	4.55878	4.48801	4.26849	4.38482	4.51495	4.68199	4.29828	4.27184	3.15389	3.09678	3.18624	3.30986	4.31708	3.57295	3.70235	3.3902
SEC61B	7.07865	6.98659	7.05193	6.82622	6.84823	7.94212	6.77399	7.76049	6.23027	6.18343	6.29042	5.25265	6.79279	6.28158	5.83639	5.64748
NR4A3	-1.8141	-2.45182	-2.65972	-2.26103	1.14345	0.77877	1.6563	0.83296	-2.42875	-3.32193	-2.61115	-2.65959	-1.51301	-2.58062	-2.55287	-2.87847
STX17	1.3814	0.88434	1.31383	0.98623	2.03267	0.69873	2.54765	0.17911	1.42674	1.06312	1.59336	1.9356	2.8234	2.08453	3.33254	2.54901
ERP44	4.03783	4.02253	4.19111	4.44484	4.03303	4.78907	4.55016	4.67394	3.7529	3.64344	3.87866	4.35879	3.59099	3.55251	3.41673	3.38043

INVS	1.36078	1.3658	1.71685	1.39992	1.62996	0.76357	1.48235	0.76545	1.81992	1.42985	1.61485	2.2229	2.71952	2.54335	2.86924	2.71398
TEX10	3.45276	3.52925	3.11865	3.36285	2.70063	2.28913	3.09439	2.33864	2.75159	2.62791	2.64538	3.29388	3.72029	3.6244	3.56262	3.67391
MSANTD3	5.2735	5.11792	5.30181	5.06618	3.62177	3.38525	4.01807	3.09224	5.19906	4.77537	4.83907	4.81949	5.766	5.42479	5.33207	4.98723
MSANTD3-TMEF	4.6269	5.04916	5.07267	5.34636	2.77714	2.52385	3.2614	2.28511	4.29527	4.31108	4.0911	3.84041	4.89778	5.62852	5.27989	5.43126
TMEFF1	3.09957	4.39133	4.2245	4.56701	-0.06122	0.95107	1.17902	0.981	1.58607	3.10654	2.45649	2.83689	2.24312	4.7028	4.08387	4.62804
CAVIN4	0.81726	1.02299	0.80951	1.40479	-2.40939	-2.18421	-0.80851	-1.52654	-1.16818	-0.24448	-1.09248	-0.72154	-0.32635	1.18769	0.72989	1.37342
BAAT	2.36128	2.16847	2.26116	2.34273	0.56669	-1.69716	0.4674	-1.6533	-3.32193	-2.58857	-3.0148	-3.03848	-2.54149	-2.88092	-1.75251	-3.32193
MRPL50	3.88049	3.74214	3.34659	3.76521	3.69383	3.86163	3.86813	3.91271	2.69211	2.80645	2.65567	2.34152	3.9645	4.17077	2.95423	3.18248
ZNF189	1.74653	1.66465	1.55537	2.15918	2.18749	2.43987	2.68027	2.67631	1.77022	2.02421	2.11737	2.63415	2.74553	2.68611	2.86546	2.7576
TMEM246-AS1	-0.85926	-0.49867	0.08713	-0.35188	-1.5996	-1.10475	-0.08559	-0.23212	-2.28702	-0.68467	-0.59741	-1.88307	0.21478	1.99086	1.9918	1.58797
RNF20	4.03297	4.20151	3.87903	4.33185	3.58274	2.67485	3.87171	2.95787	4.48255	4.11034	4.24477	4.71741	4.22705	4.38499	4.63166	4.80308
SMC2	4.00425	3.74342	3.7736	4.2169	4.28662	2.43991	4.82519	2.67994	4.09375	3.37665	3.34077	3.89387	5.1013	4.55971	5.13995	4.4949
NIPSNAP3A	2.54463	2.76683	3.14572	3.56678	1.81971	2.05927	2.19664	1.77446	2.95286	2.83498	2.6812	2.8397	4.3521	3.81924	3.54961	3.38846
NIPSNAP3B	-0.92244	-1.1204	-0.72117	-0.20965	-2.1774	-0.28528	-2.10128	-0.71641	-1.52068	-0.28348	-1.3389	-0.30506	-0.27606	1.01394	-0.29608	0.43497
ABCA1	3.21998	3.04724	3.48212	3.29827	3.93604	2.24463	3.98146	2.3941	2.96235	2.97109	3.64918	3.68889	3.50257	2.51086	3.79514	3.44585
SLC44A1	2.30812	2.39114	2.49304	2.48899	3.10033	1.49599	3.51881	1.53153	2.9091	3.03089	2.88217	3.82927	2.48024	1.71836	2.79042	1.75136
FSD1L	-0.16223	0.34826	0.38981	0.60267	-0.6728	-0.52312	0.15343	-0.14596	-0.89321	-0.81149	-1.20507	-0.47737	0.87657	0.94249	0.41995	0.2119
RALGAPA1P1	-1.51838	-1.82768	-2.01425	-2.04993	-0.93475	-2.06789	-0.22496	-2.40449	-1.06985	-1.71712	-1.90122	-0.41631	-0.42674	-0.41241	-0.50143	-0.00096
FKTN	0.86393	0.94266	0.83417	1.30958	1.88557	0.4848	2.26282	0.60895	1.29787	1.05338	0.79214	1.79535	3.52554	2.77952	2.70714	2.61039
TMEM38B	4.11885	3.90582	3.67102	4.42001	3.98212	2.84048	4.06921	2.71064	1.95595	1.42662	1.46241	1.42063	4.1656	3.96713	3.3624	3.30819
ZNF462	2.45621	2.30219	2.31697	2.19651	-3.21001	-3.23241	-3.23909	-3.32193	1.85208	1.76154	2.2911	2.35205	2.65759	2.39498	3.06505	2.80248
RAD23B	6.06245	5.65601	5.94777	5.55125	5.59621	4.30671	5.87531	4.35211	6.15093	5.33567	5.87588	5.37238	6.67461	6.15226	6.74713	6.04709
KLF4	3.40015	3.40264	3.77823	2.27487	3.61607	2.53722	3.54386	2.19078	1.70304	1.92682	1.964	1.86356	2.80658	1.83885	0.83259	1.14492
ELP1	3.38094	3.22895	3.3838	3.44268	3.86061	2.85263	4.1128	3.08917	3.54165	2.96291	3.43494	3.55363	4.51419	4.42207	4.45019	4.33981
CTNNAL1	5.28055	5.04501	5.02722	4.7387	4.05467	3.25151	4.60579	3.34395	5.94724	5.74597	5.61978	5.49057	6.63733	6.60749	6.3219	5.74373
TMEM245	3.46377	3.37549	3.24711	3.74036	4.80583	3.7411	4.9513	4.13996	4.01659	4.08371	3.4111	4.38963	6.26116	6.07565	5.98558	6.29898
EPB41L4B	1.32963	1.39609	1.53065	1.01968	2.2985	1.28128	2.93353	1.29571	2.77483	2.23769	2.31845	1.6866	2.13953	1.83824	2.04776	1.93362
PTPN3	1.20495	1.21024	1.48308	1.5083	2.97814	2.78639	3.37344	2.51252	1.39354	1.20253	1.73366	2.01899	2.0912	2.31956	2.56554	2.93639
TXN	9.1313	9.18044	9.06894	9.22132	7.87221	8.1655	8.12857	8.11982	8.16338	8.04302	7.93435	7.64584	9.17798	8.86275	7.88914	7.7975
SVEP1	-2.74443	-2.42705	-2.8481	-2.50675	-3.2888	-3.32193	-3.25009	-3.14547	-0.01443	-0.3288	-0.69991	0.38176	-2.80743	-2.2741	-2.71577	-2.31178
MUSK	-0.43487	0.27248	-0.25794	-0.03378	-3.32193	-3.32193	-3.32193	-3.32193	-2.00737	-2.28098	-2.10439	-1.96604	0.80473	1.52731	0.24124	0.8999
LPAR1	-1.39624	-0.68122	-1.85273	0.0598	-2.92465	-3.07257	-2.8907	-3.03273	5.0569	5.15979	5.34616	5.502	3.94276	4.62246	4.30652	4.74008
ECPAS	4.41535	4.24078	4.28731	4.34511	4.17673	3.46478	4.37188	3.28665	4.55881	3.93799	4.16914	4.88259	5.19352	4.47067	5.15744	4.71663
ZNF483	-2.69802	-3.32193	-2.60737	-3.09449	-3.16503	-3.32193	-2.9971	-3.10671	-1.27076	-1.10406	-1.97635	-0.93472	-1.22265	0.05622	-0.21437	0.46504
PTGR1	5.3791	5.33972	5.31468	5.70511	4.01952	0.85418	4.0721	0.9891	4.52841	4.14357	4.11227	4.23572	4.34281	4.2128	3.99344	3.89466
DNAJC25	3.74044	3.62781	3.72856	3.82723	3.72839	3.37247	3.74492	3.53536	2.62527	2.49995	2.26145	2.09842	3.35528	3.02017	2.73592	2.29373
DNAJC25-GNG1	5.90231	5.95431	5.76871	6.04598	5.26604	4.8813	5.4137	4.84101	5.62405	5.54758	5.30463	5.46779	6.8506	6.61239	5.9618	5.91293
GNG10	6.16861	6.20276	6.07163	6.20098	4.78899	4.65862	5.10246	4.66803	5.4839	5.39621	5.1377	5.38195	6.77497	6.52127	5.86288	5.8159
UGCG	5.35459	5.46084	5.24016	5.28753	3.34129	2.33572	4.37236	2.31634	6.54719	6.86545	6.63851	6.57021	6.50981	6.22724	6.46588	4.81323
SUSD1	3.84481	4.06697	4.07467	3.10475	2.78174	2.52272	3.40301	2.54543	1.37947	2.10586	2.04165	2.57037	1.93949	3.35598	3.32993	3.538
PTBP3	3.53278	3.30454	3.14561	3.47404	4.61286	2.27026	4.80193	2.60215	4.98043	3.55514	3.19164	3.68796	5.71661	4.358	5.09384	4.23603
HSDL2	4.57964	4.6647	4.55089	4.90639	4.34185	3.46563	4.91499	3.62805	4.1646	3.79461	3.89952	4.26957	4.59859	4.31456	4.32986	4.15837
C9orf147	-0.44917	0.15589	-1.07508	-1.00228	-0.99162	-1.41867	-0.72254	-0.53312	-1.46374	-1.15681	-2.0565	-1.48617	0.42085	-0.02672	0.68671	0.79174
KIAA1958	-0.47156	-0.1359	-0.25855	-0.37304	-0.02634	-0.68875	0.18673	-0.59308	-0.76554	-0.86072	-1.02259	-0.21301	1.37748	0.9408	1.73833	1.52742
INIP	2.27958	2.38426	2.60084	2.37094	1.94153	2.01387	2.52379	2.10968	2.66477	2.61044	2.5912	2.88155	3.38944	4.0831	3.68362	3.89676
SNX30	1.53038	1.60495	1.77048	1.95075	1.08498	0.09653	1.36618	0.03546	1.68558	1.8776	1.46086	2.69179	2.28503	2.12938	2.75208	2.63152
ZNF883	1.01441	1.31383	0.87858	1.43138	-0.51468	0.09851	-0.67835	0.04238	1.00933	0.97434	0.6048	1.46065	1.92044	1.92766	1.22596	1.58058
ZFP37	-0.51763	-0.65589	-0.82861	0.31158	-1.85528	-1.20607	-1.2939	-1.30367	-3.32193	-3.32193	-3.32193	-3.32193	-0.54851	-0.45537	-0.52541	-0.19299
FAM225B	-1.00528	-0.24994	-0.28631	-0.3933	-2.12624	-3.04468	-2.2682	-3.15248	1.40354	1.22843	1.47003	1.57563	-1.79183	-0.68154	-1.05843	-1.0035
FAM225A	-0.51871	0.24529	0.09046	0.11073	-1.96181	-2.99162	-2.11628	-3.11862	1.66726	1.52861	1.81841	1.87587	-1.60271	-0.40444	-0.71265	-0.74505

SLC31A2	3.09267	2.83338	3.13797	3.17958	2.60008	2.31869	2.5215	2.06768	1.83133	1.0925	1.54873	0.98589	2.85096	2.00711	1.91437	2.03303
FKBP15	4.04436	3.97803	4.08195	4.0718	2.21844	2.00338	2.44668	1.9502	2.52216	2.2901	2.63038	2.9	3.2973	2.54485	3.10693	2.86193
SLC31A1	3.79919	3.0572	3.65742	2.95309	5.19243	4.44546	5.57394	3.91632	2.5537	1.93996	2.88598	2.45834	3.83152	3.47426	4.77619	3.9003
CDC26	4.24726	4.51009	4.21475	4.06842	4.41522	4.71586	4.49065	4.74479	2.95934	3.74547	3.61823	2.9405	3.40664	4.08417	3.01955	3.55668
PRPF4	5.03728	5.00752	4.89351	4.71975	4.64843	3.90892	4.74247	3.93085	4.08992	3.50436	3.9665	3.5393	4.74367	4.64977	4.94593	4.6368
WDR31	0.40838	0.80099	0.63588	0.65055	-0.21467	-0.60656	-0.66956	-0.17469	-0.14456	0.49031	-0.26285	1.15889	-0.40876	-0.78837	-0.33828	0.42614
BSPRY	-3.32193	-3.32193	-2.84362	-2.81717	0.27894	-0.65112	-0.475	-1.01547	-3.32193	-2.35114	-3.32193	-3.32193	-3.32193	-2.98962	-3.32193	-2.60155
HDHD3	3.89221	3.76897	3.79735	3.88379	4.68735	5.16541	4.00622	5.21043	2.81614	2.7921	3.01486	2.24754	3.25044	3.32624	2.85719	2.90919
ALAD	2.04755	1.66758	2.22534	1.80801	3.36368	2.82299	2.79352	2.98509	2.57232	2.02329	2.69091	1.92832	2.23647	1.81916	2.15128	1.31401
POLE3	4.85109	4.40433	4.16729	4.35972	4.79945	4.11329	4.9841	4.1151	4.70312	4.29389	4.70925	3.8191	5.91735	6.15104	5.41709	5.43215
C9orf43	2.52605	1.84788	1.82908	1.60983	3.1989	1.6863	3.07386	1.88272	2.78977	2.87879	3.06796	1.65639	3.93929	4.21468	3.65424	3.77191
RGS3	3.50153	3.73812	3.57201	3.87471	-0.36087	-0.33263	-0.26867	-0.71632	1.44852	1.59417	1.72509	1.47395	2.02569	1.91708	2.32644	2.03475
ZNF618	0.23424	-0.2402	-0.15692	0.11275	1.84342	0.37184	1.4342	0.91599	2.32189	1.97081	2.12128	2.52107	2.01043	3.07385	3.5195	4.10099
KIF12	-3.32193	-2.21402	-2.93802	-2.34038	2.65712	4.15741	1.88873	3.90746	-0.64745	-0.00966	-0.1958	-0.65133	0.7068	3.12066	2.69229	3.44935
COL27A1	3.10717	2.8454	3.07977	2.3976	-0.05873	1.47297	-0.78174	1.09263	2.19606	2.49646	2.82449	2.45025	1.85809	1.77672	1.33395	2.53912
ORM1	-1.87684	-2.34605	-1.71736	-0.90246	5.77592	7.52389	5.03321	7.68172	-3.32193	-3.32193	-3.32193	-2.46069	-3.32193	-3.32193	-3.32193	-1.89904
ORM2	-2.30671	-3.32193	-2.17798	-2.1271	5.16167	6.88785	4.3841	7.10303	-3.32193	-3.32193	-3.32193	-3.32193	-3.32193	-2.47618	-3.32193	-1.32565
AKNA	0.6113	0.35986	0.17908	0.1351	-0.01556	1.11452	-0.64413	0.83239	1.0761	1.17652	1.05097	1.19298	0.76086	1.3992	2.04237	2.43138
WHRN	-0.11051	0.0609	0.25704	-0.62582	0.93663	1.53782	0.11159	1.3777	0.50439	0.83838	0.7496	0.11427	2.20109	2.38303	3.278	3.2689
ATP6V1G1	6.78378	6.92904	6.59858	7.18067	6.08574	6.82359	6.24454	6.74792	5.74988	6.0023	5.39637	5.11992	7.33952	7.72026	6.89621	7.12778
TMEM268	1.63807	1.42297	1.04066	1.23209	1.97251	1.69782	1.85097	1.3594	1.68933	1.41215	1.53703	1.42194	3.00318	3.09093	3.31696	3.34596
TNFSF15	-0.02118	1.56557	-0.24137	1.07007	-3.32193	-3.32193	-2.97087	-3.32193	-3.16572	-2.29085	-3.0543	-0.68437	-1.6281	1.52613	-0.69758	2.71396
TNC	2.40466	2.53265	3.18862	3.21437	-3.2734	-3.10424	-3.32193	-3.18994	2.79181	4.16514	3.10193	5.18675	3.92299	5.02264	6.39055	6.62388
PAPPA	0.40114	0.47212	1.10423	1.98569	-2.86747	-3.05479	-3.07364	-3.10839	4.11692	4.14696	4.95492	5.53354	0.26071	-0.63384	1.37335	0.52768
PAPPA-AS1	1.44854	1.60935	2.01459	3.20444	-2.46814	-3.32193	-2.9482	-2.86062	4.06869	3.73297	4.74838	5.8369	0.192	-0.24831	1.72507	0.63501
ASTN2	-0.74615	-0.60619	-1.53946	-0.9283	-0.6969	-1.53069	-0.87385	-1.48959	0.00464	-0.17556	0.14881	-0.16829	0.39784	0.20466	0.43444	0.20246
TRIM32	3.18804	3.00083	3.06112	3.28041	2.54112	1.48478	2.43021	1.58791	3.51855	3.44238	3.49401	3.81312	3.25676	3.43388	3.65838	3.97348
TLR4	0.35762	0.15187	-0.17262	1.26993	-3.15146	-3.32193	-3.32193	-3.32193	-3.0934	-2.9604	-3.32193	-2.23795	2.90984	2.29479	2.37166	3.18228
TPT1P9	2.46881	1.65564	2.58576	1.46918	0.25119	1.7251	-0.31342	1.57218	2.72114	3.67442	2.30716	3.33915	0.06538	-0.1123	-1.36465	-1.34116
BRINP1	-3.0604	-2.7897	-3.01687	-3.32193	-3.32193	-2.64129	-2.68356	-3.01579	-1.93394	-3.32193	-3.05183	-3.07289	1.35624	0.59635	2.65651	1.06882
CDK5RAP2	3.22692	3.05934	3.02277	3.15325	3.76471	2.92095	3.72819	3.16429	3.26372	2.26967	2.82034	2.86586	3.91376	3.57493	4.18109	3.9503
MEGF9	1.81293	1.86977	1.74663	2.12519	3.43546	2.37395	3.85816	2.39088	0.9078	0.84745	1.06455	1.06075	3.65453	4.35105	4.6016	4.74984
FBXW2	2.82805	2.89078	2.55981	2.99663	2.85791	2.03042	3.06333	2.16508	2.42071	2.20535	2.12253	2.88945	3.98863	4.23666	3.55235	3.92504
B3GNT10	1.25115	1.85568	1.60537	0.29972	0.60227	-0.75135	0.85634	-0.36205	-1.21318	0.26237	-0.14963	1.04873	1.24183	1.92119	1.55486	2.13475
PSMD5	3.63556	3.48103	3.38864	3.56275	3.64534	2.73743	4.10418	2.91377	3.43113	2.74473	3.29359	3.70401	5.1451	4.20841	4.49852	3.90046
CUTALP	0.7293	0.82876	0.85247	0.52879	1.22674	1.23843	1.3201	1.50316	0.13624	0.68006	0.46458	1.0748	1.06681	1.19998	1.2982	1.10764
PHF19	2.68446	2.59112	2.52793	2.18216	3.42927	3.31445	3.09896	2.96445	3.90295	2.99253	3.86285	3.32514	4.93018	4.74795	5.06522	4.57161
TRAF1	4.56389	4.52076	4.06513	3.99649	-0.00801	1.17232	0.15191	0.92873	-0.90604	-0.28716	-0.51782	0.11259	0.58053	0.14436	-0.2398	0.22494
C5	-2.44882	-2.37079	-2.33263	-1.37726	4.30541	4.32993	4.45515	4.58801	-1.52053	-2.22056	-2.24631	-2.08837	0.81284	0.34315	1.18041	0.7356
CNTRL	1.06092	0.60841	0.45348	0.94947	0.87117	0.38767	0.71925	0.374	1.71391	0.74978	0.81258	0.85684	2.67848	2.64994	2.42336	2.5266
RAB14	4.28829	4.29361	4.3426	4.72704	4.10124	3.51607	4.83269	3.4218	4.52153	4.09938	4.34163	4.768	5.61662	5.02446	5.58331	5.15783
GSN	1.45869	2.41094	2.66501	2.93541	2.40178	3.00228	2.38827	2.24282	3.84758	4.62303	4.31451	4.6627	4.33658	4.97116	5.6375	5.74067
GSN-AS1	-1.84041	-0.99319	-0.64265	-0.60954	0.00946	-0.42947	0.72373	-1.09467	1.1998	1.97021	1.56207	2.42427	1.37967	2.10357	2.69567	2.921
STOM	4.09494	4.18268	4.1336	4.47776	5.55578	5.30853	5.85692	5.21133	5.00655	4.57613	4.53164	4.84862	5.94902	6.29523	5.60622	5.89991
DAB2IP	-0.47702	0.17083	-0.40325	-0.10881	0.98678	1.17371	0.70052	1.01532	2.34743	2.74517	2.89422	2.71518	1.57093	2.60598	3.34297	3.78199
TTLL11	0.5899	0.84828	0.40329	-0.3861	0.42241	0.4954	0.16954	0.32079	0.72833	0.94167	1.13256	1.0547	0.70561	1.24023	1.32804	1.29678
NDUFA8	6.84608	6.87975	6.78168	6.87322	6.46602	6.64456	6.08558	6.57043	6.34769	6.02651	6.02389	4.81164	6.54192	6.11331	5.48222	5.65658
LHX6	-2.97704	-2.63827	-2.60835	-2.89872	-1.08086	-1.09356	-1.35887	-0.95021	-1.87492	-1.52538	-1.82705	-1.97936	-3.32193	-3.32193	-3.32193	-3.32193
RBM18	2.8145	2.83364	2.53641	3.06742	2.54392	1.86234	2.63145	1.91827	2.32662	2.11193	2.10292	2.36286	3.28511	3.04123	2.65698	2.80104
MRRF	2.10555	1.92613	1.70323	1.75467	1.70821	1.815	1.98006	1.70506	1.73795	1.78883	1.7681	2.02326	2.88476	2.86672	2.83325	2.58812

PTGS1	-2.97927	-2.94241	-3.10929	-2.90138	-2.3042	-1.78716	-1.79744	-2.61022	-1.3363	-0.79021	-1.35251	-0.77873	-3.32193	-3.17835	-3.32193	-2.48612
PDCL	3.66983	3.68803	3.54477	3.61745	2.68166	1.97821	2.86898	1.77175	3.12575	2.7669	3.10133	2.49595	3.68608	3.35698	3.76219	3.14345
RC3H2	2.17743	2.11285	2.122	2.67932	2.24649	1.02173	2.8908	1.0915	2.59423	2.27076	2.32473	3.11099	3.97799	4.0824	4.20405	4.37106
ZBTB6	1.9702	2.14667	1.59033	2.36702	1.44205	0.96629	2.0498	0.9587	1.00826	1.67746	1.06853	2.0405	2.08119	2.65137	2.48188	2.52217
ZBTB26	0.24619	0.85329	0.89668	1.10167	0.63007	0.71135	1.1309	0.39208	0.29449	0.53946	0.65194	1.70839	1.44949	0.93587	1.02167	1.2126
RABGAP1	2.15789	2.12104	2.28305	2.73452	2.29682	2.12041	2.78665	2.13494	2.51162	2.56862	2.78864	3.51083	3.39314	3.55562	3.96087	3.90337
MIR600HG	0.11865	-0.49123	-0.30967	-0.8405	0.12633	0.04413	0.15958	0.25736	-0.38962	-1.62094	-0.64964	-1.38536	-0.51561	0.07277	0.94134	0.47613
STRBP	2.4408	2.24396	2.26344	2.36681	2.49791	1.98448	3.00813	1.99142	1.62049	1.65545	1.49099	1.91497	3.20772	3.50321	3.63755	3.51799
CRB2	-1.53748	-1.26682	-1.20724	-1.34664	0.55642	0.33549	0.40157	0.26905	-1.22834	-1.38493	-1.40126	-0.88799	-1.16888	-2.04013	-0.01887	-1.84198
DENND1A	1.53227	1.48595	1.48469	1.25291	3.58279	2.47983	3.26316	2.10849	2.28057	2.18166	2.37947	2.16686	2.24191	1.82058	3.06445	2.19616
LHX2	-2.65776	-2.59404	-2.56265	-2.01385	4.49722	3.7537	4.0491	3.6413	0.97733	0.56681	0.84466	-0.6121	1.68464	2.11919	2.28468	1.74505
NEK6	4.08114	4.13793	4.25058	4.13334	3.1757	2.69884	3.10578	2.43457	5.00405	5.34982	5.16691	5.21578	4.86729	5.19042	5.24908	4.84584
PSMB7	8.00244	7.85551	7.79657	7.77822	6.48509	6.82072	6.43518	6.57839	6.73016	6.34233	6.82172	6.17709	7.57473	6.92212	7.44405	6.992
NR6A1	-0.00963	-0.22072	0.34996	-0.06991	2.4112	1.79976	2.14137	1.79455	-0.32829	-0.94281	-0.51838	-0.24536	1.35987	0.97894	1.90725	1.83425
MIR181A2HG	0.89421	0.20504	-0.09947	0.78912	-2.73897	-2.19405	-3.32193	-2.06223	-0.63242	-1.18718	-0.07375	0.40333	1.51371	1.31007	-0.86845	-0.57337
OLFML2A	-0.19954	0.33592	1.3134	1.08325	1.13178	-0.70882	1.18906	-0.89462	1.29491	2.67339	1.7762	3.41676	2.16074	3.29141	4.45817	4.68611
WDR38	6.51535	6.05771	6.26306	4.63776	5.29429	6.20149	4.40256	6.04563	5.57427	5.49511	5.05309	1.51522	6.86189	6.50852	4.54141	4.44122
ARPC5L	4.95993	4.79838	4.82512	4.66184	4.00546	3.81527	4.07527	4.0324	3.15727	2.77789	2.80364	2.22199	4.87146	4.88611	3.79209	3.84885
GOLGA1	1.2909	1.32337	1.70585	1.89918	2.89816	2.70362	3.18254	2.74965	2.27388	2.40191	2.52701	3.08917	2.7259	2.69191	2.98505	3.09055
SCAI	-0.20203	-0.20128	-0.14329	0.19721	-0.49843	-0.98852	-0.40186	-0.81563	-0.16772	-0.6687	-0.52888	0.29286	1.11131	1.17047	0.70129	0.63687
PPP6C	4.28982	4.18562	4.26005	4.6154	3.37403	3.04918	4.0504	2.90624	3.92662	2.94429	3.73249	4.041	4.80404	3.8306	4.694	3.6509
RABEPK	3.51047	3.07896	2.92389	2.8909	4.09753	3.75793	3.79515	3.48211	3.49406	3.27716	3.65059	2.68676	4.18102	3.67424	3.75948	3.43452
GAPVD1	2.92747	2.98887	2.77437	3.27905	2.58625	1.80654	3.03902	1.65793	2.06759	1.98405	2.1284	2.90548	3.35728	3.27342	3.48403	3.58313
MAPKAP1	4.11087	4.30324	4.17179	4.27488	3.59046	3.59437	3.81688	3.36679	3.53219	3.80164	4.08043	3.85001	3.92165	4.47897	4.94392	4.60896
PBX3	1.83946	1.94512	2.01387	1.95823	2.04176	1.10065	2.39691	0.982	3.08845	2.48614	2.75642	2.81825	3.10629	2.53576	3.18306	2.73816
MVB12B	-0.14046	-0.34914	-0.26058	-0.06267	0.8493	0.73349	0.45216	1.22428	0.92332	1.23835	1.18407	1.26048	2.45501	3.33048	3.41852	3.47867
LMX1B	-3.32193	-3.32193	-3.32193	-3.09782	-0.6762	-1.28779	-1.19921	-0.79831	1.49744	0.94327	0.94141	0.70226	-2.92919	-3.32193	-3.32193	-3.32193
ZBTB43	2.27276	2.33375	2.47929	2.81981	0.51345	0.90248	1.2071	1.20111	0.73178	0.89663	0.66189	1.62964	2.2366	2.00328	2.21718	2.02257
ZBTB34	0.47754	0.22421	1.07336	0.80431	1.21129	-0.01317	1.83853	0.12718	1.13054	0.34132	0.95443	1.11135	2.56489	1.3857	2.64833	1.54051
RALGPS1	-1.77569	-1.91149	-1.4638	-1.80339	-1.61761	-1.62569	-1.70439	-1.26434	-2.36256	-2.48719	-2.44086	-1.6893	-1.38247	-1.18372	-0.87751	-0.83306
ANGPTL2	-2.32348	-1.43911	-1.47363	-0.02654	-2.81219	-0.34732	-2.86505	-0.74064	-3.32193	-3.32193	-3.05081	-3.32193	-2.76705	-3.32193	-3.07031	-2.20522
GARNL3	-1.48935	-1.47164	-1.52871	-1.2059	-2.1469	-1.54448	-2.51684	-1.83181	-2.30503	-1.62653	-2.15588	-1.71357	-0.45965	-0.66385	-0.60395	-0.61118
SLC2A8	2.83872	2.53041	2.74442	2.58774	3.84961	3.60246	3.3172	3.59802	1.84436	1.99525	1.54828	1.59553	2.99622	3.05946	3.50617	3.27546
ZNF79	2.028	2.12966	1.73785	2.50478	1.8796	1.44615	1.52078	1.57883	1.50753	1.36225	1.69751	2.07829	1.91512	2.08138	2.02123	2.06342
LRSAM1	3.28875	3.40019	3.49371	3.24868	2.71127	3.56988	2.50081	3.70752	2.70654	3.14475	3.12438	2.96093	2.6646	2.7246	3.22526	3.25386
STXBP1	2.93446	2.87284	2.93495	2.98958	2.20234	2.51499	2.09566	2.19751	2.41331	2.40914	2.03357	2.28573	2.53166	2.49867	2.6484	2.85013
PTRH1	3.35613	3.45236	3.18024	2.99858	2.03522	2.78715	1.75429	2.37873	2.88534	3.28529	3.23264	2.14353	2.80707	3.12348	2.9963	3.26092
MIR3911	5.9218	5.78776	6.07864	5.87103	4.7394	5.45993	4.96986	5.43603	5.56302	5.48317	4.69582	4.92957	5.11626	5.13414	5.1198	5.36093
CFAP157	2.97081	3.16846	2.7585	2.50142	2.07043	2.40304	1.51469	2.0371	2.46689	2.97851	3.01806	1.79876	2.52352	2.98545	2.80107	3.15005
TTC16	-1.44203	-1.84799	-2.42192	-2.06836	-2.49314	-1.25054	-2.47705	-1.59132	-1.88926	-2.12508	-2.50856	-2.8927	-1.30241	-1.27274	-2.37287	-2.04288
TOR2A	2.20818	2.10493	2.34149	1.82156	1.51303	2.4644	0.99306	2.27637	2.07504	2.16958	2.18594	1.58924	1.87098	2.27109	2.09088	2.62029
SH2D3C	-2.20683	-2.11476	-1.9552	-1.89841	-2.43519	-0.1385	-2.62416	-0.52613	-1.76792	-1.48592	-1.96965	-1.95499	-3.32193	-3.32193	-3.10899	-2.11997
CDK9	5.32818	5.36618	5.447	5.1122	4.46648	5.06508	4.45009	4.82359	4.71819	5.00033	4.97774	4.80026	4.27005	4.58202	4.69827	4.80721
MIR2861	3.70436	3.66481	3.94718	3.71849	4.2026	3.27664	4.69258	3.85397	3.82101	3.74471	3.84735	3.04878	2.27177	2.90786	2.55173	4.75646
FPGS	5.26536	5.40009	5.24321	4.98546	4.62117	4.85725	3.69642	4.71352	4.69842	4.83799	4.97614	4.17687	4.49167	4.65046	4.92384	5.05063
ENG	3.21892	4.12015	4.1008	3.86949	-1.50922	-0.9468	-2.24835	-1.3902	6.38338	6.34406	7.11681	6.29152	-2.16091	-1.5953	-1.3866	-1.22699
AK1	4.92295	5.12593	5.17642	5.12322	3.57327	3.82118	2.99103	3.81949	4.27402	4.00857	4.28201	3.39995	4.00735	3.86276	3.97149	3.74924
ST6GALNAC6	3.32836	3.62012	3.56222	3.39404	2.98319	4.00491	2.41739	3.62797	3.44325	4.17749	3.55277	3.23732	2.62581	3.00208	3.21312	3.67826
ST6GALNAC4	5.15862	5.15544	4.7008	4.65374	4.38664	5.38894	3.79135	4.69479	3.9765	4.52226	4.22396	3.53232	3.4731	2.84706	3.75609	3.71966
PIP5KL1	-0.13122	0.2253	0.80841	0.38113	-1.13551	0.34979	-1.98972	-0.19488	-1.54365	-1.6259	-1.36297	-2.25078	-0.27236	-0.24553	-0.38577	-0.40084

DPM2	5.59639	5.23406	5.32137	4.55218	4.2452	4.99251	3.82837	4.85822	3.81111	3.35115	3.74895	2.40327	4.38844	3.97147	4.58424	3.92686
FAM102A	4.26095	4.04514	4.09999	4.08858	4.395	3.71495	4.73058	3.48448	2.73689	2.64028	3.18205	2.63861	2.70029	3.07097	4.07377	4.32305
NAIF1	2.92636	2.71092	2.72261	2.35975	3.45417	2.69889	2.79545	2.83517	2.07409	1.66886	2.33036	1.17238	2.24323	1.87339	2.75943	2.28362
SLC25A25	2.80791	2.56226	2.08885	2.03202	2.4861	2.4056	1.95855	2.49461	1.11453	0.91774	0.91973	0.52761	2.88124	2.68563	2.36712	2.26163
SLC25A25-AS1	0.76808	0.48015	0.14679	0.40756	0.74694	1.90562	0.08887	2.11988	-1.18845	-1.34828	-1.10522	-1.02496	0.56513	0.57876	-0.02741	0.2513
PTGES2	6.30125	6.07968	5.74479	5.33974	5.39379	5.3988	4.59984	5.2609	5.31645	4.97273	5.20972	3.94949	5.25983	4.67124	5.23924	4.98988
LCN2	3.14442	3.91041	2.50624	4.05783	1.88328	-0.25741	0.75424	0.03461	-3.32193	-3.32193	-3.32193	-2.77554	-3.32193	-3.32193	-3.32193	-3.32193
C9orf16	5.55413	5.78215	5.73066	5.39282	4.52896	6.10825	3.26342	5.76757	4.59046	4.69	4.90439	3.27895	4.49822	3.94927	4.23121	4.45473
CIZ1	4.2242	4.13052	4.13973	3.82587	3.92935	3.32154	3.37387	3.32581	4.64448	4.44086	4.66054	4.05837	4.71065	4.51367	5.53953	5.2519
DNM1	3.65915	3.68092	3.97576	3.53186	1.08549	1.35702	0.11322	1.69786	-2.09567	-1.60291	-2.34371	-2.16546	2.3083	3.30754	3.65406	3.81279
GOLGA2	3.85961	4.01845	4.09817	4.59926	3.82461	3.98215	3.82061	4.06549	3.5531	3.63098	3.76454	3.83532	3.97514	3.85238	3.99167	3.76235
SWI5	4.85087	4.46787	4.49286	4.29631	4.18509	4.5583	3.64768	4.50921	2.94568	2.88891	2.64234	1.84006	3.32464	3.01309	1.90298	2.70713
TRUB2	3.93121	4.01028	3.80902	3.83842	3.22428	3.41717	3.02137	3.27132	2.75331	3.33733	3.50957	3.47292	2.90988	3.73823	3.69332	3.79829
COQ4	3.77516	3.67434	3.67826	3.39514	3.02962	3.69068	2.592	3.59055	2.59467	2.75573	2.91307	2.49383	3.15232	3.3969	3.22512	3.45362
SLC27A4	5.19319	5.1545	5.0867	4.97193	5.92044	5.40303	5.47345	5.31525	4.96463	4.17603	4.91116	3.98481	5.17444	4.6324	5.54696	5.36742
TMSB4XP4	1.23912	1.1469	1.62091	0.27474	0.85342	1.25434	0.05355	1.55488	0.34233	1.33459	0.01534	1.56429	2.51301	1.73778	1.45305	1.28009
URM1	4.62356	4.28962	4.1674	3.91615	3.74705	3.72207	3.22225	3.6161	3.90615	4.0199	4.01453	3.16336	4.25023	4.10701	4.24433	4.19415
CERCAM	3.45449	3.67428	3.98385	3.09987	4.3125	4.46878	3.35606	4.37464	4.73026	5.28802	5.06093	4.28307	3.13759	3.51089	3.92054	4.25364
ODF2	3.60206	3.07244	3.3254	2.80981	3.3418	3.41029	3.12307	3.26972	3.70018	2.62662	3.29226	2.53241	3.61642	3.21316	3.35315	3.13487
ODF2-AS1	4.42342	4.36789	4.53255	3.65741	4.97835	4.21795	4.782	3.42763	5.23959	3.9585	4.74247	3.47604	5.14393	4.73322	4.97339	5.09401
GLE1	4.20573	4.16847	4.07132	4.31518	3.71661	2.85192	3.65169	2.85182	4.15172	3.35655	3.86546	3.85113	4.6662	4.55858	4.86676	4.77463
WDR34	6.05217	5.83045	5.84852	5.13616	6.27222	5.36366	5.17061	5.40934	6.26437	5.61012	6.20497	4.22869	6.09934	6.40109	6.86824	6.60744
PKN3	4.64281	4.46201	4.37889	3.79893	4.82601	4.44887	3.8433	4.54698	4.84571	4.58258	4.77488	3.37405	4.44328	4.80163	4.79629	4.74443
ZDHHC12	4.36467	4.57989	4.66157	4.35563	4.52974	5.55167	3.83504	5.48367	4.09772	3.81821	3.80743	3.41515	4.37831	4.61212	4.64604	4.7339
ZER1	2.9845	3.10299	3.40591	3.1114	2.82777	3.08811	2.45189	2.92181	2.97111	3.63187	3.47994	3.96096	2.80187	2.73204	3.73435	3.97414
TBC1D13	3.9558	3.71572	3.62777	3.57117	3.95774	3.43041	3.6718	3.39036	3.58424	3.18928	3.81064	3.37223	4.00373	3.69564	4.64055	4.31746
ENDOG	5.77783	5.68956	5.67734	5.08566	5.78503	5.71679	4.774	5.73515	5.52764	5.06323	5.23887	4.07568	5.51911	5.25743	5.82333	5.27353
SPOUT1	4.16273	3.79582	3.82309	3.50901	4.11946	4.09011	3.4364	4.13798	3.6878	3.29927	3.6632	2.66253	4.18351	3.83863	4.07988	3.71267
KYAT1	1.77379	1.43085	1.86873	1.30984	1.93703	2.04527	0.86331	2.28196	1.09845	0.9391	0.62487	0.33026	1.37847	1.33579	0.9255	1.10308
LRRC8A	4.27295	4.51217	4.61175	4.5383	4.49382	4.3789	4.70063	4.38247	3.371	3.79944	3.63744	4.06059	4.29766	4.9881	5.61372	5.76223
PHYHD1	-2.98136	-3.32193	-2.92639	-2.31592	-2.78358	-3.32193	-3.32193	-2.61408	1.3476	1.68105	1.42905	1.15069	-2.0232	-2.449	-2.30985	-1.96638
DOLK	4.13368	3.92093	3.75215	3.96071	5.44408	5.10977	4.80398	5.22754	3.63749	3.21212	3.69045	2.85726	4.72404	4.62881	5.05836	5.10053
NUP188	4.30531	3.96973	3.79485	3.7925	3.86456	2.92872	3.88349	2.99141	3.95456	2.81053	3.35739	3.11816	4.61747	4.21179	4.80375	4.52991
SH3GLB2	3.87011	3.68754	3.90535	3.13501	3.7314	4.20248	2.86957	3.71955	2.9497	3.08599	3.03048	2.23923	4.13373	3.90625	4.15501	3.9722
MIGA2	2.77327	2.19056	2.38439	2.02308	3.0624	3.17042	1.9696	3.07886	1.78047	1.51957	2.05113	0.92444	3.02107	2.78396	3.09662	2.96637
DOLPP1	3.63459	3.27887	3.21071	2.73004	4.63374	4.08937	3.94193	4.08839	3.10498	2.25061	3.11173	1.45938	4.26816	4.01879	4.12356	3.68898
CRAT	2.87674	3.2605	3.52507	3.22083	4.8932	5.02125	4.07423	4.7905	4.51406	4.45448	4.47888	4.12229	4.48875	4.40769	5.47022	5.23193
PTPA	5.22982	4.94268	4.81367	4.48314	5.20353	4.99354	4.92221	4.70597	5.29953	4.62986	5.20212	4.37372	5.79239	5.70532	6.61037	6.43673
IER5L	6.40034	6.45863	6.64022	5.84074	5.54698	7.6064	4.4462	7.35954	4.00356	5.06982	4.74531	3.96819	5.80598	5.6651	6.10981	6.34594
LINC01503	0.99922	1.29275	1.00258	1.20964	-2.0306	0.05013	-3.32193	-0.13483	-1.18583	-1.79683	-1.99241	-2.7744	0.3154	0.69633	0.04228	0.60923
LINC00963	1.40039	1.80445	1.81303	1.94734	3.10626	3.81162	2.92336	3.90628	0.8414	1.16492	1.10915	1.69575	0.82469	1.11464	1.67041	1.51031
NTMT1	5.46923	5.16266	5.01923	4.77613	4.32532	4.69216	3.84347	4.42384	4.0531	3.811	4.02607	3.08823	5.22795	5.27815	5.07837	5.11713
ASB6	5.12902	4.86137	4.64998	4.65527	3.99902	4.29333	3.51507	4.23681	3.66996	3.50715	3.7166	3.06929	4.71728	4.67783	4.30884	4.44598
PRRX2	-3.32193	-1.45092	-2.05725	-0.51895	0.20914	-0.00312	-2.24797	-0.08166	4.2497	4.01779	4.14577	1.81698	-2.37513	-2.37761	-1.61667	-2.21395
PTGES	7.64049	7.89874	7.4154	8.58593	3.09285	2.41004	2.87969	1.44846	1.7379	4.91545	2.44443	6.17473	-2.60701	-0.56663	-0.91619	-0.03088
TOR1B	4.77619	5.03599	5.00154	5.12524	5.03819	4.50911	5.23476	4.37464	3.3509	3.69828	3.67865	4.12213	4.69815	5.01057	4.60487	4.71415
TOR1A	3.78925	3.9439	3.81411	4.28985	3.70446	3.57968	3.72461	3.37101	3.17553	3.38045	3.27587	3.92804	4.29305	4.47488	3.82661	4.2498
C9orf78	4.72341	4.49824	4.28297	4.60011	3.43278	3.64254	3.36674	3.69531	3.89635	3.7376	3.88095	3.46435	4.21407	4.3092	3.62483	3.66798
USP20	3.56537	3.53439	3.36118	3.15128	2.16614	2.40268	1.88061	2.20093	2.9718	3.49093	3.49995	3.23704	2.94055	3.1593	3.00819	3.4015
FNBP1	4.26725	4.35514	4.79536	4.10571	3.34886	2.43687	3.52554	2.14467	3.27322	2.6766	3.15897	3.34259	3.84688	2.64185	3.77689	3.12022

GPR107	3.76765	3.95364	3.93474	4.16679	4.43752	3.43622	4.68885	3.25923	3.26837	3.51834	3.42914	4.28676	3.9983	4.07036	4.46262	4.62917
NCS1	4.53953	4.56494	4.47676	4.25178	3.09081	2.40405	2.63439	2.24038	4.37544	3.99021	4.33168	3.86193	4.09034	4.58401	4.92287	4.73681
HMCN2	-3.02481	-2.99231	-3.19719	-2.90314	-2.1543	-0.93264	-2.4314	-0.69712	-2.74921	-2.55782	-2.96986	-2.87692	-3.32193	-3.32193	-3.27036	-3.21841
FUBP3	4.74074	4.79198	4.6627	4.55869	4.23256	3.29754	4.23822	3.20198	4.213	4.29891	4.37666	3.8013	4.70425	4.90967	4.53459	4.59257
EXOSC2	4.34401	3.88854	3.71992	3.45731	3.4695	2.83072	3.30355	3.15313	3.39583	2.67841	3.20492	2.5166	4.60753	3.7659	3.77576	3.45426
ABL1	4.74279	4.69738	4.55892	4.42544	3.959	3.40468	3.58927	3.54546	5.86012	5.80808	5.5601	5.57653	4.89322	5.09946	5.70609	5.70591
FIBCD1	2.3687	2.66747	3.19731	2.79786	0.43038	-1.81426	-0.85242	-1.77216	0.48042	0.93871	-0.33539	0.51114	-1.95057	-2.24671	-2.49517	-0.23233
LAMC3	-3.05361	-3.16526	-3.00908	-3.32193	-2.80183	-2.62593	-2.95881	-2.75026	0.39145	0.47617	0.55167	0.09608	-3.01135	-2.7576	-3.18761	-2.83759
AIF1L	-0.93029	-1.0646	-1.40494	-1.47852	-1.44426	-1.28285	-1.71276	-1.04914	-1.36061	-1.38657	-1.61002	-2.15224	4.35038	3.49888	5.04334	3.56145
NUP214	3.07866	2.71275	2.82725	2.66296	2.66463	1.97778	2.41119	2.06762	2.95079	1.92434	2.85726	2.6441	3.05993	2.23448	3.6479	3.00267
FAM78A	1.33776	0.95428	0.95554	0.87049	0.44867	0.39282	0.01298	0.19005	-0.71651	-0.48914	-0.51568	-0.74891	-2.03657	-1.64225	-1.79259	-1.26263
PLPP7	-2.92205	-2.87993	-2.85894	-2.17814	1.44254	2.61034	0.66963	2.39684	-2.86644	-3.32193	-2.32727	-2.94033	-3.32193	-3.32193	-3.32193	-3.32193
PRRC2B	4.53954	4.37686	4.40081	3.95838	4.39542	3.30524	4.1511	3.58873	5.03036	5.15269	5.20988	5.24547	4.942	5.36529	6.29369	6.40687
SNORD62B	0.92974	2.62705	2.44516	1.25008	2.10973	1.50456	1.79477	0.2307	0.20036	1.84035	3.37627	-0.06562	3.42025	3.75333	2.80592	4.95065
POMT1	2.61176	2.75444	2.91193	2.97892	3.64553	3.78496	3.12259	3.79706	3.1324	3.098	3.17927	3.13871	3.47952	3.25354	3.73102	3.7206
UCK1	4.38068	4.31478	4.223	4.0478	3.87341	3.82785	3.55706	3.93351	3.52071	3.54284	3.38119	2.97703	3.30003	3.83339	3.62846	3.65655
PRRT1B	-2.65149	-3.32193	-3.32193	-3.32193	4.09878	3.88883	3.10494	4.20114	-2.56696	-1.00201	-3.32193	-2.23679	-0.71363	-0.25349	0.15914	0.87329
RAPGEF1	3.41158	3.4428	3.45935	2.84686	4.66399	3.60525	4.256	3.26584	4.65005	4.45246	4.50375	4.32962	4.57802	4.81917	5.55889	5.4266
MED27	4.10054	4.02741	3.92594	3.77871	3.0554	3.17606	3.21419	3.02106	4.15242	3.30277	3.82973	3.27842	4.04615	3.89252	3.64553	3.33529
NTNG2	-1.61024	-0.56188	-0.23425	-1.11345	-2.61683	-2.47376	-3.07741	-2.55129	1.93142	2.97904	2.05961	2.47467	-3.11402	-2.93355	-3.07229	-3.32193
SETX	2.36652	2.51926	2.516	3.09183	2.92484	2.20735	3.61962	2.23978	3.0369	3.13525	3.22835	4.38124	3.90981	3.64982	4.38513	4.4241
TTF1	4.46388	4.4447	4.2308	4.90083	2.3427	3.06175	2.47332	3.31032	2.73075	2.54265	2.23312	3.07948	3.00438	3.26837	2.57392	2.64897
DDX31	2.62282	2.41427	2.10251	2.40914	2.54777	2.32066	2.63841	2.30072	2.22368	2.05723	2.20886	2.23372	3.17731	3.09132	2.81442	2.93584
GTF3C4	2.98995	2.97532	2.70173	2.9721	2.67695	1.1933	3.01279	1.27337	2.98935	2.87284	2.9166	3.58046	4.02659	4.22384	4.23762	4.41492
AK8	1.48533	1.50751	1.92734	1.91049	-1.31566	-1.36834	-1.67937	-1.19094	-0.45873	0.1557	-0.11353	-1.54595	-0.6069	-0.87912	0.17767	-0.49385
SPACA9	1.42456	1.69934	2.08289	2.02027	-0.49988	0.10152	-1.4875	0.02234	1.58882	1.7259	2.07081	1.94601	0.76031	0.14888	1.3895	0.86161
TSC1	2.52061	2.52837	2.57163	2.94526	1.90372	2.12684	2.29351	2.18064	1.79404	1.96285	2.03254	2.79435	2.97015	3.06435	3.5672	3.9311
GFI1B	-1.54968	-1.48647	-1.16338	-1.94057	-1.0649	-1.97557	-0.52817	-2.5095	-2.19605	-2.24914	-1.74866	-2.34791	-0.76586	-0.98007	0.25407	0.55079
GTF3C5	4.85526	4.75851	4.6979	4.25433	4.49538	4.35939	4.17717	4.37769	4.27524	4.00165	4.32989	3.82497	4.65845	5.30384	5.45058	5.56408
CEL	-1.3474	-0.89302	-0.93164	-0.18301	-2.15561	-0.33782	-2.17427	-0.92703	-0.75649	-0.33889	-0.03378	-0.67886	-0.29666	-1.13354	-1.65483	-0.87627
RALGDS	0.6376	0.91262	1.08004	1.44448	1.32415	1.75406	0.4775	1.47296	2.51616	3.13224	2.78261	3.1446	2.01143	1.99782	2.36298	2.34682
GBGT1	-3.04808	-3.32193	-3.32193	-3.32193	-3.19922	-2.28159	-3.32193	-2.51799	-2.34237	-0.93433	-1.47454	-1.74585	-0.57099	-0.76238	-1.06135	-0.61982
SURF6	4.21375	3.96676	3.90186	3.97939	3.96129	3.96701	3.49792	4.06201	3.32289	3.85625	3.8042	3.54974	3.64172	3.97256	3.76589	3.80061
MED22	3.31654	2.89837	3.06361	2.11037	3.36813	2.80465	2.61758	2.79422	2.2951	2.37772	2.53843	1.75683	3.33858	3.51166	3.81568	3.95921
SNORD36B	4.53443	4.76423	4.71114	4.58089	3.09816	3.6809	3.78723	3.33339	2.89036	2.62485	2.72662	1.06249	4.4238	2.54379	2.61073	2.38336
SNORD36A	6.58175	6.3012	6.72661	6.63546	6.04493	6.31554	5.60669	6.81565	4.01483	4.13165	4.89905	4.3404	6.95659	6.59059	4.88445	3.23843
SNORD36C	5.48497	4.92872	5.18278	5.78206	5.02515	6.6262	4.59984	7.17156	0.55273	-3.32193	2.88932	3.62792	5.46991	5.23792	1.79418	2.54542
SURF1	5.0983	5.14043	5.11108	5.02399	5.21064	5.76841	4.72181	5.73056	5.08367	4.93614	5.12475	4.55698	4.48797	4.8494	4.93181	4.83218
SURF2	5.64367	5.35978	5.32715	4.65963	4.50437	4.9066	3.82648	4.93095	4.57931	4.53086	4.77919	3.36721	4.71585	4.63945	4.7708	4.52463
STKLD1	-0.34615	-0.52935	-0.16381	-0.937	-0.39964	-0.12203	-1.11536	0.03062	-1.22617	-1.36352	-0.80029	-1.2048	0.11018	-0.13457	-0.72664	-0.82644
REXO4	4.81478	4.39514	4.29318	4.04038	4.64109	3.97103	4.33756	3.87517	4.46326	4.417	4.6175	4.03326	4.78738	4.656	5.13159	4.74783
ADAMTS13	-1.92596	-0.92325	-0.59116	-0.78434	-0.65052	1.13972	-1.98676	1.26093	-0.35919	0.7763	0.48118	0.60207	-1.94461	-1.15112	-1.64074	-1.8757
CACFD1	-0.09256	0.09108	0.5951	0.37505	5.03439	5.19	4.52787	5.04494	1.86265	3.02825	2.5215	2.37274	1.44658	2.11002	3.26215	3.38239
SLC2A6	4.83249	4.94766	4.95342	5.16538	4.06758	4.43901	3.28075	4.39147	3.53204	3.52827	3.819	3.58108	3.82798	4.36842	4.45361	4.81062
DBH	-1.02	-0.39242	0.21975	-1.25488	-0.47367	-0.52242	-2.1137	-1.31982	-1.60617	-0.17587	0.16105	-0.33624	-3.32193	-3.32193	-3.32193	-3.32193
DBH-AS1	0.4154	0.31071	1.1695	1.11186	0.13967	-0.32729	-2.08634	0.76282	-0.62531	0.40557	0.57605	0.8363	-3.32193	-3.32193	-3.32193	-3.32193
SARDH	2.32957	2.77707	3.14208	2.40216	1.58665	1.40283	0.44289	1.3103	1.83636	2.3083	2.37077	2.04895	-3.32193	-3.32193	-3.12692	-2.94802
VAV2	3.9383	3.83452	4.01518	3.59917	3.76915	3.33414	3.25132	3.35354	3.28353	3.54407	3.51172	3.47166	3.79079	4.04994	4.52277	4.68105
BRD3OS	3.14829	3.27895	3.17195	3.44037	1.12347	1.81968	1.39953	1.93621	2.75217	3.11319	2.88432	3.55747	3.20151	3.55016	3.51086	3.74203
BRD3	3.29051	3.42178	3.32129	3.64019	2.59787	2.45292	2.36663	2.80471	3.01147	3.22355	3.0403	3.39095	3.70259	3.80143	4.29558	4.45639

WDR5	4.55309	4.34935	4.18714	4.02433	4.20537	3.71815	4.11006	3.94134	3.9783	3.91383	4.17535	3.78929	4.71633	5.06567	4.88778	4.54146
RXRA	3.10874	3.25293	3.10337	2.82486	2.61425	2.01962	1.9875	1.9693	2.64537	2.65716	2.44315	2.43118	2.02289	2.28162	2.94213	3.28247
COL5A1	3.38053	3.88895	4.35325	2.85357	-2.52576	-2.88826	-2.39102	-2.66427	5.60255	5.75001	6.01264	5.85944	1.17465	0.94065	2.08377	2.32995
OLFM1	-2.61372	-1.96298	-1.76014	-1.55629	-3.10594	-2.99273	-3.01551	-3.11934	2.285	2.05165	2.279	1.88004	-3.32193	-3.32193	-3.32193	-3.00301
PPP1R26-AS1	0.30838	-0.20658	0.45946	0.22801	-0.61316	-1.00697	-0.59891	-0.2971	-0.6683	-0.89146	-1.32134	-0.96571	-0.03568	-0.89909	-0.10482	0.41361
PPP1R26	4.21081	4.24957	4.34332	3.7155	3.45313	3.76448	3.44712	3.74201	4.0367	4.12386	3.93483	3.75951	3.51021	4.05027	4.26416	4.52786
C9orf116	1.61177	1.94063	1.90702	2.08831	1.43269	2.61355	1.25888	2.45894	0.72135	1.30387	0.86817	0.36083	1.7067	2.58873	1.73119	2.61202
MRPS2	5.49844	5.12613	4.97139	4.39066	4.72759	4.80562	4.1004	4.91213	5.19098	4.79074	4.90795	4.20198	5.68239	5.36759	5.46064	5.27223
CAMSAP1	2.84696	2.60348	2.609	2.57566	2.55357	1.22264	2.6884	1.24157	3.16728	3.0275	3.06858	3.51927	3.75898	3.87118	4.61184	4.45203
UBAC1	4.53986	4.23381	4.19218	4.08511	3.86849	3.74726	3.75746	3.53175	4.15015	3.97972	3.91447	3.46484	4.24795	4.17947	4.06315	3.93101
NACC2	2.83553	3.02154	3.05496	3.04223	4.71838	3.94571	4.33561	3.98001	4.69936	4.50868	5.0414	4.59431	4.69902	4.89547	5.85048	5.76176
TMEM250	5.33435	5.58161	5.66558	4.94878	5.39171	5.17303	4.75607	5.00179	5.1948	5.83629	5.9594	4.58478	4.14422	5.02151	5.2085	5.48739
QSOX2	4.45128	4.08937	4.12553	3.99582	4.68728	4.17777	4.61968	4.2869	3.6538	3.57442	3.79788	3.48665	3.92091	3.92218	3.84602	3.97569
GPSM1	1.87378	2.64097	2.69795	1.91123	1.7061	2.92869	1.36828	2.58329	3.25227	4.29094	4.34577	3.12443	2.53116	3.70961	4.38817	4.3357
DNLZ	4.07989	4.06389	4.01138	3.53983	3.46538	4.1372	2.82883	3.92522	2.9722	2.6959	2.77097	1.30077	2.776	2.88069	2.33612	2.32606
CARD9	3.11274	3.09853	3.01585	2.489	2.45496	3.07729	1.77857	2.91743	1.89628	1.61906	1.6765	0.23431	1.63849	1.61659	1.25014	1.16873
SNAPC4	4.91826	4.78603	4.66038	3.90086	2.82066	3.24714	1.83219	3.68204	2.73608	2.93562	3.26039	2.29223	3.11187	3.22453	3.42214	3.94325
ENTR1	5.10579	4.7135	4.59138	4.59201	4.67705	4.57406	4.32926	4.69056	3.76907	3.52887	3.74343	3.20716	4.62694	4.63759	4.30237	4.19975
PMPCA	4.65991	4.54138	4.2963	4.17473	4.13187	4.03681	3.81594	3.96669	3.89441	3.50376	3.8948	3.38694	4.36339	4.17803	4.32777	4.29389
INPP5E	3.24786	3.4128	3.64021	2.45668	2.13644	2.75491	0.87202	2.94402	2.27501	2.61886	2.82519	1.50767	2.5945	2.67628	2.5067	2.49355
SEC16A	4.17331	4.17015	4.0467	4.26041	4.71597	4.1144	4.55864	4.08969	4.28868	4.19156	4.26082	4.72826	4.59122	4.55251	4.88167	5.33612
C9orf163	-2.3922	-0.98201	-0.36033	-2.50506	0.71132	-0.43351	0.74567	-0.42315	-1.53354	-1.4668	-1.07407	-1.17415	-0.0729	-0.04209	0.22103	0.65242
NOTCH1	4.40384	4.46932	4.56066	3.99342	2.72225	1.16637	1.39493	1.62318	2.82479	2.94017	3.00618	2.41353	2.81597	3.32422	3.53014	4.04215
NALT1	1.56237	1.65192	2.08443	0.70567	-0.03123	0.92836	-2.10052	0.70242	-1.51974	-1.17157	-2.52369	-2.08773	-1.90155	-0.87437	-0.97528	-0.76785
EGFL7	0.98901	0.55076	0.72132	0.4904	4.7683	5.26181	3.44404	5.30425	4.7392	5.3091	4.89102	3.91239	3.42892	3.39807	3.59194	4.16188
AGPAT2	5.80735	6.01856	6.19234	5.30967	6.23985	6.54484	5.67327	6.42532	5.80849	5.93361	5.89443	4.73204	5.55057	5.65947	6.58623	6.53038
DIPK1B	-0.56906	-1.24414	-0.88347	-1.49965	4.30025	4.66214	3.42085	4.53257	2.58622	3.51352	3.15883	2.69919	1.96355	2.66707	3.25201	3.75668
SNHG7	3.92201	3.25747	3.23439	2.36488	4.97656	5.28145	4.19041	5.27201	2.90524	3.65791	3.41494	2.3917	4.32188	3.85701	3.4901	3.3593
SNORA17A	3.69356	3.10681	3.38821	1.91967	3.97395	3.70932	2.84423	4.06677	2.05996	1.54279	-3.32193	-0.6144	3.3445	4.08364	1.52893	2.90748
LCN15	-2.97843	-3.32193	-3.32193	-3.32193	-0.90546	1.98539	-2.31922	2.10056	-2.36778	-3.32193	-3.32193	-3.32193	-3.32193	-3.32193	-3.32193	-3.32193
TMEM141	4.71368	4.73346	4.7441	4.79008	6.79711	8.35163	6.02716	8.12291	3.73917	4.24632	3.95216	3.2529	4.73586	4.73301	4.06505	4.49933
CCDC183	-0.21866	0.1746	0.3194	-0.21711	3.21676	4.01527	2.26823	3.54668	-0.56003	-0.40259	-0.63819	-0.63015	-0.28985	0.51466	0.03563	0.83713
CCDC183-AS1	-0.0805	-0.14686	0.53485	-0.48206	3.48278	3.74629	2.44464	2.99867	-1.45536	-0.89128	-0.57164	-1.29207	-0.59807	0.48029	-0.53519	0.9089
RABL6	5.18455	4.97268	4.87984	4.28092	4.96617	5.78344	4.12309	5.8715	4.80107	4.50299	4.70888	3.70644	4.86165	4.89195	5.59254	5.46104
MIR4292	3.03716	2.98205	4.16517	2.16577	5.13052	5.40135	4.57824	5.67465	1.48178	2.766	1.88754	2.16755	3.95678	4.25474	3.61656	3.99331
AJM1	1.14096	1.41226	1.5813	1.24977	3.346	3.1911	2.58784	3.60873	1.80447	2.42531	2.9596	2.02078	1.00911	1.2996	2.32145	2.64296
PHPT1	5.67081	5.83845	5.99966	5.75845	6.73625	7.70658	6.01829	7.65794	6.7368	6.49753	6.64677	5.33963	6.04841	5.78166	5.49072	5.4635
MAMDC4	1.75018	1.9363	2.25518	1.66151	3.31445	4.421	2.3375	4.3616	2.68206	2.47513	2.73482	1.69958	2.69883	2.62028	1.17269	1.47854
EDF1	7.93987	7.76595	7.82303	7.45717	7.50078	8.53098	7.31233	8.28452	7.51279	7.46841	7.47997	6.08821	7.95759	7.74264	7.6619	7.58712
TRAF2	4.62021	4.29984	4.28569	3.70849	4.97051	4.65332	4.27969	4.72852	3.89261	3.89255	4.25281	3.19833	4.44595	4.56598	4.73667	4.49405
MIR4479	3.31298	4.11079	4.082	0.56556	5.18237	3.6156	3.50132	2.72266	3.67875	3.67233	3.8896	-3.32193	3.251	3.86856	3.77269	4.8531
FBXW5	5.7204	5.91387	6.1891	5.45835	6.63329	7.0255	5.73844	6.93432	5.47345	5.9268	5.85127	5.24263	5.16161	5.41959	5.97808	6.05021
C8G	-0.22106	0.05071	0.85286	-0.20848	5.72758	7.08306	4.57721	6.75981	-0.31332	-0.26731	-1.02156	-1.66404	-0.52744	-0.41343	-1.36048	-1.33696
LCN12	-2.97107	-2.37553	-3.32193	-2.56054	1.37328	2.02879	-1.04249	1.39039	-3.32193	-2.10318	-2.95992	-2.29053	-3.04015	-1.87865	-2.28529	-1.93675
PTGDS	-2.22085	-0.94974	-0.18428	0.985	-2.17406	-1.36047	-2.47257	-0.97536	-2.58594	-2.68768	-2.65005	-2.97551	-3.02999	-1.62823	-2.07908	-0.79852
PAXX	5.20296	5.01547	5.04109	4.25501	4.93672	5.66315	3.63171	5.75096	4.22065	4.11195	4.17773	2.91218	4.56253	4.56822	3.43011	3.85851
CLIC3	1.87216	2.83413	4.23643	3.54484	-0.37309	-0.04883	-1.81271	-0.45909	-0.83681	0.38752	0.95931	-0.13187	-2.72109	-2.72284	-3.32193	-3.32193
ABCA2	1.05313	1.11467	1.61858	0.83582	3.9549	3.47778	2.81946	3.1582	1.96348	2.64962	2.16519	2.52007	1.27398	1.8157	2.83994	3.28733
NPDC1	1.76102	2.76885	2.44402	2.17091	-0.78158	3.50685	-1.68152	2.94274	3.5487	4.70319	3.96171	3.81819	2.36258	2.80869	2.51203	3.19358
ENTPD2	-2.94924	-3.32193	-3.32193	-3.32193	-0.11053	0.15485	-0.56105	0.11327	-2.89687	-1.88606	-3.32193	-2.68142	-3.32193	-3.32193	-3.32193	-3.32193

SAPCD2	5.51423	5.22353	4.85864	3.49215	5.33487	4.12382	4.26939	4.18874	4.06164	3.86303	3.54798	1.38117	5.63243	5.62315	6.18385	5.85814
UAP1L1	4.62601	4.55953	5.01573	4.57411	2.61962	3.32513	2.58745	3.31796	3.01033	2.8922	3.18738	2.56595	3.67618	3.985	4.59759	4.17041
MAN1B1-DT	0.01737	-0.94673	-0.2571	-0.80832	-0.22239	-0.1577	-0.25963	0.78852	-1.36162	-0.28696	-0.06042	-0.71089	-0.03361	0.86023	0.17526	0.91088
MAN1B1	4.05301	3.88973	4.31231	4.03046	4.61402	4.58758	4.07455	4.49714	4.09326	4.12194	4.357	4.18204	3.60199	3.43363	3.94556	4.0212
DPP7	5.71732	5.54101	5.76611	5.18616	-0.4288	2.28871	-1.38612	2.12285	5.31006	6.01562	5.73533	4.83222	5.73702	6.12552	6.16451	6.52998
GRIN1	-3.16444	-2.98975	-2.97337	-3.32193	-2.10619	-1.80296	-1.93515	-1.70944	-1.7974	-1.40333	-2.11133	-3.32193	-2.5371	-2.39564	-2.41045	-1.89521
LRRC26	-1.48689	-0.72649	-0.49731	0.20605	1.04475	1.68504	0.08238	2.20317	-2.51773	-1.80738	-3.32193	-2.63615	1.35858	0.38152	0.1119	0.14056
ANAPC2	4.09581	4.19122	4.05685	3.66984	3.86782	3.82674	3.10414	3.9594	3.4284	3.651	3.98099	2.9508	3.25623	3.60375	3.52698	4.08342
SSNA1	6.7752	6.64422	6.48606	5.66166	6.97903	7.23148	6.11097	7.15886	6.54361	6.29781	6.38121	4.54216	6.59739	6.56128	6.42165	6.54703
TPRN	3.96877	4.18701	4.11338	3.25868	3.77127	3.72387	2.51912	3.97733	2.51371	2.74494	2.74679	1.36794	2.80557	2.67022	3.20697	3.16705
TMEM203	4.89768	5.01963	5.09237	4.93342	5.33204	5.10102	4.64883	5.05238	4.75714	4.9388	4.64921	4.28001	5.18038	5.40194	5.20509	5.44585
NDOR1	4.58279	4.20459	4.50656	3.7293	4.41199	4.17233	3.26729	4.42092	3.03205	2.8997	3.28378	2.0248	3.61546	4.02623	3.9508	4.17365
RNF208	0.66426	1.81095	0.76478	1.66317	1.24145	3.92412	-0.5843	4.021	0.98747	2.07039	0.8302	0.91946	-0.79623	0.83665	1.67486	1.676
CYSRT1	0.15999	1.61128	0.79123	1.57075	0.6071	0.57681	-0.27554	0.30892	-3.32193	-0.13948	-0.24489	-0.07261	-1.58035	-1.80162	-0.57493	-0.97492
SLC34A3	-3.32193	-1.31257	-1.12239	-1.67707	0.17698	-0.97459	-1.2703	0.19178	-3.32193	-1.76129	-1.15484	-2.60915	-1.81191	-1.36073	-2.12846	-1.22139
FAM166A	6.56385	6.21243	6.22285	5.50169	5.42995	5.795	4.83895	6.01417	5.57549	4.46213	4.86161	2.19969	6.16811	5.78669	4.89553	4.37785
STPG3-AS1	-1.82363	-1.12505	-0.76979	-0.44693	0.54248	0.56947	-2.81005	0.83379	-2.70806	-2.41122	-2.36191	-3.32193	-3.32193	-1.6725	-3.32193	-2.7906
STPG3	-1.36194	-1.06337	0.098	-0.76969	1.18195	0.96419	-2.08982	1.18742	-1.19764	-1.80679	-2.73785	-3.32193	-3.32193	-1.61861	-3.32193	-2.76598
NELFB	5.46407	5.39928	5.54603	4.71805	5.23527	4.91245	4.66033	4.84661	6.03054	5.8172	5.80288	5.28293	5.94438	6.23383	6.5075	6.4338
TOR4A	5.26518	5.68738	5.72585	4.86908	2.83605	4.78298	2.54316	4.32483	5.3919	5.94334	5.82475	4.94088	2.96268	2.62672	3.39189	3.83482
NRARP	-0.40024	-0.09439	-0.46717	-0.28699	1.57847	1.96818	0.72713	1.40901	1.16392	0.53909	0.50315	-0.10258	1.62697	1.74495	1.85464	1.63009
EXD3	0.79788	0.65405	0.83836	0.52223	0.21285	1.21576	-0.89702	1.18834	-0.6345	0.82242	0.41525	-0.03214	0.93952	0.87963	1.58285	1.72593
NOXA1	0.5274	1.18929	1.50581	0.74547	1.90833	3.29676	0.64886	3.18938	0.821	2.59263	1.76813	1.22417	0.90731	2.25028	1.52711	1.96031
ENTPD8	-2.45719	-3.32193	-1.61284	-1.87441	1.30512	2.45828	0.4618	2.1364	-2.92347	-0.73359	-1.49349	-0.95709	-1.32136	0.32611	0.05745	1.45624
NSMF	4.59951	4.65503	4.55453	3.93163	4.13264	4.27072	3.40414	4.32614	3.3713	4.85967	4.568	4.14324	3.35237	4.46869	4.75476	5.09558
PNPLA7	-1.8023	-1.53742	-1.24265	-1.34139	-1.18335	-0.07471	-1.90494	0.04907	-1.20227	-0.45368	-0.12973	-0.05453	-3.32193	-3.23687	-2.7179	-2.33136
MRPL41	7.1109	7.10146	7.07953	6.58934	6.67007	7.66353	5.90489	7.62963	6.18727	5.94897	6.08506	4.52067	6.95162	6.71159	6.70976	6.72337
DPH7	3.20247	2.79342	3.00192	2.75147	2.5086	3.18519	2.21697	3.33697	1.64335	1.52418	1.67368	1.31268	3.24002	3.25757	2.67259	2.53276
ZMYND19	5.40846	4.96885	4.88562	4.23671	4.53752	4.67284	3.98731	4.59866	3.8442	3.38093	3.41165	2.61296	4.83301	4.69903	4.59608	4.21875
ARRDC1	4.384	4.57489	4.62324	3.78852	4.28038	4.92171	3.57436	4.87087	3.11216	3.88847	3.54771	2.65983	3.30546	3.46063	3.75908	3.72703
ARRDC1-AS1	3.782	3.48469	3.69665	3.01864	3.16215	3.48692	2.36125	3.48033	2.07707	1.84006	2.47421	0.57246	2.56262	1.95677	2.16256	2.22314
EHMT1	1.80264	1.55329	1.74901	1.38367	2.08133	1.669	1.70188	1.86398	1.98903	1.7732	2.3375	1.77189	2.18934	2.05394	2.72257	2.46655
TUBBP5	-1.30976	-1.97266	-1.75607	-2.06328	-0.56925	-1.90338	-0.89522	-1.60107	-0.31242	-0.38666	-1.08487	-0.94802	-2.12252	-1.99589	-2.28398	-1.93517
FAM157B	1.25327	1.13823	1.34457	-0.00287	0.89916	0.47201	0.40399	1.39959	-0.68451	0.10404	0.42344	0.17541	-0.02924	-0.26766	-0.36	0.16736
LINC01001	3.43822	3.28372	3.66162	3.31344	3.01662	3.55063	2.18691	4.0608	-0.93029	0.13541	-0.11997	0.47452	0.11914	0.51726	-0.87355	0.68317
BET1L	4.30333	4.26627	4.23694	4.05004	4.86419	4.82683	4.34141	4.49249	3.97083	4.33237	4.48298	4.22228	3.05682	3.30136	3.24887	3.12035
RIC8A	5.40661	5.50366	5.50922	5.05094	5.55705	4.66855	4.93117	4.43943	5.55235	5.31668	5.49099	4.88466	4.72667	4.39539	4.69515	4.51269
SIRT3	2.84536	2.74731	2.97446	2.75056	2.53517	2.51769	1.69309	2.85827	2.70411	2.98669	2.35703	2.55699	1.83179	2.09802	1.77357	2.25508
PSMD13	6.2867	6.22857	6.00701	6.12125	5.35269	5.50572	5.43853	5.48773	5.76739	5.62682	5.71896	5.41978	5.08921	5.22213	4.80747	4.8993
PGGHG	1.82892	1.93594	2.25319	1.60552	4.01949	4.58493	2.16345	3.98731	2.34791	2.92568	3.04656	2.04561	-0.15584	0.10978	-1.28657	-0.66026
IFITM2	1.35878	1.40083	1.80599	1.65439	3.53784	6.23099	2.92832	5.50806	8.78995	8.63689	8.52816	7.2454	5.5471	5.17889	4.37838	4.45246
IFITM1	-0.28871	0.29776	0.05174	-0.44411	2.16831	4.23618	2.0676	3.48693	6.38452	6.18022	6.18505	4.17674	3.93022	3.69451	2.88221	3.07815
B4GALNT4	-1.8073	-0.09877	0.37624	-1.5801	2.90463	4.04617	1.76294	3.9759	3.43589	4.48588	4.22485	3.67765	3.08861	3.67922	3.98569	4.5183
PKP3	1.72121	2.36111	2.80142	1.72602	1.66167	0.21012	1.72017	-0.89226	5.22699	5.71833	5.69539	4.62396	0.88759	1.33101	1.34816	1.97083
SIGIRR	3.80021	4.26766	4.36377	4.12266	3.76771	4.97635	2.88238	4.84282	3.01322	3.97522	2.96852	2.70464	2.81904	3.12033	2.88996	2.97377
ANO9	0.77654	2.53905	2.20269	2.34712	-1.03604	-0.88235	-1.68924	-0.46467	-2.9538	-1.71322	-2.12323	-2.76205	-3.06381	-1.34802	-2.03944	-1.70164
PTDSS2	5.07715	4.97109	4.93353	4.51976	5.08657	4.98836	4.3755	4.94911	4.19346	4.06823	4.29509	3.86704	2.8838	2.44679	3.0479	3.01639
RNH1	6.09009	6.16012	6.27584	5.77371	5.75513	5.83533	5.00046	5.76237	5.95286	5.73036	5.92027	5.06547	4.83862	4.71979	5.08382	4.99312
HRAS	6.42558	6.25826	6.22955	5.49924	5.6964	5.66813	5.07824	5.71233	6.55923	5.57693	6.19237	4.74885	5.48334	5.14407	5.64212	5.2421
LRRC56	-1.75534	-1.06637	-1.0044	-0.9307	1.56412	2.13998	0.52038	2.01216	0.01754	1.55482	0.85848	0.31332	-0.67345	-1.75184	-1.34239	-0.25409

LMNTD2	-0.44445	0.69084	1.36501	0.73943	2.35666	3.33669	0.70567	3.44934	0.27132	2.00099	1.89423	0.85219	-2.02876	-0.28148	-0.2215	-0.76007
RASSF7	4.5191	4.47428	4.90271	3.76818	6.0225	5.73482	4.98512	5.59376	5.04627	5.0092	4.9725	3.60942	4.19777	3.69072	4.31927	3.8434
MIR210HG	2.41048	2.70443	3.89499	2.4492	1.148	3.08487	0.13597	2.64726	2.09048	2.49727	2.05414	1.00145	-0.13272	-1.46978	-0.95725	-1.60637
PHRF1	5.86474	5.70125	5.72052	4.8481	5.4791	5.02962	4.71744	5.20943	5.30288	4.92746	5.28014	4.36691	4.17554	4.26723	4.97574	4.99893
IRF7	2.48365	3.37515	3.42913	3.29729	-0.27321	-0.86802	-1.64144	1.20975	2.68944	4.00431	3.72518	4.03758	1.07922	1.2663	1.65316	1.99664
CDHR5	-0.05636	-0.49963	0.16461	-2.73125	0.15417	-0.67395	-1.02088	0.95255	-0.6535	-0.43892	-0.74124	-1.78602	-0.84871	0.23805	-0.05745	0.59264
DRD4	2.36304	2.75801	2.03007	0.70108	0.9486	2.23946	-0.64488	3.6696	0.70218	1.05392	0.24939	-0.32662	0.00816	0.41208	0.20609	1.75781
DEAF1	4.12338	4.17023	4.1165	3.66041	3.343	3.33794	2.57881	3.31758	4.13979	4.41266	4.30369	3.77276	3.13972	3.44878	3.3176	3.4756
EPS8L2	4.41457	4.65329	5.24573	4.73075	5.06144	5.27047	4.16929	5.02658	6.06547	5.8411	6.30617	5.58756	3.81892	3.58089	3.66522	3.44309
TMEM80	2.09598	1.80831	2.10469	1.79469	2.98725	2.69175	2.76366	2.60558	1.25246	1.75375	1.86203	1.41489	0.94273	0.56957	0.58645	0.98481
TALDO1	7.59444	7.59954	7.52833	7.60404	6.59503	6.44737	6.4315	6.38005	6.40938	5.95802	6.20631	5.69792	6.26848	6.14049	6.01913	6.10923
GATD1	4.47435	4.09555	4.43365	3.39995	4.02149	3.14708	3.25206	3.31486	3.86202	3.6518	3.86399	2.94697	3.35632	2.70278	3.29421	2.76137
CEND1	-1.38685	0.20982	0.21344	0.76913	-0.73761	-1.0568	-2.10904	-0.48239	-1.64914	-2.08218	-2.02167	-3.32193	-2.86909	-1.48523	-1.6229	-2.38187
SLC25A22	6.16692	5.90312	5.58388	5.304	3.9362	4.40654	3.03132	4.31221	4.89169	4.80457	4.51642	3.8822	3.5668	3.79639	2.93101	3.60417
PANO1	-0.25297	0.63401	0.55413	0.34073	1.51928	0.94235	0.52287	1.23442	1.26845	1.16241	1.07726	0.3088	1.74731	1.8759	1.65233	2.13187
PIDD1	3.43273	3.70191	3.82624	3.2144	3.15848	3.20874	1.86989	3.48601	3.29492	2.76983	3.24132	1.98136	1.22514	1.7887	1.35728	1.58669
SNORA52	5.46332	4.81013	5.21545	4.76307	3.44935	4.04413	3.87749	4.85966	2.45572	1.53236	2.60883	0.26081	4.15352	2.46615	-0.61487	-3.32193
PNPLA2	7.0373	6.94682	7.04289	6.34657	5.36194	6.35471	4.1524	6.23982	5.26079	5.01222	5.20803	4.20637	3.67327	3.50546	3.86789	3.8027
CRACR2B	3.59455	4.09099	4.17977	3.61399	2.29571	3.56702	1.23802	3.55798	-1.32297	0.2512	-1.15424	-0.76396	-1.88402	-1.18234	-1.78902	-0.98145
POLR2L	8.43643	8.30993	8.51999	8.07779	8.63301	8.39986	8.14613	8.09231	8.76739	8.20717	8.54505	7.26634	8.33357	7.94935	7.22745	7.04598
TSPAN4	5.01386	4.93541	5.17274	4.77742	5.57986	6.36364	5.12425	6.1609	5.38493	5.57128	5.55746	4.83505	4.17863	4.26586	4.14843	4.24045
CHID1	5.25179	4.93742	4.88539	4.32735	4.80224	4.56469	4.06476	4.43327	4.67086	4.42789	4.6359	3.86996	3.23537	3.14862	3.57152	3.67034
AP2A2	2.92545	3.01774	3.12347	3.08694	3.98385	3.03583	3.77349	2.86273	4.21689	4.0859	4.1761	4.61505	3.46705	3.71149	4.31005	4.24549
MUC6	-2.97	-2.53657	-2.59545	-2.66085	-2.20203	-2.30458	-2.64231	-1.55229	-2.92017	-2.88342	-2.66897	-2.288	-2.47908	-1.91408	-2.9842	-2.0905
MUC2	-3.32193	-3.32193	-3.32193	-3.32193	1.96511	1.41733	1.68316	0.78672	-3.32193	-3.16079	-3.14951	-3.09018	-3.32193	-3.32193	-3.23991	-3.32193
MUC5B	-3.15175	-3.25589	-3.18589	-3.17741	-2.53394	-0.40737	-2.84732	-0.95193	-3.32193	-3.1066	-3.32193	-3.10999	-3.1035	-2.84588	-3.21132	-2.46237
TOLLIP	4.26947	4.5109	4.43093	3.92276	5.0636	4.99515	4.60679	4.86252	4.18418	4.78893	4.51334	4.06722	3.69553	4.16453	3.92576	4.3283
TOLLIP-AS1	0.33017	0.57683	0.55671	-0.09736	0.7177	1.47208	0.41624	1.51231	-0.78496	0.61791	0.63748	-0.17861	0.26401	0.00478	-0.3036	-0.58941
BRSK2	-2.67838	-2.40862	-2.47457	-2.43315	3.18451	3.39252	2.35518	3.70541	1.23132	2.31266	1.38082	1.27713	1.7743	2.81069	2.51182	3.24336
MOB2	3.25971	3.38255	3.45442	2.91198	3.21345	3.24399	2.71605	3.09588	2.87535	3.1631	3.17717	2.77631	2.43909	2.4799	2.07236	2.45637
DUSP8	0.8055	1.57888	1.61731	0.84643	3.43161	3.73984	3.14692	3.56304	-0.67191	0.95018	0.21722	0.26281	0.47601	2.21047	2.21505	2.17955
KRTAP5-AS1	-3.32193	-2.94652	-3.32193	-3.32193	-0.26709	-0.78113	-0.81191	-1.06509	-2.9348	-3.32193	-3.32193	-3.32193	-3.32193	-1.91393	-2.73156	-1.48033
KRTAP5-1	-3.32193	-3.32193	-3.32193	-3.32193	0.68225	1.91278	1.35672	1.64979	-3.32193	-3.32193	-3.32193	-3.32193	-3.32193	-3.32193	-3.32193	-3.32193
IFITM10	0.72895	1.33446	1.39892	2.29054	0.64855	1.10458	-0.32807	1.26077	-0.16488	0.5926	0.17016	0.17687	-0.6871	-0.91432	-0.57111	-0.29969
MRPL23	5.14024	5.00734	4.99284	4.58159	5.06918	5.81665	4.61043	5.77296	5.08626	4.72597	4.79988	4.27359	4.6819	4.53949	4.4459	4.31148
H19	-2.69016	-1.52828	-1.34764	-2.89237	1.87595	8.36407	1.79055	8.07653	-1.77792	-2.2795	-1.7407	-2.1189	-2.26801	-1.99927	-2.11309	-2.27062
MIR675	0.21808	-3.32193	0.44315	0.52823	4.10923	10.5504	4.45504	10.2456	-3.32193	0.17021	0.2647	1.08119	-3.32193	0.8155	-3.32193	-3.32193
IGF2	-2.15842	-1.63179	-2.01796	-1.96275	-0.59183	-1.73409	-0.39638	-1.70612	0.9597	1.60479	1.13346	1.93599	-3.32193	-2.95411	-2.36059	-0.58049
INS-IGF2	-2.17075	-2.07679	-3.01033	-2.49763	-0.63381	-2.03492	-0.53776	-1.65366	0.64754	1.17537	0.40345	1.99884	-3.32193	-2.9239	-3.06578	-1.06702
TH	-2.54123	-2.70159	-3.32193	-3.32193	-2.82975	-0.23427	-3.32193	-1.60946	-1.40286	-0.42241	-0.20331	-1.42565	-3.32193	-3.32193	-3.32193	-3.01912
ASCL2	-3.32193	-3.32193	-3.32193	-3.32193	-2.15465	1.95272	-2.20188	1.86353	-3.32193	-3.32193	-3.32193	-3.32193	-3.32193	-2.11493	-3.32193	-2.8273
CD81-AS1	4.99138	5.06067	5.17696	4.21645	4.6502	4.70904	3.76382	4.40005	5.25718	5.13314	5.30064	4.95064	3.08057	3.06874	4.31807	4.40069
RPL26P30	1.51983	1.74045	1.69432	0.94324	0.40763	1.81004	0.5339	1.34087	0.83056	1.40747	-0.4674	-0.577	-0.58724	-1.79653	-0.56828	-1.57842
CD81	5.62499	5.59951	5.75312	5.46595	5.19648	5.88553	4.59127	5.65073	5.71599	5.50801	5.6028	5.25668	4.50029	4.42205	4.53106	4.74439
TSSC4	5.59975	5.4454	5.2957	4.80512	5.06271	5.12614	4.30733	4.99709	4.85152	4.55913	4.86881	3.32678	4.10088	3.66421	4.37116	4.25326
KCNQ1OT1	-2.2166	-2.08379	-2.48811	-1.11967	-2.25874	-1.67111	-2.45646	-0.91866	-3.06271	-2.95931	-3.0051	-2.70109	-2.85234	-2.68483	-2.92658	-2.67868
CDKN1C	-0.08873	1.19509	1.20445	-0.28654	-1.4944	-1.42236	-2.68011	-1.12983	2.87894	3.12026	3.3599	2.54775	-1.07036	-1.22431	0.17831	0.44295
SLC22A18AS	1.6143	2.95882	2.62962	3.26481	4.64903	4.90902	4.19481	4.40135	2.66148	3.98636	3.75491	3.6423	0.73947	1.53004	1.60993	2.1899
SLC22A18	1.74034	3.03764	3.21482	3.37889	4.99785	5.95063	4.20502	5.64806	3.25072	4.49898	4.20404	4.25363	0.13105	1.79166	1.57117	2.33859
PHLDA2	7.97469	8.34679	7.88317	7.91099	6.43219	6.00985	5.8366	5.98892	6.77713	6.68536	5.43313	5.92257	6.89753	7.10876	6.04386	7.2738

NAP1L4	5.46076	5.31238	5.16335	5.19883	5.09231	4.62366	5.20206	4.63086	5.21824	5.04004	4.93866	5.07728	5.2842	5.34713	5.21585	5.11896
CARS-AS1	3.95051	3.98022	4.06066	3.89625	3.74869	2.6688	4.15488	2.09193	4.7055	4.6841	4.65605	4.85953	2.65234	2.52808	2.76792	2.77542
OSBPL5	0.96376	1.04278	1.26486	0.66259	-2.16088	-1.12861	-2.34348	-1.04902	2.99825	3.9835	3.53219	3.46837	-0.27785	-0.1596	0.19172	0.59463
ZNF195	2.55192	2.60441	2.3301	2.98969	1.56743	2.48493	2.2111	2.96287	1.01994	1.55813	1.35871	2.1969	1.9993	2.47098	2.05748	2.54378
TSSC2	3.50357	2.98942	2.90671	2.53422	-2.26607	-2.60634	-3.32193	-2.73349	-3.00453	-3.32193	-2.79723	-3.32193	-3.32193	-2.58982	-3.32193	-3.32193
FAM86GP	3.7234	3.04683	2.82933	2.21433	-1.07883	-0.42175	-1.32756	-0.79749	-1.40079	-1.5941	-2.45164	-0.85038	-1.27545	-2.62241	-1.98492	-3.32193
CHRNA10	-0.29012	-0.55617	-1.42609	-1.11255	-0.99302	0.30126	-0.99415	0.90758	-1.32057	-1.76701	-0.65423	-1.42585	-0.54611	-0.70008	-1.22106	-0.59858
NUP98	4.13018	3.94678	3.78387	3.92268	4.53472	2.98395	4.61986	2.98027	4.58709	4.02679	4.32568	4.57893	4.32204	4.08856	4.27529	4.20807
PGAP2	1.97766	2.12509	1.83898	2.03232	3.90678	3.43971	3.32832	3.33424	2.68855	2.896	3.07651	3.16567	2.40004	2.10376	2.61734	2.4928
RHOG	5.89076	5.98431	5.94136	5.83305	5.1574	4.76691	4.59257	4.8762	6.37808	6.14277	6.19005	6.22399	4.82017	4.74543	4.66811	4.45589
STIM1	3.10092	2.75934	2.74198	2.66105	4.98149	3.95904	4.83697	3.7326	3.93625	3.73728	3.90435	3.83578	3.37518	3.08381	3.85634	3.73031
MIR4687	4.96092	5.17128	4.72984	4.2998	7.97641	5.06395	7.72796	3.22207	6.80398	6.96027	6.84136	6.20312	5.71543	5.77157	6.98865	6.70378
RRM1	5.87493	5.7076	5.60012	5.68368	5.34811	3.59748	5.73497	3.94424	5.81775	4.84639	5.13552	4.80743	5.71156	5.79916	5.33964	5.17073
RRM1-AS1	7.13693	6.9597	6.79334	6.93915	5.90972	4.80714	6.31967	5.23721	6.33389	5.0808	5.70464	5.32473	6.36049	6.44216	5.86141	5.74766
TRIM21	4.58492	4.37733	4.55777	4.46882	3.23724	2.46216	3.23481	2.30257	4.83462	4.30245	4.92211	4.35797	3.51695	2.73647	2.67804	2.76017
TRIM68	1.07854	0.88719	0.94503	1.17046	1.1635	0.21773	0.52264	0.2624	2.17095	1.41645	1.40796	1.77965	1.78708	1.5084	1.68478	1.53339
HBG2	3.96767	3.59064	3.36548	3.29507	-2.33957	-1.97227	-2.54388	-2.7805	-1.84673	-2.24628	-1.95087	-2.25916	-2.64391	-2.94464	-3.32193	-2.00312
HBE1	6.22951	6.24515	6.09106	6.0113	-2.5476	-3.32193	-3.32193	-3.32193	-3.32193	-1.54817	-1.47426	-1.56553	-3.32193	-3.32193	-3.32193	-3.32193
OR51B4	2.56633	2.13584	1.61191	1.26921	-3.32193	-3.32193	-3.32193	-3.32193	-3.32193	-1.55365	-3.32193	-3.32193	-3.32193	-3.32193	-3.32193	-3.32193
OR51B5	4.41106	3.76744	3.55015	3.59953	-3.32193	-3.32193	-3.32193	-2.10002	-2.33885	-2.46573	-2.21049	-2.47669	-3.32193	-3.32193	-3.32193	-2.69278
OR51J1	2.29721	0.9501	1.15517	2.52768	-3.32193	-3.32193	-3.32193	-3.32193	-3.32193	-3.32193	-3.32193	-3.32193	-3.32193	-3.32193	-3.32193	-3.32193
TRIM6	2.96861	2.93093	3.22832	3.12328	4.07131	3.10588	4.61108	2.89151	3.54696	3.18392	3.08619	3.24717	2.84525	3.06057	2.17372	2.54535
TRIM6-TRIM34	2.73742	2.69397	2.76501	2.65812	3.83811	2.27618	4.12497	1.87428	3.17614	3.06253	3.20608	3.14824	2.1962	2.83222	2.01198	2.54048
TRIM34	2.21095	2.26172	2.04734	2.2626	2.24986	1.33413	2.51142	1.1993	1.51096	1.96776	2.17746	2.59343	0.32513	1.83533	1.09656	1.55764
TRIM5	3.17796	3.08056	2.77343	3.35562	3.30314	2.40891	3.38268	2.34394	3.27178	3.06295	2.75009	3.57575	3.12212	2.82001	2.2551	2.86888
TRIM22	-0.64052	0.13549	-0.58828	0.89355	-3.03621	-3.32193	-3.15799	-2.77593	1.58121	1.96962	2.12448	2.94753	-3.18311	-3.32193	-3.32193	-3.15096
FAM160A2	3.23392	3.13452	3.04853	2.91206	3.74645	3.54824	3.44215	3.4121	2.76256	2.68281	3.0081	2.7745	2.47748	2.63871	2.83924	3.09759
CAVIN3	-1.2347	-1.10562	-0.88911	-0.97088	-2.07097	-2.76677	-3.32193	-2.6877	7.20462	7.1133	7.26658	6.51428	0.9122	0.93494	0.68421	1.47124
SMPD1	3.7971	4.15185	4.49413	4.4127	4.63666	4.75077	4.39711	4.90572	4.64945	4.77449	5.14403	5.17654	3.51913	4.00458	4.4421	4.72558
APBB1	-1.84891	-1.45092	-1.86071	-1.62502	2.33897	2.03927	1.48942	1.87023	4.49726	4.80153	4.53257	4.68086	2.72096	3.67777	3.80489	3.93353
HPX	-2.80844	-3.01169	-2.5066	-2.97729	2.8961	4.21719	2.20451	4.30766	-3.00179	-3.32193	-2.40681	-1.93178	-2.73469	-2.58429	-3.32193	-2.29128
TRIM3	2.4421	2.52724	2.71877	2.68104	1.99336	1.88883	1.62948	1.80933	1.91128	1.89728	2.32043	1.84736	1.75249	1.55823	1.71022	1.60376
ARFIP2	4.69665	4.74933	4.69633	4.68892	3.97155	3.70168	3.55255	3.75101	4.12288	4.1223	4.20038	4.04918	3.33851	3.20904	3.06799	2.70717
TIMM10B	3.82661	3.7215	3.57194	3.96917	3.44858	3.21512	3.464	3.07859	3.29288	2.8989	3.00095	3.82592	3.36698	3.5601	2.78626	3.07784
DNHD1	0.22351	0.23141	0.73108	0.40915	-0.38333	0.72344	-1.48054	0.45394	-1.29362	-0.51745	-0.80833	-0.43253	-1.27627	-0.64351	-1.18523	-0.41102
RRP8	2.40974	2.04954	1.74609	1.71579	2.08667	1.67063	2.12769	1.45447	2.40246	2.27049	2.18912	2.06407	1.82869	1.88992	1.97933	1.8309
ILK	6.09735	6.29252	6.36022	6.21666	6.29851	5.65713	6.35329	5.49645	6.76108	6.77315	6.7188	6.8561	5.95142	5.90626	6.02499	5.86262
TAF10	6.1165	6.33634	6.38589	6.19333	6.094	6.03048	5.92502	5.93101	6.42869	6.40308	6.39264	6.22328	5.39074	5.37942	5.43305	5.29522
TPP1	6.0085	6.29989	6.43299	6.71445	5.69749	5.505	5.53504	5.26134	4.63774	5.03201	4.85336	5.90652	4.23618	4.4162	4.71627	5.16865
DCHS1	-2.1677	-2.53258	-2.3662	-2.25692	1.41133	0.22843	0.78399	0.20759	3.286	4.29542	3.97701	4.5685	-2.62477	-1.72446	-1.0022	-0.10438
MRPL17	6.3541	6.3421	6.08941	6.09175	5.93174	6.04637	5.48716	5.73093	6.0551	5.99609	5.85267	5.01624	5.1776	4.97047	4.47501	4.26811
OR10A2	-2.66569	-2.12473	-3.09469	-1.28976	-3.00623	-0.58253	-3.32193	-0.09049	-3.32193	-3.32193	-3.32193	-3.13732	-3.32193	-3.32193	-3.32193	-3.32193
ZNF215	1.61974	1.12685	1.00907	1.0378	1.07024	0.40513	1.41241	0.79257	-0.59302	-0.46853	-0.48759	0.11374	-1.50245	-0.60688	-1.00526	-0.5896
ZNF214	0.17173	-0.06394	0.25252	0.33667	-0.81235	-0.72361	-0.53618	-0.73493	-1.67053	-1.72248	-1.05381	-0.56953	-1.0858	-0.28794	-0.45763	-0.33381
SYT9	-1.46314	-1.91127	-1.58458	-2.4508	-3.22468	-3.10035	-3.32193	-3.32193	0.3811	0.90555	0.20733	0.55554	-3.32193	-3.32193	-3.32193	-3.32193
OLFML1	-3.32193	-3.32193	-3.32193	-3.32193	-1.51695	-0.1169	-2.01397	0.65777	-3.32193	-3.03763	-2.0565	-2.80766	-3.32193	-3.32193	-3.32193	-3.32193
PPFIBP2	-2.83288	-2.53562	-2.9581	-2.38314	1.45734	0.42252	1.41619	0.32408	-0.91184	-1.17653	-1.2309	-0.34272	-3.23348	-2.79486	-3.11516	-2.54895
CYB5R2	-2.69738	-2.17391	-2.51559	-2.38696	-1.21835	-2.13414	-0.72338	-2.51315	1.76802	1.94253	1.39253	1.87211	-1.11796	-0.57581	-1.78763	-0.61024
EIF3F	4.95734	4.83865	4.74124	4.65533	3.82845	3.72664	3.68733	3.72957	5.65961	5.41128	5.48809	5.45889	4.58727	4.56384	4.32264	4.23542
TUB	-2.56255	-2.20667	-2.69231	-2.20686	0.63819	-0.47651	0.15456	-0.59281	2.28223	2.22527	2.17427	2.08318	1.5648	1.5376	1.34267	1.43443

LMO1	1.46656	1.65131	1.31842	1.12902	-3.32193	-3.32193	-1.17481	-3.32193	-3.32193	-3.32193	-3.32193	-2.41833	-3.32193	-3.32193	-3.32193	-3.32193
STK33	1.5353	1.20944	1.08455	1.63421	-3.0911	-3.32193	-3.07015	-2.75057	-3.32193	-3.32193	-3.32193	-3.32193	-1.59056	-1.66534	-1.24539	-1.51798
TRIM66	-0.97422	-1.13804	-0.81714	-0.76326	-0.75219	-0.35479	-1.24637	-0.55561	-0.30586	0.33625	0.43171	1.14325	-0.24802	-0.48162	-0.18277	-0.11046
SNORA3A	4.43247	3.34361	4.31501	4.2435	3.64665	3.45487	3.41796	4.03846	-0.33289	1.57459	2.91105	3.19464	2.62769	2.91778	1.25097	2.41777
SNORA3B	4.46081	4.00535	3.8215	3.98243	2.83701	3.66441	2.75196	4.25748	1.82507	0.31338	2.90014	1.54015	2.38007	1.27051	1.24038	-0.5603
AKIP1	4.19816	4.06411	4.29639	4.1624	4.12085	3.44829	3.76355	3.4669	4.43257	4.08645	4.14147	3.40933	3.92302	3.86412	3.18918	3.31412
TMEM9B	3.59772	3.68543	3.74353	3.96133	4.38583	4.1157	4.83273	3.91684	4.30078	4.40793	4.41649	4.79009	3.68704	4.16319	3.66124	3.83929
TMEM9B-AS1	0.92857	1.13691	1.77545	1.02808	1.91047	1.37777	1.50753	1.21686	1.02921	1.73134	1.8034	0.90528	0.32329	0.54166	0.70529	1.85587
NRIP3	-3.32193	-3.32193	-3.03149	-2.54548	0.05016	-0.84481	-0.19572	-0.8683	-0.4451	-1.73854	-0.8664	-1.13004	1.06412	0.50847	0.76515	0.77951
DENND5A	4.42897	4.64564	4.50152	4.79056	3.64285	2.68359	3.8345	2.69673	4.29101	4.35618	4.21227	4.66253	3.71229	3.94494	4.1547	3.81893
TMEM41B	3.3394	3.45346	3.27277	3.85597	4.615	4.49154	5.2502	4.31399	3.30037	3.33664	2.70449	4.10889	3.99211	4.37774	3.42278	3.51583
ZNF143	2.89058	2.79762	2.8616	3.18648	2.58182	2.22617	2.99508	2.19239	2.75474	2.5255	2.54031	3.02638	2.66965	2.49547	2.37199	2.19881
WEE1	3.23771	3.26083	3.28005	3.31957	3.39182	2.54846	3.986	2.48681	3.11655	2.77734	2.79314	2.88804	3.75058	4.38234	3.81552	3.6223
RPL23AP65	2.88692	2.40921	2.84257	0.82768	1.43615	2.21014	0.85024	1.41372	2.45874	4.36243	2.48479	3.6995	-0.41579	0.60791	1	0.47994
SWAP70	3.92409	4.11104	4.307	4.25416	3.93781	2.51068	4.62264	2.48747	4.41223	3.8048	4.34546	3.97976	3.54979	3.5888	3.9673	3.12727
SBF2-AS1	1.91413	2.23261	2.05173	2.21961	2.13802	1.59466	2.45734	1.72387	2.67944	2.86937	2.77659	3.31892	2.40133	2.43743	2.57301	2.33126
SBF2	1.23811	1.24514	1.15559	1.66881	1.54163	0.68016	1.8889	0.83124	2.45569	2.52603	2.32213	3.16485	2.19832	2.28385	2.41483	2.21123
ADM	8.19849	8.50791	8.80377	8.2083	3.83684	5.78623	3.84399	5.27867	6.4728	7.32315	6.41586	6.42977	5.8963	5.39409	5.00724	4.28086
AMPD3	3.70162	4.24615	4.40154	4.02145	-1.69595	-1.05526	-0.84014	-1.92647	3.49595	3.70547	3.94854	3.59833	-1.62695	-1.75527	-2.25785	-0.96428
MTRNR2L8	2.19135	2.21604	2.3534	0.87142	1.24899	4.43295	0.99305	4.46351	1.65367	2.4592	0.34754	2.07795	1.8665	2.28963	2.4601	3.81254
MIR4485	2.47157	2.63532	3.02957	3.12011	1.83581	5.3228	1.96826	5.55662	3.48269	3.86225	2.11491	4.07728	2.44192	2.69545	2.98186	4.11884
RNF141	1.7336	1.59919	1.71136	2.19705	2.3128	1.66019	2.65484	1.37995	2.50971	2.98009	2.23768	3.22912	2.18014	2.53083	2.07467	2.21788
LYVE1	-3.32193	-3.32193	-3.32193	-2.68271	-0.83779	-0.87172	1.25567	-2.71288	-3.32193	-3.32193	-3.32193	-3.32193	-3.32193	-3.32193	-3.32193	-3.32193
CTR9	4.92771	4.86945	4.70438	5.304	4.33431	3.52175	4.77341	3.54655	5.1328	4.82153	4.82874	5.39982	4.32086	4.00817	4.41253	4.33815
ZBED5	1.51131	1.4762	1.91309	2.20268	1.6596	1.39933	2.10044	1.61105	2.23146	2.47786	2.30573	3.28514	1.7278	1.5318	1.37568	1.15203
ZBED5-AS1	0.76132	1.27158	1.54175	1.9927	0.78173	0.96251	1.11763	1.12581	2.17999	2.19036	1.85048	2.6374	2.14661	1.24957	0.88418	0.99871
GALNT18	4.55651	4.35861	4.43281	3.62493	-0.24261	-1.23606	-0.32356	-1.49608	4.56555	4.74865	4.54831	4.67458	0.84252	0.91638	0.84455	0.84112
CSNK2A3	1.19688	0.71193	1.49942	0.42812	0.19853	-0.38645	0.10043	-1.75839	1.79768	1.77812	1.4393	1.73185	0.89573	1.52666	1.06251	0.81404
USP47	3.1738	3.21916	3.01968	3.50553	3.31199	2.58677	4.00416	2.51539	3.72664	3.67547	3.61032	4.20975	3.56001	3.45558	3.68973	3.61793
DKK3	2.3894	2.57399	2.50584	2.48049	4.6391	3.2643	4.92676	2.92938	5.89566	6.0194	6.20668	5.67126	2.2151	2.26141	2.88069	2.34636
LINC02547	0.47551	1.10704	0.3732	-0.41196	-3.32193	-3.32193	-3.32193	-3.32193	-2.69393	-2.7834	-3.00804	-2.40379	-2.87027	-3.32193	-3.32193	-3.32193
MICAL2	4.00234	3.1821	3.61541	2.74425	0.57196	-1.94987	1.34105	-2.05804	3.87521	2.52656	3.56783	3.1441	5.23663	3.5381	5.48745	3.46059
PARVA	3.31114	3.43471	3.28302	3.6793	0.87714	0.24859	0.98403	0.23832	4.23829	3.73742	4.38162	4.38499	3.15573	2.68393	3.57747	2.71285
TEAD1	2.97351	3.00735	2.96878	3.20954	3.17793	1.45893	3.35445	1.70143	4.49113	3.89675	3.94994	4.57114	4.68544	4.38955	4.67062	4.38345
RASSF10	-3.32193	-3.32193	-3.32193	-3.32193	1.85508	-0.00076	1.81047	-0.33414	4.23966	4.54359	4.55589	4.79324	-1.04535	0.88609	0.74982	0.59169
ARNTL	1.73594	1.75009	1.85879	1.93152	0.48912	0.06353	0.75134	-0.42457	1.77666	1.8366	1.87272	2.25758	-0.06069	0.29394	0.03622	0.26728
BTBD10	4.39664	4.11046	4.20454	4.26024	3.13833	2.02648	3.67882	1.75497	4.56004	3.9338	4.09402	3.63404	4.25007	3.48473	3.69469	3.07036
FAR1	-1.17968	-0.94875	-1.21434	-0.22164	3.32074	2.53382	4.08023	2.61015	2.77801	2.70609	2.89575	3.15833	-3.32193	-3.32193	-3.32193	-3.32193
SPON1	-1.15239	-1.29571	-1.58084	-0.69315	-2.39252	-2.77456	-2.43286	-1.92774	-1.85299	-2.10767	-1.16265	-2.21512	-1.56341	-1.46892	-1.94474	-1.38815
RRAS2	5.30138	5.05417	5.0274	4.85443	3.94953	3.10423	4.47746	2.73911	5.75832	5.5393	5.56724	4.71746	5.23697	5.00941	4.90966	4.26996
COPB1	5.49313	5.77123	5.85218	6.34573	6.34121	5.39247	7.01543	5.11374	5.7802	5.82178	5.78727	6.46602	5.44829	4.86472	5.14549	4.6251
PSMA1	7.08369	6.89336	6.99822	7.13648	5.76053	5.35713	6.01006	5.21217	6.11586	5.45102	5.91955	5.80737	5.89446	4.90418	4.83044	4.05682
PDE3B	-3.06023	-2.94604	-3.01669	-3.09865	2.85381	1.56991	3.02038	1.40002	-0.46558	-0.42338	-0.78402	-0.60089	1.46697	1.44016	2.00193	1.44712
CYP2R1	1.75076	2.11893	1.81582	1.99404	2.40315	2.70307	2.55069	2.85071	0.95443	0.98016	1.22851	1.61598	2.1091	1.52463	1.97201	1.05589
SOX6	-2.02184	-1.99518	-2.36845	-2.09098	-0.69267	-2.10856	-0.26153	-1.57751	-2.64633	-2.63502	-2.36457	-2.54631	-1.65855	-0.65792	-1.26192	-0.88973
C11orf58	5.11151	5.00959	4.95109	5.13668	4.77994	3.93771	5.17166	3.76141	5.02899	4.89323	4.92676	4.70056	4.62244	4.43974	4.69562	4.29182
PLEKHA7	1.80935	1.93002	1.92881	1.85697	2.44763	1.51084	2.22124	1.67822	-2.01278	-1.68425	-1.89803	-1.15771	0.82931	1.26936	1.27504	1.37981
OR7E14P	2.40923	2.01518	2.50351	2.24749	2.78344	2.42482	2.45781	1.97847	-2.59206	-2.25665	-3.32193	-2.26944	0.87255	1.36544	2.29684	1.94834
SNORD14A	5.3286	4.26645	5.01942	5.11077	3.54734	4.49316	3.92848	5.75126	0.12612	2.07456	2.65184	3.03303	2.76165	2.60694	0.78958	1.37678
SNORD14B	4.6501	4.35235	4.98965	5.17093	3.09148	4.05885	3.60578	4.29191	0.1406	1.77664	2.19116	3.18469	3.61597	2.45562	1.36177	0.83438

PIK3C2A	2.56315	2.50212	2.71449	2.99591	3.76759	1.96125	4.31669	2.15473	3.04695	2.40489	2.57144	3.28875	3.50969	2.45712	3.3859	2.52644
NUCB2	3.18714	3.43444	3.43876	4.24615	3.97839	5.36695	4.44717	5.38746	3.54147	3.59866	2.94107	3.90642	2.64883	2.76452	2.61399	2.88757
NCR3LG1	-1.27425	-1.7887	-1.27157	-1.47908	2.99909	0.563	2.92281	0.46063	-0.79479	-0.82369	-1.32292	-1.03338	1.68557	1.56144	1.1265	1.67537
KCNJ11	-3.00899	-2.46144	-3.32193	-3.32193	1.65786	3.53743	1.23947	2.73748	-1.62037	-1.48623	-1.61774	-1.60105	-0.79979	0.30403	0.57303	0.57985
USH1C	-3.32193	-3.32193	-3.32193	-3.32193	2.31219	2.42344	1.87195	2.27609	-2.24897	-1.985	-1.46466	-2.64374	-3.32193	-3.32193	-3.11891	-3.11473
SERGEF	2.65152	2.90934	3.06534	2.83802	2.40224	2.7073	2.25559	2.5013	2.67481	2.91401	2.81424	2.4031	1.55947	2.17366	2.31228	1.94978
SAAL1	4.00697	3.44771	3.52497	3.43181	3.25364	2.22508	3.45664	2.33095	4.10453	3.54592	3.77252	3.35581	3.46889	3.10532	3.17483	2.64224
SAA4	1.81403	2.29071	1.86448	2.90926	5.94016	4.2955	5.34547	4.25902	-0.24377	-2.30084	-1.63852	-3.32193	-3.32193	-3.32193	-3.32193	-3.32193
SAA2-SAA4	4.98872	5.41083	4.57656	5.26084	5.912	4.26425	5.30705	4.22778	2.70821	0.93592	1.69516	-0.74873	-3.32193	-3.32193	-3.32193	-3.32193
SAA2	3.74555	4.06726	3.10524	3.96221	-1.28341	-3.32193	-1.23342	-2.82162	1.45052	-0.50448	0.32802	-1.45678	-3.32193	-2.97477	-3.32193	-3.32193
ST13P5	0.10955	-1.96039	0.23496	-2.40914	-1.04084	-1.38541	0.19443	-0.95505	0.78698	0.83642	0.3322	-0.27033	-0.62643	-0.89791	-1.20495	-0.97313
SAA1	6.95273	6.91245	6.01445	6.61585	-0.80096	-2.30346	-1.78885	-3.32193	4.28967	1.23274	2.50342	0.62542	-2.47809	-2.48037	-3.32193	-3.32193
RNA5SP334	4.53111	4.52265	3.73843	4.78139	-0.60235	-3.32193	-0.4861	-3.32193	0.70402	-3.32193	-0.35593	-3.32193	-0.70068	-0.70499	-3.32193	-3.32193
HPS5	2.81994	2.46378	2.65283	3.23822	2.86468	1.17574	3.12911	1.36074	3.00922	1.92584	2.90428	2.28373	3.43415	2.11081	2.59543	1.68705
GTF2H1	3.58236	3.49802	3.26318	3.92678	3.35507	2.03678	3.67168	2.05573	3.26358	3.17996	2.81568	3.62234	3.5975	3.30311	2.91107	2.64776
LDHC	-2.86726	-3.32193	-3.32193	-2.7684	-2.61763	-2.51529	-1.40872	-2.41022	1.40087	1.18849	0.83884	0.37192	-3.32193	-3.32193	-3.32193	-3.32193
TSG101	4.55154	4.86518	4.92592	5.30398	4.01936	3.82823	4.34285	3.73088	4.48395	4.45768	4.37795	4.86376	4.22818	4.34123	4.09798	4.14069
UEVLD	2.44937	2.70681	2.84849	3.42752	2.95759	1.80175	3.48544	1.88705	2.79442	2.53595	2.35063	3.33901	3.39034	3.01751	3.14736	2.92829
SPTY2D1OS	1.96625	1.95514	2.07317	2.14878	2.65727	1.20152	3.61888	1.51074	1.70541	1.79765	1.76995	2.52909	1.96663	1.77285	1.5951	1.61003
SPTY2D1	2.93751	3.08735	2.83354	3.44857	3.63686	2.55481	4.31452	2.3052	3.12486	2.92889	3.17266	3.74614	2.96449	2.80759	2.84459	2.85574
TMEM86A	2.06949	2.7857	2.70366	3.74175	0.06732	0.60792	0.21239	0.44559	-0.4386	0.25532	-0.66526	0.52127	-3.32193	-2.80755	-2.16975	-0.92028
IGSF22	-1.62392	-0.72774	-0.70644	0.30263	-2.69459	-2.75021	-2.28802	-2.85582	-2.68126	-2.49574	-2.44975	-2.77996	-3.32193	-3.32193	-2.93662	-2.36683
ZDHHC13	0.32162	0.47557	0.40052	0.92006	1.7902	1.52597	2.18338	1.42885	1.2775	1.55306	1.57494	1.72812	1.65749	1.6199	1.37899	1.41464
E2F8	0.53583	-1.06266	-0.93791	-0.92689	2.4144	0.3507	2.678	0.28082	2.13512	1.09947	1.14851	0.10763	2.63794	2.36016	2.3283	1.44154
NAV2	4.43173	4.42076	4.41225	3.99832	-0.07681	-0.68112	0.46567	-1.07487	3.21887	3.4184	3.28905	3.40442	3.43529	4.15168	4.07922	4.34023
NAV2-AS2	4.27457	4.37214	4.48803	3.06509	1.05735	-0.7328	1.78036	-2.19729	4.39098	4.39639	4.19363	4.05008	4.16234	4.9512	4.92256	5.36676
NAV2-AS1	5.80322	5.79875	5.78477	5.91933	-0.23592	0.22659	1.71512	0.10979	3.76892	3.44262	3.52995	4.41903	4.82695	5.59651	4.9738	5.20698
HTATIP2	5.62427	5.53741	5.64014	5.90268	6.06589	5.77706	6.26098	5.47007	4.59076	4.82013	4.74286	4.71555	3.5839	3.87346	3.10504	3.41055
PRMT3	2.8448	2.497	2.26582	2.53582	2.70247	1.59158	3.00202	1.79788	2.55065	1.94522	2.24263	1.75289	2.76927	2.03587	1.7396	1.375
ANO5	0.57992	-0.33211	-0.1315	0.23778	2.71341	0.92363	3.16375	0.94073	-3.14466	-3.17336	-3.16291	-3.17575	-0.34494	-1.95426	-0.53397	-1.46021
FANCF	2.53368	2.94698	2.90038	3.02782	2.79996	2.61136	2.80528	2.29293	2.96981	3.37237	3.24763	3.27025	2.39004	3.01365	2.64262	2.52151
GAS2	-0.20134	0.15325	-0.02052	-0.1002	2.35232	0.42785	2.44838	-0.05406	0.5153	0.77549	0.90848	-0.54842	0.01888	0.45465	0.68869	0.09191
SVIP	3.71385	3.79056	3.65239	3.89543	2.67679	3.55211	2.94198	3.39489	2.09842	1.92496	1.09207	1.47723	4.08469	4.01221	2.74789	3.44915
ANO3	-0.80154	-0.00905	0.39838	0.62092	-3.32193	-3.05431	-2.96324	-3.32193	-3.16209	-3.06565	-3.04831	-2.42664	-3.32193	-2.8433	-3.32193	-3.18657
MUC15	0.50407	1.58842	1.87255	2.5983	-3.32193	-3.01879	-2.80416	-3.32193	-3.32193	-3.32193	-2.75716	-3.32193	-3.32193	-3.08249	-3.32193	-3.32193
SLC5A12	-3.32193	-3.16427	-2.87234	-2.84723	-1.79646	-2.02547	-1.10494	-1.0609	-3.32193	-3.18547	-3.32193	-3.06485	-3.32193	-3.32193	-3.32193	-3.32193
BBOX1-AS1	-3.32193	-1.21096	-1.15067	-1.32658	1.29961	1.17196	1.13638	1.39669	-3.32193	-3.32193	-3.32193	-3.32193	-3.32193	-3.32193	-3.32193	-3.32193
CCDC34	5.09492	4.77748	4.82288	4.76898	5.46183	5.59021	5.76401	5.70676	3.81931	3.16373	3.7751	3.266	3.27462	3.1559	3.59567	2.954
LGR4	2.74642	2.86514	2.68981	3.30033	6.61806	4.35651	7.12207	4.25229	3.34281	3.64326	3.53475	4.14228	4.86302	4.69552	5.38428	5.24631
LIN7C	3.06085	3.11232	3.07653	3.30161	2.9184	2.34239	3.56993	2.514	3.10204	2.94468	3.17558	3.71268	3.11007	2.56455	2.26138	1.51736
BDNF-AS	-1.79852	-1.30781	-1.74942	-1.26691	-2.88485	-2.81481	-2.34214	-2.52014	3.02473	3.00613	2.85217	2.17104	1.18294	0.85388	0.37099	0.2104
BDNF	-0.99684	-0.95336	-2.04361	-0.94972	-2.99364	-2.54819	-2.51556	-2.54321	3.38865	3.70889	3.46173	3.2894	1.70051	1.73337	1.26739	0.79424
HSP90AA2P	1.42224	1.6574	1.26478	0.4791	0.78332	1.07114	0.91186	1.05362	1.05093	1.89241	1.33138	0.98277	1.14033	1.5015	1.44994	1.48036
KIF18A	2.95076	2.54327	2.6153	2.61939	2.47467	0.87301	3.0947	1.02716	3.78681	3.40401	3.18442	3.64982	2.9799	2.98194	3.01235	2.46869
METTL15	1.31894	1.40183	1.17847	1.73871	1.66174	1.01213	2.11082	1.13797	1.94348	2.01617	1.52121	2.31646	1.91914	2.10646	1.30022	1.74936
ARL14EP	2.49898	2.50968	2.26634	2.83347	2.07502	1.26149	2.40865	1.47807	2.34632	2.81623	2.2873	2.76366	1.99787	1.79042	1.47752	1.44325
DNAJC24	1.37539	1.4717	1.24172	1.53628	1.19696	0.59044	1.61716	0.35551	0.51688	0.80427	0.70981	0.84476	0.8591	0.74856	0.44263	0.02546
IMMP1L	2.51833	2.19751	2.16401	2.65062	2.14765	2.44204	2.37207	2.19018	1.14915	0.89294	0.79405	1.14662	2.14563	2.26049	1.15641	1.0453
ELP4	-0.14955	-0.27897	-0.19905	-0.00949	-0.69182	-0.90525	-0.49906	-1.12547	-0.40708	-0.61948	-0.6938	-0.38545	-0.35179	-0.4803	-0.52074	-0.83538
PAX6	-0.78322	-0.64396	-1.01973	-0.82096	-2.94239	-2.9416	-2.9675	-2.51124	-1.73988	-2.51032	-2.40466	-2.52085	-1.08609	-1.2913	-0.85679	-1.22591

WT1	-3.32193	-3.32193	-3.32193	-3.01367	-3.21071	-3.32193	-3.32193	-3.32193	0.67038	0.55062	-0.02964	-0.14403	-3.32193	-3.32193	-3.32193	-3.07808
EIF3M	5.70531	5.74608	5.61422	5.68102	4.77118	4.56749	5.22067	4.54855	5.78188	5.83484	5.70018	6.14292	4.42719	4.27361	4.10421	3.6319
CCDC73	3.47617	3.37217	3.30005	3.40778	1.81717	2.49833	2.26884	2.73902	2.89145	2.97883	2.54291	3.29935	2.44848	2.38938	1.26059	0.75438
PRRG4	0.8008	1.39845	0.31879	1.37749	0.03407	-1.27864	0.07908	-0.61956	-1.40611	-0.83613	-2.02685	-0.85631	0.24917	1.60227	0.73791	1.04161
QSER1	2.55993	2.55146	2.34312	2.78794	2.81333	1.40027	3.08426	1.36208	3.19775	2.70034	2.64387	3.81549	3.61434	3.55239	3.27996	3.6951
DEPDC7	1.60417	1.21801	1.34073	1.64809	1.871	1.25614	2.30322	1.43774	1.53293	1.23277	0.93585	1.19536	1.65671	0.87226	0.50026	0.38615
TCP11L1	2.70103	2.64717	2.81799	3.01965	2.73057	1.67714	2.83366	1.51987	2.87258	2.71211	2.50327	3.35213	2.96965	2.90931	2.96088	3.23615
PIGCP1	2.56318	2.61697	2.80391	1.97509	3.16605	2.37443	3.85881	2.33652	3.53491	3.30505	2.75439	3.71026	3.10301	3.31954	4.0219	3.87336
LINC00294	2.69199	3.11936	2.95392	2.90863	2.68061	3.15479	2.98607	3.14857	2.9876	2.68675	2.17053	3.16669	2.81051	2.90763	3.07306	3.23285
CSTF3	4.74423	4.70153	4.73907	4.79133	3.18896	2.89022	3.12578	3.02717	3.79292	3.35849	3.50906	2.95222	3.67888	3.60228	3.14733	3.50261
HIPK3	2.24262	2.71094	2.37081	2.92326	3.76541	2.19433	4.34347	2.17248	4.14793	4.24172	3.90028	5.18908	4.22002	4.42224	4.25233	4.4823
KIAA1549L	2.10097	1.92548	2.15199	1.84151	-1.3038	-2.13664	-1.32211	-2.94368	3.93514	3.79744	4.06952	4.69208	2.23735	1.42344	2.40694	2.67721
C11orf91	0.88138	1.29069	1.67623	1.66856	-2.52293	-3.32193	-0.57957	-1.28343	0.39505	0.14791	0.70843	-0.24607	1.6335	0.84797	0.67534	0.84807
FBXO3	1.1946	1.04738	0.87667	1.33157	1.37857	0.7123	2.24996	0.70958	1.44951	1.72992	1.58832	2.15411	1.52106	1.40909	1.67355	1.32408
CAPRIN1	5.5705	5.51594	5.42007	5.75423	5.33756	3.71621	5.70479	3.84789	6.32404	5.91762	6.04936	6.86569	5.559	5.43565	5.72814	5.65522
NAT10	4.8944	4.59671	4.19297	4.36354	4.70117	4.29649	4.68421	4.29497	4.88235	4.58325	4.80338	4.71726	3.98658	3.70029	4.06017	3.84405
ABTB2	4.51737	4.38614	4.11539	3.763	3.39501	2.22042	2.47586	2.31134	4.89441	4.90922	3.97285	3.96806	3.32069	3.48842	2.97511	3.65055
CAT	3.38754	3.77738	3.91382	4.25316	4.5636	4.583	4.70335	4.46659	4.53096	4.83166	4.56471	5.30111	2.89868	2.79545	2.59295	2.8937
EHF	0.078	1.50634	0.74184	1.22272	-1.21533	-2.26987	-0.78834	-2.28465	-2.30157	-2.30485	-1.81953	-0.81171	-3.32193	-2.68729	-1.80671	-1.96909
APIP	3.49236	3.57499	3.52207	4.10362	2.98645	3.33813	3.18152	3.25721	2.38452	2.67725	2.43773	2.30419	2.09416	2.8287	1.92342	1.75017
PDHX	4.59133	4.73359	4.50747	5.04146	4.39348	4.04108	4.80435	3.9964	4.17749	4.03578	4.07592	4.08638	4.19573	4.56327	3.94605	3.97925
CD44-AS1	7.75674	7.53261	7.55633	7.8112	0.8803	0.22584	2.43482	-0.80646	8.77056	8.88449	8.84863	9.11957	7.59302	7.42413	7.5657	7.81457
PAMR1	-3.0578	-2.78487	-2.76019	-2.14041	-3.32193	-3.32193	-3.32193	-3.32193	-3.01913	-3.32193	-3.32193	-3.32193	0.57058	-0.97772	0.60357	1.21133
FJX1	5.69133	5.42322	5.18702	3.89303	2.1215	0.08525	1.05741	0.61522	6.89478	6.29882	6.42816	4.01762	6.53982	6.1457	5.92029	5.86492
TRIM44	3.81039	3.65957	3.62463	3.66615	2.81181	1.31515	3.02378	1.60581	3.95847	2.98092	3.66301	3.17637	3.65249	2.38008	4.0829	2.41256
LDLRAD3	2.54867	2.28102	2.43453	1.99142	1.72516	-1.01595	1.6141	-0.87857	3.51579	2.85121	3.37095	3.37301	3.98148	3.60302	4.02221	3.34324
COMMD9	3.64495	3.57459	3.4439	3.67562	2.17482	2.2201	2.21036	2.03248	2.8864	2.78198	2.87913	3.19231	2.27532	2.28395	2.15752	2.18109
PRR5L	0.82032	1.17592	1.73306	1.92687	-1.60305	-1.8473	-1.75096	-1.98004	3.64796	2.5448	3.03235	2.28747	3.0415	2.62562	2.8049	2.14642
TRAF6	1.10797	1.59219	1.4948	1.81535	0.81941	-0.22235	1.36949	-0.09871	1.69151	1.74068	1.80064	2.85088	1.65914	1.57806	1.48162	1.88433
RAG1	-1.22523	-0.93352	-1.38693	-0.53691	-1.96545	-2.4713	-1.43895	-2.36217	-1.87222	-1.82646	-1.81003	-1.09797	-2.20619	-1.80559	-2.58751	-1.63082
RAG2	0.76187	0.37184	0.0996	0.58388	0.20688	-0.14134	0.53073	-0.30095	-0.11351	0.57445	-0.14632	0.00492	-0.90492	-0.50324	-0.71827	-0.18675
C11orf74	3.56583	3.54639	3.42017	3.7997	3.01895	3.41875	3.44796	3.39179	3.09525	3.66006	3.03084	3.72551	2.27615	2.52915	2.22488	1.96435
API5	5.46121	4.67441	5.25237	4.9386	5.06157	3.45934	5.68482	3.30524	5.7129	4.13018	5.3948	4.8565	5.43347	3.99225	5.25466	3.65795
TTC17	2.33495	2.48456	2.46009	2.92473	2.83945	2.52451	3.37736	2.62722	2.05705	2.3185	2.07425	3.00166	2.28675	1.86966	1.91238	2.1361
HSD17B12	3.88217	3.64552	3.47603	3.99099	4.4156	4.15872	4.95287	3.91917	3.97863	3.39994	3.4853	4.13499	4.61319	3.63206	3.97521	3.62337
ALKBH3	3.80798	3.41642	3.4837	3.33572	2.43891	1.91539	2.37867	2.10538	3.08834	2.56976	3.42537	2.81088	1.82168	1.04738	1.61718	1.16307
SEC14L1P1	0.4658	0.13558	0.69397	0.29052	-1.90916	-1.12093	-0.58925	-2.43712	-0.04599	-1.70891	-0.78778	-0.24782	-0.80103	-0.4365	-0.97794	-0.95255
C11orf96	0.35324	2.31684	1.64263	1.91635	-1.83353	0.40439	-1.9848	-1.42543	-1.98813	-2.14462	-2.90097	-2.15829	-2.49925	-1.9817	-2.92987	-2.60969
ACCS	0.60921	0.4863	1.2272	0.84683	-3.24253	-2.83231	-3.32193	-3.32193	0.60841	0.99159	1.08994	1.3788	-0.44099	-0.55138	-1.09281	-0.41502
EXT2	5.41894	5.29847	5.44352	5.547	5.10603	4.29762	5.09182	4.13881	5.46485	5.09624	5.56415	5.31509	4.05167	3.59014	4.26038	4.15922
CD82	7.62515	7.56159	7.58312	7.47457	2.69478	2.26413	2.68795	1.78547	6.45173	6.58254	6.38842	6.1917	2.42343	2.28759	1.86631	2.64306
TSPAN18	-3.1428	-3.12233	-2.25408	-2.90642	0.79393	-0.43135	0.29032	0.0394	-0.45403	-0.10688	-1.47314	-0.53741	-2.93159	-2.82338	-2.7323	-3.14688
TP53I11	-2.43805	-1.91028	-1.99872	-2.5847	3.66743	2.42413	3.05249	2.29677	2.32564	2.45149	2.04171	2.26244	-1.95856	-1.08561	-0.65587	-0.56181
PRDM11	-1.55891	-1.34481	-1.28626	-1.19308	-0.96072	-0.89143	-0.92644	-0.8617	0.9253	1.40901	1.21087	1.24462	0.82065	1.3164	1.67523	2.00037
SYT13	-3.12899	-2.47151	-2.30962	-1.5027	1.3806	-2.95529	1.94606	-3.32193	-3.32193	-3.32193	-3.32193	-3.32193	4.46486	5.80407	6.1639	6.68275
SLC35C1	3.56676	3.72781	3.70489	3.78449	5.06393	4.66135	4.84503	4.57339	3.35511	3.52936	3.50835	3.54993	2.13931	2.21171	3.10222	3.22186
CRY2	1.30213	1.30714	1.27999	1.26736	2.85811	2.16732	2.74207	2.2823	2.28898	2.19484	2.80981	2.60431	2.23851	1.91247	2.83824	2.39373
MAPK8IP1	2.93009	3.27617	3.31575	2.82831	3.49154	2.99937	3.03161	3.04979	3.0931	4.10032	3.73013	3.69036	1.45529	2.24018	3.22608	3.27031
PEX16	4.88599	5.03815	4.88654	4.80235	3.69782	4.38787	3.04459	4.39199	3.82806	4.02198	3.91872	3.34147	2.16322	2.61175	2.62634	2.92905
LARGE2	-2.51666	-1.90081	-2.65257	-1.79225	3.15959	3.19278	1.65384	3.56504	2.5229	2.74052	2.61818	1.75837	-0.71902	-0.77041	-1.28896	-0.89524

PHF21A	1.85735	1.88315	2.29725	2.39909	1.78751	1.98439	1.83861	1.97977	2.43638	2.76729	2.66699	3.5262	1.37522	1.31797	2.01439	2.18487
CREB3L1	5.27011	5.01421	5.5191	4.47628	0.65798	2.07088	0.49572	1.66181	5.20048	4.79782	5.3288	4.38635	-2.39313	-2.94324	-2.8708	-2.86226
DGKZ	4.69855	4.56467	4.42995	3.88538	3.89595	3.51524	3.19957	3.57717	4.27316	3.90985	4.29047	3.61445	2.97796	2.99946	3.5639	3.6719
MDK	4.75277	5.56273	5.66454	6.00381	5.16527	7.90549	4.33328	7.66721	4.16091	4.97986	4.40772	4.24257	4.94774	5.31575	4.85215	5.128
AMBRA1	3.21638	3.30544	3.00379	3.33603	3.30225	3.06055	2.74208	3.17423	3.45929	3.67691	3.57274	3.90054	2.01378	2.25535	2.91566	3.19246
HARBI1	2.51779	2.95559	2.89865	3.17105	3.38738	2.45024	3.67299	1.83503	2.83773	3.22058	3.07658	3.39507	2.41431	2.23887	3.1532	3.2376
ATG13	3.99874	4.0169	3.83769	4.35194	4.14101	4.04227	4.18001	3.64778	3.81436	3.9396	4.02616	4.53537	3.25407	3.08798	3.67102	3.84601
ARHGAP1	4.77342	4.77343	4.77658	4.74702	4.70582	4.52338	4.25749	4.4434	4.74997	4.5856	4.87012	4.84483	3.63266	3.52737	4.70486	4.26868
ZNF408	3.83755	3.78638	3.77213	3.41523	3.57823	3.8094	3.15044	3.72313	2.90981	3.14622	3.11566	2.69118	2.05983	1.9558	2.17171	2.13144
F2	-2.93858	-2.89796	-2.53854	-2.85285	6.17373	7.80996	5.54425	7.66718	-2.55	-2.95065	-2.14484	-2.42229	-2.04057	-2.76103	-2.9539	-2.19228
CKAP5	4.82401	4.58344	4.68121	4.53749	4.96942	3.85819	5.43877	4.02839	5.68008	4.8056	5.21769	5.22083	5.53574	5.37451	5.86135	5.49425
LRP4-AS1	1.26794	0.95239	1.69302	1.03037	1.39639	-0.27005	1.11421	0.1757	0.9594	0.33256	0.317	0.55344	0.0591	0.10567	0.9534	0.72121
LRP4	1.86708	1.87451	2.38491	2.28884	2.28155	1.18422	1.83328	1.26821	2.0013	1.32349	1.6803	1.41549	1.21463	1.04208	1.6794	1.85406
C11orf49	1.96477	2.23893	2.45918	2.26275	2.27873	2.78172	2.08599	2.7515	2.00802	1.94754	2.50739	2.2907	1.89363	2.3419	2.35794	2.11511
ARFGAP2	3.82096	3.59478	3.68262	3.68161	3.75559	3.67541	3.57746	3.79102	3.95527	3.94742	4.45321	3.85767	3.26822	3.55842	3.93198	3.77116
PACSIN3	5.75473	5.95456	5.71212	5.38017	5.89299	5.51214	5.24923	5.67298	4.89404	4.8366	4.90065	4.22144	4.18899	4.28834	4.52949	4.84271
DDB2	2.89185	2.97379	2.98387	2.69654	3.31825	2.79876	3.17562	3.10463	4.29073	4.27703	4.31593	4.18147	2.03496	2.30379	1.91725	2.1475
ACP2	4.06529	4.44811	3.97731	4.41496	5.69369	6.20063	5.45179	6.04199	4.1013	4.46297	4.65052	4.52042	2.88995	3.46899	3.65847	4.12497
NR1H3	2.51065	3.0773	2.53097	2.9178	3.96846	4.31749	3.38851	3.99491	1.70678	2.67963	2.26311	2.07262	1.31476	1.67242	1.83412	2.38204
MADD	3.18861	3.25148	3.31755	3.6196	3.49138	2.89978	3.568	3.03935	3.40345	3.63233	3.72026	4.21001	2.53013	3.20101	3.37988	3.73428
SLC39A13	5.36725	5.36207	5.66357	5.16767	3.98685	4.36753	3.06991	4.09902	4.38283	4.5178	4.87213	3.92024	2.58877	1.99487	2.41615	2.46018
PSMC3	7.91911	7.80269	7.72678	7.59759	6.63022	6.35303	6.44764	6.30799	7.18042	6.73972	7.20574	6.22136	6.10904	6.0637	6.32829	5.9779
CELF1	4.28947	4.1336	4.18694	4.15366	4.14522	3.63463	4.33833	3.68012	4.59064	4.01444	4.70344	4.62947	4.51019	3.94244	4.53812	3.81465
NDUFS3	5.19047	5.42359	5.36944	5.1904	4.98873	5.45653	5.07289	5.31166	4.50086	4.95206	5.07401	4.32248	4.45851	5.03433	5.01546	4.68431
PTPMT1	4.91831	5.34129	5.27645	5.05674	4.5859	5.07686	4.82871	5.01233	3.86006	4.72714	4.82418	4.12044	3.11162	4.66654	4.80085	4.66318
KBTBD4	3.34816	3.2666	3.2242	3.57763	3.61103	2.85389	3.58395	2.97158	3.48442	3.64884	3.76183	3.53853	2.60516	3.00478	3.09387	3.10597
MTCH2	6.127	6.13695	5.95769	6.21204	5.65542	5.58954	5.87264	5.51207	5.93539	5.49762	5.46416	5.62632	5.49301	5.70991	5.02044	5.10875
AGBL2	-1.43852	-1.49735	-1.25627	-1.01997	-1.36378	-0.24432	-1.2615	-0.6852	-1.83143	-1.37093	-1.40121	-0.20943	-2.6855	-2.58814	-1.9314	-2.97
FNBP4	3.57922	3.21684	3.42982	3.80118	2.73578	3.33777	2.80447	3.59051	2.56546	2.66374	2.45895	3.20089	3.15981	3.04328	2.4032	2.70294
NUP160	3.68892	3.46199	3.40113	3.76474	3.65965	2.32322	3.9534	2.37013	3.87057	3.43981	3.56874	3.75864	4.02873	4.03547	4.03905	4.12912
PTPRJ	3.7174	3.67961	3.65995	4.21055	3.825	2.78552	4.06873	2.26939	4.48543	3.9811	3.99816	4.93623	4.09077	4.32069	5.00818	5.02471
TNKS1BP1	5.03464	5.162	5.2578	4.58341	4.54177	4.82658	4.20176	4.74226	5.77719	6.01645	6.11665	5.81831	3.82798	4.34611	4.67446	5.27526
SSRP1	6.06231	5.58173	5.55631	5.30791	5.75162	5.19178	5.59504	5.1781	6.67723	5.98184	6.29518	5.81574	5.597	5.6618	5.86739	5.65114
SLC43A3	5.89396	5.8429	5.66465	5.5057	-2.87317	-2.41995	-3.10732	-3.05365	5.84747	5.61259	5.15495	5.36786	0.94912	2.0719	1.90375	1.77398
RTN4RL2	3.35678	4.09646	4.44029	2.95897	4.40882	4.98007	2.9021	5.49059	2.49002	3.48532	2.42981	1.77777	0.431	1.1252	1.70771	2.26183
SLC43A1	3.00192	2.28098	2.42188	2.05312	5.7525	4.91244	5.22536	4.91315	1.6622	0.80964	1.43031	0.31698	0.57958	-0.51379	0.74285	0.44908
TIMM10	6.43633	5.92755	5.6653	5.58547	6.28517	6.29408	5.70396	6.48097	7.13412	6.65056	6.81612	5.34441	6.43841	6.19458	5.2282	5.08009
UBE2L6	5.69636	5.30858	5.22135	5.02331	1.26934	2.60047	0.57042	2.61082	6.01639	5.77633	6.48092	5.75983	3.41577	3.92716	3.87484	4.32875
SERPING1	-1.6647	-0.50545	-0.5417	-1.42688	3.63814	5.29897	2.8636	5.01486	-2.32082	-2.02677	-3.03133	-2.16796	-2.90237	-3.32193	-2.45589	-1.78991
CLP1	3.88209	3.55258	3.61058	3.74742	3.40165	2.47264	3.41021	2.412	3.48685	2.49742	3.71593	2.51376	2.36475	2.08517	1.93142	1.31402
ZDHHC5	4.81132	4.69764	4.73734	4.63369	5.35768	4.72834	5.2387	4.64445	5.448	4.73536	5.17637	4.87909	5.02439	4.38557	5.10032	4.93068
MED19	4.00547	4.07503	3.92486	4.34719	3.46474	3.8436	3.12219	4.06378	4.21007	4.41631	4.00696	3.37435	3.87232	4.00411	3.47947	3.45344
TMX2	5.64581	5.47604	5.26066	5.4575	6.64955	6.27265	6.69455	6.19899	5.51503	5.11221	5.39296	4.85274	5.58059	5.48102	5.18028	5.18258
TMX2-CTNND1	5.48147	5.61301	5.35081	5.3681	7.30771	5.53537	7.49712	5.50926	6.56189	6.41454	6.78382	6.79317	6.3521	6.26967	6.79849	6.76108
SELENOH	5.39303	5.37313	5.43237	5.23394	5.49103	5.94048	4.86655	5.63111	4.57633	5.3291	5.05775	4.31169	4.70505	4.95838	3.80277	4.08934
CTNND1	4.06881	4.04063	4.27121	4.44657	5.71062	4.45863	6.03609	4.51265	5.15832	5.09641	5.30665	5.96528	4.89976	4.81745	5.68277	5.45674
LPXN	6.63272	6.37889	6.47622	6.86179	-0.85131	1.05111	-0.2117	0.33341	0.02034	0.91829	0.96913	2.0132	0.24422	-0.81955	-1.02775	-0.54938
ZFP91	3.85427	3.93573	3.88662	4.24658	5.13581	3.64236	5.57309	3.65972	5.13822	4.72687	4.75697	5.4368	5.42353	5.17455	5.16366	5.08905
ZFP91-CNTF	3.4363	3.65608	3.66586	3.72174	5.31516	3.14827	5.3769	3.26455	5.23022	4.8482	4.81977	4.96818	5.06751	4.89137	5.1046	5.05815
CNTF	0.00218	0.15298	-0.112	0.11487	0.72151	-0.31413	1.2829	-0.54609	-0.79508	-1.13405	-0.93097	-0.12692	0.21338	0.59217	0.70117	0.76516

GLYATL1	-3.12952	-3.32193	-3.32193	-3.08301	-3.23706	-3.12753	-3.32193	-3.32193	-0.74299	-1.04331	-2.01277	-2.00709	-2.78878	-1.7347	-2.57255	-2.96753
FAM111B	2.93248	2.74573	1.96657	2.02029	3.51603	1.41116	4.12028	1.73381	3.50837	1.97674	2.02691	1.77951	3.83291	3.72546	3.28235	3.22258
FAM111A-DT	0.96245	1.2464	0.85072	1.54361	0.21323	0.14888	0.11907	0.40221	0.70913	0.00784	0.36233	0.43484	0.42369	-0.43173	0.18468	0.06343
FAM111A	2.86814	2.58186	2.77585	2.90065	2.40455	1.40804	2.87649	1.7151	3.10774	1.92996	2.63523	2.18671	2.57341	1.94818	1.9199	1.6125
DTX4	3.38943	3.54035	3.68035	3.45411	3.06857	0.55571	2.89948	0.88623	0.74404	1.05965	0.98569	1.24977	-3.18424	-2.83741	-2.87186	-2.86333
OSBP	3.84105	4.15522	3.86613	4.18789	5.04107	4.3965	5.06326	4.35795	4.66094	4.58346	4.3704	5.00774	3.7733	3.94909	3.98232	4.25056
PATL1	4.38981	4.24148	4.35433	4.36763	4.62477	4.13744	4.80952	4.05938	5.08695	4.46192	4.76659	5.01941	4.62992	4.75156	4.80848	4.35598
STX3	3.59903	3.64856	3.61654	3.69918	3.26307	3.40299	4.35719	3.1694	3.46737	3.29903	3.42462	3.70718	3.21466	3.87345	3.18277	3.09246
OR10Y1P	-1.94693	-0.65148	-0.22562	-0.14442	-2.9014	-3.32193	-3.32193	-3.32193	2.73901	2.25917	2.2646	1.86805	-3.32193	-3.32193	-3.32193	-3.32193
FABP5P7	4.92497	4.03975	3.99931	3.28936	5.14327	5.26543	4.84402	5.22808	6.16193	5.46811	5.70356	4.58978	1.56194	1.03193	1.19885	0.83438
MRPL16	4.90541	4.85144	4.64387	4.82791	4.29652	4.67225	4.26846	4.63546	4.50985	4.44741	4.67753	4.3854	4.12511	4.35819	3.32469	3.33702
TCN1	-3.32193	-3.32193	-3.32193	-2.62793	-3.04951	-3.32193	-3.32193	-3.32193	-2.22592	-0.30788	-1.50033	2.51432	-2.85373	-3.32193	-3.32193	-3.32193
CCDC86	6.73874	6.26728	6.0474	5.35375	6.37064	5.71224	5.71384	5.72998	6.25069	5.36266	6.18414	4.43227	6.1109	5.10909	6.05245	5.21689
PTGDR2	2.3618	1.89176	1.72591	1.52365	1.66121	1.82475	1.43433	2.13905	1.47503	0.18151	0.86544	-0.0152	2.61538	1.82909	1.74446	0.49375
ZP1	0.93814	1.68309	1.91125	0.83937	-3.13884	-3.32193	-3.32193	-3.32193	-3.32193	-3.32193	-3.32193	-2.94116	-2.51555	-3.32193	-2.93878	-2.62431
PRPF19	6.84339	6.54802	6.43367	5.95882	6.11047	5.53777	5.56578	5.51306	6.84237	6.14821	6.74513	5.71669	5.72733	5.63626	5.55908	5.47513
TMEM109	6.76182	6.05889	6.27759	5.5157	7.10618	6.33295	6.40013	6.1816	7.26569	5.88768	6.74195	5.19254	6.11514	5.29241	6.33758	5.73983
SLC15A3	3.22857	3.02841	3.18626	2.98462	-1.11723	0.37055	-2.11869	0.28281	1.67652	1.43596	2.23254	0.99139	0.73148	-0.26391	-0.55349	-0.40331
CD6	-0.05926	-0.2171	-0.12353	-0.56583	-3.24424	-3.32193	-3.32193	-3.11411	-3.32193	-2.99858	-3.32193	-2.86725	-3.32193	-3.32193	-3.32193	-3.32193
VPS37C	3.01028	2.95109	2.80656	2.54599	2.79192	2.79833	2.43928	2.63943	3.06907	2.79132	3.0987	2.48053	3.14851	3.00455	3.13155	2.93275
VWCE	-2.03911	-2.1513	-2.36345	-1.39051	-3.05543	-1.55004	-3.32193	-1.61141	-1.3359	-0.87488	-1.52517	-1.11312	-2.23599	-2.41878	-2.27633	-1.58601
TKFC	3.29581	3.27701	3.08311	3.22475	3.70444	3.84641	3.05792	3.55351	3.66127	3.70109	3.93906	3.28795	2.0004	1.73634	2.06673	2.14992
CYB561A3	3.73788	3.76881	3.52156	3.86764	3.48722	2.32468	2.92982	2.21908	2.75461	2.17442	2.64983	2.17054	2.13106	1.08675	2.06476	1.60288
TMEM138	3.0559	2.67779	2.6519	3.06572	3.28814	2.84711	3.13127	2.8847	2.09697	1.31123	1.67945	1.1208	2.66346	2.06849	1.74205	1.42068
TMEM216	-0.22582	-0.69925	-1.42559	-0.95888	2.09863	3.12057	1.70332	2.91061	1.99727	2.39516	1.87622	2.24409	1.30234	1.92241	0.47005	1.28617
CPSF7	3.76974	3.64852	3.69566	3.56707	4.35269	3.84415	4.21036	3.95426	4.06477	3.60899	4.0016	3.90294	3.96096	4.10215	3.91194	3.97242
SDHAF2	3.89361	4.09794	3.88968	4.16906	4.26006	4.40585	4.26085	4.23035	4.283	4.06247	4.0825	4.44043	4.28161	4.39292	3.67773	3.67168
SYT7	-2.84568	-3.32193	-2.37576	-3.10211	2.24322	0.34844	1.41566	0.55051	-0.93149	-1.73144	-1.85947	-2.33006	-2.82771	-3.1821	-3.32193	-2.72842
RPLP0P2	-0.48298	-0.11318	-0.11516	-0.14869	-2.81439	-2.47154	-2.37583	-2.19709	-2.5629	-1.98184	-2.03518	-1.06962	-2.76941	-2.62596	-3.32193	-3.06642
DAGLA	2.59259	2.29181	2.29498	1.65369	2.35652	1.61251	1.79598	1.22951	2.16596	1.90304	2.54595	1.12643	-0.99613	-1.23025	-0.26246	-0.19067
MYRF	1.52042	1.82459	1.6894	1.86075	2.74586	3.99868	2.11078	3.87424	1.28176	2.83787	1.70235	2.83328	1.54786	2.74853	2.2829	3.28566
TMEM258	5.6951	5.6711	5.8677	5.54021	5.6378	6.54347	5.42804	6.34589	5.22665	5.44446	5.16372	3.77585	4.52172	4.57872	3.82096	3.94077
MIR611	10.2332	10.3732	10.4483	10.1183	10.2223	10.8574	10.294	10.5657	9.89693	10.0637	9.76307	6.83677	9.17312	9.08633	8.60512	8.42996
FEN1	6.70161	6.4153	6.2497	6.02551	6.06685	5.27671	6.01268	5.43674	6.22715	4.78418	5.45195	4.05385	5.82796	6.14137	5.31294	5.60759
FADS2	2.55212	2.76548	2.62745	2.4461	3.04846	3.03671	2.65594	3.12766	3.08932	2.97671	2.69772	2.05096	3.80246	4.72928	5.11143	5.37337
FADS1	3.97324	4.02797	3.99269	4.09593	5.29137	5.37287	4.82775	5.39036	4.37708	3.87655	3.87569	3.57926	4.48623	4.74248	4.88659	4.95776
FADS3	4.7676	4.51862	4.74617	4.21075	2.21677	3.60168	1.51389	2.93859	4.17261	4.0446	4.18846	3.39054	1.94305	1.55811	1.99282	1.59897
RAB3IL1	3.47905	3.79038	3.51706	3.55288	4.30138	4.54755	3.44587	4.62992	2.665	3.01205	2.76271	2.05765	2.55705	2.82247	3.47814	3.12383
INCENP	4.95835	4.43689	4.53255	3.63345	5.05768	3.88594	4.47721	3.9538	5.59943	4.70141	4.97901	3.67233	4.26498	4.29612	4.73905	4.92723
ASRGL1	2.58243	2.37594	2.10112	2.35633	2.60409	1.82348	2.26057	2.44741	1.87205	1.07539	1.12849	0.21273	2.17051	1.79447	1.98613	1.65779
MIR6747	4.99586	4.7673	4.98889	4.01452	4.07479	4.82788	4.64619	4.1906	4.65772	5.34297	4.79593	5.3614	2.34593	3.17617	2.88529	3.13608
TUT1	4.67467	4.45212	4.38169	3.85812	3.9526	4.4642	3.07141	4.59196	3.66392	3.63468	3.75631	2.8265	2.64771	2.78304	2.40868	3.07251
MTA2	5.39806	5.1113	5.2078	5.05707	6.02041	5.67313	5.84282	5.64587	5.64933	5.1188	5.71282	5.06463	5.17678	5.18816	5.76827	5.82501
EML3	4.26084	4.20374	4.34925	3.8898	3.97586	4.17414	3.05449	4.1337	4.6948	4.29836	4.96375	3.78344	3.02711	2.71281	3.40978	3.12928
ROM1	1.90286	2.25658	2.30094	1.20107	2.01799	1.90626	1.07281	1.76253	2.65144	2.75685	3.07831	1.52205	1.10238	0.90511	1.56128	1.54751
B3GAT3	5.69455	5.82385	5.80824	5.70475	5.8475	6.272	4.95476	6.05739	4.94092	4.67933	5.10043	3.91358	4.21579	3.61498	3.73495	3.64201
INTS5	4.14002	4.31535	3.96151	4.03953	4.1025	3.61857	3.74161	3.54221	3.75211	3.86689	3.93075	3.74219	2.68902	3.04797	3.21774	3.57903
C11orf98	7.04015	6.69233	6.59565	6.39316	7.28361	7.7278	6.61874	7.66637	6.3318	5.8491	6.19475	4.15794	6.73827	6.60738	5.95622	6.01655
LBHD1	5.5584	5.17146	5.12807	4.90831	5.9164	6.35595	5.16906	6.2823	5.18288	4.60274	4.92031	3.16049	5.54107	5.26825	4.89027	4.7608
CSKMT	1.89005	1.61065	1.82189	1.93441	1.49134	2.1251	0.85276	2.40274	0.77778	0.95072	1.15086	0.24578	0.71926	0.47875	0.48837	0.1043

SNORA57	4.27596	3.87933	4.35505	3.79794	4.15363	5.56982	2.31097	5.7549	1.33447	1.63865	2.83084	1.3612	3.42089	2.72377	0.6708	2.7459
UQCC3	4.07598	3.52895	3.67282	3.11092	4.78758	5.2302	3.91502	5.11237	4.4216	3.73692	3.94068	2.32457	4.74415	4.2109	4.25347	3.79397
UBXN1	6.3127	6.30485	6.18735	6.03418	5.88124	6.55989	5.49528	6.47076	6.21836	6.2944	6.36413	5.65951	4.9887	5.38403	5.18428	5.36876
LRRN4CL	-0.28529	0.91537	0.22293	1.90251	-2.85461	-2.63744	-3.32193	-2.54438	-3.32193	-2.95509	-2.93123	-3.32193	-2.76569	-2.76733	-3.32193	-2.65655
BSCL2	3.31694	3.67546	3.60394	4.00789	3.32089	4.73229	3.16082	4.66585	3.37946	3.5331	3.69103	3.8872	3.25495	3.8574	3.54451	3.91782
HNRNPUL2-BSC	5.76233	5.45708	5.85431	5.36941	5.89	5.58952	5.92416	5.51019	6.47655	5.44098	6.41522	5.17418	5.63879	5.14251	6.16017	5.22612
GNG3	-0.02921	-0.16791	0.1922	0.1372	0.94952	1.3736	-0.25283	1.69594	0.29291	0.36508	0.55323	0.52076	-0.0055	0.91533	0.35185	0.92851
HNRNPUL2	5.28871	4.79353	5.34318	4.49173	5.42233	4.40375	5.53532	4.34808	6.13588	4.85524	6.01915	4.55597	5.37348	4.47196	5.9244	4.5508
TTC9C	4.13006	4.33143	4.35148	4.20375	3.57113	3.84743	3.28573	3.79268	4.19368	4.19569	4.2694	3.50016	3.20646	3.79419	3.322	3.30828
ZBTB3	0.45942	0.53903	1.03557	0.20605	0.79438	1.03237	0.21449	1.44328	0.53333	0.59292	0.66804	1.42931	0.03734	0.45075	0.39758	0.89881
POLR2G	5.13473	4.84674	4.82085	4.88256	4.80853	5.42996	4.44376	5.2579	5.59053	5.11358	5.32748	4.20075	5.32432	5.53846	4.18977	4.32403
TAF6L	4.30149	4.24538	4.43429	4.01111	4.23949	5.23663	3.41099	5.54839	3.31956	3.32004	3.54109	2.38964	2.74494	3.23158	2.83984	3.03658
TMEM223	5.22791	5.4252	5.39789	5.28504	5.40752	5.88403	5.01575	5.63158	4.84928	4.78355	4.81065	4.53839	4.45908	4.37301	3.96971	3.86625
TMEM179B	5.31802	5.48797	5.49673	5.43932	5.42328	6.10575	4.98303	5.90432	5.23067	4.94799	5.2577	5.25981	4.57999	4.44139	3.7679	3.92957
NXF1	3.58842	3.24713	3.32642	3.70128	3.13765	3.42007	2.8051	3.70281	3.269	2.84894	3.19336	3.1012	3.48195	3.41469	2.77553	2.88035
STX5	4.10642	4.0071	4.02806	4.1406	4.3608	4.33351	3.84638	4.09025	4.32806	4.20455	4.11912	4.48528	3.66765	3.65026	3.84944	3.8408
WDR74	4.80386	4.59699	4.32792	4.21021	3.63787	4.30042	3.26325	4.27648	4.07004	3.96253	4.08311	3.02475	3.78522	3.95485	3.50952	3.54962
SNHG1	4.92664	3.97137	4.21018	3.9164	4.24016	4.73742	4.08552	5.04157	2.94295	3.03032	2.87063	2.15973	3.80461	3.45364	1.63168	1.3217
SNORD22	5.88833	4.42844	5.61452	5.44776	5.08096	5.74577	5.57962	6.53002	2.35384	2.09012	3.61164	2.57031	5.20066	5.11774	2.43105	2.29537
SNORD30	5.64367	4.49187	3.98756	4.57861	4.64715	4.49316	4.37844	4.40599	3.18924	2.12982	3.21548	1.13892	3.97592	3.80203	1.71181	1.74242
SNORD28	5.33837	4.22373	5.10549	4.09447	5.12185	4.53096	5.31121	5.21043	4.08234	2.03264	3.69646	3.44508	4.61007	4.02294	1.61542	2.04942
SNORD27	5.24139	3.98446	4.81739	4.3584	4.85509	4.30143	4.87183	4.2729	2.67011	2.09012	4.0747	3.21676	4.46315	3.76168	2.86872	0.20405
SNORD26	4.96092	4.74494	4.82261	4.85	5.0278	3.25044	4.90903	5.00458	3.44964	2.6058	3.43565	3.68405	4.34822	4.3431	2.33343	2.04942
SNORD25	5.81611	5.38413	5.0037	4.903	5.91389	4.99745	5.74543	5.4233	5.24852	5.02855	5.4718	3.90962	5.14357	3.99143	2.9293	3.15077
SLC3A2	6.18964	5.53495	5.49328	5.42422	6.54816	6.71905	6.4904	6.78428	7.02315	6.03317	6.30652	5.14377	6.47293	5.75723	5.96035	5.6971
SLC22A8	-3.32193	-3.32193	-3.32193	-3.32193	-3.26723	-0.55746	-3.32193	-0.24018	-3.32193	-3.32193	-3.32193	-3.32193	-3.32193	-3.32193	-3.09084	-3.19921
SLC22A10	-3.32193	-3.32193	-2.95753	-3.32193	-0.26647	1.00916	-0.4025	0.67814	-3.32193	-3.32193	-3.32193	-3.32193	-3.32193	-3.32193	-2.77287	-2.76281
SLC22A9	-3.32193	-3.32193	-3.32193	-3.32193	-0.07208	0.29815	-1.19302	0.95373	-2.22041	-3.32193	-2.30673	-2.62327	-3.32193	-3.32193	-2.92827	-3.32193
ATL3	3.68196	3.76092	3.77087	3.93175	4.22244	3.69542	4.7078	3.65798	4.44852	3.87328	4.12688	4.61278	4.79398	4.43936	4.56473	4.30903
RTN3	4.75598	4.83571	4.85049	5.19862	5.6188	5.70466	5.81214	5.64666	5.42766	5.18316	5.0112	5.70409	5.1134	5.40642	5.76373	5.68477
C11orf95	2.56232	3.00156	2.81578	2.63488	2.47472	1.94859	2.2151	1.98147	2.39001	3.70709	3.69393	3.42798	1.9378	2.49079	3.76997	3.82482
SPINDOC	4.65726	4.51006	4.55388	3.91066	5.03683	5.0724	4.33851	4.77762	5.17116	4.74357	5.17181	3.7116	4.43361	4.27771	4.38191	4.16255
MARK2	3.45566	3.56324	3.47013	3.39619	4.36708	4.07294	4.28109	3.98235	3.73581	3.39704	4.08797	2.94334	3.37374	3.07367	4.10419	3.57619
RCOR2	-1.09387	-0.26575	0.35093	-0.91754	0.60462	1.67595	-0.3931	2.53553	0.63202	0.75308	0.09632	0.50257	-0.22593	0.85109	0.64287	2.14251
NAA40	2.38397	2.34859	2.3291	2.29992	3.39755	3.50492	2.95705	3.47011	2.30215	2.4374	2.22601	2.7698	3.03995	3.35207	2.80599	3.11821
COX8A	9.21222	9.021	9.00531	8.71037	8.86938	9.67037	8.17292	9.52509	9.20228	8.90155	8.87158	7.98407	9.10447	9.08606	8.29614	8.43869
OTUB1	4.9714	4.98296	4.94519	4.67417	4.95444	5.3585	4.34165	5.27025	5.19781	5.15717	5.04802	4.65981	4.4101	4.42951	4.39706	4.69197
MACROD1	4.96161	4.89679	4.88921	4.56915	2.51931	4.18633	1.84907	4.15544	3.77189	4.64174	4.21523	3.12582	2.66855	2.67663	2.52105	2.52505
FLRT1	-0.49561	-0.47863	-0.28313	-0.52397	-0.71509	-0.44246	-2.0188	-0.87793	-1.94391	-1.52959	-2.30901	-2.11766	-1.46693	-1.3529	-1.89497	-1.10654
FERMT3	4.19781	5.22408	4.91408	4.70489	0.12311	1.2804	-0.07303	1.73188	-0.2092	-0.18259	0.06218	-0.72751	-0.26229	-0.57118	-1.1124	0.02006
TRPT1	4.10162	4.52612	4.74922	4.59534	3.72033	4.67575	3.01182	4.79235	3.91467	4.19262	3.80709	3.85621	2.44294	2.54342	2.28131	2.427
NUDT22	4.29601	4.60469	4.64434	4.22823	4.46684	5.2478	3.61917	5.15184	4.38721	4.8641	4.72933	4.03849	2.67976	3.24468	2.82169	3.25774
DNAJC4	2.98134	3.63465	3.71559	3.82086	3.51351	4.61666	2.43052	4.55754	3.87369	4.5204	4.21082	3.83862	2.57254	2.83956	2.86401	3.14
VEGFB	6.60888	6.916	7.08778	6.74343	5.92746	7.23353	5.25457	7.14249	6.05506	6.19372	6.05421	5.61353	4.41628	4.6885	4.94239	5.24397
FKBP2	5.99247	6.32498	6.25406	5.84083	7.01828	8.36373	6.60542	8.11424	5.91001	5.93791	6.14773	5.0017	5.01083	5.14279	4.95732	5.13904
PLCB3	4.11388	4.39477	4.50097	4.15897	4.93696	4.36811	4.20402	4.37319	5.17617	5.24804	5.24364	5.14772	3.79239	4.27318	4.1211	4.4226
BAD	5.41695	5.5584	5.66862	4.86708	5.95892	6.34405	5.14873	6.06398	6.23785	5.9788	6.16765	4.9354	5.09459	4.86552	5.05697	4.7319
GPR137	5.08363	5.27655	5.36073	4.75525	5.82096	6.02847	4.99598	5.87817	5.54173	5.50881	5.69874	4.69742	4.20847	4.19627	4.43224	4.46488
CATSPERZ	2.55347	2.356	2.68339	2.00557	-2.63247	-1.64934	-2.09136	-1.88053	1.56101	1.66011	1.18075	0.41232	-0.47074	-0.59276	-2.56605	-3.32193
ESRRA	6.00134	5.91414	5.85648	5.34515	6.18655	6.54712	5.54978	6.48362	5.45701	5.7666	5.65109	4.29091	4.81011	5.14781	4.51217	4.71403

TRMT112	7.6039	7.75903	7.77568	7.33868	7.1699	8.14014	6.84255	7.92656	6.99508	7.34301	7.54481	5.97187	6.23117	6.66552	5.86733	5.69975
PRDX5	8.53069	8.98075	8.91837	8.75983	9.27009	10.1801	8.5895	9.83168	8.89945	9.38599	9.10479	8.17681	7.45194	7.90651	7.52342	7.80194
CCDC88B	-1.26276	-0.52281	-0.3105	-0.69557	0.0914	2.28835	-0.2832	1.72347	-0.76786	0.32179	-0.21354	-0.62692	-0.43	1.04004	-1.16202	0.59372
RPS6KA4	5.02845	5.12247	5.2265	4.0143	5.6428	5.43642	5.20047	5.38064	6.86269	6.27497	7.03896	5.11984	5.20922	5.23124	6.0205	5.88624
NRXN2	-1.34582	-1.19238	-0.74813	-1.80989	-3.07469	-3.21871	-3.05239	-2.9865	-2.72585	-2.27853	-2.63671	-2.74972	-3.32193	-3.24159	-3.32193	-3.32193
RASGRP2	-3.14871	-3.32193	-3.32193	-2.91912	-1.349	1.66436	-1.66961	1.25769	-2.94724	-2.24666	-2.84016	-3.00942	-3.32193	-3.05989	-2.34054	-2.86411
SF1	5.91216	5.53691	5.46886	5.27806	5.4124	4.87591	5.25196	5.00854	6.21148	5.67858	6.07673	5.53985	5.28703	5.34158	5.66143	5.61704
MAP4K2	2.61999	2.95691	2.87276	2.97497	2.87403	3.24127	2.00079	2.96566	3.79692	3.55258	3.72559	3.17876	1.76508	1.94358	2.12196	2.24307
MEN1	5.10856	4.83987	4.78898	4.38001	5.10423	5.12211	4.21158	5.00267	5.03088	4.80202	5.19185	4.09876	2.76592	3.01372	3.07758	3.36751
CDC42BPG	-2.5048	-1.54669	-2.08684	-1.85552	1.40355	1.2464	0.74542	1.34009	1.45525	1.82907	2.10751	1.57971	-2.73946	-2.4019	-1.65187	-1.52352
MIR194-2HG	-2.60961	-2.54239	-2.99755	-3.32193	1.26588	2.43112	-0.16071	2.77785	-3.32193	-3.32193	-3.03451	-3.32193	-3.32193	-2.90806	-2.63528	-3.32193
MIR192	0.59563	-3.32193	-3.32193	-3.32193	3.5953	4.97676	1.45123	4.99954	-3.32193	-3.32193	-3.32193	-3.32193	-3.32193	-3.32193	-3.32193	-3.32193
ATG2A	4.59354	4.84586	4.67242	4.32779	4.63681	5.33174	3.91429	4.84636	4.18858	4.55829	4.69976	4.48042	2.15121	2.39027	3.04177	3.69248
PPP2R5B	3.55246	4.0521	4.31276	3.71807	4.0438	4.89301	3.72139	4.55833	2.93274	3.50328	3.41604	3.186	1.61057	2.32174	2.22201	2.48451
BATF2	0.94742	0.90808	0.44456	0.45909	0.86023	1.71065	0.37546	1.74238	1.51585	2.68751	2.391	2.2021	-3.0424	-2.27567	-2.29181	-1.80381
ARL2	5.68006	5.60379	5.7506	5.27946	6.37001	6.86917	5.78477	6.75377	6.38474	6.21309	6.47305	5.20508	5.50959	5.4237	5.44579	5.30496
ARL2-SNX15	5.78814	5.87968	5.95214	5.58445	6.40775	6.71802	5.8821	6.70084	6.13904	6.02148	6.20184	5.15738	5.28228	5.35152	5.50037	5.4908
SNX15	3.72489	3.92606	3.91504	3.74376	3.94399	4.50915	3.40327	4.61235	3.24756	3.24487	3.08885	2.73156	2.67457	3.09906	3.1324	3.29171
SAC3D1	5.1151	5.27209	5.12805	4.38854	5.49557	6.02282	4.85619	6.31465	4.17156	4.45798	4.34921	3.49963	4.09467	4.62347	4.76541	4.52149
NAALADL1	-0.52502	-0.38125	-0.73645	-1.44809	-0.14092	0.94412	-1.18319	1.02878	-1.12997	-1.63451	-1.842	-2.13697	-1.11763	-0.36925	-1.56403	-1.62262
CDCA5	4.76291	4.17788	3.99212	3.31617	5.14281	4.23826	4.73564	4.41851	4.98132	3.88393	4.24067	2.93982	4.58235	4.92239	4.42178	4.43271
ZFPL1	4.70345	4.68168	4.74342	4.36826	4.45033	5.16579	3.69254	5.00291	4.40124	4.54057	4.68623	3.70887	2.65596	3.0501	3.08929	3.30653
TMEM262	4.01461	3.99154	4.01722	3.70253	3.77659	4.47836	3.01753	4.35162	3.72007	3.86337	4.00104	3.00531	1.95211	2.37435	2.42361	2.57752
VPS51	5.08973	5.33266	5.21781	4.87502	5.28947	5.48611	4.65701	5.33186	5.69461	5.99346	5.96736	5.44426	3.14979	3.74441	4.42447	4.73861
TM7SF2	1.77608	2.53608	2.52754	3.66587	4.92428	6.40109	3.93339	6.26577	1.52579	2.69833	1.56297	2.40681	2.02176	2.38405	3.27953	4.01704
ZNHIT2	5.5041	5.62232	5.41123	4.64858	5.20154	5.758	4.24492	5.61092	4.25519	4.45882	4.78757	3.05748	2.90738	3.14103	2.7279	3.25593
FAU	8.04006	7.7294	7.74068	7.36173	7.75057	8.51459	7.33307	8.4824	7.96222	8.04035	7.89741	7.59341	7.70275	7.65337	7.18836	7.13821
SYVN1	5.69328	5.50259	5.55134	5.50571	6.20936	6.72266	5.88447	6.58178	5.74401	5.68284	5.73106	5.81092	5.65263	5.62768	5.38269	5.31128
MRPL49	4.24018	3.98417	3.97939	4.0512	5.27594	5.01931	4.85017	4.8754	4.91135	4.56203	5.02679	4.41203	4.77206	5.12182	4.79656	4.56424
SPDYC	-1.96221	-3.32193	-1.8081	-3.32193	2.02081	3.00332	1.631	3.20614	-3.32193	-3.32193	-3.32193	-2.00875	-3.32193	-3.32193	-2.51644	-3.32193
PGAM1P8	-0.83242	-0.18914	0.4329	1.09725	-0.29666	-0.58489	0.11747	0.51936	-0.74129	0.11848	-0.70763	0.17816	-1.70539	-2.30079	-0.58508	-0.36649
CAPN1	4.86996	5.25531	5.35082	5.21067	6.16982	5.53267	6.03862	5.38083	6.25614	6.26069	6.48049	6.1711	4.64938	4.85367	5.37712	5.67781
SLC22A20P	-2.08069	-1.75225	-1.70009	-2.52453	-1.86886	0.06444	-2.6937	-0.13419	-0.85467	-0.55111	-0.77481	-0.92998	-1.98475	-1.60748	-2.37317	-1.30669
POLA2	3.45913	2.93181	3.16416	2.70791	2.74353	2.03139	2.64653	2.36347	3.45975	2.11161	2.84899	1.77737	2.95535	2.90411	2.78264	2.88004
CDC42EP2	5.67878	5.83321	5.63314	5.15413	3.72914	4.12596	3.4568	3.98092	5.34853	5.19689	5.65394	4.46381	4.0279	3.54572	3.94109	4.14065
DPF2	3.58144	3.29267	3.37151	3.38195	3.2812	2.98581	2.97042	3.05246	3.89985	3.77819	3.97659	3.9383	2.80166	2.69702	3.03505	2.81773
TIGD3	-1.1611	-0.90789	-0.84425	-0.54717	1.78329	1.41906	0.78268	1.82419	-1.83985	-1.80598	-1.73791	-2.53001	-0.56878	0.31532	0.17787	0.90246
SLC25A45	-1.40868	-1.49382	-1.4374	-0.93211	2.40465	2.9786	1.93587	3.0372	-0.38436	0.23468	0.32956	0.40822	-1.0845	0.05798	-0.21071	1.05425
FRMD8	3.58209	3.69377	3.67032	3.39151	5.28363	4.70335	4.68706	4.77152	4.2694	4.51231	4.51341	4.33935	3.68061	3.81562	3.42389	3.86568
NEAT1	3.57807	3.81726	3.87177	4.57936	2.94613	5.87574	2.67819	6.00022	2.49252	3.30541	3.7615	3.35447	2.18651	3.06355	1.45567	2.43281
MIR612	2.65741	3.71884	3.21644	4.14861	2.13261	5.82272	-0.27847	6.32934	1.89084	3.05875	2.04266	3.27275	2.12308	2.99572	2.1846	4.51233
SCYL1	5.11314	5.06629	5.06478	5.01012	5.35805	6.00005	5.01934	5.74379	5.07482	5.03107	5.23829	5.09116	4.19745	3.99009	4.74797	4.52058
LTBP3	3.82102	4.14527	4.68764	4.0767	3.73269	4.4694	2.76496	4.33034	3.99603	5.13849	4.4445	4.74029	3.42409	3.4332	4.72913	4.95714
FAM89B	6.19981	6.90562	7.12646	6.45939	6.22533	6.42895	4.90272	6.33449	6.02662	6.3634	6.29085	5.19716	5.06448	5.38041	5.97655	6.4126
EHBP1L1	5.16758	5.9545	6.0993	5.59732	4.89775	5.25597	4.859	5.0163	4.78177	5.53906	5.47589	5.36893	3.29624	4.31536	4.88449	5.32548
KCNK7	-0.08291	-0.37931	0.13761	0.14139	0.1259	1.10998	-0.5093	1.03493	-2.21467	-2.03712	-1.70952	-2.76539	-2.20619	-2.20896	-1.21136	-1.76695
MAP3K11	4.1333	4.3289	4.32173	3.74614	5.31883	5.19737	4.61674	5.14865	4.36961	4.24104	4.60529	3.99628	3.24779	3.14131	4.32647	4.33139
PCNX3	4.56735	4.55832	4.59109	4.00032	5.05825	4.54231	4.21035	4.64697	4.74328	4.67953	5.02525	4.44918	2.92407	3.26531	3.5936	4.29523
MIR4690	5.65958	5.75005	5.47709	4.41471	4.63353	4.64093	2.30152	6.29583	4.0565	2.92298	3.24491	-3.32193	2.05435	2.84166	0.41684	2.68068
SIPA1	5.22085	5.72807	5.76884	5.16284	4.70791	5.15872	3.76247	4.972	5.66102	5.29999	5.62779	4.52565	3.67513	3.57423	4.42508	4.38771

MIR4489	3.91855	4.66623	4.27647	3.67155	2.91515	4.66752	-3.32193	5.27842	2.56564	1.89582	2.71896	2.593	0.11197	0.1073	1.88186	2.89328
RELA	4.62322	4.7079	4.83063	4.31573	4.71001	4.2631	4.26149	4.21852	5.2476	5.11283	5.18941	4.84167	4.29578	4.10419	4.529	4.25476
KAT5	3.87766	3.69615	3.73667	3.53919	4.24062	4.16604	3.97871	4.22123	3.92217	4.18337	4.44589	3.77437	3.34376	3.91498	4.14501	4.07928
RNASEH2C	5.0912	4.87488	4.90307	4.56601	4.55485	4.66199	4.04627	4.63197	4.45866	4.23306	4.32343	3.7154	3.73942	3.67261	3.8531	3.72371
AP5B1	4.20747	4.29818	4.30916	3.64179	4.99258	4.13227	4.2626	4.04823	3.69846	3.64546	4.08426	3.31009	2.69934	2.22386	3.44586	3.26906
MUS81	4.07491	4.01215	4.12893	3.55871	4.23376	4.11237	3.53399	3.9875	3.46668	3.3408	3.3361	2.88932	3.76692	3.6106	3.4262	3.12836
EFEMP2	-0.47721	0.24054	1.31335	0.49272	0.20225	1.98297	-0.9542	1.34819	1.20566	2.65504	2.14898	2.2434	-2.02057	-1.8976	-2.25264	-2.44065
CTSW	2.85472	4.07671	3.44691	4.06057	-1.59901	-2.07735	-1.89659	-1.44024	-1.69003	-1.30485	-1.0765	-0.34956	-2.27848	-0.3131	-1.87606	-2.41028
FIBP	4.33238	4.5015	4.42926	4.27693	4.27414	4.91855	3.85912	4.78967	5.57414	5.53792	5.51058	5.06126	3.30896	3.69056	3.58637	3.76876
CCDC85B	7.11817	7.75179	7.71963	7.31369	6.34968	6.63703	5.5032	6.72684	8.39474	8.63654	8.80567	7.13386	6.43092	6.45876	6.57517	6.74078
C11orf68	5.91417	5.80476	5.93153	4.85401	4.55558	4.47552	4.04926	4.28119	6.82745	7.13067	7.24619	5.90017	5.26141	4.89181	4.51238	4.40132
SART1	5.90584	5.73765	5.69151	5.23736	5.59704	5.43454	5.32259	5.56026	5.86597	5.44186	5.7824	4.90214	4.62561	4.74106	5.18264	5.17547
EIF1AD	3.77121	3.57712	3.7932	3.67391	3.74478	3.67155	3.7258	3.84694	3.36012	3.28546	3.41422	3.19994	3.86898	4.4212	3.95743	3.9553
BANF1	6.98511	7.23407	7.21544	7.00577	6.69537	7.49976	6.68558	7.40146	6.3706	7.31758	7.47025	6.27488	5.46586	7.32509	6.51211	6.69298
CATSPER1	1.92494	1.75463	1.78808	1.40732	-2.08963	-2.95228	-2.46685	-2.07448	1.68812	2.20545	1.48182	1.2631	-1.95232	-1.11788	-1.21448	-1.10026
SF3B2	6.06693	5.86717	5.81752	5.8494	5.16547	5.10554	5.03036	5.09424	5.71794	5.28875	5.72353	5.42874	4.83225	4.81259	5.47719	5.17956
PACS1	3.09607	3.2031	3.29526	2.93264	3.66574	3.46367	3.52553	3.37379	5.40485	5.34448	5.34051	5.38801	3.06622	3.40676	4.13263	4.14705
KLC2	5.1629	4.96563	5.00842	4.40972	4.82199	4.5912	4.25656	4.71695	4.68111	4.22109	4.65076	3.46875	3.99725	4.08718	4.39199	4.26541
RAB1B	7.60275	7.71962	7.56915	6.98899	7.60001	7.33295	6.86702	7.14408	7.89246	7.79789	7.98163	6.80291	6.6833	6.70929	6.94868	7.13386
CNIH2	0.60924	1.43852	1.79806	2.66695	0.58686	1.462	0.28745	1.66838	-1.46748	-1.50141	-0.2544	-1.13413	0.41607	1.95514	0.94601	1.9388
YIF1A	5.14976	5.17036	5.16005	4.93396	7.00061	7.4031	6.28177	7.25867	5.59932	5.68854	5.74687	5.49652	4.74317	4.92811	4.83552	4.94088
TMEM151A	-3.32193	-2.57344	-2.26765	-3.32193	4.56891	5.02125	3.28848	5.02841	1.88425	2.61985	2.22706	1.82626	-2.04456	-1.29539	-0.62252	-0.96051
RIN1	4.36403	4.35111	4.39566	3.85657	2.31496	3.10337	1.55326	2.62285	5.31664	5.98372	5.7052	4.98649	2.94572	3.21472	2.80943	3.71498
BRMS1	5.36749	5.04064	4.9425	4.90088	5.40858	5.42795	4.72616	5.47791	5.12251	4.97487	5.16153	4.33253	4.82769	5.05054	4.64252	4.98655
B4GAT1	4.99046	5.09932	5.15925	5.17899	6.07622	5.78671	5.7184	5.98154	5.16722	5.66404	5.65949	5.71555	4.34475	4.6463	4.77353	5.22719
SLC29A2	-1.60704	-0.56389	-0.64188	-1.50575	5.23384	5.62404	4.30619	5.80727	2.73703	2.85876	2.57355	1.98846	2.46782	2.96477	2.85007	3.1217
MRPL11	5.10294	4.66011	4.54085	4.25734	5.02254	4.70384	4.43581	4.70374	5.34107	5.05321	5.27431	4.03947	4.57919	4.59558	4.4179	4.22835
PELI3	2.8553	2.87885	2.77013	2.34801	2.99098	2.11682	2.07554	2.32603	2.29351	2.6931	2.35541	2.17123	1.27686	1.02079	1.70133	1.86776
DPP3	3.97561	4.36932	4.22051	4.24761	5.16011	5.22923	4.69152	5.09191	4.39019	4.29663	4.70589	4.1193	4.14546	4.20135	4.55013	4.53609
BBS1	1.49338	1.19101	1.52014	1.43238	1.30191	1.46933	0.4943	1.39164	2.02803	2.51465	2.49429	2.72017	0.71685	0.59447	1.02959	0.95343
ZDHHC24	2.90823	2.86818	2.81877	2.42057	3.48334	4.13608	2.57576	4.08604	2.86477	3.30482	3.23897	2.66869	1.6584	1.94184	1.93104	2.39209
CTSF	1.54299	1.45789	1.5798	1.60568	5.11455	4.90237	4.3138	4.65457	3.24908	4.45531	3.85333	3.93478	2.63453	2.76884	2.80911	3.38994
CCDC87	-2.98063	-2.9439	-1.78729	-1.57412	-0.16254	0.06276	-0.51889	-0.16228	-0.24377	0.03774	0.79181	0.99826	-2.81789	-1.90684	-0.97794	-1.02716
CCS	4.0829	4.16324	3.95296	3.74912	4.51705	5.30357	3.66248	5.32417	3.76467	4.02541	4.19114	2.95291	3.16152	2.91548	3.01205	3.15157
RBM14	5.3508	4.85895	4.81742	4.28808	4.99995	4.26263	4.62874	4.33923	5.54062	4.44182	5.07442	4.14146	4.72759	4.57097	4.9553	4.77094
RBM14-RBM4	5.69436	5.68977	5.5409	5.48824	5.93491	5.78621	5.71585	5.5521	6.63132	6.28146	6.56144	5.97592	5.82357	5.76579	5.91506	6.00054
RBM4	4.5147	4.59186	4.44415	4.46653	4.64725	4.79484	4.42176	4.54841	5.40777	5.21929	5.42796	4.94956	4.65641	4.63557	4.62669	4.77275
RBM4B	2.2796	2.273	2.34335	2.63084	1.90713	2.03905	1.86171	2.00214	2.53984	2.08261	2.47166	2.44406	2.10552	1.75017	2.06269	1.72451
SPTBN2	3.17161	3.15687	2.53502	2.41492	2.35459	1.49697	1.84983	1.9816	-1.7492	-1.46549	-1.60477	-1.9376	2.07925	2.18566	2.95828	3.19406
C11orf80	2.35892	2.49901	3.03275	2.88997	2.91153	3.3129	2.91952	3.28693	1.17408	1.66049	0.98494	1.62758	2.31533	2.56298	2.67318	2.66818
C1QBPP2	0.9866	0.83952	0.91322	1.16201	1.36578	-0.06484	1.02283	0.83059	-1.22416	-0.6342	-1.34972	0.11238	0.58527	0.50781	0.34214	1.08386
RCE1	4.34432	4.26156	4.37371	4.35388	4.53954	4.65987	3.96619	4.72031	4.36334	4.06113	4.32137	3.52032	3.54246	3.57458	3.77952	3.9064
PC	3.27775	3.62391	4.14252	3.42348	4.1979	4.82275	3.66003	4.57414	4.54297	4.90816	4.8166	4.08599	2.37812	2.30606	2.56096	3.06924
LRFN4	-0.3472	1.00706	1.38048	1.02868	-1.11614	-1.03365	-1.97878	-0.12649	5.61573	6.25444	6.38641	4.51834	-2.8059	-2.27146	-2.48514	-2.47117
C11orf86	-2.11547	-0.70435	-0.63881	-0.56108	1.57919	2.42994	1.53979	0.97561	-2.50635	0.68994	-1.72164	-0.245	-2.30437	-2.72698	-3.32193	-1.49318
SYT12	-2.73538	-2.40752	-2.13699	-1.97825	-1.47913	-2.16374	-1.55292	-1.71515	-3.32193	-3.22552	-3.32193	-2.82867	-2.73189	-2.90437	-2.56985	-2.05968
RHOD	-3.32193	-3.32193	-3.32193	-2.49449	5.29513	5.50608	4.79203	5.76341	3.4134	3.41021	3.7007	2.63392	4.43199	4.49892	4.47523	4.4952
KDM2A	3.62702	3.39205	3.38739	3.51436	3.75904	3.10642	3.78301	3.16885	4.19712	4.15796	3.99178	4.59778	3.4008	3.55033	3.88322	4.15512
GRK2	3.20078	3.17631	3.29863	2.79403	4.30152	4.51837	3.8722	4.40087	4.16071	4.17703	4.36425	4.02631	3.05494	3.74211	3.48055	3.64884
ANKRD13D	3.33307	3.24029	3.43104	3.21775	3.09512	4.30768	2.30831	4.54267	2.43559	2.73241	2.83674	2.42844	1.9964	2.27578	1.72794	2.40882

SSH3	2.29165	2.5987	3.156	2.87552	4.95369	5.40329	4.06129	5.27288	2.97819	3.59498	3.81537	3.17823	2.30048	3.04213	3.60508	3.59724
RAD9A	4.33936	4.12631	4.16178	4.02822	4.55285	5.22303	3.9095	5.37527	4.26195	3.9694	4.13209	3.44402	4.22799	4.55801	4.25016	4.20404
POLD4	4.57126	4.91029	5.2743	4.92765	5.2165	5.96902	4.46458	5.48309	4.848	5.55256	5.49378	4.84153	3.94818	4.13966	3.50492	4.25573
CLCF1	3.69491	3.64827	3.52435	3.88114	2.51713	3.35498	2.2726	3.4209	2.45679	2.23973	2.50518	2.30538	3.35021	3.16933	2.59168	2.82364
PPP1CA	6.41349	6.62802	6.65631	6.31749	7.15263	7.26193	6.81614	7.1086	7.40467	7.13534	7.39376	6.73123	6.56705	6.697	7.09036	6.96298
TBC1D10C	-3.11348	-2.8901	-3.32193	-2.65409	-1.51102	-1.11568	-2.21038	-0.10163	-2.69709	-1.69036	-2.60462	-1.85293	-2.87268	-1.76538	-2.79063	-2.38825
CARNS1	-2.25494	-1.89432	-2.22758	-2.17814	-1.23259	-0.91857	-1.98094	-0.41266	-2.92335	-2.7103	-2.43377	-1.82793	-2.70258	-2.60724	-2.8447	-2.18298
RPS6KB2	3.87879	3.61994	3.58405	3.23621	4.4605	4.60172	3.75507	4.46959	4.10749	4.07906	4.00949	3.36967	3.33104	3.55524	3.65955	3.55694
RPS6KB2-AS1	6.06918	5.71569	5.83365	5.66552	6.66151	6.78302	6.3782	6.6449	6.34504	6.27592	6.1723	6.01338	5.88174	6.11259	5.99536	5.83658
CORO1B	5.42652	6.00053	6.09923	5.8511	5.9877	6.91054	5.50509	6.81174	5.97449	6.26295	6.25514	5.873	3.8321	4.59085	4.59234	4.93869
PTPRCAP	2.06518	2.65605	2.55203	2.64214	1.85628	3.28645	1.32834	3.2953	1.30686	1.52631	1.50493	2.14665	-0.49046	1.31706	-0.57018	0.69969
TMEM134	1.99966	2.45462	2.70007	2.07291	2.29113	3.34037	1.54385	3.29481	2.44455	3.16039	2.84168	2.16449	0.78257	0.93341	0.68632	0.90606
AIP	6.13703	5.97097	6.02416	5.68255	5.43471	6.19402	5.22773	6.06545	5.36425	5.27792	5.61695	4.77015	5.01594	5.10053	5.0715	4.91479
PITPNM1	4.32146	4.61522	4.67043	4.03042	5.2754	5.24435	4.43305	4.92548	5.06931	5.2436	5.17314	4.5419	3.56309	4.31022	4.5794	5.27789
CDK2AP2	5.51989	5.44311	5.46398	5.12492	6.08399	7.3823	5.24352	7.16888	6.34733	6.21046	6.40127	4.80057	4.61611	5.04654	4.66812	4.61152
NDUFV1	6.0626	5.95579	5.88969	5.57512	5.51195	5.98904	4.81116	5.94722	6.03225	5.85901	6.04237	5.28917	4.57984	4.56497	4.90073	4.65989
DOC2GP	-3.00381	-2.68613	-2.95202	-2.93078	-0.4158	1.27484	-1.24334	0.984	-2.95819	-1.67069	-2.99359	-3.32193	-2.49566	-3.06784	-3.32193	-2.34888
NUDT8	1.85816	1.93385	2.11736	0.97079	3.80274	4.27887	2.5835	4.21432	2.9533	2.69893	2.93022	1.60551	1.65557	1.38163	2.07706	1.71528
RPL37P2	2.65988	1.64859	2.83761	1.50135	0.32021	1.01002	-0.22216	0.77251	0.74128	2.33761	1.04335	1.54596	1.14964	-0.4475	-0.19336	0.20863
ALG1L8P	-0.71168	-1.17181	-0.27594	-0.42548	0.8058	2.04503	0.19983	2.08058	1.08265	0.90501	1.92702	0.88173	1.12441	0.69124	1.34341	0.25848
FAM86C2P	-0.48203	-0.70811	-0.7048	-0.9908	0.805	1.32129	0.08173	1.18319	0.9875	0.54986	1.06019	0.43763	0.64669	0.60856	1.04287	-0.01374
UNC93B1	4.17642	4.18354	4.48629	3.73035	4.26171	4.98565	3.60497	4.91662	5.64714	5.96471	5.73567	5.65203	2.76932	3.00114	3.69889	3.73946
ALDH3B1	1.45796	1.86778	2.07774	2.25925	0.74157	1.39097	-0.00497	1.13079	3.39444	4.37486	4.09387	4.21633	1.99669	2.28483	2.90322	2.71331
NDUFS8	5.55133	5.38335	5.39505	5.06061	4.92333	5.73588	4.00047	5.6661	5.29912	4.91324	5.17734	4.59546	4.32647	3.9695	4.00366	3.92163
MIR7113	5.44328	4.56825	5.7303	3.55145	5.62246	7.11294	3.89092	7.52924	2.9533	3.52562	2.47273	3.14231	3.86076	3.85564	2.3572	2.96389
TCIRG1	6.14692	6.18086	6.28998	5.97346	5.29573	6.50599	4.14831	6.13638	5.23238	6.03569	5.86975	5.82762	3.16837	3.33512	3.43511	4.01343
MIR6753	4.83262	4.58513	4.21738	3.27184	3.12805	3.47316	2.02705	2.65345	2.48563	2.79602	2.98987	3.03129	1.78105	1.77606	1.70612	2.37296
CHKA	4.31168	4.3812	4.44629	4.4468	3.9324	4.82143	4.38398	4.6808	1.00579	1.70745	1.58656	1.40217	3.46965	3.75409	4.20691	3.77575
KMT5B	1.7501	1.34034	1.87935	2.32618	2.65042	2.21297	3.24791	2.05047	2.17657	1.98486	2.30552	2.91151	2.3882	1.97021	2.42283	2.76813
C11orf24	4.16345	3.87813	3.91225	3.58607	5.97251	5.88066	5.44829	5.86047	3.92574	3.11596	3.2861	2.61516	3.71696	3.41498	3.38922	3.22848
LRP5	4.99345	4.62918	4.69358	4.21738	6.42479	6.07917	5.66149	5.8947	4.1112	3.82869	4.12715	3.10412	3.49395	3.2096	4.27936	4.533
PPP6R3	3.31603	2.9883	3.26294	3.2321	4.29333	3.38623	4.6676	3.25184	4.36288	3.3622	4.33889	4.15817	4.21047	2.6639	4.46264	3.43504
GAL	-2.64655	-1.73203	-1.38489	-1.31664	1.31233	0.17142	1.21978	-1.38068	-2.56154	-2.66593	-3.32193	-3.32193	-3.32193	-3.32193	-3.32193	-3.32193
TESMIN	-0.96444	0.13829	-0.1457	-0.53973	0.55894	0.62244	0.04475	0.36395	1.63245	1.759	1.09762	1.16629	-0.13119	0.49442	-0.11133	0.26746
CPT1A	1.60583	1.53595	1.68226	1.35755	6.02426	5.5547	6.19568	5.71211	4.20085	3.58926	4.12041	4.62906	3.8085	3.13189	3.9068	3.92286
MRPL21	4.75616	4.5867	4.45976	4.29471	4.66456	5.37271	4.30333	5.2444	4.56287	4.599	4.55251	4.10528	4.15317	3.94406	2.99915	3.13969
IGHMBP2	3.07373	2.82918	2.72551	2.66537	3.21293	3.17334	2.70261	3.28788	2.66073	2.43966	2.68763	2.41243	2.04122	2.0362	1.95694	2.23031
MRGPRF	-3.32193	-3.32193	-3.32193	-3.32193	1.09804	0.06078	-0.04302	0.7193	2.57234	2.89893	3.20256	1.62017	-3.32193	-2.58909	-3.32193	-3.32193
MRGPRF-AS1	-3.32193	-3.32193	-3.32193	-3.32193	-2.77326	-2.52749	-3.32193	-3.32193	-0.14867	-0.52415	0.38289	-2.2984	-3.32193	-3.32193	-3.32193	-3.32193
TPCN2	0.78471	0.77364	0.97078	0.52953	3.04803	2.58102	2.76105	2.85894	1.94339	1.83366	2.23907	2.08258	0.36571	0.09258	0.94886	1.11258
MYEOV	5.63102	5.29733	5.36314	4.76278	2.26533	0.13981	2.28069	-0.53694	6.1662	5.5477	6.08865	4.97579	2.28885	1.58517	1.11722	0.99528
LTO1	4.08233	3.73115	3.56546	3.71767	3.79888	4.11066	4.13407	4.17889	5.18198	4.015	4.08168	4.26195	5.09989	4.85455	4.38759	4.16803
FGF19	-3.32193	-3.32193	-3.32193	-3.32193	-1.4903	0.99424	-1.13519	0.25583	-3.32193	-3.32193	-3.32193	-3.32193	-2.73989	-3.32193	-3.32193	-2.37441
ANO1	-3.14701	-3.32193	-3.32193	-3.32193	-1.39896	-3.14518	-1.50358	-3.32193	-2.00098	-1.93462	-1.07736	-2.01953	-3.32193	-3.32193	-3.15448	-3.32193
FADD	5.0926	5.28172	5.14816	5.10323	5.40017	5.12615	5.09834	4.96294	5.41559	5.20358	5.31938	4.86169	5.01501	5.10951	5.07333	4.87532
PPFIA1	3.16841	3.09829	3.11667	3.55162	3.37514	2.46476	3.64182	2.468	3.01914	2.67146	2.98823	3.30419	3.18651	2.78566	2.95209	2.85925
CTTN	4.99133	5.22378	5.29474	5.16302	5.79216	5.40489	5.6682	5.13302	6.11819	5.81805	6.01973	5.69727	5.71698	5.17844	5.6535	5.57912
SHANK2	-3.26296	-3.19278	-3.25233	-3.24788	1.59026	1.77381	1.18455	1.78424	-0.36954	-0.4509	-0.03951	-0.77969	-3.32193	-3.27569	-3.05991	-2.96019
DHCR7	4.94003	4.75981	4.67846	4.36391	6.70267	7.60115	6.14436	7.8582	5.87463	5.33432	5.34307	4.35079	5.32622	6.1092	6.29744	6.49162
NADSYN1	1.35487	1.4404	1.64898	1.31716	1.62835	2.13883	1.09175	2.19668	2.4529	2.54335	2.6855	2.72963	1.08588	1.00802	1.0325	1.0267

FAM86C1	2.10582	1.99941	1.73014	1.28726	3.1584	3.20721	2.67561	3.24966	2.22759	1.44304	1.90771	1.55924	1.83915	1.51202	1.48168	1.56278
ALG1L9P	2.72313	2.37228	2.65465	1.94402	3.9117	4.23454	3.0499	4.05123	3.36289	2.36287	2.97487	1.84176	2.28166	2.37198	2.98704	1.93541
OR7E126P	0.20015	0.23768	0.63194	1.20583	-0.52717	0.21465	-0.76243	1.59013	-3.32193	-3.32193	-2.39182	-3.32193	-2.56942	-0.97321	-1.51318	-0.70899
RNF121	3.56676	3.45518	3.5065	3.30028	3.685	2.98764	3.56798	2.92205	2.77134	2.66255	2.94412	2.78803	2.52589	2.91773	2.71384	3.01501
IL18BP	4.37853	3.95563	4.09186	4.05307	3.09564	4.02123	2.51451	3.94186	4.16864	3.19172	3.8181	2.77753	2.95708	2.96241	3.67436	3.64635
NUMA1	4.26932	3.97253	4.09455	3.92216	3.93315	3.74843	3.64234	3.6027	5.1536	4.23571	4.75389	4.12915	4.01574	3.9654	4.99298	5.13253
LRTOMT	3.05501	2.84299	2.88231	2.84727	3.01298	3.28435	2.75748	3.36062	3.03134	2.58685	2.84653	2.12934	2.54105	2.72859	2.50385	2.58017
LAMTOR1	5.51573	5.56835	5.45037	5.48951	5.10595	5.57924	4.74861	5.42179	5.21035	5.04415	5.15194	4.72535	4.97967	5.11281	4.41498	4.68527
ANAPC15	4.12184	3.91524	3.88781	3.83163	3.84716	4.26564	3.46021	4.42553	3.96826	3.43233	3.67053	2.77346	3.23925	3.38398	2.81023	3.01549
FOLR3	-0.49095	0.78445	1.00728	1.04626	-3.01611	-2.67843	-3.32193	-2.10623	-1.24089	-0.91896	-0.69352	-1.45988	-3.32193	-3.32193	-3.32193	-2.69662
INPPL1	5.09682	5.36425	5.4899	5.35527	5.24455	5.38576	4.60116	5.28484	5.09058	5.22364	5.45965	5.2696	4.39566	4.73318	5.12818	5.30283
CLPB	3.71306	3.7085	3.39443	3.33562	4.11206	4.01981	3.82974	3.69837	3.8247	3.69465	4.04973	3.60628	3.21463	3.1917	3.52574	3.57864
LINC01537	2.57544	2.83594	2.24906	2.49025	-3.32193	-2.92107	-3.32193	-3.32193	-2.01574	-0.26337	-1.22375	0.44499	-2.74048	-3.00313	-3.32193	-3.32193
PDE2A	-0.1876	0.9315	1.47288	0.00055	-2.92	-2.41574	-2.94052	-2.85142	-0.70134	0.3055	0.17622	0.60785	-3.32193	-2.73106	-2.72178	-2.43936
ARAP1	4.89164	5.10201	4.94265	4.54803	4.10677	4.64256	3.62804	4.35017	4.43743	4.77202	4.97127	4.59241	2.69714	3.33656	3.91633	4.34264
ARAP1-AS1	7.07169	7.10097	7.0863	6.88605	5.63062	7.00592	5.3638	6.75643	5.74978	6.00314	6.1279	5.86112	4.58014	5.06006	5.03376	5.73323
ARAP1-AS2	3.40155	3.66227	3.61458	2.6987	3.088	3.03352	1.99726	2.52815	3.48269	3.89843	4.21827	3.11296	0.92608	2.14344	2.57289	2.97543
STARD10	3.65967	3.86461	3.81307	3.62441	6.1133	6.15087	5.40567	6.154	2.56158	3.25565	2.89844	2.96596	1.62499	2.34902	2.80439	2.98109
ATG16L2	1.54555	1.81433	2.2225	1.601	1.36025	1.56977	0.6286	0.942	1.63929	1.6467	1.83254	1.8311	1.03884	0.86601	0.79399	0.54831
FCHSD2	2.47518	2.53411	2.63976	2.74629	2.58402	1.58694	2.55268	1.71591	3.49804	3.11585	3.42791	3.78657	3.13111	2.84339	2.7065	2.36899
P2RY2	3.33385	3.64649	3.49213	3.11461	1.25169	0.35547	0.76153	-0.04219	0.41115	0.8896	0.50672	1.84699	-2.61037	-1.58664	-0.88816	-1.35402
P2RY6	4.16365	4.44707	4.32775	4.7567	-0.05561	-0.3031	-0.33571	-0.55547	0.97124	1.78682	2.77695	3.2452	-2.93694	-2.27462	-3.32193	-1.98626
ARHGEF17	2.75606	2.72293	3.1445	2.45744	0.85637	0.39781	-0.61172	0.72105	3.83938	3.87708	4.23226	3.68628	1.14811	1.04447	1.54972	1.71192
RELT	3.72638	3.26625	3.42968	2.59722	2.40835	2.57499	1.71621	2.49792	2.73331	2.73517	2.57292	1.71246	1.83674	1.33607	1.35032	1.21185
FAM168A	2.06821	2.41857	2.60147	2.68885	2.89184	2.3234	2.92687	2.09314	4.0275	4.35299	3.78135	4.77736	3.13307	3.67179	3.70768	4.2049
PLEKHB1	-2.14646	-1.85418	-2.11731	-1.48116	0.4253	1.06242	-0.18711	0.82006	-1.64633	0.01477	-1.53751	0.30281	-2.52321	-3.00504	-1.49264	-1.19173
RAB6A	4.66315	4.93445	4.97215	5.27212	4.80489	4.37237	5.23363	4.14805	5.30895	5.21008	4.90155	5.7299	5.02682	5.2635	4.99609	4.91295
MRPL48	2.56604	2.3907	2.38853	2.21249	3.03059	3.44236	3.36105	3.37448	1.7082	1.69586	1.36442	1.46845	2.44289	2.31505	2.00704	1.38068
COA4	5.33229	4.82653	4.60744	4.74637	5.36996	5.7392	5.12532	5.56202	4.69413	4.47848	4.50387	3.94162	5.41379	5.16902	4.08678	4.11535
PAAF1	1.3585	1.4159	1.50285	1.57947	1.65647	1.79962	1.42882	1.78644	1.7926	1.51743	1.46684	1.33312	1.72008	1.70836	1.5686	1.5149
UCP2	-1.19868	-1.43786	-1.38061	-0.77525	4.29431	4.86262	3.4267	4.95615	2.15303	1.06237	0.45075	-0.6832	3.40346	3.98729	3.84183	3.91181
UCP3	-1.8968	-1.91309	-1.864	-2.10174	-1.9879	-1.40772	-2.48471	-0.31902	-2.5273	-2.31061	-2.79938	-3.32193	-1.11059	-1.5984	-1.17808	-1.1536
C2CD3	1.51899	1.15086	1.07323	0.90244	2.03054	1.25001	2.06754	1.39292	2.14409	1.00796	1.49804	1.51138	2.03904	1.8706	2.24458	1.56355
PPME1	3.72322	3.73018	3.54731	3.79817	3.19071	3.17639	3.356	3.22621	4.14516	3.30373	3.44159	3.30538	4.17993	4.67153	4.52668	3.90459
P4HA3	0.8196	1.19827	1.01905	1.08982	1.01131	0.2818	1.61524	0.47454	1.76888	0.80141	1.21793	1.45564	2.2452	2.68532	2.51576	1.85358
PGM2L1	0.44184	1.58676	1.04986	2.37201	1.35563	1.11377	2.54634	1.46931	0.98695	1.77664	1.46561	3.14715	2.93001	4.61913	4.55446	5.3624
LIPT2	1.57384	1.73513	0.912	0.73838	1.69507	2.37585	0.99424	2.60603	1.05382	0.84989	1.09224	1.22123	0.03145	1.48812	0.88592	1.3721
POLD3	2.63912	2.10136	2.09539	2.0674	2.42232	1.83268	2.77001	1.92111	2.95092	1.83934	2.54875	2.04758	3.23527	2.70816	2.70722	2.11152
RNF169	1.66699	1.9206	1.74664	1.94759	2.22002	1.1996	2.60244	1.27776	2.83245	3.11634	2.95354	3.73692	2.4741	2.25868	2.61676	2.51727
XRRA1	1.94483	1.91167	1.86471	2.06588	0.93925	1.02473	1.27991	1.02895	1.40692	1.59258	1.55171	2.31699	1.27627	1.13781	1.66286	1.31268
SPCS2	5.68367	5.6869	5.75815	5.86025	5.83775	6.00576	6.22742	5.71281	5.54967	4.89069	5.11093	4.77291	5.08957	4.56365	4.5258	4.1059
NEU3	1.12093	0.91608	0.72912	1.12081	2.16063	1.20269	2.02482	1.24634	1.94892	1.642	1.7829	1.77829	1.82543	1.74306	1.43232	1.45219
SLCO2B1	1.79976	1.51196	1.67013	2.41277	4.02561	2.8071	3.63763	2.5313	-2.4865	-2.03147	-2.62469	-1.04741	-3.32193	-3.32193	-3.22218	-3.22005
TPBGL	-2.68431	-1.39325	-2.10977	-1.68744	0.3827	0.18846	-0.36885	-0.01102	0.26086	1.00181	-0.23709	-0.00627	-3.32193	-2.80204	-1.08489	-3.32193
ARRB1	2.69871	2.79614	2.31473	2.53583	4.06616	3.17854	4.09846	2.98636	3.21159	4.03528	3.66937	4.13109	0.88966	1.71419	1.97345	2.49471
KLHL35	-3.32193	-3.09196	-3.32193	-3.32193	-0.71305	-1.33728	-1.80174	-1.15864	-1.75217	-1.28007	-1.62909	-1.94037	-3.32193	-3.15819	-3.32193	-3.11999
GDPD5	3.10484	3.43064	3.52083	2.91787	0.36281	0.28849	-0.12817	0.4468	0.31367	1.57275	0.86341	1.03821	-2.6293	-2.20645	-1.95619	-1.14212
SERPINH1	5.61187	5.64034	6.13019	6.11533	4.52359	5.97109	4.63022	5.77373	5.92757	5.93805	6.59763	6.20926	2.24114	2.54428	2.79306	3.08747
MAP6	-2.26131	-1.10278	-0.73633	-0.6947	-2.67703	-1.70195	-3.16797	-2.12564	-2.96526	-2.46246	-3.15203	-2.3846	-3.07214	-3.32193	-3.16464	-2.76519
DGAT2	2.44128	2.26137	2.30416	2.22068	2.30118	1.89658	1.88103	2.28921	-0.55212	0.59519	0.0075	-0.16628	-0.46787	-0.66923	-0.59416	-0.11407

UVRAG-DT	1.02338	1.06901	0.97666	0.59695	2.14407	0.67018	1.87208	0.3212	1.74479	1.80076	1.72855	-0.55941	1.33945	1.56492	1.90376	1.98962
UVRAG	2.35666	2.74636	2.50781	2.73788	2.58001	1.86613	2.93981	1.89479	2.14323	1.99145	2.07942	2.53289	2.43888	2.70681	2.35508	2.66087
WNT11	-1.47955	-2.0284	-2.85417	-2.16864	-1.26619	-0.8428	-1.99501	-0.48366	-0.59162	0.09261	-0.44174	-1.41198	-1.40138	-0.61011	-0.41868	0.06535
THAP12	2.49426	2.45354	2.20476	2.54068	2.88063	1.83721	3.4414	1.95426	3.51998	3.64916	3.48439	4.03156	3.28151	3.31102	2.84536	2.94603
GVQW3	-2.05006	-2.13903	-2.0949	-1.79824	-1.81982	-1.8427	-1.95194	-1.6495	-2.7434	-1.99963	-2.65633	-2.5248	-2.24582	-1.69043	-1.38214	-1.76964
EMSY	0.91458	1.02118	0.96882	1.27991	0.60511	0.45629	1.0818	0.65339	1.35913	0.76065	1.19556	1.85669	1.15781	0.90608	1.35197	1.15132
TSKU	4.23938	4.2491	4.56508	4.11626	4.23046	3.99995	3.11406	3.85931	4.49363	4.61064	4.93996	4.26786	1.71983	2.10744	2.74438	3.06185
ACER3	2.12929	2.33126	2.23822	2.71724	3.051	2.91207	3.68396	3.11835	2.69115	2.48681	2.35858	3.67888	2.96471	1.99633	2.53303	1.9348
CAPN5	2.03658	2.82358	2.60756	2.9078	1.34047	2.06968	0.88803	1.65038	3.08215	3.71811	3.34204	3.65459	1.20102	0.93204	2.03627	2.47666
MYO7A	-3.32193	-3.32193	-3.19676	-2.85068	-1.47422	-0.99361	-1.54457	-1.19537	-0.36621	-0.82429	0.12519	-0.14398	-3.32193	-3.32193	-3.03608	-2.94494
GDPD4	-3.32193	-3.32193	-3.32193	-3.32193	-0.2617	1.88508	-0.12329	1.89512	-3.32193	-3.32193	-2.99746	-3.32193	-3.32193	-3.32193	-3.32193	-3.32193
PAK1	1.71863	1.90727	1.91783	2.19525	2.60312	1.51885	2.55425	1.26355	1.12212	1.70508	1.4282	2.17853	0.77571	1.74461	1.95025	2.15077
CLNS1A	5.16989	4.91767	4.94843	4.83392	3.95727	3.72883	4.16284	3.5899	4.9003	4.75794	4.81772	4.93277	3.9798	3.75966	3.67621	3.40012
AQP11	1.52895	1.51761	1.47836	1.8709	2.74786	2.87538	2.655	3.04078	1.3057	1.67462	1.76066	2.36927	0.76448	1.30856	1.38131	1.66276
RSF1	2.4747	2.5526	2.69451	3.54265	2.07555	2.33787	2.67935	2.60597	3.31088	3.10859	3.32773	4.08254	2.50346	2.29391	3.1375	2.95794
FTH1P16	5.38746	4.54216	5.13943	3.667	1.16974	2.95913	0.35749	2.0404	1.6804	3.06531	1.59124	2.67007	-0.02737	0.61057	-0.99218	0.55786
AAMDC	3.20279	3.39252	3.46975	3.4781	2.87321	3.83957	2.57383	3.88564	3.24471	3.00626	2.818	1.98639	3.50264	3.72888	3.11148	3.48161
INTS4	2.66928	2.64777	2.52269	2.71908	2.73691	2.77481	2.91962	2.60597	3.04508	2.46183	2.84699	2.81327	2.25244	2.0771	2.36475	2.58748
KCTD14	1.26767	1.73395	1.04723	1.51598	4.05072	4.03131	4.15271	3.72226	3.52211	3.50878	3.49267	4.07987	2.97071	3.60188	3.03188	3.4233
NDUFC2-KCTD1	7.68101	7.65563	7.58171	7.52786	8.70472	8.66143	8.68856	8.52885	7.65851	7.38133	7.51952	6.6598	7.51648	7.57931	6.9624	7.00427
NDUFC2	6.03554	6.00049	5.94908	5.87878	7.00262	6.99684	6.99662	6.88813	5.93853	5.65342	5.79099	4.94932	5.8167	5.86343	5.22933	5.25973
ALG8	4.54687	4.49732	4.28839	4.37364	5.67907	5.57418	5.83815	5.62767	4.22314	3.87852	3.88432	4.25647	4.2787	3.79818	3.72818	3.64003
KCTD21-AS1	1.12752	1.38416	0.9232	1.21731	2.25358	1.3521	2.12553	1.26265	0.67631	1.42059	1.2731	1.29496	1.00041	0.76375	1.61712	1.62188
KCTD21	1.3137	1.99682	1.57077	1.9327	3.11522	2.71455	2.9935	2.49628	1.2346	2.29245	2.08792	2.65985	1.75313	1.69077	2.51237	2.45999
USP35	2.32457	2.24624	2.58589	2.21714	2.64624	2.95097	2.10722	3.08093	1.71018	2.25168	2.38303	2.31172	1.09313	1.32665	1.53085	1.68336
GAB2	2.14822	2.35545	2.24619	2.28972	2.72011	2.61232	2.52608	2.26001	2.53666	2.70284	2.42232	2.81853	1.22958	1.47737	1.8088	2.07217
NARS2	3.4951	3.21298	3.1407	3.27828	3.52032	3.17945	3.49401	3.42722	3.51479	2.93512	2.99629	2.64089	3.34574	3.5205	2.62372	2.85964
TENM4	-0.93513	-0.98444	-0.61749	-1.01162	-3.20991	-3.25422	-3.2593	-3.32193	-3.32193	-3.32193	-3.32193	-3.32193	-1.32962	-2.19281	-0.77218	-1.73969
RPS28P7	8.23687	8.2487	8.24778	6.99929	6.29323	7.34229	5.35078	7.27866	6.97692	7.58373	6.81094	6.88901	3.71472	3.35019	2.83939	3.13025
PRCP	2.38192	2.24968	2.25182	2.60386	3.65025	3.84172	3.95394	3.72628	2.83489	3.0999	3.04407	3.43588	3.11617	3.1707	3.03158	2.96116
DDIAS	2.06016	1.41232	0.91922	1.03227	2.52333	0.85195	2.2451	1.23127	2.26224	1.02464	0.31557	0.56373	3.01902	2.67765	1.63773	1.42323
RAB30	-0.92585	-1.26332	-0.98821	-0.09487	0.63619	-0.46709	1.08445	-0.45017	-0.15109	-0.72255	-0.30989	0.06377	0.58777	-0.31919	0.70873	0.05793
PCF11	2.09515	2.11378	2.09333	2.46858	2.46532	2.46814	2.87225	2.28614	2.02209	2.47319	2.33016	3.10977	2.5574	2.57462	2.38699	2.68367
PCF11-AS1	0.56781	1.29627	-0.17059	1.11645	-0.25281	0.93484	0.47537	1.5959	-1.56478	-0.63701	-0.54923	0.59211	1.25468	0.95486	0.89502	0.80626
ANKRD42	2.19851	2.37254	2.16658	2.81474	1.15545	0.62959	1.26646	0.82775	0.70442	1.02623	0.77757	1.52574	1.23367	1.53719	0.50836	1.14167
CCDC90B	3.07921	2.72375	3.29661	3.14339	2.37972	1.55903	2.68663	1.15352	3.01591	2.04896	2.84951	2.45786	2.57907	0.49701	1.94048	0.20224
TMEM126B	4.90417	4.67558	4.64548	4.93337	4.00564	4.27505	4.45482	4.1406	3.79477	3.31929	3.48736	3.04109	4.28382	3.41381	3.55141	2.96566
TMEM126A	5.50527	5.22578	5.3173	5.62176	5.76051	5.85909	6.02426	5.66866	5.11913	5.19208	5.11389	4.6586	5.37692	4.58483	4.26713	3.73986
CREBZF	3.62021	3.29022	3.55887	3.49677	4.2045	3.71833	4.10245	3.60622	4.00043	3.68699	3.78358	3.3801	3.60904	3.82205	3.80321	3.35424
SYTL2	-0.84944	-0.46044	0.06684	-0.13706	-2.14625	-3.04798	-1.9034	-3.00463	1.75592	2.70527	1.81885	2.82505	-2.80382	-2.26789	-2.65074	-1.86293
PICALM	5.17094	5.31291	5.39176	5.39608	4.64506	4.01821	5.36668	3.82469	5.70097	5.71883	5.83044	6.81088	5.52085	5.20477	5.72717	4.95555
EED	2.52148	2.56032	2.34114	2.54586	2.55319	2.02522	2.73933	2.30891	2.61902	2.22863	2.30668	1.25468	2.3408	2.41732	1.55634	1.96742
HIKESHI	3.39415	3.6292	3.37397	3.67667	3.41677	3.18819	3.40938	3.24648	3.52916	3.48435	3.39067	3.24772	3.40656	3.46531	3.07712	2.51128
ME3	3.2576	3.09864	3.00968	2.79526	-0.2688	0.3855	-0.32479	-0.66852	4.07547	4.02547	4.05013	3.65525	2.3114	2.71985	2.82953	2.49198
FZD4	2.05944	2.03972	2.3453	2.1845	2.62779	2.02308	2.61238	2.35794	0.44208	-0.16702	0.62057	0.22196	2.63095	2.68231	3.94164	3.19184
TMEM135	1.10579	1.4863	1.40105	1.99141	3.78687	3.52603	4.42644	3.61246	2.81942	3.15756	2.80843	3.51923	2.70461	3.46112	2.9229	3.35949
RAB38	-0.93285	-1.84484	-2.14863	-0.99633	-2.78588	-1.93958	-3.32193	-3.32193	3.72425	3.89869	2.91912	4.09807	-2.15513	-1.21474	-1.72585	-1.97088
CTSC	3.63148	3.17377	3.12207	3.36938	4.23768	4.586	4.63267	4.58451	2.81135	2.76631	2.5966	3.41483	4.85601	4.9068	4.08538	4.43204
CHORDC1	3.59657	3.32322	3.02706	3.47459	3.31425	2.60586	3.95297	2.62402	2.98455	2.64618	2.66286	2.88461	3.47204	3.23133	2.7605	2.53626
TUBAP2	1.55527	1.64356	1.50398	-0.38051	0.63311	0.49931	0.12182	0.24778	1.76545	1.43207	0.84379	0.01762	-0.50813	-1.19082	-0.52033	-1.222

FAT3	-3.26499	-3.32193	-3.32193	-3.32193	-3.27342	-3.32193	-3.16783	-3.32193	-3.32193	-3.32193	-3.32193	-3.32193	-1.04468	0.8606	-1.21581	-0.68392
SLC36A4	1.36076	1.31241	1.56446	1.89203	2.55307	1.62371	3.2312	1.65102	2.05053	1.47182	1.45413	2.30206	2.15607	2.21464	2.2897	2.20792
SMCO4	3.58863	3.13584	2.88611	2.51295	5.14706	5.35882	5.23934	5.01481	3.75756	3.07424	3.04472	2.3486	3.17776	3.39277	1.82927	2.48843
CEP295	1.83752	1.75894	1.86948	2.434	2.50204	2.634	2.85175	3.00775	1.72743	1.60035	1.65073	2.08336	2.45226	2.15439	1.85274	1.74266
SCARNA9	3.32105	2.80936	2.78082	2.69684	0.67269	1.68355	1.46884	2.84011	3.13571	3.55591	1.38151	1.86525	1.42423	1.88921	1.79021	1.0222
TAF1D	4.13634	4.12002	4.05031	4.4018	3.73256	3.94076	4.17378	4.18244	3.2244	3.65605	3.41039	3.74975	3.8715	3.70938	2.94719	2.762
SNORA25	3.80743	3.20183	3.78907	3.80657	3.66053	5.65713	4.51141	6.33452	1.48178	2.5072	1.63229	1.19931	1.8992	2.96095	1.20911	1.54897
SNORA32	3.45998	4.39081	4.60982	4.70107	4.06327	5.02353	5.09709	5.1682	2.91755	2.50251	3.42379	4.14844	4.68526	3.81968	2.897	1.6914
SNORA8	5.09424	5.37374	5.49448	5.49863	4.37372	5.20145	5.19313	5.47884	3.4768	3.76135	3.54455	4.2597	4.42856	4.45245	2.6998	2.84468
SNORD5	5.13689	4.50926	3.80274	5.18342	4.03169	4.43289	5.08075	4.73637	4.01483	4.29125	2.48721	4.3404	3.65465	3.64954	2.8491	3.64964
SNORA18	5.67958	5.15934	4.97481	5.50391	4.99157	5.99344	4.92058	6.04084	4.36478	3.89156	4.37916	4.91644	5.40865	5.22026	4.21917	3.53301
MIR1304	4.98444	4.33648	3.93134	4.28393	4.56343	4.83576	3.58997	4.41973	3.00555	2.73973	2.17557	1.7373	4.01222	3.42662	2.72559	2.09135
SNORA40	5.08654	5.47078	5.04172	5.66232	5.38181	4.57083	5.81883	5.1699	4.52427	5.11064	4.84873	4.31859	5.87013	5.9572	5.30387	4.40181
C11orf54	2.34395	2.44989	2.45879	3.02383	3.56188	2.99066	4.01464	2.69511	1.2117	1.14605	0.98936	1.42962	2.75565	1.51806	2.31664	1.63612
MED17	1.41175	1.5047	1.62987	2.01237	1.44545	1.03976	1.77549	1.31602	1.25821	0.99779	1.12847	1.55769	1.70611	1.6467	1.20961	1.25918
PANX1	4.52881	4.87257	4.87716	4.85664	4.71899	3.30408	5.0609	3.03582	5.05315	5.16206	4.86186	5.59634	3.99586	4.31482	4.58319	4.95966
MRE11	2.4462	2.4456	2.35011	2.39527	2.58982	1.88456	2.90618	2.12501	2.7529	2.11248	2.28649	2.03557	2.92386	2.54508	2.48863	2.47688
ANKRD49	1.62926	1.80103	1.81399	2.06513	1.95831	1.94068	2.51846	2.21077	1.31	1.66798	1.61556	2.0883	2.08946	2.2342	1.71999	1.33757
PIWIL4	2.21611	2.16856	2.02648	1.58589	-0.77466	-1.55204	-1.56313	-2.04829	1.51153	1.53991	0.75494	0.89491	1.15124	1.60924	1.11414	1.90267
FUT4	3.25407	3.3103	3.07577	2.59326	0.01223	-0.23248	-0.22891	-0.5266	2.83944	2.84848	2.26626	2.281	2.64885	3.4659	2.51745	3.65824
AMOTL1	1.77965	1.5594	1.47752	1.60815	-2.13223	-2.84582	-2.42173	-2.60478	3.01328	3.38875	2.984	4.06616	4.98909	5.15335	5.8014	6.18701
CWC15	4.4516	4.44201	4.42203	4.68046	4.00282	4.63538	4.39294	4.37747	3.81088	4.68462	4.76304	4.54777	3.464	4.09988	3.62662	3.4416
KDM4D	-0.68678	-0.42751	-0.30374	-0.11636	-1.21071	-1.68593	-1.88742	-1.97148	-1.31139	-2.1478	-1.33285	-1.7483	-0.61866	-1.8206	-1.42002	-1.72041
SRSF8	2.65635	2.642	2.04979	2.81899	3.33159	3.28442	3.17039	3.33188	3.40653	3.38788	3.26022	3.84457	3.10916	3.45218	2.53676	3.09467
ENDOD1	0.03887	-0.15329	-0.0838	0.2217	3.00718	2.33314	3.45702	2.1027	3.92139	3.31269	3.88763	4.29946	3.98632	3.84248	4.41284	4.37852
SESN3	-3.1243	-3.10187	-2.71615	-3.07663	-3.07454	-2.8666	-3.05222	-3.08973	-3.2038	-3.32193	-3.32193	-3.04839	-2.61477	-1.15344	-1.5341	-0.45328
FAM76B	1.15024	1.43809	1.67324	2.06013	1.38981	1.65036	2.0032	1.92501	1.16996	1.44679	1.14784	1.81134	1.59379	2.13811	1.57038	1.24422
CEP57	3.02579	3.02637	2.9904	3.3069	2.88696	2.17994	2.97712	2.22527	3.37719	3.1367	2.83645	3.57559	3.35468	2.92757	2.97331	2.61501
MTMR2	3.44713	3.40493	3.29164	3.80719	3.2803	2.6001	3.93677	2.63457	3.58822	3.19198	3.23913	3.99855	4.17836	4.20612	4.18428	4.29967
MAML2	0.31769	0.60544	1.00095	0.99143	-3.13403	-3.32193	-3.18192	-2.98884	2.42735	2.37137	2.59938	3.19471	1.37711	1.07815	2.17284	1.9329
MIR1260B	0.88291	-3.32193	2.39662	2.9646	-3.32193	-3.32193	-3.32193	-3.32193	4.19734	4.40073	5.10553	3.98251	3.63196	3.04772	4.49696	4.77252
CCDC82	2.68054	3.07112	3.24939	3.82231	1.82986	2.43402	2.67166	2.33122	2.44195	2.67654	2.09315	3.08372	2.01792	2.6554	1.86288	2.39905
JRKL	1.79078	1.78639	1.84121	2.22545	0.80928	1.52177	1.61885	1.60632	2.60568	3.02435	2.85236	3.40869	2.07782	2.59775	2.16667	2.59785
PPIAP43	1.73719	1.96441	1.97497	2.97746	-1.46751	-0.46835	-1.36779	-1.10061	-0.00727	0.52251	-0.15581	-0.54807	-3.32193	-3.32193	-3.32193	-0.86033
ARHGAP42	1.01609	1.41098	1.14967	1.86454	2.59074	-0.88704	3.44462	-1.12364	1.9368	2.01602	2.34776	3.22659	0.79695	1.06303	1.86927	1.69655
CEP126	-3.32193	-3.32193	-3.32193	-3.32193	-0.59208	-0.93918	-0.1163	-0.3866	-0.27421	0.21399	-0.15991	1.24646	-1.17962	-0.41328	-0.72117	-0.35062
CFAP300	-1.34663	-2.11005	-1.86941	-1.336	-1.04087	0.63471	-0.4019	-0.0939	-0.23483	0.36458	0.05881	0.77582	1.28866	1.06442	0.71341	0.69287
YAP1	4.21173	2.41415	4.39824	2.62142	5.15216	1.81865	5.00037	1.97336	5.71503	2.89189	5.057	2.4561	5.51176	-0.26647	5.03542	-0.08608
BIRC3	5.48678	5.27025	5.26234	5.52683	5.36441	2.42522	6.25715	2.21129	5.48731	4.92235	5.41603	6.07724	4.43924	2.67854	3.61157	2.24005
BIRC2	4.93547	5.09993	5.04653	5.61796	4.89883	4.0962	5.71382	3.98413	4.94181	5.17064	4.78186	5.8106	5.907	5.58013	5.35498	5.04611
TMEM123	5.57439	5.34109	5.60925	5.5176	6.94256	5.96957	7.60921	5.65587	6.59689	6.32567	6.32908	6.6179	6.59646	6.61779	7.07905	6.46871
MMP7	-3.32193	-3.32193	-2.48065	-2.14222	-0.46527	-0.98461	-0.5965	-2.8392	-3.32193	-2.60441	-3.32193	-2.614	0.05975	2.49294	0.84046	1.25234
WTAPP1	3.69876	4.44839	4.14885	4.93695	-3.18579	-3.01674	-3.32193	-3.32193	-2.08673	-2.57859	-2.75366	-2.10452	3.48286	0.11396	0.74798	2.2235
MMP1	6.30544	7.11703	6.82025	7.73346	-2.3191	-1.74098	-2.87013	-2.36797	-0.72803	-0.75177	-1.09753	0.63838	5.7702	2.28303	2.78043	4.47541
MMP3	2.05483	3.11971	2.04988	4.18837	-3.32193	-3.32193	-3.32193	-3.32193	-2.41044	-2.02225	-2.19909	-1.50849	-2.64947	-3.32193	-3.32193	-2.86794
DCUN1D5	6.11122	5.98138	5.72491	5.8855	5.86752	5.45481	6.17914	5.21043	5.45897	5.16735	5.10783	4.59477	5.57608	5.59348	5.19484	5.01992
DYNC2H1	0.19312	0.43318	-0.05502	0.93938	-0.27019	-0.03064	0.10272	-0.03073	1.59049	1.73445	1.39667	2.72594	0.99893	1.11197	1.22396	1.52528
PDGFD	-3.08078	-3.32193	-3.32193	-3.32193	0.20268	1.17132	-0.07535	0.93937	0.97154	0.94451	0.50517	1.73344	-1.8095	-1.32826	-2.06516	-1.75977
CASP4	3.23026	3.4984	3.05291	3.48883	-0.10265	1.16434	-0.01653	0.82099	3.45665	4.10962	3.78034	4.1517	-0.63334	0.51363	-1.33586	-0.54681
CASP1	3.17309	3.14396	2.69826	3.1532	-3.32193	-3.32193	-3.32193	-3.00043	1.6662	2.08018	2.34451	2.80407	-3.32193	-2.59479	-3.32193	-3.32193

CARD16	3.09763	3.20702	2.81105	2.9778	-3.32193	-2.77115	-3.32193	-2.25584	-0.2375	0.4707	0.36824	-0.10755	-2.03455	-2.03759	-3.32193	-3.32193
MSANTD4	2.17764	2.10359	1.96363	2.43731	2.1291	1.43177	2.74698	1.47314	2.6024	2.95197	2.64208	3.29485	2.56082	2.85454	2.63817	2.80712
KBTBD3	0.89437	0.97345	1.26327	0.60501	1.24172	0.27458	1.30873	0.30802	1.35232	1.53357	1.01723	1.06175	1.16961	1.41696	1.0989	1.33148
AASDHPPT	3.3653	3.53388	3.21574	3.72818	3.26544	2.86209	3.45131	2.94195	3.72749	3.70883	3.20406	3.99013	3.88194	3.86232	3.25456	3.6491
GUCY1A2	-3.32193	-3.32193	-3.32193	-3.15873	-3.29315	-2.90429	-3.03339	-3.09632	-1.94395	-1.70912	-2.04387	-0.63455	-3.32193	-3.32193	-3.32193	-3.32193
CWF19L2	3.12173	2.84865	2.92067	3.45084	2.95746	3.51741	3.38934	3.99582	2.32552	2.28247	2.13368	2.87829	2.11931	1.97842	1.91964	1.73811
ALKBH8	1.12706	0.8177	0.33701	1.10025	2.05909	1.19804	2.64194	1.06157	3.09908	2.59996	2.85899	2.86159	2.31362	1.80254	1.6817	1.62849
ELMOD1	-2.83897	-3.32193	-2.92793	-2.58273	-3.24292	-3.32193	-3.32193	-3.32193	0.72764	0.77426	0.77093	1.19293	-1.5179	-1.60993	-2.06377	-1.28488
SLC35F2	4.13903	3.62453	3.82533	3.63034	3.95206	2.73217	4.50767	2.30535	4.15914	3.56051	4.21342	4.09788	4.84843	2.97729	4.79237	3.18687
RAB39A	-3.32193	-3.32193	-3.32193	-3.32193	0.954	0.47136	1.82545	0.59687	-1.01633	0.68991	0.85875	0.51276	-0.15614	1.36909	1.00225	0.66898
CUL5	2.57519	2.59232	2.4632	3.26148	3.54322	2.29484	4.25292	2.10091	3.15454	3.33495	3.18267	3.80544	3.42155	3.39195	3.2307	3.21033
ACAT1	4.05657	3.86409	3.82901	4.13984	4.9341	4.69338	5.24928	4.67854	4.78559	4.6494	4.6394	4.51529	4.80328	4.68343	4.28645	4.1428
NPAT	1.95631	1.97774	1.92058	2.3743	1.5053	0.44392	1.96994	0.69541	1.83209	1.49664	1.37101	2.41271	2.12474	2.22272	2.04703	2.21222
ATM	0.93589	1.2098	1.24065	1.72614	1.59153	1.39168	2.08725	1.42848	1.12256	1.56701	1.63466	2.54887	1.74926	1.56239	1.80106	1.95793
C11orf65	-0.83007	-0.47796	-0.41061	-0.09837	-0.2859	-0.11418	-0.18245	-0.21815	0.10136	0.01894	-0.48194	0.87414	0.09252	-0.37611	0.17675	0.23219
EXPH5	-1.01835	-1.14084	-1.46243	-1.23346	-2.90574	-2.95514	-3.05836	-2.81028	-1.2648	-1.70337	-1.84705	-1.40275	0.37231	-0.11813	0.25511	0.21627
DDX10	2.48403	2.6045	2.43093	2.81245	2.42623	2.24687	2.96461	2.41307	1.90963	2.49272	2.62577	2.72914	1.79808	3.01255	2.53203	2.98453
C11orf87	-3.14318	-2.94775	-3.32193	-3.09972	-3.32193	-3.32193	-3.32193	-3.32193	-3.32193	-3.14919	-3.13715	-3.15195	-2.1584	-0.80174	-1.50688	-0.64245
ZC3H12C	1.87247	1.88176	2.06593	2.59882	0.69848	-0.94383	1.46525	-0.9759	2.15488	2.32121	2.21977	3.23906	2.36771	1.56625	2.4886	1.76068
RDX	4.84998	4.91221	4.80505	5.40954	5.33048	4.81546	6.03805	5.01697	5.44371	4.70342	4.87458	5.51816	5.40003	4.98676	5.41285	4.97732
FDX1	3.83899	3.51139	3.52181	3.8513	4.46209	4.09273	4.58135	4.1262	3.86705	2.96413	3.14197	2.2366	3.26329	2.86418	2.47969	2.02462
COLCA1	-3.32193	-3.32193	-3.32193	-3.32193	-0.26493	-0.01512	-1.48533	-0.16361	-3.05098	-3.2033	-3.32193	-3.20523	-3.32193	-3.32193	-3.32193	-3.32193
COLCA2	-2.86046	-3.32193	-3.32193	-3.32193	1.97897	2.7752	0.81041	2.77084	-2.41405	-2.87463	-3.32193	-2.54399	-3.32193	-3.32193	-3.32193	-3.32193
POU2AF1	-3.17833	-3.32193	-3.32193	-2.84027	-1.89486	-1.22424	-1.21042	-1.46832	-3.32193	-3.32193	-3.32193	-3.32193	-3.32193	-3.32193	-3.32193	-3.32193
BTG4	0.52197	-0.11674	-0.48873	-0.76215	-3.32193	-3.32193	-3.32193	-3.32193	-0.51234	-0.95581	-0.87191	-1.37344	-3.32193	-3.32193	-3.32193	-3.32193
LAYN	-3.00363	-2.96909	-2.20434	-2.62303	-1.50791	-2.32311	-1.93623	-2.41055	5.8124	5.68899	5.77207	5.6081	2.1097	1.67052	1.70408	1.22673
SIK2	1.99643	2.06716	2.10289	2.38706	4.03587	3.73784	3.95946	3.68687	3.11205	2.97072	3.21478	3.70416	2.92915	2.76778	3.46639	3.50946
PPP2R1B	2.68979	3.15412	3.05004	3.58639	5.13532	5.07098	5.744	4.92553	2.92682	3.22056	3.87859	4.04355	3.60098	3.83977	4.47234	4.10319
ALG9	1.79521	2.02048	1.9755	1.95994	2.98848	3.21959	3.00685	3.19092	2.84202	2.37581	2.52332	2.9402	2.18239	2.52877	2.6796	2.60257
FDXACB1	1.21993	0.86173	0.60801	1.02248	1.48605	1.4252	1.09772	1.77716	1.09248	0.73086	1.55101	1.0193	1.69309	1.40175	1.34567	1.35209
C11orf1	2.14247	2.34477	1.90619	2.43818	0.70622	2.13799	0.67259	2.1019	1.3632	1.95248	1.47837	2.16312	2.4459	2.51457	1.80112	1.86347
CRYAB	-2.99331	-2.95778	-2.94002	-2.34461	-2.05149	1.08229	-2.35467	0.9679	-3.32193	-2.74338	-2.98279	-3.32193	-2.83566	-2.06019	-2.52891	-2.73767
HSPB2-C11orf52	-2.51107	-3.32193	-2.78908	-2.04201	0.99274	3.46011	0.75198	3.13783	-3.32193	-3.32193	-3.32193	-3.32193	-1.23449	-0.88471	-1.0114	-1.81598
C11orf52	-2.80472	-3.32193	-3.32193	-2.69496	1.20005	3.65171	0.96451	3.34367	-3.32193	-3.32193	-3.32193	-3.32193	-1.08444	-0.72525	-1.08773	-1.68787
DIXDC1	0.72299	0.53742	0.56869	1.1646	2.09363	1.09633	2.49202	1.29525	1.87275	1.02504	1.52979	1.13828	-0.44227	-0.53327	0.30112	0.03662
DLAT	4.38687	4.13972	3.86633	4.4027	5.56049	4.27897	5.80324	4.35001	4.83209	4.17309	4.29806	4.17806	4.85536	4.79785	4.38959	4.39972
PIH1D2	1.13873	1.35883	1.49217	1.44031	0.74172	1.51577	1.66663	1.92372	0.01665	-0.12536	0.26897	0.66629	1.32685	1.58003	0.72899	0.26556
NKAPD1	2.97352	3.17725	2.9607	3.47001	3.17918	2.83232	3.68252	2.93943	3.07446	3.53879	3.38574	3.88277	2.76071	2.88874	2.75908	2.81197
TIMM8B	6.48693	6.45508	6.22912	6.31606	6.66095	7.08007	6.3866	7.05986	5.85922	5.87691	5.79723	4.9755	6.6657	6.52224	5.31769	5.39443
SDHD	5.41734	5.40173	5.22838	5.77207	5.17738	5.10724	5.255	4.88287	5.19095	5.27846	5.01902	4.85573	4.73349	4.77972	3.91774	4.00532
IL18	2.52865	2.73623	2.63759	2.99395	4.19472	3.50553	5.04626	3.63976	4.24381	4.54815	4.01452	5.18078	4.32342	3.8771	3.56778	3.49965
BCO2	-2.89651	-2.49827	-2.83001	-2.53819	-2.53259	-2.21366	-2.27431	-1.85326	-2.98192	-2.90973	-2.88333	-2.59924	-2.0392	-1.07455	-1.80818	-1.35824
PTS	4.24941	4.50986	4.16207	4.59525	4.26487	4.55095	4.60351	4.68203	1.98629	2.50366	2.31631	2.37224	2.81058	3.42316	2.10903	2.26134
NCAM1	0.74692	0.95079	1.05019	1.18221	-3.32193	-3.32193	-3.32193	-3.22098	-3.32193	-3.32193	-3.32193	-3.32193	-2.46471	-2.39306	-1.02977	-1.37548
TTC12	1.60516	1.34941	1.35761	1.67177	0.79498	1.346	0.84345	0.98578	1.44376	1.57511	1.58322	2.08248	1.04337	1.02233	1.05005	1.3625
ZW10	3.67926	3.36321	3.2808	3.62228	3.50438	2.64694	3.95417	2.60538	4.018	3.33707	3.5725	3.95591	3.86486	3.81478	3.67305	3.62666
USP28	2.22866	2.18516	2.14565	2.47196	3.15024	2.21466	3.5077	2.37422	3.2719	3.07433	2.98231	4.17368	3.2282	3.10016	3.1344	3.30551
NNMT	3.75212	3.00689	3.20219	3.80695	2.07396	2.24816	2.31884	1.17023	6.33176	5.67443	6.01856	5.84786	4.42717	3.53332	3.87991	2.51211
C11orf71	1.00175	1.5	1.07274	1.83553	1.60767	2.78732	1.54998	2.42755	1.46559	2.40539	1.94827	2.52212	1.38495	2.13432	1.56514	1.69089
RBM7	1.90378	2.12945	2.05593	2.44443	2.48685	1.90397	2.93592	1.8435	3.21702	3.59888	3.00411	3.68747	2.7674	3.22754	2.32624	2.5517

REXO2	4.8947	4.78571	4.67409	5.00912	4.7716	4.4478	5.07252	4.21858	5.5463	5.3328	5.33078	4.862	5.16729	4.42017	4.26236	3.83274
CADM1	2.38642	2.54023	2.73553	2.35997	-3.15884	-3.32193	-3.23005	-3.0984	-3.00502	-3.05434	-3.12537	-3.32193	3.34422	4.31733	3.71608	3.3169
LINC00900	0.19183	-0.17471	0.0371	-0.75297	-3.32193	-3.32193	-3.32193	-3.32193	-3.32193	-3.1115	-3.32193	-3.11481	-3.32193	-3.14928	-3.32193	-3.32193
BUD13	3.80629	3.51667	3.36102	3.10798	3.47085	3.14646	2.99723	3.30049	3.90015	3.30965	3.64097	2.80083	3.57566	3.54009	3.1927	3.04859
ZPR1	4.10112	3.80359	3.74486	3.89347	3.43439	3.34917	3.2784	3.24033	3.89636	3.55669	3.68879	3.83236	3.58031	3.07567	3.40217	3.07099
APOA4	-3.32193	-3.32193	-3.32193	-1.73915	1.19724	1.0504	1.24026	-0.23101	-3.32193	-3.32193	-3.32193	-3.32193	-3.32193	-3.32193	-3.32193	-3.32193
APOC3	-3.32193	-1.73129	-3.32193	-3.32193	3.04359	5.65508	2.04107	6.05318	-3.32193	-3.32193	-3.32193	-3.32193	-3.32193	-3.32193	-2.43	-3.32193
APOA1	-1.65285	-2.63331	-2.60322	-3.32193	7.25314	7.92911	6.76985	8.25593	-1.7856	-2.2855	-1.89226	-2.72101	-1.17921	-2.81049	-3.32193	-3.32193
APOA1-AS	-1.41231	-3.32193	-1.92511	-3.32193	7.65843	8.31111	7.26317	8.64803	-1.24992	-1.45062	-2.04211	-1.46845	-1.66201	-3.32193	-3.32193	-3.32193
SIK3	1.74333	1.81977	2.07061	1.96836	2.24931	1.85931	2.22579	1.927	2.0234	1.89479	2.2556	2.29792	1.47995	0.73036	1.76751	1.4482
PAFAH1B2	4.27834	4.44979	4.31961	4.46134	4.34522	3.91442	5.08565	3.75876	5.08772	4.96977	4.88723	5.50269	4.80925	4.94139	5.61991	5.62328
SIDT2	1.43048	1.55391	1.79876	2.12802	3.83521	4.12416	3.19893	3.83814	2.31572	2.76173	2.7156	3.16595	1.1111	0.8627	1.99144	2.11143
TAGLN	0.49338	0.54262	0.984	1.27873	0.98914	2.42998	1.21378	2.19464	1.55229	1.32622	2.16755	1.02483	6.32801	4.73566	6.72973	4.59995
PCSK7	1.73524	1.91364	1.92056	2.25923	2.44472	2.42035	2.14093	2.54515	1.32579	1.84797	2.21145	1.82137	3.07157	2.38048	3.68908	2.53986
RNF214	2.45015	2.62317	2.50644	2.95672	2.65666	2.80103	2.5441	3.14953	3.0032	3.23394	2.86484	3.54969	2.47892	2.58403	2.50938	2.61528
BACE1	2.7455	2.89337	2.92486	3.08879	3.95625	3.11379	3.61846	3.24765	2.62004	3.13783	2.79568	3.55023	2.23797	2.42174	3.0926	3.16334
BACE1-AS	2.9004	3.27001	3.21364	3.33204	4.77365	3.49955	4.57574	3.39059	3.08504	3.42201	3.07708	3.67475	2.9253	2.85078	3.59256	3.6064
CEP164	2.62844	2.26326	2.13943	2.36926	2.6347	2.51027	2.30174	2.63842	2.59579	2.23487	2.71463	2.38948	2.24451	1.88499	2.27149	2.31607
DSCAML1	-3.32193	-3.32193	-3.32193	-3.32193	-3.13218	-2.68249	-3.32193	-2.84305	-3.32193	-3.17608	-3.32193	-3.32193	-0.75475	-1.58845	0.36764	-1.24134
FXYD2	-2.34299	-0.85851	-1.33711	-0.89488	2.49817	6.21053	1.7217	5.55531	-2.91869	-3.14081	-3.12822	-3.32193	4.57367	5.26512	4.4719	4.27825
FXYD6-FXYD2	-0.33145	1.73574	0.98645	1.69255	5.27936	8.97078	4.45704	8.33678	-1.5963	-2.35325	-2.30177	-3.32193	7.60582	8.30719	8.47077	8.02749
FXYD6	-3.32193	-3.32193	-3.08437	-3.32193	-2.60677	0.16797	-2.67658	-1.0135	-3.32193	-3.32193	-3.32193	-3.32193	3.98032	4.59878	6.07017	5.45773
JAML	-3.0822	-3.32193	-3.32193	-3.16605	1.04103	0.98733	0.79637	0.29723	-3.32193	-3.32193	-3.32193	-3.32193	-3.32193	-3.32193	-3.32193	-3.32193
MPZL3	-2.48812	-1.97894	-2.37564	-1.74887	3.09983	4.22721	3.96016	3.59746	-0.58126	-0.10487	-0.72128	-0.01445	0.04925	0.71559	0.17261	0.73567
MPZL2	-3.32193	-3.32193	-3.32193	-3.32193	4.16542	4.1294	4.214	3.74669	0.26406	0.45988	-0.80177	0.20493	-3.32193	-3.32193	-3.11291	-2.75823
UBE4A	3.13723	3.02284	2.93292	3.44059	4.40515	3.58606	4.72291	3.49853	4.29234	3.24976	4.08513	4.34387	3.8741	2.75729	3.80555	3.21761
ATP5MG	6.33647	6.37796	6.28477	6.11379	6.3453	6.65734	6.07708	6.47486	6.16325	6.13306	6.15248	4.77395	6.06837	6.11225	4.9416	4.93659
KMT2A	2.16991	2.19234	2.32034	2.74767	2.78456	1.81979	2.9238	2.26918	3.12744	3.13645	3.23366	4.07337	2.92923	3.00371	3.47422	3.65833
TMEM25	-2.679	-3.32193	-2.73802	-2.88426	-1.81192	0.01197	-2.38292	0.34338	1.71444	2.4454	2.06063	2.03755	-3.03511	-2.1809	-2.47063	-1.41901
IFT46	0.80595	0.89429	0.51193	1.27023	-0.15987	0.58407	0.19499	0.24995	1.84826	2.32921	2.35534	2.56703	1.08723	1.78736	1.07117	1.39066
ARCN1	5.30075	5.33228	5.27221	5.68762	5.96717	5.39147	6.60431	5.06866	5.94408	5.44637	5.89391	6.52044	5.65266	5.12324	5.72165	5.13545
PHLDB1	1.98507	2.19267	2.26643	2.26302	1.40563	1.60436	0.77755	1.7113	2.68838	2.88028	3.04058	3.05963	2.12258	2.44912	3.17485	3.16178
MIR6716	5.24549	4.8284	5.01266	5.46564	4.53441	3.88883	3.45509	4.91177	6.32488	6.15379	6.29446	5.27528	6.04928	6.2733	7.31665	7.03066
TREH	2.81792	2.96214	2.9875	3.16971	1.35336	2.65869	0.41083	2.58237	2.68309	2.74107	3.00157	2.96344	2.27469	2.78208	2.96497	3.01324
DDX6	3.252	3.62618	3.30776	3.3581	3.68544	2.90928	4.40683	3.01539	4.00568	3.9223	3.81207	4.69455	4.39208	4.32695	4.8104	4.90949
CXCR5	4.02232	4.05263	3.99396	3.79449	4.02831	3.77994	3.92979	4.14262	4.51616	4.71404	5.07177	5.11981	3.24789	3.44108	3.67538	3.72918
BCL9L	4.81469	4.91897	4.99151	3.93255	5.23165	3.86519	4.96232	4.38962	5.49571	5.74273	6.08973	5.65298	4.00111	4.30686	4.93896	5.03072
MIR4492	2.56582	3.13934	3.21644	1.91616	4.58482	2.32322	5.3523	3.03163	3.9898	4.30347	4.47316	5.09292	3.52364	2.93986	0.96533	2.53117
UPK2	1.90652	1.92671	2.17192	0.90438	3.61935	1.32902	3.50672	1.07256	3.71707	4.00627	4.35948	3.52884	2.42699	2.94756	3.39022	3.61128
CCDC84	5.11063	4.9764	5.08047	4.65776	4.53621	5.03676	4.1233	5.02336	4.48552	4.57856	4.25742	3.46078	4.74242	4.33407	3.58352	3.4166
TRAPPC4	4.37233	4.50301	4.35834	4.26732	4.8629	5.37993	4.8315	5.41131	3.88144	3.89096	3.93143	3.47535	3.47421	3.9181	3.51127	3.16993
SLC37A4	3.40447	3.31782	3.0617	2.68958	3.99425	4.68222	3.19422	5.09713	2.93625	2.63453	2.6523	2.0427	1.36567	2.21856	1.99317	2.07514
VPS11	3.30768	3.416	3.23635	3.68799	3.14745	3.56602	3.20743	3.76744	3.18513	3.18115	3.26312	3.57084	2.4766	2.71409	2.48724	2.87412
HMBS	3.76606	3.50859	3.56492	3.32481	4.52767	4.57772	4.09939	4.393	3.21686	2.652	2.92898	2.02405	2.98495	2.31583	2.98487	2.02114
DPAGT1	4.25708	4.17528	4.05993	3.96811	5.36489	5.38399	5.06358	5.27178	4.10171	3.72216	3.81546	3.69775	3.37303	3.63238	3.47649	3.70948
C2CD2L	3.35962	3.29805	2.9731	2.86316	2.55138	3.09391	2.02773	2.76789	1.08832	1.4876	0.96311	0.57447	1.29592	1.51989	0.90837	1.30746
HINFP	1.27492	1.13684	1.26844	1.36197	1.19851	1.2423	0.85502	1.31801	1.07629	1.38457	0.99741	1.07362	0.69111	0.77723	0.9264	0.66551
ABCG4	-2.86639	-2.56101	-2.52855	-2.92963	-0.14527	-0.09173	-0.58624	-0.47742	-1.9532	-2.11229	-1.54119	-2.88685	-3.06622	-3.32193	-2.36193	-2.53562
NLRX1	1.84852	2.13963	1.95152	2.13459	3.07613	2.7203	2.5375	2.97067	3.04849	3.28757	3.57328	3.09595	2.16266	2.50887	3.12001	3.37557
PDZD3	-3.32193	-3.32193	-3.32193	-2.99307	1.28851	0.7823	0.16638	-0.27991	-3.32193	-2.65591	-3.32193	-2.85255	-3.32193	-3.32193	-3.32193	-3.32193

CCDC153	-2.63729	-2.57207	-2.8786	-2.50091	0.627	1.71936	-0.358	1.03581	-1.85479	-0.42629	-0.72907	-1.87454	-2.35731	-3.32193	-2.02942	-2.40434
CBL	2.21054	2.32756	2.4809	2.60497	2.76595	1.78607	3.20679	1.69482	3.09378	3.29128	3.09725	4.02351	3.10678	3.38434	3.51391	3.65122
MCAM	5.86034	5.94023	6.15528	6.08898	-2.37135	-1.89344	-2.95139	-1.42032	3.58345	3.63016	3.67518	3.28181	2.9545	3.77005	4.36155	3.64134
RNF26	5.37925	5.21729	5.12679	4.60001	6.82518	6.01548	5.99046	5.90335	6.17301	5.60122	6.02705	5.19107	4.9272	5.14053	5.62393	5.57874
USP2	0.27701	-0.26977	-0.23499	-1.03319	1.25582	0.81561	0.92901	0.96431	-1.25818	-0.5623	-1.67758	-1.35167	-0.37719	-0.68806	0.55741	-0.04711
USP2-AS1	-1.02574	-1.28284	-2.34707	-2.62839	-0.82682	-1.52624	-1.60735	-1.97097	-0.75498	-0.93142	-0.76211	-1.8089	-0.65228	-1.13212	-1.80224	-2.61158
THY1	2.44825	2.29505	2.20177	1.86999	-2.63312	-2.47823	-2.09234	-2.62308	5.202	4.72239	5.20984	4.39823	-3.32193	-3.32193	-3.32193	-3.14838
NECTIN1	2.43057	2.62101	2.45546	2.11235	2.31476	1.84603	1.63271	2.19635	2.20479	1.81593	1.92046	1.49718	0.32509	0.21673	0.93279	1.37744
TRIM29	-3.21631	-3.2039	-3.32193	-3.18983	0.43816	-2.32311	0.23596	-2.0318	-3.32193	-3.32193	-3.21265	-3.32193	-3.23854	-3.32193	-3.32193	-3.32193
OAF	7.50127	7.67998	7.32034	7.16478	4.4641	5.73192	3.52136	5.56385	1.96577	3.17488	2.50961	2.47696	-0.08564	0.10152	-1.1521	0.00891
ARHGEF12	1.30783	0.79779	1.09905	1.48644	4.0517	2.30056	4.35109	2.30836	3.06002	2.00143	2.1243	2.85525	3.05781	2.12933	3.06908	2.46759
TBCEL	1.06354	1.05176	1.17708	1.76884	1.44115	1.33028	1.3186	1.63142	2.1888	1.89302	1.42675	2.50217	2.47648	1.58491	1.83278	1.3802
SC5D	2.03139	2.03794	1.82888	2.44193	4.06276	3.98365	4.68455	4.05474	2.20852	1.92512	1.93069	2.10554	3.74123	3.81411	3.42632	3.64029
MIR100HG	1.6173	1.85331	2.00317	2.65403	-2.309	-2.37585	-2.03048	-1.99462	1.17845	0.9828	0.83757	2.20305	-2.36667	-2.32872	-3.05658	-1.87836
UBASH3B	2.35411	1.76817	1.78958	1.44958	3.2492	0.49186	3.67385	0.66489	5.34576	4.29594	5.04701	4.61022	5.46364	4.58107	5.88915	4.62557
JHY	-0.66501	-0.52359	-0.1748	0.17099	-0.39202	-1.90145	-0.67503	-1.41062	1.15081	0.56099	0.8759	1.47125	-1.06862	-1.96945	-1.70918	-1.41458
SNORD14D	3.5481	3.18771	2.90654	3.64247	4.43935	3.32505	5.29417	4.26297	2.87942	1.82414	2.49695	3.57771	1.53384	3.73678	2.59981	1.43919
SNORD14C	4.21887	3.99445	3.77435	4.62026	4.26327	3.30873	4.42657	4.16455	4.21355	2.37929	4.19951	2.76314	4.47316	3.64302	3.2269	2.80433
CLMP	4.86257	4.67612	5.17206	4.98036	-0.25629	-1.66411	0.2734	-1.83078	5.53334	5.01152	5.95598	5.72687	4.93954	2.52261	4.8244	3.34481
GRAMD1B	0.79259	0.46652	0.42015	0.32105	-1.21947	-2.69466	-1.02268	-2.72493	2.18246	2.83035	2.15827	3.15937	-2.14176	-3.20543	-2.93393	-2.40076
ZNF202	1.80984	1.5221	1.42318	1.78325	1.62397	1.66373	1.69337	2.00603	1.95285	1.44099	1.91797	1.76133	1.98906	2.03372	1.55374	1.72838
VWA5A	-2.87447	-2.46093	-2.21838	-1.00622	-0.08274	1.86951	0.02357	1.43802	-3.13177	-2.88827	-3.15128	-2.89462	-3.19105	-3.19149	-3.32193	-2.6516
OR8G3P	-0.6903	-1.03241	-1.42021	0.63969	0.91866	2.12506	0.90982	1.70465	-3.32193	-3.32193	-3.32193	-3.32193	-3.32193	-3.32193	-3.32193	-3.32193
OR8G5	-0.02782	-0.10065	-0.89052	0.13092	0.16853	0.39922	0.34311	0.71726	-3.32193	-3.32193	-3.32193	-3.32193	-3.32193	-3.32193	-3.32193	-2.78345
OR8D1	-2.53127	-2.30471	-2.60076	-1.59795	-2.9757	-0.85728	-2.899	0.16354	-3.32193	-3.32193	-3.32193	-3.32193	-3.32193	-3.32193	-3.32193	-3.32193
OR8B7P	-0.7209	-0.38924	0.48614	0.57141	-3.32193	2.70135	-3.32193	3.61522	-3.32193	-3.32193	-3.32193	-3.32193	-3.32193	-3.32193	-3.32193	-3.32193
OR8B5P	-0.70903	-0.78927	-0.98802	-0.22824	-2.26073	2.14131	-3.32193	3.22207	-3.32193	-3.32193	-3.32193	-3.32193	-3.32193	-3.32193	-3.32193	-3.32193
OR8A1	-2.6105	-2.32076	-2.28122	-1.35962	-2.55312	-0.7617	-2.88151	0.15824	-3.32193	-3.32193	-3.32193	-3.32193	-3.32193	-3.32193	-3.32193	-3.32193
TBRG1	3.09491	3.09409	2.92456	3.50288	2.49467	3.03343	2.68719	3.05633	2.22884	2.34493	2.12783	2.26193	2.12038	2.40406	1.84344	2.13284
SIAE	2.75617	2.87274	2.8492	3.17508	1.29216	2.09908	1.41815	2.07238	2.81756	3.11298	2.80636	3.01959	2.14584	1.70322	1.5199	1.61316
RNA5SP352	3.4731	4.59415	3.85896	4.2563	1.72152	3.05285	2.31203	2.48831	4.02304	3.75536	3.73337	1.50862	2.21337	1.49203	1.5185	2.20843
SPA17	1.00621	0.85368	0.67948	0.44487	-0.61937	0.35156	-0.6066	-0.04538	1.92943	1.66768	0.9547	0.61136	1.38492	0.61517	0.77291	0.41884
NRGN	5.53441	5.54074	5.65519	4.66244	1.16376	2.08283	0.05778	2.57369	5.72118	4.94765	5.14889	3.22871	1.59759	1.77926	1.46498	1.7647
ESAM	2.99894	3.14908	3.74969	2.79598	-1.89257	-1.93654	-1.00878	-2.1427	-2.16114	-1.48881	-1.0425	-1.99319	-3.04903	-2.82115	-2.3111	-1.37588
MSANTD2	2.46592	2.41814	2.48924	2.62756	0.68306	1.40728	0.76922	2.02932	0.49677	1.21854	0.68015	1.96416	0.64036	1.41772	0.89579	0.77178
ROBO3	0.82213	0.97069	0.84904	0.90382	-0.721	0.34791	-1.70734	0.11823	2.15052	2.57755	2.84007	1.87203	-0.21498	-1.00964	-0.88109	-1.13374
ROBO4	5.83537	5.76852	5.66789	5.23863	-3.25585	-3.32193	-3.18048	-3.144	3.13276	3.57047	3.5103	3.41638	-1.58456	-2.89477	-2.81457	-2.60284
CCDC15	1.85765	2.08276	2.13407	2.48678	-0.56899	-1.8418	-0.39417	-1.77892	0.78853	-0.56886	0.46844	0.2426	0.45765	-1.10687	-0.68415	-1.16656
SLC37A2	5.78468	5.58735	5.80913	6.15216	-2.82224	-2.42493	-2.57151	-2.95479	1.66571	2.82808	2.19138	3.14253	-2.58375	-3.19087	-3.0202	-3.15987
TMEM218	1.4877	1.3546	1.30644	1.72127	2.50404	2.05342	2.13339	2.15605	2.09582	2.16157	2.04681	2.01214	2.02047	1.97092	0.90766	1.58037
FEZ1	-3.14468	-2.9507	-2.7714	-2.91044	-1.82003	-2.98354	-1.88451	-2.25952	0.1997	0.86948	0.3693	0.52448	-2.10074	-2.54427	-2.62233	-3.14871
EI24	5.36063	5.12377	4.79854	5.21588	6.28409	5.85619	6.34341	5.81299	6.53089	6.00622	5.98475	6.4466	5.87862	5.68292	5.49457	5.63018
STT3A	5.16761	5.12701	5.18105	5.39548	6.33496	5.84074	6.48908	5.85957	5.78398	5.4884	5.66779	5.88291	4.97876	4.81948	5.53625	5.29588
CHEK1	2.53419	2.62228	2.85252	2.89445	3.67879	2.66043	3.98165	2.62783	3.47848	2.63355	2.98137	3.3245	3.86431	3.61565	3.63677	3.26896
HYLS1	-0.14838	-0.5544	-0.25871	-0.1254	1.8283	1.94175	1.64136	2.01435	2.87173	2.1754	2.19445	1.82158	2.31205	2.25911	1.41666	1.74754
PUS3	4.20184	4.37362	3.96842	4.37967	3.28189	3.23138	3.51772	3.29814	2.61216	2.81262	2.90691	2.98795	2.22275	2.5941	2.16576	2.60675
CDON	0.54782	0.44953	0.30609	0.55384	0.73748	0.19386	0.84396	0.47394	-2.81043	-2.38243	-2.85804	-2.34245	-1.78939	-1.41752	-1.53671	-1.17472
RPUSD4	3.51944	3.20323	3.00081	3.05012	3.35724	3.07743	3.41617	3.13824	3.79568	4.07184	3.99029	4.10291	3.54669	3.29564	3.39717	3.28754
FAM118B	3.29375	3.24445	3.27605	3.33067	3.20997	3.73788	3.7501	3.64214	2.87274	3.02445	3.15058	2.86274	3.09195	3.339	2.90659	2.99587
SRPRA	5.73692	5.96728	6.07706	6.04144	6.3312	7.01882	6.91845	6.51751	5.0005	4.6445	6.13771	5.16126	3.5249	3.53797	4.49224	3.88794

FOXRED1	3.73763	3.55356	3.48771	3.18136	3.99327	4.2474	3.30512	4.3	2.43998	2.25802	2.62831	1.49482	2.46674	3.12459	2.32917	2.55676
TIRAP	2.16589	2.05352	1.75595	2.07721	3.11658	2.97408	2.85634	3.03972	1.83464	2.08659	1.73297	2.24199	2.61139	2.24481	2.03674	2.30772
DCPS	3.12705	2.65791	2.7465	2.54325	3.95506	5.3517	3.48101	5.36804	3.89485	3.27144	4.08425	2.64798	2.27874	2.1142	2.20467	1.58119
GSEC	-0.26945	0.01798	-0.0158	0.50166	-0.47442	0.71251	-1.42497	0.83679	0.12893	0.82252	0.2904	0.0954	-0.97626	-0.9348	-1.00559	-1.10103
ST3GAL4	3.50637	3.1631	3.20521	3.03125	3.76578	4.21225	3.01487	4.34335	4.1684	4.07855	4.26479	3.75632	2.67306	2.6337	2.76653	2.70309
KIRREL3	-1.79425	-2.37725	-1.76014	-1.84527	-2.15219	-1.42001	-2.79639	-1.45012	2.85902	2.84673	2.84237	1.78551	0.52311	0.78769	2.16796	1.34853
FLI1	2.54742	2.50465	2.34611	2.46133	-3.26488	-3.32193	-3.32193	-3.16752	1.8389	2.43854	2.09809	2.12387	2.03405	0.81617	1.08547	1.19363
SENCR	1.83513	1.54534	1.55265	0.38707	-3.32193	-3.32193	-3.32193	-2.54693	2.92717	3.24768	2.72333	1.78424	1.71606	1.55939	0.85809	0.93271
C11orf45	0.70624	0.07372	0.11208	-0.51266	-3.20038	-3.32193	-2.8506	-3.00443	1.37957	1.28395	1.48619	1.58679	-2.75008	-2.46767	-3.32193	-3.05653
ARHGAP32	0.73589	0.54279	0.85137	0.6033	1.67962	0.53658	2.0396	0.53039	1.44386	1.37222	1.41529	2.45725	1.73729	1.47751	2.63618	2.12968
TMEM45B	-1.83918	-1.2634	-2.17459	-1.03382	-0.32749	-0.95797	-0.73072	-0.26549	-3.32193	-2.74227	-2.98209	-3.00803	-1.93956	-2.18382	-3.00602	-2.73656
NFRKB	3.03314	2.77066	2.73877	2.92913	3.27409	3.19259	3.29243	3.41869	2.80578	3.03014	3.14271	3.45713	2.41698	2.65694	2.79793	2.60639
PRDM10	1.06437	1.02334	1.11614	1.37794	1.24394	0.33412	1.51915	0.46648	1.35089	0.98184	1.3164	1.49026	1.2977	1.23228	1.63092	1.45442
ST14	-0.20556	-0.08761	-0.36512	-0.40893	-1.20619	-1.11782	-1.32273	-1.25732	-2.82136	-2.42644	-3.07725	-2.57642	-2.14688	-2.81823	-2.57228	-1.27438
ZBTB44	1.83038	1.85134	1.67365	2.16394	1.2891	0.34449	1.92184	0.65862	2.02408	2.0687	1.92018	3.08268	2.23932	2.25138	2.82176	2.51166
ADAMTS15	-1.13383	-0.58174	-0.81087	-0.07967	-3.16332	-2.56352	-2.85401	-2.74839	1.68564	2.25802	2.3633	2.79657	5.19442	4.25546	6.40521	5.98875
SNX19	2.68509	2.75189	2.49233	2.95531	-2.63799	-2.75506	-2.21404	-2.57395	3.32416	2.89262	2.86786	3.73968	3.49482	3.4705	3.34388	3.75659
NTM	-3.32193	-3.32193	-3.32193	-2.96386	-3.32193	-3.32193	-3.32193	-3.32193	1.58116	1.30905	1.85812	1.56874	-3.32193	-3.32193	-3.17589	-3.32193
MIR4697HG	-3.32193	-3.32193	-3.32193	-3.32193	-2.66706	-2.78004	-2.33494	-2.54368	-0.60159	-1.30632	-1.45529	-1.60838	-3.32193	-3.32193	-3.12848	-2.41119
IGSF9B	-2.08984	-1.25848	-1.94437	-2.08164	-1.06446	-2.69376	-1.8557	-2.47209	-0.42872	-0.75063	-0.20315	-1.43998	-2.81324	-2.81476	-3.32193	-2.59226
JAM3	0.07582	-0.60092	-0.08404	-0.56667	-0.64796	-0.03188	-0.51855	0.04464	-1.39082	-2.25265	-1.70605	-2.17476	-0.65422	-0.9065	-1.05701	-0.73648
NCAPD3	3.80624	3.29721	3.45491	3.00991	4.42236	3.36978	4.20649	3.4764	4.22869	3.18976	3.79121	2.85688	4.47048	4.14267	4.17343	3.9013
VPS26B	2.92945	3.01943	2.92654	2.69211	4.16398	3.75352	3.92573	3.68941	4.01544	3.79892	4.21269	3.62858	3.71553	3.70112	4.32107	4.08985
THYN1	4.01607	3.78981	3.70387	4.06172	2.98914	3.83252	2.94297	3.42938	3.77857	3.51123	3.76635	3.75517	3.4637	3.74674	3.53667	3.42164
ACAD8	1.98631	1.86138	1.72432	2.17791	2.0347	2.63818	1.74054	2.39151	1.7686	1.69604	1.70011	1.83451	1.95293	2.00367	1.42344	1.65585
GLB1L3	0.08828	-0.18591	-0.14914	0.11876	-2.58788	-3.20061	-2.91706	-3.32193	-3.32193	-3.32193	-3.19775	-3.32193	-3.32193	-3.32193	-3.32193	-3.32193
GLB1L2	-3.32193	-3.32193	-2.99699	-3.32193	1.90142	1.48273	1.37176	1.11563	-3.32193	-3.32193	-3.32193	-3.32193	-3.09907	-2.58572	-2.46252	-2.81996
ZMYND11	3.83544	3.73317	3.67408	4.14107	3.8252	3.02234	4.33875	3.15313	3.2931	3.27622	3.3074	3.93817	5.25245	5.05186	5.14749	4.64844
DIP2C	0.69839	0.75446	1.20915	1.17062	1.38819	-0.3619	1.13631	-0.42116	2.3606	2.46893	2.37807	2.9065	2.54883	2.77907	3.41025	3.43327
PRR26	-2.37838	-2.51157	-2.55975	-1.94934	0.1114	-0.67544	0.15142	-1.0311	-3.32193	-3.32193	-3.32193	-2.83873	-2.71214	-2.59492	-2.47286	-2.06346
LARP4B	3.61226	3.6272	3.43132	3.51635	3.86344	3.43438	4.08539	3.51436	3.90076	3.4998	3.75267	3.81678	4.95526	4.87345	5.01634	4.70845
GTPBP4	5.38958	5.17886	5.02018	5.2164	4.90144	4.44026	5.3335	4.5677	4.93344	4.13634	4.57824	4.32122	6.16889	5.37282	5.13941	4.64846
IDI2	1.47724	1.83245	2.16923	2.0487	1.40422	2.07028	2.22791	2.57088	0.86863	0.76303	1.19471	1.69604	3.56888	2.71466	2.85656	2.60009
IDI2-AS1	3.40907	3.35105	3.25397	3.62162	4.46151	4.42635	4.52947	4.13812	3.69681	3.56445	3.24618	2.97898	5.1285	5.88325	4.86761	5.10716
IDI1	3.94989	4.18449	3.95395	4.7441	4.78791	5.71509	5.27633	5.87889	4.20746	4.00847	3.64768	3.47362	6.11033	6.89045	5.44154	5.72173
WDR37	2.58838	2.63921	2.59306	3.08772	1.77232	1.87406	1.60692	1.87748	2.47376	2.42751	2.27312	3.0272	3.67763	3.87041	3.62486	4.10884
PITRM1	4.06015	4.0651	3.86977	4.3275	4.15817	3.64798	4.33754	3.64468	4.5002	4.1208	4.22628	4.48932	5.47595	5.28608	5.43321	5.83204
PITRM1-AS1	3.3157	3.18433	3.06144	3.35214	3.25682	2.71862	3.2225	2.67217	3.63749	3.13189	3.43155	3.26241	4.16155	4.10272	4.29403	4.68557
KLF6	5.58997	5.88409	5.97641	5.46536	4.98683	4.77291	5.74527	4.52648	6.07601	5.97965	5.67411	6.04005	6.34072	5.78168	6.51661	5.88983
LINC00702	-1.03233	-1.04344	-1.5978	-1.72659	-3.32193	-3.32193	-3.32193	-3.32193	-1.73966	-1.85472	-1.78788	-1.47292	0.50318	-0.29626	0.404	-1.0603
MANCR	0.39253	-0.05582	1.14219	0.29002	-1.9742	-3.32193	-1.61531	-3.32193	2.71898	2.71603	2.40397	3.48216	2.87815	2.17562	1.50597	1.95864
AKR1E2	-3.32193	-3.32193	-3.32193	-3.32193	-3.32193	-3.32193	-2.94371	-3.32193	2.28615	1.85995	2.12363	1.45857	1.99063	2.1737	1.14757	1.32933
AKR1C1	3.59884	3.96937	3.93156	5.52315	3.45725	4.25872	3.28599	4.07882	-2.30099	-2.64593	-2.7773	-1.6142	4.47304	5.13499	5.30765	5.35139
AKR1C2	0.98983	1.35268	1.38113	2.71704	5.74283	5.96445	5.70212	5.6702	-2.33833	-2.56063	-2.86426	-2.38759	4.18563	5.05808	4.90299	5.06749
AKR1C3	3.56449	3.71926	3.64579	4.56862	4.95031	5.15082	4.93849	4.98699	-0.29228	1.03467	-0.50802	1.51597	5.80925	6.89256	6.82435	6.55748
AKR1C4	-1.23651	-1.10748	-0.78488	-1.71426	-0.31099	0.65622	-0.42767	0.09032	-3.32193	-2.84579	-3.32193	-3.32193	-1.94988	-1.37748	-0.63934	-0.79874
LINC02561	1.83869	0.89715	2.47192	1.68477	-1.78323	-1.18379	-3.32193	-3.32193	-3.32193	-1.23631	-1.58912	-2.27702	-0.89076	-1.86194	-3.32193	-2.25533
AKR1C7P	2.14922	2.16348	2.41672	1.7588	-1.63946	-2.41367	-3.32193	-1.70705	-2.31619	-1.90378	-3.32193	-1.22152	-1.18546	-0.64035	-1.52126	-3.32193
TUBAL3	0.56687	0.5898	0.09983	-0.76016	1.86323	1.57555	2.32646	1.41527	-0.85839	0.29144	-0.55359	0.36368	-1.72307	-1.31018	-1.52585	-1.06155
NET1	4.27216	4.14651	4.19484	4.14574	3.65241	2.65593	3.75632	2.98682	6.58717	6.27421	6.95827	7.25603	4.81952	4.63306	4.73616	4.32851

ASB13	3.89311	3.81875	3.6545	3.48873	3.9043	3.96549	3.20085	3.85049	4.40523	4.72887	4.40765	4.37192	4.42093	4.52097	4.61785	4.88179
ANKRD16	2.09118	1.9902	2.04538	1.39523	2.82206	3.14528	2.32943	3.18951	2.03941	2.33403	2.24059	1.78378	2.07537	2.04287	2.31294	2.47172
RPL12P28	0.7546	0.2251	0.50207	0.13988	1.51788	2.47407	2.03566	3.2821	0.47653	0.40533	0.65972	1.41026	1.30013	1.14162	1.04959	0.24391
FBH1	3.81376	3.75637	3.73277	3.69067	4.21113	4.13285	4.14403	4.15486	3.94202	3.84057	3.9601	4.27124	4.60284	4.3602	4.52971	4.4599
IL15RA	4.01822	3.50977	3.50904	3.21208	-1.16916	-1.56691	-1.39935	-1.21681	0.59933	0.27752	0.42114	0.04452	-0.50975	-0.73657	-1.92617	-1.69704
RBM17	4.43443	4.15902	4.20888	4.08979	3.93109	3.95337	3.99511	4.23011	4.35603	3.69991	4.05495	3.48188	4.65329	4.63507	4.13376	3.83857
PRKCQ	-3.01678	-2.9835	-2.96685	-3.32193	-3.32193	-3.32193	-3.32193	-3.32193	1.72391	1.72361	1.78364	2.1909	-3.32193	-2.87012	-3.32193	-3.02343
PRKCQ-AS1	-2.64544	-2.78822	-2.76366	-3.32193	-3.09618	-3.32193	-3.32193	-3.32193	0.89655	0.53213	0.85967	0.56562	-3.32193	-2.62355	-3.32193	-3.32193
LINP1	1.61462	1.96343	1.72626	0.46691	-3.32193	-3.32193	-3.32193	-3.32193	-0.75659	-1.9256	-3.32193	-3.32193	-3.32193	-3.32193	-3.32193	-3.32193
LINC00707	-2.99676	-3.32193	-2.94396	-3.32193	-3.17479	-3.32193	-3.32193	-3.32193	4.47806	3.58161	2.90408	2.75245	-3.32193	-3.32193	-3.32193	-3.32193
SFMBT2	-3.2136	-3.20088	-3.19445	-2.65232	-3.06045	-3.21244	-3.22056	-3.19397	-1.92604	-1.8341	-2.46769	-1.6359	-3.32193	-3.32193	-3.32193	-3.02528
ITIH5	-2.75875	-2.88118	-2.73421	-3.05777	0.90392	1.71655	1.26183	1.63407	-3.32193	-3.32193	-3.32193	-3.32193	-3.32193	-3.20711	-3.32193	-3.32193
ITIH2	-2.56552	-2.12696	-2.69488	-2.66192	6.96546	7.05377	7.31946	6.81306	-2.47298	-3.03321	-2.54453	-2.80026	-2.699	-3.08393	-2.79714	-3.32193
KIN	1.8451	2.00273	1.94927	2.64189	2.31999	2.61612	2.80988	2.81135	1.68884	1.87378	1.91725	2.00474	2.00326	2.09408	1.69109	1.58999
ATP5F1C	6.32219	6.3304	6.32392	6.49237	7.05802	7.36223	7.23218	7.28789	6.65902	6.77815	6.61357	6.51745	7.29411	7.06537	6.68872	6.36029
TAF3	3.35272	3.13289	2.9353	3.36682	2.53522	2.75188	2.66908	3.15246	2.80109	1.9268	2.2983	2.54937	2.54927	1.99275	2.50636	2.20422
GATA3-AS1	-3.32193	-3.32193	-3.32193	-3.32193	0.32585	1.85069	0.10749	1.5563	-2.85692	-2.61614	-2.90082	-2.15795	-3.32193	-3.32193	-3.32193	-3.32193
GATA3	0.10863	-0.10446	-0.12075	-1.2132	3.47699	3.35835	3.0051	3.54923	4.91862	5.06832	5.10147	4.63351	-3.32193	-3.32193	-3.32193	-3.32193
SFTA1P	-2.36446	-3.32193	-3.32193	-3.32193	-2.83647	-3.32193	-3.32193	-3.32193	3.93715	2.75068	2.00107	1.42731	4.80318	4.31317	3.11155	2.64579
CELF2	-2.43567	-1.95063	-2.99853	-1.79875	-3.12013	-3.32193	-3.14307	-3.32193	-3.32193	-3.32193	-3.32193	-3.32193	0.7614	0.7471	0.07277	0.76018
USP6NL	0.80768	0.58942	0.66232	0.68782	1.49578	1.02815	1.88409	0.6558	2.13971	1.78063	1.9355	2.09438	2.31635	1.85239	2.47852	2.07806
ECHDC3	-1.13541	0.28251	-0.22184	-1.27269	-0.70625	1.81221	-0.45409	1.76357	2.48316	2.51535	2.01338	2.00219	4.76723	4.62292	4.54205	4.21532
PROSER2	5.39848	5.29617	5.04697	3.30593	4.06743	4.13945	3.53246	4.23841	2.73892	2.46854	2.2288	0.99139	4.79206	3.95102	4.16041	3.78318
PROSER2-AS1	2.59696	2.47365	2.35023	0.12109	1.63705	1.24912	0.75975	1.45964	0.26565	-0.189	0.1141	-1.19399	2.30661	1.41806	1.92188	1.47689
UPF2	3.86478	3.60295	3.57113	4.27627	3.70578	3.71381	4.23692	4.11865	3.67905	3.50658	3.49527	4.07943	4.14942	3.57505	3.84831	3.72734
DHTKD1	2.56555	2.89539	3.1067	3.47031	3.60468	3.5888	3.63607	3.45354	3.22632	3.03173	3.04088	3.11343	5.44849	5.5454	5.55911	5.69094
SEC61A2	4.54032	4.54924	4.54942	4.63631	3.83964	4.73179	3.94482	4.70082	3.3621	3.13994	2.89729	3.29479	4.67517	5.07817	4.04324	4.56539
NUDT5	5.20612	5.16586	5.15796	5.38961	4.25839	4.76634	4.59347	4.57433	4.12568	3.64857	3.65436	3.83344	5.13627	5.25146	4.52298	4.65209
CDC123	6.65113	6.54604	6.21606	6.44551	5.86826	5.95014	6.01594	5.93893	6.27294	5.61953	5.9621	5.67234	6.45639	6.67625	6.21155	6.36112
CAMK1D	-2.10242	-1.74255	-0.9735	-1.73083	-3.21727	-2.9786	-3.32193	-3.17742	2.27692	2.7864	2.50099	3.36258	3.52201	4.05178	4.45077	4.49551
OPTN	5.12028	5.53403	5.87902	5.80862	5.10742	4.71072	5.4012	4.42856	6.03273	6.24275	6.04395	6.60336	6.06732	6.38174	6.56849	6.4575
MCM10	3.4082	2.6363	2.27924	2.34373	3.60445	1.95755	3.40694	2.36285	3.35373	1.49416	1.6288	0.942	4.91438	4.28097	3.6365	3.55131
PHYH	2.98692	2.85024	3.20329	3.3109	5.3097	4.64942	5.38388	4.33399	2.48454	1.7267	2.1999	2.08226	3.49416	1.89523	3.54769	2.30413
SEPHS1	5.15431	4.95822	4.83184	4.6867	5.40096	5.55908	5.08007	5.38763	4.91731	4.43691	4.41673	4.2711	5.92437	5.94354	5.51736	5.68836
BEND7	1.33325	1.29916	1.06724	1.12191	1.60657	1.20579	1.60998	1.27124	-0.02566	-0.06791	-0.66096	0.28326	1.39711	1.26823	1.54027	1.47788
PRPF18	4.11509	3.92476	3.73477	4.11532	3.82462	3.30796	4.14225	3.26356	3.74959	3.50796	3.36698	3.36706	4.2412	3.97507	3.81208	3.71099
FRMD4A	2.5232	2.45567	2.6239	2.50766	1.87237	1.83744	1.37269	2.40076	3.69605	3.53398	3.87643	3.9954	5.16237	5.0465	6.32141	5.40025
FAM107B	3.50544	3.39407	3.74096	3.61074	5.00321	3.97437	5.54826	3.93579	2.83399	3.00018	2.25852	3.76345	5.1847	4.71781	4.52279	4.5973
CDNF	2.12253	2.25544	2.08355	2.04057	3.33151	2.19623	3.54076	2.36749	2.5681	2.42457	2.30012	2.6934	3.67423	3.69271	3.74393	3.56226
HSPA14	3.97322	3.78402	3.68261	3.95669	4.33619	3.64928	4.65894	3.75651	3.93085	3.36146	3.4906	3.61686	4.89928	4.84817	4.49082	4.32016
SUV39H2	3.16009	2.92632	2.84138	2.70123	3.20134	2.1636	3.46109	2.4894	3.23455	2.39049	2.74102	2.49341	4.15134	3.72836	3.97169	3.51395
DCLRE1C	1.77086	1.6897	1.76531	1.94415	1.87958	1.55614	2.10148	1.99863	2.00268	1.67257	1.69567	2.17622	2.77555	2.45987	2.28663	2.45934
OLAH	-0.67739	-1.34082	-1.28222	0.42586	-2.96706	-2.32494	-3.32193	-2.48925	-3.32193	-3.32193	-3.32193	-2.931	-0.89634	-0.53645	-1.51144	-0.2027
ACBD7	1.70507	1.44194	1.5171	1.04416	0.09516	0.26312	0.02698	0.03472	1.36127	0.32088	0.16321	0.11413	3.22565	3.38598	3.49561	3.51418
RPP38	3.99131	3.89331	3.73817	3.87477	4.14357	4.01437	4.11516	4.2474	3.56467	3.22772	3.31804	3.03914	4.61379	4.57865	3.92494	4.04813
NMT2	3.76461	3.71477	3.79622	3.62976	2.83335	2.94111	3.10836	2.94873	4.1029	3.74204	3.61622	3.24205	5.61056	5.27552	5.56195	4.55647
FAM171A1	4.42427	4.13294	4.34693	3.87446	1.06481	-1.56451	0.44776	-0.70487	5.08436	4.5861	4.97113	4.25785	6.5792	5.84704	7.09609	6.60786
MINDY3	2.37313	2.6535	2.59735	3.11564	2.28762	2.01738	2.93327	1.76216	2.73694	2.42353	2.34159	2.81128	3.12826	2.97897	2.40453	2.42559
PTER	2.74246	2.58434	2.51794	2.96983	3.99931	3.33314	4.60914	3.23167	0.99498	1.61951	0.93181	2.08879	3.40129	3.12551	3.2086	3.10355
C1QL3	-0.98645	-1.04582	-0.53507	-0.11197	1.19005	1.31744	2.38254	0.59177	-1.8317	-1.99941	-2.60383	-2.01412	0.20007	-0.03667	0.33252	0.87318

RSU1	4.73338	4.68297	4.83349	4.88197	4.82012	4.85379	5.03966	4.54732	4.98442	4.69312	4.97573	4.75745	5.80135	5.57694	5.93474	5.23608
CUBN	-1.36288	-0.82858	-0.33772	-1.59659	-3.12005	-3.32193	-3.32193	-3.22418	-1.52852	-1.10859	-1.85169	-0.69051	-2.01298	-1.83938	-2.43902	-1.45569
TRDMT1	0.20601	0.2326	0.13091	0.02959	-1.22254	-1.96341	-0.63652	-1.81598	0.02327	-0.05951	-0.14986	0.15403	0.71965	-0.03778	0.62472	-0.35295
ST8SIA6	-3.32193	-3.32193	-2.55637	-3.32193	-3.32193	-3.32193	-3.32193	-2.88747	-0.34591	-0.57823	-0.11037	-0.9431	-2.77309	-3.32193	-3.32193	-2.95645
ST8SIA6-AS1	2.8482	3.10941	3.03464	2.86224	0.95762	2.43744	0.8919	1.85414	-2.40178	-3.32193	-3.32193	-3.32193	-3.32193	-3.32193	-3.32193	-3.32193
HACD1	2.64093	2.71853	3.11806	3.02726	3.02233	3.5655	3.27491	3.47373	4.35889	3.72009	4.25948	3.4955	4.56582	3.67366	3.84736	3.09156
STAM	4.54181	4.44876	4.46477	5.0124	3.53039	3.24854	4.0132	2.98563	4.0344	3.80767	4.05709	4.21516	5.66002	5.53688	5.03418	4.669
TMEM236	-2.13392	-2.3536	-2.44112	-2.54182	-3.15759	-3.32193	-2.02417	-3.32193	-2.74148	-3.13673	-2.94976	-2.46759	-1.20476	-1.63591	-2.9757	-2.24543
CACNB2	-1.51735	-2.47428	-1.56013	-1.49493	1.83763	2.41842	1.80374	2.4051	-3.13542	-3.02447	-3.15458	-2.78566	-2.17841	-1.95701	-2.15399	-1.64324
NSUN6	2.00978	1.74947	1.83583	1.73166	2.46176	2.4902	3.01322	2.60031	1.00608	0.75126	0.79238	0.96419	1.61117	1.42263	0.8266	1.15179
ARL5B	1.93395	1.78796	1.81215	2.49609	4.45492	3.47995	5.44165	2.78639	2.94902	2.09793	2.35206	2.9652	4.86206	3.48286	4.76678	3.74116
MALRD1	-3.32193	-3.32193	-3.32193	-3.32193	-3.32193	-3.18331	-3.32193	-3.16018	-2.29091	-2.95618	-2.72104	-2.36172	-0.54356	-0.23871	-0.89414	-0.58953
PLXDC2	-3.32193	-3.32193	-3.22005	-3.32193	-3.28458	-3.32193	-3.24107	-3.32193	-3.32193	-3.32193	-3.32193	-3.32193	-0.61801	-0.75693	0.06946	-0.37899
NEBL	-2.67261	-2.60999	-2.31464	-2.66744	-0.39773	0.4543	-0.09184	0.2243	-3.32193	-2.97249	-3.01679	-2.97775	1.01386	1.58805	1.72331	1.32619
NEBL-AS1	-1.60851	-3.32193	-2.08914	-3.32193	1.58696	0.67626	-0.07239	0.99839	-3.32193	-3.32193	-3.32193	-2.26506	0.22827	1.87133	2.15459	1.68441
SKIDA1	-1.23667	-1.58194	-1.08761	-1.14891	0.55837	-0.08425	0.30492	-0.31711	-1.5464	-2.48849	-0.87831	-1.85881	0.95097	1.02514	2.04465	1.08642
MLLT10	2.39588	2.11756	2.40324	2.6078	2.50386	2.5206	3.02365	2.59173	2.7716	2.74542	2.92414	3.34995	3.6293	3.23527	3.1368	2.8479
DNAJC1	4.7267	4.82162	4.80379	5.25469	5.25758	5.85614	5.54291	5.70135	4.02643	3.75874	3.74168	4.11597	4.69729	4.42401	5.00177	4.37105
COMMD3	4.96222	4.93749	5.07811	5.3999	3.47773	3.98478	3.75599	3.91386	3.37581	3.47972	3.40868	2.94224	3.63653	3.77501	3.36898	3.21753
COMMD3-BMI1	5.76001	5.56007	5.88541	6.151	5.07776	4.60245	5.63345	4.49802	5.22818	4.86143	5.25669	5.01644	5.81601	5.30357	5.54969	4.79835
BMI1	4.26027	3.71783	4.45646	4.50326	3.98115	2.78604	4.70213	2.87191	4.35678	3.7902	4.26097	4.30771	5.21595	4.21195	4.87898	3.63933
SPAG6	-3.32193	-3.32193	-3.32193	-2.99762	0.99354	0.29929	1.16568	0.39089	-3.32193	-3.32193	-3.32193	-3.32193	-3.32193	-2.05719	-2.66941	-2.84715
PIP4K2A	2.83124	3.06809	3.54199	3.49673	2.5571	2.7772	3.92669	2.87561	2.61551	3.23435	3.61306	3.88675	3.62494	5.2921	5.90375	5.06753
ARMC3	-2.58611	-2.73903	-2.98536	-3.13291	-3.07293	-2.67984	-2.37214	-2.98417	-3.14702	-3.32193	-3.32193	-3.04663	-1.43709	-0.71139	-1.02407	-1.16397
MSRB2	2.52942	3.07393	3.21504	3.16688	3.36911	4.45773	2.79685	4.58694	2.78959	3.4932	3.01669	2.95849	3.63192	3.90506	3.18455	3.43839
YWHAZP3	1.91869	0.80944	2.27336	1.46217	1.35419	-0.24416	0.97464	-3.32193	1.91349	2.67524	2.04647	2.37421	0.99636	-0.11879	0.85353	0.71317
OTUD1	2.89847	3.45945	3.49721	2.83764	2.79113	1.8392	2.00984	2.18199	2.69307	3.54023	2.93328	2.01379	0.47021	0.98534	0.44701	0.85145
KIAA1217	2.67769	2.73758	2.69437	2.94607	-0.01804	-1.18025	0.47207	-1.42143	-0.69536	0.35309	-0.55576	0.73038	1.66025	1.8315	2.54404	2.99844
ARHGAP21	3.18894	3.201	3.41519	3.3088	3.31108	2.04443	3.66285	2.16096	3.89141	3.4351	3.46258	4.46013	4.65762	4.13429	4.81433	4.5409
PRTFDC1	1.77923	1.8692	1.98879	2.26654	-2.67474	-3.32193	-2.37095	-3.32193	4.17884	4.13067	3.34281	4.21083	3.47472	4.05701	3.35747	3.34322
RN7SKP241	1.72269	1.13454	1.39386	1.29681	-2.22923	-3.32193	-1.51888	-3.32193	3.84403	3.48014	2.26212	2.13693	2.16603	1.86372	0.70296	0.94253
ENKUR	-0.74886	0.48923	0.21444	-0.46399	-2.1759	-2.66423	-1.44769	-1.83042	-2.58729	-2.52744	-2.84217	-3.32193	-2.78834	-2.00785	-2.25789	-2.68274
THNSL1	0.79836	1.11751	0.90457	1.55835	0.78117	0.63237	1.21321	1.01807	0.76183	1.3836	1.13835	2.18522	2.24456	2.30168	2.04605	2.10394
GPR158	-0.03806	0.30894	0.04025	0.51759	-0.12163	-0.21464	0.94045	-0.73257	-1.39503	-0.63831	-0.64129	-0.12188	-1.11398	-0.82963	-0.65042	-1.20654
APBB1IP	3.9045	4.01838	3.78139	4.00654	2.11746	1.42342	2.98312	1.00104	3.8041	3.31484	3.70551	3.69842	6.05282	6.01274	5.78405	5.70467
PDSS1	3.5621	3.09112	3.1065	3.03894	3.26696	2.9195	3.62829	3.23298	2.4099	1.84143	2.8915	2.13434	3.58639	2.93182	3.3693	2.15024
ABI1	4.17755	4.31661	4.39119	4.60725	4.0323	3.26961	4.57094	3.38265	4.54393	4.44743	4.53027	4.71794	5.1083	4.69234	4.44133	4.41684
ANKRD26	2.31245	2.68965	2.72408	4.11368	1.89996	1.81115	2.22644	2.4113	1.85215	1.63527	1.5583	2.61629	2.67325	2.42137	2.98714	2.67045
YME1L1	5.10228	5.11173	5.05122	5.31077	5.25562	4.29378	5.72416	4.02209	5.38308	4.69397	5.048	5.09824	6.11876	5.26029	5.96769	5.42047
MASTL	3.74134	3.34033	3.50057	3.39076	3.82578	2.80167	4.1856	3.0727	4.00679	2.85432	3.19828	2.87858	5.32933	5.02847	4.97855	4.45956
ACBD5	3.24956	3.39823	3.43675	4.02537	5.08061	4.80358	5.61552	4.84488	4.10342	3.88161	3.95553	4.21586	5.34813	5.50456	5.00556	5.03321
LRRC37A6P	-2.82222	-2.77142	-2.59609	-2.71544	-0.50722	-1.4538	-0.58491	-2.21841	-0.43145	-0.75563	-0.37371	-0.17656	-1.23689	-0.67167	-0.80027	-0.37102
RAB18	3.73148	4.04738	3.81383	4.30601	4.19877	4.11164	4.8915	3.93313	4.04782	3.81628	3.81684	4.32182	5.20198	5.0276	4.9453	4.76318
ARMC4	0.55178	0.55467	0.21355	0.32139	-3.13528	-3.32193	-3.32193	-3.32193	0.50898	-0.34021	0.01383	0.34658	-2.40222	-2.61292	-3.32193	-3.10944
MPP7	-2.14656	-1.96928	-2.5183	-1.63297	-1.2676	-2.61156	-1.57799	-3.12069	-0.93175	-2.09855	-1.75848	-2.03998	-2.74374	-2.94928	-3.01123	-3.15481
WAC-AS1	3.73469	4.03124	4.00868	4.53426	3.36974	3.66205	3.61305	3.88554	3.44087	3.78466	3.38222	4.22305	3.86825	4.12213	3.89039	3.89702
WAC	3.75876	3.94107	3.97213	3.81306	3.92694	3.34346	4.09342	3.34889	4.38076	4.3592	4.39221	4.54855	4.84552	4.48062	4.84416	4.64459
BAMBI	3.7744	4.23919	4.37868	5.4583	7.16363	6.65948	7.13972	6.52252	3.55062	3.91406	4.56634	3.96793	4.38618	5.81878	5.55204	6.49988
RPL21P93	1.63951	1.37518	1.23659	-0.99791	0.39323	1.49981	0.18531	0.50736	1.42373	2.56459	1.05174	-0.23055	-0.04335	-3.32193	0.54973	-1.97208
SVIL-AS1	2.59719	2.41938	2.72607	2.61397	3.81186	2.95434	4.06111	2.83618	3.77607	3.37552	3.52106	3.87893	4.49095	4.12776	5.10488	5.03657

PTCHD3P1	3.78976	3.36735	3.30158	3.48924	5.36548	4.68216	5.22246	4.46622	4.82391	3.99892	4.37586	4.02392	4.76949	4.0605	4.8272	4.57258
SVIL	2.86195	2.67283	2.86952	2.96722	3.97303	2.34117	4.33107	2.16802	4.76067	4.59475	4.80934	5.22192	5.69773	5.55437	6.86534	6.97499
JCAD	4.72144	4.61948	4.90685	4.84945	1.76319	0.93064	2.1653	0.27024	2.49492	2.08315	2.0105	2.41966	-2.72621	-2.72795	-1.10153	-1.63094
MTPAP	2.18706	2.06142	1.60403	2.23434	2.26991	1.83724	2.42554	2.17162	1.9391	1.66922	1.46311	2.32136	2.89973	2.81094	2.48054	2.57854
MAP3K8	2.58629	2.56215	2.62097	2.92781	3.95471	3.85623	3.94014	4.1487	0.85084	1.44292	1.04601	1.79547	1.66589	1.41912	0.1749	0.86765
SVIL2P	-0.2035	-0.18174	0.34077	0.05625	-1.76721	-2.5325	-1.96068	-3.32193	-1.75932	-1.40402	-0.80426	-0.84867	0.22939	-0.63569	1.40321	1.95341
ZNF438	1.47185	1.81121	1.59962	2.143	0.04923	0.45305	0.4216	0.36723	1.01716	1.65621	1.4331	2.3796	1.05707	0.86241	0.90406	0.9736
ZEB1-AS1	0.62142	0.88822	0.77238	0.77503	1.21403	1.2471	0.54751	0.96747	0.29902	0.99636	0.46488	0.5495	1.2828	1.64184	0.78639	1.0545
ZEB1	2.48547	2.65618	2.77386	3.17039	1.50969	0.90227	2.00406	0.87934	2.96481	2.79753	2.67222	3.59918	2.93933	2.7117	2.64205	2.52295
ARHGAP12	2.85006	2.97733	3.07037	3.6237	3.75847	3.11273	4.62193	2.88882	3.96875	4.07363	4.06062	4.97093	5.14009	4.85573	5.20703	4.85367
PPIAP31	6.17442	6.32187	5.68802	6.78335	2.86627	4.02413	2.46341	3.63324	4.10696	4.6979	3.92654	3.04878	2.69765	2.35567	2.05027	2.48756
EPC1	0.62037	0.45793	0.66987	1.12666	1.2604	1.04802	1.79495	0.96844	0.58384	1.1389	0.80375	1.89964	1.92031	1.86729	1.72996	1.95789
CCDC7	-1.15644	-0.92224	-0.78525	-0.38598	-1.0941	-0.32778	-0.75656	-0.71057	-1.79349	-1.12147	-1.77019	-0.85345	-1.07479	-0.91494	-1.15739	-0.70308
ITGB1-DT	3.67649	3.07454	3.37167	2.90524	2.05713	0.95735	2.25578	1.02465	2.67273	3.09913	2.55971	2.60461	3.57583	2.37254	2.47661	2.26242
PARD3	3.65404	3.54301	3.58681	3.74398	3.9448	3.36865	4.43008	3.29409	4.9309	4.85512	4.92981	5.13283	6.00469	6.13767	6.77634	6.15496
CUL2	4.17587	4.24187	4.06299	4.80278	3.46612	2.76081	4.10448	2.55462	4.04488	3.76642	4.00722	4.5155	5.2078	4.86841	4.8022	4.54444
CREM	2.92931	2.73166	2.6617	3.20886	1.76908	2.96038	2.03714	2.34738	1.13061	1.2698	0.71173	1.02613	2.79693	3.00555	1.99084	2.53308
CCNY	4.10849	4.12788	4.14663	3.71149	3.9228	3.41627	3.65837	3.45285	5.17658	4.60133	4.8661	4.55498	4.74879	3.89327	4.55809	4.12561
FZD8	0.79658	1.13537	1.46962	-0.35709	2.06399	-0.06377	1.4365	-0.04329	3.50587	4.46883	3.94693	4.62278	-1.91069	-2.51317	-2.87013	-1.61916
MIR4683	2.95706	2.7121	3.65385	1.33297	4.49068	1.991	3.17696	2.53563	6.20177	6.87582	6.31304	7.18423	0.67926	0.67443	-3.32193	-3.32193
NAMPTP1	5.65331	6.20356	5.93498	6.84098	2.17167	1.76921	2.54464	1.94313	3.34816	3.45104	2.72775	4.67261	2.52595	1.83607	1.86275	1.78412
ANKRD30A	-1.88771	-3.09086	-2.25148	-1.85814	2.70957	1.52869	3.46786	2.06572	-3.32193	-3.32193	-3.32193	-3.32193	-3.32193	-3.32193	-3.32193	-3.32193
LINC00993	-3.32193	-2.5217	-3.32193	-3.32193	0.18645	2.17241	-0.61064	1.79836	-3.32193	-3.32193	-3.32193	-3.32193	-3.32193	-3.32193	-3.32193	-3.32193
ZNF248	0.22007	0.18872	0.41818	0.53025	0.97648	1.68316	1.03427	1.61519	0.4063	0.96971	0.75565	1.58837	1.64912	1.67451	1.54901	1.5795
ZNF25	-0.70828	-0.56762	-0.50096	-0.29394	-0.67084	0.08608	-0.74894	0.51281	1.23462	1.32095	0.39227	2.06145	2.29264	2.60445	1.81646	1.95067
ZNF33A	1.30616	1.38556	1.52942	1.58703	2.13694	1.82888	2.40604	1.58298	1.55091	1.37132	1.18918	2.31623	2.48022	2.15606	2.44062	2.26955
ZNF37A	0.71515	0.61028	0.53843	1.09445	1.21514	1.25371	1.7258	1.45339	1.26598	0.925	0.82887	1.77593	2.51003	2.21216	2.20689	2.24653
HSD17B7P2	-0.70051	-0.44168	-0.49299	-0.68858	-0.00379	3.28386	0.71314	3.82139	-0.64584	0.31382	-0.54107	0.34769	1.77006	2.67264	1.17948	1.68619
CCNYL2	0.92831	1.27462	1.12968	1.49105	0.40248	0.65517	0.49039	0.41024	1.16535	1.56179	1.26818	1.93578	1.0919	1.71316	0.99678	1.3174
LINC00839	3.47152	3.34544	3.32407	3.67369	2.29975	2.21195	2.08881	2.04581	3.35244	3.56718	3.333	3.16136	3.40397	3.04273	1.9297	2.87961
ZNF37BP	0.85619	0.24757	0.30871	0.3397	-0.19868	-0.04061	-0.85704	0.49991	-0.34722	-0.32095	-0.41375	-0.04685	0.97201	1.04354	1.04124	1.10634
EIF3LP2	1.01684	1.53933	0.30963	0.52156	-2.16789	-0.77055	-3.32193	-3.32193	-0.00824	1.4312	-0.32661	-0.2699	-1.6169	-1.99912	-1.41325	-2.36697
ZNF33B	0.51269	0.91282	0.66287	1.18282	0.46641	0.26931	0.67022	-0.01594	0.852	1.9373	1.086	2.69929	1.18981	1.08426	1.16405	1.19421
LINC01518	0.67394	0.14676	0.4224	0.30181	2.10156	1.83897	2.10019	2.53399	-3.32193	-3.32193	-3.32193	-3.32193	-3.32193	-3.32193	-3.32193	-3.32193
BMS1	4.51249	4.4645	4.15338	4.3149	3.64308	3.38182	3.97658	3.31941	4.51401	4.01892	4.34277	4.60003	4.79986	4.83004	4.75769	4.87088
CSGALNACT2	4.14424	4.42891	4.34265	5.14475	2.1329	1.90287	2.97975	1.32229	2.61406	2.69164	2.24318	3.147	4.52069	4.49804	3.4472	3.8677
RASGEF1A	1.90391	2.13386	2.09721	2.20706	0.73393	1.31641	0.55764	0.73609	-1.50418	-0.58449	-1.74378	-1.03597	0.2281	0.77079	0.37379	1.04125
ZNF487	-1.22586	-0.56125	-0.28514	0.14299	-0.27114	-0.2768	-0.46187	-0.15567	-1.11219	-0.94028	-1.03568	-0.15449	0.51794	0.95498	0.28317	-0.02502
ZNF239	1.80846	1.47167	1.43402	2.04341	1.27819	1.41394	1.02474	1.69036	1.60804	1.92554	1.24805	1.48433	1.44217	1.717	0.81056	1.04415
CAP1P2	-0.11053	-2.16068	-1.75743	-2.06447	-0.12219	-0.73399	0.1742	-2.11383	-1.25109	-0.79074	-1.3759	0.86648	-2.12368	-1.66658	-2.28503	-2.70131
ZNF485	2.1396	2.00897	2.07194	2.20046	0.04977	0.56068	0.21143	0.8123	0.29757	0.46481	0.31085	0.02977	1.00179	1.36573	0.89692	0.853
ZNF32	3.24858	3.37185	3.1493	3.53988	3.1185	3.95005	3.17467	3.77908	3.89976	4.2533	3.84573	4.1602	5.23487	5.0866	4.27792	4.39137
ZNF32-AS1	3.41291	3.31256	3.23603	3.84022	2.97212	4.34701	2.68778	4.35892	3.85226	4.1864	3.38879	3.64104	5.45783	5.21854	4.1992	4.35882
ZNF32-AS2	1.55093	1.90987	1.94831	1.83366	1.88979	1.47922	2.31705	1.65996	2.6043	3.18809	2.63924	3.11043	3.99602	4.28648	3.31113	3.43459
HNRNPA3P1	0.67726	0.63519	0.96845	-1.1711	-1.4721	-1.16983	-1.78182	-0.98497	0.45129	0.49994	0.02526	-0.303	-0.15081	-0.92812	-0.41394	-1.00296
CXCL12	-3.32193	-2.54226	-3.32193	-1.41856	0.85053	3.17564	0.70127	3.23912	-3.12224	-3.15437	-3.32193	-2.87491	-3.32193	-3.32193	-3.15594	-3.15248
RASSF4	2.01054	2.67875	3.06567	2.03538	0.68916	1.29313	0.56112	1.41709	1.89365	2.85204	1.97254	2.81033	1.85632	2.31308	2.5374	2.5008
DEPP1	2.66738	4.0112	5.10528	3.28342	-1.40881	1.07173	-1.1829	1.1422	5.48438	6.64115	5.3118	5.78017	2.54987	3.408	2.88521	3.08576
C10orf25	-1.85462	-1.18567	-0.58298	-0.50471	-2.92	-3.32193	-3.32193	-3.32193	0.72541	1.08279	1.13073	1.64883	-0.61459	0.70959	0.08809	0.67897
ZNF22	3.12268	3.27225	3.37471	3.14053	-3.14661	-1.97821	-2.96129	-2.26146	4.73651	4.60833	4.64742	4.48137	5.01883	4.7954	4.62323	4.09195

CEP164P1	1.3751	1.36693	1.70733	1.14653	-3.02684	-2.39553	-3.10002	-2.81224	3.22578	3.15573	3.21564	2.79125	3.27402	3.03929	3.24092	2.6447
ALOX5	-1.27889	-0.01819	0.49212	-0.01	-2.88525	-3.04647	-2.65956	-2.74181	-3.32193	-3.32193	-3.32193	-2.84217	-3.10406	-3.10478	-3.06051	-2.31096
ZFAND4	-0.49363	-0.8332	-1.23584	-0.97493	0.49745	-0.40589	0.67772	0.147	0.85451	0.39278	0.78707	1.33357	1.7268	1.7047	1.48496	1.38721
AGAP10P	1.76811	1.10669	1.15672	1.08814	-0.02022	2.08882	-0.57935	2.35084	-0.60026	-0.19464	-0.73994	-0.696	0.56856	0.61186	-0.48822	0.50138
FAM21FP	0.23691	0.56309	1.05839	0.91378	-0.38958	-0.43596	0.00366	-0.88278	-1.1655	-1.01605	0.03505	-0.37738	-0.3769	-0.38138	0.42513	-0.04249
WASHC2C	4.30612	4.0384	4.27336	4.66833	3.94069	2.89437	4.36147	2.80334	4.14899	3.52596	4.42563	4.52784	4.77715	3.55535	5.03261	3.81899
AGAP4	3.05296	2.41705	2.78151	2.9529	0.6161	2.48257	0.62192	2.59747	0.70674	1.14485	0.85809	1.31801	2.41974	2.68499	1.81262	2.25264
PARGP1	1.24484	0.99804	0.79136	0.9928	-0.15208	-0.49332	-0.06408	0.2175	-0.59646	0.07687	-0.11166	0.70822	1.16206	1.559	0.52328	1.6934
TIMM23	7.42916	7.18724	7.07857	7.1564	6.46486	6.3605	6.61616	6.15809	6.84233	5.9912	6.52689	5.61087	7.87868	7.03715	7.64797	6.90502
NCOA4	5.52217	5.45199	5.39715	5.98952	4.91501	4.18914	5.25001	4.16275	5.76601	5.78368	5.75462	6.60765	5.98489	5.55023	5.87896	5.74101
AGAP7P	2.31994	1.72715	2.05585	1.92836	0.90643	2.93264	0.24994	3.38426	-0.78276	0.15869	-0.12213	0.22707	0.99074	1.32733	0.36277	0.80973
AGAP14P	0.57919	-0.14646	0.91488	0.46902	-0.99992	0.79743	-1.53042	0.92028	-1.46293	-0.89978	-1.42195	-1.51451	-0.25514	0.24879	-0.91145	0.47179
ANXA8L1	4.08267	4.01379	4.36855	3.92028	-2.2502	-3.32193	-1.50518	-3.32193	-0.33799	0.16562	0.20508	-0.48413	0.57436	0.26971	-0.69879	0.23402
HNRNPA1P33	2.75288	2.76066	2.62395	1.44346	-0.96778	-0.60053	0.00571	-1.22061	0.62972	0.52011	0.97315	0.02582	1.43361	0.23876	-0.67037	0.39513
NPY4R	-2.83981	-2.7905	-2.36574	-2.32062	-3.32193	-3.32193	-2.86735	-3.32193	0.15661	0.95352	0.76815	1.43054	-0.40967	-0.2997	-1.4533	-0.47485
GPRIN2	-3.32193	-3.32193	-2.76884	-3.32193	-2.90511	-3.32193	-3.32193	-2.76703	-0.43098	1.20519	0.65146	0.10036	-2.37566	-3.32193	-3.32193	-2.8522
SYT15	-1.30901	-0.76907	-0.76579	0.48578	0.4059	-0.89295	0.07228	-0.2161	-0.88601	-0.14388	-0.75092	0.42625	3.05557	3.00253	2.62925	2.8877
SHLD2P1	0.05763	0.40478	0.31018	0.13576	1.10581	0.63075	1.50294	0.72357	0.71442	0.84046	0.32395	0.74929	1.63925	2.19317	1.12575	0.60293
RHEBP1	2.79492	3.0977	3.14811	2.90712	3.74836	3.49182	4.26709	3.25757	3.57828	3.05502	2.59188	2.32084	4.44314	4.92051	3.27735	3.24781
BMS1P1	0.16477	-0.37992	-0.0486	0.15009	0.11486	0.40223	0.2582	1.11399	-1.13281	-0.71625	-0.55274	-0.1415	0.90539	1.69374	0.48514	0.82959
AGAP13P	2.88156	2.71796	2.56705	2.65717	2.24216	3.88751	1.7195	4.03347	0.12972	0.41905	-0.0204	0.60295	1.85501	1.47014	1.99223	2.22966
PTPN20	-3.32193	-3.32193	-2.82816	-3.32193	2.04893	1.81283	2.34142	1.84106	-3.32193	-3.10025	-3.32193	-3.32193	-3.32193	-3.32193	-3.10228	-3.32193
ZNF488	0.07671	-1.3255	-1.17816	-1.89887	1.4392	-1.123	1.25065	0.03447	1.93201	1.32057	1.20652	0.57436	4.46151	3.45498	4.27868	2.8877
ANXA8	4.88266	4.74064	5.10793	4.61474	-2.23047	-3.10983	-2.02205	-3.32193	-0.89713	-0.33135	-0.32603	-0.95521	-2.16675	-2.74749	-2.94442	-2.6336
AGAP9	3.19204	3.04872	2.86093	2.99655	2.36958	4.01575	2.03782	4.41491	0.34664	0.91621	1.33638	1.75159	2.07077	2.72198	1.53301	2.10041
RNA5SP312	2.61392	1.94667	2.4979	1.79848	2.53185	2.91481	2.51623	3.85044	1.28279	2.20673	1.12389	1.96556	2.26829	3.32534	1.40881	4.78255
BMS1P2	1.30987	0.78109	0.92121	1.32878	1.79418	1.53926	1.67614	2.16137	0.56826	0.64152	0.77043	1.58725	2.45788	2.44539	1.78177	2.16905
SHLD2P3	-0.84195	0.53514	-0.18475	0.1526	1.00591	-0.90502	1.09099	-0.55925	0.97292	0.85676	0.18602	1.20007	1.15699	1.82391	1.35562	1.01933
RHEBP2	3.02147	3.23143	3.30858	3.07521	3.85185	3.52084	4.26135	3.29172	3.34678	2.71945	2.55141	2.45827	4.28213	4.77953	3.14869	2.79004
NPY4R2	-2.83981	-2.40299	-1.79569	-2.32062	-3.32193	-3.32193	-2.86735	-3.32193	-0.0346	0.70531	0.62168	1.09169	-0.7433	-0.0025	-0.75515	-0.07117
AGAP12P	1.5744	1.42065	1.26227	1.56447	0.85044	2.99044	0.63127	3.56923	-1.46148	-0.89815	-1.31651	0.11238	0.86613	1.69168	0.08015	1.44324
RNA5SP315	2.61392	1.94667	2.4979	1.79848	2.53185	2.91481	2.51623	3.76856	1.28279	2.20673	1.12389	1.96556	2.26829	3.32534	1.40881	4.78255
BMS1P7	0.43743	0.51086	0.30597	0.75128	1.96024	2.01914	1.58128	2.49268	-0.19796	-0.06839	0.02449	1.69922	2.07164	2.08632	1.23511	1.80065
MAPK8	2.00042	1.77026	1.70098	2.07061	1.01466	0.81093	1.45462	0.8455	2.6277	2.19928	1.58773	2.53801	2.86763	3.01968	2.15813	2.57339
ARHGAP22	2.13449	2.62334	2.79893	1.95772	-3.06566	-3.16399	-3.17553	-3.13785	4.56158	4.99992	5.0575	4.16277	-2.09258	-0.48133	-1.34023	-0.67853
VSTM4	-1.01284	-1.04858	-0.71019	-0.46553	-3.26787	-3.19641	-2.90436	-3.32193	-3.32193	-3.32193	-3.32193	-3.32193	-3.32193	-3.32193	-3.32193	-3.32193
ERCC6	1.67909	1.42587	1.26416	1.62825	0.01136	-1.23954	0.3965	-0.97669	2.06545	2.20814	2.18838	2.58377	3.6932	2.83242	2.57682	3.09798
SLC18A3	-2.92061	-2.87836	-2.85731	-3.32193	-3.1377	-2.91674	-3.32193	-3.32193	6.22579	5.7037	6.07846	4.4528	-3.32193	-1.99876	-1.29974	-1.1705
OGDHL	0.59469	0.73772	0.48662	1.10704	-2.14121	-1.9186	-2.99639	-0.94533	1.14529	1.67161	1.74792	1.02924	-1.41048	-0.6928	-0.39551	-0.29598
PARG	2.85946	2.80294	2.98482	2.81831	2.71342	1.74795	3.032	1.76284	3.07072	2.56611	3.01976	3.05654	3.96514	3.81136	4.04672	3.61333
TIMM23B	3.16405	3.18708	2.56975	3.10905	2.35184	1.95406	2.24684	2.25764	2.22515	2.04992	1.92964	1.84439	3.59664	3.80507	3.06495	2.97857
LINC00843	3.27888	3.27152	2.71505	3.04095	2.39122	2.51822	2.72709	2.63279	2.47403	2.10785	2.31107	2.48144	3.84615	4.14887	3.19647	3.244
AGAP6	3.40763	2.85597	2.97085	3.23767	1.65824	3.64288	1.44019	3.85235	0.55613	0.871	1.17804	1.41207	2.91212	3.01706	1.93676	2.50704
FAM21EP	-1.20115	-1.8843	-0.72496	-0.26827	-1.15474	-1.97752	-0.70937	-1.35948	-2.54976	-1.34516	-1.95617	-2.21452	-0.85407	-1.4681	-1.15572	-0.9546
WASHC2A	5.05428	4.69023	4.8602	5.3625	4.43252	3.21051	4.81039	3.21681	4.39926	3.76924	4.71087	4.67	5.20814	3.96257	5.48168	4.41472
ASAH2	-0.28238	-0.25876	-0.53039	0.18648	0.05531	-0.36949	0.64377	-0.49832	-0.60965	-0.60569	-1.24368	-0.29288	1.09554	0.63896	0.10565	0.46413
DYNC1I2P1	0.53729	0.81462	0.76626	0.85265	0.51308	0.67291	0.44499	0.54207	0.82277	1.03879	0.49806	0.89634	0.08319	-0.39732	-0.01896	-0.17962
SGMS1	2.84527	3.12171	3.00679	3.76581	3.73472	2.36205	4.26814	2.15404	2.73097	2.43209	2.5148	2.65076	3.43251	3.62988	3.18797	3.36323
SGMS1-AS1	-2.03667	-1.5333	-1.81055	-1.53722	-2.52027	-2.45691	-2.32799	-1.96531	-2.07092	-1.52674	-1.64341	-0.54272	-2.04708	-1.83397	-1.72332	-1.08318
SHQ1P1	-1.99538	-1.89293	-1.56401	-1.78403	-2.00323	-2.72907	-0.93707	-0.94754	-1.35056	-0.77418	-1.96455	0.6574	-1.41249	-1.02209	-0.37353	-0.4389

BEND3P1	0.7844	1.02746	0.42227	0.5728	1.83036	0.07375	1.1379	-0.40814	-3.32193	-3.32193	-3.32193	-3.32193	-3.05289	-3.32193	-3.00019	-2.99383
ASAH2B	1.11859	0.84653	0.83654	1.70512	3.29049	2.31102	3.86384	2.03778	0.64664	0.67238	0.24149	1.17085	2.53975	2.16119	1.87849	1.72969
A1CF	-3.32193	-3.32193	-3.32193	-3.18177	3.5282	2.70007	4.2609	2.59932	-3.32193	-3.32193	-3.32193	-3.21542	-3.32193	-3.32193	-3.11488	-3.01554
PRKG1	-2.09294	-1.40164	-1.88109	-0.68487	-1.32706	-1.90374	-1.32494	-2.16509	-3.01119	-2.39801	-3.32193	-2.74657	-2.40282	-2.67581	-3.32193	-2.94045
CSTF2T	4.09409	3.88813	3.77572	3.95149	3.09547	2.43166	3.11427	2.41066	3.73182	3.43809	3.79176	3.62547	4.68841	4.66661	4.47097	4.77061
PRKG1-AS1	-1.93189	-2.38864	-1.6258	-1.88622	-3.0239	-3.32193	-2.50923	-2.91996	-0.89003	-0.62806	-1.19819	-1.29579	-0.21442	-0.49094	-1.11689	-1.56806
DKK1	2.55334	2.75149	1.67103	1.72151	2.11887	-1.84757	2.85982	-1.0691	6.00959	6.91537	6.26409	5.45369	5.92976	6.04292	5.7588	6.18961
MBL2	-3.32193	-3.32193	-3.32193	-3.32193	-0.19133	-0.83206	-0.62578	-0.57985	-3.32193	-3.32193	-3.32193	-2.82388	-3.32193	-3.32193	-3.32193	-3.32193
ZWINT	5.83834	5.15665	5.09609	4.76392	5.60085	4.8714	5.2833	4.85707	5.91603	4.90683	5.55028	3.4983	6.00512	5.83463	5.28383	5.04999
IPMK	2.26532	2.61075	2.72394	2.69114	1.63181	0.69831	2.08617	0.7617	3.12672	3.30015	2.87767	3.98766	2.58708	3.30596	3.02194	3.23062
CISD1	4.34806	4.16488	4.20262	4.42392	3.63615	3.16978	3.55325	3.17278	4.068	3.71649	3.68665	3.67182	4.67184	4.39588	3.2423	3.33924
UBE2D1	2.98046	2.60819	3.18916	3.05432	2.43418	1.63988	2.99949	1.40172	3.1067	1.76818	2.79222	2.68996	3.72046	2.46534	3.26009	2.20762
TFAM	4.74598	4.56326	4.27591	4.71882	3.45495	2.61989	4.03185	2.81404	4.2583	4.06933	3.87916	3.99944	4.50345	4.74212	4.28961	4.2521
BICC1	0.82041	0.83525	0.73787	1.38993	2.55595	1.32256	3.11589	1.64217	4.56809	4.27118	4.07936	4.54063	4.99189	4.81352	6.45992	5.24917
FAM133CP	0.26597	0.54708	-0.40919	1.03451	-2.36853	1.14915	-2.30111	2.70601	-2.13199	-2.27713	-2.22293	-1.27392	-3.32193	-1.17058	-1.68728	-1.66581
PHYHIPL	-3.32193	-2.43337	-3.32193	-3.32193	1.69276	1.93334	2.51372	1.9925	-3.32193	-3.32193	-3.32193	-3.32193	-1.84547	-1.18292	-1.74108	-1.40519
FAM13C	-3.02343	-2.49493	-3.32193	-3.32193	-1.36592	-0.71007	-0.67652	-0.45638	-3.32193	-3.32193	-3.32193	-3.32193	-3.19755	-2.87952	-3.32193	-2.48593
SLC16A9	-3.32193	-3.32193	-2.82091	-2.58757	-1.92286	-2.88432	-2.11492	-3.0488	-3.32193	-3.32193	-3.32193	-3.32193	-1.70874	-0.11908	-1.81585	-1.29402
CCDC6	3.59864	3.25587	3.2949	3.32929	2.91554	2.34139	3.5447	1.96648	4.0016	3.33423	3.85307	4.59522	4.29304	3.60891	4.3863	3.97567
ANK3	-2.81177	-2.86344	-2.63463	-2.63305	-1.77087	-2.93637	-1.62842	-2.80341	-3.13178	-2.91985	-3.19209	-2.89465	-0.49395	0.48641	0.11584	1.08177
CDK1	4.39561	3.87156	4.09832	3.60743	4.40294	3.60374	4.71128	3.71772	4.72621	3.92216	4.03752	2.98616	6.10319	6.1013	5.02336	4.57863
RHOBTB1	0.45054	0.14757	0.51557	1.01629	3.10736	3.17498	3.61491	2.65476	1.35606	1.28359	1.90837	2.00763	3.07077	2.71599	4.13589	3.27425
TMEM26	-3.32193	-2.85897	-3.32193	-3.32193	-2.71316	-3.02653	-2.08097	-3.14095	-3.32193	-3.32193	-3.32193	-3.32193	-2.28182	-0.86943	-1.00884	-1.25842
CABCOCO1	-3.32193	-3.32193	-3.32193	-3.32193	-2.84555	-1.23109	-3.32193	-2.69166	-3.32193	-3.32193	-3.32193	-2.79922	-0.41594	0.55223	0.6334	0.57861
ARID5B	-0.2819	0.08451	-0.28487	1.1917	0.8987	-0.30173	0.72502	-0.12954	1.5985	2.0183	0.94255	2.34077	2.1219	2.74362	3.24842	3.47525
RTKN2	-1.02863	-0.16205	-0.60264	-0.50342	-0.27758	-0.8572	-0.07429	-0.55619	0.75474	0.91621	0.3164	0.80658	2.30446	2.71503	3.2559	2.72174
ZNF365	-2.80823	-3.18966	-2.83097	-2.69905	-3.27044	-3.32193	-3.32193	-3.32193	0.63383	0.77274	0.27226	1.78624	-0.66789	-0.70326	0.59733	-0.41288
ADO	4.53935	4.77147	4.71214	4.43674	3.57353	3.00334	3.22466	2.85328	3.92342	4.23384	4.01537	3.70287	3.80536	4.0374	3.9546	3.86298
NRBF2	4.31478	4.42934	4.7363	4.73569	3.04734	3.09101	3.8123	2.75547	2.77372	2.91472	3.70976	3.52577	2.63439	2.42189	3.96115	2.37843
JMJD1C	2.76956	3.00348	2.8931	3.44415	2.17644	1.33234	2.94623	1.34248	3.03908	3.19363	3.13342	4.06712	3.37503	3.28253	3.57111	3.48385
JMJD1C-AS1	0.26855	1.04095	0.6289	0.5796	0.81088	1.29349	0.17769	0.63423	1.90653	2.37894	2.24409	1.16955	0.97627	0.67595	1.95397	1.52603
REEP3	3.07234	3.26473	3.17562	3.80055	3.73498	3.38091	4.42514	3.12418	4.04733	4.14899	3.79261	4.86884	5.10751	5.08088	4.89899	4.71508
RPL7AP50	0.34654	1.1256	1.58086	0.76736	-1.07961	0.83026	-0.62869	0.46182	0.54765	1.11841	0.39353	0.2757	-0.34311	-1.05574	-1.39736	-1.05566
LINC01515	-1.31713	-1.69838	-1.73034	-1.08728	-2.53791	-1.72208	-2.16743	-1.91366	-2.61824	-2.23951	-2.68012	-2.0639	-0.83641	-1.4525	-1.4518	-1.26299
DNAJC12	0.50317	0.76705	0.18959	1.37544	-1.33907	1.32471	-0.55499	1.43903	-1.4933	-0.24982	-1.99063	-0.61037	0.38298	1.93111	0.01199	1.43657
SIRT1	3.00541	3.06507	2.80103	3.59336	2.03239	0.63076	2.71086	1.27046	3.29179	2.94132	2.68759	3.27846	3.45445	3.4047	3.53343	3.29803
HERC4	2.23981	2.5646	2.47206	2.94575	1.94419	1.111	3.09873	1.13176	2.93519	3.007	2.95249	3.2789	3.60263	3.75248	2.9732	2.99315
MYPN	0.55989	-0.03496	-0.57121	-0.57994	-0.50031	-2.58476	0.22305	-2.19973	2.46745	1.86839	2.16127	2.5021	2.12821	0.32187	-0.10599	-0.60417
PBLD	-0.82318	-0.46755	-1.13427	-0.04184	-0.79088	-0.69932	-1.1072	-0.23508	-0.89952	-0.82865	-0.43955	0.05506	0.26914	1.50052	0.97272	1.1995
HNRNPH3	5.67089	5.44892	5.41887	5.29733	4.50192	4.8258	4.86509	4.99712	5.16712	4.08001	4.82385	3.91202	5.78274	5.63234	5.3998	5.01893
RUFY2	3.67432	3.53712	3.51953	3.54043	2.72372	2.84369	3.24822	2.93224	3.48658	2.51827	3.22474	3.12086	4.17861	3.83937	4.05874	3.63286
DNA2	2.38374	1.88659	1.57125	1.69619	2.51291	1.2371	2.79334	1.7413	2.65751	1.6316	1.34761	0.93665	3.49265	3.58505	2.34009	2.55801
SLC25A16	1.58013	1.55153	1.57657	1.88191	0.58526	2.15959	1.07835	2.44651	0.55943	0.99029	1.1163	1.46202	1.83279	2.81211	2.26108	1.99855
TET1	-1.20341	-1.89528	-1.56664	-0.88084	0.78396	-1.1636	1.10003	-0.95055	-0.37672	-0.75748	-0.88502	-0.16462	1.22582	0.52984	1.88055	0.95215
COX20P1	4.3135	4.23947	4.55692	3.92549	1.32228	2.30328	1.34208	1.89534	5.30454	4.90056	4.6752	4.57294	1.31293	-0.0762	0.63501	-0.07611
CCAR1	4.7887	4.80323	4.64404	5.39724	4.40209	4.78433	4.83458	5.03543	4.51416	4.29761	4.36655	5.20246	4.5743	4.56491	4.28514	4.34343
STOX1	-0.68488	-0.54381	-0.89406	0.66252	0.05978	-0.27618	-0.10085	0.14929	-3.32193	-2.81653	-3.02875	-2.82376	-1.127	-1.13102	-0.84452	-0.57103
DDX50	4.89842	4.74746	4.74287	4.8579	3.46713	3.20538	3.83889	3.16144	5.30657	5.30662	4.99585	5.10093	5.09331	4.86793	4.9289	4.54121
DDX21	6.62441	6.41218	6.18256	6.72502	6.07868	5.00933	6.67977	5.22611	6.37001	5.60944	6.04208	5.97204	7.04442	6.4099	6.69391	6.02418
VPS26A	4.98691	5.01482	5.08817	5.50175	4.40596	3.87063	5.20961	3.42873	5.32033	4.57298	4.95513	5.11473	5.75523	4.62496	5.43048	4.69796

SUPV3L1	3.92378	3.61142	3.39693	3.96439	2.87768	2.55368	3.16948	2.76618	3.25023	3.03702	3.08915	3.47843	3.60658	3.7408	3.0918	3.32997
HKDC1	5.83316	6.03681	5.54903	5.7036	5.12661	4.23134	5.31167	4.27728	4.2313	4.38462	3.67649	4.26223	3.68197	5.18206	4.29177	5.35008
HK1	3.74458	3.95205	4.07688	3.83604	2.15653	2.25627	2.30198	1.47264	6.31985	5.96223	6.16464	5.95299	3.73183	2.90353	4.11102	3.81306
TSPAN15	4.5369	4.69182	4.95548	4.65989	5.22053	4.60287	5.2225	4.26442	4.82425	4.81839	5.11726	4.57404	3.8427	4.24546	4.41654	4.69114
FAM241B	2.17953	2.08162	1.81066	1.72059	3.48141	4.13348	3.27191	3.90202	4.34787	4.44207	4.5339	2.95154	2.98142	3.65885	2.69418	2.68803
COL13A1	2.93184	2.99877	3.2553	2.7657	-3.16562	-3.1377	-2.99826	-3.32193	3.71978	3.98951	3.64948	3.27288	5.65661	4.67587	5.22847	4.98484
AIFM2	4.46245	4.35574	4.28882	4.36136	4.20422	3.83915	3.98723	3.78645	3.9401	3.48055	3.77016	3.50251	4.19281	4.00468	3.87526	3.90518
TYSND1	3.51032	3.49089	3.44374	2.92239	2.81576	3.21205	1.64424	3.40084	3.17771	3.70879	3.80059	2.58253	2.69789	3.14803	3.18756	3.57048
SAR1A	4.47558	4.6451	4.49071	4.62896	4.26721	3.92445	4.69298	3.77946	4.41385	4.39619	4.06729	4.79148	4.97788	4.80108	5.02088	5.16763
CALM2P2	1.25887	1.18764	2.09744	1.21832	0.86633	1.47257	1.61528	-0.45521	2.49462	2.5566	2.22288	3.6214	1.88477	1.36656	2.10777	1.51306
PPA1	5.60495	5.61436	5.33985	5.47677	4.99673	4.90008	5.44166	4.91981	5.73634	5.65602	5.45615	5.72642	5.54532	5.53254	5.21111	4.92412
LRRC20	3.71812	3.61867	3.31717	2.41778	3.2288	2.62322	2.54985	2.61714	4.47404	4.02193	3.92641	2.64384	4.21861	4.1429	4.67176	4.40046
EIF4EBP2	4.25343	4.70002	4.39099	4.54862	4.36352	3.92349	4.32668	3.92713	5.23618	4.86805	4.87087	5.25247	5.25033	5.80679	5.96834	6.35013
PALD1	-2.72626	-2.50228	-3.05666	-3.04085	-2.80169	-2.42556	-2.61159	-1.38185	1.00094	2.13908	1.55672	1.49245	-2.47319	-2.02144	-1.60933	-1.32446
ADAMTS14	-2.65363	-2.45273	-2.28838	-2.24079	-3.15185	-3.32193	-3.32193	-3.32193	-1.97657	-0.81418	-0.88225	-0.79894	-3.32193	-3.32193	-3.32193	-3.32193
SGPL1	2.82323	2.76858	2.75906	3.01034	3.72865	2.89078	3.83959	2.9114	4.72417	4.25665	4.51194	4.5655	4.13246	3.66691	4.45979	4.40564
PCBD1	5.71992	5.58742	5.34562	5.29925	6.1269	6.6363	5.78125	6.69041	5.95621	5.66889	5.58562	5.23682	7.0821	6.82707	6.31252	6.38511
UNC5B	-0.97589	-0.32128	-0.87952	-0.2434	-1.4164	-1.20872	-2.20763	-0.70658	-3.32193	-2.5016	-3.01735	-2.19557	-3.32193	-2.88443	-2.80262	-2.58703
SLC29A3	1.85911	2.09991	1.84572	2.70592	4.36178	4.98797	4.31362	5.04954	0.46177	0.82623	0.99074	0.99384	-0.4017	-0.09663	0.83519	0.95175
CDH23	-2.72192	-2.80174	-2.42231	-2.37908	0.00241	0.24649	-0.68614	-0.30306	-3.18454	-3.26334	-3.19887	-3.32193	-2.94006	-2.58123	-2.59378	-1.94691
VSIR	0.88012	0.70388	0.77717	0.57992	4.22564	2.74646	3.69713	2.71427	3.22007	3.12522	3.47537	2.79873	3.74584	3.22148	4.0356	2.91531
CHST3	2.51684	2.92104	2.61249	2.90031	3.3487	2.07359	3.64866	1.96388	3.52392	3.95098	3.39931	3.97384	3.14137	3.49217	4.19048	4.19558
SPOCK2	-2.36201	-2.27866	-1.68984	-1.84176	-0.28179	-0.02329	-0.94596	-1.24054	-3.13628	-2.30374	-2.33704	-1.67665	-2.86749	-2.45002	-3.16773	-1.81362
ASCC1	3.31281	3.42014	3.30416	3.30301	4.02243	3.92213	4.20059	3.62592	3.43825	3.78443	3.24775	4.10736	3.84391	4.23007	3.55747	4.27046
ANAPC16	4.49992	4.62988	4.67547	4.63612	4.14582	4.83938	4	4.8369	3.97705	4.40237	3.87191	4.35168	4.86203	4.88659	4.0459	4.66636
DNAJB12	4.08389	4.00129	3.98993	3.92665	3.86675	3.84322	3.75507	3.70514	3.66858	3.14687	3.43147	3.26031	4.14292	3.64367	4.1179	4.18481
MICU1	4.30635	4.38738	4.46185	4.72109	3.34893	3.80878	3.81881	3.5977	4.36239	4.04424	4.19877	4.55566	4.57534	5.19959	5.02214	4.94636
MCU	3.30898	3.02717	3.24666	3.24169	2.76647	2.90532	3.29998	2.85758	3.2739	2.44608	2.92987	2.7279	4.2118	3.80463	4.11244	3.33508
OIT3	-3.32193	-2.8695	-3.32193	-3.32193	-0.20662	-0.3891	0.40633	-0.71922	-3.32193	-2.92527	-3.32193	-3.32193	-3.32193	-2.99288	-2.92871	-2.13226
NPM1P24	1.80148	0.45073	0.80374	0.70869	-0.61472	0.20578	0.91288	-0.11082	1.33447	1.28925	0.85499	1.40669	-0.48982	-0.79989	-0.74343	-1.89197
PLA2G12B	-3.32193	-2.49633	-3.32193	-3.32193	3.55217	3.13682	3.4278	2.9469	-2.47412	-3.32193	-3.32193	-3.32193	-3.32193	-3.32193	-3.32193	-3.32193
RPL17P50	1.86935	0.76168	2.16302	-0.004	-0.17655	1.8848	0.37203	1.48807	0.93921	2.33187	1.20716	2.09673	0.14046	-0.61082	0.67162	-0.61073
P4HA1	5.17726	5.56129	5.87514	5.62891	4.39951	4.61957	4.92309	4.23138	5.76808	6.34656	5.82833	6.5024	4.49127	3.98454	3.9613	3.87478
NUDT13	1.99243	2.10958	1.90586	2.4291	0.99703	1.32007	0.91204	1.58149	0.50998	1.16187	0.91532	1.21293	1.61664	1.40462	0.47851	0.70682
ECD	4.81234	4.83525	4.52629	5.04664	3.6041	3.26188	4.12842	3.22323	4.11685	3.812	3.9859	4.28811	4.30915	4.24148	3.70519	3.55679
FAM149B1	3.23511	3.30963	3.261	3.44497	2.7073	2.62758	2.90815	2.54086	3.52152	2.88964	2.87804	2.98502	4.38014	4.23606	3.65237	3.46145
DNAJC9	4.60149	4.41799	4.41298	4.43316	4.12049	3.75835	4.16669	3.73958	4.88497	3.89704	4.15434	3.78186	5.69022	5.62455	5.28385	5.05524
DNAJC9-AS1	3.60248	3.69224	3.56479	3.99446	2.47771	3.35867	2.95733	3.42588	2.69875	2.61231	2.50432	2.87227	3.76789	4.18453	3.57802	3.38706
MRPS16	6.1518	6.26769	6.22568	5.95323	5.72728	5.83435	5.60098	5.83358	6.1505	5.9499	5.99898	5.71435	6.59904	6.77277	6.53551	6.52858
CFAP70	-1.79548	-1.39039	-1.47412	-1.68481	-2.37192	-1.7335	-2.33985	-1.86782	-2.63733	-2.4691	-2.69787	-2.25872	-1.60296	-1.50286	-2.39758	-2.63327
ANXA7	6.04552	6.08953	6.06586	6.43702	5.25763	5.08584	5.50093	5.12527	5.52438	5.23162	5.41672	5.59555	5.95784	6.0444	5.73833	5.62107
MSS51	-0.20298	0.04929	-0.09717	0.25312	-1.71758	-0.69391	-2.18706	-0.21429	-2.23994	-1.40358	-1.45222	-1.20416	0.14244	0.43322	-1.53677	-0.98177
PPP3CB	3.62997	3.88151	3.87269	4.133	1.97241	2.32155	2.54736	2.06261	2.783	3.17282	3.08155	3.9199	3.94843	4.16229	4.10124	3.99679
PPP3CB-AS1	1.74333	1.64044	1.34563	1.59875	0.41637	0.14702	0.73716	0.16688	1.21464	1.04324	1.1325	1.84525	1.72107	1.35068	2.0079	1.68544
USP54	1.93898	1.84575	1.72859	2.06166	0.90298	0.20071	1.05348	0.21336	1.89114	1.6296	1.75168	2.70184	2.15068	1.83339	2.3082	2.09978
AGAP5	1.35989	0.9603	1.22859	1.45528	0.2763	1.71111	-0.50494	2.20304	-0.55806	0.01862	0.04152	0.35726	1.00929	0.7277	-0.34034	0.59476
BMS1P4	1.06839	1.22745	1.35545	1.87154	0.24001	0.84997	0.64347	0.97307	0.41027	-0.22565	0.39722	0.59516	0.95655	1.24005	0.24393	0.73704
GLUD1P3	1.41519	1.73123	1.8069	2.03667	1.10576	0.83135	1.23581	1.4823	0.6707	-0.03855	0.92513	-0.17252	1.15659	1.42933	1.22314	0.84362
DUSP8P5	0.05763	0.66709	0.78336	-0.13649	0.06264	0.80151	-0.51719	1.12783	-0.8695	-0.39639	-0.0204	-0.67349	-0.31561	-0.04705	-0.06287	0.08376
SEC24C	5.47458	5.56878	5.45321	5.63846	5.32767	4.84549	5.25454	4.71236	5.5433	5.68566	5.89704	5.77836	5.09863	5.02779	5.45842	5.49445

FUT11	3.06199	3.54444	3.83743	3.1078	3.59172	3.3863	3.6868	3.28743	4.35197	4.26525	4.36467	4.68673	2.82211	2.67374	3.43699	3.06223
CHCHD1	5.52184	5.48358	5.45934	5.37588	5.09856	5.65596	4.74269	5.56326	5.33957	5.24984	5.18682	4.21381	5.89306	5.78799	4.51316	4.61843
ZSWIM8	4.50967	4.96281	4.91013	4.98022	3.66247	4.48283	3.32613	4.38945	3.59708	4.35038	4.21255	4.53379	3.32863	3.96162	3.87134	4.55276
ZSWIM8-AS1	5.44044	5.87948	5.84956	6.05025	4.41886	5.47733	4.17098	5.41455	4.3172	5.15062	4.95391	5.00226	4.22297	4.82367	4.50046	5.11476
NDST2	2.97659	2.81645	2.93492	2.93296	3.18954	2.66032	2.56984	2.48396	2.65601	3.04212	3.08385	3.05672	2.33299	2.47707	2.32764	2.82074
CAMK2G	3.54512	3.53277	3.62442	3.60998	1.34239	1.79411	1.36681	2.02222	2.73834	3.01648	3.0726	3.13981	2.98062	3.29215	2.92552	3.13969
C10orf55	6.83818	6.32014	5.9185	6.08561	-0.77319	-1.17348	0.07172	-2.5275	7.11156	7.22901	6.66552	7.54483	6.88213	7.26273	5.65828	6.52996
AP3M1	3.63998	3.43262	3.43923	3.82141	3.47254	2.491	4.13238	2.27703	3.96943	3.70741	3.56351	4.71204	4.99224	4.76424	4.85626	4.6135
ADK	4.35085	4.49232	4.49801	4.40664	3.60168	3.35464	4.18318	3.33249	5.41278	4.90796	5.07834	4.73862	4.93803	4.76838	4.53376	4.18792
KAT6B	0.91374	0.97031	1.02992	1.66823	0.76461	0.07661	1.06694	0.21525	1.01824	1.00728	0.94862	1.98349	0.56503	0.05093	1.23645	1.27681
DUSP13	-3.32193	-1.08763	-1.14562	-0.09928	-1.68823	2.08368	-1.08994	0.96012	-3.32193	-3.32193	-3.32193	-3.32193	-3.32193	-2.72222	-3.32193	-3.32193
SAMD8	2.69741	3.15563	2.97122	3.36713	2.16329	1.94237	3.15112	1.89092	2.82109	3.20394	2.90191	3.83594	3.13777	3.58202	3.38701	3.59553
VDAC2	7.06896	7.10071	7.03107	7.14576	5.8822	5.97025	6.58373	5.45041	6.39715	5.90967	6.06224	6.04094	6.82916	6.49502	6.43277	6.02092
COMTD1	5.02327	5.07604	5.14431	4.36203	4.20133	5.55715	3.19107	5.31153	4.5898	5.0861	4.69202	3.56497	3.23464	3.37528	2.85372	3.2326
ZNF503	5.14445	5.22785	5.08047	4.68284	1.554	2.16487	0.82104	2.44643	3.95831	3.54907	3.98717	2.23767	2.68709	3.03413	2.65004	3.35901
ZNF503-AS2	2.33276	2.59894	2.2946	2.2926	1.12909	1.11275	0.10265	0.92007	1.08468	1.47637	1.36374	0.38812	0.92444	1.13697	0.44526	1.59371
LRMDA	0.35965	0.79357	0.58656	1.53997	-3.18378	-2.03774	-3.1708	-2.33276	-1.46878	-0.60159	-1.05906	-0.43576	-1.1748	-0.495	-0.79982	-0.39955
KCNMA1	-0.45116	-0.4773	-0.3903	-1.03629	-3.26953	-3.14294	-3.23654	-3.14625	3.55022	3.46815	3.8499	3.83226	3.61942	2.73466	3.87182	3.59048
KCNMA1-AS1	-2.17848	-1.52807	-2.11891	-2.15275	-3.32193	-3.1699	-3.18102	-3.32193	2.48382	2.67476	2.87615	3.5235	2.03103	1.27817	3.05425	2.49985
DLG5	2.86964	2.99778	2.94997	3.10904	3.48263	2.88829	3.42731	2.57531	4.35656	4.37405	4.44464	4.54197	4.08355	4.01458	5.01145	5.10017
DLG5-AS1	-1.05613	-0.92263	-1.31885	-1.73522	-0.2723	-0.04907	-2.94752	-0.04635	-2.01399	-2.16852	-2.91176	-1.99734	-1.42061	-0.8089	-1.10879	-1.08398
POLR3A	3.43024	3.50639	3.07584	3.47517	2.89342	2.05551	2.97433	2.1019	3.0904	2.41383	2.90045	3.14641	3.79005	3.2277	3.90207	3.58289
LINC00856	0.57912	0.63487	0.43124	0.25016	-3.32193	-3.32193	-3.32193	-3.32193	-3.32193	-3.21088	-3.20297	-3.11115	-3.23107	-3.32193	-3.10975	-3.32193
ZMIZ1-AS1	3.14825	2.48792	2.45948	2.28046	-2.94583	-1.94602	-3.10328	-2.29151	-1.92674	-1.0326	-1.35629	0.08943	-1.52606	-2.56979	-1.65473	-1.41992
ZMIZ1	3.61012	3.48973	3.78389	3.56148	2.13139	1.66375	1.41001	1.57661	4.10217	4.20193	4.49976	4.66107	4.11488	3.57573	5.25232	4.86185
PPIF	7.38815	7.18739	6.81257	6.68632	5.08742	4.58069	4.70798	4.60494	6.80352	6.40063	6.48222	6.02134	6.59638	6.6675	5.86253	6.07767
ZCCHC24	2.88127	3.39936	3.54345	3.49481	0.80822	1.60966	0.33546	1.579	2.5792	3.59715	2.83827	4.10199	1.00143	1.63726	2.31341	2.9198
EIF5AL1	2.6885	2.97007	2.36524	2.40737	1.02246	1.51539	0.30097	1.48153	2.18275	2.00629	1.78662	0.20642	0.13429	0.24935	0.11656	0.72959
NUTM2B-AS1	1.36597	1.26809	1.23244	1.24144	0.60271	0.23831	0.98587	0.30878	0.58508	0.45747	-0.18571	0.85629	1.11132	1.16042	0.42811	0.64899
BEND3P3	-0.71564	-0.42928	0.05949	-0.68499	-0.24639	-0.04643	-1.84608	-0.28837	0.62474	0.93333	0.36317	-0.27981	1.82329	1.69094	2.08072	2.02616
NUTM2B	-0.76391	-0.5142	-0.904	-1.04175	0.4492	0.31393	-0.27349	0.6691	-0.1534	-0.0019	0.11273	-0.25385	-0.39915	-0.29408	-0.51859	-0.19218
NUTM2E	-0.8635	-0.6696	-1.1357	-1.33166	0.0263	0.16018	-0.15295	0.31725	-0.62705	-0.65111	-0.22751	-0.19879	-0.85496	-0.51109	-0.61724	-0.46036
TMEM254-AS1	-0.47729	-1.40505	-0.74147	-1.07499	0.04946	0.17028	-0.29391	-0.4784	-0.69555	-0.79336	-0.83343	-1.48231	0.31407	0.24028	0.7704	0.96973
TMEM254	1.87772	1.94517	1.90773	1.87366	2.07922	2.87877	1.8505	2.6396	1.58047	1.74306	1.69592	2.28949	2.92773	2.85167	2.63438	2.63639
LINC00857	1.27722	1.01865	1.59568	0.62683	3.79083	2.57423	3.98038	2.20389	2.40441	2.3501	2.36083	2.46531	3.41774	3.48993	2.94699	2.95885
EIF5AP4	4.11199	4.42335	4.29838	3.01913	2.81698	3.53299	1.81867	3.07689	4.31387	4.41584	3.66927	2.22916	0.8479	1.90621	1.56968	0.78976
MAT1A	-2.01606	-3.01616	-1.8655	-3.32193	3.82287	3.28296	3.26045	3.78233	-3.00639	-2.8307	-2.59626	-3.05962	-2.74242	-1.92034	-2.83483	-2.3031
PRXL2A	3.81262	4.07047	3.94894	4.40178	3.59535	4.40751	3.80968	4.39216	3.6984	4.02343	3.41021	3.92696	5.2592	5.64548	5.33873	5.53144
GHITM	7.2686	7.30594	7.12627	7.52566	6.37533	6.56114	6.60785	6.32125	7.14447	7.11623	7.03027	6.84887	7.30243	7.34303	6.94642	7.03427
CCSER2	2.14136	2.20435	2.27416	2.70748	0.99838	0.82165	1.46331	0.98901	2.80666	2.62894	2.10416	3.50345	3.42578	3.38039	3.61548	3.41471
RPS3AP5	2.72745	2.28532	2.81635	0.82436	0.62794	1.5692	0.41725	0.83721	1.66753	2.90378	0.64981	0.98693	0.08785	-0.53456	0.78627	1.21042
GRID1	-2.59533	-2.5271	-2.62545	-2.74089	-3.24385	-2.98303	-3.32193	-3.32193	-2.07713	-2.31461	-2.00452	-2.86522	-1.92063	-1.53394	-0.36752	-0.56696
WAPL	3.78674	3.9443	3.77733	4.00735	3.06251	2.05671	3.64715	1.99369	4.08104	3.90688	4.02348	4.424	4.21515	4.23336	4.60376	4.31578
LDB3	-2.89029	-2.98739	-2.82297	-2.65559	-2.69608	-2.88616	-3.32193	-2.56373	-2.36148	-2.78745	-2.43985	-2.32779	-2.38777	-2.19774	-1.90783	-1.14658
BMPR1A	1.71195	1.57424	1.78735	2.15473	2.72285	1.26928	3.20813	1.42246	1.91076	1.72549	1.84011	2.49852	2.79629	2.55228	3.22339	2.94469
MMRN2	-1.58724	-1.28525	-0.77925	-0.83853	-3.07657	-2.52651	-2.57424	-2.56298	-2.31131	-1.62373	-2.18058	-1.99004	-3.32193	-3.02659	-3.32193	-2.22706
SNCG	3.48266	4.1056	3.87772	4.07344	0.24338	2.17152	-0.22557	0.51853	0.56026	2.0639	1.89167	1.58068	-1.02892	0.033	-0.95467	1.0311
ADIRF-AS1	0.13643	0.59401	0.76525	0.76747	-3.32193	-3.32193	-3.32193	-3.32193	-1.16721	-0.65782	-0.60054	-1.34225	-3.17686	-2.61491	-3.14699	-2.84136
AGAP11	-0.45328	-0.58634	-0.86751	-1.05296	-2.70327	-0.29718	-2.19969	-0.4544	-1.58004	-0.89521	-1.69446	-0.79067	-3.06185	-2.4841	-2.75489	-3.32193
GLUD1	5.6009	5.70827	5.56981	5.80252	6.52501	5.60952	6.52261	5.58183	6.14	5.93852	5.82744	6.06081	5.83809	6.18055	6.01577	6.10345

SHLD2	4.07704	4.06115	3.92676	3.93089	5.20132	3.25033	5.15058	3.19422	5.03223	4.90796	4.60872	4.66413	4.93014	4.89208	4.93214	4.8698
NUTM2A-AS1	2.76452	2.62414	2.62756	2.89463	1.50713	1.08906	1.64842	1.1737	1.94098	1.85937	1.50901	2.05268	2.69824	2.54583	1.57388	2.10466
NUTM2A	-0.04319	0.0122	-0.10117	-0.4045	1.33303	0.96564	0.72583	1.00525	-0.33587	0.13936	0.51495	0.15503	-0.31132	0.02571	0.20142	1.17695
LINC00863	1.45656	1.90969	1.87994	1.71783	2.15451	1.44682	1.96826	1.24782	0.84929	1.6242	1.15578	1.57895	1.56911	1.77307	1.3798	1.83969
NUTM2D	-0.58494	-0.17512	-0.18991	-0.62806	0.98071	0.89823	0.60495	0.9139	-0.50018	-0.19851	0.24122	-0.10138	-0.56807	-0.48228	-0.14203	0.68454
MINPP1	4.08805	4.26892	4.12743	4.64669	4.14088	3.38257	4.45831	3.56722	3.87155	4.01834	3.98201	4.02017	3.65989	3.90743	3.54802	3.77725
PAPSS2	4.08412	4.13534	4.36724	4.68528	4.15021	1.51982	4.45213	0.93261	5.99946	5.32274	6.00398	5.6629	4.60902	4.34204	4.64698	4.44402
ATAD1	3.95789	4.0445	3.92408	4.616	4.10508	3.01911	4.87614	2.91673	3.84766	3.79193	3.74896	4.17836	4.58158	4.46605	4.36376	4.06364
CFL1P1	0.25622	0.37404	-0.41841	0.20702	1.60189	0.40374	1.75684	-0.41348	0.62236	0.2425	0.67054	0.15073	0.70857	0.34372	1.21855	0.61662
KLLN	-1.22892	-1.23148	-1.5714	-1.5064	-0.91163	-1.48656	-0.88789	-2.42404	-0.0205	-0.08938	0.20504	0.2642	-2.96091	-1.23171	-1.34179	-1.03037
PTEN	2.93851	3.09672	3.33117	3.34231	3.21708	2.4144	3.61305	2.19275	4.12497	4.74222	4.52421	5.14353	3.16558	3.36406	4.02631	3.80371
RNLS	0.22109	0.34925	0.01689	0.73936	-1.7722	-3.32193	-2.01531	-2.76668	1.28666	1.1943	0.68953	1.36141	1.4232	1.88335	0.96079	1.48337
STAMBPL1	1.50449	1.54809	1.28762	1.68463	-2.81904	-3.32193	-3.02426	-3.32193	1.91877	1.79633	1.20151	1.9217	3.39297	2.46556	2.42904	2.79983
ACTA2-AS1	-0.14457	0.3213	0.98381	0.91482	-3.32193	-3.00401	-3.32193	-3.32193	2.47769	2.99155	3.43093	3.17722	-0.61269	1.1705	1.56727	1.03645
ACTA2	0.48951	1.12319	1.88128	1.97049	-3.17038	-2.71089	-3.32193	-3.32193	3.8119	4.30311	4.7481	4.22051	0.31516	2.12938	2.47325	2.12106
FAS	1.9953	1.88145	1.74113	2.66519	-3.11403	-3.07764	-3.32193	-3.32193	3.48734	3.05927	3.4899	3.31627	2.16181	2.39422	1.27338	2.09928
LIPA	4.14383	4.57307	4.61808	4.90171	4.25742	4.16349	4.62967	4.22741	4.96205	5.19281	4.76817	5.74042	5.54035	5.92829	5.41833	5.75025
IFIT2	2.16692	2.55526	2.75203	3.70322	-1.73245	-2.50194	-0.66665	-3.02903	2.95926	3.18249	3.64954	3.61001	2.49321	1.71209	2.60854	1.56686
IFIT3	4.37624	4.88427	4.67582	5.8252	-1.36896	-1.44412	-0.07294	-1.15306	4.89369	4.76411	5.17262	5.65069	3.4188	3.06382	3.3231	3.12687
IFIT1	1.82058	1.76753	1.67335	3.00783	-0.47061	-0.92886	0.50508	-0.05816	3.11531	3.39448	3.07871	3.36343	0.97943	0.91549	1.2418	0.87999
IFIT5	2.76307	2.78473	2.34837	2.96556	1.67585	1.54321	2.19942	1.61634	3.71995	3.8041	3.81327	4.44606	3.00094	3.34769	3.04892	3.0801
SLC16A12	-1.62269	-0.95906	-1.30658	-1.23709	-3.32193	-3.10495	-3.32193	-3.32193	-1.46906	-1.52423	-1.73914	-1.67557	3.09819	3.48412	3.33301	2.94437
SLC16A12-AS1	-2.23459	-3.32193	-2.09999	-1.38085	-3.32193	-3.32193	-3.32193	-3.32193	-1.46798	-3.32193	-3.32193	-3.32193	1.9918	2.88518	3.23926	2.56502
PANK1	0.66317	1.09863	0.72504	1.21362	1.06314	0.39211	1.40679	0.4784	0.24686	0.28337	0.64448	0.28382	1.19984	2.30181	1.59925	2.13867
KIF20B	4.22159	3.93496	3.94613	4.51613	2.98169	2.5025	3.89562	2.9193	4.17164	3.12237	3.43021	3.36191	4.09707	3.50111	3.84461	3.38052
HTR7	0.77504	1.20036	0.80287	1.39765	-3.32193	-2.99928	-3.02165	-3.32193	0.25688	0.72571	1.2697	1.65759	-3.32193	-3.32193	-3.32193	-3.32193
RPP30	4.18966	3.97741	3.84673	4.02616	2.48106	2.7098	2.88492	2.79176	3.25722	2.94334	2.92914	2.93704	3.89398	3.59913	3.29256	3.0566
ANKRD1	5.40521	5.26453	4.32426	3.86849	-0.62227	-3.32193	0.50373	-1.80297	4.12287	4.16591	3.56079	2.3241	5.66923	4.38879	3.82183	1.28365
PCGF5	3.07158	3.07688	2.92916	3.21187	2.7928	2.44053	3.41684	2.17739	3.17439	2.81887	2.57208	3.67136	3.70515	3.36013	3.42534	3.07628
HECTD2	-0.79311	-0.165	-0.30664	0.36469	-0.1489	-0.2741	0.64291	-0.38954	0.68478	0.94171	0.81291	2.0514	1.2151	1.60539	1.50562	1.36666
PPP1R3C	2.05622	2.68167	2.71107	2.07545	1.39701	1.90209	0.97504	1.64876	4.91452	5.49067	4.5825	4.79665	-0.90611	0.50613	-0.60426	-0.08895
TNKS2	3.58188	3.90128	3.56173	4.25071	3.32485	2.39205	3.96966	2.30094	4.27775	4.3718	4.02382	5.26257	4.81539	5.21142	4.98551	5.43595
SRP9P1	5.67939	5.72341	5.65759	5.2051	5.69358	4.85614	6.2456	4.00144	6.3395	5.85855	5.72183	5.0844	6.67766	6.34601	6.30944	5.57564
FGFBP3	-0.37536	0.17231	-0.34656	-0.48203	0.72614	2.52942	0.6859	1.82613	0.17373	0.70414	-0.27472	-0.75762	2.5025	3.52097	2.07223	2.57596
BTAF1	2.79875	2.69267	2.7278	3.14121	3.18711	2.6576	3.69079	1.98534	2.80757	2.66636	2.77042	3.56346	3.52503	3.26321	3.70376	3.44075
CPEB3	-0.36683	-0.39731	-0.55661	-0.22792	-0.83263	-1.29747	-0.3319	-0.68044	-0.13938	0.27969	-0.11802	0.84666	-0.68612	0.51574	-0.30339	-0.03076
NHP2P1	2.58008	1.45405	1.62543	0.21458	0.91355	1.20669	0.26023	1.06909	1.5027	0.63964	0.73661	0.07797	1.05251	-1.46482	0.76884	0.11586
IDE	2.94505	2.91085	2.65	3.3647	3.16719	2.31893	3.76197	2.20611	3.43573	2.71238	3.08612	4.04474	3.93196	3.64672	3.87122	3.73144
KIF11	4.06938	3.81568	3.84452	3.4037	3.85922	2.4618	4.35806	2.36527	5.1979	3.90159	4.53983	3.95809	5.36715	4.7186	5.66924	4.70221
EIF2S2P3	0.24812	0.58033	0.8129	0.73917	-1.21775	-0.30945	-2.53517	1.02893	-0.85852	-0.36914	-2.47008	-0.70006	-1.10412	-1.54545	-2.00956	-2.50787
HHEX	3.59179	3.50423	3.37928	3.56298	5.01602	4.05601	4.66547	4.82952	2.91975	3.03938	2.98789	2.66351	3.23929	3.13523	2.85424	2.73097
EXOC6	1.23622	1.15316	0.90058	1.30632	2.60213	1.62184	3.15142	1.46893	1.20139	1.13137	0.57448	1.17776	3.08985	2.713	2.90813	2.56891
CYP26A1	-2.98719	-2.95108	-3.32193	-3.32193	-1.84968	2.09483	-2.74901	1.65258	-2.93949	-3.32193	-3.32193	-3.32193	-3.32193	-2.63346	-3.32193	-2.99442
CEP55	5.41103	5.06821	5.20321	4.75799	4.58453	3.42002	5.23253	3.40961	5.75611	4.40101	4.69127	4.1058	6.21107	5.7544	6.20363	5.18245
RBP4	-1.07419	-0.64145	-0.8778	-0.13383	7.07533	9.2492	6.59388	9.11699	-2.62363	-1.28665	-2.68513	-2.72951	-3.32193	-2.81794	-3.32193	-1.69395
FRA10AC1	2.63907	2.80092	2.6605	3.0011	1.34321	1.95533	1.48797	2.28488	1.94299	1.8744	1.70922	1.81757	2.31297	2.41648	2.0081	2.13061
SLC35G1	1.69455	0.99126	1.03727	1.36853	1.65781	0.10665	1.71016	0.04645	1.33198	0.42051	0.28508	0.46687	2.64648	1.20735	1.2955	0.70534
PIPSL	-0.5581	-1.19084	-0.29755	-1.7144	-0.90796	-0.44918	-1.4759	-0.24466	-0.84219	0.05423	-0.72736	-1.21946	-1.65379	-1.57728	-1.13952	-1.51996
PLCE1	0.53063	0.3087	0.61963	0.34995	1.57441	-0.31474	1.94005	0.21881	1.29762	0.18407	0.81578	0.42887	1.37686	0.69019	1.54577	0.71932
PLCE1-AS1	-3.32193	-1.99776	-1.56445	-2.43774	-0.35317	-2.31041	0.36531	-1.56044	-1.2811	-1.76707	-0.95342	-1.95024	0.52442	-0.68275	-0.05366	-1.02251

NOC3L	3.33089	3.49841	3.29907	3.85342	3.14086	2.26109	3.80486	2.20417	4.01671	3.83629	3.81337	3.80167	4.90547	4.25354	4.10413	3.64415
TBC1D12	2.32531	2.29517	2.03056	2.16624	2.20334	1.2572	2.24402	1.41913	3.48446	3.51373	2.90242	2.69329	2.87388	2.73293	2.55357	2.46101
HELLS	3.12042	2.67437	2.63389	2.86266	2.20935	1.261	2.38762	2.07128	1.68321	0.42115	0.76589	0.27416	3.13257	3.22834	2.68663	2.51789
CYP2C18	-3.32193	-2.87804	-2.50591	-2.46562	0.49434	3.22483	0.37679	3.06113	-2.8645	-3.32193	-2.58639	-3.32193	-2.99831	-2.3203	-3.32193	-1.96232
CYP2C9	0.4893	0.21617	0.84481	0.66404	-2.91516	-0.47327	-3.01319	-0.43728	-3.32193	-3.32193	-3.32193	-3.32193	-3.32193	-3.32193	-3.00686	-2.73799
PDLIM1	6.82636	6.71093	6.93058	6.56007	4.94256	5.57151	5.01178	5.34002	7.31663	6.70306	6.92748	6.16068	7.02586	6.6851	6.88731	5.85322
SORBS1	-0.35568	-0.32102	0.06125	0.13247	2.6364	1.20473	2.59604	1.2755	-2.09904	-2.24687	-1.96544	-1.92569	-0.07608	1.41404	1.40855	1.50447
ALDH18A1	5.24712	5.05214	4.73947	5.04372	5.91806	5.98314	5.90421	5.82212	5.88216	5.61316	5.63996	5.7007	5.54421	5.6314	5.47091	5.69392
TCTN3	3.84598	3.68452	3.57076	3.79716	4.95498	4.20807	4.74678	4.2018	4.37941	4.05802	4.23721	4.53903	4.45093	4.27141	3.97158	4.12065
ENTPD1	-0.11495	-0.39227	-0.42285	-0.02186	-0.64671	0.31909	-0.31063	0.48234	-1.97027	-1.82415	-1.81384	-2.07625	-0.04137	0.04185	0.29409	-0.37102
ENTPD1-AS1	-0.63225	-0.63853	-1.12144	-1.09674	-0.36064	-1.34229	0.21912	-1.39592	-1.07568	-1.53562	-1.06297	-1.48032	0.37717	0.09386	-0.33638	-0.12634
CC2D2B	-1.6123	-1.79686	-1.6786	-1.91071	-1.1191	-2.87566	-1.09497	-2.67245	-1.57278	-2.14069	-1.42516	-2.57365	-0.79207	-0.81989	-1.10427	-0.81981
CCNJ	2.15692	1.52212	1.76676	1.90209	2.7854	2.00576	3.39839	1.83663	2.21582	0.85922	1.93416	1.60618	3.55322	2.40595	3.2474	2.83385
ZNF518A	0.50422	0.88259	0.88567	1.43771	1.97067	2.2206	2.96156	2.17505	0.89224	1.256	1.08594	2.33875	2.18573	2.44669	2.3584	2.79989
TM9SF3	5.33899	5.48001	5.43827	5.71556	5.81995	4.96761	6.57435	4.91558	5.57106	5.60419	5.57781	5.81468	5.94118	5.90616	5.84261	6.00241
PIK3AP1	0.45239	0.57815	-0.11403	0.52894	3.86992	1.44353	3.94499	1.22557	-0.32648	-0.35137	-0.49921	-0.89012	-1.32498	-0.45775	-0.54387	-0.2425
RPL13AP5	6.44665	6.33895	6.4519	5.33212	6.37487	6.81278	5.69573	6.43764	7.4747	7.88411	7.47131	6.36127	5.41355	4.8876	5.17718	5.24206
LCOR	0.48901	0.55183	0.68609	1.05963	1.25264	0.53413	2.07653	0.73514	2.13723	1.77368	1.96029	2.99037	2.45768	2.39248	2.94608	2.79984
ARHGAP19-SLIT	-0.01145	-0.17096	0.03134	-1.55804	1.30921	-1.10586	0.95799	-1.04755	1.88603	0.19317	0.74246	0.49931	1.46557	0.20415	1.18563	0.55647
ARHGAP19	1.70437	0.97421	1.08932	0.33317	2.04243	0.87225	1.73576	0.95692	2.55537	1.18415	1.52454	1.16832	2.74754	1.40835	2.06118	1.2757
FRAT1	1.49814	2.1401	2.36954	2.08492	3.86631	4.21835	3.0105	4.13413	0.40708	1.25608	1.43841	0.8434	-2.07266	-1.10177	-1.39696	-0.34704
FRAT2	3.8489	4.0144	4.12318	3.52084	5.26227	5.17499	4.95892	5.11606	3.25663	2.9529	3.88536	2.19798	2.27077	2.0054	2.85442	2.5142
RRP12	5.03182	4.92376	4.55423	4.58668	4.16849	4.47307	4.01381	4.34161	3.94039	3.77437	4.14259	3.79369	4.20444	3.83365	4.57347	4.45817
EXOSC1	4.23631	4.16713	4.11564	4.0266	3.29806	3.98775	3.18054	3.88574	4.07912	3.6146	3.78917	3.42277	4.09251	3.72197	3.62648	3.29811
ZDHHC16	4.27533	4.36191	4.34934	4.39323	4.97132	4.99047	4.47561	4.80383	4.74103	4.70422	4.50263	4.62766	4.45862	4.56687	4.50498	4.83393
MMS19	4.24759	4.02717	4.1256	4.04288	3.30499	3.83737	3.36717	3.62981	4.70451	4.79979	4.89217	5.08751	4.40902	4.82726	4.73147	4.79892
UBTD1	5.82744	5.91292	5.79598	5.40495	4.37401	5.36855	3.57355	5.0655	5.41151	5.94394	5.80964	5.02379	5.86458	6.09128	6.38362	6.41964
ANKRD2	-1.07576	-1.83595	-1.26241	-0.64342	-3.32193	-3.32193	-2.73765	-2.13771	3.29263	2.46357	1.75242	0.28161	1.889	2.70715	2.54093	1.09472
HOGA1	-1.70925	-1.89965	-2.24581	-1.47463	0.32452	1.74583	-0.42233	2.22789	-1.47	-1.57968	-2.2089	-1.85008	1.55765	2.65376	1.84854	1.29597
MORN4	1.79399	1.71827	1.95241	1.73869	0.7551	2.00174	0.62481	1.95796	1.5544	2.14406	1.99016	1.8539	3.36661	3.33418	2.77719	2.65788
PI4K2A	4.11062	4.12633	4.37844	4.17122	3.04062	2.78659	3.00996	2.6178	3.56785	3.31386	3.61011	3.75128	4.40138	4.22272	4.47324	4.12126
AVPI1	4.38736	4.41447	4.56697	4.09136	6.32895	5.8792	5.46449	5.8692	4.41113	4.35341	4.08157	3.29214	3.5331	3.1896	2.97843	2.65213
MARVELD1	0.23823	0.6021	1.11643	1.11017	-3.18764	-2.77155	-3.32193	-3.32193	4.44839	4.88548	5.18533	4.96118	1.80775	1.78784	2.62632	2.91488
ZFYVE27	3.62783	3.25898	3.23498	3.37192	4.22878	4.27205	3.76246	4.15074	2.61253	3.04912	2.88749	2.37188	3.03175	3.28766	2.90553	3.25409
GOLGA7B	0.31233	1.39092	1.40523	0.70693	2.03079	1.509	2.41653	1.2291	-1.24711	0.50704	-1.57112	0.63997	-2.59156	-3.07384	-2.29855	-2.12654
CRTAC1	-1.6509	-1.43558	-1.58678	-2.24945	-0.27993	-1.97369	-0.2618	-1.69969	-3.32193	-1.5032	-2.78331	-2.62483	-3.09402	-3.32193	-2.62089	-2.43232
R3HCC1L	2.61361	2.33687	2.61476	2.69274	2.04099	1.62693	2.55629	1.94424	2.22421	2.56203	2.41237	3.0603	2.78529	2.63015	2.53764	2.57381
LOXL4	0.36713	0.80358	1.61831	1.18429	6.16136	4.21187	5.75233	3.97889	2.12023	3.00884	3.61203	2.75846	2.82933	2.6898	4.89611	2.80394
PYROXD2	0.21876	0.58176	0.67627	0.57872	-2.57182	-1.38046	-1.89641	-1.90453	1.74102	2.21665	2.1043	2.18657	0.0829	0.60953	0.25368	0.08367
HPS1	3.68725	3.59452	3.71441	3.64195	3.5186	4.10662	2.84109	3.78347	4.39803	4.56287	4.53225	4.15577	3.51507	3.38296	3.45067	3.47454
CNNM1	-3.32193	-3.32193	-2.80578	-3.32193	4.1664	2.26266	3.84477	2.55037	2.81458	2.16982	2.88687	2.39898	0.63286	0.11442	0.40577	0.0955
GOT1	4.91328	4.54988	4.42076	4.41689	5.70621	5.55583	5.84657	5.11916	5.17644	4.28919	4.63289	4.2918	5.1196	4.97008	4.69491	4.58888
SLC25A28	4.24667	4.13521	3.97388	4.0793	3.86042	4.26583	3.38128	4.54204	3.54421	3.40471	3.74754	3.23374	4.01575	4.12066	3.50759	3.3924
ENTPD7	2.89591	3.30254	3.11248	3.38057	4.62631	3.25421	5.11062	2.66333	3.49188	3.25005	3.664	4.14439	5.0705	5.09714	5.28481	5.3533
CUTC	4.4464	4.19635	3.97547	4.30693	4.79067	4.66114	4.7707	4.51709	4.11132	3.8683	4.11139	3.68843	4.869	5.1964	4.61547	4.71528
COX15	4.04662	4.1877	4.09357	4.22323	4.06411	3.68016	4.30004	3.68638	3.68529	3.80406	4.03254	4.39652	4.44074	4.82998	4.64937	4.69904
ABCC2	3.677	3.1343	3.26994	4.11266	6.70368	5.42922	6.78671	5.62027	4.32744	4.84809	4.81229	6.2144	5.17596	5.2005	4.87763	5.53843
DNMBP	2.1826	2.0265	1.97281	2.04607	1.80617	1.307	2.12284	1.27083	4.42763	4.65605	4.43958	5.39221	4.00289	2.74155	3.60817	3.30771
[truncated: 1,158,051 more chars]
